
JOURNAL INFORMATION
==============================
NLM Title Abbreviation: J Physiol Sci
Journal ID: J Physiol Sci
NLM Journal ID: 101262417

The Journal of Physiological Sciences

ISSN: 1880-6546
EISSN: 1880-6562

ARTICLE INFORMATION
==============================
PMCID: PMC10227792
DOI: 10.1186/s12576-023-00867-3
Article ID: 867
Article version: 1
Subjects: Meeting Abstracts

Proceedings of the 100th Annual Meeting of The Physiological Society of Japan

Electronic publication date: 2023 May 30
Print publication date: 2023
Volume: 73
Issue: Suppl 1
Issue sponsor: Publication costs for this supplement were funded by The Physiological Society of Japan.
Electronic Location ID: 11
Copyright: © The Author(s) 2023
License: Open AccessThis article is licensed under a Creative Commons Attribution 4.0 International License, which permits use, sharing, adaptation, distribution and reproduction in any medium or format, as long as you give appropriate credit to the original author(s) and the source, provide a link to the Creative Commons licence, and indicate if changes were made. The images or other third party material in this article are included in the article's Creative Commons licence, unless indicated otherwise in a credit line to the material. If material is not included in the article's Creative Commons licence and your intended use is not permitted by statutory regulation or exceeds the permitted use, you will need to obtain permission directly from the copyright holder. To view a copy of this licence, visit http://creativecommons.org/licenses/by/4.0/.
License URL: https://creativecommons.org/licenses/by/4.0/

Conference name: The 100th Annual Meeting of The Physiological Society of Japan
Conference location: Kyoto, Japan
Conference date: 14-16 March 2023
issue-copyright-statement: © The Physiological Society of Japan 2023

==============================

### Plenary Lectures

*J Physiol Sci* (2023) **73** (Suppl. 1):11
https://doi.org/10.1186/s12576-023-00867-3
Published: 30 May 2023

### Plenary Lecture 01

March 14 (Tue.), 11:10 - 12:10, Room 1

### 1PL01

**Recent Progress in iPS Cell Research and Application**

*Shinya Yamanaka[1] (*[1]Center for iPS Cell Research and Application, Kyoto University)*

Induced pluripotent stem cells (iPSCs) can proliferate almost indefinitely and differentiate into multiple lineages, giving them wide medical applications. As a result, they are being used for new cell-based therapies, disease models, and drug development around the world.
As translational research, we are proceeding with an iPSC stock project in which clinical-grade iPSC clones are being established from healthy donors with homologous HLA haplotypes to lower the risk of transplant rejection. We started distributing the iPSC stock to organizations in Japan, and related clinical studies have begun for age-related macular degeneration (AMD), Parkinson's disease, corneal epithelial stem cell deficiency, and other diseases, giving expectation that iPSC-based regenerative medicine, which includes iPSC-derived CAR T-Cell therapy, will be widely used in the future. Additionally, we reported HLA gene-edited iPSCs that could expand the range of patients who benefit from iPSC therapies faster than the homologous HLA haplotype strategy.
Over the past decade, iPSC research has made great progress, moving toward innovative therapeutics for people with intractable diseases by the application of new findings from basic science and reverse translation from clinics.

### Plenary Lecture 02

March 15 (Wed.), 11:10 - 12:10, Room 1

### 2PL01

**Archaic Genomics**

*Svante Pääbo[1,2] (*[1]Max Planck Institute for Evolutionary Anthropology, Leipzig, Germany, [2]Okinawa Institute of Science and Technology, Onna-son, Okinawa, Japan)*

Our laboratory works on methods to retrieve DNA from ancient bones and other tissue remains and its application to recent human evolution. We take a particular interest in the physiological consequences of genetic differences between present-day people and our closest evolutionary relatives.
I will describe our work to sequence genomes from Neandertals as well as a previously unknown extinct Asian hominin group related to Neandertals, which we named "Denisovans". Analyses of these genomes show that gene flow occurred among modern human ancestors and these archaic hominins. As a consequence, about 2.0% of the genomes of people living outside Africa come from Neandertals while about 4.0% of the genomes of people living in Oceania come from Denisovans. These genetic contributions have numerous physiological and medical consequences today. I will discuss recent insights into archaic genetic variants that affect pain sensitivity, the risk for miscarriages, and the risk to develop severe disease when infected by SARS-CoV-2. I will also describe recent insights into genetic changes that affected modern humans after their divergence from the ancestors of Neandertals and Denisovans and their consequences for modern human physiology and brain development.

### Plenary Lecture 03

March 16 (Thu.), 11:10 - 12:10, Room 1

### 3PL01

**Exercise as Medicine in a translational perspective**

*Bente Klarlund Pedersen[1] (*[1]The Department of Infectious Diseases and The Copenhagen Muscle Research Centre, Rigshospitalet, University of Copenhagen)*

Physical activity decreases the risk of a network of diseases, and exercise may be prescribed as medicine for lifestyle-related disorders such as type 2 diabetes, dementia, cardiovascular diseases, and cancer. During the past couple of decades, it has been apparent that skeletal muscle works as an endocrine organ, which can produce and secrete hundreds of myokines that exert their effects in either autocrine, paracrine, or endocrine manners. Recent advances show that skeletal muscle produces myokines in response to exercise, which allow for crosstalk between the muscle and other organs, including brain, adipose tissue, bone, liver, gut, pancreas, vascular bed, and skin, as well as communication within the muscle itself. Although only some of these myokines have been allocated to a specific function in humans, it has been identified that the biological roles of myokines include effects on, for example, cognition, lipid and glucose metabolism, browning of white fat, bone formation, endothelial cell function, hypertrophy, skin structure, and tumor growth. This suggests that myokines may be useful biomarkers for monitoring exercise prescription for people with, for example, cancer, diabetes, or neurodegenerative diseases. The talk will include suggestions about how to translate basic research to clinical praxis and political decisions.

### Memorial Lectures

### S. Tawara Memorial Lecture

March 15 (Wed.), 14:20 - 15:20, Room 1

### 2ML01

**Hypothalamic regulation of fat and carbohydrate metabolism**

*Yasuhiko Minokoshi[1] ([1]*Division of Endocrinology and Metabolism, National Institute for Physiological Sciences, Okazaki, Aichi, Japan)*

Over 20 years, critical neural pathways in the hypothalamus and brainstem mediating energy homeostasis have been illuminated by genetics, neuroscience, and physiological sciences. However, the brain regulation of macronutrient metabolism remains largely unknown. We have recently found that carbohydrate intake is increased in response to fasting and glucoprivation by 2DG (2-deoxyglucose) through AMPK (AMP-activated protein kinase)-regulated CRH (corticotropin-releasing hormone) neurons in the paraventricular hypothalamus (PVH) in mice. Intraperitoneal 2DG administration increased the selection of an HCD (high carbohydrate diet) and inhibited that of an HFD (high fat diet) via NPY (neuropeptide Y) neurons projecting to the PVH and activation of the CRH neurons. Because mice highly prefer HFD under a normal energy status, the results suggest that the NPY-CRH neural axis in the PVH is a homeostatic system to maintain whole-body glucose availability. We also recently found that the diurnal cycle for glucose and fat utilization is regulated in the PVH. PVH NOS (nitric oxide synthase) 1 neurons are essential to increase fat utilization during the light (resting) period in mice. Suppression of the neural activity of PVH NOS 1 neurons increased carbohydrate utilization throughout the day independently of the diurnal cycle of feeding. Our findings thus provide a novel insight to unravel the homeostatic regulation of macronutrient metabolism.

### S. Hagiwara Memorial Lecture

March 16 (Thu.), 14:20 - 15:20, Room 1

### 3ML01

**Formation of mature neural circuits through activity-dependent synapse pruning**

*Masanobu Kano[1,2] ([1]*Department of Neurophysiology, Graduate School of Medicine, The University of Tokyo, Tokyo, Japan,* [2]*International Research Center for Neurointelligence (WPI-IRCN), The University of Tokyo Institutes for Advanced study (UTIAS), The University of Tokyo, Tokyo, Japan)*

Precise formation of neural circuits during development is prerequisite for proper functions of the brain. Diverse and redundant synaptic connections are formed initially, and they are refined subsequently by strengthening some connections and eliminating others. This process is known as synapse pruning and is widely thought to be crucial for shaping mature neural circuits. Postnatal development of climbing fiber (CF) to Purkinje cell (PC) synapses in the cerebellum has been a representative model of synapse pruning in the developing brain. Each PC initially receives excitatory synapses from more than five CFs on the soma with similar strengths. During the first three postnatal weeks, synaptic inputs from a single CF are selectively strengthened in each PC and the strengthened CF extends its innervation territories along proximal dendrites, while redundant synapses from the other CFs are eliminated. Most PCs eventually become innervated by single strong CFs on their proximal dendrites by postnatal day 20. In this lecture, I will review the mechanisms of CF synapse pruning and discuss how neural activity contributes to the formation of mature neural circuits in the cerebellum.

### Special Lectures

### Special Lecture 01

March 14 (Tue.), 14:20 - 15:20, Room 1

### 1SL01-01

**A novel B cell-derived metabolite elicits anti-inflammatory macrophages and limits anti-tumor cytotoxic responses**

*Sidonia Fagarasan[1,2] ([1]*Laboratory for Mucosal Immunity, RIKEN Center for Integrative Medical Sciences,* [2]*Division of Integrated High-Order Regulatory Systems, Center for Cancer Immunotherapy and Immunobiology, Kyoto University)*

The evolving field of immunoregulation studies how the activity of lymphocytes is shaped by their local environment via a variety of receptor interactions with soluble and cell-bound proteins. However, small metabolites derived from metabolism of immune cells are likely present in both intracellular and extracellular milieu in vivo, many of which may have signaling potential we have yet to understand. A growing body of research addresses the flux in metabolic products produced and consumed by different immune cells in various stages of differentiation and activation. We hypothesized that water-soluble metabolites provide environmental cues which mediate interactions between immune cells and thereby regulate their functions. I will discuss how a small metabolite produced and secreted by B cells and plasma cells promotes monocyte differentiation into anti-inflammatory macrophages which secrete IL-10 and inhibit CD8+ T cell killer function and anti-tumor responses. We propose that in addition to cytokines and membrane proteins, small metabolites derived from B lineage cells have immunoregulatory functions, which may be pharmaceutical targets allowing fine-tuning of immune responses.

### Special Lecture 02

March 14 (Tue.), 15:30 - 16:20, Room 1

### 1SL01-02

**iPSCs-based Regenerative Medicine and Drug Development of CNS disorders**

*Hideyuki Okano[1] ([1]*Dept. Physiology, Keio University School of Medicine, Japan / Dept. Brain & Cognitive Sciences, MIT)*

It has been 15 years since the birth of human iPSC technology in 2007, and the scope of its application has been expanding. In addition to developing cell therapies using iPSC-derived cells, pathological analyses using disease-specific PSCs and clinical trials to confirm the safety and efficacy of drugs developed using iPSCs are progressing. With the innovation of related technologies, iPSC applications are about to enter a new stage (Okano and Morimoto, Cell Stem Cell, 2022).

We have investigated cell therapy for spinal cord injury (SCI) for more than 20 years. Following SCI, ischemia induces secondary damage processes, leading to axonal degeneration and death of both neuronal and glial cells, and consequently to dysfunction of motor and sensory systems. It is hoped that cell replacement therapy could be an effective treatment. Our group has been developing the transplantation of allogeneic iPSC-derived neural stem/progenitor cells (NS/PCs) into patients with complete SCI in the subacute phase. Pre-clinical studies have shown that such NS/PCs could induce long-term functional recovery of SCI model animals without tumor formation. DREADD-mediated modulation of graft-derived neuronal activity showed that activity of graft-derived neurons and synapse formation between host and graft neurons are essential for full functional recovery, leading to the conclusion that these cell replacement effects contribute to the graft-mediated functional recovery of SCI animals. In December 2021, our group at Keio University performed cell transplantation of human iPSC-derived NS/PCs for the first participant in the clinical research of "Regenerative Medicine using iPSC-derived Neural Progenitor Cells for Subacute Spinal Cord Injury." The transplanted NS/PCs were prepared at a Good Manufacturing Practice-grade cell processing facility using a clinical-grade integration-free hiPSC line established by the CiRA. After performing all quality checks, the long-term safety and efficacy of cells were confirmed using immunodeficient mouse models. After the consent was obtained, the cryopreserved cells were thawed and prepared through a multi-step process, including treatment with γ-secretase inhibitors to promote cell differentiation. Approximately 2 million iPSC-NS/PCs were transplanted into the injury's epicenter. The surgery was successfully completed, and the patients are in good condition.

iPSC-based techniques also offer new opportunities for disease modeling and developing new drugs, especially for conditions such as neurological and psychiatric diseases in which access to the affected cells and pathogenic sites is limited. Today, I am going to focus on the iPSCs-based investigation of ALS. Using iPSCs-Motor Neurons (MNs) derived from familial ALS patients with FUS and TDP-43 mutations, we could identify Ropinirole (ROPI: Already approved as an anti-PD drug with D2R agonist ) as a potential anti-ALS Drug which is further being investigated as a clinical trial (ROPALS trial)(Fujimori et al., Nat Med, 2018). As a result of the ROPALS trial, we conclude that ROPI is a safe and effective drug for ALS patients. ROPI significantly suppressed the progression of ALS and prolonged the time to respiratory failure over the 1-year treatment period. This clinical trial proved that iPS cell drug discovery was useful as a new drug screening tool.

### Special Lecture 03

(March 15 (Wed.), 15:30 - 16:20, Room 1)

### 2SL01

### Mechanism and *In Vitro* Reconstitution of Mammalian Germ-Cell Development

*Mitinori Saitou[1,2,3] *([1]Institute for the Advanced Study of Human Biology, Kyoto University, [2]Department of Anatomy and Cell Biology, Graduate School of Medicine, Kyoto University, [3]Center for iPS Cell Research and Application, Kyoto University)*

The germ-cell lineage ensures the creation of new individuals, perpetuating/diversifying the genetic and epigenetic information across the generations. We have been investigating the mechanism for germ-cell development, and have shown that mouse embryonic stem cells (mESCs)/induced pluripotent stem cells (miPSCs) are induced into primordial germ cell-like cells (mPGCLCs) with a robust capacity both for spermatogenesis and oogenesis and for contributing to offspring. These works have served as a basis for exploring the mechanism of key events during germ-cell development such as epigenetic reprogramming, sex determination, meiotic entry, and nucleome programming.

By investigating the development of cynomolgus monkeys as a primate model, we have defined a developmental coordinate of the spectrum of pluripotency among mice, monkeys, and humans, identified the origin of the primate germ-cell lineage in the amnion, and have elucidated the X-chromosome dosage compensation program in primates. Accordingly, we have succeeded in inducing human iPSCs (hiPSCs) with a primed pluripotency into human PGCLCs (hPGCLCs) and then into early oocytes with appropriate epigenetic reprogramming. We have also shown that hPGCLCs can be propagated to ~$10^6$-fold over a period of 4 months under a defined condition. These studies serve as a oundation for promoting human *in vitro* gametogenesis.

Here, I would like to provide a brief overview of our work and discuss our latest findings regarding the *in vitro* reconstitution of mammalian germ-cell development.

### Special Lecture 04

(March 16 (Thu.), 15:30 - 16:20, Room 1)

### 3SL01

### Towards Systems Biology of Human Sleep/Wake Cycles: Phosphorylation Hypothesis of Sleep

*Hiroki R. Ueda[1,2] *([1]Systems Pharmacology, Graduate School of Medicine, University of Tokyo, [2]Laboratory for Synthetic Biology, Center for Biosystems Dynamics Research, RIKEN)*

The detailed molecular and cellular mechanisms underlying NREM and REM sleep in mammals are elusive. To address these challenges, we constructed a mathematical model, Averaged Neuron Model (AN Model), which recapitulates the electrophysiological characteristics of the slow-wave sleep. Comprehensive bifurcation analysis predicted that a Ca2+-dependent hyperpolarization pathway may play a role in slow-wave sleep. To experimentally validate this prediction, we generate and analyze 26 KO mice, and found that impaired Ca2+-dependent K+ channels, voltage-gated Ca2+ channels, or Ca2+/calmodulin-dependent kinases (*Camk2a* and *Camk2b*) decrease sleep duration, while impaired plasma membrane Ca2+ ATPase increases sleep duration. Genetical and pharmacological intervention and whole-brain imaging validated that impaired NMDA receptors reduce sleep duration and directly increase the excitability of cells. Based on these results, we propose phosphorylation hypothesis of sleep that phosphorylation-dependent regulation of Ca2+-dependent hyperpolarization pathway underlies the regulation of sleep duration in mammals. In this talk, I will also present how we identify essential genes (*Chrm1* and *Chrm3*) in REM sleep regulation as well as a new project on human sleep/wake cycle measurements for next-generation sleep medicine.

References:1.Tatsuki et al. Neuron, 90(1) : 70–85 (2016). 2. Sunagawa et al, Cell Reports, 14(3):662-77 (2016). 3. Susaki et al. Cell, 157(3): 726–39, (2014). 4. Tainaka et al. Cell, 159(6):911-24(2014). 5. Susaki et al. Nature Protocols, 10(11):1709-27(2015). 6. Susaki and Ueda. Cell Chemical Biology, 23(1):137-57 (2016). 7. Tainaka et al. Ann. Rev. of Cell and Devel. Biol. 32: 713-741 (2016). 8. Ode et al. Mol. Cell, 65, 176–190 (2017). 9. Tatsuki et al, Neurosci. Res. 118, 48-55 (2017). 10.Ode et al, Curr. Opin. Neurobiol. 44, 212-221 (2017). 11. Susaki et al, NPJ. Syst. Biol. Appl. 3, 15 (2017). 12. Shinohara et al, Mol. Cell 67, 783-798 (2017). 13. Ukai et al, Nat. Protoc. 12, 2513-2530 (2017). 14. Shi and Ueda.BioEssays 40, 1700105 (2018). 15. Yoshida et al, PNAS 115, E9459-E9468 (2018). 16. Niwa et al, Cell report, 24, 2231-2247. e7 (2018). 17. Ode and Ueda, Front. Psychol. 11, 575328 (2020). 18. Katori et al, PNAS 119, e2116729119 (2022). 19. Ode K.L. et al, iScience 25, 103727 (2022), 20. Tone D. et al, PLOS Biology in press.

### Symposia

### Symposium

[1AS01m]
Committee for 100th anniversary
**Homeostasis for sustainability -Toward the next century of physiological sciences-**

March 14 (Tue.), 9:00 - 11:00, Room 1

**Part 1**
**Talks from International Physiological Society presidents**

### [1AS01m-01]

*Yoshihiro Ishikawa[1] ([1]*President of Physiological Society of Japan, Japan*)

### [1AS01m-02]

*Sue Wray[1] ([1]*President of International Union of Physiological Sciences and President of Federation of European Physiological Societies, UK*)

### [1AS01m-03]

*Robyn Murphy[1] ([1]*President of Australian Physiological Society, Australia*)

### [1AS01m-04]

*Chae Hun Leem[1] ([1]*President of Federation of the Asian and Oceanian Physiological Societies and President of The Korean Physiological Society, Korea*)

### [1AS01m-05]

*Dee U. Silverthorn[1] ([1]*President of American Physiological Society, USA*)

**Part 2**
**Pannel Discussion**

*Utako Yokoyama[1] ([1]*Tokyo Med University*)

*Kazuhiro Nakamura[1] ([1]*Nagoya University*)

*Lin Kurahara[1] ([1]*Kagawa University*)

*Yuki Suda[1] ([1]*University of Yamanashi*)

### Symposium

[1AS02a]

**Neurobiology of the social brain**

March 14 (Tue.), 14:20 - 16:20, Room 2

### [1AS02a-01]

### Group living and social affiliation in mammals: the neural basis and the regulation by the environmental factors

*Kumi O Kuroda[1], Kansai Fukumitsu[1], Nami Ohmura[1], Takuma Kurachi[1], Kazutaka Shinozuka[1], Chihiro Yoshihara[1] ([1]*RIKEN Center for Brain Science*)

Eibl-Eibesfeldt's seminal work in 1972 has suggested two evolutionary origins of sociality in mammals; one is self-defense, dealing with the conspecific group as their home to hide, and the other is parental care. For the neural basis of the latter lineage of sociality, here I would like to propose that calcitonin receptor (Calcr) and its ligand amylin in the medial preoptic area (MPOA), which acts as a common signaling pathway for parental care and female social affiliation in mice (Yoshihara, 2021; Fukumitsu 2022). This pathway is not involved in defensive huddle of mice, in harmony with the independent circuity of these two lineage of sociality. It is postulated that females tend to be more gregarious in mammals for the benefits in maternal care. The group living of multiple females enables commural nursing in Mus musculus, in which mothers make a large communal nest and nurse any pups within it. However group living has a large drawback under severe foraging demand, and this fact seems to underly the puzzle why the amylin-Calcr signaling, which regulates feeding behavior and energy metabolism is involved in regulation of group living.

### [1AS02a-02]

### Neurobiology of stress and aggressive behavior

*Aki Takahashi[1] ([1]*Faculty of Human Sciences, University of Tsukuba*)

Although aggressive behavior is adaptive behavior which helps animal's survival and reproductive success, social stress such as social isolation escalates aggression to maladaptive level. Understanding the neurobiological mechanisms of escalation of aggression in animal models would provide important insight into the biological basis of anger outburst and violence in human. One of the models to study escalated aggression is social instigation, in which a brief provocation by a rival male increases aggressive behavior of male mice. Previously, we identified an involvement of the glutamatergic input from the lateral habenula (LHb) to the dorsal raphe nucleus (DRN) on social instigation-heightened aggression. We then examined which cell types in the DRN are responsible for escalation of aggression. Anatomical analysis showed that LHb neurons synapse on DRN neurons that project to the ventral tegmental area (VTA), and optogenetic activation of the DRN-VTA projection increased aggressive behavior. By contrast, optogenetic manipulation of 5-HT neurons in the DRN showed rather suppressive effect on instigation-heightened aggression. These results indicate an important modulatory role of the DRN on escalation of aggression.

### [1AS02a-03]

### Neurobiology for social behavior

*Toru Takumi[1] ([1]*Kobe University School of Medicine*)

Social behavior is complex, including social interaction, attack, sexual behavior, maternal care, etc. Recent progress in neuroscience, such as optogenetics and chemogenetics, combined with network analysis using imaging techniques, started to understand the neural circuit and network of social behavior; however, it's just like the mediocre cannot understand the great. We focused on social interaction as social behavior and developed different systems to understand brain regions and neural networks for social interaction behavior. Those systems include fMRI using awake mice, Ca imaging using microendoscope, and our unique virtual reality system for analyzing cortical functional network dynamics during behavior. I'll introduce these systems using a mouse model of autism spectrum disorder (ASD) with impaired social behavior.

### [1AS02a-04]

### Neural basis of social action monitoring in the macaque

*Taihei Ninomiya[1,2] ([1]*NIPS*, [2]*Grad. Univ. Adv. Sci.*)

We often make our own decision based on others' actions and their consequences. The ventral premotor cortex (PMv) and the medial prefrontal cortex (MPFC), core nodes in social brain networks, are involved in processing social action information. Despite rich anatomical connections between the PMv and MPFC, these frontal areas are rarely concurrently active in neuroimaging, inviting the hypothesis that they are functionally independent. To investigate possible interactions between the PMv and MPFC, we recorded neural activities simultaneously in the two areas while macaque monkeys performed a turn-taking choice task. We found that information flow from the PMv to the MPFC increased as the biological nature of observed actions increased. We also found impairments in the processing of observed, but not executed, action when the PMv-to-MPFC pathway was selectively blocked by a double viral vector infection technique. These findings demonstrate that coordinated activity in the PMv-to-MPFC pathway is causally involved in social action monitoring.

### [1AS02a-05]

### Molecular and neural basis of self-awareness arising from social comparison

*Makiko Yamada[1] ([1]*National Institutes for Quantum Science and Technology*)

Humans have a desire to be respected and recognized as valuable. In order to find their own value, they constantly evaluate themselves relative to others, and this characteristic of human self-evaluation has been theorized as social comparison theory (Festinger 1954). Such self-evaluation based on comparisons with others is known to lead, in many cases, to the belief that one is better than average. For example, in a survey that asked university professors whether their teaching abilities were above or below the average professor, 94% rated themselves as above (Cross, 1977), and many similar results have been reported. Statistically, however, it is impossible for the majority of a group to be above average. Therefore, this is considered an illusion to which everyone is prone and is called the "superiority illusion". The superiority illusion is known to have adaptive aspects, such as believing in one's own potential, having hope for the future, and reaching one's goals, which leads to physical and mental health. My talk will provide an overview of the cost and benefits of the superiority illusion from a psychological perspective and outline the molecular and neural bases that produce the superiority illusion.

### Symposium

[1S03a]
International Relations Committee
**Dynamics & homeostasis of organella/cellular function**

March 14 (Tue.), 14:20 - 16:20, Room 3

### [1S03a-01]

### Ion homeostasis and organelle function in cardiovascular diseases

*Yong Zhang[1] ([1]*Harbin Medical University*)

Rationale: Our previous study has identified ZFAS1 as a new lncRNA biomarker of acute myocardial infarction (AMI). However, the regulatory roles of ZFAS1 in MI and myocardial ischemia/reperfusion injury (MIRI) need further exploration. Objective: To evaluate the effects of ZFAS1 on ion homeostasis and organelle function of cardiomyocytes in the setting of MI and MIRI. Methods and results: Part one: function and molecular mechanisms of ZFAS1 in MI.ZFAS1 expression was robustly increased in cytoplasm and sarcoplasmic reticulum in a mouse model of MI and a cellular model of hypoxia. Knockdown of endogenous ZFAS1 by virus-mediated silencing shRNA partially abrogated the ischemia-induced contractile dysfunction. Normal mice with overexpression of ZFAS1 and ZFAS1 cardiac-specific knock-in mice presented similar impairment of cardiac function as that observed in MI mice. Moreover, at the cellular level, ZFAS1 overexpression weakened the contractility of cardiac muscles and induced cardiomyocytes apoptosis. At the subcellular level, ZFAS1 deleteriously altered the $Ca^{2+}$ transient leading to intracellular $Ca^{2+}$ overload in cardiomyocytes, induced mitochondrial swelling and showed a pronounced decrease in mitochondrial membrane potential. At the molecular level, ZFAS1 was found to directly bind SERCA2a (sarcoplasmic reticulum $Ca^{2+}$-ATPase 2a) protein and to limit its activity, as well as to repress its expression, which contributed to $Ca^{2+}$ overload and activated the mitochondria apoptosis pathway subsequently. Notably, ZFAS1-FD (only functional domain) mimicked the effects of full-length ZFAS1 in regulation of cardiomyocyte function. Part two: function and molecular mechanisms of ZFAS1 in MIRI.The level of ZFAS1 was elevated in MIRI hearts, and artificial knockdown of ZFAS1 in mice improved cardiac function. Notch1 is a potential target of ZFAS1, and ZFAS1 could bind to the promoter region of Notch1 and recruit DNMT3b to induce Notch1 methylation. Nicotinamide mononucleotide could promote the expression of Notch1 by competitively inhibiting the expression of DNMT3b and improving the apoptosis of cardiomyocytes and cardiac function.Conclusions: Our study shows that ZFAS1 is a key regulator in MI and MIRI. As an endogenous SERCA2a inhibitor, ZFAS1 binds to SERCA2a protein to limit its intracellular level and inhibit its activity, which contributes to the impairment of cardiac contractile function and induction of mitochondria-mediated apoptosis in MI. Morover, in MIRI, ZFAS1 triggers cardiomyocytes apoptosis and ROS production by regulating DNA methylation of Notch1 promoter. Therefore, anti-ZFAS1 might be considered as a new therapeutic strategy for preserving cardiac function under pathological conditions of the heart.

### [1S03a-02]

### Impact of mitochondrial $Ca^{2+}$ dynamics on cardiomyocyte function

*Satoshi Matsuoka[1,2], Ayako Takeuchi[1,2], Yukari Takeda[1,2] ([1]*Department of Integrative and Systems Physiology, Faculty of Medical Sciences, University of Fukui,* [2]*Life Science Innovation Center, University of Fukui*)

Mitochondrial Ca2+ is one of regulators of mitochondrial NADH production, opening of mitochondrial permeability transition pore, and various cellular functions. In cardiomyocytes, mitochondrial Ca2+ is maintained mainly by the influx via Ca2+ uniport and the efflux via Na+-Ca2+ exchange (NCXmit). We previously demonstrated using HL-1 cardiomyocytes that NCXmit activity is associated with the Ca2+ content of sarcoplasmic reticulum (SR), and thereby modulates the frequency of spontaneous generation of action potential, automaticity. We recently revealed that NCXmit and sarco/endoplasmic reticulum Ca2+-ATPase (SERCA) are closely localized in mouse ventricular cardiomyocytes, and demonstrated with mathematical modelling that this spatial coupling enables functional Ca2+ coupling between mitochondria and SR. Furthermore, using mouse sinoatrial cells, we found that pharmacological inhibition of NCXmit reduced the amplitude, duration and size of local calcium release from SR and prolonged the cycle length of Ca2+ transient. It was suggested that the spatial and functional coupling of NCXmit and SERCA are pivotal in cardiomyocyte functions.

### [1S03a-03]

### Substrates dependent changes of mitochondrial function and its stimulation

Jeong Hoon Leem[1], Ji Yeon Song[1], Hajar Ibrahimi[1], Eun Seok Park[1], Jaer Kyung Song[1], Jae Boum Youm[2], *Chae-Hun Leem[1] ([1]*Department of Physiology University of Ulsan College of Medicine/Asan Medical Center,* [2]*Department of Physiology, College of Medicine Inje University*)

Mitochondria are essential organelles to support many cellular functions and the most important is the generation of ATP. The biochemical textbook explained this process orthodoxically to the link to the glycolysis. However, nowadays, for this essential role, the proper mitochondrial environment is required including substrates. In this regard, we are interested in the status of de-energized mitochondrial and what kind of factors can affect mitochondrial functions. Using laboratory-made multifactorial microspectrofluorometry system, we can monitor NADH, FAD, and the mitochondrial membrane potential ($\Delta\Psi m$) in a permeabilized cardiac myocytes of rats. The $\Delta\Psi m$ was measured in a quantitative way using out developed method. In this presentation, we would like to present three topics. Firstly, the characteristics of the de-energized mitochondria to pursue the basis of mitochondria bioenergetics. Secondly, the substrate dependent changes of mitochondrial function. Finally, the simulation-based analysis to pursue the mechanisms. From this report, we would like to show that the dogmatical understanding about mitochondrial substrates need to be corrected, the relatively simple treatment about the mitochondrial energization is not simple at all, and the mitochondrial homeostasis in an bioenergetical aspect need to be reconstructed. This work was supported by the Funding, R0005739, 2016M3C1A6936605, and 2014M3A9D7034366.

### [1S03a-04]

### Regulation of cardiac mitochondrial robustness by reactive sulfur species

*Motohiro Nishida[1,2] ([1]*Department of Physiology, Graduate School of Pharmaceutical Sciences, Kyushu University,* [2]*Division of Cardiocirculatory Signaling, National Institute for Physiological Sciences (Exploratory Research Center on Life and Living Systems), National Institutes of Natural Sciences*)

Electron transfer plays a pivotal role in maintaining cellular homeostasis, including energy metabolism, membrane potential formation, and quality controls of protein and organelles. Sulfur atom has a nucleophilic activity, while catenated sulfur molecules, called reactive sulfur species (RSS), have both nucleophilic and electrophilic activities. RSS are highly redox-active and responsible for the metabolism / elimination (stress adaptation) of the electrophiles, energy metabolism and membrane potential formation. We have reported that protein-bound RSS, such as cysteine persulfide (Cys-SSH) and cysteine polysulfide (Cys-$SS_nH$)) in proteins, participate in stress resistance of myocardium, through maintaining mitochondrial quality control by negatively regulating mitochondrial division-promoting GTP binding protein (Drp1) activity. We found that depolysulfidation of Drp1 at Cys644 induces mitochondrial hyperdivision, resulting in premature myocardial aging, which causes stress-resistant attenuation of the myocardium. Using sulfur-selective fluorescence imaging, RSS act as electron acceptors instead of oxygen in cells under hypoxic condition, resulting in the accumulation of sulfide catabolites such as $H_2S$ / $HS^-$ and reducd mitochondrial respiratory function. We thus suggest that suppressing sulfide catabolism under ischemia or fulfilling RSS after ischemia/reperfusion will become a new strategy to maintain mitochondrial quality and cardiac robustness.

### Symposium

### [1S04a]
### Physiological approach to elucidate the behavioral basis-Toward the next century of physiology-WPJ Sponsored Symposium

March 14 (Tue.), 14:20 - 16:20, Room 4

### [1S04a-01]
### Physiological changes during physical contact with caregivers in mammalian infants and their involvement in attachment formation

*Sachine Yoshida[1], Hiromasa Funato[1,2] ([1]*Faculty of Medicine, Toho University,* [2]*WPI-IIIS, Univ of Tsukuba*)

Previous studies on various young mammals, including human infants, have shown that physical contact with caregivers is essential for physical and mental development. However, little research has been conducted on how physical contact with caregivers affects infants immediately. We examined heart rate responses evaluated from R-R interval (RRI) in the first-year infants during a hug, a common parent-infant physical contact. We found that infants older than four months showed an increased RRI during a hug, indicating reduced heart rates and pronounced parasympathetic activity. Decision tree classification suggested that the RRI increase ratio can be predicted if both the infant's age and the head movement type immediately before hugging are known. Such a context-dependent RRI change was apparent in maternal and paternal hugs, but absent in infants hugged by female strangers. Physical contact with a caregiver, such as a hug, provides somatosensory inputs to infants. How these somatosensory inputs are processed in the infant's brain is still largely unknown. We are also studying the neural basis of this input using preweaning mouse pups. Preliminary results suggest that gentle strokes that mimic the dam's licking/grooming behavior reduce the stress response in the pups. Somatosensory input caused by intimate physical contact with caregivers may relax infants and promote attachment formation toward caregivers.

### [1S04a-02]
### Canine social cognition and behavior toward humans acquired through domestication.

*Miho Nagasawa[1] ([1]*Azabu University*)

Canines are the oldest domesticated animals and have supported human life in various roles, such as guard dogs, hunting dogs, herding dogs, and as companions. It is assumed that canines had already formed close relationships with humans before other domesticated animals appeared, but the process of domestication is still unclear. Recently, it has been hypothesized that canines were separated from wolves due to stress-responsive mutations and acquired human-like social cognitive abilities during the process of coexistence with humans. Based on this hypothesis, we found that canines and humans promote each other's oxytocin secretion by starting from the canine's gazing behavior toward its owner, and that the canine's gazing behavior toward its owner is controlled by oxytocin, suggesting that interspecies bonds via oxytocin nervous system can be formed like intraspecies bonds. It has been also found that canines are greatly stressed by separation from their owners and secrete tears when reunited with their owners. We would like to consider this extremely unique interspecies relationship, between canines and humans, from the perspective of the endocrine system.

### [1S04a-03]
### Auto and allo communication of sounds - echolocation behavior in bats

*Shizuko Hiryu[1] ([1]*Doshisha University*)

While many animals use visual information for navigation, the perceptual mechanisms of bats using auditory information have many biologically interesting features and also contain useful engineering insights. By comparing emitted ultrasounds with listening to returning echoes, bats can instantly recognize the physical characteristics of their surroundings, echolocation, which enable them to capture prey and avoid collisions with obstacles in complete darkness. On the other hand, most bats live in confined spaces such as caves, where they form colonies with other individuals. In order for bats to correctly sense their surroundings, they need to extract only their echoes from the complex mixture of ultrasounds belonging to neighboring conspecifics. If echolocation is defined as “auto communication” to one's own echoes, then “allo communication” to the sounds of other conspecific individuals is also a very important behavior for bats. In this presentation, I would like to introduce some experimental data obtained so far on the acoustic behavior of bats from the individual level to the group level and the results on how bats cooperate during flight and foraging with conspecifics.

### [1S04a-04]
### Emotions and subjective feelings change through joint emotional experiences with others

*Aiko Murata[1] ([1]*NTT Communication Science Laboratories*)

Many of us have experienced smiling when we see a friend’s happy face or shedding tears when we see that person cry. Our emotions are not only affected by external factors such as positive or negative events and internal conditions such as hormonal changes but are also influenced by the emotions of those around us. We conducted several experiments to investigate how a person’s emotions and subjective feelings are changed through interaction with others. In these experiments, participants had emotionally induced experiences together, and their physiological responses (e.g., blood volume pulse, and heart rate) were recorded to assess changes in autonomic responses during these experiences. Our findings suggest that physiological responses tend to converge among pairs due to face-to-face interactions and that the strength of physiological synchrony is related to the intensity of subjective feelings. Furthermore, we also found that physiological synchrony between members is gradually strengthened throughout the group performance. That is, emotions, when shared by multiple individuals, have the potential to alter the intensity of the emotion itself, subjective feeling, and group performance. These findings will help deepen understanding of the psychophysiological mechanisms underlying larger group phenomena such as crowd frenzy and group cohesion. In this talk, I would like to report the findings of our experimental studies and link them to a discussion of the social process of emotion.

### [1S04a-05]
### Conscious and subconscious neural processing of self

*Tamami Nakano[1] ([1]*Graduate School of Frontiers Bioscience, Osaka University*)

Our brains have created the concept of self in order to distinguish ourselves and act in our environment. However, the neural system that generates the self remains unclear. We found that the salience network is involved in self-recognition and the activity of it dramatically change in association with deformation of self-images. Furthermore, the saliency network consistently showed activation in relation to self-recognition, even when the viewpoint of the self was changed. Next, we measured brain activity using fMRI while the participants were subliminally presented their own images. The nucleus accumbens, the center of the dopamine reward system, showed strong activity for their own images, while the amygdala showed strong activity to the images of others. We further found that people tend to evaluate same-sex faces that resemble their own faces as more trustable. These findings suggest that we select optimal behaviors that enhance our own survival in the environment by activating the reward system to preferentially collect information relevant to the self and integrating various information relevant to the self in the cerebral cortex.

### Symposium

[1AS05a]
Committee for Young Physiologists
**Discussion for the future of physiological sciences**

March 14 (Tue.), 14:20 - 16:20, Room 5

### [1AS05a-01]
### Discussion for the future of physiological sciences

*Daisuke Yamada[1], Yuki Suda[2] ([1]*Laboratory of Pharmacology, Faculty of Pharmaceutical Sciences, Tokyo University of Science,* [2]*Department of Integrative Physiology, Graduate School of Medicine, University of Yamanashi*)

With the declining birthrate and aging population, there is a strong demand for the development of young people who will lead the future of Japan. In the academic field as well, many academic societies are trying to encourage young researchers, but in principle, they often targeted undergraduate students and above. On the other hand, we often hear undergraduate students say that they had an interest in scientific research and motivation to become a scientist even before entering university and their motivation to become a scientist was stronger before entering university. Therefore, in this symposium, we will invite high school students and undergraduate students and try to discuss the current issues and future direction of physiological science in Japan with members of the PSJ youth group. By holding it as a convention plan, not as a regular event sponsored by the PSJ youth group, we would like to provide the young people with the experience of attending an academic conference and create a starting point for revitalizing the PSJ.

### Symposium

[1S06a]
Cooperation with Other Societies Committee
**New Era of Sensory Neuroscience**

March 14 (Tue.), 14:20 - 16:20, Room 6

### [1S06a-01]

### Sound detection and processing in the cochlea of the inner ear

*Takeru Ota[1], Hiroshi Hibino[1] ([1]*Division of Glocal Pharmacology, Department of Pharmacology, Graduate School of Medicine, Osaka University*)

A cochlea receives the middle ear oscillation induced by sounds. The stimulation evokes nanometer-scale motions in the sensory epithelium which contains hair cells. The cells expose their hair bundles to endolymph, the extracellular solution characterized by high [$K^+$]. The epithelium vibration changes the opening of mechanosensitive channels on the bundles and modulates the ion entering from the fluid. Inner hair cells release neurotransmitters to the auditory nerves and outer hair cells shrink and elongate their soma depending on the receptor potentials. The electromotive response amplifies the vibration of the sensory epithelium and contributes to the faint sound sensitivity and sharp frequency selectivity. These physiologically essential functions are boosted by +80 mV environment in the endolymph. The high potential comes from the battery operation of stria vascularis in the lateral cochlear wall. With developed technique, we observed the sound-evoked vibrations in the sensory epithelium and the response of the vascularis. The results suggest that the process of sounds is based on the loop networking formed by the hair cells' active controlling of sensory epithelium and by the stria vascularis.

### [1S06a-02]

### Cellular and molecular mechanisms underlying airway protective reflexes

*Akiyuki Taruno[1,2] ([1]*Department of Molecular Cell Physiology, Kyoto Prefectural University of Medicine,* [2]*JST-CREST*)

Airway protection is an important function of the throat, a crossroad of the food and air ways, where complex reflex systems against diverse chemical and physical stimuli are at work to avoid suffocation. Although free vagal nerve endings have been supposed to serve as multimodal sensors, full understanding of the identity and mechanisms of peripheral sensors in the throat remains to be achieved. Here we show a role for sensory epithelial cells in the throat as sensors of vagal airway protective reflexes. In mice, a whole-body search for a purinergic, synaptic vesicle-independent neurotransmission machinery, termed channel synapse, discovered rare epithelial cell types that express diverse G protein-coupled receptors and communicate with the vagal nerve via channel synapses. Upon activation by receptor agonists, they trigger airway protective reflexes such as swallowing. Dysfunction of the channel synapse abolish the responses. These findings broaden our mechanistic understanding of airway protection programs.

### [1S06a-03]

### In vivo imaging of odor coding in the peripheral olfactory system

*Takeshi Imai[1] ([1]*Kyushu University*)

Olfactory perception starts at the olfactory sensory neurons (OSNs) in the olfactory epithelium. However, it has not been fully understood how odors are perceived and processed at the most peripheral level in the physiological conditions in vivo. To address this issue, we established two-photon calcium imaging of OSNs in the olfactory epithelium in freely breathing conditions. Firstly, we examined how the mixture of odors, which is natural in the environment, is perceived in OSNs. We found that non-agonist odors often suppress or enhance the OSN responses to the agonist. As for the enhancement, non-agonists demonstrated an upward or leftward shift of dose-response curves of agonists, suggesting allosteric interactions at the level of odorant receptors. Furthermore, this allosteric modulation was found for specific sets of odorants for different OSNs. Thus, the combinatorial ligand-receptor interactions at both orthosteric and allosteric sites shape the OSN responses. Secondly, we addressed the mechanisms of olfactory adaptation in the olfactory system. Olfactory adaptation is well known in human psychophysics studies but has not been fully investigated where and how this is happening. We found that minutes-long adaptation occurs in OSNs. We also found that long-lasting stimulation of a subset of OSNs suppressed odor responses in all OSNs. This result raises the possibility of the non-cell autonomous mechanism for olfactory adaptation. Together, in vivo recording revealed previously unappreciated roles of peripheral modulation for odor perception.

### [1S06a-04]

### Unraveling a novel mechanism of somatosensory information processing by spinal astrocytes

*Yuta Kohro[1], Tsuyoshi Matsuda[1], Kohei Yoshihara[1], Makoto Tsuda[1] ([1]*Dept. Mol. Syst. Pharmacol., Grad. Sch. Pharm. Sci., Kyushu Univ.*)

Somatosensory information from the periphery is conveyed to the spinal dorsal horn (SDH) through the primary afferents. The information is processed by the neuronal circuit in the SDH and then relayed to the brain. In addition, spinal sensory transmission is also modulated by the top-down pathway. The descending noradrenergic (NAergic) pathway from the locus coeruleus (LC) to the SDH is well known as an endogenous pain inhibition system. Recently, we found that a population of the SDH astrocytes, which express hairy and enhancer of split 5 (Hes5), were activated by descending LC-NAergic signals after peripheral noxious stimuli. Interestingly, this pathway activated by noxious stimuli induced mechanical hypersensitivity through Hes5 astrocytes in the SDH. Furthermore, the inhibition of NAergic signaling in Hes5 astrocytes enhanced the analgesic effect of duloxetine (which increases spinal NA levels by inhibiting its transporters at descending NAergic terminals) on chronic pain. These findings reveal a novel role of NAergic signaling in somatosensory information processing.

### Symposium

### [1S07a]
### Neurobiology of addiction science

March 14 (Tue.), 14:20 - 16:20, Room 7

### [1S07a-01]
### Neurotoxicity of cannabinoids and Cannabis Control Law

*Yuko Sekino[1] ([1]*Graduate School of Agricultural and Life Sciences, the University of Tokyo*)

Cannabinoids are a group of substances found in the cannabis plants (Marijuana). Marijuana can lead to a marijuana use disorder which are often associated with 'addiction'. Tetrahydrocannabinol (THC) and cannabidiol (CBD) are the main cannabinoids of marijuana, and THC is the main psychotropic component. While Epidiolex made from CBD has been approved by the FDA, unregulated CBD products are subject to strict regulation because contamination of THC might harm the human brain. Regular cannabinoids use particularly during infancy and adolescence is known to take the risk of long-lasting neurobiological changes in the adult brain. Here we examined the chronic effects of the CB1/CB2 receptor agonist CP55940 on dendritic spine formation. Hippocampal neurons in low density culture were exposed to various concentrations of CP55940 from DIV7 to DIV21, DIV14 to DIV21, and DIV21 to DIV28. Neuronal cell death was observed when 10 μM of CP55940 was applied from DIV7 and 14, and but not from DIV21. Low concentrations of CP55940 significantly altered the ratio of high concentration drebrin clusters and increased the number of drebrin clusters per dendrite length. Identification of drebrin clusters and analysis of their number and distribution using hippocampal neuronal cultures is promising for cannabis risk detection. Supported by a grant from LRI of JCIA and the Health and Labour Science Research grant.

### [1S07a-02]
### Addiction-like behaviors in laboratory animals

*Soichiro Ide[1], Kazutaka Ikeda[1] ([1]*Tokyo Metropolitan Institute of Medical Science*)

In recent years, various problems of addiction have become prevalent in the world, and the situation has become more serious. While it is very important to uncover the mechanisms underlying addiction, there are still many things that remain unknown. Animal studies have been crucial in understanding the biology and pathophysiology of drug addiction. Currently, there are several animal behavioral models of addiction using rodents and monkeys. Each model evaluates a different aspect of addiction, and each provides important insights. On the other hand, these procedures take many times to evaluate the addiction. Addictive substances are diversified, and it is required to establish a more accurate and quicker evaluation system for their risk assessment. The nematode *Caenorhabditis elegans* (*C. elegans*) is an excellent invertebrate model to study neurobiological disease states. Recently, it was also proposed that *C. elegans* can be used to model aspects of drug addiction. We also demonstrated that morphine caused addiction-like behaviors in *C. elegans*. Consistent responses to addictive drugs across phyla lead us to hypothesize that *C. elegans* may be a useful model system to study addiction processes and screen potential treatments for substance use disorders.

### [1S07a-03]
### Understanding the neuronal mechanisms of behavioral addiction by quantifying the motivation for wheel-running in mice

*Naoya Nishitani[1], Katsuyuki Kaneda[1] ([1]*Lab. Mol. Pharmacol., Inst. Med., Pharmaceut., Health Sci., Kanazawa Univ.*)

In behavioral addiction, addicts repeat certain behaviors, such as internet gaming and gambling, despite negative consequences. No cure has been established due to the lack of animal models to explore neuronal mechanisms of behavioral addiction. Here, by focusing on voluntary wheel-running in rodents, we have developed a novel operant conditioning task as a model of behavioral addiction, in which male C57BL/6J mice were allowed for wheel-running after certain numbers of nose pokes (NPs), a measure of motivation for wheel-running. We first monitored dopamine (DA) release using fiber photometry with GRAB-DA sensors and found that DA was increased in the nucleus accumbens (NAc) immediately after the last NP in the fixed ratio 10 schedule, by which mice recognized the availability of wheel-running. Systemic administration of antagonists for DA D1 receptor, D2 receptor, or an agonist for adenosine A2A receptor dose-dependently decreased the number of NPs. Intra-NAc infusion of these drugs also reduced the number of NPs. Additionally, systemic administration of an antagonist for serotonin (5-HT)2A or 5-HT2C dose-dependently decreased the number of NPs. These results suggest that neurotransmission via D1, D2, and A2A receptors in the NAc and 5-HT2A and 2C receptors are involved in the motivation for wheel-running.

### [1S07a-04]
### Cell-type-specific control of reward and aversive signaling in the Nucleus Accumbens

*Tom Macpherson[1], Suthinee Attachaipanich[1], Tadaaki Nishioka[2], Takaaki Ozawa[1], Takatoshi Hikida[1] , ([1]*Institute for Protein Research, Osaka University, Osaka, Japan,* [2]*Icahn School of Medicine at Mount Sinai, New York, USA*)

The nucleus accumbens (NAc) is a key brain structure within a basal ganglia neurocircuit implicated in limbic processing and decision-making; however, the precise NAc cell types and circuits that control reward and aversive learning are still unclear. Here we use miniature microscope-based in-vivo calcium imaging to record the neural activity of the NAc's two main neuron types, dopamine D1 or D2 receptor-expressing medium spiny neurons (D1/D2-MSNs), during performance of a Pavlovian conditioning task in which mice learned to associate previously neutral auditory tones (conditioned stimulus (CS)) with the delivery of rewarding (sucrose) and aversive (airpuff) unconditioned stimuli (US). We revealed the existence of several subpopulations of NAc neurons that were activated or inhibited by the delivery of rewarding or aversive stimuli. Interestingly, many of these subpopulations comprised both D1- and D2-MSNs, which appear to act collaboratively to signal a specific type of CS or US. Our findings suggest remarkable heterogeneity in the responses of NAc core D1- and D2-MSNs and indicate that the previous roles ascribed to these neurons (uniformly controlling reward and aversive signaling, respectively) have been oversimplified. This new understanding of the precise roles of specific NAc subpopulations provides novel insight into reward and aversion learning in the brain and will likely help to better understand how dysfunction of such learning can contribute to pathological conditions including addiction.

### [1S07a-05]
### Neural mechanisms for flexible risk-return decision making

*Ryo Sasaki[1] ([1]*Kyoto University*)

Humans encounter situations where they are forced to choose an action with either high-risk but high-return (HH; risky choice) or low-risk and low-return (LL; safer choice). Pathological decision-making is known to underlie some symptoms of neuropsychiatric disorders, such as gambling disorder. Then, what is the neural mechanisms to flexibly take the risky-safer choices depending on situations? To answer this question, we first trained macaque monkeys HH-LL decision-making task where they were required to handle risky choices to get reward. We then implemented the systematic mapping in the prefrontal cortices (PFCs) by the optogenetic activation of their inputs from ventral tegmental area (VTA). The terminals of the VTA-PFC pathway were optogenetically activated with high dense LED arrays while the monkeys were performing HH-LL decision. We found that the selective optogenetical activation of the pathway from VTA to ventrolateral prefrontal cortex (vlPFC) facilitated risky choices without affecting the expected value-dependency. Interestingly, we also found the optogenetic activation of the pathway from VTA to dorsolateral prefrontal cortex (dlPFC) facilitated safer choices (reduced risky choices) without affecting the expected value-dependency. These findings suggest that the pathway specific control in VTA-PFC for flexible risk-return decision making. It may contribute to develop therapeutic methods in the clinical field by elucidating the neural mechanisms of neuropsychiatric disorders.

### Symposium

[1S08a]
**A challenge from physiology for exercise medicine: scientific evidence and its social implementation**

March 14 (Tue.), 14:20 - 16:20, Room 8

### [1S08a-01]

### Exercise Training in Cardiovascular Disease Patients with Physical Deconditioning

*Qi Fu[1] ([1]*Institute for Exercise and Environmental Medicine, UT Southwestern Medical Center*)

A sedentary lifestyle or physical inactivity is one of the most important modifiable risk factors for cardiovascular morbidity and mortality in humans. Increased physical activity or exercise training is necessary to maintain overall health and functional capacity, and it plays a crucial role in the prevention and/or treatment of hypertension, heart failure, myocardial infarction, and even sudden cardiac death. In addition, exercise training (endurance combined with resistance training) has been proven to be effective for the treatment of postural orthostatic tachycardia syndrome (POTS) – a multifactorial disorder with cardiovascular deconditioning (i.e., cardiac atrophy and hypovolemia) as a common feature or a final common pathway. Adaptations involving the autonomic nervous system and the cardiovascular system play a large role in the protective and therapeutic effects of exercise training in the patient population, especially patients with physical deconditioning. Indeed, numerous studies have demonstrated that exercise training improves vagal modulation, decreases sympathetic tone, augments baroreflex sensitivity and increases heart rate variability, all of which could reduce cardiovascular risk and improve clinical outcomes. Furthermore, training-induced improvement in arterial stiffness and endothelial function, cardiac remodeling, and blood volume expansion may also contribute to the protection and treatment of cardiovascular disease and POTS. Moderate-intensity exercise at least 30 minutes per day and at least 5 days per week is recommended for the vast majority of people. Supervised exercise training is preferable to maximize function capacity and may be particularly important for cardiovascular disease patients with significant physical deconditioning.

### [1S08a-02]

### Metformin and physical activity: a pharma-physiological interaction?

*Kristian Karstoft[1,2] ([1]*Department of Clinical Pharmacology, Bispebjerg-Frederiksberg Hospital, Denmark,* [2]*Centre for Physical Activity Research, Rigshospitalet, Denmark*)

Metformin and exercise training both improve glycemic control and other cardiovascular risk factors in glucose-intolerant individuals, when prescribed independently. Several studies, however, suggest that an interaction between metformin and exercise does occur, leading to, among other things, metformin blunting or even antagonizing the exercise training-induced improvements in insulin sensitivity. A metformin-exercise interaction would be of great clinical relevance given that metformin and exercise training are both part of the first line treatment of type 2 diabetes. This lecture will give an overview of the literature on the potential interaction between metformin and exercise. The focus will be on clinically relevant data obtained in human studies and will discuss the type and extent of the interaction.

### [1S08a-03]

### Molecular Mechanisms of Exercise Training Effects

*Keiichi Higuchi[1] ([1]*Nagano University of Health and Medicine*)

Exercise training is beneficial for reducing the risk of age-related diseases, including amyloidosis, but the underlying molecular links remain unclear. Here, we investigated the protective role of interval exercise training in a mouse model of age-related systemic amyloidosis and identified potential molecular mechanisms. Mice subjected to 16 weeks of exercise training promoted whole-body physiologic functions and inhibited the progression of amyloidosis. Exercise training activated the hepatic p38 mitogen-activated protein kinase (p38 MAPK) signaling pathway and the downstream transcription factor tumor suppressor p53. This activation resulted in elevated expression and phosphorylation of heat shock protein beta-1 (HSPB1), a chaperone that defends against protein aggregation. Exercise training also increased the primary regulator of mitochondrial function PGC-1α. In amyloidosis-induced mice, exercise training additively enhanced the hepatic p38 MAPK-related adaptive responses induced by amyloid deposition. As a result, we observed more significant amounts of phosphorylated HSPB1 with exercise, which we suspect inhibits amyloid deposition. We suggest that exercise training may amplify intracellular stress-related protective adaptation pathways against age-associated disorders like amyloidosis.

### [1S08a-04]

### Scientific evidence and social implementation of interval walking training: a challenge for exercise medicine

*Shizue Masuki[1,3,4], Mayuko Morikawa[1,3,4], Hiroshi Nose[2,4] ([1]*Sports Medical Sciences,* [2]*e-Health Sciences, Shinshu University Graduate School of Medicine,* [3]*Institute for Biomedical Sciences, Shinshu University,* [4]*Jukunen Taiikudaigaku Research Center*)

The rapid rise in healthcare costs with rapidly growing elderly populations has highlighted the importance of exercise training for therapeutic as well as preventive medicine. However, exercise training programs that guarantee effectiveness in the field are not widely available. Against this problem, we have developed an exercise training system which comprises three elements: 1) interval walking training (IWT), repeating fast and slow walking for 3 min each, equivalent to >70% and ~40% peak aerobic capacity ($VO_{2peak}$), respectively; 2) an original portable calorimeter equipped with a tri-axial accelerometer and a barometer, measuring energy expenditure during IWT; and 3) an IoT system that enables users to receive instructions from trainers according to their walking records. Using this system, we examined the effects of 5-mo IWT in more than 9,600 middle-aged and older people, and found that the training increased $VO_{2peak}$ by 15%, improved lifestyle-related disease (LSD) symptoms by 20%, and reduced healthcare costs by 20% on average. Recently, we also developed a mobile application program to provide participants with this service on their smartphone. We confirmed that 5-mo IWT using the new system was as effective as that of our ordinary system in university students, company workers, older people, and LSD patients. Thus, the new system has great potential for implementation of IWT for clinical as well as preventive medicine to establish and promote a society for health and longevity.

### Symposium

### [1S09a]
### Circadian Rhythm in Health and Disease

March 14 (Tue.), 14:20 - 16:20, Room 9

### [1S09a-01]
### Roles of $Ca^{2+}$ Signaling in Generation of Circadian Rhythms

*Naohiro Kon[1] ([1]*Institute of Transformative Bio-Molecules (ITbM), Nagoya University*)

$Ca^{2+}$ is the most important intracellular second messenger in physiology and pathology. In studies of mammalian circadian rhythms, dynamics and functions of $Ca^{2+}$ signaling have been studied intensively in the central pacemaker SCN neurons. First, role of $Ca^{2+}$ signaling in photic input pathway was clarified in the SCN, and $Ca^{2+}$ activates transcription of *Period1/2* genes in response to light illumination during night period. Then, clear circadian rhythms of intracellular $Ca^{2+}$ levels were reported in the SCN (Ikeda *et al*., Neuron, 2003), and $Ca^{2+}$ influx is shown to be essential for oscillation of transcriptional and translational feedback loops (TTFL) of clock genes (Lundkvist *et al*., J. Neurosci, 2005). Our group identified $Ca^{2+}$/calmodulin-dependent protein kinase II (CaMKII) and $Na^{+}/Ca^{2+}$ exchanger (NCX) as essential mediators of circadian $Ca^{2+}$ signaling both in the SCN and peripheral cellular clocks (Kon *et al*., *Genes and Development,* 2014; Kon *et al*., *Science Advances*, 2021). Importantly, $Ca^{2+}$ rhythm is observed in the SCN from mice lacking *Cry1* and *Cry2* (Enoki *et al*., Scientific Reports, 2017), showing that circadian $Ca^{2+}$ oscillation is independent of the TTFL. In addition, roles of $Ca^{2+}$ in circadian clock are well conserved among mammals, insects, fungi, plants and bacteria, whereas homologies of transcriptional factors for the TTFL are limited among the organisms. These evidences indicate that circadian $Ca^{2+}$ oscillator is an original time keeping system inherited from a common ancestor of the organisms.

### [1S09a-02]
### Recapitulation of pre-symptomatic pathophysiology in chronic jet-lag mouse cohort model

*Nobuya Koike[1], Yasuhiro Yasuhiro[1], Kazuhiro Yagita[1] ([1]*Department of Physiology and Systems Bioscience, Kyoto Prefectural University of Medicine*)

Circadian misalignment is a significant risk factor for various diseases in human and mouse models. Numerous epidemiological and genetic studies have illustrated the pathophysiological consequences of circadian misalignment. For example, in a recent study, we showed that when subjected to a chronic jet-lag (CJL) paradigm, wild-type C57BL/6J mice exhibited a significantly shorter lifespan strongly accompanied by non-alcoholic steatohepatitis (NASH)-like low-grade chronic inflammation and immune senescence. Despite our growing understanding of the adverse events occurring at the disease stage, the pre-symptomatic state in age-related chronic diseases induced by circadian misalignment remains poorly characterized, hindering the development of preventive approaches. Here, we set out to recapitulate the pre-symptomatic process for circadian misalignment-induced diseases in mice. Utilizing an integrated pipeline of physiological, transcriptional and histological phenotyping in individual wild-type C57BL/6J mice exposed to CJL, we illustrate the pathological progression during the pre-symptomatic state prior to transitioning to the disease state during chronic environmental misalignment.

### [1S09a-03]
### Establishment of a model system for analyzing circadian rhythms in diurnal non-human primates

*Keiko Tominaga[1], Hitoshi Okamura[2] ([1]*Graduate School of Frontier Biosciences, Osaka University,* [2]*Graduate School of Medicine, Kyoto University*)

Circadian rhythms in behaviors and internal physiological phenomena are observed in humans and other living organisms on the earth. The internal machinery that controls the circadian rhythms is a circadian clock, which is susceptible to environmental lighting conditions. In humans, nocturnal lifestyles, lighting at night disturb the circadian clock, increasing the risk of developing lifestyle-related diseases and mental disorders. In humans and diurnal animals, sleep-wake cycles are differently regulated from nocturnal rodents. Therefore, research using diurnal animals is valid to understand the role of the circadian clock on sleep-wake rhythms and how to manage its disruptions in humans. We introduce our recent research on circadian rhythms in the common marmoset (*Callithrix jacchus*), a diurnal non-human primate. Using an activity ring in the neck and an EEG electrode on the scull, whose data are wirelessly collected, we observed precise activity rhythms and the relationship between activity rhythms and EEG properties under free-moving conditions. We observed synchronization of activity rhythms between individuals using social interactions similar to humans, which we had never observed in rodents. Therefore, we hope that circadian analyses in common marmosets may help to understand the physiology and pathology of the human circadian system.

### [1S09a-04]
### Phase adjustment mechanism of the human sleep-wake cycle and circadian rhythms

*Yujiro Yamanaka[1,2], Sato Honma[3,4], Ken-ichi Honma[3,4] ([1]*Laboratory of Life & Health Sciences, Graduate School of Education and Faculty of Education, Hokkaido University,* [2]*Research and Education Center for Brain Science,* [3]*Department of Chronomedicine, Graduate School of Medicine, Hokkaido University,* [4]*Center for Sleep and Biological Rhythm Disorders, Sapporo Hanazono Hospital*)

The circadian system in mammals consists of the central circadian pacemaker in the hypothalamic suprachiasmatic nucleus (SCN) and the peripheral clocks in various organs and outside the SCN brain regions. From the results in nocturnal rodents, the SCN circadian pacemaker entrains to a light-dark cycle, whereas circadian rhythms of peripheral organs and extra-SCN brain regions entrain to non-photic zeitgebers such as timed physical exercise and restricted daily feeding. From the results of experiments under the human isolation experiment, we reported that scheduled physical exercise under dim light could be a non-photic zeitgeber for the sleep-wake cycle but not for the circadian rhythm of plasma melatonin. Regarding the effect of a restricted feeding schedule on the sleep-wake cycle and human circadian rhythms, we recently examined the effect of a fixed single meal schedule on the sleep-wake cycle and circadian rhythms under free-running conditions. In this study, we assessed the entrainability of a single fixed meal per day for the human circadian system and the development of prefeeding peaks in plasma hormones. The result indicated that a fixed single meal schedule prevented the free-running of the sleep-wake cycle but not of the circadian pacemaker. In contrast, a daily single ad-libitum meal failed to prevent the free-running of the sleep-wake cycle and circadian rhythms of plasma melatonin. These findings suggest that timed physical exercise and meal schedules could be a non-photic zeitgeber for the sleep-wake cycle. These findings support the two-oscillator model of the human circadian system.

### [1S09a-05]
### Entrainment of human circadian rhythms and its disturbances: conflict between theory and practice in the treatment of circadian sleep-wake disorders

*Sato Honma[1] ([1]*Sapporo Hanazono Hospital*)

Much information has been accumulated concerning molecular and cellular mechanisms of the circadian clock, but that of the human circadian clock is still limited. Circadian rhythm sleep-wake disorders (CRSWD) due to the mutation in clock and clock-controlled genes have also been reported in humans. Intrinsic period of human circadian clock is about 25 h and environmental light entrains human circadian clock with similar phase-responses to that of other living organisms. CRSWD is not difficult to diagnose, and treatment strategy is established. Nevertheless, treatment of CRSWD is difficult because the strategy based on animal experiments is not always applicable to human circadian system. Importantly, the two oscillators regulating light-entrainable rhythms and sleep-wake rhythm could be desynchronized in humans. Since sleep-wake rhythm is entrained not by light but by social cues, CRSWD patients often have neurodevelopmental disorders who have difficulties in social relations. Social time cue is critically involved in the treatment of CRSWD.

### Symposium

[2AS01m]
**The Future of Science Driven by Cross-Disciplinary Projects**

March 15 (Wed.), 9:00 - 11:00, Room 1

### [2AS01m-01]

### Mission and Progress of Tohoku Medical Megabank Project

*Masayuki Yamamoto[1] ([1]*Tohoku University Tohoku Medical Megabank Organization*)

The Tohoku Medical Megabank Project (TMM) has been launched to accomplish creative reconstruction in the aftermath of the Great East Japan Earthquake and ensuing tsunami on March 11, 2011. TMM aims to establish an integrated biobank based on two prospective large-scale cohort studies; a population-based cohort study and a birth and three-generation cohort study, which in combination have recruited more than 157,000 participants. Two prospective cohorts of TMM show in combination a highly unique and strategical design. Collected information and samples are stored to the TMM Biobank system, including the large-scale sample storage system and the dbTMM database system. TMM also conducts genome and omics analyses of the collected cohort samples. The integrated biobank of TMM storages both bio-specimens and genome-omics big data generated in-house. TMM will share both information data and samples with the research community to facilitate biomedical research and personalized health care. TMM believes that constructing the integrated biobank by way of large-scale genome cohort studies will be effective in establishing the personalized health care and medicine.

### [2AS01m-02]

### Innovations in Medicine and Life Sciences through Creation of Quantum Life Science

*Yoshinobu Baba[1,2] ([1]*Nagoya University,* [2]*QST*)

Based on the major developments in biology and quantum science and technology, the National Institute for Quantum Science and Technology (QST) has launched the Institute for Quantum Life Science in 2019 in order to combine quantum science and life science. In 2020, the Institute for Quantum Life Science established the collaborating research team between universities, national institutes, and companies to start the MEXT Quantum Leap Flagship Program (Q-LEAP) "Innovations in Medicine and Life Sciences through Development of Quantum Life Technology", developing Biological Nano Quantum Sensors, Quantum Technology Based MRI/NMR, and Quantum Biology and Biotechnology. Full-scale research and development of quantum life science has been launched, with researchers from quantum science and technology to biology and medicine conducting close collaboration. Here, I would like to introduce the current research activities of quantum life science that is being promoted in the MEXT Q-LEAP project [1-5]. [1] *Sci. Adv.* (2022) eabl7002. [2] *Sci. Adv.* 7 (2021) eabd7888. [3] *Nat. Ener.*6 (2021) 1176. [4] *Sci. Adv.*, (2022) 10.1126/sciadv.abj2667. [5] *Nat. Commun.,* 12 (2021) 3062.

### [2AS01m-03]

### NanoTerasu: 3GeV Synchrotron Radiation Facility of Japan; - Building a New Range of Medical & Biological Science via a unique collaboration between industry and Academy on funding and utilization -

*Masaki TakataTakata[1] ([1]*Tohoku University*)

synchrotron radiation (SR) is recognized as an essential research tool for the development of science and technology as well as for industrial applications. A number of advanced SR facilities have been constructed worldwide. This also mirrors society's strong demand for public health problems and achieving sustainable development goals (SDGs). SR facilities worldwide now work together to study, understand and solve the COVID-19 pandemic, including new drugs, therapeutic strategies, and medical equipment developments. At SR facilities, powerful X-rays with unprecedented brightness are increasingly becoming available because of recent technical advances in low-emittance ring accelerators. Nanoterasu based on this technology will revolutionize our ability to investigate with nanometer resolution, chemical specificity, etc. with high-brilliance soft X-rays, which will provide unique analytical capability in the visualization of the state inside cells not only the protein structure analysis. The prospect of NanoTerasu application in Medical & Biological Science, to expand to drug discovery research such as pharmacology and pharmacokinetics will be presented.

### [2AS01m-04]

### ERATO Ueda Biological Timing Project: Toward Human Sysmtems Biology

*Yoichi Minami[1], Hiroki. R Ueda[1,2] ([1]*The University of Tokyo, Graduate School of Medicine, Department of Systems Pharmacology,* [2]*Laboratory for Synthetic Biology, RIKEN Center for Biosystems Dynamics Research*)

In the ERATO Ueda Biological Timing project, we will develop human systems biology using sleep-wake rhythms as a model system. Our challenge is to understand the "biological timing" information of sleep and wakefulness from the molecular level to the individual level.Recently, increasing numbers of people dissatisfied with sleep have increased awareness of "healthy sleep". In October 2020, we started an experiment to measure human sleep on a large scale as part of the ERATO Ueda Biological Timing Project. We have established an algorithm of defining sleep and wakefulness using accelerometer data, named ACCEL, with high accuracy, sensitivity, and specificity (Ode, Shi, Katori, et al., iScience, 2022). Using this algorithm, we analyzed sleep-wake profiles from acceleration data of approximately 100,000 adults registered in the UK Biobank and succeeded in drawing a sleep landscape (Katori, Shi, et al, PNAS, 2022). In October 2022, we further started the "Children's Sleep Health Checkup" Project. In this symposium, we will introduce our project and our attempts to connect laboratory results to society.

### Symposium

[2S02m]

**Current perspective of hibernation and torpor in mammals**

March 15 (Wed.), 9:00 - 11:00, Room 2

### [2S02m-01]

### Autorecovery from hibernation period in Syrian hamsters involves a resetting of circadian body temperature rhythms

*Yoshifumi Yamaguchi[1,2,3] ([1]*ILTS, Hokkaido Univ.*, [2]*Grad. Sch. Env. Sci., Hokkaido Univ.*, [3]*InaRIS*)

Hibernation metabolism, physiology, and development group, Institute of Low Temperature Science, Hokkaido University Mammalian hibernation consists of multiday hypothermic deep torpor and normothermic periodic arousal. Deep torpor is characterized by the profound suppression of metabolism, body temperature, heart rate, and locomotive activity. Hibernating animals repeat "torpor-arousal cycle" for several months of hibernation period and then quit hibernation even under a constant cold, short photoperiod condition. Mechanisms of the "torpor-arousal cycle" and such autorecovery from hibernation period are still poorly understood. Syrian hamster (*Mesocricetus auratus*) is a facultative hibernator, in that they are induced to hibernate when chronically exposed to cold, short photoperiod condition similar to winter. In this species, the set-point of core body temperature (Tb) decreases by the onset of hibernation, then gradually increases during the repetition of torpor-arousal cycle, and finally recovers to the level observed before the exposure to the winter-like condition at post-hibernation period. Here we analyzed the pattern of Tb fluctuation throughout all hibernation events . When hamsters were transferred to a winter-like (4ºC , L:D=8:16) condition from a summer-like condition (23ºC, L:D = 14:10), they exhibited a gradual adaptation of circadian Tb fluctuation rhythm to the winter-like condition. Once hibernation began, no clear pattern of circadian Tb fluctuation was detected. Unexpectedly, we found that circadian Tb fluctuation rhythm similar to that observed in a summer-like condition emerged at post-hibernation period while the animals were still kept in a winter-like condition. This resetting of circadian Tb fluctuation rhythm was not observed in animals that never hibernated in the winter-like condition. These data suggest that autorecovery from hibernation period in Syrian hamsters involves unknown mechanism that resets circadian Tb fluctuation rhythm for adaptation to a predicted summer-like, long photoperiod condition.

### [2S02m-02]

### Circadian Rhythms under Cold Temperatures in the Master Clock Neurons

*Ryosuke Enoki[1], Naohiro Kon[2], Yoshifumi Yamaguchi[3], Tomomi Nemoto[1] ([1]*NINS, ExCELLS/NIPS*, [2]*Nagoya University, ITbM*, [3]*Hokkaido University, ILTS*)

Mammalian circadian rhythms are coordinated by the master clock located in the hypothalamic suprachiasmatic nucleus (SCN). Under severe environmental conditions, such as during the harsh winter season for food, certain mammalian species reduce their basal metabolism and thermogenesis, thereby undergoing torpor, a controlled state of hypothermia, which naturally returns to the normothermic state. A long-lasting debate focused on whether the SCN with a temperature-compensated clock remains functional during hypothermia. However, so far, no direct and quantitative evidence has been reported of temperature sensitivity in living SCN neurons. Toward this goal, we performed dual-color fluorescence imaging of clock gene transcriptions and intracellular $Ca^{2+}$ in mouse SCN neurons, using slices at various temperatures. We found that SCN neurons remain functional under moderate hypothermic conditions at approximately 22°C–28°C but stop ticking time in deep hypothermia at 15°C and that the rhythms reset after deep hypothermia. Our data also indicate that the stable $Ca^{2+}$ oscillation precedes clock gene transcriptional rhythms in the SCN neurons.

### [2S02m-03]

### Cellular, Molecular, and Physiological Adaptations of Hibernation

*Elena O Gracheva[1] ([1]*Yale University School of Medicine*)

Mammalian hibernation is fascinating. During a short period of time, hibernating animals undergo dramatic adaptive changes, including a reduction in heart and respiration rate and a decrease in core body temperature from 37°C (98.6°F) to 4°C (39°F), yet they do not experience cold-induced pain, and their organs continue to function despite being cold and deprived of oxygen for 8 month out of the year! Moreover, since these animals do not eat or drink during hibernation, they must rely solely on the management and utilization of their internal resources for long-term survival. How hibernators achieve such a remarkable physiological adaptation, remains unknown. We use hibernating 13-lined Ground squirrels (an obligatory hibernator) and Syrian hamsters (a non-obligatory hibernator), to tackle fundamental biological questions from perspectives unachievable using the standard animal models alone. Specifically, we are interested in studying molecular evolution of mammalian hibernation and cellular adaptations that these animals evolve in order to survive prolonged periods of hypothermia, water deprivation and starvation. We are also trying to pinpoint the molecular and physiological basis of hibernation induction. Comparative analysis of three rodent species—such as ground squirrels, hamsters and mice (non-hibernator)—at the behavioral, cellular and molecular levels, will help us to delineate the multitude of adaptations that hibernators evolved in order to survive harsh environment and as a result came to inhabit a wide geographical range.

### [2S02m-04]

### Analysis of physiological functions in hibernation-like state using mice.

*Takeshi SAKURAI[1] ([1]*University of Tsukuba*)

Some mammals hibernate, significantly lowering their body temperature and metabolic rates, to adapt to the winter climate, food scarcity, and other environmental factors. During hibernation, the physiological functions of their entire body, including brain function, are greatly reduced. However, once they recover from hibernation, their biological functions completely return. There are many questions, such as how the chronological continuity of self- and external-world awareness is maintained before and after hibernation, and how other physiological functions are maintained. Until now, the biology of hibernation has been hampered by the need to use hibernating animals. We have recently reported that it is possible to induce a hibernation-like state in mice by neural manipulation. Using this mimicked hibernation state, we can examine the behavior of physiological functions in the hibernation-like state using mice, which are an excellent experimental model system. In this symposium, I will present our findings on the body clock, memory, and cardiac functions in the induced hibernation-like state.

### [2S02m-05]

### A warm hibernation can protect organs from ischemia

*Genshiro A. Sunagawa[1], Hidetoshi Masumoto[1,2] ([1]*RIKEN BDR*, [2]*Department of Cardiovascular Surgery, Graduate School of Medicine, Kyoto University*)

Hibernation is a hypometabolic state in which animals lower their metabolism actively. We recently reported that mice enter a hibernation-like hypometabolic state by exciting the QRFP-positive neurons in the hypothalamus (Takahashi et al., Nature, 2020). The torpid state was named QIH after Q neurons–induced hypometabolism. Acute kidney injury (AKI) is a complication after cardiovascular surgery requiring circulatory arrest. Hypothermia is the mainstay to protect AKI during surgery. However, the lowered temperature is harmful in not a few aspects for humans. In this study, we investigated the efficacy of a warm but hypometabolic state induced by QIH for ameliorating AKI in a circulatory arrest mouse model. We compared the QIH mice to the control mice without lowering the body temperature. The descending aorta cross-clamping induced ischemic stress to the kidneys, and after reperfusion, we evaluated the renal function. We found that normothermic QIH exhibited lower NGAL and Cystatin C levels than those in control mice. QIH protects AKI in a mouse ischemia model, even in normothermia. These results show the potential medical application of warm hibernation.

### Symposium

[2S03m]
**Physiology in the kidney revisited**

March 15 (Wed.), 9:00 - 11:00, Room 3

### [2S03m-01]

### ATP visualization technique reveals the pathophysiology of renal disease

*Shinya Yamamoto[1], Masamichi Yamamoto[2], Shigenori Yamamoto[1,3], Masahiro Takahashi[1], Motoko Yanagita[1,3] ([1]*Department of nephrology, kyoto university,* [2]*National Cerebral and Cardiovascular Center Research Institute, Osaka, Japan.,* [3]*Institute for the Advanced Study of Human Biology (WPI-ASHBi), Kyoto University, Kyoto, Japan*)

Acute kidney injury (AKI) is a common clinical condition associated with high mortality. AKI predisposes to the progression of chronic kidney disease (CKD). However, the underlying mechanisms of AKI to CKD transition remain unclear. ATP depletion plays the central role in the pathogenesis of kidney diseases. Nevertheless, *in vivo* ATP dynamics in the kidney remained unclear due to technical difficulty. Using two-photon microscopy and the novel mouse strain to visualize ATP dynamics at single cell resolution, we revealed spatiotemporal ATP dynamics during AKI, ischemic reperfusion injury (IRI). We also showed the strong correlation between ATP recovery of proximal tubules in acute phase and renal fibrosis in chronic phase (JASN 2020). Further, we succeeded in visualizing ATP dynamics of glomeruli during IRI. ATP depletion and mitochondrial fragmentation in podocytes in acute phase could contribute to foot process effacement in chronic phase. Furthermore, pharmacological inhibition of mitochondrial fragmentation ameliorated foot process effacement and mitochondrial fragmentation in podocytes *in vivo*. Finally, we also established the novel *ex vivo* system using slice culture of mouse kidney to visualize ATP dynamics in whole kidney. Our ATP visualization technique has the potential to elucidate cellular mechanism of AKI to CKD progression, and might be useful for drug screening.

### [2S03m-02]

### Impact of increased central venous pressure on kidney injury progression

*Tetsuro Kusaba[1] ([1]*Division of Nephrology, Kyoto Prefectural University of Medicine*)

Recent clinical observations revealed that increased renal venous pressure, rather than decreased cardiac output, causes the deterioration of kidney function in heart failure (HF) patients. To uncover the pathophysiology and hemodynamics of renal congestion, we generated a novel mouse model with unilateral renal congestion by constriction of the inferior vena cava between renal veins. Intravital imaging highlighted the notable dilatation of PTCs and decreased renal blood flow velocity in the congestive kidney. Renal damage after ischemia reperfusion injury (IRI) was exacerbated in the congestive kidney and accumulation of polymorphonuclear leukocytes (PMNs) within PTCs was noted at the acute phase after injury. Similar results were obtained in vitro, in which PMNs adhesion on activated endothelial cells was decreased in flow velocity-dependent manner and it was cancelled by inhibition of NF-κB signaling. Pharmacological inhibition of NF-κB for the mice subjected by both renal congestion and IRI ameliorated the accumulation of PMNs and subsequent exacerbation of kidney injury. Our study demonstrates the importance of decreased blood flow velocity accompanying activated NF-κB signaling in the exacerbation of renal injury. Inhibition of NF-κB signaling can be a therapeutic candidate for the vicious cycle between heart and kidney failure with increased renal venous pressure.

### [2S03m-03]

### Novel renoprotective effects of an old antihistamine discovered by novel cellular energy metabolism screening and big data analysis.

*Seiji Kishi[1], Yasumasa Ikeda[2] ([1]*Department of Nephrology and Hypertension, Kawasaki Medical School,* [2]*Department of Pharmacology, Institute of Biomedical Sciences, Tokushima University Graduate School*)

Acute kidney injury (AKI) occurs in approximately 13% of hospitalized patients and is associated with high mortality. AKI is increasing worldwide as the population ages, but there are no drugs to prevent or treat AKI. Renal proximal tubule epithelial cells (RPTECs) comprise the bulk of the renal parenchyma and are the primary targets of a large variety of ischemic and toxic insults which leads to AKI. In this presentation, two studies will be presented that demonstrate that older antihistamines are protective against AKI. These studies screened candidate drugs based on basic and clinical data and revealed the novel mechanism against kidney injury. Meclizine, an over-the-counter anti-nausea and -dizziness drug, was identified in a 'nutrient-sensitized' chemical screen. Pretreatment with meclizine protected mice from ischemic AKI. Meclizine inhibited the Kennedy pathway and caused rapid accumulation of phosphoethanolamine. Phosphoethanolamine recapitulated meclizine-induced protection both in vitro and in vivo. Based on a repositioning analysis of the Food and Drug Administration Adverse Events Reporting System, we found that diphenhydramine may provide a novel treatment for cisplatin-induced kidney toxicity , an important clinical condition. Diphenhydramine inhibited cisplatin-induced cell death and attenuated AKI in mice. Importantly, diphenhydramine did not influence or interfere with the anti-tumor effect of cisplatin. A cohort study revealed the renoprotective effect of diphenhydramine.Thus, by using new techniques and methods, we have successfully identified the potential for drug repositioning of old antihistamines.

### [2S03m-04]

### DNA damage repair and DNA methylation changes in kidney diseases

*Kaori Hayashi[1] ([1]*Department of Nephrology, Endocrinology and Metabolism, Keio University School of Medicine*)

Recently the results of large clinical trials have suggested that transient treatment for lifestyle-related diseases has sustained effects on prevention for cardiovascular complication over a long period of time, which has been noted as "memory effect". One of the possible mechanisms of the memory effect is epigenetic changes. Epigenetic alterations have sustained effects on gene expressions, which contribute to the pathogenesis of chronic diseases, including chronic kidney diseases (CKD). DNA damage repair is an important chance to rewrite the epigenetic marks. We have demonstrated the importance of DNA damage repair in the kidney and its relations to kidney diseases through DNA methylation changes. The DNA repair system is strikingly cell-type specific, depending on the expression of DNA repair factors; therefore, DNA repair systems may differ among various types of kidney cells. In this talk, I would like to focus on DNA damage repair in glomerular podocytes, and discuss its possibility as a prognostic marker and a therapeutic target for CKD. Moreover, a role of peripheral blood cell DNA methylation caused by podocyte DNA damage on CKD progression is also discussed. Focusing on DNA damage repair and epigenetic changes may achieve a profound understanding of the pathogenesis of kidney diseases, which leads to develop novel therapeutic strategies.

### [2S03m-05]

### Crosstalk between kidneys and nerves

*Tsuyoshi Inoue[1] ([1]*Nagasaki University*)

The kidney is a highly developed organ, and has various functions such as regulation of fluid and electrolyte, regulation of blood pressure, production of erythropoietin, and activation of vitamin D. Progression to kidney disease is caused by various causes such as diabetes and hypertension, hereditary disease, and glomerulonephritis characterized by urine protein. At present, there is no drug for chronic kidney disease other than angiotensin II receptor antagonist (ARB) and sodium-glucose co-transporter-2 (Sglt2) inhibitor, which have inhibitory effects on the progression of kidney disease. The kidneys are very rich in sympathetic innervation, while little parasympathetic innervation has been identified. Despite this, we have previously shown that vagus nerve stimulation exerts a very strong renal protective effect. Using optogenetics and single-cell RNA-seq techniques, we have also found that vagus afferent stimulation exerts tubular protection via C1 neurons in the medulla oblongata, sympathetic nerves, and the spleen. I will briefly show anti-inflammatory and kidney protection mechanisms mediated by the autonomic-immune system and I would like to discuss them with you.

### Symposium

[2S04m]

### Cellular senescence: its role in organ homeostasis and life span

March 15 (Wed.), 9:00 - 11:00, Room 4

### [2S04m-01]

### Targeting senescent cells to improve aging pathologies

*Makoto Nakanishi[1] ([1]*Division of Cancer Cell Biology, Institute of Medical Science, University of Tokyo*)

Why do we age? This physiological phenomenon, which is experienced by everyone, is still largely unknown and remains a great mystery even today with the advancement of science and technology. Aging is a major risk factor for the development of many diseases such as cancer, but there is no clear answer as to why the elderly are more susceptible to cancer and various other diseases. Very recently, the mechanisms that control aging are gradually becoming clearer. In other words, it is now known that inflammation-inducing cells, such as senescent cells, accumulate in various organs and tissues with aging and adversely affect the microenvironment, which is the pathological basis for the decline of organ functions and the development of diseases such as cancer. Cellular senescence is induced *in vitro* by a variety of senescence-inducing stimuli and exhibits unique features, including the secretion of inflammatory cytokines. However, senescent cell research to date has mainly been conducted using cultured cells, so it is difficult to determine whether senescent cells in individuals have the same characteristics as cultured cells. When and where do they exist? Also, can accumulation of senescent cells really induce age-related changes in individuals? Can senescent cells be efficiently removed from individuals? Many questions remain unanswered. Thus, in order to understand integrated changes in individuals such as senescent traits, it is essential to analyze them at the individual level using laboratory animals. In this symposium, I would like to introduce our recent findings and discuss whether it is possible to control human aging in the future.

### [2S04m-02]

### Loss of matrix integrity underlies age-dependent impairment of epidermal stem cell heterogeneity

*Aiko Sada[1,2] ([1]*Kumamoto Univ,* [2]*Univ of Tsukuba*)

Tissue stem cells divide infrequently as a protective mechanism against internal and external stresses associated with aging. However, it is unclear whether “slow-cycling” nature confers protection to stem cells and delays their aging. Taking advantage of a mouse model labeling slow- and fast-cycling stem cell populations in the skin epidermis, we demonstrated that slow- and fast-cycling epidermal stem cells undergo distinct aging processes. Two years of lineage tracing reveals that Dlx1+ slow-cycling clones expanded into the fast-cycling stem cell territory, while the number of Slc1a3+ fast-cycling clones gradually declined. Transcriptome analysis further indicated that the molecular properties of each stem cell population are altered with age. Mice lacking fibulin-7, an extracellular matrix (ECM), show early impairments resembling epidermal stem cell aging, such as the loss of fast-cycling clones, delayed wound healing, and increased expression of inflammation- and differentiation-related genes. Fibulin-7 interacts with structural ECM and matricellular proteins, and the overexpression of fibulin-7 in primary keratinocytes results in slower proliferation in the absence or presence of inflammatory cytokine IL-6. Together, our results suggest that fibulin-7 maintains epidermal stem cell heterogeneity and long-term tissue resilience.

### [2S04m-03]

### Targeting senescent cells for the treatment of age-associated disease

*Tohru Minamino[1] ([1]*Department of Cardiovascular Biology and Medicine, Juntendo University Graduate School of Medicine*)

Epidemiological studies have shown that age is the dominant risk factor for lifestyle-related diseases. The incidence and the prevalence of diabetes, heart failure, coronary heart disease and hypertension increase with advancing age. However, the molecular mechanisms underlying the increased risk of such diseases that is conferred by aging remain unclear. Cellular senescence is originally described as the finite replicative lifespan of human somatic cells in culture. Cellular senescence is accompanied by a specific set of phenotypic changes in morphology and gene expression including negative regulators of the cell cycle such as p53. Primary cultured cells from patients with premature aging syndromes are known to have a shorter lifespan than cells from age-matched healthy persons. It is also reported that the number of senescent cells increases in various tissues with advancing age. Interestingly, such accumulation of senescent cells in aged animals is attenuated by caloric restriction that regulates the lifespan regulatory system and delays age-associate phenotypes. I therefore hypothesize that cellular senescence in vivo contributes to the pathogenesis of age-associated disease and have shown a critical role of cellular senescence in age-related pathologies. However, a direct inhibition of cellular aging signaling would lead to the increased incidence of cancer, so we need to develop anti-senescent therapy without cancer development. Here I will show our recent data on a novel strategy of anti-senescent therapy for age-associated disease by targeting cellular senescence (Seno-antigens, Seno-metabolites, SASP), which would not promote tumorigenesis.

### [2S04m-04]

### The roles of cellular senescence and SASP in tumor microenvironment: Gasdermin D-mediated release of SASP factors from senescent hepatic stellate cells promotes obesity-associated hepatocellular carcinoma

*Ryota Yamagishi[1], Fumitaka Kamchi[1], Tomonori Kamiya[1], Masaki Takasugi[1], Yi Cheng[1], Yoshiki Nonaka[1], Norifumi Kawada[2], Susumu Nakae[3], Eiji Hara[4], Naoko Ohtani[1] ([1]*Department of Pathophysiology, Osaka Metropolitan University Graduate School of Medicine,* [2]*Department of Hepatology, Osaka Metropolitan University, Graduate School of Medicine,* [3]*Graduate School of Integrated Sciences for Life, Hiroshima University,* [4]*Research Institute for Microbial Diseases, Osaka University*)

While cellular senescence functions as tumor suppression mechanism, long-term senescent cells produce detrimental secretome, termed the senescence-associated secretory phenotype (SASP). Although mechanism of SASP factor induction have been intensively studied, it remains unclear whether senescent cells have distinct mechanisms to secrete SASP factors. In this study, we show that a SASP factor, IL-33, is induced in senescent hepatic stellate cells (HSCs) in the obesity-associated liver tumor, and is cleaved by chymotrypsin-like elastase family member 1 (CELA1). Interestingly, we found activated form of IL-33 is released from senescent HSCs triggered by lipoteichoic acid (LTA), and the release of IL-33 was mediated through cell membrane pores formed by the gasdermin D (GSDMD) N-terminus, which was cleaved by LTA-induced caspase-11. Moreover, we demonstrated that IL-33 release promoted hepatocellular carcinoma (HCC) development through activating ST2-positive Treg cells. These results uncover a release mechanism of IL-33 from senescent HSCs, thereby facilitating obesity-associated HCC progression. Our findings could lead to new insights for understanding obesity-associated HCC progression.

### [2S04m-05]

### Cellular senescence and cancer: relevance to microorganisms

*Eiji Hara[1] ([1]*Research Institute for Microbial Diseases, Osaka University*)

Although cellular senescence act as an important tumor suppression mechanism, the accumulation of senescent cells *in vivo* has been shown to cause a phenomenon called SASP, in which various pro-inflammatory and/or pro-tumorigenic factors are secreted, resulting in harmful side effects. It is therefore conceivable that the accumulation of senescent cells *in vivo* may contribute to aging-associated inflammatory diseases, such as cancer. Thus, the development of methods to remove senescent cells accumulated in the body as a means of tumor suppression and anti-aging has been actively pursued, and more than 20 candidates for senescent cell removal drugs (senolytic drugs) have already been reported so far. On the other hand, it has been reported that SASP may play important roles in wound healing, immune activation, and other aspects of homeostasis in the body. Therefore, rather than eliminating senescent cells accumulated in the body, we thought it would be safer and more effective to identify the causes of cellular senescence in the body and prevent them. Here, we will focus on changes in the gut microflora as one of the causes of cellular senescence and discuss their mechanism of action.

### Symposium

### [2S05m]
### Neurophysiology in the big data era: uncovering ultra-complex emotion circuits connecting self and others

March 15 (Wed.), 9:00 - 11:00, Room 5

### [2S05m-01]
### Introduction: Data driven approach to visualize dynamic information processing in the brain - toward the next 100 years of neurophysiology linking brain and mind -

*Masakazu Agetsuma[1,2] ([1]*National Institute for Physiological Sciences,* [2]*National Institutes for Quantum Science and Technology*)

Throughout the COVID-19 era, people around the world have come to reaffirm the importance of the connection between individuals. The brain regulates the underlying "recognition of self and others" and the resulting social interaction, by processing multidimensional information including sensory information from the external environments and temporal components such as memory and prediction. This symposium introduces the ambitious interdisciplinary studies, based on data-driven and computational approaches combined with cutting-edge methods generating big data, such as next-generation whole-brain-wide anatomy, and large-scale recording and fine manipulation of neural activity. These approaches enable the dissection of the ultra-complex computational architecture underlying social interactions, encouraging the next 100 years of neurophysiology.

### [2S05m-02]
### Neural representation of self- and other-states during observational fear

*Ziyan Okuyama Huang[1], Myung Chung[1], Kentaro Tao[1], Akiyuki Watarai[1], Mu-Yun Wang[1], Hiroh Ito[1], Teruhiro Okuyama[1] ([1]*Univ. Tokyo*)

Emotional contagion of fear as a fundamental form of empathy is observed across species through social observation of others suffering from aversive stimuli. Observational fear (OF) task in rodents enables quantification of the vicarious fear responses of an observer, while a demonstrator receives repetitive foot shocks. However, it remains unclear how the complex behavioral states of self are neurophysiologically represented during OF. Moreover, the function of the ventromedial prefrontal cortex (vmPFC), an important site for empathy, has not been clarified yet. Here, by combining behavioral tracking and dimension reduction clustering, eight types of stereotypic behavioral clusters of the observers were classified. We found that optogenetic inhibition of the vmPFC disrupted the OF-induced escape behavior among other behaviors. Furthermore, in vivo microendoscopic $Ca^{2+}$ imaging revealed that the multifaceted behavioral states of self and others' fear responses were represented and intermingled in vmPFC neural populations. Our study suggests that vmPFC neurons represent both self- and other-states to elicit escape behavior during OF.

### [2S05m-03]
### Subspace interactions between cortical and subcortical areas in social reward processing

*Atsushi Noritake[1], Hirokazu Tanaka[2], Masaki Isoda[1] ([1]*Division of Behavioral Development, National Institute for Physiological Sciences,* [2]*Faculty of Information Technology, Tokyo City University*)

Elucidating neural functions and algorithms underlying behaviors is an important goal in neurophysiology. This presentation aims to characterize what computational components in a cortical brain region sculpt activities in a subcortical brain region through machine-learning analyses of neural population activities. Our previous study simultaneously recorded single-unit activity from the medial prefrontal cortex (MPFC) and midbrain dopaminergic nuclei (DA) of the macaque during social reward valuation. The two brain regions played distinct functional roles, and the MPFC activities causally influenced the DA activity clarified by a Granger analysis. Our new study finds that single-unit activities in the MPFC (distinct coding of self and other rewards) successfully reconstruct those in the DA (integrated coding of self and other rewards). In this reconstruction, only specific combinations of MFPC activities contribute to the DA activities, thus supporting the idea that a subspace of MFPC activity space dominates the DA activity space. The two brain regions hence communicate through “an interaction subspace.” We propose that explicit reconstruction of population activities serves as a concrete understanding of inter-regional interactions in neural information processing.

### [2S05m-04]
### Brain-wide neuronal activation mapping shed light on new ensembles controlling brain functions

*Atsushi Kasai[1] ([1]*Osaka University*)

In the brain, neurons in various brain regions communicate with each other to control complex brain functions such as emotion. Therefore, it is very important to analyze neuronal activation patterns in a wide range of brain regions in order to systematically understand brain function and dysfunction. To this end, we have developed an automated very high-speed imaging system for block-face serial microscopy tomography (termed FAST; Seiriki et al, Neuron, 2017; Seiriki et al, Nat Protocols, 2019). We then combined FAST imaging with the immediate early gene reporter mice to generate brain-wide activation maps of mice exposed to various stimuli, including stress exposure and drugs administration. By performing classification analysis using machine learning on these maps, we were able to identify brain regions and cell populations that have been overlooked in the mechanisms of antidepressant effects and stress responses. This hypothesis-free exploration approach of functional changes in the whole brain can be applied to various animal conditions and disease models, and is expected to make a significant contribution to our understanding of the mechanisms of brain function and disease pathology in the future.

### [2S05m-05]
### Structure and Functions of the Frontal Cortex-Dopamine System Network That Facilitate Maternal Learning

Gen-ichi Tasaka[1], *Kazunari Miyamichi[1] ([1]*RIKEN BDR*)

Maternal behaviors are crucial for the health of mammalian infants. Although mothers are innately motivated to provide care for their young, how this high motivation state is implemented at the level of neural circuitry remains elusive. Recent molecular neuroscience studies have identified essential functions of the specific preoptic neurons for maternal behaviors. However, little is known about the involvement of the higher cognitive brain areas such as the frontal cortex. We herein introduce a cell-type specific chronic Ca2+ imaging of the orbitofrontal cortex (OFC). We revealed distinct populations of OFC that represented pup-directed premotor activities, ongoing pup-retrieval motions, and non-social rewards. By viral tracing and manipulations, we identified the submedius thalamus as a prominent pre-synaptic structure of the OFC that shaped the pup-related representations in the OFC. We also functionally revealed that the OFC facilitated the dopamine reward system to allow efficient learning of maternal care. Collectively, our data illuminate a frontal cortex-reward system network that contributes to the high maternal behavioral motivation.

### Symposium

[2S06m]
**Changing Trends in Gastrointestinal Research: From Molecules and Cells to Organisms**

March 15 (Wed.), 9:00 - 11:00, Room 6

### [2S06m-01]

### Pathophysiological function of the sodium pump abnormally expressed in intracellular vesicles of gastrointestinal cancer cells

*Takuto Fujii[1], Mizuki Kato[1], Takahiro Shimizu[1], Hideki Sakai[1] ([1]*Department of Pharmaceutical Physiology, Faculty of Pharmaceutical Sciences, University of Toyama*)

Sodium pump ($Na^+$,$K^+$-ATPase) is primarily located in the plasma membrane (PM) and plays an important role in regulating membrane potential and cellular ion homeostasis in almost all cells. However, we recently found that $Na^+$,$K^+$-ATPase α3-isoform (α3NaK) is abnormally expressed in intracellular vesicles in various human cancer cells. The percentage of α3NaK-positive tissue was particularly high in colon and gastric cancers. We have studied the pathophysiological function of α3NaK in cancer cells. First, we found that α3NaK is dynamically translocated from intracellular vesicles to the PM by loss of anchorage in the cancer cells. Our *in vitro* and *in vivo* studies showed that the translocation of α3NaK contributes to the survival of metastatic cancer cells. Cardiac glycosides, $Na^+$,$K^+$-ATPase inhibitors, significantly blocked the α3NaK translocation. Next, we found that cardiac glycosides stimulate the endocytosis of glucose transporter GLUT1 and inhibit glycolysis and proliferation in liver, colon, and gastric cancer cells by targeting intracellular α3NaK. Our study suggests that α3NaK is one of the therapeutic targets for inhibiting the growth and metastasis of gastrointestinal cancers.

### [2S06m-02]

### Aging-related changes of claudins and amino acid transporters expression in the colon.

*Yuta Yoshino[1], Ema Okamoto[1], Shunsuke Matsuda[1], Yoshifumi Morikawa[2], Toshiyuki Matsunaga[3], Satoshi Endo[1], Yoshiaki Tabuchi[4], Akira Ikari[1] ([1]*Laboratory of Biochemistry, Gifu Pharmaceutical University,* [2]*Forensic Science Laboratory, Gifu Prefectural Police Headquarters,* [3]*Laboratory of Bioinformatics, Gifu Pharmaceutical University,* [4]*Life Science Research Center, University of Toyama*)

Frailty is a condition in which muscle strength and vitality decline in old age, partially due to a deficiency of some nutrients, such as amino acids. Claudins (CLDNs), essential component proteins in a tight junction, are responsible for the absorption of small molecules and electrolyte ions through the intercellular space. However, it remains unknown whether CLDN protein is involved in the regulation of absorption of amino acids in the intestine. In this study, we examined the effects of aging on the expression and role of CLDNs in the mouse colon. We investigated the expression levels of amino acid transporters in the colon tissue compared with young and aged mice. The expression levels of the cystine/glutamate antiporter (xCT) and amino acid transporter (LAT) 2 and LAT3 significantly increased in aged mice. These results suggest that amino acid transporter expression increases toward the attenuation of amino acid deficiency associated with aging. We also found that CLDN8 expression decreased with aging. The removal of amino acid from the culture medium did not significantly alter the transcriptional activity of CLDN8, suggesting the involvement of other regulatory mechanisms. TargetScan program showed that fourteen miRNAs predicted to bind to the 3'-untranslated region of CLDN8. The expression of miR-153-5p was only increased by the removal of amino acids. The treatment with an inhibitor of miR-153-5p suppressed the amino acid removal-induced decrease in CLDN8 expression. Next, we examined the effect of CLDN8 expression on intercellular amino acid permeability and demonstrated that knockdown of CLDN8 expression increased amino acid permeability from the luminal side to vascular side. These results suggest that amino acid deficiency enhances intestinal amino acid absorption via increased expression levels of amino acid transporters and decreased CLDN8 expression. In conclusion, CLDN8 may be a novel drug target for mitigating amino acid deficiency associated with aging.

### [2S06m-03]

### Development of personalized therapies using patient-derived gastrointestinal epithelial stem cells

*Hiroyuki Miyoshi[1], Makoto Taketo[1,2] ([1]*Kyoto University,* [2]*Kitano Hospital*)

Recent technical advances have allowed culturing of mammalian tissue-derived epithelial stem cells as three-dimensional cell aggregates termed "organoids" or "spheroids". We have previously developed a powerful method for propagating adult intestinal epithelial stem cell spheroids using conditioned media from a supportive cell line (L-WRN) that simultaneously secretes the critical stem cell niche factors, Wnt3a, R-spondin 3, and noggin. We have applied this strategy to human colorectal cancer and established over 200 lines of patient-derived colorectal cancer stem cell (PD–CRC-SC) spheroids. Whereas these spheroids can be employed as the source of high-quality DNA, RNA, and histological specimens for personalized clinical diagnosis, a more direct approach is to test their drug sensitivity in vitro. We recently found that EGFR and FGFR inhibitors suppressed the growth of CRC-SC spheroids differently depending on the tumor line. These results reveal that evaluating drug efficacy with PD–CRC-SC spheroids can be a promising strategy to find the best regimen for individual patients. We are investigating the time- and cost-efficient operation of drug-sensitivity tests by refining the procedures to establish spheroids, estimate spheroid volumes, and predict efficacy of therapy. Our goal is to provide clinical services that can stratify patients for current and upcoming cancer therapies.

### [2S06m-04]

### Claudin-15 is responsible for the conductance and permselectivity of the murine cecum and large intestine

*Wendy Leanne Hempstock[1,2], Nozomi Nagata[2], Noriko Ishizuka[2], Hisayoshi Hayashi[2] ([1]*University of Shizuoka, School of Nursing, Department of Nursing,* [2]*University of Shizuoka, Graduate School of Nutritional and Environmental Sciences, Laboratory of Physiology*)

The large intestine functions in electrolyte and water homeostasis and as a barrier. As a part of the barrier function of the large intestine, tight junction (TJ) proteins, especially claudin-15, play an important role in nutrient uptake in the small intestine by recycling the $Na^+$ necessary for nutrient absorption back to the lumen, but their role in the cecum and colon has been largely ignored. The aim of this study was to explore the significance of claudin-15 in the murine cecum and large intestine using claudin-15 knockout (*Cldn15* KO) mice. Using chambers were used to assess baseline electric parameters, $Na^+$ flux, and dilution potentials in the cecum and colon from *Cldn15* KO mice. Conductance and $Na^+$ flux were decreased in the cecum but not the middle colon of *Cldn15* KO mice, while TJ permeability to $Na^+$ was reduced. These results suggest that claudin-15 and paracellular transport have a segment-dependent role in the cecum and large intestine. In contrast to previous ideas, TJ in the cecum and large intestine are cationic selective and we show that claudin-15 is the main molecule responsible for paracellular transport of $Na^+$ in the cecum and large intestine.

### [2S06m-05]

### Findings from single cell analysis using intestinal epithelial organoids

*Yoshimasa Saito[1] ([1]*Keio University Faculty of Pharmacy*)

It is known that organ function and tissue building capacity decline with aging, and the incidence of diseases such as malignant tumors increases; however, the molecular mechanisms underlying these changes remain unclear. Recently, the stem cell aging hypothesis, which proposes that aging of stem cells, the source of all cells, is the cause of aging of tissues and organs, has been attracting attention. We have previously established organoids from the intestinal epithelium of aged mice by organoid culture, a three-dimensional culture method of tissue stem cells, and analyzed the changes in the constituent cells of intestinal epithelial cells with aging by single cell analysis. The expression of Lgr5, a marker of stem cells, is decreased in intestinal epithelial organoids from aged mice. In addition, the cell number ratio of tuft cells, a rare cell type in the intestinal epithelium, decreased with aging, and we found that some tuft cells express Lgr5. These findings indicate that tuft cells have potential ability as stem cells in the intestinal epithelium and play a critical role in the molecular mechanisms of aging and aging-associated tumorigenesis.

### Symposium

[2S07m]

**Recent advances in the regulation of homeostasis by immune-vascular systems**

March 15 (Wed.), 9:00 - 11:00, Room 7

### [2S07m-01]

### Systemic integration toward the central nervous system regeneration

*Rieko Muramatsu[1] ([1]*Department of Molecular Pharmacology, National Institute of Neuroscience, National Center of Neurology and Psychiatry*)

Age-related regeneration failure in the central nervous system can occur as a result of a decline in remyelination efficacy. The responsiveness of myelin-forming cells to signals for remyelination is affected by aging-related epigenetic modification; however, the molecular mechanism is not fully clarified. In the present study, we report that the apelin receptor (APJ) mediates remyelination efficiency with age. APJ expression in myelin-forming cells is correlated with age-associated changes in remyelination efficiency, and the activation of APJ promotes remyelination through the translocation of myelin regulatory factor. APJ signaling activation promoted remyelination in both aged mice with toxin-induced demyelination and mice with experimental autoimmune encephalomyelitis. In human cells, APJ activation enhanced the expression of remyelination markers. Impaired oligodendrocyte function in aged animals can be reversibly reactivated; thus, the results demonstrate that dysfunction of the apelin–APJ system mediates remyelination failure in aged animals, and that their myelinating function can be reactivated by APJ activation. Ito et al. show that regeneration in the aging brain is impaired due to reduced expression of the apelin receptor APJ. Circulating apelin signals oligodendrocytes via APJ to support remyelination, and this pathway can be restored in older mice with an APJ agonist.

### [2S07m-02]

### The role of reactive astrocytes in the spinal dorsal horn in chronic itch

*Miho Shiratori-Hayashi[1], Makoto Tsuda[1] ([1]*Department of Molecular and System Pharmacology, Graduate School of Pharmaceutical Sciences, Kyushu University*)

Chronic itch associated with inflammatory skin diseases such as atopic dermatitis significantly reduces the quality of life of patients and should be treated appropriately. Such itch is difficult to treat with existing therapeutic agents, including antihistamines, and the underlying mechanisms are poorly understood. To elucidate the mechanisms of chronic itch, we have focused on changes in the spinal dorsal horn (SDH). In this context, we have recently demonstrated that astrocytes, a type of glial cells, in the SDH become reactive during the chronic phase in a STAT3-dependent manner and play an important role in chronic itch. This finding reveals for the first time the importance of SDH changes in chronic itch. In this talk, we will show the factor that is upregulated in SDH astrocytes in a STAT3-dependent manner during the chronic phase and its influence on itch signaling in the SDH, as well as the mechanisms of SDH astrocyte STAT3 activation in chronic itch.

### [2S07m-03]

### Tissue-Resident Macrophages in Homeostasis

*Yasutaka Okabe[1] ([1]*WPI Immunology Frontier Research Center, Osaka University*)

Tissue-resident macrophages are essential compontents for the maintenance of tissue homeostasis, present in virtually every mammalian tissue where they monitor local tissue environment. They have many tissue-specific functions that reflect functional adaptation to each tissue environment. Accordingly, abnormalities of tissue-resident macrophage functions link to various pathologies including osteopetrosis, type 2 diabetes, immune disorders, and neurodevelopmental diseases. In this talk, I will discuss the functional specialization of tissue-resident macrophages and their roles in the maintenance of tissue homeostasis.

### [2S07m-04]

### Vascular endothelial cell heterogeneity and mechanism of vascular formation

*Hisamichi Naito[1] ([1]*Kanazawa University*)

Blood vessels form vast vascular network throughout the body and maintain homeostasis. Blood-vessel dysfunction and dysregulation of new blood-vessel formation are related to the onset and progression of many diseases including cancer and ischemic disease. Endothelial cells (ECs) cover inner surface of the blood vessels and their proliferation is essential for new vessel formation from pre-existing blood vessel, termed angiogenesis. For the emergence of a new sprout from a blood vessel, angiogenic stimuli such as VEGF induce specification of ECs into functionally and morphologically characterized EC phenotypes known as tip or stalk cells. These phenotypic specifications are not stable, and it is now considered all ECs possess equal potential to proliferate and guide EC sprouting, depending on their microenvironment. However, recent works have greatly improved the understanding of the highly diverse cell heterogeneity and now it is becoming clear that vascular ECs show previously unknown heterogeneity in vascular formation, organ homeostasis, and disease. In this session, I would like to introduce our recent work on celler heterogeneity and their function for organ specific vascular formation. Our data provide a novel insight into the organ specific vascular formation based on cellular heterogeneity.

### [2S07m-05]

### Discovery of novel physiologically active peptide, JIP, that promotes epithelial tissue repair

*Yukako Oda[1] ([1]*CiRA, Kyoto University*)

Tight junctions (TJs) are cell-cell adhesion apparatuses that function as barriers between epithelial cells, and their disruption leads to various inflammatory diseases and tissue destruction. However, a therapeutic strategy to overcome TJ disruption in diseases has not been established because of the lack of clinically applicable TJ-inducing molecules. Recently, we discovered TJ-inducing peptides (JIPs) in mice and humans, which corresponded to 35–42 residue peptides of the C-terminus of alpha 1-antitrypsin (A1AT), a serine protease inhibitor abundant in circulating blood. JIPs were inserted into the plasma membrane of epithelial cells, which induced TJs by directly activating the heterotrimeric G protein G13. *In vivo*, subcutaneous administration of JIP into intestinal epithelial injury model mice established by dextran sodium sulfate (DSS) restored TJ integrity and strongly prevented colitis, whereas inhibition of JIPs impeded the restoration of TJs in regenerating intestinal epithelial cells. Our study has revealed TJ-inducing anti-inflammatory physiological peptides that play a critical role in tissue repair and proposes a novel therapeutic strategy for TJ-disrupted diseases.

### Symposium

[2S08m]
Cooperation with Other Societies Committee
**Coordination of visceral organ functions: Basic and clinical aspects**

March 15 (Wed.), 9:00 - 11:00, Room 8

### [2S08m-01]

### Cooperative interaction with intestinal GLP-1 and pancreatic insulin on vagal afferents enhances insulin action

*Yusaku Iwasaki[1] ([1]*Kyoto Prefectural University*)

Physically stable glucagon-like peptide-1 receptor (GLP-1R) agonist promotes insulin release from pancreas to improve hyperglycemia, whereas the physiological function of endogenous intestinal GLP-1 has not been completely elucidated due to its instability in vivo. Here, we examined the effects of intestinal GLP-1 on hyperglycemia using a novel GLP-1 releaser, rare sugar Allulose (Y. Iwasaki, Nat Commun 2018). In healthy or type 1 diabetic Akita mice, single po administration of Allulose significantly promoted GLP-1 secretion but did not alter blood glucose. In hyperinsulinemic type 2 diabetic model mice, po Allulose markedly ameliorated hyperglycemia without stimulating insulin secretion. These beneficial effects were blunted by the blockage of GLP-1R genetically or pharmacologically. These results suggested that intestinal GLP-1 improves hyperglycemia in a plasma insulin level dependent manner. Insulin-induced lowing blood glucose was potentiated by GLP-1 release in Akita mice. Furthermore, the hypoglycemic effects of sulfonylureas and thereby vagal afferent activation were also enhanced by GLP-1 release. These effects were completely abolished by chemical denervation of vagal afferents. In conclusion, intestinal GLP-1 collaborates with pancreatic insulin to activate vagal afferents, thereby enhancing insulin action and improving hyperglycemia.

### [2S08m-02]

### Regulation of ovarian estradiol secretion and change in autonomic functions by somatosensory stimulation

*Fusako Kagitani[1,2], Sae Uchida[2] ([1]*Univ. Human Arts Sci.*, [2]*Dept. Auton Neurosci, Tokyo Metropol. Inst. Gerontol.*)

It is well known that ovarian estradiol secretion is cyclically regulated by the hypothalamic-pituitary-ovarian axis. In this symposium, I will talk about our recent animal studies on the autonomic nerve-mediated reflex regulation of ovarian estradiol secretion integrated in the brainstem. In anesthetized rats, noxious stimulation of a hindpaw for 5 min decreases the ovarian estradiol secretion rate. The largest reduction compared with pre-stimulus values is 29%. The ovarian estradiol decrease responses are abolished by bilateral severance of the ovarian sympathetic nerves. These responses are observed even after decerebration but are abolished after spinal transection. Thus, the inhibition of ovarian estradiol secretion during hindpaw stimulation is integrated mainly in the brainstem, and this ovarian inhibitory response is due to reflex activation of the sympathetic nerves to the ovary. The sympathetic inhibitory regulation of ovarian estradiol secretion becomes pronounced when the HPO axis is inhibited by chronic estradiol treatment in rats. Considering the entire female cycle, extensive physical stress may easily inhibit ovarian function, especially during periods of before puberty and old ages when the HPO axis is inactive.

### [2S08m-03]

### The photosensitive characteristics of intrinsically photosensitive retinal ganglion cell in migraine

*Eiichiro Nagata[1] ([1]*Department of Neurology, Tokai University School of Medicine*)

Migraine is a genetically influenced complex disorder characterized by episodes of moderate-to-severe headache, most often unilateral and generally associated with nausea and increased sensitivity to light (photophobia), sound (phonophobia), and smell (hyperosmia). Notably, Photophobia is regarded as a most bothersome accompanying common symptom commonly seen in migraine attacks. However, the underlying mechanism is uncertain. The discovery of the intrinsically photosensitive retinal ganglion cells (ipRGCs) which signal the intensity of light on the retina has led tosparked a discussion aboutof their potential role in the pathophysiology of migraine. The ipRGCs recognize light and dark senses and non-imaging vision. The ipRGCs react the wavelength 484 nm (blue light), which does not activate although the cone orand rod cells of retina don't react the wavelength. Moreover, the ipRGCs are closely associated with pupillary response via the network of brain stem, especially the speed of mydriasis. Based on those findings, we investigate the relationship between the pathophysiology of migraine and ipRGCs function by focusing onaccording to pupillary response and using an animal model of migraine (CSD model).

### [2S08m-04]

### Considering adaptive changes of the near response in an ICT environment

*Naoto Hara[1] ([1]*International University of Health and Welfare, Department of Orthoptics and Visual Sciences*)

In today's advanced information society, computers are essential for communication and the overall functioning of society through digital technologies (e.g., IoT, AI, and Big Data). While advanced information and communications technology (ICT) has brought tremendous benefits to society, there are adverse effects as well. Widespread work using visual display terminals has caused health problems that manifest as visual, musculoskeletal, and neuropsychiatric symptoms. Gazing at a close display elicits the near response, which comprises convergence, accommodation, and pupillary constriction, and we have studied health problems based on these three reactions. Symptoms such as eyestrain and headache develop when distant viewing changes to close viewing and the near response cannot adapt at first. Recently, we have seen rapid increases in acute acquired comitant esotropia caused by excessive habitual computer use, as well as now ubiquitous smartphones. I believe that these are excessive adaptive changes of the near response induced by a decreased frequency of distant viewing. In this presentation, I will describe "adaptive changes" of the near response in the modern ICT environment as encountered in my experience.

### Symposium

[2S09m]

**Pericytes -Functional diversity and commonality in health and disease**

March 15 (Wed.), 9:00 - 11:00, Room 9

### [2S09m-01]

### Investigation of molecular mechanisms underlying pericyte development

*Koji Ando[1] ([1]*National Cerebral and Cardiovascular Center Research Institute, Department of Cardiac Regeneration Biology*)

Despite the importance of organ development and homeostasis, the molecular mechanisms underlying pericyte developmental are not fully understood. Here, we investigated pericyte formation and subsequent differentiation using zebrafish and mouse models. Using zebrafish models, we succeeded in capturing the process of specification into pericyte lineage from peri-arterial *pdgfrb*$^{low}$ mesenchyme and revealed that this specification is induced by Notch2 and Notch3 activation. Notch2 and Notch3 function predominantly in trunk and brain pericyte, respectively, suggesting that the characteristics of pericytes differs at their early development stages. Subsequent analysis in the zebrafish and mouse revealed that pericytes function as a progenitor for vascular smooth muscle cells (VSMCs) in the brain. We found that pericyte selective K-ATP channel in the brain, composed of ABCC9 and KCNJ8, cell-autonomously regulates differentiation of pericyte into VSMCs in an brain-specific manner. Taken together, we revealed the indispensable role of Notch activation in the first pericyte formation and brain-selective regulatory mechanisms of VSMC differentiation from pericyte modulated by K-ATP channel.

### [2S09m-02]

### The TMEM16A channel as a key modulator of pericyte tone: physiology and implications for therapy

*Paolo Tammaro[1] ([1]*University of Oxford*)

Increased neuronal activity is accompanied by a rise in local brain metabolism, which triggers a parallel rise in cerebral blood flow (CBF). Contractile pericytes, cells that surround capillaries, participate in the modulation of CBF by altering capillary diameter and microvascular resistance to blood flow. In pathology such as ischaemia, pericytes contract and then die in rigor, hampering CBF. The determinants of pericyte tone are poorly understood. We show that the $Ca^{2+}$-gated anion channel TMEM16A is expressed in pericytes and constitutes a depolarising force activated by a rise in intracellular $Ca^{2+}$ or ischaemia. Pharmacological inhibition of TMEM16A reduced pericyte $Ca^{2+}$ rise and capillary constriction in response to GqPCR agonists, with no effect on the electrical activity of cortical neurons. Exposure of cortical slices to oxygen-glucose deprivation (ODG), to simulate ischaemia, led to significant pericyte death, which was prevented by pharmacological inhibition or enhanced by activation of TMEM16A. In a rodent stroke model, TMEM16A inhibition reduced the ischemia-evoked $Ca^{2+}$ rise, capillary constriction, and pericyte death. Thus, pericyte TMEM16A is a crucial regulator of cerebral capillary function and a potential drug target for CBF disorders.

### [2S09m-03]

### Roles of pericyte in brain health and cerebrovascular diseases

*Tetsuro Ago[1] ([1]*Department of Medicine and Clinical Science, Graduate School of Medical Sciences, Kyushu University*)

Pericytes are mural cells interacting directly with endothelial cells in micro-vessels neighboring neurons and glial cells in the central nervous system. They play crucial roles in maintaining the blood-brain barrier formed by endothelial cells and regulating blood flow in response to neuronal activity in the intact brain. Because pericytes are more vulnerable to ischemic insults or metabolic disturbances than endothelial cells, they are often lost from around endothelial tubes within ischemic areas. Early reperfusion can rescue neural death, and can ameliorate tissue repair and functional recovery in animals, including humans, with infarcts of similar size: the pericyte re-coverage, achieved by its survival or recruitment, around endothelial tubes can restore blood flow even within infarct areas and enable macrophages to infiltrate and remove myelin debris within infarct areas. Through reciprocal interaction, macrophages and pericytes/pericyte-derived fibroblasts promote intra-infarct repair and produce various trophic factors activating astrocytes and inducing OPC differentiation and remyelination in peri-infarct areas, thereby leading to functional recovery. Tissue repair and functional recovery are significantly attenuated in pericyte-deficient *Pdgfrb+/–* mice. In this talk, we would like to discuss roles of pericytes in brain health and post-stroke functional recovery.

### [2S09m-04]

### Mechanism underlying pericyte spontaneous activity in viscera

*Hikaru Hashitani[1] ([1]*Department of Cell Physiology, Nagoya City University Graduate School of Medical Sciences*)

In visceral organs that undergo wall distension, pericytes in capillaries and pre/post- capillary vascular segments develop spontaneous $Ca^{2+}$ transients with or without associated constriction. The spontaneous $Ca^{2+}$ transients primarily arise from cyclical release of $Ca^{2+}$ from the endoplasmic reticulum (ER) resulting in spontaneous transient depolarisations (STDs) upon the opening of $Ca^{2+}$-activated $Cl^-$ (TMEM16A) channels. STDs by their own or subsequent depolarisations due to the opening of L/T-type voltage-dependent $Ca^{2+}$ channels appear to spread to neighboring pericytes via gap junction allowing the synchrony or the spread of spontaneous activity amongst pericytes. Endothelial cells also play a fundamental role in maintaining the synchrony of pericyte spontaneous activity. Thus, hyperpolarisation of pericytes either upon the transmission of activated endothelium inward rectifier $K^+$ channels (Kir2.1) and/ or endothelium-derived prostaglandin $I_2$ activating pericyte ATP-sensitive $K^+$ channels promotes the cyclical release of ER $Ca^{2+}$. Non-contractile capillary pericytes in viscera function as pacemaker cells driving vasomotion in 'upstream' pre-capillary arterioles to facilitate tissue perfusion. Venular pericytes generate vasomotion at their own rhythms to ensure tissue drainage.

### Symposium

[2S10m]
J Physiol - PSJ Joint Symposium
**Distinguished research published in J Physiol by Japanese authors**

March 15 (Wed.), 9:00 - 11:00, Room 10

### [2S10m-01]

### Intracellular staining by microelectrode for morphological identification of recorded cells

*Akimichi Kaneko[1] ([1]*Keio University*)

The vertebrate retina consists of 5 types of cells arranged in 3 layers. To elucidate the neuronal circuit of the retina it is important to reveal the light-evoked response of each retinal cells. Intracellular recordings were made from individual retinal cells, but identification of recorded cells was difficult because penetration of retinal cells had to be made without visual control in the dark room. To identify the recorded cells a dye, Procion yellow, was intracellularly injected from the recording micro- electrode and the stained cells were identified under the fluorescent microscope. Injected Procion yellow diffused within the cell into even very fine tip of the cell process and revealed the entire morphology of the cell like Golgi's silver impregnation method. Horizontal cell, bipolar cell and amacrine cells were thus identified unequivocally.

### [2S10m-02]

### Muscular thin-fiber receptors and their functions in physiological and pathological conditions

*Kazue Mizumura[1,2] ([1]*Department of Physiology, Nihon University School of Dentistry,* [2]*Nagoya University)*

Muscular pain is quite common, but characteristics of peripheral sensory receptors for it were only minimally reported when I, with collaboration with late Prof. Kumazawa, started my study. We demonstrated that most muscular thin-fiber receptors were the polymodal receptor responding to mechanical and heat stimuli, and to algogenic substances in 1977 (J. Physiol. 273:179-194). These receptors play roles in pain and cardiovascular/respiratory responses during exercise. Later we developed delayed onset muscle soreness (DOMS) model in rats and showed augmented mechanical sensitivity of muscle thin-fiber receptors in this model. We proposed two pathways involving nerve growth factor and glial cell-line derived neurotrophic factor in the development and maintenance of DOMS and found both neurotrophic factors interact synergistically at the primary afferent level. In addition, we found a brand-new sensitizing mechanism of mechanical response by acid in cultured primary afferent neurons, namely, via extracellular matrix proteoglycans. There are still many points to be clarified in muscle thin-fiber receptors and their functional roles, and more studies are needed for their thorough understanding.

### [2S10m-03]

### Discovery of cerebellar LTD and LTP

*Masaki Sakurai[1] ([1]*Department of Physiology, Teikyo University School of Medicine*)

Each Purkinje cell (PC) receives two types of excitatory inputs: numerous parallel fibres (PFs) and a single climbing fibre (CF). Marr (1969) and Albus (1970) proposed the learning hypothesis with plasticity assumption that PF-PC synaptic efficacy is modified by instruction signals conveyed by CF. There was substantial controversy about the hypothesis through 1970s, during which Ito's group had supported it in the studies of the vestibulo-ocular reflex (VOR) adaptation.

At the dawn of 1980s, Ito shifted his target from VOR to direct testing of the hypothesis, based on the knowledge learned from VOR studies. We activated PFs (via mossy fibres (MFs)) and CF and recorded PC firings. We found that conjunctive stimulation of MF-PF and CF or application of glutamate along with CF stimulation produced long-term depression (LTD) of PC response to MF-PF activation or glutamate sensitivity of PCs. This study1 presented first, direct evidence for the basic assumption of the hypothesis.

I started experiments using in vitro slice preparation in its early days to provide more direct evidence for the plasticity and to pave the way for the further mechanistic studies. Paired stimulation of PFs and CF in vitro produced LTD of PF-EPSPs recorded from PC dendrites for more than 1 h2, which provided convincing evidence for the plasticity. I also found repetitive stimulation of PFs alone gave rise to long-term potentiation (LTP)2, thus showing bidirectional modifiability of PF-PC synapses .

1) Ito M, Sakurai M & Tongroach P: Climbing fibre induced depression of both mossy fibre responsiveness and glutamate sensitivity of cerebellar Purkinje cells. J. Physiol. 324, 113-34. 1982
2) Sakurai M: Synaptic modification of parallel fibre – Purkinje cell transmission in in vitro guinea-pig cerebellar slices. J. Physiol. 394:463-80, 1987

### [2S10m-04]

### Hair Cell Mechano-Electrical Transduction

*Harunori Ohmori[1] ([1]*Kyoto University*)

A series of my hair cell studies were published on The Journal of Physiology (1984~1994). On the MET, the stepwise current was detected when the hair bundle was mechanically stimulated in the whole cell clamped isolated hair cell (Ohmori 1985). The step conductance was 50~100 pS, and Ca2+ was highly permeable through the MET channel (4 times of K+) which had nearly equal permeability to alkaline cations (Px/PCs was 1~1.4). Stimulation of the hair bundle of different length at different heights showed that the angular displacement of the hair bundle was crucial in gating of the MET channel (Ohmori 1987). A possible localization of MET channel was shown as the rise of fluorescence by Ca2+ influx and the quenching of fluorescence by Mn2+ near the base of the hair bundle using fura-2 imaging (Ohmori 1988). Release of glutamate for afferent transmission was detected by NMDA receptors using the granule cell in the cerebellar culture, on depolarization of the hair cell (Kataoka Ohmori 1994). The hair cell membrane had Ca2+ current, Ca2+-activated K+ current, anomalous rectifier K+ current but no Na+ current, and a high resistance (4 Gohm) about the resting potential region (Ohmori 1984).

### [2S10m-05]

### Biophysical characteristics of the cardiac Na/K pump

*Akinori Noma[1] ([1]*Research Organization of Science and Technology, Ritsumeikan University*)

Many significant developments in the cardiac electrophysiology have been achieved after the introduction of the patch clamp technique and the single cardiac myocyte preparations in the 1980s. These new methods allowed experimental intervention of the intracellular medium, which was almost impossible in the traditional multicellular cardiac preparations. The activity of the ion transporters such as Na/K pump and the Na/Ca exchange are mainly regulated by concentrations of intracellular ions and ATP and related substrates. Indeed, dissection of the INa/K as well as INa/Ca from the whole cell current enabled clarification of the biophysical characteristics of the ion transporters. The findings in electrophysiological experiments were finally used to determine individual parameters of the theoretical biophysical models. Thus, the realistic model equations are now incorporated in various mathematical models of cardiac myocytes. In near future, feasible mathematical models will be available in clarifying mechanisms underlying cardiac cell activities under various experimental as well as pathophysiological conditions. In my presentation, key experimental findings and simulation of cell responses will be demonstrated by using the human ventricular cell model.

### Symposium

[2S02a]
Committee for Research Ethics
**Development and ethics of medical care and research - A review of the history of medical ethics in Japan**

March 15 (Wed.), 15:30 - 16:20, Room 2

### [2S02a-01]

**Development and ethics of medical care and research - A review of the history of medical ethics in Japan -**

*Fumio Eto ([1]*Japan Academy for Comprehensive Rehabilitation*)

### Symposium

[2AS03a]
Committee for 100th anniversary
**AI technology pioneers a new era of medicine, physiology, and life sciences**

March 15 (Wed.), 14:20 - 16:20, Room 3

### [2AS03a-01]

### AI-based pattern recognition for complex dynamics

*Gouhei Tanaka[1] ([1]*International Research Center for Neurointelligence, Institutes for Advanced Study, The University of Tokyo*)

In recent years, artificial intelligence (AI) has been becoming a ubiquitous technology in medical, physiological, and life science. However, it is still not fully clear what kind of AI methods are suited for specific issues in general. Reservoir computing (RC) is one of the machine learning frameworks suited for dealing with time series data, which requires much less computational time than deep learning. Recently, advanced RC models and methods have been intensively proposed to enhance their computational ability. For instance, we proposed a diverse-time-scale RC model which enables an improvement of the prediction performance for complex multiscale dynamics, inspired by the diversity of biological neurons in the brain. Considering that the brain exhibits fast fluctuations in neuronal activities and slow variations in brain waves, the proposed model is thought to be useful for treating interactions between them. The possible RC applications include prediction and classification with EEG, fMRI, ECG, and EMG signals. In this presentation, the fundamental concept of RC is introduced, some applications are demonstrated, and future directions toward AI-based healthcare are discussed.

### [2AS03a-02]

### Cerebral Cortex and Artificial Intelligence

*Kenichi Ohki[1] ([1]*The University of Tokyo*)

The human cerebral cortex contains as many as 180 areas, and the hierarchical and parallel information processing by thousands of precise neural circuits connecting these areas is the basis for the complex and flexible intelligence of our brain. Although studies of neural circuit development to date have investigated in detail the formation of neural circuits from the retina to the primary visual cortex, the mechanism by which the connections between cortical areas are wired precisely and without confusion within the three-dimensional brain has remained unknown. We used the mouse visual system to elucidate the mechanism by which the connections between the numerous visual cortical areas are formed in a short period of time. First, we found that the pathways connecting the retina to the many visual cortical areas are formed first, before the formation of the inter-areal connections. Furthermore, the spontaneous retinal activity propagating along this pathway conveys retinal location information to visual areas, which then act as an instruction signal to form connections that precisely link the retinotopically corresponding parts of visual cortical areas. This study not only clarified the formation mechanism of inter-areal connections in the cerebral cortex, but will contribute to the development of brain-inspired artificial intelligence.

### [2AS03a-03]

### Patient-specific modeling of signaling networks for stratification of human diseases

*Mariko Okada[1] ([1]*Institute for Protein Research, Osaka University*)

Patient heterogeneity makes disease treatment and drug development difficult and costly. Therefore, there is an urgent need to develop computational methods to identify prognostic and predictive markers for individual patient treatment and drug development. Mechanistic descriptions of gene network followed by the computational simulations are considered to be one of the approaches to simultaneously elucidate gene regulatory mechanisms and drug responses. Therefore, we developed a computational framework Pasmopy (Patient-Specific Modeling in Python) for stratification of patients based on *in silico* signaling dynamics. Using this framework, we constructed a model of the ErbB receptor - c-Myc signaling network, trained it with four breast cancer cell lines, and performed simulation of 377 breast cancer patients using TCGA (The Cancer Genome Atlas) transcriptome datasets. The temporal dynamics of each patient's signaling activity was found to better predict prognosis and sensitivity to kinase inhibitors in triple negative breast cancer than the standard PAM50 panel. Our approach can be applied to other types of diseases.

### [2AS03a-04]

### Development of Technologies for Reducing Risks of Lifestyle-Related Diseases Using Large-Scale Health Checkup Data and Future Prospects

*Takahiro Tanaka[1], Kenichi Doniwa[1], Masahiro Ozawa[1], Chenyuan Xu[1], Kosuke Haruki[1], Hideyuki Nakagawa[1], Maiko Yagi[2], Masashi Ebisawa[2], Taihei Yamaguchi[2]
([1]*Toshiba corporation, Corporate research & development center,* [2]*Toshiba corporation, Corporate technology planning div.*)

Toshiba has accumulated large-scale health checkup data from employees and their dependents over multiple years with few missing data. These data are linked to health insurance claims, making them very useful for analysis. Using this set of big data, we have developed technologies for accurately predicting the risks of five lifestyle-related diseases within the following 6 years by using random survival forests, as well as for recommending lifestyle changes to effectively reduce the risks of these diseases. The technologies are widely applicable because they do not require health insurance claims as input, and they are expected to lead to behavioral change because they recommend lifestyle changes that minimize the number of areas that need improvement. These technologies are expected to support corporate health management and reduce medical costs. In addition, we are working with Johns Hopkins University to develop technologies for predicting the risks of cardiovascular diseases, and we are also collecting genetic data from our employees with the aim of realizing precision medicine that takes into account the genetic risks of developing diseases in the future.

### Symposium

### [2AS04a]
### Toward understanding the regulation of channel proteins by lipids or lipid-analogues and its importance in physiological context

March 15 (Wed.), 14:20 - 16:20, Room 4

### [2AS04a-01]
### Structures and gating of gap junction family proteins in phospholipids

*Atsunori Oshima[1,2,3] ([1]*Cellular and Structural Physiology Institute, Nagoya University,* [2]*Department of Basic Medicinal Sciences, Graduate School of Pharmaceutical Sciences, Nagoya University,* [3]*Institute for Glyco-core Research (iGCORE), Nagoya University*)

Gap junction channels form intercellular conduits with a large pore size whose closed and open states regulate communication between adjacent cells. We show the cryo-electron microscopy structures of *Caenorhabditis elegans* innexin-6 gap junction proteins in detergent or nanodisc. In a nanodisc-reconstituted structure of the wild-type innexin-6 (INX-6) hemichannel, flat double-layer densities obstruct the channel pore. The structure in detergent reveals the N-terminal funnels, suggesting an open form. Together with molecular dynamics simulations and electrophysiology functional assays, our results provide insight into the closure of the INX-6 hemichannel in a lipid bilayer before docking of two hemichannels. Pannexin (PANX) family proteins form large pore channels that mediate purinergic signaling. We present the high-resolution structures of human PANX1 in lipid nanodiscs to elucidate the gating mechanism and its regulation by the N-terminus in phospholipids. The wild-type channel in the apo state has an N-terminal funnel in the pore, but in the presence of the inhibitor probenecid, a cytoplasmically oriented N-terminus and phospholipids obstruct the pore. Functional analysis using whole-cell patch-clamp and oocyte voltage clamp showed that PANX1 lacking the N-terminus did not open and had a dominant-negative effect on channel activity, thus confirming that the N-terminal domain played an essential role in channel opening. Our observations demonstrate a possible functional gating mechanism of human PANX1 in a lipid bilayer. The dynamic N-terminal conformational change is associated with the lipid migration in and out of the pore for channel closure, which provides critical insight into understanding the gating mechanism of large pore channel family proteins.

### [2AS04a-02]
### Lipophilic potassium channel activators

*Fredrik Elinder[1] ([1]*Linköping University*)

Voltage-gated ion channels play essential roles in generating and shaping the electrical excitability of nerve and heart cells. While many pharmaceuticals cause their effects by blocking the ion-conducting pore to prevent the ion current, only a few exert their therapeutic effect by opening (or activating) the channel. Here, I will present data from different types of lipophilic compounds capable of activating voltage-gated potassium (Kv) channels. Negatively charged resin acids and polyunsaturated fatty acids have close interactions with the channel's positively charged voltage sensor, opening the channel by pulling the sensor to an activated position. This interaction occurs at the interface between the phospholipid bilayer and the channel protein. In contrast, warfarine-like tautomers activate Kv1.5 channels via close interactions with the linker connecting the voltage sensor and the channel's ion conducting pore domain. Characterizing different binding sites and compounds that use them on different ion channels will hopefully lead to better treatments for diseases caused by altered excitability.

### [2AS04a-03]
### Inverse effect of phosphatidylinositol 4,5-bisphosphate on diacylglycerol-activated TRPC channels

*Masayuki X. Mori[1] ([1]*University of Occupation and Environmental Health*)

A minor phospholipid, phosphatidylinositol 4,5-bisphosphate (PI(4,5)$P_2$ or $PIP_2$) regulates numerous membrane proteins, including diacylglycerol(DAG)-activated TRPC3/6/7 channels. These TRPC channels could negatively regulate by the reduction of $PIP_2$ caused by phospholipase C (PLC) digestion, thus their activity is autonomously controlled by the DAG and $PIP_2$ signaling. To understand this autonomous regulatory system, we have screened for $PIP_2$ binding sites within TRPC6 channels. Using voltage-sensitive phosphatase (DrVSP), we found that Arg437 and Lys442, located in the channel's pre-S1 domain/shoulder, are crucial for interaction with $PIP_2$. Further, the functional significance of $PIP_2$ binding to the pre-S1 shoulder was assessed for receptor-operated channel functions and cross-reactivity to DAG activation. In addition, neutralizing or negatively charged mutation of K771 in the distal TRP box reversed the effect of $PIP_2$ depletion from inhibiting to potentiating channel activity. This result suggests that TRPC6 possesses a robust polarity switch mediating the $PIP_2$-dependent effect. The polarity mechanism may contribute to modify the physiological context of these channels via post-translational modifications such as phosphorylation

### [2AS04a-04]
### Lipid metabolite-dependent channel regulation: lessons from the phototransduction cascade in *Drosophila*

*Takaaki Sokabe[1], Heather Bradshaw[2], Craig Montell[3] ([1]*Exploratory Research Center on Life and Living Systems (ExCELLS),* [2]*Indiana University,* [3]*University of California, Santa Barbara*)

*Drosophila* phototransduction represents a classical model for signaling cascades that culminate with activation of TRP channels. It is established that light stimulation of rhodopsin and engagement of Gq and phospholipase C beta (PLC) are required for TRPC channels in photoreceptor cells, however, the key lipids produced after PLC stimulation and the gating mechanisms of the TRPC channels are still under debate. Here, we found that light increased the levels of an abundant endocannabinoid, 2-linoleoyl glycerol (2-LG) *in vivo*. The elevation in 2-LG strictly depended on the PLC and diacylglycerol lipase encoded by *norpA* and *inaE*, respectively. This endocannabinoid facilitated $Ca^{2+}$ influx both in *Drosophila* and mammalian TRPC channels *in vitro*, and in dissociated photoreceptor cells from compound eyes. A previous study reported that a mechanical deformation of plasma membrane upon $PIP_2$ degradation contributed to the channel activation. We further demonstrated that 2-LG and the mechanical stimulation cooperatively activated TRPC channels in photoreceptor cells. We will discuss the physiological relevance of the endocannabinoid on TRPC channel regulation in photoreceptor cells and possible conserved mechanisms in other cell types.

### [2AS04a-05]
### Ion channel regulation by phosphoinositides phosphatase and its voltage-dependence in mice sperm flagellum

*Takafumi Kawai[1], Yasushi Okamura[1] ([1]*Graduate School of Medicine, Osaka University*)

$PIP_2$, a type of phosphoinositides, plays diverse and crucial roles in cellular physiology. Voltage-sensing phosphatase (VSP), which was first identified in seq squirt, *Ciona intestinalis*, is an interesting voltage-sensing protein, because it shows phosphatase activity toward $PIP_2$ in response to the membrane potential. Previously, we reported that VSP-deficient sperm show severe defect in their motility after capacitation, resulting in significant reduction in success rate of fertilization in *in vitro* fertilization experiment. Electrophysiological analysis indicated that $K^+$ current that derived from sperm specific $K^+$ channel, Slo3, is regulated by phosphoinositides phosphatase function of VSP. Interestingly, we observed that the polarized $PIP_2$ distribution is generated by VSP in sperm flagellum. Our results indicate that VSP optimizes $PIP_2$ distribution so that Slo3 appropriately functions. In spite of the important function of VSP in sperm physiology, we still do not know how and when such specialized $PIP_2$ distribution is formed by VSP activity. In the present study, we report the maturation-dependent VSP activity by examining PtdIns(4,5)$P_2$ level of sperm at different maturation stages. We also discuss the detailed mechanism how membrane potential regulates mouse VSP activity and how it is important for regulating VSP activity during sperm maturation.

### Symposium

[2S05a]

### New roles of hormones that contribute to glucoregulation and diabetes

March 15 (Wed.), 14:20 - 16:20, Room 5

### [2S05a-01]

### Physiological roles of glucagon and diabetes mellitus

*Yoshitaka Hayashi[1] ([1]*Department of Endocrinology, Research Institute of Environmental Medicine, Nagoya University*)

The major physiological role of glucagon has been regarded as to elevate blood glucose levels. However, mice deficient in glucagon gene or proglucagon, a precursor of multiple peptides including glucagon and GLP-1(GCGKO: glucagon gene knockout mice), are normoglycemic but display hyperaminoacidemia. On the other hand, mice deficient in glucagon receptor are hypoglycemic with increased plasma glucagon and GLP-1 levels. Phenotypic analyses of various animal models with glucagon deficiency suggest that regulation of amino acid metabolism is more essential as a physiological role of glucagon than regulation of glucose metabolism. Accumulating data indicate presence of mutual feedback control between liver and islet alpha cells: whereas glucagon stimulates amino acid catabolism in the liver and lower plasma amino acid levels, blocking glucagon action increases plasma amino acid levels and stimulate islet alpha cell proliferation. Interestingly, clinical studies in Europe have shown that subjects with insulin resistance harbor both hyperglucagonemia and hyperaminoaciemia, suggesting that above mentioned feedback control is impaired. In this section, regulation of amino acid by glucagon and possible treatment of hyperglucagonemia in patients with diabetes mellitus will be discussed.

### [2S05a-02]

### Genetically induced immortalization of *Ppy*-expressing cells results in pancreatic ductal adenocarcinoma

*Ofejiro Blessing Pereye[1], Takashi Sato[1], Yuko Nakagawa[1], Ayako Fukunaka[1], Akihisa Fukuda[2], Hiroki Mizukami[3], Yoshio Fujitani[1] ([1]*Lab of Developmental Biology & Metabolism, Institute for Molecular & Cellular Regulation, Gunma University,* [2]*Department of Gastroenterology and Hepatology, Kyoto University Graduate School of Medicine,* [3]*Department of Pathology and Molecular Medicine, Hirosaki University Graduate School of Medicine*)

The *Ppy*-gene encodes the pancreatic polypeptide (PP) secreted by PP cells located in the islet periphery. The pancreatic islet cells consist of *Ppy* lineage and non-*Ppy*-lineage cells. *Ppy*-lineage β-cells are immature when compared to non-*Ppy* lineage β-cells. For a detailed characterization of *Ppy* cells, we aimed to genetically immortalize *Ppy* cells for the establishment of a *Ppy* cell line. To do that, we generated a mouse model which harbors the SV40 Large T oncogene under the control of the *Ppy* promoter. Surprisingly, SV40 activation resulted in the malignant transformation of *Ppy* cells. Mice had reduced blood glucose and body weight, islet depletion, and developed aggressive pancreatic ductal adenocarcinoma (PDAC) at 4 weeks after birth, having a life span of 4-6 weeks. *Ppy*-expressing cells have been shown to occupy the islets and can secrete insulin following induced β-cell ablation in an experimental diabetes model. This suggests their plasticity and regenerative capability and could be susceptible to malignant transformation as tissue regeneration has been linked with malignancy and cancer. We establish and characterize a mouse model with PDAC originating from *Ppy* cells of the pancreas. This study revealed the endocrine pancreas may be one of the origins of PDAC and could provide insights into studying pancreatic carcinomas originating from the endocrine pancreas

### [2S05a-03]

### The role of the FGF21-oxytocin system in the appetite alteration in diabetes and obesity

*Sho Matsui[1] ([1]*Graduate School of Agriculture, Kyoto University*)

We previously reported that simple sugar intake is regulated by the hepatokine fibroblast growth factor 21 (FGF21) and hypothalamic oxytocin (Oxt) neurons. Simple sugar ingestion stimulates FGF21 secretion, and it activates a part of the hypothalamic Oxt neurons. The secreted Oxt within the central nervous system acts on the Oxt receptor and suppresses simple sugar intake. In this symposium, we will describe this regulatory mechanism and introduce its pathophysiological significance. Moreover, we identified food ingredients that strongly generate the secretion of FGF21. Although rare sugars have a sweet taste similar to that of other simple sugars, such as sucrose, they are not easily metabolized *in vivo*, thereby leading to a decrease in the caloric intake. Therefore, rare sugars are drawing attention as a food component with anti-diabetic and anti-obesity effects. We have discovered that certain rare sugars strongly induce the expression and secretion of FGF21. We would like to also introduce these latest research findings at the symposium.

### [2S05a-04]

### Role of intestinal hormone GIP in fat intake and obesity

*Tomonobu Hatoko[1], Norio Harada[1], Nobuya Inagaki[1] ([1]*Department of Diabetes, Endocrinology and Nutrition, Kyoto University Graduate School of Medicine*)

Intestinal hormones are secreted from enteroendocrine cells by the stimulation of nutrients and exert several physiological effects related to energy regulation, such as the decomposition, absorption and accumulation of energy, and appetite regulation. Glucose-dependent insulinotropic polypeptide (GIP) is an intestinal hormone secreted from enteroendocrine K cells and induces glucose-dependent insulin secretion. GIP secretion is strongly stimulated by fat intake and increased GIP secretion affects energy accumulation in adipose tissue. We clarified that free fatty acid receptor GPR40 and GPR120 are essential for GIP secretion in response to a single intake of fat and that transcription factor Rfx6 and Pdx1 are involved in GIP production under high-fat diet (HFD) feeding condition by increasing GIP gene expression. K cell number remained unclear because two-dimensional imaging using immunohistological analysis cannot show the precise number of cells in intestine. We clarified not only intestinal morphology and the number of intestinal epithelial cells but also the precise K cell number in normal mice and the change of those factors in control-fat diet-fed lean mice and HFD-fed obese mice using tissue optical clearing and three-dimensional imaging. Therefore, fat intake induces GIP hypersecretion by increasing K cells number and enhancing GIP production and GIP secretory signals in K cells. Physiological enhancement of GIP activity induces obesity and insulin resistance under HFD intake.

### Symposium

[2S06a]
Cooperation with Other Societies Committee
**Evolving imaging technologies: novel probes and their applications in physiology**

March 15 (Wed.), 14:20 - 16:20, Room 6

### [2S06a-01]

### Molecular evolution paves the way for innovations in fluorescent protein-based sensors

*Tetsuya Kitaguchi[1] ([1]*Tokyo Tech*)

I have been developing several fluorescent protein-based sensors with the thought, “I want to see everything in the cells”. Fortunately, I have been able to realize some of my wishes despite a lot of difficulties and troubles. About the development of fluorescent protein-based sensors, I consider the length of the binding protein for target molecules and the position of its insertion into the fluorescent protein, but it is difficult to accomplish a sensor with a large signal response by itself. I have developed a unique method to increase the response by combining molecular evolution and semi-rational molecular design obtained in the developing process for these sensors. However, it is still far from my initial wishes. This is because if the binding protein for the target molecule is unknown or does not exist in the first place, it is impossible to develop the sensor. Therefore, I wondered if it is possible to design and develop a sensor with high specificity and affinity, and I came up with the idea of applying antibodies to molecular recognition domain. In this presentation, I will introduce the unique method for freely developing fluorescent protein-based sensors, leading to pave the way for visualizing a myriad of molecules of interest in the living cells.

### [2S06a-02]

### Activatable Raman probes using aggregate formation for selective imaging of enzyme activities

*Mako Kamiya[1] ([1]*Tokyo Institute of Technology*)

Detecting multiple enzyme activities with high spatial specificity will expect to reveal biological phenomena more deeply, and Raman imaging is promising modality due to its multiplexing capability. We previously developed activatable Raman probes based on 9CN-pyronin scaffold for simultaneous detection of plural enzyme activities in live-cultured cells. However, these probes were not able to distinguish target enzyme expressing cells or region because the hydrolysis products of probes tend to leak out from target cells. Here, we developed novel Raman probes based on 9CN-rhodol scaffold which have improved ability of cellular retention by aggregate formation. We first show that 9CN-rhodols has higher tendency to aggregate in aqueous solution than 9CN-pyronins, and found 9-CN-JCR as a promising scaffold of activatable Raman probes. Further, SRS signal intensity and aggregate forming ability of 9CN-JCR based probes is activated upon reaction with target enzymes, so that the hydrolysis product forms aggregate to be retained in the target cells. The isotope-edited probes allowed us to perform simultaneous detection of three different enzyme activities in live cells, and to distinguish regions with target enzyme activity in live Drosophila tissues.

### [2S06a-03]

### Development of calcium sensor proteins for imaging intraorganellar calcium dynamics and application to in vivo imaging

*Kazunori Kanemaru[1] ([1]*Nihon University School of Medicine*)

Spatiotemporal $Ca^{2+}$ dynamics in cytosol and organella provide important clues to understand ongoing cellular activity and function. Our research group has developed tools and methodologies to analyze the $Ca^{2+}$ dynamics. In this presentation, I will introduce the tools, methods and applications, mainly focused on (1) intraorganellar $Ca^{2+}$ sensor proteins and (2) in vivo cytosolic $Ca^{2+}$ imaging technique for peripheral organs. The first topic (1) is about intraorganellar $Ca^{2+}$ sensor proteins, Calcium-measuring organelle-Entrapped Protein IndicAtors (CEPIA). CEPIA enables to visualize intraluminal $Ca^{2+}$ signals in the endoplasmic reticulum and mitochondria with high spatiotemporal resolution. CEPIA allowed to monitor wave-like propagations of $Ca^{2+}$ release in the ER, ER $Ca^{2+}$ oscillation which is inversely correlated with kinetics of cytosolic $Ca^{2+}$ oscillation, and localized mitochondrial $Ca^{2+}$ transients. Moreover, simultaneous $Ca^{2+}$ imaging among cytosol, mitochondria and ER is also possible with multiple-colored variations of CEPIA. The second topic (2) is about cytosolic $Ca^{2+}$ imaging in peripheral tissues in intact mice. We are trying to establish in vivo $Ca^{2+}$ imaging method for monitoring pancreatic β cells or liver hepatocytes, using transgenic mouse lines expressing cytosolic $Ca^{2+}$ sensor protein, yellow cameleon-Nano 50 (YC-Nano50). Ratiometric property of YC-Nano50 enabled to monitor $Ca^{2+}$ dynamics clearly in these cells. These methods will contribute to obtain deeper insight into cellular function in vivo.

### [2S06a-04]

### Lattice Light-Sheet Microscopy Opens Up New Possibilities for Live Imaging

*Yuko Mimori-Kiyosue[1] ([1]*RIKEN Center for Biosystems Dynamics Research (BDR)*)

The Lattice Light-Sheet Microscope (LLSM) is a high-resolution light-sheet microscope that uses an ultra-thin light sheet less than 1 µm thick generated by applying Bessel beam technology. By arranging massive Bessel beams at optimum intervals, side lobes are canceled to eliminate background light and reduce phototoxicity, while at the same time increasing scanning speed, enabling 3D live imaging with high resolution, high speed, and low phototoxicity. With this technology, high spatiotemporal resolution images with accurate 3D information can now be collected in quantities hundreds to thousands of times greater than previously possible.
On the other hand, analysis of such a large amount of image data has become difficult using conventional methods. Therefore, our laboratory is promoting the application of LLSM to life science while developing image and data analysis methods. In this talk, I would like to introduce the behavior of cellular fine structures such as cytoskeletons, plasma membranes, organelles, secretory granules, and extracellular vesicles revealed by LLSM, LLSM image analysis, and new developments in various research fields that are expanding with these technologies.

### [2S06a-05]

### Studies on muscle cells heating up

*Madoka Suzuki[1] ([1]*Osaka Univ.*)

Cellular thermogenesis is the internal source of heat that is required to maintain our body temperature above the environmental one. It is also necessary to correctly regulate the heat power to avoid high body temperature. Fluorescence temperature imaging has allowed us to examine the thermogenesis in individual cells with spatial and temporal resolutions at the subcellular level. In this presentation, I will introduce our recent studies where we combined the fluorescence temperature imaging with optical heating methodologies to examine contributions of the thermogenesis on temperature-sensitive pathways in muscle cells. Particularly, we consider malignant hyperthermia that is an unstoppable thermogenesis in skeletal muscle due to abnormal $Ca^{2+}$ homeostasis. I will demonstrate that a $Ca^{2+}$ release channel in skeletal muscles, ryanodine receptor type 1 (RyR1), is activated by heating. We denoted this novel response as "heat-induced $Ca^{2+}$ release (HICR)." RyR1 mutants implicated in malignant hyperthermia are more heat sensitive than their WT, thus possibly form a positive feedback to cause unstoppable thermogenesis during malignant hyperthermia.

### Symposium

### [2AS07a]
**Multimodal approach for age-related decline in biological function and anti-aging**

March 15 (Wed.), 14:20 - 16:20, Room 7

### [2AS07a-01]
**Resistance exercise training as a senolytic treatment for sarcopenia**

*Shuichi Machida[1] ([1]*Graduate School of Health and Sports Science, Juntendo University*)

Aging is associated with skeletal muscle decline (sarcopenia), which is defined as reductions in skeletal muscle mass, strength, and physical function. Skeletal muscles are comprised of aggregated muscle fibers which are classified as slow-twitch and fast-twitch muscle fibers based on their contractile characteristics. A typical feature of sarcopenia is selective atrophy in the fast-twitch muscle fibers; additionally, cellular senescence is also believed to be associated with sarcopenia as the senescent cells release senescence-associated secretory phenotype (SASP) and lead to chronic inflammation. On the other hand, hypertrophy of muscle fibers due to resistance exercise training is dominant in fast-twitch muscle fibers. Resistance exercise training improves skeletal muscle mass, and suppresses senescent cells and SASP in aging skeletal muscle. Therefore, resistance exercise training is recommended as a preventive measure for counteracting sarcopenia. In this symposium, I will present basic research findings on the mechanisms of skeletal muscle mass increase through resistance exercise training at the molecular, cellular, and tissue levels using rat models of sarcopenia and menopause. I will also attempt to make recommendations for resistance exercise training that take aging and gender differences into consideration as a measure to prevent sarcopenia.

### [2AS07a-02]
**Possibility of a new anti-aging mechanism of vitamin C**

*Ayami Sato[1] ([1]*Tokyo Metropolitan Institute of Gerontology*)

Vitamin C (VC: L-ascorbic acid) is known as one of the most effective nutrient candidates for anti-aging. Our laboratory has demonstrated that long-term VC shortage shortens lifespan in mice. VC is believed its anti-aging effect because of the strong antioxidant function. However, the anti-aging mechanism of VC is not fully understood yet. Notably, it was newly found that VC regulates epigenetics. Epigenetic changes are hallmarks of aging. Currently, the epigenetic clock, also known as DNA methylation age, has been developing to predict biological age. DNA methylation regulates gene expression. Early studies suggested a loss of global methylation during aging. However, recent studies indicate that either hypermethylation or hypomethylation may occur in different areas of the genome change with age in different tissues. Moreover, the epigenetic changes during aging can be modified by nutrients. Here, we introduce our study of the epigenetic regulation by VC in epidermal keratinization. We revealed that VC is a cofactor of DNA demethylation enzyme as a model. A project studying the effect of VC on epigenetic changes during aging in mice is ongoing. These studies lead to the elucidation of epigenetics-mediated aging mechanisms and the aging regulation.

### [2AS07a-03]
**Hypothalamic regulation of aging and longevity through sleep control**

*Akiko Satoh[1,2] ([1]*Geroscience Research Center, National Center for Geriatrics and Gerontology*, [2]*Institute of Development, Aging and Cancer, Tohoku University*)

Aging has significant impacts on sleep, resulting in increases in sleep fragmentation and sleep onset latency, and a decrease in sleep quality in mammals. We have found that diet restriction (DR), a dietary regimen well known to delay the aging process and to extend lifespan in several organisms, can dramatically attenuate some of age-associated sleep alterations. For example, DR significantly reduces the levels of age-associated sleep fragmentation. Therefore, DR may delay the aging process and extend lifespan by critically altering sleep control in aged animals. To further elucidate mechanisms by which DR ameliorates age-associated sleep alterations, we focus on the gene called PR-domain containing factor 13 (Prdm13). Prdm13 is one of downstream genes of Sirt1 and is exclusively expressed in the dorsomedial hypothalamus (DMH). The DMH has been shown to control age-associated physiological changes and longevity through Sirt1. We have recently found that conditional DMH-specific *Prdm13*-knockout mice mimic age-associated sleep alterations. We are currently testing whether Prdm13 is necessary to promote DR-induced sleep phenotypes. In this talk, I will discuss about the role of DMH neurons on age-associated sleep alterations and how poor sleep quality promotes age-associated pathophysiology in peripheral organs/tissues and ultimately affects longevity.

### [2AS07a-04]
**Aging of olfaction and cognitive function**

*Sae Uchida[1] ([1]*Tokyo Metropolitan Institute of Gerontology*)

A decline in olfactory function is an early symptom of Alzheimer's disease. The olfactory bulb, the first processing station of olfactory information in the brain, receives cholinergic basal forebrain input, as does the neocortex contributing cognitive function. We conducted (1) basic study on the role of cholinergic input to the olfactory bulb in rats, (2) a pilot study on the relationship between olfaction and cognitive function in older adults. (1) In anesthetized rats, olfactory stimulation increased blood flow in the olfactory bulb. Activation of nicotinic acetylcholine receptors ($\alpha4\beta2$ subtype) by nicotine potentiated the blood flow response in the olfactory bulb. This indicates that nicotinic cholinergic transmission enhances olfactory sensitivity. (2) In the community-dwelling older adults (n=12, 70-90 years), participants with a higher olfactory threshold for rose odor declined more in attentional ability and discrimination ability. Because both attention and discrimination relate to the basal forebrain cholinergic system, our results suggest that olfactory impairment links to the decline in cognitive function relating the cholinergic system.

### [2AS07a-05]
**Involvement of ROS signal in regulation and aging of brain function**

*Sho Kakizawa[1] ([1]*Grad. Sch. Pharmaceu. Sci., Kyoto University*)

Reactive oxygen species (ROS) is a redox-signaling molecule, and indicated to be involved in various pathophysiological events in organisms, such as aging and lifestyle-related diseases. In addition, recent studies demonstrate expression of ROS synthases (NADPH oxidase (Nox) and dual oxidase (Duox)) in various tissues including brain regions. These facts suggest regulated production of ROS and also involvement of ROS in both physiological and pathophysiological functions in brain. However, molecular mechanisms of ROS related aging and physiological functions of endogenous ROS in brain systems are yet to be determined. In this symposium, I introduce recent studies demonstrating involvement of ROS signals in long-term depression (LTD) in the cerebellum, a cellular basis for cerebellar-dependent motor learning. Essential role of ROS signal in age-dependent decline in PF-LTP in aged cerebellum and antioxidant effects of polyphenols are also shown. Taken together, these observations suggest dual functions of ROS in physiological and pathophysiological events in brain systems.

### Symposium

[2S08a]
**Hyperadaptability for overcoming body-brain dysfunction.**

March 15 (Wed.), 14:20 - 16:20, Room 8

### [2S08a-01]

### Neural mechanisms inducing hyper-adaptation after tendon transfer in the upper limb of non-human primates.

*Roland Philipp[1], Uchida Naoki[2], Hara Yuki[3], Funato Tetsuro[4], Seki Kazuhiko[1]
([1]*National Institute of Neuroscience, Department of Neurophysiology, NCNP, Kodaira, Japan,* [2]*University of ElectroCommunications, Graduate School of Informatics and Engineering, Department of Mechanical and Intelligent Systems Engineering,* [3]*Department of Orthopaedic Surgery, NCNP, Kodaira, Japan,* [4]*The University of Electro-Communications, Chofu-shi, Japan*)

The human body changes gradually over decades of physical development and aging. It also changes transiently by traumatic injury like limb amputation and the surgical compensations to the affected body parts. The human central nervous system (CNS) is well capable to adapt to these bodily changes. Healthy children, adults, and the elderly, for example, make locomotive gait by operating their own body with different size and posture. Amputees are known to overcome the maladaptive reorganization of sensory-motor cortices i.e. phantom pain with an appropriate intervention and prostheses replacing hands are embodied in the CNS. However, little information is available about cortical and subcortical adaptations to this physically modified body and its underlying mechanisms. Here, we performed a tendon cross-union between the *Extensor Digitorum Communis* (EDC) and *Flexor Digitorum Superficialis* (FDS), an antagonistic muscle pair in the forearm controlling the fingers in order to study to which extend two monkeys (*Macaca fuscata*) recover and perform in a simple grasping task. Furthermore, we studied how muscle synergies adapt to changes in the musculoskeletal system particularly with respect to the time course following those changes. Eventually, we hope for improvements in current and novel rehabilitation programs and fostering our understanding of cortical and subcortical plasticity.

### [2S08a-02]

### Induction of hyper-adaptation through sensory and motor interventions

*Hironobu Osaki[1] ([1]*Doshisha Univ.*)

The sensory-motor system receives information from the external environment and engages in goal-directed behavior, such as avoiding harmful conditions. The primary somatosensory cortex of mice receives predominantly nociceptive input in the dysgranular region (Dys) adjacent to the barrel field. Consequently, selective optogenetic inhibition of Dys successfully reduced pain-like behavior, such as escaping from noxious stimuli (Osaki et al., 2022). It is unclear, however, whether continuous suppression or lesion of the somatosensory cortex produces long-lasting analgesia. Studies on mice using lesion techniques such as infarction or optogenetic inhibition indicate that suppressed function would recover within a few days or weeks. Restoration of lost function would occur as a result of plastic adaptations in regions connected to the lesioned site. In this presentation, I will show examples from our studies to illustrate what occurred during long-term selective inhibition and lesion of the cerebral cortex. In addition, I will discuss the use of sensory and motor interventions to promote hyper-adaptation. (COI: NO)

### [2S08a-03]

### Overcoming stroke learned non-use through hyper-adaptability?

*Rieko Osu[1] ([1]*Waseda University*)

Hand choices—deciding which hand to use to reach targets—represent continuous, daily, unconscious decisions. Hand choices are important for stroke hemiparetic patients since patients must use the paretic hand in their daily life to recover its function. However, patients prefer to use the non-paretic hand because it gives more rewards, such as quick and accurate reach to food (learned non-use), which will deprive the chance of learning paretic hand function. Therefore, it is crucial to increase the use of the paretic hand, in addition to improving its function, in the subacute rehabilitation process. In this talk, I will introduce the experiments showing that non-invasive stimulations of the brain and the peripheral nerves can bias the hand choice of healthy participants. Our results suggest the possibility of intervening in the unconscious decision-making of hand choices, expecting future rehabilitation applications for stroke patients.

### [2S08a-04]

### Computational approaches to neuromuscular learning: An autonomous machine learning approach

*Francisco Valero-Cuevas[1] ([1]*University of Southern California*)

Lifelong learning is a defining factor of neural systems. Understanding the mechanisms by which animals learn quickly, autonomously and driven by their own limited experience would empower approaches to behavior, disability and rehabilitation. This knowledge can also drive the emerging field of bio-robotics. We are developing "robots with a nervous system " by selecting fundamental neurophysiological mechanisms—as understood today—and implementing them as physiologically-faithful algorithms and circuits. I will present examples ranging from high-level algorithms for autonomous learning of locomotor and dexterous manipulation, to low-level spinal circuits for muscle tone and stretch reflexes. This will highlight the importance of brain-body co-evolution in biological systems that holds valuable lessons for robotics based on the co-design of learning algorithms, neuromorphic controllers and bio-inspired bodies.

References:Valero-Cuevas FJ, Erwin A Nature Machine Intelligence, 2022Kudithipudi D, et al. Nature Machine Intelligence, 2022Hagen DA et al. Frontiers in Neurorobotics, 2021Berry JA, Valero-Cuevas FJ. Artificial Life Conference Proceedings, 2020Marjaninejad A, et al. Nature Machine Intelligence, 2019

### [2S08a-05]

### Variability of motor commands facilitates motor learning

*Jun Izawa[1] ([1]*University of Tsukuba*)

Variabilities of motor movements are often troublesome for both the brain controlling the arm and for motor control scientists analyzing participants' movements. However, we found theoretical and empirical evidence that motor variabilities are essential for facilitating motor learning when structures of motor apparatus (e.g., descending pathway, musculoskeletal system, and motor task) are largely changed. In general, the degree of freedom of motor command space is much larger than that of the task space. Thus, for efficient motor learning, the observed task error must be transferred to the task-irrelevant motor command space efficiently. If the structure of the body and task is changed largely, the brain must re-learn this structure to map the task error efficiently to the motor commands space. In this framework, the update process of the cortical representation of motor error to be corrected can be modeled by linear recurrence relations. The solution of this update equation suggests that the covariance matrix of motor commands should be regular to make it converge to the ideal motor command errors. This suggests that the exploration across task-relevant and task-irrelevant dimensions are necessary for efficiently transforming the task error into motor commands. This prediction was further confirmed by a numerical simulation and by simple behavioral experiments of the de-novo motor learning task.

### Symposium

[2S09a]
### The frontier of gastrointestinal and vascular pathophysiology research associated with the intestinal environment

March 15 (Wed.), 14:20 - 16:20, Room 9

### [2S09a-01]
### Redox-dependent alternative internalization (REDAI) of GPCRs regulates colitis

*Kazuhiro Nishiyama[1], Akiyuki Nishimura[2,3,4], Kakeru Shimoda[2,3,4,1], Yuri Kato[1], Yoshito Kumagai[5], Takaaki Akaike[6], Philip Eaton[7], Koji Uchida[8], Motohiro Nishida[1,2,3,4] (*[1]Graduate School of Pharmaceutical Sciences, Kyushu University, [2]National Institute for Physiological Sciences (NIPS), National Institutes of Natural Sciences (NINS), [3]Exploratory Research Center on Life and Living Systems (ExCELLS), NINS, [4]Department of Physiological Sciences, SOKENDAI, [5]Faculty of Medicine, University of Tsukuba, [6]Graduate School of Medicine, Tohoku University, [7]The William Harvey Research Institute, Charterhouse Square, Barts and the London School of Medicine and Dentistry, Queen Mary University of London, [8]Graduate School of Agricultural and Life Sciences, The University of Tokyo*)

G protein-coupled receptors (GPCRs) play pivotal roles in converting physicochemical stimuli due to environmental changes to intracellular responses. After ligand stimulation, many GPCRs are desensitized and then recycled or degraded through b-arrestin-dependent internalization, an important process to maintain protein quality control of GPCRs. However, it is unknown how GPCRs with low β-arrestin sensitivity are controlled. Here we unmasked a b-arrestin-independent GPCR internalization, named Redox-dependent alternative internalization (REDAI), using b-arrestin-resistant purinergic $P2Y_6$ receptor ($P2Y_6R$). Natural isothiocyanates (ITCs) covalently bind with $Cys^{220}$ in the intracellular 3rd loop of $P2Y_6R$, and promote internalization and degradation of $P2Y_6R$ through ubiquitination of $Lys^{137}$ in the 2nd loop. $P2Y_6R$ is highly expressed in macrophage and pathologically contributes to the development of colitis in mice. Endogenous electrophiles, such as S-nitrosoglutathione, also induce $P2Y_6R$ degradation leading to anti-inflammation in macrophages. Prevention of $Cys^{220}$ modification on $P2Y_6R$ resulted in aggravation of the colitis. Accordingly, targeting REDAI on GPCRs will be a breakthrough strategy for the prevention and treatment of inflammatory diseases.

### [2S09a-02]
### Characterization of neuromuscular transmissions in the colon of inflammatory bowel disease model animals.

*Takahiko Shiina[1,2], Yasutake Shimizu[1,2] (*[1]Laboratory of Physiology, Joint Department of Veterinary Medicine, Faculty of Applied Biological Sciences, Gifu University, [2]Joint Graduate School of Veterinary Sciences, Gifu University*)

Inflammatory bowel disease (IBD) is a disorder of the gastrointestinal tract. During inflammatory processes, the regulation of gastrointestinal functions by the enteric nervous system is disrupted. We analyzed alteration of neuromuscular transmissions in the inflamed colon. We made rodent models of IBD by intraluminal application of trinitrobenzene sulfonic acid and assessed the mechanical responses induced by application of electrical field stimulation (EFS) in isolated segments of the distal colon. EFS evoked nitrergic relaxation followed by cholinergic and tachykininergic contractions, which were attenuated in the inflamed colon. Both the nitrergic relaxation and the cholinergic and tachykininergic contraction were recovered at post-inflammatory stage. In addition, non-tachykininergic and non-cholinergic components were also expressed. Next, we investigated whether probiotics affects the alteration of neuromuscular transmissions. Recovery of the nitrergic component was greater and appearance of non-tachykininergic and non-cholinergic excitatory components was less in the probiotics group than in the control group. These results suggest that colonic inflammation causes alteration of enteric neuromuscular transmissions, which can be affected by probiotics to influence the gut microbiome. (COI: Properly Declared)

### [2S09a-03]
### Protective role of the $M_3$ muscarinic acetylcholine receptor signaling in indomethacin-induced small intestinal injury

*Yoko Igarashi[1,2], Eikichi Ihara[2,3], Yoshihiro Ogawa[2] (*[1]Mochida Pharmaceutical Co., Ltd. Research Center, [2]Department of Medicine and Bioregulatory Science, Graduate School of Medical Sciences, Kyushu University, [3]Department of Gastroenterology and Metabolism, Graduate School of Medical Sciences, Kyushu University*)

Background: Given that $M_3$ muscarinic acetylcholine receptor ($M_3R$)-selective agents are unavailable, how $M_3R$ regulates intestinal epithelial barrier function (IEBF) remains unclear. Here, we used a novel highly-selective $M_3$ positive allosteric modulator PAM-369 to explore how $M_3R$ is involved in the regulation of IEBF and its pathophysiological role in non-steroidal anti-inflammatory drug (NSAID)-induced enteropathy. Methods: We evaluated the role of $M_3R$ in IEBF *ex vivo* by measuring short circuit current (Isc) of ileal epithelium with Ussing chamber system. We also examined if potentiation of $M_3R$ is protective for indomethacin-induced small intestinal injury *in vivo*. Results: Carbachol increased the Isc in a concentration-dependent manner. The carbachol-induced Isc increase was abolished by the receptor antagonist or by removal of extracellular $Cl^-$. PAM-369 enhanced the carbachol-induced Isc increase, which could contribute to enhanced IEBF. Oral administration of PAM-369 ameliorated extents of small intestinal injury in indomethacin-treated mice. Importantly, expression of $M_3R$ was significantly up-regulated, and PAM-369-induced Isc was augmented in indomethacin-treated mice compared to untreated mice. Conclusions: This study provides evidence that $M_3R$ plays a role in maintenance of IEBF. $M_3R$ is a promising target to treat or prevent NSAID-induced enteropathy.

### [2S09a-04]
### Effects of Lactulose and breast milk derived probiotics on colitis-associated carcinogenesis in mice

*Keizo Hiraishi[1], Xiaodong Li[2], Feiyan Zhao[3], Heping Zhang[3], Katsuya Hirano[2], Takayuki Fujita[1], Lin-Hai Kurahara[2] (*[1]Dept. Physiol., Sch. Med., Fukuoka univ., Japan, [2]Dept. Cardiovasc. Physiol., Fac. Med., Kagawa univ., Japan, [3]Key Laboratory of Dairy Biotechnology and Engineering, Ministry of Education, Inner Mongolia Agricultural univ., P.R.China*)

Milk derived oligosaccharides or breastfeeding reduced the risk of inflammatory bowel disease (IBD) according to numbers of global studies. Intestinal bacteria play important roles during the progress of inflammation and carcinogenesis. We investigated the effects of the milk derived oligosaccharide lactulose or breast milk-derived probiotic *Lactobacillus rhamnosus* M9 (Probio-M9) on colitis-associated tumorigenesis. An inflammatory colorectal cancer model was prepared by mouse administrated with azoxymethane (AOM) and dextran sulfate sodium (DSS), and treated with Lactulose or probio-M9. The AOM/DSS group showed weight loss, diarrhea, intestinal shortening, increased colon tumors, proliferating tumorigenesis, increased inflammation and fibrosis, upregulated macrophage cells in the subserosal layer of non-tumor areas. Lactulose or Probio-M9 treatment ameliorate inflammation and colorectal tumorigenesis, respectively. Both treatments showed greater potential for the restoration of gut microbial structure and diversity, which was reflected by the distance of samples between treated group and control groups. Our results showed milk-derived prebiotics and probiotics could reduce tumorigenesis by regulating the structure and composition of intestinal microbiota and improving the inflammation caused by AOM/DSS.

### [2S09a-05]
### Metabolic Syndrome and Dysbiosis: New Insight into the Pathogenesis of Hypertension

*Masashi Mukohda[1], Risuke Mizuno[1], Hiroshi Ozaki[1] (*[1]Okayama University of Science*)

A microbial imbalance (dysbiosis) is associated with several diseases including metabolic cardiovascular diseases. A fecal microbiota transplantation from hypertensive human donor to germ-free mice caused blood pressure elevation, suggesting that gut microbiome may mediate development of hypertension. However, the pathophysiological mechanism by which the dysbiosis increases blood pressure remains unclear. Some cohort studies showed an increasing number of gram-positive Streptococcus was found in the feces of hypertensive patient. We reported that plasma level of streptolysin O (SLO), a streptococcal exotoxin, was elevated in adult spontaneously hypertensive rat (SHR). Treatment with SLO impaired acetylcholine-induced endothelial-dependent vasorelaxation via PKCβ-mediated phosphorylation of endothelial nitric oxide synthase, possibly through TLR4. Intravenous administration of SLO in Wistar rat impaired an acetylcholine-induced decrease in blood pressure. In addition, long-term treatment with SLO impaired endothelial cell via increasing reactive oxygen species. Consistent with this concept, SHR exhibited increased intestinal permeability with decreased tight junction proteins. It was concluded that streptococcal exotoxin causes vascular endothelial dysfunction and may contribute to a dysregulation of vascular homeostasis and blood pressure control.

### Symposium

[2S01e]
Committee for Promotion of Physiome and Systems Biology
**Brain Computer Interface: Neurophysiological Perspectives**

March 15 (Wed.), 16:30 - 18:30, Room 1

### [2S01e-01]

### Physiological architecture of the mammalian inner ear and prospects of the cochlear implant

*Hiroshi Hibino[1], Takeru Ota[1], Hiroki Yamazaki[1], Satoyuki Kawano[1] ([1]*Osaka Univ.*)

Hearing loss affects 5−10 % of global population. This disease, which lowers quality of life and significantly increases the risk for dementia, stems primarily from damage of the cochlea in the inner ear. Sounds, when reaching the cochlea, induce nanoscale vibrations in the sensory epithelium. This motion is transduced by sensory hair cells in the epithelium into electrical signals, which are transmitted by neurons to the brain. Hair cells are electrically connected to a biological battery implemented in another cellular layer. In this cochlear system, the signals are amplified by the active motility of the hair cells and the battery, accounting for high sensitivity and sharp tuning of hearing. In this symposium, we will overview the physiological architecture and operating principles of the cochlea, which is composed of different types of highly differentiated cells. Cochlear prosthesis is an effective therapy for deafness and currently implanted in ~500,000 patients worldwide. In addition to the outline of this conventional approach, we will show the prototypes of a next-generation device and discuss the prospects.

### [2S01e-02]

### Cortical visual prosthesis that mimics the response and the communication in the visual neuronal networks

*Tetsuya Yagi[1], Yoshinori Takeuchi[2], Yuki hayashida[3] ([1]*Fukui Institute of Technology,* [2]*Kindai University,* [3]*Mie University*)

The sensory organs of human process and represent the information of ambient world and efficiently communicate with the brain in real time. To design the neuronal prosthesis that can restore useful and natural percepts to patients with severe sensory dysfunction, it is critical to consider physiological and anatomical properties of the sensory systems of the brain. Based on this requirement, we are designing a visual cortical prosthesis that mimics the computation and the communication in the visual networks of the brain. The prosthesis consists of a neuromorphic retina (NM retina) and wireless communication modules implemented with micro-processors. The NM retina provides spike responses mimicking retinal ganglion cells' to the communication module. The wireless communication modules compress the image information represented with spatio-temporal distributions of the spikes and communicate wirelessly each other. Simulated phosphen images taking account for visuotopic map of the primary visual cortex showed the designed prosthesis evokes image percepts effectively and efficiently with low power consumption, in other words low heat dissipation as required for implantable prosthetic devices.

### [2S01e-03]

### Sensorimotor Augmentation and Embodied Connection Using Tactile Interfaces

*Yoshihiro Tanaka[1] ([1]*Nagoya Institute of Technology*)

Tactile sensation is personal information, depending on our bodies as well as the objects touched. We developed a wearable tactile sensor that detects skin-propagated vibration allowing users to touch the object with their fingertips. Experiments showed that the skin vibration well-reflected subjective roughness ratings. Then, we proposed tactile sharing and enhancement with this sensor and vibrotactile actuators. One of the possible applications is remote palpation. An expert could evaluate the affected area by sharing the tactile sensation during the palpation for patients by another person in a remote area. Tactile sense furthermore contributes to manipulation. Some stroke patients cannot perceive tactile sense and hardly manipulate small and/or flexible objects. We developed a substitute tactile feedback system. The skin vibration on the fingertip is fed back to other parts (e.g. shoulder or head) where the tactile sensation can be perceived. Clinical tests showed potential effects for rehabilitation. Moreover, we have investigated body integration, in which multiple people control a single robot avatar, sharing sensorimotor controls. The effects of stabilization, concentration, and motion augmentation were demonstrated. Future applications and challenges of tactile interfaces for augmenting our abilities and inducing embodied collaboration will be discussed.

### [2S01e-04]

### BCI to explore brain representation of imagery

*Takufumi Yanagisawa[1,2] ([1]*Osaka University, Institute for advanced co-creation studies,* [2]*Osaka University Graduate School of Medicine, Department of Neurosurgery*)

Humans perceive visual and somatosensory sensations in response to external stimuli and can also imagine them in the absence of external stimuli. Neurophysiological understanding of the imagery is an important issue so that we can develop novel brain-computer interfaces (BCI) and treatment of neuropsychiatric disorders. We have developed a visual imagery BCI with that a patient implanted subdural electrodes on the visual cortex controls some external images based on the semantic contents of their imagined images. The imagined contents were decoded using a representational space to vectorize images of various semantic meanings based on the latent space of language AI (word2vec). The subjects were shown some images that were selected based on the semantic vector inferred from ECoG signals online. Four subjects succeeded to present some images on a monitor following three types of instructions by imagining images of the instructed meaning. Imagery BCI can visualize the mental contents and afford us to manipulate the corresponding cortical representation to unveil the neurophysiological properties of the imagery.

### Symposium

[2AS02e]
**pH and life on earth ; transient and persistent chronology of life adaptation and environments**

March 15 (Wed.), 16:30 - 18:30, Room 2

### [2AS02e-01]

### Organic compounds created by atmospheric $CO_2$

*Nobuaki Takahashi[1] ([1]*Kyoto University*)

### [2AS02e-02]

### Ocean acidification impacts on the future marine organisms and ecosystems

*Haruko Kurihara[1] ([1]*University of the Ryukyus*)

Ocean is now being quickly acidified by the increase of anthropogenic carbon dioxide in the atmosphere, which phenomenon is called as “Ocean acidification”. Ocean is absorbing about one third of the emitted carbon dioxide which dissolve into the seawater and release proton. The present ocean pH (8.1) has already decreased by 0.1 unit compared to the era of industrial revolution. According to the different $CO_2$ emission scenarios, ocean pH is expected to further decrease for about 0.3-0.4 units by the end of this century (IPCC 2021). As the seawater pH decrease, due to the change of carbon chemistry, the concentration of carbonate ion ($[CO_3^{2-}]$) will decrease which causes the decrease of calcium carbonate saturation (Ω). Ocean acidification is expected to affect the physiology of marine organisms, particularly the marine calcifiers such as corals, bivalves, sea urchins and crustaceans. Additionally, since those organisms includes several ecological functioning species and species that is consumed as a human food source, ocean acidification is now highly concerned to threat marine ecosystems and fisheries.Here in this presentation, first I will introduce several experimental studies evaluating how the marine organisms are expected to be affected by the ocean acidification at individual to molecular scale. Those includes physiological effects such as decrease on respiration, calcification and reproduction of the marine organisms. Additionally few examples showing effects of ocean acidification on gene expression will also be introduced. Secondly, $CO_2$ vent and enclosed bay field studies aiming to scale up the effects of ocean acidification at community and ecological scale will be introduced. Those sites which show high $CO_2$ concentration at natural condition can also be used as natural analogues to investigate if marine organisms have the potential to adapt or evolve when exposed to those conditions for a long-term. Additionally, interaction among organisms and interactive effects between other environmental factor such as high temperature or low oxygen will be addressed. Finally, interpretation of those effects to the human society and adaptation strategies for the future climate change will be discussed.

### [2AS02e-03]

### The history of ocean acidification and its impact on marine organisms in geological time: evidence from fossil foraminifera records

*Yoshimi Kubota[1], Katsunori Kimoto[2], Tatsuhiko Yamaguchi[1], Shunichi Kinoshita[1] ([1]*National Museum of Nature and Science,* [2]*Japan Agency for Marine-Earth Science and Technology*)

Ocean acidification repeatedly occurs in geological time. These events had various impacts on marine organisms especially those with carbonate skeletons. We review historical ocean acidification events over the past ~500 million years of Earth’s history, some of which are accompanied by severe extinction and evolutionary turnover of marine plankton. Foraminifers are marine organisms with calcium carbonate skeletons living in the water column as plankton and on the seafloor as benthos. We can use fossil foraminifers to understand the biotic response to ocean acidification as their calcite shells are preserved in marine sediment for a long time. The Paleocene-Eocene thermal maximum (PETM; ~56 million years ago) is one of the most severe events and is thought to be an analog for future climate change. Ocean acidification at the PETM is accompanied by global warming, where benthos such as some of the benthic foraminifers and ostracods are extinct. We focus on the change in the shell density of fossil planktic foraminifers through the PETM event. The micro X-ray tomography results show that the shell density has not changed significantly through the event. We review the biotic response thourhg the PETM including our results.

### [2AS02e-04]

### Interweaving of pH in cancer biology

*Yosuke Funato[1], Osamu Hashizume[1], Hiroaki Miki[1,2] ([1]*Department of Cellular Regulation, Research Institute for Microbial Diseases, Osaka University,* [2]*Center for Infectious Disease Education and Research (CiDER), Osaka University*)

Tumor microenvironment surrounding cancer cells is significantly different from the extracellular environment of normal tissues, and hypoxia and tissue acidification are known as its major chemical hallmarks. While the response mechanisms against hypoxia, orchestrated by HIF-1, have been extensively studied, why cancer cells can continuously proliferate in an acidic environment harmful to normal cells is not well understood. In this symposium, we will introduce the current understanding of the acid adaptation mechanism of cancer cells, with emphasis on our findings from the studies on PRL, a molecule overexpressed in malignant cancer cells and promotes the malignancy. PRL induces acid adaptation by promoting lysosomal exocytosis, the fusion of lysosomes with plasma membrane and subsequent extrusion of their components including the condensed $H^+$. Furthermore, chemical screening with PRL-overexpressing cells using a library of target-known compounds suggests the importance of a signaling pathway in promoting lysosomal exocytosis and acid adaptation, which may lead to the clarification of more general mechanism of acid adaptation across various cancer types and even species.

### [2AS02e-05]

### Programed Cellular Acidification (PCA) might be the novel biological process that suspend cellular activity in the multicellular organisms

*Masayuki Oginuma[1] ([1]*Research Institute for Microbial Diseases, Osaka University*)

The African turquoise killifish (N. furzeri) lives in ephemeral ponds that completely dry up for ~6 months each year. In dry season, they enter diapause that is the state arresting the embryonic development and protecting organisms from extreme environments. How diapause arrest development and protect organism are largely unknown. To understand molecular mechanism of diapause, we generated transgenic reporter lines, which can monitor metabolic activities of the energy metabolism for the cellular resolution. Using these reporter lines, we found diapause is the highly organized biological process, they did not suspend metabolic activity all cells together but gradually arrest from the anterior to posterior direction, correlated with the axial elongation process of the embryo. Furthermore, some cells were still active even in the long-term diapause period. Interestingly intra cellular pH of the arresting cells sifted into the acidic states in the diapause embryo, and acidification might read to suspend cellular activity and protect from extreme environments, that we called Programed Cellular Acidification (PCA). In this meeting, we wish to discuss about significance of this novel programed process during the N. furzeri’s diapause.

### Symposium

### [2S03e]

### Dynamic neural mechanisms for adaptation to uncertain external environments: Next-generation physiological research

March 15 (Wed.), 16:30 - 18:30, Room 3

### [2S03e-01]

### Neural activity that enables stable behavioral output even when visual input changes slightly

*Rie Kimura[1,2,4,5], Yumiko Yoshimura[4,5], Kenichi Ohki[3,1,2] ([1]*International Research Center for Neurointelligence, The University of Tokyo,* [2]*Institute for AI and Beyond, The University of Tokyo,* [3]*Department of Physiology, Graduate School of Medicine, The University of Tokyo,* [4]*Division of Visual Information Processing, National Institute for Physiological Sciences,* [5]*Department of Physiological Sciences, SOKENDAI*)

Animals can often recognize an object once they are familiar with it, even if it has changed to some degree, making behavioral output stable. We explored the neural basis that allows stable perception despite changing contrasts. The head-restrained rats were trained to push or pull a lever, depending on whether the presented visual stimuli were vertical or horizontal. After learning, the rats could discriminate the orientation above chance levels even at low contrast. We performed multiple single-unit recordings from deep layers of the primary visual cortex during the task. The neurons whose visual responses increased with a contrast reduction were observed more often after learning. The low contrast-preferring neurons responded more strongly to preferred orientations in correct-choice than incorrect-choice trials. In addition, the neurons efficiently represented low-contrast orientations. Following training, excitation was enhanced irrespective of stimulus contrast, and the phase synchronization of spikes to the beta oscillations at high contrast was stronger in putative inhibitory than excitatory neurons. The change in excitation and inhibition balance might contribute to low-contrast preference. The repeated experience strengthened the low-contrast preference, which may lead to a stable perception of familiar objects at any contrast. We would also like to present our recent data on perceived stability to other changes.

### [2S03e-02]

### Prospective Value Representation in Mouse Frontal Cortex Supports Predictive Choice Behavior

*Kosuke Hamaguchi[1] ([1]*Kyoto University Graduate School of Medicine Department of Biological Sciences*)

To make a deliberate action in a volatile environment, the brain must frequently reassess the value of each action (action-value). Choice can be initially made from the experience of trial-and-errors, but once the dynamics of the environment is learned, the choice can be made from the knowledge of the environment. The action-values constructed from the experience (retrospective value) and the ones from the knowledge (prospective value) were identified in various regions of the brain. However, how and which neural circuit integrates these values and executes the chosen action remains unknown. Combining reinforcement learning and two-photon calcium imaging, we found that the preparatory activity of neurons in a part of the frontal cortex, the anterior-lateral motor (ALM) area initially encodes retrospective value, but after extensive training, they jointly encode the retrospective and prospective value. Optogenetic inhibition of ALM preparatory activity specifically abolished the expert mice's predictive choice behavior and return them to the novice-like state. These findings suggest that ALM preparatory activity encodes the integrated action-value which plays an important role to bias the action toward the knowledge-dependent, predictive choice behavior.

### [2S03e-03]

### Serotonin mechanism for regulating reward waiting behavior

*Katsuhiko Miyazaki[1] ([1]*Okinawa Institute of Science and Technology Graduate University (OIST) Neural Computation Unit*)

Being patient to obtain future rewards is an adaptive behavior based on anticipation of future rewards. We have previously reported the following results from studies in rats and mice that demonstrate a causal relationship between serotonergic neural activity in the dorsal raphe nucleus (DRN) and waiting behavior for future rewards. (1) Rat serotonergic neurons sustainedly enhanced their activity during reward waiting behavior and decreased when they gave up waiting (Miyazaki et al., J Neurosci 2011). (2) Local pharmacological inhibition of the DRN serotonergic neural activity impaired the rats' patience for waiting for delayed rewards (Miyazaki et al., J Neurosci 2012). (3) Optogenetic activation of serotonergic neurons in the DRN enhanced the patience of mice in waiting for both the conditioned reinforcer tone and food reward (Miyazaki et al., Curr Biol 2014). (4) Serotonin stimulation promoted waiting most effectively when the probability of reward delivery is high, but timing of delivery is uncertain (Miyazaki et al., Nat Commun 2018). Furthermore, recent study has shown that when the delay time is constant and the timing of rewards is predictable (low temporal uncertainty), waiting is promoted only by serotonin stimulation in the orbitofrontal cortex (OFC). On the other hand, when the timing of rewards is difficult to predict (high temporal uncertainty), serotonin stimulation not only in the OFC but also in the medial prefrontal cortex (mPFC) promotes waiting (Miyazaki et al., Sci Adv 2020). This result suggests that the serotonergic system acts on higher brain regions to produce flexible adaptive behaviors in response to changes in temporal uncertainty of future rewards.

### [2S03e-04]

### In vivo optical approaches to memory circuit dynamics

*Daisuke Miyamoto[1] ([1]*University of Toyama*)

Motor behavior during wakefulness increases brain excitability, which is counteracted by sleep. Cooling the brain during sleep stabilizes function but may impair plasticity that allows learning to store memory information. To elucidate the synaptic and molecular mechanisms that resolve the brain's stability-plasticity dilemma, we investigated AMPA receptors, which mediate excitatory synaptic transmission, during motor learning and sleep in mice by in vivo two-photon imaging. As expected, the amount of AMPA receptors in the primary motor cortex increased on average with motor learning and decreased with sleep after learning. This decrease in AMPA receptors was impaired by sleep deprivation after learning, supporting the role of sleep in cooling the brain. However, AMPA receptors at spines, whose expression was most upregulated by learning, were not affected by post-learning sleep or sleep deprivation, while AMPA receptors at other synapses were downregulated during post-learning sleep. AMPA receptor decrease during post-learning sleep correlated with motor memory performance. Thus, sleep supports memory by reducing noise rather than enhancing signals, a brain mechanism that balances stability and plasticity.

### [2S03e-05]

### Neural mechanism to actively cope with lack of expected reward: role of dopamine

*Masaaki Ogawa[1] ([1]*Kyoto University*)

Rewards are often uncertain and not easily obtained. When we need to continue to pursue a particular uncertain reward, how do we achieve the goal of obtaining more of that reward? Even if the expected reward is not obtained and the negative outcome is disappointing, we should not give up on obtaining the reward. Rather, we need to learn to actively overcome the lack of expected reward and adjust behavior to obtain it again. This ability to cope with lack of expected reward is the key to pursue uncertain rewards and ultimately obtain more rewards. However, the neural mechanisms underlying this ability remain unclear. We developed a task in rats to monitor active behavioral switch toward the next reward after no-reward. We found that some dopamine neurons in the ventral tegmental area exhibited increased responses to unexpected reward omission and decreased responses to unexpected reward, following the opposite responses of the well-known dopamine neurons that signal reward prediction error (RPE). The dopamine increase reflected in the nucleus accumbens correlated with behavioral adjustment to actively overcome unexpected no-reward. We propose that these responses signal error to actively cope with lack of expected reward. The dopamine error signal thus cooperates with the RPE signal, enabling adaptive and robust pursuit of uncertain rewards to ultimately obtain more rewards.

### Symposium

[2S05e]
**Understanding chronic inflammatory diseases from a metabolic perspective**

March 15 (Wed.), 16:30 - 18:30, Room 5

### [2S05e-01]

### Gut microbiota at the crossroads of pancreas-intestinal barrier axis

*Yosuke Kurashima[1,2] ([1]*Institute for Advanced Academic Research, Chiba University,* [2]*Department of Innovative Medicine, Chiba University*)

The pancreas contains exocrine glands, which release enzymes (e.g., amylase, trypsin, and lipase) that are important for digestion and islets, which produce hormones. Digestive enzymes and hormones are secreted from the pancreas into the duodenum and bloodstream, respectively. Growing evidence suggests that the roles of the pancreas extend to not only the secretion of digestive enzymes and hormones but also to the regulation of intestinal homeostasis and inflammation (e.g., mucosal defense to pathogens and pathobionts). Organ crosstalk between the pancreas and intestine is linked to a range of physiological, immunological, and pathological activities, such as the regulation of the gut microbiota by the pancreatic proteins and lipids, the retroaction of the gut microbiota on the pancreas, the relationship between inflammatory bowel disease, and pancreatic diseases. We herein discuss the current understanding of the pancreas-intestinal barrier axis and the control of commensal bacteria in intestinal inflammation.

### [2S05e-02]

### Therapeutic and diagnostic impacts of AIM on various refractory chronic diseases

*Satoko Arai[1,2] ([1]*Graduate school of Medicine, The University of Tokyo,* [2]*The Institute for AIM Medicine*)

Many refractory, chronic diseases are often caused by the accumulation of types of body-derived biological wastes such as dead cells, inflammatory elements, toxic metabolites, and abnormal proteins. Our overall goal is to challenge such diseases through efficient removal of the multiple wastes by employing apoptosis inhibitor of macrophage (AIM, or CD5L). AIM is a macrophage-derived circulating protein present in blood at relatively high levels (~5 mg/mL in humans). We initially identified AIM as a supporter of macrophage survival, and it is now featured as a facilitator of repair in many diseases. In healthy state, AIM associates with IgM pentamer through disulfide-bond formation and charge-based interaction with the Fc region of IgM. During various diseases, AIM dissociates from IgM and binds to the wastes using the identical sites necessary for binding to IgM, thereby promoting their phagocytic removal by phagocytes, suppressing excessive inflammation, and facilitating the disease repair. By such mechanism, AIM exhibits profound therapeutic effects in acute kidney injury and ischemic cerebral infarction. In addition, we discovered diagnostic impacts of AIM on chronic kidney disease by a cohort analysis of patients' sera. Overall, the newly discovered waste removal system mediated by AIM could be the basis for the development of next-generation therapies and diagnoses applicable to many intractable diseases.

### [2S05e-03]

### Altered lipid metabolism in macrophages as a novel molecular mechanism of non-alcoholic steatohepatitis

*Michiko Itoh[1,2], Takayoshi Suganami[1] ([1]*Department of Molecular Medicine and Metabolism, Research Institute of Environmental Medicine, Nagoya University,* [2]*Kanagawa Institute of Industrial Science and Technology*)

Nonalcoholic steatohepatitis (NASH) is characterized by chronic inflammation and fibrosis based on hepatic lipid accumulation, and is becoming the leading cause of cirrhosis and hepatocellular carcinoma. In contrast to benign simple steatosis with triglyceride accumulation, NASH is characterized by accumulation of lipotoxic lipids including cholesterol, which induces hepatocyte cell death. Macrophages play important roles in uptake of remnant lipids from dead hepatocytes and engulfment of cell debris. We have reported an unique histological structure termed "crown-like structure (CLS)" in mouse and human NASH, where dead or dying hepatocytes are surrounded by macrophages and fibroblasts, thereby accelerating liver fibrosis. We found cholesterol crystallization in dead hepatocytes, and cholesterol accumulation in CLS-foaming macrophages. Intriguingly, intervention to reduce lysosomal free cholesterol in macrophages effectively ameliorated liver fibrosis. *In vitro* experiments revealed that loading of cholesterol crystals induces lysosomal dysfunction and profibrotic changes of macrophages. These findings provide the evidence that altered cholesterol metabolism triggers disease-specific activation of macrophages, which contributes to the development of NASH.

### [2S05e-04]

### Developing the approaches to treat aging and chronic inflammatory diseases by targeting senescent cells

*Yoshikazu Johmura[1] ([1]*Division of Cancer and Senescence Biology, Cancer Research Institute, Kanazawa University*)

Healthy life expectancy and average life expectancy have diverged by 9.2 years for men and 12.5 years for women as of 2018 in Japan, and extending healthy life expectancy is an urgent issue to realize a sustainable society. Fundamental solutions require an understanding of the regulatory mechanisms of individual aging and chronic inflammatory diseases, and the development of therapeutic and preventive technologies. Senescent cells accumulate in various tissues as individuals age and develop chronic inflammatory diseases, and that removal of senescent cells using genetic engineering techniques delay aging traits in mice. In other words, the development of drugs (senolytics) that can remove senescent cells from individuals would lead to an increase in healthy life expectancy. Recently, we have shown that glutaminase inhibitors are effective as senolytics by targeting the metabolic characteristics of senescent cells and can ameliorate various age-related changes and symptoms of chronic inflammatory diseases. It was also found that senolytics can be applied through immunological approaches based on single-cell analysis of senescent cells in vivo. Moreover, we are trying to develop the innovative approaches to control senescent cells and will present the latest findings of these studies.

### [2S05e-05]

### Molecular crosstalk between lipid metabolism, inflammation and autoimmunity

*Ayaka Ito[1,2] ([1]*Department of Molecular Medicine and Metabolism, Research Institute of Environmental Medicine, Nagoya University,* [2]*Research Unit for Phsyconeuroimmunology and Psychosomatic medicine, Institute for Advanced Research, Nagoya University*)

The activation, differentiation and function of immune cells are dependent on energy supply and metabolic reprogramming, and how energy is supplied to immune cells is critical for an appropriate immune response. Accumulating evidence suggests that lipid metabolism is greatly altered in response to inflammatory stimuli, and which results in dynamic modification in the quantity and quality of cellular lipids. However, the molecular mechanism by which lipid metabolism influences autoimmune responses is largely unknown.Mice lacking the nuclear receptor liver X receptors (LXRα and LXRβ), which are pivotal regulators of lipid homeostasis, develop age-dependent lupus-like autoimmunity, and treatment with an LXR agonist ameliorates disease progression in a spontaneous lupus mouse model (*Immunity* 31:245-258, 2009). We demonstrated that cholesterol overload in CD11c+ antigen-presenting cells causes systemic autoimmunity in LXR-defcient mice by stimulating the production of the B cell growth factors, which support B cell expansion and autoantibody production (*Immunity* 45: 1311-1326, 2016). As an underlying mechanism, increased cholesterol content in cellular membranes enhances the lipid raft-dependent signaling of immune pathways such as toll-like receptor signaling (*eLife* 4: e08009, 2015). In addition, we recently reported that dietary EPA supplementation ameliorates representative lupus manifestations, including autoantibody production and immunocomplex deposition in the kidneys. A combination of lipidomic and membrane dynamics analyses revealed that EPA remodels the lipid composition and fluidity of B cell membranes, thereby preventing B cell differentiation into autoantibody-producing plasma cells (*Front Immunol* 12: 650856, 2021). These results highlight a previously unrecognized mechanism by which cholesterol content and/or fatty acid composition affects immune cell function during inflammation and autoimmunity.

### Symposium

### [2S06e]
### Regulatory mechanisms of voltage-gated cation channels and their significance in maintaining homeostasis

March 15 (Wed.), 16:30 - 18:30, Room 6

### [2S06e-01]

### Gating modulation of voltage-gated $K^+$ channels by auxiliary subunits via voltage-sensing domains

*Koichi Nakajo[1] ([1]*Division of Integrative Physiology, Department of Physiology, Jichi Medical University*)

Voltage-gated $K^+$ channels play a critical role in action potential repolarization. They are a tetramer of α subunits and sense membrane potential by the voltage-sensing domain (VSD) from each α subunit. The fourth segment (S4) is the center of voltage sensing, and all four S4 segments need to be in the up (or intermediate) position to open the channel's pore. Some voltage-gated $K^+$ channels have auxiliary subunits (or β subunits), which regulate the gating properties of the channel. One of the most prominent examples is the KCNQ1 channel, which owns five KCNE proteins as regulatory β subunits. For example, KCNE1 is mainly expressed in the heart and slows the activation and deactivation kinetics of KCNQ1 channels, thus regulating cardiac excitability. In another example, KCNE3 makes the KCNQ1 channel constitutively open, helping ion transport in epithelial cells by potassium recycling. Recent reports of electrophysiological experiments and structural models suggest that these regulations of the KCNQ1 channels are via the VSD: KCNE proteins directly interact with the VSD and affect the equilibrium of the states (down, intermediate, and up positions of the S4 segment). I will introduce recent advancements in the field and discuss how the KCNE proteins regulate the VSD equilibrium.

### [2S06e-02]

### Functional regulation of voltage-dependent L-Type $Ca^{2+}$ channels and its physiological significance

*Satomi Adachi-Akahane[1] ([1]*Toho Univ.*)

Dysregulation of $Ca^{2+}$ dynamics in cardiac myocytes causes contractile/diastolic dysfunction and arrhythmias. L-type $Ca^{2+}$ channels (LTCCs) are responsible for $Ca^{2+}$ influx, shaping action potential (AP), and triggering $Ca^{2+}$-induced $Ca^{2+}$ release (CICR) from the ryanodine receptor (RyR). LTCC is, in turn, inactivated by CICR. In ventricular myocytes, LTCC and RyR are highly localized in the junctional membrane structure composed of T-tubular and SR membranes. Through the cross-communication with RyR, LTCC senses subsarcolemmal $Ca^{2+}$ concentration to regulate CICR and $Ca^{2+}$ content in the SR. Accordingly, APD is also affected by the coupling between LTCC and RyR. In addition, LTCC activity is regulated by transcription, delivery, cluster formation, degradation, and post-translational modifications. Accessory subunits such as β and $\alpha_2/\delta$ subunits play essential roles in regulating LTCC activities. In this symposium, I would like to review the latest research on the regulatory mechanisms of LTCC activity in ventricular myocytes via accessary subunits, delivery, and degradation. I will further discuss their significance in cardiac function and dysfunction such as diabetic cardiomyopathy.

### [2S06e-03]

### Functional analysis of voltage-gated ion channels with voltage sensor mutations in channelopathies.

*Tomoya Kubota[1] ([1]*Osaka Univ.*)

Voltage Sensor Domains (VSDs) are small voltmeters which control the conformation of voltage-gated ion channels (VGICs), and have been involved in a variety of physiological roles in excitable organs such as neuron, skeletal muscles and heart. Mutations in VSDs of VGIC genes have been associated with several rare genetic disorders, channelopathies. Hypokalemic periodic paralysis (HypoPP) is one of the rare genetic disease associated with mutations in VSDs of *CACNA1S* or *SCN4A*, encoding Cav1.1 or Nav1.4, respectively. Most HypoPP-associated missense changes occur at the arginine residues within the VSDs and previous structural and functional studies suggest that mutations of the arginine residues destroy the hydrophobic seal separating the external water and the internal cytosolic crevices, resulting in the generation of an aberrant ion permeation pathway, the gating pore. Presently, non-physiological leak currents through the gating pore, known as the gating pore currents, are thought to underlie HypoPP. In this symposium, we review functional analysis of HypoPP and introduce our on-going project to convert gating pore currents into optical signals in HypoPP model cells.

### [2S06e-04]

### Involvement of HCN channels in neuropathic pain

*Mitsuhiko Yamada[1] ([1]*Dept. Mol. Pharmacol., Shinshu Univ. Sch. Med.*)

An injury of the somatosensory system causes neuropathic pain, which is usually refractory to conventional analgesics. The mechanism of neuropathic pain in rats that had undergone left L5 spinal nerve transection was analyzed. Ten days after surgery, these rats acquired neuropathic pain. The current-clamped L5 dorsal root ganglion neurons on the ipsilateral side exhibited significantly higher excitability than those on the contralateral side. However, only neurons with diameters of 40–50 um (i.e., A beta neurons) on the ipsilateral side exhibited significantly larger voltage sags in response to hyperpolarizing current pulses than those on the contralateral side. Under the voltage clamp, only these neurons on the ipsilateral side showed a significantly larger density of an inward current at < -80 mV (hyperpolarization-activated nonselective cation ($I_h$) current) with a rightward-shifted activation curve than that on the contralateral side. Ivabradine, a HCN channel inhibitor inhibited $I_h$ currents in these neurons on both sides in a similar concentration-dependent manner with an $IC_{50}$ value of ~3 uM. Moreover, the oral administration of ivabradine significantly alleviated the neuropathic pain on the ipsilateral side. Thus, ivabradine may be utilized for treatment of some forms of neuropathic pain.

### [2S06e-05]

### A novel point mutation of the human $Na_V1.7$ gene induces congenital insensitivity to pain by raising the threshold of action potential in small dorsal root ganglion neurons

*Hiroyuki Nakamura[1], Kenkichi Kiyosawa[1], Tsutomu Nakada[2], Takuro Numaga-Tomita[3], Mitsuhiko Yamada[3], Mikito Kawamata[1] ([1]*Department of Anesthesiology and Resuscitology, Shinshu University School of Medicine,* [2]*Departmeny of Instrumental Analysis, Research Center for Supports to Advanced Science, Shinshu University,* [3]*Department of Molecular Pharmacology, Shinshu University School of Medicine*)

[Introduction]A voltage-dependent $Na^+$ channel, $Na_V1.7$ is dominantly expressed in small dorsal root ganglion neurons (SDN) and mediates inward currents at the threshold of action potential (AP). As it is essential for pain signaling, its loss-of-function mutations cause Congenital Insensitivity to Pain (CIP), a loss of nociception against any harmful stimulations. We found a CIP patient bearing a novel point mutation in $Na_V1.7$ gene. [Method]The nociceptive behavior of knock-in mice carrying the mutation and the electrophysiological properties of their SDN were analyzed. [Result]Their homozygotes showed significantly higher pain thresholds to mechanical, heat, and inflammatory noxious stimulations than wildtype littermates. SDN of homozygotes exhibited significantly higher threshold of AP than that of wildtype littermates due to the significantly smaller maximum current density and depolarized activation curve of $Na_V1.7$ channels. [Conclusion]This study revealed that this novel point mutation reduces the inward current amplitude at the threshold of AP in SDN and thereby, causes CIP. Our findings may delineate a unique site of action of a novel pain relief drug.

### Symposium

[2S07e]

**Circadian rhythms serve as platform for homeostasis and sustainability of life: Toward overcoming health problems by the clock disfunction**

March 15 (Wed.), 16:30 - 18:30, Room 7

### [2S07e-01]

### Glucocorticoids and Circadian Rhythm Regulation

*Masaaki Ikeda[1], Shinnosuke Yanagisawa[1,2], Megumi Kumagai[1], Yasuhiro Takenaka[3], Yoshihiro Nakajima[4] ([1]*Department of Physiology, Faculty of Medicine, Saitama Medical University,* [2]*Department of Diabetes and Endocrinology, Saitama Medical University,* [3]*Department of Physiology, Graduate School of Medicine, Nippon Medical School,* [4]*Health Research Institute, National Institute of Advanced Industrial Science and Technology*)

Transmission of rhythm signals from the suprachiasmatic nucleus, the rhythm center, to cells in peripheral organs via glucocorticoid hormones and the autonomic nervous system is important for the entrainment of circadian rhythms in peripheral tissues. The glucocorticoid receptor binding sequence, GRE, exists in the regulatory region of clock genes and is involved in induction of their expression. In particular, for the *Per2* gene, a GRE, located 22.8 kb downstream of the transcription start site, has been shown to be involved in glucocorticoid-induced transcription of the *Per2* gene, circadian rhythm expression, and rhythm phase synchronization. These studies suggest that the so-called genomic effect of glucocorticoids is central to circadian rhythm induction, although non-genomic effects of glucocorticoids that do not involve transcriptional regulation by binding the glucocorticoid receptor have been reported. In this symposium, we will provide an overview of the overall mechanism of circadian rhythm regulation by glucocorticoids, including recent results from our laboratory.

### [2S07e-02]

### Clock protein codes triggering synchronous cellular clock oscillation to new strategies for jet lag-associated health disorders

*Teruya Tamaru[1], Genki Kawamura[2], Hikari Yoshitane[3,4], Mamoru Nagano[5], Yasufumi Shigeyoshi[5], Kimiko Shimizu[6,7], Yoshitaka Fukada[4], Takeaki Ozawa[2], Ken Takamatsu[1]
([1]*Department of Physiology, Toho Universiity School off Medicine,* [2]*Department of Chemistry, School of Science, The University of Tokyo,* [3]*Circadian Clock Project, Tokyo Metropolitan Institute of Medical Science,* [4]*Department of Biological Sciences, School of Science, The University of Tokyo,* [5]*Department of Anatomy, Kindai University,* [6]*Department of Pathological Cell Biology, Medical Research Institute Tokyo Medical and Dental University,* [7]*Center for Disease Biology and Integrative Medicine, School of Medicine, The University of Tokyo*)

Molecular/cellular clocks which function in almost all tissues are platforms for the harmonized physiological functions, by their synchronizing ability to the environments and sustainable/autonomous oscillation with clock genes/proteins such as BMAL1.. Central clock, the suprachiasmatic nucleus (SCN), which can be entrained by light, orchestrates peripheral clocks for coordinated functions. Abrupt changes in environment and lifestyle perturb clocks and cause various health problems and diseases by time difference disorders such as jet lag. As evidence, we demonstrate that impairment of adaptation system driven by clock synchronization responses cooperating with numerous adaptation system results in decreased adaptabilities to cellular stress [Commun. Biol. 2018]. In this research, we sought to elucidate the initial clock protein signal during synchronization which drives synchronous cellular clock oscillation as the basis for environmental adaptation and its physiological significance. Additionally, we developed a transient clock dysfunction model using a BMAL1-targeted clock inhibitor. We will present data obtained using genetic engineering and live cell imaging which was applied to both peripheral and central clocks. We demonstrate that BMAL1-S region (BMAL1-S) and BMAL1-modifications (S90-phosphorylation) are essential protein codes for synchronous clock oscillation. Additionally, we developed a novel BMAL1-targeted inhibitor that can inhibit central clock oscillation reversibly. These results demonstrate a potential unique medical strategy to address health problems by overcoming a jet lag disorder.

### [2S07e-03]

### Regulation of clock gene expression and circadian rhythm in human iPS cells

*Hitomi Kaneko[1], Taku Kaitsuka[2], Kazuhito To[1] ([1]*Department of Molecular Physiology, Faculty of Life Sciences, Kumamoto University,* [2]*School of Pharmacy at Fukuoka, International University of Health and Welfare*)

Circadian rhythm is an essential function of the organism, occurring during development and regulating cell proliferation and organ maturation. However, circadian rhythm is absent in early development, and a circadian clock is not developed in pluripotent stem cells (PSCs), and it is formed gradually during differentiation.We found that BMAL1 mRNA and protein levels are low in human induced pluripotent stem cells (iPSCs) and that clock gene expression is suppressed by repressive modification of histone H3K27 trimethylation around the transcription start site of PER1. Then, we found overexpression of BMAL1 and inhibition of the histone methyltransferase EZH2 caused circadian rhythms in BMAL1 and PER2. To rule out the possibility of effects on differentiation, we investigated whether these treatments affect pluripotency and self-renewal, the core functions of PSCs. The results from several analyzes showed that iPSCs even with both treatments maintain pluripotency. Our results suggest that the trimethylation modification of H3K27 could regulate clock gene expression and the one of the possible mechanisms how circadian rhythm is not induced early in development of human embryos. It is further needed to clarify the necessity which the circadian rhythm is developed at specific time point during development.

### [2S07e-04]

### The effects of crude drugs on clock gene expression rhythms and locomotor activity rhythms and the mechanisms of these effects

*Atsushi Haraguchi[1], Shigenobu Shibata[1] ([1]*Laboratory of Physiology and Pharmacology, School of Advanced Science and Engineering, Waseda University*)

The mammalian circadian clock system regulates many physiological functions. Crude drugs and Kampō, combining multiple crude drugs, have been reported to have effects on various diseases, but there were no studies examining these effects on the circadian clock system. Therefore, we aimed to screen crude drugs for the shortening effect on period length of clock gene expression and locomotor activity rhythms. In *in vitro* experiments, we used MEFs from PERIOD2::LUCIFERASE K.I. mice and examined the effects and mechanism of crude drugs on PER2 expression period length. In *in vivo* experiments, we fed mice with AIN-93M or AIN-93M with crude drug that has the most shortening effects, and evaluated the free-running period length of mouse locomotor activity rhythm in the absence of light signals. In the *in vitro* experiments, we found that *Polygalae Radix* (*PR*) had the most shortened the PER2 expression period length in 40 crude drugs and that the shortening effect of *PR* was mediated by the CaMKII pathway. In the *in vivo* experiment, long-term feeding with AIN-93M with *PR* slightly shortened the free-running period. Taken together, our results indicate that *PR* and the CaMKII pathway may be regarded as a new therapy and a target pathway for circadian rhythm disorders, respectively.

### [2S07e-05]

### Clock Aging: Molecular basis for age-related functional decline

*Hikari Yoshitane[1,2] ([1]*Tokyo Metropolitan Institute of Medical Science,* [2]*Graduate School of Science, The University of Tokyo*)

The circadian clock generates transcriptional rhythms with a cycle of about 24 hours, and makes genes function only at the required time. Therefore, deficiency of the clock gene not only causes sleep disorders (insomnia), but also causes a number of symptoms such as hypertension, cardiac diseases, metabolic disorders, cancer, immune depression, depression, cataract, and loss of muscle mass (sarcopenia), and significantly reduces the lifespan. These functional decline due to abnormality of the circadian clock resembles the aging-associated symptoms in humans. Here we defined the age-related circadian clock abnormality as "clock aging", and aimed to show that part of the aging-associated symptoms is caused by disruption of the functional rhythms of the circadian clock. In this study, we prepared a series of mouse tissues from 2 Mo, 18 Mo, and 24 Mo mice at 6 time points thoughout that day, and performed RNA-Seq analysis, proteome analysis, and phospho-proteome analysis to examine the effect of aging on circadian rhythms at RNA levels, post-transcriptional modification levels, total protein levels, subcellular localazation levels, and post-translational modification levels. Furthermore, we established new mouse models by imitation of the "clock aging" state. We will elucidate the mechanisms of aging-associated symptoms as phenotypes in the clock aging mice, and will pursue the molecular mechanisms that cause abnormality in clock output with aging.

### Symposium

[2S08e]
**Environmental Adaptation of Skeletal Muscles and Adipose Tissues**

March 15 (Wed.), 16:30 - 18:30, Room 8

### [2S08e-01]

### The skeletal muscle that is "easy-to-lose weight" in a thrifty phenotype model rat

*Takahiro Nemoto[1] ([1]*Dept. Bioregulatory Science, Nippon Medical School*)

The DOHaD theory hypothesizes that the mismatch between the thrifty phenotype acquired in the prenatal period and the excessive nutritional environment after birth increases the risk of developing future diseases. To understand the thrifty phenotype acquired by fetal undernutrition, we generated a model rat. The weight loss rate of low birthweight (LBW) rats was significantly lower than that of normal birthweight (NBW). Soleus muscle weight per bodyweight of NBW turned to increase 48 hours after refeeding, while its weight of LBW decreased. The recovery rate of soleus weight of LBW was significantly lower than that of NBW. Corticosterone concentrations in LBW rats were significantly higher than those in NBW after 48 hours of fasting and after 48 hours of refeeding. Blood IGF-1 concentrations in LBW were lower than those in NBW at baseline and decreased further after 48 hours of fasting. A sustained high glucocorticoid concentration due to metabolic stress exposure may induce sarcopenic obesity. Thus, the skeletal muscles of LBW that it difficult to gain weight after refeeding, which may increase the risk of developing non-communicable diseases.

### [2S08e-02]

### Fasting increases 18:2-containing phosphatidylcholines to complement the decrease in 22:6-containing phosphatidylcholines in mouse skeletal muscle

*Shinji Miura[1] ([1]*University of Shizuoka*)

Fasting stimulates catabolic reactions in skeletal muscle to survive nutrient deprivation. Cellular phospholipids have large structural diversity due to various polar-heads and acylchains that affect many cellular functions. Skeletal muscle phospholipid profiles have been suggested to be associated with muscle adaptations to nutritional and environmental status. However, the effect of fasting on skeletal muscle phospholipid profiles remains unknown. Here, we analyzed phospholipids using liquid chromatography mass spectrometry. We determined that fasting resulted in a decrease in 22:6-containing phosphatidylcholines (PCs) (22:6-PCs) and an increase in 18:2-containing PCs (18:2-PCs). The fasting-induced increase in 18:2-PCs was sufficient to complement 22:6-PCs loss, resulting in the maintenance of the total amount of polyunsaturated fatty acid (PUFA)-containing PCs. Similar phospholipid alterations occurred in insulin-deficient mice, which indicate that these observed phospholipid perturbations were characteristic of catabolic skeletal muscle. In lysophosphatidic acid acyltransferase 3-knockout muscles that mostly lack 22:6-PCs, other PUFA-containing PCs, mainly 18:2-PCs, accumulated. This suggests a compensatory mechanism for skeletal muscles to maintain PUFA-containing PCs.

### [2S08e-03]

### Fetal Origins of Obesity: Newer insights into fetal growth and body composition

*Satoru Ikenoue[1] ([1]*Department of Obstetrics and Gynecology, Keio University School of Medicine*)

Objectives: Newborns exhibit substantial variation in fat mass, which relates to metabolic dysfunction in later life. We aimed (i) to examine whether fetal fat mass measures are associated with newborn percentage body fat (NB%BF), (ii) to determine whether fetal liver blood flow (*f*LBF) influences NB%BF, and (iii) to investigate whether placental corticotrophin-releasing hormone (*p*CRH) is associated with variations in *f*LBF. Methods:(i) Fetal upper-arm and thigh percent fat area and abdominal wall thickness were measured at 20 and 30 weeks in 109 uncomplicated pregnancies. NB%BF was quantified by DXA. The association between fetal fat mass and NB%BF was examined using multiple regression analysis. (ii) *f*LBF was quantified at 30 weeks in 62 pregnancies, and examined the association with NB%BF. (iii) *p*CRH was measured in maternal circulation across gestation in 79 pregnancies. The correlation between *f*LBF and *p*CRH was determined. Results: (i) Fetal fat mass measures (especially in the arm) at 30 weeks was significantly associated with NB%BF. (ii) *f*LBF at 30 weeks significantly correlated with NB%BF. (iii) *p*CRH at 30weeks was significantly associated with *f*LBF at 30 weeks. Conclusions: Fetal fat mass is an early indicator of newborn adiposity, which is affected by *f*LBF. *p*CRH in late gestation is a possible modulator of *f*LBF and may constitute a biochemical marker in clinical investigations of fetal growth and body composition.

### [2S08e-04]

### Seasonal adaptation of growth, muscle, and brain via early-life photoperiod

*Shinobu Yasuo[1] ([1]*Faculty of Agriculture, Kyushu University*)

Season of birth or perinatal photoperiod has long-lasting effects on physiological and behavioral phenotype in many mammalian species. The adaptation to early-life photoperiod is essential to ensure survival and development in appropriate seasons in nature. We have clarified using mice that postnatal photoperiod alters body weight gain, muscle gene expression, and metabolomic profiles in adulthood. To utilize the ensuring effect of early-life photoperiod on growth and metabolism in livestock production, we addressed effect of postnatal photoperiod (short-day: SD, long-day: LD, until 3-month-old) in Japanese Black cattle. Even though cow is a non-seasonal breeder, calves under SD or LD exhibited winter- or summer-patterns of coat, respectively, irrespective of actual seasons. In cows exposed to LD postnatally, larger muscle fiber diameters were observed at least until 10-month-old; carcass weight at slaughter (>40-month-old) was heavier in them. We have identified *Syntaxin 16* as a photoperiod-imprinting gene in both mice and cows, and its functional role is analyzed. Our focus also includes the hypothalamus as an integration center of metabolism, and the role of photoperiod-dependent neuronal modeling will be discussed.

### [2S08e-05]

### Effects of maternal nutrition on skeletal muscle and adipose tissue in fetuses of Wagyu cattle and its potential for sustainable meat production

*Takafumi Gotoh[1] ([1]*Kagoshima University*)

Maternal low or high nutrition give unique effects on morphological and molecular dynamics in skeletal muscle and adipose tissue of fetuses of fatty breed Wagyu (Japanese Black) cattle which produce highly marbled beef. We have investigated to determine the effects of maternal energy intake in Wagyu cows, during gestation on fetal muscle and adipose tissue development, histochemical properties, metabolism, and gene and microRNA (miRNA) expression. Cows were allocated to one of two nutritional energy groups: 120% (HIGH) or 60% nutritional requirements of (LOW). We investigated their fetuses (fetal age 260 ± 8 days). The whole-body, total muscle, adipose, and bone masses of the fetal half-carcasses were significantly higher in the high-nutrition group than in the low-nutrition group. Wagyu cattle maternal undernutrition delayed fetal myofibers hyperplasia, while elevated maternal nutrition stimulated fetal myofiber hyperplasia, adipogenesis, and glucose metabolism. Moreover, maternal undernutrition altered the levels of amino acids and expression of genes associated with energy expenditure, glucose homeostasis, and angiogenesis in the fetal muscle. Maternal undernutrition in Wagyu cattle increased fetal brown adipose tissue development in subcutaneous, visceral, and perirenal adipose tissues, while elevated maternal nutrition stimulated fetal subcutaneous adipose tissue development compared with that of visceral and perirenal adipose tissues.

### Symposium

[2S09e]
**Hot topics on nuclear envelope**

March 15 (Wed.), 16:30 - 18:30, Room 9

### [2S09e-01]

### Nuclear envelope and research hot topics on nuclear envelopathy

*Yukiko K. Hayashi[1] ([1]*Department of Pathophysiology, Tokyo Medical University*)

The nuclear envelope (NE) is a biological membrane that separates the nucleoplasm of eukaryotes from the cytoplasm. NE has a double membrane structure of the inner and outer membranes. The nuclear lamina underlies inner nuclear membrane and linked to chromatin. Material transport between the nucleus and cytoplasm is carried out by a giant protein complex called the nuclear pore complex. NE changes dynamically during cell cycle and has important roles in gene regulation. Emerin is the firstly identified nuclear envelope protein associated with a human disease; Emery-Dreifuss muscular dystrophy. Later, mutations in the several genes encoding NE proteins are known to cause various diseases such as myopathy, cardiomyopathy, and progeria, and they are collectively called as nuclear envelopathy. Further, recent studies revealed novel important physiological functions of NE proteins. In this paper, I will overview the NE and introduce recent advances on nuclear envelopathy.

### [2S09e-02]

### Mechanical dynamics of the nucleus in mouse early embryos

*Yuta Shimamoto[1] ([1]*National Institute of Genetics*)

After fertilization, the embryo undergoes genome reprogramming to develop into a fully functional organism. Whereas the nucleus, the “container” that packages DNA, maintains overall integrity while acting as the site for diverse genomic events, its physical and biochemical properties during development are largely unknown. Using mouse embryos as a model, we have been analyzing the dynamics of nuclear size, shape, and mechanical properties. We found their drastic changes, in that the nuclear membrane becomes unstable and deformable associated with the progression of the development. In this symposium, I'll discuss the mechanism underlying the changes in the nuclear state and the possible roles in genome regulation.

### [2S09e-03]

### Toward understanding the molecular mechanism of nuclear envelope stress response and its physiological impact

*Yasunao Kamikawa[1], Zuqian Wu[1], Atsushi Saio[1], Kazunori Imaizumi[1] ([1]*Hiroshima University*)

Recent works have revealed that the nuclear envelope (NE) is damaged by various stress, such as mechanical confinement and virus infection. The stresses that injure the integrity of the NE are referred to as “NE stress”. NE stress often causes the rupture of the NE, leading to the loss of barrier between the nucleus and the cytoplasm. Although ruptured NE is repaired in most cases, prolonged rupture likely results in cellular dysfunctions. The molecular mechanisms for repairing the NE are intensively studied and the crucial factors have been identified including a DNA binding protein BAF, its binding partner LEM domain proteins, and membrane remodeling ESCRT III complexes. We previously reported that an endoplasmic reticulum resident trans-membrane transcription factor OASIS responds to NE stress and is required for maintaining the integrity of the NE. The expression of OASIS is restricted to specific types of cells such as astrocytes and osteoblasts, suggesting cell-type specific response to NE stress. Indeed, we found that nuclear deformation as well as DNA damage induced by mechanical confinement varies among a panel of cancer cell line. Furthermore, live cell imaging revealed the failure of repairing of ruptured NE in a certain type of cell line. We will present the molecular mechanism underlying such cell-type specific response to NE stress and discuss its physiological significance.

### [2S09e-04]

### Molecular mechanisms underlying defective repair of nuclear envelope ruptures in laminopathies

*Takeshi Shimi[1], Yohei Kono[1], Stephen Adam[2], Yuko Sato[1], Karen Reddy[5], Yixian Zheng[3], Ohad Medalia[4], Robert Goldman[2], Hiroshi Kimura[1] ([1]*Tokyo Institute of Technology*, [2]*Northwestern University*, [3]*Carnegie Institution for Science*, [4]*University of Zurich*, [5]*Johns Hopkins University*)

In mammalian cell nuclei, the nuclear lamina (NL) underlies the inner nuclear membrane of the nuclear envelope (NE) to maintain nuclear structure. The major NL components, A-type lamins (LA, LC) and B-type lamins (LB1, LB2) contribute to the protection against NE rupture induced by mechanical stress. Numerous lamin mutations are known to cause a wide spectrum of human genetic disorders, collectively termed as laminopathies. Some of the mutations result in spontaneous NE rupture but the molecular mechanisms still remain unclear. Our analyses using immunofluorescence and live-cell imaging revealed that among all the lamin isoforms, only nucleoplasmic LC rapidly accumulated at sites of NE rupture in mouse embryonic fibroblasts. Some of laminopathy mutations in the immunoglobulin-like fold domain of LC disrupted the binding to barrier-to-autointegration factor (BAF), leading to the inhibition of the recruitment from the nucleoplasm to the rupture sites. BAF accumulation at the rupture sites and DNA sensing of cytoplasmic cyclic GMP-AMP synthase (cGAS) were partially dependent on LA/C. These results suggest that nucleoplasmic LC, BAF and cGAS concertedly accumulate for rapid repair of NE ruptures. Our findings implicate a possible link between NE rupture and the physiological properties of and pathological changes in the laminopathies.

### [2S09e-05]

### Why do abnormalities in the nuclear envelope cause muscular dystrophy?

*Eiji Wada[1], Yukiko Hayashi[1] ([1]*Tokyo Medical University*)

Muscular dystrophy is a group of diseases that cause progressive weakness and loss of muscle mass. Approximately 30 types of muscular dystrophy are known, mostly caused by genetic mutations in proteins related to muscle structure and function. Exceptionally, Emery-Dreifuss muscular dystrophy (EDMD) causes by mutations in *LMNA* encoding lamin A/C, genes encoding a nuclear membrane protein, *EMD* (emerin), *SYNE1* (nesprin-1), *SYNE2* (nesprin-2), or *LUMA* (TMEM43). EDMD is characterized by skeletal muscle weakness, early joint contractures, and cardiomyopathy. Potential hypotheses are proposed to underpin the pathogenesis of this complicated disease: 1) the structural hypothesis focuses on abnormal nuclear shape associated with fragile nuclear membrane; 2) the gene expression hypothesis concerns impairment of chromatin organization and signaling pathways; 3) the stem cell hypothesis relates to abnormality in skeletal muscle stem cells. We have analyzed different types of EDMD model mice to provide new insights into the role of nuclear membrane proteins in striated muscles, helping to understand the tissue-specific pathological mechanisms of nuclear envelopathies.

### Symposium

[3AS01m]
**Understanding "Pre-Diseases" (Mibyo): Challenges from Cutting-Edge Mathematical Sciences**

March 16 (Thu.), 9:00 - 11:00, Room 1

### [3AS01m-01]

### Mathematical model driven "Pre-Disease" (Mibyo) research -Heterogenity and Stratification-

*Shingo Iwami[1] ([1]*Nagoya University*)

To acheive a precision medicine at a very early stage, detecting "pre-disease" (Mibyo) states is an ideal goal. However, in general, disease progressions and its prognosis are highly variable, that is, there is a huge heterogenity for disease states. Therefore, understanding and quntifying the heterogenity based on process-based mathematical model are an important step for the Mibyo research. Once the heterogenity is quitified as parameters in the mathematical model, we may stratify the variable disease progressions and its prognosis employing "metrics": estimated paramters, reconstructed time-course dynamics of biomaker(s) and its engineered features, and etc. The stratification may be a key process to detect Mibyo states. In this talk, I will discuss how we make a mathematical model and apply it to real world datasets including clinical data, then show an example for a stratification of disease progression.

### [3AS01m-02]

### The benefits and challenges of detecting the pre-disease state

*Keiichi Koizumi[1] ([1]*Institute of Natural Medicine, University of Toyama*)

The pre-disease state, which is known as Mibyou in traditional Japanese (Kampo) medicine, is a concept of Oriental medicine that has not yet been scientifically understood. Therefore, in collaboration with our division and the Research Center for Pre-Disease Science, University of Toyama, we are conducting research to scientifically detect the Mibyou state by analyzing fluctuations and changes in the expression of biometric information, such as genes, proteins, and behaviors, during the disease onset process to clarify the biological meaning of this state. Today, we are seeing marked increases in the incidence of complex diseases, such as metabolic syndrome, which are hard to treat with only modern medicines. In contrast to the symptomatic treatments offered by modern medical drugs at the disease state, drugs targeting Mibyou may have significant potential advantages as preventive and preemptive medicine. To achieve our objectives, we have joined with the Aihara Moonshot (AMS) Project, which is supported by JST Moonshot R&D, to work on developing detection systems for Mibyou and, consequently, to create novel medical strategies in collaboration with the AMS Project. In my lecture, I would like to talk about the challenges we have faced while conducting research in the field of pre-disease science.

### [3AS01m-03]

### Statistical genetics, disease biology, drug discovery, and personalized medicine

*Okada Yukinori[1,2,3] ([1]*Department of Statistical Genetics, Osaka University Graduate School of Medicine,* [2]*Department of Genome Informatics, Graduate School of Medicine, the University of Tokyo,* [3]*Laboratory for Systems Genetics, RIKEN Center for Integrative Medical Sciences*)

Statistical genetics is a research field that evaluates causality of human genetic variations on diseases, using statistical and bioinformatics approaches. Recent developments of sequencing technologies have provided human disease genome data of hundreds of thousands of the subjects, and successfully identified comprehensive catalogues of genetic susceptible loci. However, little is known regarding how to develop methodology to integrate large-scale human genome data with diverse biological resources. We have developed such methods and applied to a pioneering example of large-scale genetic association studies on a variety of human complex traits. Tran-layer omics analysis identified the cell types and microbiomes implicated in disease biology. Network analysis between the disease risk genes and the drug target genes could identify novel candidates of drug repositioning. Integration of cell type-specific gene expression profiles estimated from GWAS with compound perturbation databases can pinpoint novel therapeutic targets and compounds. Application of the machine learning methods into population genome data can classify the samples without prior biological information. These results should empirically show the value of statistical genetics to dissect disease biology, novel drug discovery, and personalized medicine. Finally, we would like to introduce our activity on young researcher developments (“Summer school of statistical genetics” in Osaka Univeristy).

### [3AS01m-04]

### Multi-layered Approach for Early Detection and Prediction of Cardiovascular Events

*Katsuhito Fujiu[1] ([1]*Department of Advanced Cardiology, the University of Tokyo*)

While cancer is Japan's most major life-threatening disease, cardiovascular diseases have been the no.1 killer worldwide. Cardiac diseases are known to be polygenic and lifestyle-associated diseases. As of now, future risks of cardiovascular diseases can be deeply determined by recent individual genome analysis. However, the following questions are about how we should handle those high-risk populations. Changing modifiable lifestyles is a common strategy as primary prevention for it. Using over two million population databases, we recently revisited what factors would impact cardiovascular disease development in young adults, the 20 to 65-year-old population. The results clearly showed mild hypertension had a significant influence on cardiovascular events. Versatile wearable devices have enabled us to spread home blood pressure monitoring in patients and healthy persons. The easier we can wear these devices, the more device users would increase, leading to good home health care management. However, even if those wearable devices develop or improve significantly, a part of the population indifferent to health may not use them. The concern is an upcoming issue in the ICT/IoT-developed society era. Therefore, we developed a new system that could detect early features of hypertension without any contact with the person. Next, we also try to resolve concerns about the secondary prevention of heart diseases. The most medical and economic issue among them is heart failure. We collected time-series multi-modality data from twenty-thousand heart failure patients and generated home monitoring systems for heart failure and sudden cardiac death. With these systems, we plan to manipulate the patients to prevent heart failure recurrence or cardiac death by early intervention based on prediction or early detection of heart events.

### Symposium

[3AS02m]

**Generational interaction in the study on thermo-sensitive Transient Receptor Potential (TRP) channel**

March 16 (Thu.), 9:00 - 11:00, Room 2

### [3AS02m-01]

### Clinining of thermosensitive TRP channels

*Makoto Tominaga[1] ([1]*National Institute for Physiological Sciences*)

The Nobel Assembly at Karolinska Institute has decided to award the 2021 Nobel Prize in Physiology or Medicine jointly to David Julius and Ardem Patapoutian for their discoveries of receptors for temperature and touch. I would like to sincerely say congratulations on their receiving the award.A gene for capsaicin receptor TRPV1 was isolated by a group of David Julius in 1997, and it was clarified that TRPV1 is activated by noxious heat stimulus with a temperature over 43 ℃, getting a lot of attention as the first temperature sensor. Then, eleven so called thermosensitive TRP channels including TRPM8 which is activated by both menthol and cold stimulus were reported. Ardem Patapoutian reported Piezo 1 and Piezo 2 as mechanosensors in 2010. Although mechanisms for detection of physical stimuli made a slow progress without clear molecular entities, discoveries of thermosensitive TRP channels and Piezo channels pushed forward the progress of researches. Because TRPV1 works as a sensor for nociceptive stimuli, this award would lead to not only the progress of research for regulating our sensory systems, but also the development of novel analgesic agents. I will talk about the reserach history of thermosensitive TRP channesl which I have been working on for more than 25 years.

### [3AS02m-02]

### A new perspective in the study of TRPM2, a body temperature sensor

*Makiko Kashio[1] ([1]*Div. Cell Signaling, NIPS*)

Temperature-sensitive transient receptor potential (TRP) channels, so-called thermo-TRPs, constitute important sensors for wide range of temperatures. Thermo-TRPs are expressed not only in sensory neurons to detect environmental temperature changes but also in deep organs which are not exposed to drastic temperature changes exceeding daily fluctuation of body temperature. These facts strongly suggest that their activities at body temperature are necessary to be regulated to exert the physiological functions as body temperature sensors. TRPM2 is one of thermo-TRP working at core body temperature. Temperature threshold of TRPM2 is regulated by multiple physiological factors, adenosine diphosphate ribose (ADPR), redox signal [1], cytosolic $Ca^{2+}$ and phosphorylation of TRPM2 [2]. Moreover, TRPM2 is reported to interact with other molecules to form a signalosome for specific functions. TRPM2 activity at body temperature could be modified responding to physiological statuses through the threshold regulation and the intermolecular interactions. Recent progress in understanding of TRPM2 will be discussed. [1] Kashio et al., *Proc Nat Acad Sci USA.* 2012;109;6745-50 [2] Kashio et al., *J Physiol.* 2022;600;4287–302

### [3AS02m-03]

### TRPV4 modulates adult neurogenesis via microglial engulfment

*Ryuta Koyama[1] ([1]*Laboratory of Chemical Pharmacology, Graduate School of Pharmaceutical Sciences, The University of Tokyo*)

Social stress impairs hippocampal neurogenesis and causes psychiatric disorders such as depression. Recent studies have highlighted the significance of increased body temperature in stress responses. However, whether and how social stress-induced hyperthermia affects hippocampal neurogenesis remains unknown. Here, using transgenic mice in which the thermosensitive transient receptor potential vanilloid 4 (TRPV4) is conditionally knocked out in Nestin-expressing neural stem cells (NSCs), we found that social defeat stress (SDS)-induced hyperthermia activates TRPV4 in NSCs in the dentate gyrus and thereby impairs hippocampal neurogenesis. Specifically, SDS activated TRPV4 in NSCs, and induced the externalization of phosphatidylserine in NSCs, which was recognized by the brain-resident macrophage, microglia, and promoted the microglial engulfment of NSCs. SDS-induced impairment of hippocampal neurogenesis was ameliorated by NSC-specific knockout of TRPV4 or pharmacological removal of microglia. Thus, this study reveals a previously unknown role of thermosensitive receptors expressed by NSCs in stress responses.

### [3AS02m-04]

### Opposing roles of glial TRP channels in vascular cognitive impairment induced by chronic cerebral hypoperfusion

*Hisashi Shirakawa[1] ([1]*Department of Molecular Pharmacology, Graduate School of Pharmaceutical Sciences, Kyoto University*)

Vascular cognitive impairment (VCI) is a syndrome defined as cognitive decline due to vascular disease. Growing evidence suggests that chronic cerebral hypoperfusion (CCH), which is caused by aging, metabolic syndromes, atherosclerosis, hypertension and hypotension, is common to various types of dementia, and CCH-associated small vessel disease and white matter injury resulting from CCH are the primary causes of VCI; however the precise molecular mechanisms of pathogenesis and its protection associated with VCI remain to be elucidated. We used bilateral carotid artery stenosis (BCAS) mice as a model of CCH-induced VCI and have previously demonstrated that mild blood-brain barrier disruption and reactive oxygen species (ROS) generation lead to CNS inflammation and white matter injury, which in turn led to cognitive impairment. In the pathogenesis of BCAS-induced cognitive impairment, we investigated the involvement of TRPM2, a moderate temperature- and ROS-sensitive $Ca^{2+}$-permeable cation channel, and found that TRPM2 mediates the activation of microglia, the glial cells responsible for immunity in the CNS, and contribute to the worsening of the symptoms. In addition, we have recently found that TRPA1, another temperature- and ROS-sensitive TRP channel expressed in astrocytes, the most abundant glial cells, inhibited white matter injury by producing LIF in the pathogenesis of BCAS. These findings suggest that TRP channels expressed in glial cells have a variety of roles and that TRPM2 and TRPA1 could be potential therapeutic targets for CCH-related VCI.

### Symposium

[3S03m]
**Elucidation of the molecular and neural basis of sleep quality and its physiological action**

March 16 (Thu.), 9:00 - 11:00, Room 3

### [3S03m-01]

### Investigation of the bidirectional relationship between REM sleep and stress using a mouse model of depression

*Shinnosuke Yasugaki[1,2], Mitsuaki Kashiwagi[1], Mika Kanuka[1], Iyo Koyanagi[1,3], Masanori Sakaguchi[1], Masashi Yanagisawa[1], Yu Hayashi[1,4,5] ([1]*WPI-IIIS, Univ of Tsukuba*, [2]*Doc Program in Biomed Sci, Grad Sch of Comprehensive Hum Sci, Univ of Tsukuba*, [3]*Doc Program in Neurosci, Degree Programs in Comprehensive Hum Sci, Grad Sch of Comprehensive Hum Sci, Univ of Tsukuba*, [4]*Dep of Bio Sci, Grad Sch of Sci, Univ of Tokyo*, [5]*Hum Health Sci, Grad Sch of Med, Kyoto Univ*)

Our sleep comprises 2 states: rapid eye movement (REM) sleep and non-REM (NREM) sleep. Currently, the physiological function of REM sleep is largely unknown. Here, we focused on depression. Patients with depression almost inevitably exhibit sleep disorders. In particular, abnormalities in REM sleep, most commonly increases in REM sleep amounts, are frequently observed. There are controversies as to whether increased REM sleep helps recovery from depression or rather contributes to worsening of the depression-related symptoms. As a first step to address this using mice, we analyzed the effects of 10 days of social defeat stress on sleep in mice. Similar to patients with depression, stress exposure in mice dramatically changed some of the parameters in REM sleep. For example, the stress exposure increased REM sleep amount and this increase in REM sleep tended to attenuate after chronic stress exposure. Based on these results, we next investigated the effects of artificially increasing REM sleep at specific timings by chemogenetic activation of the REM sleep-promoting neurons recently identified in our group. We found that repeated activation of these neurons altered the behavioral phenotype induced by chronic social defeat stress. Now we are trying to elucidate how the activation of REM sleep-promoting neurons contributed to behavioral phenotypes by REM sleep-specific optogenetic intervention. Our future work will provide new insights to address the causal relations between stress resilience and REM sleep.

### [3S03m-02]

### Kinase signaling pathway regulating non-REM sleep

*Hiromasa Funato[1,2] ([1]*Toho Univ*, [2]*Univ Tsukuba*)

Sleep is homeostatically regulated, and several neuronal groups have recently been reported to be involved in sleep homeostasis. However, it remains unclear what intracellular events regulate the amount and depth of sleep through specific cell groups. Previously, we identified SIK3 as a key component in sleep homeostasis through EEG/EMG-based screening of randomly mutagenized mice. A splice mutation in the *Sik3* gene leads to an increase in NREMS amounts and EEG delta power during NREMS. Here, through a forward genetic approach in mice, we identified HDAC 4 as a sleep-regulating molecule. Haploinsufficiency of HDAC4, a substrate of salt-inducible kinase (SIK3)3, increased sleep. In contrast, mice showed decreased sleep when lacking SIK3 or having an HDAC4(S245A) mutation conferring resistance to phosphorylation by SIK3. Neuron type- and brain region-specific manipulation of SIK3 and HDAC4 revealed that SIK3 signalling in excitatory neurons located in the cerebral cortex and hypothalamus positively regulates EEG delta power during NREMS quality and quantity, respectively. This study provides the basis for linking the intracellular events and the circuit-level mechanisms that control NREMS.

### [3S03m-03]

### Sleep state-dependent reciprocal interaction between PGO waves and hippocampal neural activity

*Tomomi Tsunematsu[1] ([1]*Tohoku University*)

Ponto-geniculo-occipital (PGO) waves are a prominent electrophysiological marker of rapid eye movement (REM) sleep. PGO waves were discovered in cats in the 1960s; for more than 50 years they were thought to be absent in mice, but we have recently succeeded in recording PGO waves in mice. Although PGO waves have long been recognized, they have been fewer studies compared to other sleep-related neural events. Here, by performing *in vivo* electrophysiological experiments in mice, we unveiled the state-dependent modulatory effects of PGO waves on hippocampal excitability. During non-REM (NREM) sleep, hippocampal sharp wave-ripples (SWRs) precede PGO waves. Although PGO waves are functionally coupled with SWRs, we discover that SWRs coupled with PGO waves are short-lasting compared to SWRs without PGO waves. In contrast, PGO waves during REM sleep are followed by the firing of hippocampal neurons and phase-locked with hippocampal theta rhythms. Theta oscillations and SWRs have both been implicated in memory consolidation, so PGO waves may also contribute to this process in a coordinated way. We additionally suggest that PGO waves may also have a state-dependent physiological role.

### [3S03m-04]

### Inspiring cognitive inference in cortical network during REM sleep

*Kaoru Inokuchi[1] ([1]*University of Toyama*)

Sleep has been proposed to facilitate inference, insight and innovative problem-solving. However, it remains unclear how and when the subconscious, but not conscious, brain can create novel ideas. In a transitive inference paradigm, mice gained the inference one day, but not shortly, after complete training. Inhibiting the neuronal computations in the anterior cingulate cortex (ACC) during post-learning sleep, but not during wakefulness, disrupted the inference without affecting the original memories. Furthermore, after insufficient learning, artificial activation of medial entorhinal cortex-ACC dialogue during only REM sleep created inferential knowledge. These findings establish causal evidence for the necessity and sufficiency of REM sleep in reorganizing existing knowledge to achieve novel inference, thereby highlighting the power of the idling brain in creativity and cognitive flexibility. Evolution of neuronal dynamics representing the inference will be also discussed.

### [3S03m-05]

### Genetic dissection of neural mechanisms underlying the central circadian clock

*Michihiro Mieda[1] ([1]*Kanazawa Univ.*)

The circadian rhythm regulates when to sleep. The central circadian clock of the suprachiasmatic nucleus (SCN) is a network consisting of various types of GABAergic neurons. Although individual cells have a molecular clock driven by autoregulatory transcriptional/translational feedback loops of clock genes, intercellular communication among SCN neurons is essential for the SCN to generate a highly robust circadian rhythm. However, such network mechanisms of the SCN remain unclear. We have been focusing on the roles of arginine vasopressin (AVP)-producing GABA neurons in the SCN shell. Disruption of molecular clocks, specifically in AVP neurons, attenuated circadian behavior rhythm. In addition, the period of molecular clocks in AVP neurons is likely the primary determinant of the ensemble period of the SCN network. Furthermore, GABAergic transmission from AVP neurons regulates the timing of SCN neuronal firing to temporally restrict circadian behavior to appropriate time windows in SCN molecular clocks. Thus, AVP neurons of the SCN may be an essential component for the generation and period-setting of circadian rhythm and the coordination of the time at which the SCN allows the animal's daily behavior.

### Symposium

### [3AS04m]
**Next-generation technologies for the functional analysis of life at the organism level**

March 16 (Thu.), 9:00 - 11:00, Room 4

### [3AS04m-01]
**PITT, *Easi*-CRISPR and *i*-GONAD: genome engineering tools in mice**

*Masato Ohtsuka[1] ([1]*School of Medicine, Tokai University*)

Genetically engineered mice (GEM) have been used in various studies as animal models for human diseases. In this talk, I will introduce three original methods to generate GEM using site-specific recombination (PITT) and CRISPR genome editing system (*Easi*-CRISPR and *i*-GONAD). PITT: We have developed a microinjection-based method to generate Tg mice in which a single copy transgene can be inserted at a predetermined locus for stable and reproducible transgene expression.*Easi*-CRISPR: One of the major challenges of CRISPR system was poor efficiency of insertion of longer DNA cassettes. We recently demonstrated that long single-stranded DNAs serve as very efficient donors for DNA cassette insertion experiments. This *Easi*-CRISPR approach generates flox and insertion alleles in 8.5-100% of live offspring.*i*-GONAD: Mouse transgenesis usually involves three major technical steps; isolation, *ex vivo* handling and subsequent transfer of eggs. We developed a new system called *i*-GONAD that could bypass these steps of traditional methods. The *i*-GONAD method enables genome editing in mice through intraoviductal injection of genome editing components followed by an *in vivo* electroporation.

### [3AS04m-02]
**Development of *in vivo* genome editing technology in somatic cells**

*Keiichiro Suzuki[1] ([1]*Osaka University*)

Recently, various engineered nucleases including CRISPR-Cas9 have been developed and gene manipulation (i.e. genome editing) is revolutionizing basic biology and biomedical research. Despite rapid advances in the field, *in vivo* targeted transgene integration was inefficient especially for non-dividing somatic cells which compose most adult tissues. This poses a huge barrier for uncovering fundamental biological principles and developing treatments for a broad range of devastating genetic disorders. Here, I describe a versatile *in vivo* gene-manipulation methodology “Homology-Independent Targeted Integration (HITI)” that enables targeting a broad range of cell types and mutations in non-dividing somatic cells. As a proof of concept of their therapeutic potential, we demonstrated the efficacy of our developed method in improving disease-related phenotypes of several animal models. The developed methods establish new avenues for basic research and genome-editing therapies.

### [3AS04m-03]
**Photo-isolation chemistry for high-resolution and deep spatial transcriptome with tissue sections**

*Shinya Oki[1] ([1]*Kyoto University*)

Tissues are composed of a wide variety of cell types with spatially specific gene expression in multicellular systems. Spatial transcriptomics have been developed with various resolutions to understand gene expression with spatial relationships among cells. We have developed photo-isolation chemistry (PIC) that enables to isolate transcriptome information from locally defined areas by photo-irradiation. PIC has the advantages of detection depth and spatial resolution, ranging from large to small areas such as brain fields, embryonic tissues, single cells, and intracellular structures. PIC protocols have been optimized for formalin-fixed frozen and paraffin sections, as well as the original protocols for fresh-frozen sections. Therefore, PIC would be applied for pathological tissues to perform clinical diagnosis and to find disease-specific marker genes.

### [3AS04m-04]
**Establishing Cell-omics technology by systematic observation and analysis of cell circuits**

*Etsuo A. Susaki[1,2] ([1]*DBSB, Juntendo Univ. Grad. Sch. Med.*, [2]*Lab. for Synthetic Biology, RIKEN BDR*)

Modern tissue clearing and three-dimensional (3D) imaging have enabled cellomics, a framework for comprehensively observing and analyzing a whole organ/body at the single-cell level. This presentation will provide an overview of the cellomics workflow, with a focus on our latest achievements in 1) a highly efficient 3D tissue staining and imaging method (CUBIC-HistoVIsion or CUBIC-HV) and 2) a cross-state analysis of multicellular 3D point clouds (CellClouds). CUBIC-HV is a rationally designed 3D staining and imaging protocol based on our discovery of biological tissue as an electrolyte gel. The world's best 3D staining technique enabled uniform dye and antibody labeling of cm-cubic 3D organs, such as whole mouse and rat adult brains. The 3D imaging data can be processed computationally and converted into multicellular 3D point clouds for subsequent quantitative analysis. As part of the post-imaging workflow, a web-based cloud software (CUBIC-Cloud) enabled single-cell resolution quantitative analysis of the HV-processed whole mouse brains. Furthermore, CellClouds allowed for a comparative analysis of muticellular states based on biological spatial contexts but without their contents (molecular expressions). CellClouds successfully classified multiple brain and organoid states using their 3D point cloud data, paving the way for a novel strategy to analyze collective multicellular systems. These cellomics techniques serve as an advanced basis for systematic analysis of various multicellular systems in various biomedical fields.

### [3AS04m-05]
**Redundant and specific roles of growth factors in collective cell migration**

*Michiyuki Matsuda[1], Naoya Hino[1], Eriko Deguchi[1], Shuhao Lin[1], Kimiya Matsuda[1], Kenta Sumiyama[3], Kenta Terai[2] ([1]*Graduate School of Biostudies, Kyoto University*, [2]*Graduate School of Medicine, Kyoto University*, [3]*Graduate School of Bioagricultural Sciences, Nagoya University*)

Epidermal growth factor (EGF) and EGF receptor (EGFR) play a critical role in the propagation of ERK MAP kinase activation during collective cell migration of Madin-Darby canine kidney (MDCK) cells. The ERK activation waves generated at the leader cells promote follower cell migration by providing directional information. MDCK cells express four EGF receptor ligands, EGF, HB-EGF, TGFα, and EREG, and three EGFR-family proteins, EGFR, ErbB2, and ErbB3. By the CRISPR/Cas9-mediated gene knockout, we revealed that all of the four EGFR ligands contribute to the ERK activation waves during collective cell migration. It was also found that EGFR plays the principal role and ErbB2 and ErbB3 play an auxiliary role in the propagation of ERK activation. Meanwhile, lamellipodial extension at the front increases the cellular sensitivity to hepatocyte growth factor (HGF). The HGF-dependent sustained ERK activation in turn promotes lamellipodial extension and traction force generation. Collectively, our findings reveal that HGF and EGF ligands cooperatively drive the organized collective cell migration of the epithelial cells.

### Symposium

[3S06m]
International Relations Committee
**Adaptative regulation of muscle contraction in health and disease**

March 16 (Thu.), 9:00 - 11:00, Room 6

### [3S06m-01]

### Role of the mechanosensing machinery in skeletal muscle homeostasis

Kotaro Hirano[1,2], *Yuji Hara[1] ([1]*Univ. of Shizuoka,* [2]*Kyoto Univ.*)

Skeletal muscle is composed of thousands of multinucleated cells called myofibers, which possesses regenerative capacity after muscle injuries caused by excessive exercise. Muscle-resident stem cells, called muscle satellite cells (MuSCs), play a fundamental role in regeneration of myofibers to maintain skeletal muscle homeostasis. Mechanosensation is presumed to be involved in myofber regeneration, but the molecular entity that converts the mechanical stimuli into biochemical signals for myofiber regeneration remains to be elucidated. Here we identify PIEZO1, a mechanosensitive ion channel that is activated by membrane tension, as a key regulator for muscle regeneration. Fluorometric $Ca^{2+}$ imaging detected PIEZO1-dependent $Ca^{2+}$ fluctuation in freshly isolated MuSCs. Moreover, regeneration of myofibers after muscle injury was significantly delayed in MuSC-specific *Piezo1*-deficient mice, at least in part due to mitotic defects of MuSCs. These results suggest that mechanosensation through PIEZO1 acts as a critical determinant for MuSC-dependent myofiber regeneration.

### [3S06m-02]

### Functional analysis of type 1 ryanodine receptor in skeletal muscle using a novel animal model

*Toshiko Yamazawa[1] ([1]*Core Research Facilities, The Jikei University School of Medicine*)

Type 1 ryanodine receptor (RYR1) / $Ca^{2+}$ release channel on the sarcoplasmic reticulum (SR) is required for excitation-contraction coupling in skeletal muscle. Mutations in RYR1 cause severe muscle diseases, such as malignant hyperthermia (MH), a disorder of $Ca^{2+}$-induced $Ca^{2+}$ release (CICR) through RYR1 from the SR. Recently we generated an MH model (R2509C-RYR1 mice) carrying a p.R2509C mutation in RYR1 using the CRISPR/Cas9 system. In R2509C-RYR1 heterozygous mice, MH-like episodes were induced by volatile anesthetics as well as by an increase in environmental temperature. On the other hand, because R2509C-RYR1 homozygotes mice died *in utero*, we generated primary cultured skeletal myocytes to analyze morphological and functional changes. In this symposium, we would like to introduce that the relationship between thermal signaling and $Ca^{2+}$ homeostasis using this MH model mouse.

### [3S06m-03]

### Furthering insights into sarcoplasmic reticulum $Ca^{2+}$-ATPase (SERCA) regulation in skeletal muscle

*Robyn Murphy[1] ([1]*La Trobe University*)

Skeletal muscle is heterogeneous, being comprised of two major cell or fibre types (Type I/II) distinct in metabolic and/or contractile properties. A large proportion of ATP consumption in skeletal muscle is due to the activity of the sarcoplasmic reticulum (SR) $Ca^{2+}$-ATPase (SERCA). The activity and abundance of SERCA is isoform dependent and fibre type specific. Given the presence of Type I and II fibres side by side in the typically studied *vastus lateralis* muscle of humans, studies at individual muscle fibre level are necessary to elucidate how SERCA might play a functional role in skeletal muscle overall. Adding complexity, the function of SERCA can be influenced by its regulatory proteins, phospholambdan and sarcolipin, which are also fibre type specific. The presence of these proteins will be presented and discussed in relation to calcium regulation in skeletal muscle. This work adds to understanding how muscle fibres are able to differentially regulate $Ca^{2+}$ uptake into the SR. It demonstrates that when muscle is examined at the single fibre level it is possible to obtain the most meaningful mechanistic data about the function of SERCA as well as its regulatory properties.

### [3S06m-04]

### Adaptative regulation of myometrial contractility in pregnancy and labour

*Susan Wray[1] ([1]*university of Liverpool*)

The physiology and function of the myometrial smooth muscle provides one of the most extreme examples of adaptive regulation and control of contraction. How does a small hollow organ increase in size, and overcome the effects of stretch on contraction, which could produce premature birth? I will also address research showing that during labour there are endogenous feedback mechanisms that help prevent contractility being too prolonged (tetanic) and stopping blood flow to the fetus. The intrinsic links between metabolism and contractility are key adaptive mechanisms that help ensure successful abours. I will show how this physiological understanding can be applied clinically to help difficult labours.

### Symposium

[3S07m]
Cooperation with Other Societies Committee
**Frontiers of structure-function relationship analysis: Visualization of dynamic structural features underlying life phenomena**

March 16 (Thu.), 9:00 - 11:00, Room 7

### [3S07m-01]

### Intracellular live imaging of organelle structures and mechanical properties by nanoendoscopy using AFM

*Keisuke Miyazawa[1,2], Takeshi Fukuma[1,2] ([1]*Kanazawa Univ.*, [2]*WPI-NanoLSI*)

Atomic force microscopy (AFM) is the only technique that allows label-free imaging of nanoscale biomolecular dynamics, playing a crucial role in solving biological questions. However, such imaging is possible only for systems either extracted from cells or reconstructed on solid substrates. Thus, nanodynamics inside living cells largely remain inaccessible with the current nanoimaging techniques. Here, we overcome this limitation by the nanoendoscopy-AFM, where we fabricate a needle-like tip, and insert it into a living cell to measure a 3D force image. By using the nanoendoscopy-AFM, we visualized 2D or 3D internal structures of living cells by the measured 3D force during three-dimensional tip scanning. Our results showed undetectable changes by the previous methods such as actin fiber three-dimensional (3D) maps, and 2D nanodynamics of the membrane inner scaffold in the living cells. Unlike previous AFM methods, the needle tip directly accesses the target intracellular components, exploiting all the AFM capabilities, such as high-resolution imaging, nanomechanical mapping, and molecular recognition. These features should greatly expand the range of intracellular structures observable in living cells, and contribute to the various life science research fields.

### [3S07m-02]

### Ultrastructural observation of organelles by serial electron microscopy and Deep-learning based image analysis

*Yusuke Hirabayashi[1], Shogo Suga[1], Koki Nakamura[1], Yu Nakanishi[1], Bruno Humbel[2], Hiroki Kawai[1] ([1]*University of Tokyo*, [2]*Okinawa Institute of Science and Technology Graduate University*)

Recent advances in automated serial scanning electron microscopy (SEM) provide the community with large volume 3D EM datasets. Although Deep-Learning (DL) algorithms have been implemented recently to facilitate the segmentation of SEM volumes, DL-based image analysis remains a nascent field, and DL-based prediction lacks robust validation in most of the studies. Therefore, laborious manual proofreading is required to enable biological analyses. This often hampers or even misleads biological conclusions.Here, to overcome this major roadblock, we implemented a Human-in-the-loop (HITL) approach for creating highly accurate deep learning model by engineering a new open-source analysis platform called Python-based human-in-the-loop workflow (PHILOW) equipped with a GUI and workflows suitable for handling 3D data. We applied our HITL-TAP method to segmentation of mitochondrial cristae and showed that this method leads to an unprecedented quality of cristae segmentation, largely surpassing human performance.

### [3S07m-03]

### Three-dimensional structural analysis of dendritic spines of memory-related neurons using the super-resolution technique

*Yutaro Kashiwagi[1], Hiroshi Terashima[1], Shigeo Okabe[1] ([1]*The University of Tokyo*)

The hippocampus is a brain region that plays an essential role in memory acquisition and storage. Memory is thought to be encoded by a subpopulation of synaptically connected neurons. Most excitatory synapses in the hippocampal pyramidal neurons are formed onto dendritic spines, micron-scale postsynaptic structures. Therefore, imaging-based analysis of spine size and morphology, which are regulated by learning-related neuronal firing, is necessary to understand the encoding process of memory in the hippocampus. However, with current microscopic techniques, it is challenging to achieve nano-scale imaging of spines distributed across the broad area of the hippocampus. Here, we utilized a recently developed super-resolution technique, expansion microscopy (ExM), to analyze the morphological characteristics of dendritic spines in neurons involved in forming memory engrams. We first established an efficient method to analyze more than a thousand spines by combining ExM-based spine imaging and computational 3D-image analysis. Next, we combined the identification of immediate-early gene-positive neurons after fear conditioning with the subsequent ExM-based spine imaging. To identify synaptically connected engram neurons, we are currently developing a tracing technique of axons from the CA3 pyramidal neurons to their synaptic counterparts. These technologies will enable us to analyze the morphology of spine synapses in the neural circuits underlying learning and memory processes.

### [3S07m-04]

### Multidimensional intravital imaging based on novel multi-photon microscopic technologies

*Kohei Otomo[1,2,3] ([1]*Graduate School of Medicine, Juntendo University*, [2]*Exploratory Research Center on Life and Living Systems, National Institutes of Natural Sciences*, [3]*National Institute for Physiological Sciences, National Institutes of Natural Sciences*)

Multi-photon excitation microscopy is used for thick biological specimens because of its superior penetration depth and less invasiveness owing to its near-infrared excitation laser light pulses. Our technological improvement studies for multidimensional intravital imaging are introduced in this presentation. To improve the spatial resolution, we applied stimulated emission depletion (STED) microscopic technologies, resulting in 5-times higher spatial resolution than conventional [Otomo+ *OEx* 2014; Otomo+ *BOEx* 2018; Ishii+ *BOEx* 2019; Ishii+ *in revision*]. In addition, we also introduced adaptive optics [Yamaguchi+ *PLOS One* 2020; Yamaguchi+ *ACS Omega* 2021] and nano-sheet-based observation methods [Takahashi+ *iSci.* 2020; Takahashi+ *Star Protoc.* 2021] for sharper visualization of intravital microstructures. For higher temporal resolution, a confocal spinning-disk scanner and high-peak-power laser light sources were applied, enabling simultaneous approximately 200 foci scanning [Otomo+ *Anal. Sci.* 2015; Goto+ *Front. Phys.* 2019; Otomo+ *BBRC* 2020; Kamada+ *Sci. Rep.* 2022]. Moreover, we recently proposed an easy-to-use light-needle creating devise for volumetric imaging [Chang+ *Sci. Rep.* 2022]. These developed systems are being used for several biological applications.

### Symposium

[3S08m]
**New insights into central dopaminergic system**

March 16 (Thu.), 9:00 - 11:00, Room 8

### [3S08m-01]

### Role of cholinergic interneurons of the nucleus accumbens in dopamine responses to rewarding stimuli

*Akinori Nishi[1], Yukie Kawahara[1] ([1]*Dept of Pharmacology, Kurume Univ Sch of Med*)

Dopaminergic neurons projecting to the nucleus accumbens (NAc) are responsible for reward-related behaviors, and their function is impaired in depression. p11 is a key regulator of depression-like behaviors. We recently reported that p11 expressed in cholinergic interneurons (CINs) of the NAc plays a critical role in activating CINs to mediate dopamine responses to rewarding stimuli (cocaine, palatable food and female mouse encounter). The dopamine responses to rewarding stimuli and acetylcholine release were attenuated (cocaine) or abolished (palatable food and female mouse encounter) in mice with deletion of p11 from cholinergic neurons (ChAT-p11 cKO mice). Chemogenetic studies revealed that p11 is required for activation of CINs in response to rewarding stimuli. We are now investigating the excitatory inputs to CINs of the NAc when mice are exposed to rewarding stimuli, and found that the hippocampus plays an important role in the excitation of CINs in response to platable food and female mouse encounter, but not cocaine. The role of CINs of the NAc in dopamine responses to rewarding stimuli and their roles in depression will be discussed.

### [3S08m-02]

### Dopamine neurotransmission mechanisms in the nucleus accumbens for flexible cognitive behavior

*Takatoshi Hikida[1], Tadaaki Nishioka[1], Tom Macpherson[1], Kosuke Hamaguchi[2] ([1]*Osaka University Institute for Protein Research,* [2]*Kyoto University*)

Cognitive flexibility is required for decision-making behavior, and cognitive impairment can be observed in various mental disorders. Nucleus accumbens (NAc) is one of key neural substrates for cognitive behavior in the cortico-basal ganglia circuit, and is regulated by dopamine neurotransmission. Within the NAc, dopamine D1 and D2 receptor-expressing medium spiny neuron (D1-/D2-MSNs) in the direct and indirect pathways have been revealed to play important roles in controlling reward and aversive behavior, respectively. However, the collaborative role of NAc D1-/D2-MSNs in flexible cognitive behavior has been remained. In this study, using a visual discrimination task in mice, we assessed the role of NAc D1-/D2-MSNs in cue-guided reward-based decision-making behavior. Cell-type specific neuronal silencing and in-vivo calcium imaging revealed NAc D1-/D2-MSNs to separately contribute to cue-guided reward-based decision-making behavior. Our findings indicate that neural circuit mechanisms within NAc underlies flexible cognitive behavior and the pathophysiology of mental disorders.

### [3S08m-03]

### Extracellular sugar modification regulates neuronal signaling in the nucleus accumbens

*Masayuki Masu[1], Ken Miya[1], Kazuko Keino-Masu[1], Takuya Okada[1], Kent Ohta[2], Tom Macpherson[2], Takatoshi Hikida[2], Etsuko Suzuki[3], Toshihiko Momiyama[3] ([1]*University of Tsukuba,* [2]*University of Osaka Institute for Protein Research,* [3]*The Jikei University School of Medicine*)

Heparan sulfate (HS) is a polysaccharide attached to the core proteins of proteoglycans. It plays critical roles in cell growth, differentiation, migration, axon guidance, synapse formation, and synaptic plasticity in the nervous system. HS endosulfatases, Sulf1 and Sulf2, are extracellular enzymes that remove 6-*O*-sulfate from HS and regulate cellular signaling. We previously reported that *Sulf1/2* are required for cell differentiation and axon guidance. Although the roles of *Sulf1/2* in development have been extensively studied, their functions in the mature brain remain largely unknown. Recently we found that *Sulf1* mRNA is highly expressed in the nucleus accumbens, paraventricular nucleus of the thalamus, the posterior tail of the striatum, and layer 6 of the cerebral cortex in the adult mouse brain. Moreover, we showed that *Sulf1*-expressing neurons in these regions coincided with the cells expressing dopamine D1 receptor (D1R) and/or D2R. To test whether *Sulf1* is involved in neuronal transmission in the nucleus accumbens, we performed behavioral tests and electrophysiological measurement using *Sulf1* knockout mice. In this symposium, we discuss the roles of HS modification in neuronal signaling.

### [3S08m-04]

### Chemogenetic manipulation of ventral tegmental area dopamine neurons alters multidimensional roles in motivated behavior

*Kazuto Kobayashi[1], Yoshio Iguchi[1] ([1]*Fukushima Medical University*)

Dopamine (DA) transmission is associated with reinforcement of goal-directed behavior and its dysfunction leads to the pathogenesis of anhedonia and amotivation shared by many psychiatric disorders. We developed a new reinforcement schedule for the operant lever-pressing with food reward in rats that allows to evaluate two motivational parameters "hedonic set point" and "demand elasticity", which are proportional to the inverse of the reward and motivational effects, respectively. To investigate the role of DA neurons in the ventral tegmental area (VTA) in the regulation of these parameters, we employed an ionotropic chemogenetic tool, ivermectin (IVM)-gated chloride channel (GluCl) derived from C. elegans to induce a reduction in the activity of target neurons. We induced an expression of GluCl in the VTA-DA neurons using the Cre-loxP system in transgenic rats and tested the behavioral effects of IVM treatment. Systemic administration and microinjection of IVM into the nucleus accumbens (NAc) core significantly increased both demand elasticity and hedonic set point in the rats. IVM microinjection into the VTA increased only demand elasticity. These results indicate the multidimensional roles of the VTA-NAc circuit in the control of reinforcement, suggesting the presence of the complex mechanism to modulate the reward effect through the NAc.

### [3S08m-05]

### Repairs of neurons and synapses in central dopaminergic system

*Etsuko Suzuki[1], Yutaka Mine[1,2,3,4], Toshihiko Momiyama[1] ([1]*Jikei University, Sch. of Med. Dept. Pharmacology,* [2]*National Hospital Organization, Tokyo Medical Center, Dept. of Neurosurgery,* [3]*Keiko University Sch. of Med. Dept. Physiology,* [4]*National Hospital Organization, Tochigi Medical Center, Dept. Clinical Research*)

We have been investigating the repair process of neurons and synapses in the basal ganglia of Parkinson's disease (PD) model rats and mice. Grafted mecencephalic neuroepithelial stem cells (M-NESCs,) of mini swine survived after implanted in PD rats. Many of the rafted cells differentiated to tyrosine hydroxylase (TH)-positive neurons. Amphetamine-induced rotation in host PD rats was reduced after implantation of swine M-NESCs. Whole-cell recordings from donor-derived neurons in the host rat brain slices revealed the presence of multiple types of neurons including dopaminergic, and showed that donor-derived cells receive both excitatory and inhibitory synaptic inputs from host area. These results shows that non-rodent mammalian M-NESCs can differentiate into functionally active neurons in the diseased xenogeneic environment and could improve the parkinsonian behavioural defects over the species. Since recent progress in the use of human iPS cell-derived midbrain dopaminergic progenitors is remarkable, we are carrying out implantation of human iPS- neural stem cells (NSCs) into PD mice. In this symposium, we'll also report the results of these experiments using human iPS-NSCs.

### Symposium

[3S09m]

**Dynamic correlation between subcellular structures and functions for neuronal signaling**

March 16 (Thu.), 9:00 - 11:00, Room 9

### [3S09m-01]

### Activity-dependent regulatory mechanism of axonal excitability

*Haruyuki Kamiya[1] ([1]*Department of Neurobiology, Hokkaido University Graduate School of Medicine*)

Modification of axonal excitability impacts information transfer through the neuronal networks. However, the significance of excitability tuning of the axon by the preceding neuronal activity largely remains elusive. One remarkable exception is the activity-dependent broadening of action potential (AP) propagating along the hippocampal mossy fibers. The duration of AP is progressively prolonged during repetitive stimuli and facilitated presynaptic $Ca^{2+}$ entry and subsequent transmitter release. As an underlying mechanism, accumulated inactivation of axonal $K^+$ channels during AP train has been postulated. Since the inactivation of axonal $K^+$ channels proceeds on a time scale of several tens ms slower than the ms scale of AP, the contribution of $K^+$ channel inactivation in AP broadening is waiting to be tested and evaluated quantitatively. Using the simulation approach, it was attempted to explore the effects of the removal of inactivation of axonal $K^+$ channels and found that the use-dependent AP broadening was abolished. The results demonstrated the critical roles of $K^+$ channel inactivation in activity-dependent regulation of axonal excitability.

### [3S09m-02]

### Actin-dependent spatial confinement of synaptic vesicles for high-frequency neurotransmission

*Takafumi Miki[1] ([1]*Doshisha Univ.*)

High rates of synaptic vesicle reloading and release at a release site are required for rapid information processing at cerebellar mossy fiber terminals. In a recent study, we directly observed rapid vesicle tethering that occurred with a similar time course to exocytosis at the terminals. However, the mechanism of the rapid tethering remains unknown. Here, we found that actin disruption abolished the rapid tethering, while induction of actin polymerization did not affect the time course of the tethering, suggesting that the existence of actin filaments was necessary for the tethering. Analysis of synaptic vesicle trajectories obtained from single-particle tracking experiments indicated that actin disruption made vesicles diffuse faster. Inferring vesicle diffusion states by Hidden Markov modeling revealed that synaptic vesicles under normal conditions had two diffusion states: free-diffusing and trapped. After actin disruption, vesicles tended to have only the free-diffusion state. Based on these results, it is suggested that actin filaments limit synaptic vesicle movement to achieve the rapid tethering which is essential for the sustained activity at the terminals.

### [3S09m-03]

### Dynamic changes in the sizes of various pools of synaptic vesicles at simple cerebellar glutamatergic synapse

Van Tran[1], Melissa Silva[1], Takafumi Miki[2], *Alain Marty[1] ([1]*Saints Peres Paris Institute for Neuroscience,* [2]*Doshisha University Kyoto*)

In recent years our laboratory has developed methods to monitor the release of individual synaptic vesicles (SVs) at simple synapses containing one active zone (AZ) and a few vesicular docking sites (DSs). These methods allow for improved accuracy and time resolution, as well as for a direct scaling of SV pool estimates with respect to AZ and DS numbers. Here we use the simple synapse recording method to study the structure of the readily releasable pool (RRP) of SVs in cerebellar glutamatergic synapses. When increasing the probabilities of SV docking and SV release, we found a maximum value of 2 SVs for the RRP size per DS. This result constrains models of SV docking and it indicates that each DS can bind up to 2 SVs in 2 different stages of docking. Next, we examined SV pools upstream of the RRP. We found that SVs flow from a recycling pool of ~180 SVs/AZ into an intermediate pool (IP) of ~10 SVs/AZ, and then into the RRP (~7 SVs/AZ). When stimulating the synapse with repeated action potential trains at short inter-train intervals, a rapid (~10 s) recycling pathway that feeds directly into the IP was turned on. The SVs coming from this pathway are preferentially recruited to DSs compared to SVs coming from slower recycling routes. The rapid recycling pathway depends on dynamin and on myosin light chain kinase, and it provides ~200 SVs/AZ before being exhausted.

### [3S09m-04]

### Dissecting the Organisation of Morphological Synaptic Vesicle Pools by Electron Microscopy

*Cordelia Imig[1,2], Francisco José López-Murcia[2,3], Lydia Maus[2,4], Nils Brose[2], Benjamin H. Cooper[2] ([1]*University of Copenhagen, Faculty of Health and Medical Sciences, Department of Neuroscience, Copenhagen, Denmark,* [2]*Max Planck Institute for Multidisciplinary Sciences, Department of Molecular Neurobiology, Goettingen, Germany,* [3]*Present Affiliation: University of Barcelona, Institute of Neurosciences, and Bellvitge Biomedical Research Institute, L'Hospitalet de Llobregat, Barcelona, Spain,* [4]*Present Affiliation: Microscopy Core Facility, Medical Faculty, Bonn Technology Campus Life Sciences, University of Bonn, Bonn, Germany*)

Synaptic neurotransmitter release is mediated by a highly complex molecular machinery that determines the speed and spatiotemporal precision of fusion of docked vesicles at active zones, and the ability to purposefully adapt the efficacy of information transfer during ongoing synaptic activity. Although fundamental molecular and cell biological principles are highly conserved between different synapse types, distinct neuronal connections exhibit strikingly different functional properties including the probability of neurotransmitter release and short-term plasticity characteristics. The goal of our research is to understand how distinct active zone-proximal synaptic vesicle sub-pools contribute to neurotransmitter release during distinct synaptic activity states and thereby determine functional synaptic heterogeneity. We employ a combination of electrophysiology, optogenetics, light stimulation-coupled high-pressure freezing ("flash-and-freeze"), and electron microscopy techniques to probe ultrastructure-function relationships at high spatial and temporal resolution. Our research has initially focused on understanding the functional relevance of synaptic vesicle organization in thestructurally and functionally highly complex hippocampal mossy fiber synapse.

### [3S09m-05]

### Asymmetric spread of membrane potential changes in neuronal dendrites

*Shin-ya Kawaguchi[1] ([1]*Graduate School of Science, Kyoto University*)

Characteristic patterns of dendritic arborization in distinct types of neurons would impact on the integration of synaptic inputs. However, technical limitation to detect local membrane potential changes at tiny size of synapses has made it difficult to precisely tell how synaptic inputs are computed in somatodendritic regions. Recent advances of fluorescent imaging of membrane potential changes based on genetically-encoded voltage indicators (GEVIs) are making it possible to provide high spatiotemporal information about subcellular voltage signals in individual neurons even at sub-μm level of small structures such as spines and axon terminals. Using a type of GEVI, ASAP, we are studying how synaptic inputs are processed during the travelling along a dendritic tree in hippocampal pyramidal neurons and cerebellar Purkinje cells. In this symposium, I am going to demonstrate how distinct morphological design of dendrite together with different membrane excitability in those two-types of neurons give rise to diverse signal processing in dendrites, shedding light on tight relation of function and morphology of neuronal processes.

### Symposium

[3S10m]

### Circadian rhythms of intra- and extra-ocular physiological functions

March 16 (Thu.), 9:00 - 11:00, Room 10

### [3S10m-01]

**Molecular regulatory mechanisms in circadian rhythm of intraocular pressure**

*Keisuke Ikegami[1] ([1]*Aichi Med. Univ.*)

Glaucoma is the leading cause of blindness in elderly people; however, no effective cure exists. Abnormal intraocular pressure (IOP) contributes to glaucoma development and progression characterized by vision loss. IOP is formed by aqueous humor (AH) dynamics, with inflow from the ciliary body and outflow through the trabecular meshwork (TM). Since IOP has a circadian rhythm with nocturnal increase in both nocturnal and diurnal animals, controlled by the circadian pacemaker suprachiasmatic nucleus, the regulation of nocturnal IOP increase is central to glaucoma management, and the circadian mechanism in AH dynamics is important for glaucoma therapy. However, the detailed mechanisms underlying IOP rhythmicity remain unclear. We discovered direct driving of the IOP rhythm by the suprachiasmatic nucleus via the dual adrenal glucocorticoids (GCs) and sympathetic noradrenaline (NE) pathway, but not the ciliary clock. In outflow, we also revealed that TM phagocytosis suppression by NE could regulate IOP rhythm through AH outflow through β1-adrenergic receptor-mediated exchange proteins directly activated by cyclic adenosine monophosphate (EPAC)-SHIP1 signal activation. In inflow, we discovered that both NE/GC activated the key AH producer ciliary $Na^+K^+$ATPase, and explored the molecular regulatory mechanism. In this symposium, I would like to introduce these efforts.

### [3S10m-02]

**Atypical opsins regulate the circadian rhythms of the mammalian eye**

*Ethan D Buhr[1] ([1]*University of Washington*)

Circadian rhythms are present in most cell types in the eye. In the retina, circadian clocks are directly sensitive to environmental light cycles. The retina's clocks can be entrained by violet-blue light independently of the circadian phase of the animal. This does not require the rods/cones of the visual system; rather, we have found that the atypical opsin, Opn5, is necessary for direct light synchronization. The mechanism of signal transduction from Opn5-retinal ganglion cells to the rest of the retina is still unknown. Recently, we have found that light information is transmitted via diffusible signals which do not require direct cell-cell interactions. Another ocular tissue which exhibits direct circadian light-synchronization is the cornea. However, the cornea only exhibits photoreception after damage. Following an epithelial debridement, Opn5-expressing cells are induced on the surface of the cornea, and they render the tissue temporarily light sensitive. Both Opn5 and environmental short-wavelength light regulate the rate and integrity of healing in damaged corneas. Similar to the retina, the Opn5-mediated photic information is carried through paracrine signals.

### [3S10m-03]

**Circadian rhythms and corneal endothelial cell function**

*Yoshiki Tsuchiya[1], Hiroko Nakai[1,2], Kazuhiro Yagita[1] ([1]*Department of Physiology and Systems Bioscience, Kyoto Prefectural University of Medicine*, [2]*Department of Ophthalmology, Kyoto Prefectural University of Medicine*)

Circadian regulation of corneal function is one of the topics of interest in circadian physiology of the eye. The cornea is exposed to diurnal changes of environment including light and oxygen availability regulated by eye-lid closure during sleep. Circadian rhythms are observed in corneal thickness, which is regulated by water flux through the corneal endothelium that is critically important for the transparency of the cornea. However, circadian regulation of corneal physiology remains poorly understood. This talk will focus on cornea to share a better understanding of corneal circadian physiology by introducing related studies including our findings on corneal endothelial cells. Our recent study has revealed circadian clock oscillation and circadian global gene expression in cultured human corneal endothelial cells (cHCECs). Oscillating genes are involved in various physiological processes including glycolysis, mitochondrial function, antioxidative systems, and hypoxic responses, which represent the physiological functions of HCECs. A functional circadian clock in corneal endothelial cells suggests a potential contribution of circadian regulation to fine-tune corneal functions for daily changes in the environment.

### [3S10m-04]

**Reactivation of circadian clock-regulated intracrine activity ameliorates meibomian gland dysfunction and its associated dry eye disease**

*Masao Doi[1] ([1]*Kyoto University, Graduate School of Pharmaceutical Sciences*)

Canonically, hormones are produced in the endocrine organs and delivered to target tissues. However, for steroids, the concept of tissue intracrinology, whereby hormones are produced in the tissues where they exert their effect without release into circulation, has been proposed, but its role in physiology/disease remains unclear. The meibomian glands in the eyelids produce oil to prevent tear evaporation, which reduces with aging. Here, we demonstrate that (re)activation of local intracrine activity through nicotinamide adenine dinucleotide ($NAD^+$)-dependent circadian 3β-hydroxyl-steroid dehydrogenase (3β-HSD) activity ameliorates age-associated meibomian gland dysfunction and accompanying evaporative dry eye disease. Genetic ablation of 3β-HSD nullified local steroidogenesis and led to atrophy of the meibomian gland. Conversely, reactivation of 3β-HSD activity by boosting its coenzyme $NAD^+$ availability improved glandular cell proliferation and alleviated dry eye disease phenotype. Both women and men express the 3β-HSD in the meibomian gland. Enhancing local steroidogenesis may help combat age-associated meibomian gland dysfunction.Sasaki *et al.*, Nature Aging 2, 105-114 (2022); Hamada *et al.*, Ocul Surf 26, 268-270 (2022)

### Symposium

[3S03a]
International Relations Committee
**Prenatal and perinatal physiology - Adaptation to drastic environmental change**

March 16 (Thu.), 14:20 - 16:20, Room 3

### [3S03a-01]

### Hidden clocks: the importance of understanding time for diagnosis and treatment of evolving perinatal brain injury

*Laura Bennet[1], Victoria King[1], Chris Lear[1], Simerdeep Dhillon[1], Tomoaki Ikeda[2], Alistair Gunn[1] ([1]*The University of Auckland,* [2]*Mie University*)

Neonatal brain injury is associated with hypoxia-ischemia during birth. Less well appreciated, however, is the fact that such insults can occur well before birth during preterm fetal life. Such insults can cause brain injury, but fetuses survive in utero and continue to grow and develop to full term. Indeed, brain injury well before birth is a key cause of cerebral palsy, and underpins many other post-natal neurodevelopmental impairments. Detection of such injury before birth would allow us to implement neuroprotection or neurorepair treatments during pregnancy. Further, it is important to understand how pre-existing brain injury might alter the fetal adaptive responses to hypoxia experienced during labour and the physiological signals used by obstetricians to determine fetal well-being. This talk will examine how the preterm fetus can survive a severe hypoxic insult *in utero* leading to brain injury and continues to grow and develop *in utero* to full term. Data will be presented on how brain injury evolves over time, the temporal changes in fetal physiology and behaviour associated with evolving injury, and the impact of clinical drugs on these physiological and behavioural changes. Finally, research will be presented on how the temporal changes in fetal physiology and behaviour, including circadian changes, informs the search for prognostic and diagnostic biomarkers, which are important for implementation of therapeutic treatments and clinical monitoring during labour.

### [3S03a-02]

### A prenatal epigenetic fluctuation underlies systemic immune dysregulation in autism

*Chiawen Lin[1,2], Kenji Atarashi[3], Kenya Honda[3], Thomas McHugh[2], Toru Takumi[1] ([1]*Kobe University School of Medicine,* [2]*RIKEN Center for Brain Science,* [3]*RIKEN Center for Integrative Medical Sciences*)

The established role of immune dysregulation on autism etiology has emerged for over two decades. However, the molecular mechanism behind these changes remaining elusive. Considering the critical developmental windows for immune insult of autism, we suggest that tracking the origin of immune dysregulation back to embryonic stage in specific cell types should provide a rational direction to explore the underlying mechanism. In parallel, the emergence of gut-microbiota-brain axis starts another boom to pursue the role of dysbiosis in the pathogenesis of autism. However, the findings in autistic patients are often heterogenous and contradictory between studies. By using a valid mouse model of immune dysregulation, the BTBR strain, we found the increased HDAC1 activity affects the definitive hematopoiesis in yolk sac and AGM, which therefore affects the development of microglia and hematopoietic stem cells and subsequently leads to brain inflammation and skewed immune cell profiles. We also demonstrated the causality of a specific autistic immune profile with a specific pattern of dysbiosis, which reveals the potential of microbiome for the diagnosis of ASD caused by immune dysfunction. Furthermore, we identified the active endogenous retrovirus (ERV) during embryonic development works at the most upstream to predominate the epigenetic machinery. The analogy between cellular responses induced by ERV and exogenous viral infection again echoes the etiology of epigenetic perturbation in the autism models of environmental risk factor, such as maternal immune activation (MIA) and valproic acid (VPA)-induced models of autism. With the new advance in the old model, our study unravels the idiopathic etiology of BTBR strain, which not only provides evidence for the origins of immune dysregulation in autism, but also provide new insights to how the ancient viral infection affects autism susceptibility.

### [3S03a-03]

### Dynamic cardiovascular remodeling to adapt extrauterine life

*Utako Yokoyama[1], Satoko Ito[1] ([1]*Tokyo Medical University*)

Mammals undergo a sudden change from a life in the water to land at birth. The ductus arteriosus (DA) begins to close immediately after birth to adapt to neonatal circulation. Substantial progress has been made to understand how the DA matures during the fetal period. Differentiated contractile smooth muscle cells (SMCs) and prominent intimal thickening are essential for postnatal DA closure. The concentration of circulating prostaglandin $E_2$ ($PGE_2$) derived from the placenta increases toward birth, reaching a more than 10-fold concentration versus neonates after birth. $PGE_2$ receptor EP4 is specifically expressed in DA SMCs and dilates the DA through cAMP-protein kinase A pathway. Studies using EP4-deficient mice revealed that the DA utilizes $PGE_2$ for its maturation. EP4 signaling contributes to intimal thickening formation via forming a hyaluronan, fibulin-1, and versican complex and through impairment of elastic fiber formation via cAMP-independent pathways. EP4 also promotes DA SMC differentiation. $PGE_2$, therefore, plays two major roles during the fetal period, i.e., maintaining DA patency and promoting phenotypic and structural remodeling of the DA for postnatal closure. COI: None.

### [3S03a-04]

### Watching the small baby grow

*Janna Leigh Morrison[1] ([1]*University of South Australia*)

10% of babies are small for gestational age (SGA). Some were always going to be small (SGA) but many experience fetal growth restriction (FGR; ~20,000 babies/yr). This is due to adverse events such as impaired placental function and maternal hypertension, reducing oxygen and nutrient delivery to the fetus, which the fetus must adapt to. FGR is associated with low fetal oxygenation and major adverse pregnancy outcomes such as preterm birth and stillbirth. In adulthood, FGR individuals are at 40% greater risk of hypertension, a major risk for cardiovascular disease (CVD). This CVD risk is programmed during fetal life via changes to cardiac structure and function, and these programmed effects persist after birth and are dependent on the timing, duration and severity of the reduction in oxygen and nutrients. Our work focusses on two main goals: better detection of FGR to improve outcomes and finding ways to treat the FGR pregnancy to improve fetal growth.

### Symposium

[3S04a]

**Elucidation of brain information decoded by glia**

March 16 (Thu.), 14:20 - 16:20, Room 4

### [3S04a-01]

### Oligodendrocytic regulation of neuronal synchrony

*Hiroaki WAKE[1,2] ([1]*Department of Anatomy and Molecular Cell Biology Nagoya University Graduate School of Medicine,* [2]*Division of Multicellular Circuit Dynamics, National Institute for Physiological Sciences, National Institute of Natural Sciences*)

ligodendrocyte (OC) forms myelin approximately in around 10 different axons to regulate conduction velocity. Myelin formation in the central nervous system is required for the rapid and the coordinated action potential (AP) propagation across distinct brain regions including cortical and subcortical gray matter, which is important for the information processing. In contrast with the Schwan cell that only form myelin around single axon in peripheral nerve, OC forms myelin around 10 different axons. However, the functional significance for OC to form myelin around different axons remains unknown. We previously found that Oligodendrocyte form myelin depends on neuronal activity and that regulate during the learning. In this session, we focused on thalamo-cortical projection and found the arrival of AP on primary motor cortex through thalamo-cortical axons increase synchrony with the motor learning, The regulation of AP synchronized arrival was neuronal activity dependent and also required OC activity. Inhibition of OC activity resulted in asynchronized AP arrival and impaired motor learning. This neuronal activity induced increase synchrony via OC activity was associated with reduced size of node of ranvier that triggered by expression of 3'UTR of MBP in OC. In addition, that myelin regulation also through the lipid synthesis. We suggested activity dependent functional response of OC promote is required for synchrony of neuronal circuit activity. And impaired OC function may result in neurological and psychiatric diseases.

### [3S04a-02]

### Imaging neuron-glia interactions

*Shigeo Okabe[1] ([1]*Graduate School of Medicine, The University of Tokyo*)

Glial cells have diverse functions and are associated with the pathogenesis of various brain diseases. In vivo analysis of the dynamic responses of glial cells and neural circuits requires the development of new imaging techniques and integration with other methodologies. In this presentation, we will introduce novel glial cell imaging techniques and discuss the future of glial research using these techniques. First, we will show the long-term in vivo two-photon imaging techniques from deep brain tissue with minimal invasion. By optimizing surgical conditions, we have been able to observe ramified microglia and capture the events of microglial engulfing of newly born neurons in the dentate gyrus. We also introduce a technique that combines brain cell transplantation and two-photon microscopy imaging, which has a variety of potential applications in studying animal models of brain disease. The transplanted cells are incorporated into brain tissue and can be imaged in vivo. By isolating progenitor cells from disease model animals and transplanting them into wild-type animals, it is possible to verify cell-autonomous factors from the environmental effects. These technologies are expected to accelerate new discoveries about the involvement of glial cells in the pathogenesis of brain diseases.

### [3S04a-03]

### Zooming into synapse-astrocyte interactions using advanced microscopy techniques

*Misa Arizono[1,2], Valentin Nägerl[1] ([1]*University of Bordeaux,* [2]*Department of Pharmacology Kyoto University Graduate School of Medicine*)

While accumulating evidence shows the involvement of astrocytes in neuronal circuits and cognitive functions, their activity and structure at individual synapses have been challenging to address with conventional light microscopy. Using live STED microscopy, we observed that the fine processes of astrocytes are composed of bulbous nodes and thin shafts. FRAP experiments and $Ca^{2+}$ imaging revealed nodes are biochemical compartments hosting $Ca^{2+}$ microdomains. Node $Ca^{2+}$ signals were associated with individual synapses, identifying nodes as the likely synaptic partner. To further characterize astrocytic nodes, we established $Ca^{2+}$ imaging using lattice light-sheet microscopy, which reconciles high spatio-temporal resolution with low phototoxicity. This approach enabled us to observe the detailed temporal nature of astrocytic $Ca^{2+}$dynamics both in 2D and 3D. We found that node $Ca^{2+}$ events can be <100 ms and be elicited by glutamate uncaging. This work presents the framework for dissecting bidirectional crosstalk between neurons and glia with unprecedented spatial and temporal resolution, bringing us closer to evaluating the impact of astrocyte-neuron interaction on neural circuit function.

### [3S04a-04]

### Synapse and network remodeling by glia

*Schuichi Koizumi[1,2] ([1]*Dept Pharmacol, Interdisc Grad Sch Med, Univ Yamanashi,* [2]*GLIA Center, Interdisc Grad Sch Med, Univ Yamanashi*)

When sense environmental changes, glial cells become reactive and greatly change brain functions. Here, we show reactive astrocyte-mediated network remodeling and its pathophysiological consequences in the primary somatosensory cortex (S1) and the hippocampus. Mechanical allodynia is caused by peripheral nerve injury, and its pathology remains unknown. After peripheral nerve injury, we found that S1 cortical astrocytes become reactive and remodel neuronal circuits by excess synapse remodeling. For these network remodeling, a re-emergence of metabotropic glutamate receptor 5 (mGluR5) and $Ca^{2+}$ elevation in S1 astrocytes have essential roles. Similar events also occurred during formation of epileptogenesis i.e., a condition in which the brain becomes prone to epileptic seizures. After status epilepticus, hippocampal astrocytes become reactive, again, increase mGluR5 and $Ca^{2+}$ signals, leading to remodel hippocampal circuits. Taken together, network remodeling by mGluR5 and $Ca^{2+}$excitation in reactive astrocyte may be a cross-disease molecular pathogenesis. We will also discuss how manipulation of these two molecules can induce 2nd remodeling and lead to treatment of these diseases.

### Symposium

[3S05a]
Science and Research Committee / The Japanese Medical Science Federation Member society Collaborative Forum
**Inter-organ communication underlying homeostasis and sustainability: mechanisms of its regulation and failure**

March 16 (Thu.), 14:20 - 16:20, Room 5

### [3S05a-01]

### A central integrated controller of multi-organ responses for body temperature regulation

*Kazuhiro Nakamura[1], Yoshiko Nakamura[1], Takaki Yahiro[1], Akihiro Fukushima[1]
([1]*Department of Integrative Physiology, Nagoya University Graduate School of Medicine*)

The central mechanism for integrated control of multi-organ responses is unknown. Using rats, here we show that prostaglandin EP3 receptor-expressing neurons in the thermoregulatory center, preoptic area (POA) ($POA^{EP3R}$ neurons) are an integrated controller of multi-organ responses for thermal homeostasis. $POA^{EP3R}$ neurons were activated in response to elevated ambient temperature, but inhibited by prostaglandin $E_2$, a pyrogenic mediator. Chemogenetic stimulation of $POA^{EP3R}$ neurons at room temperature reduced body temperature by eliciting skin vasodilation, whereas inhibition of them elicited hyperthermia involving brown fat thermogenesis and tachycardia, mimicking fever. We found that $POA^{EP3R}$ neurons innervate sympathoexcitatory neurons in the dorsomedial hypothalamus (DMH) via tonic inhibitory signaling. Although many $POA^{EP3R}$ neuronal cell bodies expressed a glutamatergic mRNA marker, their axons in the DMH predominantly released GABA and their GABAergic terminals were increased by chronic heat exposure. These findings demonstrate that tonic GABAergic inhibitory signaling from $POA^{EP3R}$ neurons is a fundamental determinant of body temperature for thermal homeostasis and fever.

### [3S05a-02]

### Exosome-mediated inter-organ communication: roles in disease etiology and biomarker potential

*Ayuko Hoshino[1] ([1]*Tokyo Institute of Technology*)

Exosomes, first thought to function only as cellular garbage disposal, are secreted by all cells and have recently been discovered that they also function as a cell-cell communication tool. We have previously shown that cancer cells send "exosomes" to the future site of metastasis to alter the environment to a favorable place where cancer cells can successfully metastasize. Furthermore, we have reported a proof of principal study showing that machine learning classification of plasma-derived exosome proteomes could identify cancer *versus* non-cancer in 95% sensitivity/90% specificity. In addition, we defined a panel of tumor-type specific exosomal proteins in plasma, which may help classify tumors of unknown primary origin. Expanding our knowledge and techniques on inter-organ communication of exosomes through cancer, we have analyzed exosomal changes and how it could contribute to different disease etiology, such as preeclampsia, Alzheimer's Disease, and autism spectrum disorder. In this talk, I will share our most recent findings on the role of exosomes in multiple disease etiology and biomarker potential.

### [3S05a-03]

### Immunoregulatory mechanisms in the gut mediated by the liver-brain-gut axis

*YOHEI MIKAMI[1], TOSHIAKI TERATANI[1], TAKANORI KANAI[1] ([1]*Division of Gastroenterology and Hepatology Department of Internal Medicine, Keio University School of Medicine*)

Inflammatory bowel disease (IBD) is currently recognised as a state of aberrant immune activation in the intestine, which are affected immunological, genetic and environmental factors. T-helper (Th) cell plays a central role on intestinal immune response in physiological and pathogenic conditions. Activation, differentiation, and maintenance of Intestinal Th cells are highly affected by the local microenvironment of the intestinal tract, including the intestinal microbiota, metabolites, food antigens, and neurotransmitters. The gut is innervated by the vagus nerve (VN), the X cranial nerve. VN has long been considered provide functional regulation of movement and gland secretion, but recent large-scale clinical studies have highlighted the immunoregulatory roles of the autonomic nervous system in the gut. In order to understand the immunoregulatory roles of VN, we utilised surgical and chemical perturbation of VN, significantly reduced the number of excited and sensory neurons in the nodose ganglion (NG), which is a sensory ganglion of VN. Disruption of VN branches connecting the liver and the brain is sufficient to decrease intestinal Treg cells and increase colitis burden. This study reveals a novel cholinergic anti-inflammatory pathway (CAIP), "Liver-Brain-Gut neural arc", that governs homeostasis in the gut. Further study is needed to elucidate the whole picture of interorgan networks in IBD pathogenesis.

### [3S05a-04]

### Regulation of pancreatic β cells by liver-β cell-inter-organ neuronal network: the role of vagal nerves signals

*Junta Imai[1] ([1]*Tohoku University Graduate School of Medicine*)

In insulin resistant states such as obesity, pancreatic β cells undergo compensatory proliferation and secrete more insulin to prevent blood glucose elevation. Previously, we discovered that pancreatic vagal nerve signals, elicited by activation of the hepatic extracellular signal-regulated kinase (ERK) pathway, play critical roles in triggering compensatory β cell proliferation during obesity development. Recently, to explore whether activation of efferent vagal nerves *in vivo* alone is sufficient to promote glucose-stimulated insulin secretion (GSIS) and β-cell proliferation, we newly developed two optogenetic vagal nerve stimulation methods. Employing these strategies, we stably and selectively activated vagal nerves innervating the pancreas and showed vagal activation to be sufficient to promote both GSIS and β-cell proliferation, thereby increasing functional β-cell mass and ameliorating insulin-deficient diabetes. Thus, vagal nerve signals are not only necessary but also sufficient to induce GSIS and β-cell proliferation, thereby increasing functional β-cell mass. Furthermore, activation of vagal nerves is a promising therapeutic option for insulin-deficient diabetes.

### [3S05a-05]

### Does Parkinson's disease start from the peripheral nervous system?

*Ryosuke Takahashi[1] ([1]*Department of Neurology, Kyoto university Graduate School of Medicine*)

Parkinson' s disease (PD) is a multisystem neurodegenerative disorder characterized by motor and non-motor symptoms. These symptoms are caused by alpha-synuclein (a-Syn) pathology such as Lewy bodies and Lewy neurites throughout the central and peripheral nervous systems. Recently, accumulating evidence suggests that a-Syn, alike prion, propagates from neurons to neurons, leading to systemic a-Syn lesions. Heiko Braak, a German neuropathologist, proposed an attractive hypothesis stating that a-Syn pathology is initially formed in the olfactory bulb and the dorsal motor nucleus of vagus and spreads upwards in a consecutive manner as the disease progresses. He also postulated that a-Syn pathology is early formed in the nerve plexus of the gut and transmitted retrogradely through the vagus nerve, suggesting the gut origin of PD. Moreover, it is proposed that PD is subdivided into brain-first and body-first subtypes, in which a-Syn pathology is originally formed in and spread from the central and peripheral nervous systems, respectively. In this lecture, I will raise the evidence suggesting the peripheral origin of PD and discuss what needs to be done to clarify this important issue.

### Symposium

[3S06a]

**Mechanisms of hippocampal function revealed by in vivo large-scale recording**

March 16 (Thu.), 14:20 - 16:20, Room 6

### [3S06a-01]

### Representation of space and direction in the avian hippocampus

*Susumu Takahashi[1] ([1]*Graduate School of Brain Science, Doshisha University*)

In the rodent hippocampus, place cells were found, and the mammalian hippocampal damage leads to deficits in episodic memory function. Does the avian hippocampus have a similar function? The medial pallium (MP) is considered homologous to the mammalian hippocampus. Pioneering studies have found cells with patchy multiple place fields on the ventral MP of pigeons. Recently, with the advent of neurologgers that can wirelessly record many single neuronal activities, the MP of various avian species has become a subject of study. Unlike the pigeon report, some cells in the dorsal MP of the titmouse, a food-caching bird, were found to have a single confined place field, like rodent place cells. However, not place cells but head direction cells were found in the dorsal MP of quails. We also found head direction cells in the dorsal MP of juvenile streaked shearwaters, a migratory bird. However, unlike rodents, their head direction preference was biased toward the geomagnetic north orientation. In this presentation, to understand the hippocampal function, we will discuss the neural representation in the MP of these avian species.

### [3S06a-02]

### Dynamic Nature of Memory Representation and the Hippocampal Network

Yu-Ju Lin[1], Thato Mokhothu[1], Miyu Nambu[1], *Kazumasa Z Tanaka[1] ([1]*Okinawa Institute of Science and Technology Graduate University*)

Memories are encoded through long-lasting changes in the network of the brain. These memory traces can interfere with each other and therefore lead to instability of the representations. Indeed, a previous study discovered that instability is preferentially embedded within spatial maps in memory engram cells in the hippocampus even though their activity is still functionally linked to memory-relevant behaviors (Tanaka et al., 2018). Notably, higher instability of place cells is often observed in aberrant network states caused by various factors, including aging, stress, or epileptic seizure, which cause memory impairments. These studies highlight two distinct types of instability leading to the opposite outcomes of hippocampal memory. In my talk, I will introduce our unpublished studies aiming at 1) elucidation of neuronal underpinning that survives extreme plasticity yet supports memory and 2) the development of a novel approach to reset the aberrant network state. These studies will provide fundamental insight into physiological and pathological instability in the neuronal network.

### [3S06a-03]

### Social memory representation in the hippocampus

*Teruhiro Okuyama[1] ([1]*Institute for Quantitative Biosciences (IQB), The University of Tokyo*)

For social animals, it is crucial to remember and recognize different conspecific individuals (i.e., having “social memory”) and exhibit appropriate social behaviors, such as preference behavior or avoidance behavior, to each individual. In humans, lesion of the hippocampus leads to multiple memory deficits including social memory, suggesting that the hippocampus, at least in part, stores memory information on the individual as well as other components of episodic memory such as spatial or temporal memory. Since mice naturally tend to spend more time interacting with novel mice rather than familiar mice (social discrimination behavior), we can quantify the degree of memory of individuals by calculating the total duration of time spent with novel versus familiar individuals. Using the social discrimination behavioral assay, we recently demonstrated that vCA1 pyramidal neurons in the hippocampus store social memory (social memory engram). Even if the memory seemed lost after long separation periods, optogenetic activation of the engram can fully restore that social memory. Additionally, an artificial association between social engram encoding the memory of a specific individual with fear or reward events can elicit avoidance from or preference to that individual, respectively. Additionally, one tiny dissonance in social memory can easily disrupt the appropriate social behavior, even for humans. Social impairments caused by a genetic mutation, especially those related to the familiarization with other individuals, are commonly exhibited by patients diagnosed with autism spectrum disorder (ASD). Patients with ASD have difficulty either with social memory itself or showing normal social communication driven by social memory. In this meeting, we will show our recent findings regarding neural mechanisms underlying social familiarization impairments observed in ASD.

### [3S06a-04]

### Hippocampal-cortical network dynamics elucidated with multi-regional in vivo large-scale electrophysiology

*Hiroyuki Miyawaki[1] ([1]*Osaka Metropolitan University*)

Animals choose their behavior by integrating various types of information, which are processed in parallel in distinct brain regions. Furthermore, animals change their behavior based on their previous experience. These indicate that neuronal networks across brain regions change in an experience-dependent manner, however, the details of the changes are largely unknown. To investigate this point, we performed multi-regional large-scale electrophysiological recording from fear-conditioned rats and found that memory-related neuronal ensembles in the prefrontal cortex (PFC) were simultaneously reactivated with those in the ventral hippocampus (vHPC) and basolateral nucleus of the amygdala (BLA) during sleep following the conditioning sessions. Coactivations of PFC-BLA ensemble pairs were also detected during the conditioning and retention sessions. In contrast, virtually no PFC-vHPC coactivations were observed during conditioning sessions, whereas the coactivations re-emerged during retention sessions. These results demonstrate that hippocampal-cortical networks dynamically change after memory acquisition, presumably driving the initial stage of memory consolidation.

### Symposium

[3S07a]

**Molecular and neural circuit mechanisms of eating behavior and metabolic process**

March 16 (Thu.), 14:20 - 16:20, Room 7

### [3S07a-01]

### Neural mechanism of salt and umami seeking behavior

*Takaaki Ozawa[1], Tomohiro Shibata[1], Yoshinobu Oyama[1], Mayuka Abe[1], Kentaro Goto[1], Hinano Yonemaru[1], Yuma Matsumoto[1], Ryotaro Iwamoto[1], Koki Sakurai, Tom Macpherson[1], Takatoshi Hikida[1] ([1]*Osaka University, Institute for Protein Research*)

Salt is not only a vital nutrient for maintaining health but also an essential seasoning for our rich dietary lifestyle. On the other hand, it has been suggested that overconsumption of salt can be a serious risk factor for high blood pressure, a trigger for stroke and heart attack. Human studies have suggested that umami substances enhance the palatability of salty food, and are useful in reducing sodium intake without compromising the palatability of our daily meals. However, the biological mechanism underlying umami's effect has not been investigated. In the present study, we investigated the roles of cortical and subcortical taste circuit and neurotransmitter dopamine, which is known to play an important role in hedonic responses and reward-seeking behavior, in the regulation of salt-seeking and umami's enhancing effect by using behavioral analysis, fluorescent imaging, and neural silencing. In this symposium, current datasets supporting their regulatory roles will be shown. Our results provide new evidence for the effectiveness of umami substances in sustainable salt intake reduction in our dietary lifestyle.

### [3S07a-02]

### Neurosecretory protein GL accelerates feeding behavior and lipid metabolism

*Yuki Narimatsu[1], Masaki Kato[1], Eiko Iwakoshi[1], Megumi Furumitsu[1], Kazuyoshi Ukena[1] ([1]*Laboratory of Neurometabolism, Graduate School of Integrated Sciences for Life, Hiroshima University*)

Hypothalamic dysfunction readily leads to obesity and associated metabolic diseases. Of note, feeding behavior is regulated by a highly complex process in the hypothalamus. We have recently identified a novel gene encoding a small secretory protein, termed neurosecretory protein GL (NPGL) in the avian hypothalamus. A database search revealed that the *Npgl* gene is conserved in vertebrates, including mice and rats. Judging from the robust expression of NPGL in the mediobasal hypothalamus, which is the central regulatory region of energy homeostasis, we predicted that NPGL had a crucial role in feeding behavior. We have employed functional analysis of NPGL in birds, rats, and mice. In birds, intracerebroventricular infusion of NPGL stimulated food intake and fat accumulation. Analysis using mammals demonstrated that the mRNA expression of *Npgl* was strictly regulated by energy status in individuals. Furthermore, overexpression of *Npgl* elicited feeding behavior and fat accumulation via *de novo* lipogenesis, resulting in obesity. Taken together, NPGL is a novel orexigenic and obesogenic neuropeptide in vertebrates.

### [3S07a-03]

### Cortical and hypothalamic cooperation in the control of appetite

*Ikue Kusumoto-Yoshida[1], Jihao Ma[1], Mira Masumitsu[1], Ran Yamaguchi[1], Tomoyuki Kuwaki[1] ([1]*Department of Physiology Graduate School of Medical and Dental Sciences, Kagoshima University*)

Maintaining good health requires properly controlling food consumption. Understanding the brain regions that underlie aberrant food consumption is crucial since growing obesity, diabetes, and cachexia pose serious health risks. It is generally known that the hypothalamus controls both energy homeostasis and food intake. However, further research is needed to fully understand relationships between other brain regions, such as cortical regions.We have been investigating neuronal circuits downstream of hypothalamic orexin neurons using histology and genetic approach. Increased c-Fos signal-positive neurons were seen in hypothalamic orexin neurons and the insular cortex in a study of restricted feeding-induced c-Fos mapping. Similar to the earlier study, mice in the limited feeding paradigm displayed elevated locomotor activity soon before feeding time that resembled meal anticipation. The insular cortex, a higher-order sensory cortex, integrates various modalities and is crucial for maintaining homeostasis in the body. These findings imply a critical function for coordinated insular and orexin neuron activity in anticipation of eating. Food intake and c-Fos expression increased in the insular cortex after activating orexin neurons, showing that the insular cortex is crucial for orexin-regulated food intake.

### [3S07a-04]

### Ingestive behaviors in mouse offspring are affected by maternal dietary balance of polyunsaturated fatty acids

*Nobuyuki Sakayori[1] ([1]*Hiroshima Univ.*)

Most animals cannot synthesize omega-6 (n-6) or omega-3 (n-3) polyunsaturated fatty acids (PUFAs). In the brain, these PUFAs work as essential structural components of the cellular membrane and also serve as precursors for bioactive lipid metabolites. Importantly, these PUFAs are generally competitive in various metabolic processes, and thus the n-6/n-3 ratio in the brain warrants particular attention. Focusing on recent nutritional trends leading to foods that are rich in n-6 PUFAs and poor in n-3 PUFAs, we found that lifelong exposure to a diet high in n-6 PUFAs and low in n-3 PUFAs (n-6 high/n-3 low) increased the brain n-6/n-3 ratio and the intake of sucrose and fat, despite no difference in the intake of water or standard diets. In vivo microdialysis and histological experiments showed that dopamine release in the nucleus accumbens and the number of dopaminergic neurons in the ventral tegmental area were increased in the offspring fed the n-6 high/n-3 low diet. Neurodevelopmental and lipidomics analyses further revealed that this induced hedonic consumption was driven by maternal diet, as dopaminergic neurogenesis and several n-6 PUFA-containing lipids in the embryonic brain were increased during in utero access to the n-6 high/n-3 low diet. Our findings reveal that maternal consumption of PUFAs can have long-lasting effects on offspring's ingestive behaviors for highly palatable foods.

### Symposium

[3S08a]
**The basal ganglia -beyond motor control functions-**

March 16 (Thu.), 14:20 - 16:20, Room 8

### [3S08a-01]

### The cortex-basal ganglia loop and its role in procedural learning and decision processes in vertebrates.

*Thomas Boraud[1,2] ([1]*CNRS*, [2]*University of Bordeaux*)

The cortex of mammals makes a loop circuit with the basal ganglia and the thalamus known to control and adapt behaviour but the who's who of the functional roles of these structures and their dynamic properties are still debated. We reevaluated the function of the network in light of the current experimental evidence concerning the comparative anatomy and physiology of the basal ganglia-cortical circuits in vertebrates and how oscillation regimes can be generated in the network. We propose here a minimal computational framework, which predicts that early vertebrates that are deprived of a proper cortex should be able to perform associative learning but could not automatize. We validated this hypothesis in Pleurodeles Waltl, rats and primates. We showed that pallium/cortex is not necessary for a dopaminergic dependent operant learning but is important to develop proper habits. It also has implications concerning the condition in which oscillations can be generated and the role of the different cortico-sub cortical pathways in the generation of physiological and pathological oscillations such as the one that appears in Parkinson's Disease.

### [3S08a-02]

### Subthalamic nucleus modulates the stability of movements and neural activity in the basal ganglia

*Taku Hasegawa[1,2], Satomi Chiken[2,3], Kenta Kobayashi[3,4], Atsushi Nambu[2,3,4] ([1]*RIKEN Center for Brain Science*, [2]*Department of Physiological Sciences, National Institute for Physiological Sciences*, [3]*Department of Physiological Sciences, SOKENDAI*, [4]*Section of Viral Vector Development, National Institute for Physiological Sciences*)

The subthalamic nucleus (STN) is a small nucleus in the basal ganglia (BG) but plays an indispensable role in voluntary movements. The STN receives inputs from the cortex and the external pallidum (GPe) and projects to the internal pallidum (GPi) and the GPe, constituting the cortico-STN-GPi hyperdirect and cortico-striato-GPe-STN-GPi indirect pathways. Although the STN is considered to suppress movements, its neural mechanism has remained elusive. Here, we reversibly suppressed the neural activity of the STN of three macaque monkeys (*Macaca fuscata*) using DREADD (Designer Receptors Exclusively Activated by Designer Drugs) and induced involuntary movements and unstable reaching movements on their contralateral upper limbs. Single unit recordings in the STN, GPe, and GPi showed that the STN suppression increased spike train variability. Across-trial analyses revealed that the increased variability in neural activity is correlated with the abnormal movements. Our results suggest that the STN modulates the stability of the BG output and adjusts the movement variability, possibly related to exploration in reinforcement learning.

### [3S08a-03]

### Tic disorders -disruption of neuronal processing in the cortico-basal ganglia motor and limbic loops-

*Yoshihisa Tachibana[1] ([1]*Department of Physiology and Cell Biology, Kobe University Graduate School of Medicine*)

Tourette syndrome (TS) is a neurodevelopmental and neuropsychiatric disorder characterized by chronic multiple motor and vocal tics. We have recently reported that motor and vocal tics in TS patients are ameliorated by a removable oral splint. Then, our question is why the oral splint is effective for improving tic symptoms. To answer this question, we investigated the central pathway of masticatory muscle spindles, which are activated by the insertion of oral splint. A series of tracer injection studies in rats revealed that the proprioceptive inputs from orofacial muscle spindles are finally conveyed to the dorsal part of granular insular cortex. Interestingly, human imaging studies have reported the abnormal activity in the striatum and insula in TS patients. On the other hand, the disinhibition of the striatum by local injection of GABA antagonist bicuculline induced tic-like symptoms in rodents. In the tic mouse model, the c-Fos immunohistochemistry revealed the activation of the insula. Viral tracing studies revealed that the outputs from the globus pallidus and the substantia nigra originating from the striatal motor region are conveyed to the insula via the intralaminar thalamic nuclei. Further, inactivation of the insula by means of DREADD system ameliorated motor symptoms in the tic mouse model. These data suggest that the motor tic may be generated by abnormal information processing in the motor (striatum)-limbic (insula) network.

### [3S08a-04]

### Striosome; from the birth to death in Huntington's disease

*Ayano Matsushima[1,2], Sergio Sebastian Pineda[3,4,5,6], Jill R. Crittenden[1,2], Hyeseung Lee[3,4], Kyriakitsa Galani[4,6], Julio Mantero[4,6], Manolis Kellis[4,5,6], Myriam Heiman[2,3,4], Ann M. Graybiel[1,2] ([1]*McGovern Institute for Brain Research, MIT*, [2]*Department of Brain and Cognitive Sciences, MIT*, [3]*Picower Institute for Learning and Memory, MIT*, [4]*Broad Institute of MIT and Harvard*, [5]*Department of Electrical Engineering and Computer Science, MIT*, [6]*MIT Computer Science and Artificial Intelligence Laboratory*)

The striosomes exist as 3-D labyrinths winding through the matrix, which has differential developmental origin and vulnerabilities in diseases. Striatal projection neurons (SPNs) in striosomes are born earlier (E11-14 in mice) than those in the matrix (E14 -P1 in mice). To capture SPNs born in close succession, we administered fast-acting 4-Hydroxytamoxifen (4-OHT) to CreER driver mice. This allows us to see fine developmental programs. First, SPNs at the center of striosome born earlier than those in the periphery of the same striosome. Second, E12- and E13-born, but not E11- nor E14-born, SPNs project to dopaminergic cells participating the so- called "striosome-dendron bouquets". In Huntington's disease, preferential disturbance of D2R-expressing SPNs, as compared to D1R-expressing SPNs, is well-documented. However, how this organizational axis relates to the striosome-matrix axis of SPN organization has not been clearly understood. To address this issue, we identified with snRNA-sequencing striosome and matrix subclusters within D1 and D2 parent clusters in human and mouse. In early grade human HD, striosomal SPNs were the most depleted SPN population. Surprisingly, in mouse models of HD, the transcriptomic distinctiveness of striosome-matrix SPNs was diminished more than that of D1-D2 SPNs. Thus, the canonical D1-D2 and striosome-matrix organizations of the striatum are differentially compromised in HD.

### Symposium

### [3S09a]
### Divergent roles of mitochondria: regulation of excitability, cell death/survival, metabolism and beyond

March 16 (Thu.), 14:20 - 16:20, Room 9

### [3S09a-01]

### Impact of mitochondrial dynamics on functional maturation in cardiomyocytes

*Takaya Ishihara[1], Naotada Ishihara[1] ([1]*Dept. of Biol. Sci, Grad. Sch. of Sci., Osaka Univ.*)

Mitochondria are double-membraned organelles derived from bacterial symbioses that serve essential functions not only in energy production but also in differentiation, development and diseases. Mitochondria are also known to frequently repeat fission and fusion within the cell which is defined as "mitochondrial dynamics," and this morphological change is important for maintaining these cellular functions. We have analyzed the physiological roles in various tissue-specific Drp1 KO mice deficient in mitochondrial fission. From these analyses, we have found that mitochondrial fission is essential for different functions. One of them is proper cardiac functioning. Furthermore, we found that mtDNA which encodes genes essential for the maintenance of mitochondrial activity, is heterogeneously distributed in Drp1 KO cardiomyocytes, suggesting that its dynamics also may play a role in the maintenance of cardiac function. Thus, the analysis of the mitochondrial fission factor Drp1 enabled us to understand the importance of the cooperative dynamic behavior between the mitochondrial membrane and mtDNA. Now, we are discovering various mitochondrial properties through screening of factors regulating mitochondrial dynamics. In the future, we would like to elucidate the molecular mechanisms and their relationship to physiological functions by analyzing of related factors.

### [3S09a-02]

### Regulation of mitochondrial quality and cardiac homeostasis by supersulfides

*Akiyuki Nishimura[1], Kakeru Shimoda[2], Tang Xiakang[1], Kazuhiro Nishiyama[2], Yuri Kato[2], Motohiro Nishida[2,1] ([1]*Div. Cardiocirc. Signal., NIPS,* [2]*Grad. Sch. Pharmaceut. Sci., Kyushu Univ.*)

Proper mitochondrial quality control is important for cardiac homeostasis and its defect is implicated in the development of cardiac diseases. Our group has investigated the molecular mechanism underlying the development of maladaptive cardiac remodeling, especially myocardial senescence, and found that mitochondrial hyperfission induced by aberrant activation of Dynamin-related protein 1 (Drp1), a mitochondrial fission-accelerating protein, is a key determinant of cardiac remodeling and fragility. Supersulfides have been recently recognized as a key molecule to regulate redox homeostasis and are abundantly discovered in both prokaryotes and eukaryotes. We found that Drp1 activity is negatively regulated by supersulfide-mediated polysulfidation of Drp1 at $Cys^{644}$. Ischemic stress induced by myocardial infarction converted supersulfides into hydrogen sulfide, and reduced supersulfides promoted Drp1 hyperactivation via depolysulfidation of $Cys^{644}$, causing myocardial senescence and cardiac fragility. Exposure of cardiomyocytes to environmental electrophiles such as methylmercury also induced supersulfide depletion and triggered mitochondrial hyperfission-associated myocardial senescence.

### [3S09a-03]

### Distinct characteristics of mitochondrial $Ca^{2+}$ dynamics in mouse heart and brain

*Ayako Takeuchi[1,2], Satoshi Matsuoka[1,2] ([1]*Department of Integrative and Systems Physiology, Faculty of Medical Sciences, University of Fukui,* [2]*Life Science Innovation Center, University of Fukui*)

Mitochondrial $Ca^{2+}$ is a key factor regulating ATP synthesis, cytosolic $Ca^{2+}$ signaling, cell death, and so on. It is not static, but changes dynamically according to the dynamical changes of intra- and extra-mitochondrial circumstances. In excitable tissues such as the heart and the brain, it is well understood that the mitochondrial $Ca^{2+}$ influx is mainly mediated via the $Ca^{2+}$ uniporter complex, and the efflux via the $Na^+$-$Ca^{2+}$ exchange ($NCX_{mit}$) and the $H^+$-$Ca^{2+}$ exchange, with the $NCX_{mit}$ being dominant. In the present study, we analyzed the distinct characteristics of mitochondrial $Ca^{2+}$ dynamics in the heart and the brain, especially focusing on the $NCX_{mit}$. In both heart and brain mitochondria, the cytosolic $Na^+$-dependent $Ca^{2+}$ efflux from mitochondria, i.e., the forward mode of the $NCX_{mit}$, comprised the major component of the mitochondrial $Ca^{2+}$ efflux. The $Na^+$ was substitutable with $Li^+$ and the activity was sensitive to a classic $NCX_{mit}$ blocker CGP-37157. On the other hand, the mitochondrial $Na^+$-dependent $Ca^{2+}$ influx into mitochondria, i.e., the reverse mode of the $NCX_{mit}$, was much faster in the brain than in the heart. This may contribute to the vulnerability to mitochondrial $Ca^{2+}$ overload in the brain.

### [3S09a-04]

### Regulation of mitochondrial function by an intracellular $Ca^{2+}$ sensor is critical for myocardial stress tolerance and systemic energy metabolism.

*Tomoe Y Nakamura-Nishitani[1] ([1]*Wakayama Medical Univ.*)

Intracellular $Ca^{2+}$ is crucial for determining cell viability and is also implicated in the obesity regulation. Mitochondria play important roles in both cell survival and metabolism, however, the mechanisms linking all these factors including $Ca^{2+}$ signaling are unknown. Here, we found that a $Ca^{2+}$ sensor NCS-1 contributes to myocardial stress tolerance and energy metabolism through regulation of mitochondrial functions. Cardiomyocytes isolated from NCS-1 KO mice exhibited vulnerable to various stressors. This was due to reduced $Ca^{2+}$-dependent mitochondrial biosynthesis, resulting in impaired ATP synthesis and detoxification mechanisms. Furthermore NCS-1 KO mice become obese with age. Individual-level analysis revealed that energy metabolism was reduced in KO mice, while food intake and locomotion remained the same. NCS-1 was actually expressed in both brown and white adipose tissues (BAT and WAT). Metabolomic analysis revealed that metabolites involved in energy metabolism were decreased in BAT and metabolites that promote fat accumulation were increased in WAT in KO mice. Taken together, these data suggest a novel NCS-1-mediated regulation of mitochondrial function that contributes to myocardial stress tolerance and energy metabolism.

### [3S09a-05]

### Pathophysiological roles of p13/FMC1, a protein localized in mitochondria.

*Norihito Shintani[1,2] ([1]*Laboratory of Pharmacology, School of Pharmaceutical Sciences, Wakayama Medical University.,* [2]*Laboratory of Molocular Neuropharmacology, Guraduate School of Pharmaceutical Sciences, Osaka University.*)

Mitochondrial dysfunction has recently attracted attention as a molecular pathogenesis of chronic inflammatory diseases such as diabetes and neurodegenerative diseases. Here I show the recent results of p13/FMC1, which I was identified as a novel functional protein localized in mitochondria. Through the transcriptome analysis focusing on the pancreatic remodeling, many mitochondrial proteins including p13 was shown to decreased in pancreatic islets under type II diabetes. The studies using p13 transgenic animals revealed the possibly beneficial roles of p13 in the diabetes-related pancreatic islet remodeling. Interestingly, comprehensive phenotypic analyses of its knockout mice disclosed that p13 is involved in the formation of various central and peripheral organs, not just in the pancreas. The possible mechanism underlying the p13 action is also discussed.

### Symposium

[3S10a]
Cooperation with Other Societies Committee
**Physiological research as a basis for rehabilitation (physical therapy)**

March 16 (Thu.), 14:20 - 16:20, Room 10

### [3S10a-01]

### Maximize therapeutic effect on damaged brain via Kampo medicine on rehabilitative training

*Naoki Tajiri[1], Shinya Ueno[1], Takeshi Shimizu[1], Dewi Mustika[1], Eisuke Haneda[2], Keita Mizuno[2], Hideki Hida[1] (*[1]Department of Neurophysiology & Brain Science, Graduate School of Medical Sciences & Medical School, Nagoya City University, [2]Tsumura Kampo Research Laboratories, Tsumura and Co*)

Exercise ameliorates physical and cognitive impairment of patients with neurological diseases, by enhancing brain plasticity, including increased neurogenesis and angiogenesis, as major mechanism of action. Key to neuroplasticity is brain remodeling towards recapitulation of a neurodevelopmental microenvironment conducive to synapse formation. Rehabilitative training of forced limb use (FLU) after internal capsule hemorrhage (ICH) shows a causal relationship between the cortico-rubral tract and the functional recovery. As the interests of Kampo medicine on rehabilitative training are recently increasing, we focused on Ninjin'yoeito (NYT) that has various effects on the muscle (sarcopenia) and the brain (cognitive dysfunction). We first investigate whether the combination of NYT and FLU can promote motor function after ICH, and then tried to know the mechanism of NYT in rehabilitative training. ICH model rat was made by the injection of Type IV collagenase (15 units/ml, 1.4μl) into the left internal capsule of male rats. FLU was given from 1 day after the region (D1) for 7 days with the oral administration of 1% NYT until D56. Motor deficit score and horizontal ladder tests were used for behavioral assessment at D28. Motor activities were assessed by the open field test using Smart software. We found that FLU + NYT group showed significantly better functional recovery in both tests, showing that the effect of NYT administration is additive to the effect of rehabilitative training. This work was a breakthrough in the field, highlighting the effects of rehabilitation on ICH and in the repair of central nervous system.

### [3S10a-02]

### The Role of Physical Therapy in the Prevention and Improvement of Atherosclerosis and its Mechanisms

*Erika Iwamoto[1] (*[1]Sapporo Medical University, School of Health Sciences*)

Physical therapists are involved in cardiovascular and cerebrovascular disease treatments, and our target patients have a high prevalence of atherosclerosis. Endothelial dysfunction is an early marker for atherosclerosis. Importantly, endothelial dysfunction can also occur in patients with respiratory disease and diabetes or healthy adults with postprandial hyperglycemia. Physical therapy involves aerobic and resistance exercises, electrical stimulation, heating, etc., and these interventions can improve endothelial function through different mechanisms. Therefore, understanding the physiological mechanisms is crucial for developing effective intervention programs. Aerobic exercises and heating are well-known methods to improve endothelial function in the peripheral arteries. Therefore, we have recently examined the mechanism underlying aerobic exercise to prevent hyperglycemia-induced endothelial dysfunction in peripheral arteries. In contrast, since atherosclerosis also occurs in cerebral arteries, we evaluated the effects of age, sympathetic activation, and menopause on cerebrovascular function, as well as potential effective interventions (e.g., aerobic and resistance exercises, and intermittent hypoxic stimulation) to improve cerebrovascular function. This symposium will discuss our findings and ongoing studies.

### [3S10a-03]

### Motor training promotes both synaptic and intrinsic plasticity of layer V pyramidal neurons in the primary motor cortex

*Dai Mitsushima[1] (*[1]Department of Physiology, Yamaguchi University Graduate School of Medicine*)

Layer V neurons in primary motor cortex (M1) are important for motor skill learning. Since pretreatment of either CNQX or APV in rats M1 layer V impaired rotor rod learning, we analyzed training-induced synaptic plasticity by whole-cell patch-clamp technique in acute brain slices. One-day trained rats showed a decrease in small inhibitory postsynaptic current (mIPSC) frequency and an increase in the paired-pulse ratio of evoked IPSCs, suggesting a transient decrease in presynaptic GABA release in early phase. Two-days trained rats showed an increase in miniature excitatory postsynaptic current (mEPSC) amplitudes/frequency and elevated AMPA/NMDA ratios, suggesting a long-term strengthening of AMPA receptor-mediated excitatory synapses. Importantly, rotor rod performance in trained rats was correlated with the mean mEPSC amplitude and the frequency obtained from that animal. In current-clamp analysis, 1-day-trained rats showed an increase in action potential threshold and a decrease in firing rate, while 2-day-trained rats returned to pre-training levels, suggesting a dynamic changes in intrinsic properties. Furthermore, western-blot analysis of layer V detected decreased phosphorylation of $Ser^{408-409}$ in $GABA_A$ receptor $\beta_3$ subunits in 1-day trained rats, and increased phosphorylation of $Ser^{831}$ in AMPA receptor GluA1 subunits in 2-days trained rats. Finally, live-imaging analysis of Thy1-YFP transgenic mice showed that the training rapidly recruited a substantial number of spines for long-term plasticity in M1 layer V neurons. Taken together, these results indicate that motor training induces complex and diverse plasticity in M1 layer V pyramidal neurons.

### [3S10a-04]

### New mechanisms of muscular pain

*Kazue Mizumura[1,2] (*[1]Department of Physiology, Nihon University School of Dentistry, [2]Nagoya University*)

We have been analyzing the mechanism of delayed onset muscle soreness (DOMS) as a model for myofascial pain syndrome, and have reported two pathways, B2 bradykinin receptor-NGF pathway and COX-2 -GDNF pathway, are implicated. We have recently found these two pathways interact synergistically at the primary afferents (DRG neurons) in rats, and that NGF receptor TrkA and GDNF receptor GFRa1, which are reported to be expressed in different groups of DRG neurons, are co-expressed in about 8-15 % of small to medium sized putative nociceptive DRG neurons. In another muscle pain model, repeated cold stress (RCS) model, a model for fibromyalgia, we have found muscle pH decreases, and long-lasting muscular mechanical hyperalgesia exists. Both the decrease in muscle pH and muscular mechanical hyperalgesia are reversed by administration of bafilomycin, an antagonist against vacuolar(V)-ATPase. Thus, the mechanical hyperalgesia after RCS is considered to be induced by pH decrease through activation of V-ATPase. These two new mechanisms for muscular mechanical hyperalgesia may also work in other muscular painful conditions. No COI.

### Symposium

### [3AS01e]
### Innovative Brain Research - Toward a comprehensive understanding of the neural circuits underlying higher brain functions

March 16 (Thu.), 16:30 - 18:30, Room 1

### [3AS01e-01]

### Bi-directional translational approach from the findings in human neuroimaging studies for schizophrenia

*Shinsuke Koike[1,2] ([1]*UTokyo Institute for Diversity & Adaptation of Human Mind (UTIDAHM)*, [2]*Center for Evolutionary Cognitive Sciences, Department of Arts and Sciences, The University of Tokyo*)

Schizophrenia is a syndrome which characterizes a set of severe psychological symptoms such as persecutory delusion and auditory verbal hallucination. Around 1% of general population receive diagnosis of schizophrenia and encounter their onset during adolescence and young adulthood. Human brain magnetic resonance imaging (MRI) studies for schizophrenia have indicated structural abnormalities in the auditory-related areas such as the superior temporal gyrus and inferior frontal gyrus, as well as in the subcortical areas such as the globus pallidus. However, there has been no definitive neural basis founded from both clinical and basic researches. Here, we want to talk about the human brain MRI findings for schizophrenia from the clinical multi-site studies and adolescent development from Tokyo TEEN Cohort (TTC), and discuss about the brain change in schizophrenia along with adolescent development. Then, we intend to discuss that human brain MRI could have the possibility of translatable brain markers between human, common marmoset, and rodent for the development during adolescence, progression of schizophrenia-related brain pathology, and their interactions.

### [3AS01e-02]

### Mapping axonal projections of the marmoset prefrontal cortex

*Akiya Watakabe[1] ([1]*RIKEN CBS*)

The expansion of the prefrontal cortex (PFC) is a hallmark of primate brain evolution. Whereas the increase in the number of neurons leads to the formation of more complex neural circuits, the burden of wiring costs becomes exponentially large. What would be the neural basis for efficient information processing for an enlarged brain? To explore the connectional architecture of primate brains, we performed a connectomic mapping of axonal projections for the marmoset PFC. Adaptation of Serial Two-Photon Tomography imaging technique allowed accurate and high-resolution volume imaging, which enabled a quantitative description of PFC projections in unprecedented detail. Here we show that PFC projections consist of many arrays of columnar patches, dispersed in a diffuse spread of low-density axonal extensions. Interestingly, the co-application of retrograde and anterograde tracers revealed tight reciprocal connections, raising the possibility of mosaic-like architecture. These columnar patches were enriched in the dorsolateral PFC, suggesting area-specific differences in columnar projections. We also found that the topographic rules govern the distribution of the columnar and diffuse projections in the extra-frontal association areas. Functionally, columnar and diffuse projections involve different populations of neurons with differential effects. The columnar projections are expected to convey highly efficient neurotransmission between restricted neural populations, while the diffuse projections would be effective only when the neural activities of a large number of neurons are synchronized. The refinement of the population connectivity architecture may have been an important factor for efficient information processing in the primate brain.

### [3AS01e-03]

### Fast and wide field-of-view two photon microscope to understand the brain's large-scale network dynamics

*Masanori Murayama[1] ([1]*RIKEN Center for Brain Science*)

Fast and wide field-of-view imaging with single-cell resolution, high signal-to-noise ratio, and no optical aberrations can inspire new avenues of investigation in biology. Here, we combine a resonant scanning system, a large objective with low magnification and high numerical aperture, and highly sensitive large-aperture photodetectors. The result is a practically aberration-free, fast-scanning high optical invariant two-photon microscopy (FASHIO-2PM) that enables calcium imaging from a large network composed of ~16,000 neurons at 7.5 Hz from a 3 mm x 3 mm contiguous image plane. Network analysis based on single-cell activities revealed that the brain exhibits small-world rather than scale-free behavior. I will introduce recent results from network analysis and a new wide-FOV microscope that allows us to explore causal relationships between brain functions and network dynamics.

### [3AS01e-04]

### Marmoset database provides next-generation neuroscience research

*Tomomi Shimogori[1] ([1]*RIKEN Center for Brain Science*)

The Marmoset Gene Expression Atlas (MGA) has published more than 2,000 gene expression patterns since 2016, focusing on genes involved in psychiatric mental and neurological disorders in the marmoset brain. The database has a multifaceted search function, including a search function for genes expressed in specific brain regions. By making full use of these functions, gene expression patterns that could not be clarified before can be revealed. As an example, genes related to psychiatric disorders mental and neurological disorders were found to be expressed in clusters in specific brain regions and neurons. In addition, by linking databases, it is possible to compare the expression patterns of marmoset, mouse, and human brains. As a result, many common expression patterns were found in marmosets and humans. Using these databases and their functions, we will propose new neuroscience research using large-scale data.

### [3AS01e-05]

### Data-driven framework for integrating the brain waves and connectivity

*Ken Nakae[1] ([1]*National Institutes of Natural Sciences*)

Epilepsy is a disease that causes recurrent unprovoked seizures, in which a brain waves with a high frequency propagate through the brain. The spatial mechanism of the propagation is thought to depend on the brain connectivity. However, the quantitative relation between the brain waves and connectivity in the epileptic seizure remains unclear. For understanding the relation, we develop a data assimilation approach for predicting the brain waves of ECoG data from the brain connectivity of diffusion MRI. We applied the proposed method to two marmosets with epilepsy, in which the brain wave is originating from the temporal region, and the brain connectivity around the region are different from normal marmosets. We found that the prediction accuracy in the temporal-occipital regions of ECoG data was lower than the other regions, which implies a network anormal in the epileptic seizure. This framework will contribute to understand the mechanism of the epilepsy and a better prediction for epileptic seizure.

### Symposium

[3S02e]
Cooperation with Other Societies Committee
**State-of-the-art in stress research**

March 16 (Thu.), 16:30 - 18:30, Room 2

### [3S02e-01]

### Subpopulations of medullary tyrosine hydroxylase-expressing neurons and their roles in stress responses

*Masahide Yoshida[1], Anir Khurelbaatar[1], Tatsushi Onaka[1] ([1]*Division of Brain and Neurophysiology, Department of Physiology, Jichi Medical University*)

The nucleus tractus solitarii (NTS) in the medulla oblongata receives direct inputs from the vagus nerve, spinal cord and peripheral hormones as well as from several brain areas. The NTS responds to signals of stress and metabolic status. Stress-related diseases are known to increase the risk of developing metabolic dysregulations and vise versa. Thus, stress responses and energy metabolism share a common neural pathway possibly via the NTS. We have focused on tyrosine hydroxylase (TH)-positive noradrenergic neurons in the NTS. β-Klotho, a co-receptor of FGF21, is expressed locally in the NTS. FGF21 has been reported to increase energy expenditure. We found that β-Klotho was expressed in TH neurons in the NTS. FGF21 injection or social defeat stress (SDS) activated TH neurons. FGF21-deficient mice enhanced depressive behavior after chronic SDS. In the NTS, prolactin-releasing peptide (PrRP) is expressed specifically in TH neurons. We reported that endogenous PrRP suppresses feeding. Feeding, conditioned fear or SDS activated PrRP/TH neurons. PrRP-deficient mice increased freezing behavior after conditioned fear and enhanced depressive behavior after chronic SDS. Histological analysis suggested that β-Klotho and PrRP are expressed in different subpopulations of TH neurons. It is possible that stress responses and energy metabolism form a common circuit via the TH neurons in the NTS.

### [3S02e-02]

### Hypothalamic control of defensive responses to potential threats -possible relation to human psychiatric disorder-

*Noriko Horii[1], Mayumi Nishi[1] ([1]*Nara Medical Unviersity*)

Defensive behaviors are a set of evolved responses to threats. Threats can be categorized as either acute or potential. An acute threat is imminent and is an obvious risk to safety. Animals perceiving an acute threat (e.g., predators, intruders) exhibit defensive responses including fight, flight, and freezing. In contrast, a potential threat has ambiguous and uncertain risks. Animals encountering a potential threat, such as a novel object, exhibit risk assessment/checking behavior. While a number of studies have investigated the neural mechanisms underlying defensive responses to acute threats, that to potential threats remains unclear. Here, we will present evidence that hypothalamic perifornical urocortin-3 neurons are involved in the modulation of defensive responses to potential threats in mice and that mice whose perifornical urocortin-3 neurons are activated can serve as a model of obsessive-compulsive disorder. Our results indicated that the hypothalamic defensive system against potential threats could associate with the pathophysiology of obsessive-compulsive disorder.

### [3S02e-03]

### Psychosomatic correlation mechanism driving psychological stress responses

*Naoya Kataoka[1,2], Kazuhiro Nakamura[1] ([1]*Department of Integrative Physiology, Nagoya University Graduate School of Medicine, Nagoya, Japan,* [2]*Nagoya University Institute for Advanced Research, Japan*)

The brain has the neural circuits of “mind” that process psychological stress and emotion and the neural circuits that regulate “body” states, such as body temperature and heart rate. Close association of these brain circuits causes a phenomenon called “psychosomatic correlation”. We recently found that the dorsomedial hypothalamus (DMH) receives glutamatergic stress signals from the medial prefrontal cortex, which processes stress and emotion. We have proposed that this neural pathway connects the “mind” and “body” brain circuits to achieve the psychosomatic correlation, including sympathetic responses to psychological stress. However, the “mind” neural circuits involved in psychosomatic correlation have been poorly understood. To investigate the correlation between neural activities in the “mind” neural circuitry and activities of peripheral effector organs, we are simultaneously monitoring neural activities in six brain regions including the limbic system by fiber photometry and vital parameters by telemetry in mice under psychological stress. We will present our approach to elucidate functional “mind” circuit by analyzing synchronization of neural activity patterns among the brain regions with sympathetic effector responses.

### [3S02e-04]

### Detection of malnutrition and the maintenance of energy homeostasis by the hindbrain

*Fuko Matsuda[1], Marimo Sato[1] ([1]*Graduate School of Agricultural and Life Sciences, The University of Tokyo*)

Hypoglycemia, major metabolic stress, is monitored by the brain, which system is essential for the maintenance of energy homeostasis. Administration of 2-deoxy-D-glucose (2DG), a glucose utilization inhibitor, to rats induces glucoprivation and therefore have been used as an animal model of malnutrition. An infusion of 2DG into the fourth ventricle (4V) causes counterregulatory responses against hypoglycemia in rats, suggesting that hindbrain is involved in glucose sensing. To clarify the hindbrain glucose sensor-hypothalamic neural pathway, we administered 2DG into 4V of rats and morphologically analyzed the expression of c-Fos, a marker for cellular activation, in the brain. As a result, it was suggested that lowered glucose availability, sensed by 4V ependymal cells, activates hindbrain catecholaminergic neurons followed by corticotropin-releasing hormone neurons in the hypothalamic paraventricular nucleus and neuropeptide Y (NPY) neurons in the arcuate nucleus, thereby leading to counterregulatory responses against hypoglycemia. To further confirm the role of 4V ependymal cells as a glucose sensor, we generated rats whose 4V ependymal cells were denuded and analyzed the effect of hindbrain glucoprivation on their counterregulatory responses. 4V 2DG infusion failed to increase the food intake of rats with ependymal denudation, suggesting that the 4V ependymal cells function as a key glucose sensor to regulate feeding during malnutrition. Taken together, the mechanism detecting malnutrition and maintaining energy homeostasis by the hindbrain was clarified using 4V 2DG-infused rat model.

### [3S02e-05]

### Principle of the sensory medicine: inducing the artificial hibernation/life-protective state using innate fear odors

*Reiko Kobayakawa[1], Ko Kobayakawa[1] ([1]*Kansai Medical University*)

Innate fear intimately connects to the life preservation in crises, although their relationships are not fully understood. We have developed thiazoline-related innate fear odors (tFOs), and found that they orchestrate hypothermia, hypometabolism, and anti-hypoxia, which enable survival in lethal hypoxic conditions via activation of trigeminal/vagal TRPA1. These responses exerted potent therapeutic effects in cutaneous and cerebral ischemia/reperfusion injury models. TFO presentation activates the NTS-Parabrachial nucleus pathway to induce hypothermia and hypometabolism; this activation was suppressed in Trpa1 knockout mice. However, Importantly, TRPA1 activation is insufficient to trigger tFO-mediated anti-hypoxic effects; Sp5/NTS activation is also necessary. According to this rule, we find a novel molecule that enables mice to survive in a lethal hypoxic condition ten times longer than known tFOs. Combinations of appropriate tFOs and TRPA1 command intrinsic physiological responses relevant to survival fate. If this system is preserved in humans, it may be utilized to give rise to a new field: "sensory medicine."

### Symposium

[3S03e]
Committee for Editorial Board of the Journal of Physiological Sciences
**Discuss how to publish our physiology research**

March 16 (Thu.), 16:30 - 18:30, Room 3

### [3S03e-01]
### Current status of the Journal of physiological sciences (JPS)

*Motohiko Sato[1] ([1]*Dpt. of Physiology, Aichi Medical University*)

The Journal of physiological sciences (JPS) is the official journal of Japanese Physiological Society. JPS was first issued in 1950, since then, JPS has been contributing to the development of research in the physiological fields. JPS covers over all fields of physiology including adaptation and environment, autonomic nervous function, biophysics, cell sensors and signaling, central nervous system and brain sciences, endocrinology and metabolism, excitable membranes and neural cell physiology, exercise physiology, gastrointestinal and kidney physiology, heart and circulatory physiology, molecular and cellular physiology, muscle physiology, physiome/systems biology, respiration physiology and senses. In 2018, all of JPS articles were published online, and 2 years later, JPS restarted as an open access journal. JPS published 39 papers submitted from 11 countries in 2021. In this section, the current status of JPS will be reported including the acceptance rate, citations and impact factor etc. Standard reviewing process and tips for smooth entry to reviewing process will also be reviewed.

### [3S03e-02]
### Excellence and Integrity in Publishing Physiology

*Sharona E Gordon[1] ([1]*University of Washington*)

The “publish or perish” imperative has never been more intense than in the last decade. Our choices in publishing venues are greater than ever, with a near-exponential expansion in the number of journals  and the introduction of preprint servers into biomedical research. Prestige journals, such as eLife, and community-based programs, such as Sciety, are driving large-scale changes in the publishing landscape. This talk with discuss how to navigate many of the recent changes in scientific publishing with an emphasis on preserving excellence and integrity for authors, reviewers, and readers.

### [3S03e-03]
### Science Colab: empowering scientific communities to publish, review and curate their research

*Lesley Anson[1,2], Marie Sweet[1,3], Kenton Swartz[1,4] ([1]*Science Colab,* [2]*Anson Scientific,* [3]*NYU Langone,* [4]*NINDS, NIH*)

Research articles are submitted to scholarly journals to enable peer evaluation, public endorsement, and wide communication. By authoring peer-reviewed publications, researchers gain recognition for their work, particularly when published in high-impact journals. However, these opportunities aren't inclusive, equitable, or transparent. Traditional journal publishing is dominated by a small number of highly selective journals that charge large fees to generate profit. Moreover, binary publish/reject decisions are often arbitrary and opaque, and associated with an adversarial and judgemental peer review process. We are leveraging the increasing popularity of preprints to provide an alternative publishing experience. In our model, authors publish their work as a preprint so that it can be openly evaluated by the community and curated as a version of record. Our first endeavour, Biophysics Colab, provides collaborative, collegiate and constructive peer review of biophysical preprints, aiming to improve the rigour of studies within a transparent, journal-agnostic framework. We publicly endorse studies that address a research question in a rigorous way, and will soon begin to curate such studies as versions of record that will be indexed in PubMed. In the future, our not-for-profit business model will compensate reviewers and curators for their time and expertise by levying a modest fee for peer review and curation. By encouraging others to adopt this model, we envisage a future in which research is fairly evaluated, openly communicated and its significance recognised independently of publication venue.

### Symposium

[3AS04e]
Committee for 100th anniversary / Education Committee
**Physiology Education - present, past, and future-**

March 16 (Thu.), 16:30 - 18:30, Room 4

### [3AS04e-01]

### The achievement of the Physiology Society of Japan in Physiology education

*Noriyuki Koibuchi[1], Akira Nakashima[2], Michio Shiibashi[3], Minamisawa Susumu[4]
([1]*Department of Integrative Physiology, Gunma University Graduate School of Medicine,* [2]*Department of Physiological Chemistry, School of Medicine, Fujita Health University,* [3]*Information Technology Center, Saitama Medical University,* [4]*Department of Cell Physiology, The Jikei University School of Medicine*)

The Physiological Society Japan (PSJ), particularly the Education Committee, has contributed developing Physiology education. Until around 2000, discipline-based education was a gold standard, and the Committee discussed mainly teaching Physiology systematically with keeping identity. Then, various knowledge integration such as Physiology, Anatomy and Biochemistry through active learning became standard in teaching. To keep up such changes, the Committee started Physiology Model Lectures in PSJ annual meeting in 2005. Besides, due to subdivision of research, researchers preferred attending specialized meetings such as Neuroscience and Cardiology rather than PSJ meeting. To share the awareness of the importance of education to reunite PSJ members, Physiology Educator Certification started in 2014 by the effort of the Physiology Educator Certification Committee, together with Education Lecture in the annual PSJ meeting in 2013. This Committee is planning a new certification for those playing a major role in educating Physiology teachers and researchers. The effort of the PSJ to improve Physiology education will nurture human resources in the research and higher education.

### [3AS04e-02]

### What key aspects were changed and unchanged in medical education?

*Yasushi Okamura[1] ([1]*Osaka University*)

The word “Education” in Japanese consists of two Chinese characters. The first character means “teaching”, whereas the second character means “nurturing” or “forstering”. Probably, these two aspects could be translated into two; one is “let students acquire knowledge and skill” as represented well by core curriculum for medical students and the other is “giving high spirits or urging self-motivation”. Recent new digital technology in teaching has changed situations of the former part, whereas much effort and endeavor are still necessary for the latter part as before. In my talk, I will talk about what I think is important currently from my some experiences in medial school.(COI:No)

### [3AS04e-03]

### Molecular Mechanisms and Physiological Significance of Neurogenesis

*Noriko Osumi[1] ([1]*Tohoku University*)

Santiago Ramón y Cajal, a Spanish neuroanatomist who proposed the "neuron theory," and Camillo Golgi, who advocated the opposing "reticular theory," shared the Nobel Prize Physiology or Medicine in 1906. Cajal was an exceptional observer, and his keen sense of observation led him to develop many hypotheses and discussions based on morphological data, some of which were later confirmed at the molecular and cellular levels. Cajal's only error was that he denied neurogenesis, writing, "Once the development ended, … Everything may die, nothing may be regenerated." In the 1960s, however, Joseph Altman in the US discovered newborn neurons in the postnatal rat brain. Initially, neuroscientists dismissed Altman's discovery, but in the 1990s, a link between song learning and neurogenesis in songbirds was proposed, and the role of neurogenesis in memory and learning began to gain attention. Because decreased neurogenesis can be linked to depression and dementia, it is becoming more popular as a drug discovery target. I will discuss the molecular mechanisms and physiological significance of neurogenesis in this session.

### [3AS04e-04]

### What the Education Committee of the Japanese Physiological Society should do and wants to do

*Susumu Minamisawa[1] ([1]*The Jikei University School of Medicine*)

### Symposium

[3S05e]
**Innovative neuro-chemical biology for understanding neuronal and synaptic functions**

March 16 (Thu.), 16:30 - 18:30, Room 5

### [3S05e-01]

### Chemical labeling of endogenous neurotransmitter receptors in the live mouse brain by Ligand-directed acyl imidazole chemistry

*Hiroshi Nonaka[1,2], Itaru Hamachi[1,2] ([1]*Graduate School of Engineering, Kyoto Univ.*, [2]*JST, ERATO*)

Chemical modification of proteins provides valuable tools to realize a wide range of biological applications, including functional studies of individual proteins in biological systems. However, site-specific and target-selective modification of proteins remains challenging due to the limited availability of suitable chemical strategies for such modifications. To solve this problem, we have developed a method for selective chemical modification of proteins, called "Ligand-directed acyl imidazole chemistry (LDAI chemistry)". This method allows us to introduce functional molecules into target proteins without requiring genetic manipulation and compromising the function of proteins. Our recent studies have demonstrated that LDAI chemistry is applicable to selective labeling of endogenous neurotransmitter receptors in primary cultured neurons and in a live mouse brain. In this talk, we will present our chemical labeling method for endogenous neurotransmitter receptors and discuss its application.

### [3S05e-02]

### *In vivo* molecular nanoarchitecture of synapse revealed by expansion microscopy techniques

*Kazuya Nozawa[1], Taku Sogabe[1], Ayumi Hayashi[1], Michisuke Yuzaki[1] ([1]*Department of Neurophysiology, Keio University School of Medicine*)

Neuronal circuits are connected via small adhesion structure, synapse. In synapses, many molecules are enriched and involved in the regulation of synaptic structure and function. Recent developments of super-resolution microscopy have revealed that some molecules, including presynaptic release machinery and postsynaptic neurotransmitter receptor, are organized into subsynaptic nanodomain. However, it has been difficult to perform multicolor super-resolution imaging of endogenous proteins without overexpressing tagged proteins, particularly in tissue sample. Here, we demonstrate the imaging approach using epitope-tag knock-in mouse lines and super resolution technique, expansion microscopy: the former enables specific detection of endogenous proteins, and the latter enables triple-color super-resolution imaging by isotropic physical expansion of the specimen. Using this approach, we revealed nanoscale relationship between Nlgn1, LRRTM1, and Cbln1, which is known to regulate the different types of ionotropic glutamate receptors. We will also discuss our current development and application of expansion microscopy techniques toward the nanoscale understanding of molecular biology of the synapse.

### [3S05e-03]

### Mapping neurotransmitter receptor interactomes in live mice by photoactivated proximity labeling

*Mikiko Takato[1], Seiji Sakamoto[1,2], Hiroshi Nonaka[1,2], Tomonori Tamura[1,2], Itaru Hamachi[1,2] ([1]*Graduate School of Engineering, Kyoto University*, [2]*JST ERATO*)

Identifying the protein networks that modulate neurotransmitter receptor function are critical to furthering our understanding of the brain at the molecular level. In recent years, enzymatic proximity labeling has emerged as a powerful tool for discovering protein interactomes in and on living cells. However, in live animal models, it lacks the temporal resolution necessary to capture rapid changes in synaptic proteomes. Moreover, genetic fusion of an exogenous enzyme to the protein of interest may perturb its native function and obstruct protein-protein interactions. To address these issues, we developed a nongenetic, optochemical proximity labeling method called PhoxID (photooxidation-driven proximity labeling for proteome identification). In PhoxID, a photosensitizer is chemically tethered to a protein of interest inside a living organism. Upon irradiation with visible light, the photosensitizer locally generates $^1O_2$, which, based on its half-life, oxidizes proteins within several tens of nanometers from the target protein. The oxidized proteins can be tagged by a nucleophilic biotin labeling reagent, purified, and identified by LC-MS/MS. In this presentation, we report the use of PhoxID in mapping the interactomes of the AMPA-type glutamate receptor and the $GABA_A$ receptor in the live mouse brain.

### [3S05e-04]

### Optical inactivation of molecular functions by CALI and its application for memory analysis.

*Kiwamu Takemoto[1] ([1]*Department of Biochemistry Mie University, Graduate School of Medicine*)

CALI (chromophore-assisted light inactivation) method is one of the promising technologies for molecular inactivation by using photosensitizers that release reactive oxygen species upon light irradiation. We have been developing elemental technologies important for the CALI method (Takemoto K et al. *ACS. Chem. Biol.* 2011, Takemoto K et al. *Sci. Rep.* 2013). We have further applied these techniques to develop a CALI method for the AMPA receptor GluA1 homomer and found that this molecule is critical for memory acquisition (Takemoto K et al. *Nat. Biotechnol.* 2017). Our method is attracting a lot of attention as a next-generation technology for neuroscience (Humeau Y et al. *Nat. Neurosci.* 2019, Frank JA et al. *Nat. Biotechnol.* 2019, Paoletti P et al. *Nat. Rev. Neurosci.* 2019 etc.). In this session, we describe a new CALI method for another AMPA receptor, GluA2/3 and its application to memory analysis. We applied this method to the hippocampal CA1 region and found a novel function related to memory that is distinct from the GluA1 homomer. Finally, we will present HyperNova, a novel photosensitized fluorescent protein with fast and efficient maturation at 37°C, which is useful for CALI of intracellular molecules in mammalian cells. We are establishing techniques to manipulate both membrane proteins and intracellular molecules, and expect to realize optical manipulation of more molecules in the future.

### [3S05e-05]

### A novel chemogenetics for cell-type-specific activation of glutamate receptors

*Shigeki Kiyonaka[1] ([1]*Nagoya University*)

Chemogenetics is a powerful approach for selective activation of target receptors in a cell-type-specific manner. One of the typical examples is DREADD, which has been widely utilized for cell-type-specific activation of G-protein signaling in live animals. Although powerful, original ligand-binding properties are lost in DREADD. Thus, DREADD is not applicable for understanding the physiological roles of receptors of interest. Our research direction is developing chemogenetic methods in which original ligand-binding properties of receptors are retained. We have recently developed a chemogenetic method, termed coordination-based chemogenetics (CBC) for selective activation of ion-channel-type or GPCR-type glutamate receptors. CBC is a unique chemogenetic approach in which histidine mutations are introduced into an appropriate position of the receptor of interest using structural information of the ligand-binding site. A designed metal complex induces a structural change for receptor activation, which allowed chemogenetic regulation of glutamate receptors in target neurons. In this talk, I will present our recent progress of our chemogenetic approaches.

### Symposium

[3S06e]
**Mind sensing by an integration of neuroscience and engineering technologies and its use for communication aid**

March 16 (Thu.), 16:30 - 18:30, Room 6

### [3S06e-01]

### Mind sensing by an integration of neuroscience and engineering technologies and its use for communication aid

*Ken-Ichiro Tsutsui[1] ([1]*Tohoku University*)

This symposium aims to introduce a new research and development project which aims to realize mind sensing and perceptual, motor, and cognitive interventions by integrating state-of-the-art neuroscience and engineering technologies. (Moonshot Goal #9, "Development of "At-will Translator (Jizai hon-yaku ki)" connecting various minds based on brain and body functions") We aim to showcase the element technologies for mind sensing and perceptual, motor, and cognitive interventions and discuss the future that will be brought by this project.

### [3S06e-02]

### Deciphering mental states from multidimensional physiological signals in rodents

*Takuya Sasaki[1,2] ([1]*Graduate School of Pharmaceutical Sciences, Tohoku University,* [2]*Tohoku University School of Medicine*)

In this symposium, I would like to summarize our neurophysiological reports related to these brain functions, highlighting the importance of network-level studies and introduce our recent studies to understand brain-body interactions using our novel techniques. Peripheral organ functions such as cardiovascular and respiratory activity are controlled by the brain. While most early studies have mainly focused on physiological events within a single organ, it remains largely unknown how the brain and peripheral organs interact with each other. We hereby developed a recording method that comprehensively monitors electrical biosignals representing cardiac rhythm, breathing rhythm, vagus nerve spikes, awake/sleep-related muscle contraction, and collective neuronal activity of multiple brain regions. Multi-dimensional analysis applied to these physiomics datasets revealed that certain activity patterns in the brain reflected future peripheral activity state. We are now extending this idea to a number of biological research issues of how the brain-body association is altered in response to various environmental changes, emotional challenges in health, and dysfunction of internal organs in disease.

### [3S06e-03]

### Decoding mental states from metastable synchrony dynamics of the brain and the body in humans

*Keiichi Kitajo[1] ([1]*National Institute for Physiological Sciences*)

I present our approaches to decoding mental states from metastable synchronization dynamics across the brain and the body in humans. First, I introduce the theoretical background of metastable synchrony dynamics from a dynamical systems theory viewpoint. Next, I introduce our scalp electroencephalography (EEG) studies showing that metastable EEG synchrony is associated with individual differences in psychological traits and brain disorders. Next, I show our data regarding synchrony between the brain (EEG) and multimodal biological signals from the body, such as electrocardiographic and respiration recordings. Since these brain and body signals show distinct frequency characteristics, we propose novel methods to assess the degree of synchrony between distinct frequency bands. Finally, I discuss how we can decode mental states from these multimodal signals focusing on metastable synchrony.

### [3S06e-04]

### Reconstruction of Non-verbal Expressions of Humans in a Virtual Environment by Ubiquitous Optical Sensing

*Maki Sugimoto[1] ([1]*Keio University*)

This talk presents a technology to reproduce non-verbal expressions of humans with embedded optical sensing in cyberspace. With the aid of machine intelligence, our human body and behavior can be extended in various ways. For instance, we are able to have an augmented avatar as our embodiment in the metaverse. By reflecting our expressions to the avatar, we are able to retain a sense of agency and ownership for the embodiment. Recent technologies allow us to make such reproduction of non-verbal expressions with various methods. This talk introduces a highly flexible sensing system with low dimensional embedded optical sensors for wearable devices such as a head-mounted display and machine learning with a low computational cost. By utilizing such a ubiquitous sensing technique, we can make synchronization with the virtual embodiment. Furthermore, it is possible to remap a degree of freedom between physical and virtual elements with this technique. We can use our expressions as a command set to drive augmented embodiment in cyberspace. Through such remapping of the physical and virtual elements, it is possible to design new embodiments beyond physical restrictions.

### [3S06e-05]

### From Jizai Body to Jizai Mind

*Masahiko Inami[1] ([1]*The University of Tokyo*)

In this talk, we introduce a concept called “JIZAI Body”[1] that allows each person to live the way they wish to live in society. One who acquires a JIZAI Body can (simultaneously) control (or delegate control) of their natural body and extensions of it, both in physical and cyberspace. We begin by describing the JIZAI Body and the associated JIZAI state in more detail. We then provide a review of the literature, focusing on human augmentation and cybernetics, robotics and virtual reality, neuro and cognitive sciences, and the humanities; fields which are necessary for the conception, design, and understanding of the JIZAI Body. We then illustrate the five key aspects of a JIZAI Body through existing works. Finally, we present a series of example scenarios to suggest what a JIZAI society may look like. Overall, we present the JIZAI Body as a preferred state to aspire towards when developing and designing augmented humans.   In this symposium, examples of JIZAI Bodies will be introduced, as well as a perspective on JIZAI Mind.   [1] Masahiko Inami, Daisuke Uriu, Zendai Kashino, Shigeo Yoshida, Hiroto Saito, Azumi Maekawa, and Michiteru Kitazaki. 2022. Cyborgs, Human Augmentation, Cybernetics, and JIZAI Body. In Augmented Humans 2022 (AHs 2022). Association for Computing Machinery, New York, NY, USA, 230–242. https://doi.org/10.1145/3519391.3519401

### Symposium

[3S07e]
**Homeostatic regulation by a cooperation of sensory function and autonomic nervous system**

March 16 (Thu.), 16:30 - 18:30, Room 7

### [3S07e-01]

### Anti-inflammatory effect via the autonomic nervous system

*Chikara Abe[1] ([1]*Gifu University*)

Activation of the immune system via the autonomic nerves, including sympathetic and parasympathetic nerves, is a possibility for the preventive medicine. Electrical stimulation of the vagal afferents and/or efferents reduces subsequent inflammation. In the pathway through the vagal afferent, stimulation of C1 neuron in medulla oblongata, which is a center of the autonomic nervous system, is important for the anti-inflammatory effect. Furthermore, activation of immune cells in the spleen via the splenic sympathetic nerves is indispensable for the protective effect. In this symposium, we will introduce the mechanism of the anti-inflammatory via the autonomic nervous system and C1 neurons using a disease animal model including kidney injury and pneumonia.

### [3S07e-02]

### Sensing mechanisms of gut osmolality in the vagus nerve

*Takako Ichiki[1], Tongtong Wang[2], Ann Kennedy[2], Allan-Hermann Pool[2], Haruka Ebisu[2], David J. Anderson[2], Miho Terunuma[1], Yuki Oka[2] ([1]*Division of Oral Biochemistry, Graduate School of Medical and Dental Sciences, Niigata University,* [2]*California Institute of Technology*)

Ingested food and water stimulate sensory systems in the oropharyngeal and gastrointestinal areas before absorption. These sensory signals send feed-forward modulation signals to brain appetite circuits. Emerging evidence suggests that gut osmolality sensing rapidly inhibits thirst neurons upon water intake. Nevertheless, it remains unclear how visceral sensory neurons detect gut osmolality changes, and how they transmit the signals to the brain to modulate thirst. We used optical and electrical recording to show that the vagal, but not the spinal pathway mediates visceral osmolality responses. Gut hypotonic stimuli activate a dedicated vagal population distinct from mechanical-, hypertonic, or nutrient-sensitive neurons. These hypotonic responses are partly mediated by a genetically defined vagal population. We demonstrate that hypotonic responses are mediated by vagal afferents innervating the hepatic portal area (HPA), through which the majority of water and nutrients are absorbed. These responses are mediated partly by vasoactive intestinal peptide secreted after water ingestion. Together, our results revealed gut hypoosmolality as an important vagal sensory modality, and that intestinal osmolality change is translated into hormonal signals to regulate thirst circuit activity through the HPA pathway.

### [3S07e-03]

### Neural circuits mediating visual input for body homeostasis in mammals

Kota Tokuoka[3,4], *KEISUKE YONEHARA[1,2,3,4] ([1]*National Institute of Genetics,* [2]*The Graduate University for Advanced Studies, SOKENDAI,* [3]*DANDRITE- Danish Research Institute of Translational Neuroscience,* [4]*Dept. of Biomedicine, Aarhus University*)

The superior colliculus translates various sensory inputs into innate behaviors important for animal survival, such as hunting and escape behaviors. The superior colliculus may also regulate autonomic functions that are important in preparing for the initiation of innate behaviors. My laboratory combines calcium imaging of freely moving mice, anatomical neuronal tracing, and quantification of behavioral and physiological responses to decipher the neural logic by which visual input is translated into systemic homeostatic control. In this talk, I will present our laboratory's recent work on this topic.

### [3S07e-04]

### Neural circuits for taste perception in hunger

*Ken-ichiro Nakajima[1] ([1]*Division of Endocrinology and Metabolism, National Institute for Physiological Sciences (NIPS)*)

The gustatory system plays a critical role in sensing appetitive and aversive taste stimuli for evaluating food quality. Although taste preference is known to change depending on internal states as shown in a famous Western proverb "Hunger is the best sauce", a mechanistic insight remains unclear. To answer this question, we examine the neuronal mechanisms regulating hunger-induced taste modification in mice. Starved mice exhibit an increased preference for sweetness and tolerance for aversive taste. Since Agouti-related peptide (AgRP)-expressing neurons in the arcuate nucleus of the hypothalamus play a pivotal role in triggering appetite, we evaluate the role of AgRP neurons with optogenetic experiments. We found that the hunger-induced taste modification is recapitulated by selective activation of AgRP neurons projecting to the lateral hypothalamus, but not to other regions. Importantly, both appetitive and aversive tastes are modified by these lateral hypothalamus projecting AgRP neurons. We next characterized the lateral hypothalamic neurons that function as downstream neurons of AgRP neurons. Pathway specific chemogenetic experiments showed that glutamatergic neurons in the lateral hypothalamus play a key role in modulating preferences for both appetitive and aversive tastes by using distinct pathways projecting to the lateral septum or the lateral habenula, respectively.

### [3S07e-05]

### regulation of feeding by tuberal nucleus somatostatin neurons

*Yu Fu[1] ([1]*Agency for Science Technology and Research*)

An environment that promotes excessive food intake is a major cause of obesity epidemic, but how food palatability and the environmental context information are integrated to influence feeding behavior is unknown. We found that tuberal nucleus somatostatin ($^{TN}$SST) neurons showed specific higher activity to palatable food. Repeated pairing of activating $^{TN}$SST neurons with a context led to contextual conditioned consumption of normal chow in sated mice.
The $^{TN}$SST neurons received subiculum (Sub) input that was required for both formation and expression of contextual feeding conditioning (CFC). Physiological CFC driven by hunger and palatable food specifically required $^{TN}$SST neurons and potentiated synaptic transmission between Sub and $^{TN}$SST neurons. Brain-wide input-output tracing further reveals that $^{TN}$SST neurons integrate multimodal information for regulating complex feeding behaviors.

### Symposium

### [3S08e]
### Frontiers in structure-based molecular physiology focusing on membrane transport proteins

March 16 (Thu.), 16:30 - 18:30, Room 8

### [3S08e-01]

### The structural basis of the divalent cation block generated in prokaryotic cation channel

*Katsumasa Irie[1] ([1]*Department of Biophysical chemistry, School of Pharmaceutical Sciences, Wakayama Medical University*)

Divalent cation blocking is observed in important tetrameric cation channels. For the blocking, divalent cation is thought to stack in the ion pathway of the channel, but this has not yet been directly observed, so the blocking mechanism by these small divalent cations remains uncertain. Prokaryotic tetrameric channels are one of the channel groups whose structural analysis is the most advanced. By utilizing the accumulated knowledge of structural analysis of them, it has become possible to know the molecular basis of the various primary mechanism of eukaryotic ion channels.Here, we elucidated the divalent cation blocking mechanism by reproducing the blocking effect into NavAb, which is a well-studied tetrameric sodium channel. Our crystal structures of NavAb mutants showed that the mutations increasing the hydrophilicity of the inner vestibule of the pore domain enable a divalent cation to stack on the ion pathway. Furthermore, molecular dynamics simulation showed that the stacking calcium ion repels the sodium ions at the bottom of the selectivity filter. These results suggest the primary mechanism of the divalent cation block in biologically essential channels.

### [3S08e-02]

### Proper interaction between the S1 segment of KCNQ1 and KCNE3 is required for the constitutively open nature of the KCNQ1-KCNE3 $K^+$ channel complex.

*Go KASUYA[1], Koichi NAKAJO[1] ([1]*Division of Integrative Physiology, Department of Physiology Jichi Medical University School of Medicine*)

KCNQ1 channel is a voltage-gated $K^+$ channel expressed both in excitable and non-excitable cells. Five KCNE proteins (KCNE1-3) are single transmembrane proteins that function as auxiliary subunits for the KCNQ1 channel. Among the KCNE proteins, KCNE3 protein binds to the voltage sensor domain (VSD) of KCNQ1 and stabilizes VSD at an intermediate position, thereby making the KCNQ1-KCNE3 channel complex a constitutively open $K^+$ channel. However, it remains unknown how KCNE3 proteins stabilize KCNQ1 VSDs at an intermediate position. The recent cryo-EM structure of the KCNQ1-KCNE3 channel complex shows that the S1 segment of KCNQ1 and KCNE3 are tightly interacted with each other, suggesting that the interaction is crucial for stabilizing VSD at a specific position. To investigate the functional importance of the interaction, we mutated all the amino acid residues on the S1 segment of KCNQ1 and KCNE3 facing each other to various sizes of amino acid residues (“volume scanning”) and found that the distance between the S1 segment and KCNE3 is properly optimized to achieve the constitutive activity. Besides, we identified two pairs of KCNQ1 and KCNE3 mutants that partially restored constitutive activity. These results indicate that the proper interaction between the S1 segment and KCNE3 is required to stabilize VSD at an intermediate position to achieve the constitutive channel activity.

### [3S08e-03]

### Integrative multi-omics and synthetic biochemistry unveil the hidden functions of the known transporters

*Pattama Wiriyasermkul[1], Shushi Nagamori[1] ([1]*Jikei University School of Medicine*)

All organisms maintain their metabolic homeostasis through the functions of membrane transport proteins, which are expressed in every cell. Traditionally, dissecting functions of transporters have relied on sequence similarities and putative substrates. However, 30% of transporters are still orphans and this approach limits physiological functions, especially at the tissue level where multiple transporters function coordinately and differently in distinct local environments. Here, we utilized various approaches, such as proteomics, metabolomics, and reconstituted transport assays, to reveal the physiological roles of the transporters. As a paradigm, we identified transport systems for D-serine in the kidney, an organ containing diverse and abundant transporters. D-Serine has been identified as a promising biomarker for diagnosing acute kidney injury (AKI) and chronic kidney disease (CKD). By integrated approach, we discovered D-serine to be a non-canonical but physiological substrate of sodium-monocarboxylate transporters (SMCTs). Our study enlightens the hidden physiological functions of transporters and provides a platform for investigations of other membrane transport systems across all tissues.

### [3S08e-04]

### Structural basis for $Ca^{2+}$-induced opening of cardiac ryanodine receptor

*Haruo Ogawa[1] ([1]*Graduate School of Pharmaceutical Sciences, Kyoto University*)

Cardiac ryanodine receptor (RyR2) is a $Ca^{2+}$ release channel localized in the sarcoplasmic reticulum and plays a key role in excitation-contraction coupling in the heart. RyR2 is a large molecule with a total molecular weight of 2.2 MDa and functions as a homotetramer. It is also well known that mutations in RyR2 (more than 300 locations) are implicated in severe arrhythmogenic diseases. RyR2 opens by the binding of $Ca^{2+}$, yet the mechanism by which a small molecule like $Ca^{2+}$ regulates the opening of RyR2, a supermolecule 60,000 times larger than RyR2, and the structural basis for the abnormal channel activity induced by disease mutations remains unknown. Here, by combining high-resolution structures determined by cryo-electron microscopy with quantitative functional analysis of channels carrying various mutations in specific residues, we have succeeded in elucidating the gating mechanism of RyR2 by $Ca^{2+}$ binding and the mechanism by which disease mutations cause channel abnormalities at the atomic level.

### [3S08e-05]

### Development of reconstituted skeletal muscle depolarization-induced $Ca^{2+}$ release and its application to elucidation of structural mechanism

*Takashi Murayama[1] ([1]*Juntendo University*)

In skeletal muscle, depolarization of the plasma membrane triggers $Ca^{2+}$ release from the sarcoplasmic reticulum (SR), referred to as depolarization-induced $Ca^{2+}$ release (DICR). DICR occurs via the type 1 ryanodine receptor (RyR1), which physically interacts with the dihydropyridine receptor Cav1.1 subunit. Recent studies have revealed that DICR requires specific machinery formed with additional essential components including DHPR β1a subunit, Stac3 adaptor protein and junctophilins. RyR1 also mediates $Ca^{2+}$-induced $Ca^{2+}$ release (CICR), in which binding of $Ca^{2+}$ opens the channel. It remains so far unclear about mechanism of DICR and its relationship with CICR. We have recently established the platform for reconstituted DICR in HEK293 cells using baculovirus infection of the essential components. High $[K^+]$ depolarization triggered rapid $Ca^{2+}$ release from ER in a $[K^+]$-dependent manner. The platform has great advantage in easily testing the mutant components. Based on the recent structures at near-atomic resolution, we generated several mutations in RyR1 and tested them in our DICR platform. This approach will accelerate elucidation of structural mechanism of DICR.

### Symposium

[3S09e]
**Mereological Neurophysiology**

March 16 (Thu.), 16:30 - 18:30, Room 9

### [3S09e-01]

### Neurophysiology with the mereological imaging

*Makoto Osanai[1,2,3] ([1]*Osaka University,* [2]*CiNet/NICT,* [3]*Tohoku University*)

"Mereological fallacy" (Bennett and Hacker, 2003) is ascribed to the idea that the whole can be represented by a set of parts. The nervous system has a highly hierarchical structure and interactions within and between each level of the hierarchy. Therefore, directly linking phenotypes, the output results of the brain, to measurement results in one part of the brain involves a logical fallacy. To avoid this fallacy, we have been developing a multi-scale and multi-modal imaging method and applying it to neurophysiological research. For in vivo whole-brain activity analysis, we developed and used the quantitative activation-induced manganese-enhanced MRI (qAIM-MRI). qAIM-MRI is based on the use of $Mn^{2+}$, which shortens the longitudinal relaxation time (T1) of $H^{+}$, as a surrogate marker of $Ca^{2+}$ influx. Using qAIM-MRI, we can see where the neural activity has changed. To see how it changes, we use, we are developing the ultra-thin fluorescence endoscope imaging system (U-FEIS). U-FEIS can record the multicellular neuronal activities from the deep brain region. To see why the neural activity has changed, we use in vitro imaging methods. We have been conducting $Ca^{2+}$ imaging on brain slice preparations. By combining these techniques and applying them to the same animal, we can get closer to elucidating the function expression mechanisms of the nervous system, avoiding the mereological fallacy.

### [3S09e-02]

### Multimodal microelectronic fiber technologies for brain mereology

*Yuanyuan Guo[1] ([1]*Tohoku University*)

Establishing the causal relationship between molecules, cells, circuits, and systems to behavior levels of neural networks within the brain and nervous system is critical for the full understanding of the part-whole relation underlying brain functions. However, a formidable obstacle is a lack of technologies to achieve such a mereological approach to neural networks, deciphering their functions across their multi-level and multi-modal signaling mechanisms. Recently, leveraging the thermal drawing process, we have successfully developed multifunctional fibers, with optical, chemical, and electrical functionalities within a thin strand of fiber (Y. Guo et al, ACS Nano 2017; S. Park, Y. Guo, et al, Nature Neuroscience, 2017.). Lately, we further expanded fiber functionality with electrochemical sensing and actuation modalities, not only for advancing fundamental neuroscience but also for studying brain-body interactions.   In this talk, in the first part, I will discuss our work in advancing fiber-based multimodal bio-interface with a focus on deciphering in-brain intrinsic chemical release (Y. Guo et al., Biosensors and Bioelectronics, 2021; PLOS One, 2020; R. Nishimoto et al, Biosensors 2022). I will introduce our recent development - the aptamer functionalization on microelectronic fibers for in vivo neurochemical sensing.  I will also discuss deep-brain chemical imaging via polymer-fiber-coupled field effect sensors as well as multielectrode-fiber-enabled bipolar electrochemistry. In the second part, I will talk about the mechanical actuation within fibers, which we recently succeeded in incorporating within fiber devices (PCT/JP2022/17664). Such fiber could move guided by sensing information. Lastly, not limited to fundamental studies in vivo, we recently developed fiber-based textiles, that could be seamlessly integrated into our clothes for monitoring physiological signals, such as brain waves, chemical molecules in the sweat, and respiration rate, in our daily life. Such fiber-based smart textiles open the possibilities for investigating brain-body interactions as well as monitoring our mental and physical health status.

### [3S09e-03]

### Mereology for the basal ganglia

*Yoshio Iguchi[1], Kazuto Kobayashi[1] ([1]*Fukushima Medical University*)

One of the roles of the central nervous system is to order and orchestrate the organism's behavior toward its goal, which requires information about the environment, including oneself. The brain can be regarded as an organ responsible for converting environmental information (stimuli) into motor information (responses), and the basal ganglia are thought to play an essential role in learning for stimuli-responses mapping. In particular, the striatum, the input layer of the basal ganglia, is divided into two anatomically distinct subnuclei, the medial (caudate) and lateral (putamen), each composed of dopamine D1 receptor-positive direct-pathway neurons and D2 receptor-positive indirect-pathway neurons. Much work has studied the effects of the selective lesion in the subnuclei or cell type-specific ablation in each in stimulus-response learning. In order to understand the relationship between detailed information about such parts and the function of the overall structure of the striatum and basal ganglia (i.e., mereology of the striatum and basal ganglia), a system is needed to manipulate the activity of direct and indirect pathway neurons in a single organism in a cell-specific, arbitrary, and reversible manner. We will present our attempts to development of a bland new chemogenetic toolkit for this purpose.

### [3S09e-04]

### Mereology for organizing episodic memories in the hippocampus

*Noriaki Ohkawa[1] ([1]*Dokkyo Med Univ*)

Memories of novel episodes in daily life are stored through a subset of neurons, termed engram cells. However, how different sets of engram cells are selected for current and next episodes remains unclear. To elucidate organization of engram cells, we established a unique imaging system to identify engram cells and to record the $Ca^{2+}$ events corresponding to the activity of engram cells and non-engram cells during memory processing of a novel episodic event. By this system, we found out that information of one episodic experience is composed of several hippocampal engram sub-ensembles defined by individual synchronous activities, and a portion of the engram sub-ensemble activities survive from post-experience sleep through retrieval session. On the other hand, a subset of non-engram cells developed population activity, which was constructed during post-learning offline periods, and then emerged to represent new learning. These results proposes that a circulation of appearance of ensemble activities from engram and non-engram cells is principle of mereological organization of daily episodic memories in the hippocampus.

### [3S09e-05]

### Mereological reinforcement learning

*Kazuhiro Sakamoto[1] ([1]*Tohoku Medical and Pharmaceutical University*)

Unlike dice, which, though uncertain, are known to produce a number from 1 to 6, the real world changes from moment to moment, sometimes unexpectedly. In order to adapt and survive in such an environment where even the probability space is not fixed, i.e., an indefinite environment, a mereological structure is essential to determine the role to be played by each part within the individual while maintaining the consistency of the function of the whole. Here, the authors introduce a reinforcement learning model, constructed based on behavioral neuroscience experiments on primates, that learns while determining the probability space or state. The model determines the state/learning framework of "what to learn" so that it can uniquely determine its behavior. The reinforcement learning model that dynamically changes the state space based on this decision uniqueness is also useful for understanding higher brain functions. This learning mechanism provides an critical strategy for organisms to adapt to indefinite environments.

### Symposium

### [3S10e]
### Inter-relation of biosensing functions and their role in the regulation of circulation during exercise

March 16 (Thu.), 16:30 - 18:30, Room 10

### [3S10e-01]
### Cardiopulmonary baroreflex control of sympathetic vasomotor outflow regulation during dynamic exercise

*Keisho Katayama[1] ([1]*Nagoya University*)

Appropriate regulation of sympathetic vasomotor outflow is necessary during dynamic exercise to maintain arterial blood pressure and facilitate the delivery of blood flow to active muscles. Multiple neural mechanisms, mainly central command, the exercise pressor reflex (i.e., metaboreflex and mechanoreflex), the arterial baroreflex, and the cardiopulmonary baroreflex, work in concert to regulate sympathetic vasomotor outflow during exercise. Interestingly, muscle sympathetic nerve activity (MSNA) decreases or does not change during dynamic exercise (i.e., leg cycling) with light or mild intensity from that at rest despite activation of central command. The decreased MSNA in dynamic leg exercise is linked to the loading of cardiopulmonary baroreceptors, which is attributable to muscle pump-induced increases in venous return and central blood volume. In contrast, MSNA increases during dynamic leg exercise above moderate intensity. From these reports, it has been suggested that the sympathoinhibitory effect of the cardiopulmonary baroreflex could be modulated by a powerful sympathoexcitatory drive from the exercise pressor reflex (i.e., metaboreflex) above moderate intensity. In my presentation, I will focus on the interaction between the sympathoinhibitory influence of cardiopulmonary baroreflex and sympathoexcitatory effects of the skeletal muscle metaboreflex.

### [3S10e-02]
### Effect of exercise on sensing functions of blood vessels

*Shigehiko Ogoh[1] ([1]*Toyo University*)

Sensing of various physiological factors (blood pressure, blood $O_2$/$CO_2$, etc.) plays an important role in the respiratory and circulatory homeostasis of living organisms. In fact, it has been shown that a decline in this sensing function (baroreceptor reflex, respiratory reflex, vascular endothelial function, etc.) is one factor in the onset of respiratory and cardiovascular diseases. Since this sensing function is mediated by autonomic nerve activity, it has been confirmed that the sensing function of the respiratory system affects the circulatory system and vice versa, suggesting an interrelationship between the sensing functions of each physiological system. Thus, for example, in exercise therapy, different sensing can have different effects on the circulatory respiratory system. In this symposium, we will focus on the interaction of sensing of each physiological function and will also review the findings of related previous studies and the effects of exercise training on the sensing function of blood vessels.

### [3S10e-03]
### Influence of sensory functions, primarily thermal factors, that modulate circulatory response during exercise

*Manabu Shibasaki[1] ([1]*Nara Women's University*)

Cardiovascular responses during exercise are influenced not only by the internal demands of the body, but also by the environmental temperature and various pressures (i.e., atmospheric, hydraulic, and gravitational pressures). In addition to environmental temperature, exercise itself increases body temperature, resulting in complicated changes of the circulatory system. Given that the increase in heat stroke and exhaustion due to global warming and urban heat islands is a serious issue, sufficient knowledge is needed to prevent heat-related diseases during exercise. This presentation will focus on temperature factors, and discuss the following topics: 1) the relationship between sympathetic thermoregulatory responses and exercise commands from higher centers, called "central commands", muscle afferent inputs from metaboreceptors and mechanoreceptors, 2) the effects of elevated body temperature on circulation, and 3) the relationship between elevated body temperature and brain function. From the perspective of heat stroke prevention, I will summarize the contribution of thermal sensing to the cardiovascular system.

### [3S10e-04]
### Impact of cardiovascular disease on sensory afferent function during physical exercise

*Masaki Mizuno[1] ([1]*University of Texas Southwestern Medical Center*)

In patients with cardiovascular disease or diabetes, physical exercise elicits an abnormal increase in blood pressure (BP). As potentiated BP responses to physical exertion are associated with increased risk for adverse cardiovascular events during exercise, elucidating the mechanisms responsible is clinically important. In addition, the aberrant circulatory response limits the safety of exercise prescription, which is problematic since habitual physical activity is known to be a therapy with demonstrated potential for improving overall cardiovascular health. Afferent signals from working skeletal muscle are one of the important sources of neural input to the cardiovascular centers controlling cardiovascular function during physical activity (exercise pressor reflex, EPR). Increasing evidence suggests that the abnormal EPR function significantly contributes to the generation of the enhanced circulatory responses in these disease. Moreover, it has been demonstrated that the EPR dysfunction is mediated by both mechanosensitive ion channel (Piezo1) and chemically-sensitive receptors (transient receptor potential vanilloid 1, TRPV1). The focus of this symposium presentation will be on the recent evidence aimed at determining the mechanisms underlying the heightened BP response to exercise in cardiovascular disease or diabetes.

### Educational Programs

### Model Lecture

March 15 (Wed.), 16:30 - 18:30, Room 4

**2EL04-01**

**An ingenious combination of platelets, coagulation factors and fibrinolytic factors**

*MARIKO NAKAMURA[1] *([1]Dept.of Molecular and Cellular Physiology, Graduate schoo of medicine, University of the Ryukyus)*

### Model Lecture

March 15 (Wed.), 16:30 - 18:30, Room 4

**2EL04-02**

**Glucose homeostasis and endocrine pancreas**

*Noriko Takahashi[1] *([1]Department of Physiology, Kitasato University School of Medicine)*

### Model Lecture

March 15 (Wed.), 16:30 - 18:30, Room 4

**2EL04-03**

*Masaki Morishima[1] *([1]Kindai University)*

### Educational Lecture

March 16 (Thu.), 9:00 - 11:00, Room 5

**3EL05-01**

*Takeshi SAKURAI[1] *([1]University of Tsukuba)*

### Educational Lecture

March 16 (Thu.), 9:00 - 11:00, Room 5

**3EL05-02**

**Physiology of Pain**

*Kazue Mizumura[1,2] *([1]Department of Physiology, Nihon University School of Dentistry, [2]Nagoya University)*

### Educational Lecture

March 16 (Thu.), 9:00 - 11:00, Room 5

**3EL05-03**

**Integration of learning and assessment**

*Yoshihiro Kitamura[1] *([1]Nippon Medical School)*

### Poster Presentation

### Day 1
### (March 14, 12:10 - 14:10)

[1P] Neurophysiology, Neuronal cell biology - Plasticity
[1P] Neurophysiology, Neuronal cell biology - Neural network
[1P] Neurophysiology, Neuronal cell biology - Neurons, Synapses
[1P] Neurophysiology, Neuronal cell biology - Higher brain function
[1P] Neurophysiology, Neuronal cell biology - Motor function
[1P] Neurophysiology, Neuronal cell biology - Sensory function, Sensory organ
[1P] Neurophysiology, Neuronal cell biology - Others
[1P] Molecular physiology, Cell physiology - Ion channels, Receptors
[1P] Molecular physiology, Cell physiology - Others
[1P] Muscle
[1P] Digestion, Digestive system
[1P] Oral physiology
[1P] Circulation
[1P] Respiration
[1P] Urinary organ, Renal function, Urination
[1P] Autonomic nervous system
[1P] Physical fitness and sports medicine
[1P] Nutritional and metabolic physiology, Thermoregulation
[1P] Behavior, Biological rhythm, Sleep
[1P] Stress
[1P] Anthropology
[1P] Pathophysiology

### Poster Presentation

### [1P]
### Neurophysiology, Neuronal cell biology
### Plasticity

March 14 (Tue.), 12:10 - 14:10, Poster Room

### [1P-001]

**Motor training promotes both synaptic and intrinsic plasticity of layer V pyramidal neurons in the primary motor cortex**

*Hiroyuki Kida[1], Kawakami Ryosuke[2], Sakimoto Yuya[1], MItsushima Dai[1] ([1]*Department of Physiology Yamaguchi University Graduate School of Medicine,* [2]*Department of Molecular Medicine for Pathogenesis, Graduate School of Medicine, Ehime University*)

Layer V neurons in primary motor cortex (M1) are important for motor skill learning. Since pretreatment of either CNQX or APV in rats M1 layer V impaired rotor rod learning, we analyzed training-induced synaptic plasticity by whole-cell patch-clamp technique in acute brain slices. One-day trained rats showed a decrease in small inhibitory postsynaptic current (mIPSC) frequency and an increase in the paired-pulseratio of evoked IPSCs, suggesting a transient decrease in presynaptic GABA release in early phase. Two-days trained rats showed an increase in miniature excitatory postsynaptic current (mEPSC) amplitudes/frequency and elevated AMPA/NMDA ratios, suggesting a long-term strengthening of AMPA receptor-mediated excitatory synapses. In current-clamp analysis, 1-day-trained rats showed an increase in action potential threshold and a decrease in firing rate, while 2-day-trained rats returned to pretraining levels, suggesting a dynamic changes in intrinsic properties. Furthermore, western-blot analysis of layer V detected decreased phosphorylation of Ser408–409 in GABAA receptor β3 subunits in 1-day trained rats, and increased phosphorylation of Ser831 in AMPA receptor GluA1 subunits in 2-days trained rats. Finally, live-imaging analysis of Thy1-YFP transgenic mice showed that the training rapidly recruited a substantial number of spines for long-term plasticity in M1 layer V neurons. Taken together, these results indicate that motor training induces complex and diverse plasticity in M1 layer V pyramidal neurons.

### [1P-002]

**The effect of acetylcholine on associative synaptic plasticity at the medial and the lateral perforant path synapses in the hippocampal dentate gyrus**

*Eriko Sugisaki[1], Kazuhisa Kamei[1], Tadayoshi Monden[1], Yoshikazu Isomura[2,1], Takeshi Aihara[1] ([1]*Tamagawa University,* [2]*Tokyo Medical and Dental University*)

The cells in the entorhinal cortex have projections to granule cells (GC) in the hippocampal dentate gyrus via the medial (MPP) and lateral (LPP) perforant path to convey spatial and non-spatial information respectively. Both MPP and LPP synapses are reported to induce synaptic plasticity. When attentional processes are paid, acetylcholine (ACh) can be released in the hippocampus. However, it is unknown how the associative synaptic plasticity at the MPP and LPP synapses are modulated under ACh treatment. To clarify the role of ACh in the associative synaptic plasticity, patch clamp recordings were made to the soma of GC along with the stimuli in the MPP and LPP using rat hippocampal slices. As the results, spike timing-dependent plasticity (STDP) at the MPP synapse was enhanced along with ACh if only MPP stimulation was applied, so as at the LPP synapse with LPP stimulation. Interestingly, paring stimulation of the MPP and LPP under ACh treatment decreased STDP at the MPP synapse, while the LPP synapse was similar to that induced by the LPP stimulation only. These results suggest that non-spatial input can influence on spatial learning as a form of information integration if attentional processes are paid.

### [1P-003]

**Investigation of effect of a casein kinase 2 inhibitor on the short-term presynaptic plasticity at the frog neuromuscular junction: irreversible enhancement of augmentation and potentiation.**

*Naoya Suzuki[1] ([1]*Dept. physics, Sch. Science, Nagoya Univ.*)

To investigate mechanism of stimulation induced increment of transmitter release, we analyzed effect of a casein kinase 2 inhibitor, tetrabromobenzotriazole (TBB), on the short-term synaptic plasticity at the frog neuromuscular junction (NMJ), especially augmentation, and potentiation. The NMJ preparation was firstly stimulated by 30 stimuli at 8 sec intervals to measure normal level of transmitter release, then the presynaptic plasticity was induced by 300 stimuli at 20Hz, lastly its decay process was monitored by a stimulation pattern (combination of 6 stimuli at 1.5 sec intervals, 7 stimuli at 4 sec intervals, and 41 stimuli at 8 sec intervals). Endplate currents (EPCs) were recorded extracellularly with a surface glass microelectrode. Both rising (during 20Hz tetanus) and decaying processes of amplitudes of EPCs were analyzed and compared before and after a treatment of TBB (bath application of 0.01mM TBB for 30 min then wash out it for 30 min). The augmentation and potentiation were irreversibly enhanced after the treatment of TBB.

### [1P-004]

**Visualization of a LTP-related signaling loop in a hippocampal neuron**

*Yusuke Sugimoto[1], Shin-ya Kawaguchi[2] ([1]*Faculty of Science, Kyoto University,* [2]*Graduate School of Science, Kyoto University*)

Long-term potentiation (LTP) is a major form of synaptic plasticity in hippocampus underlying learning and memory in animals. Molecular mechanisms for LTP induction have been extensively studied. A positive feedback loop including protein kinase C (PKC) and MAPK has been suggested as a key factor for LTP induction. However, it remains elusive how that signaling loop is activated and mediates the LTP establishment. To address this issue, we used a fluorescent protein consisting of a mutated PKCα and venus, which can detect activation of PKC-MAPK signaling loop implicated in the long-term depression (LTD) in cerebellar Purkinje cells. We found that chemical LTP stimulation caused sustained accumulation of the probe into spines in cultured hippocampal neurons, while it is ubiquitously distributed in the cytoplasm before the chemical induction. This recruitment lasted for at least 1 hour, suggesting that altered distribution of the probe may be related to the LTP occurrence. Together with electrophysiological analysis, we are going to demonstrate the possibility of the probe as a tool for LTP imaging in hippocampal neurons.

### [1P-005]

**Quantitative measurement of the activity of transcription factors in the brain during memory formation.**

*Ryoma Onodera[1], Ryotaro Fukue[1], Takeru Shiraishi[1], Kentaro Abe[1,2] ([1]*Graduate School of Life Sciences,Thoku University Brain Developmet,* [2]*Organization for Advanced Studies, Tohoku University*)

The formation of long-term memory requires new gene expression and protein synthesis. Transcription factors directly regulate gene transcription, thus, play an essential function during memory formation. However, their dynamic activity in vivo during memory formation is still needs to be understood. We have developed a battery of AAV-vector-based transcription factor activity reporters that can be used to measure the activity of dozens of transcription factors in the brain. In this research, we utilized this method to focus on the changes in the activity of transcription factors during memory formation in mice (Mus musculus). We presented learning stimulation in a social learning memory task. We then assayed transcription factor activity profiles in the mouse hippocampus and analyzed the time course of their change. We revealed that the activity of several transcription factors changed during memory formation, and some of them differed among long-term and short-term memory. Experimental manipulation of the activity of transcription factor by knockdown with the CRISPR/Cas9 system hindered the formation of memory, suggesting that the change of transcription factor activity is necessary for learning. By quantitatively analyzing the activity of multiple transcription factors in vivo, our method provides a landscape of transcription factor activity to investigate the molecular mechanisms behind memory formation.

### [1P-006]

### Supramammillary glutamatergic inputs exhibit NMDA receptor-dependent long-term potentiation in the dentate gyrus

*Yuki Hashimotodani[1], Himawari Hirai[1], Takeshi Sakaba[1] ([1]*Graduate School of Brain Science, Doshisha University*)

The supramammillary nucleus (SuM) projects to the dentate gyrus (DG) granule cells (GCs) and regulates GC activity through the co-transmission of glutamate and GABA. Although coordinated activity between the SuM and DG is implicated in spatial memory processing, it remains unclear whether the correlated activity of SuM and DG could induce Hebbian form of plasticity, which is thought to be a cellular model for associative learning and memory. We show that NMDA receptor-dependent long-term potentiation (LTP) is induced by pairing SuM input with GC spikes at glutamatergic, but not GABAergic, SuM-GC synapses. We found that this Hebbian form of LTP is input-specific, requires CaMKII activation, and is expressed postsynaptically. By inducing LTP, SuM inputs associated with perforant-path inputs effectively discharge GCs. Our results highlight the important role of associative plasticity at SuM-GC synapses in the regulation of DG activity.

### [1P-007]

### $GABA_B$ receptor- and NK1 receptor-mediated long-term plasticity in GABAergic synaptic connections in the rat cerebral cortex

*Kiyofumi Yamamoto[1], Masayuki Kobayashi[1] ([1]*Department of Pharmacology, Nihon University School of Dentistry*)

The cerebral cortex contains ~20% GABAergic neurons in the total neurons. GABAergic neurons, especially fast-spiking neurons (FSN), have many tight inhibitory synaptic connections to the adjacent neurons. As for long-term potentiation (LTP) and depression (LTD) in long-term synaptic plasticity, the plasticity in excitatory synapses has been widely studied, and presynaptic and postsynaptic mechanisms for induction were hypothesized. Although long-term plasticity in inhibitory synapses was also reported, the detailed mechanisms underlying the induction of LTP/LTD in the synapses were still unknown. Here, to examine whether LTP/LTD appeared in FSN→pyramidal neurons (PYR) and identify the mechanism, we performed the quadra whole-cell patch-clamp and recorded unitary IPSCs. θ-burst stimulation (TBS) to the presynaptic FSN elicited a variety of plasticity, including LTP/LTD. The degree of enhanced and decreased uIPSC amplitude in responses to TBS depended on the paired-pulse ratio (PPR) calculated from the amplitudes measured before the stimulation. Bath application of $GABA_B$ receptor antagonist and intracellular perfusion of GTPase inhibitor blocked to induce LTP. Surprisingly, a low concentration of baclofen (1 μM) induced LTP-like synaptic potentiation in FSN→PYR synapses. The results that U73122 suppressed LTP-induction indicated participation of $G_q$-protein to induced LTP. In contrast, LTD was suppressed by SR140333, an NK1 receptor antagonist. These results indicated that the direction of plasticity was determined by PPR, especially the release probability of GABA, and the LTP/LTD was mediated by these receptors and downstream pathways. COI: NO

### [1P-008]

### Coenzyme $Q_{10}$ supplementation improves age-related reduction of fEPSP in the motor cortex of middle-aged mice.

*Ritsuko Inoue[1], Masami Miura[1,3], Shuichi Yanai[2], Hiroshi Nishimune[1,4] ([1]*Laboratory of Neurobiology of Aging, Tokyo Metropolitan Institute of Gerontology,* [2]*Laboratory of Memory Neuroscience, Tokyo Metropolitan Institute of Gerontology,* [3]*Saitama Central Hospital,* [4]*Department of Applied Biological Science, Tokyo University of Agriculture and Technology*)

Physiological aging causes motor function decline and anatomical and biochemical changes in the motor cortex. Middle-aged mice at 15 to 18 months old show motor function and brain mitochondrial function declines, which can be restored to the young adult level by supplementing with mitochondrial electron transporter coenzyme $Q_{10}$ ($CoQ_{10}$) as a water-soluble nanoformula by drinking water for one week. We previously reported that field excitatory postsynaptic potential (fEPSP) amplitudes in the primary motor area of middle-aged mice show an age-related decline, which was restored to the young adult level by supplementing middle-aged mice with $CoQ_{10}$. Now, we examined the effects of administering $CoQ_{10}$ acutely to brain slices to seek the mechanism of fEPSP improvement. However, the acute $CoQ_{10}$ administration did not enhance the fEPSP amplitudes in the primary motor area of slices prepared from middle-aged mice. Interestingly, $CoQ_{10}$ administration with high-frequency stimulation induced NMDA receptor-dependent long-term potentiation (LTP) in the primary motor cortex of middle-aged mice. The fEPSP amplitude showed a larger input-output relationship after $CoQ_{10}$-dependent LTP expression. These data suggest that the $CoQ_{10}$ supplementation potentially enhances synaptic plasticity efficacy, resulting in improved basal fEPSP levels of the motor cortex and motor function in middle-aged mice.

### [1P-009]

### Fluorescent visualization of cerebellar LTD induction

*Riku Okawa[1], Shin-ya Kawaguchi[1] ([1]*Graduate School of Science, Kyoto University*)

Long-term depression (LTD) at cerebellar parallel fiber-Purkinje cell (PC) synapses has been extensively studied as a fundamental phenomenon of motor learning in animals. Molecular mechanisms of LTD induction have been well clarified, and it is thought that a positive feedback loop including protein kinase C (PKC) and MAPK cascades is important for LTD establishment. Activated PKC upon a strong $Ca^{2+}$ increase is known to translocate to plasma membrane, but previous studies showed that PKC exhibits rapid returning to the cytoplasm as $Ca^{2+}$ decays, precluding the sustained activity of the positive feedback loop. To examine how and where the positive feedback loop operates in a PC, we have developed a fluorescent probe protein consisting of mutated PKCα and venus. We succeeded to detect sustained activation of the positive feedback loop for a few hours as accumulation of the probe at the LTD-induced sites in living cultured PCs. In addition, we used this probe together with fluorescent $Ca^{2+}$ imaging and electrophysiological recording in a PC. Here, we are going to demonstrate a relationship between the positive feedback loop activation and the local $Ca^{2+}$ increase during LTD induction.

### [1P-010]

### Synaptic and intrinsic plasticity mechanisms for peripheral nerve injury-induced reorganization of input and output functions in the somatosensory thalamus

*Yoshifumi Ueta[1], Mariko Miyata[1] ([1]*Dept Physiol, Div Neurophysiol, Sch Med, Tokyo Women's Med Univ*)

The somatosensory thalamus undergoes structural and functional reorganization after peripheral nerve injury. We previously report that the infraorbital nerve cut remodels whisker-specific topography of afferent axons from the brainstem in the mouse thalamic ventral posteromedial nucleus (VPM) by newly recruiting ectopic non-whisker-derived afferent synapses in the VPM whisker region. Our previous reports also indicate that aberrant increase of GABAergic tonic inhibition in VPM and microglial activation in the brainstem both regulate this synaptic remodeling. Thalamic reorganization associates with ectopic mechanical hypersensitivity, an allodynia-like response. Thus, peripheral nerve injury may recruit ectopic inputs relaying non-whisker tactile information to the VPM whisker region. VPM activity also undergoes plastic changes under chronic pain conditions. Nerve injury changes intrinsic properties of thalamocortical neurons to facilitate burst activity, which is mediated by tonic inhibition. Furthermore, microglial ablation prevents nerve injury-induced enhancement of tonic inhibition and tonic inhibition-mediated changes in intrinsic properties. Thus, a common mechanism may regulate plastic changes in VPM input/output functions to alter ascending somatosensory processing after peripheral nerve injury.

### Poster Presentation

[1P]
### Neurophysiology, Neuronal cell biology
**Neural network**

March 14 (Tue.), 12:10 - 14:10, Poster Room

### [1P-011]

**Perioral sensory signaling pathways to the cerebellum via the mesencephalic and pontine regions**

*Reika Kubo[1], Takayuki Yoshida[1], Kouichi Hashimoto[1] ([1]*Hiroshima University*)

Perioral sensory signals are transmitted to the cerebellar Purkinje cells (PCs) via the mossy fibers emerging from the pontine nuclei, modulating simple spike (SS) activities of the PCs. To find the signaling pathways from the perioral area to the PCs to control the SS generations, we recorded SSs from the PCs in CrusII evoked by electrical stimulations of the infraorbital nerve (ION) in anaesthetized mice. The ION stimulations enhanced SS firing rate around 5-15 ms (1st SS peak) and 20-33 ms (2nd SS peak), which was followed by SS silence. We found that the 2nd SS peak and the SS silence were suppressed by muscimol, a $GABA_A$ receptor agonist, injections into the pontine gray (PG). They were also inhibited by muscimol injections into the mesencephalic reticular nucleus (MRN) or the pontine reticular nucleus caudal part (PRNc). Injections of a retrograde tracer, Fluoro-Gold, into the PG suggested projections from the MRN to the PG. These data suggest that the 2nd SS peak and the SS silence are relayed at the MRN and thereafter sent to PCs via the PG.

### [1P-012]

**The mPFC suppresses the value of reward under the risk of overt punishment**

*Monami Nishio[1], Masashi Kondo[1], Yoshida Eriko[1], Masanori Matsuzaki[1] ([1]*The University of Tokyo*)

The medial prefrontal cortex (mPFC) is important for the avoidance of punishment. However, neither whether the role of mPFC depends on the type of punishment nor which intrinsic variable is regulated by mPFC has been determined. Therefore, the present study compared the reward-seeking behavior of mice under the risks of two types of punishment: overt punishment (airpuff) and covert punishment (reward omission). In the first, airpuff task, mice that pulled a lever in response to tones A and B received airpuff punishment at probabilities of 90% and 10%, respectively. In both tone trials, a water reward was delivered at a probability of 100%. In the second, omission task, another group of mice that pulled a lever in response to tones A and B received water reward at probabilities of 10% and 90%, respectively. Airpuffs were not delivered in response to either tone. After the 2 weeks of training, mice in both groups learned to suppress lever pulling in response to toneA. However, when GABA-A receptor agonist was applied to mPFC, the lever-pull rate was increased in trials with a high probability of overt punishment, but not in the case of covert punishment. The animal's choice was simulated with reinforcement learning models and the effect of mPFC inhibition was most well-explained as the increase of the subjective value of water reward. These findings suggest that the mPFC controls reward-seeking behavior by suppressing the value of the reward specifically under the risk of overt punishment.

### [1P-013]

**Neural mechanisms of retrospective fear; revisiting James-Lange theory of emotion with optogenetics**

*Kaoru Isa[1], Thongchai Sooksawate[1,2], Kota Tokuoka[1,3,4], Sara Karimi[1,5], Sakura Hiramatsu[1], Kenta Kobayashi[6], Tadashi Isa[1] ([1]*Kyoto University,* [2]*Fac. Pharmaceutical Sci., Chulalongkorn University,,* [3]*DANDRITE – Danish Research Institute of Translational Neuroscience, Nordic EMBL Partnership for Molecular Medicine, Department of Biomedicine, Aarhus University,* [4]*National Institute of Genetics,* [5]*Kashan University of Medical Sciences,* [6]*National Institute for Physiological Sciences*)

Classical James-Lange theory of emotion proposed that emotions occur as a result of physiological reactions to events, that is, the retrospective interpretation of physical responses to environmental stimuli results in an emotional experience. This theory is so fascinating, however, it has been difficult to prove. Here, in mice , we selectively activate the motor command for innate fear responses originating from the motor layers of superior colliculus and demonstrate that it forms the memory of the fearful experience in the passive avoidance paradigm through its efference copy pathway to the amygdala via the posterior thalamic nucleus triangular.
We propose that such immediate activation of the amygdala by the efference copy of motor commands underlies the induction of retrospective fear which was suggested by the James-Lange theory of emotion.

### [1P-014]

**Indirect pathway from the rat interstitial nucleus of Cajal to the vestibulo-cerebellum**

*Taketoshi Sugimura[1], Yasuhiko Saito[1] ([1]*Nara Medical University*)

Gaze holding is primarily controlled by the oculomotor neural integrators, which are separated into the prepositus hypoglossi nucleus (PHN) for horizontal gaze and the interstitial nucleus of Cajal (INC) for vertical gaze. Although neural connections between the neural integrators and the vestibulo-cerebellum (VC) are suggested to be significant for gaze holding, the neural pathway from the INC to the VC have not been revealed, contrary to the pathway from the PHN to the VC. In the present study, we aimed to obtain the anatomical evidence of the INC-VC pathway. When we injected a dextran-conjugated Alexa 488 into the cerebellar flocculus or uvula/nodulus of rats, the retrogradely labeled neurons were not observed in the INC. This result indicates that the INC does not project directly to the VC. Next, we used rabies virus-based trans-synaptic tracing to examine indirect INC-VC pathway. Starter neurons which project to the VC were observed in the PHN and medial vestibular nucleus (MVN). Fluorescent labeled neurons, which received a retrograde trans-synaptic infection with rabies virus from the starter cells, were observed in both sides of the INC. All these results strongly suggest that connections from the INC to the VC occur indirectly via the PHN and MVN.

### [1P-015]

**Diverse alterations of sensory cell activity in the thalamic reticular nucleus by prefrontal cortex activation**

*Akihisa Kimura[1] ([1]*Department of Physiology, Wakayama Medical University*)

Thalamic reticular nucleus (TRN), a GABAergic nucleus, regulates interactive neural processing in the loop circuitry between the cortex and thalamus. Higher brain functions influence this regulation from the prefrontal cortex (PFC). The present study examined the effects of medial PFC activation (electrical micro-stimulation) on auditory or visual cell activity in the TRN of anesthetized rats, using juxta-cellular recording and labeling techniques. Cells were labeled with neurobiotin, and their locations and targets of axonal projection were histologically verified. PFC activation alone did not evoke cell activity, but it altered sensory response in the majority of auditory (39/43) and visual cells (19/20) projecting to the first- and/or higher-order thalamic nuclei. Modulation took place in early (onset) and late responses. Response magnitude modulation was bi-directional, i.e., facilitation and suppression, including induction of de novo cell activity. Modulation also took place in response latency and burst spiking. Facilitation was more frequently observed than that in the intra- or cross-modal sensory interplay in the TRN. The results suggest that the top-down (PFC activation) and bottom-up (sensory interplay) effects interact in the TRN to flexibly modulate sensory attention and perception depending on internal demands and sensory signals from the environment.

### [1P-016]

### Parvalbumin interneuron circuits mediate functional recovery in post-stroke rehabilitation

Naohiko Okabe[1], *Thomas Carmichael[1] ([1]*Department of Neurology, David Geffen School of Medicine at UCLA*)

The damaged brain modifies its neural network to restore function in response to environmental demands. Rehabilitation in clinical practice employs the modification of neural networks. However, it is unclear which neural circuits in the brain are associated with functional recovery. This study aimed to identify neuronal circuits responsible for experience-dependent recovery after stroke. We investigated synaptic alterations in the rostral forelimb area (RFA) after photothrombotic stroke in the caudal forelimb area (CFA) in the mouse motor cortex using multiple virus labeling approaches (dendritic spine analysis, rabies virus tracing, and GRASP assay). The mice received intensive skilled reaching training (>800 reaches/day, 5 days/week, 3 weeks) from ten days after the stroke. To investigate the involvement of modified neuronal circuits in functional recovery, we also performed acute and chronic chemogenetic studies combined with behavioral assessment by single seed reaching test and grid walk test. Our histological studies revealed that the CFA stroke dramatically reduced dendritic spine density in the neurons projecting to the stroke site (stroke-projecting neurons) from the RFA. The stroke-projecting neurons lost synaptic connections from many brain areas apart from the stroke site, such as the somatosensory cortex, thalamus, and contralesional RFA. These alterations did not occur in the corticospinal neurons in the RFA. Rehabilitative training restored dendritic spine density and part of the synaptic connections. Furthermore, using the rabies virus tracing and the GRASP assay, we identified that the rehabilitative training increased local synaptic inputs from the parvalbumin interneurons (PV-INs) to the stroke-projecting neurons. The acute chemogenetic studies revealed that inhibition of the stroke-projecting neurons disturbed motor function after rehabilitation, suggesting the involvement of stroke-projecting neurons in functional recovery. Furthermore, chronic inhibition of either stroke-projecting neurons or PV-INs blocked functional recovery during rehabilitative training. These data suggest that neuronal activities in the stroke-projecting neurons and the PV-IN are necessary for functional recovery. Our results suggest that the neuronal circuits formed by the stroke-projecting neurons and PV-INs may play a critical role in the experience-dependent functional recovery after stroke.

### [1P-017]

### Novel cranial window for in vivo calcium imaging

*Satoshi Manita[1], Shigetomi Eiji[2,3], Haruhiko Bito[4], Schuichi Koizumi[2,3], Kazuo Kitamura[1] ([1]*Department of Neurophysiology, Division of Medicine, University of Yamanashi,* [2]*Department of Neuropharmacology, Faculty of Medicine, University of Yamanashi,* [3]*Yamanashi GLIA center, Interdisciplinary Graduate School of Medicine, University of Yamanashi,* [4]*Department of Neurochemistry, Graduate School of Medicine, The University of Tokyo*)

We have developed a new cranial window that allows two-photon and wide-field calcium imaging in the same mouse. The cranial window is made of polyvinylidene chloride (PVDC) wrapping film, a transparent silicone plug, and a cover glass, and can be made as large as the size of the hemisphere of the mouse cerebral cortex (approximately 6 x 3 mm). Wide-field calcium imaging was performed using mice that express calcium sensors in neurons or glial cells. In each mouse, the cortical activity induced by the sensory stimulation of the whiskers could be observed to propagate throughout the cortex. Two-photon imaging could also be performed and fluorescence signals specific to single neurons and glial cells were observed through the same window. This cranial window also allows two-photon imaging at different locations within the window. To determine the degree of vibration artifact under the new cranial window, we measured the position of small fluorescent particles in the brain, and found that the standard deviation of the centroid of the fluorescent particles was about 0.3 µm, comparable to that under the conventional glass window. We didn't observe any signs of infection, bleeding, or regrowth more than one month after surgery. In addition, we have successfully expressed genetically-encoded calcium indicator in cortical neurons by applying thin films of adeno-associated virus onto PVDC films. This technique for making a large cranial window is reliable and cost-effective and facilitates the investigation of the neural and glial dynamics and their interactions during behavior at the macroscopic and microscopic levels.

### [1P-018]

### Development of a compact and strong inhibitory neuron-specific promoter available for adeno-associated virus vectors

*Yuuki Fukai[1], Ayumu Konno[1,2], Yasunori Matsuzaki[1,2], Hirokazu Hirai[1,2] ([1]*Department of Neurophysiology and Neural Repair, Gunma University Graduate School of Medicine,* [2]*Viral Vector Core, Gunma University Initiative for Advanced Research (GIAR)*)

[Purpose] Recently, we published an inhibitory neuron-specific promoter (mGAD65 promoter) (Hoshino et al. *Mol Brain*. 2021). However, the mGAD65 promoter has 2.5 kb in length and occupies over a half of the accommodation space of adeno-associated virus (AAV) vector, which substantially compromises the usefulness of the mGAD65 promoter. Here, we aimed to develop a compact inhibitory neuron-specific promoter.[Methods] We prepared deletion constructs from the mGAD65 promoter and similar genomic regions upstream of the mouse GAD67 gene. AAV-PHP.eB expressing GFP by one of the mGAD65 or mGAD67 promoter candidates was intravenously administered to VGAT-tdTomato mice, which expressed tdTomato specifically in GABAergic neurons. Three weeks after the injection, transduced cell types and expression levels of GFP in the cerebral cortex were assessed by immunohistochemistry and quantitative RT-PCR, respectively. [Result and Discussion] The 0.4-kb mGAD67 promoter (named as compact mGAD67 promoter; cmGAD67 promoter) showed significantly higher promoter activity than the original (2.5-kb) mGAD65 promoter without compromising GABAergic neuron specificity. Thus, the cmGAD67 promoter allows larger transgene accommodation in AAV and is useful for studying the pathogenesis of inhibitory GABAergic neurons.

### [1P-019]

### Chemogenetic strategies to investigate the role of projections from the insular cortex to the trigeminal spinal subnucleus caudalis in pain-related behaviors of rats

*Yuka Nakaya[1], Kiyofumi Yamamoto[1], Masayuki Kobayashi[1] ([1]*Department of Pharmacology Nihon University School of Dentistry*)

Orofacial noxious information is transmitted to the insular cortex (IC) via the trigeminal spinal subnucleus caudalis (Sp5C). The IC plays a major role in processing nociception, and direct descending projections from IC to Sp5C have been reported. However, it remains unclear whether this descending projection regulates pain behavior. Here, we examined whether IC projections modulate pain behaviors in rats using a chemogenetic technique. Behavior tests were performed by using VGAT-Venus transgenic rats that received AAV-hSyn-hM3D(Gq)-mCherry injection into IC. We injected CNO via the cannula implanted in the cisterna magna of the rats. The head withdrawal threshold (HWT) to mechanical stimulation of the whisker pads using von Frey hairs significantly decreased 2.5 and 3 h after CNO injection. The HWT to radiant heat stimulation of the whisker pads also decreased 3 h after CNO injection. These results suggest that excitatory inputs from the IC to the Sp5C decrease the threshold of nociception induced by mechanical and heat stimulation of the peripheral region innervated by the trigeminal nerve.No COI.

### [1P-020]

### Upstream regions underlying central amygdala activation in inflammatory pain

*Yukari Takahashi[1], Takao Okuda[1], Sawako Uchiyama[1,2], Fusao Kato[1] ([1]*Jikei University School of Medicine,* [2]*Kyusyu University*)

The central nucleus of the amygdala (CeA) is activated in various types of pain animal models. The spino(trigemino)-parabrachio-amygdaloid pathway is the major ascending pain pathway conveying nociceptive information to the CeA. However, in various pain models, neurons expressing c-Fos in the CeA can be found outside of the regions without termination of excitatory fibers arising from the lateral parabrachial nucleus (LPB) (Miyazawa et al., 2018). To identify the origins of excitatory inputs underlying this pain-associated activation in the CeA, we used a cre-reporter mouse line and retrograde AAV to visualize the connections from inflammation-activated cells to those in the CeA in formalin-induced orofacial inflammation model. The pain-activated cells with direct projections to the pain-activated CeA were distributed in regions including the paraventricular thalamus, subthalamic nucleus, LPB, and insular cortex. These results indicate that transient activation of pain/inflammation-associated neurons widely distributed in various brain areas contribute to establishing "nociplastic" activation of the CeA network, which might underlie the widespread sensitization.

### [1P-021]

### The analysis of protein-interaction of RNF39 for dendritic spine formation

*Yumi Nakagaki[1], Shinichirou Suzuki[1], Ikuko Yao[1], Michinori Toriyama[1] ([1]*Biomedical Chemistry major, Graduate School of Science and Technology, Kwansei Gakuin University*)

Docosahexaenoic acid (DHA) is an essential fatty acid involving in the brain development and functions. Accumulated studies demonstrated that DHA positively regulates neural circuit formation, including synapse formation. However, its molecular mechanism in neural circuit formation is poorly understood. Recently, we identified RNF39 (Ring Finger Protein 39), an E3 ubiquitin ligase, as an up-regulated gene upon DHA stimulation in cultured neurons. Over-expression of RNF39 in cultured neurons increased dendritic spine number. These results indicated that DHA enhances dendritic spine formation via up-regulation of RNF39 expression. To clarify the molecular functions of RNF39, we screened the interacting proteins of RNF39 by immunoprecipitation followed by mass spectrometry. MDM2, p53, IRS4, and WDR62 were identified by immunoprecipitation. First, we examined the interaction between RNF39 and MDM2. *In vitro* binding assay showed that RNF39 directory is bound to MDM2 via RING domain in RNF39. Also, we found MDM2 protein levels were reduced in RNF39 overexpressed cells. However, the reduction of the RNF39 protein level was not observed in MDM2 overexpressed cells. These results exhibited that RNF39 interacts and ubiquitinates MDM2 for protein degradation. Second, we addressed the interaction between RNF39 and IRS4. Co-immunoprecipitation assay showed IRS4 interacts with RNF39 via its RING domain. Also, a significant reduction of the protein level of IRS4 was observed in RNF39 overexpressed cells. These results indicated that RNF39 is involved in dendritic spine formation via protein degradation of MDM2 or IRF4.

### [1P-022]

### A medium-scale spiking neural network model of rodent cerebral cortex-basal ganglia-cerebellar circuit that can be run in a lab desktop environment

*Hinako Yamamoto[1], Jun Igarashi[2], Yoshikazu Isomura[1], Riichiro Hira[1] (*[1]Tokyo Medical and Dental University, [2]RIKEN*)

State-of-the-art supercomputers and the open-source connectome database for rodent brains have made it possible to simulate realistic-sized brains with actual circuit topology. However, giant supercomputers are not practical for routine analyses, such as condition searches to reproduce experimental data obtained in individual laboratories. Here, we constructed neural circuits for the cerebral cortex, basal ganglia, and cerebellum using realistic neuron numbers, firing characteristics, and connection probabilities (number of neurons > 100,000, number of synapses >50,000,000). We created a spiking neural circuit with the Izhikevich model in the python-based Brian2 simulator. The results showed that it takes only 15 minutes to simulate 3 seconds of brain activity with a time step of 0.1 ms in a normal desktop environment. Starting from the parameters obtained from the connectome data, we tuned the parameters based on our own data recorded from cerebellum and SNr when neurons in the cerebral cortex were stimulated. As a result, we successfully determined circuit parameters that reproduced the experimental data simultaneously retaining the consistency to the connectome data. Thus, we demonstrated that the medium-scale simulation environment, which does not require special computational resources, works as an important tool to link experimental data with large-scale simulations using a supercomputer.

### [1P-023]

### Bidirectional GABAergic and cholinergic modulation of delta-band activities in a hippocampal-like circuit *in silico*

*Kota Itagaki[1], Masabumi Minami[2], Yuichi Takeuchi[2] (*[1]Hokkaido University, School of pharmaceutical sciences, [2]Hokkaido University, Faculty of pharmaceutical sciences*)

### Poster Presentation

[1P]
**Neurophysiology, Neuronal cell biology**
**Neurons, Synapses**

March 14 (Tue.), 12:10 - 14:10, Poster Room

### [1P-024]

**Taurine depletion during fetal and postnatal development blunts firing responses of neocortical layer II/III pyramidal neurons.**

Yasushi Hosoi[3,2], *Tenpei Akita[1,2], Miho Watanabe[2], Takashi Ito[4], Hiroaki Miyajima[3], Atsuo Fukuda[2] ([1]*Division of Health Science, Department of Basic Nursing, Hamamatsu University School of Medicine,* [2]*Department of Neurophysiology, Hamamatsu University School of Medicine,* [3]*First Department of Medicine, Hamamatsu University School of Medicine,* [4]*Department of Biosciences and Biotechnology, Fukui Prefectural University*)

Fetal and infant brains are rich in taurine of maternal origin. Here, we used whole-cell patch-clamp analysis to examine the effects of fetal and infant taurine depletion by knockout of the taurine transporter Slc6a6 on the firing properties of layer II/III pyramidal neurons in the mouse somatosensory cortex at 3 weeks postnatal age. Membrane excitability at rest was comparable between neurons from knockout mice and wild-type littermates. However, neurons from knockout mice showed a marked decrease in the repetitive firing frequency of action potential spikes during moderate current injection, as well as a decrease in the level of membrane voltage between spikes. In addition, when a strong current was injected, by which repetitive firing rapidly disappeared in wild-type neurons due to inactivation of voltage-gated $Na^+$ channels, firing persisted much longer in the knockout neurons than in wild-type neurons. This was due to much lower membrane voltage levels between spikes in the knockout neurons, accelerating faster recovery of voltage-gated $Na^+$ channel from inactivation. Thus, taurine deficiency in pyramidal cells blunted neuronal responses to external stimuli through increased stability of repetitive firing, presumably via larger increases in membrane $K^+$ conductance during interspike intervals.

### [1P-025]

**Optical recording of spontaneous oscillatory activity in the absence of external $Ca^{2+}$ observed in the embryonic chick olfactory bulb**

*Katsushige Sato[1], Yoko Momose-Sato[2] ([1]*Department of Health and Nutrition Sciences, Faculty of Human Health, Komazawa Women's University,* [2]*Department of Nutrition and Dietetics, College of Nutrition, Kanto Gakuin University*)

Using 464/1020-site optical recording systems with a voltage-sensitive dye (NK2761, a merocyanine-rhodanine dye), we have pursued functiogenesis of the embryonic central nervous system. In our previous studies focused on the embryonic chick olfactory system, we revealed that the glutamatergic synaptic connections between the olfactory nerve (N.I) and olfactory bulb (OB) were generated from the embryonic 6-7 day (E6-E7) stage, and (2) oscillatory activity was evoked in the E9-E12 OB by N.I stimulation. We also investigated N.I-related neural circuits in the E8-E12 forebrain and suggested that (3) the N.I-related neural circuit from the periphery to the subpallium functionally matures earlier than that to the pallium during ontogenesis. In the present study, we examined the effects of a removal of $Ca^{2+}$ in the external solution in the E8-E10 N.I-OB-forebrain preparations. In the absence of $Ca^{2+}$, the slow signals corresponding to the glutamatergic EPSPs as well as the oscillation following the EPSP were completely blocked, whereas spontaneous oscillatory activity newly appeared in the OB. The spatiotemporal patterns of the spontaneous oscillatory activity observed in a $Ca^{2+}$-free solution were different from those of the evoked oscillatory activity detected previously in the normal physiological solution.

### [1P-026]

**Effects of intermittent hypoxia on synaptic activities in pyramidal cells of the somatosensory cortex in developing rats**

*Hiroki Toyoda[1], Atsuko Hayata-Takano[2], Ayano Katagiri[1], Masaharu Yamada[1], Kazuhiro Takuma[2], Takafumi Kato[1] ([1]*Dept. Oral Physiol., Osaka Univ. Grad. Sch. Dent.,* [2]*Dept. Pharmacol., Osaka Univ. Grad. Sch. Dent.*)

Exposure to intermittent hypoxia (IH) in developing rats is associated with an impairment of brain development. In this study, we examined how synaptic activities in layer II/III pyramidal cells of the masticatory cortex in developing rats are affected by IH. Male SD rats were exposed to IH ($O_2$:21%-5%; 6 min cycle, 14:00-20:00) from postnatal day 14 (P14) to P20 or to room air. On P21, Golgi staining and whole-cell patch clamp recordings were performed. Golgi staining revealed that primary dendrite number and total dendrite length were reduced in IH rats compared with the control rats. Whole-cell patch-clamp recordings revealed that the frequency of spontaneous excitatory postsynaptic currents was increased in IH rats compared with the control rats, while that of spontaneous inhibitory postsynaptic currents was not different between control and IH rats. These data suggest that IH during P14 to P20 may cause abnormal synapse formation and enhancement of excitatory synaptic transmission in layer II/III pyramidal cells of the somatosensory cortex in developing rats.

### [1P-027]

**Cholinergic induction of synchronous oscillation in the slug neuronal network *in vitro***

*Suguru Kobayashi[1,2], Futori Riho[1], Sadamoto Hisayo[1] ([1]*Kagawa Schl Pharmaceut Sci, Tokushima Bunri Univ,* [2]*Inst Neurosci, Tokushima Bunri Univ*)

Synchronous oscillatory network is vital for cognitive functions of the brain in both vertebrates and invertebrates. In the central nervous system of the terrestrial slugs, spontaneous periodic slow oscillation (0.5 - 1.0 Hz) is recorded from the surface of the laminar structure of procerebrum (PC), and its frequency changes are suggested to encode the olfactory information and memory. We recently found oscillatory activity is generated spontaneously in dispersed cell culture of PC neurons. Application of acetylcholinesterase inhibitor or nicotine increased the number of spontaneous activities in cultured PC neurons, and furthermore, induced synchronous oscillation in *in vitro* network activity. On the other hand, biogenic amines or neuropeptides often changed the number of spontaneous activities without generating synchronous oscillation on PC neurons. To investigate how such synchronous oscillation is generated, we tested cholinergic activation and compared between synchronous and asynchronous networks. Previous results suggest that acetylcholine could be function as a driving force on the synchronous oscillatory activity of the PC neuron network via nicotinic acetylcholine receptors activation *in vivo*. In present study, differences between synchronous and asynchronous network were examined in cultured PC neuron (7-21 days). First, PC neurons cultured more than 10 days could induce synchronous oscillation *in vitro* by the application of cholinesterase inhibitor physostigmine. It is suggested that synchronous networks included two groups of neurons, (a) acetylcholine-sensitive PC neurons, (b) acetylcholine-insensitive PC neurons that activated or driven by excitatory input from a. Second, we found a lower excitability and a higher sensibility to cholinergic activation of PC neurons in synchronous networks than asynchronous networks. Third, muscarinic receptor agonist, pilocarpine, did not induce synchronous oscillation and did not occlude the effects of physostigmine. These results suggest that: 1) *in vitro* synchronous oscillation was induced by activation of cholinergic synaptic transmission via nicotinic ACh receptors; 2) "synchronous *in vitro* networks" were characterized by higher ACh-sensitivity (than "asynchronous networks") together with the lateral inhibition of excessive activity in resting states. Furthermore, another cholinesterase inhibitor, neostigmine, increased in spontaneous activity of PC neurons like physostigmine, but never induced synchronous oscillation. Difference in effects of physostigmine and neostigmine may be essential for generation of synchronous oscillation.

### [1P-028]

**Widespread EPSPs in dendrites of a cerebellar Purkinje cell**

*Reo Higashi[1], Shin-ya Kawaguchi[1] ([1]*Graduate School of Science, Kyoto University*)

Neuronal dendrites receive a lot of synaptic inputs and integrate them to serve computational functions. How EPSPs travel and summate in dendrites is an important question. However, technical limitation of patch-clamp method in acquiring spatial information and its invasive nature have made it difficult to evaluate them. To study this issue, here we used a genetically encoded voltage sensor, ASAP, and spot uncaging of glutamate on a cultured Purkinje cell, together with mathematical simulation. An illumination of 405 nm laser spot on a dendrite locally generated glutamate, evoking EPSP which was monitored as a decrease of ASAP fluorescence. The amplitude of EPSP decreased substantially around the uncaged site, whereas in distant areas EPSP widely spread throughout the dendritic branches with limited attenuation. To theoretically evaluate this result, we built cable-theory models of individual cells. The widespread nature of EPSPs was reproduced by a simple model assuming only passive electrical membrane property. Taken all these results together, thick, short, and highly branching dendritic topology of a Purkinje cell enables EPSP spreading to broad area of the cell.

### [1P-029]

### The primary source and molecular requirements of somatodendritic dopamine release

*Takuya Hikima[1], Paul Witkovsky[2], Margaret Rice[2,3] ([1]*Div. Memory and Cognitive Function, Dokkyo Medical University,* [2]*Dept. Neurosurgery, NYU School of Medicine,* [3]*Dept. Neuroscience and Physiology, NYU School of Medicine*)

Midbrain dopamine (DA) neurons exhibit somatodendritic release of DA. DA acts at somatodendritic D2 autoreceptors to regulate DA neuron firing patterns that govern DA release throughout the brain. The primary source and mechanism of somatodendritic DA release remain unsolved despite the passing of decades since its discovery. We focused on the two key questions about this process: 1) what is the source of the somatodendritic DA release? and 2) what cellular proteins underlies the unique $Ca^{2+}$ sensitivity of somatodendritic DA release? *We* hypothesized that D2 receptors on a given DA neuron in substantia nigra pars compacta (SNc) are activated primarily by DA released from that same cell, literally *auto*regulation, via exocytosis involving the SNARE protein SNAP-25. We tested this using voltage-clamp recording of SNc DA neurons in acute midbrain slices. Locally evoked D2 DA-dependent inhibitory currents (D2ICs) resulting from G-protein-coupled, inwardly rectifying $K^+$ channels (GIRKs) were used as an index of somatodendritic DA release. Consistent with our hypothesis, single-cell application of botulinum neurotoxin-A (BoNT/A) to cleave SNAP-25 abolished D2ICs by pulse-train evoked D2ICs. In contrast, BoNT/A had no effect on synaptically activated GABA-B receptor GIRK currents. Thus, in SNc, somatodendritic DA release indeed inhibits the neuron that released it. We reported previously that robust release of somatodendritic DA in submillimolar extracellular $Ca^{2+}$ concentration ($[Ca^{2+}]_o$) (Chen et al., 2011). We tested the hypothesis that the high-affinity $Ca^{2+}$ sensor, synaptotagmin 7 (Syt7), is a key player of somatodendritic DA release and its $Ca^{2+}$ dependence. Single-cell application of an antibody to Syt7 (Syt7 Ab) decreased pulse-train evoked D2ICs. In addition, pulse-train evoked D2ICs persisted in low $[Ca^{2+}]_o$. However, this sensitivity to $Ca^{2+}$ was lost when with Syt7 Ab in the pipette and in Syt7 knockout (KO) mice. Notably, interference with Syt7 had no effect on single-pulse evoked DA release,. In contrast, single-cell application of an antibody to Syt1 (Syt1 Ab) had no effect on train-evoked D2ICs in wildtype SNc DA neurons, but decreased D2IC amplitude in Syt7 KOs, indicating a functional substitution of Syt1 for Syt7. Moreover, Syt1 Ab decreased single-pulse evoked D2ICs in WT cells, indicating the involvement of Syt1 in synchronous, tonic DA release. Thus, Syt1 and Syt7 subserve different aspects of the somatodendritic DA release processes.

### [1P-030]

### Effects of unilateral nasal obstruction during the growth period on cerebellar neural circuit and function.

*Moe TANIGAWA[1], Chiho Kato[1], Takashi Ono[1], Naofumi Uesaka[1] ([1]*graduate school of Medical and Dental science*)

There is accumulating evidence that nasal obstruction induces cognitive decline including memory and learning deficits. However, little is known about effects of nasal obstruction during brain development on neural circuit formation and brain function other than memory and learning. In the present study, we examined possible association among nasal obstruction during development, synapse development, and behaviors using the mouse model of nasal obstruction. Male mice were subjected to unilateral nasal obstruction (UNO) by the cauterization to the right nasal at postnatal day 3. We focused on cerebellum as a brain region affected by UNO and analyzed cerebellar neural circuits and behaviors. We examined effects of UNO on synapse elimination in which some synapses are selectively strengthened and the others are eliminated during neural circuit formation. We found that UNO mice showed the impairment of synapse elimination and neural innervation pattern in the cerebellum. We also performed a comprehensive behavioral test battery to assess motor function/learning, repetitive movement, depression, activity and anxiety. UNO mice showed impaired motor function in the Rota-Rod test and a depressive-like behavior in a forced swim test. UNO did not alter activity and an anxiety-related behavior in the Open Field test. Our findings suggest that adequate oxygen inhalation through the nose from early developmental stage is essential for the development of cerebellar circuits, and motor function and mental health.
COI: NO

### [1P-031]

### cAMP modulates membrane excitability controlling action potential conduction in cerebellar Purkinje cells

*Kei Furukawa[1], Shin-ya Kawaguchi[1] ([1]*Graduate School of Science, Kyoto University*)

An action potential (AP) is propagated in an axon to its terminals. Recent studies showed that cAMP modulates the AP conduction velocity in cerebellar parallel and mossy fibers. Here, we studied whether cAMP modulates the AP conduction in an axon of a cerebellar Purkinje cell (PC) in both culture and slice preparation. Primary cerebellar cultures (from P0 rats, and used at ≥26 DIV) and acute slices of cerebellar vermis (from P17-20 rats) were used. Adeno-associated virus (AAV) vector was used (for slice preparation, injected into P2-5 rats' cerebellum) to fluorescently visualize PC axons. We directly recorded a spontaneous AP or that elicited by a current injection to the PC soma in culture or by an axonal electrical stimulation with a glass pipette in slices. Increase in cAMP by forskolin delayed the AP conduction in both culture and slices. Furthermore, we measured membrane properties such as membrane resistances and resting potentials at either a soma or an axon before and after forskolin application, and found cAMP-dependent change of axonal membrane excitability. Additionally, we attempted to identify the cAMP's downstream molecule using pharmacological agents such as KT5720, H89 (protein kinase A inhibitor), and ZD7288 (HCN channel blocker). Here, we show the cAMP's role and mechanism in regulation of APs in PC axons.

### [1P-032]

### New approach of cell types and their networks of red nucleus

*Takae Ohno[1], Satoshi Fukuda[1], Naoyuki Murabe[1], Hideo Miyake[1], Shu Takai[1], Toshihiro Hayashi[1] ([1]*Dept Physiol, Teikyo Univ Sch Med*)

The red nucleus consists of the magnocellular and parvocellular neurons, which have generally been believed to project to the spinal cord and inferior olive, respectively. It is generally believed that there is a progressive segregation between the two systems during their phylogenesis. Although they play prominent roles in different aspects of limb movements in mammals, little is known for their interactions. In the first step, we retrogradely and dually labeled them with a pair of retrograde tracers, CTB-Alexa and AAV2retro-Channel rhodopsin (ChR) 2-fluorescent proteins (FP) from each main target, the inferior olive nucleus and spinal cord. The rubro-olivary (RO) neurons were mostly smaller than the rubrospinal (RS) neurons but unexpectedly, significant proportion of large cells were also included. Moreover, RO and RS neurons were intermingled along with the rostrocaudal axis. Next, we studied their synaptic connections and cell characters by whole cell recordings with optogenetic stimulation after alternately expressing AAV2retro-ChR2-FP and CTB-Alexa 594 in RO and RS neurons respectively. Specific cell characteristics and differences in the synaptic plasticity were found.

### [1P-033]

### Chloride-dependent modulation of excitatory synaptic inputs in hippocampal neuron

*Masato Morita[1], Shin-ya Kawaguchi[1] ([1]*Graduate School of Science, Kyoto University*)

How do membrane potential changes propagate spatially in dendrites of hippocampal neurons? To study this issue, we used a genetically encoded voltage indicator, called ASAP, combined with spot uncaging of glutamate or GABA on a local area of dendrite. We previously found that excitatory synaptic inputs were oppositely modulated in a dendrite of hippocampal neuron depending on the direction of propagation. Here, we examined the mechanism of that modulation by direct patch-clamp recordings from a thin dendrite. Surprisingly, resting membrane potentials were more negative in dendrites than in the soma, which was abolished by inhibition of $Cl^-$ export by KCC2 that contributes to low level of intracellular chloride ($[Cl^-]_{in}$). Thus, it was suggested that the lower $[Cl^-]_{in}$ underlies more negative resting potential in the dendrite. Moreover, direction-dependent augmentation of excitatory synaptic inputs in a dendritic tree disappeared by the disturbance of low $[Cl^-]_{in}$ by KCC2 inhibition. Taken all these results together, it was suggested that very low $[Cl^-]_{in}$ in a dendrite impacts local computation of synaptic inputs in hippocampal neurons.

### [1P-034]

### $Ca^{2+}$ mobilization by activation of metabotropic glutamate receptor 1 in the hippocampal marginal zone.

*Megumi Taketo[1] ([1]*Kansai Medical Univ.*)

In the central nervous system, G-protein-coupled metabotropic glutamate receptors (mGluRs) participate in the regulation of cell excitability and synaptic plasticity. Group I mGluRs which consist of mGluR1 and mGluR5, mainly couple to $G_{q/11}$ proteins and increase intracellular $Ca^{2+}$ concentration ($[Ca^{2+}]_i$). The receptors also regulate several channels and other signaling proteins. In the hippocampal marginal zone, Cajal-Retzius (CR) cells control the radial migration of neurons by production and secretion of the glycoprotein, reelin. CR cells also project their dendrites to other neurons and modulate network activity. In a previous study, it was demonstrated that mGluR1 is expressed by hippocampal CR cells, however, the role of mGluR1 in CR cells has not yet been elucidated. In this study, fluorescence imaging revealed that selective activation of mGluR1 induced $[Ca^{2+}]_i$ elevation in CR cells. The $[Ca^{2+}]_i$ elevation was insensitive to several $Ca^{2+}$ channel blockers, and was prevented by depletion of intracellular $Ca^{2+}$ store. Possible interactions between mGluR1 and other G-protein-coupled receptors were also elucidated.

### [1P-035]

### Long-term exposure to high glucose induces changes in the expression of AMPA receptor subunits and glutamate transmission in primary cultured cortical neurons

*Sachie Hamada[1,2], Emi Sanai[2], Mariko Kanemaru[2], Gaku Kamanaka[2], Jun-Ichiro Oka[2]
([1]*Kitasato Univ.*, [2]*Tokyo Univ. of Science*)

Hyperglycemia, which occurs under the diabetic conditions, induces serious diabetic complications. Diabetic encephalopathy has been defined as one of the major complications of diabetes, and is characterized by neurochemical and neurodegenerative changes. However, little is known about the effect of long-term exposure to high glucose on neuronal cells. In the present study, we showed that exposure to glutamate (100 mM) for 7days induced toxicity in primary cortical neurons using the MTT assay. Additionally, high glucose increased the sensitivity of AMPA- or NMDA-induced neurotoxicity, and decreased extracellular glutamate levels in primary cortical neurons. In Western blot analyses, the protein levels of the GluA1 and GluA2 subunits of the AMPA receptor as well as synaptophysin in neurons treated with high glucose were significantly increased compared with the control (25 mM glucose). Therefore, long-term exposure to high glucose induced neuronal death through the disruption of glutamate homeostasis.

### [1P-036]

### The effect of β3 subunit phosphorylation on trafficking and function of GABAA receptor.

*Aogi Kobayashi[1], Moeka Fujii[1], Tomonori Furukawa[2], Shuji Shimoyama[2], Shinya Ueno[2] ([1]*Department of Neurophysiology, Hirosaki University School of Medicine*, [2]*Department of Neurophysiology, Hirosaki University Graduate School of Medicine*)

GABAA receptor (GABAAR) is generally composed of two α, two β, and one γ subunits. The β3 subunit contains phosphorylation sites at serine 408 and 409 (S408/9). The phosphorylation state of these sites affects receptor function and plays an important role in receptor trafficking, but the detailed mechanism has not yet been explored. To assess the role of phosphorylation state of S408/9, we established stable Neuro2A cell lines with mutant β3, wild type α1 and γ2 subunits. The S408/9 of the β3 subunit were mutated into alanine (S408/9A) or glutamic acid (S408/9E) to mimic dephosphorylated or phosphorylated state. These established Neuro2A cells were analyzed electrophysiologically and immunohistochemically. ln electrophysiological analysis, the wild type and the S408/9A mutant containing GABAAR showed response to GABA in a dose-dependent manner, and the response was enhanced by diazepam and propofol applications. However, there was no response in the S408/9E mutant. In immunohistochemical analysis, the S408/9E mutant β3 subunit was not localized to cell membrane. These results suggest that the phosphorylation state in S408/9 of β3 subunit reduced receptor trafficking to the cell membrane in Neuro2A cell.

### Poster Presentation

[1P]
### Neurophysiology, Neuronal cell biology
### Higher brain function

March 14 (Tue.), 12:10 - 14:10, Poster Room

### [1P-037]

**Comprehensive behavioral analysis of C57BL/6.KOR-ApoE$^{shl}$ Mice**

*Hiroshi Ueno[1], Yu Takahashi[2], Okamoto Motoi[3], Takeshi Ishihara[2] ([1]*KawasakiUniversity of Medical Welfare,* [2]*Kawasaki Medical School,* [3]*Okayama University*)

Apolipoprotein E (ApoE) is a protein with a molecular weight of 34,200 Da that consists of 299 amino acids. In addition to lipid metabolism, ApoE and ApoE isoforms are involved in the maintenance of normal brain function. ApoE is associated with Alzheimer's disease (AD) and cognitive dysfunction in elderly individuals. Worldwide, studies have used ApoE-deficient mice as an AD model. ApoE-deficient mice are commercially available as B6.129P2-Apoe$^{tm1Unc}$ mice and are homozygous to the mutant Apo-E$^{tm1Unc}$. There have been studies on behavioral abnormalities in ApoE-deficient mice. ApoE-deficient mice show reduced contact with the herd during sleep and reduced motor activity in new environments as well as learning and memory deficits. On the other hand, Matsushima et al. have discovered spontaneously hyperlipidemic mice (SHL mice) in the process of producing from wild mice to inbred mice. This mouse is a naturally mutated mouse. Spontaneous hyperlipidemia (Apoe$^{shl}$) mice have been shown to develop due to apolipoprotein E deficiency due to mutations in the apolipoprotein E gene. However, behavioral and central nervous system abnormalities in Apoe$^{shl}$ mice remain unclear. Accordingly, we aimed to investigate the behavioral abnormalities of Apoe$^{shl}$ mice. Apoe$^{shl}$ mice showed decreased motor skill learning and increased anxiety-like behavior toward heights. Our findings demonstrated that Apoe$^{shl}$ mice are useful as a model for investigating ApoE function in hyperlipidemia and in the central nervous system.

### [1P-038]

**Representation of multi-sensory information in hippocampal CA1 neurons**

*Yuki Takemoto[1], Kumiko Kawabuchi[2], Susumu Setogawa[1,3], Chikako Kamiyoshihara[1], Mikiko Ishikawa[1], Yoshito Saitoh[1], Makoto Osanai[2], Noriaki Ohkawa[1] ([1]*Dokkyo Medical Univ.,* [2]*Osaka Univ.,* [3]*Osaka Metropolitan Univ.*)

Hippocampus is a vital brain region to form episodic memories which are composed of sensory information sensed by novel experiences. It has not been elucidated whether the hippocampus represents and integrates multi-sensory information for constructing one episodic memory. Moreover, although a brain has two hippocampi bilaterally, it is unknown that if there are any differences in feature of processing sensory information between the two hippocampi. Currently, $Ca^{2+}$imaging using miniature fluorescence microscope is prevalent, however it is difficult to apply microscopes to cover both sides of CA1 due to space limitation on the skull of experimental animals, especially rodents. In this study, we have developed an imaging fiber-mediated $Ca^{2+}$imaging technique to allow measuring both sides of CA1 simultaneously. By this technique, from both side CA1, we recorded neuronal activities evoked by sensory stimuli including sound, light, tactile, and odor. Over a half of recorded cells showed sensory response. Moreover, we confirmed existence of some neurons which represented multimodal sensory information. There was no difference in feature of responding to sensory stimulations between left and right CA1. These data suggest that the CA1 neurons function to integrate multi-sensory information.

### [1P-039]

**Respiration-timing-dependent modulation of neural substrates during cognitive processes**

*Nozomu Nakamura[1], Masaki Fukunaga[2], Tetsuya Yamamoto[2], Norihiro Sadato[2], Yoshitaka Oku[1] ([1]*Department of Physiology, Hyogo Medical University,* [2]*Division of Cerebral Integration, Department of System Neuroscience, National Institute of Physiological Sciences*)

We previously showed that cognitive performance declines when the retrieval process spans an expiratory-to-inspiratory (EI) phase transition (an onset of inspiration). To identify the neural underpinning of this phenomenon, we conducted functional magnetic resonance imaging while participants performed a delayed matching-to-sample (DMTS) recognition memory task with a short delay. Respiration during the task was monitored using a nasal cannula. Behavioral data replicated the decline in memory performance specific to the EI transition during the retrieval process, while an extensive array of frontoparietal regions were activated during the encoding, delay, and retrieval processes of the task. Within these regions, when the retrieval process spanned the EI transition, activation was reduced in the anterior cluster of the right temporoparietal junction (TPJa, compared to cases when the retrieval process spanned the inspiratory-to-expiratory phase transition) and the left and right middle frontal gyrus, dorsomedial prefrontal cortex, and somatosensory areas (compared to cases when the retrieval process did not span any phase transition). Such EI transition-specific changes in activation were greater than any changes during the encoding process or the delay period. These results in task-related activity may represent respiratory interference specific in information manipulation rather than memory storage. These findings demonstrate a cortical-level effect of respiratory phases on cognitive processes and highlight the importance of the timing of breathing for successful performance.

### [1P-040]

**Neural manifold represents diverse spatial information in the subiculum**

*Shinya Nakai[1,2], Takuma Kitanishi[1,3], Kenji Mizuseki[1,2] ([1]*Osaka City Univ.,* [2]*Osaka Metropolitan Univ.,* [3]*The Univ. of Tokyo*)

The subiculum (SUB), which receives significant input from the hippocampal CA1 area, is crucial for spatial navigation. The activity of CA1 single neurons is correlated with variables associated with spatial navigation (position, speed, and trajectory), and CA1 population activity creates a low-dimensional neural manifold embedded in a high-dimensional space. Individual SUB neurons also represent spatial information such as position, speed, and trajectory, but their coding approach differs from that of CA1 neurons. Thus, the geometry of SUB population activity is unknown. To address this question, we analyzed the CA1 and SUB neuronal activity data recorded from rats performing the spatial task (Kitanishi et al., 2021). We found that the SUB population activity generated a low-dimensional neural manifold that was homeomorphic to the surrounding space and that the manifold contained a variety of spatial information. According to regression-based decoding analysis, the SUB neural manifold represented diverse spatial information, including position, speed, and trajectory. Additionally, the structures of the SUB neural manifolds remained consistent across rats and tasks. These results indicate that the low-dimensional neural manifold formed by the SUB's neuronal population underlies the representation of various spatial information.

### [1P-041]

**Analysis of frontal and accumbal dopamine release dynamics in mice during reward prediction task**

*Yuma Matsumoto[1,2], Takaaki Ozawa[1,2], Yoshinobu Oyama[1,2], Ryotaro Iwamoto[1,2], Tomohiro Shibata[1,2], Tom Macpherson[1,2], Takatoshi Hikida[1,2] ([1]*Osaka University, Institute for protein research,* [2]*Osaka University, Department of biological science*)

In animals, an adaptive prediction of future reward (e.g., food) by using various environmental cues is an essential ability for survival. The dopamine is the one of key neuromodulators playing important roles in the reward prediction. Dopamine neurons in the ventral tegmental area (VTA), a part of the midbrain, project mainly to the nucleus accumbens (NAc) while other VTA dopamine neurons send sparse but significant projections to the medial prefrontal cortex (mPFC). Although the dopamine release in NAc has been considered to function as a teaching signal which facilitates reward-prediction learning, its role in the mPFC remains largely unknown.
In this study, we analyzed dopamine release dynamics in both mPFC and NAc during mice's performing pavlovian conditioning task.
In conditioning, two types of sound stimuli (CS-High or CS-Low) were paired with food reward (sweetened milk (US)) in different probabilities (CS-High, 80%; CS-Low, 20%). We simultaneously measured the changes of dopamine level in mPFC and NAc by taking advantage of fluorescent dopamine sensor (GRAB-DA). As a result, we found a learning-dependent increase in dopamine particularly in response to CS-High in both mPFC and NAc, (2) reward prediction error (RPE) cording of dopamine in the NAc, (3) error insensitive, monotonic dopamine response to reward in the mPFC. These results suggest that dopamine release dynamics in the mPFC and NAc contribute to reward-prediction learning in a different way.

### [1P-042]

### Dynamics of cortical, striatal and amygdaloid dopamine release during differential auditory fear conditioning in mice.

*Kazuhiro Umemoto[1], Takaaki Ozawa[1], Moe Nakamura, Yuma Matsumoto[1], Tomohiro Shibata[1], Ryotaro Iwamoto[1], Oyama Yoshinobu[1], Tom Macpherson[1], Takatoshi Hikida[1] ([1]*Laboratory for Advanced Brain Functions, Institute for Protein Research, Osaka University*)

Prediction and avoidance of future aversive events are vital abilities for survival in animals. The midbrain dopamine plays an important role in aversive learnings such as fear conditioning. Previous studies found that striatal dopamine release is inhibited in response to aversive predictive cues and aversive stimuli themselves; however, little is known about how cortical and subcortical dopamine release dynamics change during learning of associations between cues and aversive events. To address this question, we recorded dopamine release in the frontal cortex, the nucleus accumbens and the amygdala in mice during differential auditory fear conditioning by taking advantage of the fluorescence dopamine sensor, GRAB-DA2m. In the present study, we trained mice in an auditory fear conditioning paradigm where one auditory stimulus (conditioned stimulus, $CS^+$) is followed by a mild electrical shock (unconditioned stimulus, US) and another is not ($CS^-$). As a result, we found learning dependent changes of dopamine release during $CS^+$. In the nucleus accumbens, dopamine levels were significantly decreased during the $CS^+$ following conditioning. On the other hand, dopamine releases in the frontal cortex and amygdala were increased during $CS^+$ especially after several days of conditioning. These results suggest the possibility that experience-dependent changes in both cortical and subcortical dopamine releases are important for adaptive fear learning and prediction in mice.

### [1P-043]

### Odor associated with memory-recalling could be a preventive method for cognitive impairment: investigating in human and animal studies

*Yuri Masaoka[1], Akira Yoshikawa[2], Sawa Kamimura[1,3], Hirokazu Ohtaki[4], Motoyasu Honma[1], Kei Sakikawa[1,3], Shotaro Kamijo[5], Masaki Yoshida[6], Masahiko Izumizaki[1]
([1]*Department of Physiology, Showa University School of Medicine*, [2]*Division of Health Science Education Showa University School of Nursing and Rehabilitation Sciences*, [3]*Department of Otorhinolaryngology Head and Neck Surgery, Showa University School of Medicine*, [4]*Department of Functional Neurobiology, Tokyo Univerisity of Pharmacy and Life Science, School of Pharmacy*, [5]*Department of Physiology, Showa University School of Pharmacology*, [6]*Department of Ophthalmology, Jikei University School of Medicine*)

Olfactory impairment is the first sign of mild cognitive impairment and Alzheimer's disease, however, whether the olfactory stimuli could be uses as a preventive measure prior to their typical symptoms is unknown. In this study, two studies were performed to test whether olfactory stimuli could be a preventive method. First, we used functional magnetic resonance imaging (fMRI) to specify the brain regions associated with individual memory (odor memory) in human. fMRI analysis indicated activations in the left orbitofrontal region, and this area of activation strengthened the connectivity with the hippocampus and parahippocampus during odor memory. Next study was to investigate in animals whether enriched olfactory stimuli affect neurogenesis of mitral and granule cells of the olfactory bulb and dentate gyrus of the hippocampus using 5-bromo-2′-deoxyuridine. An enriched olfactory environment significantly increased neurogenesis of mitral and granule cells in the olfactory bulb, but not in the dentate gyrus. Odor associated with memory-recalling might be important for activating hippocampus and frontal regions, and might be an factor for neural genesis of the dentate gyrus.

### [1P-044]

### Effects of saccadic eye movements on face-responsive neurons in the inferior temporal cortex of macaque monkeys

*Kenji Kawano[1,2], Kazuko Hayashi[2], Narihisa Matsumoto[2], Keiji Matsuda[2], Kenichiro Miura[3], Mark Eldridge[4], Richard Saunders[4], Barry Richmond[4], Yasuko Sugase-Miyamoto[2] ([1]*Dokkyo Medical University*, [2]*AIST*, [3]*NCNP*, [4]*NIMH*)

We perceive the outside world by constantly moving our eyes with saccadic eye movements and capturing objects located at various positions in space on the fovea, which has high resolution within the retina. When a person is in the environment, we shift our gaze to the face of the person. By shifting our gaze and recapturing the face on the fovea, we can recognize its details. To study effects of saccadic eye movements on face-responsive neurons, we recorded neuronal activity using microelectrode arrays implanted in the inferior temporal cortex (areas TE and TEO) of macaque monkeys while they performed fixation or saccadic tasks. We presented monkey and human face stimuli (three models with three expressions, respectively) either at foveal or peripheral locations. Both in areas TE and TEO, face-responsive neurons, characterized by the fixation task, responded to facial stimuli when the eyes arrived at the peripheral facial stimulus. We studied the latencies of the response of neurons in both areas to face stimuli. In the fixation task, the latencies of TEO neurons and TE neurons were similar, but in the saccade task, the latencies of the TEO neurons were shorter than those of the TE neurons.

### [1P-045]

### The active spots movement of responses to the FM sounds were analyzed by the frequency-band responses in AI of guinea pigs observed by optical recording.

*Yutaka Hosokawa[1] ([1]*Dept. of Systems Physiol., Grad. Sch. Univ. of Ryukyus*)

The responses to the FM sounds in the primary auditory cortex (AI) of the guinea pig were investigated using optical imaging with a voltage-sensitive dye (RH795). Eight guinea pigs were anesthetized with ketamine (80 mg/kg) and xylazine (40 mg/kg). The patterns of the active spots to the tones (0.5, 1, 2, 4, 8, 16 kHz: duration 200ms) were recorded from the AI near onset time and then measured the 0.5, 1, 2, 4, 8, 16-kHz frequency bands (FB) in the AI. Then, activity patterns to the FM sounds (upward and downward linearly swept frequency: 0.5-16 kHz in 16-200ms duration or FM sweep rate 0.04-0.5 kHz/ms) at 55-75 dB SPL were also recorded. All FB responses to upward FM simultaneously started at onset in dependent of the sound intensities and sweep rates. To the downward FM, the FB responses of higher frequency first appeared and following by the lower frequency band responses. When the FM sweep rate was larger, the off response additionally appeared. These results show that the FB responses to upward FM were different from ones to downward FM. In other words, these results suggest the direction of FM sweep is easily detected by the FB responses.

### [1P-046]

### Working memory-based and -free reward prediction in dual dopamine system of the basal ganglia

*Tomohiko Yoshizawa[1,2,4], Yuuto Miyamura[2], Yuta Ochi[3], Riichiro Hira[2], Makoto Funahashi[1], Yutaka Sakai[4], Yilong Cui[3], Yoshikazu Isomura[2,4] ([1]*Oral Physiology, Department of Oral Functional Science, Faculty of Dental Medicine and Graduate School of Dental Medicine, Hokkaido University*, [2]*Department of Physiology and Cell Biology, Graduate School of Medical and Dental Sciences, Tokyo Medical and Dental University*, [3]*Laboratory for Biofunction Dynamics Imaging, RIKEN, BDR*, [4]*4.Brain Science Institute, Tamagawa University*)

Animals can memorize previous events that lead to good or bad outcomes in their working memory (WM), then use the information to predict the next rewards. To elucidate neuronal bases of the WM-based reward prediction, we compared rat's neuronal activity during two different tasks: one in which an operant behavior was alternately rewarded (WM-based task) and another in which the same operant behavior was randomly rewarded with a 50% probability (WM-free task). The 2-deoxy-2-[18F]fluoro-D-glucose-positron emission tomography revealed that midbrain dopamine (DA) area was more strongly activated by the WM-based task than by the WM-free. Neurons electrophysiologically recorded from the lateral portion tended to have weaker reward responses in the WM-based task than in the WM-free, reflecting WM-based reward prediction errors (RPE). At the time when mice got rewards in the WM-based task, suppressed DA releases (DA dips) optically recorded in the dorsomedial striatum (DMS), whereas phasic DA releases were observed in the nucleus accumbens, reflecting WM-free RPE. Our results suggest that DA system including the DMS is a neuronal basis of the WM-based reward prediction.

### [1P-047]

### Information processing for time estimation and production in prefrontal cortex and medial premotor areas of the monkey

*Atsushi Chiba[1], Kazunori Morita[2], Ken-ichi Oshio[1], Masahiko Inase[1] ([1]*Dept. Physiol., Faculty Med., Kind Univ.*, [2]*Dept. Physiol., Sch. Med., Iwate Med. Univ.*)

To investigate Information processing for time estimation and production, we examined neuronal activity in dorsolateral prefrontal cortex (PFC) and medial premotor areas (MPA) of monkeys during the task involved in estimation of visual stimulus duration and production of motor preparation duration. When monkeys pressed the hold key, a visual cue was presented for 0.8, 1.6, or 3.2 sec. Following a delay period, a retention signal was presented. Monkeys were trained to keep pressing the hold key until the start of the allowed press interval, and to press a target button during that interval. When the cue was 0.8, 1.6, or 3.2 sec, the interval was 3.2 to 4.8, 1.6 to 3.2, or 0.8 to 1.6 sec after the retention signal was presented, respectively. In PFC, cue-responsive neurons showed build-up activity during the cue presentation. Delay-responsive neurons showed activity that changed depending on cue duration. In MPA, delay-responsive neurons also exhibited activity representing cue duration. Retention-responsive neurons showed build-up activity toward the end of the retention period. No neurons were related to both estimation of cue duration and duration production in PFC or MPA. These results suggest that PFC engaged more in duration estimation of the cue, and that MPA was involved more in production of retention duration based on the cue information from PFC. COI: NO

### [1P-048]

### Clarification of pathophysiology of neuropsychiatric disorders of genome-edited macaque monkeys

*Yang Fang[1], Hirotaka Onoe[2], Sayaka Takihata-Matsushita[1], Hideaki Tsuchiya[3], Chizuru Iwatani[3], Masataka Nakaya[3], Setsuko Tsukiyama-Fujii[3], Tomoyuki Tsukiyama[1,3], Tadashi Isa[1,2,4] ([1]*Institute for the Advanced Study of Human Biology (WPI-ASHBi), Kyoto University,* [2]*Human Brain Research Centre, Graduate School of Medicine, Kyoto University,* [3]*Research Center for Animal Life Science, Shiga University of Medical Science,* [4]*Division of Neurobiology and Physiology, Department of Neuroscience, Graduate School of Medicine, Kyoto University*)

Psychiatric disorders, such like schizophrenia and bipolar disorder, affect more than 10% of population worldwide. Disrupted-In-Schizophrenia-1 (DISC1) was initially identified as a promising candidate gene for schizophrenia and bipolar disorder in a Scottish pedigree. Animal studies using gene mutant mice model from multiple groups demonstrated that DISC1 is involved in cerebral cortex development and synaptic functions, and suggested that DISC1 mutant may underlie neurodevelopmental dysfunction in schizophrenia. However, the direct relationship between gene mutant and animal mental illness still remains unclear. Furthermore, it is difficult to identify human-specific psychiatric disorders in rodents. To tackle this problem, we have generated the DISC1 knockout (KO) macaque monkeys (*Macaca fascicularis*) in collaboration with Shiga University of Medical Science. In order to elucidate the pathophysiology of psychiatric disorders in DISC1 KO monkeys, we designed and implemented multiple testing environments (individual/grouping) and recording methods (e.g. movement trace tracking, locomotion activity recording, etc.), for exploring the monkey brain development, cognitive ability, and individual/social behaviors. By analyzing the behavior and functional brain network organization, we are trying to clarify the pathophysiology of neuropsychiatric disorders for identifying the mechanism of emergence of primate-specific structural and functional abnormalities of the developing brain.

### [1P-050]

### Effects of Color in Mental Illness

*Yuka Ito[1], Midori Shibushita[1,2], Ikuko Takeda[1,2], Hiroaki Wake[1,2] ([1]*Department of Anatomy and Molecular Cell Biology Nagoya University Graduate School of Medcine,* [2]*Division of Multicellular Circuit Dynamics National Institute for Physiological Sciences, National Institutes of Natural Sciences*)

### [1P-049]

### Social context-dependent usage of communicative signals in songbirds

*Mizuki Fujibayashi[1], Kentaro Abe[1,2,3] ([1]*Faculty of Science, Tohoku University,* [2]*Division for the Establishment of Frontier Sciences, Tohoku University,* [3]*Graduate School of Life Sciences, Tohoku University*)

Songs are the vocal signals songbirds use to communicate with others, composed of sound elements—syllables—weaved into sequences. Though some species of songbirds, including the Bengalese finch, use a variety of syllable sequence for communication, how they utter different songs in different contexts remain poorly understood. To explore the social modulation of songs and the neural mechanism underlying them, we utilized an experimental system we have previously developed to analyze syllable sequences under virtual social contexts, along with a neural network-assisted automated analysis of song contents. We found that the presentation of the video-recorded social signals evoked changes in their behavioral response. Concerning the ordering of syllables, the structure of songs also displayed stimuli-dependent changes varied in the persistence of the structural change in songs; some were transient, and some were long-lasting. These songs evoked different behavioral reactions to conspecifics when presented, implying that they function as a communicative signal distinct from before. We observed a differential pattern of neural activity according to the social situation by calcium imaging of the brain. Our results reveal that the Bengalese finches flexibly change the contents of their song according to the social and environmental situation for communication. Our experimental system establishes an efficient method to explore the neural mechanism underlying the flexible modulation of communicative signals in songbirds.

### Poster Presentation

[1P]
### Neurophysiology, Neuronal cell biology
### Motor function

March 14 (Tue.), 12:10 - 14:10, Poster Room

### [1P-051]

**Modulation of the swallowing reflex by electrical stimulation of the gigantocellular reticular nucleus**

*Arisa Murakawa[1], Yoshihide Satoh[1], Tomohito Sakazume[1] ([1]*Nippon Dental Univ.*)

The previous study reported that the swallowing reflex was suppressed by stimulation of the pedunculopontine tegmental nucleus (PTg). Morphological studies have demonstrated that the fibers of the PTg project to the gigantocellular reticular nucleus (Gi). From these studies, it is assumed that the Gi is involved in control of swallowing. This study investigated whether the swallowing reflex is modulated by stimulation of the Gi. The experiments were used anesthetized rats by urethane. The swallowing reflex was evoked by repetitive electrical stimulation of the superior laryngeal nerve (SLN). The electromyogram was recorded from the mylohyoid muscle to identify the swallowing reflex. Electrical stimulation was applied to the Gi. During recording sessions, the SLN and the Gi were simultaneously stimulated. As a control, the SLN was solely stimulated before (pre-control) and after (post-control) the simultaneous stimulation. After each recording, the stimulus sites were confirmed histologically. The number of swallowing reflexes were decreased or increased by the Gi stimulation. When the number of swallowing reflexes were decreased, the onset latency of the first swallow was significantly longer than in the pre-control or post-control. The present study suggests that the Gi is involved in the control of the swallowing reflex.

### [1P-052]

**Neural circuits for suppressing saccades during visual fixation and its release for saccade initiation**

*Mayu Takahashi[1], Yuriko Sugiuchi[1], Yoshikazu Shinoda[1] ([1]*Tokyo Medical and Dental University*)

The role of the rostral superior colliculus is somewhat controversial; the ideas of a "fixation zone" located in the rostral pole of the SC vs only extension of the caudal saccade zone. This study was to investigate whether there are differences in the neural circuit from rostral/caudal SC to omnipause neurons (OPNs), which are known to fire during fixation and are suppressed during saccades. We made a series of studies using electrophysiology, intracellular recordings, and pathway tracing in anesthetized cats. The results showed that OPNs received monosynaptic excitation from the rostral SC and disynaptic inhibition from the caudal SC, and this latter disynaptic inhibition was mediated via inhibitory burst neurons (IBNs). Intracellular staining of single IBNs with HRP demonstrated that they projected to the contralateral OPNs. We conclude that OPNs are activated by the rostral SC during fixation, and prior to a saccade they receive decreased activation from the rostral SC and also strong IBN inhibition that results from increased caudal SC activity. Together this study provides direct proof for the circuit that suppresses OPN activity to trigger saccades.

### [1P-053]

**Chemogenetic activation of convergent inputs to the cervical motoneurons enhances forelimb motor performance in monkeys**

*Michiaki Suzuki[1], Kenta Kobayashi[2], Yukio Nishimura[1] ([1]*Tokyo Metropolitan Institute of Medical Science*, [2]*National Institute for Physiological Sciences*)

Inactivation of neural activity disrupts functions, while how enhancement of neural activity influences behavior remains unknown. Here we show chemogenetic activation of convergent inputs to spinal motoneurons enhances forelimb motor performance in monkeys. We injected retrograde viral vector expressing the excitatory designer receptors exclusively activated by designer drug (DREADD) hM3Dq unilaterally into the cervical enlargement innervating forelimb in macaque monkeys. Under anesthesia the administration of DREADD agonist deschloroclozapine (DCZ) increased the neuronal activity of primary motor cortex innervating cervical enlargement and the forelimb muscle activity. This chemogenetic activation improved motor performances such as arm reaching time and grasping force in behaving monkeys. These findings highlight a methodology for investigating the causal role of enhancement of neural activities in nonhuman primate sensorimotor system, and open up the possibility of a new generation of neuromodulatory therapy facilitating impaired motor functions after neural damage via chemogenetic neural control prosthetics.

### [1P-054]

**Suppression of the swallowing reflex by stimulation of the center of lateral reticular nucleus in rats**

*Tomohito Sakazume[1], Yoshihide Satoh[1], Arisa Murakawa[1], Shogo Ohkoshi[1] ([1]*Nippon Dental Univ.*)

The previous study reported that the swallowing reflex was suppressed by stimulation of the red nucleus. Morphological studies have demonstrated that the lateral reticular nucleus (LRN) receives projection fibers from the red nucleus. This study examines whether the swallowing reflex is modulated by stimulation of the LRN. These experiments were performed on rats anesthetized by urethane. The swallowing reflex was evoked by repetitive electrical stimulation of the superior laryngeal nerve. The electromyogram was recorded from the mylohyoid muscle to identify the swallowing reflex. Electrical stimulation was applied to the LRN or glutamate was injected into the LRN. Electrical stimulation of the LRN had suppressive effect on the number of swallows. The onset latency of the first swallow during electrical stimulation of the LRN was significantly increased. The number of swallows was significantly reduced at 0, 2, 5, 10, 20, and 25 min after injection. The onset latency of the first swallow was also significantly increased at 10 min after injection. These results suggest that the LRN is involved in the control of swallowing and the LRN indirectly inhibits the swallowing central pattern generator.

### [1P-055]

**Motor skill training induces activation of rubrospinal tract to compensate for corticospinal tract disruption in experimental diabetic rats**

*Ken Muramatsu[1], Niimi Naoko[2], Ikutomo Masako[3], Shimo Satoshi[4], Tamaki Toru[5], Niwa Masatoshi[6], Sango Kazunori[2] ([1]*Dept Physical Therapy, Kyorin University*, [2]*Diabetic Neuropathy Project, Tokyo Metropolitan Institute of Medical Scienc*, [3]*Dept. Physical Therapy, University of Tokyo Health Sciences.*, [4]*Dept. Occupational Therapy, Health Science University*, [5]*Dept. Physical Therapy, Nagoya Women's University*, [6]*Dept. Occupational Therapy, Kyorin University*)

We previously demonstrated that diabetes disrupts the corticospinal tract (CST) system components controlling hindlimb and trunk movement, which causes size reduction of the hindlimb motor area of the motor cortex. In this study, rehabilitative effects of a 2-week aerobic training (DM-AT) and complex motor skill training (DM-ST) on motor disorder of streptozotocin-induced type 1 diabetes in rats were investigated. In the electrophysiological mapping of the motor cortex, only the DM-ST group showed extension of the motor cortical area while the sedentary diabetic animals and rats in the DM-AT group did not show any changes. Hand grip strength and rotarod latency also increased in the DM-ST group; however, no change after intervention was noted in others. Immunohistochemical analysis indicated that the rubrospinal tract of the DM-ST group expressed 43-kD phosphorylated growth-associated proteins, a specific marker of axons with plastic changes. Additionally, electrical stimulation of the hindlimb area of the red nucleus elicited larger motor-evoked potentials of the hindlimb in the DM-ST group than that in other experimental groups, suggesting strengthening of the synaptic connections between the red nucleus and spinal motoneurons by motor skill training. These results show that motor skill training in a diabetic rat model induced plastic changes of the rubrospinal tract that could compensate for diabetes-induced disruption of the CST system components controlling the hindlimb.

### [1P-056]

### Effect of ketamine on eye-movement characteristics of macaque monkeys

*Aya Takemura[1], Kenichiro Miura[2] ([1]*National Institute of Advanced Industrial Science and Technology (AIST),* [2]*National Institute of Mental Health, National Center of Neurology and Psychiatry*)

Eye-movement characteristics are often associated with psychiatric disorders. However, it is unclear physiological and pathological mechanisms of these eye movement abnormalities. This study aimed to determine whether macaque monkey is a good animal model of humans to investigate eye movement abnormalities in schizophrenia (SCZ) and its underlying neuronal mechanism. In the present study, we investigated NMDA hypothesis by recording the effect of ketamine intramuscular injection (0.3 mg/kg) on the eye movements of two monkeys (*Macaca fuscata*). As for oculomotor tasks, we used a free-viewing task adapted from an eye movement examination for SCZ (Miura et al., 2014) and a tracking eye movement task. The pre-injection eye-movement data were obtained in the same day. In the free-viewing task, we found that the macaque monkeys in pre-injection showed exploratory visual behaviors as in healthy humans. After intramuscular injection of ketamine, the monkeys showed a reduced exploratory visual scanning in the free viewing task consistent with an eye movement abnormality observed in patients with SCZ. We also found that smooth tracking eye movements decreased after injection. These results suggest that the macaque monkeys could be an appropriate animal model to uncover the underlying neural mechanisms.

### [1P-057]

### Establishment of a behavioral task to study neural mechanisms of behavioral adaptations driven by different types of prediction error

*Masayoshi Murakami[1], Tomoki Ishimaru[1], Keisuke Kameda[1], Kazuo Kitamura[1] ([1]*University of Yamanashi*)

According to Marr-Albus-Ito theory, climbing fiber inputs to the cerebellum serve as a teaching signal that induces synaptic plasticity in the cerebellum and allows an organism to adapt to a new sensorimotor context. In line with this theory, climbing fiber inputs are activated by prediction errors, mismatches between predicted and actual outcomes of behavior. But we do not know how different types of prediction errors, such as prediction errors for spatial and temporal aspects of sensory inputs and reward prediction errors, are represented in a population of climbing fibers. To address this, we devised a behavioral task where mice experienced different types of prediction errors. In this task, a head-fixed mouse obtained a water reward by licking a spout that was presented after a short delay from a trial initiation cue. To evoke sensory prediction errors or reward prediction errors, we introduced non-standard trial blocks where the position or timing of spout presentation was changed, or the reward was omitted. Mice quickly adjusted their anticipatory licking in response to these changes.We are currently investigating how climbing fiber inputs represent different types of prediction errors with 2-photon calcium imaging.

### [1P-058]

### Contribution of the interhemispheric pathways to the recovery of dexterous hand movements after the corticospinal tract lesion in macaque monkeys

*Masahiro Mitsuhashi[1], Reona Yamaguchi[2], Toshinari Kawasaki[1], Satoko Ueno[1,2], Isa Tadashi[1,2] ([1]*Kyoto Univ Grad Sch of Med.,* [2]*Institute for the Advanced Study of Human Biology (WPI-ASHBi), Kyoto Univ.*)

Corticospinal tract (CST) lesions impair dexterous hand movements in higher primates. It is known that damaged neurons hardly regenerate, but the impaired motor function recovers to some extent by rehabilitation. Therefore, the mechanism of the functional recovery is due to plastic change of residual neural circuits. Based on our previous studies, we hypothesized that the interhemispheric pathways from the contralesional to the ipsilesional premotor cortex (PM) are playing a role for the recovery. In this study, we blocked the interhemispheric pathways with chemogenetic techniques during the recovery after the CST lesion and investigated whether the recovered motor function was impaired. In addition, we recorded the electrocorticography at the bilateral sensorimotor areas to evaluate the effect of the pathway blocking on brain activities and connectivities. In the intact states, the hand dexterity was not impaired by the blocking, but the connectivity from contralesional to ipsilesional PMs was decreased and the brain activities at ipsilesional PM was increased. During the early phase of the recovery, the hand dexterity was impaired and the brain activities at ipsilesional PM was decreased by blocking the pathways. These results suggested that the interhemispheric pathways from contralesional PM inhibited the ipsilesional PM in the intact states, while during the recovery after the CST lesion, these pathways facilitated the ipsilesional PM and contributed to the recovery of the hand dexterity.

### [1P-059]

### The effects of DREADD ligands treatments on motivational food seeking in mice

*Yoshinobu Oyama[1,2], Yoshiatsu Aomine[1,2], Takaaki Ozawa[1,2], Tom Macpherson[1,2], Takatoshi Hikida[1,2] ([1]*Osaka Univ. Graduate School of Science, Department of Biological Sciences,* [2]*Osaka Univ. Institute for Protein Research , Advanced Brain Function lab.*)

Clozapine N-oxide (CNO) has been developed as a ligand to selectively activate designer receptors exclusively activated by designer drugs (DREADD). However, previous studies have revealed that peripherally injected CNO is reverse-metabolized into clozapine and activates DREADD receptors in the brain suggesting an off-target effect of CNO on animal physiology and behaviors by itself. The second-generation DREADD agonists compound 21 (C21) and JHU37160 (J60), which are not reverse-metabolized into clozapine, have also been developed. Although the off-target effect of these drugs is assumed to be limited, it has not been fully understood. To address this issue, we tested the possible unexpected effect of three different designer drugs (CNO, C21, J60) by themselves on motivational food-seeking behavior. We investigated the effect of acute injection (0.1 to 10 mg/kg, i.p.) on the performance in the operant licking task in a progressive-ratio schedule. As a result, we found the treatments of these designer drugs do not change motivational food-seeking, whereas only a high dose of J60 (10 mg/kg) seriously impaired mice's locomotor activity. On the other hand, we found the injection of clozapine (1 or 2 mg/kg) significantly decreased motivational food seeking in mice. This study suggested the off-target effects of CNO, C21, and J60 with moderate doses (0.1 to 3 mg/kg) on motivational food seeking are very limited.

### [1P-060]

### Rapid changes in cortico-striatal signal transmission during adaptive behavior in monkeys

*Okada Ken-ichi[1], Tanaka Masaki[1] ([1]*Hokkaido Univ.*)

The basal ganglia play a pivotal role in adaptive behavior. Flexible changes in neuronal activity in the caudate nucleus of the striatum have been reported both during long-term learning and during rapid changes in task demands. Alterations of striatal neuron activity during long-term learning are causally related to changes in cortico-striatal transmission and synchrony of local field potentials (LFPs). However, it remains to be seen whether similar changes will occur during trial-by-trial adjustment of behavioral strategies. To address this, we evaluated cortico-striatal transmission by examining the LFPs and spiking responses in the caudate nucleus to electrical stimulation of the supplementary eye field (SEF) in monkeys performing the oculomotor tasks. Striatal neurons showed spiking activity with a latency of 26.5 ± 3.8 ms (N = 51) in response to a single-pulse electrical stimulation of the SEF. LFPs 10–20 ms after electrical stimulation were more negative when spikes were elicited, indicating that negative potentials were associated with excitatory post-synaptic potentials. Evoked LFP was greater when electrical stimulation was delivered during the task than during the inter-trial interval and earlier than later in the task. Furthermore, the evoked LFPs and spikes were highly dependent on the phase and amplitude of low-frequency oscillatory activity. These results suggest that the signal transduction in the cortico-striatal pathway is dynamically regulated by network states, thereby altering striatal neuronal activity for rapid behavioral control.

### [1P-061]

### Influence of exercise patterns on the cortical facilitation and inhibition during an attentional focus task

*Amiri Matsumoto[1], Akari Ogawa[1], Chihiro Oshima[1], Rieko Aruga[1], Mai Ikeda[1], Keisuke Irie[1], Nan Liang[1] ([1]*Cognitive Motor Neuroscience, Department of Human Health Sciences, Graduate School of Medicine, Kyoto University*)

It remains unclear whether the exercise patterns differentially modulate the corticospinal excitability during an attentional focus task. The healthy participants were asked to perform a dynamic or static exercise of right index-finger abduction under the external (object) and internal (finger movement) focus conditions. We applied transcranial magnetic stimulation of the left primary motor cortex or electrical stimulation of the ulnar nerve at -70 ms before and 500 and 3000 ms after the agonist muscle onset, then motor evoked potential (MEP) or F-wave was recorded from the right first dorsal interosseous muscle. In the dynamic exercise task, MEP amplitude was significantly larger with external than internal focus condition at all stimulus timings ($P<0.05$, respectively) and the silent period was significantly longer with external than internal focus condition at 500 ms and 3000 ms ($P<0.05$, respectively), while no differences were observed in the static exercise task. F-wave showed no difference in the amplitude and occurrence between the focus conditions in both exercise tasks. This study revealed that the cortical contribution to an attentional focus might be modulated depending on the exercise patterns. (COI: NO)

### [1P-062]

**The effects of poly(I:C) on the body movement activity in the perinatal period.**

*Yuki Kosaka[1], Naoko Masutani[1], Daisuke Sakuma[1], Saki Yoshida[1], Akiko Arata[1]
([1]*Dept. of Physiome, Hyogo Medical University*)

Poly ((I:C): PIC) is an immunostimulant in viral infections and it has been reported that experimental procedure of pregnant mice with PIC, which induces a virus infection-like immune response, results in development disorders in the neonatal mice. However, the effects of PIC on the medulla oblongata and spinal cord during the perinatal period are still unknown. In this study, we investigated the effects of PIC as an indicator of body movement activity. The data were recorded from the C8 and L4 ventral roots of spinal cord isolated from the embryonic 17-19-day-old and postnatal 0-2-day-old rat isolated brainstem-whole spinal cord preparation and recorded as upper and lower limb respectively. In embryonic preparations, 5-HT was applied to stimulate body movement activity, then PIC was applied under 5-HT. In neonatal period, the preparations were applied with strychnine as a glycine antagonist to remove the blockade of body movement, and PIC was applied. We examined the effects of PIC on body movement activity during the embryonic and neonatal stages. In the embryonic period, small movement activity accompanied with body movement activity was observed when PIC was applied under 5-HT. Moreover, during the neonatal stage, application of 5-HT under strychnine induced large body movement followed by a small activity, which was more significant in the P0 than in the P2. These results showed that PIC changed normal body movement to body movement with shaking activity, sort of the movement with PIC treated embryonic rat in pregnant rat.

### [1P-063]

**Formation pattern of climbing fiber at P15 in rats of neonatal white matter injury with paralysis**

*Kenta Kajiura[1], Taiga Sato[1], Shiori Tominaga[1], Shinya Ueno[1], Naoki Tajiri[1], Takeshi Shimizu[1], Hideki Hida[1] ([1]*Dept Neurophysiol and Brain Sci, Nagoya City Univ*)

### [1P-064]

**Functional linkage between cerebellum and medial frontal cortex in behavioral error detection**

*Kaede Abe[1], Ken-ichi Okada[1], Masaki Tanaka[1] ([1]*Hokkaido Univ.*)

### Poster Presentation

### [1P]
### Neurophysiology, Neuronal cell biology
### Sensory function, Sensory organ

March 14 (Tue.), 12:10 - 14:10, Poster Room

### [1P-065]

### Temporal calibration in taste temporal order judgments is influenced by empathizing tendency

*Makoto Wada[1], Kouji Takano[1], Tatsu Kobayakawa[2] ([1]*National Rehabilitation Center for Persons with Disabilities,* [2]*National Institute of Advanced Industrial Science and Technology*)

Latency of gustatory evoked magnetic fields of salty tastant occurs faster than sweet's one by approximately 100 ms (Kobayakawa et al., 1999). It would be caused by differences in receptor type (i.e., ionotropic or metabotropic). However, its effect on the perception is still unknown. In this study, we examined how the delay is perceived and how autistic-like traits affect the perception. Participants (n=14) were required to contact tips of their tongues with a small hole in a tube that purified water was running. Tastants (Salty: 0.5M NaCl, Sweet: 1M Sucrose, Their mixture) sectioned with air bubbles were sequentially delivered to the participants. The participants were required to reproduce the orders by pressing buttons. The orders were correctly reproduced (86%) with stimulus onset asynchrony of approximately 500 ms. When the mixture was delivered, "sweet first" judgment ratio was correlated with empathy quotient score (r=-0.75, p<0.0018). The result indicates that temporal calibration may occur in individuals with higher empathy, which may complement the delay from the receptor characteristics. We will investigate relationships between the taste perception and food selectivity.

### [1P-066]

### The effect of green tea and tea catechin on the optokinetic responses in aging mice

*Yuko Sugita[1], Takahisa Furukawa[1] ([1]*Osaka University*)

The intake of catechins, especially epigallocatechin gallate (EGCG), which is abundant in green tea, is effective for retinal tissue protection; however, it remains unclear whether and how EGCG modulates visual function. The optokinetic response (OKR) is a reflexive eye movement elicited by a moving visual pattern that has been studied in several animal species, including humans, and serves as a useful tool for evaluating visual motion processing. In present study, to investigate the effect of EGCG contained in green tea on visual motion processing in young and aging mice, we investigated the OKRs of mice fed a diet containing matcha or green tea. The OKR the was examined by measuring the slow phase eye velocity of the optokinetic nystagmus induced by sinusoidal gratings of various spatiotemporal frequencies moving for 30s. The mice after administrated green tea showed the OKRs with a temporal frequency tuning that was higher from those in control mice. In addition, to investigate the effect of EGCG on visual motion processing, EGCG was intraperitoneally administered to young and aging mice, and eye movements were measured. We found that the OKR of the mice after EGCG administration became higher in temporal sensitivity than control mice. From the above results, the visual motion processing for optokinetic responses by ingesting green tea was enhanced, which may be related to the effect of EGCG.

### [1P-067]

### Physiological Properties of Utricular Hair Cells in *Gpr156-/-* Mice Lacking Reversal of Hair Bundle Orientation

*Kazuya Ono[1,2], Ruth Anne Eatock[2], Basile Tarchini[3] ([1]*Osaka University,* [2]*Univerisity of Chicago,* [3]*The Jackson Laboratory*)

In otolith organs, hair bundles of hair cells have varying orientations that reverse along the line of polarity reversal (LPR) located within or at the edge of the central striolar zone. This striking anatomical feature creates simultaneous excitatory and inhibitory responses to translational stimuli, but the significance for vestibular function is not clear. Recent work has shown that the reversal is controlled by the transcription factor *Emx2*, localized on one side of the LPR, acting via G protein-coupled receptor GPR156, which is uniformly expressed in vestibular hair cells (Kindt *et al., Nat Commun* 2021). *Gpr156*$^{del/del}$ otolith organs lacking *Gpr156* expression lose the LPR without clear macroscopic anatomical defects. In this study, we examined whether loss of LPR affects physiological properties of individual hair cells. Whole-cell patch clamp recordings were made from hair cells in excised utricles from post-natal (P12-100) *Gpr156*$^{del/+}$ and *Gpr156*$^{del/del}$ mice. Responses of hair cells were recorded to steps of hair bundle deflection, applied by a rigid probe, or of membrane voltage or current, applied by the whole-cell recording electrode. *Gpr156* deletion had no significant effect on mechanoelectrical transduction by hair cells or on the physiological differentiation between type I and type II hair cells, as indicated by expression of type-specific voltage-gated potassium conductances (n = 9-29 for each combination of cell type (I and II) and genotype). In summary, we find that the deletion of *Gpr156* and resulting loss of bundle orientation reversal in the utricular LES does not disrupt key properties of individual hair cells, suggesting that *Gpr156*$^{del/del}$ mouse is an useful model to study the role of LPR in the vestibular function.

### [1P-068]

### Atypical responses of auditory temporal order judgment in adults with autism spectrum disorders

*Aya Sato[1], Naomi Ishii[1], Michiru Makuuchi[2], Makoto Wada[1] ([1]*Developmental Disorders Section, Department of Rehabilitation for Brain Functions, Research Institute of National Rehabilitation Center for Persons with Disabilities,* [2]*Section of neuropsychology, Department of Rehabilitation for Brain Functions, Research Institute of National Rehabilitation Center for Persons with Disabilities*)

Autism spectrum disorder (ASD) is characterized atypical sensory characteristics. In the auditory fields, there are known some difficulties related to selective hearing and sound localization. However, it is not fully understood why they develop such sensory characteristics. To investigate the mechanism, we conducted psychophysical experiments on auditory functions related to individuals with ASD. Two individuals with ASD (1 male, 1 female) and 5 typically developed individuals (2 males, 3 females) as a control group participated in the initial study. Auditory temporal order judgment (TOJ) task was employed to assess effect of perceived spatial position on temporal resolution. Whereas the temporal resolution tended to be lower in typically developed individuals when the interval of two sound position perceived in the cranium was narrower, in individuals with ASD, the temporal resolution was not changed even when the perceived positional sense was narrow. These results suggest that though the typically developed individuals once position the sound in the space, then judge the order, individuals with ASD might simply discriminate the temporal order judgment without positioning it in space. In the future, it is necessary to investigate which brain area was responsible of the auditory characteristics in the individuals with ASD.

### [1P-069]

### Neural response in the posterior insular cortex evoked by stimulation of the basolateral amygdala in central post-stroke pain model rats

*Tomofumi Otsuki[1,2,3,4], Kazuaki Nagasaka[4,5], Naofumi Otsuru[4,5], Hideaki Onishi[4,5] ([1]*System Emotional Science, Faculty of Medicine, University of Toyama,* [2]*Major of Cognitive and Emotional Neuroscience, University of Toyama Graduate School of Innovative Science,* [3]*Graduate School, Niigata University of Health and Welfare,* [4]*Institute for Human Movement and Medical Sciences, Niigata University of Health and Welfare,* [5]*Department of Physical Therapy, Niigata University of Health and Welfare*)

Central post-stroke pain (CPSP) is chronic pain with delayed onset after stroke in the ventral posterolateral nucleus (VPL) of the thalamus. Previous brain imaging studies in CPSP model animals showed increased neuronal activity in the basolateral amygdala (BLA) and posterior insular cortex (PIC) related to mechanical allodynia, but it is unclear how the functional connections between the two regions are altered following VPL lesion. Here, we investigated this by measuring neural response of the PIC induced by electrical stimulation of the BLA in CPSP model rats. Collagenase type IV was injected into the left VPL to generate an artificial hemorrhagic lesion under anesthesia. The occurrence of tactile allodynia in the hind limb was confirmed by von Frey test from after one week of the lesion to the end of the behavioral analysis. In electrophysiological experiments, local field potentials (LFP) in PIC evoked by single-pulse stimulation of BLA were obtained and amplitude was calculated. One week after thalamus injury LFP amplitude was not different as compared to a healthy individual, but after four weeks the significantly increased activity was found (p<0.05). Our results suggest that changes in electrophysiological activity from the BLA to the PIC occur late after injury and may be involved in the mechanism of CPSP. This work was supported by JST SPRING, Grand Number JPMJSP2145.

### [1P-070]

### Bone-conducted inaudible-ultrasound evokes synchronized hair cells excitation in guinea pig cochleae.

*Bakushi Ogawa[1,2], Kazuhiro Horii[1], Iori Morimoto[1], Noriko Nagase[1,2], Chikara Abe[1], Fumiaki Nin[1] ([1]*Division of Biological Principles, Department of Physiology, Graduate School of Medicine, Gifu University,* [2]*Department of Otolaryngology-Head and Neck Surgery, Graduate School of Medicine, Gifu University*)

Although the hearing range of humans is under 20 kHz, we hear inaudible-ultrasound via bone conduction. However, details of its physiological mechanism are unknown. In this study, to investigate the possibility of ultrasound reception in the cochlea, we measured the local field potentials (LFP) evoked by sound and ultrasound stimulations. Under general anesthesia, we recorded the potentials from the temporal bone in guinea pigs. The stimulus waveform was 127 kHz 110 dB tone burst with a duration of 200 msec. 127 kHz is above the guinea pig's hearing range. We performed frequency analysis by fast Fourier transform on LFP. In the analysis, we observed three signal peaks: 127 kHz, 202.85 kHz, 245.85 kHz. Examining the relationship between these signals and stimuli in waveforms, only 127 kHz was synchronized with the stimulus. This synchronized LFP is likely the cochlear microphonics (CM), which reflects the excitation of the primary auditory receptor cells. Furthermore, as we increased the strength of the stimulus at 127 kHz, CM amplitude gradually increased. Its stimulus-potential relationship was 'nonlinear'; cochlear amplifies smaller sound more, and bigger sound less. Additionally, in comparison with the potential recorded under anoxia, CM was actively amplified. We finally measured CM at frequencies within the range from 80 to 301 kHz. At a frequency of 127 kHz or lower, CM exhibited significant nonlinearity and amplification. To the contrary, these features were lessened in more than 127 kHz, and disappeared at frequencies over 201 kHz.

### [1P-071]

### Starburst amacrine cells form gap junctions in the early postnatal stage of the mouse retina.

*Toshiyuki Ishii[1], Takuma Maruyama[1], Makoto Kaneda[1] ([1]*Nippon Medical School*)

In the central nervous system, gap junctional coupling is important for the maturation of neuronal network. Similarly, in the retina, many types of cells have known to form gap junctional couplings during developmental period. Currently, starburst amacrine cells (SACs) are recognized as a neuron which does not form gap junctional couplings in the retina. However, no systematic analysis of gap junctional couplings in SACs has been carried out. In the present study, therefore, we examined whether gap junctional couplings by SACs occur during the developmental period in the mouse retina. When we injected Neurobiotin into SACs, many tracer-coupled cells were detected before eye-opening. Some tracer-coupled cells were retinal ganglion cells, and tracer coupling was not detected between SACs. The number of tracer-coupled cells significantly decreased after eye-opening. Membrane capacitance (Cm) before eye opening was larger than Cm after eye opening in SACs. The application of meclofenamic acid (MFA), a gap junction blocker, reduced the Cm of SACs before eye opening. At the mRNA level, connexin 43 expression levels significantly decreased after eye opening. Dark rearing did not change the time course of Cm reduction in SACs. The application of MFA reduced the frequency of retinal waves before eye opening. These results suggest that SACs form gap junctions in early postnatal period, and inherently reduce the connection to neighboring cells during the development.

### [1P-072]

### Ionomycin-induced currents recorded from the olfactory receptor neurons in the goldfish olfactory epithelium slices

*Kana Sakamoto[1], Takashi Tsunenari[1] ([1]*Dept. Appl. Chem., Chem. Eng., and Biochem. Eng., Grad. Sch. Sci. and Eng., Yamagata Univ.*)

The olfactory epithelium (OE) of teleosts contains several types of olfactory receptor neurons (ORNs) including ciliated ORNs (cORNs) and microvillous ORNs (mORNs). We recorded whole-cell currents from ORNs of goldfish OE slices and tested effects of ionomycin on the cells that did not respond to IBMX and forskolin. With a patch-pipette solution containing 0.3 mM EGTA for weak $Ca^{2+}$ buffering, bath application of 1 or 2.5 μM ionomycin induced slow (35-43 s to peak) inward currents in a subset of cells. Even slower (72-93 s to peak) currents were observed with 3 mM EGTA in the whole-cell pipette, consisting with the notion that the currents were activated by the increased internal $[Ca^{2+}]$. Reduction of $[Cl^-]_{out}$ caused no apparent shift of reversal potential in the preliminary tests, suggesting the existence of $Ca^{2+}$-dependent cation conductance in the goldfish mORNs. Even in the cells that responded to odorants (amino acids) but not to IBMX and forskolin, not all cells responded to ionomycin. mORNs with various amounts of the $Ca^{2+}$-dependent conductance may be involved in the olfactory transduction.

### [1P-073]

### $\alpha_2\delta$-1 expressed in spinal dorsal horn neurons is involved in aberrant excitatory synaptic transmission and mechanical hypersensitivity after peripheral nerve injury.

*Keisuke Koga[1], Kenta Kobayashi[2], Makoto Tsuda[3], Kazufumi Kubota[4], Kitano Yutaka[4], Hidemasa Furue[1] ([1]*Hyogo Medical University,* [2]*National Instutute for Physiological Sciences,* [3]*Kyushu University,* [4]*Daiichi Sankyo Company, Limited*)

Neuropathic pain, a highly debilitating pain condition that commonly appears after nerve damage, is caused by the aberrant excitability of spinal dorsal horn neurons. Gabapentinoids, the current therapeutic drugs, inhibit spinal calcium-mediated neurotransmitter releases by binding to $\alpha_2\delta$-1 subunits and alleviate neuropathic pain. However, the mechanisms of $\alpha_2\delta$-1-mediated synaptic alterations in the spinal dorsal horn following nerve injury are not fully elucidated. In this study, we investigated the role of spinal $\alpha_2\delta$-1 subunits in mechanical hypersensitivity and how spinal synaptic transmission is altered after peripheral nerve injury. Using *in situ* hybridization technique, we found that *Cacna2d1* mRNA, coding $\alpha_2\delta$-1 subunit, was expressed in excitatory neurons but not in inhibitory neurons in spinal dorsal horn. We also found that excitatory post-synaptic responses evoked by electrical stimulation applied to spinal dorsal horn neurons were significantly enhanced after nerve injury, and that the evoked responses were significantly decreased by application of mirogabalin, a potent $\alpha_2\delta$-1 inhibitor. Using clustered regularly interspaced short palindromic repeats (CRISPR)-Cas9 system, spinal dorsal horn neuron-specific ablation of *Cacna2d1* alleviated aberrant synaptic transmission and mechanical hypersensitivity induced following nerve injury. These results suggest that $\alpha_2\delta$-1 expressed in excitatory neurons in the spinal dorsal horn facilitates spinal nociceptive synaptic transmission, and contributes to the development of peripheral nerve injury-induced mechanical hypersensitivity.

### [1P-074]

### Comparison of response patterns to sound amplitude changes among neurons in different auditory fields

*Sohei Chimoto[1] ([1]*Department of Neurophysiology, Division of Medicine, University of Yamanashi*)

Sounds under natural acoustic environments exhibit a broad of time envelope changes in amplitude. They contain a sound with a quick attack in amplitude and a sound with a slow increase. In the previous studies we showed that the higher auditory cortices as well as the primary auditory cortex (A1) had velocity-sensitive cells for the damped- and ramped-sounds and some neurons in the secondary auditory cortex (A2) and posterior auditory fields (PAF) had the sensitivities to direction of the amplitude change. It is well known that a core area of auditory fields, the anterior auditory fields (AAF) show a tonotopy to pure tone stimulus and the neurons respond to various kinds of sounds such as noise bursts, AM sounds, and natural sounds. However, little is known about the responses during the asymmetrical stimuli. In the present study we recorded single unit activities from AAF of awake animals and examined response patterns during amplitude changes. In the damped sound, the amplitude of sound wave exponentially decreases with a time constant set to 1/5th of the stimulus duration. The ramped stimulus is just the time-reversed version of the damped stimulus. We found that most of AAF neurons showed sensitivities to the abrupt change of stimulus envelope and weaker selectivity for damped- or ramped-sounds than those in A2 and PAF. These results might suggest that the majority of AAF neurons are tuned to the velocity but not to the direction of the amplitude change. (COI:No)

### [1P-075]

### A mathematical model for explaining an impact of genetic mutations on ionic currents of photoreceptor

*Yuttamol Muangkram[1], Saya Ito[1], Yukiko Himeno[1], Akira Amano[1] ([1]*Dept. of Bioinformatics, Coll. of Life Sciences, Ritsumeikan Univ.*)

Mutations in ion channel-encoding genes of photoreceptors can lead to vision impairment and blindness. Understanding of pathophysiological mechanisms underlying the electrophysiological deficiency in photoreceptors may assist improving the therapeutic approaches. The goal of this study is to construct a mathematical model of a mammalian photoreceptor for evaluating the changes in electrophysiological properties due to missense mutations in *Cacna1f* gene. The proposed model is based on the ionic current model of vertebrate photoreceptor proposed by Kamiyama et al. 2009, and we have incorporated the Goldman-Hodgkin-Katz formulation together with ion concentration calculation. Various ionic currents were presented in this model based on the gene expression database of mouse, and also corresponding electrophysiological models were incorporated if the model of the ion current of a specific gene was available. The simulation results can quantitatively reveal the biophysical processes of ionic current dynamics, including the equilibrium potential of ion channels, the electrophysiological activities, and ion homeostasis. Furthermore, the new model can explore a shift in the voltage dependence of L-type calcium channels due to the *Cacna1f* mutation, which is the cause of X-linked congenital stationary night blindness. This work was supported by JSPS KAKENHI Grant Number 22K20514.

### [1P-076]

### Synaptic homeostasis elicited by intranasal administration of rotenone in mouse olfactory bulbs.

*Tomohiro Noguchi[1], Hitoshi Sasajima[1], Sadaharu Miyazono[1], Kaoru Takakusaki[1]
([1]*Asahikawa Medical University*)

Intranasal administration of rotenone, a mitochondrial inhibitor, induced cell death specific to olfactory bulb dopaminergic neurons and reduced olfactory function in mice. Patch-clamp recordings from projection neurons in olfactory bulb slices after rotenone administration revealed that the frequency of spontaneous inhibitory postsynaptic currents decreased but current amplitude increased. We considered that increased input amplitude suggests the existence of a compensatory mechanism for decreased frequency of synaptic inputs. In other types of olfactory dysfunction, inhibition of mastication with soft-diet feeding reduced olfactory function in mice. Immunohistochemistry with BrdU labelling showed that the number of newly generated interneurons migrating from the subventricular layer to the olfactory bulb was reduced. In patch-clamp experiments, the frequency of inhibitory synaptic inputs to projection neurons decreased as expected. However, unlike intranasal administration of rotenone, no increase in amplitude was observed. Therefore, it was inferred that the compensatory increase in inhibitory postsynaptic currents depends on the mechanism of olfactory impairments. Analyzing dependence of compensatory mechanisms on pathogenesis provides new insights into synaptic homeostasis which is important for maintaining brain function.

### Poster Presentation

[1P]
**Neurophysiology, Neuronal cell biology**
**Others**

March 14 (Tue.), 12:10 - 14:10, Poster Room

### [1P-077]

**Neuritin-1 protects neural function against cerebral infarction in the mouse auditory cortex.**

*Hideki Derek Kawai[1], Kenichi Kunii[1], Makoto Nakanishi[1], Yumi Fukuzaki[1] ([1]*Soka University*)

A neurotrophic factor nuritin-1 reportedly protects neurons against transient ischemia-induced injury. However, it is unclear whether it does so in a permanent infarction model or whether the neural function is protected. Here we tested these in the photo-thrombosis model of focal middle cerebral artery occlusion (PT-MCAO). This permanent MCAO model resulted in unilateral cortical injuries from about 2 mm anterior and about 3 mm or more posterior to the bregma at 3 days after stroke. Local injection of neuritin-1 (2 ng) at 1 mm posterior to the bregma immediately after PT-MCAO reduced the size of the injury at 1 mm posterior to the injection site and beyond. Having confirmed the structural protection, we next examined if the cortical function is also protected by neuritin-1 treatment. Since the protected area contained the primary auditory cortex (A1), we recorded sound-evoked activities monitoring the changes in flavoprotein fluorescence on the cortical surface in vivo. Sound exposure via a speaker in front of a mouse elicited fluorescence increases bilaterally to a similar extent. In mice with unilateral PT-MCAO, sound-evoked activities were nearly absent on the ipsilateral A1, while preserving the activities in the contralateral A1. Neuritin-1 injection near the infarct, however, kept the total intensity and area size at the levels like those of the contralateral side. These data indicate that neuritin-1 could protect against stroke-induced injury and sustain neuronal function in A1.

### [1P-078]

**Transcranial direct current stimulation alters the clearance of brain metabolic waste in mice**

*Yan Wang[1], Hiromu Monai[1] ([1]*Ochanomizu Univ.*)

Cerebrovascular diseases, including ischemic stroke, are among the most severe conditions. Not only is the mortality rate high, but the secondary effects are not negligible and result in long-term nursing and rehabilitation. In clinical research, transcranial direct current stimulation (tDCS), which involves passing a very weak direct current through the skull or scalp for 10-30 minutes, has been increasingly investigated as an adjunct to facilitate the rehabilitation of diseases including stroke. Many studies have shown that tDCS has positive therapeutic effects on stroke, but the mechanisms are still not clearly understood. The cellular mechanisms of anodal tDCS have been suggested by Monai et al. (2016), in which activation of adrenergic receptors has a significant role in the mouse brain. In contrast, Monai et al. (2019) found that adrenergic receptor blockade also facilitated the recovery from motor dysfunctions after acute ischemic stroke in mice. The results suggested that the adrenergic receptor blockade facilitated the normalization of the brain's extracellular ion milieu by the cerebrospinal fluid (CSF) and interstitial fluid (ISF) exchange. However, it is still unclear how tDCS affects the dynamics of CSF-ISF exchange in the brain. In this study, we report that after tDCS, the CSF tracer over the cortex increased, suggesting that tDCS alters the CSF-ISF exchange in the clearance of brain metabolic waste.

### [1P-079]

**Serotonergic regulation of cerebral energy metabolism**

*Akiyo Natsubori[1] ([1]*Sleep Disorders Project, Tokyo Metropolitan Institute of Medical Science*)

The central serotonergic system has multiple roles in animal physiology and behavior, including sleep-wake control. However, its function in controlling brain energy metabolism according to the state of animals remains unclear. Through *in vivo* monitoring of energy metabolites and signaling, we demonstrated that optogenetic activation of raphe serotonergic neurons increased cortical neuronal intracellular concentration of adenosine triphosphate (ATP), an indispensable cellular energy molecule. The serotonergic neuronal activation-induced increase of neuronal ATP level was suppressed by inhibiting neuronal uptake of lactate derived from astrocytes, a type of glial cells. Raphe serotonergic neuronal activation induced cortical astrocytic $Ca^{2+}$ and cAMP surges and increased extracellular lactate concentrations, suggesting the facilitation of lactate release from astrocytes. Furthermore, chemogenetic inhibition of raphe serotonergic neurons partly attenuated the increase in cortical neuronal intracellular ATP levels as arousal increased in mice. In conclusion, serotonergic neuronal activation promoted an increase in cortical neuronal intracellular ATP levels, partly mediated by the facilitation of the astrocyte-neuron lactate shuttle, contributing to state-dependent optimization of neuronal intracellular energy levels.

### [1P-080]

**Cortical depth of the first dorsal interosseous region in the human primary motor cortex revealed by fMRI and TMS**

*Takahiro Osada[1], Koji Nakajima[1,2], Akitoshi Ogawa[1], Satoshi Oka[1], Koji Kamagata[1], Shigeki Aoki[1], Yasushi Oshima[2], Sakae Tanaka[2], Seiki Konishi[1] ([1]*Juntendo University*, [2]*The University of Tokyo*)

The first dorsal interosseous (FDI) representation in the primary motor cortex (M1) is a standard stimulation target for various transcranial magnetic stimulation (TMS) and transcranial ultrasound stimulation (TUS) experiments. For TUS experiments, the depth of the stimulation target must be set somehow. Ideally, the depth of the stimulation target should be determined based on subject-wise brain activation using functional magnetic resonance imaging (fMRI). Here, we report the distribution of the cortical depth of the FDI region in the M1 (FDI-M1) based on fMRI from 50 subjects. fMRI scans were administered to identify the FDI-M1 in each subject while the subjects performed a motor task designed to activate the FDI-M1. There was variability in depth from the cortical surface to the FDI-M1. The median was 2.7 mm, and 80% of the population was located within 6 mm from the cortical surface. The FDI-M1 in each subject was targeted by TMS with the aid of an online navigator, and motor-evoked potentials were measured. The motor thresholds depended on the distance between the cortical surface and FDI-M1, which validates the FDI-M1 detected by fMRI. We further propose an easy and practical solution for the focus length parameter in TUS experiments of the FDI-M1, consisting of setting the target to approximately 3 mm away from the cortical surface.

### [1P-081]

***Cnpy32xHA* mice reveal neuronal expression of Cnpy3 in the brain**

*MD MONIRUL ISLAM[1], Hiroki Mutoh[1], Kazushi Aoto[1], Hirotomo Saitsu[1] ([1]*Department of Biochemistry, Hamamatsu University School of Medicine*)

Identified biallelic *CNPY3* variants in patients and *Cnpy3* knock-out mice have indicated that the loss of CNPY3 function causes neurological disorders such as epilepsy. However, the basic property of CNPY3 in the brain remains unclear. In this study, *Cnpy3-2xHA* knock-in mice were generated by C-terminal attaching with two HA epitope tags to investigate the role of CNPY3 in the brain. HA-tagged CNPY3 was confirmed by immunoblot analysis using HA and CNPY3 antibodies, although HA tagging caused the decreased CNPY3 protein level. Immunohistochemical analysis of *Cnpy3-2xHA* knock-in mice showed that CNPY3-2xHA was specifically expressed in the neuron. In addition, CNPY3 and CNPY3-2xHA were localized in the endoplasmic reticulum and synaptosome, and showed age-dependent expression changes in the brain. Taken together, we demonstrated that *Cnpy3-2xHA* knock-in mice could be generated to further elucidate the role of CNPY3 in brain function and neurological disorders.

### [1P-082]

### Deletion of Glutamate decarboxylase 67-kDa isoform causes object location memory impairment in rats.

*Kazuyuki Fujihara[1,2], Toshikazu Kakizaki[2], Yuchio Yanagawa[2] ([1]*Department of Psychiatry and Neuroscience, Graduate School of Medicine, Gunma University,* [2]*Departments of Genetic and Behavioral Neuroscience, Graduate School of Medicine, Gunma University*)

γ-Aminobutyric acid (GABA), the major inhibitory neurotransmitter in the central nervous system, plays an important role in the regulation of cognitive functions. Dysfunctions of the GABAergic system have been reported in psychiatric disorders such as major depressive disorder and schizophrenia. Since these psychiatric disorders are accompanied by cognitive dysfunction, GABAergic dysfunction is considered to be one of the hypothetical backgrounds of these disorders. Recently, we developed the glutamate decarboxylase 67-kDa (GAD67) knockout rats using the CRISPR/Cas9 technique. GAD67 is one of the two GABA-synthesizing enzymes, which is downregulated in the postmortem brains of schizophrenia patients. Although we have reported that GAD67 knockout rats show various behavioral alterations, it is still elusive which domains of cognition are mostly affected. In the present study, we carried out both novel object recognition (NOR) and object location recognition tasks (OLR) to address this issue. As a result, GAD67 knockout rats displayed a reduced performance selectively in the OLR task. The present result suggests that GAD67 affects hippocampus-dependent memory. This is an important finding in elucidating the physiological function of GAD67.

### [1P-083]

### Neuroprotection by the mitochondrial uncoupler FCCP from ischemia reperfusion injury revealed by glucose metabolism of rat brain slices

*Tatsuya Asai[1,2,3], Yuta Tanaka[1], Yasushi Kiyono[2,3], Hidehiko Okazawa[2,3] ([1]*Graduate School of Engineering, University of Fukui,* [2]*Biomedical Imaging Research Center, University of Fukui,* [3]*Life Science Innovation Center, University of Fukui*)

Objectives: Mitochondrial dysfunction is considered to be one of the main mechanisms involved in ischemia-reperfusion injury, and mild mitochondrial uncoupling may have neuroprotective effects by restoring mitochondrial function. We have measured glucose uptake in rat brain slices as index of tissue viability, and examined whether the mitochondrial uncoupler FCCP has neuroprotective effects against ischemia-reperfusion injury. Methods: Sagittal brain slices were prepared from Wistar rats (7–8 weeks old) and incubated with 100 kBq/mL [$^{18}$F]FDG in oxygenated Krebs-Ringer solution at 36°C. Serial two-dimensional time-resolved images of [$^{18}$F]FDG uptake were obtained by replacing imaging plates every 15 min. Results: Reperfusion after 45-min ischemia, [$^{18}$F]FDG uptake became almost zero, indicating the ischemia-reperfusion load caused irreversible damage to the brain tissue. Addition of 1 μM FCCP during reperfusion, [$^{18}$F]FDG uptake decreased but remained, indicating less damage, and the neuroprotective effects of FCCP could be observed even when the uncoupler was applied 30 min after the start of reperfusion. Conclusion: These results suggest that FCCP has neuroprotective effects by restoring mitochondrial function.

### [1P-084]

### Physiological roles of mastication in depression model mice

*Mie Kamate[1,2], Hitoshi Teranishi[1], Kenji Kawano[2], Reiko Hanada[1] ([1]*Department of Neurophysiology, Oita University, Faculty of Medicine,* [2]*Department of Oral and Maxillofacial Surgery, Oita University*)

Background: Modern society is a "stress society", and it is known that excessive stress destroys the homeostasis in health and progresses to mental and neurological disorders such as depression. It has been reported that the "chewing" (mastication) decreases stress levels, however the details of this phenomena and molecular mechanisms are not fully clarified yet. Methods: Groups of mice with different chewing intensity (solid feed group and powder feed group) were subjected to “Repeated Social Defeat Stress (R-SDS)” , and behavioral analysis was performed. To address examine, a social interaction test (SIT) was performed after 10 days of R-SDS. A series of behavioral analysis was performed using a video tracking system. Furthermore, we examined neurotransmitter dynamics such as catecholamine and serotonin, acetylcholine, excitatory glutamic acid, inhibitory GABA in R-SDS using HPLC and GC-MS systems. To evaluate the stress level of R-SDS, blood corticosterone level was measured. Results: R-SDS for 10 days increased blood corticosterone levels in defeat group mice compared to non-defeat group mice. In addition, in SIT, the time spent in the stress avoidance zone was significantly increased in the powder-fed group of the defeat group mice compared to the solid-fed group. We are currently measuring neurotransmitters in the prefrontal cortex, dorsal hippocampus, ventral hippocampus, amygdala, and locus coeruleus. Conclusion: It was found that the difference in food texture (difference in mastication intensity) affects stress resilience, and it was clarified that a decrease in mastication intensity reduces resilience to stress.

### Poster Presentation

[1P]
### Molecular physiology, Cell physiology
### Ion channels, Receptors

March 14 (Tue.), 12:10 - 14:10, Poster Room

### [1P-085]

### Effects and underlying mechanisms of bioactive components of licorice on GIRK channel activity

*I-Shan Chen[1], Tomoe Y. Nakamura-Nishitani[1] ([1]*Wakayama Medical University*)

Licorice is a common food additive and also a Chinese herbal medicine. Excess licorice intake is known to induce side effects, such as hypertension and cardiac arrhythmias. Recent studies suggest that cardiac type G-protein-gated inwardly rectifying $K^+$ channels (GIRK1/4) are constitutively activated in patients with chronic atrial fibrillation. We therefore hypothesized that excess licorice intake may affect the activity of GIRK. In the present study, we investigated the effects of the main ingredient of licorice, glycyrrhizic acid (GA), and its metabolite 18β-glycyrrhetic acid (18β-GA) on GIRK channel activity and the underlying mechanism. By electrophysiological recordings using *Xenopus* oocytes expressing different GIRK subunits (GIRK1, 2, 4), we observed that GA inhibits the current of GIRK1-containing channels in a voltage-independent manner. In contrast, 18β-GA activated the current of all combinations of GIRK channels in a voltage-dependent manner. Mutation of a GIRK1-specific amino acid residue in the pore helix, Phe137, to Ser abolished the inhibitory effect by GA, while it potentiated the activation effect by 18β-GA. Taken together, these data indicate that GA and 18β-GA have distinct actions on GIRK currents and diverse mechanisms underlying their regulations.

### [1P-086]

### A switching mechanism of $PIP_2$- and voltage- gating modes depending on the conformations of the second S4 helix in two-pore channels

*Takushi Shimomura[1,2], Kiichi Hirazawa[1,2], Yoshihiro Kubo[1,2] ([1]*Division of Biophysics and Neurobiology, National Institute for Physiological Sciences,* [2]*Department of Physiological Sciences, SOKENDAI*)

Two-pore channels (TPCs) contain two repeats of the 6-transmembrane functional unit, which is commonly observed in the superfamily of voltage-gated cation channels, in a single subunit. Domain I (DI) is responsible for $PIP_2$ binding, while DII plays a major role in voltage sensing. TPC3 generates voltage-dependent $Na^+$ currents that are enhanced by $PIP_2$. TPC2 normally shows only $PIP_2$-gated currents, but exhibits voltage-gated currents in the presence of an antidepressant, desipramine. It remains unclear how these complex gating modes in TPCs are induced.We analyzed the structure-function relationships of TPCs in *Xenopus* oocyte expression system. Voltage clamp fluorometry analysis of DII-S4 movement in TPC3 revealed that it has an intermediate state. Immobilization of this conformation converted TPC3 to a strongly $PIP_2$-dependent channel. A stabilized intermediate conformation in TPC2 similarly attenuated desipramine-induced voltage-dependent gating, while it did not affect the $PIP_2$-evoked currents. Our results show that the two gating modes which optimally respond to voltage and $PIP_2$ are switched depending on the DII-S4 conformations and that the mechanism is shared in TPC subtypes. (COI: NO)

### [1P-087]

### EP4 promoted cell migration via CaMKK2 and AMPK in oral cancer cells

*Soichiro Ishikawa[1,2], Masanari Umemura[1], Rina Nakakaji[1,2], Akane Nagasako[1], Kagemichi Nagao[1], Yuto Mizuno[1], Kohei Osawa[1,2], Mitomu Kioi[2], Kenji Mitsudo[2], Yoshihiro Ishikawa[1] ([1]*Cardiovascular Research Institute (CVRI), Yokohama City University Graduate School of Medicine,* [2]*Department of Oral and Maxillofacial Surgery, Yokohama City University Graduate School of Medicine*)

[Introduction] We have previously reported that EP4, one of prostaglandin E2 (PGE2) receptors, promoted cell migration via $Ca^{2+}$ signaling in oral cancer cells. However, it remains unknown how $Ca^{2+}$ signaling regulates cell migration. Therefore, we focus on the role of calcium/calmodulin-dependent protein kinase kinase 2 (CaMKK2) and its downstream. [Materials and Methods] STO-609 (CaMKK2 inhibitor) and ONO-AE1-437 (EP4 agonist) were used. Human gingival fibroblasts, HGnF and Human tongue squamous cell carcinoma cell lines, HSC-3 were used. Changes in intracellular $Ca^{2+}$ levels were examined by Fura-2, a $Ca^{2+}$-sensing fluorescent dye. Migration was examined with the scratch assay. Lamellipodia formation was observed by cell immunostaining. Intracellular energy production was evaluated using an ATP assay kit. To ablate CaMKK2, shRNA was induced with lentiviral infection in HSC-3. [Results] EP4 agonist increased the intracellular $Ca^{2+}$ and promoted the cell migration in HSC-3, not in HGnF. Western blots showed that EP4 agonist increased the phosphorylation of CaMKK2 and AMP-activated protein kinase (AMPK). Furthermore, EP4 agonist increased the lamellipodia formation and the intracellular ATP. STO-609 and CaMKK2-knockdown negated the EP4 agonist-induced cell migration in HSC-3. [Conclusion] Our results suggest that EP4 enhanced the mitochondrial biogenesis via CaMKK2 and AMPK, and promoted the cell migration of oral cancer.

### [1P-088]

### Regulation of voltage-sensing phosphatase (VSP) by its substrate, phosphoinositide $PI(4,5)P_2$

*Natsuki Mizutani[1], Yasushi Okamura[1] ([1]*Integrative Physiology, Graduate School of Medicine, Osaka University, Suita, Japan*)

Among the voltage-dependent membrane protein superfamily, voltage-sensing phosphatase (VSP) is the only protein in which a voltage sensor domain (VSD) regulates an enzyme activity voltage-dependently. The cytoplasmic catalytic region (CCR) has remarkable structural similarity to phosphatase and tensin homolog (PTEN) which dephosphorylates mainly $PI(3,4,5)P_3$, but the principal substrate of VSP is $PI(4,5)P_2$. $PI(4,5)P_2$ is thought to bind to the N-terminal $PI(4,5)P_2$-binding motif (PBM) of PTEN which is also conserved in the C-terminal half of the linker connecting the VSD and the CCR among VSP orthologs. Interestingly, previous studies suggest that $PI(4,5)P_2$ modulates VSD motion and VSD-CCR coupling through the PBM-like region of VSP. However, whether $PI(4,5)P_2$ directly binds to the linker has not been known. In this study, we expressed *Ciona intestinalis* VSP with a fluorescent unnatural amino acid (Anap) incorporated in the PBM-like region or its vicinities in *Xenopus* oocytes, and analyzed $PI(4,5)P_2$-dependent structural rearrangements of these residues by measuring fluorescence upon membrane depolarization by voltage clamp fluorometry. We compared fluorescence signals between in the presence or absence of pre-depolarization. We found activity-dependent changes in the kinetics of the signals at several residues, consistent with the idea that $PI(4,5)P_2$ binds to the PBM-like region or its vicinities. (COI: NO)

### [1P-089]

### Regulation of pro-tumorigenic cytokine expression by $Ca^{2+}$-activated $K^+$ channel, $K_{Ca}3.1$ in $M_2$-like macrophages

*Susumu Ohya[1], Miki matsui[1], Junko Kajikuri[1], Hiroaki Kito[1], Kyoko Endo[1] ([1]*Dept. Pharamcol., Grad. Sch. Med Sci., Nagoya City Univ.*)

The human monocytic leukemia cell line, THP-1-differentiated $M_2$-like macrophages are a useful tool to investigate the physiological significance of tumor-associated macrophages (TAMs). In the tumor microenvironment (TME), TAMs with the $M_2$-like phenotype play a critical role in the promotion of cancer progression and metastasis by inhibiting the immune surveillance system. We examined the role of $K_{Ca}3.1$ in the expression of pro-tumorigenic cytokines and angiogenic growth factors in THP-1-derived $M_2$ macrophages. THP-1 cells into $M_0$ macrophages were induced by treatment with PMA treatment, and then cells were treated with IL-4 and IL-13 to induce the polarization of $M_2$ macrophages. The expression levels of IL-8 and IL-10 were significantly decreased by treatment with the selective $K_{Ca}3.1$ activator, SKA-121 in THP-1-derived $M_2$ macrophages. Furthermore, under in vitro experimental conditions that mimic extracellular $K^+$ levels in the TME, IL-8 and IL-10 levels were both significantly elevated, and these increases were reversed by treatment with SKA-121. Among several signaling pathways potentially involved in the transcriptional regulation of IL-8 and IL-10, respective treatments with ERK and JNK inhibitors significantly suppressed their transcriptions, and treatment with SKA-121 significantly reduced the phosphorylated ERK, JNK, c-Jun, and CREB levels. These results strongly suggest that the $K_{Ca}3.1$ activator may suppress IL-10-induced tumor immune surveillance escape and IL-8-induced tumorigenicity and metastasis by inhibiting their production from TAMs through both ERK and JNK signaling pathways.

### [1P-090]

### Quantitative analysis of *Trpm4*-knockout microglia movement by temperature-controlled time-lapse imaging

*Rei Nishimoto[1,4], Yoshikazu Matsuoka[2], Makoto Tominaga[3] ([1]*Okayama University*, [2]*Okayama University Hospital*, [3]*National Institute for Physiological Sciences*, [4]*Fukuyama Medical Center*)

Thermosensation is an essential property for the survival of living organisms and is critical even at the cellular level (Hoffstaetter et al., 2018). Even though we found that mouse microglia exhibit temperature-dependent motility through activation of transient receptor potential receptor vanilloid 4 (TRPV4) (Nishimoto and Derouiche et al., 2021), the involvement of TRP melastatin 4 (TRPM4), a calcium-activated TRP channel, is still being investigated. In this study, we generated *Trpm4*-knockout (M4KO) mice by CRISPR-Cas9 system and evaluated whether TRPM4 is involved in the temperature-dependent microglia movement by *in vitro* time-lapse imaging. Mouse microglia from whole brains of mouse pups were prepared for time-lapse imagings at 33 °C, 37 °C or 40 °C in 2 hrs. As a result, M4KO microglia moved in a temperature-dependent manner (the distance traveled by M4KO microglia 113.5 ± 5.1 µm at 33 °C n=151, 175.3 ± 4.1 µm at 37 °C n=240, and 201.4 ± 5.6 µm at 40 °C n=175) comparable to Wt (112.9 ± 6.0 µm at 33 °C n=117, 175.3 ± 5.2 µm at 37 °C n=235, and 204.3 ± 7.2 µm at 40 °C n=150). Those results suggested that TRPM4 is not solely involved in the temperature-dependent microglia movement. It might be possible that activation of TRPM4 could be involved in other mechanisms like secretion in the digestive system requiring calcium-activated signal transduction to trigger the actin remodeling.

### [1P-091]

### The transmembrane domains of THIK-1 channel play critical roles in the regulation of the channel activity

*Michihiro Tateyama[1,2], Yoshihiro Kubo[1,2] ([1]*Dev. of Biophysics and Neurobiology, National Institute for Physiological Sciences*, [2]*Dept. of Physiological Sciences, SOKENDAI*)

A two-pore domain $K^+$ (K2P) channel, THIK-1, is activated by arachidonic acid (AA) and stimulation of Gq-coupled receptors (Gq-Rs). We previously observed that two amino acid residues in the 4th transmembrane domain (M4) of THIK-1 play critical roles in the AA and Gq-R dependent activation, using a UV-dependent cross-linking unnatural amino acid, 4 amido-phenyalanine (AzP). In this study, we focused on M2, another inner helix, and introduced AzP one at a time to bulky residues in the lower part, to search for the residues critical for the channel regulation. Seven mutants incorporating AzP showed an increase in current and one showed decrease, upon application of AA or activation of Gq-coupled adrenergic receptor α1A-AR. Upon UV-exposure, six of them showed an increase in the current amplitude and one showed a decrease, possibly due to the UV-induced cross-linking reaction. Especially, I139AzP and L140AzP showed a marked increase in the current amplitude upon UV exposure, but a decrease in the response to α1A-AR and AA. The results suggest that a high conductive conformation is induced and stabilized by the cross-linking reaction of AzP. The two residues are shown to locate at a crossing point of inner helices (M2 and M4) of THIK-1 by a prediction using AlphaFold2. Taken together, this study shows that the M2 as well as M4 play critical roles in the regulation of THIK-1.

### [1P-092]

### $Ca^{2+}$ dynamics in primary skeletal myocytes from malignant hyperthermia mouse model

*Yoshitaka Tsuboi[1], Kotaro Oyama[2], Fuyu Kobirumaki-Shimozawa[1], Takashi Murayama[3], Nagomi Kurebayashi[3], Toshiaki Tachibana[1], Yoshinobu Manome[1], Emi Kikuchi[1], Satoru Noguchi[4], Takayoshi Inoue[4], Yukiko U. Inoue[4], Ichizo Nishino[4], Shuichi Mori[5], Ryosuke Ishida[5], Hiroyuki Kagechika[5], Madoka Suzuki[6], Norio Fukuda[1], Toshiko Yamazawa[1] ([1]*Jikei Univ.*, [2]*National Institutes for Quantum Science and Technology*, [3]*Juntendo Univ.*, [4]*National Center of Neurology and Psychiatry*, [5]*Tokyo Medical and Dental Univ.*, [6]*Osaka Univ.*)

Type 1 ryanodine receptor (RYR1) is a $Ca^{2+}$ release channel in the sarcoplasmic reticulum (SR) of the skeletal muscle and is important for excitation-contraction coupling. Mutations in RYR1 lead to serious muscle diseases, such as malignant hyperthermia (MH), a disorder of $Ca^{2+}$-induced $Ca^{2+}$ release (CICR) through RYR1 from the SR. We recently described that volatile anesthetics trigger MH -like episodes via enhanced CICR in heterozygous R2509C-RYR1 mice. However, the characterization of $Ca^{2+}$ dynamics has yet to be investigated in skeletal muscle cells from homozygous mice because these animals die in utero. In the present study, we generated primary cultured skeletal myocytes from R2509C-RYR1 mice. No differences in cellular morphology were detected between wild type (WT) and mutant myocytes. Electron microscopic observation revealed that the sarcomere length was shortened in homozygous myocytes, as compared to WT and heterozygous myocytes, respectively. Consistently, the resting intracellular $Ca^{2+}$ concentration was higher in homozygous myocytes than in WT or heterozygous myocytes, which may have been coupled with a reduced $Ca^{2+}$ concentration in the SR. Finally, using infrared laser-based microheating, we found that heterozygous myocytes showed larger heat-induced $Ca^{2+}$ transients than WT myocytes. Our findings suggest that the R2509C mutation in RYR1 causes dysfunctional $Ca^{2+}$ dynamics in a mutant-gene dose-dependent manner in the skeletal muscles, in turn provoking MH-like episodes and embryonic lethality in heterozygous and homozygous mice, respectively.

### [1P-093]

### Analysis of the dynamic structure of voltage-gated cation channels using transition metal ion Förster resonance energy transfer

*Kiichi Hirazawa[1,2], Takushi Shimomura[1,2], Yoshihiro Kubo[1,2] ([1]*National Institute for Physiological Sciences*, [2]*The Graduate Univ. for Advanced Studies*)

Voltage-gated cation channels have a canonical motif composed of 6 transmembrane helices, and the 4th helix (S4) moves in a voltage-dependent manner. By applying voltage-clamp fluorometry (VCF) technique to Two-pore channel 3 (TPC3), we previously investigated the S4 movement, and found that it is regulated by phosphoinositide (PI) binding. Although the standard VCF analysis can detect the S4 movement using the attached fluorophore, the detailed information about the S4 movement, such as vertical, horizontal, and/or tilting movements, cannot be obtained. Transition metal ion Förster resonance energy transfer (tmFRET) is recently utilized to obtain more detailed information about the S4 movement by analyzing the change in the distance between an attached fluorophore (donor) and a transition metal ion (acceptor). We aim to investigate the detail of the S4 movement and its PI dependence in TPC3 by tmFRET analysis. S506 or Q508 in the extracellular region of the S4 was labeled by incorporation of a fluorescent unnatural amino acid, Anap, and the neighboring S3 or S5 helix was labeled by Cys-reactive TETAC-$Cu^{2+}$ at the introduced Cys residue. These constructs were expressed in *Xenopus* oocytes for the analysis of their currents and FRET changes. The screening of the constructs and the optimization of the experimental conditions are ongoing. (COI : NO)

### [1P-094]

### Investigation on the arrhythmogenicity caused by gain-of-function mutations of TRPM4

*Yaopeng Hu[1], Qin Li[2], Takayuki Fujita[1], Xin Zhu[2], Ryuji Inoue[1] ([1]*Dept. Physiol., Sch. Med., Fukuoka Univ.*, [2]*Dept. Biomed. Info. Technol., Aizu Univ.*)

The $Ca^{2+}$-activated monovalent cation-selective channel TRPM4 plays non-trivial roles in cardiac electrophysiology. Many of its gain-of-function mutations were found to cause conduction block associated with sudden death. To elucidate the mechanism underlying, we performed gating analysis of its E7K or Q854R mutants expressed in HEK293 cells by the ionomycin-perforated cell-attached recording. The results indicated that the closed-to-open transition is greatly accelerated and a sojourn in the open state is much stabilized in these two mutants with increased voltage-and $Ca^{2+}$-sensitivities. Single-cell action potential (AP) and 1D-cable simulations with a human Purkinje fiber AP model respectively showed that enhanced activity of E7K or Q854R mutants strikingly prolongs AP duration with depolarized resting membrane potential and slows AP propagation resulting in both partial and complete conduction blocks. Furthermore, 2D simulations considering cellular heterogeneity disclosed that altered gating of the two mutants not only generates a different degree of conduction blocks but complex reentrant activities, suggesting the pleiotropy of TRPM4 mutations in arrhythmogenesis. These results clearly demonstrate that the pathologically enhanced activities in gain-of-function mutations of TRPM4 can account for observed arrhythmogenicity. (COI:No)

### [1P-095]

### Direct interaction between the voltage-gated proton channel and ATP

*Akira Kawanabe[1], Maki Takata[1], Kohei Takeshita[2], Yuichiro Fujiwara[1] ([1]*Faculty of Medicine, Kagawa University*, [2]*RIKEN*)

The voltage-gated proton channel (Hv1) transports protons across the cell membrane in response to the membrane potential [Sasaki et al. 2006]. We have shown that intracellular ATP, which has not been considered a regulator of Hv1, works as an activator that enhances the proton current of Hv1 using electrophysiological techniques [The 99th Annual meeting of the physiological society of Japan]. Furthermore, the experiments with ATP analogs revealed that the potentiation effects depended on the number of phosphate groups (Adenosine < AMP < ADP <ATP). In the present study, we examined whether ATP binds directly to Hv1 using Microscale Thermophoresis (MST) method. The truncated mouse-Hv1 protein (Thr57-Ile224), which is responsive to ATP confirmed by electrophysiological analysis, was expressed in *E.coli*, purified, and analyzed. The MST experiments showed that the binding affinities of ATP analogs to purified Hv1 were Adenosine < AMP < ADP < ATP, which is consistent with the results in the electrophysiological experiments. These results indicate that ATP binds directly to Hv1 and regulates its activity. We will discuss the molecular mechanisms and physiological significance of these regulations. COI: No

### [1P-096]

### A rapid efflux of 5-Fluorouracil inducive calcium signal in anti-cancer drug resistant gastric cancer cells

*Hayato Hiraki[1], Satoshi Nishizuka[1] ([1]*Iwate Med. Univ.*)

The $Ca^{2+}$ signal is known as a second messenger. It has been reported that cancer cells express multiple $Ca^{2+}$ receptors, channels, and pumps in different levels depending on the cancer type. However, it remains unknown whether the $Ca^{2+}$ signal is involved in anti-cancer drug resistance of cancer cells. Understanding the mechanism of drug resistance is an important processes to provide a better therapy. In this study, we observed 5-Fluorouracil (5-FU) inducive $Ca^{2+}$ signals in gastric cancer cell lines, MKN45 and its 5-FU resistant, MKN45/5FU. The 5-FU-inducive $Ca^{2+}$ signals were observed under a laser scanning confocal microscope with a $Ca^{2+}$ sensor agent CaTM-2 AM. The $Ca^{2+}$ uptake was induced in both cell lines, while the efflux speed of $Ca^{2+}$ was higher in MKN45/5FU than that of MKN45. The expression level of *PMCA2*, a $Ca^{2+}$ pump-encoded gene, was constitutively higher in MKN45/5FU than that of MKN45. In contrast, MKN45 could induce the expression level of *PMCA2* and *SPCA1* depending on the volume of $Ca^{2+}$ signal in response to 5-FU treatment. Our findings suggest that the $Ca^{2+}$ signal related factors such as $Ca^{2+}$ pumps may be involved in the early stage of mechanism of drug resistance. COI: properly declared

### [1P-097]

### Dissociation and inter-channel dimerization of voltage-sensor domains (VSD) of voltage-gated $Na^+$ channel in the resting state

*Ayumi Sumino[1,2], Takashi Sumikama[1,4], Leonardo Puppulin[1], Mikihiro Shibata[1,2], Katsumasa Irie[3] ([1]*WPI-NanoLSI, Kanazawa Univ.*, [2]*Infiniti, Kanazawa Univ*, [3]*Sch. of Pharm. Sci., Wakayama Med. Univ.*, [4]*PRESTO/JST*)

Gating of voltage-gated sodium channels ($Na_v$) is essential for action potential generation; it is important to understand the molecular mechanism of the gating. Through tremendous efforts by researchers, including structural analysis, the molecular mechanism has settled on a sliding helix model: the outward sliding of the positively charged S4 helix in the voltage-sensor domain (VSD) opens the activation gate in pore domain (PD) in a voltage-dependent manner. Within this gating model, the VSD has been considered to interact with the PD; however, it is unclear whether the VSDs are always attached to the PD during gating. Furthermore, it is known that there is positive cooperativity between $Na_v$ channels in action potential generation, but it is not clear what kind of interactions between channels cause the positive cooperativity. In this study, we reconstituted three types of $Na_v$Ab channels with different voltage dependence into a lipid membrane and analyzed their structural dynamics by high-speed atomic force microscopy (HS-AFM). Interestingly, in the resting state, the VSDs dissociated from the PD and further dimerized to form a cross-link between the channels. This unexpected conformational change may be the molecular basis of cooperative activation of $Na_v$ channels.

### [1P-098]

### The intracellular C-terminal domain of mGluR6 contains ER retention motifs.

*Atsushi Shimohata[1], Takumi Akagi[1], Ikuo Ogiwara[1], Makoto Kaneda[1] ([1]*Dept Physiol, Nippon Med Sch, Tokyo, Japan*)

Metabotropic glutamate receptor 6 (mGluR6) is predominantly localized at postsynaptic sites of the retinal ON-bipolar cells where this receptor recognizes glutamate released from the photoreceptors. We have previously reported that C-terminal deletions involving a cluster of basic amino acids within the C-terminal domain (CTD) attenuated mGluR6 cell-surface localization and response to glutamate. We herein examined whether the basic residues within the cluster participated in mGluR6 intracellular trafficking, using 293T cells expressing mGluR6 CTD mutants with immunocytochemistry, immunoprecipitation and flow cytometry. We showed that the mGluR6 mutants with 15-, 16-, 19- and 20-amino acid deletions at the C-terminus displayed significant reductions in mGluR6 cell-surface levels, and that their deficiencies in cell-surface localization were rescued by introducing alanine substitution at the basic residues. We also showed that surface-deficiency on the surface-deficient mutant was not improved with co-expression of the surface-expressible constructs, even though the surface-deficient and surface-expressible constructs formed heterodimeric complexes. These observations suggest that the basic residues in the mGluR6 CTD may serve as motifs for ER retention, which can prevent aberrant mGluR6 assemblies from being transported to the cell-surface.

### [1P-099]

### Dimerization of sea urchin Hv1 voltage-gated proton channel is required for its own glycosylation

*Yoshifumi Okochi[1], Jinno Yuka[1], Okamura Yasushi[1,2] ([1]*Graduate School of medicine Osaka University*, [2]*Graduate School of Frontier Bioscience Osaka University*)

Hv1 forms dimer and each protomer permeates proton. However, we still do not know the organelle where Hv1 is dimerized. We show that dimerization of Hv1 takes place in ER. Sea urchin Hv1 (SpHv1) has a consensus sequence N-X-T/S for N-linked glycosylation. We addressed whether the N96 is glycosylated by two ways: One is an enzyme, PNGaseF, which cut N-linked glycans added to protein in ER and the other is a mutant that is substituted N to Q. We found that N96 in SpHv1 is glycosylated. Next, we performed whole cell patch-clamp technique for HEK cells expressing N96Q mutant to see whether the glycosylation is involved in the function of SpHv1. The current density and activation kinetics in N96Q mutant did not differ from that in WT, indicating that glycans of SpHv1 do not affect the channel function. Intriguingly, monomer made by deleting C-terminal cytoplasmic region did not undergo glycosylation. Tandem dimer concatenating each monomer underwent glycosylation. Thus, dimerization of Hv1 is necessary for glycosylation, which suggests that Hv1 is dimerized in ER, leading to glycosylation.

### [1P-100]

### IFN-γ regulates PD-L1 transcription via PYK2 in melanoma

*Fumina Suzuki[1], Yuto Mizuno[1,2], Masanari Umemura[1], Akane Nagasako[1], Rina Nakakaji[1,3], Kagemichi Nagao[1], Soichiro Ishikawa[1,3], Yoshihiro Ishikawa[1] ([1]*Cardiovascular Research Institute (CVRI), Yokohama City University Graduate School of Medicine*, [2]*Department of Environmental Immuno-Dermatology, Yokohama City University Graduate School of Medicine*, [3]*Department of Oral and Maxillofacial Surgery, Yokohama City University Graduate School of Medicine*)

[Introduction] Interferon-γ (IFN-γ) increases the immune checkpoint molecule, programmed death-ligand (PD-L1). PD-L1 suppresses the tumor immunity and enhances tumor growth, in various cancer cell types, including melanoma. However, the mechanism of IFN-γ/ PD-L1 signaling is not fully understood. Our aim is to elucidate this signaling and to explore novel candidate inhibitors in melanoma. [Materials and methods] C8161 cell line (human melanoma) was used. quantitative reverse transcription PCR(q-PCR) was performed. VS-6063 ([Proline-rich tyrosine kinase 2 (PYK2)/ Focal adhesion kinase (FAK) inhibitor)] and PF-573228 (FAK inhibitor) were used. [Results] To explore the candidate genes which regulate the expression of PD-L1, we performed the linear regression analysis using mRNA samples of human melanoma. We found that PYK2 expression was significantly correlated with PD-L1. Therefore, we evaluated the mRNA transcription of PD-L1 in the presence of IFN-γ by q-PCR in C8161. Indeed, IFN-γ promoted the mRNA transcription of PD-L1. VS-6063 negated the IFN-γ-induced mRNA transcription of PD-L1. We also examined whether PYK2 or FAK is involved in this signaling by the used of PF573228. PF573228 did not change the IFN-γ-induced mRNA transcription of PD-L1. [Conclusion] PYK2 plays an important role as the intermediate molecule in IFN-γ-induced PD-L1 transcription.

### [1P-101]

### $PI(4,5)P_2$ regulates human $GABA_AR$ channel activity through K312 of α1 subunit

*Risa Marie Mori-Kreiner[1], Takafumi Kawai[1], Naoyuki Miyashita[2,4], Yasushige Yonezawa[3,4], Yasushi Okamura[1] ([1]*Osaka University, Graduate School of Medicine, Laboratory of Integrative Physiology*, [2]*Department of Computational Systems Biology, Faculty of Biology-Oriented Science and Technology*, [3]*High Pressure Protein Research Center, Institute of Advanced Technology*, [4]*KINDAI University, 930 Nishimitani, Kinokawa, Wakayama 649-6493, Japan*)

$PI(4,5)P_2$ is known to directly regulate the activities of over 50 ion channels. Of note, the recent Cryo-EM structure of the human α1β3γ2L $GABA_A$ receptor ($GABA_AR$) revealed two $PI(4,5)P_2$ bound at the intracellular regions of the α1 subunits. However, their functional significance remains unresolved. Here we present an electrophysiological analysis of $PI(4,5)P_2$ regulation of $GABA_AR$ channel activity in a heterologous expression system (*Xenopus* oocyte). Endogenous $PI(4,5)P_2$ was depleted via voltage-sensing phosphatase (VSP) and the GABA-induced currents were measured using two-electrode voltage clamp (TEVC). We focused on the K312 and R313 of the α1 subunit, which are two residues important for $PI(4,5)P_2$ binding in $GABA_AR$ as suggested by the Cryo-EM structure. The K312N/R313L double mutant's TEVC results demonstrated that the GABA-induced currents were more noticeably reduced than the wild type upon PI(4,5)P2 depletion. In addition, coarse-grained molecular dynamics simulation suggests that K312 is more critical for the $PI(4,5)P_2$ interaction. TEVC results from single-point mutant, K312N, show an even more robust reduction of GABA-induced current after $PI(4,5)P_2$-depletion. Altogether, current results suggest that K312 plays a more important role than R313 in binding to $PI(4,5)P_2$ which regulates $GABA_AR$ channel activity.

### [1P-102]

### Asymmetric manipulation of the lipid bilayer tension revealed an inner leaflet tension dependence in the single TRAAK channel gating

Takahisa Maki[1], Yuka Matsuki[2], Toshiyuki Yoshida[3], Shigetoshi Oiki[4], *Masayuki Iwamoto[1] ([1]*Dept. Mol. Neurosci., Univ. Fukui. Facul. Med. Sci.,* [2]*Dept. Anesth. Reanimatol., Univ. Fukui. Facul. Med. Sci.,* [3]*Dept. Info. Sci., Univ. Fukui. Facul. Eng.,* [4]*Biomed. Imaging Res. Cent., Univ. Fukui*)

Among the two-pore domain potassium (K2P) channels, only TRAAK and its close relatives (TREK-1 and TREK-2) exhibit membrane tension-dependent gating. To date, crystallographic and electrophysiological approaches have been applied to explore their tension dependence molecular mechanisms. However, which part of these channels senses the membrane tension and how the tension governs the gating remains elusive. In this study, we performed the single-channel current analysis using the contact bubble bilayer (CBB) to understand the tension-dependent gating of TRAAK deeply. Manipulation of the CBB tension revealed for the first time the tension vs. activation relation of a single TRAAK channel. Further, the tension of monolayers establishing the CBB was independently manipulated to elucidate the sidedness of the tension acting on the channel. We unequivocally identified that the cytoplasmic leaflet governs the gating of TRAAK. The present methodology using the CBB will also allow for a deeper exploration of the effects of lipid bilayer tension on various ion channels.

### [1P-103]

### Mechanism of voltage-dependent H+ channel activity regulated by membrane stretch and lipids

*Maki Takata[1], Kohei Takeshita[2], Akira Kawanabe[1], Yuichiro Fujiwara[1] ([1]*Faculty of Medicine, Kagawa University,* [2]*RIKEN*)

Voltage-gated H+ channels (Hv), which are responsible for the production of reactive oxygen species (bactericidal effect) in leukocytes, have sensor functions for a wide variety of physicochemical stimuli, including membrane potential, temperature, pH, membrane stretch, and unsaturated fatty acids. It is known that when leukocytes phagocytose and digest pathogens, the phagocytic membrane stretches to envelop the pathogens and arachidonic acid (lipid) is produced, which activates the Hv channel. However, the mechanism of Hv channel activation through these membranes remains unresolved, even with today's advanced understanding of molecular structures. In this study, we approached the mechanism by which Hv channels open in response to phagocyte biomembrane dynamics. We observed by pressure-clamp electrophysiology analysis that the stretch response of Hv channels was dramatically increased by the addition of arachidonic acid. We also observed by microscale thermophoretic analysis that arachidonic acid bound directly to Hv channels. These results highlight the relationship between the stretch response and fatty acids addition and indicate that direct binding of arachidonic acid to Hv channels has the effect of increasing Hv channel activity. In this presentation we would like to discuss the binding affinity of fatty acids (saturated and unsaturated) to Hv channels, their differential regulation of activity against Hv channels, and their relationship to changes in membrane fluidity and stretch response.

### [1P-104]

### The mechanisms of intracellular Ca2+ increase by timosaponin AIII in HeLa cells

*Rin Takahashi[1], Hiroki Takanari[2], Takeshi Terabayashi[3], Toshimasa Ishizaki[3] ([1]*Tokushima University Faculty of Medicine,* [2]*Tokushima University,* [3]*Oita University*)

Timosaponin AIII (TAIII) is a component of crude drugs, which has thermos genic and hypoglycemic effects. In the preliminary study, we found that TAIII increased intracellular $Ca^{2+}$ ($[Ca^{2+}]_i$). In this study, we examined the mechanism of TAIII-induced $[Ca^{2+}]_i$ elevation in detail. HeLa cells were stained with Flou-4 AM for $Ca^{2+}$ imaging using high-speed confocal laser microscopy (Nikon A1R). To determine whether $IP_3$ and ryanodine receptors are involved in $[Ca^{2+}]_i$ elevation due to TAIII, some cells were treated with the respective blockers, 2-APB and ryanodine, in prior to the imaging experiments. Some cells were also subjected to $Ca^{2+}$ imaging under $Ca^{2+}$-free condition to test the effect of the influx of extracellular $Ca^{2+}$. It was shown that 2-APB reduced the speed of TAIII-induced $[Ca^{2+}]_i$ elevation, while ryanodine had no effect on TAIII-induced $[Ca^{2+}]_i$ elevation. In addition, $[Ca^{2+}]_i$ elevation by TAIII was completely inhibited when extracellular $Ca^{2+}$ was removed from the medium. The results indicated that TAIII-induced $[Ca^{2+}]_i$ elevation was triggered by the extracellular $Ca^{2+}$ influx, and was predominantly regulated by $IP_3$ receptors intracellularly.

### Poster Presentation

[1P]

**Molecular physiology, Cell physiology**

**Others**

March 14 (Tue.), 12:10 - 14:10, Poster Room

### [1P-105]

**Decreased maturity of cell-cell contact in senescent vascular endothelial cells**

*Katsuya Hirano[1], Mayumi Hirano[1] ([1]*Department of Cardiovascular Physiology, Faculty of Medicine,Kagawa University*)

Purpose: Our earlier studies suggest that there are different levels of maturity in the cell-cell contact in vascular endothelial cells, which determine the responsiveness to inflammatory stimuli. Disturbed cell-cell contact leads to endothelial dysfunction. We herein elucidate the state of maturity of the cell-cell contact in the senescent endothelial cells. Main findings: In confluent porcine aortic endothelial cells at passages 9-15, 1 unit/mL thrombin decreased trans-endothelial electrical resistance and transiently increased a level of diphosphorylation of myosin light chain (MLC). Di-phosphorylation of MLC was colocalized with actin bundles at the cell periphery 3 min after thrombin stimulation. Later the actin bundles were reorganized into stress fibers. In the cells at early culture days (3 days) or in the confluent cells exposed to the $Ca^{2+}$-free media, thrombin abruptly induced actin stress fiber formation with no transient appearance of peripheral bundles. In the confluent senescent cells at passages 26-30, some actin stress fibers were observed at the basal condition, and thrombin further induced formation of actin stress fibers, with no peripheral bundles. Conclusion: The cell-cell contact of the senescent endothelial cells is suggested to be immature, which might be related to dysfunction of the senescent cells.

### [1P-106]

**Intracellular cGMP dynamics during incretin secretion revealed by red fluorescent protein-based cGMP sensor**

*Yuri Osuga[1], Mai Takizawa[1], Rika Ishida[1], Marie Mita[1], Kazuki Harada[1], Tetsuya Kitaguchi[2], Takashi Tsuboi[1] ([1]*Department of Life Sciences, Graduate School of Arts and Sciences, The University of Tokyo,* [2]*Laboratory for Chemical and Life Science, Institute of Innovative Research, Tokyo Institute of Technology*)

cGMP (cyclic guanosine 3', 5'-monophosphate) is an intracellular second messenger known to be involved in phototransduction and hormone secretion. To visualize the intracellular dynamics of cGMP, green fluorescent protein (GFP)-based cGMP sensors have been developed. However, since most existing sensors utilize GFP, development of red fluorescent protein-based sensors has been desired. One of incretin hormone, glucagon like peptide-1 (GLP-1) is secreted from enteroendocrine cells in the small intestine when they sense nutrients in the gut lumen. Although intracellular $Ca^{2+}$ and cAMP are known to be involved in GLP-1 secretion, the relationship between GLP-1 secretion and cGMP has been unclear. In the study, we have developed a red fluorescent protein-based cGMP sensor, Red cGull. Red cGull can detect intracellular cGMP dynamics as changes in the fluorescence intensity and can be used for dual-color imaging in combination with green $Ca^{2+}$-sensitive dyes or optogenetic tools. GLP-1 secretagogue, L-arginine, was administered to Red cGull expressing enteroendocrine cell line and performed cGMP imaging. We found that L-arginine treatment increased the intracellular cGMP levels via nitric oxide synthase. Taken together, these results suggest that elevated intracellular cGMP concentrations may be involve in GLP-1 secretion.

### [1P-107]

**Protective effect of magnesium on ultraviolet-induced damage of keratinocytes mediated by production of polyamine**

Shokoku Shu[1], Ema Okamoto[1], Shunsuke Matsuda[1], Yuta Yoshino[1], Makiko Goto[2], Yuji Katsuta[2], *Akira Ikari[1] ([1]*Gifu Pharmaceutical Univ.,* [2]*Shiseido Co. Ltd.*)

Magnesium ion ($Mg^{2+}$) has the functions such as acceleration of tissue repair and suppression of inflammatory response in the skin. However, the functional mechanism of $Mg^{2+}$ has not been fully resolved. Microarray data demonstrated that polyamine synthase expression is increased by $Mg^{2+}$ supplementation in human HaCaT keratinocytes. We examined the production mechanism and function of polyamine. The expression level of polyamine synthase was dose-dependently increased by $MgCl_2$ supplementation, which was inhibited by U0126, a MEK inhibitor, CHIR-99021, a glycogen synthase kinase-3 (GSK3) inhibitor, and Naphthol AS-E, a cyclic AMP-response element-binding protein (CREB) inhibitor. In the reporter assay, luciferase activities of polyamine synthases were downregulated by these inhibitors. We suggest that MEK, GSK3, and CREB are involved in the transcriptional regulation of polyamine synthases. The viability of HaCaT cells was decreased by ultraviolet B (UVB) irradiation, which was partially recoverd by $Mg^{2+}$ supplementation. The production of reactive oxygen species was increased by UVB irradiation, which was suppressed by $Mg^{2+}$ supplementation. Our results indicate that the expression of polyamine synthases may be upregulated by $Mg^{2+}$ supplementation mediated by the activation of MEK/GSK3/CREB pathway. Magnesium may be favorable to reduce the risk of UVB-induced damage to the skin.

### [1P-108]

**intra-single cell sequencing (iSCseq) spotlights transcriptomic, epigenetic and differentiational heterogeneity inside multinucleated osteoclast**

*Hiroyuki Okada[1,2], Yuta Terui[3], Yasunori Omata[2,4], Masahide Seki[5], Shoichiro Tani[1,2], Junya Miyahara[2], Kenta Makabe[2], Asuka Terashima[4], Sanshiro Kanazawa[6], Masahiro Hosonuma[7], Shoko Onodera[8], Fumiko Yano[9], Hiroshi Kajiya[10], Saito Taku[2], Yutaka Suzuki[11], Koji Okabe[10], Roland Baron[12], Sakae Tanaka[2] ([1]*Center of Disease Biology and Integrative Medicine, Graduate school of Medicine, the University of Tokyo,* [2]*Department of Orthopaedic Surgery,* [3]*Biotechnology Section 1, Product Strategy Department, Marketing Center, Life Business HQ, Yokogawa Electric Corporation,* [4]*Bone and Cartilage Regenerative Medicine, the University of Tokyo Hospital;,* [5]*Department of Medical Sciences, Graduate School of Frontier Sciences, the University of Tokyo,* [6]*Department of Oral and Maxillofacial Surgery, Graduate School of Medicine, the University of Tokyo,* [7]*Department of Clinical Immuno Oncology, Clinical Research Institute for Clinical Pharmacology and Therapeutics, Showa University; Showa University Pharmacological Research Center,* [8]*Department of Biochemistry, Tokyo Dental College,* [9]*Department of Biochemistry, Showa University School of Dentistry,* [10]*Department of Physiological Science and Molecular Biology, Fukuoka Dental College,* [11]*Department of Computational Biology, Graduate School of Frontier Sciences, the University of Tokyo,* [12]*Department of Oral Medicine, Infection, and Immunity, Harvard School of Dental Medicine,* [13]*Department of Bioengineering, Graduate School of Engineering, the University of Tokyo*)

Multinucleated giant cells like osteoclasts (OCs) often contain >10 nuclei. This raises the question of whether nuclei residing in the same cytoplasm are all regulated similarly or whether they are heterogeneous, expressing a different set of genes regulated by nucleus-specific epigenetic mechanisms. We combined imaging of living cells with confocal microscope, picking of intracellular components inside a single cell using the Single Cellome™ System SS2000 (Yokogawa, Japan), allowing next generation sequencing at high resolution for mRNA (iscRNA-seq) with SMART-Seq Single Cell PLUS kit (Takara bio, Japan) and for DNA methylation (iscWGBS; whole genome bisulfite sequencing) with Pico Methyl-Seq Library Prep Kit (Zymo research, US) of any cellular components including a single OC nucleus. Our results revealed that individual nuclei within the same cell are heterogeneous in terms of gene expression and epigenetic regulation, possibly affecting regional physiological function. Integrative analysis with conventional scRNA-seq showed that many stages of differentiation can coexist in a multinucleated cell.

### [1P-109]

**Relationship between cell cycle and intracellular $Cl^-$ in the mouse fibroblast cell line NIH3T3 cells**

*Miu Kora[1], Hiroaki Miyazaki[1] ([1]*Setsunan UniversIity*)

In general, the cancer microenvironment around the primary tumor is assumed to have a high partial pressure of $CO_2$ due to the high metabolism of cancer cells. Since carbonic anhydrase that convert $CO_2$ and $H_2O$ to $HCO_3^-$ and $H^+$ are highly expressed in tumor cells, large fluctuations in $HCO_3^-$ concentration can be expected in the cancer microenvironment. $HCO_3^-$ and $Cl^-$ can be easily exchanged via anion exchange transporters, so the cells constituting the cancer microenvironment are affected by these large intracellular $Cl^-$ fluctuations. Therefore, the purpose of this experiment was to investigate the effect of intracellular $Cl^-$ on the mouse fibroblast cell line, NIH3T3.The results of a proliferation assay of NIH3T3 cells showed that cell proliferation was completely blocked when NIH3T3 cells were cultured in the low $Cl^-$ concentration media (replacement of NaCl with $NaNO_3$). To confirm whether cell cycle progression is inhibited in the low $Cl^-$ concentration media, cell cycle analysis was performed. Results of cell cycle analysis showed that there were no significant differences in cell cycle profile patterns between cells cultured in normal or low $Cl^-$ media. We also found that the exposure of the cells to low $Cl^-$ concentration media elevated protein expression of p21 and phosphorylation of cdc2, and reduced EdU incorporation. These results suggest that cell cycle progression was arrested at all cell cycle stages. These observations suggest that the change in intracellular $Cl^-$ concentration play an important role in control of cell proliferation.

### [1P-110]

### Apoptosis-induced decline of intracellular $Cl^-$ concentration activates apoptotic signaling pathway in MDA-MB231 breast cancer cells

*Mizuki Sada[1], Hiroaki Miyazaki[1] ([1]*Setsunan Univ.*)

Apoptotic cell death is performed using a programed signaling pathway, accompanied apoptotic volume decrease (AVD) during induction of apoptosis. AVD is induced by activation of the volume-sensitive outwardly rectifying (VSOR) anion channel and $K^+$ channels, which cause effluxes $Cl^-$, $K^+$ and $H_2O$, leading to cell volume decreases. This efflux of $Cl^-$ and $K^+$ may induce the reduction of intracellular $Cl^-$ concentration, which is possibly affect the signal pathway of apoptosis. Therefore, the aim of this study was to investigate whether the decline in intracellular $Cl^-$concentration is involved in the activation of the apoptotic signaling cascade using MDA-MB231 breast cancer cells. First, we investigated the effect of intracellular $Cl^-$ on staurosporine (STS)-induced activation of caspase-3 and externalization of phosphatidylserine (PS) on the cell plasma membrane, which were observed in the early stage of apoptosis. As a result, the activation of caspase-3 and the externalization of PS were detected earlier in cells cultured in the low $Cl^-$ medium than those in the normal $Cl^-$ medium after induction of apoptosis with STS. These results suggest that the intracellular $Cl^-$ functions as an important regulator of apoptotic signaling pathways. Based on these results, we are investigating the effect of intracellular $Cl^-$ on the apoptotic events in the AVD prevented cells by blocking volume-regulatory $Cl^-$ and $K^+$ channels to elucidate the role of $Cl^-$ in apoptotic signaling pathway.

### [1P-111]

### Monoacylglycerol acyl transferases (MGATs) mediate cool temperature avoidance via regulation of gene expression of ionotropic receptors in *Drosophila* larvae

*Xiangmei Deng[1,2,3], Takuto Suito[2,3], Makoto Tominaga[1,2,3], Takaaki Sokabe[1,2,3]
([1]*Department of Physiological Sciences, SOKENDAI, Hayama, Japan,* [2]*Division of Cell Signaling, National Institute for Physiological Sciences, Okazaki, Japan,* [3]*Thermal Biology Group, Exploratory Research Center on Life and Living Systems (ExCELLS), Okazaki, Japan*)

Elucidating mechanisms underlying thermotaxis of animals is essential for coping with recent extreme climate changes. In *Drosophila* larvae, ionotropic receptor genes (*Irs*) expressed in dorsal organ cool cells (DOCCs) are involved in aversion to innocuous cool temperatures. Recent studies showed that exogenous modification of lipids in DOCCs altered thermal preference in *Drosophila*, however, physiological components potentially involved in this regulation have not been investigated. Here, we report that genes encoding MGAT regulate thermal preference. Compared with control larvae that preferred 24°C, *MGAT-2* and *MGAT-3* knock out (KO) larvae did not discriminate 20°C and 24°C in a two-way choice assay. Knocking down of *MGAT-2* in DOCCs was sufficient to recapitulate the thermotactic phenotype. The defect in cool temperature avoidance was compensated by overexpressing *MGAT-2* or human MOGAT2 in DOCCs in the *MGAT-2* KO background . We also observed that cooling-induced response in the DOCCs was reduced in the absence of *MGAT-2*, and such reduction could be caused by downregulation in the expression level of multiple *Irs* in DOCCs. Our findings provide a line of evidence for the physiological importance of lipid metabolic enzymes in the thermotaxis of animals.

### [1P-112]

### SREBP1-mediated *GJA5* expression in cholesterol excess and depletion.

*Naoya Kanada[1], Hiroki Takanari[1] ([1]*Tokushima University*)

*Gap junction alpha-5 protein* (*GJA5*) is selectively expressed in atrial myocytes. *GJA5* encodes Connexin 40, a component of GAP junction that transports molecule and electronic signals between cells, and plays a significant role in cell physiology. We hypothesized that excess or depletion of cholesterol may affect *GJA5* expression via Sterol regulatory element-binding protein1 (SREBP1), which is involved in cholesterol metabolism, and conducted dual-luciferase reporter assay. *GJA5* promoter (-1209 bp to +275 bp) reporter vector, SREBP1 expression vector and control reporter vector were transfected to HeLa cells treated with Methyl-β-cyclodextrin (MβCD) or cholesterol-saturated MβCD for 3 hours to deplete or excess intracellular cholesterol concentration. In a luciferase assay preformed 24 hours after transfection, *GJA5* promoter activity increased when cholesterol was depleted for 3 hours, while the same duration of cholesterol excess did not change in activity compared to control. These results indicated that SREBP1 was not involved in *GJA5* expression in 3-hour cholesterol excess, while SREBP1-mediated *GJA5* expression pathway responded quickly and sensitively to cholesterol depletion.

### [1P-113]

### Intracellular $Cl^-$ affects cell migratory ability and formation of filopodia in esophageal cancer-derived cell line TE-5

*Sasuga Otonari[1], Hiroaki Miyazaki[1] ([1]*Setsunan university*)

Sodium and chloride ions ($Na^+$ and $Cl^-$) are the most abundant ions in body fluids. However, the physiological functions of $Cl^-$ are less well known than those of $Na^+$. Therefore, we focused on the physiological function of $Cl^-$ and found that $Cl^-$ is an important regulator of the growth of cancer, a familiar disease in recent years. In this study, we decided to investigate whether intracellular $Cl^-$ also affects migration capacity, another characteristic feature of cancer, in esophageal cancer-derived cell line TE-5. First, we observed the effect of intracellular $Cl^-$ on migration ability by the wound-healing and the transwell migration assays. The results of the wound-healing assay showed that cell migration was declined in TE-5 cells under $Cl^-$ conditions. Interestingly, cell migration ability of TE-5 cells was significantly enhanced in the low $Cl^-$ condition as measured by transwell migration assay. The formations of filopodia, which are involved in cell migration, also decreased as measured by the wound-healing assay and did not change as measured by the transwell migration assay under low $Cl^-$ conditions. These results strongly suggest that the effect of intracellular $Cl^-$ on filopodia mediated migration ability differs under chemotactic (transwell migration assay) or non-chemotactic (wound healing assay) conditions. Since the Rho family GTPase Rac and Cdc42 are well known to control cell migration and formation of filopodia, we are investigating the relationship between the intracellular $Cl^-$ and the Rho family GTPase.

### [1P-114]

### Proteomic analysis of the metabolic pathway involved in the developmental process of cardiac primordium just after heartbeat initiation to primordial heart tube formation in rats.

*TAKURO KARAUSHI[1], Tatsuya Sato[1], Nobutoshi Ichise[1], Hiroyori Fusagawa[1], Taiki Kudo[1], Noritsugu Tohse[1] ([1]*Sapporo Medical University*)

Backgrounds: We recently found that the rat heart primordium starts beating at around embryonic day 10.0 (E10.0) and that the energy required to initiate the heartbeat is supported by HIF-1α-mediated enhancement of glycolysis. Although the heart primordium requires more energy during the development to the primordial heart tube formation while maintaining beating, underlying molecular characteristics remain unknown. Methods: The heart primordium at E10.0 after heartbeat initiation and the primordial heart tube at E11.0 were isolated from rat embryos and were subjected to data-independent acquisition mass spectrometry (DIA-MS) to identify proteins that changed more than 1.5-fold between the two groups. Results: A total of 8,514 proteins were identified by DIA-MS and of those, 1,293 proteins were significantly upregulated and 1,724 proteins were significantly downregulated in the primordial heart tube compared to the heart primordium. Among upregulated proteins, the top three enriched gene ontology biological processes were energy metabolism, energy derivation by oxidation, and circulatory system development, In the pathway enrichment analysis, the pathway of citrate cycle ranked as the highest pathway in the up-regulated proteins. Conclusions: The results indicate that proteins involved in energy metabolism, especially in the citrate cycle, are predominantly upregulated during the developmental process from the heart primordium after heartbeat initiation to primordial heart tube formation.

### Poster Presentation

[1P]
**Muscle**

March 14 (Tue.), 12:10 - 14:10, Poster Room

### [1P-115]

### Receptor subunit compositions underly distinct potencies of a muscle relaxant in fast and slow muscle fibers

*Manami Yamashita[1], Yoshihiro Egashira[1], Shuntaro Nakamura[1], Souhei Sakata[1], Fumihito Ono[1] ([1]*Osaka Medical and Pharmaceutical University*)

A line of studies in the1960s-1980s suggested that muscle relaxants do not work uniformly on all skeletal muscles, though its mechanism has not been clarified. We showed here that a classical non-depolarizing muscle relaxant pancuronium inhibits fast muscle fibers at lower concentration compared to slow muscle fibers in zebrafish. The difference of effective concentration was observed in locomotion caused by tactile stimulation as well as in synaptic currents of the neuromuscular junction induced by motor neuron excitation. We further showed that this difference arises from the different composition of acetylcholine receptors between slow and fast muscle fibers in the neuromuscular junction of zebrafish. It will be interesting to examine the difference of subunit composition and sensitivity to muscle relaxants in other species.

### [1P-116]

### Effect of Postsynaptic receptors, beta-adrenergic receptor and CGRP receptor, on expression level of myosin heavy chain class IIa ($MyHCII_a$) mRNA in murine skeletal myocytes

*Junko Yamaji[1], Reiko Hiroshima[2], Yoshiaki Mori[2] ([1]*Dept. of Nutr. Sci., Kansai Univ. of Welf. Sci.,* [2]*Dept. of Rehab., Kansai Univ. of Welf. Sci., Kashiwara*)

Postsynaptic receptors, beta-2 adrenergic receptor and calcitonin gene related peptide (CGRP) receptor, on the sarcolemma at neuromuscular junctions receive neurotransmitters from motor nerves and also stimulates activates adenylate cyclase pathway in skeletal muscle cells. However, the roles of these receptors and the cAMP-PKA pathway on mRNA expression of MyHC and myokine including interleukin (IL) -6 in skeletal muscle remains elusive. In the present study, we examined that these agonists, catecholamine and CGRP, on mRNA levels of $MyHCII_a$ and myokine in mouse skeltal muscle cells. The results from our study are as follows: The mRNA level of IL-6 was significantly upregulated by supplemented with calcineurin activatiors, but was not also affected by medium supplemented with forskolin, with beta-2 agonists and with PKA inhibitor. (2) The mRNA level of $MyHCII_a$ was significantly increased induced by calcineurin activatiors but not by IL-6, and was significantly attenuated by calcineurin inhibitor. (3) The $MyHCII_a$ mRNA level was decreased by medium supplemented with CGRP. (4) The $MyHCII_a$ mRNA level was elevated by medium supplemented with cAMP and with beta-2 agonist, and inhibited with CREB inhibitor. These results suggested that cAMP pathway is affected for $MyHCII_a$ mRNA level and that CGRP pathway and IL-6 is not affected for the mRNA level in C2C12 cells.

### [1P-117]

### Effect of high-intensity interval training using electrical stimulation on mitochondrial dysfunction and muscle fatigue in 5/6 nephrectomy rat model of chronic kidney disease

*Hiroyori Fusagawa[1,2], Takashi Yamada[3], Tatsuya Sato[1,4], Yuki Ashida[3], Azuma Naito[3], Nao Tokuda[3], Nao Yamauchi[3], Nobutoshi Ichise[1], Takuro Karaushi[1], Atsushi Teramoto[2], Toshihiko Yamashita[2], Noritsugu Tohse[1] ([1]*Department of Cellular Physiology and Signal Transduction, School of Medicine, Sapporo Medical University,* [2]*Department of Orthopaedic Surgery,* [3]*Graduate School of Health Science,* [4]*Department of Cardiovascular, Renal and Metabolic Medicine, Sapporo Medical University School of Medicine*)

Background: Mitochondrial dysfunction in skeletal muscle has recently been highlighted as a major pathophysiological factor of chronic kidney disease (CKD)-associated muscle fatigue. High-intensity interval training (HIIT) is known for its effectiveness in improving mitochondrial function, but due to the high-intensity load of whole-body exercise, its use has been limited to specific targets such as athletes. We have recently developed a HIIT application system that utilizes electrical stimulation of local muscles and have verified its effectiveness. Here we examined the effects of HIIT using electrical stimulation (ES) intervention on skeletal muscle in CKD rats. Methods and Results: CKD was induced by 5/6 nephrectomy (Nx) in male Wistar rats, and sham-operated rats served as controls (Sham). We defined the left leg of Nx rat trained with HIIT as the Nx-HIIT leg and the untrained right leg as the Nx-CNT leg. HIIT was enforced by electrically stimulating plantar flexor muscles every other day for 4 weeks. *In* vivo muscle endurance analysis using repeated fatiguing stimulation measured 24h after the last training session showed a significantly greater force depression in the Nx-CNT leg compared to that in the Sham leg, which was significantly improved in the Nx-HIIT leg. Pyruvate/malate-driven ADP- and uncoupler-stimulated mitochondrial respiration in isolated muscle mitochondria from the Nx-CNT leg assessed by Seahorse XFe96 analyzer were significantly decreased compared with those from the Sham leg, which were also significantly recovered in those of the Nx-HIIT leg. The maximal activities of citrate synthase, biomarker of mitochondrial content, measured spectrophotometrically using whole muscle homogenates showed a significant decrease in the Nx-CNT leg compared to that in the Sham leg, which was significantly repaired in the Nx-HIIT leg. Consistent with improved mitochondrial function and amount, myosin heavy chain (MyHC) electrophoresis showed a significant increase in the proportion of fast-twitch MyHC IIB fibers in the Nx-CNT leg compared to that in the Sham and the Nx-HIIT leg. Conclusions: HIIT with ES improved muscle fatigue and mitochondrial function and content in a rat model of CKD-associated cachexia. HIIT with ES may lead to an innovative therapeutic intervention that can be utilized for patients with CKD-associated cachexia who have difficulty with full-body exercise.

### [1P-118]

### The mechanistic target of rapamycin inhibition suppresses mitochondrial protein expressions and respiratory capacity in skeletal muscle cells

*Kazuki Uemichi[1], Takanaga Shirai[2], Tohru Takemasa[2] ([1]*Graduate School of Comprehensive Human Sciences, University of Tsukuba,* [2]*Faculty of Health and Sport Sciences, University of Tsukuba*)

The mechanistic target of rapamycin (mTOR) is a well-known serine-threonine kinase that activates muscle protein synthesis in response to anabolic conditions after exercise or nutrition intake. Rapamycin, a canonical inhibitor of mTOR complex 1 (mTORC1), induces mitochondrial dysfunction in addition to attenuating the muscle protein synthesis response. These findings suggest that mTORC1 contributes to mitochondrial homeostasis, but the involvement of mTOR complex 2 (mTORC2) and the relationship between mTORC1 and mTORC2 are unclear. The purpose of this study was to determine the role of the mTOR complex on mitochondrial function in skeletal muscle cells using AZD8055, as an ATP-competitive mTOR inhibitor that inhibits both mTORC1 and mTORC2, and rapamycin. AZD8055 treatment more significantly reduced the content of proteins associated with mitochondrial oxidative phosphorylation, fusion, fission, and decreased respiratory capacity than rapamycin treatment. In conclusion, we observed that mTORC1 and mTORC2 cooperatively regulate mitochondrial protein expressions and respiratory function in skeletal muscle cells.

### [1P-119]

### Tension responses to pure water in the guinea pig taenia caecum

*Yukisato Ishida[1], Masaru Watanabe[1], Naoya Nakahara[2], Shigeru Takemori[2] ([1]*Graduate School of Human Health Sciences, Tokyo Metropolitan University,* [2]*Department of Molecular Physiology, Jikei University School of Medicine*)

With respect to effects of water on muscle contractile system, Kühne (~1860) already reported a precipitation of muscle proteins induced by distilled water, now known as actomyosin precipitation at low ionic strength. Muscle contractile responses to water were also reported (Herman, 1879; Asano, 1916; Noguchi et al., 1955). Natori (1954) reported distilled water produced a contractile response of Natori's fiber, which was recently confirmed by us. Here we present the properties of contractile responses to pure water ($H_2O$) in smooth muscle of the guinea pig taenia caecum. When the taenia was properly stretched, the exposure to $H_2O$ reproducibly elicited a sustained tension with the magnitude comparable with that induced by high-$K^+$. The tension response to $H_2O$ was not affected by the presence of sucrose 100-300 mM, suggesting the osmolality independent mechanism. The presence of mono- or divalent cation reduced the $H_2O$-tension. In mono-valent ions, $Li^+$, $Na^+$ $K^+$ and $NH4^+$, reduction in tension was prompt and the amount of reduction was concentration-dependent with ED50 ~3 mM. Divalent ions, $Mg^{2+}$, $Ca^{2+}$, $Cu^{2+}$ and $Cd^{2+}$, also reduced the tension to $H_2O$. Their low concentration, 0.1 mM, caused a gradual reduction to 20% of tension in 15-45 min exposure, and the rate of reduction was concentration-dependent. $H^+$, though mono-valent, behaved like divalent cations. Results suggest that the simple reduction in ionic strength may not attribute to the $H_2O$-induced tension, since the manner and concentration in tension reduction were markedly different between mono- and divalent cations. (COI: NO)

### [1P-120]

### Study of zebrafish lacking voltage-gated sodium channels in muscles

*Souhei Sakata[1], Chifumi Akiyama*(Terai)*[1], *Fumihito Ono*[1] ([1]*Dept. Physiology, Faculty of Medicine, Osaka Medical and Pharmaceutical University*)

Extensive studies over years established that the action potential(AP) in muscle is indispensable for muscle contraction. To reexamine the significance of the AP, we generated zebrafish lacking APs by editing scn4aa and scn4ab genes encoding Nav1.4 (NavKO) by CRISPR-Cas9 system. Surprisingly the escape response and the elevation of the cytoplasmic $Ca^{2+}$ ($[Ca^{2+}]_i$) in NavKOs were not distinct from WT fish. The mathematical simulation showed that the end plate potential was able to elicit membrane potential change large enough to stimulate the dihydropyridine receptor, which spread over the entire muscle fiber because of its small size. Our data demonstrate that the AP is not essential for the muscle contraction in zebrafish larvae. Some reports showed that invertebrates lack the AP in muscle. Our PCR experiments confirmed that sodium channels are expressed in the muscle of the lamprey and the *polypterus senegalus*. It is known that the whole genome was duplicated in the process of evolution from invertebrates to vertebrates, supporting the idea that one of sodium channel gene duplicons evolved to express in the muscle, which gave rise to the AP in vertebrate muscle cells.

### [1P-121]

### Effects of endurance training on exercise performance and muscle metabolic enzyme activity in orchiectomized mice

*Kenya Takahashi[1], Yu Kitaoka[2], Hideo Hatta[1] ([1]*The University of Tokyo,* [2]*Kanagawa University*)

This study investigated whether endurance training attenuates orchiectomy (ORX)-induced metabolic alterations. Male ICR mice underwent sham operation or ORX surgery. On day 7 of recovery, the mice were randomized to remain sedentary or undergo 5 weeks of treadmill running training (15–20 m/min, 60 min, 5 days/week). During week 5 of the training, all animals performed a treadmill running test (15 m/min, 60 min). ORX reduced glycogen level in the gastrocnemius muscle, increased phosphofructokinase activity in the plantaris muscle, and decreased lactate dehydrogenase activity in the plantaris and soleus muscles. Mitochondrial enzyme activities and protein content in the plantaris and soleus muscles were also decreased after ORX, but preserved, in part, by endurance training. In the treadmill running test, orchiectomized sedentary mice showed impaired exercise performance, which was restored by endurance training. The present findings suggest that endurance training would be a potential therapeutic strategy to counteract the hypoandrogenism-induced decline in muscle mitochondrial content and physical performance.

### [1P-122]

### Synchrony of sarcomere dynamics in the *in vivo* beating mouse heart

*Fuyu Shimozawa Kobirumaki[1], Togo Shimozawa[2], Kotaro Oyama[3], Takako Terui[4], Shin'ichi Ishiwata[5], Norio Fukuda[1] ([1]*Department of Cell Physiology, The Jikei University School of Medicine,* [2]*Technical Division, School of Science, The University of Tokyo,* [3]*National Institutes for Quantum Science and Technology,* [4]*Department of Anesthesiology, The Jikei University School of Medicine,* [5]*Department of Physics, Faculty of Science and Engineering*)

In the present study, we investigated the dynamic behaviors of sequentially connected individual sarcomeres along a myofibril in a left ventricular myocyte of the *in vivo* beating mouse heart by expressing α-actinin-AcGFP in Z-disks (spatial resolution, 10 nm at 100 fps). To analyze the contribution of individual sarcomeres to myofibrillar dynamics, the novel parameter “contribution index” (CI) was introduced to quantify the synchrony in movements between a sarcomere and a myofibril (from −1 [complete asynchrony] to 1 [complete synchrony]). First, during normal systole, CI varied markedly between sarcomeres, with an average value of ~0.3. Second, when the movements between adjacent sarcomeres were asynchronous (CI<0), a sarcomere and those next to the adjacent sarcomeres and farther away moved in synchrony (CI>0) along a myofibril. Third, a linear relationship (R=0.93; P<0.01) was observed between CI and left ventricular pressure, in the range between ~136 and ~5 mmHg. Fourth, following moderate reduction (~30%) of left ventricular volume by inferior vena cava clamping, the sarcomere length was transiently shortened with increased inhomogeneity, but CI was barely changed. These findings suggest that sarcomeres heterogeneously contribute to myofibrillar dynamics due to an imbalance of active and passive force between neighboring sarcomeres, and (2) sarcomere synchrony via the distal inter-sarcomere interaction regulates the heart’s pump function in coordination with myofibrillar contractility.

### [1P-123]

### Effects of omecamtiv mecarbil on $Ca^{2+}$ sensitivity in skinned porcine left atrial and ventricular muscles

*Tomohiro Nakanishi[1,2], Kotaro Oyama[3], Hiroyuki Tanaka[4], Fuyu Shimozawa Kobirumaki[1], Shuya Ishii[3], Takako Terui[2], Shin'ichi Ishiwata[5], Norio Fukuda[1] ([1]*Department of Cell Physiology, The Jikei University School of Medicine,* [2]*Department of Anesthesiology, The Jikei University School of Medicine,* [3]*National Institutes for Quantum Science and Technology,* [4]*Laboratory of Marine Biotechnology and Microbiology, Graduate School of Fisheries Sciences, Hokkaido University,* [5]*Department of Physics, Faculty of Science and Engineering, Waseda University*)

Omecamtiv mecarbil (OM) is a novel inotropic agent for heart failure with systolic dysfunction. OM prolongs the actomyosin attachment duration, which enhances thin filament cooperative activation and accordingly promotes the binding of neighboring myosin to actin. We investigated the effects of OM on $Ca^{2+}$ sensitivity in skinned porcine left ventricular (PLV) and atrial (PLA) muscles. OM increased $Ca^{2+}$ sensitivity in a concentration-dependent manner in PLV, by left shifting the mid-point ($pCa_{50}$) of the force-pCa curve ($\Delta pCa_{50}$) by ~0.16 and ~0.33 pCa units at 0.5 and 1.0 μM, respectively. The $Ca^{2+}$-sensitizing effect was likewise observed in PLA, but less pronounced with $\Delta pCa_{50}$ values of ~0.08 and ~0.22 pCa units at 0.5 and 1.0 μM, respectively. The effect of OM (1.0 μM) was attenuated under enhanced thin filament cooperative activation in both PLV and PLA; this attenuation occurred directly via treatment with fast skeletal troponin ($\Delta pCa_{50}$: ~0.16 and ~0.10 pCa units in PLV and PLA, respectively) and indirectly by increasing the number of strongly bound cross-bridges in the presence of 3 mM MgADP ($\Delta pCa_{50}$: ~0.21 and ~0.08 pCa units in PLV and PLA, respectively). This attenuation of the $Ca^{2+}$-sensitizing effect of OM is presumably coupled with a decrease in the number of “recruitable” cross-bridges that can potentially produce active force. These findings suggest that the positive inotropy of OM is more markedly exerted in the ventricle than in the atrium, which results from the strongly bound cross-bridge-dependent allosteric activation of thin filaments.

### [1P-124]

### Preventive effects of hyaluronic acid upregulated in the muscle after exercise on the development of delayed onset muscle soreness

*Hiroki Ota[1,2], Katsuyuki Tsuboshima[3], Toru Taguchi[1,2] ([1]*Dept. Phys. Ther., Fac. Rehabil., Niigata Univ. Health Welfare,* [2]*Inst. Human Move. Med. Sci., Niigata Univ. Health Welfare,* [3]*Dept. Judo Ther., Fac. Med. Tech., Teikyo Univ.*)

Repetition of the same exercise prevents the development of delayed onset muscle soreness (DOMS). The mechanical adaptation, also referred to as the “repeated bout effect”, is important for maintaining and improving muscle function. However, the molecular basis has not been fully elucidated. In this study, we investigated roles of hyaluronic acid (HA), which is located in the extracellular matrix surrounding muscle cells, for preventing the development of DOMS. Under isoflurane anesthesia, rats were given lengthening contractions (LC) to the lower leg extensor muscles. Immunohistochemical labeling of HA-binding protein revealed that the expression level of HA was upregulated 24 h after LC. Intramuscular injection of HA 24 h prior to LC prevented the development of DOMS. Activities of C-fiber muscle nociceptors decreased by the prior intramuscular injection of HA. Taken together, these results suggest that HA upregulated in the muscle after LC could prevent the development of DOMS. This work was supported by JSPS KAKENHI (JP19H03987 and JP20K11246), and partly by the AMED Grant (JP21gm0810010h0606). There were no conflicts of interest in this study.

### [1P-125]

### Seasonal effects of muscle hypertrophy and metabolism in Mangalica.

*Chisato Nakayama[1], Sangwoo Kim[1], Tatsuki Okazaki[1], Erina Yoneda[1], Kisaki Tomita[1], Daisuke Kondoh[1], Yuki Muranishi[1] ([1]*Obihiro University of Agriculture and Veterinary Medicine*)

**[1P-126]**

**Withdrawn**

### Poster Presentation

[1P]
**Digestion, Digestive system**

March 14 (Tue.), 12:10 - 14:10, Poster Room

### [1P-127]

**Lipopolysaccharide accelerates peristalsis by promoting glucagon-like peptide-1 secretion in the rat proximal colon**

*Hiroyuki Nakamori[1], Atsuko Niimi[1], Hikaru Hashitani[1] ([1]*Department of Cell Physiology, Nagoya City University Graduate School of Medical Sciences, Nagoya, Japan.*)

In an intestinal injury model, lipopolysaccharide (LPS) stimulates glucagon-like peptide-1 (GLP-1) secretion from enteroendocrine L cells by activating toll-like receptor 4 (TLR4). Since GLP-1 accelerates peristalsis in the rat proximal colon, effects of LPS on colonic peristalsis were investigated. Isolated segments of rat proximal colon were serosally perfused with oxygenated Krebs solution and luminally perfused with disgassed 0.9% saline. Colonic wall motion was recorded using a video camera and converted into spatio-temporal maps. Fluorescence immunohistochemistry was also carried out to visualize TLR4 or GLP-1 expression. Intraluminal but not serosal administration of LPS (1 μg/mL) increased the frequency of oro-aboral propagating peristaltic contractions in a TLR4 antagonist (TAK-242)-sensitive manner. The LPS-induced acceleration of colonic peristalsis was blocked by luminally-applied exendin-3, a GLP-1 receptor antagonist. In aspirin-pretreated preparations in which gut epithelial barrier function had been impaired, a lower dose of LPS (0.1 μg/mL) that failed to accelerate peristalsis in normal preparations became capable of increasing the frequency of peristalsis. TLR4 immunoreactivity was co-localized with GLP-1-positive epithelial cells. In conclusion, luminal LPS promotes GLP-1 secretion via the activation of TLR4 on L cells resulting in the acceleration of colonic peristalsis.

### [1P-128]

**Dysmotility of gastric wall induced by high salt fed**

*Masumi Eto[1], Yohei Mochizuki[1], Toshiyasu Matsui[1], Minami Ohashi[1], Yuki Shimojima[1], Shuichi Katsuki[1], Kosuke Takeya[1], Yoshinori Tanaka[1], Risuke Mizuno[1]
([1]*Okayama University of Science*)

Dietary salt intake has been linked to a high prevalence of hypertension and overall cardiovascular and renal risks, although its effect on digestive system has yet to be fully evaluated. Gastric smooth muscle contraction is governed by $Ca^{2+}$-dependent phosphorylation of myosin regulatory light chan (LC20). The extent of LC20 phosphorylation is defined by $Ca^{2+}$ signaling as well as the $Ca^{2+}$ sensitization/de-sensitization signaling, which are mediated by ROCK, PKC, PKA, and PKG. Here we determined impacts of dietary salt intake on gastric motility in mice and on the $Ca^{2+}$ sensitization signaling in smooth muscle. High salt diet resulted in a deformation of stomach with an extended fundus (forestomach), which was associated with a loss of motility of pyloric walls. In the extended fundus, each smooth muscle cell was highly elongated without sign of apoptosis. High salt diet conferred an enhanced sensitivity to inhibitors against ROCK, PKC and PDE on smooth muscle strips from fundic walls, which was associated with an increased ROCK2 expression. High salt diet may alter the excitation-contraction couplings of gastric smooth muscle due to the disturbed $Ca^{2+}$ sensitization signaling.

### [1P-129]

**Measurement of SGLT1 and CFTR activity in stem-cell derived mouse enteroids**

*Noriko Ishizuka[1], Yuka Suzuki[1], Nozomi Nagata[1], Hisayoshi Hayashi[1] ([1]*University of Shizuoka*)

The small intestinal epithelium has a function of nutrient absorption and NaCl secretion. These functions are performed by intestinal stem cells localized in the crypts, which differentiate into intestinal cells with the respective functions. It is also thought that the crypt has a Cl secretory function and the villi has a nutrient absorption function. However, it was difficult to distinguish in crypt and villi function using tissues isolated from animals. It has been reported that only spherical small intestinal stem cells can be cultured in 3D and further differentiated into cells with small intestine-specific functions. In this study, we used these techniques to culture stem cells in 2D on Transwell insert, induce further differentiation, and measure their functions. The small intestine was harvested from mice, and the crypts containing stem cells were isolated. Microenvironmental factors necessary for stem cell passaging were added to the medium and stem cells were cultured in Matrigel. To create a monolayer epithelium, spherical groups of stem cells were treated with trypsin and seeded onto Matrigel-coated Transwells insert. After 3 days, the culture medium was replaced with differentiation medium to induce differentiation. mRNA expression related to transport function was observed before and after induction of differentiation. The number of transporters related to nutrient absorption increased in response to differentiation induction. However, CFTR did not change significantly before and after differentiation. SGLT1 and CFTR activities before and after induction of differentiation were analyzed electrophysiologically using the Ussing chamber method. SGLT1 activity could not be measured before induction of differentiation, but Cl secretion activity could be measured.

### [1P-130]

**Regulations of transepithelial ion transport and permeability by enteric nervous system in mice small intestine**

*Shin-Ichiro Karaki[1], Mao Ikeya[1], Yuichi Suzuki[1,2] ([1]*University of Shizuoka,* [2]*Sendai Seiyo Gakuin College*)

Intestinal epithelia have two contradictory functions; one is substance transport for nutrient absorption, and the other is barrier function for prevention of harmful substance invasion. To conduct both functions, the barrier function must be regulated according to circumstances in the luminal environment. Enteric nervous system (ENS) is known to regulate intestinal motility and mucosal secretion by chemical and mechanical stimulations; therefore, we have hypothesized that ENS also regulates the epithelial barrier function. In the large intestinal mucosa-submucosa preparations including intact submucosal plexus (SMP) mounted on the Ussing chamber, electrical field stimulation (EFS) to SMP has been generally reported to induce simultaneous increases in short-circuit current (Isc) as an index of fluid secretion and tissue conductance (Gt) as an index of ion permeability. However, different from large intestine, we found that, in small intestine, EFS induced an increase in Isc but a decrease in Gt. Moreover, EFS- and luminal heat-stable enterotoxin b (STb)-induced decreases in Gt were tetrodotoxin (TTX) insensitive, so that it might involve TTX-resistant voltage-gated sodium (NaV) channel activities. These results suggest that the ENS regulates to increase the barrier functions of small intestinal epithelia as neural reflexes involved in the TTX-resistant NaV by sensing harmful luminal conditions.

### [1P-131]

**Study on the effect of Bisphenol A in colorectal cancer based on network toxicology**

*Muhamad Fikri Shazlan Saad[1], Yoke Keong Yong[1] ([1]*Department of Human Anatomy, Faculty of Medicine and Health Sciences, Universiti Putra Malaysia*)

Colorectal cancer (CRC) is the second largest cause of death due to cancer worldwide. However, the effects of environmental chemicals on CRC may be understated due to a lack of knowledge about interacting genes and tumours. So, the current study aimed to predict the interaction between Bisphenol A (BPA) and CRC through a network toxicology approach. The interactive genes of BPA were obtained from Swiss Target Prediction and CompTox Chemicals Dashboard databases. The CRC interactive genes were retrieved from Online Mendelian Inheritance in Man, DisGeNET and GeneCards databases. The intersection genes between BPA and CRC were identified, and the protein-protein interaction network had 156 nodes and 1182 edges. The gene ontology analysis indicated that the biological processes were correlated with the transcription activation from the RNA polymerase II promoter (p = 3.31E-23). Meanwhile, Kyoto Encyclopedia of Gene and Genomes pathway analysis found that the interactive genes were enriched in pathways in cancer, chemical carcinogenesis - receptor activation, estrogen signalling pathway, and CRC. Thus, we predict that BPA can promote the development of cancer in the colon.

### Poster Presentation

### [1P]
### Oral physiology

March 14 (Tue.), 12:10 - 14:10, Poster Room

### [1P-132]

**Effects of the contagion of social defeat stress on masseter muscle nociception in male mice**

*Kajita Piriyaprasath[1,3], Mana Hasegawa[1,2], Noritaka Fujii[2], Kensuke Yamamura[1], Keiichiro Okamoto[1] ([1]*Division of Oral Physiology, Graduate School of Medical and Dental Sciences, Niigata University, Niigata, Japan,* [2]*Division of General Dentistry and Dental Clinical Education Unit, Graduate School of Medical and Dental Sciences, Niigata University, Niigata, Japan,* [3]*Department of Restorative Dentistry, Faculty of Dentistry, Naresuan University, Phitsanulok, Thailand*)

Psychological stress spreads like an infectious disease across social networks in a phenomenon called stress contagion. This study determines whether cohabitation with a mouse subjected to Social Defeat Stress (SDS) increases anxiety-like behaviors and masseter muscle nociception in the cage mate of a mouse (Stress Contagion, SCO). Male mice (C57BL/6J) were divided into 3 groups: sham, SDS and SCO. SDS and SCO mice were housed in pairs for 11 days. One animal of each pair was subjected to SDS, which were daily exposed to the aggressor mouse for 10 mins, and then brought back to the cage in which a cage mate was housed. The cage mate was subjected to stress contagion (SCO). The social interaction (SI) was employed to test the anxiety conditions on Day 10. Masseter muscle nociception was quantified by the orofacial formalin behavioral test and nociceptive neural activities indicated by c-Fos and FosB immunoreactivities in the subnucleus caudalis (Vc) region on Day 11. The duration of SI was shorter in SDS (7.6 sec) and SCO (44.3 sec) compared with sham (83.8 sec) mice, indicating that anxiety-like behaviors were greater in SDS and SCO than sham mice. Formalin-evoked orofacial behaviors were significantly greater in SDS and SCO, with increases in c-Fos and FosB expression in the Vc region compared with sham mice. These findings indicate that stress contagion can increase the MM nociception.

### [1P-133]

**Relationship between salivary buffering capacity and taste sensitivity in humans**

*Aiko Hyodo[1,2], Yoshihiro Mitoh[1], Ryusuke Yoshida[1] ([1]*Department of Oral Physiology, Graduate School of Medicine, Dentistry and Pharmaceutical Sciences, Okayama University,* [2]*Department of Oral and Maxillofacial Reconstructive Surgery, Graduate School of Medicine, Dentistry and Pharmaceutical Sciences, Okayama University*)

Saliva has multiple functions, such as digestion, buffering, remineralization and cleansing. Among them, buffering capacity depends on bicarbonate ions in saliva. Buffering capacity of saliva may affect taste sensitivity because acids will be neutralized by bicarbonate, leading to reduction of sour taste. Here, we examined relationship between salivary buffering capacity and taste sensitivity in healthy young human subjects. Salivary buffering capacity was measured using Salivary Risk Test CAT21 Buff (Morita). The recognition thresholds of various tastes were measured by the whole mouth method. We found negative relationship between salivary buffering capacity and recognition threshold for umami and salt taste. There was no relationship between salivary buffering capacity and recognition threshold for sweet, bitter and sour tastes. If the subjects were divided into two groups according to buffering capacity, recognition thresholds for salty and umami tastes were significantly higher in low buffering capacity group than high buffering capacity group. These results suggest that salivary buffering capacity could affect salt and umami taste sensitivities but not sour taste.

### [1P-134]

**Analysis of emetine-induced nausea in rats using a taste reactivity test.**

*Shaoyi Su[1], Emi Kikuchi[1], Zimo Wei[1], Helai Huang[1], Tomohiko Yoshizawa[1], Tadashi Inui[1], Makoto Funahashi[1] ([1]*Hokkaido University*)

Emetine is an emetic substance contained in Cephaelis Ipecacuanha. To obtain objective evidence of whether the rats felt nausea due to emetine administration, we performed a taste reactivity (TR) test to measure the gaping reactions as an indicator of nausea. We investigated conditioned nausea after the taste conditioning procedure. Intraoral catheterization and intragastric cannulation surgeries were performed on adult male Sprague-Dawley rats under anesthesia. After recovery days, rats were conditioned with the intraoral administration of 0.1% saccharin solution (for 8 min with a flow rate of 0.5 ml/min) paired with the intraperitoneal (i.p.) or intragastric (i.g.) administration of emetine (5.54 mg/kg, 1%BW). The TR test was performed at the time of re-administration of the saccharin solution into the oral cavity. We also observed the gaping reactions during the administration of emetine without any conditioning procedure. There were no or few gaping reactions on the conditioning day, whereas a lot of gaping reactions ($58.0 \pm 10.7$ times / 8 minutes, n = 5, emetine i.p.; $137.3 \pm 33.4$ times / 8 minutes, n = 5, emetine i.g.) occurred when rats took saccharin again after conditioning. These findings indicated that emetine induces conditioned nausea. Administration of emetine without taste conditioning caused gaping reactions ($14.5 \pm 5.3$ times, n = 4, i.p. and $37.2 \pm 9.5$ times, n = 5, i.g.) within 1 hour. This provided the objective index for emetine-induced unconditioned nausea.

### [1P-135]

**Changes of taste intensity and preference elicited by mixing of sodium and commercial grade thickener**

*Chikayo Maeda[1], Fumihiko Nakamura[2], Shinpei Takahashi[2], Toshiaki Yasuo[2], Takeshi Suwabe[2], Keika Gen[1], Noritaka Sako[2] ([1]*Dept. Dent. for Disabil. & Oral Health, Asahi Univ. Sch. Dent.,* [2]*Dept. Dent. for Disabil. & Oral Health, Asahi Univ. Sch. Dent.*)

We conducted the electrophysiological and behavioral experiments to investigate how the intensity and preference for taste substances change when they were mixed with the commercial grade thickeners, which were used for the patients with dysphagia, in rats. Our electrophysiological experiment demonstrated that the chorda tympani nerve responses to 0.1M NaCl was suppressed by mixing with some of the commercial grade thickeners. In behavioral experiment, a 48-hour two-bottle preference test between NaCl mixed with one of three tested thickeners and that dissolved in distilled water was conducted. As this result, preference for NaCl mixed with some thickeners were lower than those for NaCl without thickener. These results suggest that the mixing taste substances and thickeners change taste nerve responses and that also change their preference.

### [1P-136]

**The role of $Ca^{2+}$-dependent aggregation of cargo proteins in selective transport of salivary acinar cells**

*Junko Fujita-Yoshigaki[1], Megumi Yokoyama[1], Osamu Katsumata-Kato[1] ([1]*Nihon University School of Dentistry at Matsudo*)

Salivary acinar cells have two pathways for protein secretion: the regulated and constitutive pathways. Proteins in the regulated pathway are stored in secretory granules until stimulation by secretagogues and those in the constitutive pathway are released immediately after synthesis. To analyze the mechanism, two HaloTag-fused proteins with parotid secretory protein (Psp-Halo) and erythropoietin (Epo-Halo) were expressed in the primary culture of salivary acinar cells. We have previously found that most Epo-Halo was released to the medium without retention in the cells while Psp-Halo was more accumulated in secretory granules. In this study, we investigated their tendency of aggregation. Since the Golgi lumen was reported to retain high concentration of $Ca^{2+}$, the two proteins were incubated in the presence or absence of 1 mM $Ca^{2+}$ and were centrifugated. While Epo-Halo was rarely precipitated, precipitated Psp-Halo was increased by addition of $Ca^{2+}$. These result suggest that aggregation properties may be one of the reasons that Psp was efficiently transported to and stored in secretory granules.

### [1P-137]

### Elucidation of the mechanism of the gustatory salivary reflex using mice: c-Fos expression in the central nervous system.

*Reimi Adachi[1,2], Ryusuke Yoshida[1], Sumihiro Mito[1] ([1]*Department of Oral Physiology, Okayama University Graduate School of Medical, Dental and Pharmaceutical Sciences,* [2]*Okayama University Hospital, Department of Complementary Dentistry*)

Saliva is reflexively secreted upon gustatory stimulation (gustatory salivary reflex). However, the peripheral and central mechanisms of this reflex are not well understood. For detection of acids in the oral cavity, both OTOP1 in taste cells and TRP channels in trigeminal neurons may have important roles. Thus, there may be two sensory pathways for the detection of acid in the oral cavity: gustatory and somatosensory. Therefore, to clarify the pathways that contribute to the gustatory salivary reflex induced by acids, we examined activation of neurons in the supra-salivary nucleus in wild type and TRPV1-KO mice after oral acid stimulation by c-Fos immunohistochemistry. We used awake animals with transection of hypoglossal nerve to prevent movement of the tongue. We found a concentration-dependent increase in the numbers of c-Fos positive cells in the supra-salivary nucleus of wild type mice. In TRPV1-KO mice, the number of c-Fos positive cells was reduced compared to wild-type mice. These results suggest that the pathway mediated by TRPV1 channels is considered to be greatly involved in acidity perception.

### [1P-138]

### Characteristics of children's sense of taste in objective taste assessment using functional taste tests

*Rie Fujiyama[1] ([1]*Department for Clinical Education in General Dentistry, Nagasaki University*)

Genetic and environmental factors purportedly affect taste. Environmental factors such as diet change individuals' sense of taste and form preferences. This study compared objective taste assessments between adults and children using functional taste tests. Fourteen adults (mean age, range: 46.3, 32–59 years) and children (8.7, 4–12 years) without subjective taste abnormalities were tested using the electrogustometer, a four-basic taste discrimination test using the drop method of the whole-mouth gustatory test procedure, and the Saxon test. The average threshold values determined by the electrogustometer were approximately 3.5 times higher for adults than for children. Children's taste sensitivity tended to be higher than that of adults. However, adults were significantly more sensitive than children in discriminating sweet tastes. No significant differences were observed, but adults tended to be more sensitive to salty and sour tastes than were children. However, both groups' sensitivity to bitter tastes did not differ. The Saxon test showed no difference in salivary secretion between adults and children. In conclusion, the sensitivity of taste cells and taste nerves tends to be higher in children than in adults, but that adults are more sensitive to the four basic tastes, except for bitterness. Resultantly, taste discrimination in children may be affected by environmental factors.

### [1P-139]

### Developmental changes of jaw-closing muscle activities for rhythmic ingestive behaviors

*Masaharu Yamada[1,2], Ayano Katagiri[1], Yuji Masuda[3], Hiroki Toyoda[1], Hitoshi Niwa[2], Takafumi kato[1] ([1]*Department of Oral Physiology, Graduate school of Dentistry, Osaka University,* [2]*Department of Dental Anesthesiology, Graduate school of Dentistry, Osaka University,* [3]*Division of Oral and Maxillofacial Biology, Institute for Oral Science, Matsumoto Dental University*)

[Introduction] This study aimed to clarify developmental processes in suckling and mastication using longitudinal electromyographic recordings.[Methods]The electrodes for electromyography of the masseter and temporalis muscles were installed in the rat pups at the postnatal day 10 (P10). Nipple attachment and rhythmic sucking were recorded as suckling at P14. Pasta biting and pellet chewing were recorded as mastication from P21 to 49. Burst rhythm and muscle activity coordination were quantified.[Results]At P14, burst rhythms for nipple attachment and rhythmic sucking were slower than those for pasta biting and pellet chewing. Muscle activity coordination differed between suckling and mastication. After P21, burst rhythms for biting and chewing increased as growing. Muscle activity coordination did not change from P21 to 49 for biting. However, for chewing, muscle activity coordination changed from P21 to 24 but was stable after P24.[Conclusion] Suckling and mastication can be differently controlled. Biting and chewing exhibit the unique developmental processes of masticatory motor dynamics after weaning. The authors declare no COI associated with this manuscript.

### [1P-140]

### Spatial separation of amiloride sensitivity, threshold and responses to NaCl of taste-sensitive neurons in rat rostral nucleus of the solitary tract

*Tatsuko Yokota[1], Katsunari Hiraba[1] ([1]*Department of Physiology, School of Dentistry, Aichi-Gakuin University*)

We reported that the N-best of taste-sensitive neurons in rat rostral nucleus of the solitary tract (rNST) was located rostral to the NH-best. In the present study, we investigated histological distributions of neurons for NaCl and amiloride (ENaC antagonist) sensitivities. Here, we recorded extracellular single unit activities in the rNST neurons using multi-barrel glass micropipettes while under anesthesia. The recording sites marked by dye spots were reconstructed on the rostrocaudal and mediolateral axes. Seventy-three taste-sensitive neurons were classified into the best-taste category. In thirty-one neurons, amiloride sensitivities were examined for 0.1, 0.2, 0.4 and 0.8 M NaCl. Taste-sensitive neurons were identified as amiloride-sensitive (AS) and amiloride-insensitive (AI), low-threshold (LT) and high-threshold (HT). Low-response frequency (LR) and high-response frequency (HR) were applied to all recorded neurons. AS, LT and HR were located rostrolateral to AI, HT and LR ($p<0.05$, Discriminant analysis), and AS consisted mostly LT and/or HR. Spatial differences for the concentration and amiloride sensitivities of the taste-sensitive neurons suggest that palatable and aversive sodium salts were processed separately the rostral-lateral and caudal-medial regions of the rNST. No conflicts of interest, financial or otherwise, are declared by the authors.

### [1P-141]

### Heated Tobacco Products (HTPs) Affect Human Oral Cancer Cells.

*Shota Takayasu[1], Masanari Umemura[1], Kagemichi Nagao[1], Rina Nakakaji[1], Akane Nagasako[1], Soichiro Ishikawa[1], Yuto Mizuno[1], Fumina Suzuki[1], Takahiro Horinouchi[2], Yoshihiro Ishikawa[1] ([1]*Yokohama City Univ.,* [2]*Hokkaido Univ.*)

### Poster Presentation

[1P]
**Circulation**

March 14 (Tue.), 12:10 - 14:10, Poster Room

### [1P-142]

### A distinguishable role of PVAT in obesity-related cardiovascular disease through the buffering activity of ATF3 by vasoconstrictors

*Yu Han Su[1,2], Hsiao Fen Li[4,5,6], Po Yi Chen[1,2], Heng Lin[4,5,6], Tzu Ling Tseng[1,2,3]
([1]*CardioVascular Research Center, Hualien Tzu Chi Hospital,* [2]*Department of Medical Research, Hualien Tzu Chi Hospital,* [3]*Tzu Chi University of Science and Technology,* [4]*Department of Physiology, School of Medicine, College of Medicine, Taipei Medical University,* [5]*Graduate Institute of Clinical Medicine, College of Medicine, Taipei Medical University,* [6]*Division of Nephrology, Department of Internal Medicine, School of Medicine, College of Medicine, Taipei Medical University*)

Perivascular adipose tissue (PVAT) has emerged as an adipose organ that exhibits similarities to brown adipose tissue (BAT), including cellular morphology and thermogenic gene expression. However, it is unclear whether the phenotype of PVAT is indistinguishable from BAT in the vasculature under physiological conditions. We demonstrated that PVAT is distinguishable from BAT given its specific vessel tone-controlling function not found in BAT. Activating transcription factor 3 (ATF3) is a key factor in hypertension. Compared to wild-type (WT) mice, ATF3-deficient ($ATF3^{-/-}$) mice fed a high-fat diet (HFD) exhibited elevated mean arterial pressure, increased monocyte chemoattractant protein-1 expression and hypertrophy plus abnormal fatty tissue accumulation in the PVAT, and enhanced vascular wall tension and vasoconstrictive responses of potassium chloride, U46619, and norepinephrine in the isolated aortic rings. These changes in the $ATF3^{-/-}$ mice were restored after administration of an adeno-associated ATF3 vector. Our findings suggest that PVAT, but not BAT, modulates obesity-related vascular dysfunction. ATF3 within PVAT could provide new insights into the pathophysiology of obesity-related cardiovascular diseases.

### [1P-143]

### Effects of empagliflozin on baroreflex-mediated sympathetic arterial pressure regulation in type 2 diabetic model rats

*Toru Kawada[1], Meihua Li[1], Aimi Yokoi[1], Akitsugu Nishiura[1], Keita Saku[1] ([1]*Department of Cardiovascular Dynamics, National Cerebral and Cardiovascular Center*)

Background: Empagliflozin is an inhibitor of sodium-glucose cotransporter 2 (SGLT2). Although cardiovascular benefits have been reported as a class effect of SGLT2 inhibitors, the effects of empagliflozin on baroreflex-mediated sympathetic arterial pressure (AP) regulation remain to be quantified. Methods: Using type 2 diabetic model rats (Goto-Kakizaki rats, n = 7), the carotid sinus pressure was changed stepwise from 60 to 180 mmHg. The baroreflex responses in sympathetic nerve activity (SNA) and AP were compared before and 44 min after the intravenous administration of empagliflozin (10 mg/kg). Results: Empagliflozin did not significantly affect the response range of SNA (45.0 ± 6.1% vs. 45.6 ± 9.4%) or the slope of AP versus SNA (1.30 ± 0.12 vs. 1.50 ± 0.22 mmHg/%). It did not affect the operating-point AP in the baroreflex equilibrium diagram, either (125.7 ± 4.2 vs. 130.0 ± 2.6 mmHg). Conclusion: Empagliflozin did not acutely affect the baroreflex-mediated sympathetic AP regulation in type 2 diabetic model rats.

### [1P-144]

### Donepezil Markedly Attenuates the Cardiac/Renal Remodeling in Rats with Renal Artery Stenosis-Induced Hypertension

*Meihua Li[1], Toru Kawada[1], Masaru Sugimachi[1], Keita Saku[1] ([1]*Department of Cardiovascular Dynamics, National Cerebral and Cardiovascular Center*)

Acetylcholinesterase inhibition by donepezil has been shown to improve prognosis in chronic heart failure (CHF) rats following permanent myocardial infarction. This study investigated whether donepezil is effective in the treatment of another CHF rat model complicated with renal artery stenosis-induced hypertension (RASH). RASH was induced by ligating the left renal artery down to 50% in rats. At the 11th week, survived RASH rats were randomly assigned to the untreated (UT) or donepezil treated (DT, 3 mg/kg/day) group. After 6 weeks of treatment, we evaluated hemodynamics, blood levels of neurohumoral markers, histology, and morphology. Compared with UT, DT significantly suppressed the progression of cardiac hypertrophy and left kidney atrophy and prevented cardiac and renal dysfunction. DT decreased creatinine, norepinephrine, aldosterone, and BNP in the blood and attenuated systemic inflammation. Donepezil treatment markedly prevented the progression of cardiac/renal remodeling and dysfunction in RASH rats. The results suggest that donepezil may be used as a new pharmacotherapy for CHF complicated with RASH.

### [1P-145]

### Regulation of Blood Pressure and Cardiac Output during Chronic α1-Adrenergic Stimulation

*Jean-Pierre Montani[1], Bruce N. Van Vliet[2] ([1]*Physiology, University of Fribourg / Switzerland,* [2]*Memorial University of Newfoundland, St. John's, Canada*)

Objective. We evaluated the long-term control of blood pressure and cardiac output during chronic vasoconstriction induced by $\alpha_1$-adrenergic stimulation.Methods and results. We infused phenylephrine (PE) intravenously (1 µg/kg/min) for 10 days in 7 dogs maintained on a fixed sodium intake. Mean arterial pressure (MAP) and cardiac output (CO) were monitored 20 hours/day. On the first day, PE caused a rapid increase in MAP from 91 ± 8 mmHg, and decreased both CO and heart rate (HR) from 2.32 ± 0.10 L/min and 69 ± 3 beats/min to 1.77 ± 0.06 L/min and 51 ± 2 beats/min, respectively. The initial increase in MAP was not sustained as MAP stabilized then at a value of 99 ± 4 mmHg (days 8-10) whereas CO and HR remained decreased at 1.74 ± 0.09 L/min and 54 ± 2 beats/min, respectively (days 8-10). However, due to an increase in hematocrit from 37.9 symbol 177 \f"Symbol" \s 12± 1.9 to 52.2 symbol177 \f "Symbol" \s 12± 3.6, arterial oxygen delivery, estimated by the product of CO and hematocrit, was maintained. Summary and conclusions. Chronic vasoconstriction led to a mild hypertension with a sustained decrease in HR and CO. However, despite the low CO, oxygen delivery to the tissues was maintained secondary to an increase in hematocrit. Our data suggest that arterial oxygen delivery rather than CO itself is a strongly regulated variable.

### [1P-146]

### Sustained overexpression of cell cycle promoter Fam64a causes heart failure through repression of Klf15

*Ken Hashimoto[1], Aya Kodama[1], Momoko Ohira[1], Misaki Kimoto[1], Yuu Usui[1], Yoshihiro Ujihara[2], Akira Hanashima[1], Satoshi Mohri[1] ([1]*First Department of Physiology, Kawasaki Medical School,* [2]*Department of Electrical and Mechanical Engineering, Nagoya Institute of Technology*)

Postnatal loss of cardiomyocyte proliferation hinders heart regeneration in adults. Therefore, introduction of fetal cell cycle genes into damaged adult hearts is a promising strategy for achieving heart regeneration. We have recently identified Fam64a as a fetal-specific cell cycle promoter in cardiomyocytes. In this study, we analyzed transgenic mice maintaining cardiomyocyte-specific postnatal expression of Fam64a when endogenous expression was abolished. These mice showed an enhancement of cardiomyocyte proliferation as expected, but they demonstrated impaired differentiation during postnatal development, leading to heart failure in later life. Mechanistic analyses revealed that Fam64a inhibited cardiomyocyte differentiation by repressing Klf15, leading to the accumulation of immature cardiomyocytes. In contrast, transient and local induction of Fam64a in differentiated adult wildtype hearts improved functional recovery upon injury with enhanced cell cycle and no dedifferentiation in cardiomyocytes. These data indicate that Fam64a inhibits cardiomyocyte differentiation during development, but does not induce dedifferentiation in once differentiated cardiomyocytes, highlighting a promising potential of Fam64a as a cell cycle promoter to attain heart regeneration.

### [1P-147]

### The exacerbation mechanism of cardiomyocyte injury by cardiac Sigma-1 receptor knockdown

*Hideaki Tagashira[1,2], Yasuharu Shinoda[2], Tomohiro Numata[1], Kohji Fukunaga[2] ([1]*Akita Univ.*, [2]*Tohoku Univ.*)

Heart failure is a leading cause of death globally. We previously reported that the Sigma-1 receptor (Sigmar1) is down-regulated in mice with cardiac dysfunction. A recent study suggested Sigmar1-deficient mice display cardiac dysfunction via impaired mitochondrial function. However, the mechanism of mitochondrial quality control mediated by Sigmar1 has not been investigated. In this study, we investigated the role of Sigmar1 for ER-mitochondrial tethering and mitochondrial $Ca^{2+}$ signaling using primary cultured neonatal rat ventricular cardiomyocytes (NRVMs). Sigmar1 knockdown showed the disruption of ER-mitochondrial tethering and reduction of ER-mitochondrial $Ca^{2+}$ transport. Furthermore, immunohistochemical analysis demonstrated that Sigmar1 knockdown exacerbated Endothelin-1-induced cardiomyocyte hypertrophy and was associated with mitophagy induction. These data suggest that the reduction of cardiac Sigmar1 is involved in myocyte hypertrophy by maintaining intracellular $Ca^{2+}$ signaling mediated by regulation of ER-mitochondrial tethering.

### [1P-148]

### Pathophysiological analysis of heart failure in cardiac-specific TRIC-B-deficient mice

*Daiju Yamazaki[1], Kohei Matsushita[1], Miki Nonaka[2], Mika Tokutake[1], Kaori Chujo[1], Nanae Koda[1], Yasuhito Uezono[2], Kumiko Ogawa[1] ([1]*National Institute of Health Scieces*, [2]*The Jikei University*)

TRIC channel subtypes (A and B) function as counter ion channels on the ER membrane. TRIC-A is expressed in excitable tissues such as heart. TRIC-B is expressed ubiquitously throughout the body. Since TRIC-A and B double deficient (KO) mice are embryonic lethal, both subtypes may play important functions in the heart. In systemic TRIC-B-KO mice, surfactant production and secretion of type II alveolar cells are impaired, resulting in impaired alveologenesis and neonatal lethality. Thus, we generated cardiac-specific TRIC-B-KO (cKO) mice and investigated the function of TRIC-B in the heart. We found that cKO mice die 30-60 weeks of age. To confirm changes over time in the heart, we performed histopathological analysis at 12, 20, 30, and 40 weeks of age. We found that cKO mice had significantly higher blood troponin levels than wild-type mice ≥ 12 weeks of age. cKO mice also showed marked cardiac fibrosis ≥ 20 weeks of age and increased heart and lung weight ≥ 30 weeks of age. Moreover, cKO mice showed decreased body weight, cardiomyocyte degeneration, and atrial thrombus at 40 weeks of age. Since these findings indicated heart failure-like pathology, cardiac systolic function is under further investigation with echocardiography.

### [1P-149]

### Saturated fatty acids induced $Ca^{2+}$ overload is ameliorated by eicosapentaenoic acid in cardiomyocytes

*Masaki Morishima[1], Kosuke Horii[1], Pu Wang[3], Kazuki Horikawa[2], Katsushige Ono[3] ([1]*Kindai University*, [2]*Tokushima University*, [3]*Oita University*)

Omega-3 polyunsaturated fatty acids can modulate cardiac electrophysiological functions and reduce the genesis of arrhythmias. Here we investigated the possible beneficial actions of eicosapentaenoic acid (EPA) on cardiomyocyte focusing on the L-type $Ca^{2+}$ channel through the receptor FFAR4 and a transcription factor adenosine-3', 5'-cyclic monophosphate response element binding protein (CREB). Neonatal mice cardiomyocytes were cultured with oleic (500 µM) / palmitic acid (250 µM) mixture (OAPA) in the presence or absence of EPA (10 µM) for 24 h. EPA retrieved a reduction of spontaneous beating rate, L-type $Ca^{2+}$ current, mRNA, and protein expressions of the Cav1.2-L-type $Ca^{2+}$ channel caused by OAPA. Immunocytochemical analysis revealed a distinct downregulation of the Cav1.2 channel by OAPA with a concomitant decrease in the phosphorylated component of CREB in the nucleus, which was also rescued by EPA. Transcriptional regulation of Cav1.2 by EPA was blocked by an FFAR4 antagonist AH7614, whereas an FFAR4 agonist TUG-891 mimicked the action of EPA. In addition, EPA shortened the time to the peak and accelerated the decay of the $Ca^{2+}$ transient in Fluo-4 loaded cardiomyocytes. These results suggest that EPA rescues $Ca^{2+}$ overload caused by OAPA lipotoxicity through the FFAR4/CREB/Cav1.2-mediated pathways.

### [1P-150]

### Fenestration improves the hemodynamics in the failing Fontan patients under mechanical circulatory support

*Shuji Shimizu[1], Yasuhiro Kotani[2], Toshiaki Shishido[1] ([1]*National Cerebral and Cardiovascular Center*, [2]*Okayama University*)

Introduction: In the failing Fontan patients, the implantation of ventricular assist device has recently been considered as a destination therapy. However, in the failing Fontan patients under mechanical circulatory support (MCS), increased pulmonary vascular resistance (PVR) may reduce venous return to the single ventricle, resulting in pump flow deficiency. Fenestration between the conduit and the single atrium may increase venous return without additional volume load. To clarify the effects of fenestration on the hemodynamics under MCS, we performed a computational simulation. Methods: A computational model of the Fontan circulation was made using a time-varying elastance chamber and Windkessel vasculature models. Ventricular assist device was modeled as a linear function of pressure head and flow. The fenestration was modeled according to the simple form of the Bernoulli's equation. When PVR index was varied, mean arterial pressure was maintained by adjusting the stressed blood volume and rotational frequency. Results: With PVRI index of 6.1 WU $m^2$, MCS combined with fenestration (fMCS) significantly increased cardiac index (2.7 to 3.0 l/min/$m^2$) and reduced central venous pressure (16.6 to 11.1 mmHg) compared to the MCS alone. However, fMCS significantly decreased arterial oxygen saturation (97 to 77%). Conclusions: With a decrease in saturation, the fenestration significantly improves the hemodynamics in the failing Fontan patients under MCS.

### [1P-151]

### Prefrontal oxygenation was quantified with a time-resolved near-infrared spectroscopy: effect of sex on baseline oxygenation at rest and the response during exercise

*Ryota Asahara[1], Kanji Matsukawa[2] ([1]*National Institute of Advanced Industrial Science and Technology*, [2]*Hiroshima University*)

A sex difference in cerebral circulation has been reported. Resting cerebral blood flow (CBF) is higher and plasma hemoglobin (Hb) concentration is lower in young women than men. The inverse relationship between CBF and Hb may be interpreted as homeostatic regulatory mechanisms for maintaining oxygen supply to the brain. If so, it is postulated that the resting oxygenation level in cerebral microcirculation is similar between women and men. Using a time-resolved near-infrared spectroscopy that enables the quantitative measurement of oxygenated-Hb (Oxy-Hb) in the prefrontal cortex, this study aimed to examine sex differences in baseline oxygenation at rest and the responses during unilateral cycling exercise. Eighteen young participants (8 women and 10 men aged 21 to 33 years) were enrolled in this study. Baseline prefrontal Oxy-Hb was significantly lower in women (35 ± 3) than in men (46 ± 7), while deoxygenated-hemoglobin concentration (Deoxy-Hb) demonstrated no sex difference. The change of prefrontal Oxy-Hb to unilateral cycling was similar between sexes. In addition, the baseline and the response values of prefrontal Oxy-Hb to unilateral cycling were identical between the right and left prefrontal cortices. The current findings indicate that baseline prefrontal Oxy-Hb is lower in women than in men, whereas Deoxy-Hb is not different irrespective of sex, suggesting that prefrontal oxygenation in women is still insufficient in delivery of oxygen to the brain and (2) prefrontal oxygenation responds during exercise, similarly independent of sex.

### [1P-152]

### Mechanism of metabolic adaptation in the heart primordium just after heartbeat initiation in the early embryonic development in rats.

*TATSUYA SATO[1], NOBUTOSHI ICHISE[1], HIROYORI FUSAGAWA[1], TAKURO KARAUSHI[1], TAIKI KUDO[1], NORITSUGU TOHSE[1] ([1]*Department of Cellular Physiology and Signal Transduction, Sapporo Medical University School of Medicine*)

The initiation of the heartbeat is an essential step of cardiogenesis, but it remains unclear how the energy is provided after heartbeat initiation. In this study, embryos in Wistar rats at embryonic day 10.0, when the heartbeat begins in rats, were divided into two groups by the heart primordium before and after heartbeat initiation, and their metabolic characteristics were evaluated. Metabolomic analysis revealed that the major determinants in the heart primordium after heartbeat initiation were increased levels of ATP, a major product of glucose catabolism, and reduced glutathione, a byproduct of the pentose phosphate pathway. An extracellular flux analyzer revealed that glycolytic capacity was significantly increased in the heart primordium after heartbeat initiation. ATP-linked mitochondrial respiration was also increased in the heart primordium after heartbeat initiation, but expression levels of TCA cycle enzymes and subunits of mitochondrial electron transport chain were unchanged, suggesting that the energy after heartbeat initiation is supplied by increased glycolytic flux. Hypoxia-inducible factor (HIF)-1α was activated, and its downstream rate-limiting enzymes of the glycolytic and pentose phosphate pathways were upregulated in the heart primordium after heartbeat initiation. These results indicate that the HIF-1α-mediated enhancement of glycolysis with activation of the pentose phosphate pathway covers the increased energy demand in the beating and developing heart primordium.

### [1P-153]

### Myofilament protein post-translational modifications may underlie right ventricular dysfunction in pulmonary hypertension

*Mark Waddingham[1], Hirotsugu Tsuchimochi[1], Takeshi Ogo[1], James Pearson[1]
([1]*National Cerebral and Cardiovascular Center*)

Introduction: Previous data from our group indicates that impairments in regional myofilament function correlates with changes in right ventricular (RV) dysfunction in pulmonary hypertension (PH). Myofilament function is tightly regulated by phosphorylation of myofilament proteins, allowing fine-tuning of myocardial performance to match ventricular loading conditions. Under pathological conditions, overactivation of the sympathetic nervous system, metabolic imbalances, oxidative and nitrosative stress, deranged protein quality control and an altered chromatin transcription pathway activity can all induce numerous myofilament protein post-translational modifications (PTMs) and isoform shifts that can ultimately drive the development of RV dysfunction. We investigated several of these myofilament proteins PTMs and isoform shifts in the RV of rats with PH and RV dysfunction. Methods: PH was induced in rats using the sugen 5416/3-week 10% hypoxia method followed by a 6-week normoxic period. RV function was measured by pressure-volumetry. Myofilament enriched fractions were prepared from RV tissue using a Triton X-100 extraction technique and solubilized in a urea/thiourea sample buffer. Myofilament PTMs and isoforms shift were established using SDS-PAGE and Western blotting, except for titin where specialized agarose electrophoresis was utilized. Results: Compared to controls rats, PH rats exhibited heightened RV afterload, systolic and diastolic dysfunction (all $P<0.05$) along with a significant increase in the titin N2BA and b-myosin heavy chain isoforms ($P<0.001$), consistent with RV dysfunction in PH. Whole myofilament analysis revealed a significant increase in carbonylation, nitrosation and ubiquination (all $P<0.01$ vs. Control) in the RV of PH rats. In terms of phosphorylation, both titin ($P<0.001$) and MLC-2v ($P<0.01$) were lower in the RV of PH rats compared to controls. Both myofilament ubiquitin and phosphorylation levels correlated with indices of RV diastolic and systolic function ($P<0.05$). Conclusion: Myofilament proteins PTMs related to oxidative/nitrosative stress, protein quality control and phosphorylation likely contribute to the myofilament and RV dysfunction in PH.COI: Properly Declared

### [1P-155]

### Effect of mutant truncated myosin binding protein-C on early cardiac function and remodeling in a mouse model of hypertrophic cardiomyopathy

*James T Pearson[1,2], Mark Waddingham[1], Hirotsugu Tsuchimochi[1] ([1]*NCVC*, [2]*Monash Univ.*)

Cardiac myosin binding protein-C (cMyBP-C) acts as a brake on myosin filament sliding within the sarcomere, negatively regulating contractility. Mutations in Mybpc3 are one of the most common causes of familial hypertrophic cardiomyopathy. Here we assessed how deletion of Mybpc3 exon 33 (256bp) affects cardiac function and gene-protein expression. Homozygotes developed dilated heart failure as juveniles, with regional calcification, while heterozygotes showed increased fractional shortening and increased mitral E/E' ahead of hypertrophy as adult mice. Truncated cMyBP-C did not remain attached to the myofilaments and accumulated with modifications in the cytosol and was associated with increased Atf6 mRNA expression. Relative MLC-2 phosphorylation was inversely related to LV mass, while desmin phosphorylation was elevated in failing homozygotes, along with increased natriuretic peptide and inflammatory gene expression and decreased antioxidant gene expression from a young age. Utilising synchrotron X-ray scattering we also examine in situ cross-bridge cycling and sarcomere shortening in the left ventricle to understand the progression of contractile and diastolic dysfunction.

### [1P-157]

### Region-specific regulation of cerebral blood flow during cardiac pacing

*Kei Ishii[1], Hidehiko Komine[2] ([1]*AIST, HIIRI*, [2]*AIST, HCMRC*)

Cardiac tachyarrhythmias can evoke severe hypotension and restriction of cerebral blood flow, resulting in syncope and sudden death occasionally. In the situation, cerebral blood flow must be redistributed to vital brain areas which are essential for maintenance of life. However, it was still unknown whether and how cerebral blood flow is regulated depending on brain areas during tachyarrhythmia. As a first step, cerebral blood flow during cardiac pacing was compared between the dorsal hypothalamus (i.e., vital brain area) and the motor cortex (i.e., non-vital brain area) in anesthetized ventilated rats. We hypothesized that reduction of blood flow during cardiac pacing was relatively maintained in the dorsal hypothalamus in comparison to the motor cortex. Cerebral blood flow was measured by using a laser-Doppler flowmetry. Cardiac pacing caused a reduction of arterial blood pressure (AP) and a slight rise in central venous pressure, while intracranial pressure was not changed ($P > 0.05$). The reduction of blood flow in the motor cortex was proportional to the decrease in AP, whereas blood flow in the dorsal hypothalamus was relatively ($P < 0.05$) maintained against hypotension. These results indicate differential regulation of cerebral blood flow in the face of severe hypotension caused by tachyarrhythmia.

### [1P-154]

### Mechanisms of ventricular arrhythmias triggered by the development of EAD: an *in silico* study

*Kunichika Tsumoto[1], Yu-ma Aoji[2], Takao Shimamoto[2], Yukiko Himeno[2], Akira Amano[2], Yuhichi Kuda[1], Mamoru Tanida[1], Yasutaka Kurata[1] ([1]*Kanzawa Med Univ.*, [2]*Ritsumeikan Univ.*)

Torsades de Pointes (TdP), which is frequently observed in patients with long QT syndrome and also known as polymorphic ventricular tachycardia, is thought to be caused by spiral excitation waves meandering in the ventricle. Many clinical studies have reported that early afterdepolarization (EAD) which causes transient depolarization during the action potential repolarization phase, precedes the development of TdP. However, the role of EAD in the development of TdP remains unclear. Simulating excitation propagation on a two-dimensional ventricular tissue model consisting of the Kurata human ventricular myocyte model (Kurata et al., Biophys J, 2005), we have shown in the 99th annual meeting of the Physiological Society of Japan that the discontinuous distribution of EAD-evoking cardiomyocytes in islands (clusters) within ventricular tissue may promote the TdP initiation. To better understand the initiation mechanism of TdP, we investigated the relationship between TdP development and the distance between EAD clusters. In 6 cm square ventricular tissue, we placed two EAD clusters (2 cm × 4 cm) parallel at a fixed distance. The secondary TdP-triggering excitatory waves that followed the EAD onset were generated only when the distance between EAD clusters was within a range between 2.0 and 2.8 mm. These results suggest that the specific substrate formation by the EAD cluster augments the risk of triggering TdP.

### [1P-156]

### Novel molecular mechanisms of endocardial hematopoiesis and their significance in cardiac development

*Norika Liu[1,2], Naofumi Kawahira[2], Yasuhiro Nakashima[3], Haruko Nakano[2], Akiyasu Iwase[4], Yasunobu Uchijima[4], Sean Wu[5], Susumu Minamisawa[1], Hiroki Kurihara[4], Atsushi Nakano[1,2] ([1]*The Jikei University School of Medicine*, [2]*University of California, Los Angeles*, [3]*Kyoto University*, [4]*University of Tokyo*, [5]*Stanford University*)

We previously reported a subset of endocardial cells is hemogenic during early embryogenesis. They are enriched in the cushion region, the primordia of the cardiac valves and septa, where active remodeling via endothelial-mesenchymal transition (EndoMT) takes place. Hemogenic endocardial cells undergo endocardial- hematopoietic transition (EHT) in Nkx2-5-dependent manner. In the current study, we analyzed the regulatory network of hematopoiesis using scRNA-seq data. Nkx2-5-null hearts were devoid of clusters for hemogenic endocardium and cushion endocardium. Further analysis revealed that genes related to Notch signaling pathway significantly downregulated in Nkx2-5-null endocardium. Strikingly, impaired hematopoiesis and cushion defects in the Nkx2-5-null heart were both rescued by overexpression of Notch intracellular domain (NICD), suggesting that Notch signaling promotes endocardial hematopoiesis downstream of Nkx2-5. A further gene network analysis identified that Dhrs3, an enzyme that attenuates retinoic acid (RA) signal by catalyzing the reduction of all-trans-retinaldehyde to all-trans-retinol, is a signature gene of the hemogenic endocardial cells downstream of Nkx2-5. Our *ex vivo* hematopoietic colony forming assay revealed that EHT is strongly inhibited by RA signal. Notch inhibition also suppressed EHT. Consistently, *in vivo* forced activation of NICD drastically increased the number of hemogenic endocardial cells as well as macrophages in the cardiac cushion. Taken together, our study demonstrated that the Nkx2-5/Notch/RA signaling axis plays a pivotal role in EHT during early embryogenesis, thereby facilitating local tissue remodeling by inducing macrophage differentiation.

### [1P-158]

### Oxidative Stress-Induced Increase in Endothelial Permeability Is Associated with Disorganization of Adherence Junctions in Human Umbilical Vein Endothelial Cells.

*NUR AQILAH BINTI KAMARUDDIN[1,2], YOKE KEONG YONG[1], MUHAMMAD NAZRUL HAKIM ABDULLAH[4], MANRAJ SINGH CHEEMA[4], LAI YEN FONG[3] ([1]*Department of Human Anatomy, Faculty of Medicine and Health Sciences, Universiti Putra Malaysia, UPM Serdang, Selangor, Malaysia.*, [2]*Department of Anatomy, Faculty of Medicine, Universiti Kebangsaan Malaysia, Jalan Yaacob Latif, Bandar Tun Razak, Cheras, Kuala Lumpur, Malaysia.*, [3]*Department of Pre-clinical Sciences, Faculty of Medicine and Health Sciences, Universiti Tunku Abdul Rahman, Kajang, Selangor, Malaysia.*, [4]*Department of Biomedical Science, Faculty of Medicine and Health Sciences, Universiti Putra Malaysia, UPM Serdang, Selangor, Malaysia.*)

Objective: The endothelial barrier is crucial in maintaining vascular homeostasis. The endothelium barrier breaks down in response to oxidative stress, resulting in increased endothelial permeability. Omentin is an anti-diabetic and anti-inflammatory adipocytokine that is abundantly secreted in visceral adipose tissue. Withal, its effect on endothelial barrier function still remains unclear. Thus, the aim of this study is to explore whether omentin can ameliorate barrier dysfunction induced by hydrogen peroxide ($H_2O_2$). Methods: The effect of omentin in regulating human umbilical vein endothelial cells (HUVECs) paracellular micro- and macromolecules permeability was evaluated by using sodium fluorescein (Na-F) and Evans blue albumin (EBA). The distribution of adherence junctions (AJs) in cells were investigated using immunocytochemistry and confocal imaging. Western blot analysis was used to measure the total protein expression of AJs. Results: $H_2O_2$ significantly increased the permeability of Na-F and EBA by approximately 1.79-fold and 3.1-fold, respectively compared to the control group ($p<0.001$). Interestingly, omentin significantly reduced both leakage of small molecules and large molecules ($p<0.001$). Immunostaining data also demonstrated that omentin enhanced the distribution of VE-cadherin and β-catenin proteins by preserving reticular junctions at the cells border. In addition, the quantification analysis demonstrated that omentin significantly increased the $H_2O_2$-reduced junctional region of VE-Cadherin and β-catenin compared to $H_2O_2$ group ($p<0.001$). Western blot analysis demonstrated that $H_2O_2$ treatment reduced protein levels of both VE-cadherin and β-catenin by 0.39 and 0.18-folds, respectively, compared to non-stimulated cells ($p<0.05$). Surprisingly, pre-treatment of omentin at 300 and 450 ng/ml significantly increased the decreased VE-cadherin expression to 0.93 and 0.81, respectively, compared to the $H_2O_2$-induced group ($p<0.001$). Conclusion: This study implies that omentin prevented endothelial disruption induced by oxidative stress and this effect was strongly associated with the protective of AJs.

### [1P-159]

### Single cell mechanics of human cardiomyocytes assessed by cellular force-length relationships

*Hiroaki Komatsu[1], Yasuhiro Kotani[1], Keiko Kaihara[2], Keiji Naruse[2], Shingo Kasahara[1], Gentaro Iribe[3] ([1]*Okayama University Hospital Cardiovascular Surgery,* [2]*Okayama University Graduate School of medicine,Dentistry and Pharmaceutical Science Department of Cardiovascular Physiology,* [3]*Asahikawa Medical University Department of Physiology*)

Objective: In clinical practice, it is difficult to directly measure myocardial contractility in patients, and thus the mechanical characteristics of human cardiomyocytes remain poorly understood. In this study, we applied our original method, which allows to assess force-length relationship in single cardiomyocytes, to human cardiomyocytes to investigate their mechanical characteristics in detail.Methods: Right ventricular myocardium resected from patients undergoing surgery for congenital heart disease was used. Single cardiomyocytes were enzymatically isolated from the tissue. To obtain force-length relationships for the cells, a pair of carbon fibers (CFs)were attached to each cell end and the cells were stretched in several steps under electrical stimulation of 0.5 Hz at 37°C. Cell length and tension were obtained from the distance between CFs and the amount of CF bending, respectively. The slope of the obtained end-systolic force-length relation and the end-diastolic force-length relation was used to evaluate cellular contractility and diastolic cellular stiffness, respectively.Results: We found that the contractility and diastolic cellular stiffness in human cardiomyocytes were not significantly different from those in mouse cardiomyocytes. Conclusions: The present method for evaluating mechanical function of human cardiomyocytes has the potential to be applied to preoperative evaluation of cardiac function in the future.

### [1P-160]

### Mechanisms of Automaticity in HL-1 Mouse Atrial Myocytes. : role of $I_{K1}$, $I_f$ and SR $Ca^{2+}$ release.

*Yuhichi Kuda[1], Yasutaka Kurata[1], Takayuki Ikeda[2], Mamoru Tanida[1], Kunichika Tsumoto[1], Toshishige Shibamoto[1], Hideto Yonekura[2] ([1]*Department of Physiology II, Kanazawa Medical University, Japan,* [2]*Department of BiochemistryII, Kanazawa Medical University, Japan*)

Aim: HL-1 mouse atrial myocytes are often used for systematic electrophysiological studies. In this study, we determined the characteristics of $I_{K1}$ and $I_f$ channel currents and their involvement with automaticity in HL-1 cells using the superforated-patch technique (Shioya, 2020). Methods: The superforated-patch recording using nystatin were performed to determine $I_f$ and $I_{K1}$ dynamics and the effects of an $I_{K1}$ blocker, $Ba^{2+}$, and caffeine that depletes $Ca^{2+}$ in the SR on action potentials (APs) or automaticity. Results: 1) $I_f$ was detected in some HL-1 cells but the cells with $I_f$did not have automaticity. On the other hand, the HL-1 cells that had no $I_f$ detected showed automaticity. 2) $I_{K1}$ block by $Ba^{2+}$ at 1-5 mM induced automaticity. 3) Caffeine attenuated intracellular $Ca^{2+}$ transients, slowed pacemaking, and abolished automaticity. However, in a few cells, caffeine did not abolish automaticity. Conclusions: HL-1 cells showed two types of pacemaker activity, i.e., $I_{K1}$ block-induced and SR $Ca^{2+}$ release-dependent pacemaking. $I_f$ is not necessary for pacemaiking of HL-1 cells. Thus, HL-1 cells are useful for systematically investigating the mechanisms of cardiac automaticity.

### [1P-161]

### TRPV2 is crucial for the structural and functional maturation of myocyte in growing hearts

*YUBING DONG[1], Yoshihiro Ujihara[2], Yanzhu Chen[1], Kimiaki Katanosaka[3], Keiji Naruse[1], Yuki Katanosaka[1] ([1]*Department of Cardiovascular Physiology, Graduate School of Medicine, Dentistry and Pharmaceutical Sciences, Okayama University,* [2]*Department of Electrical and Mechanical Engineering, Nagoya Institute of Technology, School of Engineering,* [3]*College of Life and Health Sciences, Chubu University*)

Aims: We examined the effects of the transient receptor potential, vanilloid family type 2 (TRPV2)-deficiency in cardiac maturation during growth process from juvenile to adult of mice.Methods and Results: We injected tamoxifen-inducible TRPV2cKO mice with tamoxifen starting at 2 weeks of age and analyzed heart structure and function in juvenile and adult mice. TRPV2cKO mice had smaller hearts as adults and showed reduced contractility throughout juvenile life. TRPV2cKO mice showed abnormal T-tubule structure, myofibrillar segmentation, and mitochondrial swelling. Isolated cardiomyocytes from cKO hearts showed defects in contractility and intracellular $Ca^{2+}$ handling, representing the abnormality in subcellular localization of $Ca^{2+}$ regulatory proteins. TRPV2-deficiency from juvenile mice led to the impairment of the maturation of cardiomyocytes and defects in contractile reserve capacity. Conclusions: These results suggest that TRPV2 is crucial for the maturation of myocyte structure and function. Our results improve our understanding of the molecular processes involved in cardiomyocyte maturation and advances in knowledge and implication for cell transplantation therapies, *in vitro* modelling or drug discovery.

### [1P-162]

### Impaired automaticity of sinoatrial nodal cells in mouse model of myocardial steatosis

*YUKARI TAKEDA[1,2], Satsuki Sato[3], Jinya Suzuki[3,4], Satoshi Matsuoka[1,2] ([1]*Department of Integrative and Systems Physiology, Faculty of Medical Sciences, University of Fukui.,* [2]*Life Science Innovation Center, University of Fukui.,* [3]*Third Department of Internal Medicine, Faculty of Medial Sciences, University of Fukui.,* [4]*Department of Medicine, Medical Corporation Yasukawa Hospital*)

Sinoatrial node is the primary pacemaker of heart. We demonstrated that $Ca^{2+}$ efflux from mitochondria contributes to the automaticity of sinoatrial nodal cells (SNCs), by modulating $Ca^{2+}$ content of nearby sarcoplasmic reticulum (SR) and local calcium release (LCR), and hypothesized that spatial coupling between mitochondria-SR-plasma membrane is important for the automaticity. In order to test our hypothesis, we took an advantage of transgenic mice (Tg) with cardiac-specific overexpression of perilipin 2 (PLIN2), which induces accumulation of lipid droplets, especially around mitochondria, and partial swelling of SR. Electrocardiogram recording revealed increased variability of RR interval in PLIN2-Tg compared to WT mice. In isolated SNCs, the amplitude and size of LCR were significantly reduced and the occurrence of early LCR tended to increase in PLIN2-Tg. The variability of $Ca^{2+}$ transient cycle length tended to increase in SNCs of PLIN2-Tg. Cytosolic and mitochondrial ROS levels were increased in SNCs of PLIN2-Tg. Our findings suggest that myocardial steatosis induces sinoatrial node dysfunction, which is associated with increased ROS levels and impaired SR $Ca^{2+}$ handling.

### [1P-163]

### Wild-type troponin T overexpression on troponin T mutant-induced dilated cardiomyopathy rescued its ventricular arrhythmia.

*Hirofumi Maetani[1,4], Yuya Yamaguchi[1,2,3], Jun Tanihata[1,2,3], Shunsuke Baba[1,2], Sachio Morimoto[5], Susumu Minamisawa[1,2] ([1]*The Jikei University School of Medicine,* [2]*Department of Cell Physiology,* [3]*Division of Aerospace Medicine,* [4]*5th grade medical student,* [5]*International University of Health and Welfare School of Health Sciences at Fukuoka*)

### [1P-164]

### Diagnosis of cardiac amyloidosis using Raman spectroscopy

*Mizuki Yoshimoto[1], Takeshi Honda[2], Hiroki Takanari[3], Shin-ichiro Ynnagiya[3], Hirokazu Miki[4] ([1]*Tokushima university Faculty of Science and Technology,* [2]*Tokushima university Graduate School of Science and Technology for Innovation,* [3]*Tokushima university,* [4]*Tokushima university Hospital*)

Cardiac amyloidosis is a disease caused by amyloid deposition in the heart, resulting in poor prognosis due to severe heart failure and arrhythmias. Especially in the case of AL type amyloidosis, the disease progresses more quickly than ATTR type. With the recent development of therapeutic agents for ATTR amyloidosis, rapid and accurate classification of amyloidosis is required. In this study, we aimed to distinguish between AL and ATTR by Raman spectroscopy based on differences in molecular structure of the amyloid precursor proteins. Congo Red staining and polarized microscopy were used to identify the location of amyloid deposition in samples obtained from two patients each with AL and ATTR cardiac amyloidosis. Raman spectra of normal and amyloid-deposited areas were measured using a Raman microscope. We found a characteristic peak in the amyloid-deposited area at a Raman shift of 1680 $cm^{-1}$. A principal component analysis of the spectra could clearly distinguish between amyloid-deposited and normal areas. Moreover, the distribution of principal components was different between AL and ATTR, suggesting that Raman spectroscopy may lead to a rapid diagnosis of cardiac amyloidosis.

### Poster Presentation

[1P]
**Respiration**

March 14 (Tue.), 12:10 - 14:10, Poster Room

### [1P-165]

### Metabolic and cardiorespiratory responses to environmental temperature are suppressed by dexmedetomidine in spontaneously breathing newborn rats

*Eishi Nakamura[1], Nana Hashizume[1], Hinata Kubota[1], Ryouji Ide[1], Yoichiro Kitajima[1], Kei Anai[1], Chikako Saiki[1] ([1]*The Nippon Dental University, Graduate School of Life Dentistry at Tokyo, Department of Physiology*)

We examined whether dexmedetomidine (DEX), an $\alpha_2$-adrenoceptor/imidazoline 1 receptor agonist, suppresses metabolic and cardiorespiratory responses to cool or warm environmental temperature (Te) in newborn rats. Wistar rat (3 to 6-day-old, 7.2 to 13.6 g, n=47) was inserted an abdominal catheter for drug administration and subcutaneous electrodes for ECG recording under isoflurane anesthesia. After recovery, the rats were divided into four groups receiving normal saline (NS) or DEX (50μg/kg) at Te=27 or 40°C. Firstly, we obtained values at Te=34 °C (i.e. thermoneutral range) as control (100%). In NS group, oxygen consumption, body temperature, heart rate and respiratory frequency (mean, %) were 160, 87, 90 and 105 at 27°C, respectively, and 103, 108, 106 and 85 at 40 °C, respectively. In DEX group, these values (mean, %) were 99, 84, 62 and 48 at 27°C, respectively, and 74, 107, 84 and 37 at 40 °C, respectively. The absolute values of DEX group were significantly less than NS group at both Te (P<0.05). Our results suggest that metabolic and cardiorespiratory responses to environmental temperature are suppressed through $\alpha_2$-adrenoceptor/imidazoline 1 receptor activation in newborn rats.

### [1P-166]

### Resting-State fMRI Connectivity Analysis on Psychiatric Symptoms for COPD Patients

*Akira YOSHIKAWA[1,2], Yuri Masaoka[1], Masaki Yoshida[3], Nobuyoshi Koiwa[4], Ryo Manabe[5], Satomi Kubota[6], Natsuko Iizuka[6], Motoyasu Honma[1], Shotaro Kamijo[1,7], Masahiro Ida[8], Masahiko Izumizaki[1] ([1]*Department of Physiology, Showa University, School of Medicine,* [2]*Division of Health Science Education, Showa University, School of Nursing and Rehabilitation Sciences,* [3]*Department of Ophthalmology, The Jikei University, School of Medicine,* [4]*Department of Health and Science, University of Human Arts and Sciences,* [5]*Department of Respiratory Medicine and Allergology, Showa University, School of Medicine,* [6]*Department of Neurology, Showa University, School of Medicine,* [7]*Department of Physiology, Showa University, School of Pharmacy,* [8]*Department of Radiology, Mito Medical Center*)

Patients with chronic obstructive pulmonary disease (COPD) suffer from depression and/or anxiety due to dyspnea and inactive state. COPD patients often have dyspnea and depression/anxiety despite they have normal blood oxygen levels. In this study, we hypothesized that brain networks regarding perception of dyspnea and emotions might be altered in such patients.
18 COPD patients and age-matched 24 control subjects participated in the present study. Depression and anxiety levels were measured with the Hospital Anxiety and Depression (HAD). For brain network analysis, resting-state fMRI was recorded and evaluated the strength of functional connectivity (FC) in all brain area. The correlation coefficient between HAD and FC was statistically analyzed. COPD patients had a significantly negative correlation between HAD and FC between the middle temporal gyrus- paracentral lobule. These results suggest that level of symptom in COPD patients may not only determined by blood oxygen levels but also need to observe psychiatric symptoms reflected in brain function.

### [1P-167]

### Role of hydrogen sulfide in the modulation of inspiratory/expiratory balance in the respiratory center as compared to inhibition of synaptic transmission.

*Minako Okazaki[1,2], Tadachika Koganezawa[1,3] ([1]*Dept Neurophysiol, Fac Med, Univ Tsukuba, Tsukuba, Ibaraki, Japan,* [2]*Dr. Prog Neurosci, Grad Sch Comp Human Sci, Univ Tsukuba, Ibaraki, Japan,* [3]*Transborder Med Res Ctr, Univ Tsukuba, Ibaraki, Japan*)

We have reported that hydrogen sulfide ($H_2S$) contributes to central respiratory pattern generation. However, the underlying neural mechanism is still unclear. In this study, we aimed to evaluate the roles of $H_2S$ in each subregion of the medullary respiratory center on respiratory pattern generation. We performed *in situ* arterially perfused preparations of rats. The central respiratory outputs were recorded from the phrenic and vagus nerves. The synthesis of $H_2S$ was inhibited by an inhibitor of cystathionine β-synthase (CBS), an $H_2S$-producing enzyme. Local inhibition of the CBS in the Bötzinger complex (BötC) or the preBötzinger complex (preBötC) increased the respiratory frequency, resulting from shortening the inspiratory and expiratory durations. On the other hand, the CBS inhibition in the ventral respiratory group (VRG) decreased the respiratory frequency, resulting from longer expiration. In the BötC and the preBötC, the effects of CBS inhibition were similar to those of the inhibition of excitatory synaptic transmission. In the VRG, the CBS inhibition induced the opposite effects to those by suppressing inhibitory synaptic transmission. These results suggested that $H_2S$ in the respiratory center modulates and maintains the respiratory phase duration and its balances, possibly caused by facilitating the excitatory transmission and/or attenuating the inhibitory transmission.

### [1P-168]

### Activation of astrocytes in the ventrolateral medulla via PAR1 and modulation of respiratory rhythm in newborn rat brainstem-spinal cord preparations

*Hiroshi Onimaru[1], Isato Fukushi[2], Keiko Ikeda[3], Itaru Yazawa[4], Kotaro Takeda[5], Yasumasa Okada[6], Masahiko Izumizaki[1] ([1]*Department of Physiology, Showa University School of Medicine,* [2]*Faculty of Health Sci., Aomori Univ. of Health and Welfare, Aomori, Japan,* [3]*Dept. Oral Physiol, Showa Univ. Sch. of Dent., Tokyo, Japan,* [4]*Dept. Food & Nutrition, Kyushu Nutrition Welfare Univ, Fukuoka, Japan,* [5]*School of Health Sciences, Fujita Health Univ., Toyoake, Aichi, Japan,* [6]*Clinical Res. Ctr., Murayama Med. Ctr., Musashimurayama, Tokyo Japan*)

Proteinase-activated receptor-1 (PAR1) is expressed in astrocytes of various brain regions and is involved in the modulation of synaptic activity. Here we report the effects of PAR1-selective agonist TFLLR on respiratory rhythm in the brainstem-spinal cord preparation. The preparation was isolated from newborn rats (P0-P4) under deep anesthesia, transversely cut at the rostral medulla, and superfused with the artificial cerebrospinal fluid (25-26˚C). The cellular responses were detected by calcium imagings or membrane potential recordings. Application of 10 μM TFLLR induced a transient increase of calcium signal in 58% of cells in the ventrolateral medulla. More than 85% of responding cells were also activated by low (0.2 mM) $K^+$ solution, suggesting that they were astrocytes. Respiratory related neurons in the ventrolateral medulla showed moderate membrane hyperpolarization (-1.6 mV) in association with 10% decrease of C4 burst rate during 10 μM TFLLR. In the presence of 50 μM theophylline or 10 μM bicuculline, TFLLR did not induce excitatory effects on respiratory rhythm. In conclusion, activation of astrocytes via PAR1 induced weak inhibitory modulation of respiratory rhythm.

### [1P-169]

### Activation of the lateral habenula causes stress-induced respiratory responses in rats.

*Riko Mizukami[1,3], Masayuki Matsumoto[2,4], Tadachika Koganezawa[1,4] ([1]*Department of Neurophysiology, Institute of Medicine, University of Tsukuba, Tsukuba, Ibaraki, Japan,* [2]*Docter's Program in Neurosciences, Graduate School of Comprehensive Human Sciences, University of Tsukuba, Tsukuba, Ibaraki, Japan,* [3]*Department of Cognitive and Behavioral Neuroscience, Institute of Medicine, University of Tsukuba, Tsukuba, Ibaraki, Japan,* [4]*Transborder Medical Research Center, University of Tsukuba, Tsukuba, Ibaraki, Japan*)

Psychological stress triggers a variety of responses. It induces behavioral reactions such as the fight or flight response, the freezing response, and autonomic changes, for example, cardiovascular and respiratory responses. Recent studies have suggested that the lateral habenula (LHb), excited by aversive stress, is one of the critical brain regions for autonomic cardiovascular changes in psychological stress. However, the involvement of LHb in neurogenic respiratory regulation is still unclear. In this study, we hypothesized that the LHb regulates stress-induced respiratory responses by controlling the respiratory center in the brainstem.
To approach this hypothesis, we employed urethane-anesthetized rats, activated the LHb by electrical stimulation, and observed the effects on respiratory activity. As a result, activation of the LHb induced increases in the respiratory frequency and the tidal volume, which is also observed in stress-induced respiratory responses. Moreover, these changes were dependent on stimulus intensity. These results suggested that the LHb neurogenically modulates respiratory activity. The LHb-respiratory center circuits may be essential for respiratory regulation during psychological stress. [TK1]About 1,200 characters

### [1P-170]

### Early postnatal development in cell types and activity patterns of inspiratory neurons in the preBötzinger complex of mice

*Yoshihiko Oke[1], Fumikazu Miwakeichi[2,3], Yoshitaka Oku[1], Johannes Hirrlinger[4,5], Swen Hülsmann[6,7] ([1]*Division of Physiome, Department of Physiology, Faculty of Medicine, Hyogo Medical University, Japan.,* [2]*Department of Statistical Modeling, The Institute of Statistical Mathematics, Japan.,* [3]*Department of Statistical Science, School of Multidisciplinary Sciences, The Graduate University for Advanced Studies, Japan.,* [4]*Carl-Ludwig-Institute for Physiology, Faculty of Medicine, University of Leipzig, Germany. 5Department of Neurogenetics, Max Planck Institute for Multidisciplinary Sciences, Germany.,* [5]*Department of Neurogenetics, Max Planck Institute for Multidisciplinary Sciences, Germany.,* [6]*Clinic for Anesthesiology, University Medical Center, Germany.,* [7]*Research Center for Nanoscale Microscopy and Molecular Physiology of the Brain, Germany.*)

Spontaneous inspiratory rhythm is generated in the pre-Bötzinger complex (preBötC). Here, to examine whether and how the inspiratory neuronal network in the preBötC develop during the early postnatal period, we first quantified the composition of the population of inspiratory neurons between postnatal day 1 (p1) and p10. To classify inspiratory neuron types, we applied calcium imaging to the medullary transverse slices in double-transgenic mice expressing EGFP in GlyT2+ neurons and tdTomato in GAD65+ neurons. We found that putative excitatory and glycinergic neurons formed a majority of the population of inspiratory neurons and the composition rates of these two inspiratory neurons inverted at p5-6. We also found that activity patterns of both inspiratory neurons became significantly well synchronized with inspiratory rhythmic bursting pattern in the preBötC within the first postnatal week. GABAergic and GABA-glycine co-transmitting inspiratory neurons formed a small population just after birth, which almost disappeared until P10. In conclusion, the inspiratory neuronal network in the preBötC might mature at the level of both neuronal population and neuronal activities during early postnatal development.

### Poster Presentation

### [1P]
### Urinary organ, Renal function, Urination

March 14 (Tue.), 12:10 - 14:10, Poster Room

### [1P-171]

**Dilatation of descending vasa recta regulated by NO-sGC-cGMP pathway after hypoxia/re-oxygenation**

*Minze Xu[1], Falk Lichtenberger[1], Cem Erdogan[1], Pontus Persson[1], Andreas Patzak[1], Pratik Khedkar[1] ([1]*Charité – Universitätsmedizin Berlin*)

Purpose: Acute kidney injury (AKI) goes along with impaired renal medullary blood flow. Hypoxia is involved in the pathogenesis of renal damage and can influence the function of renal microvessels. Dilating these vessels improves renal medullary flow, and this may be renoprotective. Here, we characterize the NO-sGC-cGMP signaling pathway in descending vasa recta (DVR), and test potential vasodilators after hypoxia/re-oxygenation. Methods: Rat DVR were isolated and perfused under isobaric conditions. A hypoxia chamber was used to provide the hypoxia (0.1% oxygen) environment. Results: Sildenafil, a PDE5 inhibitor ($10^{-6}$ mol/l), induced vasodilatation in angiotensin II (Ang II, $10^{-6}$ mol/l)-pre-constricted vessels. In L-NAME ($10^{-4}$ mol/l) pre-treated and Ang II ($10^{-6}$ mol/l) pre-constricted vessels, BAY 60-2770, an sGC activator ($10^{-6}$ mol/l), dilated vessels NO-independently. The application of Ang II ($10^{-12}$ to $10^{-6}$ mol/l) showed a stronger constriction effect in vessels after hypoxia/re-oxygenation. Sildenafil failed to dilate the vessels after hypoxia/re-oxygenation. SNP, an NO donor ($10^{-3}$ mol/l), and BAY 60-2770 both induced dilatation in DVR, while BAY 60-2770 dilated DVR faster than SNP under these conditions. Conclusion: The results emphasize the role of the NO-sGC-cGMP signaling pathway in regulating the tone of renal medullary micro-vessels. The sGC activator BAY 60-2770seems to be the best choice to restore renal blood flow after hypoxia/re-oxygenation compared to SNP as well as sildenafil.

### [1P-172]

**Effects of trigonelline, a regulator of vascular function, on human renal glomerular endothelial cell function**

Yuri Nonoshita[1], Yuji Minami[1,2], *Katsuko Kajiya[1,2] ([1]*Graduate School of Agriculture, Forestry and Fisheries, Kagoshima University,* [2]*Faculty of Agriculture, Kagoshima University*)

Renal glomerulus, a bundle of capillaries lined by delicate fenestrated endothelia, plays an important role in urine production. Human renal glomerular endothelial cells (HRGECs) are the first barrier in ultrafiltration. HRGEC dysfunction causes glomerular diseases such as glomerulonephritis or glomerulosclerosis, ultimately leading to kidney failure. Therefore, it is important to conduct research on the protection of HRGECs. While searching for various agricultural products that improve vascular function, we found Sakurajima daikon, a special product in Kagoshima prefecture, Japan. The functional compound contained in Sakurajima daikon was identified as trigonelline. We hypothesized that trigonelline may delay the progression of renal failure if it can improve the function of the renal glomerulus. In this study, we investigated the effects of trigonelline on vasoactive substances [vasodilator nitric oxide (NO) and vasoconstrictor endothelin-1 (ET-1)] in HRGECs.The activation of endothelial NO synthase (eNOS) by phosphorylate of Ser1177 and expression of ET-1 in HRGECs were assessed by western blotting and flow cytometry. Bradykinin, acetylcholine, and vascular endothelial growth factor were used as positive controls. HRGECs were treated with trigonelline at various concentrations for a period of time and the effect of trigonelline on concentration of calcium ion and the expression levels of phosphorylated eNOS and/or ET-1 was also examined.

### [1P-173]

**Physiological importance and association with podocyte function of the tRNA modification enzyme, *CDKAL1*.**

*Hiroko Nagata[1], Yuu Nagayoshi[1], Takeshi Chujo[1], Hitoshi Nakazato[2], Kazuhito Tomizawa[1] ([1]*Department of Molecular Physiology, Faculty of Life Sciences, Kumamoto University,* [2]*Department of Pediatric Health Education, Faculty of Life Sciences, Kumamoto University*)

A genome-wide association study reported that Cdk5 regulatory associated protein 1-like 1 (*CDKAL1*) as a causative gene of type 2 diabetes. We have reported that *CDKAL1* thiomethylates transfer $RNA^{Lys}$ (UUU) at position 37. Moreover, the mutation of *CDKAL1* gene causes impairment of insulin processing and reduction of insulin secretion in pancreatic β cells. In addition, the mutation of *CDKAL1* gene has reported one of the risk factors for progression of chronic kidney disease (CKD). However, the physiological role of *CDKAL1* in kidney is almost unclear. We generated systemic *Cdkal1* knockout (KO) mice and these mice showed *Cdkal1* is present in podocyte by immunostaining of glomeruli. Moreover, systemic *Cdkal1* KO mice show the phenotype of albuminuria. Using electron microscopy analysis, podocyte foot process effacement was observed in the glomeruli of *Cdkal1* KO mice. Next, we generated *Cdkal1* KO podocyte culture cells using CRISPR-Cas9 systems. We found that podocin, a podocyte-specific protein, expression was reduced in *Cdkal1* KO cells. In summary, we found that *Cdkal1* is associated with podocyte function and podocin translation. These results suggest that *Cdkal1* is important for anti-albuminuria effects and CKD progression without mediating type 2 diabetes.

### [1P-174]

**Parathyroid hormone-related protein suppresses spontaneous contractions of the detrusor smooth muscle by activating BK channels.**

*Wataru Kudo[1], Hikaru Hashitani[1] ([1]*Department of cell physiology, Nagoya city University Graduate school of medicine*)

Parathyroid hormone-related protein (PTHrP) that is released from detrusor smooth muscle (DSM) cells upon bladder distension suppresses DSM spontaneous phasic contractions (SPCs). Since excessive SPCs would cause urinary urgency by stimulating afferent nerves, endogenous PTHrP is considered to facilitate urine storage by attenuating SPCs. However, detail mechanisms underlying PTHrP-induced suppression of SPCs remain to be explored. Perforated whole-cell patch clamp technique was applied to enzymatically-isolated DSM cells taken from female rat bladders. SPCs of DSM strips were also recorded isometrically, while spontaneous Ca2+ transients were visualised using Cal-520 loaded DSM preparations. In isolated DSM cells in which spontaneous transient outward currents (STOCs) arising from the opening of BK channels were developed, PTHrP (10 nM) increased STOCs. PTHrP (10 nM) increased the depolarization-induced outward currents only above +20 mV. PTHrP (10 nM) suppressed SPCs in an iberiotoxin (100 nM), a BK channel blocker, sensitive manner. PTHrP (10 nM) diminished intercellular spread of the spontaneous Ca2+ transients without reducing their ampilitude. These results indicated that PTHrP suppress SPCs in DSM through the activation of BK channels lowering the probability of propagation of L-type voltage-gated Ca2+ channels dependent action potentials.

### [1P-175]

**Regulation of renal gluconeogenesis by inter-organ crosstalk**

*Ryo Hatano[1], Takashi Miki[1] ([1]*Department of Medical Physiology, Chiba University Graduate School of Medicine*)

Gluconeogenesis is essential for the maintenance of body energy homeostasis in fasting state, On the other hand, abnormal upregulation of hepatic gluconeogenesis cause hyperglycemia as observed in Diabetic patients. Generally, liver is considered to be a major organ for gluconeogenesis, but kidney and intestine also generate glucose from glucogenic substrates. In prolonged fasting state, kidney plays a pivotal role in the regulation of blood glucose via gluconeogenesis in the proximal tubules. Pancreatic hormones, insulin and glucagon, are the most important hormones regulating hepatic gluconeogenesis. Thus, hepatic gluconeogenesis is dynamically regulated in response to the blood glucose concentration. However, the regulation mechanism for renal gluconeogenesis still remains unclear. Hepatic gluconeogenesis is induced at early stage of fasting, whereas renal gluconeogenesis is induced at late stage of fasting, suggesting that regulatory machinery is different among these organs. We recently identified that liver plays important role in the regulation of renal gluconeogenesis. In the present study, we will show a novel regulatory mechanism for renal gluconeogenesis via liver-kidney inter-organ crosstalk.

### [1P-176]

### Effects of novel TRPC3/6 channel inhibitor L862 on PAN-induced nephrotic syndrome model rats

*Reiko Sakaguchi[1], Yoshimune Matsuda[1,2], Ryo Okada[1,3], Marie Konomi[1,2], Ryu Nagata[4], Masayuki X. Mori[1] ([1]*Bio-materials and Chemistry, School of Medicine, University of Occupational and Environmental Health,* [2]*Faculty of Environmental Engineering, The University of Kitakyushu,* [3]*Human, Information and Life Sciences, School of Health Sciences, University of Occupational and Environmental Health,* [4]*Graduate School of Pharmaceutical Sciences, Osaka University*)

Nephrotic syndrome is a dysfunction of kidney that is diagnosed by proteinuria concomitantly with low serum albumin level caused by the defect of podocytes in glomerulus. It has been reported that Transient Receptor Potential Canonical 6 (TRPC6) mutations found in focal segmental glomerulosclerosis (FSGS) patients, a common type of nephrotic syndrome, often cause hyperactivated channel currents. Regarding this mechanism, we have previously reported that the disruption of $Ca^{2+}$-dependent inactivation of TRPC6 channel current results in prolonged cation influx, which leads to disorganization of cytoskeleton in the podocytes. However, compensatory increase of TRPC3 expression is reported in TRPC6 knockdown condition, making it rational to block both channels to suppress nephrotic syndrome. Here, we developed “L862”, a novel selective inhibitor of both TRPC3 and TRPC6 channels. The effect of L862 is tested in puromycin aminonucleoside (PAN)-induced nephrotic rats. L862 exerted significant improvement of proteinuria and serum albumin in PAN-induced nephrotic rats, along with the improvement of morphological observations of kidney sections. These results suggest that L862 would be a promising therapeutic compound for channel-related diseases such as nephrotic syndrome.

### [1P-177]

### Elucidation of the water secretion mechanism of Boui-ougi-to and its application to cancer therapeutic agents

*Taro Suzuki[1], Haruna Saito[1], Kaori Sato-Numata[1], Tomohiro Numata[1] ([1]*Akita Univ.*)

Boui-ougi-to (BOT) is a Japanese herbal medicine that causes the body to excrete water and is effective in relieving water weight and chronic renal failure. So far, it is used to avoid dialysis for about 13.3 million chronic renal failure patients in Japan, but its medicinal mechanism is poorly understood.In order to investigate the effect of BOT on cell volume, HEK293T cells derived from human kidney, a representative cell line, were used here. Coulter counter assay showed a concentration-dependent cell volume decrease upon administration of BOT. This cell volume decrease was significantly suppressed by the addition of $Cl^-$ channel inhibitors and $K^+$ inhibitors. Furthermore, biochemical measurements revealed that exposure to BOT for more than one day caused PI-positive cell death. These results show that BOT induces apoptosis by KCl efflux.This apoptosis-inducing effect of BOT was then tested on human cancer cell lines such as HeLa and Caco-2. We found that one-day BOT exposure induced apoptotic death due to decreased cell volume and increased caspase activity.From these results, it was found that BOT has an effect of apoptotic cell volume decrease as well as a water excretion effect in the body. This effect can be expected to induce apoptotic death in cancer cells.

### Poster Presentation

### [1P]
### Autonomic nervous system

March 14 (Tue.), 12:10 - 14:10, Poster Room

### [1P-178]

**Effect of Drinking Warm Water on Cardiovascular Autonomic Responses.**

*Om Lata Bhagat[1] ([1]*Additional Professor, Department of Physiology, All India Institute of Medical Sciences, Jodhpur, Rajasthan, India*)

Water ingestion has multi-system effects on the human body. It is reported in the literature that cold water enhances vagal modulation, but the impact of warm water ingestion has not been explored much. The present study was planned to investigate the effect of ingesting 500ml of water at $25^0$C, $37^0$C and $45^0$C. Volunteers (18-21years; n=10) were inducted into the study after consent. BP, ECG, and chest movements were recorded at rest and then for 30 mins post water ingestion. RSA, HRV and BRS were calculated in 5min epochs. After ingestion of water, heart rate showed an initial rise, then a middle fall and finally reaching back to baseline. The SBP and DBP not changed after water consumption at $45^0$C and showed a fall of ~10mmHg in BP at $25^0$C. BRS increased after water ingestion at $25^0$C and decreased after $37^0$C and $45^0$C. HRV significantly increased after 15mins of drinking. Temperature and time interaction had a significant effect on HF (p=0.043). On drinking water at $45^0$C, minimal changes were observed in all the cardiovascular parameters but RSA. It can be concluded that warm water ingestion modulates cardiovascular autonomic responses, that too, towards parasympathetic dominance.

### [1P-179]

**Dried bonito-derived extract regulates inflammatory responses in the central nervous system and blood brain barrier, associated with the modification of the cholinergic system**

Yoshinori Hokari[2], Aya Sekine[2], Yuko Kai[1], Shino Oikawa[1], Asuka Mano[1], Hisayuki Ohata[1], Shuei Sugama[3], *Yoshihiko Kakinuma[1] ([1]*Nippon Medical School,* [2]*Maruhachi Muramatsu Inc.,* [3]*International University of Health and Welfare*)

A non-neuronal cardiac cholinergic system (NNCCS), which is equipped by cardiomyocytes, is considered to be essential for cardiac homeostasis, according to effects on energy metabolism modulation, angiogenesis-acceleration, and on cell-cell communication, leading to myocardium salvage. Furthermore, it influences blood brain barrier functions via the vagus nerve. However, it remains to be fully elucidated whether any substance activates this system. With Katsuo extract (KE) derived from dried bonito, in vitro and in vivo studies were performed whether KE activates the NNCCS and influences the associated physiological responses, specifically focusing on anti-inflammatory property and potentiation of blood brain barrier (BBB) functions. KE upregulated the NNCCS as well as the parasympathetic nervous system. Murine models disclosed that KE exerted anti-inflammatory action by suppressing cytokine production and microglial activation against pathogenic and non-pathogenic factors. Furthermore, KE upregulated expression of BBB component proteins, strengthened the function, and downregulated an aggravation level of in a brain injury model, and finally modulated murine higher brain functions by suppressing depressive or anxiety-like behaviors. This study indicates that KE is involved in anti-inflammatory and blood brain barrier consolidation effects, and the NNCCS activation. The intake might be effective in influencing pathophysiology of neuroinflammation-related diseases.

### [1P-180]

**Activation of Phox2B-positive neurons in the dorsal medulla induced sucking and hiccup**

*Makito Iizuka[1], Keiko Ikeda[2], Hiroyuki Igarashi[3], Kazuto Kobayashi[4], Hiroshi Onimaru[1], Masahiko Izumizaki[1] ([1]*Dept Physiol, Showa Univ Sch Med, Tokyo, Japan,* [2]*Dept Oral Physiol, Showa Univ Sch Dent, Tokyo, Japan,* [3]*Dept Physiol Pharmacol, Schulich Sch Med Dent, Robarts Res Inst, Western Ontario Univ, Canada,* [4]*Dept Molecular Genetics, Inst Biomed Sci, Fukushima Med Univ Sch Med, Fukushima, Japan*)

We found that blue light stimulation of the dorsal skull of transgenic neonatal rats in which Phox2B-positive neurons expressed one of the channelrhodopsin variants, ChRFR (C167A), caused rhythmic opening/closing movements of the mouth under conscious free-moving conditions. In anesthetized transgenic neonatal rats, the oral cavity showed a rhythmic negative pressure during this movement. After euthanasia, the rat's stomach was filled with air that would flow from the catheter to the pressure transducer in the oral cavity. Therefore, this movement should be both sucking and swallowing. We also noticed another type of movement, which was a hiccup-like movement. That is, the thorax near the sternum was depressed during swelling movement of the abdomen. During the hiccup-like activity, the masseter and digastric muscles showed short burst activity synchronized with burst activity in the diaphragm. On the other hand, the masseter and digastric muscles active alternately during sucking-like movements. We concluded that activation of Phox2B-positive neurons in the dorsal medulla induced sucking and hiccup movements. COI: No.

### [1P-181]

**Comparison of effects of single session of classical music alone and music with guided meditation on heart rate variability in healthy individuals**

*Smruti Bhalendu Vaishnav[1], Hasmukh Shah[2], Tejas Prajapati[3], Bhalendu Vaishnav[4] ([1]*Professor and Head, Department of Obstetrics and Gynecology Pramukhshwami Medical College, Bhaikaka University, Karamsad, Gujarat, India,* [2]*Professor and Head, Department of Physiology, Pramukhswami Medical College, Bhaikaka University, Karamsad, Gujarat, India,* [3]*Assistant Professor,Department of Physiology, Pramukhswami Medical College, Bhaikaka University, Karamsad, Gujarat, India,* [4]*Professor and Head, Department of Medicine, Pramukhswami Medical College, Bhaikaka University, Karamsad, Gujarat, India*)

Heart rate variability (HRV) is known to be positively influenced by music and meditation. We compared effects of : single session of Indian classical instrumental music with (2) guided meditation combined with same music on heart rate variability in 30 individuals. Similar studies in literature are limited. Pre and post intervention HRV was measured after a session of music played on day one; and a session of guided meditation combined with same music which was played on day 2. (Duration: 16 minutes each.) Instrumental music was in Raag Yaman belonging to of North Indian classical music. Guided meditation script was structured according to the ancient Indian practice of Yoganidra. It comprised of stages of resolution, breath awareness, rotation of consciousness, visualization and relaxation. Results: SDNN, RMSSD, NN50, pNN50 increased significantly after listening music (p<0.05) and greater with combination of music and guided meditation (p=0.01). LF (n.u.) was reduced after music and guided meditation. Conclusion: Guided meditation session combined with classical music results in greater sympatho-vagal balance (LF/HF ratio) as compared to the same music alone.

### [1P-182]

**Locomotion and sympathetic cardiovascular responses by orexinergic neurons in rats**

*Emi Narai[1], Yuki Yoshimura[1], Akihiro Yamanaka[2], Naoya Kataoka[3,4], Kazuhiro Nakamura[3], Tatsuo Watanabe[1], Satoshi Koba[1] ([1]*Division of Integrative Physiology, Tottori University Faculty of Medicine,* [2]*Department of Neuroscience II, Research Institute of Environmental Medicine, Nagoya University,* [3]*Department of Integrative Physiology, Nagoya University Graduate School of Medicine,* [4]*Nagoya University Institute for Advanced Research*)

Increased orexinergic nervous system activity contributes to blood pressure elevation, yet its involvement in cardiovascular adjustments during exercise is unknown. This study aimed to determine whether orexinergic neurons play a role in autonomic cardiovascular regulation during locomotor exercise. In anesthetized orexin-Cre transgenic rats, optogenetic stimulation (0.5-s laser on/2.5-s off, repeated 20 times) of orexinergic neurons immediately elicited sympathoexcitation, that was synchronized with the intermittent manner of laser illumination. In freely-moving conscious rats, optogenetic stimulation (1-s laser on/5-s off, repeated 3 times) of orexinergic neurons at rest rapidly induced exploration-like behavior including locomotion and blood pressure elevation, and belatedly increased heart rate. Moreover, 2-s optogenetic inhibition of orexinergic neurons during voluntary wheel running immediately suppressed locomotor activity and blood pressure elevation. These observations suggest that excitation of orexinergic neurons is essential for simultaneous regulation of somatomotor and sympathetic nervous systems required for motivation-driven locomotion. This study contributes to our understandings of central autonomic circuitry mechanisms engaged for locomotor exercise. (COI: No)

### [1P-183]

### Electrical microstimulation of the peripheral sympathetic nerve enhances glycolysis and glucose release simultaneously in the liver in rats

*Daisuke Sato[1], Nozomi Imaizumi[1], Ryoichi Banno[2,3], Masataka Kusunoki[2], Takao Nakamura[4] ([1]*Department of Biochemical Engineering, Graduate School of Science and Engineering, Yamagata University,* [2]*Research Center of Health, Physical Fitness and Sports, Nagoya University,* [3]*Department of Diabetes and Endocrinology, Nagoya University Hospital,* [4]*Department of Clinical Engineering, Faculty of Health Sciences, Japan Healthcare University*)

It is suggested that the hypothalamic stimulation elevates peripheral glucose uptake. We previously reported that electrical microstimulation (MS) of the peripheral sympathetic nerve enhanced glucose uptake independently of insulin action in rats while the blood glucose level changed little. In the present study, we evaluated the effects of the MS on glucose metabolism in the liver. Under anesthetic condition, we conducted microneurography to detect the peripheral sympathetic nervous signal in the unilateral sciatic nerve in male rats, and stimulated the sympathetic nerve fascicle with the microelectrode for 60 min (MS group; n=6). After the MS, we measured expressions of mRNA involved in glucose metabolism in the liver, and compared them with those in non-stimulated, control rats (control group; n=6). As a result, glucose-6-phosphatase (*G6PC*), *ChREBP*, and *SREBP1c* expressions were significantly higher than those, respectively, in the control groups (P<0.05). G6PC and ChREBP are involved with glucose release whereas SREBP1c mediates glycolysis via glucokinase. Therefore, these results suggest that the MS may enhance glycolysis and glucose release simultaneously in the liver.

### [1P-184]

### Differences in autonomic vasomotor responses and their interactions during trigeminal afferent stimulation in rat gingiva

*Yunosuke Okada[1], Toshiya Sato[2], ST Islam[2], Masato Saitoh[1], Hisayoshi Isii[2] ([1]*Div. Pediatric., Dept. Sch. Dent., Health Sci Univ. Hokkaido,* [2]*Div. Physiol., Dept. Sch. Dent., Health Sci Univ. Hokkaido*)

Blood flow (BF) in the gingiva, which consists of interdental papilla (IP) and attached (AG) and marginal gingiva (MG), is important in the maintenance of gingival function. Marked BF changes mediated by autonomic nerves may be essentialfor gingival hemodynamics. However, differences in autonomic vasomotor responses in different parts of the gingiva and their functional significance are unclear. We examined the differences in autonomic vasomotor responses and their interactions in the gingiva of anesthetized rats. Electrical stimulation of the central cut end of the lingual nerve (LN) elicited BF increases in IP, AG, and MG, with the increases being the greatest in IP. The BF increases evoked by LN stimulation were reduced by hexamethonium (90%), atropine (50%) and VIP antagonist (50%). The BF increase produced by acetylcholine was higher in IP than in AG, whereas that evoked by VIP agonist was greater in AG than in IP. Activation of the cervical sympathetic nerve decreased the gingival BF and inhibited LN stimulation-induced BF increases. Our results suggest that parasympathetic reflex vasodilation is i) more involved in the regulation of BF in IP than in AG or MG, ii) mediated by cholinergic (IP) and VIPergic system (AG), and iii) inhibited by excess sympathetic activity.

### [1P-185]

### Short-term Heart Rate Variability: A Physiological Technique to Detect Subclinical Cardiac Autonomic Neuropathy in Type 2 Diabetes Mellitus

*Phurpa Phurpa[1,2,3] ([1]*Department of Neurophysiology, Faculty of Medicine, Oita University,* [2]*Department of Physiology, Bangabandhu Sheikh Mujib Medical University, Dhaka, Bangladesh,* [3]*Department of Basic Sciences, Faculty of Postgraduate Medicine, Khesar Gyalpo University of Medical Sciences, Thimphu, Bhutan*)

Background: Sub-clinical cardiac autonomic neuropathy (CAN), which precedes clinically overt CAN, often presents reduced heart rate variability (HRV) and yet may remain undetected by the conventional autonomic tests. Hence, this study explored the short-term HRV as a tool to detect sub-clinical CAN in type 2 diabetes mellitus (T2DM). Method: This study included 30 recently diagnosed and 54 long-term T2DM male patients (without any symptoms of CAN) with normal findings in Ewing battery of cardiovascular reflex tests. 30 age and body mass index matched healthy male subjects were recruited as controls. RMS Polyrite-D measured the short- term HRV of 5-minute ECG recording of study subjects and controls by using time and frequency domain methods. For statistical analysis, one way ANOVA and independent sample 't' test were used. Result: Standard deviation of all RR interval (SDNN), square root of mean squared differences of successive RR intervals (RMSSD), low frequency power (LF), and high frequency power (HF) in long-term T2DM patients, were significantly lower than that of healthy control and recently diagnosed T2DM patients. LF/HF ratio was significantly higher in long-term T2DM patients compared to healthy control and recently diagnosed ones. This latter group had significant reduction in SDNN, LF and HF compared to healthy controls. Conclusion: Short-term HRV, seems to be an important tool to detect sub-clinical CAN in T2DM.

### [1P-186]

### Alterations of Piezo-1 channel activity in group IV muscle afferents of Type 2 diabetic rats

*Rie Ishizawa[1], Norio Hotta[2], Han-Kyul Kim[1], Gary Iwamoto[1], Wanpen Vongpatanasin[1], Scott Smith[1], Masaki Mizuno[1] ([1]*UT Southwestern Medical Center,* [2]*Chubu University*)

Exercise blood pressure is exaggerated in Type 2 diabetes mellitus (T2D). We reported that group IV muscle sensory afferent discharge to mechanical stimulation is potentiated in T2D rats likely contributing to the exaggerated cardiovascular response during exercise. Piezo-1 channels play a crucial role in mechanotransduction. Thus, we hypothesized that the exaggerated group IV muscle afferent responsiveness in T2D is mediated by overactive Piezo-1 channels. Sprague-Dawley rats were given either a normal diet (CON) or a high fat diet in combination with a low dose of streptozotocin (T2D). Using a muscle-nerve preparation, group IV afferent neuronal discharge to the Piezo-1 activator, Yoda1, was assessed. T2D rats displayed increased fasting blood glucose (P<0.05) as well as plasma lipopolysaccharide (LPS) (P<0.05). The percentage of group IV fibers shown to be responsive to Yoda1 was similar between groups (CON, 18%; T2D, 25%). The response latency to Yoda1 tended to be shorter in T2D than CON (P=0.09). Peak response magnitude to Yoda1 was greater in T2D (0.27±0.35 vs. 0.97±1.38 Hz, P=0.035). The magnitude of neural discharge to mechanical stimulation was significantly correlated with plasma LPS but not glucose level. Moreover, LPS injection increased the Yoda1 induced neural discharge. These findings suggest that group IV muscle afferents may be sensitized by Piezo-1 overactivity in diabetic rats through a LPS-Piezo-1 pathway.

### [1P-187]

### Differences in surface temperature regulation are associated with hemodynamic changes in intra and extra-oral tissues mediated by trigeminal afferents

*Syed Taufiqul Islam[1], Toshiya Sato[1], Yunosuke Okada[2], Hisayoshi Ishii[1] ([1]*Div. Physiol., Dept. Sch. Dent., Health Sci Univ. Hokkaido,* [2]*Div. Pediatric., Dept. Sch. Dent., Health Sci Univ. Hokkaido*)

Surface temperature (Tm) in the skin or mucosa is critical in maintaining function and may be regulated by blood flow. Although the mechanisms underlying thermoregulation in the orofacial region are unclear, parasympathetic vasodilation evoked by trigeminal afferents may be crucial due to changes in blood flow velocity and magnitude. Here, using urethane-anesthetized, cervically vago-sympathectomy rats, we investigated the role of trigeminal afferents in regulating surface Tm and hemodynamics in the intra and extra-oral tissues. LN stimulation resulted in significant elevations in surface Tm, as well as vasodilation in the lower lip and tongue. Pretreatment with the autonomic ganglion cholinergic blocker hexamethonium significantly inhibited both the surface Tm and vasodilation evoked by LN stimulation in the lower lip. However, in contrast to the lower lip, hexamethonium significantly attenuated surface Tm increase on the tongue, but vasodilation induced by LN stimulation was almost the same. Our findings imply that parasympathetic reflex vasodilation regulates surface Tm in the orofacial region and that the interaction of parasympathetic and axon reflex vasodilation regulates surface Tm in some intraoral tissues.

### [1P-188]

### Effects of alternate nostril breathing exercise comprising of incremental duration and depth of breathing on heart rate variability and psychological wellbeing in Indian medical students

*Rutam Bhalendu Vaishnav[1], Dr. Hasmukh Shah[2] ([1]*Undergraduate student, Pramukhswami Medical College, Bhaikaka University, Karamsad, Gujarat, India,* [2]*Professor and Head, Department of Physiology, Pramukhswami Medical College, Bhaikaka University, Karamsad, Gujarat, India*)

Pranayama is Indian yogic practice aimed at enhancing health. We studied effects of two Alternate Nostril Breathing (ANB) cycles comprising of incremental ratios of inspiration, end-inspiratory pause and expiration on heart rate variability and psychological well-being in 30 medical students. These breathing sequences have been sparsely reported. Two ANB cycles of inspiration-pause-expiration duration of 4-6-6 seconds and 4-8-8 seconds were performed on day 1 and day 2 respectively. (8 minutes daily). Spectral HRV indices were recorded. Results: Statistically significant change in SDNN, RMSSD after both cycles, more after cycle having longer breath-holding and expiration time (4-8-8 seconds). There was significant increase in HF (n.u.), reduction in LF (n.u.), and in LF/HF ratio during both cycles, greater with 4-8-8 seconds. Increase in total power was significant and greater with 4-8-8 seconds. Participants consistently reported reduction in number of thoughts and spontaneous experience of quietude persisting for several hours. Conclusion: Prolongation of end-inspiratory pause and expiration during alternate nostril breathing increases heart rate variability and produces quietening effect.

### [1P-189]

**Two-photon *in vivo* live imaging of neural activity in nucleus tractus solitarii in response to vagus nerve stimulation in mice**

*Azumi Hatakeyama[1], Masakazu Agetsuma[2], Daisuke Yamada[1], Hiroshi Kuniishi[3], Eri Takeuchi[3], Tomoko Kobayashi[2], Ryo Fujikawa[1], Akiyoshi Saitoh[1], Junichi Nabekura[2], Masayuki Sekiguchi[3] ([1]*Tokyo University of Science,* [2]*Institute for Physiological Sciences,* [3]*National Institute of Neuroscience, National Center of Neurology and Psychiatry*)

### Poster Presentation

### [1P]
### Physical fitness and sports medicine

March 14 (Tue.), 12:10 - 14:10, Poster Room

### [1P-190]

**Effect of occlusal state on postural alignment during trunk flexion**

*Mutsumi Takahashi[1], Yogetsu Bando[2], Katsuhiko Kitaoka[3,4], Keisuke Hata[4,5], Yoshihide Satoh[1] ([1]*Dept Physiol, Nippon Dent Univ, Niigata, Japan,* [2]*BANDO Dental Clinic, Ishikawa, Japan,* [3]*Dept Orthopedic Surgery, Kijima Hosp, Ishikawa, Japan,* [4]*Japan Handball Association, Tokyo, Japan,* [5]*Dept Rehabil, Kijima Hosp, Ishikawa, Japan*)

The aim of this study was to clarify the effect of occlusal state on postural alignment during trunk flexion. Participants were 16 female handball players. Postural alignment during trunk flexion was measured using a spinal shape analyzer. Thoracic kyphosis angle (TKA), lumbar lordosis angle (LLA), sacral slope angle (SSA), and spinal inclination angle (SIA) were analyzed. The occlusal state during measurement was set as three conditions: mandibular resting position (RP), clenching in centric occlusion (CO), and clenching while wearing a mouthguard (MG). Differences in postural alignment due to occlusal conditions were analyzed using repeated measure ANOVA. The occlusal state had a significant effect on postural alignment during trunk flexion. LLA and SIA showed significant differences between RP and CO, and between RP and MG, with higher values in RP. SSA was significantly different among all conditions, and RP>MG>CO. TKA was not significantly affected by occlusal condition. As a result of this study, it was clarified that the occlusal state during trunk flexion affects the lumbar lordosis angle, sacral slope angle, and spinal inclination angle. It was suggested that clenching with or without an oral appliance may contribute to trunk stabilization.

### [1P-191]

**Differences in myokine secretion depending on the type of exercise**

*Naoyuki Himi[1], Emi Nakamura-Maruyama[1], Takehiro Nakamura[1] ([1]*Dept. Physiol. 2, Kawasaki Med. Sch.*)

Exercise therapy is effective as one of rehabilitation methods for functional recovery after stroke. The one possible mechanism for this recovery is the effects of myokines secreted from muscle by muscle contraction. However, the relationship between the type of exercise and myokines secretion has not been reported. Therefore, the intramuscular myokines after various exercises were evaluated in this study. Rats were devided to 5 groups, endurance exercise groups at three levels of intensity, voluntary exercise group, transcutaneous muscle electrical stimulation group. After exercise for 14 days, gastrocnemius and soleus muscle were desected and homogenized to measurement of brain-derived neurotrophic factor (BDNF) and insulin-like growth factor 1 (IGF-1) using ELISA or Western Blot. In the comparison of types of exercise, IGF-1 concentration in soleus was significantly higher in the electrical stimulation group than in the other groups, whereas no significant difference was found between the types of exercise and myokines concentration in the gastrocnemius. In comparison between muscles, BDNF in the endurance exercise groups and IGF-1 in the all exercise group in the soleus were significantly higher than those in the gastrocnemius. It was suggested that the types of both muscle metabolism and exercise may influence differences in myokines secretion.

### [1P-192]

**The Impact of Continuos Aquarobic Dance (CAD) on Fibroblast Growth Factor 19 (FGF-19)**

*Siti Baitul Mukarromah[1], Mohammad Arif Ali[1], Gustiana Mega Anggita[1], Ronny Lesmana[2], Aziiz Mardanarian[2] ([1]*Universitas Negeri Semarang, Semarang, Indonesia,* [2]*Universitas Padjajaran, Bandung, Indonesia*)

Fibroblast growth factor 19 (FGF-19) is a gut hormone with pleiotropic effects, it is secreted by the small intestine in response to feeding. FGF-19 has insulin like actions, such as promoting glucose uptake in adipocytes, synthesizing hepatic glycogen, and inhibiting gluconeogenesis. It also has a role in bile acid homeostasis and hepatic protein metabolism. Lower FGF-19 levels are observed in patients with type-2 diabetes mellitus and obese patients, suggesting that FGF-19 plays a role in weight loss. The purpose of this study to analyze the impact of Continuos Aquarobics Dance (CAD) on Fibroblast growth factor 19 (FGF-19) levels. Design, Randomized Experimental Pre-post-test control group design was carried out on 40 male from Sports Science Students, divided into 4 groups:P1 Group Intake Energy Bar (n = 10), P2 Group Aquarobic Dance (n = 10), P3 Group Intake Energy Bar and Aquarobic Dance (n = 10) and P4 Control Group (n = 10) treatment is given every day for 15 days, Intensity of Continous Aquarobic exercise 75% HR-max and data were collected body fat percentage, BMI, FGF-19 before and after treatment. Hypothesis testing using the test (One-Way Anova and Kruskal-wallis and mean difference test (Tukey HSD and Mann Whitney's) Result Fasted FGF-19 concentrations in plasma were 128 ± 20 and 155 ± 38 pg/ml for Group 2 and Group 3 respectively. FGF-19 was not affected in group 1 and remained unchanged in the 3-hour recovery period. Conversely, 90 minutes into the recovery period following aquarobics dance, FGF-19 was significantly lowered to 89 ± 18 pg/ml compared with the concentration before exercise ($P$ = 0.019), and this continued throughout the recovery phase to 60 ± 9 pg/ml ($P$ < 0.001 at 3 hours after exercise relative to that before exercise. However, the decrease in plasma FGF-19 concentration during the recovery phase after aquarobics dance was not statistically different compared to the control group. Conclusion, Intake Energy bar and continuos aquarobic dance can increase FGF-19 levels, there are differences in the effect of energy bar feeding and training an in athletes. Intake Energy bar and aquarobic exercise are more dominant in increasing FGF-19 levels in athletes. Plasma FGF-19 is reported to be increased with Continuos aquarobics dance and may thus contribute to the benefits of this type of exercise on glucose and lipid metabolism

### [1P-193]

**Non-targeted analysis of plasma volatile small molecules contained and water metabolic response by rapid fasting/dehydration with regular exercise**

*Kazuya Hasegawa[1], Yuya Yamacuchi[2] ([1]*Teikyo Heisei University,* [2]*Toho University*)

Purpose: The water metabolic response to rapid weight loss by simultaneous restriction of water and food, as practiced in weight class competitions, was examined in Sprague Dawley rats. We also attempted to identify plasma screening markers by non-targeted analysis of small molecule compounds in plasma.Results: Rapid restriction decreased kidney weight. Rapid restriction decreased urine output and increased urine osmolality. Circulating blood aldosterone concentration and renal expression of ion channel SGK-1 increased, suggesting that RAAS was stimulated. Furthermore, rapid restriction was shown to induce renal expression levels of inflammatory cytokines.Conclusions: Rapid restriction may have different functional significance between VPS and RAAS. Furthermore, the combination of rapid restriction and regular exercise may have detrimental effects on the kidneys.

### [1P-194]

**Local muscle contraction of atrophied muscle by denervation reduces inguinal white adipose tissue weight**

*Riku Tanimura[1], Takanaga Shirai[2], Kazuki Uemichi[1], Tomohiro Iwata[1], Ryoto Iwai[1], Shunsuke Sugiyama[1], Tohru Takemasa[2] ([1]*Graduate School of Comprehensive Human Sciences, University of Tsukuba, Tsukuba, Japan.,* [2]*Faculty of Health and Sports Sciences, University of Tsukuba, Tsukuba, Japan.*)

Introduction Skeletal muscle is known to have drastic plasticity and endocrine function (Baldwin and Haddad, 2002). We found that acute muscle contraction of atrophied muscle by denervation promotes myokine, IL-6 and FGF21, secretion like that of healthy skeletal muscle (unpublished data). These myokines act not only on muscle, but also on adipose tissue and other tissues. Thus, we aimed to investigate that effects of chronic denervated muscle contraction on skeletal muscle and adipose tissue phenotype and metabolism-related protein expression. Methods Male ICR mice aged 7 weeks were used in this study. After a week of acclimation, mice were underwent denervated surgery (denervation: DEN) or performed sham operation (Sham). Two weeks after surgery, the gastrocnemius muscles were stimulated percutaneously with electrode (ES) or let them be sedentary (CON) for 4weeks. The mice were divided 4 groups: Sham+CON group ($n$ = 6), Sham+ES group ($n$ = 5), DEN+CON group ($n$ = 6), DEN+ES group ($n$ = 6). Results and Discussion The gastrocnemius wet weight lowered significantly in DEN and DEN+ES groups, and the protein synthesis is significantly higher in ES and DEN+ES groups. Thus, we observed collateral evidence of ES and DEN interventions. The inguinal white adipose tissue (iWAT) wet weight lowered significantly in ES and ES+DEN group. Moreover, DEN induced reduction of mitochondria related proteins in iWAT, which were rescued by 4-weeks ES. Based on our previous data, several myokines secreted by muscle contractions could act adipose tissue metabolic remodeling.

### [1P-195]

### Possible involvement of methylglyoxal as a new exercise-resistance factor

*Tatsuro Egawa[1], Takeshi Ogawa[1], Kohei Kido[2], Takumi Yokokawa[3], Katsumasa Goto[4], Tatsuya Hayashi[1] ([1]*Graduate School of Human and Environmental Studies, Kyoto University,* [2]*Faculty of Sports and Health Science, Fukuoka University,* [3]*Graduate School of Agriculture, Kyoto University,* [4]*Graduate School of Health Sciences, Toyohashi SOZO University*)

Exercise causes skeletal muscle adaptations, including increased insulin signaling, glucose metabolism, and mitochondrial biogenesis. However, exercise-induced skeletal muscle adaptations may not occur in some cases, a condition known as exercise-resistance. This study aimed to clarify the effect of methylglyoxal (MG), a highly reactive dicarbonyl metabolite, on skeletal muscle adaptations following endurance exercise. Mice were randomly divided into four groups: sedentary control group, voluntary exercise group (Ex), MG-treated group (MG), and MG-treated with voluntary exercise group (MG+Ex). Mice in the Ex group were housed in a cage with a running wheel for 4 weeks, whereas mice in the MG group received drinking water containing 1% MG. In Ex group, several molecular adaptations occurred in the plantaris muscle, including increased expression of peroxisome proliferator-activated receptor gamma coactivator 1α, mitochondria complex proteins and enhanced insulin-stimulated Akt $Ser^{473}$ phosphorylation and citrate synthase activity. However, these adaptations were suppressed in MG+EX group. These findings suggest that MG is a factor that prevents exercise-induced molecular adaptations including mitochondrial biogenesis and insulin signaling activation in skeletal muscle.

### [1P-196]

### The ASIC3 inhibitor APETx2 attenuates exaggerated muscle mechanoreflex in rats under repeated cold stress

*Amane Hori[1,2], Teruaki Nasu[3], Ryuji Saito[3], Kimiaki Katanosaka[3], Kazue Mizumura[4], Masaki Mizuno[5], Norio Hotta[3] ([1]*Graduate School of Life and Health Sciences, Chubu University,* [2]*Japan Society for the Promotion of Science,* [3]*College of Life and Health Sciences, Chubu University,* [4]*Department of Physiology, Nihon University School of Dentistry,* [5]*Department of Applied Clinical Research, University of Texas Southwestern Medical Center*)

Repeated cold stress (RCS), a model for widespread pain, has been reported to induce muscle acidification and potentiate the response to mechanical stimulation in thin-fiber muscle afferents. We hypothesized that RCS exaggerates the muscle mechanoreflex by sensitizing acid-sensing ion channel 3 (ASIC3). In this study, Sprague-Dawley rats were divided into RCS and the control groups. The RCS group was alternately exposed to room temperature (22 °C) and cold stress (4 °C) for 5 days. All recordings were performed on unanesthetized decerebrated rats. Responses of mean arterial pressure (MAP), heart rate (HR), and renal sympathetic nerve activity (RSNA) to passive muscle stretch (mechanoreflex) were significantly ($P<0.05$) higher in the RCS group than in the control group. Additionally, RCS significantly ($P<0.05$) increased the responses of MAP, HR, and RSNA to injection of lactic acid (24 mM), an ASIC3 agonist. Notably, injection of APETx2, an ASIC3 inhibitor, significantly ($P<0.05$) attenuated these responses to muscle stretch in the RCS group. These results suggest that ASIC3 contributes to the RCS-induced exaggeration of the muscle mechanoreflex.

### [1P-197]

### Vocalization during the post-exercise recovery period affects muscle oxygen status

*Hajime Arikawa[1], Tomoyoshi Terada[2], Kanako Yamada[2], Teppei Takahashi[3] ([1]*Chubu Gakuin Univ.,* [2]*Gifu Univ.,* [3]*Takahashi Dental Clinic*)

[Aims] In sports, briefly meeting with teammates immediately after a play is common. In this situation, the players must vocalize to communicate, despite being out of breath immediately after exercise. The effects of this forced change in breathing patterns on ventilatory and hemodynamic dynamics have not been investigated. Therefore, this study investigated the effects of active vocalization during the post-exercise recovery period on ventilation, hemodynamics, and muscle oxygenation. [Methods] Eight male university students underwent a 10-min rest and recovery period, with or without vocalization, after a 3-min sustained lower-body exercise (80%$VO_2$peak load). We measured ventilation (respiratory rate [fB], minute ventilation [VE], fraction of end-tidal $CO_2$ [$FetCO_2$]), circulatory indices (HR, $SpO_2$), and right vastus lateralis muscle oxygen status (TSI%) during the exercise. Oxidative stress (d-ROMs test), blood lactate concentration ([$Lac^-$]), and rate of perceived exertion (RPE) were assessed before exercise, immediately after exercise, and at the end of the recovery period. [Results] We observed decreased fB and VE values due to vocalization, whereas $FetCO_2$ (≈blood $CO_2$ concentration [$PaCO_2$]) values were elevated throughout the recovery period. However, the recovery of TSI% tended to be suppressed, suggesting that the blood supply to active muscles was reduced. This suggested a decreased venous return in participants caused by an increase in intrathoracic pressure associated with vocalization. [Conclusion] Vocalization during the post-exercise recovery period was confirmed to increase $PaCO_2$ due to suppression of ventilation. Additionally, the recovery of the oxygen status in active muscles tended to be suppressed, which suggested that vocalization may also affect blood circulation.

### [1P-198]

### Interactive effects of gender and posture on sympathetic neural and vasodilator responses before beginning exercise

*Kazumasa Manabe[1,2], Ryosuke Takeda[1,2], Andrew D'Souza[1,2,3], Sarah Hissen[1,2], Takuro Washio[1,2], John Akins[1,2], Qi Fu[1,2] ([1]*Women's Heart Health Laboratory, Institute for Exercise and Environmental Medicine at Texas Health Presbyterian Hospital Dallas,* [2]*The University of Texas Southwestern Medical Center,* [3]*Neurovascular Research Laboratory, School of Kinesiology, Western University*)

[Aim] Countdown before beginning exercise induces muscle vasodilation via reductions in muscle sympathetic nerve activity (MSNA) in young men, which is likely advantageous for supplying oxygen to exercising muscles. This study, therefore, assessed the role of gender and posture in sympathetic neural and vasodilator responses to countdown before exercise.[Method] Young healthy men and women (31 ± 4 and 30 ± 5 [SD] yrs, n=11 each) performed 1-min of static handgrip at 30% of maximal contraction force twice while supine and twice during 30° head-up tilt (HUT). For each posture, participants were either given a 30 s countdown (CD+) or immediately signaled to begin exercise (CD-), with the order randomized and counterbalanced. MSNA (microneurography) and superficial femoral artery diameter (Doppler ultrasound) were measured continuously. [Results] CD+ decreased MSNA burst frequency (BF) and total activity (TA) compared to CD- in both postures (both $p<0.05$). No gender differences were observed in these responses while supine (both $p>0.50$), though during HUT CD+ reduced MSNA more in men than women (BF: $p=0.023$ for interaction; TA $p=0.014$ for gender). Vasodilation was also larger in men than women during HUT ($p=0.045$), but not while supine ($p=0.24$).[Conclusion] Countdown before exercise elicits larger vasodilation via greater reductions in MSNA in young men versus women during head-up tilt but not while the supine.

### Poster Presentation

### [1P]
### Nutritional and metabolic physiology, Thermoregulation

March 14 (Tue.), 12:10 - 14:10, Poster Room

### [1P-199]

**Effect of progesterone on thermoregulatory responses in ovariectomized rats administrated TREK agonist**

*Yuki Uchida[1], Yuki Samejima[1,2], Izumizaki Masahiko[1] ([1]*Department of Physiology, Showa University School of Medicine,* [2]*Department of Orthopaedic Surgery, Showa University Fujigaoka Hospital*)

INTRODUCTION The TWIK-related $K^+$ (TREK) channels have been reported to be new cold receptors. The effect of progesterone (P), one of the female hormones, on thermoregulatory responses through TREK has not been elucidated. METHODS Ovariectomized rats were implanted with nano tag® for the measurement of body temperature ($T_b$) in the peritoneal cavity. Before the experiment day, ovariectomized rats were implanted with two silastic tubes with P or sesame oil underneath the dorsal skin (P(+) and P(−) groups, n=32) as documented (Williams et al., 2010, Endocrinology). After intraperitoneal administration of the TREK agonist (Ostruthin Imperatorin, 4.2μg) or vehicle at 10:00, rats were exposed to 27°C for 2 hours with continuous $T_b$ and activity measurements. The changes in $T_b$ and activity from the baselines (the mean for the 30 min prior to administration) were calculated. RESULTS In the P(+) and P(−) groups, the TREK agonist increased the change in activity, but, did not affect the change in $T_{b.}$ CONCLUSION Progesterone may not affect $T_b$ and activity through TREK in ovariectomized rats. We plan to analyze the tail skin temperature, thermoregulatory behavior, blood, and brain areas.

### [1P-200]

**Fatty acids are taken up from both basolateral and apical side in the kidney: Unappreciated mechanisms of fatty acid uptake by the kidney**

*Tatsuya Iso[1], Ryo Kawakami[2], Hirofumi Hanaoka[2], Ayaka Kanai[2], Hideru Obinata[2], Daisuke Nakano[3], Hidekazu Ikeuchi[2], Keiju Hiromura[2], Akira Nishiyama[3], Motoko Yanagita[4], Hideki Ishii[2], Masahiko Kurabayashi[2] ([1]*Gunma University of Health and Welfare,* [2]*Gunma University Graduate School of Medicine,* [3]*Kagawa University Faculty of Medicine,* [4]*Kyoto University Graduate School of Medicine*)

Background: Although the kidney combusts a considerable amount of fatty acid (FA), little is known about the mechanisms of FA uptake. We aimed to determine how FAs are taken up by tubular epithelial cells. Methods and Results: CD36, known as an important FA transporter in the heart, was also expressed in the basolateral side of proximal tubule epithelial cells (PTECs). The uptake of $^{125}$I-BMIPP, radio-labeled FA tracer, was significantly reduced in CD36 knock-out mice 1min after injection. In-vivo imaging with two-photon microscopy revealed that BODIPY-$C_{12}$, fluorescence-labeled FA tracer, was accumulated in the basolateral side (blood side) of PTECs early after injection followed by the apical-side (primary urine side) accumulation. A large amount of neutral lipid was accumulated in the kidney when serum FA concentration was increased by accelerated lipolysis. Immunohistochemistry with cell-specific antibodies allows us to identify PTECs as primary lipid-accumulating cells. Importantly, urinary FA was not detected at all even in the case with remarkable albuminuria in mice and human, suggesting complete FA reabsorption from primary urine independently of albumin. Conclusions: Tubular epithelial cells take up FA from both blood (CD36-dependent) and primary urine (CD36-independent) and store excess amount of FA as neutral lipids. Further, it is noteworthy there is a robust system to completely reabsorb FA from primary urine even in diseased kidneys.

### [1P-201]

**17-hour flavor preference test in vitamin C deficient rats**

*Toshiaki Yasuo[1], Fumihiko Nakamura[1], Takeshi Suwabe[1], Shinpei Takahashi[1], Noritaka Sako[1] ([1]*Asahi university*)

How animals regulate the ingestion of deficient vitamin C (VC) is still unknown. Our previous behavioral studies have shown that Osteogenic Disorder Shionogi (ODS) rats, which lack the ability to synthesize VC, show an increased preference for VC solution in VC deficient condition compared to replete condition. However, it is unclear whether ODS rats can learn conditioned flavor preferences and whether such feeding experiences affect their ingestive behavior during vitamin C deficiency. In the present study, we conducted a 17-hour two-bottle preference test between the two flavors. In experiment 1, the rats in replete condition were presented with unsweetened cherry-flavored water mixed with 10mM VC and unsweetened grape-flavored water for 8 days and then fed VC-deficient diet for 25 days, followed by the test. In Experiment 2, rats were fed a VC-deficient diet for 25 days, then presented with unsweetened grape-flavored water for a day, and then presented with unsweetened cherry-flavored water mixed with 10 mM VC for a day, followed by the same test. As a result, the average percentage of intake of cherry flavored water (cherry flavored water intake / total intake ×100) was approximately 50% in Experiment 1 and 60% in Experiment 2. These results suggest that ODS rats under the replete condition might not acquire long-term flavor preference learning with VC solution as the unconditioned stimulus.

### [1P-202]

**Role of hypothalamic prostaglandins to monitor blood glucose levels**

*Chitoku Toda[1] ([1]*Hokkaido University*)

The hypothalamus plays a central role in monitoring and regulating systemic glucose metabolism. The brain is enriched with phospholipids containing poly-unsaturated fatty acids, which are biologically active in physiological regulation. Here, we show that intraperitoneal glucose injection induced changes in hypothalamic distribution and amounts of phospholipids, especially arachidonic-acid-containing phospholipids, that were then metabolized to produce prostaglandins. Knockdown of cytosolic phospholipase A2 (cPLA2), a key enzyme for generating arachidonic acid from phospholipids, in the hypothalamic ventromedial nucleus (VMH), lowered insulin sensitivity in muscles during regular chow diet (RCD) feeding. Conversely, the down-regulation of glucose metabolism by high fat diet (HFD) feeding was improved by knockdown of cPLA2 in the VMH through changing hepatic insulin sensitivity and hypothalamic inflammation. Our data suggest that cPLA2-mediated hypothalamic phospholipid metabolism is critical for controlling systemic glucose metabolism during RCD, while continuous activation of the same pathway to produce prostaglandins during HFD deteriorates glucose metabolism.

### [1P-203]

**A temperature-dependent diabetes-like metabolic state regulated by QIH**

*Ming-Liang LEE[1], Ching Pu CHANG[1], Tomomi NEMOTO[1], Ryosuke ENOKI[1] ([1]*NINS, ExCELLS/NINS, NIPS*)

Torpor and hibernation are states of low body temperature and low energy metabolism for animals to survive in harsh environments such as food shortages in cold seasons. Activation of Qrfp neurons in the preoptic area of hypothalamus induces hibernation-like hypothermia and hypometabolism (QIH) in non-hibernation animals such as mice (Takahashi et al., Nature, 2020). QIH mice show inappetence accompanied by very low locomotor activities. During QIH, systemic oxygen consumption and energy loss are remarkably reduced. However, it remains unclear how the metabolism of glucose, the main energy resource, is regulated during QIH. In this study, we utilized DREADD to chemogenetically activate Qrfp neurons of mice to trigger QIH and found that the QIH mice were insulin resistant with systemic glucose hypometabolism. These mice showed hyperinsulinemia and higher blood glucose after overnight fasting, suggesting that the QIH animals are in a diabetes-like metabolic state. Surprisingly, the glucose metabolism was fully recovered in an elevated ambient temperature (33°C), in which the body temperature of QIH mice was comparable with normal mice. QIH-induced suppression of appetite and locomotor activities were also recovered under the elevated ambient temperature. We concluded that activation of Qrfp neurons induced insulin resistance and glucose hypometabolism, which can be recovered by increased body temperature. These results indicate that the Qrfp neurons do not directly control glucose metabolism during QIH, instead, hypothermia alters glucose metabolism, appetite, and locomotor activity.

### [1P-204]

### Physiological function of NPGL/NPGM system in energy metabolism

*Kenshiro Shikano[1], Morisaki Ikuko[2], Higa Ryoko[1], Teranishi Hitoshi[1], Yahiro Takaki[3], Yoshimura Mitsuhiro[4], Hanada Toshikatsu[2], Nakamura Kazuhiro[3], Ueta Yoichi[4], Hanada Reiko[1] ([1]*Department of Neurophysiolgy, Faculty of Medicine, Oita University, Japan,* [2]*Department of Cell Biology, Faculty of Medicine, Oita University, Japan,* [3]*Department of Integrative Physiology, Nagoya University Graduate School of Medicine, Japan,* [4]*Department of Physiology, School of Medicine, University of Occupational and Environmental Health, Japan*)

Hypothalamus is the center of energy metabolism and feeding behavior, and several hypothalamic neurotransmitters regulate energy homeostasis. Neurosecretory protein GL (NPGL) and neurosecretory protein GM (NPGM), were discovered as a novel neuropeptide from hypothalamus in 2014, induced obese phenotype owing to increased feeding behavior and lipogenesis. However, the mechanisms of energy metabolism mediated by NPGL/NPGM system has not been totally elucidated. To investigate the loss of function of NPGL/NPGM system, we have established NPGL and NPGM double knockout mice (NPGL/NPGM dKO). NPGL/NPGM dKO showed a lean phenotype under a high fat diet condition because of decreased food intake and increased energy expenditure compared with wild type mice (WT). Furthermore, NPGL/NPGM dKO showed not only decreasing fat deposition but also augmenting thermogenic uncoupling protein 1 expression in brown adipose tissue. Finally, we analyzed expression levels of feeding regulators in hypothalamus. The expression levels of anorexigenic factors were upregulated in NPGL/NPGM dKO. These results imply that endogenous NPGL/NPGM system has important roles in central regulation of energy metabolism.

### [1P-205]

### Exploration of thermosensory neural pathways that drive thermoregulatory behavior

*Takaki Yahiro[1], Naoya Kataoka[1,2], Kazuhiro Nakamura[1] ([1]*Department of Integrative Physiology, Nagoya University Graduate School of Medicine,* [2]*Nagoya University Institute for Advanced Research*)

Behavioral thermoregulation is a thermoregulatory mechanism based on behaviors, such as avoidance from cold and hot temperature, but its central circuit mechanism is largely unknown. We previously found that the lateral parabrachial nucleus (LPB) is required for thermoregulatory behavior. In this study, we explored the neural pathways from the rat LPB that mediate themosensory signaling for thermoregulatory behavior. First, we found that LPB→central amygdala (CeA) projection neurons were activated by cold exposure. The LPB also contains neurons sending cold and warm sensory signals to the thermoregulatory center, preoptic area (POA). We investigated whether LPB→POA and LPB→CeA neurons are involved in behavioral thermoregulation. Adeno-associated viruses were used to selectively express tetanus toxin light chains in LPB→POA or LPB→CeA neurons to suppress their transmission. Behavioral analyses showed that suppressing LPB→POA neurons attenuated heat avoidance. On the other hand, suppressing LPB→CeA neurons attenuated both cold and heat avoidance. Currently, we are further investigating the roles of these two pathways in behavioral themroregulation by using chemogenetic and optogenetic techniques to suppress neurotransmission at axon endings.

### [1P-206]

### Identification of uncoupling protein 1 (UCP1) in the hypothalamus of Syrian hamster brain

*CHING PU CHANG[1,2], Yoshifumi YAMAGUCHI[3], Masatomo WATANABE[4], Tomomi NEMOTO[1,2], Ryosuke ENOKI[1,2] ([1]*National Institute for Physiological sciences,* [2]*Exploratory Research Center on Life and Living Systems,* [3]*Hokkaido University, ILTS,* [4]*Fukuyama University, Faculty of Pharmacy and Pharmaceutical Sciences*)

Thermogenesis is essential for survival in endotherms, which maintain their body temperature when exposed to a cold environment. Body temperature is mainly controlled by shivering and non-shivering thermogenesis in muscle and brown adipose tissue (BAT), respectively. The unique property of BAT is the presence of abundant mitochondria and high expression of uncoupling protein 1 (UCP1), which uncouples the proton gradient and generates heat. It has been long believed that UCP1 is restricted to BAT, however, it is yet uncertain whether UCP1 is also presented in the central nerve system. In this study, we used multiple immunohistochemical techniques and quantitative analysis to identify the regional and cell type specificity of UCP1 in the brain of the Syrian hamster, a facultative hibernator. We found that UCP1 was profoundly expressed in neurons in the paraventricular nucleus of the hypothalamus (PVN) and in astrocytes near the third ventricle, which is innervated by tanycytes. These results imply that hypothalamic UCP1 plays an unexpected role in local thermoregulation in the hamster brain during hibernation.

### [1P-207]

### Regulation of glucoprivation-induced carbohydrate selection by NPY-CRH neural axis in the paraventricular nucleus of the hypothalamus

*Nawarat Rattanajearakul[1], Ken-ichiro Nakajima[1], Yasuhiko Minokoshi[1] ([1]*Division of Endocrinology and Metabolism, National Institute for Physiological Sciences (NIPS)*)

We previously reported the essential role of a subset of corticotropin-releasing hormone (CRH) neurons expressing fasting-responsive AMP-activated protein kinase (AMPK) in the paraventricular nucleus of the hypothalamus (PVH) in a high carbohydrate diet (HCD) selection over a high fat diet (HFD) during refeeding after fasting. Here, we investigated the role of neuropeptide Y (NPY)-CRH neural axis in the PVH in 2-deoxyglucose (2DG)-induced change in food selection in mice. Two-diet choice experiment revealed that intraperitoneal (IP) injection of 2DG increased HCD intake while it suppressed HFD intake. Intracerebroventricular (ICV) or intra-PVH NPY injection increased HCD intake along with a slow increase in HFD intake. Intra-PVH injection of antagonists for Y1R and Y5R blocked 2DG-induced HCD intake preferentially. Chemogenetic inhibition or a specific knockdown of AMPK in PVH CRH neurons suppressed both 2DG and NPY-induced HCD intake. By contrast, PVH injection of melanocortin-4 receptor (MC4R) agonist, Melanotan II (MTII), inhibited NPY-induced HFD intake but not HCD intake. 2DG activated PVH-projecting NPY neurons in several brain areas including the nucleus tractus solitarius (NTS). Optogenetic activation of NTS NPY neurons projecting to the PVH increased c-fos expression in the PVH and increased HCD intake along with a slow increase in HFD intake. Collectively, these suggest that NPY-CRH neural axis in the PVH is necessary for 2DG-induced HCD selection and HFD intake is mediated by a distinct neural mechanism.

### [1P-208]

### Metformin down-regulates IL-11 expression to inhibit myocardial fibrosis

*Mengting Shan[1,2] ([1]*Department of Neurophysiology, Faculty of Medicine, Oita University, Japan,* [2]*Department of Internal Medicine, Zhengzhou University, China*)

Objective: To explore whether IL-11 is the target of Metformin (Met) inhibiting TGF-β1 pathway to delay myocardial fibrosis. Methods: 40 SD rats were averaged into 4 groups; Control group (C) with normal diet, (2) Diabetic group (DM) with high fat and high sugar diet, (3) DM+Met group, (4) DM+Insulin (Ins) group. Rats were sacrificed and myocardial tissue was extracted at 16 weeks. The mRNA expressions of TGF-β1, IL-11, alpha smooth muscle actin (α-SMA), extracellular matrix (ECM) were detected, and the proliferation of collagen fibers in myocardial tissue was surveyed by HE staining. Results: The expression levels of TGF-β1, IL-11, ECM and α-SMA were increased in DM group, compared with C group. In Met treatment, fluorescence staining level of pro IL-11 significantly increased, but there was no difference in the expression of TGF-β1 and IL-11mRNA, and the fluorescence staining of pro IL-11 was lower than DM group. Under the light microscope, the proliferation of myocardial collagen fibers was obviously reduced in Met treatment. Conclusion In myocardial tissue, Met can reduce the expression of IL-11 and inhibit the progression of fibrosis.

### [1P-209]

### PVH TH neurons regulate feeding behaviour and reward system

*Winda Ariyani[1], Haruka Tsuneoka[1], Chiharu Yoshikawa[1], Izuki Amano[2], Noriyuki Koibuchi[2], Hiroshi Ichinose[3], Tadahiro Kitamura[1], Daisuke Kohno[1] ([1]*Gunma University, Institute for Molecular and Cellular Regulation, Metabolic Signal Research Center,* [2]*Department of Integrative Physiology, Gunma University Graduate School of Medicine.,* [3]*School of Life Science and Technology, Tokyo Institute of Technology.*)

The paraventricular hypothalamus (PVH) plays a vital role in feeding regulation. Our previous study showed that the deletion of DNA methyltransferase 3a (DNMT3a) in the PVH highly increased the tyrosine hydroxylase (TH) expression level in the PVH and induced obesity. Therefore, we explored the role of TH neurons in the PVH. Histological analysis showed that PVH TH neurons were dopaminergic neurons, colocalized with GABA and received the projections from NPY/AgRP and POMC neurons. In addition, c-Fos-immunopositive cells, calcium intensity, *MC4R*, *Pdyn*, and presynaptic puncta were increased in TH neurons during refeeding. The obesity phenotype in *Dnmt3a*$^{lox/lox}$/*Sim1*-Cre mice disappeared in *Dnmt3a*$^{lox/lox}$/*Th*$^{lox/lox}$/*Sim1*-Cre mice. Then, we examined the role of TH neurons in feeding behavior using *Th*$^{lox/lox}$/*Sim1*-Cre mice. The second phase of food intake was reduced, and the sensory cue-initiated food reward task was improved in *Th*$^{lox/lox}$/*Sim1*-Cre mice. Furthermore, DREAAD activation or suppression of PVH TH neurons affected food intake and the success rate of the task. The anterograde AAV tracer showed that PVH TH neurons projected to brain regions outside of the hypothalamus. These results indicate that PVH TH neurons are dopaminergic neurons that are regulated by DNMT3A and activated by the melanocortin pathway, which may play a role in further craving food after meal initiation. COI:No

### [1P-210]

**Effects of dorsomedial hypothalamus-specific *Prdm13* deficiency in body metabolism under high fat diet**

*Shiho Maruyama[1,2], Mai Kiyozuka[1,2], Mio Goto[2], Keiko Kabetani[2], Shogo Tsuji[2], Hirobumi Tada[1,2], Akiko Satoh[2,3] (*[1]Faculty of Wellness, Shigakkan University, [2]Geroscience Research Center, National Center for Geriatrics and Gerontology, [3]Institute of Development, Aging and Cancer, Tohoku University*)

### [1P-211]

**Seasonal effects of fat formation and thermogenesis in Mangalica.**

*Tatsuki Okazaki[1], Sangwoo Kim[1], Chisato Nakayama[1], Erina Yoneda[1], Kisaki Tomita[1], Yuki Muranishi[1] (*[1]Obihiro University of Agriculture and Veterinary Medicine*)

### [1P-212]

**The expression of Piezo1 channel is increased in adipose tissues from mice exposed to a cold environment**

*Satsuki Takizawa[1], Kazuhiko Okamura[2], Manato Kenmochi[3], Satoko Kawarasaki[4], Tetsuya Hosooka[1,3], Tsuyoshi Goto[4], Kunitoshi Uchida[1,3] (*[1]University of Shizuoka, [2]Fukuoka Dental College, [3]Graduate School of Integrated Pharmaceutical and Nutritional Sciences, University of Shizuoka, [4]Graduate School of Agriculture, Kyoto University*)

### Poster Presentation

[1P]
**Behavior, Biological rhythm, Sleep**

March 14 (Tue.), 12:10 - 14:10, Poster Room

### [1P-213]

### Correlation of sleep quality with menopausal symptoms in Indian females

*Bharati Mehta[1], Shival Srivastav[1], Mahesh Arjundan Gadhvi[1] ([1]*All India Institute of Medical Sciences, Jodhpur*)

Introduction: With a gradual decline of ovarian hormones during menopause, several symptoms, such as hot flashes, sweating, mood swings, and sleep disturbances arise. There is a need to explore and address sleep disturbances because the sleep dysfunction may intensify and lead to the onset of diseases like anxiety, depression or cardiovascular diseases.Methods: This cross-sectional study was performed on postmenopausal women aged 48 to 60 years. Their menopausal symptoms were evaluated by the Menopause Rating Scale (MRS) and sleep quality by the Pittsburgh Sleep Quality Index (PSQI). Participants with PSQI scores of 5 or less were considered good sleepers and scores greater than 5 were considered poor sleepers. Results: Data is represented as median values with 1$^{st}$ and 3$^{rd}$ quartiles. The age of the participants was 56[55-57] years and their age at natural menopause was 50[48-51.75] years. Global PSQI score was 5[4-8]; 51.12% were good sleepers, while 48.89% poor sleepers. Total MRS score was 9.5[4.25-15.25]. Menopausal symptoms were found in 75.6% of participants. Pearson correlation revealed that the total MRS score was associated with poor sleep quality ($p<0.0003$). Amongst the components of PSQI, subjective sleep quality (p=0.001), sleep disturbances (0.02), daytime sleepiness, and disturbances during the day (p=0.008) showed a significant correlation with the total MRS score.

### [1P-214]

### Characteristics of sleep-wakefulness cycle and circadian activity in senescence-accelerated mice

*Akira Terao[1], Mao Sato[1], Haochen Ma[1], Nobuaki Okumura[2] ([1]*Department of Biology, School of Biological Sciences, Tokai University,* [2]*Institute for Bee Products and Health Science, Yamada Bee Company Inc.*)

The circadian rhythm and sleep architecture of senescence-accelerated mice (SAM)P8 and SAMR1 were evaluated to utilize SAM as a model of age-related sleep disorders. The evaluation of the circadian rhythm based on the locomotor activity was performed from 1 to 5 months old SAMP8 and SAMR1. At 6 months of age, mice were chronically implanted with electroencephalograph and electromyogram electrodes for polysomnographic recording of sleep-wake states. The vigilance states were automatically classified by SleepSign ver.3 software. First, the circadian rhythm was evaluated. Comparing the locomotor activity ratio of 12 h dark period vs.12 h light period, SAMR1 showed its ratio between 3.7 to 5.4, while that of SAMP8 showed between 1.6 to 2.5. Therefore, it was shown that SAMP8 has a smaller circadian rhythm amplitude than SAMR1. Next, the sleep architecture was evaluated. The non-rapid eye movement (REM) sleep amount and the REM sleep amount of SAMP8 mice showed significantly lower than those of SAMR1. The duration of non-REM sleep in SAMP8 mice was significantly shorter than that of SAMR1, suggesting that sleep quality deteriorated with sleep fragmentation. Therefore, it was shown that SAMP8 is effective as a model of age-related sleep disorders and can be used for exploratory research on food ingredients that improve sleep.

### [1P-215]

### Identification of brainstem neurons involved in sleep regulation in mice

*Mitsuaki Kashiwagi[1,2], Kanuka Mika[2], Tatsuzawa Chika[2], Tanaka Kaeko[2], Hayashi Yu[1,2,3] ([1]*The University of Tokyo,* [2]*University of Tsukuba,* [3]*Kyoto University*)

Mammalian sleep comprises two distinct states: REM (rapid eye movement) sleep and NREM (non-REM) sleep. Pharmacological and lesion studies implicated that the brainstem area plays an important role in REM/NREM sleep regulation. However, cell types and molecular identities of neural circuits responsible remain largely unknown. To identify sleep-regulating neurons, we searched for possible genetic markers and analyzed the effect of genetic manipulation of candidate neurons on sleep in mice. As a result, we found that neurotensin-positive neurons in the dorsal pons promote NREM sleep. We also identified putative downstream NREM sleep-promoting neurons in the other brainstem areas that were also neurotensinergic. Infusion of neurotensin peptide and knock-out mice analysis implicated the involvement of the neurotensin peptide itself. These findings identify a widely distributed NREM sleep-regulating circuit in the brainstem with a common molecular property (Kashiwagi, et al., Current Biology, 2020: 30(6) 1002-1010.e4). Recently, we also succeeded in identifying a neural circuit that promotes REM sleep (Kashiwagi, et al., unpublished). Our findings precisely address the cell types and molecular identities of neural circuits regulating REM/NREM sleep, respectively.

### [1P-216]

### Recalling of positive memory increases cataplexy-like behaviors in narcolepsy mice

*Mayuko Yoshida[1], Tomoyuki Kuwaki[1] ([1]*Kagoshima Univ.*)

Background: Cataplexy is loss of muscle strength and postural collapse threatening the daily life of narcolepsy patients; it is triggered by positive emotions such as laughter in humans andchocolate in mice. It also sometimes takes place without any apparent triggering stimulus. We hypothesized that spontaneous cataplexy in narcoleptic mice might indicate arecalling of happy moments.Results: To test our hypothesis, we did a conditioned place preference test on orexin/hypocretin neuron-ablated (ORX-AB) mice, one of the animal models of human narcolepsy. ORX-AB mice successfully remembered the chocolate-associated chamber, and the number of cataplexy-like behaviors significantly increased in the chocolate-associated chamber but not in the control chamber. ORX-AB mice remembered the aversive odor-associated chamber and avoided entering without affecting the number of cataplexy-like behaviors. Finally, similar activation of the nucleus accumbens, a positive emotion-related nucleus, was observed during both spontaneous and chocolate-induced cataplexy behaviors. Conclusions: These results support our hypothesis and serve as a basis for better understanding of cataplexy in narcolepsy patients.

### [1P-217]

### Motivational increase for sucrose reward and brain network variation in Importin α3 (KPNA3) deficient mice

*Yoshiatsu Aomine[1,2], Koki Sakurai[1], Tom Macpherson[1,2], Takaaki Ozawa[1,2], Yoichi Miyamoto[3], Yoshihiro Yoneda[4], Masahiro Oka[3], Takatoshi Hikida[1,2] ([1]*Osaka Univ, Inst. for Protein Research,* [2]*Osaka Univ, Dept. Biol., Grad. Sch. Sci.,* [3]*National Inst.of Biomedical Innovation,* [4]*The Res. Found. for Microbial Diseases of Osaka Univ.*)

Importin α3 (*Kpna3*) is a member of the importin α family and participates in nucleocytoplasmic and cytoplasmic transport to nucleus. Evidence from human studies has indicated that single nucleotide polymorphisms in the *KPNA3* gene are associated with the occurrence of several psychiatric disorders accompanied by abnormal reward-related behavior, including schizophrenia, major depression, and substance addiction. However, the precise roles of KPNA3 in controlling reward processing and motivation are still unclear. In this study, we evaluated the behavioral effects of *Kpna3* knockout (KO) in mice on progressive ratio schedule test, in which the number of operant responses to obtain a reward increases with each reward collected. In this schedule, the break point, defined as the number of responses needed to receive the last reward collected, allows assessment of how much effort mice are willing to expend for a single reward. KO mice showed a significantly increased motivation to instrumentally respond for sucrose. We additionally measured the number of c-Fos positive cells, a marker of neural activity, in 20 regions of the brain and identified a network of brain regions based on their interregional correlation coefficients (functional connectivity). Network and graph-theoretic analyses suggested that *Kpna3* deficiency enhanced overall interregional functional connectivity and altered hub regions, which play a central role in the control of motivation. These findings suggest the importance of *Kpna3* in motivational control.

### [1P-218]

### Differences of effects of excess betamethasone administrations at newborn or after-growth mice on motor and learning abilities assessed by suspension test and step-down passive avoidance test

*Takayoshi Hosono[1] ([1]*Osaka Electro-Communication University*)

Problems. Effects of betamethasone (Bet) administrations to human preterm infants have been widely performed in clinical medicine. However, effects of Bet have not been fully understood. We examined Bet effects after growth using newborn and grown-up mice assessed by suspension test (ST) and step-down passive avoidance test (PAT). Methods. We made two groups of mice consisted of 4-day-old mice (IG) and 7-week-old adult mice (AG). We administrated Bet of 2.5, 0.5, or 0 mg/body weight for consecutive 3 days in each IG and AG. We performed the ST 3 weeks later and PAT 6 weeks later. Results. In the ST, there was no significance among the groups. In IG group the SDT showed the staying times on the insulation in Bet 2.5mg groups were significantly shorter than the other infant groups. These results imply that excess dose administration of Bet causes impairments of behavioral tests, different from the AG group.

### [1P-219]

### Physiological functions of VRK1 in the central nervous system

*Magdeline Carrasco Apolinario[1], Ryohei Umeda[1], Kenshiro Shikano[1], Hitoshi Teranishi[1], Reiko Hanada[1] ([1]*Department of Neurophysiology, Faculty of Medicine, Oita University*)

Vaccinia-related kinase 1 (VRK1) is a predominantly nuclear serine/threonine kinase. VRK1 has been found to have implications in nucleus disassembly and reassembly, cell cycle, and transcription regulation. Mutations in the VRK1 gene have been previously associated with several neurological diseases with phenotypes of microcephaly, human motor neuropathy and pontocerebellar hypoplasia. However, the involvement of the VRK1 gene and its mutations in the nervous system has not yet been fully described. In this study, to characterize the role of VRK1 in vivo, we generated and evaluated a VRK1 gene-deficient zebrafish (VRK1$^{-/-}$) model. In the VRK1$^{-/-}$ model we found growth retardation and microcephaly, reduced brain area, and impaired locomotor activity with anxiety-like behavior. Our VRK1$^{-/-}$ zebrafish is useful to evaluate VRK1's physiological role in the nervous system; also to examine the pathophysiological mechanisms of other conditions associated with neurological involvement and motor activity.

### [1P-220]

### Fish oil intake before exposure to social defeat stress reduces stress-like behavior in mice.

*Airi Otsuka[1], Tamai Nakano[1] ([1]*Kindai Univ*)

Social-defeat stress (SDS) is a well-known rodent model of human's psycho-social stress. Recently, we reported that fish oil reduces social avoidance caused by SDS. In this study, we determined whether fish oil intake before stress exposure affects social behavior in SDS-exposure mice. We used A male C57BL/6J mouse in our experiments. For fish oil treatment, experimental mice were fed a diet containing fish oil at middle (M-FO), and high (H-FO) concentrations for two weeks before SDS exposure. Control group supplemented equivalent canola oil. After eight days SDS protocol, we performed social interaction test and compared social behaviors between FO and control groups. In control group, SDS-exposure mice showed negative social interaction compared to non-stressed mice. However, M-FO and H-FO groups did not exhibit negative social behavior. The serotonin level was not difference by SDS in all groups. In contrast, the expression of genes related to serotonin synthesis of SDS-exposure mice was increased in FO groups, but not in control group. These results suggest that fish oil intake before stress exposure improves psycho-social behavioral disorder caused by SDS. This improvement could be explained by increases in serotonin synthesis in hippocampus.

### [1P-221]

### Oscillatory Population-Level Activity of Dorsal Raphe Serotonergic Neurons Is Inscribed in Sleep Structure

*Tomonobu Kato[1], Yasue Mitsukura[2], Masaru Mimura[1], Norio Takata[3], Kenji Tanaka[3] ([1]*Department of Neuropsychiatry, Keio University School of Medicine,* [2]*Department of System Design Engineering, Faculty of Science and Technology of Keio University,* [3]*Division of Brain Sciences, Institute for Advanced Medical Research, Keio University School of Medicine*)

Dorsal raphe (DR) 5-HT neurons regulate sleep–wake transitions. Previous studies demonstrated that single-unit activity of DR 5-HT neurons is high during wakefulness, decreases during non-rapid eye movement (NREM) sleep, and ceases during rapid eye movement (REM) sleep. However, characteristics of the population-level activity of DR 5-HT neurons, which influence the entire brain, are largely unknown. Here, we measured population activities of 5-HT neurons in the male and female mouse DR across the sleep–wake cycle by ratiometric fiber photometry. We found a slow oscillatory activity of compound intracellular $Ca^{2+}$ signals during NREM sleep. The trough of the concave 5-HT activity increased across sleep progression, but 5-HT activity always returned to that seen during the wake period. When the trough reached a minimum and remained there, REM sleep was initiated. We also found a unique coupling of the oscillatory 5-HT activity and wideband EEG power fluctuation. Furthermore, optogenetic activation of 5-HT neurons during NREM sleep triggered a high EMG power and induced wakefulness, demonstrating a causal role of 5-HT neuron activation. Optogenetic inhibition induced REM sleep or sustained NREM, with an EEG power increase and EEG fluctuation, and pharmacological silencing of 5-HT activity using a selective serotonin reuptake inhibitor led to sustained NREM, with an EEG power decrease and EEG fluctuation. These inhibitory manipulations supported the association between oscillatory 5-HT activity and EEG fluctuation. We propose that NREM sleep is not a monotonous state, but rather it contains dynamic changes that coincide with the oscillatory population-level activity of DR 5-HT neurons.

### [1P-222]

### Heart rate dynamics in infant soothing and promoting sleep utilizing Transport Response

*Nami Ohmura[1], Lana Okuma[1], Anna Truzzi[1,2,3], Kazutaka Shinozuka[1], Atsuko Saito[1,4], Susumu Yokota[5], Andrea Bizzego[2], Eri Miyazawa[1], Masaki Shimizu[6], Gianluca Esposito[1,2], Kumi O. Kuroda[1] ([1]*RIKEN Center for Brain Science,* [2]*University of Trento,* [3]*Trinity College Dublin,* [4]*Sophia University,* [5]*Saitama Prefectural Cardiovascular and Respiratory Center,* [6]*Saitama Prefectural Children's Medical Center*)

Infant cry and sleep problems are the major sources of parental stress. However, there were no conclusive recommendations for on-site behavioral interventions for crying infants. Transport Response is an innate response in mammalian infants that calm down by activating parasympathetic nervous system during carrying. We examined the effects of Transport Response on the infant cry and their heart rate. We found that "Transport", but not holding, is an effective in soothing infants in a 30-second short effect. In 5-minute of carrying, all infants ceased crying and half of them fell asleep. Surprisingly, non-crying infants at the start were not promoted to sleep by five-minute carrying. We also examined the effects of being carried by non-mother persons and by car driving. Subsecond-scale, event-locked analyses of infant heart activity elucidated that the initiation of detachment from the maternal body causes the most alerted in sleeping infants. Furthermore, we found the waiting 5-8 minutes from sleep onset to start laydown reduce infant awakening. These findings regarding infant soothing and sleeping would be useful in practical child rearing.

### [1P-223]

### Postnatal roles of the transcription factor AP-2β in neurons in determining sleep amount

*Ayaka Nakai[1], Mitsuaki Kashiwagi[1,2], Tomoyuki Fujiyama[1], Kanako Iwasaki[1], Hiromasa Funato[1], Masashi Yanagisawa[1], Takeshi Sakurai[1], Yu Hayashi[1,2,3] ([1]*University of Tsukuba,* [2]*University of Tokyo,* [3]*Kyoto University*)

The molecular mechanism of sleep remains largely unclear. Human short sleepers may provide insight into molecular mechanisms of sleep. We focused on the transcription factor AP-2β (*TFAP2B*). Sleep abnormalities such as short sleep and sleep-walking have been self-reported in human families that carry mutations in *TFAP2B*. In addition, the orthologue genes of *TFAP2B* promotes sleep in invertebrate animals. Previously, we provided direct evidence that *TFAP2B* is involved in mammalian sleep. We showed that different mutations in *Tfap2b* have diverse effects on the mouse sleep architecture including the reduction or fragmentation of non-REM sleep (Nakai *et al.*, *Genetics* 2020). Thus, AP-2 transcription factors are crucial for sleep regulation across the animal phyla. However, it is unclear when and where TFAP2B functions. Therefore, we generated mice in which *Tfap2b* is knocked out specifically in the nervous system and mice in which *Tfap2b* can be postnatally knocked out specifically in neurons. Both mice exhibited reduced non-REM sleep amount but there were differences in other sleep parameters. We will discuss the results at the venue. Our study is expected to contribute to understanding the genetic mechanism that defines daily sleep amount.

### [1P-224]

### Effects of long-term living with pet-type robots on psychological stress and oxytocin secretion

*Shuhei Imamura[1], Yoko Gozu[1], Moe Tsutsumi[1], Kaname Hayashi[2], Chiaki Mori[2], Ishikawa Megumi[2], Megumi Takada[2], Tomotaka Ogiso[2], Keiko Suzuki[2], Shota Okabe[3], Takefumi Kikusui[4], Kentaro Kajiya[1] ([1]*MIRAI Technology Institute,Shiseido Co., Ltd.*, [2]*GROOVE X, Inc.*, [3]*Division of Brain and Neurophysiology, Department of Physiology, Jichi Medical University .*, [4]*Laboratory of Human-Animal Interaction and Reciprocity School of Veterinary Medicine, Azabu University.*)

[Purpose] We investigated the effect of interacting with a pet-type robot on stress reduction and the effect of living with one long-term on oxytocin secretion and affiliative behavior towards. [Method] The pet-type robot LOVOT (manufactured by GROOVE X, Inc.) was used for the test . 47 female subjects were assigned to two groups: an owner group who lived with a LOVOT and a non-owner group who did not own one. To examine the basal oxytocin levels, urine was collected upon awakening 3 days before and after the test. During the interaction test, subjects respectively interact with their own LOVOT or the LOVOT prepared for the test. Urinary oxytocin and salivary cortisol levels were measured before and after 15 minutes of interaction with the LOVOT. Behavior during interaction was recorded, and mood states were evaluated using questionnaires. [Results and Discussion] After an interaction, all subjects showed decrease of stress scores and cortisol levels, while oxytocin levels did not change. Basal oxytocin levels were higher in the owner group than the others. The owner group showed more hugging and synchronizing behavior toward their LOVOT than the others. These results suggested that short-term interaction with a LOVOT reduces stress, and indicated that living with a robot facilitates an affiliative relationship between humans and robot via an increasing of basal oxytocin level.

### [1P-225]

### Analyses of a novel mechanism of REM sleep regulation that depends on feeding condition

*Hibiki Okamura[1,2], Shimpei Taniguchi[1,3], Mitsuaki Kashiwagi[1,4], Shinnosuke Yasugaki[1,5], Mika Kanuka[1], Masashi Yanagisawa[1], Yu Hayashi[1,4,6] ([1]*WPI-IIIS, Univ. of Tsukuba*, [2]*Ph. D. Prog in Humanics, SIGMA, Univ. of Tsukuba*, [3]*Master's Prog in Med Sci, Grad Sch of Comprehensive Human Sci, Univ. of Tsukuba*, [4]*Dept of Biol Sci, Grad Sch of Sci, Univ. of Tokyo*, [5]*Doc Prog in Biomed Sci, Grad Sch of Comprehensive Human Sci, Univ. of Tsukuba*, [6]*Dept of Human Health Sci, Grad Sch of Medicine, Kyoto Univ*)

Our sleep comprises two states: rapid eye movement (REM) sleep and non-REM (NREM) sleep. The physiological function of REM sleep is poorly understood. Recent human studies revealed that low amount of REM sleep is associated with a high risk of dementia (Pase et al., 2017) and that emotional distress resolution that naturally occurs during sleep is diminished in people with low-quality REM sleep, i.e. REM sleep that is fragmented by brief awakenings (Wassing et al., 2016). Although the causal relationships are unclear, these studies imply that REM sleep is important for maintenance of our health. However, currently it is difficult to reliably increase REM sleep amount in humans. Here, we found that a certain type of diet can increase REM sleep amount in mice. Switching to a normal diet reversed the REM sleep amount to normal levels. We also found that this feed condition-dependent increase in REM sleep can be enhanced by the perturbation of certain neuronal circuits. Now, we are trying to elucidate the underlying mechanisms. This study contributes to understanding a novel regulatory system of REM sleep. Moreover, it may provide clues for developing methods to increase REM sleep in humans.

### [1P-226]

### Analyses of the anatomical and molecular bases of REM sleep deficits that accompany Parkinson's disease

*Ami Kaneko[1,2], Kanae Iwase[1], Mitsuaki Kashiwagi[1,8], Shinnosuke Yasugaki[1,3], Mika Kanuka[1], Yoshinori Hayakawa[4], Hideki Hayakawa[5], Kensuke Ikenaka[5], Cesar Aguirre[5], Kousuke Baba[5,6], Masashi Yanagisawa[1], Yo Oishi[1], Hideki Mochizuki[5], Haruka Ozaki[4], Yu Hayashi[1,7,8] ([1]*WPI-IIIS, Univ of Tsukuba*, [2]*Ph. D. Prog in Humanics, SIGMA, Univ of Tsukuba*, [3]*Doc Prog in Biomed Sci, Grad Sch of Comprehensive Human Sci, Univ. of Tsukuba*, [4]*Bioinformatics Laboratory, Institute of Medicine, Univ of Tsukuba*, [5]*Dept of Neurology, Grad Sch of Medicine, Osaka Univ.*, [6]*Dept of Neurology, Faculty of Medicine, Acad Res Division, Univ. of Toyama*, [7]*Dept of Human Health Sci, Grad Sch of Medicine, Kyoto Univ*, [8]*Dept of Biol Sci, Grad Sch of Science, Univ of Tokyo*)

Rapid eye movement (REM) sleep behavior disorder (RBD) is characterized by enactment of dreams during REM sleep. RBD can lead to reduced sleep quality and excessive sleepiness or fatigue during the daytime. Importantly, a majority of RBD patients eventually develop synucleinopathies including Parkinson's disease and dementia with Lewy bodies within 10-14 years. Synucleinopathies are neurodegenerative diseases characterized by aggregation of α-synuclein. We aimed to understand the mechanisms underlying the link between RBD and Parkinson's disease. We focused on a G51D α-synuclein mutation which was found in familial Parkinson's disease with rapid progression. We produced and injected G51D α-synuclein fibrils into the mouse sublaterodorsal tegmental nucleus (SLD) within the brainstem pons, an area involved in REM sleep regulation, and examined the effect on muscle tones during REM sleep. Moreover, to understand why REM sleep-regulating neurons in the SLD are vulnerable to α-synuclein and to identify novel therapeutic targets, we conducted transcriptome analyses of neurons in the SLD. To this end, we performed translating ribosomal affinity purification (TRAP), which allows the selective purification of specific subpopulations of neurons, and single nucleus RNA seq. We expect that this study will provide important insight into early treatment for sleep deficits that accompany Parkinson's disease.

### Poster Presentation

[1P]
**Stress**

March 14 (Tue.), 12:10 - 14:10, Poster Room

### [1P-227]

**Withdrawn**

### [1P-228]

**Increased pain-related behaviors and neuronal activation of the spinal dorsal horn in a rat repeated cold stress-induce pain model**

*Toru Taguchi[1,2], Kainuma Riku[1], Ota Hiroki[1,2] ([1]*Dept. Phys. Ther., Fac. Rehabil., Niigata Univ. Health Welfare*, [2]*Inst. Hum. Mov. Med. Sci., Niigata Univ. Health Welfare*)

Persistent psychophysical stress develops chronic pain, although the underpinning mechanisms have not been fully clarified. Here we investigated pain-related behaviors and neuronal activation in the dorsal horn of the spinal cord in response to noxious stimuli, using a repeated cold stress (RCS)-induced pain model. The formalin test was performed by injecting formalin solution into the plantar skin. Neuronal activation evoked by the injection was visualized by immunohistochemical staining of c-Fos-positive cells in the spinal dorsal horn. Duration of pain-related behaviors was significantly prolonged in phase II of the formalin test, but not in phase I, compared to the SHAM rats. The number of c-Fos-positive cells significantly increased in the entire dorsal horn laminae I−VI on the side ipsilateral to formalin injection at the segments L3−L5. The numbers in the contralateral side (laminae I−VI) did not change in the model. These results demonstrate that pain-related behaviors in response to noxious stimulus were intensified in the RCS rats, and increased activation of dorsal horn neurons could be associated with the behavioral hypersensitivity in the RCS model. This work was supported by JSPS KAKENHI (JP22H03458), and partly by the AMED Grant (JP21gm0810010h0606). There are no conflicts of interest related to this study.

### [1P-229]

**Effects of Different Exercise Loads on Oxidative Stress and Skeletal Muscle Growth in Rats**

*Yoichi Ohno[1], Riki Nakano[2], Itsuki Takahashi[3], Tetsuaki Shimokawa[4], Noriyuki Koibuchi[5] ([1]*Department of Physical Therapy, Takasaki University of Health and Welfare*, [2]*Medical Corporation Yamazakikai St-pierre Hospital*, [3]*Department of Food and Nutrition, Takasaki University of Health and Welfare Graduate School of Health and Welfare*, [4]*Department of Nutrition, Takasaki University of Health and Welfare*, [5]*Department of Integrative Physiology, Gunma University Graduate School of Medicine*)

[Introduction] Oxidative stress has been reported to be involved in the onset and progression of various diseases. In this study, we examined the effect of exercise on the changes in oxidative stress and skeletal muscle growth. [Method] Male Wistar rats (n=5, in each group) were divided into low-, and high-intensity exercise groups (LE and HE, respectively), and control group. LE underwent moderate (15 m/min), and HE underwent high-intensity (25 m/min) treadmill exercise for 30 min/day for 4 weeks. After the termination of the training, a wire hanging test was performed to assess the improvement of muscle function. Then, blood sample was collected from the tail vein. Following the sampling, rats were sacrificed, and the soleus muscle was dissected out. The oxidative stress was assessed by measuring active oxygen and free radicals using d-ROMs test, and biological antioxidant power by BAP test in serum. Myogenin and MyoD levels in the soleus muscle were measured using ELISA as a marker of muscle growth. [Results] No significant differences among groups on the levels of oxidative stress and skeletal muscle growth markers were observed. On the other hand, HE group showed lower weight gain and poorer performance in the wire hanging than those of other groups ($p<0.05$). A significant positive correlation was observed between the wire hanging test and weight gain ($p<0.05$). [Conclusion] Overtraining (OT) has been reported to increase reactive oxygen species production, and causes muscle damage, and reduces body weight. However, such tendency was not observed in our animal model, although we found OT declined the muscle performance and weight gain, indicating the involvement of other factors than oxidative stress on such declines. (COI: NO)

### [1P-230]

**FGF21 is involved in social stress-induced alteration of sleep**

*Saori Hokari[1], Sachiko Chikahisa[1,2], Tetsuya Shiuchi[1], Yoshiaki Nakayama[3], Morichika Konishi[3], Seiji Nishino[4], Nobuyuki Itoh[5], Hiroyoshi Séi[1] ([1]*Tokushima University*, [2]*Shikoku University*, [3]*Kobe Pharmaceutical University*, [4]*Stanford University*, [5]*Kyoto University*)

Although the relationship between sleep and the stress response has been suggested in several previous studies, the mechanism connecting these two remains largely unknown. Here, we show that fibroblast growth factor 21 (FGF21), a lipid metabolism-related hormone, may play a role in this relationship. In this study, we examined differences in the stress response between FGF21 knockout (KO) mice and wild-type (WT) mice after social defeat stress (SDS). When averaging the amount of non-rapid eye movement (NREM) sleep, rapid eye movement (REM) sleep and wakefulness over the dark period after SDS, only KO mice showed significant differences in NREM sleep and wakefulness. In the social interaction test, KO mice showed more vulnerability to social avoidance than WT mice. Our real-time (RT) -PCR results revealed that the mRNA expression of the stress- and sleep-related gene, gamma-aminobutyric acid A receptor subunit alpha 2, was significantly lower in WT mice than in KO mice. Moreover, KO mice showed a reduced plasma level of ketone bodies, which also affect sleep/wake, than WT mice. These results suggested that FGF21 might be involved in sleep/wake regulation by inducing anti-stress agents and/or the production of ketone bodies, which may result in resilience to social stress.

### [1P-231]

**Distinct sex-specific responses in a rat model of chronic stress**

*Faadiel Essop[1] ([1]*Stellenbosch University*)

Psychological stress can elicit the onset and progression of cardio-metabolic diseases. As the underlying mechanisms remain unclear, we first set out to establish a chronic stress model. Male and female Wistar rats were subjected to a 4-week restraint stress protocol vs. controls. Here, stressed male rats showed a decreased % change in body weight over time vs. controls ($p<0.01$). The male stressed group exhibited higher plasma corticosterone levels vs. controls ($p<0.01$), with no differences for plasma adrenocorticotropic hormone (ACTH). Male brain-derived neurotrophic factor levels were lower in the stress group vs. controls ($p<0.05$). Stressed males also displayed less attempts into the closed arms of the elevated plus maze (EPM) vs. controls ($p<0.05$). There were no weight changes for female rats. However, stressed females exhibited lower plasma corticosterone levels, while displaying higher plasma ACTH levels vs. controls ($p<0.05$). Stressed females also showed increased rears (EPM test) vs. controls ($p < 0.01$). Our findings reveal intriguing sex-based differences, with males displaying a depressive-type phenotype while females exhibited a post-traumatic stress disorder phenotype.

### [1P-232]

### The response to lower intestinal peristalsis evoked by stimulation of the hypothalamic stress center in the rat

*Naoya Kikuchi[1], Mio Mathuyama[1], Joji Horiuchi[1] ([1]*Department of Bioengineering. Toyo University*)

Irritable bowel syndrome, one of the stress-related diseases, has symptoms of diarrhea or constipation, and either mechanism has not been elucidated. In particular, diarrhea may be evoked by an increased intestinal motility consequence of parasympathetic dominance and is inconsistent with the stress-induced cardiovascular response. We hypothesized that psychological stress may disrupt the balance of the autonomic nervous system and affect intestinal motility. To investigate this, the hypothalamic stress center, the dorsomedial hypothalamic area (DMH) was chemically stimulated and recorded bowel peristalsis. The DMH stimulation evoked suppression or hyperactivity of intestinal motility. In addition, to test whether the parasympathetic activity is involved in the enhanced bowel peristalsis observed in this experiment, Atropine, a parasympathetic blocker, was administered. The Atropine administration greatly inhibited the increase in bowel motility after the DMH stimulation. These results showed that neurons in the DMH participated in the changes in intestinal motility elicited by psychological stress, suggesting that an increase in intestinal motility during the stress may involve a parasympathetic activity.

### [1P-233]

### The descending pathway from the hypothalamus to the medulla on the pressor response during social defeat stressin rats; the participation of the midbrain

*Mio Matsuyama[1], Shota Ushikubo[1], Joji Horiuchi[1] ([1]*Department of Biomedical Engineering, Toyo University*)

It has been shown that the sympathetic vasomotor pathway of psychological stress is mediated via neurons in the rostroventral medulla (RVM) indirectly from the hypothalamic stress center. Our previous study indicated that directly projecting neurons to the RVM were distributed in the midbrain lateral/ventrolateral periaqueductal grey matter (l/vl PAG) and some of them were excited by an acute psychological stressor. In addition, chemical stimulation of the neurons in the l/vl PAG caused increases in blood pressure and renal sympathetic activity in anesthetized rats. In this study, direct projections to the l/vl PAG, and neuronal excitability in regions that were located rostral to the midbrain were investigated during the social defeat stress (SDS) in conscious rats. FluoroGold (FG), a neural tracer, was injected into the unilateral l/vl PAG and exposed to the SDS a week later. The double-labeled (c-Fos and FG) neurons were locally distributed within the dorsomedial area and the perifornical area in the hypothalamus. Therefore, these results suggested that the pressor response during acute psychological stress may be mediated from the hypothalamus to the RVM via neurons in the l/vl PAG.

### [1P-234]

### Volatile compounds in exhaled air associated with scores of general physical and mental stress

*Akito Shimouchi[1], Kentaro Taniguchi[2], Naoya Jinno[1], Naoya Okumura[1] ([1]*Chubu University*, [2]*Nagahama Bio University*)

We explored volatile chemicals associated with physical and mental conditions. Study 1. Exhaled air of 95 adult volunteers at home were obtained in breath bags. Their breath was analyzed by a gas-chromatograph with a semiconductor sensor and the concentration differences were evaluated between before night sleep and after wake-up in the morning. Study 2. Exhaled air of 188 adult volunteers aged 50 years old or more were obtained during ambient air breathing and analyzed by an Ion Mobility Spectrometer. Physical and mental conditions were evaluated by the Cornel Medical Index and General Health Questionnaires 28. We found that increases in carbon monoxide and acetone during night sleep were significantly associated with depression and neurotic conditions, respectively. Three and one compounds were associated with neurotic grades and depression, respectively.

### [1P-235]

### Analysis of behavioral and immunological responses in exposures to acute as well as chronic restraint stresses

*Shunya Sasaki[1], Hiromasa Higuchi[1], Shuei Sugama[2] ([1]*International University of Health and Welfare, Department of Physical Therapy*, [2]*International University of Health and Welfare, Center for Basic Research*)

### Poster Presentation

[1P]
**Anthropology**

March 14 (Tue.), 12:10 - 14:10, Poster Room

### [1P-236]

### Comparative Primate neuroimaging to understand the evolutional and developmental basis of the human brain

*Tomoko Sakai[1] ([1]*Keio University School of Medicine*)

To understand the nature of the human mind, it is essential to trace the evolutionary origin of the human brain, namely by focusing on nonhuman primates (NHPs), our closest ancestors. The rapid advance of neuroimaging technologies has made it much easier to directly compare the brain systems among humans and NHPs. However, the underlying processes observed in humans remain unclear, in part because the developmental patterns of the brain have not been adequately explored in our closest living primate relatives, the chimpanzees. Thus, we tracked the development of the cerebral tissues in growing chimpanzees from the fetal period to the juvenile period using MRI and ultrasound scanning and compared these results with previously recorded data from humans and macaques. Our results reveal common features of the development of brain tissues among primates, common features between hominoids, as well as unique features of humans. In this presentation, I will introduce five representative topics about our comparative imaging studies of brain development patterns among humans and NHPs. This effort will increase our understanding of the human brain system underlying the high-order cognitive functions and, ultimately, contribute causal identification of the contributory factors for psychiatric disorders.

### Poster Presentation

[1P]
**Pathophysiology**

March 14 (Tue.), 12:10 - 14:10, Poster Room

### [1P-237]

**Epigenome-wide association analysis of pancreatic exocrine cells from high-fat- and normal fat-diet-fed mice.**

Tomoyuki Araki[1], Hajime Hirasawa[2], Fuminobo Tamalu[2], *Naofumi Miwa[2] ([1]*Saitama Medical Univerisy, Department of Biochemistry,* [2]*Saitama Medical University, Department of Physiology*)

Aberrant DNA methylation is associated with oncogenesis of human cancers, including pancreatic cancer (PC). PC is the seventh most common cancer, and obesity is a known high-risk factor. However, the molecular mechanisms whereby obesity promotes carcinogenesis in the pancreatic tissue have not yet been assessed. Collection of pancreatic cells from presymptomatic participants is extremely difficult; therefore, we performed experiments on diet-induced obesity (DIO) mouse model. Furthermore, we utilized the Illumina Mouse Methylation BeadChip that was recently validated for the mouse epigenome-wide association study (EWAS). Like genome-wide association studies, EWAS uses an array format; previous EWAS studies for mice have been performed using human methylation arrays. In this study, we investigated methylation profile of DIO mice, representing a presymptomatic PC status, and extracted differentially methylated regions and signaling pathways that potentially contribute to the oncogensis of human PC. There are no COIs to declare.

### [1P-238]

**Mechanism of decreased expression of the lipid mediator-degrading enzyme in fibrotic lung**

*Yasuo Okamoto[1], Yasuhiro Takenouchi[1], Keisuke Kitakaze[1], Rena Matsui[2], Hironobu Ishimaru[1], Rie Sugimoto[1], Kazuhito Tsuboi[1] ([1]*Kawasaki Medical School,* [2]*Kawasaki University of Medical Welfare*)

Lung fibrosis is a chronic lung disease characterized by excessive accumulation of extracellular matrix and remodeling of the lung architecture. Increased levels of the lipid mediators sphingosine 1-phosphate (S1P) and lysophosphatidic acid (LPA) have been shown to promote lung fibrosis via their receptors. However, the molecular mechanisms that increase S1P and LPA levels are unknown. In this study, we investigated the molecular mechanisms by which S1P and LPA are increased. DNA microarray analysis showed decreased gene expression of lipid phosphatase 3 (LPP3), which degrades both lipid mediators, and its protein expression was also decreased in the lungs of the bleomycin-treated mice compared to the saline-treated lungs. Next, we examined which cells constituting the lungs showed decreased expression of LPP3. Compared with the saline-treated group, LPP3 mRNA expression was decreased in alveolar epithelial cells isolated from the lungs of the bleomycin-treated group. Although several microRNAs have been reported to suppress LPP3 expression, expression of mir-184 was increased in the lungs and alveolar epithelial cells of the bleomycin-treated group. In fact, treatment of A549 cells with miR-184 suppressed LPP3 expression. These results suggest that the decreased expression of LPP3, a degrading enzyme of S1P and LPA, in alveolar epithelial cells in fibrotic lungs causes accumulation of S1P and LPA and promotes fibrosis.

### [1P-239]

**Large negative correlation coefficient between erythrocyte count and mean corpuscular volume (MCV) may account for upregulation of blood cells in orthopedic surgery patients with traumatic injury**

*Katsumasa Kawahara[1,2,3], Koichiro Sato[1], Junya Sekita[1], Yukiko Yasuoka[3], Mariko Nishikitani[4], Hiroshi Nonoguchi[5], Sumiyuki Mii[1], Mitsufumi Nakawaki[1] ([1]*Zama General Hospital,* [2]*Fukushima Medical University,* [3]*Kitasato University,* [4]*University of Kyusyu,* [5]*Kitasato University Medical Center*)

In almost all vertebrates including human, there is a negative (inverse) relationship between erythrocyte count (RBCC) and mean corpuscular volume (MCV) among species to keep stable blood flow for an optimum tissue $O_2$ level (Hawkey CM et al, 1991). However, this has not been well established within the same species, a human. Purpose & Hypothesis: To prevent the orthopedic surgery patients with traumatic injuries from venous thrombosis in association with iron deficiency (without anemia) (Clénin GE, 2017). We hypothesize that correlation coefficient (r) of this negative relationship may reflect blood cells activity, such as cell volume regulation and oncotic pressure tolerance. Materials & Methods: We analyzed the electric medical record test results of 2021-22 for orthopedic patients with traumatic injury and dermatology (no-injury) patients in Zama General Hospital of JMA, according to a guideline for patients' rights. Importantly, the orthopedic patients were sub-grouped into (a) PLT group (n=10): their platelet (PLT) counts exceeded beyond the normal range of 35.5 ($x10^4$/μL) after surgery within 2 wks and (b) non-increased group, control (n=11). Results: Preoperative excerpt data, such as plasma protein (g/dL), total bilirubin (TB)(mg/dL), RBCC ($x10^4$/μL), hemoglobin (Hb) (g/dL), PLT count ($x10^4$ /μL), MCV, MCHC (MC Hb concentration) of both groups, were within normal ranges and were not significantly different from each other except PLT. Basal PLT was found to be significantly ($p<0.01$) higher in PLT group: 27.8 vs. 23.2 of control. (2) PLT values of control increased also to 25.1 (n.s.) and to 28.2 ($p<0.001$) on 7 and 14 days after surgery, respectively. (3) In PLT group, TB transiently and significantly ($p < 0.05$, n=7) increased from 0.66 to 1.03 on 1 day after surgery. (4) More importantly, large negative r levels were obtained before and after surgery in PLT group: -0.74 (before surgery), -0.82 (d1), -0.87 (d3), -0.88 (d7), and -0.87 (d14), while they were not in control: -0.55, -0.46, -0.07, -0.21, and -0.15 (in sequence). Conclusion and Prospects: The present results are consistent with a view that peripheral blood cells may be relatively more activated probably through upregulated hematopoietic signaling, such as erythropoietin and thrombopoietin, which may be more secreted in perioperative patients with (latent) iron deficiency even without anemia.

### [1P-240]

**Characteristics of water intoxication model mice as a model of brain edema.**

*Emi Nakamura-Maruyama[1], Mai Ishikawa[2], Keiichiro Irie[3], Kazuhiko Narita[1], Naoyuki Himi[1], Takashi Adachi[1], Osamu Miyamoto[1,4], Takehiro Nakamura[1,2,3] ([1]*Department of Physiology2, Kawasaki Medical School,* [2]*Graduate School of Kagawa prefectural University of Health Sciences,* [3]*Department of Neurological Surgery, Kagawa University Faculty of Medicine,* [4]*Departoment of Medical Engineering, Kawasaki University of Medical Welfare Faculty of Health Science and Technology*)

Brain edema is a serious disease that in severe cases can lead to brain herniation and death. However, many treatments for brain edema itself remain classic. In this study, we investigated the characteristics of water intoxication models that can induce pure brain edema in terms of age and gender, and considered their usefulness in the search for new treatment methods. Brain edema was induced in C57BL/6J mice (♂ and ♀ 5-52 weeks old) by intraperitoneal administration of distilled water at 10% of body weight. Brain water content was calculated from the difference in weight immediately after brain tissue removal and after drying (120°C, 24 hours). DNA and proteins were extracted from brain tissue and analyzed for 8-OHdG and caspase3. In addition, aquaporin4 (involved in brain edema induction) knockout mice were intraperitoneally injected with water and were compared the result of the water content to that of wild-type mice. These results showed that the time course of water content varied with age and gender. 8-OHdG and caspase3 activity also differed with age. The aquaporin4 knockout mice showed inhibition of brain edema induction. Thus, this study demonstrated the age- and gender-specific differences in water intoxication, and the importance of considering these factors in creating animal models of water intoxication and potentially other models of brain edema. Furthermore, as it is possible to study using this model with genetically modified mice, this model would be useful in search for a treatment for brain edema.

### [1P-241]

**A novel drug for cancer stem cell-specific ion channels**

*Mikio Hayashi[1] ([1]*Kansai Medical University*)

Glioblastoma multiforme (GBM) is the most fatal malignant primary brain tumor. GBM contains functional subsets of cells called glioblastoma stem-like cells (GSCs), which are radio- and chemo-resistant and eventually lead to tumor recurrence. We had found mucolipin, a transient receptor potential channel, in the plasma membrane of GSCs. Thus, the present study aimed to develop anticancer drug targeting mucolipins in GSCs. We found 14 drugs that inhibited cell growth of GSCs using WST-8 assay. Among them, we performed *in silico* screening from a virtual library using docking of the drugs and mucolipin channel. And then, new drugs were created that inhibited GSCs proliferation. This drug increased $Na^+$ currents through mucolipin channels on the plasma membrane of GSCs. The oral administration of the drug significantly prolonged the overall survival of brain tumor-bearing mice. These results demonstrate the potential clinical use of this novel drug for GBM.

### [1P-242]

### Calcium signaling is impaired in the lumbar spine of a rat model of congenital kyphosis

*Noriaki Shimokawa[1], Itsuki Takahashi[1], Yusuke Watanabe[1], Izuki Amano[2], Noriyuki Koibuchi[2] ([1]*Takasaki University of Health and Welfare,* [2]*Gunma University*)

Kyphosis involves the spine curving excessively backward, beyond its physiological curvature. Although the normal structure of the spinal vertebrae is extremely important for maintaining one's posture as well as the normal function of the thoracic and abdominal organs, our understanding of the pathogenesis of the disease is insufficient. We found that the downregulation of the calcium-signaling pathway was involved in the pathogenesis of congenital kyphosis. By analyzing DNA microarray data, we found that the expression of genes associated with the calcium-signaling pathway decreased at flexion sites of the lumbar spine in Ishibashi (IS) rats, a rat model of congenital kyphosis. The expression of calcium-sensing receptor (CaSR) and transient receptor potential vanilloid 1 (Trpv1), two receptors that involve calcium homeostasis in this pathway, also decreased at both the gene and protein levels. We also found that the number of CaSR-immunopositive and Trpv1-immunopositive cells in the lumbar spine of IS rats was lower than in wild-type rats. These results indicate that adequate calcium signaling is extremely important in the regulation of normal bone formation and may also be a key factor for understanding the pathogenesis of congenital kyphosis.

### [1P-243]

### Role of TRPM7 in the pathophysiology of aortic dissection

*Hana Inoue[1], Takashi Nakamura[1], Hiyo Obikane[2], Toshitaka Nagao[2], Utako Yokoyama[1] ([1]*Department of Physiology, Tokyo Medical University,* [2]*Department of Anatomic Pathology, Tokyo Medical University*)

Abnormal mechanotransduction in vascular smooth muscle cells (VSMCs) has been suggested to cause aortic diseases, including aortic dissection (AD), a sudden onset, highly lethal disease. In the present study, we aimed to investigate the involvement of transient receptor potential channel melastatin 7 (TRPM7), the mechanosensitive channel, in AD. We used paraffin-embedded sections of ascending aorta of non-AD (n = 10) and AD (n = 19) patients and performed immunostaining for TRPM7. TRPM7 was extensively expressed in VSMCs of the tunica media, with especially enhanced expression in the undissected region adjacent to the false lumen. To investigate the role of TRPM7 in VSMC mechanotransduction, human aortic VSMCs were cultured under cyclic hydrostatic pressure loading that mimicked hypertension in the presence or absence of a TRPM7 inhibitor, NS8593 (20 μM). Cyclic hydrostatic pressure upregulated gene expression of early growth response 1 (*EGR1*) which mediates mechanotransduction in VSMCs, by 2.9-fold. In the presence of NS8593, *EGR1* expression was further increased to 4.3-fold. These results suggest that TRPM7 downregulates EGR1-mediated mechanotransduction in VSMCs. TRPM7 might be involved in abnormal mechanotransduction in AD.

### [1P-244]

### Assessment of collagen fiber orientation in alcoholic liver disease using polarization-resolved second harmonic generation microscopy

*Haruto Oshikata[1], Saya Matsuzaki[2], Eiji Hase[2], Hiroki Takanari[2], Satoko Kimura[3], Koichi Tsuneyama[2] ([1]*Tokushima University facalty of Med,* [2]*Tokushima University,* [3]*Tokyo examiners Office*)

Liver fibrosis is a pathophysiological change in the liverresulted from collagen accumulation, and causes cirrhosis, portal hypertension, liver failure, and carcinogenesis. It is important to assess the severity of liver fibrosis for staging chronic liver diseases and determining the efficacy of treatment. We attempted to evaluate liver fibrosis in terms of collagen fiber orientation by PR-SHG (polarization-resolved second harmonic generation; PR-SHG)microscopy.Twenty-four autopsy cases of alcoholic liver injury from stage F0 to stage F4 were involved. Unstained liver samples were scanned with an ultrashort pulsed laser (wavelength: 800 nm) to detect SHG light. Thepolarization angle of the linearly polarized irradiation light was rotated from 0° to 165° in 15° steps to acquire 12 consecutive PR-SHG images. Then the parameters for fiber orientation and crystallinity of the collagen were determined as φ and ρ, respectively.There was no apparent tendency between fibrosis stages and the value of φ, while the mean and spatial heterogeneity of ρ increased with the progression of liver fibrosis.The results suggested that PR-SHG could be used to evaluate the degree of liver fibrosis.

### Poster Presentation

Day 2
(March 15, 12:10 - 14:10)

[2P] Neurophysiology, Neuronal cell biology - Plasticity
[2P] Neurophysiology, Neuronal cell biology - Neural network
[2P] Neurophysiology, Neuronal cell biology - Neurochemistry
[2P] Neurophysiology, Neuronal cell biology - Neurons, Synapses
[2P] Neurophysiology, Neuronal cell biology - Glia
[2P] Neurophysiology, Neuronal cell biology - Higher brain function
[2P] Neurophysiology, Neuronal cell biology - Motor function
[2P] Neurophysiology, Neuronal cell biology - Sensory function, Sensory organ
[2P] Molecular physiology, Cell physiology - Membrane transport
[2P] Molecular physiology, Cell physiology - Ion channels, Receptors
[2P] Molecular physiology, Cell physiology - Others
[2P] Embryology, Regenerative Medicine, Development, Growth, Aging
[2P] Muscle
[2P] Digestion, Digestive system
[2P] Oral physiology
[2P] Blood, Lymph, Immunity
[2P] Circulation
[2P] Respiration
[2P] Reproduction
[2P] Endocrine
[2P] Autonomic nervous system
[2P] Environmental physiology
[2P] Physical fitness and sports medicine
[2P] Nutritional and metabolic physiology, Thermoregulation
[2P] Behavior, Biological rhythm, Sleep
[2P] Pathophysiology
[2P] Drug Action, Pharmacology
[2P] Study Methodology

### Poster Presentation

[2P]
### Neurophysiology, Neuronal cell biology
### Plasticity

March 15 (Wed.), 12:10 - 14:10, Poster Room

### [2P-001]

### Chemical LTD stimulation induced the drastic mitochondrial morphological changes in cultured hippocampal neurons

*Naoya Atarashi[1], Shinji Matsuda[1] ([1]*Department of Engineering Science, Graduate School of Informatics and Engineering, The University of Electro-Communications*)

The long-term depression (LTD) has been proposed to play essential roles for certain kinds of memory and learning. Although the endocytosis of AMPA-type glutamate receptor (AMPA receptor) is the molecular basis for the induction of LTD, it is still unknown how the endocytosis of AMPA receptor can be induced. Previous report indicated that the signals from mitochondria were required for the endocytosis of AMPA receptor. Here, we expressed CFP with mitochondria targeting signal (mito-CFP) in cultured hippocampal neurons and examined the morphology of mitochondria before and after chemical LTD-inducing stimulation. Before applying LTD-inducing stimulation, elongated forms of mitochondria were observed in the dendritic regions. On the other hand, after applying the chemical LTD stimulation, the morphology of mitochondria was drastically changed to the round shape. Moreover, we examined the distribution of mitochondria and endo-lysosomes by expressing mito-CFP together with mCherry-tagged Rab4, Rab7, Rab11 and Cathepsin, which are early, late, recycling endosome markers and lysosome marker, respectively. The results indicated that the mitochondria associated with endosomes and lysosomes during the induction of LTD. These results suggested that the signal transduction between mitochondria and endo-lysosomes plays important roles to induce LTD in hippocampal neurons.

### [2P-002]

### Astrocyte-mediated neuronal circuit remodeling in higher visual cortex induced by sensory deprivation

*Ikuko Takeda[1,2], Izumo Takei[1], Yuki Aoyama[1], Hiroaki Wake[1,2] ([1]*Department of Anatomy and Molecular Cell Biology Graduate School of Medicine,* [2]*Division of Multicellular Circuit Dynamics, National Institute for Physiological Sciences*)

Different modality of sensory information projected in higher brain region to integrate their information. Tactile stimulation activates higher visual cortex with congenital visual deprivation (e.g. V2L) and triggers cross-modal plasticity. However, the resulting phenotype varies with the age of visual loss – heightened tactile acuity in puberty, but visual illusions in adulthood. In this research we aim to study the astrocytic contribution on time dependent plasticity with visual loss and study how different age dependent plasticity induce different phenotype in higher visual associated area. We modelled congenital (congenital deprived mice) and acquired blindness (acquired deprived mice) by vision deprivation in 2-week and 5-week old mice, respectively. We found increased numbers of V2L astrocytes overall and of those contacting neuronal somas 5 days after visual deprivation only in congenital deprived mice, and increased intensity and synchronicity in V2L astrocyte $Ca^{2+}$ activities both spontaneously and during whisker stimulation 6 week after visual deprivation in acquired deprived mice. These results suggest V2L astrocytes as the mechanism underpinning the divergent age-related phenotypes after vision-loss. We are now studying how V2L astrocyte $Ca^{2+}$ activity affects neuronal activity on the basis that astrocyte-mediated circuit remodeling in higher visual cortex could be an effective approach for driving circuit construction in line with restoring sensory function.

### [2P-003]

### Alternation of LTP to LTD depending on the number of stimuli in mouse cerebellar Purkinje cells

*Kazuhiko Yamaguchi[1], Atsuro Daida[1], Yuji Takahashi[2], Noritaka Ichinohe[1] ([1]*Dept. Ultrastructural Res., National Institute of Neuroscience, NCNP,* [2]*Dept. of Neurology, National Central Hospital, National Center of Neurology and Psychiatry*)

Long-term depression (LTD) of synaptic transmission at parallel fiber Purkinje cell synapses plays an important role in cerebellum-related motor coordination and learning. However, whether LTD is induced in an all-or-none or graded manner depending on the number of stimuli remains elusive. Here, we investigated the relationship between the number of stimuli (combination of Purkinje cell depolarization and parallel fiber stimulation) and synaptic plasticity in mouse cerebellar slices. We found that a sufficient number of stimuli induced LTD, whereas a small number stimulated a postsynaptic form of long-term potentiation (LTP). Furthermore, we revealed hidden LTD under nitric oxide-free conditions to inhibit LTP by a small number of stimuli. We mathematically reconstituted these three phases of synaptic plasticity in Purkinje cells. In conclusion, we observed three-phase synaptic plasticity depending on the number of stimuli and NO-blocker, indicating that LTP overwrites hidden LTD, but robust LTD overcomes LTP.COI: NO

### [2P-004]

### The corticospinal projections after the motor recovery from spinal cord injury in macaque monkeys

*Satoko Ueno[1,2], Reona Yamaguchi[2], Kaoru Isa[1], Kawasaki Toshinari[1], Masahiro Mitsuhashi[1], Tadashi Isa[1,2,3] ([1]*Department of Neuroscience, Graduate School of Medicine, Kyoto University,* [2]*Institute for the Advanced Study of Human Biology (WPI-ASHBi), Kyoto University,* [3]*Human Brain Research Center, Graduate School of Medicine, Kyoto University*)

Our recent studies showed that the macaque monkeys with subhemisection of the cervical spinal cord considerably recovered their hand movements following intensive behavioral tests and cortical electrical stimulation. There must have been neural pathways carrying the motor commands in these monkeys, though the corticospinal tract (CST) and the other descending motor pathways were mostly transected on the ipsilesional side. It was known that the CST locally bypassing the lesion area in the subhemisetion or hemisection models after the recovery. However, the overall pattern of the CST projection has not been investigated.Here, we labeled the CST fibers by injecting anterograde viral tracers into the motor cortex of our subhemisection models after the recovery. A number of the labeled fibers originating from the contralesional motor cortex showed massive plasticity at the pyramidal decussation which is relatively apart from the lesion. These labeled fibers projected to the original terminal area on the ipsilesional side of the gray matter at the caudal to the lesion. This extensive plasticity of CST might have been induced by the intensive behavioral test and the cortical stimulation.

### [2P-005]

### Caudate neurons encode eye positions during intersaccadic interval in non-retinocentric coordinate frame : implication for oculomotor reinforcement learning

*Rikako Kato[1], Hiroki Bando[2], Tadashi Isa[2,1] ([1]*Kyoto University Institute for the Advanced Study of Human Biology,* [2]*Graduate School of Medicine and Faculty of Medicine*)

We recently reported oculomotor reinforcement learning during a task to find a hidden area (HA) located within the monitor screen (HA search task) in macaque monkeys (R, Kato et al. Sci. Rep. 2021). In the process of locating a HA, both the non-retinocentric (non-RC) location of the start position of final saccades to the HA and its retinocentric (RC) vector were being reinforced with a learning. The caudate nucleus (CN) is considered to play a critical role in an oculomotor reinforcement learning. Neural activity related to saccadic vector in the RC coordinates has been widely examined in the CN neurons. However, neural activity related with the non-RC coordinates has not been reported. In the present study, to identify neural activity related to the eye position signals in the non-RC coordinates in the CN neurons, we recorded neural activities while monkeys performed the HA search task and the memory guided saccade task with five different start positions. Many CN neurons showed a modulation in the non-RC coordinate frame in their activity during both the HA search task and the memory guided saccade task. The non-RC coordinate signals shifted from that at the saccade start position to that of the end position around the saccades. We found the non-RC coordinate signals in the neural activity of the CN neurons. Such neural signals suffice a necessary condition for the CN to be the locus of adaptation for the reinforcement learning of the oculomotor behavior in the non-RC coordinate.

### [2P-006]

### Regulation of plasticity of axon initial segment by LINC complex

*Koichi Hasegawa[1], Takeshi. K. Matsui[1], Junpei Kondo[1], Noriyuki Hama[1], Ken-ichiro Kuwako[1] ([1]*Shimane University School of Medicine*)

The axon initial segment (AIS) is located at the proximal site of axon and plays a pivotal role in initiation of action potentials in neurons. AIS is a highly plastic structure, that changes in length, position, and molecular composition in response to neural activity. The nuclear membrane LINC complex, composed of Sun1/2 and Nesprin-1/2, connects the nucleus to the cytoskeleton and is involved in cell polarity and migration. In this study, we focused on the regulation of AIS by the LINC complex. The Nesprin dominant-negative (nesprin-DN) mutant that inhibits LINC function significantly shortened the AIS length in a variety of neurons *in vitro* and *in vivo*. Furthermore, cortical neurons-expressing nesprin-DN lost the structural changes in the AIS under chronic depolarization conditions, suggesting that the LINC complex is involved in the regulation of AIS plasticity. To further elucidate the mechanism of the LINC complex-mediated AIS regulation, we expressed the loss-of-function mutants of nesprin2 in cortical neurons. Expression of the actin-unbound form of nesprin-2, like nesprin-DN, significantly shortened AIS length, whereas the microtubule-unbound form of nesprin-2 did not alter it. This result suggests that the LINC complex regulates AIS plasticity via actin cytoskeleton. In summary, this study demonstrates a novel nuclear-mediated control mechanism of AIS.

### [2P-008]

### Expression level of $Na^{+}$-$K^{+}$-$Cl^{-}$ co-transporter 1 on oligodendrocytes affects axonal position-dependency of myelinated fiber plasticity

*Yoshihiko Yamazaki[1], Hiroki Fujiwara[1], Jun-Ichi Goto[1], Satoshi Fujii[1] ([1]*Yamagata Univ.*)

Beyond saltatory conduction, the modulatory effects of oligodendrocytes (OLs) on neural function, which is called myelinated fiber plasticity, have come to be recognized. This OL-related plasticity includes an early-onset, short-lasting increase in conduction velocity. The activity of $Na^{+}$-$K^{+}$-$Cl^{-}$ co-transporter 1 (NKCC1), which is expressed abundantly in OLs at the juvenile stage, plays a role in myelinated fiber plasticity. We previously reported that the magnitude of the increase in conduction velocity induced by OL depolarization varies at different positions along the axon. In this study, we investigated whether the expression level of oligodendrocytic NKCC1 correlates with positional dependency for myelinated fiber plasticity. Using mice with OLs expressing channelrhodopsin-2, we performed whole-cell recordings from CA1 pyramidal cells and recorded antidromic action potentials by stimulating the axon at different positions and examined the effects of OL depolarization on axonal velocity. The change in latency was evident in action potentials with a latency of 1.5–2.5 msec. The overexpression of *Nkcc1* in OLs extended the region that showed a significant increase in the conduction velocity. These results indicate that the increase in NKCC1 activity on OLs facilitates myelinated fiber plasticity.

### [2P-007]

### Olfactory learning-dependent plasticity of neuronal connection from piriform cortex to olfactory tubercle in mice

*MD FAZLEY RABBI SHA[1], Yuriko Koga[1], Yoshihiro Murata[1], Mutsuo Taniguchi[1], Masahiro Yamaguchi[1] ([1]*Kochi Medical School,Kochi University*)

We previously revealed that the olfactory tubercle (OT), which belongs to the olfactory cortex and ventral striatum, has functional domains that represent odor-guided motivated behaviors. dependent activation of specific OT domains remains unknown. We hypothesized that neuronal connectivity of OT domains plastically changes through olfactory experience. OT domains, inputs from the piriform cortex to OT were optogenetically stimulated in mice in association with food reward for attractive learning and with electrical foot shock for aversive learning. The size of photo-activated axon boutons preferentially increased in amOT compared to lOT for attractive behavior learned mice and increased in lOT compared to amOT for aversive behavior learned mice.These results indicate the learning-dependent plasticity of synaptic connections to OT domains.

### Poster Presentation

[2P]
### Neurophysiology, Neuronal cell biology
**Neural network**

March 15 (Wed.), 12:10 - 14:10, Poster Room

### [2P-009]

**Analysis of expectation-modulated dynamics of dopamine release during food seeking behavior in mice**

*Tomohiro Shibata[1], Takaaki Ozawa[2], Yuma Matsumoto[1], Moe Nakamura[1], Ryotaro Iwamoto[1], Yoshinobu Oyama[1], Tom Macpherson[2], Takatoshi Hikida[2] ([1]*Graduate school of science, Osaka university, Japan,* [2]*Institute for Protein Research, Osaka University, Japan*)

Mesolimbic dopamine neurons have been considered to encode reward prediction error (RPE) which is basically calculated from the difference between the actual reward and the temporal difference of the estimated value function. However, learning-dependent shift of the striatal dopamine release dynamics during an associative reward learning has not yet been investigated. To address this issue, we conducted continuous recording of striatal dopamine release in mice during 15-day auditory appetitive conditioning by taking advantage of a fluorescence dopamine sensor (GRAB-DA2m). We trained head-fixed mice in an auditory pavlovian appetitive conditioning paradigm where one auditory stimulus (CS-High) is followed by a liquid reward delivery (US) with high probability (80%) but it was delivered with low probability (20%) after the other auditory cue (CS-Low). As a result, it was revealed that RPE coding in dopamine was eventually observed after 15-day trainings. Notably, we also found that very initial stage of learning (day1-3) was characterized by small but significant CS-evoked dopamine and its error insensitivity, (2) RPE cording of dopamine emerges from later stage (day 4). These results suggest that learning-dependent shift of striatal dopamine dynamics is not fully explained by typical RPE model such as a temporal difference (TD) leaning.

### [2P-010]

**Remodeling of neuronal circuits in the sensory thalamus by postweaning social isolation**

*Hisako Nakayama[1], Mariko Miyata[1] ([1]*Tokyo Women's Medical University*)

Adolescent social isolation is known to have widespread influences on brain functions, including sensory abnormalities and anxiety, and these adverse effects persist into adulthood. However, the underlying neural mechanism is almost unknown. To address this question, we investigated the impact of social isolation in the pathway of whisker sensation, the most critical sense for mice. Tactile information from whiskers is sent to the somatosensory thalamus, VPM neurons in the thalamus through medial lemniscal fibers (MLF). Most VPM neurons receive strong excitatory synaptic input from one MLF (mono-innervation) after weaning age while from multiple MLFs (multiple-innervation) in the early postnatal stage. We found an extensive remodeling at the MLF-VPM synapses (a reappearance of multiple-innervation and a reduction of EPCS) was induced in mice isolated from weaning day but not after sexual maturation (two-month-old). This suggests that MLF-VPM synapses are susceptible to social isolation during adolescence, from weaning to sexual development. Immunostaining showed glucocorticoid receptors (GRs) expressed in neurons and astrocytes in the VPM. We will discuss the contribution of GRs to social isolation-induced synapse remodeling in the VPM.

### [2P-011]

**In-house manufacture of Diesel2p mesoscope and demonstration of large field-of-view two-photon calcium imaging during a conditioning task**

*Fumiya Imamura[1], Hiroto Imamura[1], Hiroo Ikeda[1], Yoshikazu Isomura[1], Riichiro Hira[1] ([1]*Tokyo Medical and Dental University*)

Two-photon microscopy is well suited for observing highly scattering tissues such as living brain and has been used to visualize spine morphology and neural activity for years. Recently, the development of two-photon microscopes with a large field-of-view has accelerated, and it is now possible to visualize more than 100,000 neural activities simultaneously. In this study, we constructed an open-source mesoscope, Diesel2p, with the field-of-view of 7 mm (the largest in the world) and used it to visualize neural activity in multiple areas of the mouse cerebral cortex during conditioning. The objective lens, tube lens, relay lens, polarization beam splitter, and dichroic mirror were all custom-built with some modifications from the original ones. Scanners (galvano and resonant mirrors) and photomultiplier tubes were placed, and these were driven by custom-made software. The light source was a fixed wavelength fiber laser, ALCOR 920 (<100 fs, 2 W). We conducted two-photon calcium imaging during conditioning of head-fixed mice injected with AAV-syn-GCaMP8s and visualized the distribution of task-related neurons including neurons related to the reward-prediction and the omission. We are also investigating changes in the correlation structure of internal and interareal circuits before and after synaptic inhibition with eOPN3, a molecule that suppresses synaptic transmission by light in a Gi-dependent manner. The development of such new methods will deepen our understanding of the functional coordination among task-related neurons distributed across many cortical areas.

### [2P-012]

**Emotional arousal enhances perceptual memory through amygdalo-cortical inputs during NREM sleep**

*Yoshihito Saito[1,2], Yuma Osako[3], Maya Odagawa[1], Yasuhiro Oisi[1], Chie Matsubara[1], Mitsuhiro Morita[2], Masanori Murayama[1] ([1]*RIKEN Center for Brain Science,* [2]*Department of Biology, Graduate School of Science, Kobe University,* [3]*Department of Brain and Cognitive Sciences, Picower Institute for Learning and Memory, Massachusetts Institute of Technology*)

The basolateral amygdala (BLA) activation by emotional arousal is thought to enhance memory through its projections to memory-storing regions. However, how the BLA interacts with other regions to enhance memory remains unclear. Adding emotional information onto a perceptual memory task that rely on top-down inputs from frontal to sensory cortices, we demonstrated that the BLA stores emotional memory and enhances perceptual memory through BLA-frontal projections. Electrophysiological recording revealed emotional information increases reactivation of coordinated activity across the BLA-frontal-sensory region during non-rapid eye movement (NREM) but not REM sleep. Furthermore, optogenetic silencing of BLA terminals at the frontal cortex during NREM sleep disrupted only perceptual memory enhancement but not perceptual and emotional memories themselves, indicating the crucial role of the sleep-state-dependent BLA-cortical inputs on memory enhancement.

### [2P-013]

**High-throughput mapping of multi-synaptic functional pathways from the cerebrum to the SNr.**

*Hikaru Sugino[1], Tatsumi Yoshida[1], Yoshikazu Isomura[1], Riichiro Hira[1] ([1]*Tokyo Medical and Dental Univ.*)

The substantia nigra pars reticulata (SNr), the final output of the basal ganglia, sends inhibitory signals to the cortex via the thalamus and is known to contribute to the maintenance of sustained activity in working memory as well as motor control. In monkeys and rodents, Nambu and colleague found that SNr exhibits triphasic (excitation-inhibition-excitation) activity in response to cortical stimulation, which may be responsible for the organization of movement. This cortico-basal ganglia-thalamo-cortical loop structure is thought to cooperate with another multi-synaptic loop, the cerebral cortex-cerebellar loop, in motor control and working memory, but the relationship between the two is not well understood. In this study, we used scanning optogenetics and Neuropixels (high-density probes) to examine how cortical activity affects the temporal activity patterns of the SNr and cerebellar nuclei in awake head-fixed Thy1-ChR2 rats. Stimulation was performed at 100 ms intervals in a 32 x 32 grid over a 10 mm x 10 mm area that included the entire dorsal frontal and parietal cortex of the left and right hemispheres. By comparing the location of neuropixels identified by DiI fluorescence after fixation with the correlation structure of the LFP responses of each channel, we identified the electrode locations that were successfully recorded in the SNr and cerebellar nuclei. In the SNr, the response pattern of single neurons was dependent on their location within the SNr, with fast tri-phase activity patterns in the lateral part of the SNr and slow biphasic (inhibitory-excitatory) activity patterns observed more frequently in the medial part of the SNr. In the cerebellar nuclei, fast and slow activity patterns were also observed, depending on the recording location. The fast and slow activities may convey distinct information such as movement and working memory, respectively, and have different but coordinated effects on the cerebral cortex.

### [2P-014]

### Interaction between voltage-gated sodium channel and fibroblast growth factor homologous factor: Implication for pathophysiology of intractable epilepsy

*Ikuo Ogiwara[1], Chengzhu Yin[1], Atsushi Shimohata[1], Mie Gangi[1], Makoto Kaneda[1]
([1]*Department of Physiology, Nippon Medical School*)

Voltage-gated sodium channel type I, Nav1.1, encoded by the *SCN1A* gene, and fibroblast growth factor homologous factor, FHF1, encoded by the *FGF12* gene, have been associated infantile epilepsies. Loss of function mutations of *SCN1A* cause Dravet syndrome, characterized with intractable infantile epilepsy with neurodevelopmental delay. A *de novo* recurrent mutation of *FGF12* has been described in neonatal-onset epilepsy. We have previously reported that Nav1.1 is co-precipitated with FHF1 in mouse brain lysate, and *vice versa*, although it has been reported that Nav1.1 could not bind to FHF1. As was previously reported, FHF1 did not bind to the C-terminal domain of Nav1.1. We however found that FHF1 interacted with Nav1.1 through the intracellular loop between the transmembrane domains of the channel. FHF1 mutant also bound to the Nav1.1 intracellular loop but not to the C-terminal domain. We also found that wild-type FHF1 did not affect electrophysiological property of Nav1.1, but mutant FHF1 altered it. Our studies will contribute to the understanding of Nav1.1 and FHF1 in the pathophysiology of early-onset intractable epilepsy with developmental delay.

### [2P-015]

### Cortical spine dynamics during motor learning

*Yoshiyuki KUBOTA[1,2,3], Jaerin Sohn[1,4], Yasuo Kawaguchi[5] ([1]*National Institute for Physiological Sciences,* [2]*SOKENDAI,* [3]*RIKEN Center for Brain Science,* [4]*Osaka University,* [5]*Tamagawa University*)

In mammalian neocortex, learning triggers the formation and turnover of new postsynaptic spines on pyramidal cell dendrites. However, the biological principles of spine reorganization during learning remain elusive because the identity of their presynaptic neuronal partners is unknown. We show that two presynaptic neural circuits supervise distinct programs of spine dynamics to execute learning. We imaged spine dynamics in motor cortex during learning and performed post-hoc identification of their afferent presynaptic neurons. New spines that appeared during learning formed small transient contacts with corticocortical neurons that were eliminated on skill acquisition. In contrast, persistent spines with axons from thalamic neurons were formed and enlarged. These results suggest that pyramidal cell dendrites in motor cortex use a neural circuit division-of-labor during skill learning, with dynamic teaching contacts from top-down intracortical axons followed by synaptic memory formation driven by thalamic axons. Dual spine supervision may govern diverse skill learning in neocortex.

### [2P-016]

### Identification and characterization of Asef2 splicing variant bound to RNA

*Toshihiro Yamanoi[1], Manami Toriyama[2], Hiroshi Itoh[2], Ikuko Yao[1], Michinori Toriyama[1] ([1]*Biomedical Chemistry major, Graduate School of Science and Technology, Kwansei Gakuin University,* [2]*Division of Biological Science, NAIST*)

Cytoskeletal rearrangement plays pivotal role in cell morphology and motility for proper organogenesis. Asef2 (SPATA13) has been reported to function as a GEF for Cdc42 and Rac1 and positively regulate cell migration in various types of cells. These findings are from studies with the short splicing variant of Asef2 (Asef2-short), However, despite the long splicing variants of Asef2 (Asef2-long) has been identified, its function is poorly understood. In this study, we aimed to characterize the molecular function of Asef2-long. While Asef2-short is ubiquitously expressed in various tissues, Asef2-long is transiently expressed in the embryonic brain especially its expression was observed in neural stem cells. To address the molecular function of Asef2-long by focusing its long N-terminal region, we performed F-RIP (Fluorescence-based RNA ImmunoPrecipitation) assay to determine whether Asef2-long bind to RNA. We found a novel mRNA binding activity at 151-689 a.a. of the N-terminal region of Asef2-long. Furthermore, we perform the SELEX method to clarify the bound RNA sequence to Asef2-long, and identified the TAARGCCRCC [R : A or G] sequence was the essential sequence. Bioinformatics analysis identified 11 mRNAs having this sequence which are common among human, mouse, and rat. In this presentation, we focus on the interaction of Asef2 with RNA and outline its effects on neural stem cell differentiation and migration.

### [2P-017]

### Generation and in vivo functional evaluation of novel ACR2 reporter mice

*Akiyo Nakamura[1], Yasutaka Mukai[1], Yan Li[1], Noriaki Fukatsu[1], Daisuke Iijima[1], Manabu Abe[2], Kenji Sakimura[2], Keiichi Itoi[3], Akihiro Yamanaka[4] ([1]*Nagoya University,* [2]*Niigata University,* [3]*Tohoku University,* [4]*Chinese Institute for Brain Research, Beijing*)

### [2P-018]

### A blood-brain barrier-penetrating AAV2 capsid variant that evades neutralizing antibodies against AAV9 capsid protein

*Hayato Kawabata[1], Ayumu Konno[1,2], Yasunori Matsuzaki[1,2], Hirokazu Hirai[1,2]
([1]*Department of Neurophysiology and Neural Repair, Gunma University Graduate School of Medicine,* [2]*Viral Vector Core, Gunma University Initiative for Advanced Research (GIAR)*)

Recent progress of adeno-associated virus (AAV) technology brought several adeno-associated virus (AAV) capsid variants, such as PHP.B and PHP.eB, that cross mouse blood-brain barrier (BBB). Those BBB-penetrating capsid variants are all derived from AAV9. BBB-penetrating capacity of AAV-PHP.eB is mouse-strain dependent: it works in C57BL/6 mice, but not in BALB/c mice. Moreover, second intravenous infusion of AAV-PHP.eB with over one week interval after the first infusion fails to transduce the mouse brain because first systemic infusion produces neutralizing antibodies (NAbs) against AAV9 capsid. Thus, CNS transduction by intravenous infusion of AAV-PHP.eB is one time only, and limited to mice of certain strains such as C57BL/6. Here, we developed an AAV serotype 2 (AAV2) capsid mutant, AAV2-BR1N, that crosses mouse BBB and allows CNS transduction even in the presence of NAbs against AAV9. AAV2-BR1N can penetrate the BBB of BALB/c mice as well as that of C57BL/6 mice. These results suggest that AAV2-BR1N is available for mouse strains that show failure of BBB penetration by AAV-PHP.eB, and that mouse CNS can be transduced twice at arbitrary timing, using AAV9-derived capsid variants and AAV2-BR1N.

### Poster Presentation

[2P]

**Neurophysiology, Neuronal cell biology**

**Neurochemistry**

March 15 (Wed.), 12:10 - 14:10, Poster Room

### [2P-019]

**Thioredoxin regulates oxidative stress in neurons**

*Iori Ohmori[1], Mamoru Ouchida[1], Saeko Ishida[2], Tomoji Mashimo[2] ([1]*Okayama University*, [2]*University of Tokyo*)

Thioredoxin (TXN), encoded by *Txn1*, acts as a critical antioxidant in the defense against oxidative stress by regulating the dithiol/disulfide balance of interacting proteins. The role of TXN in the central nervous system (CNS) is largely unknown. A phenotype-driven study of *N*-ethyl-*N*-nitrosourea-mutated rats with wild running seizures revealed the importance of *Txn1* mutations in CNS degeneration. Genetic mapping identified *Txn1*-F54L in the epileptic rats. The insulin-reducing activity of *Txn1*-F54L was approximately one-third of that of the wild-type (WT). Bilateral symmetrical vacuolar degeneration in the midbrain, mainly in the thalamus and the inferior colliculus, was observed in the *Txn1*-F54L rats. The lesions displayed neuronal and oligodendrocytic cell death. Neurons in *Txn1*-F54L rats showed morphological changes in the mitochondria. Vacuolar degeneration peaked at five weeks of age, and spontaneous repair began at seven weeks. The TUNEL assay showed that fibroblasts derived from homozygotes were susceptible to cell death under oxidative stress. In five-week-old WT rats, energy metabolism in the thalamus was significantly higher than that in the cerebral cortex. In conclusion, in juvenile rats *Txn1* seems to play an essential role in reducing oxidative stress in the midbrain with high energy metabolism.

### [2P-020]

**Expression of Myeloid-derived growth factor in the retina after zebrafish optic nerve injury**

*Kayo Sugitani[1], Takumi Mokuya[1], Yuya Omori[1], Serika Hosoi[1], Kazuhiro Ogai[1] ([1]*Kanazawa Univ.*)

Unlike mammals, fish have regenerative capacity in the central nervous system after injury. In zebrafish, the retinotectal connection can be re-established within one month after optic nerve injury. Thus, the zebrafish optic nerve has been used as a regeneration model of the central nervous system for understanding their molecular mechanisms.  Here, we focused on myeloid-derived growth factor (MYDGF), a bone marrow-derived growth factor, as a molecule involved in optic nerve regeneration in fish. MYDGF is a secreted protein consisting of 173 amino acids in humans, and has recently been shown to protect and repair myocardium after myocardial infarction. In zebrafish, the expression of MYDGF increased in the retina 1 hour after optic nerve injury. The localization of MYDGF protein expression was the outer nuclear layer, the outer plexiform layer and the inner plexiform layer. These results suggesting that MYDGF is also synthesized and secreted by neurons, and involved in the early regenerative process after optic nerve injury.

### [2P-021]

**Calcium imaging reveals the gut-brain axis via the vagus nerve involvement in the sucrose preference reduction after psychological stress in mice**

*Serika Yamada[1], Kazuki Harada[2], Takashi Tsuboi[2], Hiromu Monai[1] ([1]*ochanomizujoshi*, [2]*Department of Life Sciences, Graduate School of Arts and Sciences, The University of Tokyo, Tokyo, Japan*)

Sucrose preference in mice is a reliable index for depression. However, its mechanism has not been fully understood. Some studies have suggested that the brain could detect glucose rapidly through the afferent vagus nerve by sodium-glucose cotransporter 1 (SGLT1) on intestinal epitheliums. This signal may activate dopaminergic neurons, which induce reward sense. Thus, we hypothesized that this signal could be related to the change in sucrose preference of depression model mice that are subjected to psychological stress. To elucidate this mechanism, we examined whether glucose injected into the gut could change cortical dopaminergic $Ca^{2+}$ activity through vagus nerve activation in mice. First, we directly injected glucose solution through a catheter inserted into the gut. Simultaneously, we monitored the cortex-wide $Ca^{2+}$ dynamics using transgenic mice that express genetically encoded calcium indicators G-CaMP7 in the brain. We found that intragastric glucose injection induced the prefrontal cortical $Ca^{2+}$ elevation within 10 s, which was reduced by SGLT1 antagonist, dopamine receptor antagonist, or psychological stress. These results suggest that the gut-brain axis via the vagus nerve plays an important role in sucrose preference and changes after psychological stress.

### [2P-022]

**Molecules mechanisms responsible for differences in hypothalamic neuronal differentiation potential under different spatial culture conditions**

*Tsukushi Suzuki[1], Shunya Tsukamoto[1], Yoshinari Mera[1], Moeka Kuno[1], Miho Kawata[1], Yu Kodani[1], Kanako Saito[1], Akira Nakashima[2], Toshiki Kameyama[1], Hiroshi Nagasaki[1] ([1]*Fujita Health University Physiology I*, [2]*Fujita Health University Physiological Chemistry*)

The hypothalamus, the centre of the autonomic nervous system, is composed of multiple nuclei and neuronal subtypes. However, molecular mechanisms of the differentiation of these diverse neurons remain unclear.With the expectation of regenerative medicine, many studies have been conducted to induce the differentiation of hypothalamic neurons in vitro.To induce hypothalamic neuronal differentiation from pluripotent ES cells in vitro, a serum-free floating culture of embryonic body-like aggregates with quick reaggregation(SFEBq) has been widely used.There are two ways to mature hypothalamic neurons in this method: dissociation monolayers in two-dimensional(2D) culture and maintaining organoid structure in three-dimensional(3D) culture.In this study, we found that AVP neurons differentiate more frequently under 3D culture conditions than 2D, while SST neurons differentiate more frequently under 2D culture conditions. We then performed RNA-seq analysis of progenitor cells in SFEBq culture and differentiated nerves in 2D and 3D. We compared the expression of patterning and neuronal cell fate genes between cultured under 2D and 3D conditions.We also validated the expression of some key transcription factors related to the differentiation of the AVP neuron by RNA-seq and qRT-PCR. We are going to over-expressed these transcription factors and investigate the possible involvement in AVP neuronal differentiation.

### [2P-023]

**Involvement of Semaphorin3A-PlexinA signaling in amyloid-β production**

*Takumi Sekiguchi[1], Takashi Sakurai[1], Naoya Yamashita[1,2] ([1]*Juntendo Univ.*, [2]*Kanagawa Inst. Tech.*)

Alzheimer's disease (AD) is one of the neurodegenerative disorders that must be overcome in countries facing an aging society like Japan.  Amyloid-β (Aβ) is thought to play a central role in AD pathology.  However, the mechanism that causes Aβ overproduction by disrupting homeostatic regulation in aging human brains has not been clarified.  Here we report a potential role for Semmaphorin3A (Sema3A) signaling, known to be upregulated in AD brains, in regulating Aβ production.  We found that PlexinA, a sema3A receptor component, interacts with Aβ precursor protein (APP).  PlexinA and APP interacted through their extracellular regions, and we were able to narrow down these regions to less than 100 amino acids.  Moreover, Sema3A inhibited the NGF-enhanced non-amyloidgenic cleavage of APP, which could reduce Aβ production, in cultured neurons.  These findings suggest that upregulation of Sema3A signaling may enhance Aβ production in AD brains.  Based on these findings, we are now investigating whether the PlexinA-APP interaction affects APP function and metabolism, which might provide a new perspective that aberrant Sema3A signaling induces Aβ overproduction.

### Poster Presentation

[2P]
**Neurophysiology, Neuronal cell biology**
**Neurons, Synapses**

March 15 (Wed.), 12:10 - 14:10, Poster Room

### [2P-024]

**Quantitative control of synaptic suppression by CB2 through decreasing $Ca^{2+}$ influx in a Purkinje cell bouton**

*Takuma Inoshita[1], Shin-ya Kawaguchi[1] ([1]*Graduate School of Science, Kyoto University*)

Synaptic transmission is weakened by cannabinoids at various synapses in the central nervous system. Cerebellar Purkinje cells (PCs) have been suggested to express cannabinoid receptor type 2 (CB2), but not type 1 (CB1), while previous slice patch clamp studies demonstrated lack of cannabinoid-mediated modulation of PC outputs. We first studied the reason why CB2 activation did not show any changes of PC outputs. Immunofluorescent staining showed little CB2 signal at PC axon terminals, although slight signal could be seen at soma and dendrites. Thus, insufficient CB2 amount at terminals might be responsible for the lack of PC output modulation by cannabinoid. To confirm this idea, we exogenously expressed CB2 in cultured PCs. Activation of transfected CB2 reduced synaptic outputs through decrease of presynaptic $Ca^{2+}$ current at a PC axon terminal. Direct patch clamp recordings from a presynaptic terminal showed that CB2 activation did not affect the amount of readily-releasable synaptic vesicles. Taken all these results together, our data suggest that insufficient CB2 level underlies the lack of cannabinoid-mediated control of PC outputs, and that CB2, if exogenously expressed, down-regulates synaptic transmission primarily by the decrease of $Ca^{2+}$ influx.

### [2P-025]

**Super-resolution imaging of the presynaptic active zone at the developing somatosensory thalamus**

*Mitsuharu Midorikawa[1], Sakamoto Hirokazu[2], Miyata Mariko[1] ([1]*Tokyo Women's Medical University*, [2]*University of Tokyo*)

During the development, it has been shown that the synaptic transmission between neurons changes drastically. On the presynaptic side, a developmental change largely relies on the kinetics of the transmitter release. It has been postulated that the developmental tightening of the coupling distance between the synaptic vesicle release site and voltage-gated calcium channels (VGCCs) is crucial to determine the maturation of the transmitter release kinetics, but direct observation is lacking. We focused on the synapse at the ventral posteromedial thalamic nucleus (VPM) at the rodent somatosensory thalamus, which mediates input from the whisker. Recently, we clarified the functional development of the single presynaptic terminal via direct patch-clamp recordings from the presynaptic terminals (Midorikawa & Miyata, 2021, PNAS). By applying two-color three-dimensional stochastic optical reconstruction microscopy (STORM), we visualized the nanoscale geometry of synaptic release sites and VGCCs. Our analysis revealed the developmental tightening of the coupling distance quantitatively, which is consistent with the developmental change of the transmitter release kinetics.

### [2P-026]

**Triple-color 3D nanoscopic analysis reveals retrograde alignment of trans-synaptic Nrxn-Cbln1-GluD2 complex**

*Taku Sogabe[1], Kazuya Nozawa[1], Ayumi Hayashi[1], Michisuke Yuzaki[1] ([1]*Keio Univ.*)

In the brain, synapse formation is driven by various types of synaptic organizers. A secreted type of synaptic organizer Cbln1 interacts directly with pre- and postsynaptic receptors, Neurexin (Nrxn) and GluD2, respectively, and plays an essential role in the formation/maintenance of synapses between parallel fibers (PFs) and Purkinje cells (PCs) in the cerebellum. Although Nrxn, Cbln1, and GluD2 form a tripartite complex in vitro, it remains unclear whether and how the complex is formed in vivo. Here, combining X10 Expansion microscopy (X10 ExM) with improved labeling strategies for endogenous molecules, we revealed that Nrxn, Cbln1, and GluD2 exist as nanoclusters aligning trans-synaptically, indicating that the tripartite complex is formed across PF-PC synapses in vivo. Genetic disruption of GluD2 caused almost complete loss of Nrxn and Cbln1 at the presynaptic terminal. In contrast, deletion of the Cbln1 gene did not affect the size of GluD2 nanoculsters but reduced the nanocluster number. These findings indicate a retrograde mechanism driven by GluD2 nanoclusters leading to the trans-synaptic alignment of Nrxn and Cbln1 nanocluster complex.

### [2P-027]

**CDK5/p35 is involved in the structural plasticity of axon initial segment by regulating microtubule dynamics**

*Ryota Adachi[1], Israt Jahan[1], Ryo Egawa[1], Haruka Nomura[1], Hiroshi Kuba[1] ([1]*Dept. of Cell Physiology, Grad. Sch. of Med., Nagoya Univ.*)

The axon initial segment (AIS), located on the axon close to the soma, is a specialized compartment that has a high density of voltage-gated ion channels, and a bundled microtubule structure. AIS is a place of the action potential generation, and changes the length or location depending on neural activity. This structural plasticity of the AIS contributes to the homeostatic control of activity and optimizes the function of neural circuits. However, the underlying mechanisms are not fully understood. In this study, we prepared a slice culture containing chicken nucleus magnocellularis (NM), a homologue of mammalian anteroventral cochlear nucleus, that reproduces most features of AIS plasticity *in vivo*. Treating the culture with a high-$K^+$ medium shortened the AIS and reduced sodium current and membrane excitability in NM neurons. Pharmacological analyses revealed that this AIS shortening was driven by multiple $Ca^{2+}$ pathways and subsequent signaling molecules that converge on CDK5. AIS shortening was suppressed by overexpression of dominant-negative CDK5, whereas it was facilitated by the overexpression of p35, an activator of CDK5. Moreover, microtubule stabilizers occluded AIS shortening during the p35 overexpression, indicating that CDK5/p35 mediated AIS shortening by regulating the dynamics of microtubules at distal AIS. This study highlights the importance of microtubule reorganization and regulation of CDK5 activity in structural AIS plasticity and the tuning of AIS characteristics in neurons.

### [2P-028]

**Elucidation of the physiological roles of ER-mitochondria contact sites on adult hippocampal neurogenesis**

*Shinobu Miyake[1], Masanobu Kuriyama[1], Hirotaka Shoji[3], Yui Sakurai[1], Masafumi Tsuboi[1], Itaru Imayoshi[2], Tsuyoshi Miyakawa[3], Yusuke Hirabayashi[1] ([1]*University of Tokyo*, [2]*Kyoto Univ.*, [3]*Fujita Health Univ.*)

Post-Traumatic Stress Disorder (PTSD) is an anxiety disorder characterized by heightened reactivity to neutral stimuli resembling past severely traumatic events. It has been thought that this disorder is ascribed to the inability in separating the patterns of external stimuli (pattern separation). Adult neurogenesis in the hippocampus plays a key role in the pattern separation. Indeed, it has been reported that the ability of pattern separation was improved by promoting adult neurogenesis. On the other hand, a previous report identified the endoplasmic reticulum (ER) - mitochondria tethering factor PDZD8 as a risk factor for PTSD. Therefore, our study aims to examine the possibility that PDZD8 regulates adult neurogenesis and associated pattern separation via ER-mitochondrial contact. To date, We have investigated the roles of PDZD8 in the fate regulation of NSCs with tamoxifen-inducible PDZD8 conditional KO mice. We have also analyzed the effect of PDZD8 deficiency on behavioral phenotypes and brain functions. These investigations revealed the roles of PDZD8 in regulating the number of NSCs in an age-dependent manner. These results provide an important clue to elucidate the mechanism of adult NSC fate regulation from a new perspective of organelle contacts.

### [2P-029]

### TRPA1 regulates neural activity in the anterior cingulate cortex

*Kawabata Ryo[1,2], Yao Ikuko[1], Arata Akiko[2], Koga Kohei[2] ([1]*Kwansei Gakuin University,* [2]*Hyogo Medical University*)

Transient receptor potential ankyrin 1 (TRPA1) is one of the TRP channel family, and express in both peripheral and central nervous systems. In the peripheral nervous system, TRPA1 senses low temperature, $O_2$ as well as pain. However, there are less reports about TRPA1 in the central nervous system. Here, we studied the role of TRPA1 in the adult mice anterior cingulate cortex (ACC) where were related pain and emotion. First, we examined the contributions of TRPA1 on the pyramidal neurons of the layer II/III in the ACC slice preparation using whole-cell patch-clamp method. Cinnamaldehyde (CA) as a TRPA1 agonist produced inward currents and these currents were blocked by a TRPA1 antagonist HC 030031. While CA changed the shape and frequency of action potentials (APs) but CA did not change the property of synaptic transmissions. Second, we examined the functional roles of TRPA1 on central hypoxia in the ACC. Biphasic effects that were inward currents in early phase and outward currents in late phase were seen in hypoxia condition. The inward currents were reduced by TRPA1 antagonist, the outward currents were completely blocked by $K_{ATP}$ channel blocker. These results suggest that TRPA1 acts on postsynaptic neurons in the ACC and plays important roles as an acute $O_2$ sensor.

### [2P-030]

### Analysis platform for amyloid precursor protein processing using hippocampal slice cultures

*Yuji Kamikubo[1], Yiyao Zhou[1], Hao Jin[1], Yoshie Hashimoto[1], Takashi Sakurai[1] ([1]*Juntendo Univ.*)

Alzheimer's disease (AD) is a progressive neurodegenerative brain disorder and the most common cause of senile dementia. The presence of large numbers of amyloid plaques is one of the characteristic features of AD. Amyloid beta peptide (Aβ) is derived from amyloid precursor protein (APP) and is a major component of amyloid plaques. APP is commonly cleaved by membrane proteases in the secretase family: α-, β-, and γ-secretase. Aβ is generated by sequential cleavage by β-secretase and γ-secretase, whereas alternative cleavage by α-secretase prevents Aβ production. In this report, we showed the organotypic hippocampal slice culture methods and consecutive analysis of Aβ and related products, which play a central role in the pathogenesis of AD. Continuous sampling and analysis of APP cleavage products is a powerful approach for elucidating AD's cellular and molecular mechanisms. Long-term and consecutive analyses are also required to develop and evaluate therapeutic agents and treatment approaches for AD.

### [2P-031]

### Genetic manipulation of neuronal activity facilitated Kv1.1 expression in period- and tonotopic-dependent manners in avian cochlear nucleus during development.

*Hesheng CHEN[1], Ryo EGAWA[1], Hiroshi KUBA[1] ([1]*Nagoya Univ.*)

The tonotopic organization is a topographic representation of sound frequency in the auditory pathway, in which neurons are arranged orderly according to their characteristic frequency (CF). Avian nucleus magnocellularis (NM) is a homolog of the mammalian anteroventral cochlear nucleus and is well known for a graded expression of potassium channels (Kv1.1) along the tonotopic axis, while the expression being higher in neurons with high CFs. Our group previously revealed that the expression of Kv1.1 is accelerated steeply in the high-CF neurons after hatch (embryonic day 21, E21). However, it remains unclear why the acceleration of Kv1.1 expression occurs specifically after hatch, even though the animals can start to hear around E12 and the auditory threshold reaches the mature level before hatch. This raises a possibility that the lack of this acceleration before the hatch could be related to the intrinsic properties of the neurons; more specifically, the ability of the neurons to express Kv1.1 in response to pre- and/or post-synaptic activities could be still immature during the period. Thus, we tested this possibility by genetically manipulating the activity of NM neurons in vivo through introducing either overexpression of Kir2.1 or PSAMs (pharmacological-selective-actuator modules), while examining the effects on Kv1.1 current with whole-cell patch-clamp recording before hatch. Overexpression of Kir2.1 hyperpolarized the cells by ~20 mV, and this reduced Kv1 current by ~70% in neurons with high-tuning frequencies. Moreover, overexpression of PSAMs augmented the generation of action potentials, which increased Kv1 current by two times. Notably, this facilitation of Kv1 current did not occur just after the hearing onset and appeared only after the late embryonic period. These results indicated that the activity-dependent mechanism of Kv1.1 expression develops during maturation, which underlies the period- and tonotopy-specific acceleration of Kv1.1 expression in the nucleus.

### [2P-032]

### Essential Role of Somatic Kv2 Channels in High-Frequency Firing in Cartwheel Cells of the Dorsal Cochlear Nucleus

*Tomohiko Irie[1] ([1]*Division of Pharmacology, National Institute of Health Sciences*)

Among all voltage-gated potassium (Kv) channels, Kv2 channels are the most widely expressed in the mammalian brain. However, studying Kv2 in neurons has been challenging because of a lack of high-selective blockers. Recently, a peptide toxin, guangxitoxin-1E (GxTX), has been identified as a specific inhibitor of Kv2, thus facilitating the study of Kv2 in neurons. The mammalian dorsal cochlear nucleus (DCN) integrates auditory and somatosensory information. In the DCN, cartwheel inhibitory interneurons receive excitatory synaptic inputs from parallel fibers conveying somatosensory information. The activation of parallel fibers drives action potentials in the cartwheel cells up to 130 Hz in vivo, and the excitation of cartwheel cells leads to the strong inhibition of principal cells. Therefore, cartwheel cells play crucial roles in monaural sound localization and cancelling detection of self-generated sounds. However, how Kv2 controls the high-frequency firing in cartwheel cells is unknown. In this study, we performed immunofluorescence labeling with anti-Kv2.1 and anti-Kv2.2 antibodies using fixed mouse brainstem slice preparations. The results revealed that Kv2.1 and Kv2.2 were largely present on the cartwheel cell body membrane but not on the axon initial segment (AIS) nor the proximal dendrite. Whole-cell patch-clamp recordings using mouse brainstem slice preparation and GxTX demonstrated that blockade of Kv2 induced failure of parallel fiber-induced action potentials when parallel fibers were stimulated at high frequencies (30-100 Hz). Thus, somatic Kv2 in cartwheel cells regulates the action potentials in a frequency-dependent manner and may play important roles in the DCN function.

### [2P-033]

### Analysis of novel function of nobiletin for neural circuit formation

*Ayame Furukawa[1], Ikuko Yao[1], Michinori Toriyama[1] ([1]*Biomedical Chemistry major, Graduate School of Science and Technology, Kwansei Gakuin University*)

Nobiletin is a polymethoxylated flavonoid enriched in *citrus* peels. Recent studies demonstrated that nobiletin and its metabolites have various physiological activities including anti-inflammation, anti-oxidation, anticancer, and neuroprotection. However, their effects on neural circuit formation were poorly understood. In this study, we aim to address the effects of nobiletin in cultured hippocampal neurons as a model. We examined dendritic spine density in cultured neurons. Dendritic spines are small protrusions forming on neuronal dendrites which is an essential structure for neural synapse connection. Neurons were treated with nobiletin at DIV (days in vitro) 7 and immunostained at DIV9 with an anti-PSD95 antibody that specifically recognize dendritic spines. The density of dendritic spines was significantly increased compared with controls. These results suggested that nobiletin has a positive activity on neural circuit formation. To clarify the molecular mechanism of nobiletin in neurons, we conducted to identify nobiletin binding proteins by affinity purification followed by mass spectrometric analysis. 5-O-Demethylnobiletin, which is a derivative form of nobiletin, was covalently coupled with magnetic beads and then incubated with mouse brain lysate. The bound proteins were eluted and then analyzed by mass spectrometry. The identified proteins contained a large number of RNA-binding proteins (RBPs). This result indicates that nobiletin is involved in regulating RNA function via its binding proteins. In this presentation, we introduce our recent findings of nobiletin function in neural circuit formation.

### Poster Presentation

[2P]
**Neurophysiology, Neuronal cell biology**
**Glia**

March 15 (Wed.), 12:10 - 14:10, Poster Room

### [2P-034]

**Acute neuroinflammation triggers a dopaminergic surge: a D1 dopamine receptors mediated compensatory role against inhibited spontaneous activity and sleep disturbances**

*Mohammed E Choudhury[1], Yuki Nishikawa[2], Hajime Yano[1], Toshihiro Yorozuya[2], Junya Tanaka[1] ([1]*Department of Molecular and Cellular Physiology, Ehime University Graduate School of Medicine,* [2]*Department of Anesthesia and Perioperative Medicine, Ehime University Graduate School of Medicine*)

Immune modulatory role of dopamine (DA) has been documented via line of evidences. This study shows that rat microglia predominantly express D1 and D4 receptors (D1R and D4R). In response to the D1R agonist SKF-81297C, cultured microglia increased intracellular cAMP and suppressed lipopolysaccharide (LPS)-induced expression of interleukin 1b (IL-1b) and tumor necrosis a (TNF-a). Since delirium is considered a neuroinflammatory disease, we prepared a delirium model in male rats by intraperitoneal administration of LPS, which increases DA levels at prefrontal cortex (PFC) following 21 hours of LPS administration. Following 10 hours of LPS administration, increased expression of TNF-a and IL-1b was seen in sorted microglia in the delirium model. However, expression of TNF-α, but not IL-1β, was sharply reduced 21 h after LPS administration, when DA levels were elevated, suggesting that DA may play a role in suppressing neuroinflammation. Although, administration of L-DOPA to the delirium model rats did not suppress expression of the cytokines but SKF-81297 did. Considering crucial role of D1R on guiding neuroinflammation, it may be a novel effective therapy for delirium.

### [2P-035]

**Developmental oligodendrocytes contribute to cerebellar maturation**

*Ryo Masumura[1], Naofumi Uesaka[1] ([1]*Tokyo Medical and Dental University, Graduate School of Medical and Dental Sciences, Cognitive Neurobiology*)

Proliferation and differentiation of oligodendrocyte (OL) and myelin formation continue throughout life, but are mostly concentrated during postnatal development. During the developmental stage, some of the over-formed synapses are selectively strengthened and maintained, while other synapses are removed. This process, known as synapse elimination, is crucial for transforming immature neural circuits into functionally mature versions. However, despite the coincidence of the timing of both processes, the relationship between OL and synapse elimination remains largely unexplored. Here, we focused on the cerebellar climbing fiber-purkinje cell synapse during postnatal development to examine the relationship between OL and synapse elimination. We further tested the effects of cerebellar OL disruption during synapse elimination on brain functions. We found that the removal of the cerebellar OL during the period of synapse elimination results in excessive residual climbing fiber synapses, increased anxiety tendency, decreased sociality, and impaired motor function. These findings indicate that the OL is essential for synapse elimination in normal development and suggest that OL destruction during postnatal development result in psychiatric disorders and motor deficits.

### [2P-036]

**Change of the blood-brain barrier in Alzheimer's disease model mice.**

*Lingnan Hou[1], Jinglei Cheng[1], Zhongtian Guo[1,2], Daisuke Kato[1,2], Hiroaki Wake[1,2] ([1]*Department of Anatomy and Molecular Cell Biology, Nagoya University Graduate School of Medicine,* [2]*Division of Multicellular Circuit Dynamics, National Institute for Physiological Sciences, National Institutes of Natural Sciences*)

Alzheimer's disease (AD) is a neurodegenerative disease with pathological features such as the accumulation of amyloid plaques and tau tangles. Previous studies showed that blood-brain barrier (BBB) leakage is associated with cognitive decline in the early stage of AD. BBB leakage due to the abnormal glial cell may be part of a cascade of pathologic events that lead to immune activation that ultimately induce cognitive decline. However, the timeline and underlying mechanism of the BBB leakage in APP knock-in mouse (as a mouse model of AD, developed and provided by Dr. Takaomi Saido, Riken) remain unclear. In this research, we investigated the structural and functional changes of BBB by electron microscopy and evaluate the BBB leakage using two-photon in vivo imaging. We found that the length of tight junctions in AD model mice decreased from around 12~16 weeks using electron microscopy. On the other hand, microglia start to accumulate with blood vessels from 11~13 weeks that detected by two-photon *in vivo* imaging. These results suggest microglia accumulation cause tight junction losing and BBB alteration. We are currently trying to detect microglia and other glial cells contribution to regulation of BBB integrity in AD model mice.

### [2P-037]

**Effects of monocular deprivation on the development of oligodendrocyte progenitor cells in primary visual cortex**

*Hyeryun Shin[1], Hideki Kawai[1] ([1]*Department of Science and Engineering for Sustainable Innovation, Faculty of Science and Engineering, Soka University*)

Sensory experience modulates proliferation and differentiation of oligodendrocyte progenitor cells (OPCs). We previously found that binocular enucleation increased the undifferentiation of OPCs during postnatal days (P) 22~25 in the primary visual cortex (V1) of mouse. In this study, we examined whether monocular deprivation (MD) promotes OPC proliferation. We compared the effects of monocular enucleation (ME) and lid suturing (LS), which does and does not serve optic nerves, respectively. We found that proliferated OPCs labeled with bromodeoxyuridine (BrdU+ NG2+ PDGFRa+) appear to increase in both contralateral binocular and monocular regions of V1 following ME during P22~25 as compared to ipsilateral V1. In LS mice, proliferated, cell cycling OPCs (BrdU+ Ki67+ NG2+ PDGFRa+) increased in the contralateral monocular region of V1, but not in the binocular region. Reactive gliosis as measured by glial fibrillary acidic protein (GFAP) and macrophage accumulation as measured by CD68 increased in the deprived side of dorsal lateral geniculate nucleus (dLGN) following ME, whereas such changes were not observed in the non-deprived side of ME mice as well as in both sides of dLGN in LS mice. Overall, it would be suggested that MD by LS or ME regulated OPC undifferentiation in V1 in a neuronal activity-dependent manner independent of thalamic gliosis.

### [2P-038]

**Role of microglia in higher visual sensory integration**

*Mai Kagamiuchi[1], Ikuko Takeda[1,2], Hiroaki Wake[1,2] ([1]*Department of Anatomy and Molecular Cell Biology Nagoya University Graduate School of Medicine,* [2]*Division of Multicellular Circuit Dynamics National Institute for Physiological Sciences, National Institutes of Natural Sciences*)

### [2P-039]

### Synergistic activation of astrocytic TRPV4 by multiple ligands

*Amane Tateishi[1], Koji Shibasaki[1] ([1]*Lab of Neurochem, Dep of Nutrition Sci, University of Nagasaki*)

We previously reported that TRPV4 is expressed in ~30% subpopulation of astrocytes in brain, and the TRPV4 activation leads to gliotransmitter (ATP and glutamate) release and increases synaptic transmission (Shibasaki et al. JBC 2014, J. Anesth 2016). It has been reported that TRPV4 is activated by various stimuli such as warmth temperature (>34°C), hypotonic stimulus, extracellular arachidonic acid and mechanical stimulus. All TRP channels have unique properties called as synergistic effects. If we apply two different agonists, thresholds of each agonist can be effectively reduced. Thus, we can observe significant TRP channel activation by combination of two different agonists. These backgrounds indicate that TRPV4 can be specifically potentiated by combinational application of two different agonists. In this study, we examined the possibility by an electrophysiological and an Ca2+-imaging experiments. Combinational application of arachidonic acid and hypotonic stimuli significantly potentiated the TRPV4 activation. These results indicate that endogenous TRPV4 is strongly activated by multiple ligands, and lead to enhanced gliotransmitter release in naïve astrocytes.

### [2P-040]

### Exploration of the interaction between microglia and cancer cells that involved with brain metastasis formation

*Misuzu Horikoshi[1], Takahiro Tsuji[1], Mariko Shindo[1,2], Hartantio Rahadian[1,2], Daisuke Kato[1], Hiroaki Wake[1,2] ([1]*Department of Anatomy and Molecular Cell Biology, Graduate School of Medicine, Nagoya University,* [2]*National Institute for Physiological Sciences, Okazaki, Japan*)

### Poster Presentation

[2P]
### Neurophysiology, Neuronal cell biology
### Higher brain function

March 15 (Wed.), 12:10 - 14:10, Poster Room

### [2P-041]

### Task-dependent encodings of decision variables in the orbitofrontal cortex.

*Tomoya Ohnuki[1,2], Yuma Osako[1,3], Kazuki Shiotani[1,4], Yuta Tanisumi[1,5], Shogo Takamiya[1], Nagi Matsui[4], Hiroyuki Manabe[1], Yoshio Sakurai[1], Junya Hirokawa[1]
(*[1]Doshisya University, Graduate School of Brain Science, [2]RIKEN, Center for Brain Science, [3]Massachusetts Institute of Technology, Picower Institute for Learning and Memory, [4]Ritsumeikan University, College of Life Sciences, [5]National Institute for Physiological Sciences*)

Neurons in the prefrontal cortex form distinct functional subpopulations that represent task-relevant cognitive information. Those subpopulations represent key decision variables such as decision confidence, uncertainty, and past success/failure experiences, suggesting that those variables are integrated to support optimal decisions based on reward expectations. However, whether and how those subpopulation-level encodings emerge with the task demands relevant to those encodings is unknown. Furthermore, the mechanisms by which neurons coordinate within and across the functional subpopulations are still unidentified. In this study, we found that the degree of demands in a decision-making task changed rats' choice strategy, single-neuron tunings to the same decision variables, and subpopulation-level encodings. To analyze higher-order cognitive representations, we trained rats in a high-dimensional behavioral task in which rats decided whether they wait for or forgo a potential water reward based on the evidence provided by an odor cue in each trial. We investigated how task demands changed the decision variable encodings by making rats alternatively perform the two tasks with longer and shorter reward delay periods during multi-channel electrophysiological recordings.

### [2P-042]

### Differential and temporally dynamic involvement of primate amygdala nuclei for social and reward information processing.

*Koji Kuraoka[1], Kae Nakamura[1] (*[1]Kansai Medical University*)

Social and reward information is independent in nature while they are closely related. While the primate amygdala has been implicated in processing both pieces of information, it is unclear whether they are computed separately or conjointly. To this end, we developed a behavioral task in which the monkeys (n=2) made saccades under different social and reward contexts. After fixation on a central fixation point (FP), a target dot was presented on the left or right of the FP, to which they made a saccade to obtain a liquid reward. During a trial, one of 8 images with two attributes: social reality: a monkey or cartoon face, and reward: large or small, was presented twice: earlier, after fixation onset (S1) and later, before target presentation (S2). We found that neurons in the distinct nuclei of the amygdala encode social and reward information separately, with a specific time course within a trial. In the lateral nucleus (LA), as a population, neurons responded more stronger to monkey- than cartoon- faces, especially during the S1 presentation. In the basal nucleus (BA), neurons showed stronger responses to large than small reward-indicating faces, especially during the S1. In the central nucleus (CE), neurons showed stronger responses to large than small reward-indicating faces, especially during the S2. These results indicate anatomically- and temporally- distinct social and reward information processing: social and reward information in the LA and BA, respectively, at the sensory encoding phase; reward information in the CE, at the time close to action and outcome phase.

### [2P-043]

### The analysis of accumbal dopamine dynamics during observational fear in mice

*Ryotaro Iwamoto[1,2], Takaaki Ozawa[1,2], Tom Macpherson[1,2], Takatoshi Hikida[1,2]
(*[1]Institute of Protein Research, Osaka University, [2]Graduate School of Science, Osaka University*)

Previous studies have demonstrated that mice show observational fear response in which the observer animal expresses fearful behavior in response to the expression of unpleasantness by another conspecific. Dopaminergic circuit plays an important role in regulation of aversive as well as appetitive behavior. Previous studies found that dopamine release was inhibited in response to aversive predictive cues and aversive stimuli themselves in the nucleus accumbens; however, little is known about how this accumbal dopamine level changes during observational fear. To address this question, we recorded accumbal dopamine release during observational fear by taking advantage of the fluorescence dopamine sensor, GRAB-DA2m. In the present study, we trained mice in the auditory observational fear conditioning paradigm where each of one animal (observer) and the other animal (demonstrator) was placed into two different behavioral compartments which are separated by perforated transparent wall. During conditioning, auditory cue (conditioned stimulus, CS) was followed by mild electric shock (unconditioned stimulus, US) only in the demonstrator side. This study will pave the way to understand the neural circuit of negative empathy in the animal model.

### [2P-044]

### Ipsilesional spatial bias after a focal cerebral infarction in the medial agranular cortex and posterior parietal cortex

*Daisuke Ishii[1,2], Hironobu Osaki[3], Arito Yozu[4], Kiyoshige Ishibashi[1], Kenta Kawamura[1], Satoshi Yamamoto[1], Mariko Miyata[5], Yutaka Kohno[1] (*[1]Ibaraki Prefectural University of Health Sciences, [2]Department of Cognitive Behavioral Physiology, Chiba University Graduate School of Medicine, [3]Laboratory of Functional Brain Circuit Construction, Graduate School of Brain Science, Doshisha University, [4]Department of Precision Engineering, The University of Tokyo, [5]Tokyo Women's Medical Univ. School of Medicine, Division of Neurophysiology, Department of Physiology*)

In this study, a mouse model of unilateral spatial neglect (USN) was created to investigate whether the size of the lesion is related to the severity of ipsilesional spatial bias and the recovery process. Photothrombosis was used to induce focal infarctions in three different areas (anterior to posterior) of the right medial agranular cortex (AGm) and posterior parietal cortex (PPC) of mice. After induction of cerebral infarction, ipsilesional spatial bias was evaluated for 9 consecutive days. We also assessed motor paralysis using the horizontal ladder rung walking test. The major findings were as follows: unilateral local infarction of the AGm and PPC resulted in ipsilateral bias during internally guided decision-making; (2) none of the mice showed gait disorder; (3) mice with anterior AGm lesions experienced lower USN recovery rates. These findings suggest that recovery from ipsilesional spatial bias requires neural plasticity within the anterior AGm. This conditional mouse model of ipsilesional spatial bias may be used to develop effective treatments for unilateral spatial neglect in humans.

### [2P-045]

### Accumbal dopamine response to salt intake depends on internal sodium level in mice

*Issei Nakagawa[1,2], Takaaki Ozawa[1,2], Tom Macpherson[1,2], Takatoshi Hikida[1,2] (*[1]Osaka Univ, Dept. Biol., Grad. Sch. Sci, [2]Osaka Univ, Inst. for Protein Research*)

The palatability of salt changes depending on our internal sodium level. For example, a low internal sodium state increases the palatability of a high concentration of salt, whereas it rather induces an aversive taste in the animal in a normal natrium state. The dopaminergic circuit plays an important role in the regulation of aversive as well as appetitive behavior. Previous studies have revealed that food reward increases, but aversive stimulus suppresses dopamine level in the nucleus accumbnens (NAc). However, it remains unknown whether dopamine release in the NAc shows an opposite response pattern to salt intake depending on internal sodium level. To address this question, we recorded dopamine release in the NAc during salt consumption behavior by using the fluorescent dopamine sensor, GRAB-DA2m. In the experiment, deionized water, and 300 or 750 mM NaCl solutions were randomly given to mice under water or sodium restriction. As a result, we found that dehydrated mice preferred deionized water compared to NaCl solution, whereas sodium-restricted mice preferred NaCl solutions. Furthermore, it was also found that, under water restriction, NAc dopamine was increased in response to water intake while it was suppressed by salt intake. On the other hand, these dopamine response patterns to water and salt were totally flipped when mice are under sodium restriction. These results suggest that the dopamine change in the NAc reflects the palatability of salt in a state-dependent manner.

### [2P-046]

### Social behavior analysis of monkeys in a group based on a novel 3D markerless motion capture system.

*Jumpei Matsumoto[1,2], Takaaki Kaneko[3], Kei Kimura[3], Salvador Negrete[4], Naoko Suda-Hashimoto[5], Akihisa Kaneko[5], Mayumi Morimoto[5], Eri Sumiya[3], Hiroshi Nishimaru[1,2], Tsuyoshi Setogawa[1,2], Yasuhiro Go[6,7], Tomohiro Shibata[4], Hisao Nishijo[1,2], Masahiko Takada[3], Ken-ichi Inoue[3] ([1]*Dept System Emotional Sci, Faculty of Med, Univ of Toyama, Toyama, Japan, [2]Research Center for Idling Brain Sci, Univ of Toyama, Toyama, Japan, [3]Systems Neurosci Section, Center for the Evolutionary Origins of Human Behavior, Kyoto Univ, Inuyama, Japan, [4]Dept of Human Intelligence Systems, Grad Sch Life Sci and Systems Engineering, Kyushu Inst of Tech, Kitakyushu, Japan, [5]Center for the Evolutionary Origins of Human Behavior, Kyoto Univ, Inuyama, Japan, [6]Cognitive Genomics Research Group, Exploratory Research Center on Life and Living Systems (ExCELLS), National Inst of Natural Sci, Okazaki, Japan, [7]Dept of System Neurosci, National Institute for Physiological Sci, Okazaki, Japan*)

Monkeys form well-organized groups and make complex social decisions based on relationships between individuals and social contexts. To achieve objective and automatic analysis of the social behavior of monkeys in a group, we developed a 3D markerless motion capture system for multiple monkeys. The present system tracked individual monkeys and estimated their 3D poses (3D locations of 15 body parts) based on the videos captured from eight cameras installed in a group cage. We applied the system for analyzing social behaviors between four monkeys in a group cage in long term recordings (8 days x 2 groups). Social behaviors (e.g., attacking, grooming, looking another monkey) were automatically detected based on the 3D motion data. Comparisons of the counts of these social behaviors revealed the different interactions depending on pairs of monkeys. Furthermore, the individuals were successfully discriminated based on the quantified social behaviors (correct ratio = 86.1 ± 14.5 % (mean ± S.D.), support vector machine). These results suggest that present system will be useful in future studies for studying complex social behaviors in non-human primates and its impairment in models of neuropsychiatric disorders.

### [2P-047]

### Arithmetic neurons in the dorsal premotor cortex of the monkey

*Sumito Okuyama[1], Mushiake Hajime[1] ([1]*Tohoku univ.*)

Arithmetic is an important cognitive skill in everyday life, and also a foundation in science and technology. The neural architecture that enables such behavior has not been specified. Here we show cells in the dorsal premotor cortex selectively exhibit activities for numerical addition or subtraction. Most of these activities changed their coding from arithmetic to motor representations such as hand to be used or number of steps(arithmetic neurons). We find a biased distribution in the relationship between operations and hand. Specifically, among cells that coded for both arithmetic and hands, we frequently found neurons coding addition and the right hand and neurons coding subtraction and the left hand, suggesting arithmetic and hand-use are intertwined with each other. Moreover, we computationally trained a statistical classifier to predict monkey's arithmetic decisions of addition or subtraction from arithmetic neurons. Without further training, addition vs subtraction classifier predicted right hand vs left hand trials. These finding implies that arithmetic operations are cognitively embodied in motor function by recycling inherent neural systems in the motor cortex.

### [2P-048]

### Transcranial ultrasound stimulation revealed a causal role of anterior prefrontal-putamen circuit for response inhibition in humans

*Koji Nakajima[1,2], Takahiro Osada[2], Akitoshi Ogawa[2], Masaki Tanaka[2], Satoshi Oka[2], Koji Kamagata[3], Shigeki Aoki[3], Yasushi Oshima[1], Sakae Tanaka[1], Seiki Konishi[2] ([1]*Department of Orthopaedic Surgery, The University of Tokyo School of Medicine, [2]Department of Neurophysiology, Juntendo University School of Medicine, [3]Department of Radiology, Juntendo University School of Medicine*)

It is known that stopping inappropriate responses requires the involvement of the prefrontal-subthalamic hyperdirect pathway. However, how the prefrontal-striatal indirect pathway contributes to stopping is not fully understood. In this study, the essentiality of the striatum for response inhibition was examined by transcranial ultrasound stimulation (TUS), which can suppress the activity of deep brain structures. Functional magnetic resonance imaging (MRI) revealed activation in the right anterior part of the putamen in the striatum during a stop-signal task, and TUS to the anterior putamen, as well as the subthalamic nucleus, resulted in significant impairments in stopping performance. Furthermore, diffusion MRI revealed prominent structural connectivity between the anterior putamen and the right anterior part of the inferior frontal cortex (IFC). TUS to the anterior IFC also showed significant impairments in stopping performance. These results indicate that the right anterior putamen and right anterior IFC causally contribute to stopping, suggesting that the anterior IFC-anterior putamen circuit in the indirect pathway serves as an essential route for stopping.

### [2P-049]

### Sex difference of the information process in the brain as revealed by autocorrelation function of the brain BOLD signal

*Tomohiro Donishi[1], Yoshiki Kaneoke[1] ([1]*Wakayama Medical University*)

Neural activity in the brain is dependent on its past activity, which is useful for the integration and maintenance of complex information. Magnitude of the dependence of the neural activity on the past activity can be measured by its autocorrelation function. Coefficient of the autocorrelation function at lag 1 ($r_1$) has been shown to be associated with cortical regions and wakefulness. In this study, we explored sex difference of the $r_1$ using the data for right-handed healthy volunteers (N=586, 18-84 years old). A 3-Tesla MRI was used to measure BOLD brain signals. We calculated the value of $r_1$ at each voxel for 15 min and the mean value for each brain region (total 388 regions including the subcortical nuclei and the cerebellum). The $r_1$ value was varied with regions from 0.18 to 0.63. The values in almost all cortical regions for males were significantly larger than those for females under 30 years old. The values at all the regions for both males and females decreased with age. The value was not affected by the menstrual phase of the young female subjects (N=59, 18-22 years old). Our results suggest that information process in the brain for young males is distinct from that for females at the same generation.

### [2P-050]

### Evaluation of sense of agency in adolescents with anorexia nervosa

*Tasuku Kitajima[1], Toshihiro Kawase[2], Toshimitsu Takahashi[3], Soichiro Fujiki[3], Kensaku Nomoto[3], Ryoichi Sakuta[1], Kenji Kansaku[3] ([1]*Child Development and Psychosomatic Medicine Center, Dokkyo Medical University Saitama Medical Center, [2]Department of Information and Communication Engineering, School of Engineering, Tokyo Denki University, [3]Department of Physiology, Dokkyo Medical University School of Medicine*)

Sense of agency (SA) is a core subjective experience in body representation. Patients with anorexia nervosa (AN) tend to engage in excessive exercise regardless of conditions of the body, but the SA in AN has not been well investigated. We have developed a robotic arm illusion task in which a robotic arm is controlled by myoelectricity (Sato et al. , 2018). In the present study, we investigated SA in adolescents with AN using the robotic arm illusion task.Eleven female adolescents with AN admitted for renourishment treatment were included in the study, and performed the robotic arm illusion task at the beginning and end of the hospitalization. The robotic arm with a single joint (wrist flexion and extension) were controlled by means of the myoelectricity on the wrist flexor and extensor. The participants took part in the in-phase and out-of-phase movement conditions for 2 min each and answered a questionnaire to assess SA immediately after each experiment.SA scores were positive (significantly above zero, p=0.01) in the in-phase for the AN at admission. After the inpatient treatment, SA scores in the in-phase significantly decreased and were not positive (p=0.01).SA changed dynamically before and after the renourishment treatment in patients with AN, suggesting that their self-body representation may change dynamically depending on the stage of the disease.

### Poster Presentation

[2P]
### Neurophysiology, Neuronal cell biology
**Motor function**

March 15 (Wed.), 12:10 - 14:10, Poster Room

### [2P-051]

**Eyeblink conditioning established robustly to the ratio of paired sensory stimuli**

*Ryota Iwase[1], Shin-ya Kawaguchi[1] ([1]*Graduate School of Science, Kyoto University*)

Eyeblink conditioning in mice has been a major model of cerebellar-dependent motor learning. Typically, high rate of pairing of conditioned stimulus (CS, tone) and unconditioned stimulus (US, air puff) is used for the training of animals in eyeblink conditioning. However, it remains elusive how the proportion of paired stimuli impacts on the success rate and dynamics of learning. To address this issue, here we examined the effect of decreasing the ratio of CS-US paired trials on the establishment of the classical learning paradigm by monitoring conditioned response (CR) percentage, peak latency, amplitude, and velocity of eyelid closure quantitatively. Mice were head-fixed on a treadmill and given 10 blocks of daily training consisting of altered combination of CS-US trial and CS-only trial per 1 block. Eyelid movements were monitored by a 100 frames/sec with a CMOS camera, analysed using ImageJ. We also examined whether the expression of conditioned response depends on cerebellum, by local inhibition by administering muscimol through an internal cannula targeting cerebellar cortex.Here we like to demonstrate how the classical reinforcement learning is robust to the change of stimulus pairing rate.

### [2P-052]

**Distinct populations of neurons in the primary motor cortex, supplementary motor area, and caudal cingulate motor area of monkeys contribute to initiations of contralateral and ipsilateral hand movements**

*Yoshihisa Nakayama[1], Osamu Yokoyama[1], Eiji Hoshi[1], Yukio Nishimura[1] ([1]*Tokyo Metropolitan Institute of Medical Science*)

The primary motor cortex (M1), supplementary motor area (SMA), and caudal cingulate motor area (CMAc) send projections to the contralateral and ipsilateral spinal cord. However, it is not well understood how the motor areas contribute to initiating contralateral and ipsilateral hand movements. To investigate the behavioral consequences of neuronal firing on contralateral and ipsilateral hand movements, we examined correlations between reaction times (RT) and spike counts of neurons in M1, SMA, and CMAc while monkeys were performing a button-press task with the right or left hand. We found that the correlations of RT for contralateral movements with spike counts of neurons selective for contralateral hand movement (contralateral neurons) were stronger than those selective for ipsilateral (ipsilateral neurons) and both contralateral and ipsilateral (bilateral neurons) hand movements. We also found that the correlations of RT for ipsilateral movements with spike counts of ipsilateral neurons were stronger than those of contralateral and bilateral neurons. Moreover, the two types of correlations in M1 were stronger than those in SMA and CMAc. These findings suggest that distinct populations of neurons in M1, SMA, and CMAc contribute to initiating contralateral and ipsilateral movements, and M1 is more involved in initiating the movement with ipsilateral hand as well as contralateral hand than SMA and CMAc.

### [2P-053]

**Postural control during bipedal behavior in Japanese macaque: Kinematic and ethopharmacological investigation.**

*Kei Mochizuki[1], Akira Murata[2], Masahiko Inase[2] ([1]*Iwate Medical University,* [2]*Kindai University*)

Deterioration of cognitive performance reduces stability of bipedal behavior and eventually causes falling. This phenomenon is a serious problem in current aging society. Animal experiments with appropriate bipedal behavioral tasks are needed to understand how the central nervous system normally acquires and pathologically loses bipedal stability.In the present study, we established a bipedal standing-up and walking task for macaque monkeys. The monkey was freed in an escape-prevented space and performed bipedal behaviors on a force platform. We combined video-based kinematic analysis with low-dose intramuscular ketamine injection (approximately one-tenth of the anesthetic dose). This technique has been used to induce psychotic cognitive impairment by modifying cortical glutamatergic transmission without immobilization by anesthetic effects. In control sessions with saline injection, the monkey showed an anticipatory adjustment of center of pressure just prior to the gait initiation, which was similar to human subjects. However, this adaptive postural adjustment was disturbed by low-dose ketamine administration. Our animal model will be useful in future studies to unravel the role of cortical control in bipedal behavior.

### [2P-054]

**Deep learning-based quantitative analysis for macaque hand dexterity**

*Yiping Sun[1], Reona Yamaguchi[2], Tadashi Isa[1,2] ([1]*Division of Neurobiology and Physiology, Department of Neuroscience, Graduate School of Medicine in Kyoto University,* [2]*Institute for the Advanced Study of Human Biology (ASHBi), Kyoto University*)

Recently, many studies have summarized the potential mechanisms of fine finger movement recovery using big data analysis. Understanding the contributions of the different neural pathways would help us develop effective therapeutic strategies in the early recovery phase. However, we still don't have a good method for quantitative analysis of hand dexterity for macaque monkeys, which would give us rich information for constructing a comprehensive database to study the potential of different neural pathways in hand dexterity recovery. Therefore, we developed a method using Deeplabcut™ to quantitatively analyze the kinematic performance of reach-and-grasp tasks with different difficulty levels in healthy non-human primates (macaque). We found that compared to the traditionally used fingertip aperture (defined as the distance between the thumb and index finger), the angle between the line connecting the thumb and index fingers' tip and the horizontal line more significantly varies between the tasks and can more sensitively reflect the hand dexterity in different grasping movements, which would be valuable for our future analysis of the recovery process.

### [2P-055]

**Development of a mouse musculoskeletal model by integrating three modal (3M) data sets: 3D Scx-GFP expression patterns, X-ray CT data, and a mouse biomechanical skeletal model**

*Satoshi Oota[1], Hiroki Mori[2], Atsushi Yoshiki[3], Ryutaro Himeno[4], Riichiro Hira[5], Hideo Yokota[1] ([1]*RIKEN RAP,* [2]*Waseda Univ.,* [3]*RIKEN BRC,* [4]*Juntendo Univ.,* [5]*TMDU*)

Our long-term objective is to introduce a biomechanics framework to neurosciences: we will integrate a computational mouse brain model with a mouse neuro-musculoskeletal model to develop a computational mouse brain-body model, by which we aim to realize a comprehensive computer simulation that connects brain functions and the physical world. In this poster, we present a framework to develop a practical yet realistic mouse neuro-musculoskeletal model by using tissue-specific gene expression patterns. Taking advantage of transgenic mice expressing Scleraxis (Scx)-green fluorescent protein (GFP) specifically in connective tissues including tendons, we reconstructed three modal (3M) data: i.e., X-ray computed tomography (CT) and Scx-GFP data from the same individual and a mouse biomechanical skeletal model. We mapped the X-ray CT and Scx-GFP data sets to the pre-developed mouse skeletal model by converting the multimodal coordinate systems to local coordinate systems of the skeletal model to decide musculotendon paths of Hill's muscle models. This systematic framework will reduce modeler-dependent biases and makes it possible to realize effective and accurate modeling. Our framework has the potential to acquire new insights into the brain, especially in terms of individual-environment interactions, which conventional methods would be difficult to achieve.

### [2P-056]

### Elucidation of pathological mechanism caused by human disease mutation in CaMKIIβ

*Hiroki Mutoh[1], Kazushi Aoto[1], Takehiro Miyazaki[2], Atsuo Fukuda[3], Hirotomo Saitsu[1] ([1]*Department of Biochemistry, Hamamatsu University School of Medicine,* [2]*Kyoto University,* [3]*Department of Neurophysiology, Hamamatsu University School of Medicine*)

Recently, we have identified CaMKIIα and CaMKIIβ mutations in patients with neurodevelopmental disorders by whole-exome sequencing study. Most CaMKII mutants have increased phosphorylation of Thr286/287, which induces autonomous activity of CaMKII, using cell culture experiments. In this study, we explored the pathological mechanism of motor dysfunction observed exclusively in a patient with Pro213Leu mutation in CaMKIIβ using a mouse model of the human disease. The homozygous CaMKIIβ P213L knockin mice showed age-dependent motor dysfunction and growth failure from 2 weeks after birth. In the cerebellum, the mutation did not alter the mRNA transcript level, but the CaMKIIβ protein level was dramatically decreased. Furthermore, in contrast to previous result from cell culture, Thr287 phosphorylation of CaMKIIβ was also reduced. CaMKIIβ Pro213Leu knockin mice showed similar motor dysfunction as CaMKIIβ knockout mice, newly providing evidence for a loss of function rather than a gain of function. Our disease model mouse showed similar phenotypes of the patient, except for epileptic seizures. We clearly demonstrated that the pathological mechanism is a reduction of mutant CaMKIIβ in the brain, and the physiological aspects of mutation were greatly different between *in vivo* and cell culture.

### [2P-058]

### Suppressive control of optokinetic nystagmus by the primate frontal eye field

*Yoshiko Izawa[1], Hisao Suzuki[1] ([1]*Department of Systems Neurophysiology, Graduate School of Medicine, Tokyo Medical and Dental University*)

When the eyes are fixated on a spot, fixation neurons in the frontal eye field (FEF) show an increase in activity. Our previous study suggested that fixation neurons in the FEF contribute to the suppression of saccades and smooth pursuit eye movements to maintain active fixation. The present study was performed to examine the role of the FEF in the suppressive control of reflexive eye movements in trained monkeys. We found that electrical stimulation in the FEF suppressed the quick and slow phases of optokinetic nystagmus at an intensity subthreshold for eliciting electrically evoked saccades. During optokinetic nystagmus, presentation of a stationary small spot to the eyes followed by fixation is known to suppress both the quick and slow phases of eye movements. We recorded the activity of fixation neurons in the FEF and found that fixation neurons usually showed a decrease in activity during optokinetic nystagmus and an increase in activity during the suppression of nystagmus by visual fixation. The present results show that the activity of fixation neurons in the FEF is related to the suppressive control of optokinetic nystagmus for maintaining active fixation. We may conclude that a common neuronal assembly in the FEF contributes to the suppressive control of different functional classes of eye movements.

### [2P-060]

### Connectome changes relating to motor improvement in cerebral palsy

*Taichi Goto[1,2], Tomokazu Tsurugizawa[3,4], Yuji Komaki[5], Ichiro Takashima[1,2], Sunao Iwaki[1,3], Nobuo Kunori[2] ([1]*Doctral Programs in Neuroscience, Degree Programs in Comprehensive Human Sciences, Graduate School of Comprehensive Human Sciences, University of Tsukuba, Japan.,* [2]*Integrative Neuroscience Research Group, Human Informatics Interaction Research Institute, National Institute of Advanced Industrial Science and Technology, Japan.,* [3]*Mental and Physical Functions Modeling Group, Human Informatics and Interatcion Research Institute, National Institute of Advanced Industrial Science and Technology, Japan.,* [4]*Faculty of Engineering, University of Tsukuba, Japan.,* [5]*Live Imaging Center, Central Institute for Experimental Animals, Japan.*)

Motor impairment in the children with cerebral palsy (CP) is considered a major problem in daily lives, and rehabilitation is often implemented to help them recover. Previous studies reported rehabilitation to children with CP increased neural connectivity in a whole brain, and the importance of plastic changes in neural activity in the undamaged sensorimotor cortex was pointed out in animal research using juvenile rat model of CP. Though these studies can suggest that increased neural connectivity including the sensorimotor cortex in undamaged side may be important to restore motor dysfunction in CP, this is not clear. In the present study, we investigated the changes of neural connectivity within the sensorimotor cortex after rehabilitation using juvenile animal model of CP. CP rat model was made by ligating unilateral common carotid artery and exposing to hypoxic condition on postnatal day 7 (PND7). From PND21, physical exercise using rotarod apparatus was started as a rehabilitation for three weeks. Following that, whole brain tissue was fixed by 4% paraformaldehyde for *ex vivo* diffusion tensor imaging (DTI) to estimate neural connectivity. The results of behavioral tasks showed, although the motor impairment was observed in CP rat model before the training, motor performance was improved by the physical exercise. In the DTI tractography, neural connectivity between the M1, M2 and S1 of undamaged side was increased and changes of white matter structures were also estimated in the connection between M1 and M2 of undamaged side. The present results suggest that the changes of neural connectivity including the sensorimotor cortex in undamaged side can be important to improve the motor recovery in CP.

### [2P-057]

### Functional mapping and anatomical tracing of the saccade related region in dorsal frontal cortex of common marmoset

*Chih-Yang Chen[1], Akiya Watakabe[2], Denis Matrov[3], Kuan-Ting Ho[4], Wajd Amly[1], Hirotaka Onoe[5], Tetsuo Yamamori[2], Tadashi Isa[1,4,5] ([1]*Evolutionary Systems Neuroscience Group, Institute for the Advanced Study of Human Biology (ASHBi), Kyoto University, Kyoto, Japan,* [2]*Laboratory for Molecular Analysis of Higher Brain Function, Center for Brain Science, RIKEN, Saitama, Japan,* [3]*Section on Behavioral Neuroscience, National Institute of Mental Health, National Institutes of Health, Bethesda, Maryland, U.S.A.,* [4]*Division of Physiology and Neurobiology, Department of Neuroscience, Graduate School of Medicine, Kyoto University, Kyoto, Japan,* [5]*Human Brain Research Center, Graduate School of Medicine, Kyoto University, Kyoto, Japan*)

The dorsal frontal cortex in primate contains several regions that are both involved in saccade generation and higher cognitive functions, such as attention and decision making. However, these areas are not fully identified in common marmoset. In the current study, we aimed to use either electrical microstimulation or viral tracer to identify these regions. We applied electrical microstimulations (biphasic current at 250 Hz for 30 trains) in the dorsal prefrontal cortex and premotor areas while two marmosets performing gap saccade task to identify the saccade evoking regions. In separate animals, we injected viral tracers with TET amplification (AAV1-Thy1S-tTA mixed with either AAV1-TRE-clover or AAV1-TRE3-Vamp2-mTFP1) and analyzed how these regions projected to the brainstem saccade center, superior colliculus (SC). We successfully evoked saccades in area 45, 8aV, 8aD, 6DR, and 6Va. Anatomically, 8aV and 45 projected to the intermediate layer whereas 8aD projected to the deeper layer of SC. We also observed a systematic decrease in evoked saccade amplitude and change from upper to lower saccade direction when the stimulation location moved laterally and caudally in area 8av and 45. Similar trends could also be identified in the projection pattern of SC. The saccade evoking regions in the dorsal frontal cortex is functionally and anatomically consistent. This result will help to fill the gap of previous primate research. "COI: NO"

### [2P-059]

### Spinal reflex representation in the primary motor cortex

*Tatsuya Umeda[1,2,3], Osamu Yokoyama[4], Michiaki Suzuki[4], Miki Kaneshige[4], Tadashi Isa[1,2,5,6], Yukio Nishimura[2,4,6,7] ([1]*Kyoto University Graduate School of Medicine,* [2]*National Institute for Physiological Sciences,* [3]*National Center of Neurology and Psychiatry,* [4]*Tokyo Metropolitan Institute of Medical Science,* [5]*Institute for the Advanced Study of Human Biology (WPI-ASHBi), Kyoto University,* [6]*The Graduate University for Advanced Studies (SOKENDAI),* [7]*Japan Science and Technology Agency (JST)*)

Descending motor drive and somatosensory feedback play important roles in modulating muscle activity. We previously revealed that spinal motor neurons are subjected to temporally organized modulation by direct activation through the descending pathway and the lagged action of the spinal reflex during voluntary limb movement (Umeda et al., PNAS in press). However, it remains unknown by what mechanism descending motor drive coordinates with the spinal reflex to control the activity of spinal motor neurons for achievement of accurate limb movements. Here, we decoded muscle activity from the activity in motor cortices and afferent neurons in monkeys performing reaching movements and found that motor cortical activity affects muscle activity through not only the direct descending pathway but also through the "transafferent pathway" composed of descending and spinal reflex pathways. Selective interruption of the afferent pathway reduced the estimated transafferent component of muscle activity, providing causal evidence for the parallel information flow from motor cortices to muscles. Moreover, among the motor-related areas, the primary motor cortex (M1) encodes most information on muscle activity transmitted via the direct descending and transafferent pathways. These results suggest that M1 implements an internal model that prospectively estimates sensorimotor transformation by spinal reflex systems.

### Poster Presentation

[2P]

**Neurophysiology, Neuronal cell biology**

**Sensory function, Sensory organ**

March 15 (Wed.), 12:10 - 14:10, Poster Room

### [2P-061]

**ON and OFF starburst amacrine cells are controlled by distinct cholinergic pathways.**

*Mie Gangi[1], Takuma Maruyama[2], Toshiyuki Ishii[1], Makoto Kaneda[1] ([1]*Nippon Medical School,* [2]*Tokyo Women's Medical Univ.*)

In the retina, ACh is released only by two types (ON and OFF) of starburst amacrine cells (SACs), a key neuron for motion detection in the retina. ON and OFF SACs are considered to have the same functions, and synaptic wirings of OFF SACs are speculated based on the data obtained from ON SACs. However, recent studies demonstrated that ON and OFF SACs were different in gene expression patterns and receptors, implying the different functions of ON and OFF SACs. Here, we compared the cholinergic signaling pathways between ON and OFF SACs in the mouse retina. ACh induced GABAergic feedback to SACs in both ON and OFF SACs. However, ACh receptors involved in this feedback in adult were different in ON and OFF SACs, which were originally same in the early developmental stage. This feedback remained even in the presence of TTX in both SACs, suggesting that the ACh-induced GABAergic feedback originated from non-spiking amacrine cells. When mGluR2 receptor agonist LY354740 was used to block the reciprocal interactions between SACs, spontaneous GABAergic inputs decreased, but ACh induced GABAergic feedback remained in both SACs. These results suggest that the release of ACh from ON and OFF SACs might be regulated by different feedback mechanisms, mediated by non-spiking amacrine cells other than SACs.

### [2P-062]

**Taste information processing elucidated by optogenetic identification of the gustatory pathway**

*Shogo Soma[1], Kengo Nomura[1], Naofumi Suematsu[2], Tatsuro Murakami[1], Akiyuki Taruno[1,3] ([1]*Kyoto Prefectural University of Medicine,* [2]*University of Pittsburgh,* [3]*CREST*)

Gustation controls feeding behaviors. A gustatory experience consists of three processes: detection of chemicals by taste cells in the taste buds, cognitive process and value judgment in the brain. Intensive studies at the periphery have recently identified the cellular and molecular mechanisms of all five basic tastes. In contrast, the neural mechanisms of gustatory information processing in the central nervous system remain enigmatic. The confusion is principally due to studies based on small-scale neuronal recordings, which are prone to contamination from other sensory inputs, i.e., tactile and visceral inputs. In this study, we developed a method for identifying "true" taste-responsive brain neurons, combining optogenetic activation of the gustatory pathway and simultaneous large-scale electrophysiological neuronal recording in the brain. We measured neuronal responses to five basic tastes in multiple brain regions of the gustatory pathway of anesthetized mice. The data demonstrate that each region contains various taste-responsive cells with distinct activity patterns, suggesting the logic of multi-layered gustatory information processing.

### [2P-063]

**Optical coherent tomography reveals ultrasonic reception at the cochlear hook region in guinea pigs.**

*Kazuhiro HORII[1], Bakushi OGAWA[1,2], Noriko NAGASE[1,2], Iori MORIMOTO[1], Chikara ABE[1], Fumiaki NIN[1] ([1]*Division of Biological Principles, Department of Physiology, Graduate School of Medicine, Gifu University,* [2]*Department of Otolaryngology-Head and Neck Surgery, Graduate School of Medicine, Gifu University*)

Humans receive sound in frequency range from 20 Hz to 20 kHz. Sound higher than 20 kHz is called ultrasound, which cannot be perceived in air-conduction through the tympanic membrane. However, in bone conduction, humans can hear ultrasound. This perception is known as ultrasound hearing, though its precise mechanism remains unclear. In this study, we hypothesized that the hook region, which is located in the basal edge of the cochlear turn, plays an important role. Using optical coherence tomography (OCT), we imaged the sensory epithelium in the hook region in an anesthetized guinea pig. Compared to the epithelium in the apical of hook region, the width in the basal side was significantly narrower. In addition, we examined the vibration response to ultrasound stimulation by OCT vibrometry. Ultrasound stimulation via bone conduction induced synchronized vibrations with the stimulus frequency (frequency at 110~120 kHz). Our results suggest that sensory epithelium in the hook region mechanically receives inaudible ultrasound and contributes to ultrasound hearing.

### [2P-064]

**Role of N-linked glycosylation in mGluR6 cell surface delivery**

*Takumi Akagi[1], Atsushi Shimohata[1], Ikuo Ogiwara[1], Makoto Kaneda[1] ([1]*Nippon Medical School*)

The metabotropic glutamate receptor 6 (mGluR6) is a retina-specific G protein-coupled receptor localized exclusively at the dendritic tips of ON-bipolar cells, and contribute to visual information processing by sensing glutamate released from photoreceptors. It is well known that N-linked glycosylation of extracellular domain (ECD) of membrane proteins is critical for the intracellular trafficking and membrane targeting. Although there are four putative N-glycosylation sites within the mGluR6 ECD (Asn-Xaa-Ser/Thr: Asn 290, Asn 445, Asn 473, Asn 561), the role of glycosylation on mGluR6 is obscure. Here, to elucidate the involvement of N-glycosylation for the delivery of mGluR6 to the cell surface, we constructed the single, triple and quadruple mutants with Asn-to-Gln substitutions, and examined their expression mGluR6 in HEK293T cells. Immunoblotting showed that all the four single substituted mutants ran slightly faster than the wild-type construct, and that the quadruple mutant migrated the fastest. Immunofluorescence cytochemistry showed that the quadruple and triple substitutions severely reduced mGluR6 cell surface levels. These findings suggest that mGluR6 is N-glycosylated at four Asn residues within the ECD, and that the ECD N-glycosylation is involved in mGluR6 delivery to the surface plasma membrane.

### [2P-065]

**Optogenetic manipulation of spinal inhibitory neurons and enhancement of mechanical nociceptive responses evoked in the spinal dorsal horn**

*Yuka Fujiwara[1,2], Keisuke Koga[1], Toshiya Tachibana[2], Hidemasa Furue[1] ([1]*Department of Neurophysiology, Hyogo medical university,* [2]*Department of Orthopaedic Surgery, Hyogo medical university*)

Inhibitory interneurons in the spinal dorsal horn play a major role in regulating pain signaling. Reduction of the inhibitory synaptic transmission is thought to contribute to the development of touch-evoked pain (allodynia) that is a common symptom of neuropathic pain. However, it remains unclear how functional loss of inhibitory neuronal activity in the spinal dorsal horn modulates sensory transmission. We developed a novel method that enable us to temporarily and specifically control spinal inhibitory neuronal activity by optogenetics, and investigated the effects of specific inhibition of spinal dorsal horn inhibitory neurons on mechanical nociceptive behaviors and spinal mechanical responses evoked by cutaneous natural mechanical stimulation. Specific spinal optogenetic suppression of inhibitory neuronal activity induced mechanical hypersensitivity, and enhanced mechanical responses of wide dynamic range neurons in the spinal dorsal horn. The present results suggest that functional reduction of spinal inhibitory neuronal activity induces an excessive excitation of WDR neurons, and it may contribute to neuropathic mechanical allodynia.

### [2P-066]

**Multiple sensory mechanisms underlying 2-methylthiazoline-evoked avoidance behavior in *Drosophila***

*Shoma Sato[1,2], Takaaki Sokabe[1,2] ([1]*Thermal Biology Group, Exploratory Research Center on Life and Living Systems, National Institutes of Natural Sciences*, [2]*Division of Cell Signaling, National Institute for Physiological Sciences, National Institutes of Natural Sciences*)

Various irritant chemicals induce nocifensive behaviors and aversive responses via the activation of multiple transient receptor potential (TRP) channels and other receptors expressed in sensory neurons. We focused on 2-methylthiazoline (2MT), which has been originally identified as a volatile TRPA1 activator evoking innate fear responses in mice. We observed strong, dose-dependent avoidance against 2MT in the wild-type flies, whereas such avoidance disappeared in the *TrpA1* null mutant flies particularly at high concentrations. Mutation of *TrpA1-A*/*B* or *C*/*D* isoforms partially impaired 2MT avoidance. Specific knockdown of *TrpA1* in bitter taste neurons diminished the aversive response against 2MT. In addition, *Odorant receptor co-receptor* mutant flies, which show olfactory dysfunction, did not avoid 2MT at low concentrations. These results suggest that 2MT is detected by a combination of TRPA1 and odorant receptor(s) in *Drosophila*. Taken together, we propose a novel strategy for insect pest management aiming at insect sensory molecules as a target.

### [2P-067]

**Peripheral neurogenesis in methylmercury exposed rat dorsal root ganglion**

*Yo Shinoda[1], Marika Abe[2], Eiko Yoshida[3], Tsutomu Takahashi[1], Toshiyuki Kaji[4], Yasuyuki Fujiwara[1] ([1]*Tokyo Univ. of Pharm.and Life Sci.*, [2]*Shibuya Senior High School*, [3]*CRIEPI*, [4]*Tokyo Univ. Sci.*)

Methylmercury (MeHg) is known to cause the severe neural degeneration in the central and the peripheral nervous system, which is called Minamata disease. Recently, we reported that the hypoalgesia was selectively induced in MeHg-exposed rats, which was recovered time-dependently. In addition, the number of neurons in the dorsal root ganglion (DRG) of MeHg-exposed rats decreased during hypoalgesia, while it also recovered to control levels after behavioral recovery. In this study, we performed immunohistochemistry in order to clarify whether neurogenesis occurred overtime in MeHg-exposed DRGs. MeHg (6.7 mg/kg/day) was orally administered to Wistar rats for 5 days and discontinued for 2 days, and this cycle was done once again. BrdU (100 mg/kg/day) was administered intraperitoneally for 5 days from a week before the fixation. Twenty-eight, 42, 56 and 70 days after the beginning of MeHg exposure, rats were fixed by paraformaldehyde and their DRGs were cryosectioned and processed for immunohistochemistry. The sections were immunostained for neuronal, nuclear markers, Ki67 and BrdU antibodies. Now we are exploring the optimal experimental condition to see the neurogenesis, and hope to show the results in the conference.

### [2P-068]

**Upregulation of the hypothalamo-neurohypophysial system and activation of vasopressin neurones attenuates hyperalgesia in a neuropathic pain model rat**

*Kazuhiko Baba[1,2], Makoto Kawasaki[1], Naofumi Ikeda[1,2], Teruaki Fujitani[1], Takanori Matsuura[1], Yoshiaki Yamanaka[1], Hitoshi Suzuki[1], Makiko Shimizu[2], Mitsuhiro Yoshimura[2], Takashi Maruyama[2], Yoichi Ueta[2], Akinori Sakai[1] ([1]*Department of Orthopaedic Surgery, School of Medicine, University of Occupational and Environmental Health*, [2]*Department of Physiology, School of Medicine, University of Occupational and Environmental Health*)

Arginine vasopressin (AVP) is a hypothalamic neurosecretory hormone well known as an antidiuretic, and recently reported to be involved in pain modulation. The expression kinetics of AVP and its potential involvement in the descending pain modulation system (DPMS) in neuropathic pain (NP) remains unclear. We investigated AVP expression and its effects on mechanical and thermal nociceptive thresholds using a unilateral spinal nerve ligation (SNL) model. All rats with SNL developed NP. Intensities of enhanced green fluorescent protein (eGFP) in the supraoptic and paraventricular nuclei, median eminence, and posterior pituitary were significantly increased at 7 and 14 days post-SNL in AVP-eGFP rats. *In situ* hybridisation histochemistry revealed significantly increased *AVP* mRNA expression at 14 days post-SNL compared with the sham control group. The chemogenetic activation of AVP neurones significantly attenuated mechanical and thermal hyperalgesia with elevated plasma AVP concentration. These analgesic effects were suppressed by pre-administration with V1a receptor antagonist. AVP neurones increased the neuronal activity of serotonergic dorsal raphe, noradrenergic locus coeruleus, and inhibitory interneurones in the spinal dorsal horn. These results suggest that the hypothalamo-neurohypophysial system of AVP is upregulated in NP and activated endogenous AVP exerts analgesic effects via the V1a receptors. AVP neurones may activate the DPMS.

### [2P-069]

**Physiological function of the neuronal projection between different sensory modality**

*Mio Inoue[1], Akari Hashimoto[1], Ikuko Takeda[1,2], Daisuke Kato[1], Hiroaki Wake[1,2] ([1]*Nagoya University school of medicine*, [2]*National Institute for Physiological Sciences, Okazaki, Japan*)

### [2P-070]

**Upper limit of hearing range in bone conduction in guinea pigs.**

*Motoya Sawa[1], Moemi Sakata[1], Kazuhiro Horii[1], Bakushi Ogawa[1,2], Noriko Nagase[1,2], Iori Morimoto[1], Fumiaki Nin[1] ([1]*Division of Biological Principles, Department of Physiology, Graduate School of Medicine, Gifu University*, [2]*Department of Otolaryngology-Head and Neck Surgery, Graduate School of Medicine, Gifu University*)

### Poster Presentation

[2P]
**Molecular physiology, Cell physiology**
**Membrane transport**

March 15 (Wed.), 12:10 - 14:10, Poster Room

### [2P-071]

**Temporal shift and involvement of actin in the docking dynamics of biphasic exocytosis of glucagon-like peptide-1**

*Kazuki Harada[1], Maoko Takashima[1], Tetsuya Kitaguchi[2], Takashi Tsuboi[1] ([1]*Department of Life Sciences, Graduate School of Arts and Sciences, The University of Tokyo,* [2]*Laboratory for Chemical and Life Science, Institute of Innovative Research, Tokyo Institute of Technology*)

Glucagon-like peptide-1 (GLP-1) is an incretin hormone secreted from enteroendocrine L cells. During peptide hormone exocytosis, hormone-containing granules are docked and fused with the plasma membrane. Exocytosis can be classified into several forms based on the duration of docking, priming, and fusion. Although various proteins and second messenger molecules are known to be involved in the regulation of exocytotic forms, mechanisms regulating the proportion of those forms in the hormone exocytosis remain unclear. In this study, we used enteroendocrine L cell line GLUTag cells and visualized a single exocytosis by live-cell imaging. GLP-1 granules showed biphasic pattern of exocytosis frequency during 30 min. In the first phase, most granules were predocked with the plasma membrane before stimulation or immediately fused to the plasma membrane without docking. In the second phase, GLP-1 granules were mainly docked with the plasma membrane after stimulation and eventually fused. Inhibition of actin polymerization altered the proportion of those exocytotic forms. These results suggest that actin network and actin-interacting proteins regulate the docking dynamics of GLP-1 granules to enable the time-dependent change of exocytotic forms during the biphasic exocytosis.

### [2P-072]

**The mechanism of mitochondrial dynamics regulation via PPI**

*Sho Aki[1], Tsuyoahi Osawa[1] ([1]*Division of Integrative Nutriomics and Oncology RCAST, The University of Tokyo*)

Mitochondria have transformed from the classical concept of "homogeneous organelles responsible for energy production" to "organelles that control higher functions through metabolic communication". The balance between mitochondrial fusion and fission is a fundamental event in the life process that maintains mitochondrial morphology and function, but the mechanism of mitochondrial homeostasis (mitochondrial dynamics) remains largely unknown. We found that the Phosphatidyl Inositol Kinases, PI3K regulates mitochondrial fission and fusion via phosphoinositide (PPI) metabolism. PI3K knockdown (KD) increased excessive mitochondrial division and fragmentation, significantly reduced the function of fragmented mitochondria, and accumulation of ROS was observed. The expression levels of mitochondrial fusion/fission factor did not change in PI3K KD cells. In addition, although Mfn1 was recurited to the mitochondrial fusion site, mitochondrial fusion was inhibited, suggesting that PI3K metabolite PPI is essential for mitochondrial fusion. In addition, cardiomyocyte-specific PI3K double KO mice showed accumulation of fragmented mitochondria in cardiomyocyte and died within 1-2days after birth due to abnormal cardiac contraction. These observations ind icate that PI3K is a novel kinase that promotes mitochondrial fusion through PPI production and controls mitochondrial dynamics.

### [2P-073]

**Sodium-glucose cotransporters (SGLT) in *Ciona intestinalis* transports various kinds of sugar**

*Kazuyo Kamitori[1,2], Yuichiro Fujiwara[1,2] ([1]*Laboratory of Molecular Physiology & Biophysics, Faculty of Medicine, Kagawa University,* [2]*International Institute of Rare Sugar Research and Education, Kagawa University*)

Sodium-glucose cotransporters (SGLTs) play major roles during the sugar absorption in various living organisms. In addition, their physiological significance in diabetes, cancer, and food intake regulation have been reported. While mammals utilize several SGLTs with different sugar specificities, some other chordates or microorganisms are reported to have just one SGLT. Molecular analyses for SGLTs in various species could lead to understand the evolution of sugar metabolism and progression of sugar-related diseases. Here we report cloning and first characterization of SGLT in *Ciona intestinalis*. It showed more than 50% homology to human SGLTs. In spite of its high similarity to the human homologues especially around the sugar binding site, it transported several kinds of sugar which could not be transported by human SGLTs, with the higher binding affinity. This was contrary to the "high selectivity and low binding affinity" characteristic of mammalian SGLTs. Meanwhile, point mutation studies revealed that several common amino acids are crucial for sugar selectivity in *Ciona* and human SGLT. Present studies could be the clue to the structural basis for the SGLT sugar selectivity and evolution of sugar metabolism and sugar-related diseases.

### [2P-074]

**The non-genomic action of Vitamin $D_3$ on the sodium phosphate cotransporter family SLC34**

*JUNXIAN ZHOU[1], Okochi Yoshifumi[1], Okamura Yasushi[1] ([1]*Integrative Physiology, Graduate school of medicine, Osaka university*)

Maintaining the balance of inorganic phosphates (Pi) in the body is fundamental to normal cellular function. Three members of the SLC34 family of sodium-driven phosphate cotransporters (NaPi-IIa, IIb, and IIc) regulate Pi homeostasis in the intestine and kidney, respectively. Vitamin $D_3$ is known to promote intestinal phosphorus absorption through genomic action. In the process of analyzing the function of NaPi, we found the possibility that vitamin $D_3$ directly regulate the activity of NaPi, which is what is called a non-genomic action. We have previously established an electrophysiological method by transfecting a gene encoding NaPi-II into Neuro2A cell line. By this method, we examined the direct effects of 1,25(OH) $D_3$ (active form) and 25(OH)$D_3$ (precursor of 1,25(OH)$D_3$) on Pi-dependent sodium currents in human NaPi-IIa and IIb. Our results show that both vitamin $D_3$ have a promotion effect on the Pi-dependent sodium current of the NaPi-II. On the other hand, cholecalciferol, precursor of 25(OH)$D_3$, did not show any effect on both NaPi-II. These results suggest that 1 and 25-hydroxyl groups of Vitamin $D_3$ are involved in the promotion of NaPi-II activity.

### [2P-075]

**Is expression of the $Na^+$,$K^+$-ATPase α4 isoform testis-specfic?**

*Mizuki Katoh[1], Moeno Ogata[1], Yoshiaki Tabuchi[2], Takuto Fujii[1], Takahiro Shimizu[1], Hideki Sakai[1] ([1]*Department of Pharmaceutical Physiology, Faculty of Pharmaceutical Sciences, University of Toyama,* [2]*Life Science Research Center, University of Toyama*)

$Na^+$,$K^+$-ATPase normally contributes to maintain the resting membrane potential. The catalytic α subunit of $Na^+$,$K^+$-ATPase has four isoforms. Among them, α1 isoform is expressed ubiquitously in the plasma membrane, whereas α4 isoform has been reported to be expressed specifically in sperm of the testis. We wonder why expression of α4 isoform is believed to be restricted in male (testis) because $Na^+$,$K^+$-ATPase is one of key proteins in mammals. To explore the possibility that α4 isoform functions in the tissues other than testis, we made a specific antibody for α4 isoform and examined detailed expression in various tissues and cells. Interestingly, Western blotting using this antibody suggested that α4 isoform is expressed in the membrane fraction of brain as well as testis. Next, we examined localization of α4 isoform in the cells exogenously expressing it. Unexpectedly, the α4 isoform was not present in the plasma membrane, but in the endoplasmic reticulum. These results suggest that the $Na^+$,$K^+$-ATPase α4 isoform has novel physiological functions in the tissues other than testis.

### [2P-076]

### Roles of F-actin in intracellular insulin granule behavior analyzed by single-particle tracking

*Hiroyasu Hatakeyama[1], Shinichiro Ono[1], Tomomi Oshima[1], Noriko Takahashi[1]
([1]*Department of Physiology, Kitasato University School of Medicine*)

Mobilization of intracellular insulin granules to the juxtamembrane regions is one of the critical processes in regulating insulin secretion. We herein quantified intracellular insulin granule movement in rat INS-1 cells using single-particle tracking of insulin granule membrane proteins labeled with Quantum dot fluorescent nanocrystals and pharmacologically investigated the roles of F-actin. Destabilization of whole-cell F-actin with latrunculin B significantly facilitated insulin granule movement, whereas stabilization with cytochalasin D and jasplakinolide significantly suppressed the movement, indicating that global F-actin has suppressive roles in insulin granule movement. In addition to the global F-actin dynamics, local intracellular actin dynamics that depend on actin nucleators formin or Arp2/3 were observed. Inhibitions of either formin or Arp2/3 suppressed insulin granule movement, implying that local F-actin plays facilitative roles in insulin granule movement. Dual-color imaging of insulin granules and F-actin suggested direct modulation of insulin granule movement by local F-actin dynamics. Our findings show that F-actin plays dual regulatory roles in dynamic insulin granule movement.

### Poster Presentation

### [2P]
### Molecular physiology, Cell physiology
### Ion channels, Receptors

March 15 (Wed.), 12:10 - 14:10, Poster Room

### [2P-077]

### Cy3-based membrane protein targeting assay for quantitative evaluation of membrane expression efficiency of ion channels

*Reina Yamamoto[1], Kosuke Yamauchi[1], Satoe Takahashi[2], Aono Fukumoto[1], Mizuki Yamamoto[1], Masanori P. Takahashi[1], Kazuaki Homma[2,3], Kubota Tomoya[1]
([1]*Osaka University Graduate School of Medicine, Division of Health Sciences,* [2]*Department of Otolaryngology - Head and Neck Surgery, Feinberg School of Medicine, Northwestern University, Chicago, IL, USA,* [3]*The Hugh Knowles Center for Clinical and Basic Science in Hearing and Its Disorders, Northwestern University, Evanston, IL, USA*)

Recent progress of next generation sequencing technique has enabled us to detect large numbers of gene variants in patients with channelopathies. Evaluating the expression levels of the mutated channel proteins in cell's plasma membrane (PM) is crucial for defining the pathogenicity and pathological roles of the variants, which is an essential element for the diagnosis. In this study, we developed a Cyanine3 (Cy3)-based fluorometric assay that quantifies the PM targeting efficacies of ion channel proteins. First, we established HEK293T-based stable cell lines expressing mTurquoise2 (mTq2)-tagged ion channels of interest using the Sleeping Beauty transposon system (Kowarz et al., 2015). The cells were treated with sulfo-Cy3 NHS ester to label channel proteins at the PM, and lysed with a mild detergent-containing buffer after quenching unreacted NHS. The mTq2-tagged channels were captured using an anti-GFP-conjugated beads (GFP-selector), and the beads were imaged to determine the fluorescence intensities of Cy3 ($F_{Cy3}$) and mTq2 ($F_{mTq2}$). The ratio of $F_{Cy3}/F_{mTq2}$ represents the PM targeting efficiency. In this study, we determined the PM targeting efficiencies of Nav1.4 and Kir2.1 channels, demonstrating the feasibility and usefulness of this Cy3-based PM protein targeting assay for evaluating the membrane expression of channels.

### [2P-078]

### Structural determinants of the inhibition of M2R by Sigma-1 receptor

*CHANG LIU[1,2], I-Shan Chen[1,2,3], Ruth Murrell-Lagnado[4], Yoshihiro Kubo[1,2] ([1]*Div. Biophys and Neurobiol, NIPS, Okazaki, Japan,* [2]*Physiol. Sci, SOKENDAI, Hayama, Japan,* [3]*Dept. Pharmacol. Wakayama Med. Univ., Wakayama, Japan,* [4]*School of Life Sciences, University of Sussex, Brighton, UK*)

Sigma-1 receptor (S1R) is a chaperone protein which is relevant to various psychiatric and neurological disorders. It has been reported to colocalize with muscarinic acetylcholine receptor M2 (M2R) on the soma of motoneurons, while the functional aspects remain unknown. To clarify whether and how S1R could interact with M2R, we performed experiments and observed the results as follows: By patch-clamp recordings from transfected HEK293T cells, we observed S1R inhibits the function of M2R, but not other $G_{i/o}$ coupled receptors such as M4R. (2) By immunohistochemical staining, we observed that the expression level of M2R on the plasma membrane (PM) is not down regulated by S1R and that S1R is closely localized to M2R. (3) By co-immunoprecipitation, the interaction of M2R and S1R was confirmed. (4) By analyzing various chimeras and mutants between M2R and M4R, we identified E172 and E175 of M2R on the extracellular loop 2 region, and also the transmembrane 6 domain (TM6) are essential for the inhibition by S1R. Taken together, our data shows that S1R inhibits, not the expression on the PM, but the function of M2R via the extracellular loop 2 and TM6.

### [2P-079]

### Specific expression of HCN channels in cardiac pacemaker cells of the ascidian *Ciona*

*Yuma Fujikake[1,2], Junko Nishino[1,2], Yasunori Sasakura[3,4], Atsuo Nishino[1,2] ([1]*The United Graduate School of Agricultural Science, Iwate University,* [2]*Graduate School of Agriculture and Life Science, Hirosaki University,* [3]*Shimoda Marine Research Center, University of Tsukuba,* [4]*Faculty of Life and Environmental Sciences, University of Tsukuba*)

The heart of *Ciona*, a basal chordate model, is composed of a contractile tube called the "heart tube". The ends of the tube are called the hypobranchial and visceral ends (the H and V ends), respectively. Heart-beatings in the tube are represented by peristaltic contractile waves which are initiated from one of the ends and propagate to the other. Interestingly, the direction of the peristaltic heart-beatings periodically reverses. Although past studies revealed that heart-beatings and its periodic reversals occurred by pacemaker activities in the heart, details of the mechanism, e.g., whether the pacemaking is neurogenic or myogenic, have remained unknown. Our recent analyses on the *Ciona* revealed that two independent populations of pacemaker cells reside within 5% regions at both ends of the heart tube (called P regions). We then performed RNA-seq analyses and revealed that the *Ciona* paralogs of HCN channels, which are essential factors in the mammalian cardiac pacemaker cells, were expressed in the P regions. A previous study has shown that cell populations expressing *PC2* gene, a crucial factor to synthesize neuropeptides, reside at both ends of the heart tube with a ring-like pattern. In the present study, we examined the relationship between these *PC2*-expressing cells and the putative pacemaker cells expressing HCN channels by in situ hybridization. We confirmed that one of the *Ciona* HCN genes (*Ci-HCNc*) was specifically expressed in a cell population at the H end with a crescent-like pattern. Co-staining of *Ci-HCNc* with cardiac muscle myosin *Ci-Myh.b* or *PC2* indicated that HCN-positive putative pacemaker cells were at the end of the heart tube and faced close to the PC2-positive putative neural tissue. We are now analyzing the role of the HCN channels in the generation of heart-beating rhythms by means of genome editing. We will report its progress.

### [2P-080]

### Synergistic activation of TRPA1 by a chemical ligand and alkaline condition

*Koji Shibasaki[1], Isaki Hanamura[1] ([1]*Laboratory of Neurochemistry, Graduate School of Human Health Science, University of Nagasaki*)

It has been reported that TRPA1 is activated by various stimuli such as cold (<17°C), Allyl isothiocyanate (AITC in Wasabi and mustard oil), extracellular alkaline condition and mechanical stimulus. All TRP channels have unique properties called as synergistic effects. If we apply two different agonists, thresholds of each agonist can be effectively reduced. Thus, we can observe significant TRP channel activation by combination of two different agonists. These backgrounds indicate that TRPA1can be potentiated by weak alkaline condition. In this study, we examined the possibility by an electrophysiological and an Ca2+-imaging experiments. We used both STC-1 cells (a model of intestinal enterochromaffin cell) and mouse intestinal enterochromaffin cells. We examined the effects of extracellular alkaline condition on TRPA1 activation by AITC. Although we failed observed TRPA1 activation in weak alkaline condition, AITC-activated TRPA1 currents were significantly potentiated in the alkaline condition compared with those in normal pH (pH7.4). These results indicate that alkaline condition significantly reduces the thresholds for AITC responses.

### [2P-081]

### Trafficking regulation of voltage-gated potassium channels associated with $PI(4,5)P_2$ binding sites

*Daisuke Yoshioka[1], Yasushi Okamura[1] ([1]*Integrative Physiology, Graduate School of Medicine, Osaka University*)

The inositol phospholipid $PI(4,5)P_2$ is well known signaling molecules, and regulate a wide variety of membrane proteins such as receptors, ion channels and transporters. In particular, a kind of the voltage-gated potassium channel, KCNQ2/3, has long been investigated for its regulation by $PI(4,5)P_2$. KCNQ2/3 is expressed in neurons to regulate action potential and is also an important drug target of epilepsy. In highly polarized neurons, KCNQ2/3 asymmetrically localized to axon initial segment (AIS), and the characteristic spatial distribution of KCNQ2/3 are directly related to the neuronal excitability. In this study, to elucidate the role of $PI(4,5)P_2$ in the regulation of channel trafficking, single molecule dynamics of KCNQ3 were quantitatively analyzed in living cells using total internal reflection illumination fluorescence microscopy (TIRFM). As a result, a correlation was found between the activity and single molecule dynamics of KCNQ3 with mutations in the $PI(4,5)P_2$ binding site. Furthermore, although these mutant KCNQ3s were transported to the AIS same as the wild-type KCNQ3, their expression level on the cell surface was decreased depending on their channel activity.

### [2P-082]

### Role of ether phospholipid in temperature-sensitive TRP channel functions

*Takuto SUITO[1], Xiangmei Deng[1], Kohjiro Nagao[2], Makoto Tominaga[1,3], Takaaki Sokabe[1,3] ([1]*Division of Cell Signaling, National Institute for Physiological Sciences (NIPS),* [2]*Department of Biophysical Chemistry, Kyoto Pharmaceutical University,* [3]*Thermal biology Group, ExCELLS*)

Ether phospholipids (ePLs) are the class of phospholipids which are distributed in specific tissues or cell types. It has been known that ePLs are enriched in neurons and related to neurodegenerative diseases, however, involvement of ePLs in neural function is still unclear. In this study, we explored the role of ePLs in sensory function using a *Drosophila* model. In a temperature preference assay of *Drosophila* third instar larvae, we found that knock-out of ePL synthesis genes, alkylglycerone phosphate synthase (AGPS), and specific knock-down of AGPS in the warm sensitive Transient Receptor Potential A1(TRPA1)-expressing neurons resulted in impairment of warmth sensation. To address the functional interaction between ePL and thermoreceptor TRPA1, we established the ePL-engineered cell lines by knock-out of AGPS or supplementation with the precursor of ePL. The electrophysiological recordings of *Drosophila* TRPA1 showed the increase in the temperature thresholds for activation when ePLs were depleted. In conclusion, we identified the novel function of ePLs in the thermosensation by modulating the property of thermoreceptor protein in *Drosophila*.

### [2P-083]

### Physiological significance of the $Ca^{2+}$ influx through slow muscle-type AChR on locomotor activity of zebrafish.

*Buntaro Zempo[1], Fumihito Ono[2], Koichi Nakajo[1] ([1]*Jichi medical univ.,* [2]*Osaka Medical and Pharmaceutical Univ.*)

Skeletal muscle in vertebrates consists of slow muscle and fast muscle. Recent studies in zebrafish found that the subunit composition of AChR in slow muscle differs from that of fast muscle. While the AChR in fast muscle is composed of α, β, δ, and ε (or γ), AChR in the slow muscle is composed of only α, β, and δ. However, the physiological significance of slow muscle-type AChR has not been understood. In the present study, we compared the channel properties of slow and fast muscle-type AChRs expressed in *Xenopus* oocytes by two-electrode voltage clamp. We found that the slow muscle-type AChR shows much higher $Ca^{2+}$ permeability than the fast muscle-type one. To clarify the physiological roles of the $Ca^{2+}$ influx through the slow muscle-type AChR, we mutated Glu (E) of channel pore in the d subunit, which is considered to be a key amino acid residue for $Ca^{2+}$ permeability, to Gln (Q). We confirmed that the AChR containing the mutant d subunit lost the $Ca^{2+}$ permeability. Then, to analyze the physiological functions of the $Ca^{2+}$ permeability of slow muscle-type AChR, we generated a transgenic (Tg) zebrafish line that expresses the mutant d subunit in slow muscle. We recorded spontaneous locomotion of one-day-old larvae and found that the trunk angles during swimming in Tg were significantly smaller than the wild type. This result suggests that $Ca^{2+}$ influx through AChR plays an important role in slow muscle contraction.

### [2P-084]

### Ion-conducting property of aquaporin 6 in a contact bubble bilayer

*Takahisa Maki[1], Shigetoshi Oiki[2], Masayuki Iwamoto[1] ([1]*Dept. Mol. Neurosci., Facul. Med. Sci., Univ. Fukui,* [2]*Biomed. Imaging. Res. Ctr., Univ. Fukui*)

Aquaporins (AQPs) are a family of water channels that accelerate water transport across cell membranes. It has been proposed that a mechanism for regulating AQP function in cells is the trafficking of AQPs from intracellular vesicles to the plasma membrane. On the other hand, the intra-molecular gating machinery of a water-conducting pore remains elusive. Among AQPs, AQP6 possesses a unique property of conducting ions in addition to water. Such a property of AQP6 allows for the elucidation of gating propertyies via ionic current measurements. In this study, the purified AQP6 was reconstituted into the contact bubble bilayer (CBB), and single-AQP6 currents were recorded. AQP6 exhibited an anion-selective current at acidic pH, consistent with the previous results in the cell membrane. We found that the ion-conducting pore of AQP6 opened 100% at ±50 mV, while it closed at higher membrane potentials. We further attempted to explore the molecular determinants of acid activation of AQP6 using mutants.

### [2P-085]

### The role of LRRC8D in the regulatory volume decrease in human epithelial cells.

*Kaori Sato-Numata[1], Taro Suzuki[2], Ayako Sakai[1], Shuntaro Mori[2], Yasunobu Okada[1,3,4,5,6], Tomohiro Numata[1] ([1]*Department of Integrative Physiology, Graduate School of Medicine, Akita University,* [2]*School of Medicine, Akita University,* [3]*National Institute for Physiological Sciences,* [4]*Department of Physiology, School of Medicine, Aichi Medical University,* [5]*Department of Physiology, Kyoto Prefectural University of Medicine,* [6]*Cardiovascular Research Institute, Yokohama City University*)

Volume-sensitive outward rectifying anion channels (VSOR) are activated during cell swelling induced by hypotonic stimulation, thereby causing $Cl^-$ to flow out of the cell and promote the regulatory volume decrease (RVD). VSOR forms a hetero-hexamer composed of LRRC8A (8A) as the core molecule and its isoform(s), 8B, 8C, 8D, and/or 8E, as the sub-core molecule(s). We previously showed a predominant role of the 8A+8D combination in the VSOR current formation (2016 Pflugers Arch). Here, we investigated the role of the 8A+8D channel in the RVD process by monitoring the cross-sectional cell area (CSA) after hypotonic swelling Among human epithelial HeLa and HEK293 cells overexpressing these LRRC8 members, the cells transfected with 8A+8D were found to show the most prominent recovery of cell volume. Hypotonicity-induced whole-cell $Cl^-$ currents in -8D overexpressing cells were significantly more prominent than in control cells. Together, it is concluded that not only LRRC8A but also LRRC8D predominantly contribute to cell volume regulation after cell swelling.

### [2P-086]

### Creation of Photoswitchable Ion Channels and Measurement of Their Dynamic Structural Changes

*Yuichiro Fujiwara[1,2], Maiko Hirano[1], Kazuyo Kamitori[1,2] ([1]*Molecular Physiology & Biophysics, Faculty of Medicine, Kagawa University,* [2]*International Institute of Rare Sugar Research and Education, Kagawa University*)

In recent years, light-driven channels such as algal-derived channelrhodopsin have been studied as a tool for analyzing neural circuit function, under the title of optogenetics. Efforts are underway to find new, more user-friendly light-driven rhodopsins from prokaryotes. On the other hand, there have been attempts to artificially create light-driven channels, but it is not easy. In this study, we attempted to create light-driven ion channels by cross-linking azobenzene derivatives to the channel, which are isomerized by light irradiation. Azobenzene di-maleimide isomerizes to the cis form (5-12Å) by UV light and to the trans form (18Å) by blue visible light. Cys-introduced insect fructose receptor channel mutants were expressed in Xenopus oocytes, cross-linked with azobenzene di-maleimide, and analyzed by electrophysiology while being exposed to light. Many mutant channels that opened and closed in response to light irradiation were obtained. We also obtained channels that opened and closed by the disulfide bond of the introduced Cys themselves. The second objective of this study is to understand the open/close structure of ion channels. Based on the site information of the obtained mutants and the distance information changed by azobenzene isomerization, models of the open/closed structure of the fructose receptor channel were created. In this poster, we would like to discuss the usefulness of the new light-driven channel and the dynamic structural changes of the channel.
COI: NO

### [2P-087]

### A new gating model of $K^+$ channel based on cryo-EM structures and single-molecule dynamics measurements using X-ray

*Hirofumi Shimizu[1], Hiroko Takazaki[2], Yoshikazu Hirai[3], Takuo Yasunaga[4] ([1]*University of Fukui,* [2]*Osaka University,* [3]*Kyoto University,* [4]*Kyusyu Institute of Technology*)

Since the crystal structure of the KcsA channel was reported in 1998, some structures related to open, closed, and inactivated states have been reported, which gave insights into structural changes of the functioning $K^+$ channel. To elucidate the dynamic picture of conformational changes during gating, we used X-ray single-molecule dynamics measurements that film the structural changes using gold nanocrystals as observation probes. A nanocrystal, attached to a KcsA channel, reports the conformational changes as motions of the diffraction spot when a synchrotron X-ray is irradiated to the nanocrystal. With this method, large twisting motions were observed under acidic conditions in which the KcsA channel opens and closes. We attempted to interpret the movement by comparing it with the structures reported by X-ray crystallography; however, the reported structures could not explain the large structural changes. Therefore, here we elucidated the structures on single-particle analysis using a cryo-electron microscope (cryo-EM), which has no restrictions in solution conditions compared with those required for crystal structure analysis. The obtained structures under neutral and acidic pH conditions can explain the large twisting motions. In this presentation, we propose a new gating model based on the cryo-EM structures and the single-molecule dynamics measurements using X-ray.

### [2P-088]

### The concentration-dependent permeation modes for $Ca^{2+}$ and $Na^{+}$ in $Ca_V1.3$ L-type calcium channels

*Futoshi Toyoda[1], Yukiko Himeno[2], Wei-Guang Ding[1], Mariko Omatsu-Kanbe[1], Hiroshi Matsuura[1] (*[1]Department of Physiology, Shiga University of Medical Science, [2]Department of Bioinformatics, College of Life Sciences, Ritsumeikan University*)

It is generally believed that L-type $Ca^{2+}$ channels are highly selective for $Ca^{2+}$ and hardly permeable to $Na^{+}$ under physiological conditions. However, we have recently identified the $Ca_V1.3$ L-type $Ca^{2+}$ channel as a molecular determinant for the sustained inward $Na^{+}$ current ($I_{st}$), an important player in cardiac pacemaker activity. Here, we report the experimental and theoretical realization of the competitive permeation of $Ca^{2+}$ and $Na^{+}$ through $Ca_V1.3$ channels. In patch-clamp experiments, $Ca_V1.3$ evoked a typical L-type $Ca^{2+}$ current in the presence of external $Ca^{2+}$ at 1.8 mM, which was gradually decreased as the $[Ca^{2+}]_o^{+}$ was lowered but bottomed out even at 0.1 mM, suggesting a switch of conducting ion from $Ca^{2+}$ to $Na^{+}$. A large $Na^{+}$ current was abruptly relieved from the $Ca^{2+}$ block when the $[Ca^{2+}]_o^{+}$ was reduced to submicromolar levels. Theoretical analysis using a classical permeation model (Almers & McCleskey, J Physiol, 1984) well explained the experimental observation of the anomalous mole-fraction effect between $Ca^{2+}$ and $Na^{+}$ but predicted the presence of two types of concentration-dependent permeation modes with different $Ca^{2+}$ selectivity. Single-channel recordings and computer simulation were attempted to distinguish the pattern of $Ca^{2+}$ block kinetics for the permeation modes.

### [2P-089]

### Functional regulation and intermolecular interaction of phosphoinositides on potassium channel KcsA

*Takunari Kiya[1], Kohei Takeshita[2], Akira Kawanabe[1], Yuichiro Fujiwara[1] (*[1]Faculty of Medicine, Kagawa University, [2]RIKEN*)

Phosphoinositides (PIPns) are known to play important roles in the physiological responses in living cells and modulate functions of various membrane proteins, such as ion channels. The bacterial KcsA channel, considered as a representative model of potassium channels, has been analyzed broadly from the stand point of crystallography, in silico molecular analysis and electrophysiology. However, it has not been reported whether PIPns regulate the function of KcsA directly. In this study, we analyzed the effect of PIPns on the activity of KcsA using an electrophysiological technique with artificial membranes, contact bubble bilayer method. Here we show that a few percent content of PIPns mixed in the POPC membrane in the side of inner leaflet induced higher open probability of KcsA. We also analyzed the intermolecular binding between PIPns and KcsA using the microscale thermophoresis (Kiya.et al, 2022, *JBC*). Moreover, decreases in both open probability and direct interaction with PIPns were shown in KcsA lacking the positively charged M0 helix. These results suggest that KcsA and PIPns maintain their functional regulation and binding properties through their electrostatic interactions with each other.

### [2P-090]

### EP4 promotes mitochondrial respiration and biogenesis in oral cancer cells

*Rina Nakakaji[1,2], Masanari Umemura[1], Soichiro Ishikawa[1,2], Akane Nagasako[1], Kagemichi Nagao[1], Yuto Mizuno[1], Kohei Osawa[1,2], Yoshihiro Ishikawa[1] (*[1]Cardiovascular Research Institute, Yokohama City University Graduate School of Medicine, [2]Department of Oral and Maxillofacial Surgery, Yokohama City University Graduate School of Medicine*)

[Introduction]EP4 is one of the prostaglandin $E_2$ ($PGE_2$) receptors. We have investigated the function of EP4 in oral cancer and previously reported that EP4 promoted the cell migration via $Ca^{2+}$ signaling. Furthermore, we found that EP4 promoted the hosphorylation of calcium/calmodulin-dependent protein kinase kinase 2 (CaMKK2) and AMP-activated protein kinase (AMPK), *i.e.* cell energy regulator. Therefore, we focused on the relationship between EP4 and the mitochondrial function.[Materials and Methods]HSC-3, human tongue squamous cell carcinoma cell line was used. ONO-AE1-437 was used as EP4 agonist. To evaluate the EP4 effect in mitochondrial aspiration, the oxygen consumption rate (OCR) was measured by XF Cell Mito Stress Test using Extracellular Flux Analyzer. The mRNA transcriptions of mitochondrial biogenesis associated factors were evaluated by real-time qPCR. [Results]EP4 agonist increased the OCR value, which indicated maximal respiration 3h after the stimulation. This result indicated that EP4 promoted the mitochondrial respiration. EP4 agonist also increased the expression of mtDNA and mitochondrial transcription factor A (TFAM) 3h after the stimulation, indicating that EP4 promoted the mitochondrial biogenesis.[Conclusion]Our results suggested that EP4 was involved in the mitochondrial respiration and biogenesis in oral cancer.

### [2P-091]

### Voltage-Sensing Phosphatase (VSP) in the Trophotaenia of a Viviparous Teleost: Molecular and Functional Characterization

*Adisorn Ratanayotha[1,2], Atsuo Iida[3], Takafumi Kawai[1], Eiichi Hondo[3], Yasushi Okamura[1] (*[1]Laboratory of Integrative Physiology, Department of Physiology, Graduate School of Medicine, Osaka University, Suita, Osaka, Japan, [2]Department of Anatomy, Faculty of Medicine Siriraj Hospital, Mahidol University, Bangkoknoi, Bangkok, Thailand, [3]Department of Animal Sciences, Graduate School of Bioagricultural Sciences, Nagoya University, Nagoya, Aichi, Japan.*)

Nutrient absorption is essential for animal development. Our previous study reported that nutrient absorption in lysosome-rich enterocytes (LREs) of larval zebrafish is promoted by the voltage-sensing phosphatase (VSP), a membrane protein that regulates phosphoinositides (PIPs) homeostasis via electrical signaling in biological membranes. However, it remains unknown whether VSP function is shared by different absorptive tissues in other species. Here we present our study on VSP in the viviparous teleost *Xenotoca eiseni*, whose intra-ovarian embryos absorb nutrients from maternal ovarian fluid via trophotaenia. *X. eiseni* VSP (Xe-VSP) is expressed in trophotaenia epithelium, which is an absorptive tissue functionally similar to zebrafish LREs. Electrophysiological analysis using a heterologous expression system revealed the voltage response of Xe-VSP, with difference in voltage threshold compared to zebrafish VSP (Dr-VSP). Furthermore, while the molecular architectures of Xe-VSP and Dr-VSP are virtually identical, we found that a single amino acid variation in the S4 transmembrane helix may be responsible for the difference in voltage ranges between the two orthologs. This study emphasizes the biological variation and significance of VSP in various animal species. It also hints at the potential role of VSP in nutrient absorption in *X. eiseni* trophotaenia, along with other known endocytosis-associated molecules. (COI:No)

### Poster Presentation

[2P]
**Molecular physiology, Cell physiology**
**Others**

March 15 (Wed.), 12:10 - 14:10, Poster Room

### [2P-092]

### Microelectrode array analysis of the inhibitory effect of indole 3 propionic acid on pacemaker activity of the small intestine

*Md Sajjad Hossen[1], Naoko Iwata[1], Shinsuke Nakayama[1] ([1]*Department of Cell Physiology, Graduate School of Medicine, Nagoya University*)

Interstitial Cells of Cajal (ICC) are the pacemaker cell in the GI tract that generate basic electric rhythms for gut motility, such as peristalsis and segmentation. Indole 3 propionic acid (3-IPA) is a microbiota-derived tryptophan metabolite that is known to improve gut barrier function and contains anti-inflammatory properties. We examine the effect of 3-IPA on ICC by dialysis membrane-reinforced 8×8 microelectrode array system using musculature samples with the myenteric plexus from the mouse ileum. In the presence of secondary bile acid ursodeoxycholic acid (UDCA) and L-type calcium channel blocker nifedipine (2μM), 3mM 3-IPA inhibited the pacemaker frequency from 25.79cpm to 15.49cpm. In addition, the frequency of micro-coordination patterns changed significantly: expanding and migrating activities were 50.26% and 47.12% in the control and 70.87%, and 21.84% in 3-IPA, respectively. Pretreatment of TTX (1μM), a potent neuronal blocker did not alter this effect, indicating the action of 3-IPA solely on ICC. 5-HT (100μM) did not restore these 3-IPA mediated changes in pacemaker activity. Our data suggest a possible role of commensal bacteria, e.g., C. *sporogenes* in the reciprocal regulation of ICC pacemaker activity via tryptophan metabolites.

### [2P-093]

### Membrane potential modulates ERK activity

*Mari Sasaki[1], Masanobu Nakahara[1], Takuya Hashiguchi[1], Fumihito Ono[1] ([1]*Osaka Medical and Pharmaceutical University*)

While the knowledge about physiological function of membrane potential in non-excitable cells is limited, membrane potential has been linked to cell survival and proliferation. In particular, there is no evidence that directly connects membrane potential and proliferative signal such as ERK. Here we show ERK activity is regulated by membrane potential. We monitored ERK activity using a FRET probe EKAREV while the membrane potential was changing. The ERK activity was upregulated when the membrane was depolarized. The upstream MAPK cascade was also upregulated with membrane depolarization. The voltage-dependent ERK activation was diminished by depletion of phosphatidylserine, suggesting phosphatidylserine dynamics was involved in the voltage-dependent ERK activation. COI:NO

### [2P-094]

### Effect of cesium on actin elongation might affect cell migration in NIH/3T3 cells

*Daisuke Kobayashi[1], Natsumi Nishimura[1], Akihiro Hazama[1] ([1]*Dept. Cellular and Integrative Physiology, Sch. Medicine, Fukushima Medical University*)

We previously showed the effects of cesium (Cs) on murine fibroblast cells (NIH/3T3) proliferation and migration. The treatment of Cs inhibited the migration of fibroblast cells compared with the control and the migration inhibition showed a dose-dependent manner; however, it is unclear how cell migration was affected by Cs. We assume that one of the targets of Cs is actin filaments, which turn over constantly to remodel cell shape and migration. In this study, cytoplasmic filamentous actin (F-actin) and globular actin (G-actin) was detected by immunostaining. NIH/3T3 cells were treated with cytochalasin D, which was inhibitor of actin polymerization, then cytoplasmic F- and G-actin were detected after washout. Releasing from cytochalasin D blocking, F-actin localization ratio decreased in Cs-treatment compared with control treatment. On the other hand, G-actin localization ratio increased in Cs-treatment. The results indicated Cs inhibited actin polymerization step (G-actin -> F-actin) or facilitated actin depolymerization step (F-actin -> G-actin). (COI: NO)

### [2P-095]

### Acetylcholine induced suppression of $Ca^{2+}$ signaling in pancreatic β-cells.

*Isamu Taiko[1], Kazunori Kanemaru[1], Masamitsu Iino[1] ([1]*Nihon university school of medicine department of physiology*)

Intracellular $Ca^{2+}$ signal plays an essential role in insulin secretion from pancreatic β-cells. Recent reports suggest that $Ca^{2+}$ release from the endoplasmic reticulum (ER) through cholinergic receptor stimulation mediated by parasympathetic nerves contributes to $Ca^{2+}$ signals in β-cells. However, how the $Ca^{2+}$ release from the ER shapes the intracellular $Ca^{2+}$ signal remains elusive due to limitations in the methods for direct visualization analysis. We recently developed transgenic mouse lines expressing a genetically encoded cytosolic $Ca^{2+}$ indicator, YC-Nano50, or an ER $Ca^{2+}$ indicator, CEPIA specifically in β-cells. We successfully observed periodic oscillations of both cytosolic and ER $Ca^{2+}$ signals evoked by high glucose in isolated pancreatic islets. We also confirmed an acetylcholine induced decrease in ER $Ca^{2+}$. Surprisingly, during a high glucose condition, short-term cholinergic agonist application induced a transient suppression of cytosolic $Ca^{2+}$ level to the extent comparable with the resting level, despite the release of $Ca^{2+}$ from the ER. Our results suggest that parasympathetic nerves mediate suppressive regulation of $Ca^{2+}$ signaling. Further analysis is required to reveal the physiological roles and underlying mechanisms of this unexpected $Ca^{2+}$ suppression.

### [2P-096]

### The effects of Transforming growth factor-β1 and bone morphogenetic protein-2 on differentiation into mature ependymal cells.

*Takuya Hirao[1], Beak Gyu Kim[1], Hinako Habuchi[1], Kotoku Kawaguchi[1], Shinji Asano[1], Takashi Nakahari[2] ([1]*Department of Molecular Physiology, Ritsumeikan University,* [2]*Research Organization of Science and Technology, Ritsumeikan University*)

Background: Multiciliated ependymal cells (MCCs) lining the ventricular surface have essential roles in cerebrospinal fluid flow. In the primary culture system, it has been identified that fetal bovine serum (FBS) inhibits the differentiation of MCCs. In this study, we identified inhibitory factors involved in differentiation from undifferentiated glial cells to mature MCCs. Methods: Cells prepared from the whole brain of a newborn mouse were cultured on Transwell permeable support filter. Test cytokines and inhibitors were added to the upper chamber (ventricle side). Cilia were visualized with an anti-AcTub antibody, and ciliary movement was observed under a microscope. Results: FBS inhibited the differentiation into MCCs as reported previously. We newly found that transforming growth factor-b1 (TGF-b1) and bone morphogenetic protein-2 (BMP-2) inhibited the differentiation into mature MCCs with beating cilia. The inhibition was suppressed by the treatment TGF-b1 and BMP-2 inhibitors, respectively.

### [2P-097]

### Effects of PAI-1 knock-out in vascular endothelial cells on their motilities

*HIDETO SANO[1], MASAHIKO ITO[2], TETSURO SUZUKI[2], TETSUMEI URANO[1,3], YUKO SUZUKI[1] ([1]*Dept of Medical Physiology, Hamamatsu University School of Medicine,* [2]*Dept of Microbiolgy & immunity, Hamamatsu University School of Medicine,* [3]*Shizuoka Graduate University of Public Health*)

The plasminogen activation system plays important roles in various pathophysiological processes such as vascular- and tissue-remodeling. Plasminogen Activator inhibitor-1 (PAI-1) is the principal regulator of plasminogen activation system. We have reported PAI-1 deficient patients who had lethal bleeding also showed delayed wound healing. In this study, we aimed to evaluate the accurate PAI-1 functions in human vascular remodeling in pathophysiological phases such as wound healing. To observe PAI-1 function in endothelial cells, PAI-1 deficient human endothelial cell lines (PAIKO-ECs) were generated by using CRISPR/Cas9. The plasmin generations on the surface of the PAIKO-ECs were strongly enhanced compared to WT-ECs. PAIKO-ECs were also subjected to cell migration scratch assay. PAIKO-ECs showed lower cell invasion to the scratched area, and the cells in the confluent area started to independently move in a disorganized way, which were reversed by a plasmin inhibitor. Disorganized movements were the unique characteristics of PAIKO-ECs, and seemed caused by less tight attachment to the neighboring cells. PAI-1 appeared to play critical role for the regulation of cell motility.

### [2P-098]

### Identification of pH-inducible transcription factor that normalizes intracellular pH levels

*Koya Nagata[1], Ryoko Kagami[1], Yasuo Mori[1], Nobuaki Takahashi[1] ([1]*Department of Synthetic Chemistry and Biological Chemistry, Graduate School of Engineering, Kyoto University*)

All organisms are exposed to various cellular stresses, such as oxidative stress, hypoxic stress, and pH stress. It is therefore essential for organisms to sense stresses and induce defense programs. Stress defense mechanisms for protection against oxidative and hypoxic stress are becoming increasingly clear, owing to the identification of the oxidant-defense transcription factor NRF2 and the hypoxia-inducible transcription factor HIF. Yet, little is known about the molecular mechanisms underlying acid adaptation even though cells are susceptible to acidic stress due to the continuous production of protons by glycolysis. Here, we provide evidence that most cells intrinsically express the program that induces the expression of acid extruders, which extrude proton from the inside to the outside of the cell, in response to acidic stress, strongly suggesting the existence of acid-inducible transcription factors. To identify acid-inducible transcription factors, we generated various mutant cell lines, each of which has 400 bp deletion in the specific region of 5' flanking sequence for acid extruder genes, and determined the enhancer sequence responsible for the induction of acid extruders by acidic stress. We are currently carrying out comprehensive analyses for the proteins that bind to the enhancer sequence by the proteomics approach and will hopefully identify acid-inducible transcription factors in the near future.

### [2P-099]

### Decreased intracellular $Cl^-$ enhances cell migration and invasion via activation of the Ras-ERK signaling pathway in human prostate cancer cell line, DU145

*Hiroaki Miyazaki[1], Nakano Koya[1], Sato Junichi[1] ([1]*Department of Life Science, Faculty of Science and Engineering, Setsunan University*)

Our previous studies indicated that reducing the intracellular $Cl^-$ concentration of the prostate cancer cell line DU145 facilitates cell migration and invasion, but its mechanism remains unclear. Therefore, we investigated the mechanism of enhancing cell migration and invasion of DU145 cells in the low $Cl^-$ condition. Since the previous studies suggested that ERK/MAPK signaling has been involved in cell migration and invasion in several types of cancer, we first examined the effect of $Cl^-$ on the activation of ERK. In the low $Cl^-$ condition, phosphorylation levels of ERK were transiently upregulated. The inhibition of ERK activation by the application of MEK inhibitor, U0126, completely abolished the enhancement of cell migration and invasion in the low $Cl^-$ condition. From these results, we concluded that the enhancement of cell migration and invasion of DU145 cells in the low $Cl^-$ condition is due to the activation of ERK. We subsequently tested whether the increased levels of ERK phosphorylation in the low $Cl^-$ environment was accompanied by activation of its upstream regulator, Ras small GTPase. The enhancement of ERK phosphorylation level in the low $Cl^-$ condition was significantly inhibited by the application of Ras specific inhibitor, Lonafanib. These results suggest that the intracellular $Cl^-$ may regulate the activation of ERK by affecting Ras activity. We are continuing to confirm the effect of $Cl^-$ on Ras activity directly.

### [2P-100]

### Analysis of the regulatory mechanisms of migration and invasive potential of the human prostate-derived cancer cell line DU145 via the ERK1/2 signal cascade mediated by intracellular $Cl^-$

*Shiko Tsujimoto[1], Hiroaki Miyazaki[1] ([1]*Dept. Life Sci, Fac Sci Eng, Setsunan Univ.*)

Infinite proliferation and metastatic potential are known to be characteristic features of cancer cells, and suppressing these capacities is important for cancer therapeutics. In our previous study, lowering the intracellular $Cl^-$ concentration in the human prostate-derived cancer cell line, DU145, enhanced their migratory and invasive capacities. Since the previous studies suggested that ERK/MAPK signaling has been involved in cell migration and invasion in several types of cancer, we examined the effect of $Cl^-$ on the activation of ERK1/2. Phosphorylation of ERK1/2 increased rapidly and then decreased in DU145 cells shortly after the treatment of low $Cl^-$ medium. In other words, transient phosphorylation of ERK1/2 was observed. The inhibition of this transient ERK1/2 activation by the application of MEK inhibitor, U0126, completely abolished the enhancement of cell migration and invasion in the low $Cl^-$ condition. From these results, the enhancement of cell migration and invasion of DU145 cells in the low $Cl^-$ condition is due to the activation of ERK1/2. We are currently focusing on the possibility that DUSPs, phosphatases of ERK1/2, are involved in this transient phosphorylation of ERK1/2 and the enhancement of cell motility in the low $Cl^-$ condition. We are investigating the changes in mRNA levels of dual specific phosphatases (DUSPs) after treatment low $Cl^-$ media by using RT-PCR and the effects of inhibition of DUSPs on the cell motility.

### [2P-101]

### Effects of intracellular $Cl^-$ concentration on mitochondrial activities in human breast cancer MCF-7 cells.

*Haruka Hatsumura[1], Hiroaki Miyazaki[1] ([1]*Dept. Life Sci, Fac Sci Eng, Setsunan Univ.*)

The cancer microenvironment is known to influence the proliferative and metastatic potential of cancer cells, which are abnormal characteristic features of cancer cells. Although mitochondrial dysfunction is often observed in the cancer microenvironment, its mechanism has been largely unknown. The intracellular $Cl^-$ concentration ($[Cl^-]_i$) is assumed to change significantly in the cancer microenvironment, and the changes in the $[Cl^-]_i$ may affect the properties of cancer cells. In this experiment, we analyzed the effects of $[Cl^-]_i$ on mitochondria activities by using human breast cancer MCF-7 cells. At first, we cultured MCF-7 cells in normal or low $Cl^-$ medium ($Cl^-$ is replaced by $NO_3^-$) and evaluated mitochondrial activity by measuring intracellular NADH production by the WST assay. The results showed that a decrease in $[Cl^-]_i$ significantly decreased intracellular NADH production. Thus, a decrease in the $[Cl^-]_i$ was expected to decrease mitochondrial activity. Our results strongly suggest that changes of $[Cl^-]_i$ would play important roles in mitochondrial activities in tumor cells. We continue to measure the membrane potential of the mitochondrial inner membrane in MCF-7 cells by using MT-1 dye to confirm the effect of intracellular $Cl^-$ on mitochondrial activity more directly.

### Poster Presentation

[2P]
**Embryology, Regenerative Medicine, Development, Growth, Aging**

March 15 (Wed.), 12:10 - 14:10, Poster Room

### [2P-102]

**Regulation of aging by balancing mitochondrial function and antioxidant levels.**

*Sawako Yoshina[1], Luna Izuhara[1], Naoyuki Kamatani[2], Shohei Mitani[1] ([1]*TWMU School of Medicine*, [2]*StaGen Co., Ltd*)

Aging is the deterioration of physiological mechanisms that is associated with getting old. Aging is closely related to age-related diseases and human beings' death. The accumulation of DNA damage, mitochondrial damage, and damaged proteins are believed to cause aging. In *C. elegans*, it was reported that inhibiting the electron transfer system decreases ATP production and extends lifespan and that suppressing oxygen consumption extends lifespan. However, it has also been suggested that energy loss due to mitochondrial dysfunction or impairment is a mechanism of diseases and aging. Thus, there is an unresolved relationship between ATP levels and aging. To address this issue, we administered febuxostat (FBX), an inhibitor of human xanthine oxidase (XO)/xanthine dehydrogenase (XDH), to *C. elegans*. We showed that FBX protects mitochondria and prevents age-related muscle deterioration in *C. elegans*. In addition, we showed that FBX administration could increase ATP levels without overloading the mitochondria while extending the lifespan. We also showed that the combination of FBX and an antioxidant as a protection against ROS prolongs lifespan more. Furthermore, we found that FBX has potential as a therapeutic or preventive agent for Alzheimer's disease and Parkinson's disease.

### [2P-103]

**Transcriptome analyses of mouse cardiac myocytes and cardiac non-myocyte cells: postmitotic vs. proliferative cells**

*Yasuhiro Takenaka[1,3], Masataka Hirasaki[2], Ikuo Inoue[3], Masaaki Ikeda[4], Hisayuki Ohata[5], Yoshihiko Kakinuma[1] ([1]*Department of Physiology, Nippon Medical School*, [2]*Department of Clinical Cancer Genomics, International Medical Center, Saitama Medical University*, [3]*Department of Diabetes and Endocrinology, Saitama Medical University*, [4]*Department of Physiology, Saitama Medical University*, [5]*Division of Laboratory Animal Science, Nippon Medical School*)

Cellular senescence is basically defined as the limited replicative potential of normal diploid cells. Therefore, studies of cellular senescence have been predominantly attempted to elucidate the molecular mechanism of irreversible cell cycle arrest and senescence-related phenomenon in proliferating cells. However, many tissues in mammals such as brain, retina, and heart are largely composed of postmitotic terminally differentiated cells and limited number of proliferative cells. Recent reports suggest that postmitotic cells also show phenotypic characteristics of cellular senescence, and play important roles in organismal aging or contribute to pathology. Adult heart mostly contains postmitotic cardiac myocyte and cardiac non-myocyte cells that have proliferative potential. Here, we isolated cardiac myocyte and cardiac non-myocyte cells from young and aged mouse heart, and performed transcriptomic analyses by RNA-sequence to understand the differences of gene expression in postmitotic and proliferative cells. Our results revealed that genes associated with positive regulation of transcription were notably upregulated, whereas mitochondria-related genes were downregulated in aged cardiac myocyte.

### [2P-104]

**Regulation of cortical actin dynamics and cytoplasmic flow by intracellular $Ca^{2+}$ in mouse oocytes**

*Hideki Shirakawa[1], Ryo Yonekura[1], Kento Kondo[1] ([1]*The University of Electro-Communications*)

In fertilized mammalian oocytes, actin filaments (F-actin) localized in the cell cortex are critically involved in the events on the plasma membrane, such as sperm incorporation and cortical reaction. We investigated cortical F-actin dynamics accompanying directional cytoplasmic flow in mouse oocytes and their changes correlated with the increase in intracellular $Ca^{2+}$ concentration during oocyte activation. In unfertilized eggs at metaphase of the second meiosis, steady movement of cortical actin from the animal pole towards the equator generated the cortical cytoplasmic flow in the same direction. The movements of cortical F-actin and cytoplasm were reversed, when the oocyte was activated and resumed the cell cycle after a few transient $Ca^{2+}$ increases of PLCζ-induced $Ca^{2+}$ oscillations. Experiments with inhibitors indicated that forward movements at the resting state are driven by actin polymerization mediated by Arp2/3, and reversed movements require myosin II and Rho kinase. The cytoplasmic flow was also dependent on dynamics of fine F-actin network that is spread out in deeper cytoplasm and is maintained by formin activities. Furthermore, it was suggested that Src kinase regulates the interaction between cortical layer and cytoplasmic network of F-actin. In the presentation, the results obtained from the observations of local dynamics of cortical F-actin around the sperm attachment site in fertilized oocytes will be discussed.

### [2P-105]

**Mechanism of stemness maintenance in human amniotic epithelial cells**

*Chika Takano[1], Millei Kaga[2], Isamu Taiko[2], Toshi Miki[2] ([1]*Department of Pathology and Microbiology, Nihon University School of Medicine*, [2]*Department of Physiology, Nihon University School of Medicine*)

Introduction: The human amniotic epithelial cell (hAEC), a type of placental stem cell, has been investigated as a new source of regenerative therapy. We previously demonstrated that the hAECs underwent TGF-β-dependent epithelial-mesenchymal transition (EMT) shortly after starting cell culture. The inhibition of the EMT using the TGF-β pathway inhibitor, SB-431542, maintained the expression of stemness-related genes in hAECs. Comprehensive transcriptome analysis identified a differentially expressed gene, Traf2- and Nck-interacting kinase (*TNIK*) significantly enriched in SB-431542-treated hAECs. TNIK is a member of the germinal center kinase family and is known as an important activator of the Wnt pathway. In this study, we explored the role of TNIK in hAECs to identify the missing link between TGF-β inhibition and stemness. Methods: hAECs were isolated from the placentae of 6 patients who underwent scheduled Caesarean sections. The cells were cultured for 7 days with or without SB-431542. Total RNA was extracted on day 0 (naïve cell), day 1, 4, and 7, and then the expressions of *TNIK* were analyzed by RT-qPCR. We cultured the hAECs with supplementation of a TNIK inhibitor, NCB-0846, which binds to TNIK in an inactive conformation and inhibits the phosphorylation of TCF4, and examined the cell proliferation. Results: We confirmed that TNIK was significantly expressed in cultured hAECs with SB-431542 for 7 days by RT-qPCR. *TNIK* was not observed in naïve hAECs but gradually expressed in inhibited-EMT hAECs over days. The supplementation of NCB-0846 influenced cell viability and proliferation. Conclusion: Our data showed that the *TNIK* expression in hAECs was upregulated by the inhibition of TGF-β-dependent EMT. The blocking TNIK/TCF4 interaction using NCB-0846 interfered with cell proliferation. Further study is needed to clarify the crosstalk between TGF-β and Wnt pathways, which might be associated with the regulation of an unknown switching mechanism of Wnt/β-catenin signaling in placental stem cells. Understanding these signaling pathways might be useful to develop a clinical protocol for cell transplantation therapy using hAECs.

### [2P-106]

**Directed differentiation of human amnion epithelial cells to type II alveolar epithelial cells**

*Masayuki Nomoto[1], Isamu Taiko[1], Chika Takano[1,2], Kazunori Kanemaru[1], Toshio Miki[1] ([1]*Division of Biomedical Sciences, Department of Physiology, Nihon University School of Medicine*, [2]*Division of Microbiology, Department of Pathology and Microbiology, Nihon University School of Medicine*)

The alveolar wall of the lung is composed of type I and type II alveolar epithelial cells (AT-I and AT-II), where AT-I is involved in gas exchange and AT-II in the production of surfactant to prevent alveolar collapse. Dysfunction of AT-II cells causes respiratory diseases such as chronic obstructive pulmonary disease and interstitial pneumonia. It is suggested that these intractable respiratory diseases can be treated by transplantation of healthy AT-II cells. However, the limited availability of healthy donor AT-II cells prevents to provide the therapeutic option for the patients. Human amniotic epithelial cells (hAECs) are a type of placental stem cell that possesses pluripotent stem cell-like differentiation potential as well as have a number of advantages for clinical applications. In this study, we aimed to develop a method to derive AT-II cells from the hAECs. We utilized a direct programming approach by introducing NKX2.1, a master regulator gene of AT-II cells. After 2 weeks of induction, the upregulation of AT-II cell-specific marker gene, SPC, was confirmed by RT-qPCR. Interestingly, the exogenous NKX2.1 induction consequently induced the expression of endogenous NKX2.1, which indicated the existence of a self-activation mechanism in the differentiation pathway. Comprehensive gene expression analysis revealed that the majority of alveolar-associated genes were highly expressed in the NKX2.1-induced cells. Our study showed the differentiation potential of hAECs into the AT-II cells, which may lead to the development of a novel cell therapy for intractable respiratory diseases.

### [2P-107]

**Macrophages derived from Nkx2-5-dependent endocardial hematopoiesis regulate mitral valve remodeling**

*Amane Tada[1], Norika Liu[1,2], Susumu Minamisawa[1], Atushi Nakano[1,2] ([1]*The Jikei University School of Medicine*, [2]*University of California, Los Angels*)

### Poster Presentation

[2P]
**Muscle**

March 15 (Wed.), 12:10 - 14:10, Poster Room

### [2P-108]

**Effects of spermidine administration on hypertrophy response of skeletal muscle in mice**

*Ryoto Iwai[1], Tomohiro Iwata[1], Kazuki Uemichi[1], Riku Tanimura[1], Takanaga Shirai[2], Tohru Takemasa[2] (*[1]Graduate School of Comprehensive Human Sciences, University of Tsukuba, [2]Faculty of Health and Sports Sciences, University of Tsukuba*)

Skeletal muscle mass is regulated by the balance between protein synthesis and degradation, and it increases when synthesis exceeds degradation. Resistance exercise/mechanical overload stimulates mTOR, a master regulator of skeletal muscle mass, and induces muscle hypertrophy. On the other hand, skeletal muscle mass, autophagy deficiency induces muscle atrophy by accumulating waste substances in cells. It is also known that autophagy is suppressed and intracellular clearance is impaired during skeletal muscle hypertrophy. Therefore, in this study, we applied spermidine, which promotes autophagy in an mTOR signaling pathway independent manner.The purpose of this study was to examine whether spermidine stimulates skeletal muscle hypertrophy response induced by synergists ablation. We administered 50 mg/kg/day of spermidine to mice for 14-day mechanical overload model.Spermidine administration to the mechanical overload model did not increase plantaris muscle wet weight, compared with those in mice with synergist ablation alone. Spermidine tended to promote autophagy by increasing LC3-II expression without suppressing the expression of protein synthesis-related signals, p70S6K and S6. Spermidine also tended to decrease the expression of p62, a marker of waste substances.In conclusion, although spermidine tended to promote autophagy but did not stimulate muscle hypertrophy response.

### [2P-109]

**TRPV2 of muscle satellite cells is crucial for the muscle regeneration**

*Yanzhu Chen[1], YUBING DONG[1], Kimiaki Katanosaka[2], So-Ichiro Fukada[3], Keiji Naruse[1], Yuki Katanosaka[1] (*[1]Department of Cardiovascular Physiology, Graduate School of Medicine, Dentistry and Pharmaceutical Sciences, Okayama University, [2]College of Life and Health Sciences, Chubu University, [3]Graduate School of Pharmaceutical Sciences, Osaka University*)

Aims: It is known that $Ca^{2+}$ signaling of muscle satellite cells (MuSCs) plays an important role in muscle regeneration and formation, but the detailed molecular mechanism is not clear. Among the various $Ca^{2+}$ channels expressed in myocytes and satellite cells, this study investigated the role of transient receptor potential vanilloid family type 2 (TRPV2) channel on the myogenesis. Methods and Results: Detailed analysis of TRPV2 expression in muscle tissue and isolated muscle fibers revealed that TRPV2 is strongly expressed in specific fast-twitch muscle fibers and in MuSCs during muscle regeneration. Therefore, we generated MuSCs-specific *TRPV2*-deficient mice and analyzed muscle regeneration from muscle injury. These *TRPV2*-deficient mice had significantly fewer MuSCs at the site of injury, and their ability to regenerate muscle after muscle injury was severely impaired. Elimination of *TRPV2* from the MuSCs resulted in a significant reduction in the number of *Pax7*-positive cells. In cultures of muscle fibers isolated from these *TRPV2*-deficient mice, the proliferation of MuSCs showing Pax-7 positivity was also markedly reduced. Conclusions: These results suggest that TRPV2 in MuSCs is an essential molecule for muscle regeneration. In particular, the expression of TRPV2 in muscle satellite cells may regulate Pax7 expression, and the mechanism should be analyzed in detail in the future.

### [2P-110]

**A useful zebrafish model for cisplatin-treated muscle atrophy**

Rei Ito[1], Chiharu Tomono[1], Haruka Hara[1], Mami Nakayashiki[2], *Genri Kawahara[2], Yukiko Hayashi[2] (*[1]Tokyo Medical University, [2]Department of Pathophysiology, Tokyo Medical University*)

Cisplatin is an anti-cancer drug used for a number of different types of cancers, but severe side effects are problems including skeletal muscle atrophy. Muscle RING-finger protein-1 (MuRF1) is a muscle-specific ubiquitin ligase and one of a marker molecule of muscle atrophy. To understand the molecular mechanism of muscle atrophy caused by cisplatin treatment, we used *murf1*:EGFP transgenic zebrafish and analyzed the expression of the *murf1* gene in cisplatin-treated larva and adult zebrafish. The expression analysis of *murf1* and EGFP showed that treatment with cisplatin at 4 day post-fertilization larva for 24 hours increased*murf1* expression associated with muscle atrophy. In skeletal muscle of adult zebrafish, treatment with cisplatin for 24 hours also induced up-regulation of *murf1* expression. Previously, we identified five candidate drugs to reduce the expression of zebrafish *murf1* using the *murf1*:EGFP zebrafish among a total of 1,280 drugs in a drug library. Co-treatment with one of the five candidate drugs can reduced the expression of *murf1* in both larva and adult cisplatin-treated fish and improved muscle atrophy in zebrafish larva. Our results indicated that zebrafish will be a useful animal model to analyze mechanisms of muscle atrophy induced by anti-cancer drugs.

### [2P-111]

**Microscopic heat pulses induce activation of cardiac and skeletal thin filaments in the absence of $Ca^{2+}$**

*Shuya Ishii[1], Kotaro Oyama[1], Fuyu Kobirumaki-Shimozawa[2], Nakanishi Tomohiro[2], Shin'ichi Ishiwata[3], Norio Fukuda[2] (*[1]QST, [2]Dept Cell Physiol, Sch Med, Jikei Univ, [3]Fac Sci Engn, Waseda Univ*)

In the present study, we investigated the effects of increased temperature on the thin filament regulatory function of cardiac and skeletal muscles. By taking advantage of the *in vitro* motility assay and using focused infrared (IR) laser irradiation, we analyzed the effects of rapid heating (up to 40°C) on the sliding of thin filaments composed of rabbit fast skeletal actin, and either human α-tropomyosin (Tm) and bovine ventricular troponin (Tn) or rabbit fast skeletal Tm-Tn complex. In the presence of $Ca^{2+}$ (pCa 5), heating from room temperature (23°C) monotonically increased the sliding velocity of cardiac and fast skeletal thin filaments, coupled presumably with acceleration of actomyosin ATPase. In the absence of $Ca^{2+}$ (pCa 9), cardiac thin filaments did not move at room temperature; however, IR laser irradiation elicited sliding with a $Q_{10}$ of 3.6. Likewise, fast skeletal thin filaments slid upon IR laser irradiation with a $Q_{10}$ of 8.5, showing higher sensitivity compared to cardiac thin filaments. It is important that at pCa 9, moderate sliding was observed for both types of thin filaments at 37°C. These findings suggest that 1) the "*on-off*" equilibrium of striated muscle thin filaments is partially shifted toward the "*on*" state under the relaxing condition at the physiological temperature, enabling rapid and efficient contraction in response to $Ca^{2+}$ during activation, and 2) the higher temperature dependency of fast skeletal thin filaments is optimized for the muscle's physiological properties *in vivo*.

### [2P-112]

**Assessment of myofibrillogenesis in rat embryonic hearts in the initiation of heartbeats by transmission electron microscopy and evaluation of its molecular expression patterns by proteomic analysis.**

*Nobutoshi Ichise[1], Tatsuya Sato[1], Takuro Karaushi[1], Hiroyori Fsagawa[1], Taiki Kudo[1], Noritsugu Tohse[1] (*[1]Department of cellular physiology and signal transduction, Sapporo Medical University*)

Backgrounds: The heart is the first functioning organ in embryo. We found that heart primordium begins to contract at embryonic day (E) 9.99 to 10.13; however, it remains unclear how myofibrils get formed in early embryonic heart. Methods: Heart primordium at E10.0 before and after initiation of heartbeats and primordial heart tube at E11.0 were isolated from rat embryos. Samples were subjected to transmission electron microscopy and data-independent acquisition mass spectrometry (DIA-MS). Results: The bundles of myofilaments were observed in cells of heart primordium after initiation of heartbeats, while no typical sarcomeres were observed. Sarcomeres with Z-lines were identified in cells of primitive heart tube, although myofilaments were not aligned. DIA-MS proteome analysis revealed that 43 proteins were significantly upregulated by more than 2.0-fold among a total of 7,762 detected proteins in heart primordium after initiation of heartbeats, compared with that before initiation of heartbeats. 27.9% of those upregulated proteins were constituent proteins of myofibrils and 23.3% of them were accessory proteins for myofibrillogenesis. Conclusions: These results indicate that initial heartbeats might be correlated with the formation of bundles of myofilaments along with upregulated myofibrils-related proteins.

### [2P-113]

### The role of vimentin cleavage in the signal transduction of abnormal vascular smooth muscle contraction

*Hiroko Kishi[1], Keisuke Shigenobu[1,2], Tatsuo Miyamoto[1] ([1]*Department of Molecular and Cellular Physiology, Yamaguchi University Graduate School of Medicine*, [2]*Third year at Yamaguchi University School of Medicine*)

Rho-kinase (ROK) modulates the phosphorylation level of myosin light chain (MLC) and plays a critical role in the signal transduction of abnormal vascular smooth muscle (VSM) contraction leading to vasospasm. Previously we identified sphingosylphosphorylcholine (SPC)/Fyn/ROK pathway which mediates abnormal VSM contraction. As possible downstream targets of Fyn tyrosine kinase, we identified vimentin by focused proteomics. Interestingly, SPC induced limited proteolysis of vimentin in human coronary artery smooth muscle cells (CASMCs) and VSM strips of the porcine coronary artery. Since vimentin is reported as the target of calpain, we examined the involvement of calpain. In CASMCs, SPC increased calpain activity, which was blocked by PD150606, a calpain inhibitor. Furthermore, PD150606 inhibited the SPC-induced VSM contractions, ROK activation and MLC phosphorylation, suggesting that calpain is involved in the signal transduction of abnormal VSM contraction mediated by the SPC/Fyn/ROK pathway. In the present study, we overexpressed a vimentin fragment whose length was corresponding to the vimentin fragment generated by the limited proteolysis in CASMCs. Overexpression of the vimentin fragment induced ROK activation and MLC phosphorylation in CASMCs. Furthermore, we observed impaired filament assembly of those vimentin fragment. Those findings suggested the involvement of vimentin cleavage in the signal transduction of abnormal VSM contraction.

### [2P-114]

### Abnormal differentiation of C2C12 myoblasts due to low expression level of ryanodine receptor 3

*Rika Ito[1], Yokoyama Shingo[2], Ohashi Kazuya[2], Hagiwara Arisa[2], Takeo Yusuke[1], Tatsuro Egawa[1,3], Atsushi Asakura[4], Katsumasa Goto[1,2] ([1]*Graduate School of Health Sciences, Toyohashi SOZO University*, [2]*School of Health Sciences, Toyohashi SOZO University*, [3]*Gradeate School of Human and Environmental Studies, Kyoto Univeristy*, [4]*Stem Cell Institute, University of Minnesota Medical School*)

The purpose of this study was to investigate the effects of knock down of ryanodine receptor 3 (Ryr3) on myogenic differentiation of mouse myoblasts-derived C2C12 cells. Ryr3 in C2C12 cells was knocked down by siRNA 3 days after the initiation of differentiation. On the 7$^{th}$ day of differentiation, numerous huge multinucleated myotubes having wide width were observed by knockdown of Ryr3. Ryr3-knockdown myotubes have aggregation of nuclei and the abnormal nuclear arrangement. Phalloidin- and α-actinin-staining showed no distinctive differentiation in the arrangement of cytoskelton between the control and Ryr3-knockdown myotubes. On the other hand, the knockdown of Ryr3 stimulated spontaneous contraction of C2C12 myotubes. Ryr3 may play a prat in the arrangement myonuclei during myogenic differentiation. This study was partially supported by JSPS KAKENHI (18H03160, K.G.; 19K22825, K.G.; 19KK0254, K.G.; 22H03474, K.G.; 22K19722, K.G.; 22H03319, K.G.; 22K18413, K.G.), a grant from Graduate School of Health Science, Toyohashi SOZO University (K.G.), and a research grant from Toyohashi SOZO university (K.G.). The authors declare no COI associated this study.

### [2P-115]

### Regulation of protein and oxidative energy metabolism are down-regulated in the skeletal muscles of Asiatic black bears during hibernation

*Mitsunori Miyazaki[1], Michito Shimozuru[2], Yu Kitaoka[3], Kenya Takahashi[4], Toshio Tsubota[2] ([1]*Department of Integrative Physiology, Graduate School of Biomedical and Health Sciences, Hiroshima University*, [2]*Laboratory of Wildlife Biology and Medicine, Graduate School of Veterinary Medicine, Hokkaido University*, [3]*Department of Human Sciences, Kanagawa University*, [4]*Department of Sports Sciences, The University of Tokyo*)

Hibernating animals exhibit an unexplained physiological characteristic of skeletal muscles being atrophy resistance, in which case muscle mass and strength remain almost unchanged both before and after hibernation. In this study, we examined the alterations in the regulatory systems of protein and energy metabolism in the skeletal muscles of Asiatic black bears during hibernation. Skeletal muscle samples (vastus lateralis muscle) were collected from identical individuals (n = 8) during the active (July) and hibernating (February) periods, while histochemical and biochemical analyses were performed. We observed no significant alterations in body weight, muscle fiber size, and fiber type composition during the active and hibernating periods, indicating that the skeletal muscles of bears are very well preserved during hibernation. In hibernating bear skeletal muscles, both regulatory pathways of muscle protein synthesis (Akt/mechanistic target of rapamycin and mitogen-activated protein kinase systems) and proteolysis (ubiquitin-proteasome and autophagy systems) were down-regulated. Gene expression levels of factors regulating oxidative metabolism were also decreased in hibernating bear skeletal muscles. This is likely an adaptive strategy to minimize the energy wasting of amino acids and lipids during hibernation, which is accompanied by a prolonged period of disuse and starvation.

### [2P-116]

### Upregulation of mitochondrial calcium regulation proteins following the eccentric contraction in rat skeletal muscle

*Ayaka Tabuchi[1,2], Ryo Takagi[3], Yudai Kikuchi[1], Yutaka Kano[1,4] ([1]*Department of Engineering Science., University of Electro-Communications*, [2]*Japan Society for the Promotion of Science Research fellowship*, [3]*Ritsumeikan Global Innovation Research Organization, Ritsumeikan University*, [4]*Center for Neuroscience and Biomedical Engineering, University of Electro-Communication*)

[Backgrounds] Repeated bouts of eccentric contractions (ECC) reduce exercise-induced skeletal muscle damage. We investigated the hypothesis that the first bout of ECC increases the mitochondrial calcium regulation proteins and leads protective effect on the consequent bouts. [Methods] Male Wistar rats were divided into two groups: Single bout (SB) and repeated bouts (RB). ECC (40 times, 5 sets) was performed once (SB) or twice 2 weeks apart (RB). The expression level of mitochondrial $Ca^{2+}$ regulating proteins in the tibialis anterior (TA) muscle were measured by western blots. $[Ca^{2+}]_i$ changes in TA with ECC was also measured *in vivo* using Fura-2 under anesthesia. Muscle damage was assessed 4 days after ECC in HE staining. [Results] The expressions of MCU and MICU2, components of the mitochondria $Ca^{2+}$ uniporter complex, were significantly increased in RB compared to SB ($p < 0.01$). $[Ca^{2+}]_i$ during ECC were significantly suppressed in RB ($p < 0.01$). After 5 hours ECC there were locally increased $[Ca^{2+}]_i$ area in SB, whereas $[Ca^{2+}]_i$ was significantly lower in RB (S:1.79 ± 0.04 vs R:1.57 ± 0.05, $p < 0.01$). Muscle damage was significantly decreased in RB ($p < 0.01$). [Conclusions] Upregulation of the mitochondrial $Ca^{2+}$ regulating proteins and suppressed $[Ca^{2+}]_i$ accumulation underlie the reduced muscle damage in the repeated bout effect.

### [2P-117]

### A novel assessment of neuromuscular junction transmission in living mice

*Tatsuhiro Yamaguchi[1], Koichi Nakazato[2], Karina KozakiKozaki[2], Kazushige Sasaki[1] ([1]*Graduate School of Arts and Sciences, The University of Tokyo*, [2]*Nippon Sport Science University*)

Purpose: Synaptic transmission at the neuromuscular junction is usually examined by electrophysiological techniques, but little is known about its relation to contractile performance. In the present study, we aimed to determine the neuromuscular junction transmission by measuring electrically evoked joint torques in living mice.Methods: Young (5 months old) and aged (24 months old) male C57BL/6J mice were anesthetized and subjected to two types of electrical stimulation: nerve stimulation (NS) and direct muscle stimulation (MS). The evoked plantar flexion torque was measured isometrically with a dynamometer at stimulation frequencies of 5 to 300 Hz.Results: We observed a smaller joint torque during NS than during MS. Blocking of neuromuscular transmission with pancuronium bromide resulted in a complete loss of joint torque during NS but not during MS. The ratio of evoked torques between NS and MS decreased with age and with stimulation frequency. Conclusion: These results suggest that the ratio of evoked torques between NS and MS represents the efficacy of synaptic transmission at the neuromuscular junction and is useful for studying its plasticity in aging and disease.

### Poster Presentation

[2P]
### Digestion, Digestive system

March 15 (Wed.), 12:10 - 14:10, Poster Room

### [2P-118]

**Analysis of effects of purple sweet potato extracts on glucose absorption in mice isolated small intestine by transmural potential method**

*Tomoo Homma[1], Shinya Hoshikawa[1], Nao Shoji[1], Mika Kobayashi[1], Yuka Suzuki[2], Takumi Koga[2] ([1]*Maebashi Institute of Technology,* [2]*Nichino Kagaku Kogyo Co.,Ltd.*)

Purple sweet potato extracts (PSPE) made by Nichino Kagaku Kogyo include much quantity of polyphenols (main component is anthocyanidin). It is reported that anthocyanidin shows various functions (antioxidative activity, increased effects of liver function and vascular flow, and so on), but effects of intake of PSPE on intestinal functions are not yet clear. In the present study, we measured transmural potential (TMP) generated by glucose applications in mice everted sacs of small intestine and investigated effects of PSPE on glucose absorption. Repeated changes of TMP by glucose applications were obtained. In the presence of PSPE (0.2, 0.4, 0.8%), amplitude of glucose-induced TMP was decreased with PSPE-concentration dependently. After washing PSPE, amplitude of glucose-induced TMP returned to the initial level. When treating times of PSPE with everted sacs (1, 5, 10 min) were changed, glucose-induced TMP were inhibited without treating time-dependency. The results showed that PSPE induced temporary inhibition of glucose absorption and suggested that intake of PSPE before eating or drinking something including glucose would prevent an increase of blood glucose level. Inhibitory mechanism of glucose absorption by PSPE has been studied.

### [2P-119]

**Proteinase-activated receptor 1 antagonist ameliorates colitis-associated tumorigenesis**

*XIAODONG LI[1], Lin-Hai Kurahara[1], Gaopeng Li[1], Katsuya Hirano[1] ([1]*Dept. Cardiovasc. Physiol., Fac. Med., Kagawa univ.*)

Abstract Objectives: Inflammatory bowel disease (IBD) is an intractable disease associated with tumorigenesis. The level of proteinases including thrombin increases in the colon of IBD patients. Proteinase-activated receptor 1 ($PAR_1$), a receptor for thrombin, induces inflammation and cell proliferation. We herein explored the therapeutic efficacy of a $PAR_1$ antagonist E5555 in colitis-associated tumorigenesis. Main findings: A colitis-associated tumorigenesis mouse model was prepared by azoxymethane (AOM)/dextran sulfate sodium (DSS) treatment. In AOM/DSS model, E5555 treatment started after completing AOM/DSS treatment ameliorated inflammation, epithelial proliferation, and tumorigenesis in the colon 20 weeks after AOM injection. $PAR_1$ expression was higher in tumor areas than in non-tumor areas in AOM/DSS model. Thrombin (1 unit/mL) induced an increase in cytosolic $Ca^{2+}$ concentration and ERK phosphorylation in intestinal myofibroblasts of the patients with Crohn's disease (CD), which were all inhibited by E5555. $PAR_1$ expression was co-expressed with α-smooth muscle actin in the CD colonic mucosa. Conclusion: $PAR_1$ plays an important role in intestinal inflammation and tumorigenesis. $PAR_1$ antagonist could be a potential strategy for the treatment of IBD and associated tumorigenesis.

### [2P-120]

**Tumor suppressive effect of rare sugar D-allose in inflammatory carcinogenesis mice model**

*Lin Hai Kurahara[1], Xiaodong Li[1], Tetsuo Yamashita[1], Takeshi Hashimoto[1], Katsuya Hirano[1] ([1]*Dept. Cardiovasc. Physiol., Fac. Med., Kagawa univ., Japan*)

[Background]: Chronic inflammation is known to be a risk factor for colorectal cancer. Immune system abnormality and oxidative stress are involved in the tumorigenesis. D-allose, a kind of rare sugar, has been reported to have an inhibitory effect on cancer cell proliferation. [Method]: The inflammatory colon tumor model was prepared by administration of azoxymethane (AOM) and 2% dextran sulfate sodium (DSS). Mice were given 5% D-allose in drinking water for 5 weeks. The evaluation of pathophysiology was performed 20 weeks after AOM adminstration. Colon cancer cell Caco-2 was used to examine direct effect of D-allose on cell proliferation. [Result]: Weight loss, loose stools, splenomegaly, bowel shortening, hematochezia, colonic tumor formation, and increase in inflammation score were observed in the AOM/DSS group. D-allose treatment significantly suppressed hematochezia, tumor formation, and increase in inflammation score. D-allose at 50 mM significantly suppressed Caco-2 proliferation. [Conclusion]: D-allose exerts preventive effect on inflammatory carcinogenesis indirectly by suppressing inflammation and directly by inhibiting cell proliferation.

### [2P-121]

**Multipotent dental pulp stem cells of deciduous teeth improve gastrointestinal function in a murine model of entero-neuropathy**

*Shinsuke Nakayama[1], Naoko Iwata[1], Chiho Takai[1], Shunichi Kajioka[2] ([1]*Department of Cell Physiology, Nagoya University Graduate School of Medicine,* [2]*Department of Pharmaceutical Sciences, International University of Health and Welfare*)

Hirschsprung's disease (HSCR) and its allied disorders are congenital entero-neuropathies with life-long implications in many cases. In this study, we thus examined the effects of intravenous transplantation of cultured dental pulp stem cells derived from human deciduous teeth (dDPSCs) in a mouse model of entero-neuropathy. Japanese fancy-1 (JF1) mouse carries a homozygous mutation at the piebald locus (Ednrbs) that encodes the endothelin-B receptor (ETBR), and the phenotype is characterized by a sparse network of myenteric ganglia, especially in the proximal colon. Intravenously injected dDPSCs (multipotent neural crest cells with low immunogenicity) migrated to affected regions of the intestine, and differentiated into both enteric neurons and pacemaker interstitial cells to correct abnormalities in the electrical and mechanical activities of the proximal colon. We anticipate that dDPSC transplantation could be developed into a novel cell-based therapy for HSCR and its allied disorders. In addition, the results indicate the importance of cooperating multiple motor systems in gastrointestinal motility.

### [2P-122]

**Alterations in central regulatory mechanisms of colonic motility in visceral hyperalgesia induced by colonic inflammation in rats**

*Yuuno Hiroki[1], Tomoya Sawamura[2], Natsufu Yuki[1], Kazuhiro Horii[2], Takahiko Shiina[1,2], Yasutake Shimizu[1,2] ([1]*Laboratory of Physiology, Joint Department of Veterinary Medicine, Faculty of Applied Biological Sciences, Gifu University,* [2]*Laboratory of Physiology, Joint Graduate School of Veterinary Sciences, Gifu University*)

### [2P-123]

### Anticancer drugs directly affects taste responce

*Isaki Hanamura[1], Koji Shibasaki[1] ([1]*University of Nagasaki Graduate School of Human Health Science*)

Although anticancer drugs induce taste disorders, the mechanism is still unclear. It is necessary to identify the mechanism, and develop the good cure methods. An intestinal endocrine cells, STC-1 cells, are ideal model for taste study, because they respond to all taste substances (sweet, salty, umami, sour and bitter tastes). We examined how anticancer drugs affected the taste responses by a $Ca^{2+}$ imaging method in STC-1 cells. We applied docetaxel (DOX) as a representative cancer drug. DOX application in high concentration (High DOX) increased the $Ca^{2+}$ response for quinine and citric acid compared with control group. On the other hand, (glutamate) and salty (NaCl) taste responses were reduced in high DOX condition. It is reported that high DOX inhibits cell proliferation and microtubuleformation, these might be affected the expression levels or function of taste receptors. Especially, salty response decreased in DOX concentration-dependent manner. Responses to bitter, sweet, umami, acid, and fat also changed, but the outcome were different from salty responses. Clinical trial reported taxane-based anticancer drugs (including DOX) cause strong salt taste disorder, consistent with this study. Based on our new findings, saltiness is caused by the DOX concentration-dependent manner, and exacerbate taste response. The authors declare no conflicts of interest associated with this presentation.

### Poster Presentation

### [2P]
### Oral physiology

March 15 (Wed.), 12:10 - 14:10, Poster Room

### [2P-124]

**Can do the rats recognize the components of binary sweet taste solutions?**

*Shinpei Takahashi[1], Fumihiko Nakamura[1], Toshiaki Yasuo[1], Takeshi Suwabe[1], Noritaka Sako[1] ([1]*Department of Oral Physiology, Asahi University School of Dentistry*)

Katagawa *et al*. (2016) revealed that rats could recognize the components in binary taste mixture contained different taste qualities. In this study, we conducted behavioral studies by using conditioned taste aversion technique to be clear whether rats could recognize the components in binary taste mixture made of same quality substances classified as sweet. Male Wistar/ST rats (8 weeks; n=27) were divided into conditioned (n=20) and control groups (n=7). During the 5 days, the rats deprived water were allowed presentation of distilled water (DW) for 10 min. On the next day, rats were allowed presentation of 0.5M glucose (G) for 10 min just before the injection of either 0.15M LiCl (conditioned group) or physiological saline (control group). On the following 5 test days after a recovery day, the number of licks for 11 test solutions, including sweet substances with or without G and others, were measured for 10 sec. As results, 3 of 20 rats acquired the conditioning to G did not suppress the number of licks to F, but they did those to all tested solutions contained G. Following 5 progressed test days, the number of rats, which did not avoid F but did all tested solutions contained G, was increased. These results suggest that rats can recognize the components in binary mixtures made of same taste quality (i.e. sweet) as well as those made of different quality.

### [2P-125]

**Functional analysis of fatty acid receptors expressed in mouse posterior tongue**

*Keiko Yasumatsu[1], Yumiko Nagai[1] ([1]*Tokyo Dental Junior College*)

Fatty acid (FA) sensors, CD36, GPR40 and GPR120, have been reported to be expressed in rodents' taste systems in the tongue. Among the chorda tympani (CT) nerve fibers, 17.9% in WT and 4.0% in GPR120-KO mice showed a maximal response to FAs (F-type). In behavioral test, GPR120-KO conditioned to avoid linoleic acid showed generalized avoidance to glutamate, indicating that GPR120 and F-type fibers are necessery for taste information of long chain FA. However, the function of FA receptors expressed in mouse posterior tongue is not clear so far. The present study, therefore, examined single fiber recordings from the glossopharyngeal nerve (GL) and behavioral preference based on 5-minute intake by using antagonists for FA receptors. The results showed that percentage of F-type fibers was 7% in WT and more than a half of M-type (glutamate-best) and S-type (sucrose-best) fibers are also responsive to FAs in the GL. The residual responses to FAs significantly suppressed by antagonists for CD36 and GPR40 in GPR120-KO mice. After transection of the CT, the mice were given two bottles, one containing vehicle and the other 10 mM oleic acid (OA) with or without an antagonist under a condition of 23 h water deprivation. The results showed that the preference scores were 67.0%, 49.9%, 33.4% and 64.7% for OA, OA with CD36 antagonist, OA with GPR40 antagonist, OA with GPR120 antagonist respectively. These results suggest that CD36 and GPR40 mediate preferable taste of FAs. Authors have no COI to disclose in relation to the presentation.

### [2P-126]

**Involvement of calcitonin gene-related peptide in developing tongue mechanical allodynia induced by sleep apnea in a rat.**

*Ayano Katagiri[1], Saki Kishimoto[1,2], Hiroki Toyoda[1], Hitoshi Niwa[2], Takafumi Kato[1] ([1]*Dept. of Oral Physiology, Osaka Univ. Grad. Sch. of Dentistry,* [2]*Dept. of Dental Anesthesiology, Osaka Univ. Grad. Sch. of Dentistry*)

Obstructive sleep apnea (OSA) is associated with several conditions characterized by orofacial pain. This study aimed to determine the role of calcitonin gene-related peptide (CGRP) in mediating ocular and tongue mechanical allodynia in a rat model of OSA. Rats were exposed to chronic intermittent hypoxia (CIH) during the light phase for 16 days. CIH rats exhibited ocular surface and tongue mechanical allodynia. The number of CGRP-immunoreactive neurons and activated satellite glial cells in the trigeminal ganglion (TG) was greater in CIH than in normoxic rats. The number of cFos-immunopositive neurons in the lamina I-II of Vc was significantly higher in CIH rats. Administration of CGRP antagonist into the TG of CIH rats suppressed CGRP expression in the TG and the density of CGRP-positive fibers and cFos expression in the Vc. These changes were associated with alleviating ocular and tongue mechanical allodynia in CIH rats. These results suggest that CGRP mediates orofacial mechanical allodynia induced by CIH in the peripheral and central nervous mechanisms. COI: NO

### [2P-127]

**Ghrelin-induced enhancement of swallowing motor activity in an arterially perfused rat preparation**

*Mitsunori Ishiguro[1,2], Kiyomi Nakayama[1], Shiro Nakamura[1], Ayako Mochizuki[1], Masanori Dantsuji[1], Yoshiaki Ihara[2], Tomio Inoue[1] ([1]*Department of Oral Physiology, Showa University School of Dentistry,* [2]*Division of Oral Functional Rehabilitation Medicine, Department of Special Needs Dentistry, Showa University School of Dentistry*)

Ghrelin is a gastrointestinal peptide hormone that is an endogenous ligand for the growth hormone secretagogue receptor (GHS-R). Although ghrelin is known to regulate food intake and body weight, the effects of ghrelin on swallowing have not been studied in detail. In this study, we investigated the effects of ghrelin on swallowing activity in motor nerves innervating the pharyngeal muscles in an arterially perfused rat preparation. Swallowing burst activity was evoked in the cervical vagus nerve by injection of 0.5 ml distilled water into the oral cavity. Administration of ghrelin (6 nM), but not des-acylated ghrelin (6 nM), in the perfusate increased the peak amplitude and duration of the swallowing burst activity. Furthermore, the first burst interval was shortened by ghrelin. These ghrelin-induced effects were blocked by administration of JMV2959 (6 μM), a GHS-R antagonist. In preparation without the hypothalamus, ghrelin failed to enhance swallowing activity. Furthermore, the administration of BIBO3304 (1 μM), a neuropeptide Y (NPY) Y1 antagonist, or L-152,804 (1 μM), a NPY Y5 antagonist, antagonized the effects of ghrelin on the swallowing activity. These results suggest that ghrelin enhanced swallowing activity via the hypothalamic neural network and that NPY Y1 and Y5 receptors are involved in this enhancement.

### [2P-128]

**Differences in somatosensory and gustatory inputs in the hemodynamics of major salivary glands in rats**

*Toshiya Sato[1], Yunosuke Okada[1], Hisayoshi Ishii[1] ([1]*Div. of Physiol., Dept. of Oral Biol., Sch. of Dent., Health Sci. Univ. Hokkaido. Japan*)

We reported that oral sensory stimulus input induces increases in blood flow (BF) in the major salivary gland via parasympathetic nerves (PN), suggesting the importance of PN activation due to oral sensory input in salivation and glandular hemodynamics. Mechanical input from the oral cavity is known to increase the rate of salivation in the parotid gland (PG), while gustatory input on the tongue in rats promotes salivation in the submandibular gland (SMG) rather than PG. Studies show that glandular hemodynamics is regulated according to differences in sensory input. However, no details are available. Thus, we analyzed the glandular hemodynamics during electrical stimulation of the inferior alveolar nerve (IAN; somatosensory input) or taste nerve (TN; gustatory input) in rats. IAN stimuli induced increases in BF in the PG and SMG, and the increases in the PG were significantly higher than in the SMG; Moreover, most salivation was from the SMG. The TN stimuli induced increases in BF and salivation in the SMG, but no BF increase and salivation in the PG. Thus, our results indicate that there is a difference in parasympathetic glandular BF increase depending on the type of sensory input, which suggests that this difference is important for the variation in relative secretion ratios of major salivary gland. Furthermore, there is a discrepancy between BF increase and salivation induced by somatosensory input in the PG; Further studies on this discrepancy will provide better understanding of the relationship between parasympathetic increase in glandular BF and salivation.

### [2P-129]

### Ligation of the infraorbital nerve increases neural responses in murine barrel cortex

*Kouhei Kitano[1], Kazunori Oohashi[1], Masayuki Kobayashi[1] ([1]*Department of Pharmacology, Nihon University School of Dentistry*)

Infraorbital nerve (ION) injury by surgical operation is clinically known to induce abnormal pain. ION is a branch of the maxillary nerve, and processes sensory information of the maxillofacial region to central nervous system (CNS). The postoperative neuropathic pain is presumed to be caused by plastic changes in the cortical local circuits. However, little has been known how the plastic changes occur in the oral and maxillofacial areas of the cerebral cortex. In this study, to clarify the mechanism of neuropathic pain in the orofacial area, we recorded the ION injury-induced plastic changes of the barrel cortex, a part of the primary somatosensory cortex, which processes tactile information of whiskers. ION injury was performed by ligating the infraorbital nerve approached from the oral cavity with a 6-0 nylon thread. Wide-field calcium imaging was performed over time under isoflurane anesthesia. We measured the fractional changes of fluorescence responding to the whisker stimulation with a CMOS camera. In control, whisker stimulation increased in the GCaMP6s fluorescence of the barrel cortex, which was followed by motor cortical activities. The responses in the barrel cortex were dominant in the contralateral side. In the ION-injury model mice, the amplitude of $Ca^{2+}$ responses was increased in the barrel cortex, whereas that of the motor cortex was unchanged. These results suggest that ION-injury induces plastic changes in a cortical region-dependent manner. No COI.

### [2P-131]

### Effect of chronic smoking on salivary proteins in umami -induced jaw/ sublingual gland saliva.

*Akira Furuyama[1] ([1]*Department of Oral Function and Molecular Biology, Ohu University School of Dentistry*)

In this study, we investigated salivary proteins in persistent stimulating saliva induced by umami stimuli and searched for candidate proteins for smoking biomarkers.Jaw/sublingual gland saliva were collected from non-smokers (6 males and 1 female (25 ± 1.7 years old)) and smokers (7 males (24 ± 1.3 years old)). Saliva samples were collected from one subject three times : at rest, (2) 2 min after umami stimulation (200 mM sodium glutamate + 40 mM sodium inosinate), and (3) 22 min after umami stimulation. Pooled saliva samples from 7 subjects were analyzed by Genomine's protein analysis service.As a result of two-dimensional electrophoresis, 198 protein spots were selected that showed more than 2-fold or less than 1/2 intensity change compared to the control group (non-smoking, resting saliva). Among them, 11 spots were selected as those with high spot intensity, high molecular weight, and large intensity change, and protein identification was performed. Four of them (CATD, ZG16, AMY1C, and AMY2B) had already been reported to be significantly down-regulated by smoking, but the difference of expression levels between the non-smoking and smoking groups 2 or 22 minutes after umami stimulation was more than 3-fold larger than in previous reports. In addition, I newly identified three proteins (CST5, IGHA, and ACTG1) whose expression levels were significantly reduced by smoking in persistently stimulated saliva 22 minutes after umami stimulation.In addition to conventional resting or stimulated saliva, there is a possibility to improve the quality of known marker proteins by using persistently stimulated saliva induced by umami stimulation. In addition, new candidate proteins for smoking biomarkers can be identified, thereby increasing the number of biomarker options available for diagnosis.

### [2P-130]

### Electromyographic assessment of Masticatory muscles (Masseter &Temporalis) and; their asymmetries in Adult Indian Population

*Bhupendra Singh Yadav[1], Mehul Hirani[2], Naresh Sharma[2] ([1]*Department of Physiology, Institute of Medical Sciences, Baranas Hindu University, Varanasi, India,* [2]*Department of Oral surgery, Institute of Medical Sciences, Baranas Hindu University, Varanasi, India*)

ObjectiveTo study the surface electromyography (sEMG) and it's asymmetry under restingand dynamic conditions in masticatory muscles.Materials and MethodssEMG of the masticatory muscles (masseter- right & left; temporalis- right & left)was done in 61 healthy subjects aged between 20 to 50 years with bilateralfunctional molar occlusal relationship. Root mean square (RMS) values sEMG foreach activity (rest, clenching, maximum mouth opening (MMO), left and rightexcursion) for a 10 seconds period were recorded and analysed. Indices forasymmetry of muscles, relative activity and resultant torque were assessed andanalysed.ResultsRMS values of sEMG during clenching were significantly higher for allmasticatory muscles in males compared to females ($p<0.05$). During excursivemandibular movement a statistically significant difference seen in ipsilateraltemporalis muscle in males. sEMG activity of masticatory muscles during rest aswell as functional activities of mandible was asymmetrical. A predominant masseteric activity was observed for all functional activities of mandible except during rest for which temporalis muscle activity was higher. Right sided torque was observed during rest, MMO and right lateral movements while a predominant left sided torque was present during left lateral movement and clenching.ConclusionsEMG values of masticatory muscles obtained in our study can be used asreference for healthy Indian population. A perfect muscular symmetry might beillusive and a controlled asymmetry criterion appears to be more useful corresponding to reality. sEMG of human masticatory muscles can provide valuable information of diagnostic, prognostic & causal relationships of muscular activity and related disorders.

### Poster Presentation

### [2P]
### Blood, Lymph, Immunity

March 15 (Wed.), 12:10 - 14:10, Poster Room

### [2P-132]

**Hepatic neutrophil pool possibly contributes to the postnatal neutrophil surge**

*Ryo Ishiwata[1], Yuji Morimoto[1] ([1]*Dept. of Physiology, National Defense Medical College*)

Mammalian neonates experience an abrupt surge of blood neutrophil counts in the first 72 hours of life. The neutrophil surge is considered an adaptive reaction of neonates facing the acute transition from a bacteria-free to a bacteria-rich environment. In this study, we aimed to elucidate the mechanism of the neutrophil surge.
Full-term (embryonic day 21, e21) Wistar rat fetuses were delivered vaginally or by caesarian section. Flow cytometric analysis revealed that the blood neutrophil counts increased from 484 ± 70.5/mL at e21 to 1,109 ± 80.8/mL at 6h after birth ($n = 8$, $P < 0.001$), while the counts of monocytes and lymphocytes did not change during this period. The proportion and the maturity of the bone marrow neutrophils did not change from e21 to 6h. Thus the neutrophil surge was not attributable to the bone marrow neurtophil pool. Immunohistochemistry for myeloperoxidase showed that at e21 fetuses, the spleen and liver contained a significant portion of neutrophils. Then we performed flow cytometric analysis of a whole organ. The neutrophil counts per liver decreased from e21 to day1 ($1.05\times10^7 \pm 5.09\times10^5$ vs. $0.85\times10^7 \pm 5.03\times10^5$, $n = 8$, $P < 0.05$), while the counts per spleen did not significantly change ($4.7\times10^5 \pm 7.23\times10^4$ vs. $7.4\times10^5 \pm 1.26\times10^5$, $n = 8$, $P = 0.09$).
These results indicate that the neutrophil surge possibly arises from the hepatic neutrophil pool.

### [2P-133]

**PYK2 regulates PD-L1 expression in melanoma**

*Yuto Mizuno[1,2], Masanari Umemura[1], Fumina Suzuki[1], Akane Nagasako[1], Soichiro Ishikawa[1], Tomoya Watanabe[2], Yukie Yamaguchi[2], Yoshihiro Ishikawa[1] ([1]*Cardiovascular Research Institute (CVRI), Yokohama City University Graduate School of Medicine,* [2]*Department of Environmental Immuno-Dermatology, Yokohama City University Graduate School of Medicine*)

[Introduction] Programmed cell death ligand 1(PD-L1) is expressed as an adaptive immune response to escape anti-tumor mechanisms in various cancer cell types, including melanoma. However, the complex mechanisms regulating PD-L1 expression are not completely understood. Our aim is to elucidate the signaling in PD-L1 expression and to explore candidate inhibitors in melanoma. [Materials and methods] The C8161, SK-MEL-2 and SK-MEL-24 cell lines (human melanoma) were used. Western blotting (WB) analysis was performed. VS6063 was used as PYK2 inhibitor. Melanoma RNAseq data (TCGA-SKCM) were obtained from The Cancer Genome Atlas (TCGA). [results] To explore the candidate genes which regulate the expression of PD-L1, we performed the linear regression analysis of mRNA PD-L1 and PYK2 mRNA in human melanoma. We found that PYK2 was significantly correlated with PD-L1 (R=0.52, p<0.001). To confirm the relationship between PYK2 and PD-L1, we examined whether VS-6063 affects the PD-L1 expression in C8161 cell line. We evaluated the IFN-γ-induced PD-L1 expression in the presence of VS-6063. PYK2 significantly negated the IFN-γ-induced PD-L1 expression, signal transducer and activator of transcription 1 (STAT1) and -interferon regulatory factor 1 (IRF1). In conclusion, our results show that PYK2 inhibitor might strengthen tumor immunity and exhibit the positive synergistic effect when combined to immune checkpoint inhibitors.

### [2P-134]

**Leukocytoclastic debris sustains ADP-induced platelet aggregation**

Chiaki Kijima[1], *Mariko Nakamura[1], Kazuhiko Hanashiro[2], Masanori Sunagawa[2], Gakuya Takamatsu[1], Masayuki Matsushita[1] ([1]*Department of Molecular and Cellular Physiology, Graduate School of Medicine, University of the Ryukyus, Nishihara, Okinawa, Japan,* [2]*Department of Pathophysiology, Graduate School of Nursing, Meio University, Nago, Okinawa, Japan*)

Inflammation, infection, and sepsis activate leukocyte, followed by its fragmentation. Although it has been reported that leukocytoclastic debris may have some effects on platelet aggregation, but the details are not clear. In this study, we investigated whether leukocytoclastic debris activate platelet aggregation, especially ADP aggregation. Blood was collected from rabbit auricular vein in the presence of sodium citrate, leukocytes in buffy coat were collected in microtube. After the leukocytes were physically disrupted with a pestle, they were centrifuged to obtain the disrupted leukocyte supernatant and the disrupted leukocyte-suspended plasma. ADP-induced platelet aggregation activity was measured using platelet-rich plasma (PRP) prepared from the same blood sample. As a result, the maximum ADP aggregation rates of the control plasma and the disrupted leukocyte supernatant were 45.3±7.5% and 44.5±9.2%, respectively. The maximum ADP aggregation rate with the disrupted leukocyte-suspended plasma was 33.3±5.4%. By comparing the aggregation retention rate (ARR), calculated from the maximum aggregation rate and the aggregation rate 3 minutes after the addition of the elicitor shows that ARR with the disrupted leukocyte-suspended plasma was 60.4±11.6%. The control plasma and the disrupted leukocyte supernatant were 46.8±19.8%, 51.4±14.0% (not statistically significant). The duration of ADP secondary aggregation by the disrupted leukocyte-suspended plasma was prolonged compared to the control plasma. Fluorescent immunostaining observation using P-selectin antibody showed adhesion of leukocytoclastic debris to platelets, and strong fluorescence of P-selectin was also confirmed at the adhesion sites. These results suggest that the leukocytoclastic debris adhered to the platelets and sustained the ADP-induced platelet aggregation activity. It is presumed that cell death accompanied by leukocyte crushing in inflammation/infectious disease causes sustained platelet aggregation, in addition to substances released from leukocytes. In pathological conditions such as inflammation, infection, and sepsis, platelet activation by leukocytosis may contribute to thrombus formation.

### [2P-135]

**Feature of chondroitin sulfate in stem cells derived from rat umbilical cord blood**

*Keiko Nakanishi[1,2], Kyohei Higashi[3], Toshihiko Toida[4], Masato Asai[2] ([1]*Ctl Hosp, Aichi Developmental Disability Center,* [2]*Inst Dev Res, Aichi Developmental Disability Center,* [3]*Dept Pharmacy, Tokyo Univ of Science,* [4]*Center for Preventive Medical Sciences, Chiba Univ*)

Chondroitin sulfate (CS) is a complex glycosaminoglycan (GAG) with repeating disaccharide units and is ubiquitous components of the extracellular matrix especially in the central nervous system (CNS), cartilage, and skin. While CS in the CNS has been well investigated, those in the hematopoietic stem cells are largely unknown. Wharton jelly, a gelatinous substance within the umbilical cord, is well known to contain mucopolysaccharides such as hyaluronic acid and CS, indicating that CS could have some physiological function in hematopoietic stem cells. We investigated the characteristics of CS in stem cell enriched-umbilical cord blood cells (SCE-UCBCs) which were expanded from rat umbilical cord blood cells. CS was detected in media and intima of vasculatures in rat umbilical cord at E19 by immunohistochemistry. CS was also detected in the SCE-UCBCs. Disaccharide composition analysis revealed that CS was more abundant than heparan sulfate (HS) in SCE-UCBCs and the major component of CS in UCBCs was A-unit. A colony-forming cell assay revealed that the percentage of colony-forming cells decreased in culture with CS degradation enzyme. It is possible that CS of UCBCs is involved in biological activities such as stem cell proliferation and/or differentiation.

### [2P-136]

**Coagulation activity-dependent regulation of fibrinolysis on activated platelets**

*Yuko Suzuki[1], Sano Hideto[1], Morooka Nanami[1], Honkura Naoki[1], Urano Tetsumei[1,2] ([1]*Medical Physiology, Hamamatsu University School of Medicine,* [2]*Shizuoka Graduate University of Public Health*)

Background: Platelets play crucial roles in the hemostatic response to provide the coagulation and fibrinolysis activation surface. Our real-time imaging analysis of the platelet-containing plasma (PLT-P) clot formation and lysis revealed the impact of thrombin-activatable fibrinolysis inhibitor (TAFI) activation for fibrinolysis inhibition. Aim: We further evaluate how inhibition of coagulation cascade modifies TAFI-dependent inhibition of fibrinolysis.Methods: Tissue factor-triggering clotting time (CT) and tissue plasminogen activator-initiated lysis time (LT) in PLT-P treated with anticoagulants were measured by turbidimetric assay. Fibrin clot structure and plasminogen accumulation were visualized by confocal laser scanning microscopy. Results: 1) LT was prolonged by the activation of TAFI, depending on the activated platelet-augmented thrombin generation. 2) Both rivaroxaban (activated factor X inhibitor) and dabigatran (thrombin inhibitor) dose-dependently prolonged CT and shortened LT, which was not shortened further by activated TAFI inhibitor at the highest concentration. 3) Two anticoagulants differently modified platelet-associated fibrin network structure and localization of plasminogen accumulation. Conclusion: Two anticoagulants-modified thrombin activity on the activated platelets differently affected TAFI activation.

### [2P-137]

### Immunoregulatory mechanism analysis of primary cilia in skin epidermal keratinocytes

Moe Nishizawa[1,2], *Hiroko Kato[1,2], Defri Rizaldy[1,2,3], Ken Ishii[2,5], Fumitaka Fujita[1,2,4]
([1]*Osaka University,* [2]*National Institutes of Biomedical Innovation, Health and Nutrition,* [3]*Institut Teknologi Bandung,* [4]*Mandom Corp.,* [5]*The University of Tokyo*)

Skin produces cytokines and chemokines in response to external stimuli for immune regulation, in addition to the protection from mechanical defense by barriers. Disruption or overreaction of immune system in epidermal keratinocytes contributes to inflammatory skin diseases. For example, psoriasis and atopic dermatitis are diseases characterized by dryness and recurrent eczema caused by Th17/22 and Th2 cytokines, respectively. There are no fundamental therapies for these diseases, but rather symptomatic treatment with antihistamines, steroids, immunosuppressive drugs, and other drugs that aim to alleviate symptoms. Therefore, understanding the pathogenesis of these diseases and developing treatments are important. Primary cilia are structural organelles protruding from the cell surface. Various ion channels and receptors are localized in primary cilia and are involved in development, differentiation, and homeostasis in the skin by accepting signals and stimuli, but their role in skin immunity is unknown. Previous results from our laboratory have shown that primary cilia are increased in the pathological skin of psoriasis and atopy. Furthermore, it has been suggested that primary cilia are involved in differentiation inhibition and inflammatory immune responses via the JAK/STAT pathway, but the role of primary cilium-expressing cells remains unclear. In this study, we focused on primary cilia in skin to elucidate the immune regulatory mechanism. Gene expression levels of human epidermal keratinocytes (NHEK) induced by inflammation with IL-13, knockdown of primary cilia, and untreated cells were analyzed using DNA microarray results. As a result, we found 13 molecules, including endothelin receptor A (EDNRA) and Transforming Growth Factor-β2 (TGF-β2), whose expression was altered upon inflammation induction in relation to primary cilia. Furthermore, knockdown of primary cilia resulted in a decrease in EDNRA and an increase in TGFB2, even at the protein level. In epidermal keratinocytes, these two molecules and primary cilia were not co-localized, suggesting that EDNRA and primary cilia may be expressed in the same basal layer in human skin.In the future, we plan to elucidate the detailed immune mechanism of primary cilia and to research and develop products for inflammatory skin that target primary cilia to improve skin barrier function.

### [2P-138]

### The effect of acidic microenvironment on the antitumor activity of γδT cells against lung cancer cell lines

*Shigekuni Hosogi[1], shura Yarimizu[1], Nobuhisa Todo[1], Yusuke Sano[1], Yuki Toda[1], Eishi Ashihara[1] ([1]*Kyoto Pharmaceutical University*)

The microenvironment of cancer becomes acidic because cancer cells produce a large amount of acidic metabolites. Many investigators have demonstrated that γδT cells exert anti-tumor effects by recognizing isopentenyl pyrophosphate (IPP) produced through the mevalonate pathway in cancer cells. However, the anti-tumor activity of γδT cells under a low pH condition remain unknown. The purpose of the present study is to clarify the antitumor effect of γδT cells against a lung adenocarcinoma cell line, A549 and H1299, under an acidic condition. γδT cells were expanded from peripheral blood mononuclear cells obtained from a healthy donor using ZOL and rhIL- 2. The anti-tumor effect of γδT cells was investigated using CSFE assay. The anti-tumor activity of γδT cells was enhanced in a low pH (pH 6.9) condition compared to that in a normal pH condition (pH 7.5) in A549 cells however conflicting reactions were observed in H1299. The mRNA expression of the IPP transporter (ABCA1) in cancer cells were increased in a low pH condition in A549, on the other hand, conflicting reactions were observed in H1299. These findings suggest that the IPP transporter (ABCA1) expression in lung cancer cells is associated with the anti-tumor activity of γδT cells

### [2P-139]

### The molecular mechanism of innate immune responses by bacteria specific modified nucleosides

*Miho Shimamura[1], Yu Nagayoshi[1], Kayo Nishiguchi[1], Ryosuke Yamamura[1], Takeshi Chujo[1], Kazuhito Tomizawa[1] ([1]*Department of Molecular Physiology, Faculty of Life Sciences, Kumamoto University*)

### [2P-140]

### The direction of gravity influences the morphic characteristics and molecular localization of HUVECs

*Taiga Nakayama[1], Susumu Minamisawa[1], Hiroki Bochimoto[1] ([1]*The Jikei University School of Medicine, Division of Aerospace Medicine, Department of Cell Physiology*)

Venous thrombosis during spaceflight has recently been reported. Spaceflight triggers vascular endothelial dysfunction, which may activate the human coagulation system. Autophagy in endothelial cells is enhanced under simulated microgravity. Simulated microgravity using a 3D clinostat is the accumulation of gravity by different angles, which means the analysis of separation of the direction of gravity is needed. In the present study, we analyzed the gravity response of human umbilical vein endothelial cells (HUVECs) as a model of vascular endothelial cells by using morphological analysis. HUVECs were cultured for a couple of days under the following conditions: the gravity direction in the cell axis from A (apical) to B (basal) (control group), (2) perpendicular against A to B (90° group), (3) from B to A (180° group), and (4) simulated microgravity using a 3D clinostat (SMG group). After fixation with 4% PFA, the cells were fluorescently immunolabeled for p62, an indicator for autophagic flux, and were observed with confocal microscopy. The cells fixed with 2% glutaraldehyde were freeze-dried and coated with osmium and observed with scanning electron microscopy (SEM). Immuno-staining revealed the tendency of increase in the cell number with strong cytoplasmic p62 localization in 180° and SMG group. The observation with SEM showed enhanced linear hairs on the cell surface of the 180° group and an abnormal invagination on the cell surface of the SMG group.These results indicate that changes in the direction of gravity may induce changes in cellular functions such as autophagy, as well as the environment of simulated microgravity, which can result in abnormal cell surface ultra-structures.

### Poster Presentation

[2P]
**Circulation**

March 15 (Wed.), 12:10 - 14:10, Poster Room

### [2P-141]

### Stress-induced changes in neural modulation of contractility in the rat rectal arterioles

*Retsu Mitsui[1], Mizuki Yamori[1], Hiroyuki Nakamori[1], Hikaru Hashitani[1] ([1]*Nagoya City University*)

Psychological or physical stress-induced reduction in the mucosal blood flow of the rectum is prolonged in irritable bowel syndrome (IBS) patients. Here, we compared the sympathetic and nitrergic modulations of the contractility in submucosal arterioles in the rectum between normal and IBS model rats exposed to water avoidance stress (WAS, 10 days in total, 1 h per day). The submucosal preparations of rectum were made, and changes in arteriolar diameter were measured using edge-tracking software. In normal rats, electrical field stimulation (EFS)-induced sympathetic vasoconstrictions were enhanced by the neuronal nitric oxide synthase (nNOS) inhibitor L-NPA in accordance with perivascular projection of nNOS-immunoreactive nerve fibres. The inhibitor of cGMP-specific phosphodiesterase 5 tadalafil attenuated the EFS-induced vasoconstrictions. In WAS rats, EFS-induced sympathetic vasoconstrictions were enlarged compared with those in sham-treated controls. L-NPA enhanced EFS-induced arteriolar constriction in sham-treated control rats but had no effect in WAS rats. Thus, sympathetic constrictions of submucosal arteriole of rat rectum appears to be negatively modulated by perivascular nitrergic innervation in normal conditions. In stress condition, sympathetic nerve-mediated arteriolar constriction is enlarged due to a decrease in nitrergic nerve-mediated inhibition.

### [2P-142]

### Warmed cardiomyocytes achieve contraction rhythm homeostasis by producing chaotic instability

*Seine A. Shintani[1,2,3] ([1]*Department of Biomedical Sciences, College of Life and Health Sciences, Chubu University,* [2]*Center for Mathematical Science and Artificial Intelligence, Chubu University,* [3]*Nagoya University, Institute for Advanced Research*)

The presenter constructed an experimental system to measure a movement of individual sarcomere in cardiomyocytes with nanometer precision. Then, we observed the case where the temperature near cardiomyocytes was warmed by near-infrared laser irradiation. As a result, the presenter discovered that when the cardiomyocytes are heated to a temperature similar to the core body temperature, the sarcomere inside the cardiomyocytes undergoes an oscillating state of repeated contraction and relaxation. The presenter named this oscillation HSOs (Hyperthermal Sarcomeric Oscillations). It was revealed that HSOs exhibit both homeostatic stability and chaotic instability when the calcium concentration in cardiomyocytes changes periodically due to excitation-contraction coupling. During HSOs, the phase relationship between adjacent sarcomeres changes irregularly to an in-phase or an anti-phase synchronous state. By utilizing this property, contraction rhythm homeostasis was achieved, in which the oscillation amplitude was changed in response to changes in calcium concentration and the oscillation period was kept constant. This property likely contributes to heartbeat robustness.

### [2P-143]

### Distinct contribution of local and circulating transforming growth factor β1 to amelioration of aortic aneurysms in *Tgfb1*$^{L/L}$ mice

*Masao Kakoki[1], Kenji Kansaku[1] ([1]*Dokkyo Medical University*)

Loss-of-function mutations in the component genes of transforming growth factor (TGF)-β signaling lead to fatal aortic aneurysm syndromes (Loeys BL et al, NEJM 2006). To study the contribution of TGF-β signaling in different tissues to the aortopathy, we generated mice having homozygous hypomorphic alleles (*Tgfb1*$^{L/L}$) (Kakoki M et al. PNAS 2013), which are tissue-specifically switched to hypermorphic alleles by Cre-loxP recombination. *Tgfb1*$^{L/L}$ mice spontaneously developed aortic aneurysms, which were associated with markedly shortened lifespan (median: 80 days vs. 802 days in WT mice). The aortic dilatation was mitigated by vascular smooth muscle cell-specific overexpression of *Tgfb1.* Their median lifespan was 169 days. In contrast, megakaryocyte lineage cell-specific overexpression of *Tgfb1* almost abolished the aortic aneurysms, and considerably elongated their median lifespan (673 days). Thus, supplementing TGF-β1 via circulating platelets prevented the aortic aneurysm and markedly improved vital prognosis in *Tgfb1*$^{L/L}$ mice. Our findings suggest that platelet manipulation has a therapeutic potential for aortic aneurysm syndromes caused by genetic insufficiency of TGF-β signaling.

### [2P-144]

### Angiotensin II-treated zebrafish as a new experimental model to study vascular elastic fiber formation

*Shota Tanifuji[1], Genri Kawahara[2], Takashi Nakamura[1], Saki Iida[1], Yukiko Hayashi[2], Utako Yokoyama[1] ([1]*Department of Physiology, Tokyo Medical University,* [2]*Department of Pathophysiology, Tokyo Medical University*)

Zebrafish is an attractive model to study cardiovascular disease. Angiotensin II (AngII) plays a role in elastic fiber dysregulation, i.e., aortic aneurysm, in mice and humans. It has been suggested that AngII induces extracellular matrix degradation through matrix metalloproteinase (MMP)-2 activation. However, the effects of AngII on vascular elastic fibers in zebrafish remain largely unknown. We aimed to examine the effects of AngII on the dorsal aorta in zebrafish. We microinjected AngII into *Tg(kdrl:EGFP)* zebrafish at the 1-cell stage, and measured dorsal aorta diameter at 5 days post fertilization (dpf) by visualization of EGFP expression. The average aortic diameter of five serial positions was significantly increased in AngII (160 ng)-injected zebrafish compared to buffer-injected controls (AngII, 23.2±0.7 µm; controls, 19.1±0.8 µm; n=13-18, $p$<0.05). Elastica van Gieson staining revealed that the dorsal aorta of AngII-injected 2-month-old zebrafish exhibited increased elastic fiber fragmentation (1.9±0.1-fold vs controls, n=5-8, $p$<0.05) with enhanced MMP-2 expression, although aortic diameter was similar in two groups. These results suggest that AngII-induced aortic expansion in early larvae associates with elastic fiber dysregulation in adult zebrafish. Monitoring AngII-induced aortic expansion at 5 dpf may be useful for high-throughput screening to discover the drugs that inhibit vascular elastic fiber dysregulation.

### [2P-145]

### Effect of prolonged sitting on cerebrovascular endothelial function

*Shotaro Saito[1], Kento Dora[1], Marino Karaki[1], Erika Iwamoto[2], Jun Sugawara[3], Shigehiko Ogoh[1] ([1]*Toyo university,* [2]*Sapporo Medical University,* [3]*National Institute of Advanced Industrial Science and Technology*)

Prolonged sedentary behavior increases the risk of brain disease. Although prolonged sitting has been reported to gradually decrease cerebral blood flow (CBF), its mechanism has not been clarified. The aim of the present study was to test the hypothesis that cerebrovascular endothelial function, an important CBF regulatory mechanism, is attenuated after prolonged sitting in healthy young adults. As a pilot study, three participants were asked to sit for 4-h without moving their lower limbs. During sitting, the blood flow and shear rate (SR) in the internal carotid artery (ICA) were hourly assessed using duplex Doppler ultrasound. The cerebrovascular endothelial function was evaluated by hypercapnia (30 s of hypercapnia stimulation, end-tidal partial pressure of $CO_2$: +9 mmHg)-induced flow-mediated dilation in the ICA (cFMD) before and after 4-h sitting. ICA blood flow and SR were gradually decreased during 4-h sitting in all participants (-17±12 % and -19±11 %, respectively). However, a decrease in cFMD following 4-h sitting was observed in only one participant. These results suggest that cerebrovascular endothelial function may not be affected by prolonged sitting but further investigations are necessary to identify our hypothesis.

### [2P-146]

### Analysis of Mechanism which determines $Ca^{2+}$ Ion Concentration Equilibrium in Ventricular Myocyte Mathematical Model

*Ryosuke Hara[1], Koki Koyama[1], Yukiko Himeno[1], Akira Amano[1] ([1]*Ritsumeikan University faculty of Life Sciences*)

$Ca^{2+}$ concentration determines contraction force of ventricular myocytes. Ryanodine receptor opens by $Ca^{2+}$ influx from $Ca^{2+}$ channel, and increase the cytosolic $Ca^{2+}$ concentration, and cause contraction force. The equilibrium points of ion concentrations under rest and exercise conditions are determined by the ion channels and transporter activities. However it's mechanism is not quantitatively explained yet. We analyzed the mechanism determining the equilibrium point of $Ca^{2+}$ concentration using human ventricular myocyte mathematical model O'Hara-Rudy model. First, the relationships between the $Ca^{2+}$ concentrations of four adjacent $Ca^{2+}$ compartments were obtained by simulating the model under various conditions, where the model parameters such as maximum conductances of $Ca^{2+}$ currents or cycle length were changed. The relations were approximated by multiple regression analysis. Then the changes in the $Ca^{2+}$ equilibrium concentrations with respect to the changes in parameters could be explained by the movement of these regression relations.

### [2P-147]

### Effects of AMPK activation on skeletal muscle contraction-induced vasodilation in the hindlimb of non-obese type 2 diabetes Goto-Kakizaki rats

*Takashi Sonobe[1,2], Hirotsugu Tsuchimochi[2], James Pearson[2] ([1]*Nippon Medical School,* [2]*National Cerebral and Cardiovascular Center Research Institute*)

In resistance arteries, exercise-induced endothelium-dependent vasodilatation is depressed in diabetes. We evaluated the effects of AMPK activation, which is suggested to regulate vascular tone, on skeletal muscle contraction-induced vasodilation in isoflurane anesthetized male Wistar rat and Goto-Kakizaki (GK) rat, a model of non-obese type 2 diabetes. Using in vivo X-ray microangiography, the hindlimb vasculature was visualized before and after tetanic muscle contraction (40 Hz, 3-5 V, 60 s). The angiography was repeated after an administration of AMPK activator, AICAR (20 mg/kg, i.v.). Angiographic images were analyzed, and longitudinal distribution of vessel diameter of the popliteal artery was presented as a color map and a histogram.The muscle contraction induced a right shift of the distribution of vessel diameter in both Wistar and GK rats. AICAR increased the number of large diameter sections of the popliteal artery (225-275 µm) in Wistar rats; however, the distribution of vessel diameters was similar between the groups after the muscle contraction. These data suggest that AMPK-dependent mechanisms to induce vasodilation is impaired in GK rats. (COI: No)

### [2P-148]

### Facilitated differentiation of induced pluripotent stem cells into cardiomyocytes in a microfluidic chip

*Rumaisa Kamran[1], Yun Liu[1], Qiang Li[1], Keiji Naruse[1], Ken Takahashi[1] ([1]*Department of Cardiovascular Physiology, Graduate School of Medicine, Dentistry and Pharmaceutical Sciences, Okayama University*)

Cardiovascular diseases have always posed a serious threat to mankind. With the advent of pluripotent stem cell technology, the pathophysiology of human heart diseases can now be mimicked using tissue culture methods, including trans-well and microfluidic chip models. In this study, we developed a tri-culture model comprising human gingival fibroblasts (HGFs), human umbilical vein endothelial cells (HUVECs), and induced pluripotent stem cells (iPSCs) using both trans-wells and microfluidic chips. We seeded iPSCs and HGFs onto the top channel of a chip and the apical side of a trans-well, whereas HUVECs were seeded onto the bottom channel of the chip and the basolateral side of the trans-well. After iPSCs were differentiated into cardiomyocytes in each model, their contractility was compared. Interestingly, cardiomyocytes in the chip model exhibited spontaneous contraction as early as on day 16 post-commencement of differentiation whereas those in the trans-well model showed contractions on day 23. Moreover, 100% spontaneous contraction was observed in the chip model compared with 11% in the trans-well model. Fluorescence-activated cell sorting revealed that 12.6% of the cells collected from the top channels in the chip model expressed cardiac troponin T, a specific cardiomyocyte marker. Our study suggests that the continuous supply of medium to the cells may be an important factor governing the differentiation of iPSCs into cardiomyocytes.

### [2P-149]

### Upregulation of neuregulin-1 contributes to suppressing the progression to systolic dysfunction in the diabetic cardiomyopathy

*Yoshinori Mikami[1], Fumiki Iwase[1], Daisuke Ohshima[1], Taichiro Tomida[1], Satomi Adachi-Akahane[1] ([1]*Department of Physiology, Faculty of Medicine, Toho University*)

Diabetic cardiomyopathy is characterized by an early left ventricular (LV) diastolic dysfunction and subsequent progression to systolic dysfunction. However, its underlying mechanism has not yet been fully understood. We aimed to identify the key signaling pathway to the development of diabetic cardiomyopathy. In the streptozotocin-induced T1DM model mouse 4 weeks after injection (STZ-4W), diastolic function was impaired without systolic dysfunction. In the ventricles of STZ-4W mice, the expression levels of neuregulin-1 (Nrg1) were significantly higher than those of control. Nrg1 was localized in the epicardium, endocardium, and vascular endothelial cells. We found that KLF binding sites are located at the promoter region of *Nrg1*. KLF9/10 showed similar expression and localization as those of Nrg1. To clarify the physiological role of Nrg1, trastuzumab (TRZ), an antibody against Nrg1 receptor ErbB2, was administered to mice for 3 weeks. The systolic function and T-tubule structure were significantly impaired in the TRZ-injected STZ-4W mice compared to STZ-4W mice. These results suggest that a compensatory upregulation of Nrg1-ErbB signaling preserves the LV systolic function. COI:NO.

### [2P-150]

### Influence of $Ca^{2+}$-stimulated adenylyl cyclase AC3 on pulmonary venous arrhythmias, which is expressed predominantly in the supraventricular area of the heart.

*Yosuke Okamoto[1], Naing Ye Aung[2], Daiki Kobayashi[1], Kyoichi Ono[1] ([1]*Department of Cell Physiology, Akita University Graduate School of Medicine,* [2]*Pathological and Image Analysis Center, Yamagata University Faculty of Medicine*)

Ectopic excitability in pulmonary veins (PVs) is the major cause of atrial fibrillation. We previously reported that the inositol trisphosphate receptor in rat PV cardiomyocytes cooperates with the $Na^{+}$-$Ca^{2+}$ exchanger to provoke ectopic automaticity in response to norepinephrine. Here, we focused on adenylyl cyclase (AC) as another effector of norepinephrine stimulation. RT-PCR, immunohistochemistry, and Western blotting revealed that the abundant expression of $Ca^{2+}$-stimulable AC3 was restricted to the supraventricular area, including the PVs. All the other AC isotypes hardly displayed any region-specific expressions. Immunostaining of isolated cardiomyocytes showed an enriched expression of AC3 along the t-tubules in PV myocytes. The cAMP-dependent response of L-type $Ca^{2+}$ currents in the PV and LA cells is strengthened by the 0.1 mM intracellular Ca2+ condition, unlike in the ventricular cells. The norepinephrine-induced au-tomaticity of PV cardiomyocytes was reversibly suppressed by 100 µM SQ22536, an adenine-like AC inhibitor. These findings suggest that the specific expression of AC3 along t-tubules may contribute to arrhythmogenic automaticity in rat PV cardiomyocytes.There is no conflict of interest in this study. (Reference; Okamoto, Y.et al. *Biomolecules* 2022, 12, 724).

### [2P-151]

### Protective effect of the conditioned medium from human adipose-derived stem cell culture on oxidative stress-induced cytotoxicity in primary cultured rat cardiomyocytes and human iPS cell-derived cardiomyocytes

*Takuya Notomi[1], Jumpei Yasuda[1], I-Shan Chen[1], Yoshinobu Matsuo[4,2], Takahiro Tsujimura[3], Hiroyuki Kaiya[2], Tomoe Nakamura-Nishitani[1] ([1]*Department of Pharmacology, Faculty of Medicine, Wakayama Medical University,* [2]*Grandsoul Research Institute for Immunology, Inc.,* [3]*Grandsoul Nara,* [4]*U-Medics, Inc.*)

Human mesenchymal stem cells (hMSC) are commonly used in clinical applications. Several studies in various tissues have shown that the biological effects of hMSCs depend on their secretary factors. However, their beneficial effects on cardiomyocytes (CMs) remain unknown. In this study, we investigated whether human adipose-derived mesenchymal stem cell culture-conditioned medium (ADMSC-CM) exhibit anti-stress effects in CMs. When cultured rat CMs were exposed to 75µM $H_2O_2$, spontaneous beating rate (SBR) and contractile deformation distance (CDD) decreased, and the beating stopped at ~10 min in control medium. In contrast, in ADMSC-CM, beating was maintained even 30 min after $H_2O_2$ exposure. This protective effect was lost when boiled ADMSC-CM (100°C, 10 min) was used, suggesting that the bioactive factors in ADMSC-CM may be higher-order structural molecules such as growth factors and cytokines. To investigate the possibility for clinical applications, we used human iPS-derived CMs (hiPS-CMs). These CMs were more resistant to $H_2O_2$ toxicity than rat CMs, but SBR and CDD were zero within 30 min at 300µM $H_2O_2$ in control medium. In ADMSC-CM, on the other hand, these values were still positive, suggesting a protective effects of ADMSC-CM against $H_2O_2$ toxicity in hiPS-CMs. Taken together, ADMSC-CM would be a potential candidate for treatment of stress-associated cardiovascular diseases.

### [2P-152]

### Suppression of TRPC6 augments Frank–Starling mechanism in mouse cardiomyocyte

*Yohei Yamaguchi[1], Toshiyuki Kaneko[2], Gentaro Iribe[2] ([1]*Department of Pharmacology, Graduate School of Medical Sciences, Nagoya City University,* [2]*Department of Physiology, Asahikawa Medical University*)

TRPC6 has been reported to contribute to cardiac mechanosensitive responses, e.g., the Anrep effect. However, its involvement in the Frank–Starling mechanism has not been known yet. Our echocardiographic studies showed that the cardiac performance of the volume-loaded heart was better in $TRPC6^{-/-}$ mice than in WT mice. To investigate the mechanism of this response, we used isolated ventricular cardiomyocytes from the wild-type (WT) and $TRPC6^{-/-}$ mice hearts. The cells were electrically stimulated at 4 Hz in normal Tyrode solution at 37 °C, and several steps of axial stretch were applied using the carbon fiber technique to obtain the end–systolic force–length relation (ESFLR) curve. The slope of the ESFLR, an indicator of cellular contractility, was significantly steeper in the $TRPC6^{-/-}$ mouse cardiomyocytes than the WT mouse cardiomyocytes. Transcriptome and real-time polymerase chain reaction analysis revealed that the genetic deletion of TRPC6 increased metallothionein 1 and 2, which are indicators of intracellular $Zn^{2+}$ concentrations ($[Zn^{2+}]_i$). Zinc imaging revealed that $[Zn^{2+}]_i$ increased to a greater extent in $TRPC6^{-/-}$ mouse cardiomyocytes than in WT mice. These results suggest that TRPC6 may regulate changes in cardiac muscle contractility via changes in zinc metabolism.

### [2P-153]

### Visualization of arrhythmia-like abnormal electrical activities in isolated rat atrial preparation using the optivcal recording of membrane potential

*Tetsuro SAKAI[1] ([1]*Dept Systems Physiol, Univ Ryukyus Sch Med*)

Using the optical recording of membrane potential activities using a voltage-sensitive absorption dye (NK2761), we visualized the excitation spread during arrhythmia-like abnormal electrical activities (tachycardia-like excitation, TE) in an isolated rat atrial preparation. We recorded optical action potentials simultaneously from 256 contiguous sites in the preparation using a 16 X 16 -element photodiode array (PDA) and mapped the excitation spread pattern during TE. Furthermore, we introduced a complementary metal-oxide semiconductor image sensor camera (CMOS : ORCA flash4.0, Hamamatsu Photonics Ltd., Hamamatsu, Japan, or Zyla 5.5 10-tap, Andor Technology Ltd., Belfast, UK) as the photodetector, in order to improve the spatial resolution of the optical imaging and to directly visualize the spatiotemporal pattern of the excitation spread. We could record the optical action potential with a spatial resolution of 1000 X 1000 (ORCA) or 1392 X 1040 (Zyla) and a temporal resolution of about 100 frame/second. Using digital image processing, we succeeded in visualizing the excitation spread during TE and made video clips of the excitation spread. In these movies, we demonstrated the circus movement of the excitatory wave (i.e. micro re-entry) and the abnormal automatism during TE. Chaotic electrical activities were also found. This study was approved by the Animal Care and Use Committee, University of the Ryukyus, and was conducted in accordance with its recommendations. No COI.

### [2P-154]

### Pore opening, not voltage sensor movement, underpins the voltage-dependence of facilitation by a hERG blocker

*Kazuharu Furutani[1,2], Ryotaro Kawano[1], Minami Ichiwara[1], Ryo Adachi[1], Colleen Clancy[2], Jon Sack[2], Satomi Kita[1] ([1]*Department of Pharmacology, Faculty of Pharmaceutical Sciences, Tokushima Bunri University,* [2]*Department of Physiology and Membrane Biology, University of California, Davis*)

A drug that blocks the cardiac myocyte voltage-gated K+ channels encoded by hERG carries a potential risk of long QT syndrome and life-threatening cardiac arrhythmia. Interestingly, certain hERG blockers can also facilitate hERG activation to increase hERG currents, which may reduce proarrhythmic potential. However, the molecular mechanism involved in the facilitation effect of hERG blockers remains unclear. The hallmark feature of the facilitation effect by hERG blockers is that a depolarizing preconditioning pulse shifts voltage-dependence of hERG activation to more negative voltages. Here we utilize a D540K hERG mutant to study the mechanism of the facilitation effect. D540K hERG is activated by not only depolarization but also hyperpolarization. This unusual gating property enables tests of the mechanism by which voltage induces facilitation of hERG by blockers. With D540K hERG, we find that nifekalant, a hERG blocker and Class III antiarrhythmic agent, blocks and facilitates not only current activation by depolarization but also current activation by hyperpolarization, suggesting a shared gating process upon depolarization and hyperpolarization. Moreover, in response to hyperpolarizing conditioning pulses, nifekalant facilitates D540K hERG currents but not wild-type currents. Our results indicate that induction of facilitation is coupled to pore opening, not voltage per se.

### [2P-155]

### Allosteric gate mechanism underlying the propofol inhibitory effects on the HCN channels

*Morihiro Shimizu[1], Toyoda Futoshi[2], Kojima Akiko[1], Fukushima Yutaka[1], Ding Wei-Guang[2], Omatsu-Kanbe Mariko[2], Kitagawa Hirotoshi[1], Matsuura Hiroshi[2] ([1]*Department of Anesthesiology, Shiga University of Medical Science,* [2]*Department of Physiology, Shiga University of Medical Science*)

The hyperpolarization-activated, cyclic nucleotide-gated (HCN1-4) channels contribute to the proper function in heart pacemaker cells and neurons. The opening of HCN channels is operated by both voltage-sensing and cAMP-bindings to the cyclic nucleotide binding domains (CNBDs). Recently Flynn and Zagotta developed an allosteric gate model (FZ model, PNAS, 2018) to describe the dual gate property of HCN channels. In the present study, we investigate the anesthetic agent propofol (2,6-diisopropylphenol) inhibition of HCN channels by a combined approach of patch-clamp experiments and analysis using the FZ model. Propofol caused a reduction of maximum current density and a hyperpolarizing shift of voltage dependence for channel activation, which were substantially attenuated by intracellular loading of cAMP. The FZ model optimization to our experimental data revealed that propofol only affected the autoinhibitory coupling between the pore domain and CNBDs. Our simulations successfully predicted that propofol effects were gradually attenuated by stepwise activation of four CNBDs and eventually abolished when the CNBDs were fully activated. These results suggested that propofol inhibits HCN channels by functionally interacting with the cAMP-dependent gate. Thus, our model-based approach provided a perspective for examinations and understanding of the functional interaction of HCN channels with various anesthetics.

### [2P-156]

### Echocardiographic and electrocardiographic analyses of cardiac function analysis during bathing

*Takeshi Yamaguchi[1], Hideki Yano[2], Yasuko Okuda[3] ([1]*Laboratory of anatomy, physiology and cardiovascular sciences, Faculty of Nursing, Shikoku university,* [2]*Adult health Nursing, Faculty of Nursing, Shikoku university,* [3]*Graduate school of Nursing, Hiroshima Bunka Gakuen university*)

[Background] Many elderly have accidents in their bathroom in winter due to consciousness disturbance, lethargy, and elevated body temperature. [Aim] The aim of this study was to establish an index of criteria for safe bathing in order to prevent bath-related accidents. [Methods] A Holter ECG was attached to healthy adult males (33 ± 13 years old) in the supine position for 10 minutes before echocardiography. Blood pressure and pulse rate were measured. Fatigue was measured using a visual analogue scale. Measurements were performed before, after 2 and 9 min in warm water immersion (WWI), and immediately and 10, 20, and 30 min after WWI. The water temperature of the bath was maintained at 40 ± 1°C. [Results] During WWI, pulse rate and sympathetic activity (L/H) were increased but parasympathetic activity (HF) was lower than before WWI. The left ventricular ejection fraction (LVEF) was significantly higher immediately after WWI than before WWI. Furthermore, there was a negative correlation between HF and LVEF and a regression equation was obtained. The E wave for blood flow velocity in the early diastolic phase increased immediately after WWI and returned to baseline 20 min after WWI. [Conclusion] EF increased in situations where parasympathetic activity was suppressed during WWI.

### [2P-157]

### Contribution of $I_{K1}$ to action potential repolarization in human ventricular myocyte and one-dimensional cell array evaluated using mathematical models

*Hiroto Nomura[1], Suzuka Enomoto[1], Yukiko Himeno[1], Akira Amano[1] ([1]*Ritsumeikan Univ.*)

### [2P-158]

**Effect of Voltage-dependence and Reaction Rate of Ion Binding Process of Mathematical Model of Na/Ca Exchanger (NCX) Considering Thermodynamics Constraints**

*Shaocong Ou[1], Keika Oyama[1], Masaaki Furukawa[1], Yuttamol Muangkram[1], Yukiko Himeno[1], Akira Amano[1] ([1]*Ritsumeikan University*)

### [2P-159]

**Dynamical Mechanisms of Human Sinoatrial Node Pacemaking: Roles of sarcolemmal ion channel currents and SR $Ca^{2+}$ handling determined by bifurcation analyses of a mathematical model**

*Yasutaka Kurata[1], Kunichika Tsumoto[1], Yuhichi Kuda[1], Mamoru Tanida[1] ([1]*Kanazawa Medical University*)

To provide insights into dynamical mechanisms of human sinoatrial node (SAN) pacemaking, we theoretically investigated the roles of sarcolemmal ion channel currents and sarcoplasmic reticulum (SR) $Ca^{2+}$ handling in the SAN pacemaking. Bifurcation analyses as well as numerical simulations were performed using a mathematical model for human SAN cells developed by Fabbri et al (J Physiol 595, 2017): stabilities of equilibrium points (EPs) and limit cycles (LCs) and bifurcation points were determined as functions of the maximum ion channel conductance or other parameters. Stabilities of EPs and spontaneous LC oscillations were strongly affected by modulating L-type $Ca^{2+}$ channel current ($I_{CaL}$) and rapidly-activating delayed-rectifier $K^+$ channel current ($I_{Kr}$) but not by inhibiting hyperpolarization-activated cation channel current ($I_f$); instability of EP at depolarized potentials was ascribable mainly to $I_{CaL}$. Robustness against hyperpolarizing loads was much lower in the human SAN cell model than in rabbit SAN cell models, which was due to smaller $I_f$ in the human SAN. Slow-fast decomposition analysis revealed slow $I_{Kr}$ deactivation-dependent phase 4 depolarization with smaller contribution of $I_f$ activation, especially when acetylcholine was applied. SR $Ca^{2+}$ cycling ($Ca^{2+}$ uptake rate) did not significantly affect stability or robustness against hyperpolarizing loads of the model cell. Spontaneous intracellular $Ca^{2+}$ oscillations around unstable EPs did not occur under voltage-clamped conditions. These results suggest that human SAN cell stability and pacemaking depend on $I_{CaL}$ and $I_{Kr}$ kinetics, and that $I_f$ or SR $Ca^{2+}$ clock is not essential for human SAN pacemaker activity.

### [2P-160]

**Pathophysiological characterization of left ventricular-derived cardiomyocytes from Goto-Kakizaki type 2 diabetic rats**

*Yuko Iwata[1], Hirotsugu Tsuchimochi[1], James Pearson[1] ([1]*National Cerebral and cardiovascular Center Research Institute*)

Heart failure with reduced or preserved ejection fraction (HFrEF and HFpEF, respectively) is an important cause of morbidity and mortality worldwide. We have shown that the transient receptor potential cation channel TRPV2 is a principal candidate for abnormal $Ca^{2+}$-entry in HFrEF models such as dilated cardiomyopathy. However, very little is known about calcium dynamics and the role of TRPV2 in HFpEF models. To elucidate the mechanisms leading to diastolic dysfunction in HFpEF, we characterized the phenotypes of cardiomyocytes from middle-aged Goto-Kakizaki (GK) type 2 diabetic rats based on morphological properties, high-speed video imaging and $Ca^{2+}$ imaging and compared with Wistar control rats. TRPV2 was concentrated in the cardiomyocyte sarcolemma of GK rats compared to Wistar. Furthermore, the relaxation rate was reduced in GK cardiomyocytes and a negative relationship between TRPV2 surface accumulation and relaxation rate was observed. These results suggest that TRPV2 accumulation in cardiomyocytes might have a significant role in the progression of HFpEF and that TRPV2 may be a potential therapeutic target for HFpEF as well as HFrEF.

### Poster Presentation

[2P]
**Respiration**

March 15 (Wed.), 12:10 - 14:10, Poster Room

### [2P-161]

**5-$HT_{1A}$ receptors in lateral parabrachial nucleus may intensify respiratory-body movement coupling in the neonatal rat pons-medulla-spinal cord preparation**

*Yoko Tsukamoto[1], Hirotaka Ooka[2], Chiaki Uchida-Nishiyama[2], Akiko Arata[2]
([1]*Hagoromo University of International Studies,* [2]*Hyogo Medical University*)

It is known that serotonin strongly modulates the rhythm generator like respiratory or body movement. In the previous studies, we analyzed the relationship between respiratory rhythm and body movement using neonatal rat medulla-spinal cord preparation with or without pons. We also examined the effect of serotonin on the relationship between respiratory rhythm and body movement in parabrachial nucleus (PB). We showed that the respiratory rhythm was synchronized to body movement when serotonin was applied in the pons-medulla-spinal cord preparation. On the other hand, even if serotonin was applied, the synchronization between respiratory rhythm and body movement was not observed without pons. In this study, we examined how serotonin coordinate respiration-body movement coupling. We found the optical signals induced by body movement in the dorsal lateral pons using voltage sensitive dye. The respiration-body movement coupling was abolished by 5-HT1A receptor blocker. A lot of 5-HT1A receptor immunoreactive cell bodies were found in the lateral PB in the pons and vestibular nucleus in the medulla in P2 rat. These results suggest that dorsolateral pons is essential structure for the respiratory-body movement coupling, and 5-HT1A receptor in lateral PB may play a crucial role in the functional connection between medulla and spinal cord.

### [2P-162]

**Development of a lung fibrosis model using lung epithelial cells, fibroblasts, and vascular endothelial cells**

*QIANG LI[1], Yun LIU[1], MengXue WANG[1], Yin LIANG[1], Keiji NARUSE[1], Ken TAKAHASHI[1]
([1]*Department of Cardiovascular Physiology, Graduate School of Medicine, Dentistry and Pharmaceutical Sciences, Okayama University*)

Pulmonary fibrosis is a severe lung disease causing scarring and thickening of the lungs, which makes breathing difficult. Although animal models are often used to study lung fibrosis, models using human cells are necessary to develop better therapeutic strategies. To develop a human lung fibrosis model, NCI-H441 lung epithelial cells and human gingival fibroblasts (HGF) were first monocultured. The cells were treated with transforming growth factor beta-1 (TGF-β1) to induce fibrosis-like changes. TGF-β1 treatment increased the area of adhesion to the culture plate of fibroblasts, but not the lung epithelial cells. Gene expression analysis of fibrosis markers using quantitative reverse transcribed polymerase chain reaction (qRT-PCR) revealed that alpha-smooth muscle actin (α-*SMA*) expression increased with increasing doses of TGF-β1 in HGF, but not in NCI-H441 cells. In contrast, expression of fibrosis marker genes, including *Slug* and *Vimentin*, increased in a dose-dependent manner in NCI-H441 cells. Transwells were used to recapitulate lung structure. Human umbilical vein endothelial cells were seeded on the basolateral side of the transwells, which were coated with Matrigel to facilitate cell adhesion. NCI-H441 cells and fibroblasts were seeded on the apical side of the transwells. After the cells reached 100% confluency, cells were treated with TGF-β1 for 2 days, and NCI-H441 and HGF were collected from the apical side of the transwells. The expression of fibrosis marker genes, including *α-SMA*, *Slug*, and *Vimentin*, were measured using qRT-PCR. These three marker genes tended to increase in response to TGF-β1. To achieve an air-liquid interface (ALI), similar to lung alveoli, the medium was removed from the apical side of the transwells. Uptake of an intracellular calcium indicator was observed under a confocal microscope even two days after removal of the medium, indicating the successful formation of an ALI. Additionally, immunostaining for PECAM-1 indicated high integrity of the intercellular junctions between the endothelial cells. We will further develop this human triculture model to facilitate the development of treatments for pulmonary fibrosis.

### [2P-163]

**Interrelation of pulmonary functions parameters with plasma progesterone level during different phases of normal menstrual cycle**

*Ashish Kumar Gupta[1] ([1]*Department of physiology Institute of medical Sciences Banaras Hindu University Varanasi*)

Background and objective The present study was carried out to observe the correlation of some lung function parameters with the endogenous plasma progesterone level during different phases of menstrual cycle. Methods The study was conducted on 30 healthy young female volunteers with age range of 20-25 years in the Dept of Physiology of SKMC, Muzaffarpur, during Jan 2019- Jan 2020. All the subjects were studied in 3 phases of menstrual cycle for 2 consecutive cycles. lung function parameters and plasma Progesterone level during each phase of menstrual cycle were measured by spiroexcel and by ELIZA method in auto analyzer respectively. Comparison of the values between different phases were done by paired 't' test considering menstrual phase data as baseline due to negligible amount of progesterone detected in this phase. Correlation of FVC, FEV1 and FEV1/FVC%with Progesterone level in each 3 phases were analyzed by Pearson's correlation– coefficient test. Results Plasma progesterone was much higher during luteal phase compared to those of follicular phases of both cycles (24.54ng/ml vs1.41 ng/ml; 26.56 ng/ml vs. 1.48 ng/ml). Both FVC and FEV1 were significantly higher ($p<0.001$) during luteal phase than those of follicular phases in both the cycles.PFT Parameters were positively correlated with plasma progesterone level but these relationships had failed to show any statistical significance. Discussion and conclusion Study observed increased ventilation and high endogenous progesterone level during luteal phase. Therefore increased ventilation might be related to high progesterone level during luteal phase owing to increased inspiratory muscle endurance and bronchial relaxation effect.

### [2P-164]

### Blockade of microglial activation delays the occurrence of severe hypoxia-induced seizure

*Isato Fukushi[1,2], Kotaro Takeda[2,3], Masashi Yoshizawa[2,4], Yosuke Kono[2,4], Yohei Hasebe[2,4], Keiko Ikeda[5], Mieczyslaw Pokorski[6], Yasumasa Okada[2] ([1]*Aomori University of Health and Welfare,* [2]*Murayama Medical Center,* [3]*Fujita Health University,* [4]*University of Yamanashi,* [5]*Tokyo Institute of Technology,* [6]*University of Opole*)

Recently, it has been elucidated that microglia modulate neuronal functions in various brain regions. Severe hypoxia induces seizures, which reduces ventilation and further worsens hypoxia and the ictal state. This is a serious threat to patients with underlying hypoxic respiratory pathologies such as severe asthmatic attacks. However, the mechanisms of hypoxia-induced seizure occurrence remain unknown. We investigated the hypothesis that microglia could interact with hypoxia-induced seizures. We measured ventilation by whole-body-plethysmography in conscious, spontaneously breathing, adult mice. Ventilatory responses to hypoxia were analyzed in two groups (n=10 each), one without and the other with the administration of minocycline, an inhibitory modulator of microglial activation. The time from the onset of hypoxia to the occurrence of seizures was significantly longer in the group with minocycline than without minocycline. We suggest microglia play a crucial role in the generation of seizure activity in severe hypoxia. Blockade of microglial activation may prevent hypoxia-induced seizures, which would be worthy to investigate in a clinical trial.

### [2P-166]

### Shoseiryuto improved TDI-induced allergic rhinitis symptoms via inhibiting IL-33 release from nasal epithelial cells

*Naoki Adachi[1], Manabu Kitano[1], Seiya Fukuoka[1], Tadashi Hisamitsu[1], Masataka Sunagawa[1] ([1]*Department of Physiology, School of Medicine, Showa University*)

Allergic rhinitis (AR) is induced by allergens such as ragweed and other pollens, mites, and chemical substances. To block the entry of such substances from the environment, airway epithelialium has a barrier function in the superficial layer of the airways. The airway epithelial cells regulate inflammatory responses via secreting cytokines and chemokines. Recent studies have shown that IL-33 that regulates the TH2 cytokine response is released by nasal epithelial cells, which is an important step in developing the inflammatory response. Shoseiryuto (SST) is one of the traditional herbal medicines (Kampo medicine) that has long been used as a natural medicine for allergic diseases, such as AR and asthma. In this study, we investigated whether SST ameliorates the AR-related symptoms induced by Toluene diisocyanate (TDI), a major cause of occupational asthma and rhinitis, in rats and inhibits IL-33 release from nasal epithelial cells. We found an ameliorative effect of SST on TDI-induced AR symptoms and suppressive effect on IL-33 release from nasal epithelial cells.

### [2P-165]

### Effect of chronic Hyperglycemia on the pulmonary system and its response to acute lung injury.

*Rinkoo Yadav[1] ([1]*IMS BHU*)

ABSTRACT Introduction: In Diabetes, there is proinflammatory dysfunction, which delays the inflammatory response. A large prospective multicentric study of patients with septic shock found that Diabetes delays the development of acute lung injury (ALI). However, covid -19 induced ALI has worsened outcomes in Diabetic patients and deteriorated various cardiopulmonary parameters. Objective- To study the effect of chronic Hyperglycemia on the pulmonary system and its response to acute injury. Material and Method: The experiments were performed on healthy adult male albino rats weighing 150-180 gm. The trachea, jugular vein and carotid artery of anaesthetized rats were cannulated to keep the respiratory tract patent, deliver saline/ drugs and record BP, respectively. Animals were randomly divided into four groups. In group I/ control, normal saline (65µl) was injected. In group II, Oleic acid (OA 65µl) was administered to induce ALI in rats. In group III, for a hyperglycemic model, rats were fed a high-fat diet for 2 weeks; after that, streptozotocin was injected (35mg/kg i.p). In hyperglycemic model rats, Group IV was injected with oleic acid to induce acute lung injury (ALI). Cardio-respiratory parameters were recorded, and pulmonary water content and histological examination of the lung in all the animals were determined. Result: A histological examination of the Hyperglycemic rat model showed minimal focal interstitial fibrosis and peribronchial inflammatory cell infiltration. Injection of OA produces ALI, indicated by a moderate to severe increase in respiratory frequency followed by a progressive decrease and, ultimately, death of the animal. OA-induced ALI in the hyperglycemic animal model shows early deterioration of all cardiorespiratory parameters. Pulmonary water content was significantly increased, and a histological examination of the lungs showed moderate focal interstitial fibrosis, alveolar septal infiltration, alveolar oedema, alveolar exudate and peribronchial inflammatory cell infiltration. The survival time of animals in this group is less compared to the OA group. Discussion: In this study, OA-induced ALI in the hyperglycemic animal model shows moderate to severe lung injury, the same as in normal rats. However, cardiopulmonary parameters were early deteriorated and the survival time of animals in this group was also less. Other studies in the mouse with type 2 diabetes demonstrated that Diabetes causes pulmonary fibrosis. They explained that Diabetes trigger a cascade, starting with increased DNA damage, impaired DNA repair, and leading to persistent DNA damage signalling. Other studies show that hyperglycemia leads to significant oxidative stress and promote inflammation by increasing proinflammatory cytokines, which also leads to pulmonary fibrosis. Nuclear factor kB (nf-kb), a transcription factor whose activation is tightly regulated under normal conditions, probably shifts the balance in favour of a pro-inflammatory state in Hyperglycemia, thus exaggerating acute lung injury. Also, the hyperglycemic state induces the formation of advanced glycation end products (AGE), which are now recognized to promote inflammation and endothelial dysfunction. All these factors may lead to early deterioration of all cardiorespiratory parameters and survival time. Conclusion- OA-induced ALI in the hyperglycemic animal model shows early deterioration of all cardiorespiratory parameters.

### Poster Presentation

[2P]
**Reproduction**

March 15 (Wed.), 12:10 - 14:10, Poster Room

### [2P-167]

**Functional coupling between BK and L-type $Ca^{2+}$ channels found in cricket oviducts**

*Tomohiro Numata[1], Kaori Sato-Numata[1], Masami Yoshino[2] ([1]*Akita Univ.*, [2]*Tokyo Gakugei Univ.*)

Large-conductance calcium ($Ca^{2+}$)-activated potassium ($K^+$) (BK) channel activation is essential for resting membrane potential and electrical rhythm formation. To characterize endogenously expressed BK channels and assess the functional relevance of $Ca^{2+}$ sources leading to BK activity, a patch-clamp method was performed in cricket oviduct myocytes to analyze single-channel recordings. The single channel conductance of 120 pS, voltage dependence, and intracellular $Ca^{2+}$ sensitivity demonstrated the categorical nature of vertebrate BK channels. Single-channel current amplitudes were sensitive to iberiotoxin (IbTX). These biophysical and pharmacological results indicate that BK channels are endogenously expressed in the muscle cells. Extracellular $Ca^{2+}$ removal and L-type $Ca^{2+}$ channel (L-Ca) modulators affected BK activity. Administration of ryanodine abolished BK activity. Finally, the proximity between L-Ca and BK was investigated. Administration of L-Ca modulators by the backfill method successfully allowed small regions of the pipette's tip to affect BK activity. This effect was further found to be membrane potential dependent. These results indicate that BK channels are endogenously expressed in cricket oviduct myocytes and that BK activity is regulated by L-Ca activity and $Ca^{2+}$ release from $Ca^{2+}$ stores.

### [2P-168]

**Exercise effect on superimposed preeclampsia in the Dahl salt-sensitive rat**

*Toru Kobayashi[1,2], Ko Yamanaka[1], Itakura atsuo[2], Hidefumi Waki[1] ([1]*Juntendo University*, [2]*Juntendo University, Department of Obstetrics and Gynecology*)

Preeclampsia is a major cause of maternal death and preterm delivery. Currently, no effective treatment exists; however, exercise has been proposed as a potential modality to reduce blood pressure and improve fetal growth. We hypothesized that exercise would improve the maternal syndrome and fetal outcomes of superimposed preeclampsia (SPE). An SPE model of Dahl salt-sensitive rats were divided into an exercise (EX) and a non-exercise (NE) group. The EX rats were housed in wheel cages for 4 weeks prior to conception and during the last trimester of pregnancy. We compared the blood pressure, urinary protein levels and the fetal body weight between the groups. The NE group had a significant rise in blood pressure during early pregnancy compared with the EX group (114.1±1.1 vs. 106.5 ± 2.1 mmHg; $p < 0.01$). Exercise significantly increased fetal growth (EX: 2.99 ± 0.48 g, NE: 2.32g ± 0.44 g; $p < 0.01$). There was no difference in proteinuria between the groups. These results suggest that exercise before and after pregnancy improves fetal and maternal symptoms of SPE. However, the non-improvement of urinary protein indicates that several mechanisms, except for renal, may be involved in exercise.

### [2P-169]

**Selection of mouse early embryos by membrane potential measurement**

*Masao MIYAKE[1], Susumu YOSHIE[1], Satoru KANEKO[2], Akihiro HAZAMA[1] ([1]*Department of Cellular and Integrative Physiology, Fukushima Medical University School of Medicine*, [2]*2Ichikawa General Hospital, Tokyo Dental College*)

Morphological inspection is the most commonly used technique to pick quality oocytes and embryos for artificial fertilization. To raise reproductive ratio, a new selection method from a new point of view is needed. The membrane potential reflects expression of ion channels and completeness of cell membrane, it may evaluate ovum quality. We previously showed that there was a wide dispersion of membrane potential among eggs without morphological difference. It implied this technique could be applied for quality selection. In this study, we analyzed the relationships between embryogenic outcome and membrane potential of mouse embryos after freeze-thaw cycle. Single-cell, two-cell and four-cell embryos were applied to the freeze-thaw cycle, and measured membrane potential. Some embryos performed good morphological characteristics, and could reach blastocysts. But most embryos which performed near zero voltage stopped development. The near zero voltage embryos are possible to be scratched during conventional protocol. This method may be applicable to ignore damaged embryos. All experiments were planned toward institutional guidelines and reviewed by institutional animal care and use committee.(COI:NO)

### [2P-170]

**Role of hypoxia-mediated autophagy in Rat ovary**

*Anil Kumar Yadav[1], Kumar Sarvottam[1] ([1]*Department of Physiology, Institute of Medical Sciences, Banaras Hindu University, Varanasi India*)

Hypoxia is known to affect ovarian function in several ways and chronic exposure may even lead to ovarian dysfunction and altered hormonal profile. Hypoxia induces autophagy, a self-degradatory process involved in elimination of damaged proteins and organelles in cell. Primarily it acts as a Protective mechanism to maintain the cellular homeostasis and longevity, but in some extreme case it also leads to cell death. The specific role autophagy plays in ovary during hypoxia is not known. In this study we tried to identify the hormonal, morphological, ultrastructural, and molecular change related to autophagy role of autophagy in ovaries of female rats of different age groups subjected to varying degree and duration of hypoxia by utilizing the techniques like histology, immunohistochemistry, western blotting and transmission electron microscopy. We found that Autophagy is involved and helps in overcoming the hypoxia related stress in ovary of female rats subjected to mild and moderate chronic hypoxia whereas it acts as cell death during severe chronic hypoxia. More numbers of cell in corpus luteum were positive to autophagic markers in all types of hypoxia as well control.

### [2P-171]

**Establishment of endometritis model and elucidation the inflammatory mechanism by LPS in mouse**

*Kisaki Tomita[1], Erina Yoneda[1], Sangwoo Kim[1], Hideomi Sanai[2], Yuki Muranisi[1] ([1]*Obihiro University of Agriculture and Veterinary Medicine*, [2]*Obihiro ART Clinic*)

### Poster Presentation

### [2P]
### Endocrine

March 15 (Wed.), 12:10 - 14:10, Poster Room

### [2P-172]

**Changes in serum magnesium, calcium, and parathyroid hormone levels with long-term use of proton pump inhibitors in outpatients with gastroesophageal reflux disease**

*Toru Shizuma[1] ([1]*Department of Physiology, Tokai University School of Medicine*)

Background: Hypomagnesemia is induced by the long-term use of proton pump inhibitors (PPIs), which potentially inhibit the absorption of magnesium. Hypomagnesemia can induce hypocalcemia by blockading parathyroid hormone (PTH) release. Although cases of PPI-induced hypomagnesemia and/or hypocalcemia have been reported sporadically, few studies have evaluated changes in the levels of PTH after long-term use of PPIs. Subjects and methods: We prospectively investigated changes in the serum levels of intact PTH as well as magnesium and calcium before and at 6 and 12 months after the continuous administration of PPI in 43 outpatients with gastroesophageal reflux disease. Results: None of the patients developed hypomagnesemia or hypocalcemia after PPI treatments. There were no significant differences in the magnesium and calcium levels before and after PPI administration. However, the PTH levels at 6 and 12 months after PPI administration were significantly higher than those before PPI treatment, although these increases were within the normal range. Discussion: One of the mechanisms that could be responsible for the increase in serum PTH levels may be decreased absorption of magnesium or calcium with PPI use, which could promote the compensatory secretion of PTH via a feedback mechanism.

### [2P-173]

**Effects of growth hormone on skeletal muscle and bone in mice**

*Naoyuki Kawao[1], Takashi Ohira[1], Yuya Mizukami[1], Yoshimasa Takafuji[1], Hiroshi Kaji[1] ([1]*Department of Physiology and Regenerative Medicine, Kindai University Faculty of Medicine*)

The interactions between muscle and bone have been noted. The skeletal muscles influence bone metabolism by releasing humoral factors called myokines. Although growth hormone (GH) affects both muscle and bone, its effects on myokine expression remain unknown. We therefore examined the effects of GH administration for 8 weeks on myokine expression, muscle and bone in mice. GH significantly increased muscle mass in the whole body and lower limbs, tissue weights of the extensor digitorum longus (EDL) and soleus muscles, and grip strength in mice. GH significantly increased cortical thickness and area at femurs of mice. Moreover, GH attenuated the decrease in bone volume of trabecular bone at the femurs of ovariectomized mice. As for expression of myokines linking muscle to bone, GH significantly reduced levels of follistatin mRNA, but not the mRNA of other myokine examined, in the EDL of mice. Meanwhile, GH increased the levels of serum follistatin in mice. In conclusion, we showed that GH administration increases muscle mass, grip strength and bone mass in mice. Myokines linking muscle to bone might not be important for GH effects on bone. COI: NO

### [2P-174]

**Role of 1,5-anhydro-D-glucitol in carbohydrate metabolism**

*Asuka Morita[1], Motoshi Ouchi[1], Kobayashi Shunsuke[3], Keitaro Satoh[2], Tomoo Homma[4], Mika Kobayashi[4], Tatsuya Suzuki[5], Kenzo Oba[6], Masahiro Yasutake[5,7], Anzai Naohiko[8], Tomoe Fujita[1] ([1]*Department of Pharmacology and Toxicology, Dokkyo Medical University School of Medicine,* [2]*Department of Pharmacology, Meikai University School of Dentistry,* [3]*Depertment of Endocrinology, Metabolism and Nephrology, Graduate School of Medicine, Nippon Medical School,* [4]*Department of Biotechnology, Maebashi Institute of Technology,* [5]*Division of Geriatric Medicine, Department of General Medicine and Health Science, Nippon Medical School,* [6]*Kawaguchi Sakura Clinic,* [7]*Department of General Medicine and Health Science, Graduate School of Medicine, Nippon Medical School,* [8]*Department of Pharmacology, Chiba University Graduate School of Medicine*)

[Purpose]The polyol 1,5-anhydro-D-glucitol (1,5-AG) has a similar structure to that of glucose. A previous study reports that 1,5-AG stimulates insulin secretion, whereas another report suggests no association. The aim of this study was to evaluate the association between 1,5-AG and carbohydrate metabolism in several organs.[Methods/Results]To determine the effect of 1,5-AG on insulin secretion, we used an organ bath set-up with isolated rat pancreas preparations. At normal (100 mg/dL) and high (400 mg/dL) glucose concentrations, 1,5-AG did not affect insulin or amylase release. Subsequently, mice were treated with 1,5-AG for 7 days through continuous subcutaneous administration. Blood 1,5-AG levels in 1,5-AG-treated groups were significantly higher than those in the controls, but no changes in blood glucose and insulin levels were observed. Lastly, the everted intestinal sac model was used in mice to examine 1,5-AG absorption, which tended to be higher in the upper and middle small intestine than in the lower small intestine. [Conclusion] Ex vivo experiments with the organ bath set-up suggested that 1,5-AG had no effect on insulin secretion. High blood 1,5-AG levels through continuous subcutaneous administration suggested that there was most likely no direct impact of 1,5-AG on glucose control, in vivo. The site of intestinal 1,5-AG absorption might be similar to that for glucose.

### [2P-175]

**Alteration of oxytocin levels in breast milk of lactating mice**

*Yusuke Watanabe[1], Itsuki Takahashi[1], Kaoru Uchida[1], Izuki Amano[2], Wataru Miyazaki[3], Noriyuki Koibuchi[2], Noriaki Shimokawa[1,2] ([1]*Takasaki University of Health and Welfare,* [2]*Gunma University,* [3]*Hirosaki University*)

In mammals, although breast milk is an essential for the growth of offspring, our understanding regarding the factors in breast milk which influence the future behavior, particularly that with regard to nurturing, of offspring is still limited. We previously reported the possibility that maternal prolactin (PRL) during the prenatal period may be a critical factor for the development of nurturing behavior in the subsequent generation. In this study, we focused on oxytocin (OXT), a posterior pituitary hormone, as a candidate factor influencing future nurturing behavior. First, breast milk was expressed from lactating mothers, and then the milk was centrifuged, and the OXT content in the whey (supernatant) was measured by an enzyme-linked immunosorbent assay. OXT levels were high on the third day of lactation and then dropped to basal levels. Interestingly, we also observed an increase in OXT levels at middle lactation. We suspect that the high OXT levels on the third day of lactation are due to the contractions of the uterine muscle during parturition, and that the mid-lactation rise is necessary for influencing the future behavior of offspring, such as their nurturing behavior. We are currently planning to analyze the nurturing behavior of offspring fed milk lacking OXT.

### Poster Presentation

[2P]
### Autonomic nervous system

March 15 (Wed.), 12:10 - 14:10, Poster Room

### [2P-176]

**The hypothalamic A11 region and the medullary raphe nuclei regulate colorectal motility mediated through the spinal defecation center.**

*Tomoya Sawamura[1], Kazuhiro Horii[1], Natsufu Yuki[2], Hiroshi Yamaguchi[3], Akihiro Yamanaka[3], Takahiko Shiina[1,2], Yasutake Shimizu[1,2] (*[1]Laboratory of Physiology, Joint Graduate School of Veterinary Sciences, Gifu University, [2]Laboratory of Physiology, Joint Department of Veterinary Medicine, Faculty of Applied Biological Sciences, Gifu University, [3]Department of NeuroscienceII, Research Institute of Environmental Medicine, Nagoya University*)

We have previously shown that the brain regulates colorectal motility via the L6-S1 spinal cord. In this study, we aimed to identify the mechanisms on regulation of colorectal motility by using DREADD system. Adeno-associated virus (AAV)-retro encoding Cre recombinase was injected into the L6-S1 in male SD rats. Another AAV encoding hM3Dq or hM4Di was injected into the A11 region and/or raphe nuclei. Colorectal motility was assessed in vivo in anesthetized rats. Injection of CNO, a ligand for the artificial receptors, enhanced colorectal motility in rats expressing hM3Dq in the A11 region, only when a GABAergic inhibitor bicuculline was preinjected into the L6-S1. This colorectal motility was inhibited by spinal injection of a dopaminergic inhibitor. Similar results were obtained in rats expressing hM3Dq in raphe nuclei. In rats expressing hM4Di in the A11 region and raphe nuclei, enhancement of colorectal motility due to noxious stimuli in the colon was diminished by CNO injection. Fecal outputs by water avoidance stress (WAS) in conscious rats were also reduced by CNO injection in these rats. Our results show that the A11 region and raphe nuclei have important roles in colorectal motility.The authors have no COI to disclose.

### [2P-177]

**Evaluation of autonomic dysfunction in patients with chronic pain**

Atanu Roy[1], *Sanjeev Singh[1] (*[1]Institute of Medical Sciences, Banaras Hindu University*)

Introduction: Heart rate variability (HRV) is a non invasive method for assessment of cardiac autonomic tone, which is performed when the body is in a resting state. Chronic pain especially of musculoskeletal origin changes cardiac autonomic tone and affects the cardiovascular system. Many studies have shown the effect of chronic pain on heart rate variability (HRV), but there is lack of data for the same from Indian population. Thus, the present study was designed to study the autonomic changes in patients with musculoskeletal chronic pain. Methods: This study was carried out after getting approval from institutional ethical committee. Only the patients with chronicity of >6 months duration and severity of >3 on visual analog scale were selected. The exclusion criteria were medications affecting autonomic system like α/β blockers , chronic diseases etc. Age-sex matched controls were selected and five minute electrocardiogram was recorded in the resting state and was analyzed for the HRV by using time and frequency domains. Results: In time domain parameters the RR intervals in male cases were significantly different than the controls, whereas the minimum RR interval is significantly different than female controls. The rest of the time domain parameters didn't show any significant difference between the cases and controls. In frequency domain parameters low frequency value in female cases were significantly different than controls and low frequency/high frequency ratio in male cases were significantly different than controls. Conclusion: The male and female cases had elevated sympathetic and reduced parasympathetic parameters in comparison to their respective controls.[1] Nilsen KB, Sand T, Westgaard RH, Stovner LJ, White LR, Bang Leistad R. 2007 Autonomic activation and pain in response to low-grade mental stress in fibromyalgia and shoulder/neck pain patients. Eur J Pain.;11(7):743-55.Elsevier.[2]Fazalbhoy A, Birznieks I, Macefield VG. 2012, Individual differences in the cardiovascular responses to tonic muscle pain: Parallel increases or decreases in muscle sympathetic nerve activity, blood pressure and heart rate. Exp Physiol, 97(10):1084-92.Wiley[3] Saraswathi PV, Neelambikai N, Mahesh A, Govindarajan K. 2013, Cardiovascular parasympathetic nervous system dysfunction in female rheumatoid arthritis patients. Indian J Physiol Pharmacol, 57(1):23-30.Scientific Scholar.

### [2P-178]

**Autonomic and cardiovascular regulation by exercise-indued hormone in rats and mice**

*Mamoru Tanida[1], Mikako Ikeda[1], Yasutaka Kurata[1] (*[1]Department of physiology 2, Kanazawa medical university*)

Irisin, one of hormone produced in the skeletal muscle, activated systemic metabolism by direct action on the adipose tissue. In the present study, we examined effect of irisin on efferent autonomic nervous system and cardiovascular function in rats and mice. After treadmill running for 5 day and rest for 2day in rats, acute treadmill running elevated blood irisin levels. Next, intracerebral ventricular (ICV) injection of irisin activated efferent sympathetic nerve outflows to the kidney and brown adipose tissue in anesthetized mice, however it suppressed efferent vagal nerve outflow to the stomach. Similarly, in anesthetized rats, ICV irisin elevated renal sympathetic nerve activity, blood pressure and heart rate. Intracellular signal molecules such as mitogen-activated protein kinase, phosphoinositide 3-kinase and protein kinase A, functioned as hypothalamic autonomic regulator, were not changed by central irisin in mice. To clarify the hypothalamic signaling mechanism of autonomic and cardiovascular controls by irisin, further examination will be needed in the future.

### [2P-179]

**Dietary GABA potentiates postprandial activation of vagal afferents thereby enhancing satiation**

*Kento Ohbayashi[1], Taichi Nohmi[1], Utano Nakamura[2], Riho Sagane[2], Maiko Miyazaki[2], Atsushi Yamatsu[2], Mujo Kim[2], Yusaku Iwasaki[1] (*[1]Laboratory of Animal Science, Graduate School of Life and Environmental Sciences, Kyoto Prefectural University, [2]Department of Research and Development, Pharma Foods International Co., Ltd*)

Gamma-aminobutyric acid (GABA) is known as the main inhibitory neurotransmitter, and it is also present in foods. Recently, GABA widely available as dietary supplements which have beneficial effects on brain functions. However, GABA has long been thought to be unable to cross the blood-brain barrier, which raises questions about the mechanisms of these beneficial actions. In the present study, we explored the effects of peroral (po) administration of GABA on feeding behavior as a brain function and the involvement of vagal afferents.Po GABA immediately before refeeding reduced food intake without aversion in overnight fasted mice. This effect was blunted by surgical and chemical denervation of vagal afferents. However, po GABA alone did not evoke vagal afferents activation and po GABA 30 min before refeeding did not alter feeding. These results suggested that GABA interacts with meal-evoked factors and regulates feeding. Simultaneously administration of GABA and liquid diet potentiated the postprandial activation of vagal afferents and robustly enhanced meal-evoked satiation. The present study demonstrates that dietary GABA potentiates postprandial vagal afferents activation by possibly collaborating with meal-evoked factors, and thereby regulate brain function such as feeding behavior.

### [2P-180]

**Chronic activation of hypothalamic orexin neurons in menopause model mice**

*Hideki Kashiwadani[1], Sho Ueno[1], Tomoyuki Kuwaki[1] (*[1]Graduate School of Medical and Dental Sciences, Kagoshima University*)

In menopausal patients, plasma orexin level was increased three times, and the increase was suppressed after hormone replacement treatment. Furthermore, the menopause model mice (ovariectomized (OVEX) mice) showed that the stress-induced hot flash-like phenomenon, one of the major menopause symptoms, is significantly attenuated by systemic administration of orexin receptor antagonist. These findings have supported the hypothesis that menopause symptoms are induced by the hyperactivation of hypothalamic orexin neurons. However, the hyperactivation during menopause or in OVEX mice has not yet been reported. In this study, we examined the expression of delta FosB, one of the FosB isoforms induced by chronic stimulation, in hypothalamic orexin neurons after OVEX. We found that the number of delta FosB-positive orexin neurons was significantly increased after OVEX, indicating that orexin neurons were chronically activated after OVEX. Our findings support the hypothesis that hyperactivation of hypothalamic orexin neurons mediates, at least in part, the menopausal symptoms.

### [2P-181]

### GABA neurons affected by inflammation in the cardiovascular center of spontaneously hypertensive rats

*Shawn Tate[2], Minori Shibutani[2], Ryo Kawamoto[2], Yoshii Nishino[1,2], Toshiyuki Saito[1,2]
([1]*Faculty of Life Sciences, Kyoto Sangyo University,* [2]*Graduate School of Life Sciences, Kyoto Sangyo University*)

Elevated activity of the rostral ventrolateral medulla (RVLM) neurons, referring to the cardiovascular center, is considered to be the primary cause of the essential hypertension. Because of the synaptic pathway for arterial baroreflex being in the medulla oblongata, tonically inhibition via GABA neurons has to be taken into consideration from the caudal VML (CVLM) neurons to the RVLM neurons to regulate the sympathetic tone. In this study, we have examined whether GABA-neurons are degenerated in the cardiovascular center of the hypertensive rats (male spontaneously hypertensive rats; (SHRs), in comparison with the normotensive rats (male Wistar Kyoto rats; WKYRs). Immunohistochemical (IHC) studies have shown the GAD65+GAD67 positive area and the number of Iba-1 cells in the CVLM of SHRs significantly decreased than in WKYRs. A decrease in the number of the Iba-1 cells was also found in the RVLM of SHRs. In both areas of SHRs, there were found the ramified Iba-1 cells, with fewer and shorter processes than of WKYRs. Another line of our study indicated the plasma level of S-100 beta in SHRs was significantly higher than that in WKYRs, suggesting blood-brain barrier disruption. In SHRs, there may occur mild inflammation in the cardiovascular center, in which suppression (via GABA) may be impaired to the RVLM neurons through the nucleus of the tractus solitarius (NTS). We would express our sincere appreciation for kindly supply of SHRs and WKYs to Disease Model Cooperative Research Association (DMCRA), Kyoto, Japan.

### [2P-182]

### Simultaneous suppression of cervical vagal nerve and sympathetic nerve activity induced by intravenous alpha-2 adrenergic receptor agonist administration in conscious rats

*Kenju Miki[1], Haruka Inoue[1], Yuki Kitagawa[1], Shizuka Ikegame[1], Misa Yoshimoto[1]
([1]*Autonomic Physiology, Faculty of Human life and Environment, Nara Women's Univ.*)

A limited number of reports have attempted to record vagal nerve activity (VNA) in the conscious state. Thus, the nature of changes in VNA still remains unclear. In this study, an alpha-2 adrenergic receptor agonist (dexmedetomidine) was used to search for ways to alter VNA in the conscious state. Electroencephalography, electromyography, electrocardiography, cervical VNA, renal sympathetic nerve activity, and catheters for arterial pressure measurement and intravenous drug administration were measured after male Wistar rats were anesthetized and implanted with electrodes. Dexmedetomidine (50 μg/kg) was intravenously administered in conscious rats. Cervical VNA and EEG theta/delta ratio gradually decreased and reached their lowest values at about 10 min after intravenous dexmedetomidine administration. On the other hand, renal sympathetic nerve activity, electromyography, and heart rate decreased immediately after administration, while arterial pressure immediately increased. They recovered to their pre-administration levels 60 min after the administration. These results suggest that alpha-2 adrenergic receptor agonists simultaneously inhibit VNA and sympathetic nerve activity, but with different action mechanisms. No conflict of interest.

### [2P-183]

### Physiological function of basal forebrain cholinergic fibers projecting to the olfactory cortex

*Jura Moriya[1,2], Daichi Morihara[1,2], Fusako Kagitani[1], Sae Uchida[1] ([1]*Department of Autonomic Neuroscience, Tokyo Metropolitan Institute of Gerontology,* [2]*Tokyo University of Agriculture and Technology*)

The piriform cortex, a major olfactory cortical area, receives odor information directly from the olfactory bulb, and send the signals to the higher-odor association areas such as orbitofrontal cortex. The piriform cortex as well as the olfactory bulb receives cholinergic axonal projections from neurons originating in the nucleus of the horizontal limb of the diagonal band of Broca (HDB) in the basal forebrain. However, it is unclear whether HDB stimulation increases extracellular acetylcholine (ACh) release in the piriform cortex. We used microdialysis with high performance liquid chromatography and electrochemical detection (HPLC–ECD) to measure the extracellular ACh concentrations in the piriform cortex of urethane-anesthetized rats. Focal chemical stimulation by microinjection of L-glutamate into the HDB in the basal forebrain increased extracellular ACh concentrations in the ipsilateral piriform cortex. The present study showed that the activation of the HDB in the basal forebrain produces an increase in extracellular ACh release in the piriform cortex. The increased ACh may contribute modulation of olfactory information processing in the piriform cortex.

### [2P-184]

### The effects of chronic mild stress on GABAergic system in the paraventricular nucleus of hypothalamus associated with cardiac autonomic activity.

*Janpen Bangsumruaj[1,2], Anusak Kijtawornrat[3], Sarinee Kalandakanond-Thongsongb[3] ([1]*Interdisciplinary Program in Physiology, Graduate School, Chulalongkorn University, Bangkok, Thailand,* [2]*Division of physiology, Faculty of Science and Technology, Huachiew Chalermprakiet University, Samut Prakan, Thailand,* [3]*Department of Veterinary Physiology, Faculty of Veterinary Science, Chulalongkorn University, Bangkok, Thailand*)

Stress is known to be associated with cardiovascular diseases. One possible mechanism is the reduction in gamma-aminobutyric acid (GABA)ergic transmission at the paraventricular nucleus (PVN), which contributes to the disinhibition of sympathoexcitatory circuits and activates sympathetic outflow. At present, the mechanism of chronic mild stress (CMS) on GABAergic transmission at the PVN and cardiac autonomic activity is not yet fully clarified. Therefore, this study was designed to investigate the effects of CMS on the GABAergic system at the PVN and on the cardiac autonomic activity. Adult male Sprague–Dawley rats were randomly assigned to control (left undisturbed in their home cage) or CMS (subjected to various mild stressors for 4 weeks). Cardiac autonomic activities were determined by heart rate variability (HRV) analysis, and GABAergic alterations at the PVN were determined from GABA levels and mRNA expression of GABA-related activities. Results showed that the CMS group had decreased HRV as determined by the standard deviation of all R–R intervals (SDNN). The low frequency (LF) and high frequency (HF) powers of the CMS group were higher than those of the control. Therefore, the LF/HF ratio was consequently unaffected. These findings indicated that following 4-week CMS, despite the increased in sympathetic and parasympathetic activities, the autonomic balance was preserved. For the GABAergic related parameters, the CMS group had decreased mRNA expression of glutamic acid decarboxylase-65 (GAD-65), the GABA-synthesizing enzyme, and increased mRNA expression of gamma-aminobutyric acid transporter-1 (GAT-1). Moreover, the GAD-65 mRNA expression was negatively correlated with LF. In conclusion, 4-week CMS exposure in male rats could attenuate GABAergic transmission at the PVN and alter cardiac autonomic activities. COI:No

### [2P-185]

### Regulation of afferent vagal nerves in the abdominal organs by lactate after exercise

*Yuichiro Kimoto[1], Mamoru Tanida[1], Yasutaka Kurata[1] ([1]*Department of physiology 2, Kanazawa medical university*)

Physical exercise has beneficial effects of the obesity, diabetes and hypertension. Generally, acute exercise causes elevation of blood lactate levels in humans and animals, and elevated lactate enters to the liver and brain to be metabolized. There are afferent vagal nerves in the abdominal organs such as liver, stomach and small intestine. Here, to examine effect of lactate on the vagal afferent nerve outflows, we performed whether intravenous injection of lactate affect afferent vagal nerve in the anesthetized mice. In exercise experiment, training of treadmill running was forced in the rats and mice for 5 days. After training, 60 min treadmill running elevated blood lactate levels. Next, to determine concentration equal to lactate concentration raised by exercise, effect of intravenous injection of various doses of lactate on blood lactate levels was investigated in the anesthetized mice. Injection of 0.1g/kg b.w. lactate elevated blood lactate levels. Finally, we performed measurement of afferent activity of celiac vagal branch innervating the small intestine in anesthetized mice. It activated afferent celiac vagal nerve outflow. These data suggest that elevated lactate after acute exercise may activate afferent vagal pathway and send signals to the brain to regulate whole body functions such as metabolism or cardiovascular system.

### [2P-186]

### Activation of the dorsomedial hypothalamus enhances colorectal motility by activating spinal defecation center via the medullary raphe in rats.

*Natsufu Yuki[1], Kazuhiro Horii[2], Tomoya Sawamura[2], Hiroshi Yamaguchi[3], Akihiro Yamanaka[3], Takahiko Shiina[1,2], Yasutake Shimizu[1,2] ([1]*Laboratory of Physiology, Joint Department of Veterinary Medicine, Faculty of Applied Biological Sciences, Gifu University,* [2]*Laboratory of Physiology, Joint Graduate School of Veterinary Sciences, Gifu University,* [3]*Department of NeuroscienceII, Research Institute of Environmental Medicine, Nagoya University*)

### Poster Presentation

### [2P]
### Environmental physiology

March 15 (Wed.), 12:10 - 14:10, Poster Room

### [2P-187]

**Augmented evaporative cooling by wetted inner clothing and ventilation garments reduces heat strain in hot-dry and warm-humid environments**

*Ken Tokizawa[1] ([1]*National Institute of Occupational Safety and Health, Japan*)

The present study examined 1) the effect of wearing a water-soaked inner t-shirt with a ventilation garment on heat strain in undertaking moderate-intensity exercise (metabolic rate: 200 W/m$^2$) in a hot environment (37 °C/50% RH), and 2) whether the augmented evaporative cooling were effective in hot-dry (40 °C/30% RH) and warm-humid (32 °C/80% RH) environments. In study 1, while intermittent walking in hot conditions for 60 min, eight male subjects wore a dry inner t-shirt without fanning of a ventilation jacket as a control (CON). Meanwhile, under a fanned ventilation jacket, the subject wore a dry inner t-shirt (DRY) or an inner t-shirt soaked with 350 mL of tap water (WET). Increases in rectal temperature in the WET trial (0.7 ± 0.2 °C) were lower than in the CON (1.3 ± 0.3 °C) and DRY (1.1 ± 0.2 °C) (both $p<0.05$) trials during exercise in hot conditions. Heart rate and sweat loss were lowest in the WET, followed by DRY, and then CON conditions in both groups (all $p<0.05$). In study 2, the attenuated elevation in rectal temperature, heart rate, and sweat loss between CON and WET were observed in both hot-dry and warm-humid environments. These findings demonstrate that wearing a water-soaked inner t-shirt while using a ventilation garment is an effective and practical cooling strategy to mitigate heat strain during moderate-intensity exercise in hot conditions.

### [2P-188]

**Exercise training of paternal mice subjected to continuous psychological stress prior to mating may modify changes in their offspring's emotional behaviors**

*Noriyuki Shimizu[1,2], Sachiko Chikahisa[2,3], Tetsuya Shiuchi[2], Hiroyoshi Sei[2] ([1]*Dept Anat Cell Biol, Inst of Biomed Sci, Grad Sch Univ of Tokushima, Tokushima, Japan.*, [2]*Dept Physiol, Inst of Biomed Sci, Grad Sch Univ of Tokushima, Tokushima, Japan.*, [3]*Dept Hlth Nut, Fac of Hum Life Sci, Univ of Shikoku, Tokushima, Japan.*)

Psychological stress is thought to be one of the factors that induces disturbances in emotional behavior, and affect brain functions in the next generation, but the mechanism is unclear. In this study, we investigated how exercise training on psychologically stressed paternal mice induces changes in the emotional behavior of their offspring. Male C57/BL6 mice aged 8-9 weeks were subjected to restraint stress for 2 hours (started at zeitgeber time 0) using a 50-mL conical centrifuge tube. Stress period was 2 weeks. After the restraint stress, the mice were subjected to a 1-hour treadmill run. After these processes, male mice were mated with same aged virgin females for only 2 days. When the offspring has reached 10 weeks of age, emotional behaviors was examined by using several behavioral analysis systems. The restraint-stressed mice showed depression-like behavior, and stronger fear response to electrical stimulation than non-stressed group. The impairment of emotional behaviors observed in restraint-stressed group was improved by application of treadmill running to paternal mouse during stress period, and which was nearly equal level observed in non-stressed group. These results indicate that the impairment of offspring's emotional behaviors derived from paternal mice exposed to psychological stress prior to mating might be recovered by exercise training.

### [2P-189]

**The anorectic effect of central administered xenin was partially mediated by the nesfatin-1 in rat**

*Shota Saito[1], Hirofum Hashimoto[1], Norie Hamaguchi[1], Sangjon Pae[1], Shinpei Saito[1], Yoshie Reien[1], Yuri Hirayama[1], Naohiko Anzai[1] ([1]*Chiba University Graduate School of Medicine School of Medicine Department of Pharmacology*)

The hypothalamus contains many appetite-related neuropeptides, which form a neuronal network to regulate feeding behavior. Xenin is a 25-amino acid peptide identified in human gastric mucosa. Xenin is widely expressed in peripheral and central tissues. Central and peripherally administered xenin decreased food intake in rodents. The central mechanism of xenin-induced anorexia has been unclear yet. Nesfatin-1/NUCB2 (nesfatin-1) was identified as an anorexic neuropeptide consisting of 82 amino acids. Nesfatin-1 is widely expressed in peripheral organs and the central nervous system. Nesfatin-1 has been reported to co-localize with many peptides in each nucleus. We examined the effect of icv administration of xenin on nesfain-1-like immunoreactive (LI) neurons, on food intake and water intake in rats. Fos-LI neurons were observed in the supraoptic nucleus (SON), paraventricular nucleus (PVN), arcuate nucleus (Arc), and nucleus of the solitary tract (NTS) after icv administration of xenin. In double immunohistochemistry for Fos and nesfatin-1, nesfatin-1-LI neurons expressing Fos were significantly increased in the SON, PVN, Arc, and NTS compared with a control group after icv administration of xenin. Anorexigenic effects of xenin were attenuated by nesfain-1 antisense pretreatment. These results suggested that central nesfatin-1 neurons may be involved in xenin-induced anorexia in rats. The authors declare no conflicts of interest associated with this poster.

### [2P-190]

**Effects of developmental exposure to polyhalogenated dibenzofuran on ultrasonic vocalization in newborn mouse pups.**

*Fumihiko Maekawa[1], Eiki Kimura[1,2], Go Suzuki[1], Naoto Uramaru[3], Masaki Kakeyama[4] ([1]*National Institute for Environmental Studies*, [2]*Japan Society for the Promotion of Science*, [3]*Nihon Pharmaceutical University*, [4]*Waseda University*)

There is concern that developmental exposure to environmental chemicals affects children's brain and induces neuropsychological diseases later. Developmental exposure to dioxins, even at low concentrations, is thought to affect neurodevelopment, and therefore environmental dioxin concentrations should be reduced. Although significant improvement in the concentration of chlorinated dioxins in the atmosphere has been reported with the improvement of incinerators, on the other hand, it has been also reported that brominated dioxins are generated in the recycling process of wastes containing brominated flame retardants, and a risk assessment is required. The purpose of this study was to evaluate the developmental neurotoxicity of 1,2,3,7,8-tetrabromodibenzofuran (Penta-BDF) and 2-Cl-3,7,8-Br-dibenzofuran (Tetra-C/BDF), whose presence in the environment has recently been reported, and to provide toxicity information useful for risk assessment. Penta-BDF or Tetra-C/BDF was administered orally to mice at 12.5 days gestation, and ultrasonic vocalizations of the pups were studied. We found that the number and duration of ultrasonic vocalizations were significantly reduced in the Tetra-C/BDF-exposed group. Our unpublished data indicate that the amount of Penta-BDF transferred to the brain is less than that of Tetra-C/BDF, and it is possible that differences in brain transfer may affect behavior. Previous studies have also shown that chlorinated and brominated dioxins cause a decrease in ultrasonic vocalizations, and the results of this study were consistent with those of previous studies.

### [2P-191]

**Magnetic vestibular stimulation amplifies posture and arterial pressure control**

*Kunihiko Tanaka[1], Akihiro Sugiura[2] ([1]*Graduate School of Health and Medicine, Gifu University of Medical Science*, [2]*Department of Radiotechnology, Gifu University of Medical Science*)

It is known that stochastic resonance phenomenon can be seen in vivo. According to the input, applying weak noise to the signal inputs sometimes improves the response output.
Nystagmus often occurs in the MRI system with 1.5 or 3T, which is used medical imaging, and the patient complains of dizziness. It is thought that a stochastic resonance may be caused by a weaker magnetic stimulus. In this study, we investigated the effects of lower magnetic vestibular stimulation on posture and arterial pressure (AP) control those are related to the vestibular system.
Twelve healthy subjects were recruited. Posturography was measured in eight subjects placed upright on a rubber foam with their eyes closed. AP change at the onset of head-up tilt (HUT), that is related to the vestibular function were measured in four subjects. A 0.4 T magnets or aluminum disks were attached behind the auricle. The average trajectory length, sway area, frequency components of the X and Y directions of posturography were significantly decreased with the magnets, compared to those with aluminum disks. Mean AP at the onset of HUT did not change without the magnet, but increased with the magnet. Magnetic stimulation around the inner ear was suggested to amplifies the vestibular-related reflex.

### [2P-192]

### Effects of monoacyl glycerol lipase deficiency on fever induced by Zymosan

*Ayato Nakata[1], Kouki Kuniyoshi[1], Kenji sakimura[2], Masanobu Kano[3], Kiyoshi Matsumura[1] (*[1]Dept of Biomedical Engineering, Osaka Institute of Technology, [2]Dept of Cellular Neurobiology, Brain Research Institute, Niigata University, [3]Dept of Neurophysiology, Graduate School of Medicine, The University of Tokyo*)

Fever is mediated by prostaglandin E2 (PGE2) in the brain. PGE2 is produced from arachidonic acid (AA) by the actions of cyclooxygenase-2 (COX-2) followed by prostaglandin E synthase. It had been unclear how AA is supplied to COX-2 during fever. In 2015, two groups demonstrated that AA is supplied from 2-arachidonoyl glycerol by the action of monoacyl glycerol lipase (MGL) during fever induced by lipopolysaccharide (LPS). It is still unclear, however, if MGL is responsible for fever induced by pyrogens other than LPS. In this study, we examined the involvement of MGL in fever induced by zymosan, a cell wall component of yeast. In wild-type mice and MGL-deficient mice, zymosan was injected subcutaneously in the hind paw plantar. Peritoneal temperatures were recorded with a telemetry system under free-moving conditions. In wild-type mice, zymosan induced fever 3-9 hours after administration. MGL-deficient mice also showed similar increases in peritoneal temperature that was not statistically different from those of wild-type mice. On the other hand, LPS-induced fever was suppressed in MGL-deficient mice as reported earlier. These results suggest that there is a difference in the AA supply pathway between zymosan- and LPS-induced fever.

### [2P-193]

### Cerebral blood velocity with or without warning stimuli before the start of exercise

*Sayuri Chishiro[1], Keiko Yamamoto[1], Manabu Shibasaki[1] (*[1]Nara Women's Univ.*)

### Poster Presentation

[2P]
### Physical fitness and sports medicine

March 15 (Wed.), 12:10 - 14:10, Poster Room

### [2P-194]

**Microstructural Organization of the Corpus Callosum in Young Endurance Athletes: A Global Tractography Study**

*Takashi Tarumi[1,2], Marina Fukuie[1,2], Yamabe Takayuki[1,2], David Zhu[3], Keigo Ohyama-Byun[2], Seiji Maeda[2,4], Jun Sugawara[1,2] ([1]*National Institute of Advanced Industrial Science and Technology,* [2]*University of Tsukuba,* [3]*Michigan State University,* [4]*Waseda University*)

Aerobic exercise training has been shown to improve microstructural organization of the corpus callosum (CC); however, evidence of this topographic effect is limited. Purpose: To compare the CC microstructural organization between endurance athletes and sedentary adults using a white-matter fiber tractography approach. Methods: Diffusion tensor imaging (DTI) data were collected from 15 young endurance athletes and 16 age- and sex-matched sedentary adults and analyzed with global probabilistic tractography based on neighborhood anatomical information. Fractional anisotropy (FA) and mean, radial, and axial diffusivities were measured in the eight CC tracts. Cortical thickness of the CC tract cortical endpoints was also measured. Physical activity level was assessed by metabolic equivalents. Results: The athlete group had an average VO2max of 69.5±3.1 ml/kg/min. Compared with the sedentary group, athletes had higher FA in the body's premotor and parietal tracts and the splenium. The voxelwise analysis confirmed that the athlete group had higher FA in the CC and other white matter regions than the sedentary group, including the corona radiata, internal capsule, and superior longitudinal fasciculus.
The cortical thickness of the CC tract endpoints was similar between both groups. Physical activity levels were positively correlated with FA in the body's parietal (r = 0.486, p = 0.006) and temporal (r = 0.425, p = 0.017) tracts and the splenium (r = 0.408, p = 0.023). Conclusion: Aerobic exercise is associated with a higher microstructural organization of the CC tracts connected to the sensorimotor and visual cortices.

### [2P-195]

**Lower limb muscle activity during squat exercise under optokinetic stimulation with a head-mounted display**

*Junya Komagata[1], Atsushi Sugiura[2], Atsuya Otsuka[1], Yuki Komatsu[1], Toshihiro Kitama[2] ([1]*Dept of Physical Therapy, Health Science University,* [2]*Center for Life Science Research, Univ of Yamanashi*)

Strength loss is a common and critical impairment after a stroke. Muscle strengthening and weight-bearing (WB) adjustments are important for stroke rehabilitation. This study examined the effects of optokinetic stimulation (OKS) via a head-mounted display (HMD) on postural stability, WB, and electromyography (EMG) from the lower limbs in normal participants.
Six healthy students wearing HMD were asked to repeat a metronome-paced squat with an approximately 60° knee flexion and extension. Regarding OKS, a random dots pattern motion in virtual three-dimensional space was presented in horizontal or torsional directions (HOKS or TOKS). The center of pressure position (CoP) and sway mean position (SM) were measured for WB evaluation. EMG was recorded from four limb muscles—lateral and medial vastus, semitendinosus, and femoral biceps. During TOKS, CoP and SM clearly shifted to the OKS direction, and mean EMG activities in the same direction showed a clear increase compared to those during stationary OKS. By contrast, no clear change was observed in either WB or EMG during HOKS. The results indicated that, during squat, OKS could effectively increase EMG activity of the lower limbs in the OKS direction, suggesting that the squat-OKS combination might be useful for resistance training of paralyzed limb muscles in patients with strokes.

### [2P-196]

**Effects of 5-month interval walking training on cognitive function in middle-aged and older people: a randomized controlled study**

*Mayuka Furihata[1,4], Mayuko Morikawa[1,3,4], Ryoichi Hayashi[1], Wayumi Tomita[5], Hiroshi Nose[1,2,4], Shizue Masuki[1,3] ([1]*Sports Medical Sciences,* [2]*e-Health Sciences, Shinshu University Graduate School of Medicine,* [3]*Institute for Biomedical Sciences,* [4]*Jukunen Taiikudaigaku Research Center,* [5]*Health and Welfare, Yurihonjo*)

We examined whether 5-mo interval walking training (IWT) improved cognitive function (CF) with increased physical fitness in middle-aged and older people. We randomly divided 202 participants (n=202, ~65 yr) into the control (CNT) and IWT groups. Subjects in the CNT group were instructed to maintain a sedentary lifestyle while those in the IWT group were instructed to perform IWT, repeating ≥5 sets of fast and slow walking for 3 min each at ≥70% and 40% peak aerobic capacity ($VO_{2peak}$), respectively, per day ≥4 days/wk for 5 mo. Before and after the intervention, we measured $VO_{2peak}$ and CF with Urakami-style Screening Test. Since 5 subjects dropped out during the intervention and additionally 41 subjects lacked ≥1 of the measurements, we analyzed the remaining 156 subjects (CNT, n=81 and IWT, n=75). Moreover, since subjects who marked ≤12 points in CF were clinically defined as mild cognitive impairment, they were used for further analyses (CNT', n=15 and IWT', n=18). After the intervention, $VO_{2peak}$ and CF increased by 3.8±1.2% and 3.9±3.0% in the IWT group, more than -2.7±1.2% and -7.7±2.6% in the CNT group, respectively (P<0.004). These effects were enhanced in the mild cognitive impairment subjects, 7.4±2.2% and 33.2±6.5% in the IWT' group vs -2.4±2.4% and 3.4±7.1% in the CNT' group, respectively (P<0.006), with positive correlation between changes in $VO_{2peak}$ and CF (P<0.001). The 5-month IWT improved CF with increased $VO_{2peak}$, especially in the mild cognitive impairment individuals.

### [2P-197]

**Polyamine metabolism in atrophying skeletal muscle**

*Hideki Yamauchi[1], Shigeru Takemori[1] ([1]*The Jikei University School of Medicine*)

Aim: Polyamines are reported to regulate skeletal muscle growth and atrophy. To study polyamine metabolism in atrophying skeletal muscle, we observed polyamine metabolizing enzymes in soleus muscle of hindlimb unloaded rats.Methods: Adult (6 months, n=42) F344 female rats were randomly divided into control and unloading groups. Rats of the unloading group have their hindlimbs unloaded by tail-suspension for 1, 3, and 8 weeks. Protein expressions in soleus muscle were examined with western blotting.Results: Muscle mass progressively decreased with unloading by 19, 48, and 56% in 1, 3, and 8 weeks, respectively. Histological disorders were evident in 3 weeks and prominent in 8 weeks. The progress of biochemical processes of atrophy was also evident from a decrease in translation initiation factors and an increase in muscle-specific ubiquitin ligase and ubiquitinated proteins. These changes were found to be accompanied by significant alterations in polyamine metabolizing enzyme system that likely to decrease one of the biologically abundant polyamine, spermidine. Conclusion: Performance dependent modulation of polyamine metabolism may be involved in the process of muscle atrophy.

### [2P-198]

**Roles of interleukin-4 in myoblast fusion via induction of myomerger expression**

*Mitsutoshi Kurosaka[1], Yung-Li Hung[2], Shuichi Machida[3], Kazuhisa Kohda[1]
([1]*Department of Physiology, St. Marianna University School of Medicine,* [2]*Institute of Health and Sports & Medicine, Juntendo University,* [3]*Graduate School of Health and Sports Science, Juntendo University*)

Myoblast fusion is essential for development, growth, and regeneration of skeletal muscle. Recently, two membrane proteins, myomaker and myomerger, which drive myoblast fusion, have been identified. However, the regulation of these proteins remains unclear. Since, interleukin-4 (IL-4) promotes myoblast fusion, as we reported previously, the two proteins might be regulated by IL-4 signaling. In this study, we demonstrated that the differentiation and fusion of C2C12 cells through the induction of MyoD, myogenin, and myomerger was promoted by IL-4. Knockdown of IL-4 receptor alpha (IL-4RαKD) by small interfering RNA impaired myoblast fusion. We also demonstrated the reduction in expression of MyoD, myogenin, and myomerger in IL-4RαKD C2C12 cells. Finally, we elucidated that IL-4RαKD C2C12 cells fused with wild-type C2C12 cells in the cell mixing assay. Collectively, these results suggest that the IL-4/IL-4Rα axis promotes myoblast fusion via induction of MyoD, myogenin and myomerger expression. (COI: None)

### [2P-199]

### Interval walking training over 10 years prevents age-associated declines in physical fitness and improves lifestyle-related diseases

*Mayuko Morikawa[1,3,4], Shizue Masuki[1,3,4], Shunichi Furuhata[1,4], Hirokazu Shimodaira[1,4], Mayuka Furihata[1,4], Hiroshi Nose[2,4] (*[1]Sports Medical Sciences, [2]e-Health Sciences, Shinshu University Graduate School of Medicine, [3]Institute for Biomedical Sciences, Shinshu University, [4]Jukunen Taiikudaigaku Research Center*)

Exercise training above a given intensity throughout the lifespan is the most effective strategy to prevent age-associated declines in physical fitness. We assessed the effects of 10-year continuation of interval walking training (IWT) on peak aerobic capacity ($VO_{2peak}$) and lifestyle-related disease (LSD) symptoms in older people. One hundred eighty men (~68 yr) and 445 women (~63 yr) started IWT in April of 2005 or 2006 and thereafter, we measured $VO_{2peak}$ and LSD symptoms every year for 10 years. We assigned 49 men and 76 women who continued IWT for 10 years with no intermission as a 10-year IWT group (10-year IWT) while 131 men and 369 women who dropped out as a DO group (DO). Separately, we assigned a control group (CNT) by selecting subjects from 212 men and 474 women before starting IWT in both groups so that their number and age distribution were matched to those at every year of 10-year IWT. We found that $VO_{2peak}$ in CNT gradually decreased with aging ($P<0.001$). In contract, $VO_{2peak}$ in 10-year IWT sharply increased for the 1st year and thereafter gradually decreased but remained higher than in CNT in both sexes (both, $P<0.001$), which was negatively correlated with the LSD symptoms (both, $P<0.001$). The profile of $VO_{2peak}$ in response to IWT before dropping out in DO was similar to that in 10-year IWT in both sexes (all, $P>0.19$). Thus, the 10-year IWT continuation markedly prevented an age-associated decline in $VO_{2peak}$ with improved LSD symptoms.

### [2P-200]

### Cross-adaptation of the muscle by lengthening contraction observed in the animal model of delayed onset muscle soreness.

*Masayo Suzuki[1], Teruaki Nasu[2], Kimiaki Katanosaka[1,2] (*[1]Graduate School of Life and Health Sciences, Chubu University, [2]College of Life and Health Sciences, Chubu University*)

Although an unaccustomed exercise results in delayed-onset muscle soreness (DOMS), the muscle adapts to the exercise to protect against subsequent damages. This phenomenon, called the repeated bout effect, has been reported to show a cross effect in human, in which a unilateral exercise produces adaptive effect on the unexercised contralateral side of the body (cross-adaptation). However, its underlying mechanisms have not yet been elucidated, because it has not examined using animal models. Here, to elucidate the mechanism of cross-adaptation of the muscle using animal model, we examined whether cross-adaptation occurs in a rat model of DOMS. Lengthening contraction (LC) was applied to the flexor muscles in right lower leg of rats, and, after 5 days, the second LC was applied to the left leg. We measured mechanical withdrawal threshold of the muscle in the left leg by Randall-Selitto test, before and 2days after the second LC, but we could not observe decrease of the threshold, suggesting occurrence of cross-adaptation by LC to the contralateral side. In the present study, we showed that the animal model of DOMS could be used to analyze the mechanisms of cross-adaptation of muscle soreness after unilateral exercise.

### [2P-201]

### Five-month interval walking training and bone mineral density (BMD) in postmenopausal women: impact of baseline BMD and exercise intensity

*Rizka Nugraheni Martyanti[1], Mayuko Morikawa[1,4,5], Yukio Nakamura[3], Masaaki Hanaoka[2,5], Hiroshi Nose[2,5], Shizue Masuki[1,4,5] (*[1]Department of Sports Medical Sciences, Shinshu University Graduate School of Medicine, [2]Department of e-Health Sciences, Shinshu University Graduate School of Medicine, [3]Department of Orthopaedic Surgery, Shinshu University School of Medicine, [4]Institute for Biomedical Sciences, Shinshu University, [5]Jukunen Taiikudaigaku Research Center*)

We examined whether interval walking training (IWT) increased bone mineral density (BMD) in postmenopausal women (PW) while focusing on baseline BMD and fast walking intensity. PW (n=234, ~64 yr) performed IWT for 5 mo. We measured BMDs for the lumbar spine (LS) and femoral neck (FN) by DEXA before and after IWT. By multiple regression analysis, we found that the baseline was a significant predictor for an increase in BMD after training for LS or FN (both, $P<0.01$) and the intensity (% peak aerobic capacity, $\%VO_{2peak}$) was that for LS ($P<0.05$), where the increase in BMD was negatively correlated with the baseline for LS or FN (both, $r= -0.92$, $P<0.05$) while positively correlated with the intensity for LS ($r = 0.93$, $P<0.05$). Accordingly, we determined cut-off values for each predictor by ROC method, which was baseline BMD of 0.961 g/cm² for LS and 0.709 g/cm² for FN and $\%VO_{2peak}$ of 85% for LS. When we determined the change in BMD for LS or FN after training while dividing the subjects into low and high groups according to the cut-off values, we confirmed that BMD increased in the low group of baselines for LS or FN while in the high group of $\%VO_{2peak}$ for LS (all, $P<0.05$), with significant differences between the high and low groups, respectively (all, $P<0.01$). Thus, IWT for 5 mo increased BMDs in PW who had lower BMDs than the given baseline for LS or FN and who performed IWT above the given $\%VO_{2peak}$ for LS.

### [2P-202]

### Effect of maximal isometric contraction at different ankle angles on muscle anabolism, mass, and strength

*Taiga Mishima[1,2], Sota Sugimura[1], Riki Ogasawara[1,2] (*[1]Nagoya Institute of Technology, [2]National Institute of Advanced Industrial Science and Technology*)

Although joint angle affects force-length relationships, it is unclear whether differences in joint angle during muscle contraction affect muscle size and strength adaptation. The purpose of this study was to investigate the effects of differences in contraction intensity depending on joint angle on acute muscle anabolism, muscle mass, and force adaptation. According to ankle joint angle during muscle contraction, male Sprague-Dawley rats were grouped into 20° dorsiflexion (DF) and 40° plantar flexion (PF) groups. The right gastrocnemius muscle was contracted isometrically via percutaneous electrical stimulation, and the left gastrocnemius muscle served as a control. The number of sets was modified to match the force-time integral between groups. Acute muscle contraction increased the phosphorylation of p70S6K, 4E-BP1, and rpS6, which are downstream of mTORC1 signaling, and protein synthesis in both groups, the extent of increase in phosphorylation of p70S6K and rpS6 was greater in the PF group. While chronic muscle contraction increased gastrocnemius muscle mass similarly in both groups, muscular strength was higher in the DF group than in the PF group. Considering that the force-time integral was matched between groups, differences in contraction intensity and/or joint angle may affect strength adaptation independent of muscle size.

### Poster Presentation

### [2P]
### Nutritional and metabolic physiology, Thermoregulation

March 15 (Wed.), 12:10 - 14:10, Poster Room

### [2P-203]

**Effects of 5-aminolevulinic acid phosphate with iron supplementation on exercise-induced blood lactate responses and endurance exercise performance in young men.**

*Shigeki Ikegawa[1], Tsubasa Kondo[1], Mitsuhiro Ueno[1], Atsuko Kamiya[2], Kiwamu Takahashi[2], Masahiro Ishizuka[2] (*[1]Joetsu Univ of Educ, Grad Sch of Educ, [2]SBI Pharmaceuticals Co., Ltd.*)

Aims: We examined whether 5-aminolevulinic acid phosphate with iron (ALA) intake affected blood lactate response and endurance exercise performance. Methods: We conducted two experiments. In the laboratory experiment, eight young men underwent two trials for 10 days each in which they performed aerobic training at the 70 % peak oxygen consumption rate with ALA or placebo (CNT) intake. Before and after each trial, subjects underwent a graded cycling test, and blood lactate was measured. In the field experiment, eight young men completed a 10-min running time trial before and after two separate 7-day periods of supplementation, ALA or CNT. Exercise performance was evaluated by the running speed of the time trial. Results: In the laboratory experiment, the lactate threshold after the intervention shifted to a higher value in the ALA trial (P = 0.046). In the field experiment, the running speed of the time trial significantly increased after the ALA supplementation period (P = 0.0231). These effects were not observed in the CNT trial, respectively. Conclusions: ALA intake might improve endurance exercise performance by enhancing aerobic metabolism. COI: The COI has been reported to the conference secretariat.

### [2P-204]

**Role of gut microbiota formation in the induction of beige adipocytes in pre-weaning mice**

*Anju Tsukada[1], Yuko Okamatsu-Ogura[1], Mira Suzuki[1], Emi Futagawa[1], Yuko Kato[2], Yuki Habu[1], Natsumi Takahashi[1], Satoshi Ishizuka[2], Kei Sonoyama[3], Kazuhiro Kimura[1] (*[1]Laboratory of Biochemistry, Faculty of Veterinary Medicine, Hokkaido University, [2]Laboratory of Nutritional Biochemistry, Research Faculty of Agriculture, Hokkaido University, [3]Laboratory of Food Biochemistry, Research Faculty of Agriculture, Hokkaido University*)

Intestinal microbiota has been suggested to influence host metabolism by modulating adipose tissue biology. We examined the role of gut microbiota formation during pre-weaning period on adipose tissue development in mice. In pre-weaning pups, the expression level of beige adipocyte marker *Ucp1* in inguinal white adipose tissue (iWAT) gradually increased after birth and peaked at postnatal day 17. The induction of beige adipocytes with multilocular lipid droplets was also confirmed by histological analysis. Metagenomic analysis showed the drastic change in gut microbiota during pre-weaning period. When gut microbiota formation in pups was disrupted by the treatment of dam with antibiotics, the expression of *Ucp1* in iWAT at postnatal day 17 was significantly reduced compared to the normal pups. In addition, antibiotic treatment significantly decreased the expression of *Cyp7a1*, a rate-limiting enzyme in bile acid synthesis, in liver and changed bile acid profiles in serum. These results indicate that the pre-weaning gut microbiota formation is important for the beige adipocytes induction, suggesting the possible role of bile acids.

### [2P-205]

**Interactive effect of estrogen and leptin on palatable sucrose solution in ovariectomized rats**

*Natsumi Kosugi[1], Sayaka Eguchi[1], Ayano Oka[1], Mayuna Nagata[1], Akira Takamata[2] (*[1]Graduate School of Humanities and Sciences, Nara Women's University, [2]Department of Environmental Health, Nara Women's University*)

Estrogens are known to exert hypophagic and antiobesity actions. We have reported that estrogens also enhance palatable 10% sucrose solution intake. Leptin attenuates food intake and body fat accumulation. In the present study, we examined the interactive effect of estrogen and leptin on 10% sucrose solution (SS) and energy intakes. Female Wistar rats were ovariectomized and implanted a silicon capsule containing estradiol (E2) or cholesterol, and these rats were then continuously infused with leptin (Lep; 38.4 µg/day) or saline using a subcutaneous osmotic pump for 14 days. All rats were provided access to 10% SS, water, and standard chow, and intakes of these were measured. E2 decreased body weight but Lep with the dose in the present study did not. E2 enhanced SS intake and percent of energy intake from the SS in total energy intake, and Lep enhanced SS intake only with E2 replacement. The results suggest that low concentrations of leptin may synergistically act with estrogen to increase sucrose preference.

### [2P-206]

**Ameliorative effects of high-protein ketogenic diet on abnormal behaviors in rats with neonatal dopamine depletion**

*Masanori Ogata[1], Kei Eto[1], Hitoshi Ishibashi[1] (*[1]Department of Physiology, School of Allied health Sciences, Kitasato University*)

Standard ketogenic diet (KD) is a low carbohydrate, moderate protein and high fat diet. Nowadays, there are several types of KD. High-protein ketogenic diet (HPKD) is similar to the standard KD but includes more protein, and is effective for treatment-resistant epilepsy. Effects of HPKD on other neurological disorders are still unknown. In the present study, effects of HPKD on abnormal behaviors in rats with neonatal dopamine (DA) depletion induced by 6-hydroxydopamine treatment were investigated using open field (OF), elevated plus maze (EPM) and 24-hour home cage (24-h) tests. Rats were fed normal diet (ND) or HPKD for 5 weeks after weaning. In the OF and/or EPM tests, ND-fed rats with DA depletion showed increases in locomotor activity, and decreases in anxiety-related and exploratory behaviors. The ND-fed rats with DA depletion also showed a decrease in locomotor activity in the 24-h test. The abnormal locomotor activities induced by neonatal DA depletion in the OF and 24-h tests were ameliorated by the HPKD feeding, but not the exploratory behavior. In the present study, there was no significant difference in the behaviors between control rats fed with the ND and HPKD. These results suggest that the HPKD may ameliorate several symptoms of DA-related disorders. COI: NO

### [2P-207]

**Physiological analysis of spontaneous dwarf mutant mice**

*Takahiro Sato[1], Kanae Oishi[1], Masayasu Kojima[1] (*[1]Institute of Life Science, Kurume University*)

Purpose: Spontaneous dwarf mutant mice (dwarf mice) were incidentally obtained in our laboratory. However, it is not known what kind of abnormality they have other than dwarf features. In this study, we will analyze the physiological functions of these dwarf mice to clarify what kind of abnormalities they have.Materials and Methods: All experiments were performed by comparing dwarf mice with normal mice born from the same mother. In the glucose tolerance test, glucose (1 g/kg) was administered, and blood was collected over time to measure blood glucose and insulin levels. Biochemistry blood tests were also measured using VetScan (ABAXIS). The high-fat diet was High-Fat Diet (D12492, EP Trading) fed ad libitum.Results: Compared to normal littermate mice, the dwarf mice had lower blood glucose levels at rest, and the glucose levels were quickly lowered by the glucose tolerance test. The blood insulin level was not abnormal and showed the same trend as that of the blood glucose level. The dwarf mice did not become obese even after being fed a high-fat diet.Discussion: Since dwarf mice are able to live their entire life in good health, the high glucose tolerance exhibited by dwarf mice is thought to be due to an unknown regulation of glucose metabolism.

### [2P-208]

### Transcriptome and lipidome analyses of mouse Harderian glands in aging

*Takehito Uruno[1], Masatomo Takahashi[1] ([1]*Kyushu Univ., Medical Institute of Bioregulation*)

The Harderian gland (HG) is a lipid-rich endocrine/exocrine orbital gland that is closely related to the lacrimal gland. In addition to supplying tear lipids for protecting the ocular surface, HG has been implicated in the maintenance of homeostasis through various biological functions, such as thermoregulation, feeding behavior, weight control, immune regulation, interaction with the pineal gland, etc. However, the underlying molecular mechanisms have not been fully elucidated. Here we performed a cross-tissue transcriptome analysis of eleven mouse organs and tissues, and found that a set of genes involved in de novo lipogenesis were robustly upregulated in HG, indicating that HG is a tissue specialized in de novo lipogenesis, comparative to the liver. In particular, members of the SCD (stearoyl-CoA desaturase) and ELOVL (elongation of very long-chain fatty acids) families, both implicated in long-chain fatty acid synthesis, adipogenesis, insulin sensitivity, and obesity, were distinctively expressed. The result was supported by lipidome analysis of HGs. We are currently analyzing the transcriptomic and lipidomic changes in aging HGs. Through the analyses, we aim to identify specific lipid-metabolizing enzymes and bioactive lipids that play a key role in age-related biological functions of HG. (COI: NO)

### [2P-209]

### Rare Sugar Modulation on Hepatic FGF21 Expression Controls Weight Gain in C57BL/6 Mice Fed with High-Fat High-Sucrose Diet

*Oulan Gustav Hakim Nata Buana Efendi[1], Sho Matsui[1], Yasuo Oguri[1], Satoshi Tsuzuki[1], Tsutomu Sasaki[1] ([1]*Laboratory of Nutrition Chemistry, Division of Food Science and Biotechnology, Graduate School of Agriculture, Kyoto University*)

Excessive intake of high-fat high-sucrose diet (HFHSD) promotes obesity and impairs glucose control. Simple sugar intake is regulated by a negative feedback signaling, consisted of hepatic fibroblast growth factor 21 (FGF21) and hypothalamic oxytocin neurons. We identified that D-tagatose, D-allulose, and D-sorbitol as *Fgf21* inducers using primary mouse hepatocytes. One month *ad libitum* supplementation with rare sugar solutions significantly reduced caloric intake and weight gain accompanied by smaller adipocyte size, and improved glucose tolerance and insulin sensitivity. Among the sugars, D-allulose showed the most potent effect. Mechanistically, the *Fgf21* promoter luciferase reporter assay revealed that the region at approximately -1050 bp relative to the transcription start site is required for the effect of rare sugars. Animal TFDB 3.0 database predicted that the region contains a binding element for interferon regulatory factor 5 (IRF5). The rare sugars' effect was abrogated by deletion and mutation of the IRF5-binding element, but not by the knock down of *Irf5*, indicating that unreported transcription factors may mediate the rare sugar-induced *Fgf21* transcription through this region.

### [2P-210]

### Tumor suppressor homologue *let-7* that is LET7 micro RNA is regulated across generations by starvation in *C. elegans*.

*Luna Izuhara[1], Sawako Yoshina[1], Shohei Mitani[1] ([1]*Tokyo Women's Medical University*)

The effects of starvation on the next generation are thought to be due not to changes in gene sequence, but to changes in epigenetic status, that is, regulation of chromosomal structures involved in gene expression. *let-7* is a microRNA found in *C. elegans*, whose human homologue functions as a tumor suppressor. Recent studies in *C.elegans* suggest an intrinsic strategy in which parental experiences during developmental stages form transmissible epigenetic memories, that elicit enhanced robustness and viability in their descendants. In this study, we investigated the transgenerational inheritance of the expression control of the nematode homologous gene *let-7*. We used a temperature-sensitive *let-7* mutant allele, which has sterile and vulval malformation at the restrictive temperature. We found that starvation suppresses the phenotypes of *let-7* mutant animals. Also, starvation changes the expression stage of *let-7*; this effect is inherited through the F4 generation. In addition, we found that downregulating the expression of certain genes involved in epigenetics repressed the infertility phenotype of *let-7* mutant animals. Thus, the regulation of *let-7* expression suggests that dependence of this microRNA on nutrition and epigenetic regulation.

### [2P-211]

### Nos1 neurons in the paraventricular nucleus of the hypothalamus control the circadian rhythm of lipid metabolism

*Kunio Kondoh[1], Yasuhiko Minokoshi[1] ([1]*National Institute for Physiological Sciences, National Institute of Natural Sciences*)

The energy homeostasis keeps the balance among production/intake, utilization and storage of energy substances. For example, there is a circadian rhythm for carbohydrate and lipid utilization: carbohydrate utilization increases during active periods with increased food intake, while lipid utilization increases during resting periods. The communication between the central nervous system, including the hypothalamus, and peripheral tissues is thought to be important in the control of energy homeostasis. However, the mechanism that forms circadian rhythms of energy-consuming substances remains unknown. To identify neurons in the hypothalamus that regulate peripheral tissues, we used transsynaptic viral tracers and found that neurons expressing Nos1 (Nitric oxide synthase 1) in the paraventricular nucleus of the hypothalamus (PVH) project to peripheral tissues important for energy metabolism. Interestingly, activation of PVH Nos1 neurons increased lipid utilization, whereas the inactivation of Nos1 neurons suppressed lipid utilization preferentially during the light periods. Furthermore, Nos1 neurons were activated during the light periods and inactivated during the dark periods. Chronic suppression of Nos1 neuron activity disrupted the circadian rhythms of lipid utilization without affecting total energy expenditure, resulting in the high carbohydrate utilization throughout the day. These results indicate that PVH Nos1 neurons are involved in the formation of circadian regulation of lipid metabolism, which could be important for energy homeostasis.

### [2P-212]

### Acute effects of branched-chain keto acids on blood glucose levels in normal rats

*Kotaro Shibata[1], Yuri Yoshimi[1], Futoshi Furuya[1], Eri Mukai[1] ([1]*Ritsumei University*)

Branched-chain amino acids (BCAA), essential amino acids consisting of valine, leucine, and isoleucine, are converted into branched-chain α-keto acids (BCKA) by aminotransferases. The blood levels of BCAA and BCKA have been reported to be higher in obesity and type 2 diabetes. However, the detailed mechanism is not clear. In this study, we investigated the acute effects of BCAA and BCKA on blood glucose regulation in normal rats. Single BCKA administration enhanced blood glucose levels during the oral glucose tolerance test. Serum insulin levels were not different with BCKA treatment. The blood glucose levels in during the insulin tolerance test were enhanced by BCKA treatment. These results indicate that BCKA attenuates insulin sensitivity. In the pyruvate tolerance test, BCKA treatment significantly enhanced the blood glucose levels. However, glucose production in isolated hepatocytes was suppressed by BCKA treatment. This study showed that BCKA acutely increased blood glucose levels by attenuating insulin sensitivity and enhancing hepatic gluconeogenesis in normal state. The enhancement of hepatic gluconeogenesis *in vivo* is thought to be caused by attenuated insulin sensitivity rather than a direct effect of BCKA. Future studies will examine the mechanism of insulin sensitivity attenuation by BCKA to clarify the relationship between BCKA and blood glucose regulation.

### [2P-213]

### Neuroprotective effects of heat acclimation in a rat model of heat stroke

*Kentaro Matsuzaki[1], Naotoshi Sugimoto[2], Osamu Shido[1] ([1]*Shimane Univ.*, [2]*Kanazawa Univ.*)

Heat acclimation is beneficial in the prevention of heat stroke, but the mechanism remains unclear. In this study, we investigated neuroprotective effect of short-term heat acclimation in a rat model of heat stroke. Male Wistar rats were subjected to an ambient temperature ($T_a$) of 32 ± 0.2°C for 5 days for heat acclimation (HA), while control (CN) rats were maintained at $T_a$ of 25 ± 0.1°C. Rats were then exposed to $T_a$ of 38°C for heat stroke (HS). Rats kept at $T_a$ of 25°C were used as the normal rats (NS). The rats were then anesthetized, and brain and blood were collected. In CN-HS rats, neuronal degeneration, apoptosis-like cell death, and lipid peroxidation were significantly increased in the cerebral cortex, hippocampus, thalamus, and cerebellum, whereas those were significantly inhibited in HA-HS rats. In addition, increased inflammatory cytokines such as interleukin-1b and tumor necrosis factor-a, increased creatinine levels, and decreased platelet counts observed in CN-HS group were significantly suppressed in HA-HS group. Heat acclimation may prevent apoptosis, inflammation and oxidative stress during heat stroke and may exert a neuroprotective effect in rats.

### [2P-214]

### Optogenetic Induction of Hibernation-like state with modified OPN4

*Tohru M Takahashi[1,2], Arisa Hirano[1,2], Takeshi Sakurai[1,2] ([1]*University of Tsukuba,* [2]*WPI-IIIS, Tsukuba, Japan*)

Optogenetics has been used for neuronal manipulation at a high temporal resolution to understand the neuronal mechanisms of many physiologies. Current tools have been reported to be effective for short-term manipulation (seconds to minutes), although it has been challenging for continuous stimulation of neurons on much longer time scale (hours to days) to induce long-term behavioral and physiological responses such as hibernation. In this study, we aimed to establish a method allowing stable 24-hour neuronal manipulation with high reproducibility by using OPN4 (melanopsin), which is a GPCR(Gq)-type photosensor with high-photosensitivity. We recently found that excitatory manipulations of *Qrfp*-expressing neurons in the preoptic area of the hypothalamus (Q neurons) induced a hibernation-like hypothermic/hypometabolic state (QIH) in mice. To control QIH with higher time resolution, we developed an optogenetic method using modified OPN4. The engineered-OPN4 stably and reproducibly induced QIH for at least 24 hours by illuminating low-power light (3 μW, 473 nm laser) with high temporal resolution. The optogenetic method would enable us to identify neural mechanisms underlying long-term dormancy states such as sleep, daily torpor and hibernation.

### Poster Presentation

[2P]

**Behavior, Biological rhythm, Sleep**

March 15 (Wed.), 12:10 - 14:10, Poster Room

### [2P-215]

### Neonatal MK-801 treatment and peripubertal social isolation increase impulsivity in cliff avoidance response test in adult mice

*Hinano Yonemaru[1,2], Takaaki Ozawa[1], Takatoshi Hikida[1] ([1]*Laboratory for Advanced Brain Functions, Institute for Protein Research, Osaka University,* [2]*Graduate School of Frontier Biosciences, Osaka University*)

The dual hit hypothesis of schizophrenia suggests that both the genetic factor of an individual and its developmental environment determine the disease risk in the future. However, the detailed mechanism remains largely unknown. NMDA receptor dysfunction has been considered to be associated with schizophrenia. Supporting this, neonatal injection of MK-801, an NMDA receptor antagonist, induces schizophrenia-like phenotype, such as impaired prepulse inhibition and increased reactivity to psychostimulants, in adult rodents. Furthermore, it has been known that social isolation stress during development also causes psychosis-like abnormalities like increased anxiety and impaired behavioral flexibility. In this study, we examined possible schizophrenia-like behavioral abnormalities in adult mice that experienced neonatal sub-chronic MK-801 treatment and/or peripubertal social isolation. Mice received daily subcutaneous injections of MK-801 from postnatal day (PND) 5 to 8 and/or were socially isolated from PND 28 to 35. As a result, neonatal MK-801 treatment and peripubertal social isolation particularly increased impulsivity in the cliff avoidance reaction test, while locomotor activity in the open-field test and motor function in the rotarod test were not changed. This study suggests developmental NMDA receptor hypofunction and peripubertal social stress trigger elevated impulsivity, which typifies schizophrenia, in later life.

### [2P-216]

### Arginine vasopressin neurons of the suprachiasmatic nucleus act as the principal circadian pacesetter cells in vivo

*Yusuke Tsuno[1], Yubo Peng[1], Shin-ichi Horike[2], Kanato Yamagata[3], Mizuki Sugiyama[4], Takahiro J. Nakamura[4], Takiko Daikoku[5], Takashi Maejima[1], Michihiro Mieda[1]
([1]*Department of Integrative Neurophysiology, Graduate School of Medical Science, Kanazawa University,* [2]*Division of Integrated Omics research, Research Center for Experimental Modeling of Human Disease, Kanazawa University,* [3]*Child Brain Project, Tokyo Metropolitan Institute of Medical Science,* [4]*Laboratory of Animal Physiology, School of Agriculture, Meiji University,* [5]*Division of Animal Disease Model, Research Center for Experimental Modeling of Human Disease, Graduate School of Medical Science, Kanazawa University*)

The central circadian clock of the suprachiasmatic nucleus (SCN) is a network consisting of various types of neurons and glial cells. Individual cells have the autonomous molecular machinery of a cellular clock, but their intrinsic periods vary considerably. Here, we show that arginine vasopressin (AVP) neurons set the ensemble period of the SCN network to control circadian behavior rhythm. Artificial lengthening of cellular periods by deleting casein kinase 1 delta (CK1δ) in the whole SCN lengthened the free-running period of behavior rhythm to an extent similar to CK1δ deletion specific to AVP neurons. In SCN slices, PER2::LUC reporter rhythms of these mice did not recapitulate the period lengthening, showing a dissociation between the SCN shell and core with a period instability in the shell. However, in vivo calcium rhythms of both AVP and vasoactive intestinal peptide (VIP) neurons in the SCN of freely-moving mice demonstrated stably lengthened periods similar to the behavioral rhythm upon AVP neuron-specific CK1δ deletion, without changing the phase relationship between each other. These results indicate that AVP neurons entrain other SCN neurons such as VIP neurons in vivo and thereby act as the primary determinant of the SCN ensemble period.

### [2P-217]

### A smartphone-based system to assess the modification of episodic memory performance.

*Yukari Saito[1], Yokoyama Akane[2], Tabata Toshihide[1] ([1]*Univ. of Toyama,* [2]*Cure Code Corp.*)

Episodic memory (EM) is thought to be sensitive to age. To elucidate the detailed nature and underlying mechanism, it is required to assess EM performance in many people of various ages. Here we devised a smartphone-based system which automatically executes a two-session EM recognition test. In the first session, the system asked the subject to do an encoding task on 90 photographs. In the second session taken place 48 hr later, the system displayed the above photographs and 90 distracting photographs in random order and asked whether the subject remembered each photograph. A comparison in recognition score between 26 young (20-26 yo) and 20 older (60-69 yo) participants showed that the system could detect an age-related EM decline. Moreover, the system had a functionality to examine the effect of post-encoding aerobic exercise (10 min) which has previously been reported to facilitate EM consolidation. The system monitored the suject's heart rate using a wearable terminal and instructed her/him to increase or decrease the exercise intensity so that the heart rate stayed around the level determined by the Karvonen formula (40%). For both younger and older participants, aerobic exercise indeed improved the recognition score. This system would be a powerful tool to promote large-scale EM studies.

### [2P-218]

### Longitudinal analysis of the primate progressive synucleinopathies model revealed prolongation in saccade reaction time

*Wajd Amly[1], Chih-Yang Chen[1], Hirotaka Onoe[1], Masanori Sawamura[1], Ryosuke Takahashi[1], Tadashi Isa[1] ([1]*Kyoto University*)

Recent studies suggested that the α-Synuclein fibrils can lead to synucleinopathies, including Parkinson's disease (PD) and dementia with Lewy bodies. Patients showed oculomotor deficits correlated with the disease progression. However, whether the animal models show similar oculomotor deficits to human patients was not tested. Motivated by this, we trained three marmosets on the externally and the internally driven visual tasks, the gap and the oculomotor delayed response (ODR) tasks respectively. After collecting baseline data, we injected α-Synuclein fibrils into the olfactory bulb (OB) bilaterally, following Braak's dual hypothesis (Heiko Braak, et.al, 2004). With a longitudinal follow-up, we found a gradual prolongation of saccade reaction time (SRT) and deceleration period in gap task after α-Synuclein injection. The marmosets did not show similar progressive symptoms when performing ODR task. Our primate model captured oculomotor deficits which were previously reported in human patients with Lewy body dementia (Mosimann et al., 2005, Yasuo Terao, et.al, 2019). This result suggested that cortical saccade generation may be affected by α-Syn fibrils and further research is needed with this established synucleinopathies primate model.

### [2P-219]

### Changes in the rhythmicity of behaviors in female mice during the pup-raising period

*Atsumi Murakami[1], Hitoshi Okamura[2], Keiko Tominaga[1] ([1]*Osaka university,* [2]*Kyoto university*)

Most organisms on the earth display circadian rhythms (about 24-hr rhythmicities) in their physiological processes and behaviors, which are driven by a circadian clock. Since mice are nocturnal animals, they are active during a dark phase under 12 hr light:12 hr dark (LD) cycles. And in constant darkness (DD), the free-running period of activity rhythms is a little shorter than 24 hr. However, the activity rate decreases in a female mouse raising pups (dam), and its activity rhythm becomes unclear. To elucidate whether the dam's rhythms disappear during raising pups, we observed murine maternal behavior for the postpartum period and investigated its rhythmicity. We focused on crouching posture, which a dam displays to lactate and maintain the body temperature of pups for several weeks after parturition. We recorded murine behaviors using a video camera with infrared light and analyzed crouching posture and feeding behavior. As a result, crouching behavior in WT dams, but not *Per*-null mice (*Per1-/-Per2-/-Per3-/-*), showed robust daily rhythms in LD and DD conditions. During the pup-raising period, feeding behavior in WT dams increased in the light phase of the LD condition. In conclusion, the murine maternal behavior is controlled by a circadian clock, and the rhythmicity of feeding behavior is disrupted during the pup-raising period.

### [2P-220]

**Difference of environmental enrichment condition effect of anxiety-like behavior response time and hindlimb muscle.**

*Mizuki Sudo[1], Yutaka Kano[2], Soichi Ando[2] ([1]*Meiji Yasuda Life Foundation of Health and Welfare,* [2]*University of Electro-Communications*)

[Aim] The purpose of the present study was to examine which components of the environmental enrichment (EE) (i.e., wheel running activity and locomotor activity (LA)) mitigate anxiety-like behaviors and increase skeletal muscle mass. [Methods] Wistar rats were divided into four different housing groups: EE (running wheel, slope, tunnel, and hut), EE with running wheel only (EE-W), EE without running wheel (EE-NW), and standard environment group (SE, N=6 for each group). LA of each rat was continuously recorded using a Nano-Tag. Four weeks later, the rats were submitted to the elevated plus maze test (EPM) to assess anxiety-like behavior. Hindlimb muscles were removed and immediately weighed and examined by immuno-histological staining to asses cross-sectional area. [Results] LA was higher in the EE and EE-W compared with the EE-NW (Ps < 0.05). In the EPM, the OPEN/CLOSE (O/C) ratio of spent time in the EE-W was significantly higher than the EE-NW in the early phase (P=0.005). However, O/C ratios in the later phase were significantly higher in the EE-NW compared with the EE-W (P=0.01). Soleus muscles were greater in the EE, EE-W, EE-NW groups compared with the SE group. The present study suggests that wheel running activity and LA in the absence of wheel running may have differential effects on anxiety-like behavior. Muscle hypertrophy is likely to occur irrespective of LA in the EE.

### [2P-221]

**Comparison of the heart rate during sleep in freely behaving mice acquired by a novel noninvasive electrocardiogram system and a telemetry system**

*Shinichi Sato[1], Nishijima Tsuguo[1] ([1]*Iwate Medical Univ.*)

Purpose: The telemetry system is a gold standard for modern cardiovascular research and literature that the effect of device implantation surgery is negligible after a substantial recovery period. On the other hand, we recently developed a noninvasive electrocardiogram system, which requires no surgery and is suitable for noninvasive electrocardiogram recording in freely behaving mice during sleep. This study sought to determine whether the sleeping heart rate (HR) acquired by the above two systems is the same. Methods: Male C57BL/6 mice aged 9 – 16 w underwent electrocardiogram recording during the daytime using our multi-dry-electrode plate (MDEP) sensor system. Eight recordings that contained sleep stages were used to determine sleeping HR. To determine the sleeping HR for the telemetry system, we estimated it from a figure in the literature. These sleeping HRs were the averaged HR over 10 s. Results: The sleeping HR for the MDEP-sensor system was 351±23 bpm, which was considerably lower than that of 441 bpm for the telemetry system. The mouse group for the telemetry system consisted of ten C57BL/6 mice (5 male and female) aged 10 - 16 w. Conclusion: Further study is needed to clarify whether the telemetry device implantation affects the nervous system control of the heart during sleep after a substantial recovery period.

### [2P-222]

**Analysis of the function of neurotransmitters coexisting with orexin in sleep regulation**

*Yuki Cleo Saito[1,2], Yasutaka Niwa[1,2], Yoan Cherasse[1,2], Takeshi Sakurai[1,2] ([1]*Faculty of Medicine, Univ. of Tsukuba,* [2]*WPI-IIIS, Univ. of Tsukuba*)

Deficiency of orexin causes a severe hypersomnia symptom called narcolepsy. Narcolepsy is characterized by cataplexy and excessive daytime sleepiness. Orexin-deficient mice suffer from behavioral instability, characterized by sudden and abrupt transition from wakefulness (WAKE) to non-REM sleep (NREM) and pathological transition from WAKE to REM sleep (REM). Although orexin KO mice recapitulate the phenotype of narcolepsy, most cases of human narcolepsy are caused by the degeneration of orexin neurons, which express several co-existing neurotransmitters, including glutamate, dynorphin A, and neurotensin. In this study, we aimed to elucidate the functions of neurotransmitters other than orexin that coexist with orexin in orexin-producing neurons. We generated orexin-iCre knock-in mice, in which endogenous orexin alleles were replaced by iCre. In homozygous mice, we histologically confirmed that orexin production was completely knocked out. Furthermore, we found these mice exhibited cataplexy-like symptoms (CLEs) by EEG/EMG analysis. Next, we injected AAV10-FLEx-hM3Dq-mCherry into the lateral hypothalamic area of orexin-iCre heterozygous and homozygous mice to examine the effect of an excitatory manipulation of orexin neurons on the sleep-wake state. When orexin neurons in the heterozygous mice were specifically excited by deschloroclozapine (DCZ) during the light period, there was a marked difference in the amount of NREM sleep and REM sleep up to 3 hours after administration. The total time of both NREM and REM sleep was shorter. On the other hand, in homozygous mice lacking orexin, only the total time of REM sleep was drastically decreased. This result suggests that other neurotransmitter(s) present in orexin neurons other than orexin have a role in inhibition of REM sleep. We are going to analyze the effects of conditional knocking out of candidate factors expressed in orexin neurons on DREADD-mediated excitation of orexin neurons on REM sleep amount.

### [2P-223]

**Network-driven intracellular cAMP coordinates circadian rhythm in the suprachiasmatic nucleus**

*Daisuke Ono[1], Huan Wang[2], Chi Jung Hung[1], Hsin-tzu Wang[1], Naohiro Kon[1], Akihiro Yamanaka[1], Yulong Li[2], Takashi Sugiyama[3] ([1]*Nagoya University,* [2]*Peking University,* [3]*Evident Corporation*)

Second messengers such as cAMP and $Ca^{2+}$ play a variety of roles in biological functions. Signaling mediates signals from receptors on the cell surface to target molecules inside the cell and amplifies the signaling. Then, the signal alters gene expression and finally changes cellular functions. In the mammalian central circadian clock, the suprachiasmatic nucleus (SCN), several neurotransmitters are suggested to be involved in the SCN neuronal network. Receptors of these ligands are coupled with G-protein and second messenger signalings, such as cAMP and $Ca^{2+}$. However, the functional roles of cAMP and $Ca^{2+}$ and their dynamics in the SCN neuronal network remain largely unclear. In the present study, we visualized the spatiotemporal patterns of circadian rhythms of second messengers and neurotransmitter release in the SCN. Here, we show that circadian rhythms of intracellular cAMP, but not $Ca^{2+}$, in the SCN are driven by the neuronal network, and that this depends on the rhythmic release of vasoactive intestinal peptides (VIP) from the SCN. Importantly, rhythmic VIP release is regulated by neuronal activity in the SCN. Furthermore, optical manipulation of intracellular cAMP levels in the SCN shifts molecular and behavioral circadian rhythms. Together, our study demonstrates that intracellular cAMP is a key molecule in the composition of the SCN circadian neuronal network.

### [2P-224]

**Studies on early detection of sleep-onset signal during driving**

*Akitoshi Seiyama[1,2], Tatsuro Miura[5,2,1], Sayaka Okahashi[3,2,1], Nami Konishi[4], Monte Cassim[1,2] ([1]*Akita International University,* [2]*Kyoto University,* [3]*National Center for Geriatrics and Gerontology,* [4]*Kyoto Tachibana University,* [5]*National Hospital Organization Kyoto Medical Center*)

More than 60% of traffic accidents take place on simple and even roadways due to human errors. We considered that the main human error is the occurrence of drowsiness during driving and hypothesized that the human body sends some bio-signal preceding the drowsiness. To evaluate our hypothesis, we performed simultaneous measurements of changes in the cerebral blood flow (CBF), brain waves and autonomic nerve function by using near-infrared spectroscopy (NIRS), electroencephalogram (EEG) and pulse oximeter (Pulse), respectively. Six healthy subjects participated in this study. We used Gran Turismo 5 Prologue on Play Station 3 as a driving simulator, and used the Karolinska Sleepiness Scale (KSS) as a declaration of feeling drowsy. We analyzed NIRS, EEG and Pulse signals for five minutes before and after declaration of the "slightly drowsy state (KSS-6)". We report characteristic changes in the CBF, brain wave and the autonomic nerve function before and after declaration of KSS-6. This study was approved by the Ethics Committee of Kyoto University Faculty of Medicine, and both written and verbal informed consents were obtained from the participants.

### [2P-225]

**Timelapse observation of the intestinal microbiome during chronic jet lag in mice**

*Hironari Yamasaki[1], Nobuya Koike[1], Kazuhiro Yagita[1] ([1]*Department of Physiology and Systems Bioscience, Kyoto Prefectural University of Medicine*)

In mammals, daily cycles in physiology require the synchronized activity of circadian clocks for the maintenance of homeostasis. Circadian misalignment has been implicated in a number of health problems including metabolic syndrome and chronic inflammation. The alteration of gut microbiota balance is likely to be associated with pathophysiology of these diseases. Although the disruption of the circadian rhythms may affect to gut microbiota, it has uncertain when and how the intestinal flora is affected by the circadian misalignment. To investigate the dynamics of gut microbes by chronic circadian misalignment, we examined the "Timelapse" observation of gut microbiota composition during chronic jet lag characterized by 8-hour advance every 4 days in wild-type C57BL6/J mice over 11 months. Meta-analysis of 16S-rRNA microbial data revealed that significant alterations of gut microbiota composition by chronic circadian misalignments in addition to aging-associated changes. These results reveal that the circadian misalignment-related mechanism impairs the balance of gut microbiota as a non-dietary factor.

### [2P-226]

**The alternative splicing of Cold-inducible RNA-binding protein gene in mice during daily torpor**

*Manaka Takahashi[1], Monami Shiraishi[1], Yuuki Horii[2], Shingo Miyawaki[3], Takahiko Shiina[1], Yasutake Shimizu[1] ([1]*Laboratory of Physiology, Joint Department of Veterinary Medicine, Faculty of Applied Biological Sciences, Gifu University,* [2]*Institute for Glyco-core Research (iGCORE),* [3]*Laboratory of Surgery, Joint Department of Veterinary Medicine, Faculty of Applied Biological Sciences, Gifu University*)

### Poster Presentation

[2P]
**Pathophysiology**

March 15 (Wed.), 12:10 - 14:10, Poster Room

### [2P-227]

### Heated Tobacco Products (HTPs) Promotes Cell Proliferation and Migration in Human Oral Cancer Cells.

*Kagemichi Nagao[1,3], Masanari Umemura[1], Shota Takayasu[1], Fumina Suzuki[1], Yuto Mizuno[1], Soichiro Ishikawa[1], Rina Nakakaji[1], Akane Nagasako[1], Takahiro Horinouchi[2], Tetsuya Yamamoto[3], Yoshihiro Ishikawa[1] (*[1]Yokohama City University Graduate School of Medicine, Cardiovascular Research Institute (CVRI),, [2]Hokkaido University Graduate School of Medicine, Department of Cellular Pharmacology, [3]Yokohama City University Graduate School of Medicine, Department of Neurosurgery*)

[Background] The mainstream tobacco smoke exits the filter of a lit tobacco consists of liquid/solid droplets, called the particulate phase (PP) and suspended in a mixture of gases and semi-volatiles, called the vapor phase (VP). We focused on VP of the heated tobacco products (HTPs), which does not contain tar and nicotine, and investigated its effects on the cell proliferation and the cell migration in human oral cancer cells. [Materials and Method] HSC-3 (human oral cancer cells) was used. We removed tar and nicotine from VP of traditional tobacco products (1R6F) and HTPs (IQOS, Phillip Morris) using a Cambridge filter, and suspended in a culture medium (VP extract). Cell Counting Kit-8 assay and scratch assay were performed. Changes in intracellular $Ca^{2+}$ level were measured by Fluo-4. The phosphorylation antibody array and the western blotting were performed. [Result] 3% VP extract of HTPs significantly promoted the cell proliferation of HSC-3. HTPs increased the cell migration and increased the intracellular Ca2+. Western blotting showed that 3% HTPs phosphorylated CAMKK2 (calcium/calmodulin dependent protein kinase kinase 2). The phosphorylation array showed that 3% VP extract of HTPs dephosphorylated EGFR (epidermal growth factor receptor). [Conclusion] We found that the VP extracts of HTPs at a some concentration might promote the cell proliferation and migration in human oral cancer cells.

### [2P-228]

### Relation between severity of headache and basal ganglia volume in migraine patients.

*Shota Kosuge[1,2], Yuri Masaoka[1], Hideyo Kasai[2], Motoyasu Honma[1], Miku Kosuge[1,3], Kouzou Murakami[4], Nobuyuki Yoshii[5], Hidetomo Murakami[2], Masahiko Izumizaki[1] (*[1]Department of Physiology, Showa University School of Medicine., [2]Department of Neurology, Showa University School of Medicine., [3]Department of Respiratory Medicine, Showa University Fujigaoka Hospital., [4]Department of Radiology, Showa University School of Medicine., [5]Department of Radiological Technology, Showa University Hospital.*)

Migraine (MG) is a complex disorder of the brain that involves multi-sensory disturbances. Symptoms of MG include the primary headache accompanied with disordered perceptions of light, sound and smell. Patients often complain of hypersensitivities toward normal light or sound and these induce headache as well as nausea. In this study, we measured the structural volume of brain regions in patients with MG, and investigated a relationship between structural volumes and headache severity. 16 migraine patients (aged 16-64 years) and 27 healthy subjects (aged 24-55 years) measured whole brain T1-weighted magnetic resonance imaging (MRI) and measured brain volume with Freesurfer software. MG were measured Migraine Disability Assessment Questionnaire (MIDAS) and Migraine-Specific-Quality of Life Questionnaire (MSQ) to assess headache severity. Analysis of covariance showed decreased volumes in the left thalamus, left globus pallidus, left temporal nucleus, left middle temporal gyrus, left lingual gyrus, right superior frontal gyrus, right central posterior gyrus, and bilateral occipital poles in MG compared with those of controls. Correlation analysis showed a negative correlation between the volume of the left globus pallidus and MIDAS and MSQ scores in MG, indicating that individuals with smaller left globus pallidus volume have more severe migraine symptoms. The globus pallidus play a role for inhibiting the activity of the thalamus, so that volume reduction of the globus pallidus might be one of the factors for inducing dysregulation of the pain processing of the thalamus.

### [2P-229]

### Effect of microglia-specific cyclooxygenase-1 knockout on fever induced by cerebral hemorrhage

*ISHIDA SHUNTO[1], Shiho Kitaoka[2], Tomoyuki Furuyashiki[3], Shu Narumiya[4], Kiyoshi Matsumura[1] (*[1]Department of Biomedical Engineering, Osaka Institute of Technology, [2]Department of Pharmacology, School of Medicine, Hyogo Medical University, [3]Division of Pharmacology, Graduate School of Medicine, Kobe University, [4]Medical Innovation Center, Kyoto University Graduate School of Medicine*)

Our laboratory has previously found that prostaglandin E2 (PGE2) is involved in fever due to cerebral hemorrhage, and the enzyme cyclooxygenase-1 (COX-1) is responsible for PGE2 production. COX-1 is highly expressed in microglia constitutively, and in platelets of the hemorrhage region. In this study, microglia-specific COX-1 knockout mice (KO mice) were used to investigate its contribution to cerebral hemorrhagic fever. Under isoflurane anesthesia, collagenase (400 nl) was injected near the hypothalamus of KO and control mice to induce brain hemorrhage. After recovery from anesthesia, changes in body temperature were measured for 24 hours under free-moving conditions. The brains of the mice were then removed to confirm the site of the brain hemorrhage and immunostained for COX-1. Control mice with cerebral hemorrhage showed an increase in body temperature, reaching a peak after 4 to 5 hours. KO mice showed similar temperature changes. There was no difference in the location or size of the cerebral hemorrhage between the two groups of mice, and COX-1 expression in microglia was almost completely suppressed in the KO mice. These results suggest that the contribution of microglial COX-1 to cerebral hemorrhage fever is small. The contribution of platelets to COX-1 is currently under investigation.

### [2P-230]

### Involvement of secondary thrombus formation in acute ischemic stroke

*Yuuki Moriike[1], Yasuki Matano[1], Yasuhiro Suzuki[2], Kazuo Umemura[3], Nobuo Nagai[1] (*[1]Department of Animal Physiology, Faculty of Bioscience, Nagahama Institute of Bio-Science and Technology, [2]Faculty of Pharmacy, Ohu University, [3]Department of Pharmacology, Hamamatsu University School of Medicine*)

We investigated the roles of secondary thrombus (ST) on the pathogenesis of cerebral infarction using a murine photo-thrombotic stroke model. It was found that ST was induced at the surrounding region of ischemic core. The ST positive area expanded until 16 hours after stroke induction, but disappeared at 24 hours in wild type mice. It was also found the expansion of ST positive area was trailing behind the expansion of the area with the increase in vascular permeability (VP) associated with ischemic stroke, which prevented the increase in VP. In mice with gene deficiency of plasminogen, ST was not dissolved until 24 hours and the infarct size was significantly larger than that of wild type controls. When a recombinant tissue plasminogen activator (t-PA) was intravenously administered at 4 hours after stroke, the ST disappeared, the increase in VP reversed, and hemorrhage at the damage region was accelerated in wild type mice. These findings suggest that ST is induced at the surrounding region of ischemic core in this model, which is involved in both deterioration of ischemic damage and suppression of the increase in VP and associating hemorrhagic transformation by t-PA.

### [2P-231]

### Dedifferentiated Schwann cells that maintain the adrenergic microenvironment contributes to chemotherapy resistance in lung cancer patient.

*Yusuke Otani[1,3,4], Haruyoshi Katayama[2,3], Shinichi Toyooka[1,4], Atsushi Fujimura[3] (*[1]Department of General Thoracic Surgery and Breast and Endocrinological Surgery, Okayama University Graduate School of Medicine, Dentistry and Pharmaceutical Sciences, [2]Department of Orthopaedic Surgery, Okayama University Graduate School of Medicine, Dentistry and Pharmaceutical Sciences, [3]Department of Cellular Physiology, Okayama University Graduate School of Medicine, Dentistry and Pharmaceutical Sciences, [4]Department of General Thoracic Surgery and Breast and Endocrinological Surgery, Okayama University Hospital*)

Doublecortin (DCX)-positive neural progenitor-like cells are reported to be a component of the cancer microenvironment in a previous paper. The number of DCX-positive cells in a tissue has been reported to correlate with cancer progression. Although the number of DCX-positive cells has been reported to correlate with cancer progression, the mechanism by which these cells affect cancer progression is largely unknown. In this study, we show that DCX-positive cells in all major tissue subtypes of lung cancer originate from Schwann cells and contribute to the chemotherapy resistance of lung cancer cells by creating an adrenaline-rich microenvironment. The results revealed that the de-differentiation of Schwann cells to DCX-positive cells involves activation of the Hippo transducer YAP/TAZ, and that these cells also express catecholamine synthase and synthesize adrenaline, which enhances the chemotherapy resistance of lung cancer cells via activation of YAP/TAZ. Furthermore, in patients with lung adenocarcinoma, it was suggested that patients with a higher density of Schwann cells had more recurrences and worse prognosis. These findings demonstrate that cancer-associated Schwann cells form an adrenergic microenvironment in lung cancer tissues through the regulation of YAP/TAZ.Disclosures: We DO NOT have a Conflict of Interest.

### [2P-232]

### Presence of catecholamine synthases and the role in maintaining cancer stem-like cells in malignant peripheral nerve sheath tumors

*Haruyoshi Katayama[1,2,3], Yusuke Otani[2,4], Eiji Nakata[1,3], Toshifumi Ozaki[1,3], Atsushi Fujimura[2] ([1]*Department of Orthopaedic Surgery, Okayama University Graduate School of Medicine, Dentistry and Pharmaceutical Sciences,* [2]*Department of Cellular Physiology, Okayama University Graduate School of Medicine, Dentistry and Pharmaceutical Sciences,* [3]*Department of Orthopedic Surgery, Okayama University Hospital,* [4]*Department of General Thoracic Surgery and Breast and Endocrinological Surgery, Okayama University Graduate School of Medicine, Dentistry and Pharmaceutical Sciences*)

Malignant peripheral Nerve Sheath Tumor (MPNST) is a rare soft-tissue sarcoma arising from Schwann cells (SCs). The prognosis is poor due to the chemo-resistance. Our previous study showed that exogenous adrenaline (Ad) increases stemness of MPNST. SCs have plasticity and can dedifferentiate into Schwann cell precursors (SCPs). SCPs are origin of chromaffin cells, main cells of Ad synthesis. The purpose of this study is to clarify the presence and the role of catecholamine (CA) synthases for the cancer stemness in MPNST.Three human MPNST cell lines (FMS-1, HS-PSS, and HS-Sch-2) were examined in vitro. Western blotting (WB) and immunofluorescence staining (IF) were performed to confirm the presence and localization of intracellular CA synthases and Ad. RNAi of CA synthases were used to confirm changes in stemness.WB showed intracellular CA synthases and de novo Ad, and IF showed that their localization was in cytoplasm. RNAi experiment showed the decrease of YAP/TAZ, a cancer stem cell factor, activity and cancer stem cell markers and self-renewal capacity.This study shows that the endogenous Ad synthesis pathway is involved in the maintenance of cancer stem-like cells, thus, inhibition of this pathway is expected to be a new therapeutic target.Disclosures: We DO NOT have a Conflict of Interest.

### [2P-233]

### Anti-tumor effect mediated by the downregulation of PD-L1 induced by the combinatorial suppression of multiple factors correlates with tumor metastases

*Nagomi Yano[1], Yuji Hayashi[2], Shoko Miyoshi[1], Itaru Watanabe[1], Hajime Yano[1], Naohito Hato[2], junya Tanaka[1] ([1]*Department of Molecular and Cellular Physiology, Graduate School of Ehime University Medical School,* [2]*Department of Otorhinolaryngology, Head and Neck Surgery, Ehime University Medical School*)

Cancer metastasis is a multifactorial and multidimensional process. Although many factors involved in this process have already been identified, suppression of these factors has not yet been achieved in the clinical setting to prevent cancer metastasis. We have discovered several metastasis-related factors including NHE1 and LOXL2, which are upregulated in a tongue cancer cell SASL1m, in originally established mouse model of lymph node metastasis of tongue cancer. Aiming at getting a breakthrough that can substantially suppress metastasis in clinical practice, herein, we assessed the effectiveness of multiple and parallel suppression of metastasis-related factors against tumor metastases. The SASL1m cell in which NHE1 and LOXL2 are doubly knocked down, exhibited lower lymph node metastasis than knockdown cells of each alone. Moreover, double knockdown cells showed reduced xenograft-primary lesion. Elimination by the immune system upon xenograft accompanying double-knockdown was implied, and decreased PD-L1 gene expression as well as protein mass in double-knockdown cells were observed. We also observed a decrease in cell number of double knockdown under co-culture with phagocytotic microglial line cells, BV2. We would like to discuss the possibility that suppressing multiple such factors in parallel may lead to efficient inhibition of cancer metastasis and elimination of tumor cells.

### [2P-234]

### Adrenoceptor switching is required to maintain the cellular plasticity of glioblastoma

*Atsushi Fujimura[1] ([1]*Department of Cellular Physiology, Okayama university Okayama University Graduate School of Medicine, Dentistry, and Pharmaceutical Sciences*)

Glioblastoma cells are known to regulate their plasticity in response to the surrounding microenvironment, but what factors contribute to the formation of plasticity is not fully understood. Here, we found that glioblastoma cells change the expression levels of adrenoceptors depending on their differentiation state. Among the catecholamines that are abundant in the central nervous system, we found that noradrenaline enhances the stemness of glioblastoma cells and promotes the dedifferentiation of differentiated glioblastoma cells. Experiments using adrenoceptor antagonists and RNAi revealed that noradrenaline's effects on glioblastoma cell plasticity are mediated by alpha1D-adrenoceptor signaling. We also found that high expression of alpha1D-adrenoceptor is associated with poor prognosis in malignant brain tumor patients. These data suggest that glioblastoma cells alter their own adrenoceptor expression levels to be adapted to the surrounding tumor microenvironment for their survival. We believe that this may contribute to the development of new therapeutic strategies for glioblastoma.

### Poster Presentation

[2P]
### Drug Action, Pharmacology

March 15 (Wed.), 12:10 - 14:10, Poster Room

### [2P-235]

**Network pharmacological analysis of the effect of *Smilacis Glabrae Rhixoma* on gastrointestinal motility disorder**

NARI CHOI[1,4], KANGWOOK LEE[2,3,4], MUJIN SEO[1,4], SEOK-JAE KO[2,3,4], WOO-GYUN CHOI[1], JAE-WOO PARK[2,3], *BYUNGJOO KIM[1] (*[1]Pusan National University School of Korean Medicine, Republic of Korea, [2]Department of Clinical Korean Medicine, Graduate School of Kyung Hee University, Republic of Korea, [3]Department of Gastroenterology, College of Korean Medicine, Kyung Hee University, Republic of Korea, [4]These authors contributed equally to this work*)

Background: Gastrointestinal motility disorder is a disease that causes digestive problems due to inhibition of the movement of the gastrointestinal tract, and is one of the diseases that reduce the quality of life of modern people. *Smilacis Glabrae Rhixoma* (SGR) is a traditional herbal medicine for many diseases and is sometimes prescribed to improve digestion. Methods: As a network pharmacological approach, we searched the TCMSP database for SGR, reviewed its constituents and target genes, and analyzed its relevance to Gastrointestinal motility disorder. And using the GMD mouse model through acetic acid (AA), we investigated the locomotor effect of SGR on intestinal transit rate (ITR). Results: As a result of network pharmacology analysis, 56 compounds out of 74 candidate compounds of SGR have targets, the number of targets is 390 targets, and there are 904 combinations. Seventeen compounds of SGR related to GMD, and as a result of comparing the related gene with the GMD-related gene, 17 genes (Active only) corresponded to both. When looking at the relationship network between GMD and SGR, it was confirmed that Quercetin, Resveratrol, SCN5A, TNF, and FOS were most closely related to GMD. As a result of feeding SGR to AA-induced GMD mice, it was confirmed that the ITR decreased by AA was restored by SGR. Conclusions: Through network pharmacology, it was confirmed that Quercetin, Resveratrol and SCN5A, TNF, FOS were related to GMD in SGR, and these were closely related to intestinal motility. confirmed to do. Based on these results, it is suggested that SGR in GMD restores digestion through the recovery of intestinal motility.

### [2P-236]

**Investigation of Piezo1 inhibition by liquiritigenin, and anti-colon cancer.**

*Yasunori Takayama[1], Kenta Maruyama[2,1], Takeshi Kondo[3], Mami Kato[4], Makoto Tominaga[2], Masataka Sunagawa[1] (*[1]Dept Physiol, Showa Univ Sch Med, [2]Thermal Biol, ExCELLS, Nat Inst Nat Sci, [3]Dept Biochem, Hokkaido Univ, [4]Dept Mol Sys Pharmacol, Kyushu Univ*)

Liquiritigenin, an aglycone derived from *Glycyrrhiza glabra*, reportedly inhibits the growth of colon cancer cell, HC-116 cells. However, the primary molecular target of liquiritigenin is still unclear. Here, we found that liquiritigenin inhibits a mechanosensitive ion channel, Piezo1. Piezo1 has been also reported that the expression is enhanced in cancer cells, and the inhibition of Piezo1 expression induces anti-cancer effect. Therefore, we hypothesized that Piezo1 inhibition by liquiritigenin is the molecular mechanism of its anti-cancer effect on colon cancer. To clarify it, we performed MTT assay in CMT-93 cells derived from mouse colon cancer. Although liquiritigenin inhibited the growth of CMT-93 cells, the anti-cancer effect was also similarly observed in Piezo1-knock out CMT-93 cells. Therefore, the anti-cancer effect of liquiritigenin could be Piezo1 independent pathway. However, the starting time point of growth of Piezo1-KO CMT-93 cells after passage was faster than that of wild type CMT-93 cells. These facts indicated that Piezo1 is a suppression factor against cancer malignant. Namely, selective Piezo1 inhibition could induce the expansion of colon cancer invasion.

### [2P-237]

**The effect of acute nicotine treatment on visual discrimination and its reversal learning in mice.**

*Yuto Shimo[1,2], Takaaki Ozawa[1,2], Tom Macpherson[1,2], Takatoshi Hikida[1,2] (*[1]Osaka Univ. Institute for Protein Research, Advanced Brain Functions Lab., [2]Osaka Univ. Graduate School of Science, Department of Biological Sciences*)

Nicotine is known as an addictive chemical compound that has been found in tobacco. After being absorbed in a periphery, nicotine acts as a ligand for nicotinic acetylcholine receptors in the central nervous system and increases neuronal excitation. Interestingly, nicotine has also been reported to improve some cognitive functions in humans and animals, suggesting that nicotinic acetylcholine receptors and their related mechanisms can be good targets to develop effective cognitive enhancers. However, the detailed mechanism of the cognitive enhancing effect of nicotine remains largely unknown. To address this question, we tested the effect of acute nicotine administration (nicotine tartrate; 0.125, 0.25, 0.5 mg/kg, i.p.) on the performance in visual discrimination and its reversal tasks in a touch screen device, which can assess learning and cognitive functions in model animals under the conditions more similar to human cognitive tasks. As a result, it was found that nicotine treatment facilitated discrimination learning and its reversal learning in male mice, while it impaired these learnings in female mice. These results suggest the cognitive enhancing effect of nicotine is dependent on the sex of animals, and the roles of nicotinic receptors in-memory processing and cognitive flexibility are different between males and females.

### [2P-238]

**Flavacitropone A, from *Glycosmis citrifolia* (Rutaceae)., induce an apoptotic effect on human pre-B cell leukaemia cells**

*Takuya Matsui[1], Chihiro Ito[2], Masataka Itoigawa[3] (*[1]Department of physiology, School of Medicine, Aichi Medical Univeristy, [2]Faculty of Pharmacy, Meijo university, [3]School of Sport and Health Science, Tokai Gakuen University*)

Acute lymphoblastic leukemia (ALL) is treated by combination chemotherapy or targeted small-molecule drugs. However, these conventional therapeutic agents have problems with safety and efficacy. Compounds from plants may be useful in developing anticancer drugs for ALL with fewer side effects. We investigated the biological activities of flavacitropone A, a homoacridone–flavanone dimer isolated from *Glycosmis citrifolia* (Willd.) Lindl., against a human pre-B cell leukemia cell line (NALM6). Flavacitropone A suppressed the growth of NALM6 cells but not that of peripheral blood mononuclear cells, and it time-dependently increased the number of annexin V positive cells and decreased the cell population of the G2/M and S phases. Additionally, caspase 3/7 activity in NALM6 cells treated with flavacitropone A was higher than that in non-treated cells. These results show that flavacitropone A induced apoptosis in NALM6 cells. Comprehensive analysis of flavacitropone A–treated cells indicated the elevated expression of some genes (*ATF3* and *DDIT3*) and intracellular proteins (cleaved caspase 3 and 7). Flavacitropone A may be useful in the treatment of various types of leukemia.

### [2P-239]

**Reduced pain sensitivity of episodic pain syndrome model mice carrying a Nav1.9mutation by ANP-230, a novel sodium channel blocker**

*Hiroko Okuda[1], Sumiko Inoue[1], Yoshihiro Oyamada[1,2], Akio Koizumi[1], Shohab Youssefian[1,3] (*[1]Department of Pain Pharmacogenetic, Kyoto Univ., [2]AlphaNavi Pharma Inc., [3]Laboratory of Molecular Biosciences, Kyoto Univ.*)

The sodium channel Nav1.9 is expressed in the sensory neurons of small diameter dorsal root ganglia (DRG) that transmit pain signals. Gain-of-function Nav1.9 mutations have been associated with painful disorders, and we initially determined that some Nav1.9 mutations are responsible for the familial episodic pain syndrome observed in the Japanese population. We therefore generated model mice harboring one of the more painful Japanese mutations, R222S, and determined that DRG hyperexcitability was the cause of the associated pain. A novel non-opioid drug, ANP-230, with strong inhibitory effects on Nav1.7, 1.8 and 1.9, is currently under clinical trials for patients suffering from familial episodic pain syndrome. However, little is known about its mechanism of action and effects on pain sensitivity. We therefore investigated the inhibitory effects of ANP-230 on the pain hypersensitivity of Nav1.9 p.R222S mutant model mouse. In behavioral tests, ANP-230 reduced the pain response of the mice, particularly to heat or mechanical stimuli, in a concentration-dependent manner. Furthermore, ANP-230 suppressed the repetitive firing of DRG neurons of these mutant mice. Our results suggest that ANP-230 is an effective analgesic for familial episodic pain syndrome resulting from DRG neuron hyperexcitability, and that such analgesic effects are likely to be of clinical significance.

### [2P-240]

**Analysis of intracerebral neurotransmitter dynamics during antiepileptic drug administration in epilepsy model zebrafish.**

*Ryohei Umeda[1,2], Kazuo Okanari[3], Hitoshi Teranishi[1], Kenshiro Shikano[1], Reiko Hanada[1] ([1]*Oita university Faculty of Medicine Department of Neurophysiology,* [2]*Oita university Faculty of Medicine Department of Advanced Medical Science,* [3]*Oita university Faculty of Medicine Department of Pediatrics*)

Epilepsy is a chronic neurological disease associated with abnormal neuronal activity in the brain, and a serious health problem in the world. The pathogenesis of epileptic seizures is thought to result from an imbalance between excitatory and inhibitory neurotransmitters in the brain. However, detailed neurotransmitter dynamics during epileptic seizures and Antiepileptic drugs (AEDs) administration are still unclear. At first, we established a pentylenetetrazole (PTZ)-induced epileptic seizure model using adult zebrafish and analyzed a series of neurotransmitter dynamics in the brain. Next, we analyzed the effects on behavior and major neurotransmitters at administrated of the AEDs carbamazepine (CBZ), levetiracetam (LEV), and fenfluramine (FFR) in seizure. We found that the levels of serotonin and dopamine significantly increased in the brain immediately after seizures. Glutamate decreased in the brain in the CBZ group, and GABA and choline levels increased in the brain in the FFR group. These data suggested that each AED has a different pharmacological mechanism depending on the dynamics of different neurotransmitters.

### Poster Presentation

### [2P]
### Study Methodology

March 15 (Wed.), 12:10 - 14:10, Poster Room

### [2P-241]

**How to describe the quality of itching with objective probability of success**

*Kotaro Honda[1,2], Mitsutoshi Tominaga[1,2], Kenji Takamori[1,2,3] ([1]*Institute for Environmental and Gender-Specific Medicine, Juntendo University Graduate School of Medicine,* [2]*Juntendo Itch Research Center,* [3]*Department of Dermatology, Juntendo University Urayasu Hospital*)

We perceive itching as a warning about the presence of a foreign body. Scratching behavior targets the area causing the itch, but there is no guarantee that the foreign substance will be removed by the scratching behavior. This is because allergens, parasites such as mites, and chemicals that cause itch are too small for the animal to recognize with its senses, and changes in their presence before and after the scratching behavior are essentially unknown. So what does the scratching behavior mean for its role as an itch alert?  We found that scratching behavior is temporally characterized by clusters of scratching bouts. By classifying groups of scratching bouts by repetition length (n=1, 2, 3, ...), we found that the pattern of occurrence of the groups can be modeled by a geometric distribution. The geometric distribution is a distribution determined only by the success probability $p$, and this maximum likelihood estimator is a consistent estimator, although it is not an unbiased estimator. For healthy mice, $p$ = 0.43-0.44, and for the dry skin model mice, $p$ = 0.21-0.27. For acute itch, $p$ = 0.15 for histamine, $p$ = 0.08 for chloroquine, and p = 0.15 for capsaicin.  These results suggest the existence of a risk estimation mechanism in which risk measures for foreign substances are stored in the central nervous system in analog form ($0<p<1$) and digitally transformed (1, 2, 3, ...) when they are embodied in repeated scratching bouts.

### [2P-242]

**Label-free voltage measurement by dual-comb interferometry: A fundamental study for label-free imaging of action potentials**

Satoshi Araoka[1], *Hiroki Takanari[1], Ryo Mitsumoto[1], Akira Emoto[1], Kazumichi Yoshii[1] ([1]*Tokushima University*)

Action potential (AP) measurement is essential to understand cell and organ physiology. Patch clamp or optical mapping using voltage-sensitive fluorescence are commonly used to measure APs. However, it is difficult to apply these methods to humans or living animals. We hypothesized that APs would alter the optical properties of cytoplasmic membranes, and that ultra-precise measurement of these minute changes in the membrane would allow label-free detection of APs. To prove the hypothesis, we established a custom-made dual-comb optical device including two optical frequency combs (a central wavelength: 1,560 nm). A cover glass (0.25 mm thick) with Indium Tin Oxide (ITO, 200 Å thick) on both sides, which could be considered as a transparent parallel plate capacitor, was prepared to mimic a cytoplasmic membrane. A signal comb was transmitted through ITO glass and interferogram was acquired by interfering with a local comb that was phase-locked to the signal comb. When a voltage of 3 V was applied to the ITO glass, the phase of the interferogram changed significantly. It was shown that the potential applied to thin films such as cell membranes may change the phase of the light.

### [2P-243]

**Development of an fMRI Neurofeedback System for Cognitive Functions Using Machine Learning.**

*Kouji Takano[1], Tomoaki Komatsu[1], Kimihiro Nakamura[1] ([1]*Department of Rehabilitation for Brain Function, Research Institute of National Rehabilitation Center for Persons with Disabilities*)

The efficacy of fMRI neurofeedback was investigated in this decade. Simple fMRI neurofeedback systems feedback the intensity of brain activity in the region of interests to the subject. Nowadays, almost fMRI neurofeedback, such as DecNef, perform more advanced analysis and feedback scores related to the target behavior or brain function. To use machine learning methods for neurofeedback, performing a task related to the target function before feedback, and creating a model based on the brain activity during the task is necessary. In this study, we investigated the use of 3D convolutional neural networks to create a linear regression model for estimating scores for brain functions related to the visual recognition task. Supervised learning was performed using EPI as input, BOLD signal intensity calculated from visual stimulus presentation time, and the hemodynamics response function as output. As a result, we obtained a model that showed a significant correlation between the predicted values and the BOLD signal. This correlation coefficient was better than using the intensity of the brain or/and the random forest method.

### [2P-244]

**Elastase treatment improves adhesiveness of primary human hepatocytes**

*Rieko Tanaka[1], Kazuko Aizawa[1], Hidenori Akutsu[2], Kazuaki Nakamura[1] ([1]*Department of Pharmacology, National Research Institute for Child Health and Development,* [2]*Center for Regenerative Medicine, National Research Institute for Child Health and Development*)

Primary human hepatocytes (PHHs) are important tools for physiological studies of human hepatocytes in vitro, but has the problem of unstable adhesion to culture vessel coated by collagen. Long-term and stable monolayer culture of PHHs including formation of bile canaliculi is desired for physiological investigating hepatocyte functions including drug metabolism. In this study, we investigated the cause of decreased adhesiveness and attempted to improve the adhesiveness of PHHs.We hypothesized that extracellular matrix (ECM) covering the PHHs surface inhibit cell adhesion to culture vessel and analyzed the difference of ECM on cell surface between adhesion and non-adhesion PHHs. We found that non-adhesion PHHs had more ECM on the cell surface than adhesion PHHs and one of main components of these ECM is elastin. When elastin was removed from cell surface of non-adhesion PHHs by elastase treatment, consequently removed whole ECM from cell surface, and these cells could attach to culture vessel. These results suggest that ECM on the surface of PHHs is the cause of poor adhesion and show that removal of ECM including elastin as the main ingredient improved hepatocyte adhesion.

### [2P-245]

**Optimization of DNA-PAINT for Super-Resolution Imaging of Neuronal Synapses**

*Ikumi Tobishima[1], Michinori Toriyama[1], Ikuko Yao[1] ([1]*Kwansei Gakuin University*)

Optical microscopy is generally used to reveal molecular localization and biological structures. The development of super-resolution microscopy has reached high resolution, making it possible to visualize nano-scale structures such as neuronal synapses. STORM detects the blinking of photoswitchable fluorophores and records them as coordinate data. These processes are repeated to reconstruct pointillistic super-resolution images. STORM generally uses fixed labeling, thus this labeling method has the problem that it is prone to photobleaching, and the reduction of blinking events due to photobleaching may affect the accuracy and quantitative precision of acquired images.
In this study, we introduced DNA-PAINT to solve this problem. The advantages of this method are not restricted to the photoswitchable fluorophore and photobleaching. The purpose of this experiment was to optimize super-resolution imaging with DNA-PAINT for stable super-resolution image acquisition of neuronal synapses.
We found that about 15,000 frames per channel were required to detect and record for stable acquisition of super-resolution images of synapses. This result suggests that it is useful to introduce DNA-PAINT, which can reduce photobleaching. In fact, compared to conventional STORM, the number of photons per frame was hardly reduced. In the future, it is expected to accurately capture molecular localization changes at the level of single synapses, leading to their quantitative evaluation.

### Poster Presentation

### Day 3
### (March 16, 12:10 - 14:10)

[3P] Neurophysiology, Neuronal cell biology - Plasticity
[3P] Neurophysiology, Neuronal cell biology - Neural network
[3P] Neurophysiology, Neuronal cell biology - Neurochemistry
[3P] Neurophysiology, Neuronal cell biology - Neurons, Synapses
[3P] Neurophysiology, Neuronal cell biology - Glia
[3P] Neurophysiology, Neuronal cell biology - Higher brain function
[3P] Neurophysiology, Neuronal cell biology - Motor function
[3P] Neurophysiology, Neuronal cell biology - Sensory function, Sensory organ
[3P] Molecular physiology, Cell physiology - Membrane transport
[3P] Molecular physiology, Cell physiology - Ion channels, Receptors
[3P] Embryology, Regenerative Medicine, Development, Growth, Aging
[3P] Muscle
[3P] Circulation
[3P] Endocrine
[3P] Environmental physiology
[3P] Nutritional and metabolic physiology, Thermoregulation
[3P] Behavior, Biological rhythm, Sleep
[3P] Pathophysiology
[3P] Drug Action, Pharmacology
[3P] Medical education, Medical histology
[3P] Study Methodology
[3P] Others

### Poster Presentation

[3P]
### Neurophysiology, Neuronal cell biology
### Plasticity

March 16 (Thu.), 12:10 - 14:10, Poster Room

### [3P-001]

### Neurofeedback Learning of Cerebellar Brain Inhibition in Patients with Spinocerebellar Degeneration -A proof of concept study-

*Erika Omae[1], Atsushi Shima[1], Kazuki Tanaka[1], Akari Ogawa[1], Masako Yamada[1], Tadashi Isa[1], Satoko Koganemaru[1] ([1]*Kyoto Univ.*)

Neurofeedback learning is a method for self-regulating brain activities. Recently, we have reported the volitional control of the physiological activity of the primary motor cortex (M1) by the neurofeedback learning using transcranial magnetic stimulation (TMS), cerebellar function can be evaluated by cerebellar inhibition (CBI) using a paired TMS technique. The CBI is disturbed in spinocerebellar degeneration (SCD) patients. Here, we preliminarily investigated whether the CBI neurofeedback learning could be achieved and improve cerebellar function in SCD patients. Three SCA-6 patients (all male) participated in the study. Their CBIs were disrupted before the intervention. During the neurofeedback learning, they were trying to enhance their CBIs by the intention to reduce the size of a circle, which reflected degrees of the CBI. After the neurofeedback learning, their CBIs were improved in the intention condition in which they were trying to reduce the size of a circle, while they were not without any intention, just resting. It suggests that the CBI feedback learning can be achieved in SCD patients and may become a novel approach to improve the cerebellar function in SCD patients.

### [3P-002]

### Effects of hypoxic stress on periventricular glial cells in neonatal common marmosets

*Rie Suge[1], Ting Tao[2], Hideo Yamanouchi[1], Yoshimasa Kamei[1], Mamiko Koshiba[1,2,3] ([1]*Saitama Medical Univ.*, [2]*Yamaguchi Univ.*, [3]*Tohoku Univ.*)

We developed a common marmoset neonatal model with hypoxic-ischemic stress for investigating mechanisms and treatment methods of neonatal hypoxic-ischemia and periventricular leukomalacia, with behavioral and molecular biological approaches. Perinatal hypoxic-ischemic stress, varying effects due to gestational-age or the stress level, causes extrapyramidal dysfunction (have residual neurological sequelae, cerebral palsy) or failure to differentiation of oligodendrocyte in periventricular area. The animal model was established with 30 min exposure to a low level oxygen environment (6%) on ~24 h after the birth. Based on our previous work (CNS Neurol Disord Drug Targets. 2016;15(5):578-86), hanging behavior (anti-gravity motion test, we hypothesize a part of human primitive reflex is reflected on this behavior) was used as a behavioral index. Brain samples were collected for immunohistochemical analysis. In the anti-gravity motion test, the hypoxia group tended to fall down in a shorter time than control, indicating weaker grip force. Glial cells in periventricular area were examined signal intensity and number of positive cells per unit area of marker proteins. In the hypoxia group generally, there was an increase of signal intensity of Ionized calcium binding adapter protein 1-positive cells and subjects with decreased intensity of Olig2 positive cells showed weaker grip force. This work was supported by JSPS KAKENHI Grant Numbers JP15K15404 and JP19K08305.

### [3P-003]

### KCC2 downregulation after sciatic nerve injury enhances motor function recovery

*Dennis Lawrence Cheung[1], Takuya Toda[1], Junichi Nabekura[1] ([1]*The National Institute for Physiological Sciences*)

Injury to mature neurons induces KCC2 downregulation, resulting in elevated intracellular [Cl-] and depolarized GABAergic signaling. This is hypothesized to facilitate neural circuit repair as neural circuit formation by immature neurons depends on low KCC2 expression. We test this in spinal cord motoneurons injured by sciatic nerve crush using transgenic (CaMKII-KCC2) mice wherein conditional CaMKIIα promoter-KCC2 expression coupling selectively prevents injury-induced KCC2 downregulation. We observe impaired motor function recovery in CaMKII-KCC2 mice relative to wild-type mice in an accelerating rotarod assay. We correlate this with differing post-injury reorganization patterns of synaptic input to motoneuron somas – for wild-type, both VGLUT1-positive (excitatory) and GAD67-positive (inhibitory) terminal counts decrease; for CaMKII-KCC2, only VGLUT1-positive terminal counts decrease. Furthermore, we recapitulate impaired motor function recovery in wild-type mice via local spinal cord injections of bicuculline (GABAA receptor blockade) or bumetanide (lowers intracellular [Cl-] by NKCC1 blockade) during the early post-injury period. Thus, we confirm injury-induced KCC2 downregulation enhances motor function recovery and suggest an underlying mechanism of depolarizing GABAergic signaling driving adaptive neural circuit reconfiguration that preserves appropriate excitation-inhibition balance.

### [3P-004]

### Ocular dominance plasticity and visual response selectivity of surviving subplate neurons in layer 6b of mouse primary visual cortex

*Taisuke Yoneda[1,2], Kenji Hayashi[1,2], Yumiko Yoshimura[1,2] ([1]*Natl Inst for Physiol Sci*, [2]*Sch of Life Sci, SOKENDAI*)

Subplate neurons are early-born cortical neurons that transiently form neural circuits during perinatal development and guide cortical maturation. Thereafter, most subplate neurons undergo cell death, while some survive and renew their target areas for synaptic connections. However, the functional properties of the surviving subplate neurons remain largely unknown. Here we characterize the visual responses and experience-dependent functional plasticity of layer 6b (L6b) neurons, the remnants of subplate neurons, in the primary visual cortex (V1). Two-photon $Ca^{2+}$ imaging was performed in V1 of awake juvenile mice. L6b neurons showed broader tunings for orientation, direction, and spatial frequency than did L2/3 and L6a neurons. In addition, L6b neurons showed lower matching of preferred orientation between the left and right eyes compared with other layers. Post hoc 3D immunocytochemistry confirmed that the majority of recorded L6b neurons expressed a subplate neuron marker. Moreover, chronic two-photon imaging showed that L6b neurons exhibited ocular dominance (OD) plasticity by monocular deprivation during critical periods. The OD shift to the open-eye depended on the response strength to the stimulation of the eye to be deprived before starting monocular deprivation. In conclusion, our results provide strong evidence that surviving subplate neurons exhibit sensory responses and experience-dependent plasticity.

### [3P-005]

### Visual awareness of blindsight in macaque monkeys

*Xiyao Yu[1], Reona Yamaguchi[2], Hakwan Lau[3], Tadashi Isa[1,2] ([1]*Department of Neuroscience, Graduate school of Medicine, Kyoto University*, [2]*Institute for the Advanced Study of Human Biology (WPI-ASHBi), Kyoto University*, [3]*RIKEN Center for Brain Science*)

The damage to the primary visual cortex may lead to an intriguing phenomenon known as blindsight. The blindsight subjects can discriminate the target presented in the affected visual field, while having little visual awareness of seeing the target. The purpose of this study is to investigate the brain activity associated with visual awareness in blindsight subjects. To this end, we trained a unilateral V1-lesioned monkey to perform 2AFC (2 alternative forced choice) saccade-fixation task, in which the monkey needed to fixate at the position where the target appeared for 5 s to get the reward to measure the visual awareness level of monkeys. Fixation break was considered to be a sign of having less confidence about seeing the target. The current result showed that under the same correct rate, fixation break rate was much higher in the affected visual field than in the normal visual field, suggesting a lower visual awareness level. In the future, the activity of prefrontal cortex will be recorded using electrocorticography, to search for the brain activity reflecting the different visual awareness level.

### [3P-006]

### Enhancement of hippocampal LTP by synaptic $Zn^{2+}$ under the cooperation of noradrenaline and glucocorticoid

*Satoshi Watanabe[1], Risako Machidera[1], Suzuki Miki[1], Atsushi Takeda[1], Yuji Hara[1]
([1]*University of Shizuoka*)

Stress increases intracellular $Zn^{2+}$ in hippocampal CA1 via the increase in glucocorticoid secretion and affects memory formation. Noradrenaline secretion is increased prior to glucocorticoid secretion after exposure to stress. To clarify the stress-induced modulation of memory formation, the present study examined whether LTP induction is affected by $Zn^{2+}$ signaling via the cooperative action of noradrenaline and glucocorticoid.In anesthetized rats, CA1 LTP was not changed by perfusion of isoproterenol, an adrenergic β-receptor agonist, or 50 ng/ml corticosterone but enhanced by both perfusions. CaEDTA, an extracellular $Zn^{2+}$ chelator, canceled the LTP enhancement. When isoproterenol and corticosterone were added to hippocampal slices, intracellular $Zn^{2+}$ was decreased in the CA1. These data suggested that the decrease in intracellular $Zn^{2+}$ under the cooperation of noradrenaline and glucocorticoid enhances CA1 LTP. In the hippocampal DG, DG LTP was not attenuated by 50 ng/ml corticosterone but by 500 ng/ml. Additionally, DG LTP was not enhanced in the presence of noradrenaline and corticosterone.The present study suggests that CA1 LTP is vulnerable to stress-related $Zn^{2+}$ signaling via the cooperative action of noradrenaline and glucocorticoid compared with DG LTP.

### [3P-007]

### The effects of the paired associative premotor-cerebellar stimulation on the non-targeted cerebellar function in healthy subjects

*Satoko Koganemaru[1], Kazuki Tanaka[1], Atsushi Shima[1], Erika Omae[1], Akari Ogawa[1]
([1]*Department of Regenerative Systems Neuroscience, Human Brain Research Center, Graduate school of Medicine Kyoto University*)

[Background] Paired associative stimulation (PAS) is a combination of two stimuli over pre- and post-synaptic sites to induce spike-timing dependent plasticity (STDP). Recently, we have found that the PAS of the cerebellum and the contralateral premotor area (PMA) could enhance cerebellar function in the targeted side by using transcranial magnetic stimulation (TMS) in healthy participants. However, the change in the non-targeted side has been unknown. [Methods] Six healthy adults (3 females) were given the PAS in which 120 times of paired stimuli of the first stimulus on the right PMC (intensity: 90% rMT) followed by the second stimulus on the left cerebellum (intensity: SI 1mV) with 20ms of interstimulus interval every six seconds, 120 times in total. We evaluated the cerebellar brain inhibition (CBI) in the right and left cerebellum. [Results] After the PAS, CBI in the non-targeted side tended to decrease while CBI in the targeted side increased. [Discussion] Our findings suggested that the change of CBI in the non-targeted side may have been brought by the modulation of the cerebellum and M1 activity in the PAS targeted side.

### [3P-008]

### Dose-dependent bidirectional modification of synaptic transmission by gadolinium ion

Zorigt Odgerel[2], *Hiroki Yasuda[1], Takahito Nakajima[2], Yoshito Tsushima[2] ([1]*Saga University,* [2]*Gunma University*)

Gadolinium-based contrast agents (GBCAs) are commonly used in magnetic resonance imaging (MRI) examination. GBCAs remain in some brain regions after MRI examinations and they could damage brain tissues. Here, we report that gadolinium induces bidirectional changes in efficacy of synaptic transmission in the CA1 region of the mouse hippocampus depending on its concentration. A low concentration of gadolinium (100 μM) potentiated field excitatory postsynaptic potentials (fEPSPs) with a decrease in paired-pulse ratio (PPR) and induced a robust increase in frequency but no significant change in amplitude of miniature and spontaneous excitatory postsynaptic currents (mEPSCs and sEPSCs, respectively), indicating that 100 μM gadolinium enhances glutamate release at presynaptic sites. On the other hand, high concentrations of gadolinium (500–1000 μM) induced group 1 metabotropic glutamate receptor (mGluR)- and endocannabinoid (eCB)-dependent long-term depression (LTD). Finally, we found that EPSCs were not affected by a macrocyclic GBCA (Gd-GOTA), however, evoked EPSCs was enhanced by a liner GBCA (Gd-DTPA) at 100 μM. Thus, gadolinium has concentration-dependent bidirectional effects on synaptic transmission and high concentration of gadolinium activate group 1 mGluRs and endocannabinoid signaling. Furthermore, gadolinium is fully chelated and its effects on synaptic transmission are inhibited by Gd-GOTA, but not by Gd-DTPA.

### [3P-009]

### Temporal and quantitative analysis of functional expression of $Ca^{2+}$-permeable AMPA receptors during LTP

*Yoshihiko Wakazono[1], Ryosuke Midorikawa[1], Kogo Takamiya[1] ([1]*Dept. of Integr. Physiol., Univ. of Miyazaki*)

In the present study, we attempted to examine whether $Ca^{2+}$-permeable AMPA receptors (CP-AMPARs) functionally contribute to long-term potentiation (LTP) expression, by using an electrophysiological and pharmacological approaches. Under whole-cell voltage-clamp conditions, using a CP-AMPAR-selective antagonist NASPM, we first demonstrated that NASPM-sensitive components functionally contributed to about 15% of AMPAR-mediated EPSC amplitude in basal conditions. Moreover, biochemical and immunochemical studies suggested that the components mainly arose from GluA1 homomer. Next, to examine whether CP-AMPARs play some role in LTP, we attempted to treat with NASPM at different time points (3 to 30 min) after LTP induction. LTP was almost completely impaired when NASPM was administrated at 3 or 10 min, whereas LTP was maintained when at 20 or 30 min although its potentiation was reduced. The results suggest that CP-AMPARs in the first 3-10 min of LTP might be involved in LTP maintenance, but it is unclear whether CP-AMPARs in the 20-30 min are involved. In addition, temporal and quantitative analysis in this experiment also revealed that functional expression of CP-AMPARs started to increase from approximately 10 min after LTP induction and reached up to more than 2-fold increase of basal levels at 30 min, and that the decay time significantly increased at 30 min. These results suggest that CP-AMPARs changed not only quantitative but also qualitative during LTP expression.

### [3P-010]

### Optical inactivation of hippocampal GluA2/3 AMPA receptor.

*Susumu Jitsuki[1,2], Takuya Takahashi[2], Kiwamu Takemoto[1] ([1]*Mie University, Graduate School of Medicine, Department of Biochemistry,* [2]*Yokohama City University*)

Ionotropic neurotransmitter receptors commonly form protein complex by various combinations with subunits and have distinct functional properties. For example, AMPA-type glutamate receptors (AMPA-Rs), which are well known to be important glutamate receptors for learning, are composed of variable combinations of four subunits, GluA1-4. The combinations of GluA1 homomer, GluA1/2 and GluA2/3 were known to be expressed in adult brain. AMPA-Rs with GluA1 subunits require plasticity-inducing stimuli and NMDA-Rs activation to be driven into synapses and serve to enhance neurotransmission. In contrast, GluA2/3 complex continuously replace synaptic receptors in a manner that maintains transmission (Shi, S-H. et al. Cell 2001, Takahashi, T. et al. Science 2003 etc.). Since subunit combinations affect the functions of ion channel, complexes lacking GluA2 subunit shows calcium permeability and high single channel conductance (Dingledine R et al. Pharmacol Rev 1999, Coombs ID et al. J. Neurosci. 2012). These observations support the idea that AMPA-Rs complexes should have different physiological functions in vivo. To elucidate their complex-specific functions in vivo, we have developed an optical technology for acute inactivation of synaptic GluA1 homomeric AMPA-Rs in *vivo* by chromophore-assisted light inactivation (Takemoto et al. Nat. Biotechnol. 2017). This technology enabled us to elucidate the function of GluA1 homomer in the acquisition of contextual fear memory in hippocampus. Based on this study, here we show that CALI method for GluA2/3, an AMPA receptor that does not contain GluA1. First, we screened GluA3 antibody, and obtained antibodies that showed high CALI efficiency of GluA2/3. By using eosin labeled this antibody for CALI, we found that CALI with this antibody impaired performance in hippocampus-dependent memory task 24h after learning but not after 1h. Our optical technique for inactivating synaptic proteins will enable elucidation of their physiological roles in cognition.

### Poster Presentation

[3P]
### Neurophysiology, Neuronal cell biology
**Neural network**

March 16 (Thu.), 12:10 - 14:10, Poster Room

### [3P-011]

**Hippocampal spike activity and sharp-wave ripples augmented and attenuated during reward expectation and acquisition respectively**

*Tomomi Sakairi[1], Masanori Kawabata[1], Alain Rios[1], Satoshi Kaneko[1], Yutaka Sakai[2], Yoshikazu Isomura[1] ([1]*Tokyo Medical and Dental Univsesity,* [2]*Tamagawa University*)

The hippocampus plays a key role in storing episodic experiences, which may help predict and confirm an outcome (reward) to learn an optimal action. Hippocampal neurons encode information on predicting or responding to reward. Their synchronous oscillatory activity, sharp-wave ripple (SWR), also often occurs in a reward-expectable situation as well as in “consummatory” behaviors, e.g., drinking the reward after the action. However, it remains unclear how hippocampal neurons, individually or populationally, represent reward information if reward confirmation concurrently conflicts with prediction. Here, we recorded rats’ hippocampal CA1 activity regarding reward prediction and confirmation during the reward-alternation task under the head-fixed condition. They were required to release a pedal in response to a go cue tone. The reward was presented every other time so that they could predict reward or no reward before the action in each trial. The rate of SWR occurrence was augmented prior to the action following the go cue but attenuated in response to a success cue with reward. Likewise, some of task-related neurons augmented the spike activity by expecting reward and attenuated it by acquiring reward. Nevertheless, these and other task-related neurons similarly displayed synchronous activation with the SWRs despite different changes of spike activity by reward expectation and/or acquisition. In summary, the hippocampus can prioritize the prediction of the next reward over the confirmation of the current reward.

### [3P-012]

**Effects of enhanced KCC2 activity induced by dephosphorylation of Thr906 and Thr1007 on neuronal function.**

*Miho Watanabe[1], Kristopher Kahle[2], Atsuo Fukuda[1] ([1]*Department of Neurophysiology, Hamamatsu University School of Medicine,* [2]*Dept Neurosurgery, Pediatrics, and Cellular & Molecular Physiology, Centers for Mendelian Genomics, Yale School of Medicine*)

The $K^+/Cl^-$ cotransporter KCC2 is the main $Cl^-$ extrusion mechanism of CNS neurons and its role in $Cl^-$ homeostasis is essential for $GABA_A$ receptor mediated inhibition. KCC2 dysfunction has been implicated in neurological disorders. However, the regulatory mechanisms of KCC2 are not entirely understood. We previously reported that knockin mice expressing the homozygous phosphomimetic KCC2 mutations T906E/T1007E ($Kcc2^{E/E}$), which prevented the normal developmentally regulated dephosphorylation of these sites, exhibited early postnatal death from respiratory arrest and touch or pain-evoked status epilepticus associated with impaired KCC2-dependent $Cl^-$ extrusion. To further examine the role of phosphorylation in the regulation of KCC2, we generated knockin mice expressing the homozygous dephosphorylation of KCC2 mutations T906A/T1007A ($Kcc2^{A/A}$). $Kcc2^{A/A}$ mice exhibited reduced anxiety, deficit in social novelty recognition, and reduced startle response with enhanced KCC2-dependent $Cl^-$ extrusion. γ band power was reduced in resting EEG. Synchronized neuronal activity in the cortex was increased in $Kcc2^{A/A}$mice. Furthermore, susceptibility to pilocarpine induced seizures was increased in $Kcc2^{A/A}$ mice. These data demonstrated that precisely regulated KCC2 Thr906/Thr1007 phosphorylation is essential for GABA-mediated inhibition.

### [3P-013]

**Holographic microscopy for biological applications**

*Daisuke Kato[1,2], Quan Xiangyu[3], Osamu Matoba[4], Hiroaki Wake[1,2,4] ([1]*Department of Anatomy and Molecular Cell Biology, Nagoya University Graduate School of Medicine,* [2]*Division of Multicellular Circuit Dynamics, National Institute for Physiological Sciences, National Institutes of Natural Sciences,* [3]*Department of System Science, Kobe University Graduate School of System Informatics,* [4]*Center of Optical Scattering Image Science, Kobe University*)

Recent advances in optical imaging and optogenetics have enabled to visualize and manipulate the biological phenomena in living animals. Detailed neural activity related to learning and memory has now been revealed and it has become feasible to manipulate this activity to express brain functions. However, quantification of neural activity by two-photon $Ca^{2+}$ imaging has the problem of low temporal resolution. In addition, manipulation of neural activity by conventional optogenetics through the optic fiber can only simultaneously regulate the activity of neurons which has the same genetic background at same timing and making it difficult to control the activity of individual neurons. To resolve this issue, we recently developed a microscope with a high spatiotemporal resolution for biological applications by combining optogenetics with digital holographic technology that can modify femtosecond infrared laser beams. Using the holographic microscope, we have successfully integrated functional and molecular information of optical labeled cells by cell extraction with flow cytometry and single cell analysis. These findings provide accurate spatiotemporal information on neural activity, which may be useful in elucidating the pathogenesis of neuropsychiatric disorders that lead to abnormalities in neural activity.

### [3P-014]

**Functional characterization of bitter taste-relaying neurons in the parabrachial nuclei**

*Makoto Sugita[1], Kuniyo Yamamoto[1] ([1]*Department of Physiology and Oral Physiology, Graduate School of Biomedical and Health Sciences, Hiroshima University*)

Bitter taste information is transmitted to the parabrachial nuclei (PBN) via neurons in the solitary tract nuclei. We combined genetic tracing with immunohistochemical analyses in mice to functionally characterize bitter taste-relaying neurons in the PBN, which were fluorescently labeled by the transneuronal tracer tWGA-DsRed originating from bitter taste receptor cells. In the PBN, the tWGA-DsRed-labeled neurons were located rostrally in the external lateral PBN, and caudally in the medial PBN. Immunohistochemical detection for induction of the immediate early gene c-fos suggested that the tracer-labeled neurons in the external lateral PBN may receive the convergent inputs of bitter taste and viscerosensory aversive information, whereas those in the medial PBN may be selectively activated by bitter taste. Immunohistochemical characterization of the types of tWGA-DsRed-labeled neurons revealed that subsets of tracer-labeled neurons in the external lateral PBN, but not in the medial PBN, were CGRP-immunoreactive. Our data show functional differences between the bitter taste-relaying neurons in the medial PBN and those in the external lateral PBN. (There is no conflict of interest.)

### [3P-015]

**Cortical beta-band activity in common marmoset performing visually-guided saccade task**

*KUAN TING HO[1], CHIH YANG CHEN[2], HIROTAKA ONOE[3], TADASHI ISA[1,2,3] ([1]*Department of Neurobiology, Graduate School of Medicine, Kyoto University,* [2]*Evolutionary Systems Neuroscience Group, Institute for the Advanced Study of Human Biology (ASHBi), Kyoto University,* [3]*Human Brain Research Center, Graduate School of Medicine, Kyoto University*)

Saccadic eye movements reflect not only the ability of motor control but also higher brain functions such as attention. Although the subcortical networks for the pure motor component of saccades have been well-studied, how saccades are influenced by top-down signals remains less clear. To address this issue, we implanted a whole-hemispheric electrocorticography array on a marmoset performing visually guided saccade task. In this task, the marmoset has to fixate on a central stimulus for 250 ms. Then the central stimulus disappears, and a peripheral stimulus shows up, which the marmoset is required to make a saccade to. In another condition, a 150 ms gap period was inserted between the offset of the central stimulus and the onset of the peripheral stimulus, during which the marmoset is required to maintain fixation. Catch trials are also included in both conditions, where central fixation is still required but the peripheral stimulus never shows up. In the results of time-frequency analysis, we focused on the beta-band (12~35 Hz) power, which is considered to mediate top-down signal, and observed differences not only between ipsi- and contra-lateral saccades but also saccades of different response latencies. The gap and catch designs enabled us to dissociate influence of various task events on these activity differences, and helped us identify top-down effects for saccade modification.

### [3P-016]

### Gamma frequency light flicker and auditory tone stimulation induces rapid cortex-wide neroglial $Ca^{2+}$ elevations

*Zihan Xu[1], Hiromu Monai[1] ([1]*Ochanomizu Univ.*)

Gamma oscillations (20-120 Hz ), a synchronous neural activity, are associated with higher neural activities. Abnormal reductions in gamma oscillations are found in patients with Alzheimer's disease. Non-invasive light flicker or auditory tone stimulation at gamma frequencies, especially 40 Hz, can increase gamma frequency oscillations in mice's visual and auditory cortex, even from peripheral sensory organs such as the eyes or ears. It has been shown that amyloid-β levels were halved, spatial learning ability and memory were enhanced, and cognitive function was improved after the stimulation. These previous studies have focused on changes before and after gamma-frequency light flicker or auditory tone stimulation. However, it is unclear when and how the neuroglial network responds to the stimulus, as there are no examples of in vivo brain imaging observations of the activation process of neurons and glial cells during stimulation. To monitor cortex-wide $Ca^{2+}$ changes during/after 40 Hz light flicker and auditory tone stimulation, we employed transgenic mice expressing the $Ca^{2+}$ sensor protein G-CaMP7 in astrocytes and some neurons with transcranial macro imaging. So far, we found that 40 Hz visual stimulation for 1 minute induced astrocytic-like $Ca^{2+}$ elevation, and its frequency was significantly increased during/after the stimulus.

### [3P-017]

### The role of feeding-related appetite-stimulating signalling molecules in the higher olfactory cortical region in mice

*Ahasan Md Monjurul[1], Murata Yoshihiro[1], Taniguchi Mutsuo[1], Yamaguchi Masahiro[1] ([1]*Kochi Medical School*)

The appetite stimulating and suppressing neuromodulatory molecules regulate neuronal activity reflecting metabolic state and hedonic value, and also contribute to learning and memory. These molecules regulate feeding behavior, in which olfaction is heavily involved. The expression of feeding-related neuromodulatory signaling molecules were examined in the olfactory system including the olfactory bulb, olfactory tubercle (OT), and the other olfactory cortical area in mice, by quantitative real-time PCR. The OT was further divided into attraction-related anteromedial OT, aversion-related lateral OT and remaining central OT. Many molecules showed higher expression in the OT, especially in the anteromedial and central OT. Among the molecules examined we first chose orexin, an orexigenic neuropeptide produced in the hypothalamus, for functional analysis because its receptor is abundantly expressed in the anteromedial OT. Suppression of orexin signals by the local injection of receptor antagonist in the anteromedial OT, but not in the lateral OT or nucleus accumbens, reduced attraction and conversely induced aversion to the food-associated cue odor. These results indicating the crucial role of appetitive signal in the anteromedial OT in the odor-guided feeding behavior.

### [3P-018]

### Excitatory and inhibitory connectivities associated with global disinhibition promoting functional recovery from spinal cord injury

*Reona Yamaguchi[1], Yusuke Imoto[1], Tadashi Isa[1,2,3] ([1]*Institute for the Advanced Study of Human Biology (WPI-ASHBi), Kyoto University,* [2]*Department of Neuroscience, Graduate School of Medicine, Kyoto University,* [3]*Human Brain Research Center, Graduate School of Medicine, Kyoto University*)

Balance between excitation and inhibition plays a key role for cortical network reorganization. The change of excitation-inhibition balance is also considered as neural basis of plastic changes in brain. Our recent study showed that disinhibition occurred globally and nonspecifically across wide cortical areas in both hemispheres after the spinal cord injury. We hypothesized that excitatory and inhibitory connectivites would change to induce disinhibition associated with functional recovery. The aim of this study is to reveal the polarity of the connectivity associated with the motor recovery after spinal cord injury using statistical causal discovery method (linear non-Gaussian acyclic model, LiNGAM). As a result, inhibitory connectivies from ipsilesional premotor cortex to contralesional primary motor cortex significantly decreased in the recovery process. On the other hand, excitatory connectivites in interhemispheric interaction did not change. These results suggested that decrease of inhibitory connectivies induced the disinhibition associated with functional recovery after spinal cord injury.

### [3P-019]

### Electrophysiological comparison of synaptic properties between zebrin-positive and -negative Purkinje cells in the mouse cerebellum

*Tianzhuo Wang[1], Izumi Sugihara[1] ([1]*Tokyo Medical and Dental University*)

Subsets of cerebellar Purkinje cells (PCs) that have a particular molecular expression profile are arranged into separate longitudinal stripes, which have different topographic afferent and efferent axonal connections to be involved in different functions. Expression levels of many molecules such as glycolysis enzyme aldolase C, glutamate transporter EAAT4, postsynaptic enzymes PLCβ4, and PKCδ, and ion channel TRPC3 are linked together among PC subsets, suggesting different physiological properties among them. In our previous study in which we recorded from PCs of different “zebrin types” (zebrin-positive = aldolase C-positive = Z+; and Z-) in identified neighboring stripes in vermal lobules IV–V and VIII in cerebellar slice preparation from Aldoc-Venus mice, we reported that intrinsic excitability and plasticity of intrinsic excitability and parallel fiber (PF)-PC synaptic long-term potentiation are enhanced more in Z- PCs than in Z+ PCs. This study focused on the climbing fiber (CF)-PC synaptic property and PF-PC synaptic long-term depression. Our preliminary results showed some significant differences in these CF and PF synaptic properties between Z+ and Z- PCs in lobule IV–V. The results suggest that cellular and synaptic physiological properties of Z+ and Z- PCs differentially tune these PCs to the cerebellar compartments involved in the control of different motor and non-motor functions.

### [3P-020]

### Functional connectivity of multiple regions of the brainstem induced by high-intensity exercise in rats

*Hiroyasu Ichihara[1], Ko Yamanaka[1], Hidefumi Waki[1] ([1]*Graduate School of Health and Sports Science, Juntendo university*)

To exert and maintain a high performance, understanding the physiological responses during high-intensity exercise (HIE) is vital for athletes. The neuronal regions of the brainstem, including the area postrema (AP), nucleus tractus solitarius (NTS), and lateral parabrachial nucleus (LPBN), receive inputs from higher brain areas and the periphery and are involved in cardiovascular regulation. This study aimed to determine whether these brainstem areas are activated and functionally connected during HIE. Male Wistar rats ($n = 26$) were subjected to treadmill exercise (HIE [34 m/min] or low-intensity exercise [LIE; 20 m/min]) for 90 min. To determine which brainstem neurons were activated during HIE and have neuronal projections, a retrograde tracer (cholera toxin subunit B [CTB]) was injected before the treadmill exercise, and c-Fos immunohistochemistry was performed thereafter. Activity of the AP, NTS, and LPBN was increased with exercise intensity and revealed a significant correlation between the AP-LPBN in HIE ($r = 0.78$, $p < 0.05$) and NTS-LPBN in LIE ($r = 0.76$, $p < 0.05$) exercise. In addition, CTB injected into the LPBN was labeled with AP neurons and double-labeled with c-Fos in HIE. The study demonstrated that the activity and functional connectivity of these brainstem areas change with exercise intensity, and functional connectivity between AP and LPBN occurs during HIE.

### [3P-021]

### Cortical adaptation of the night monkey to a nocturnal environment indicated by T1w/T2w myelin mapping

*Takuro Ikeda[1], Joonas Autio[1], Akihiro Kawasaki[1], Chiho Takeda[1], Takayuki Ose[1], Masahiko Takada[2], David Van Essen[3], Matthew Glasser[3], Takuya Hayashi[1] ([1]*RIKEN,* [2]*Kyoto University,* [3]*Washington University in St. Louis*)

Neuroimaging studies using nonhuman primates (NHPs) are crucial for understanding the structure, function, and evolution of the human brain. Here we extended high-quality MRI and cortical surface-based analysis methods in humans to NHPs and applied them to the night monkeys (*Aotus lemurinus*), macaques (*Macaca mulatt*a and *Macaca fascicularis*), and marmosets (*Callithrix jacchus*). MRI data were collected using a 3T MRI scanner and multi-array RF coils designed for NHPs. Structural images were acquired using 3D T1 and T2-weighted (T1w and T2w) scans under deep anesthesia and were analyzed using a NHP version of Human Connectome Project (HCP) pipeline, which generated myelin maps with T1w/T2w ratio. Despite the significant difference in gyrification pattern, these three species share a similar pattern of cortical thickness and myelination: thin and heavily myelinated in early sensory areas and thick and lightly myelinated in association areas. Detailed analysis of cortical myelin isolated three regions in the parieto-temporal cortex: MT+ complex, auditory cortex, and Brodmann area 7 (BA7). Quantitative comparisons revealed that MT+ complex and auditory cortex were significantly larger in night monkeys than in marmosets or macaques. The expansion of areas related to visual motion and audition in night monkeys suggests the cortical adaptation to the nocturnal niche environment.

### [3P-022]

### The role of the medial prefrontal cortex in the nicotine-induced facilitation of object recognition memory encoding and retrieval in mice

Hirohito Esaki[1], Takanori Kitanaka[1], Shoma Izumi[1], Ayumu Inutsuka[2], Akihiro Yamanaka[3], Kazuki Nagayasu[4], Shuji Kaneko[4], Naoya Nishitani[1], Satoshi Deyama[1], *Katsuyuki Kaneda[1] (*[1]Lab Mol Pharmacol, Inst Med Pharmaceut Health Sci, Kanazawa Univ, [2]Division of Brain and Neurophysiology, Dept Physiol, Jichi Med Univ, [3]Res Inst Environ Med, Nagoya Univ, [4]Dept Mol Pharm, Grad Sch Pharm Sci, Kyoto Univ*)

We have previously reported that nicotine (Nic) facilitates object recognition memory (ORM) encoding via acting on the medial prefrontal cortex (mPFC) in mice. In this study, we examined the detailed mechanisms of Nic-induced facilitation of ORM encoding and whether Nic also facilitates ORM retrieval using the novel object recognition test in male C57BL/6J mice (7 – 12 weeks). Consistent with the facilitated effect of intra-mPFC Nic infusion (0.3 µg/side) on ORM encoding, suppression of mPFC excitatory neurons with inhibitory DREADD (hM4Di) or optogenetics (eArchT3.0), or selective suppression of mPFC-perirhinal cortex (PRH) pathway with hM4Di significantly inhibited the systemic Nic-induced (0.1 mg/kg; s.c.) facilitation of ORM encoding. Moreover, selective activation of mPFC-PRH pathway with excitatory DREADD (hM3Dq) facilitated ORM encoding. On the other hand, systemic injection, but not intra-mPFC infusion, of Nic facilitates ORM retrieval, and this effect was inhibited by suppression of mPFC excitatory neurons with hM4Di. Additionally, activation of mPFC excitatory neurons with hM3Dq facilitated ORM retrieval. These data suggest that Nic facilitates ORM encoding and retrieval, respectively, via activation of mPFC-PRH pathway and indirect activation of mPFC excitatory neurons.

### [3P-023]

### A multi-reservoir model enabling flexible behavior based on the presence of rule without reorganization of neural circuits.

*Yuuto Miyamura[1], Tomohiko Yoshizawa[2], Riichiro Hira[1], Yoshikazu Isomura[1] (*[1]Tokyo Medical and Dental University, [2]Hokkaido University*)

Animals live in a changing world by detecting rules in their surrounding environment and transforming their behaviors based on these rules. Although reorganization of neural circuits by synaptic plasticity is important for flexible behavior, repeating synaptic plasticity may lead to unneeded learning in rapidly changing environments. How can the brain enable flexible behavior to rule changings without reorganization of neural circuits? To address this issue, we first trained rats in an environment where rewards were alternately given on each trial and randomly given. The rats showed stronger anticipation of reward in the alternate condition, which quickly disappeared when the environment was switched to the random condition. Next, we prepared three reservoir models as follows. The first model input cue and reward, and output behavior. The second model input cue and reward plus the reward prediction error and output behavior. The third model had the main-reservoir, which input cue and reward and output behavior, and sub-reservoir, which input only reward prediction error and output to main-reservoir (multi-reservoir model). The results showed that the multi-reservoir model reproduced the behavioral patterns of rats most robustly. These results predict the presence of a sub-reservoir in the actual brain such that the reward prediction error holds for seconds. We suggest that hierarchical organization of mammalian frontal cortex may reflect a mechanism by which multiple reservoirs allow for highly adaptive behavior without neural circuit reorganization.

### [3P-024]

### Modulation of TRPA1 and TRPV1 on respiratory rhythm at the pons in isolated brainstem-spinal cord preparation from neonatal rat

*Naoko Masutani[1], Yuki Kosaka[1], Takuya Tsujimura[1], Chihiro Tarumi[1], Akiko Arata[1] (*[1]Dept. of Physiome , Hyogo Medical University*)

The thermosensory TRP channels TRPA1 and TRPV1 are known to be activated by endogenous factors that not only cause acute pain but are also involved in inflammation of primary afferent nerves. TRPA1 and TRPV1 are predominantly expressed on myelinated A delta fibers and unmyelinated C fibers of peripheral nerves and are found in the axons of the spinal, vagus, and trigeminal nerves. However, the relationship between respiratory rhythm and pontine-level nociception has not been fully investigated. In this study, we investigated the effects of TRPA1 and TRPV1 on the respiratory rhythm in the parabrachial nucleus (PBN), which is known as a nociceptive coupling system. First, we examined the effects of TRPA1 and TRPV1 on respiratory rhythms in pons-medullary-spinal cord preparations isolated from postnatal day 0–2 rats and compared them with medulla-spinal cord preparations. Respiratory activity was recorded from the cervical fourth (C4) ventral nerve root. The TRPV1 agonist capsaicin promoted respiratory rhythm, whereas the TRPA1 agonist cinnamaldehyde inhibited respiratory rhythm and decreased respiratory activity with shorter amplitude. These effects were shown in the preparations with the pons but not in the preparation without the pons. Furthermore, we investigated whether the involvement of GABAergic inhibition in the PBN was responsible for inhibition induction by TRPA1. Because respiratory depression was blocked by the GABAA antagonist bicuculline, we hypothesized that TRPA1 might be mediated by the GABAergic inhibitory system in PBNs and have descending depression in response to nociception. These results suggested that 1) TRPA1 and TRPV1 regulate respiratory rhythm with opposite effects; 2) the analgesic effect of TRPA1 on respiration may be modulated using the PBN inhibition system.

### [3P-025]

### Effect of dopamine on input frequency dependency of neuronal activities in striatal projection neurons

*Haruka Fujie[1], Atsushi Tamura[1], Tomoki Sueoka[1], Kazuto Kobayashi[2], Makoto Osanai[1,3,4] (*[1]Osaka Univ, [2]Fukushima Medical University, [3]Tohoku Univ, [4]Cinet*)

### [3P-026]

### Modulation of Sense of Agency during visual feedback task brings altered hemodynamic responses in cerebral cortex

*Hiroki Kuwamura[1], Masato Tsuyuhara[2], Kitano Kento[1], Duc Le Trung[2], Hisato Nishijo[3], Masahito Mihara[4], Naoto Fujita[2], Susumu Urakawa[2] (*[1]Hiroshima University, [2]Dept of Musculoskeletal Functional Research and Regeneration, Grad Sch of Biomedical and Health Sci, Hiroshima University, [3]Dept of System Emotional Sci, Grad Sch of Medicine and Pharmaceutical Sci, University of Toyama, [4]Dept of Neurology, Kawasaki Medical School*)

In rehabilitation medicine, the Sense of Agency (SoA) is thought to decrease when motor dysfunction occurs by diseases such as stroke hemiplegia, or Parkinson's disease. It is clinically accepted that maintaining the SoA is an important factor in improving rehabilitation effectiveness. But the neurophysiological mechanisms for the perception of SoA are still not understood. The objective of this study was to develop the experimental set-up which modified SoA by visual-feedback task and evaluate the neurophysiological mechanisms. In this study, the subject performed totally 16 circular-tracking tasks under two different feedback conditions, Normal (congruent with monitor cursor and subject's movement by stylus pen) and Modified (incongruent with those because of modified output conversion) condition. SoA under each condition was assessed by questionnaires on a seven-point scale. Task performance (how subjects could trace the target cursor) in the Modified condition was lower than Normal condition. It was also clear that task performance in the Modified condition gradually increased in the later tasks. The questionnaire results revealed that the SoA of Modified condition was lower than those of Normal condition. We found that hemodynamic responses in some cerebral cortex correlated with SoA. Our results might improve the basic understanding of neurophysiological mechanisms of SoA.

### Poster Presentation

[3P]

### Neurophysiology, Neuronal cell biology

**Neurochemistry**

March 16 (Thu.), 12:10 - 14:10, Poster Room

### [3P-027]

**Decreased cell density and augmented oxidative stress in parvalbumin-expressing interneurons in the medial prefrontal cortex of schizophrenia-like *PlexinA1*-deficient mice**

*Kazunori Yukawa[1], Mst Sharifa Jahan[1], Takamasa Tsuzuki[1], Takuji Ito[4], Md. Eliusur Rahman[1], Ikuko Takahashi[2], Hyota Takamatsu[3], Atsushi Kumanogoh[3], Takayuki Negishi[1] ([1]*Department of Physiology, Faculty of Pharmacy, Meijo University,* [2]*Radioisotope Center, Faculty of Pharmacy, Meijo University,* [3]*Department of Immunopathology, Immunology Frontier Research Center, Osaka University,* [4]*Department of Neurology, Aichi Medical University*)

PlexinA1 (PlxnA1) is a transmembrane receptor for semaphorins (Semas), a family of axonal guidance cues vital during neural development. PlxnA1 is expressed in embryonic interneurons, and *PlxnA1* deletion in mice leads to less interneurons in the developing cortex. *PlxnA1* KO mice exhibited significantly increased self-grooming and reduced prepulse inhibition, a schizophrenia endophenotype. However, the mechanism underlying the abnormal behavior of *PlxnA1* KO mice remains unclear. We confirmed *PlxnA1* mRNA expression in parvalbumin-expressing interneurons (PV cells) in the medial prefrontal cortex (mPFC) of adult mice. Immunohistochemical analysis (IHC) showed significantly decreased densities of both GABAergic neurons and PV cells in the mPFC of *PlxnA1* KO mice compared with wild type mice (WT). PV cells were shown to express flavoenzyme MICAL1, an effector in Sema-Plxn signaling for axon guidance, indicating co-expression of MICAL1 and PlxnA1 in PV cells. IHC of 8-oxo-dG revealed significantly increased oxidative stress in *PlxnA1*-deficient PV cells compared with WT. Thus, increased oxidative stress and decreased PV cell density in the mPFC may determine the onset of *PlxnA1* KO mice's abnormal behavior. Accordingly, deficient PlxnA1-mediated signaling may increase oxidative stress in PV cells, thereby disrupting PV-cell networks in the mPFC and causing the abnormal behavior.

### [3P-028]

**The impacts of occlusal disharmony as a risk factor on dementia**

*Munehisa Maeshiba[1,3], Hiroshi Kajiya[2,3], Takashi Tsutsumi[4], Keisuke Migita[5], Kazuko Goto-T[6], Yuri Kono[3], Kei Egashira[3], Takashi Tsuzuki[1], Jun Ohno[3] ([1]*Department of Oral Rehabilitation, Fukuoka Dental College,* [2]*Department of Physiological Science and Molecular Biology, Fukuoka Dental College,* [3]*Oral Medicine Research Center,* [4]*Department of General Density, Fukuoka Dental College,* [5]*Department of Drug Informatics, Faculty of Pharmaceutical Sciences, Fukuoka University,* [6]*Department of Dental Hygiene, Fukuoka College of Health Sciences*)

Occlusal disharmony has been reported to affect not only by cytokine and steroid hormone secretion and sympathetic activation in peripheral organs, but also by neurotransmitter release in the central nervous system. We previously reported that occlusal disharmony using hyperocclusion model mice promotes the secretion of inflammatory factors such as chemokines in periodontal ligament tissues and alveolar bone resorption, leading to occlusal traumatism. The dementia associated with Alzheimer's disease has been known to be dependent on the balance between amyloid-b (Aβ) and phosphorylated tau accumulation and clearance of these molecules in the brain. However, little is known about whether occlusal disharmony have effect on the expression of cognitive suppressor molecules and cognitive abilities. The present study is aimed to elucidate the relationships among occlusal disharmony, cytokine and cognitive suppressor molecule expression in the brain, and the impairment of learning and memory cognition. Hyperocclusion dramatically increased interleukin-1β expression in the serum and hippocampus 1 week after hyperocclusal loading in 2-month-old mice, but no effects in 12-month-old mice. The expression levels of Aβ and phosphorylated tau were significantly upregulated 1 week after hyperocclusal loading in the hippocampus of 2-month-old mice but were constant in 12-month-old mice. The social and long-term cognitive abilities in the 2-month-old mice were transiently downregulated close to the level of the 12-month-old mice 1 week after hyperocclusion and recovered to close to basal level via the expression of cognitive suppressor clearing/extrusion proteins. The cognitive abilities in Aβ precursor knock-in mice (C57BL/6-$App^{<tm1(NL-G-F)>}$) also downregulated at 1 week after hyperocclusion. The expression levels of Aβ and phosphorylated tau were significantly upregulated 1 week after hyperocclusal loading in the hippocampus of 2-month-old mice but were constant in 12-month-old mice. Occlusal disharmony-induced expression of cognitive suppressor molecules may contribute to the subsequent increase in their clearing proteins, which may be a defense against dementia progression in young individuals.

### [3P-029]

**CaMKII domain-mediated CASK function is involved in the molecular mechanism underlying the cerebellar hypoplasia in MICPCH syndrome.**

*Qi Guo[1], Emi Kouyama-Suzuki[1], Yoshinori Shirai[1], Takuma Mori[1], Katsuhiko Tabuchi[1] ([1]*Department of Molecular and Cellular Physiology, Shinshu University, School of Medicine*)

Microcephaly with pontine and cerebellar hypoplasia (MICPCH) syndrome is a neurodevelopmental disorder caused by the deficiency of X-chromosomal gene CASK. CASK has been shown to be involved in the synapse functions and cognitive behaviors, but the molecular mechanism that causes cerebellar hypoplasia in MICPCH syndrome remains elusive. In this study, we used CASK knockout (KO) mice as models for MICPCH syndrome and investigated the molecular mechanism by which CASK deficiency causes cerebellar hypoplasia. CASK KO cultured cerebellar granule cells (CGCs), generated by the infection with lentivirus expressing Cre recombinase in the CASK floxed cultured neurons, also showed progressive cell death that was rescued by co-infection with lentivirus expressing wild-type CASK. Rescue experiments by co-infection with lentiviruses expressing CASK deletion mutants identified that the CaMK, PDZ, and SH3, but not L27 and guanylate kinase, domains of CASK were required for the survival of CGCs. Missense mutations in the CaMK domain of CASK (R106P, L209P, R255H, and Y268H) failed to rescue the cell-death of cultured CASK KO CGCs. Machine learning based structural analysis using Alpha-Fold 2.2 predicted that the some mutations affect the binding to Liprin-a. These results suggest that the interaction with Liprin-a via CaMK domain of CASK may be involved in the mechanism underlying the cerebellar hypoplasia in MICPCH syndrome.

### [3P-030]

**Inflammatory factor expression alteration by very early exercise in rats with cortical infarction**

*Keigo Tamakoshi[1], Ami Saito, Akane Watanabe[1], Masahiro Motoyama[1], Wataru Arioka[1] ([1]*Niigata University of Health and Welfare*)

This study examined very early exercise's effects at 6 hours post-infarction on proinflammatory factors in rats with cortical ischemia (CIS). Subjects were randomly assigned to no training post-CIS (CIS), no training after sham surgery (SHAM), and very early treadmill exercise post-CIS (CIS + VET). CIS + VET performed treadmill exercise for 60 minutes at 6 hours post-CIS. All groups were evaluated for motor function using the ladder test and Rotarod test at 4 and 8 hours post-CIS. At 10 hours post-CIS, the brain was removed and analyzed for lesion volume and proinflammatory factors. For analyzing proinflammatory factors, IL-1b and TNF-b protein expression levels were determined using ELISA. Additionally, 40 chemokine, 25 interleukin, 8 tumor necrosis factor, and 11 other inflammation-related genes were analyzed using RT2 Profiler PCR Arrays (QIAGEN). Inflammatory factors were analyzed as cortical damage areas. Injury volume in the CIS + VET group was significantly higher than in the CIS group. TNF-b protein expression showed a trend toward higher levels in the CIS + VET group compared to the SHAM group. IL-1b was not significantly different among all groups. Inflammation-related genes in the CIS + VET group showed upregulation of CxCr1, Tnfsfl4, Ccl5, Ccr3, and Cd40lg and downregulation of Cxcl11, Cxcr5, Il11, Il7, Ccl20, Ccl9, and Cxcr2. The infarct volume expansion due to very early exercise post-CIS may involve changes in the expression of inflammation-related factors, primarily in the chemokine system. [Editor1]Remark: For clarity, please consider elaborating this.

### [3P-031]

**Alternative strategy of neocortex for driving voltage-oscillator of rats**

*Takako Fukuda[1], Takashi Tominaga[2], Yoko Tominaga[2], Nobuo Kato[3], Hiroshi Yoshimura[1] ([1]*Dept. of Molecular Oral Physiology , Institute of Biomedical Sciences, Tokushima Univ. Graduate School, Tokushima, Japan,* [2]*Institute of Neuroscience, Tokushima Bunri Univ. Kagawa, Japan,* [3]*Department of Physiology, Kanazawa Medical Univ. Ishikawa, Japan*)

Information integration in the brain requires functional connectivity between local networks. However, strategy for inter-regional coupling by the neocortex has been unclear. In the present study, we investigated the coupling mechanism from a viewpoint of oscillation, using optical recording method. Application of caffeine to rat visual cortex slice induced oscillatory activities between the Oc1 and secondary visual cortex (Oc2), in which the oscillation generator was located in the Oc2, and was triggered by feedforward signal. During to-and-fro oscillatory activities, neural excitation was marked in the layer II/III. When, upper layer were disrupted between the Oc1 and Oc2, feedforward signal could propagate through deep layer, and switched on the oscillator in the Oc2. Whereas, when lower layers were disrupted between the Oc1 and Oc2, feedforward signal could propagate through upper layer, and switched on oscillator in the Oc2. As for backward direction, neither upper layer cut nor lower layer cut could disrupt propagation of the oscillations. In all cases, horizontal and vertical pathways were used as needed. Fluctuation of oscillatory waveforms of local field potential at the upper and lower layers in the Oc2 was reverse. Thus, neocortex may work as a safety device of inter-regional communications, and layer II/III in the Oc2 play a role as a booster of synchronized oscillation.

### [3P-032]

### Analysis of Glial cells missing 1 binding site by ChIP-seq

*NUR AZRAH FAZERA BINTI MOHD ARIFFIN[1], Asmaa Abdullah[1], Yoshitaka Hayashi[1], Kazuhiko Nakabayashi[3], Yasuhiro Go[2], Seiji Hitoshi[1] (*[1]Shiga University of Medical Science, [2]National Institutes of Natural Sciences, [3]National Center for Health and Development*)

*Glial cells missing* (*Gcm*) was first discovered in *Drosophila* as a regulator for differentiation of neuroblasts into glial cells in the developing nervous system. This protein is a transcription factor and has been reported to bind to the GCM motif [(A/G)CCC(T/G)CAT)]. In the mammalian, *Gcm 1* and *2* present as homologues. We have reported that Gcms regulated *Hes5* expression in the developing brain. *Gcm1* knock out (KO) mice were embryonic lethal at E10 because of placental hypoplasia. On the other hand, Gcm2 KO (ICR background) also embryonic lethal at E12 because of abnormal development. The Gcm2 KO mice are particularly interesting because they show a phenotype in which the cortex is divided into several pieces. To know the target genes of *Gcm1* and *2*, we performed Chip-seq analysis using *Gcm1* and *2* overexpressed Neuro2a cells. Now, we finish to analyse of Gcm1 ChIP-seq data from three biological replicate data and revealed 6108 ChIP region common to all samples. This result also included *Hes5* gene, and we are currently in the process of analysing the detailed binding site to investigate new targets of Gcm1.

### Poster Presentation

[3P]
**Neurophysiology, Neuronal cell biology**
**Neurons, Synapses**

March 16 (Thu.), 12:10 - 14:10, Poster Room

### [3P-033]

**Deletion of *Setd1a*, a H3K4 methyltransferase, in cerebellar Purkinje cells impairs climbing fiber synapse elimination and attenuates inhibitory synaptic inputs during postnatal development**

*Takaki Watanabe[1,2], Shu Zhang[1], Kyoko Matsuyama[1], Masanobu Kano[1,2] ([1]*Graduate School of Medicine, The University of Tokyo,* [2]*WPI-IRCN, UTIAS, Univ. of Tokyo*)

In the mouse cerebellum, each Purkinje cell (PC) is innervated by multiple climbing fibers (CFs) at birth. Only single CF is strengthened by postnatal day 7 (P7) and extends its innervation along PC dendrites from P9. In parallel, the other CFs are eliminated from P7 to around P17. While many molecules involved in CF synapse elimination have been identified, little is known about the roles of epigenetic factors. Here, we investigated whether the H3K4 methyltransferase *Setd1a* is involved in CF synapse elimination using PC-specific conditional *Setd1a* knockout (Setd1a-cKO) mice. We examined CF innervation by counting the number of steps of CF-induced EPSCs from PCs in acute cerebellar slices. We found that the degree of multiple CF innervation was higher in PCs of Setd1a-cKO mice than control littermates at P8-P11 and P24-P26. In addition, miniature IPSCs were attenuated at P7-12 in Setd1a-cKO PCs. Because reduced GABAergic inhibition from around P7 to P12 is reported to cause impairment of CF synapse elimination (Nakayama et al., 2012), our results suggest that the deletion of *Setd1a* in PCs caused impairment of CF synapse elimination by reducing inhibitory synaptic inputs to PCs.

### [3P-034]

**Impact of a novel slow afterhyperpolarization (sAHP) on spike encoding by serotonergic (5-HT) dorsal raphe (DR) neurons**

*Masaru Ishibashi[1], Atsuo Fukuda[1], Christopher S. Leonard[2] ([1]*Department of Neurophysiology, Hamamatsu University School of Medicine,* [2]*Department of Physiology, New York Medical College*)

Serotonergic (5-HT) dorsal raphe (DR) neurons regulate numerous functions including behavioral state. We recently reported that in addition to activating a depolarizing cation current, orexin-A strongly enhances the post-spike afterhyperpolarization in 5-HT DR neurons. This orexin-enhanced AHP (oeAHP) requires $Ca^{2+}$ influx and has two distinct components: a shorter one, mediated by SK channel activation; and a longer one, that is apamin-insensitive (ai-oeAHP). The ai-oeAHP is a novel sAHP results from transient $Ca^{2+}$-mediated inhibition of the depolarizing orexin cation current. Here we have utilized current clamp and dynamic clamp recordings in mouse brain slices to investigate the role of the ai-oeAHP in regulating 5-HT DR neuron firing. Since membrane conductance is decreased during the ai-oeAHP, we postulated that synaptic responsiveness might be preserved during the ai-oeAHP. To test this, we delivered either a virtual ai-oeAHP or virtual classical sAHP conductance by dynamic clamp and compared their effects on responsiveness to a virtual EPSP burst. Preliminary findings indicate that spike induction by the virtual EPSP burst was better preserved during the ai-oeAHP.

### [3P-035]

**Ghrelin-induced cerebellar neuronal excitation depends on nutritional states**

*Moritoshi Hirono[1], Boyang Zhang[1], Masanori Nakata[1] ([1]*Dept Physiol, Wakayama Med Univ*)

Ghrelin, an orexigenic peptide and an endogenous ligand for growth hormone secretagogue receptor 1a (GHS-R1a), is expressed not only in the stomach but also in the brain. Our previous study reported that ghrelin facilitated not only firing of cerebellar Purkinje cells (PCs) but also GABAergic transmission onto them. The latter was attributed to presynaptic mechanisms involving the facilitation of action potential induction most likely in somatodendritic sites of molecular layer interneurons (MLIs). It has been reported that food deprivation or fasting enhanced the effects of ghrelin on functions in other brain areas to regulate feeding behavior and metabolism. Thus, we examined whether 24 h-fasting affects the ghrelin-mediated firing facilitation of MLIs and PCs using patch clamp recordings applied to mouse cerebellar slices. We found that fasting enhanced the ghrelin-induced facilitation of PC firing, whereas it attenuated the effect of ghrelin on MLIs. Thus, the attenuation was thought to in turn result in reinforcement of ghrelin-induced excitation of PCs. Our findings suggest that ghrelin regulates firing of PCs directly and indirectly, thereby impacting motor coordination and motor learning depending on nutritional states.

### [3P-036]

**Lobule-dependence of ghrelin-induced increase in spontaneous firing of cerebellar Purkinje cells**

*Fumiya Mima[1], Ryohei Aoki[1], Yuto Masuda[1], Boyang Zhang[1], Moritoshi Hirono[1], Masanori Nakata[1] ([1]*Dept Physiol, Wakayama Med Univ*)

The cerebellum plays a role in motor coordination and motor learning, and has been reported to be involved in cognition, emotion and reward. So far, the relationship between the cerebellum and feeding behavior has not been fully understood. Ghrelin, the main orexigenic peptide, is secreted during fasting, but little is known about its role in cerebellar function. Thus, we investigated expression patterns of ghrelin and its receptor growth hormone secretagogue receptor 1a (GHS-R1a) in the mouse cerebellar cortex using immunohistochemical techniques. We further clarified the magnitude of ghrelin-induced firing facilitation of Purkinje cells (PCs) among cerebellar lobes using patch clamp recordings under blocking synaptic transmission. Ghrelin was strongly expressed in PCs of the rostral cerebellar cortex, i.e, lobules 1–6, whereas GHS-R1a showed no obvious localization. Furthermore, PCs in lobules 1–6 showed a greater magnitude of ghrelin-induced firing facilitation than those in lobule 9. The rostral cerebellar cortex is thought to control complex movements of the trunk and limbs. Thus, it is conceivable that ghrelin could regulate motor learning for these movements during food deprivation or fasting.

### [3P-037]

**Simulation analysis on the contribution of inactivating potassium channel in frequency facilitation at hippocampal mossy fiber synapse**

*Fumeng Zheng[1], Haruyuki Kamiya[1] ([1]*Hokkaido University Graduate School of Medicine, Department of Neurobiology, Sapporo, Japan*)

Hippocampal mossy fiber synapse displays characteristic large frequency facilitation in response to repetitive stimuli. Activity-dependent broadening of axonal action potentials was demonstrated to contribute partly to mediating frequency facilitation at this synapse. Although accumulated inactivation of axonal potassium channels during repetitive action potentials has been postulated, the inactivation proceeds much more slowly (~ several tens ms) compared with the time course of action potentials (~ ms). In this study, we aimed at evaluating the contribution of potassium channel inactivation in use-dependent broadening of action potentials. In the computer simulation approach, we tested the effects of removing the inactivation of axonal potassium channels and found that the broadening of action potentials and the facilitation of calcium entry to the terminals were both completely abolished. These results suggest that potassium channel inactivation progressively accumulates during trains of action potentials and enhances calcium entry to support wide dynamic range frequency facilitation at this synapse.

### [3P-038]

### Effects of *GRIA1* mutations associated with neurodevelopmental disorders on the function of AMPA Receptors

*Taku Uchida[1], Kogo Takamiya[1] ([1]*Department of Neuroscience, Section of Integrative Physiology, Faculty of Medicine, University of Miyazaki.*)

The cause of neurodevelopmental disorders, including autism spectrum disorder (ASD) and attention-deficit/hyperactivity disorder (ADHD), is not clear. However, previous reports have described a strong influence of genetic factors and abnormal morphology of excitatory synaptic spines in the neuronal dendrites. Recently, a series of missense mutations in *GRIA1*, the gene of GluA1, a subunit of AMPA-type glutamate receptor (AMPA receptor), have been reported from patients with ASD and ADHD. At excitatory synapses, AMPA receptors form tetramers with GluA1-4 subunits in the postsynaptic membrane and function as ion channels. These genetic mutations could alter the function of AMPA receptors and affect the pathogenesis of neurodevelopmental disorders through excitatory synaptic dysfunctions. To investigate the above, we focused on missense mutations in *GRIA1* from intellectual disability patients diagnosed with ASD and/or ADHD. We generated mutant GluA1 expression vectors and performed the following experiments by gene transfection into HEK293T cells. The expression of AMPA receptors formed by mutant GluA1 on the membrane surface was examined using immunofluorescence staining. (2) The channel function of the mutant AMPA receptor was examined using the whole-cell patch clamp recordings. Immunostaining results showed that some of the mutants had significantly reduced cell surface expression compared to the wild type. On the other hand, electrophysiology results showed a diverse appearance for each mutation. In some mutations show none of agonist-induced current generation, while in the other was increased reversely. It is possible that dysfunctional AMPA receptors formed by the *GRIA1* mutations affect synapse formation and function, which in turn affects the pathophysiology of patients with these genetic mutations.

### [3P-039]

### Optical imaging of swallowing-related neurons in arterially perfused decerebrate rats.

*Shiori koyama[1], Nakayama Kiyomi[2], Nakamura Shiro[2], Ayako Mochizuki[2], Masanori Dantsuji[2], Kotaro Maki[1], Tomio Inoue[2] ([1]*Department of Orthodontics, Showa University School of Dentistry,* [2]*Department of Oral Physiology, Showa University School of Dentistry*)

The series of movements of pharyngeal muscles associated with the swallowing reflex are known to be produced by neural circuits in and around the nucleus tractus solitarius (NTS) in the medulla oblongata. In this study, we investigated the distribution of neurons associated with swallowing by real-time imaging of neuronal activity using intracellular $Ca^{2+}$concentration changes in the dorsal brainstem, mainly in the rostral part of the NTS, in arterially perfused decerebrate rats. To induce the expression of a $Ca^{2+}$-sensitive fluorescent protein in the dorsal brainstem, adeno-associated virus vector expressing GCaMP6f was injected at postnatal day 2 to 3, and the rats were kept in the cage with the mother for 3-4 weeks to allow for adequate protein expression. The swallowing reflex was induced by electrical stimulation of the superior laryngeal nerve (0.05-5 mA, 100 µs, 5 Hz), and compound action potentials were recorded from the cervical vagus nerve to monitor the swallowing reflex. Neurons with enhanced fluorescence intensity in response to the swallowing reflex were scattered at level from 0 to 3,000 µm rostrally to the obex and 2,000 µm bilaterally from the center. These results suggest that swallowing-related neurons are widely distributed in and around the rostral part of the nucleus tractus solitarius.

### [3P-040]

### Granule cells in the dentate gyrus produce 2-arachidonoyl glycerol and ameliorates kainate-induced seizures

*Yuki Sugaya Sugaya[1], Kenji Sakimura[2], Masanobu Kano[1] ([1]*The University of Tokyo,* [2]*Niigata University*)

The endocannabinoid 2-arachidonoyl glycerol (2-AG) is produced by diacylglycerol lipase α (DGLα) and mediates retrograde suppression of synaptic transmission, which is necessary for suppressing excessive excitability of neural circuits such as epileptic seizures. Previously, we have demonstrated that global DGLα knockout mice exhibit severe seizures in a kainate model of the temporal lobe epilepsy. However, it remains unclear whether the production of 2-AG in a specific cell population is sufficient to ameliorate seizures. To address this issue, we created dentate gyrus granule cell-specific DGLα knockout mice (gcDGLα -/- mice) by crossing pro-opiomelanocortin (POMC)-cre mice with DGLα floxed mice. The gcDGLα -/- mice lacked DGLα in dentate granule cells but exhibited intact DGLα expression in other hippocampal neurons when it was compared to their cre negative littermates (gcDGLα fl/fl mice) in immunofluorescent staning. We injected kainate (30 mg/kg, i.p.) to gcDGLα -/- and gcDGLα fl/fl mice and determined the latency to the onset of generalized tonic clonic seizures. We found that the latency was significantly shorter in gcDGLα -/- mice than in gcDGLα fl/fl mice. Furthermore, the latency of gcDGLα -/- mice was comparable to that of global DGLα knockout mice. These results suggest that the 2-AG-mediated signaling from granule cells in the dentate gyrus is crucial for ameliorating kainate-induced seizures. The authors declare no conflicts of interest associated with this presentation.

### [3P-041]

### Analysis of neurite degeneration caused by amyloid plaque using hippocampal slice culture

*Yiyao Zhou[1], Yuji Kamikubo[1], Takashi Sakurai[1], Hiroyuki Hioki[1], Yoko Ishida[1] ([1]*Juntendo University Graduate School of Medicine*)

Alzheimer's disease (AD) is usually a progressive neurodegenerative disease and is one of the most common causes of senile dementia. The formation of amyloid plaques and degeneration of neurites in the AD brain are pathognomonic features. Since the neurodegeneration associated with AD progresses gradually, it is difficult to analyze the early stages of the disease in patient brains or animal models. Therefore, we established a method to analyze amyloid plaque formation chronologically using rodent brain organotypic cultures. Slice cultures of rat and mouse hippocampus could be maintained for several months while retaining in vivo neural circuits and environment. We formed amyloid plaques in hippocampal slice cultures by seed injection and continuous administration of amyloid β. Furthermore, we found that neurites degenerated around these plaques. Our findings are useful for clarifying AD's initial pathophysiology and analyzing its progression. By elucidating the molecular mechanisms of neurite degeneration in cultured hippocampal slices, we can reveal the process of propagation of degeneration in neurodegenerative diseases.

### [3P-042]

### High-throughput screening method using frozen neurons

*Reiko T. Roppongi[1], Noriko Koganezawa[2], Yuko Sekino[4], Ayaka Higa[3], Tomoaki Shirao[3] ([1]*Gunma University Initiative for Advanced Research,* [2]*Department of Pharmacology, Gunma University Graduate School of Medicine,* [3]*AlzMed, Inc.,* [4]*Department of Veterinary Pathophysiology and Animal Health, Graduate school of Agricultural and Life Sciences, The University of Tokyo*)

Neuron culture is a valuable system that can be used for evaluating synaptic function and drug screening. However, the neuronal culture requires specific techniques to obtain good quality. Also, it takes time to check the quality of neurons in terms of subcellular localization of protein components, spine morphology, synapse development and plasticity. Here, we have developed a simple method of neuron culture using ready-to-use frozen stock (SKY neuron) to assess synaptic function. We cultured the SKY neuron in a 96-well plate for 21 days and then treated them with several concentrations of glutamate for 10 min and fixed them. Immunocytochemistry was performed, and we observed the dose-dependent reduction of drebrin cluster densities against glutamate stimulation. We performed validation studies using this method with several facilities. The method described is also useful to investigate the effect of Aβ on the synaptic state. These results suggest that the SKY neuron can be applied to evaluate the effect of drugs on the synaptic state. The culture method is useful in drug screenings, safety pharmacological studies, and evaluation of synaptic function.

### [3P-043]

### Presynaptic mu-opioid receptors modulate excitatory synaptic transmission from CGRP-containing lateral parabrachial neurons to the capsular central amygdala neurons in mice with inflammation

*Naoko Sato[1], Yukari Takahashi[1], Yae Sugimura[1], Fusao Kato[1] ([1]*Department of Neuroscience, The Jikei University School of Medicine*)

The inputs to the capsular central amygdala (CeC) from the lateral parabrachial nucleus (LPB) are potentiated in inflammatory pain models (Kato et al., 2018). We examined whether opioids affect this transmission and the influence of general inflammation. The LPB-CeC transmission was optogenetically activated in CGRP-cre mice. Lipopolysaccharide (LPS, 0. 5 mg/kg, i.p.) was administered 2 h before brain sampling, and light-evoked excitatory postsynaptic currents (leEPSCs) were measured in CeC neurons in slices. [D-Ala2,N-Me-Phe4,Gly5-ol]-enkephalin (DAMGO) significantly decreased leEPSC amplitude in a concentration-dependent manner (0.01-1 µM; EC50 = 0.09 µM), accompanied with a significant increase in the paired-pulse ratio, suggesting a presence of presynaptic mu-opioid receptors (MORs) at the CGRPergic/glutamatergic terminals. An inverse agonist for MOR, D-Phe-Cys-Tyr-D-Trp-Arg-Thr-Pen-Thr-NH2 (CTAP, 1 µM), significantly increased leEPSC amplitude, an effect likely to be more potent in LPS-treated animals, suggesting a constitutive activation of the presynaptic MORs. These results suggest that the opioid systems regulate the nociception/inflammation-associated inputs to the CeC [no COI to declare].

### [3P-044]

### Spatiotemporal dynamics of glutamate release from individual ribbon and non-ribbon regions in the goldfish retinal bipolar cell terminal

*Tomoko Oshima-Takago[1,2], Hirokazu Sakamoto[2], Yukihiro Nakamura[3,2], Shigeyuki Namiki[2], Kenzo Hirose[2], Masao Tachibana[4,1,2], Hideki Takago[1,2] ([1]*Dept. of Rehabilitation for Sensory Functions, Research Inst., Nat'l Rehabilitation Ctr. for Persons with Disabilities, Saitama, Japan,* [2]*Dept. of Pharmacol, Grad. Sch. of Med., The Univ. of Tokyo, Tokyo, Japan,,* [3]*Dept. of Pharmacol, Jikei Univ. Sch. of Med. ,Tokyo, Japan,* [4]*Ctr. for Systems Vision Science, Research Organization of Science and Technology, Ritsumeikan University, Shiga, Japan*)

The ribbon synapse in the retinal bipolar cell (BC) terminal continuously transmits visual information via $Ca^{2+}$-driven glutamate (Glu) release. Electrophysiological studies suggest that long depolarization of the goldfish Mb1 BC terminal triggers kinetically different (i.e., the fast and slow) components of Glu release. Here, we monitored the Glu release using a retinal bipolar cell-targeted type of enhanced glutamate optical sensor (BC-eEOS) under the whole-cell voltage-clamp. The spatiotemporal dynamics of the evoked Glu release as well as the spontaneous Glu release was examined in relation to the location of the fluorescently labeled synaptic ribbons. Our major findings are as follows: First, the evoked fast Glu signal is detected predominantly at active zones within 500 nm of the nearest neighbor ribbons (i.e., ribbon-associated regions). Second, the active zone was not located at every ribbon-associated region. Third, the deconvolution analysis of the evoked slow Glu signal enabled us to measure the latency of its peak around each ribbon in a single BC terminal. Finally, the spontaneous Glu signal is mainly detected away from the ribbon-associated regions (i.e., non-ribbon regions). We believe that the Glu imaging using the BC-eEOS provides a novel platform to optically analyze spatiotemporal dynamics of Glu release from individual active zones in the retinal BC terminal.

### Poster Presentation

[3P]
**Neurophysiology, Neuronal cell biology**
**Glia**

March 16 (Thu.), 12:10 - 14:10, Poster Room

### [3P-045]

### Glial cells missing 1 induce glial cells and angiogenesis in the injured region of brain

*Yoshitaka Hayashi[1], Yasuhiro Go[2], Kazuhiko Nakabayashi[3], Asmaa Abdullah[1], Nur Azrah Fazera Binti Mohd Ariffin[1], Zakiyyah Munirah Mohd Zaki[1], Seiji Hitoshi[1]
([1]*Department of Integrative Physiology, Shiga University of Medical Science,* [2]*Exploratory Research Center on Life and Living Systems (ExCELLS), National Institutes of Natural Sciences,* [3]*Department of Maternal-Fetal Biology, National Center for Health and Development*)

*Glial cell missing* (*gcm*) plays a critical role in glial cell development in Drosophila. However, the function of Gcm1 in the mammalian brain remains to be investigated because Gcm1-deficient mice are embryonic lethal around embryonic day 10. Using an *in utero* electroporation, we revealed that Gcm1 overexpression in the embryonic brain promotes the differentiation of neural progenitor cells into astrocytes and oligodendrocytes, and angiogenesis by Lif and Vegf. On the other hand, when the brain is injured, the increase of glial cells and angiogenesis are promoted and play an important role in repair. To clarify the relationship between Gcm1 and brain damage, we studied a cryo brain injury model using wild type and heterozygous *Gcm1* knockout mice (*Gcm1* het) and quantified gene expression level after brain injury. The results showed that *Gcm1* expression was upregulated in the brain 1-2 days after injury and suppressed in the *Gcm1* het. Furthermore, the expression of Lif, Vegfc, angiogenesis-related genes 1, and neural progenitor-related gene (Nestin) was significantly decreased in *Gcm1* het compared to wild type at 1-3 days after injury. These results suggest that Gcm1 induces neuro-gliogenesis and angiogenesis in brain injury through the secretion of growth factors.

### [3P-046]

### Astrocytic Na-K-Cl cotransporter type 1 attenuates seizures promoted by GABAergic excitation

*Dao Trong Nguyen[1], Masaru Ishibashi[1], Miho Watanabe[1], Adya Saran Sinha[1], Ruksana Yesmin[1], Atsuo Fukuda[1] ([1]*Hamamatsu University School of Medicine*)

During seizure-like events (SLEs), the action of GABA switches from inhibition to excitation. The specific NKCC1 inhibitor bumetanide can reduce the intracellular $Cl^-$ concentration ($[Cl^-]_i$) and thereby sustain the inhibitor effect of GABA in *in vitro* experiments. However, NKCC1 knock-out mice exhibited a more status epilepticus than WT littermates. NKCC1 expresses not only in neurons but in astrocytes. Therefore, the role of astrocytic NKCC1 in epilepsy generation needs to be investigated. We use the astrocyte conditional NKCC1 knock-out (astroNKCC1 KO) mice to reduce the astrocytic $[Cl^-]_i$. The SLEs were obtained from hippocampal CA1 pyramidal neurons and triggered by tetanus stimulation. In addition, the pilocarpine-induced seizure model was observed using the Racine scale in *in vivo* experiment. The SLEs in astroNKCC1 KO pyramidal neurons were more severe than in WT neurons. The underlying excitatory GABA current was significantly larger in astroNKCC1 KO neurons than in WT neurons. Consistent with *in vitro* results, astroNKCC1 KO mice were prone to seizure with *in vivo* seizure model. Our findings suggest a protective role of astrocytic NKCC1 in excitatory GABA-mediated seizures.

### [3P-047]

### Oligodendrocytes regulate the synchronized axonal conduction and required for motor learning

*Shouta Sugio[1,2], Akari Suzuki[1], Satoshi Hamada[1], Daisuke Kato[1,2], Hiroaki Wake[1,2]
([1]*Department of Molecular Cell Biology, Nagoya University Graduate School of Medicine,* [2]*Division of Multicellular Circuit Synamics, National Institute for Physiological Sciences (NIPS)*)

Oligodendrocytes (OCs) are myelin-forming glia in the central nervous system and regulated axon conduction. Recent studies have suggested that OCs are responded to the changes in neural activities and altered their myelin morphology to regulate the temporal patterns of neural activities, which are important for the motor learning acquisition. Although, the physiological significances of OCs on motor learning have been known in last decade, while the effects on the axonal conduction have not been cleared. Here, we examined whether the myelin structure and axonal conduction affected before and after motor learning task in mice. We found that the motor learning task induced the upregulation of *Mpb* mRNA (a myelin gene) and shortened the length of node of Ranvier in the motor learning associated neural tract. Using a combinational method of optogenetics (ChR2) and extracellular recording, we further measured the axon conduction and found that the propagation of ChR2-evoked antidromic spikes on the motor learning associated tract is synchronized by the motor learning task. The synchronization was significantly reduced by the chemogenic inhibition of OCs during motor learning session. Moreover, the increment of success trial of the motor task was highly correlated with the spike synchronization by OCs. These results suggested the oligodendrocyte responded to the changes in neuronal activity and adapt their morphology and myelination to regulate the temporal activity of neural populations via synchronizing axon conduction, which are important for the motor learning.

### [3P-048]

### High-speed imaging of calcium elevations under Aβ dimers exposure in astrocytes

*Kaito Nakata[1,2], Kohei Otomo[2,3,4], Hirokazu Ishii[1,2,3], Motosuke Tsutsumi[2,3], Ryosuke Enoki[1,2,3], Tomomi Nemoto[1,2,3,5] ([1]*School of Life Science, The Graduate University for Advanced Studies,* [2]*National Institute for Physiological Sciences, National Institutes of Natural Sciences (NINS),* [3]*Exploratory Research Center on Life and Living Systems, NINS,* [4]*Graduate School of Medicine, Juntendo University,* [5]*Research Institute for Electronic Science Hokkaido University*)

In the early stage of Alzheimer's disease (AD), it has been hypothesized that dendritic spine loss was induced by astrocytic glutamate release excessively via amyloid-β (Aβ) oligomers-evoked intracellular Ca2+ elevations [Liu *et al., Front. Neurosci*, 2019]. In this study, we applied a home-built two-photon laser-scanning microscope utilizing a spinning disk scanner [Otomo *et al., Anal. Sci.*, 2015] and performed high-speed $Ca^{2+}$ imaging of the primary cultured astrocytes expressing fluorescent indicators, cytosolic GCaMP6f. To quantify complex astrocytic calcium events, we utilize Astrocyte Quantification Analysis [Wang *et al., Nat Neurosci.*, 2019] which is an event-based machine-learning model. By applying 500 nM of $A\beta_{1\text{-}40}$ dimers, the frequency and the amplitude of the localized fast $Ca^{2+}$ elevation in the astrocytes were significantly increased compared to the pre-dose of the $A\beta_{1\text{-}40}$ dimers. This might be the fast demonstration that Aβ oligomers evoked localized fast $Ca^{2+}$ elevations. Our finding will help to understand the mechanism of astrocyte dysfunction in AD.

### [3P-049]

### Forelimb reaching exercise after intracerebral hemorrhage in rats causes better motor function recovery with adaptive cerebellar oligodendrogenesis

*Takeshi Shimizu[1], Shinya Ueno[1], Dewi Mustika[1], Hideki Hida[1] ([1]*Department of Neurophysiology and Brain Science, Graduate School of Medical Sciences, Nagoya City University*)

We previously reported that rehabilitative effects of forced limb use (FLU) after intracerebral hemorrhage (ICH) is due to a causal relationship between the cortico-rubral tract and motor function recovery. However, it is still unclear whether dynamic change of the cerebellum in the motor regulatory system is also induced by rehabilitation after ICH. Growing number of studies demonstrates that oligodendrocyte (OL) remodeling in adult mice is involved in motor learning, in which neural activity increase modulates myelination. In this study, we assessed whether rehabilitation after ICH or a motor skill learning itself influence oligodendrogenesis and cause adaptive changes in cerebellum-dependent motor regulatory system. We observed that forelimb reaching exercise significantly induced better functional recovery after ICH and increased the number of newly-born OLs and CC1+ mature OLs in the cerebellar nucleus. When we injected GSK3 inhibitor (SB216763) into the cerebellum to inhibit oligodendrogenesis, the effect of the reaching exercise on motor function recovery was attenuated. In addition, the reaching exercise caused a switch from the cortico-spinal pathway to the cortico-rubral pathway. These data suggest that OL remodeling under reconstruction of injured brain circuits by intervention therapy plays roles in functional recovery.

### [3P-050]

### $IP_3$ receptor type-1 is a key $Ca^{2+}$ channel controlling NMDAR-coagonist availability and synaptic plasticity

*Mark William Sherwood[1], Aurélie Amadio[1], Aymeric Oliveira[1], Philippe Ciofi[1], Thierry Lesté-Lasserre[1], Misa Arizono[1], Chihiro Hisatsune[2], Etsuko Ebisui[3], Katsuhiko Mikoshiba[4], Stéphane Oliet[1], Aude Panatier[1] (*[1]Univ. Bordeaux, INSERM, Neurocentre Magendie, Bordeaux, France, [2]Calpain Project, Tokyo Metropolitan Institute of Medical Science, [3]Laboratory for Developmental Neurobiology, RIKEN Brain Science Institute, Wako, Saitama, Japan, [4]ShanghaiTech University, Shanghai, China*)

The role of astrocytes in brain function was overlooked because they are electrically silent. It is now appreciated that astrocytes, in response to neuronal activity, exhibit $Ca^{2+}$-based excitability. In turn, astrocytes regulate neuronal function via $Ca^{2+}$-dependent gliotransmission. Numerous gliotransmitters, like d-serine/glycine (NMDAR co-agonists), have been identified and investigated using tools that broadly impact astrocytic $Ca^{2+}$ signalling. However, because the molecular mechanisms of gliotransmission are poorly understood, it has been impossible to dissect the various roles of gliotransmission precisely. Here we identify a novel astrocytic $Ca^{2+}$ release channel that regulates NMDAR-coagonist availability in the synaptic cleft and synaptic plasticity. Through identifying the molecular machinery of gliotransmission, we serendipitously discovered a new mode of gliotransmission with a critical role in synaptic plasticity. These findings enrich the $Ca^{2+}$ signalling toolkit of astrocytes and provide a unique avenue for understanding the role of astrocytes in physiology and pathophysiology.

### [3P-051]

### Single-cell Resolution Imaging of Microglial $Ca^{2+}$ Activity and Spatiotemporal Analysis of Its Dynamics

*Hiroshi Horiuchi[1,2], Junko Ishida[1], Junichi Nabekura[1,2] (*[1]National Institute for Physiological Sciences, [2]The Graduate University for Advanced Studies*)

Microglia are the sole resident immune cells in the central nervous system. Microglial $Ca^{2+}$ activity is critical to their surrounding environment according to various spatiotemporal scales. However, region-of-interest-based analyses on microglial activity used in previous reports have remained limited in their scope. Here, we accurately characterize the spatiotemporal properties of individual microglial $Ca^{2+}$ events in an awake state according to onset location using an event-based approach. Almost all events originate at their processes, and had non-propagative features. Their spatiotemporal dynamics significantly depended on the origin and propagative features. Microglial $Ca^{2+}$ propagative features of events generated at processes are unrelated to $Ca^{2+}$ amplitude. Surprisingly, their propagation and direction are precisely regulated at their process branches. Furthermore, the $Ca^{2+}$ activity is clearly suppressed by both the inhibition of neuronal activity and purinergic P2 receptor signaling. These results indicate a certain degree of spatiotemporal diversity in microglial $Ca^{2+}$ activity.

### Poster Presentation

[3P]

### Neurophysiology, Neuronal cell biology

**Higher brain function**

March 16 (Thu.), 12:10 - 14:10, Poster Room

### [3P-052]

**Decoding loss aversion of non-human primates during Balloon Analogue Risk Task using a utility-based computational model**

*Ryo Ito[1,2], Rikako Kato[1], Shin Ishii[2,3], Ken-ichi Amemori[1] ([1]*Institute for the Advanced Study of Human Biology, Kyoto University,* [2]*Graduate School of Informatics, Kyoto University,* [3]*International Research Center for Neurointelligence, The University of Tokyo*)

Loss aversion is the tendency to avoid losses rather than acquire equivalent gains. To characterize the degree of loss aversion, we adopted the Balloon Analogue Risk Task (BART), which can measure how the subjects make a decision under a probabilistic loss, and we modified it for macaque monkeys.In this task, a balloon was presented on a screen, and it swelled while the monkey gazed at the fixation point. The time of the burst was probabilistically determined by a uniform distribution. If the monkey made a saccade to the peripheral target, the balloon stopped swelling, and the monkey received a reward in an amount proportional to the size of the balloon (cash-out). However, the monkey could not receive a reward if the balloon burst before the cash-out.We found that the monkey basically searched for the optimal time at which the expected amount of rewards was maximized within a single daily session under an identical condition. Interestingly, however, the monkey showed a conservative strategy by choosing cash-out at an earlier time than optimal, especially in the early stage of a session.To explain such conservative behavior, we performed a behavioral analysis using a utility-based computational model. We decoded the degree of loss aversion and found that the loss aversion was high while the monkey exhibited such conservative behavior. This modeling approach allowed us to measure the dynamically changing degree of loss aversion.

### [3P-053]

**Effects of Food Viscoelasticity on Memory and Learning in Mice**

*Minori Tajima[1], Hiromu Monai[1] ([1]*Ochanomizu University*)

Mastication is a vital function not only to eat but also to maintain hippocampal functions. Molar loss has been shown to attenuate the ability of memory and learning dependent on the hippocampus in mice. In addition, it has been shown that the degree of neurogenesis in the dentate gyrus was less in mice with mastication disorders. Furthermore, human studies reported a correlation between bite force and cognitive function in subjects without mastication disorders. These findings suggest a link between chewing and the maintenance of hippocampal function. However, whether the strength of chewing affects this function has not been fully understood. In this study, we explored how chewing affects memory and learning ability in mice using gummy candies. Since gummy candies have unique viscoelasticity characteristics, we considered that feeding them with different viscoelasticity mice made it possible to evaluate the effects on memory and learning under conditions in which chewing itself occurred but at different strengths. The ability of memory and learning was evaluated by behavioral tests and the degree of neurogenesis in the dentate gyrus. We found that the mice given soft gummy candies in the behavioral test scored significantly lower than the mice given hard gummy candies so far. These results suggest that chewing may be essential to memory and learning ability.

### [3P-054]

**Decoding oscillatory power signals during risk-return decision-making in the prefrontal and premotor cortices**

*Takeshi Miyamoto[1,2], Reona Yamaguchi[3], Hirotaka Onoe[4], Tadashi Isa[1,3,4], Ryo Sasaki[1] ([1]*Graduate school of Medicine, Kyoto University,* [2]*Japan Society for the Promotion of Science,* [3]*Institute for the Advanced Study of Human Biology (WPI-ASHBi), Kyoto University,* [4]*Human Brain Research Center, Graduate School of Medicine, Kyoto University*)

The purpose of this study was to clarify the brain mechanisms for taking a balance across risk-return options, i.e., a high risk-high return (HH) option with occasional large rewards and a low risk-low return (LL) option with frequent small rewards. We trained macaque monkeys to choose one of two options assigned with various combinations of HH-LL and expected values (EVs). Behavioral data clearly showed that the monkeys preferred the HH option with higher EVs, suggesting that monkeys could flexibly integrate and/or segregate multiscale reward parameters for their decision. We then recorded neural oscillatory signals using electrocorticography (ECoG) implanted over prefrontal brain regions, including the prefrontal and premotor cortices, to ask how the brain guides the balanced risk-return decision-making. We found that the oscillatory powers were systematically modulated for HH-LL and EV variables. We further attempted to decode monkeys' choices from the oscillatory powers using Fisher's linear discriminant. We found the oscillatory powers, mainly in lower frequency bands, clearly explained the monkeys' choice behavior. Our findings imply that these regions are involved in computing the risk-return balance of given options in reward-dependent decision-making. In addition to the above results, we will discuss about our additional data obtained by application of optogenetic manipulations.

### [3P-055]

**Distinct expression patterns of Aldolase C, Phospholipase C beta 3, and beta 4 in the macaque cerebellum**

*Tatsuya Yamamoto[1,2], Yuko Yoshida[2], Takayuki Ose[3], Yumi Murata[2], Takuya Hayashi[3], Noriyuki Higo[2] ([1]*Tsukuba International Univ.,* [2]*National Institute of Advanced Industrial Science and Technology (AIST),* [3]*Institute of Physical and Chemical Research (RIKEN)*)

The parasagittal stripe-shaped compartments have been well characterized in the rodent cerebellar cortex by multiple molecular markers such as Aldolase C (Aldoc), Phospholipase C beta 3 (PLCB3) and Phospholipase C beta 4 (PLCB4). Here, we examined the immunoreactive expression of these markers in cerebellar cortex and deep cerebellar nuclei of adult rhesus macaque monkeys (Macaca mulatta) and compared it with previous reports in rodents. Signals of the three markers were all expressed homogeneously and intensively throughout the cerebellar hemisphere in macaque, of which pattern is strikingly different from that in rodents, a pattern of parasagittal stripes alternating with high and low immunoreactivity. In addition, the dentate nucleus (DN) of macaque showed strong signals both in the ventral and dorsal parts, although that of rodents has weaker Aldoc signals in its dorsal part than in the ventral. Thus, the macaque has a higher proportion of compartments with positive markers in the cerebellar hemisphere and dorsal DN than in the rodents. Since these molecular markers are involved in neural plasticity, their distinct expression patterns in macaque cerebrocerebellum may be associated with mechanism underlying cognitive and motor function highly developed in primates.

### [3P-056]

**How do the Cingulate-Motor Cortical Circuits Control Reward Learning and Decision-Making Behaviors?**

*Daigo Takeuchi[1] ([1]*The University of Tokyo School of Medicine*)

The prefrontal cortex is known to serve as a crucial hub in the brain for adaptive decision-making. Previous studies in humans and animals have demonstrated that dopamine signals a reinforcement signal such as a reward prediction error, (2) basal ganglia circuits code action values for learning and decision-making, and (3) the prefrontal cortex including anterior cingulate cortex underlies an ability to flexibly adapt the choice behaviors upon rule changes in the environment. The network dynamics underlying these processes, however, have remained to be understood. To address this problem, I investigated how cortical circuits connecting the anterior cingulate cortex (ACC) and the secondary motor cortex (M2) play a role in reward learning and decision-making. First, by using the modified rabies virus method, I identified anatomical projections from ACC to M2 in mice and rats. Next, I developed a novel behavioral paradigm that I called a "conditional action sequencing task" with which I could probe a rat's ability to flexibly update its sequential decision-making behaviors upon rule switches in the environment. With these foundations, I conducted two experiments using optogenetics and chemogenetics in combination with neural activity measurements in task-behaving rats. First, I tested how the silencing of activities of anterior cingulate cortical neurons affects an animal's behavioral performance in the task. I showed that the silencing of activities of anterior cingulate cortical neurons decreased animals' behavioral performance in trials that immediately followed rule changes in the task. Then, I tested how silencing of activities of anterior cingulate cortical neurons affects the neural activities of the downstream motor cortical neurons in M2. I found that silencing of activities of anterior cingulate cortical neurons excessively increased the neural activities of a class of downstream motor cortical neurons (positive outcome-activated neurons) during the outcome feedback period specifically in trials that immediately followed the rule changes. These data suggest that the ACC-M2 connections are a part of brain circuits that, based on the outcome feedback information, update the internal representations of how the environment works and use them for planning subsequent choice actions. This study highlights the roles of specific prefrontal cortical projections from ACC to M2 in flexibly updating the rule representations of sequential choice decisions upon a sudden change of rules in the environment.

### [3P-057]

### The importance of the thalamic reticular nucleus for motor sequence learning in mice

*Takako Ohno-Shosaku[1,2], Mitsugu Yoneda[1], Takashi Maejima[2], Yui Kikuchi[1], Mohan Wang[2], Michihiro Mieda[2] ([1]*Faculty of Health Sciences, Kanazawa University,* [2]*Department of Integrative Neurophysiology, Graduate School of Medical Sciences, Kanazawa University*)

The thalamic reticular nucleus (TRN) receives excitatory inputs from the thalamus and cortex, and sends inhibitory output to the thalamus. Therefore, it is possible that the TRN contributes to various brain functions through regulating the activity of thalamic relay neurons. In the present study, we examined whether the TRN plays an important role in motor sequence learning, by analyzing the performance of Avp-Vgat$^{-/-}$ mice in the Yoneda three-lever operant task, in which animals learn the sequence of lever presses by trial and error. Our histological and electrophysiological data demonstrated that vesicular GABA transporter (Vgat) was deleted in most of the TRN neurons and the inhibitory synaptic transmission from TRN to thalamic neurons was largely suppressed in Avp-Vgat$^{-/-}$ mice. Simple operant learning to press a lever for food reward in one-lever task was intact in Avp-Vgat$^{-/-}$ mice. However, the motor sequence learning in the three-lever task, in which the mice need to press three levers in a correct order for food reward, was severely impaired in Avp-Vgat$^{-/-}$ mice. These results strongly suggest that the TRN plays an important role in motor sequence learning. COI: NO.

### [3P-058]

### Decision-invariant memory traces of spatial goals in the dopamine-striatal circuits

*Norihiro Takakuwa[1], Hiroshi T. Ito[1] ([1]*Max Planck Institute for Brain Research*)

Flexible navigation requires the brain to choose a desired destination from numerous positions in the environment. The dopamine-striatal system is thought to support such a decision-making process, and previous studies reported that the dopamine release in the ventral striatum (vStr) increases as an animal approaches a destination, potentially reflecting the reward expectation at goals. However, it remains unclear if this increase in dopamine release is updated flexibly depending on the animal's goal choice, and if so, whether this activity influences navigational decisions. To address this question, we measured the dopamine release in the vStr, as well as spiking activity of vStr neurons, under a navigation task where an animal is required to update start and goal locations repeatedly. We found that both dopamine release and spiking activity increased as an animal approached a destination and changed flexibly depending on a pair of start and goal locations. However, in contrast to the idea of dopamine's role in decision making, these signals failed to discriminate the distances of remote navigational goals, and continued to increase at non-goal locations that had been rewarded in previous trials. These results suggest that the goal-directed ramp-up signals do not necessarily reflect an animal's decision, but rather represent the memory of spatial values from previous experiences.

### [3P-059]

### Coding of elapsed time in the hippocampal CA1 of rats during a time estimation task performance

*Fumiya Sawatani[1], Kaoru Ide[1], Hirotsugu Azechi[1], Susumu Takahashi[1] ([1]*Doshisha University*)

For most animal species, time perception ranging from few seconds to few minutes is essential for survival. Time perception has been suggested to be supported by calculations in multiple brain regions. However, how the brain estimates elapsed time remains unsolved. The hippocampus is involved in the temporal component of memory because several lines of evidence suggested that lesions in the hippocampus disrupt various types of the timing behavior. To address how the hippocampus contributes to time estimation, we established a behavioral task in which animals require explicit tracking of elapsed time for several seconds. We examined the representation of the time estimation in the hippocampal CA1 of four rats during the task performance by means of extracellular multiple single-unit recording. We found that many neurons in the hippocampus exhibited sustained activity with diverse temporal profiles of firing rate modulation during the monitoring of elapsed time. These firing activities may support the monitoring of elapsed time. In this presentation, we will report and discuss the features of the neural representation and its role in time estimation.

### [3P-060]

### The damaged area of mouse brain by depression studied using Mn MRI method

*Akio Inoue[1], Yuriko Inoue[2], Hiromitsu Ezure[2], Hiroshi Moriyama[2], Jyunji Ito[2], Chika Sawa[2], Maruto Ohtsuka[3], Koichi Shiraisi[3], Yoshinobu Manome[4] ([1]*Human Brain Research Center, Graduate School of Medicine, Kyoto University,* [2]*Dep. Anat., Showa Univ. Sch. Med.,,* [3]*Dev. Med. Eng. Jikei Uni. Med.,* [4]*Div. Mol. Cell Biol. Res. Cent. for Med. Sci., Jikei Uni. Med.,*)

The monoamine hypothesis is proposed as a mechanism of depression. However, it takes more than three weeks to recover from depression by anti-depressant drugs. Therefore, depression is not induced by the decrease in noradrenaline or serotonin, but that they may help the recovery of damaged area. Mn ions enter into nerve cells through membrane Ca-channel, depending on nerve activity, and Mn ions induce the increase of T1 signal of MRI. So, Mn-MRI is used to measure the nerve activity in vivo. We found that Mn ions inside the cells are released and disappeared by nerve activation. This study suggests that mouse should be activated only when Mn ions were charged. When Mn ions were injected into *abdominal cavity, Mn ions enter into blood vessel and finally Mn ions enter into brain through* chorioid plexus. *Mn concentration in the brain is maintained for few hours. Therefore, we applied restraint stress for three hours. Next day, we measured T1 signal of mouse brain* using Bruker 9.4T MRI machine. We found that mouse brain is strongly activated by restraint stress. Mouse becomes depression when restraint stress (3h) was repeated for three days. After they become depression we applied restraint stress with Mn ions, and we found that several place of brain were damaged when they become depression.

### [3P-061]

### Properties of olfaction and verbal ability in community-dwelling late or super elderly women

Fumino Okutani[1], *Risa Mitsuuchi[2], Tomoyuki Uematsu[2], Satoshi Ike[2] ([1]*Department of Occupational Health, Kochi Medical School,* [2]*Kochi Professional University of Rehabilitation*)

Forty-nine community-dwelling elderly women over 74 years old participated in this study. They attend community-based day programs every week. We excluded individuals with a history of neurological diseases which induce dementia as well as psychiatric diseases. Using Open Essence, odor-identification test card, their olfaction was examined. Verbal fluency test which consists of category fluency test (CFT) and letter fluency test (LFT) was subscribed. We also assessed their cognitive and basic living function by the Japanese version of the Montreal Cognitive Assessment (MoCA-J) and the Dementia Assessment Sheet for Community-based Integrated Care System-8 items (DASC-8). Only 5 people showed normal olfactory identification ability. Data of Open Essence had no significant relation with scores of MoCA-J and DASC-8. In DASC-8, all subjects had enough basic ADL (Activities of Daily Living) which consists of using toilet, eating and mobility although instrumental ADL was lost. CFT and LFT scores were significantly correlated with DASC-8 scores. Taken together, these results suggest that cognitive function of late and super elderly women is assessed by verbal fluency. It seems also important to practice verbal ability to prevent dementia in elderly people.

### [3P-062]

### fNIRS evidence of frontoparietal cortex activities during curved reach planning in different feedback conditions

*Duc Trung Le[1], Masato Tsuyuhara[1], Hiroki Ogawa[1], Kazuki Watanabe[1], Tatsunori Watanabe[5], Hisao Nishijo[2,3], Masahito MIhara[4], Naoto Fujita[1], Susumu Urakawa[1]
([1]*Hiroshima University, Graduate School of Biomedical and Health Sciences, Department of Musculoskeletal Functional Research and Regeneration,* [2]*University of Toyama, Graduate School of Medicine and Pharmaceutical Science, Department of System Emotional Science,* [3]*University of Toyama, Research Center for Idling Brain Science (RCIBS),* [4]*Kawasaki Medical School, Department of Neurology,* [5]*Hiroshima University, Graduate School of Biomedical and Health Sciences, Department of Sensorimotor Neuroscience*)

Planning reaching movements along given trajectory paths is necessary for daily life. While linear reaching planning (LRP) has been widely studied, little attention has been given to curved reaching planning (CRP). Reaching movements could be planned and executed under two types of visual feedback conditions: spatially coupled feedback (CF) (e.g., directly reaching for an object with congruent visual direction), and spatially decoupled feedback (DF) (e.g., maneuvering a computer cursor). The neural mechanism of CRP under these feedback conditions remains unclear. In this study, participants performed LRP and CRP in different feedback conditions, CF and DF. We measured their task-related frontoparietal activities by fNIRS. Our findings showed that, during the cue period, CRP exhibited stronger activity in the superior parietal and dorsal premotor cortices than LRP. Regarding the delay period, DF showed greater activity in the right prefrontal cortex during CRP than CF did. These results imply that the frontoparietal activities may play a critical role in processing trajectory-path representations for CRP. This work provides insight into the brain processes of reach planning under various feedback conditions.

### [3P-063]

### Emotional behavior affected by the absence of calmodulin kinase IIα activity

*Yoko Yamagata[1,2], Yuchio Yanagawa[3] ([1]*National Institute for Physiological Sciences,* [2]*SOKENDAI (The Graduate University for Advanced Studies),* [3]*Gunma University Gradudate School of Medicine*)

$Ca^{2+}$/calmodulin-dependent protein kinase IIα (CaMKIIα) is a key mediator of activity-dependent neuronal modifications and involved in the molecular mechanisms of learning and memory. It is also implicated in the regulation of emotional behavior. We previously reported that the kinase-dead CaMKIIα (K42R)-KI mouse showed decreased fear response after single training and generalized fear response after repeated training of fear conditioning. To understand more about the relationship between kinase activity of CaMKIIα and emotional behavior, here we performed unconditioned response tests to evaluate emotional behavior in the kinase-dead CaMKIIα-KI mouse. In the open field locomotion test, the KI mouse showed decreased time spent in the center area and increased traveling distance in the peripheral area, indicating the avoidance of an open space. On the other hand, in the elevated plus maze test, the KI mouse showed increased entry into and time spent in the open arms, indicating the preference for an open space. Based on these and other behavioral test results, we will discuss the emotional traits affected by learning and memory deficits in the absence of kinase activity of CaMKIIα.

### [3P-064]

### A novel virtual reality task for measuring strategic decision-making for balancing the reward and cost

*Masanobu Ishio[1], Tadashi Isa[1,2,3], Ryo Sasaki[1] ([1]*Department of Neuroscience, Graduate School of Medicine, Kyoto University,* [2]*Human Brain Research Center, Graduate School of Medicine, Kyoto University,* [3]*Institute for the Advanced Study of Human Biology (WPI-ASHBi), Kyoto University*)

Foraging theories in behavioral ecology predict that the animals efficiently take the balance between reward and cost, and temporally maximize the reward rate (reward amount per unit cost) in their decision-making in the natural environment. This behavior requires the brain to temporally integrate reward amount and cost (distance, complexity, effort etc..) and their analysis may lead to clarification of its computational mechanism in the brain.Here we successfully reproduced this naturalistic behavior in the laboratory setup. We developed a novel foraging task that requires control of a joystick for the subject to move in the virtual reality natural environment (300 m x 300 m) to harvest prey items under time requirement. We first tested this task in human subjects. They immediately could learn the reward environment within a few training sessions (5 min/session). We analyzed their foraging trajectory and found that the human subjects continuously maximized the reward rate by taking the strategy to choose in a descending order from the items with higher reward rate, which follows the optimal foraging theory. To understand which parameter is more effective, the reward or cost in their choice, we also calculated the intake efficiency of the reward amount and cost separately from their trajectory, and found that both the reward and cost efficiency were well balanced when they maximized the reward rate. We are currently applying the same method and analysis to macaque monkeys.

### [3P-065]

### Visual attention in 9- and 12-month human infants and adults versus macaque and marmoset monkeys

*Richard E Veale[1], Chih-Yang Chen[1], Tadashi Isa[1] ([1]*Kyoto University*)

In daily life, humans move their eyes several times per second. The neural pathways by which information flows from the retina to the ocular muscles are understood in broad terms, but we have yet to elucidate the local computations that determine how visual information is converted into the precise timings and targets of each eye movement. While several excellent models of bottom-up visual attention have been proposed (e.g. Itti et al.'s 1998 saliency map model), these models only explain 20% of the variance in looking targets of human adults and macaque monkeys. Recently (Chen et al., 2021), we showed that looking behavior of another primate species, the common marmoset, has similar properties to human adults and macaques. Even in infant humans, such bottom-up models explain only 20% of variance (Veale, Chen, & Isa, 2021). Here, we address the question of whether the 20% explained by saliency is the same among all species and ages. We directly compare the video-watching behavior of 9-month-old human infants (N=3) and 12-month olds (N=3) against marmosets (N=3), macaques (N=2), and human adults (N=4) under the same conditions. Human infant behavior at 9 months postnatal is similar to marmoset behavior in that 20% of looking behavior variance is explained by salient visual targets, but different subjects tend to look to *different* salient targets. At 12 months the infants appear in a state of change, transitioning to a more adult regime depending on the subject, implying that further investigation at more advanced ages is necessary to identify the start and end of the "transition period" of visual attention. After transition, we expect infant behavior to follow that of human adults and macaques, whose looking is likewise explained 20% by saliency. However, in contrast to the younger infants and marmosets, subjects tend to look at the *same* salient target.

### [3P-066]

### Neural and behavioral correlates of discriminating facial expressions with different skin textures in macaque monkeys

*Kazuko Hayashi[1,2], Narihisa Matsumoto[2], Keiji Matsuda[2], Kenichiro Miura[3], Shigeru Yamane[2], Mark Eldridge[4], Richard Saunders[4], Barry Richmond[4], Yuji Nagai[5], Naohisa Miyakawa[5], Takafumi Minamimoto[5], Masato Okada[6], Kenji Kawano[2], Yasuko Sugase-Miyamoto[2] ([1]*JSPS,* [2]*AIST,* [3]*NCNP,* [4]*NIMH,* [5]*QST,* [6]*University of Tokyo*)

Face perception and recognition are critical for social interaction. In primates, the ability to detect faces quickly is assumed to rely on neural mechanisms in the temporal lobe. We have previously reported that many temporal cortical neurons coded the differences of monkey/human facial surface properties while three monkeys performed a fixation task. In areas TE and TEO, amount of mutual information in the neural responses suggested that the strength of the expression representation was significantly reduced in the style-transferred images, compared to the original. To find behavioral correlates of these neuronal findings, we further investigated effects of the different skin textures on face discrimination learning. Two monkeys were trained to choose one expression of two monkey/human facial images in a saccade task. By analyzing their performance and reaction time, discrimination learning was impaired in monkey expressions of the style-transferred images, but not human expressions. The results are partially consistent with neuronal findings and indicate that facial information processing of the style-transferred images might be different from that of other images.

### [3P-067]

### Neural population dynamics of multidimensional economic variables in mesocortical pathway during risk-return decision making

*Koh Ikuchi[1], Tadashi Isa[1,2,3], Ryo Sasaki[1] ([1]*Department of Neuroscience, School of Medicine, Kyoto University,* [2]*Institute for the Advanced Study of Human Biology (WPI-ASHBi), Kyoto University, Kyoto, Japan,* [3]*Human Brain Research Center, Graduate School of Medicine, Kyoto University, Kyoto, Japan*)

It is essential for one's capability of economic decision-making to flexibly handle the conflict between high-risk high-return (HH) and low-risk low-return (LL) choices. The mesocortical pathway has been reported to be involved in economic decisions, while the precise neural mechanisms remain largely unknown, especially in such multidimensional conditions. In this study, we simultaneously recorded populations of single neurons in macaque vlPFC or VTA, during an HH vs LL choice task. Monkeys performed accurately in selecting higher EVs and showed a clear preference for HH choices. To quantify the neural sensitivity for the parameters, we used a regression analysis and found that many single neurons had mixed-selectivity for EV, HH-LL, and/or choice. Next, to understand the population dynamics in the task-related space, we applied a regression-based dimensionality reduction method. It visualized the clear task-relevant manifold where the peak of choice-related signals follows that of the stimuli-related signal representing EV or HH-LL, which might reflect the internal process of economic decision. Finally, we succeeded in decoding the stimuli and choice information from the neural population. Importantly, the population encoding and decoding analysis consistently showed that both populations had abundant information on EV. In contrast, vlPFC, compared to VTA, had more information on HH-LL. Our findings suggest that the mesocortical neurons flexibly encode the multidimensional economic variables concurrently, with significant area-specificity.

### [3P-068]

### Distinct sex difference in the relationship between personality and resting-state brain functional network in young volunteers

*Taisei Hirata[1], Tomohiro Donishi[1], Masaki Terada[2], Yoshiki Kaneoke[1] ([1]*Dept. of System Neurophysiology, Graduate School of Wakayama Medical University,* [2]*Wakayama-Minami Radiology Clinic*)

Several researches have shown that human personality is associated with resting-state brain functional networks. Its sex difference, however, has yet to be elucidated even though brain functions are affected by sex hormones. We studied the relationship between personality scores and brain regional functional connectivity parameters using the data from 214 young (18-22 years old) volunteers (103 males). All female volunteers were estimated to be in the follicular phase. 3T MRI was used to acquire 15 min BOLD images for each subject. Brain regions including the subcortical nuclei and the cerebellum were divided into 388 regions by the atlas (AICHA and AAL) to determine network parameters using the time course of the mean BOLD signal for each region. We found that the score of the neuroticism in the big five personality traits was negatively related to the degree centrality (DC) of the left intra-parietal sulcus (IPS) in males (FDR corrected $p<0.01$). Score of the harm avoidance in the Temperament and Character Inventory (TCI) was negatively related to the DC of the right intra-occipital sulcus for males and score of the directedness in the TCI was significantly related to the DC of the left IPS and the left anterior insula and negatively related to the right parieto-occipital sulcus. The DC value at any region in females was related to any of the personality score. The results suggest that the neural correlates of the personality traits for females are distinct from those for the males.

### [3P-069]

**Contribution of white matter impairment to cognitive decline with aging**

*Shiho Kunishima[1], Daisuke Kato[1,2], Hiroaki Wake[1,2] ([1]*Department of Anatomy and Molecular Cell Biology, Nagoya University Graduate School of Medicine,* [2]*Division of Multicellular Circuit Dynamics, National Institute for Physiological Sciences, National Institutes of Natural Sciences, Okazaki, Japan*)

### Poster Presentation

### [3P]
### Neurophysiology, Neuronal cell biology
### Motor function

March 16 (Thu.), 12:10 - 14:10, Poster Room

### [3P-070]

### Maturation of forelimb motor representation of motor cortex in postnatal rats

*Yaoko Iwasaki[1], Naomi Oshiro[1], Ikutomo Masako[2], Masatoshi Niwa[1], Yuhki Fujisawa[3], Ken Muramatsu[3] (*[1]Dept. Occupational Therapy, Kyorin University., [2]Dept. Physical Therapy, University of Tokyo Health Sciences., [3]Dept. Physical Therapy, Kyorin University.*)

The purpose of this study was to investigate maturation of forelimb motor cortex in postnatal rats.Fifty-four male Wistar rats were used for analysis (4 weeks old [W]: n = 11, 5W: n = 13, 6 W: n = 10, 7 W: n = 10, 8W: n = 7). We used intracortical micro-stimulation (ICMS) to examine motor representation of forelimbs in the cerebral cortex. Additionally, we recorded motor evoked potentials (MEPs) caused by ICMS from dorsiflexors of the wrist and maximal evoked potential (M-max) by stimulation of radial nerves. Forelimb area was 2.0 ± 0.5 $mm^2$ at 4W, expanded to 4.0 ± 0.89 $mm^2$ at 5W, and did not change thereafter. Similar changes were observed for increased amplitude of MEPs and MEPs/M-max. Additionally, the electrical threshold of ICMS was decreased. During the aforementioned period, the movement evoked by ICMS was altered as dorsiflexions and elbow flexions appeared at 4W, finger flexions at 5W, and finger extensions and shoulder extensions appeared to replace wrist dorsiflexion at 6W and after.These results suggest that dramatic expansion of the motor cortex and strengthening of synaptic connections with the spinal cord occur as rats grow from 4 to 5 weeks of age. We also found that after 5 weeks, the types of forelimb movements represented on the motor cortex increased, indicating that internal maturation occurred.

### [3P-071]

### Identification of gamma motor neuron by expression of transcription factor Err3 and distribution feature of motor neuron at lower cervical region of macaque monkey

*Shun Nakamura[1,2], kikuta satomi[1], moeko kudo[1], kazuhiko seki[1] (*[1]NCNP, [2]University of Texas at Austin, Department of Kinesiology and Health Education*)

Motor neurons of the spinal cord are the neuronal cells that transmit impulses to the skeletal muscles. One of the neurons, γ motor neurons, regulate the gain of muscle stretch by modulating the level of tension in the intrafusal muscle fibers of the muscle spindles. The disruption of γ motor neurons leads to severe motor deficits. Recently reported the technique using a molecular marker that selectively identifies γ neurons in rodents but never examined in non-human primates. To address this issue, we investigate whether the transcription factor Err3 can selectively identify γ motor neurons of a macaque monkey and characterize the distribution feature of motor neurons in the spinal cord. We measured the size of the cell body (CS) and fluorescence intensity (FI) of motor neurons stained with anti-Err3 and anti-ChAT. We found the bimodal distributions of CS and IF. This result suggests that Err3 works as a marker of γ motor neurons in primates, although their separation between α and γ was not as clear as in rodents. By comparing the ratio between motoneurons of Err+ and Err- in the three motoneuron pools potentially innervating finger, elbow, and axial muscles, we found a higher probability of Err+ in the proximal muscles suggesting the predominance of gamma motoneurons. This distal-proximal gradient may reflect the specific role of the gamma system in controlling limb movement.

### [3P-072]

### Improvement in muscle strength parameters of patients with depression following electroconvulsive therapy (ECT)

*Kumar Sarvottam[1], Zoha Deeba Khan[1], Pankaj Surekha[1] (*[1]Institute of Medical Sciences, Banaras Hindu University, Varanasi, Uttar Pradesh, India*)

Introduction: Patients with depression characteristically have reduced physical activity. The physical activity improves cognition and has been shown to have inverse correlation with severity of depression. Electroconvulsive therapy (ECT) is an important treatment modality for depression and there is associated improvement in cognition following ECT in patients with depression. Muscle strength is an important prerequisite for physical activity and an independent predictor of cognition. So, in this study we have explored the effect of ECT on muscle strength parameters in patients with depression. Materials and methods: 50 patients with depression were recruited in the study from psychiatry in-patient department after obtaining informed written consent. Patients were not suffering from any other neuropsychiatric or muscular diseases. A brief history of present illness was taken and for assessment of the severity of depression patient health questionnaire ( PHQ-9) was used. Anthropometric measurements were performed in all patients prior to study. 20 patients among them undergone ECT along with pharmacotherapy (ECT group) while 30 patients received pharmacotherapy (PT). Isometric hand grip strength maximum force (HGMF) and Endurance time (HGET) were recorded pre and post intervention by digital hand dynamometer. Maximum force of plantar flexion (MFPF) and endurance time (PFET) were also recorded in similar timeline using pre-standardized force transducers. Independent sample T test and Mann Whitney U test were used for comparing ECT group and PT, while Wilcoxan sign-rank test was used for pre-post comparison. SPSS 22 was used for all statistical analysis. Study was approved by institute ethics committee and ethical standards of Helsinki Declaration were followed while taking all measurements. Results: HGMF was significantly higher in ECT group (z=-3.29, p= 0.001) as well as in PT (z= -2.82, p= 0.005) post treatment. MFPF was also similarly higher in ECT group (z= -3.92, p= 0.000) as well as in PT (z= -2.654, p= 0.008). HGET and PFET were significantly higher post treatment only in ECT group (t= -2.63, p= 0.017 and t=-2.02, p=0.044 respectively). Change in HGET was significantly higher in ECT group in comparison to PT (z= -2.546, p= 0.011). Change in MPPF was significantly higher in ECT group in comparison to PT (z= -3.091, p= 0.002). There were no statistical difference in change in HGMF and PFET post treatment in ECT group and PT. Conclusion: Improvement in muscle strength parameters in ECT group may be indicative of reduction in severity of depression as compared to PT. Since endurance time is more related with motivational component of physical activity, it may be implied that ECT may be an important modality affecting motivational component of physical activity than pharmacotherapy alone.

### [3P-073]

### Dynamic activity model of movement disorders: A unified view of their pathophysiology

*Atsushi Nambu[1,2], Satomi Chiken[1,2], Hiromi Sano[1,2,3], Nobuhiko Hatanaka[1,2] (*[1]Division of System Neurophysiology, National Institute for Physiological Sciences, [2]Department of Physiological Sciences, SOKENDAI, [3]International Center for Brain Science, Fujita Health University*)

Malfunction of the basal ganglia causes movement disorders such as Parkinson's disease and dystonia, but their pathophysiology is still under debate. Here, we propose a "dynamic activity model" to explain their pathophysiology in a unified manner. In the normal state of monkeys, rodents, and probably humans, cortical stimulation induces a triphasic response consisting of early excitation, inhibition, and late excitation in the output nuclei of the basal ganglia. Cortically induced inhibition mediated by the *direct* pathway disinhibs thalamocortical activity and releases movements, whereas early and late excitation mediated by the *hyperdirect* and *indirect* pathways resets cortical activity and stops movements, respectively. In animal models of movement disorders, the triphasic response patterns are systematically altered, which could well explain their pathophysiology. In Parkinson's disease, inhibition is reduced and cannot release movements, causing akinesia. Conversely, in dystonia, inhibition is enhanced and releases movements unintentionally, resulting in involuntary movements. Moreover, blocking the subthalamic nucleus in Parkinson's disease restores inhibition and recovers movements, which could explain the beneficial mechanism of stereotactic surgery.

### [3P-074]

### Assessment of concentration status by SMR during dart throwing

*Kairi Sato[1], Shingo Murakami[1] (*[1]Chuo Univ.*)

The (sensorimotor rhythm) SMR wave has been shown to be related to the concentrated mental status required for sports and games, and appears just before an action is performed during sports and games. However, the relationship between SMR wave and concentration during sports and games has not been shown. In the present study, we adopted a dart game and quantitatively evaluated the concentration state during dart throwing by correlating the dart scores with the SMR waves. In this experiment, total scores of 90 throws in three days by male subjects (21-25 years old) with limited darts experience were evaluated while EEG signals were measured. The subjects were instructed to throw darts after five seconds countdown. The EEG signals during the countdown were measured with channels Cz, C3, and C4 in the 10-20 method and analyzed. The EEG signals were filtered at 12-15 Hz and their peak values were calculated based on the averaged scores during 10 throws. The dart scores were evaluated with 15 levels of scores, with the closer to the center point of the dart, the higher the score. EEG signals were analyzed for five seconds before throwing, which shows an increase in SMR power 1.5 seconds before throwing the darts was found. This result suggests that the SMR wave-related concentration increased just before throwing the darts, and therefore, SMR waves may be used to evaluate the state of concentration in sports in which players make actions from a state of rest.

### [3P-075]

### Neural dynamics of motor cortex for flexible feedback motor control

Kuroki Yosuke[2], Masui Ryota[2], *Tomohiko Takei[1,3] ([1]*Brain Science Institute, Tamagawa University,* [2]*Faculty of Medicine, Kyoto University,* [3]*Graduate School of Medicine, Kyoto Univeristy*)

A hallmark of our motor system is how flexibly it can switch the association between sensory input and motor response. In the motor control theories, this association is called the "control policy" and is assumed to be prepared according to the behavioural context prior to the initiation of action, but the neural mechanism for the preparation of control policy in the cortical areas is still unclear. In this study, we constructed a recurrent neural network model that reproduces the flexible motor response of animals to mechanical perturbations applied to the limb, and analyzed the dynamics of the neural state. We found that the neural state of the trained network moved along the preparation and response dimensions that were orthogonally arranged, suggesting that the preparation of the control policy was achieved in the null space of motor output. We, then, recorded electrocorticograms (ECoGs) from the fronto-parietal cortical areas of macaques performing a flexible motor response task to a mechanical disturbance of the limb. The dimensional reduction analyses showed that the cortical activity spanned orthogonal dimensions during the preparatory and response phases. Furthermore, we identified different areal and different frequency signals underlying the preparation and response dimensions. These results suggest that the control policy is prepared in the neural subspace that is separated from motor execution to achieve flexible motor control.

### [3P-076]

### Changes in temporal and spatial parameters of the postural control for forelimb reaching following microinjection of muscimol into the posterior parietal cortex in the cat.

*Mirai Takahashi[1], Toshi Nakajima[2], Shusei Fukuyama[1], Tomohiro Noguchi[1], Ryosuke Chiba[1], Kaoru Takakusaki[1] ([1]*Department of physiology, Division of neuroscience, Asahikawa Medical University,* [2]*Department of Integrative Neuroscience, Faculty of Medicine, Toyama University*)

Appropriate postural control precedes the purposeful action so that the subject maintains its equilibrium throughout the movements. We have been investigating preceding postural control during forelimb reaching movement in cats and revealed that the posture at the end of the reaching was provided before the onset of lifting the forelimb to be reached. In addition, the time required for forelimb reaching was optimized to the preceding postural control. Because the posterior parietal cortex (PPC) is considered to integrates the spatial and temporal information necessary for the target reaching task, we hypothesized that the PPC plays an important role in the preceding postural control. Then, attempts were made to elucidate whether and how temporal and spatial parameters of preceding postural control were altered by inactivation of the PPC by microinjection of muscimol, a GABA-A receptor agonist. Two adult female cats weighing from 2.4 to 3.0 kg, which were well trained to perform forelimb reaching task, were employed. Under general anesthesia, a craniotomy was performed to attach a chamber on the right frontal cranium. After recover from the surgery, locations of the PPC and sensori-motor cortices were identified by recording cortical neuronal activities. Then, muscimol was microinjected (5 µg/µl, 10-20 µg/site) into the target site. After the microinjection, each cat performed the forelimb reaching task on force transducers. We measured the time required for preceding postural control and reaching movement, together with center of vertical pressure (CVP) as an index of postural change. Inactivation of the PPC attenuated the body sway during reaching movement, and the cat adopted a more stable postural control strategy. Shorting of the CVP shift or increasing movement time was traded off to maintain equilibrium during the preceding postural control. This result suggests that the PPC has a role for spacio-temporal coordination of postural control. Here we present the result in detail, leading to this conclusion.

### [3P-077]

### Detection of shared muscle activity patterns during cyclic and discrete lower limb movements

*Shinya Suzuki[1], Susumu Yoshida[1] ([1]*Health Sciences University of Hokkaido*)

It remains controversial whether shared neural mechanisms contribute to the control of cyclic (e.g., walking) and discrete (e.g., stepping) limb movements. To address this question, the present study examined the relationship among the basic components of muscle activity during walking and stepping movements. Surface electromyograms (EMGs) were recorded from 16 lower limb and trunk muscles in healthy human participants while they performed two visually guided motor tasks: walking on a treadmill; and one-leg stepping onto a visual target. Using a non-negative matrix factorization algorithm, time series signals of the full-wave rectified and low-pass filtered EMG of the 16 muscles could be decomposed to approximately 5 temporal and spatial components in both tasks. The accuracy of reconstruction of muscle activity using this model was > 90% in all participants. Coefficients of each component demonstrated a significant positive linear correlation between the tasks. These findings suggest that spatiotemporal components of lower limb muscle activity patterns during walking and stepping are analogous.

### [3P-078]

### The effect of the forced limb using and stress for recovery of the motor paralysis in ischemic model rat by photochemically induced thrombosis (PIT)

*Chihiro Sato[1], Kazuki Akahira[2], Shuhei Koeda[1], Misaki Mikami[1], Kai Hatakenaka[1,2], Tomohito Nunomura[1,2], Kana Umamichi[1], Hiroyuki Miura[1], Junko Yamada[1] ([1]*Department of Comprehensive Rehabil Rehabilitation Science, Hirosaki University Graduate School of Health,* [2]*Hirosaki Stroke and Rehabilitation Center*)

The forced impaired limb use (FLU) and motor skills training (MST) like the pellet reaching task promotes recovery of upper limb function and neural plasticity after stroke. However, it is unclear which training is better method, because the benefits of exercise are affected by stress response in recovery. We demonstrated the effect of FLU or MST for functional recovery using Photochemically Induced Thrombosis (PIT) model We used the PIT model rats have the infarct in the area of upper limb. The rats were assigned into the three groups; no exercise (non-Ex group, n=10), trained with only FLU (FLU group, n=8), pellet reaching task and FLU (MST group, n=8). The functional assessments were the wire hang test, forelimb placing test, beam walk test (BWT) and the pellet reach test. To investigate the effect of stress, the corticosterone in serum was assessed after all of experiments. The scores for gross motor function (wire hang, forelimb placing test) were higher in the trained groups than the non-Ex group. Notably, the score of the fine function (pellet reach) was recovered in MST group than the other groups ($P<0.05$). In the BWT as the assessment for hindlimb function, there were no difference among all groups. The concentration of corticosterone was higher in the FLU group than the non-Ex group. These data suggested that the use of upper limb promotes the gross motor function, but not the fine function because of the effect by highly stress.

### [3P-079]

### Verbal encouragement induces better pegboard performance and increased hemodynamic responses in the prefrontal cortex

*Susumu Urakawa[1], Hiroki Ogawa[1], Masato Tsuyuhara[1], Le Trung Duc[1], Hisao Nishijo[2], Masahito Mihara[3], Naoto Fujita[1] ([1]*Dept of Musculoskeletal Functional Research and Regeneration, Grad Sch of Biomedical and Health Sci, Hiroshima University,* [2]*Dept of System Emotional Sci, Grad Sch of Medicine and Pharmaceutical Sci, University of Toyama,* [3]*Dept of Neurology, Kawasaki Medical School*)

To improve the motor function of patients who suffer from central nervous system disorder, therapist verbally instructs and encourages their performance as an exercise therapy. However, neuronal mechanisms underly such an encouragement to improve their motor performance is not clarified. In the present study, we evaluated the motor performance of pegboard task with or without the verbal encouragement, and related hemodynamic responses of cerebral cortex using the functional near-infrared spectroscopy. Healthy subjects were divided into two groups: a group presented with encouraging auditory stimuli (ENC) and a group presented with nonsensical auditory stimuli (NON). Each group performed eight trials of pegboard tasks divided into two stages: the first four trials as the control stage without auditory stimuli and the last four trials as the auditory stimulation stage. As the results, although both ENC and NON group showed improved performance at later stage, ENC showed better performance by encouragement. The ENC group substantially increased oxyhemoglobin changes of in the prefrontal regions. Meanwhile, the similar activity was not observed in the NON group. These results suggest that encouragement as an intervention to improve motor performance induces neuronal activation of prefrontal regions.

### [3P-080]

### Subdural spinal electrical stimulation for muscle activations of upper limb in an incomplete tetraparesis: A case report in a patient with spinal tumor

*Kenji Kato[1], Kyousuke Kamada[2], Yukio Nishimura[3] ([1]*National Center for Geriatrics and Gerontology,* [2]*Megumino Hospital,* [3]*Tokyo Metropolitan Institute of Medical Science*)

Spinal electrical stimulation is a promising approach for restoring the motor functions of paralyzed limbs following neurological damage to descending pathways. The present study examined the muscle responses upper limb evoked by subdural spinal electrical stimulation of the cervical cord in a patient with tetraparesis. We temporarily installed a platinum subdural electrode array over the dorsal-lateral aspect of the cervical enlargement locating caudal to the vicinity of epidural tumor. Electrical stimulation was delivered at four sites under anesthesia. The subdural electrical spinal stimulation over the cervical enlargement (C5–C7), which was located caudal to the vicinity of epidural tumor, could activate multiple muscles of the upper limb in a tetraparesis. At smaller current of 1 mA, stimulation through the rostral electrode activated muscle responses in proximal muscles, stimulation through the caudal electrode activated distal muscles. Increasing the stimulus current associated number of the activated muscles and amplitude of muscle responses. Once muscles are recruited, while the waveform of muscle response sin proximal muscles were similar among different intensity, that in distal muscles changed as current increased. These results indicate that spinal electrical stimulation is a promising neuroprosthetic technology to regain motor function in upper-limb after neural damage to the descending pathways.

### [3P-081]

### Reticulospinal control of synergy in hindlimb muscles and its modulation by cholinergic -serotonergic interaction in the pontine reticular formation

*Kaoru Karl Takakusaki[1] ([1]*Asahikawa Medical University*)

Postural muscle synergy is altered depending on behavioral states such as sleep-wake cycles. Moreover, disturbance in postural muscle synergy is observed in elderly persons and patients with degenerative diseases such as Parkinson's disease and Alzheimer's disease. We hypothesized that homeostatic changes in neurotransmitters might regulate the state-dependent postural muscle synergy, whereas deficiency in neurotransmitter actions may elicit pathophysiological postural synergy. Because the pontomedullary reticular formation (PMRF), which receives influences from various neurotransmitter systems in the brainstem, has diverse projections to spinal segments of the whole neuroaxis via the reticulospinal tract (RST) to organize contractions of the neck-trunk-limb muscles to control postural muscle synergy. The purpose of this study was to understand the mechanisms of how convergent inputs of cholinergic projection from the pedunculopontine tegmental nucleus (PPN) and serotonergic projection from the raphe nuclei (RN) to the pontine reticular formation (PRF) altered the reticulospinal evoked postural muscle synergy. For this purpose, we used decerebrate cat preparations (n=12) in which forebrain structures were removed at the rostral midbrain level. Repetitive electrical microstimulation (up to 40 μA with 50 Hz trains) was applied to the whole regions in the PMRF. Contraction of each hindlimb muscle was substituted by the amplitude of monosynaptic Ia reflex recorded in the ventral loots so that postural muscle synergies in knee and ankle joints were examined. Then, solutions containing carbachol, a muscarinic cholinomimetic agent resistant to choline-esterases, or serotonin were microinjected (1.6 μg /0.1 μl ~ 4.0 μg/0.1~0.25 μl) into the rostral PRF. Hypotonic or atonic postural synergy in the knee and ankle joints was evoked by stimuli applied to the PPN and the dorsomedial part of the PMRF. In contrast, co-contraction muscle synergy was preferentially induced by stimuli delivered to the PMRF, including the RN. On the other hand, the lateral part of the PMRF elicited synergies with either flexion, extension, or a mixture of both. We also observed that pontine carbachol injection enlarged areas that induced hypotonic-atonic synergy. In contrast, pontine serotonin injection increased the site from which synergies with a mixture of co-contraction, extension, and flexion were evoked. These results suggest that the functional topography concerning the postural muscle synergy exists in the PMRF. The functional synergy map can be reorganized by the interaction between the cholinergic and serotonin systems in the PRF.

### [3P-082]

### Role of the lateral cerebellum in the visuo-motor association learning

*Yusuke Akiyama[1], Hiroshi Yamada[1], Masayuki Matsumoto[1], Jun Kunimatsu[1]
([1]*University of Tsukuba*)

Recent imaging studies reported an increase in activity in the cerebellum during visuo-motor association learning, although it remains unclear what information is encoded by the neuronal activity. To examine it, we recorded the single-neuron activity in the cerebellar dentate nucleus (DN), an output structure of the cerebellum, while monkeys performed a visuo-motor association task. In this task, one of two fractal objects (A or B) was presented on a monitor. After a delay period, the fractal object was disappeared, then two identical saccade target points were presented on left and right hemifields. If the monkeys made a saccade to one of the directions that associated with the fractal object (e.g. A-right, B-left), they got a liquid reward. We recorded the activity of DN neurons in two conditions: i) the learning condition in which novel fractal objects were used and ii) the over-trained condition in which well-learned fractal objects were used. We first found that many neurons showed an increase in activity during a delay-period, and it was greater in the learning condition than in the over-trained condition. The delay-period activity was modulated by saccade directions in the learning condition. The direction selectivity was gradually increased according to the progress of learning. Our results suggest that the delay-period activity of DN neurons is involved in visuo-motor association learning, but not in retention or retrieval.

### [3P-083]

### Involvement of the cerebello-rubral regulatory system in functional recovery by intensive use of the paralyzed side after cerebral hemorrhage

*Shinya Ueno[1], Takeshi Shimizu[1], Kenta Kobayashi[2], Shiori Tominaga[1], Naoki Tajiri[1], Hideki Hhida[1] ([1]*Nagoya City University, Graduate school of Medical Sciences, Neurophysiology and Brain Science,* [2]*National Institute for Physiological Sciences, Section of Viral Vector Development*)

Intensive forced limb use (FLU) after intracerebral hemorrhage (ICH) improves paralyzed limb motor function, and the cortico-rubral pathway is involved in this functional recovery (J Neurosci 36:455, 2016). However, the contributions in the motor regulatory system associated with this recovery remain unclear. We focused on the pathway from the lateral cerebellar nucleus (LCN) to the parvocellular red nucleus (RNp) as an output system from the cerebellum and investigated the role of this regulatory system by neural silencing.The cerebello-rubral pathway was blocked by infecting the LCN with AAV-DJ-EF1a-DIO-hM4D(Gi)-mCherry and the RNp with FuG-E-MSCV-Cre, respectively. The unimpaired upper limb was cast immobilized 1-8 days after the ICH, and paralyzed upper limb was subjected to intensive use. Upper limb motor function was evaluated by the skilled reach test until 28 days after the ICH, and the detailed function was analyzed using deep learning technique.The success rate of the skilled reach test was higher in the group with FLU than without FLU, and the success rate was significantly lower when the pathway blocked with clozapine-N-oxide (CNO). Furthermore, the effect of this blocking was confirmed electrophysiologically. In addition, there was no effect in the animals without ICH when CNO was treated.The motor regulatory circuits in the cerebello-rubral pathway were found to be involved in the improvement of impaired motor function by FLU.

### [3P-084]

### Cerebellar Purkinje cells encode behavioral information of multiplex modalities with their complex spikes and form functional modules

*Koji Ikezoe[1], Naoki Hidaka[1], Satoshi Manita[1], Masayoshi Murakami[1], Shinichiro Tsutsumi[3], Yoshikazu Isomura[4], Masanobu Kano[2], Kazuo Kitamura[1] ([1]*Faculty of Medicine, University of Yamanashi,* [2]*Graduate School of Medicine, The University of Tokyo,* [3]*RIKEN Center for Brain Science,* [4]*Graduate School of Medical and Dental Sciences, Tokyo Medical and Dental University*)

Cerebellar climbing fiber (CF) inputs to Purkinje cells (PCs) generate complex spikes in PCs. CFs convey sensorimotor information and their errors, which are used for motor control and learning. Moreover, they encode reward-related information. Although CFs convey such multiple signals, it is still unclear whether each CF conveys the information of single or multiple modalities and how the CFs conveying different information are distributed over the cerebellar cortex. We performed two-photon calcium imaging from PCs in mice engaged in a voluntary forelimb lever-pull task. We found that CF responses, inferred from transient fluorescent changes, in 93% of PCs were partially explained by the combination of multiple behavioral variables, such as lever movement, licking, and reward delivery. Neighboring PCs had similar CF response properties, formed functional clusters, and shared noise fluctuations of responses. The results suggest that individual CFs convey behavioral information on multiplex variables and are spatially organized into the functional modules of the cerebellar cortex. Authors have no COI to disclose in relation to the presentation.

### [3P-085]

### The motor and parietal cortices contribute to the online modification of the forthcoming reaching movement based on the memory of the error in the previous trial

*Masato Inoue[1], Shigeru Kitazawa[1,2,3] ([1]*Center for Information and Neural Networks (CiNet), Advanced ICT research institute, National Institute of Information and Communications Technology,* [2]*Graduate School of Frontier Bioscience, Osaka University,* [3]*Graduate School of Medicine, Osaka University*)

We have recently shown that motor cortices (primary motor cortex (M1) and premotor cortex (PM)) and parietal areas (area 5 and 7) encode information on visual end-point errors in reaching (apparent error, AE) toward a target when the vision is given after each movement (Inoue et al. 2016, Inoue & Kitazawa 2018). In these studies, we displaced the visual field in a random direction using motor driven wedge prisms to dissociate the AE with motor errors in reaching (ME), that is, errors between the hand and the perceived target location. We further found that the ME was modulated every trial by a small amount in the direction opposite to the AE in the previous trial. In the present study, we examined whether the motor and parietal cortices represented AE in the previous trial as well as the ME in the present trial. Neurons in M1, PM, and area 5 encoded information on the AE of the previous reaching movement as well as information on the ME of the forthcoming reaching movement "before" the target presentation. These results suggest that M1, PM, and area 5 contributes to the online modification of the forthcoming reaching movement based on the memory of the error in the previous trial.

### [3P-086]

### Motor evaluation of cuprizone-induced demyelinating mice with three-dimensional kinematic analysis

*Tatsuro Kumada[1], Saho Morishita[1] ([1]*Tokoha Univ.*)

Chronic demyelinating diseases caused by loss of oligodendrocytes, such as multiple sclerosis, can cause changes in conduction velocity and firing frequency in the central nervous system, resulting in various neurological symptoms including motor function. To study the roles of neurorehabilitation with the rodent model of these diseases, a quantitative assessment of motor behavior is required to detect its deficit even during the recovery stage. Though a battery of behavioral tests can assess multiple aspects of animal behavior, it is not yet enough to quantify the motor behavior in detail, especially in rodent models. To overcome this issue, we have evaluated the motor ability of the mice with cuprizone-induced demyelination in a Spatio-temporal manner through three-dimensional (3D) kinetic analysis methods. We fed 10-week-old C57BL6 mice a 0.2 % cuprizone diet and evaluated the kinematic movement of mice on a treadmill over time. Approximately 5-7 weeks after cuprizone treatment, changes in walking/running behavior were observed. We would like to discuss these motor deficits in the meeting.

### [3P-087]

### Kinematics and EMG activity related to postural transformation during gait in Japanese macaques

*Takashi Suzuki[1], Kei Mochizuki[1], Morita Kazunori[1], Yoshiro Suzuki[1], Masahiko Inase[2], Katsumi Nakajima[1] ([1]*Iwate Medical University,* [2]*Kindai university*)

To investigate the CNS mechanism that controls posture during gait, we trained monkeys to transform trunk posture from horizontal to vertical during locomotion. We compared kinematics and EMG activity among periods of quadrupedal (QP) gait, postural transformation (PT) and bipedal (BP) gait. Postural transformation was accomplished within 1-2 step cycles. Step cycle frequency increased for PT and decreased for BP gait. During QP gait, the hip and head positions fluctuated cyclically and medio-laterally in an out-of-phase manner. The head and hip shifted to the opposite side during stance of the fore and hind limbs, respectively, forming a diagonal pair. For PT, they became to shift together to the side opposite to the supporting hindlimb. Bilateral back muscles during QP gait showed co-activation around touchdown of each hindlimb. For PT, the co-activation was enhanced and alternate burst activity additionally emerged in the left and right EMGs. Patterns of trunk sway and EMG activity for PT were preserved for Bp gait. The results suggest that, for postural transformation during gait, brainstem-spinal cord circuits in macaques accommodate descending commands and adjust stepping rhythm and patterns of inter-limb and trunk-limb coordination.

### Poster Presentation

[3P]

**Neurophysiology, Neuronal cell biology**

**Sensory function, Sensory organ**

March 16 (Thu.), 12:10 - 14:10, Poster Room

### [3P-088]

**Effect of Vasopressin V1a receptor activation on granule cell activities at the synapse in the mouse accessory olfactory bulb**

*Mutsuo Taniguchi[1], Yoshihiro Murata[1], Masahiro Yamaguchi[1], Hideto Kaba[1]
([1]*Department of Physiology, Kochi Medical School, Kochi University*)

Central vasopressin (AVP) facilitates social recognition and modulates many complex social behaviors in mammals. By measuring the reciprocal synaptic currents (IPSCs) from mitral cells (MC) in the accessory olfactory bulb (AOB), the first relay in the vomeronasal system, we have demonstrated that AVP significantly reduced the IPSCs via V1a receptors. The reciprocal transmission, however, contains both glutamatergic transmission from MCs to granule cells (GCs) and GABAergic one from GCs to MCs. Thus, it is unclear whether AVP acts on the excitatory and/or the inhibitory transmissions.
In the present study, we have given attention to the effect of V1a receptor activation on presynaptic properties in the GABAergic transmission (that is, granule cell activities). AOB slices were prepared from 23- to 35-day-old Balb/c mice. Using the whole-cell voltage clamps, the current response of GCs or MCs was recorded in the presence of antagonists for glutamatergic transmission, CNQX and AP5. Recording from GCs, an extracellular application of AVP significantly diminished the $Ca^{2+}$ currents. Analysis of its I-V relationship suggests that reduction of the GABAergic transmission by AVP involves the inhibition of high-voltage-activated $Ca^{2+}$ channels on GCs.

### [3P-089]

**Olfactory marker protein (OMP) contains a leucine-rich domain in the Ω-loop important for nuclear export**

*Noriyuki Nakashima[1], Akiko Nakashima[1], Kie Nakashima[2], Makoto Takano[1] ([1]*Dept. Physiol., Kurume Univ. Sch. Med.*, [2]*Dept. Physiol., Facult. Med., Kobe Univ.*)

Olfactory receptor neurons (ORNs) are located in the olfactory epithelium and sense environmental chemicals for olfaction. Odourant binding to the odourant receptor in the cilia of ORNs induces intracellular cAMP signalling, which in turn opens cAMP-gated channels. ORNs are unique in their ability to renew throughout life. cAMP is crucial in both phasic olfaction and the maintenance of anatomical neural projections. Olfactory marker protein (OMP), exclusively expressed in cytoplasm of ORNs, regulates cAMP kinetics. Therefore, it is important that OMP is retained in the cytosol to interact with cAMP. Although OMP, at 19 kDa, is small enough to passively diffuse between the nucleus and cytoplasm, OMP is only sparsely detected in the nuclear regions of ORNs. However, the mechanisms by which OMP is retained in the cytosol remain unclear. We hypothesized that OMP might contain nuclear export signals (NESs). In this study, we investigated possible nuclear transport signals in the OMP primary sequence by using several bioinformatics methods and evaluated whether the sequence could direct OMP to either the cytosol or nucleus.

### [3P-090]

**Two modes of 5-HT release from gut enterochromaffin cells**

*Kouki Touhara[1], Nathan Rossen[1], Fei Deng[2], Yulong Li[2], David Julius[1] ([1]*University of California, San Francisco*, [2]*Peking University*)

Enterochromaffin (EC) cells are sensory gut epithelial cells that release serotonin (5-HT) in response to chemical and mechanical stimuli. EC-derived 5-HT acts as a paracrine substance to stimulate mucosal sensory neurons regulating local gastrointestinal (GI) functions and the gut-brain axis. EC-derived 5-HT also acts as a hormone to supply 5-HT to platelets and other distant organs through intestinal capillaries. The critical role of gut 5-HT signaling is evident from severe GI symptoms resulting from carcinoid tumors. Despite its physiological importance, it is unknown how EC cells activate spatially and molecularly diverse targets inside and outside the gut. Using genetically encoded fluorescent 5-HT sensors, we found that EC cells exhibit tonic activity that continuously supplies nanomolar of 5-HT to the mucosa. Whereas when EC cells are stimulated by irritants, micromolar of 5-HT is released to activate 5-HT3 receptors expressed in mucosal sensory neurons. Our study demonstrates that EC cells exhibit two modes of 5-HT release to regulate different targets in the intestinal mucosa.

### [3P-091]

**Elucidation of the dynamic function for the transmembrane domain of the sweet taste receptor subunit, TAS1R3**

*Keisuke Sanematsu[1,2,3], Masato Yamamoto[1], Yuko Kawabata[1], Yu Watanabe[1], Shusuke Iwata[1,3], Shingo Takai[1], Noriatsu Shigemura[1,3] ([1]*Section of Oral Neuroscience, Graduate School of Dental Science, Kyushu University*, [2]*Oral Health/Brain Health/Total Health Research Center, Graduate School of Dental Science, Kyushu University*, [3]*Research and Development Center for Five-Sense Devices, Kyushu University*)

The sweet taste is mediated by the taste G-protein-coupled receptors (GPCR), TAS1R2 + TAS1R3. Although binding sites for sweet compounds or modulators were determined, the dynamic mechanisms of receptor activation or inactivation remain unclear. Here, we used molecular dynamics simulations and functional assay to elucidate the function of the transmembrane domain of TAS1R3, an interaction site for G-protein. The molecular dynamics simulations predicted dynamic allostery induced by artificial sweeteners and sweet inhibitors for the transmembrane domain of TAS1R3. Our simulations also reproduced species-specific sensitivity of the sweet taste receptor to these compounds. Upon receptor activation, the allostery induced by the artificial sweetener destabilized the intracellular region of the TAS1R3, an interface of the Gα subunit, along with an ionic lock opening. Our predictions were confirmed by the functional assays of the TAS1R3 mutations. This study provides important insights not only into the functions of the sweet taste receptor but also into predicting dynamic activation for other G-protein-coupled receptors.

### [3P-092]

**Identification and intersectional optogenetic control of sodium cells in fungiform taste buds**

*Kengo Nomura[1], Hayatsu Norihito[2], Shogo Soma[1], Yasushi Okazaki[2], Akiyuki Taruno[1,3] ([1]*Dept. of Mol Cell Physiolology, Kyoto Pref Univ of Medicine, Kyoto, Japan*, [2]*Lab for Comprehensive Genomic Analysis, RIKEN Center for Integrative Medical Sciences, Yokohama, Japan*, [3]*JST CREST*)

Understanding salt taste is crucial to fight health problems linked to sodium overconsumption. However, we know little about the cellular and neural mechanisms involved. The epithelial sodium channel (ENaC) is a putative taste sensor for sodium. Indeed, we previously reported that a subset of taste cells expressing the α-subunit of ENaC mediate the taste of sodium. Nevertheless, functional ENaC is typically composed of three subunits, i.e. α, β, and γ, and their co-expression is controversial; thus, the sodium sensor mechanisms are unresolved. Using single-cell transcriptome analysis of murine fungiform taste buds, we identified a distinct taste cell cluster expressing all ENaC subunits. Furthermore, we show that intersectionally-targeted optogenetic activation of these cells, using the ENaCα and β promoters, specifically stimulates sodium-responding neurons in the brain and mimics behaviors to sodium taste. These data provide the precise identification of sodium taste cells. In addition, the transcriptomic data and optogenetic mice offer a basis and tool for studying the peripheral and central processing of sodium taste.

### [3P-093]

### Temporal profiles of neuronal responses to repeated tone stimuli in the mouse primary auditory cortex

*Wen-Jie Song[1], Ryohei Tomioka[1], Bo Zhou[1] ([1]*Graduate School of Medical Sciences, Kumamoto University*)

How the auditory system processes temporal information of sound has been investigated extensively using repeated stimuli. Recent studies on how the response of neurons in the primary auditory cortex (A1) changes with the progression of stimulus repetition, have reported response temporal profiles of two categories: “adaptation”, i.e., gradual decrease, and “facilitation”, i.e., gradual increase. To explore the existence of profiles of other categories and to examine the tone-frequency-dependence of the profile category in single neurons, here we studied the response of mouse A1 neurons to five tone-trains; each train comprised 10 identical tone pips, with 0.5-sec inter-tone-intervals, and the five trains differed only in tone frequency. The response to each tone in a train was evaluated using the peak of the ON response, and how the peak response changed with the tone number in the train, i.e., the response temporal profile, was examined. We confirmed the existence of profiles of both “adaptation” and “facilitation” categories; “adaptation” could be further subcategorized into “slow adaptation” and “fast adaptation” profiles, with the latter being encountered more frequently. Moreover, two new categories of non-monotonic profiles were identified: an “adaptation with recovery” profile and a “facilitation followed by adaptation” profile. Examination of single neurons with trains of different tone frequencies revealed that some A1 neurons exhibited profiles of the same category to tone trains of different tone frequencies, whereas others exhibited profiles of different categories, depending on the tone frequency. These results demonstrate the variety in the response temporal profiles of mouse A1 neurons, which may benefit the encoding of individual tones in a train.

### [3P-094]

### Bottom-up/top-down neural circuits modulating whisking-related activities in the barrel cortex.

*Masahiro kawatani[1,2], Takayuki Yamashita[1] ([1]*Fujita Health University,* [2]*Nagoya University*)

In the primary somatosensory barrel cortex of the whisker region (wS1), spontaneous movements of whiskers (whisking) influence neuronal activities and modulate sensory perception (Poulet and Petersen, 2008, PMID: 18633351). However, neural circuit mechanisms of such motor-related wS1 modulation are not fully understood. Here, we performed 32-Ch silicone probe recordings from wS1 in awake behaving mice while inhibiting synaptic inputs sent from specific brain regions using optogenetics. We introduced the expression of eOPN3, an inhibitory GPCR opsin, in the neurons of the whisker thalamus (Thal), the primary whisker-motor cortex(wM1), or the secondary whisker somatosensory cortex (wS2) and photo-inhibited their axonal terminals in wS1 during spontaneous whisking behaviors. Spontaneous whisking induced an increase or a decrease in the spike rate of a fraction of wS1 neurons (increase: 20±3 %, decrease: 42±10 %, n = 209 units, from 6 mice). Photo-inhibition of wM1→wS1 inputs did not affect such whisking-related spike rate changes in wS1 (n = 2 mice). In contrast, photo-inhibition of wS2→wS1 inputs abolished the whisking-related changes in wS1 (n = 2 mice). Photo-inhibition of Thal→wS1 inputs increased overall spike rates in wS1 during quiet wakefulness and during whisking by inhibiting the reduction of whisking-related spiking (n = 2 mice). The spike rates in wS1 during quiet wakefulness were not affected by photo-inhibition of wM1→wS1 and wS2→wS1 inputs. These results thus suggest the differential contribution of top-down and bottom-up signal inputs to whisking-related activity dynamics in wS1, highlighting wS2, not wM1, critical for whisking-related changes in the wS1 activity.

### [3P-095]

### α1-adrenergic receptor antagonist inhibits c-fos expression at the spinomedullary junction evoked by light stimulation in mild traumatic brain injury rats

*Akimasa Tashiro[1], Yuji Morimoto[1] ([1]*National Defense Medical College*)

Headaches and photophobia are common persistent postconcussive symptoms following mild traumatic brain injury (mTBI). Enhanced central nervous system (CNS) noradrenergic activity in mTBI provides a rationale for using anti-adrenergic medications to ameliorate these symptoms. There is also preliminary clinical evidence specifically supporting the efficacy of blocking the CNS postsynaptic α1-adrenoreceptor (AR) with the α1-AR antagonist, , for treating chronic postconcussive headaches. Previously, we reported that after laser induced shock wave (LISW) irradiated, light-evoked neuronal activity at the trigeminal subnucleus caudalis (Vc) and light aversive behavior enhanced, resulting in photoallodynia. This study used c-fos immunohistochemistry to assess the effects of LISW on activation of trigeminal brain stem neurons produced by bright light stimulation and secondly, whether the adrenergic system contributes to this response. The parietal region of male rats was irradiated with laser-induced shock waves (diameter 3mm, $4J/cm^2$) under barbiturate anesthesia. After 7days of LISW, irradiated, male rats (LISW rats) were exposed to light ($300W/m^2$) delivered from a thermal neutral source for 30 min (30 s ON, 30 s OFF) under barbiturate anesthesia, and allowed to survive for 2h. Light evoked Fos-LI in laminae I–II at the trigeminal subnucleus caudalis/upper cervical cord (Vc/C1-2) junction, the trigeminal interpolaris/caudalis transition (Vi/Vc) was significantly greater in LISW rats than naïve rats. LISW also modified the influence of the α1-AR antagonist (prazosin) on light -evoked Fos-LI. Prazosin (1mg/kg, ip, 10min before light stimulation) reduced the Fos-LI response at the Vc/C1-2 junction and Vi/Vc transition in LISW rats but not naïve rat. These results suggest that LISW plays a significant role in light-evoked nociceptive processing at the caudal trigeminal brainstem complex mediated, in part, through α-1 AR-dependent mechanisms. This study provides evidence for the efficacy of prazosin in photoallodynia during post-traumatic headache.

### [3P-096]

### The properties of slow potential induced by taste stimuli on the human tongue

Satoshi Fukuda[1], Naoyuki Murabe[1], Takashi Yamamoto[2], *Takatoshi Nagai[1,3] ([1]*Teikyo University,* [2]*Kio University,* [3]*Keio University*)

In response to a taste solution the extracellular electrode placed on the human tongue should record a hyperpolarization of the potential of lingual surface (LSP). However, recent papers have shown taste stimuli evoked either depolarization or hyperpolarization of the LSP depending on the taste quality. We examined if the taste transduction in humans is reflected in either depolarization or hyperpolarization of the LSP. By Ag-AgCl electrodes placed on the lingual surface we recorded the LSPs by taste solutions after a rinse with 10 mM NaCl which mimics the electrolyte environment on the surface. Sucrose dissolved in distilled water (sucrose/water) evoked a large depolarizing potential, while sucrose dissolved in 10 mM NaCl (sucrose/Na) evoked a hyperpolarizing potential. The depolarizing potential by sucrose/water was shown to derive from the junction potential generated by reduction in Na concentration from 10 mM to near zero. Sweet taste inhibitor, lactisole (3.75 mM), significantly reduced the magnitude of LSP by sucrose/10 Na. Bitter taste stimulus, 6-n-propylthiouracil (PROP), evoked hyperpolarizing LSPs, which contain a large junction potential generated by PROP.

### [3P-097]

### Role of tachykinin peptides in pruriceptive processing in mice

*Rumi Nakayama-Naono[1], Akihiro Ambo[1], Fumito Naganuma[1], Tadaho Nakamura[1], Toshikazu Nishimori[2], Hideki Funahashi[2] ([1]*Tohoku Med. and Pharm. Univ.,* [2]*Univ. of Miyazaki*)

Tachykinin peptide family is a member of peptides that has a common sequence F-X-G-L-M at the C-terminus of this peptide. This peptide family includes substance P, hemokinin-1 and endokinins, and their roles, in pruriceptive processing remain poorly understood. Recent study on pruriceptive mechanisms, demonstrated that substance P could contribute to pruriceptive processing. On the other hand, there are little reports on the role of other tachykinin peptides, such as hemokinin-1 and endokinins, in pruritus. Therefore, to investigate the effect of these tachykinin peptides on pruriceptive processing, we investigated the expression levels of these peptides in skin tissue and blood of pathological model animals with chronic pruritus. In addition, we also conducted behavioral and anatomical analyses. As the expression of gastrin-releasing peptideis known to increase in the peripheral tissues and spinal cord in animal models with chronic pruritus, the expression level of this peptide was used as a control group for evaluating the effects of these tachykinin peptides. These data suggested that these tachykinin peptides appear to have a crucial role in pruriceptive processing.

### [3P-098]

### Cortical representation for orofacial noxious information in mouse

*Kazunori O'Hashi[1], Masayuki Kobayashi[1] ([1]*Nihon university*)

The somatosensory cortex exhibits a typical somatotopic organization that precisely corresponds to parts of the body. However, clinical observations in the orofacial region question the accuracy of the organization, as patients often fail to detect the tooth pain. Indeed, the detailed topographic organization of nociception in the oral structures remains poorly understood. To clarify the orofacial nociceptive cortical representation, here, the neural activation topography to electrical stimulation of the maxillary and mandibular periodontal ligament (PDL) was compared in anesthetized GCaMP6s mice by two-photon calcium imaging. First, we identified three distinct nociception-processing areas corresponding to the primary, secondary somatosensory and insular cortices (IC). These cortical areas did not show any difference in the pattern of activation responding to both maxillary and mandibular inputs. Next, we focused on the nociceptive representation in IC which is one of the critical cortical foci for nociception. The spatial profiles of maxillomandibular PDL inputs in IC were consistent throughout the depth. These results suggest that the maxillary and mandibular PDLs inputs cannot be discriminated by the somatosensory and insular cortical maps. These characteristics may contribute to explain the difficulty in identifing the source of maxillary and mandibular pain in human patients.

### [3P-099]

### Function of the anterior amygdaloid area during odor information processing.

*Shingo Fukumoto[1], Nana Kitamura[1], Kengo Ehara[1], Yuta Tanisumi[2,3,4], Junya Hirokawa[5], Yoshio Sakurai[5], Takashi Kitsukawa[1], Kazuki Shiotani[1,5], Hiroyuki Manabe[5]
([1]*Laboratory of Brain Network Information, College of Life Sciences, Ritsumeikan University, Shiga, Japan,* [2]*Department of Anatomy and Molecular Cell Biology, Nagoya University Graduate School of Medicine, Nagoya, Japan,* [3]*Division of Multicellular Circuit Dynamics, National Institute for Physiological Sciences, National Institute for Physiological Sciences, Nagoya, Japan,* [4]*Research Fellow of the Japan Society for the Promotion of Science, Tokyo, Japan,* [5]*Laboratory of Neural Information, Graduate School of Brain Science, Doshisha University, Kyoto, Japan*)

The anterior amygdaloid area (AAA) is an amygdaloid region adjacent to the olfactory cortex, which processes the odor information. The AAA has been shown to anatomically connect to the olfactory cortex and the prefrontal cortex, which is considered to be responsible for behavioral switching and cognitive functions. Based on these anatomical characteristics, we hypothesized that the AAA relayed the sensory and behavioral information. In this study, we investigated whether the AAA is involved in the processing of sensory and behavioral information during the odor information process. We conducted an odor discrimination task in mice and recorded neural activities from the AAA of the mice during the task. Many AAA neurons responded to the timing of behavioral switches in the behavioral task, such as the starting point of the task and the timing of the reward. In addition, some AAA neurons had different response characteristics depending on the type of odor stimulus. These results indicate that neurons in the AAA process both behavior-related information and odor information. These results suggest that the AAA plays a relay role in transmitting sensory and behavioral information in the neural circuit of goal-directed behavior.

### Poster Presentation

[3P]
**Molecular physiology, Cell physiology**
**Membrane transport**

March 16 (Thu.), 12:10 - 14:10, Poster Room

### [3P-100]

### Eps15/Pan1p is a master regulator of the late stages of the endocytic pathway

*Junko Toshima[1], Enshoji Mariko[2], Yoshiko Miyano[2], Nao Yoshida[2], Makoto Nagano[2], Jiro Toshima[2] ([1]*Tokyo University of Technology*, [2]*Tokyo University of Science*)

Endocytosis is a multistep process involving the sequential recruitment and action of numerous proteins. It proceeds through four functional modules, and the whole process can be divided into two phases: an early phase, in which sites of endocytosis are formed, and a late phase in which clathrin-coated vesicles are formed and internalized into the cytosol, but how these phases link to each other remains unclear. In this study, we demonstrate that anchoring the yeast Eps15-like protein Pan1p to the peroxisome triggers most of the events, occurring during the late phase, including actin polymerization, at the peroxisome. These observations suggest that Pan1p is a key regulator for initiating, processing, and completing the late phase of endocytosis.

### [3P-101]

### Contextual learning requires phosphorylation at $Ser^{408\text{-}409}$ of $GABA_A$ receptor $\beta_3$ subunit

*Yuya Sakimoto[1], Mitsushima Dai[1] ([1]*yamaguchi university*)

Although contextual learning requires plasticity at both AMPA receptor-mediated excitatory and $GABA_A$ receptor-mediated inhibitory synapses in CA1 neurons, detailed mechanism of the learning-induced plasticity at $GABA_A$ receptor-mediated synapses has been unknown. We recently reported learning-induced increase in postsynaptic number of $GABA_A$ receptor channel (Sakimoto et al, *Cerebral Cortex* 2019), and phosphorylation of intracellular loop ($Ser^{408\text{-}409}$) of $GABA_A$ receptor $\beta_3$ subunit (Sakimoto et al., *FASEB J* 2019). To further examine the causal relationship among the $Ser^{408\text{-}409}$ phosphorylation, synaptic plasticity, and the learning, we used cell permeable HIV tag peptide and synthesized with the novel peptide-based phosphorylation inhibitor targeting sites at $Ser^{408\text{-}409}$ (Tat pep β3-*SS*). Under the freely-moving condition, we bilaterally microinjected the Tat pep β3-*SS* or site-specific mutated control (Tat pep β3-*AA*) into the CA1 region, and used them for the following behavioral test battery: contextual learning (IA task), emotion (open field task), perception (visual task) and social behaviors (pairing test). Tat pep β3-*SS* but not Tat pep β3-*AA* impaired the performance of retrieval test in IA task, while the effect was not observed in any other behavioral tests. *Ex vivo* whole cell patch clamp analysis further revealed that unilateral Tat pep β3-*SS* microinjection clearly blocked the learning-induced increase in the postsynaptic $GABA_A$ receptor-mediated $Cl^-$ current in CA1 pyramidal neurons. These results suggest a causal relationship among the $Ser^{408\text{-}409}$ phosphorylation, $GABA_A$ receptor-mediated synaptic plasticity, and the learning. Understanding the functional role of $Ser^{408\text{-}409}$ phosphorylation of the subunit might be beneficial for the drug discovery and development for multiple cognitive disorders.

### [3P-102]

### Analysis of the mechanism underlying the selective uptake of fluorescently labeled L-glucose analogue 2-NBDLG into mouse insulinoma cells

*Koki Ono[1], Kazuto Kimura[2], Taku Ozaki[2], Ayumu Sugiura[3], Ayako Sasaki[4], Katuya Yamada[1] ([1]*Hirosaki Univ. Grad. Sch. Med., Dept. Molecular Transport*, [2]*Iwate Univ. Sch. Sci. Eng., Dept. Biological Science*, [3]*Juntendo Univ. Grad. Sch. Med., Intractable Disease Research Center*, [4]*Hirosaki Univ. Grad. Sch. Med., Dept. Orthopaedic Surgery*)

L-glucose, the mirror isomer of D-glucose, has been considered a useless sugar because it is not transported into cells through glucose transporters and is not metabolized except in some soil bacteria. A fluorescently labeled D-glucose analogue 2-NBDG has been effectively used for monitoring transport of D-glucose into cells. However, since fluorescence is an arbitrary measure, a control substrate is needs. We have developed 2-NBDLG, the mirror image isomer of 2-NBDG, as the negative control substrate. 2-NBDLG, which was not taken up by non-cancellous cells such as erythrocytes, peritoneal mesothelial cells, muscle cells in human subjects, was taken up into cells in various human biopsy tissue and ascites specimens obtained from patients with adenocarcinoma including gastric, ovarian, uterine body cancers. Moreover, in a preliminary study, the presence of 2-NBDLG-positive cells was correlated well with patient outcome. However, the molecular mechanism underlying the 2-NBDLG uptake is not well understood. In the present study, we show evidence that a channel-like membrane protein contributes to the uptake mechanism.

### [3P-103]

### Nasal ciliary motility maintained by a high intracellular pH under the air condition in ciliated human nasal epithelial cells (c-hNECs)

*Shota Okamoto[1,2], Takashi Nakahari[1,4], Makoto Yasuda[2], Shigeru Hirano[2], Kotoku Kawaguchi[3], Shinji Asano[3], Yoshinori Marunaka[1,4] ([1]*Research Laboratory for Epithelial Physiology, Research Organization of Science and Technology, BKC Ritsumeikan University*, [2]*Department of Otolaryngology Head and Neck Surgery, Kyoto Prefectural University of Medicine*, [3]*Department of Molecular Physiology, College of Pharmaceutical Sciences, Ritsumeikan University*, [4]*Medical Research Institute, Kyoto Industrial Health Association*)

The ciliary beating of nasal epithelia is maintained at an adequate level in the air. In this study, we explored the mechanism maintaining an adequate ciliary beating by a high intracellular pH ($pH_i$) in c-hNECs. Ciliary beat frequency (CBF) was measured by the high-speed video microscopy. An application of $CO_2/HCO_3^-$-free solution did not increase CBF and $pH_i$ in c-hNECs, whereas it increased CBF and $pH_i$ in ciliated human bronchial epithelial cells (c-hBECs). An application on the $NH4^+$ pulse under the $CO_2/HCO_3^-$-free condition, caused $pH_i$ to decrease in c-hNECs, but it did not in c-hBECs. These suggests that $H^+$ is produced even under the $CO_2/HCO_3^-$-freecondition. Under the $CO_2/HCO_3^-$-containing condition, the $pH_i$ measured was higher in c-hNECs (7.66) than in c-hBECs (7.11). S0859 (an inhibitor of $Na\text{-}HCO_3^-$ cotransporter (NBC)) decreased CBF and $pH_i$ in c-hNECs, but did not in c-hBECs, suggesting $HCO_3^-$ entered via NBC increased $pH_i$ in c-hNECs. Thus, a large amount of $HCO_3^-$ entered via NBC maintains a high $pH_i$, which keeps an adequate CBF even in the air.

### [3P-104]

### Pharmacological Investigation of $H^+$ Permeation Mechanism in Outer Nuclear Membrane

*Yoshimichi Murata[1], Yoh Ishihara[1], Masanori Kato[1] ([1]*Graduate School of Medicine, Tohoku University*)

The pH regulation mechanisms of the perinuclear space are not well understood. In this study, we investigated the proton permeation properties of the outer nuclear membrane using isolated nuclear envelope (NE) preparations. pH measurements of the inside of NEs ($pH_{NEi}$) using a genetically encoded ratiometric fluorescent pH probe, mCherrySEpHluorin-ER, showed that The $pH_{NEi}$ followed the external pH change, whereas the degree of change was smaller than the external pH change, indicating that the $pH_{NEi}$ was kept within a regulated range. Ion substitution experiments of inner and outer $K^+$ and $Cl^-$ indicated that luminal $K^+$ and cytosolic KCl contribute to proton transport in response to external acidification. This result suggests the presence of a KCl co-transporter (KCC) in the NE membrane. We investigated the contribution of KCC to $H^+$ transport in NE membranes using KCC activators and inhibitors.

### [3P-105]

### A novel method for analysis of the dynamics of insulin secretory granules

*Daisuke Ohshima[1], Yoshinori Mikami[1], Taichiro Tomida[1], Satomi Adachi-Akahane[1]
([1]*Dept Physiol. Fac Med. Toho Univ.*)

Calcium signaling through voltage-dependent calcium channels (VDCCs) is essential for insulin secretion from pancreatic β cells. Fine-tuning of calcium signaling is necessary for adequate insulin secretion. However, the details of the molecular mechanism still need to be elucidated. We aimed to elucidate the functional relationship between calcium signaling and the dynamics of insulin secretory granules. We have previously generated MIN6 cell lines stably expressing mCherry-labeled insulin and the calcium indicator GCamp7. We have obtained time-lapse imaging using total internal reflection fluorescence microscopy (TIRF) and have successfully applied the machine learning algorithm to segment insulin secretory granules. In this study, we attempted to segment unlabeled insulin secretory granules on time-lapse imaging data from confocal microscopy. As a result, we successfully segmented insulin secretory granules in transmitted light images. This novel method allowed us to quantify calcium signal-dependent changes in the dynamics of insulin secretory granules. In conclusion, we developed a novel method to analyze the subcellular dynamics of native insulin secretory granules.

### [3P-106]

### Effect of medium-chain fatty acids and carnitine to fluid secretion from guinea pig pancreatic duct cells

*Mayuko Higuchi[1], Hiroshi Ishiguro[1], Akiko Yamamoto[1], Miyuki Nakakuki[1], Itsuka Taniguchi[1], Nao Nomura[1], Erina Niwa[1] ([1]*Nagoya university graduate school of medicine*)

Medium-chain fatty acids are fatty acids made up of 8-12 carbons, which are rapidly absorbed in the small intestine and provide a source of energy. Medium-chain fatty acids may therefore promote pancreatic juice secretion, although this remains unclear. Carnitine has been shown to promote membrane transport of long-chain fatty acids. However, it may also facilitate membrane transport of medium-chain fatty acids. So I investigated the effect of medium-chain fatty acids and carnitine on pancreatic secretion. I first isolated pancreatic ducts from the pancreas of guinea pigs. It was incubated for several hours until both ends were closed. The pancreatic ducts were superficially perfused. The effect of medium-chain fatty acids and carnitine on pancreatic secretion were determined from the change in ductal volume. Changes in intracellular $Ca^{2+}$ concentration were measured using fura-2 to investigate the mechanism by which pancreatic secretion is stimulated. Medium-chain fatty acids stimulated pancreatic secretion at about 500 pM or higher. And carnitine enhanced the effects of medium-chain fatty acids. $Ca^{2+}$ concentrations in the duct cells increased during the reaction of medium-chain fatty acids and carnitine on the pancreatic ducts.

### Poster Presentation

[3P]
**Molecular physiology, Cell physiology**
**Ion channels, Receptors**

March 16 (Thu.), 12:10 - 14:10, Poster Room

### [3P-107]

### Analysis of cytokine expression in osteoarthritis and rheumatoid arthritis using synovial fibroblasts of the knee joint.

*Kosuke Kumagai[1,2], Futoshi Toyoda[2], Noriaki Okumura[1], Tsutomu Maeda[1], Hitoshi Tanigawa[1], Mitsuhiko Kubo[1], Mimura Tomohiro[1], Takafumi Yayama[1], Hideki Saito[1], Yasutaka Amano[1], Battulga Khaltar[1], Mariko Omatsu[2], Hiroshi Matsuura[2], Shinji Imai[1]
([1]*Department of Orthopaedic Surgery, Shiga University of Medical Science,* [2]*Department of Physiology, Shiga University of Medical Science*)

Purpose: For degenerative joint diseases (OA and RA) centering on the knee joint, many studies have been conducted on OA cartilage regeneration treatment and the etiology of RA, but the methods and causes have not yet been elucidated. In this study, we investigated synovial membrane cytokine expression levels and cell membrane ion channels, which we have been analyzing for the maintenance of cell homeostasis, to investigate causative factors.Method: Human synovial cells were used with permission from the clinical research ethics committee of Shiga University of Medical Science. In the control (normal) group, the synovium that had to be resected during arthroscopic surgery to secure the visual field was collected, and in the OA/RA group, the resected synovium during TKA was used. Cytokines were measured by ELISA, and cell membrane ion channels were analyzed by PCR after exhaustive analysis using microarrays. Results: Cytokine measurement experiments showed that almost all items were expressed in the RA synovium at the highest level. In OA, the expression level of cytokines with inhibitory activity such as IL-10/G-CSF/MIP-1 was increased. Comprehensive analysis of cell membrane ion channels confirmed increased expression of TRPV1 and others in OA compared to RA. However, no clear significant difference was observed by the qPCR.Discussion and conclusion: From the results of cytokine expression measurement, it was found that the expression of inhibitory cytokines was high in OA-derived synovium. Therefore, it is considered that there is still a possibility that reversible changes can occur compared to RA. In addition, OA and RA have many related items in the inflammatory response. In other words, not only joint deformation but also inflammatory OA is a pathological condition that should be investigated. However, in this experiment, it was not possible to elucidate the ion channels that are the onset factors of OA/RA. In the future, we will continue to analyze candidate ion channels in RA samples subjected to homogenization of drug treatment conditions, and to continue research on whether pharmacological inhibition of these channels can suppress the onset/ progression of joint degenerative diseases.

### [3P-108]

### The role of hydrophilic subunit cavity in the function of transmembrane protein 16F

*Takahiro Shimizu[1], Kanon Shirai[1], Shota Nabeshima[1], Takuto Fujii[1], Hideki Sakai[1]
([1]*Univ. of Toyama, Fac. of Pharm. Sci., Dept. of Pharm. Physiol.*)

Transmembrane protein 16F (TMEM16F) is a member of the TMEM16 family including ion channels and phospholipid scramblases. TMEM16F has a pivotal role in phosphatidylserine exposure during blood coagulation and its dysfunction relates to Scott syndrome, a blood clotting disorder. Interestingly, we have found that human TMEM16F functions as not only a phospholipid scramblase but also a $Ca^{2+}$-activated $Cl^-$ channel with low $Ca^{2+}$ sensitivity. In this study, we investigated the role of the subunit cavity, which is a hydrophilic pore, in the function of TMEM16F. Whole-cell patch-clamp recordings and flow cytometry analysis using annexin V-phycoerythrin (PE) were applied to HEK293T cells overexpressing human TMEM16F. The mutation of cytoplasmic and extracellular phospholipid-stabilizing sites in its subunit cavity (called Sc and Se, respectively) reduced the phosphatidylserine exposure and the $Cl^-$ current triggered by the elevation of intracellular calcium concentrations. Besides, the bulky mutation of the inner gate in the central region of its subunit cavity decreased the scrambling activities as well as ion channel activities. These results suggest that human TMEM16F permeates phospholipids and ions through a highly hydrophilic subunit cavity.

### [3P-109]

### The functional analysis of human TMEM16F at the single-molecule level

*Teppei Kageyama[1], Taiga Sakurai[1], Takahiro Shimizu[1], Hiroyuki Nakao[2], Masayuki Iwamoto[3], Takuto Fujii[1], Shushi Nagamori[4], Minoru Nakano[2], Shigetoshi Oiki[5], Hideki Sakai[1] ([1]*Department of Pharmaceutical Physiology, Faculty of Pharmaceutical Sciences, University of Toyama,* [2]*Department of Biointerface Chemistry, Faculty of Pharmaceutical Sciences, University of Toyama,* [3]*Department of Molecular Physiology and Biophysics, Faculty of Medical Science, University of Fukui,* [4]*Department of Laboratory Medicine, The Jikei University School of Medicine,* [5]*Biomedical Imaging Research Center, University of Fukui*)

The asymmetrical environments of ions across the cell membrane and phospholipids between inner and outer leaflets of the membrane were individually formed by ion or phospholipid transporters, which underlie the various physiological reactions ranging from cell survival to cell death. Transmembrane protein 16F (TMEM16F) functions as a scramblase that disrupts the asymmetry of membrane phospholipids and is associated with blood coagulation. Interestingly, TMEM16F-expressing cells are reported to exhibit $Ca^{2+}$-activated ion currents. To clarify whether TMEM16F is a unique membrane protein that transports both phospholipids and ions, we analyzed the functions of purified human TMEM16F at the single-molecule level. The scramblase activities were analyzed in fluorescent phospholipid-containing liposomes by a quenching assay. The TMEM16F-containing liposomes exhibited a greater fluorescent decay than the protein-free liposomes, suggesting that the TMEM16F transports inner fluorescent phospholipids to the outer leaflet. The ion channel currents were measured by a contact bubble bilayer method. When the TMEM16F protein was incorporated into the bilayer, the single-channel currents were detected. The currents were not observed in the absence of the TMEM16F. These results suggest that human TMEM16F itself has a dual function of scramblase and ion channel.

### [3P-110]

### Intercellular communication between trigeminal ganglion neurons and vascular endothelial cells via $G_s$ coupled receptor axis

*Akira Iwasaki[1], Takehito Ouchi[2], Maki Kimura[2], Akihiro Nishiyama[1], Hidetaka Kuroda[3], Yoshiyuki Shibukawa[2], Akira Katakura[1] ([1]*Department of Oral Pathobiological Science and Surgery, Tokyo Dental College,* [2]*Department of physiology, Tokyo Dental College,* [3]*Department of Dental Anesthesiology, Kanagawa Dental University*)

Pulpitis is caused by neurogenic inflammation in which neuropeptides released from nerve endings into the pulp tissue (axon reflex). To elucidate the detailed mechanism of axon reflex in dental pulp, we examined intercellular communication between rat brain microvascular endothelial cells (RBMECs) and trigeminal ganglion (TG) neurons. We also investigated functional CGRP receptor expression in RBMECs. Adenylyl cyclase (AC) activator, forskolin (FSK), increased intracellular cAMP levels ($[cAMP]_i$) in RBMECs. FSK induced-$[cAMP]_i$ increases were augmented by phosphodiesterase inhibitor. CGRP application to the RBMECs increased $[cAMP]_i$, which was sensitive to CGRP receptor antagonist and AC inhibitor. Direct mechanical stimulation to the TG neurons also increased $[cAMP]_i$ in the surrounding RBMECs in co-culture. RBMECs were immunopositive for $G_s$ protein coupled CGRP receptor, while CGRP-positive peptidergic TG neurons were immunopositive for Piezo channels. CGRP application upregulated pCREB in RBMECs. The results suggested that TG neurons could release CGRP by direct mechanical stimulation mimicking the tissue pressure increases by pulpal inflammation. The released CGRP might be capable of activating CGRP receptors and $G_s$ signaling pathways in RBMECs to establish intercellular communication, as axon reflex, between them.

### [3P-111]

### Evaluation of the cell surface expression of $Ca_V1.2$ channel encoded by the psychiatric risk gene *CACNA1C*

*Mizuki Omori[1], Akito Nakao[1], Yutaro Sugihara[2], Yuta Uchida[1], Shigeki Kiyonaka[2], Yasuo Mori[1] ([1]*Department of Synthetic Chemistry and Biological Chemistry, Graduate School of Engineering, Kyoto University,* [2]*Department of Biomolecular Engineering, Graduate School of Engineering, Nagoya University*)

Calcium influx *via* voltage-gated calcium channel (VGCC) mediates numerous intracellular events such as neuronal excitability, neurotransmitter release and gene expression. Recently, genes encoding VGCC subunits have attracted attention because they are frequently associated with a variety of psychiatric disorders. However, the regulatory mechanisms that control channel trafficking to and from the cell surface remains unclear. In this study, we evaluated the membrane trafficking of $Ca_V1.2$, which is encoded by the psychiatric risk gene *CACNA1C*. We focused on VGCC β subunit, which is reported to regulate the cell surface expression of $\alpha_1$ subunit, and its interacting protein, VGCC β-anchoring and -regulatory protein (BARP). A new method was developed for labeling the cell surface proteins based on click chemistry technology. The results of cell surface labeling by this method and the conventional surface biotinylation assay showed that β subunit and BARP regulate both the surface and total expression of $Ca_V1.2$. It was also suggested that the alteration of $Ca_V1.2$ expression is mediated by proteasomal degradation of the channels. Further investigation by live cell imaging based on our new click chemistry method would provide new insight into the mechanism regulating the trafficking of $Ca_V1.2$.

### [3P-112]

### Differential effects of the *Lurcher* mutation on the channel activity of human and mouse GluD2 receptors.

*Masayuki Itoh[1], Wataru Kakegawa[1], Michisuke Yuzaki[1] ([1]*Department of Neurophysiology, Keio University School of Medicine*)

Ionotropic glutamate receptors (iGluRs), consisting of GluAs, GluKs, GluNs and GluDs, play vital roles at excitatory synapses in vertebrates. Unlike other iGluRs, GluDs do not form functional ion channels in heterologous cells *in vitro*. However, a single amino acid mutation (A654T) in the third transmembrane region, originally found in *Lurcher* (*Lc*) mice, caused GluD2 channels to open spontaneously. Thus, it remains unclear whether and how GluDs show ion channel activities *in vivo*. To obtain clues, we examined the effect of *Lc* mutation on the channel activity of mouse ($mGluD2^{Lc}$) and human ($hGluD2^{Lc}$) receptors in HEK293 cells. Surprisingly, HEK293 cells expressing $hGluD2^{Lc}$ showed less than one-tenth of the leak current of those expressing $mGluD2^{Lc}$. To understand the differential effects of *Lc* mutation on mouse and human GluD2, we examined the effect of *Lc* on mouse-human GluD2 chimeric receptors. Site-directed mutagenesis revealed that single amino acid at the fourth transmembrane region was mainly responsible for the differential effect of the *Lc* mutation. Further, our preliminary data showed that a point mutation mimicking hGluD2 in the corresponding region reduced glutamate-gated currents in homomeric GluA2 or GluK2 in HEK293 cells. These findings indicate that GluD2 shares a common channel-gating mechanism with other iGluRs but hGluD2 may be difficult to be gated.

### [3P-113]

### Nitrergic modulation of muscle contraction in different regions of the murine colon

*YAO YU[1], Shinsuke Nakayama[1], Chiho Takai[1] ([1]*Nagoya University*)

Gastrointestinal tract shows spontaneous movement that is operated by at least three motor systems: smooth muscle bundles, intrinsic neural circuits and pacemaker interstitial cells, referred to as interstitial cells of Cajal (ICCs). In the myenteric plexus nitrergic neurons release nitric oxide (NO) synthesized by nitric oxide synthase (NOS) to cause smooth muscle relaxation via cGMP pathway. In this study, we examined the effects of L-NNA, an inhibitor of NOS on spontaneous contractions in three different regions of colon isolated from transgenic mouse expressing fluorescent $Ca^{2+}$ indicator YC-Nano50 in the musculature. Contractile movement was separately assessed in circular and longitudinal directions by tracking an ROI of fluorescent images. In the middle and distal regions L-NNA amplified the vertical movement, but had little effect in the transverse movement. Conversely in the proximal, movement was increased in both directions. The results suggest nitrergic innervation differently suppress circular and longitudinal muscle contractions between proximal and middle/distal regions. Interestingly, in the presence of L-NNA the basal $Ca^{2+}$ concentration decreased in the middle/distal regions, indicating a paradoxical regulatory mechanism in intestinal muscle contraction.

### [3P-114]

### Two molecules of calmodulin also contribute to $Ca^{2+}$-dependent inactivation and its responsible binding site in Cav1.2 channel

*Etsuko Minobe[1], Jianjun Xu[1], Masaki Kameyama[1] ([1]*Kagoshima University*)

Calmodulin (CaM) is essential to regulate activity of Cav1.2 channels, but the its molecular mechanism is not fully clarified. We have previously proposed the two-site model, in which two independent CaM binding sites responsible for the channel modulations, activation and inactivation, respectively, are hypothesized in the intracellular domain of the channel. CaM vs. channel activity curve shows bell-shaped relationship at a constant $Ca^{2+}$ concentration. In this study, we utilized the Cav1.2 channel linked with CaM through a glycine linker to the C-terminal side and its N-terminal domain was deleted (Ndel-Cav1.2-CaM). The Ndel-Cav1.2-CaM inactivated in a CaM-dependent manner, but not in a $Ca^{2+}$-dependent manner (CDI) without external CaM, revealing that CDI was caused by one CaM through the N-terminal domain of the channel. To test the contribution of two molecules of CaM in CDI, CDI of the Ndel-Cav1.2 in the presence of external CaM (1 microM) was compared with that of the wild-type. Then we introduced point mutations into the predicted CaM-binding sites in the channel, and evaluated their effects on CDI using patch-clamp recording and pull-dawn assay. (COI: NO)

### [3P-115]

### Mitochondrial TRPA1 Regulates the Activity of Mitochondria under Stressful Conditions in Cancer Cells

*JIACHUN XU[1] ([1]*Mori Laboratory, Department of Synthetic and Biological Chemistry, Graduate School of Engineering, Kyoto University*)

TRPA1 is a member of TRP family, which has been reported to be activated by environmental factors to mediate calcium influx, thereby activating intracellular signaling in response to environmental stress. However, it is unknown whether TRPA1, as a membrane protein, can also localize to membrane-bound organelles and regulate their activities. Here, we found that TRPA1 also localize on mitochondria and involved in mitochondrial oxidative stress response. TRPA1 enhances the level of mitochondrial tolerance to ROS, supporting ATP production and defending oxidative stress. This finding provides insight into the internal metabolic mechanisms of cancer cells and the response to external oxidative stress. This finding provides new clues to understand the internal metabolic mechanism and response to external oxidative stress in cancer cells.

### [3P-116]

### Inhibitory modulation of voltage- and pH-gated Slo3 potassium channel by zinc

*Rizki Tsari Andriani[1], Yasushi Okamura[1], Takafumi Kawai[1] ([1]*Integrative Physiology, Graduate School of Medicine, Osaka University.*)

Voltage- and pH-gated Slo3 potassium channel is exclusively expressed in mammalian spermatocyte. Its sensitivity to both voltage and alkalization plays an important role in sperm fertility, and Slo3-deficiency in mice sperm causes severe infertility in vivo. Modulation of the activities of Slo3 is thought to have a significant influence in sperm physiology. Here we show that divalent cation $Zn^{2+}$ show the dynamic alteration in sperm flagellum upon sperm capacitation and it regulates alkalinization-induced hyperpolarization in mouse sperm cells which is mediated by Slo3 channel. We further examined the detailed zinc modulation in mouse Slo3 (mSlo3). In *Xenopus* oocyte expression system, zinc inhibited mSlo3 currents dose-dependently at micromolar concentrations, with an exceptionally long washout period. By point-mutation analysis, we also identified some amino acid residues that are important for $Zn^{2+}$ suppression of Slo3 activities. Taken together, these will provide us with a clue to study the detailed inhibitory modulation of mSlo3 currents by zinc.

### [3P-117]

### Effects of FMRFamide analogues on the FMRFamide-gated $Na^+$ channel

*Yasuo Furukawa[1], Megumi Furumitsu[2], Kazuyoshi Ukena[2] ([1]*Laboratory of Neurobiology, Graduate School of Integrated Sciences for Life, Hiroshima University,* [2]*Laboratory of Neurometabolism, Graduate School of Integrated Sciences for Life, Hiroshima University*)

FMRFamide-gated $Na^+$ channel (FaNaC) is a homo-trimeric peptide-gated sodium channel, which is activated by a molluscan cardioactive peptide, FMRFamide (FMRFa). We previously reported that six aromatic residues in the extracellular domain of FaNaC which are conserved among molluscan FaNaCs are involved in the FMRFa sensitivity of *Aplysia* FaNaC, AkFaNaC, (Furukawa and Tagashira, FAOPS2019). Among the tested mutants, W167V was not activated even by millimolar FMRFa. To address the functionality of aromatic moiety at position 167, we examined two conservative mutants of W167, W167F and W167Y. These mutants expressed the functional FaNaC but their EC50s were nearly 100-fold larger than that of the wild-type channel (Furukawa and Tagashira, ZSJP2022). Based on these results, we hypothesized that W167 is a key residue for the activation of FaNaC, and that the aromatic moiety of W167 interacts with 1st or 4th phenylalanine of FMRFa. In the present study, we made FMRFa analogues which have different aromatic amino acids at its 1st and/or 4th position and checked their potencies in FaNaC. EC50 of FMRWa was similar to that of FMRFa in FaNaC but the maximum response by FMRWa was approximately one half of that by FMRFa. We also checked WMRFa as well as WMRWa and found that they are less effective agonists than FMRWa. In a preliminary experiment in W167F, we found that the potency of FMRFa analogues depends on the tested concentration. These results may suggest that the aromatic moiety at position 167 interacts with the aromatic amino acids in FMRFa.

### [3P-118]

### The interplay between the membrane thickness and tension on the gating of the KcsA potassium channel

*Yuka Matsuki[1], Masayuki Iwamoto[2], Masako Takashima[2], Shigetoshi Oiki[3] ([1]*Dept. Anesth. Reanimatol., Univ. Fukui Fac. Med. Sci.*, [2]*Dept. Mol. Neurosci., Univ. Fukui Fac. Med. Sci.*, [3]*Biomed. Imag. Res. Cent., Univ. Fukui*)

At the interface between a channel protein and the surrounding membrane, the hydrophobic part of the transmembrane domain (hydrophobic band) governs the local thickness of the hydrophobic core of the membrane (hydrophobic match). Generally, potassium channels undergo global conformational changes upon gating and alter the width of the hydrophobic band, thus leading to further modification of the local membrane thickness. To examine the interplay between channel proteins and membrane, the KcsA potassium channel was reconstituted into the lipid bilayer called the contact bubble bilayer, and the effect of the membrane thickness on the activation gate was examined. The membrane thickness was changed using phosphatidylcholine of different acyl lengths and evaluated by the capacitance and membrane area measurements. The open probability ($P_{open}$) decreased with the increased membrane thickness. Even in thick membranes, the $P_{open}$ increased substantially as the membrane tension increased. The interplay between membrane thickness and tension on the gating channel was discussed.

### [3P-119]

### The mechanism of hydrogen sulfide-induced activation of HCN channel in cultured rat dorsal root ganglion neurons.

*You Komagiri[1] ([1]*Department of Physiology, School of Medicine, Iwate Medical University, Japan*)

Hydrogen sulfide ($H_2S$) and Hyperpolarization-activated cyclic nucleotide-gated (HCN) channels are thought to be involved in generating chronic pain after nerve injury. We previously reported that $H_2S$ donor shifted the voltage-dependence of HCN channel current ($I_h$) toward positive potential without the adenylate cyclase activity. Here we investigated the mechanism of the $H_2S$-induced activation of HCN channel in cultured DRG neurons with gramicidin perforated patch-clamp technique. The pretreatment of LNMMA, a Nitric oxide synthase (NOS) inhibitor, and ODQ, a guanylate cyclase inhibitor, significantly inhibited the $H_2S$-induced shift in the voltage-dependence of $I_h$, respectively. In addition, application of NO scavenger (C-PTIO) abolished the effect of $H_2S$ on $I_h$. These results suggest that $H_2S$ regulates the voltage-dependence of $I_h$ activation via NO signaling in rat DRG neurons. We will further examine a cross talk among $H_2S$ and NO in the mechanisms underlying the $H_2S$-induced activation of $I_{h.}$

### [3P-120]

### The lack of dystrophin attenuates voltage-sensitive potassium current of nucleus tractus solitarius neurons in muscular dystrophy model rats

*Akitoshi Chikamoto[1], Shin-ichi Sekizawa[1], Ryota Tochinai[1], Masayoshi Kuwahara[1] ([1]*Laboratory of Veterinary Pathophysiology and Animal Health, Graduate School of Agricultural and Life Sciences, The University of Tokyo*)

[Background]Duchenne muscular dystrophy (DMD) is a severe and progressive neuromuscular disorder, resulting in cardiac and respiratory failure. Using DMD model rats (W-*Dmd*$^{em1Kykn}$, DMDRs), we have found that the signaling mechanism of neurons in the nucleus tractus solitarius (NTS), a brainstem region important for cardio-respiratory regulation, is different from its wild-type (WT), particularly being characterized by low excitability of the neurons. To clarify the mechanism of low neuronal excitability, we hypothesized that voltage-activated potassium channels were responsible and performed *ex vivo* whole-cell patch-clamp experiments in NTS neurons from DMDRs and WT rats.[Methods]Three-month-old male DMDRs (DMD group) and their WT littermates (WT group) were used for the study. A neuronal tracer, DiI, was implanted onto the right carotid body of animals to identify second-order NTS neurons in brainstem slices >3 weeks before performing electrophysiological experiments. After the whole-cell configuration was formed on the NTS neurons and their membrane properties were tested, inactivation and non-inactivation of outward currents were studied under the voltage-clamp conditions in the presence of sodium channel blockers. [Results]There was no significant difference in intrinsic membrane properties, such as resting membrane potential, membrane capacitance, and input resistance between WT and DMD groups. Low neuronal excitability of DMDRs was reconfirmed by blunted spiking responses to depolarizing current injection as had been observed before. In voltage-clamp configuration, smaller transient and steady-states outward current densities as well as smaller non-inactivating current densities were observed in DMDRs compared to WT rats, while voltage sensitivities of those currents were similar between groups. [Discussion]The lack of dystrophin may influence the number of expressions of voltage-sensitive K+ channels which can contribute to excitability of NTS neurons. However, properties of other ion channels may also be different in DMDRs and thus further studies will be needed to clarify the precise mechanism for the role of dystrophin on neural excitation. It may be hoped that compensating NTS neuronal excitability will decelerate the pathological progression of the cardio-respiratory system in DMD patients.

### [3P-121]

### Involvement of skin TRPV3 in temperature detection regulated by TMEM79 in mice

*Jing Lei[1,2,3], Reiko Yoshimoto[4], Takeshi Matsui[5,6], Masayuki Amagai[5,7], Mizuho Kido[4], Makoto Tominaga[1,2,3] ([1]*Thermal Biol., ExCELLS, Okazaki, Japan*, [2]*Dept. Physiol. Sci., SOKENDAI, Okazaki, Japan*, [3]*Div. Cell Signal., NIPS, Okazaki, Japan*, [4]*Div. Histol. and Neuroanat., Saga Univ, Saga, Japan*, [5]*Skin Homeost., RIKEN, Yokohama, Japan*, [6]*Evol. Cell Biol. of Skin, Sch. Biosci. and Biotech., Tokyo Univ. of Tech., Tokyo, Japan*, [7]*Dept. Derm., Keio Univ. Sch. of Medicine, Tokyo, Japan*)

TRPV3, a non-selective cation transient receptor potential (TRP) ion channel, is activated by warm temperature. It is predominantly expressed in skin keratinocytes, and it participates in various somatic processes. Although TRPV3 was suggested to be required for thermosensation, this interpretation is still controversial because of the lack of direct evidence. Here we identified a transmembrane protein, TMEM79, that acts as a regulator of TRPV3. Heterologous expression of TMEM79 was capable of suppressing TRPV3-involved currents in HEK293 cells. In addition, TMEM79 modulated TRPV3 translocalization and promoted its degradation in the lysosomes. TRPV3-related currents and Ca2+ influx were potentiated in primary mouse keratinocytes lacking TMEM79. Furthermore, TMEM79-deficient mice preferred a higher temperature than wild-type mice due to elevated TRPV3 function. Overall, our study revealed an interaction between TRPV3 and TMEM79 in vitro and in vivo, which provided direct support for the underlying involvement of TRPV3 in thermosensation.

### [3P-122]

### Involvement of proteasome in gamma-ENaC surface expression in renal epithelial A6 cells

*Naomi Niisato[1], Yoshinori Marunaka[2,3,4] ([1]*Kyoto University of Advanced Science*, [2]*Kyoto prefectural University of Medicine*, [3]*Medical Research Institute, Kyoto Industrial Health Association*, [4]*Research Organization of Science and Technology, Ritsumeikan University*)

Epithelial $Na^+$ channel (ENaC)-mediated $Na^+$ reabsorption in the cortical collecting duct plays an important role in regulation of extracelullar fluid volume and blood pressure. The $Na^+$ entry step via ENaC expressed at the apical surface membrane is the rate-limiting step for transepithelial $Na^+$ reabsorption. Previous our study indicates that SB202190 (a p38 inhibitor) drastically reduces gamma-ENaC surface expression through suppression of Nedd4-2-dependent ubiquitination in aldosterone-treated renal epithelial A6 cells. In this study, we investigated possible roles of p97 (an AAA$^+$ ATPase) and proteasome for ENaC degradation to regulate surface expression and found that 1) inhibitory effect of SB202190 on ENaC surface expression was recovered with proteasome inhibitors (MG132 and epoxomicin), 2) treatment with a p97 inhibitor (MBDM) induced accumulation of gamma-ENaC and p97 in the apical membrane., and 3) deubiquitinating enzyme (DUB) inhibitor (WP1130) abolished the recovery of ENaC-mediated Na+ entry by proteasome inhibitors. These results suggest that p97 might be a crucial factor for ENaC retrieval and degradation, and that DUBs (regulatory subunits of 19S proteasome such as RPN11, UCH37 and USP14) inhibitors suppressed the recycling of ENaC rescued by proteasome inhibitors to the apical membrane.

### [3P-123]

### Therapeutic drugs developed for CFTR mutants found in Caucasian Cystic Fibrosis patients also succeeded to rescue CFTR mutants found in Japanese patients.

Rio Kimishima[1], Hikaru Sohma[1], Hiroshi Nakagawa[2], Tadaomi Furuta[3], Hiroshi Ishiguro[4], Kanako Nakao[1], *Yoshiro Sohma[1] ([1]*International University of Health and Welfare*, [2]*Chubu University*, [3]*Tokyo Institute of Technology*, [4]*Nagoya University*)

Cystic Fibrosis (CF) is the most popular, life-shorten, inheritance disease in Caucasians, which is caused by function-loss mutations in Cystic Fibrosis Transmembrane conductance Regulator (CFTR). The most frequent mutation in Caucasians is a deletion of phenylalanine508 (ΔF508), classified into the class II (trafficking defect). Recently a few chemical chaperons (correctors) for rescuing the ΔF508 mutant from the trafficking defect were developed by Vertex Inc and approved by FDA in USA. CF is very rare but does exist in Japanese. However the CFTR mutation profiles in Japanese are different from those in Caucasians. At present, twenty-two mutations were identified from twenty-four Japanese CF patients definitely diagnosed. The first to third most frequent Japanese CF mutations, Δ(G970-T1122), H1085R and L441P are all classified into class II. Our previous study found that unfortunately the most frequent Δ(G970-T1122)-mutant could not be rescued by the Vertex drugs. In 2019, the FDA granted Vertex Inc. approval for "Trikafta", a combination of two expression correctors, elexacaftor and tezacaftor, and one channel function potentiator, ivacaftor. In Japan, at present, the CF treatment is limited to some symptomatic ones and no radical treatment has not been approved yet. Trikafta could be effective against disease-associated mutations other than ΔF508 including Japanese-specific mutations. In this presentation, we will discuss about the therapeutic effects of Tricafta against H1085R and L441P, based on our *in vitro* data.

### [3P-124]

### Impacts of impairments of the $Ca^{2+}$-dependent inactivation in TRPC6 channel on the differentiation and morphology in podocytes and the age at onset of Nephrotic syndrome

*Ryo Okada[1,2], Reiko Sakaguchi[2], Masayuki X. Mori[2] ([1]*Human,Information and Life Sciences, School of Health Sciences, University of Occupational and Environmental Health,* [2]*Laboratory of Biomaterials and Chemistry*)

Transient Receptor Potential Canonical 6 (TRPC6) is a tetrameric $Ca^{2+}$/$Na^{+}$-permeable nonselective cation channel, and is expressed in the various types of cells including podocytes in kidney glomerulus. Mutations of this gene are known to be associated with the two major causes of nephrotic syndrome (NS), namely minimal change and focal segmental glomerular sclerosis (FSGS). It has been shown that impairment of $Ca^{2+}$-dependent inactivation (CDI) in TRPC6 channels is a cause of NS. However, the physiological roles of CDI in development and maintenance of the glomerular filtration barrier are largely unknown. Here, we quantitatively evaluated the activities of NS-associated TRPC6 channels by the patch-clamp recording. CDI of NS-associated channel currents were significantly delayed compared to that of wild-type, and TRPC6 mutations associated with the early-onset NS exhibited increased integration of the current density compared to that of mutations associated with the late-onset NS. Furthermore, we edited the TRPC6 gene in mouse podocyte MPC5 cells by the CRISPR/Cas9 system, and the cell line which expressed CDI impaired-TRPC6 was established. In this meeting, we would like to discuss the physiological significance of CDI in the glomerular filtration barrier through quantitative evaluation of the channel activities by patch-clamp recording and podocyte differentiation and cell morphologic assay with the established cell line.

### Poster Presentation

[3P]
**Embryology, Regenerative Medicine, Development, Growth, Aging**

March 16 (Thu.), 12:10 - 14:10, Poster Room

### [3P-125]

### Study on catecholamine administration for browning in primary culture of fat tissue from pig.

*Sangwoo Kim[1], Akari Koide, Erina Yoneda[1], Yuki Muranishi[1] ([1]*Obihiro University of Agriculture and Veterinary Medicine*)

White adipocyte is essential for energy storage and endocrine regulation of metabolism and regulation of homeostasis. Recently, white adipocyte has been reported that browning to beige adipocyte which consume energy to thermogenesis, by cold exposure or catecholamine administration. However, it is unclear the detail of browning mechanism in homeothermal animal. Pig has focused on human medical research because that physiology is similar to human biological function. The aim of this study is to examine the browning condition using primary culture of pig fat and investigate the molecular mechanism *in vitro*.Primary fat culture were induced to mature adipocyte and to examine the optimal concentration of isoproterenol which is catecholamine preparation (adrenergic-β receptor agonist). These cells were evaluated the gene expression related to thermogenesis. In result, 1 μM isoproterenol concentration increased significantly UCP3 and PGC-1a ($P < 0.05$). And, the gene expression of COX1, COX2 and COX3 related to mitochondria activity were upregulated in 1 μM ($P < 0.05$). Furthermore, the matured adipocytes were significantly reduced the droplet size in 1 μM isoproterenol. We confirmed the browning condition at primary pig fat culture and this *in vitro* system is available for metabolic research.

### [3P-126]

### Involvement of glycine receptors in the development of hippocampal neurons

*Shoichiro Ikeda[1], Masahiro Mori[1] ([1]*Kobe University*)

Glycine is a major inhibitory neurotransmitter in the central nervous system and its receptors, glycine receptors are ligand-gated chloride channel receptors. Functional glycine receptors are expressed in the hippocampus, where glycinergic synaptic transmission has not been reported. Thus, roles of glycine receptors in the hippocampus have not been clarified yet. We studied if glycine receptors might be involved in the development of hippocampal neurons. The responses to pressure application of glycine (0.3 mM, 0.5-1 s) peaked at DIV 8 in CA3 pyramidal cells of the cultured hippocampal slices made from newborn rats (P0-1). We analyzed two-dimensionally morphological parameters such as total dendritic length and number of branching points in biocytin-labeled CA3 pyramidal cells and dentate granule cells of the hippocampal slices cultured with a glycine receptor blocker, strychnine (1-10 microM). The mechanisms, by which glycine receptors could affect the development of hippocampal neurons will be discussed.

### [3P-127]

### In vitro generation of goblet cell metaplasia model using iPS cell-derived airway epithelium

*Susumu Yoshie[1], Akihiro Hazama[1] ([1]*Dept Physiol, Grad Sch Med, Fukushima Med Univ, Fukushima, Japan*)

[Background] Goblet cell metaplasia caused by asthma and habitual cigarette smoking leads to excessive mucus production and airway obstruction. However, the pathogenic mechanism of goblet cell metaplasia has not yet been fully elucidated. The aim of this study is to generate goblet cell metaplasia model using iPS cell-derived airway epithelium in order to elucidate the pathogenic mechanism of goblet cell metaplasia.[Methods] We generated airway epithelium via spheroid formed from iPS cells based on serum-free conditions. Goblet cell metaplasia model was generated from iPS cell-derived airway epithelium by the use of cigarette smoking solution. [Results] Airway epithelium generated from iPS cells expressed airway epithelium markers and had functional characteristics such as ciliary movement and $Cl^-$ transport. Furthermore, iPS cell-derived airway epithelium treated with cigarette smoking solution strongly expressed goblet cell markers. Mucin-positive cells were also appeared.[Conclusions] We succeeded in the generation of goblet cell metaplasia model from iPS cell-derived airway epithelium.

### [3P-128]

### *Fut9* Deficiency Causes Abnormal Neural Development in the Mouse Cerebral Cortex and Retina

*Asmaa Abdullah[1], Yoshitaka Hayashi[1], Naoko Morimura[1], Akhilesh Kumar[2], Kazuhiro Ikenaka[2], Akira Togayachi[3], Hisashi Narimatsu[3], Seiji Hitoshi[1,2] ([1]*Shiga University of Medical Science,* [2]*The Graduate University for Advanced Studies,* [3]*National Institute of Advanced Industrial Science and Technology*)

α1,3-Fucosyltransferase 9 (Fut9) is responsible for the synthesis of Lewis X [LeX, Galβ1-4(Fucα1-3)GlcNAc] carbohydrate epitope, a marker for pluripotent or multipotent tissue-specific stem cells. In this study, using *in situ* hybridization and immunohistochemistry, we clarified the spatiotemporal expression of Fut9, together with LeX, in the brain and retina. We found that Fut9-expressing cells are positive for Ctip2, a marker of neurons residing in layer V/VI, and TLE4, a marker of corticothalamic projection neurons (CThPNs) in layer VI, of the cortex. A birthdating analysis using EdU at embryonic day (E)11.5 and BrdU at E12.5 revealed a reduction in the percentage of neurons produced at E11.5 in layer VI/subplate of the cortex and in the ganglion cell layer of the retina in P0 Fut9 KO mice. Furthermore, this reduction in layer VI/subplate neurons persisted into adulthood, leading to a reduction in the number of $Ctip2^{strong}$/$Satb2^{weak}$ excitatory neurons in layer V/VI of the adult Fut9 KO cortex. To investigate behavioral abnormalities in Fut9 KO mice, we are performing open field, 3-chamber social interaction, and acoustic startle response using Fut9 KO mice. Our current data suggest that Fut9 plays significant roles in the differentiation, migration, and maturation of neural precursor cells in the developing cortex and retina.

### [3P-129]

### $PGE_1$ responsiveness of 3D spheroids with ductus smooth muscle cells in fetus rats

*Takahiro Inoue[1], Toru Akaike[1], Hiroki Bochimoto[1], Susumu Minamisawa[1]
([1]*Department of Cell Physiology, The Jikei University School of Medicine*)

Background) Recently, characters of smooth muscle cells (SMCs) in ductus arteriosus (DA) have been well described. However, the major issue is that assessment of drug response to DA-SMCs have been generally performed in the setting of 2D-monolayer culture instead of 3D-culture, which more represents an authentic cell environment.Materials and Methods) SMCs were isolated from DA and descending thoracic aorta (dAo) in Wistar rat fetuses on embryonic day 21. Following passages 4 to 6, $4x10^4$cells were employed for one spheroid and maintained for 5 days to create a spheroid. $PGE_1$ responsiveness was evaluated after 2 days of $PGE_1$(1μM) stimulation under fetal bovine serum-free medium. Results) Production of hyaluronic acid per unit number of cells was greater in 2D-monolayer culture than in 3D-spheroids (5.50±0.73 vs 3.72±0.17ng/ml, $p<0.01$). H&E staining showed that cells were secured and filled inside a spheroid without central necrosis, and EVG staining revealed that elastic fibers were located around DA spheroid after $PGE_1$ administration as well as the similar staining pattern on hyaluronan binding protein(Versican). On scanning electron microscopic examination, the surface of DA spheroid was rough with bumps and dips at 6 hours, but it altered smooth with uniform formation at 48 hours following $PGE_1$ administration. These morphological changes may demonstrate that hyaluronic acid is released from SMCs in the outside of DA spheroids that are exposed to and stimulated by $PGE_1$. Conclusion) To our knowledge, this is the first study to create spheroids with single smooth muscle cells in ductus arteriosus. DA spheroids can be a better tool to observe the configurational modification after $PGE_1$ administration. (COI:No)

### [3P-130]

### Brain commissure development in marmoset from infancy to juvenile: longitudinal MRI study

*Akiko Uematsu[1], Makoto Fukushima[1], Junichi Hata[2], Noriyuki Kishi[1], Ayako Murayama[1], Takuya Hayashi[1], Hideyuki Okano[1,3] (*[1]RIKEN, [2]Tokyo Metropolitan Univ., [3]Keio Univ.*)

A featured animal in neuroscience research field, common marmoset, is a usable animal model for primate development study in that it grows relatively fast among primates. In the current study, we delineate the maturational process of brain commissural structure in both qualitatively and quantitatively in marmoset. We collected longitudinal multi-modal MRI images, including T1- (T1w), T2- (T2w), and diffusion- (DWI) weighted images, from infancy (2 weeks old) to Juvenile (6 months old) stage (N=21). T1w and T2w images are used to examine age-related image contrast changes and to estimate its volume. DWI is used to calculate diffusion tensor image (DTI) metrics including fractional anisotropy (FA) and mean diffusivity (MD). The intensity values of anterior commissure and corpus callosum in T1w and T2w images showed similar values as gray matter region until 2 to 3 months old. Consistent with the contrast change, the FA values dramatically increased during first 3 months after birth. On the other hand, the MD values showed no significant change along with development. These findings might demonstrate a primate specific feature of commissure development that both axonal development and axon pruning occur in parallel.

### Poster Presentation

### [3P]
### Muscle

March 16 (Thu.), 12:10 - 14:10, Poster Room

### [3P-131]

**Development of EAD in heart cells involves reverse E-C coupling and reverse electrotonic conduction along T-tubules.**

*Takao Shioya[1] ([1]*Department of Physiology, Faculty of Medicine, Saga University*)

Early after depolarization (EAD) underlies the development of life-threatening ventricular arrhythmias. Since EADs develop preferentially in damaged heart cells with abnormal $Ca^{2+}$-signaling, I studied the causal link between the development of EADs and aberrant intracellular $Ca^{2+}$ ($[Ca^{2+}]_i$) dynamics, using nystatin "superforated-patch" recording technique and $[Ca^{2+}]_i$ imaging by fluo-3 AM in mouse heart cells. My results show: 1) The generation of EADs was preceded by the development of depolarizing membrane potential ($V_m$) fluctuation. 2) The depolarizing Vm fluctuation occured concurrently with a local brief $[Ca^{2+}]_i$ elevation, and the $V_m$ fluctuation was eliminated when $Ni^{2+}$ was used to block the $Na^+/Ca^{2+}$ exchanger. 3) The generation of the $V_m$ fluctuation and EADs were suppressed when the T-tubule system of was detubulated. 4) Abbreviating the T-tubule's length constant by increasing the extracellular $K^+$ level suppressed the $V_m$ fluctuation and EADs accordingly. I conclude that EADs are caused by the depolarizing $V_m$ fluctuation, which is induced locally in the T-tubule membrane by aberrant $[Ca^{2+}]_i$ elevation and is conducted electoronically along the T-tubules back to the surface membrane.

### [3P-132]

**Effects of contraction mode and intensity on sarcomere structure of skeletal muscle**

*Kazuhiro Hirano[1], Hideki Yamauchi[1], Naoya Nakahara[1], Maki Yamaguchi[1], Tomonori Hayashi[1], Shigeru Takemori[1] ([1]*Jikei Univ.*)

We previously compared the effects of tetanic eccentric contractions (ECC) of varied intensity and fixed number of iterations, and found that low-intensity ECC successfully triggered biochemical processes for muscle hypertrophy without deteriorating sarcomere fine structure. In the present study, we aimed to study the effects of high-intensity ECC of fewer iterations. Plantaris muscles with maintained blood perfusion of 7-week healthy rats were divided into control group (CON), isometric contraction group (ISO), low-intensity ECC group (L-ECC), and high-intensity ECC group (H-ECC). The stimulation frequencies were 50 Hz for L-ECC, 100 Hz for ISO and H-ECC. L-ECC muscles received tetanus stimulation of 0.3 sec duration iterated 30 times every 3 seconds. The iteration number of ISO and H-ECC were adjusted to match its tension-time integral to that of L-ECC. After respective contraction load, the muscle was excised to prepare skinned fibers, and the sarcomere structure was evaluated by x-ray diffraction. Fine sarcomere structure of H-ECC muscles was well preserved. We are focusing on the meridional troponin reflection, which were proposed as tentative mechanical sensor for triggering muscle hypertrophy.

### [3P-133]

**CGRP-cAMP-dependent signal transduction pathways upregulate MyHC I mRNA through the activation of PKA in C2C12 cells.**

*Yoshiaki Mori[1], Hiroshima Reiko[1], Yamaji Junko[2] ([1]*Dept. of Rehab. Sci., Kansai Univ. of Welf. Sci.*, [2]*Dept. of Nutr. Sci., Kansai Univ. of Welf. Sci.*)

Our previous study using differentiated C2C12 cells indicated that myosin heavy chain type I (MyHC I) mRNA expression level was significantly increased by the application of CGRP. CGRP peptides are mainly localized in sensory and central neurons and have been implicated in a variety of physiological processes. CGRP has also been identified in spinal motoneurons of several species and in the nerve terminals of the rodent neuromuscular junction. C2C12 cell line appears to express CGRP receptors coupled to adenylyl cyclase activity, therefore, we examined the contribution of cAMP-dependent pathways on the upregulation of MyHC I mRNA levels in C2C12 cells. MyHC I mRNA expression levels were measured by the real-time PCR method. MyHC I mRNA levels were significantly increased by the administration of isoprotelenole, forskolin, or 8-Br-cAMP. The effects of forskolin on MyHC I mRNA expression levels were significantly inhibited by the co-administration of PKA inhibitor, H-89. However, MyHC I mRNA expression levels were not affected by the application of CREB inhibitor, 665-15, and Epac2 inhibitor, HCJ1350. These results suggested that the upregulation of MyHC I mRNA level by the activation of CGRP-cAMP-dependent signal transduction pathways was involved in PKA-mediated mechanisms in C2C12 cells.

### [3P-134]

**Structural stability of sarcomere structure of in vivo muscle with maintained blood supply**

*Naoya Nakahara[1], Hideki Yamauchi[1], Maki Yamaguchi[1], Tomonori Hayashi[1], Kazuhiro Hirano[1], Shigeru Takemori[1] ([1]*The Jikei University School of Medicine*)

We obtained x-ray diffraction patterns from in vivo extensor digitorum longus muscle of anesthetized 6-month female ICR mice in KEK. Tetanic 100 Hz electrical stimulation of 0.5 msec duration at supramaximal intensity through nerve was iterated 10 times every 10 second to contract the muscle. Due to maintained blood supply, tetanus tension showed no sign of fatigue. In the x-ray diffraction patterns, myosin and actin layer lines were clearly visible. Contraction induced general intensity decrease of myosin layer lines and relative increase of actin layer lines. Equatorial 1,1/1,0 intensity ratios were surprisingly low; 0.1 at rest and 0.15 at contraction. For comparison, resting skinned muscle showed the ratio of 0.4. In skinned fibers, most vulnerable is the myosin layer lines, which represents ordered helical arrangement of myosin heads around the shaft of thick filament. The present study suggested that in the sarcomere of in vivo muscle, radial positioning of thin actin filaments on the 1.1 plane of hexagonal thick filament lattice is more vulnerable than the helical arrangement of myosin heads. Some unknown factor may act to destabilize the positioning in vivo.

### [3P-135]

**Correlation between muscle pain and the meandering structures of muscle fibers and stretching treatment for contracted calf muscle.**

*Akihiro Kaizu[1], Yoshiyuki Tsuboi[1] ([1]*Department of Physiology, Nihon University School of Dentistry*)

Background
Leg cramps are painful contraction of muscle and usually common in old people. They affect a significant impact on quality of life such as sleep in older people. After involuntary muscle contraction, muscle become hard and remain discomfort. However, the mechanism of remaining pain in muscle is unclear.
Objective
In this study, we aimed to acquire fundamental knowledge for abnormal contraction in rat calf muscle.
Method
Tibial nerve stimulation under general anesthesia (2% isoflurane) is used for obtain abnormal contraction model in rat. Hardness (stiffness and tone) of contracted muscle were measured using Myotone Pro®. The structure of muscle fibers was observed under microscope. pERK expression in DRG was analyzed with immunohistochemical staining method.
Results
After artificial abnormal contraction (ACC), muscle hardness such as stiffness and tone were significantly increased. Ankle ROM were limited against dorsiflexion. The meandering structures of muscle fibers were observed in ACC group. The pERK expression in DRG was induced by ACC. These structural and physiological changes in ACC muscle were improved by stretch treatment.
Conclusion
These results indicated that muscle pain which caused by ACC are associate with meandering structural changes in muscles and stretching treatment can help improve muscle pain.

### [3P-136]

### Functional characteristics of myosin heavy chain isoforms in the sarcomere of extraocular muscle

*Mai Yamaguchi[1], Maki Yamaguchi[1], Naoya Nakahara[1], Shigeru Takemori[1] ([1]*Molecular Physiology, The Jikei University School of Medicine*)

To realize complex eye movements, extraocular muscle consists of various types of muscle fibers expressing specific isoforms of myosin heavy chain (MHC). Contractile characteristics of each fiber type would depend mainly on excitation-contraction coupling and contractile processes, in which motor head domain of MHC plays crucial roles. The head domain binds to $Ca^{2+}$-activated thin filament to form a crossbridge, which not only generate force and myofilament displacement but also activate the thin filament. We observed muscle force generation at low [MgATP] without $Ca^{2+}$, where self-regenerative formation of crossbridges progresses slowly. Chemically skinned fibers of rabbit were used. With gradual decrease in [MgATP], extraocular and psoas muscle started to develop tension at nearly ten times higher [MgATP] compared with soleus muscle. Electrophoretic analysis of MHC isoforms identified mainly fast type fibers in extraocular and psoas muscle, and slow type fibers in soleus muscle. The higher [MgATP] threshold for self-regenerative contractile crossbridge formation in fast type fibers suggested lower affinity of myosin heads to MgATP, or higher activating potency of the crossbridges.

### [3P-137]

### *In vivo* mitochondrial $Ca^{2+}$ dynamics during tetanic muscle contraction in male and female mice

*Yoshinori Tanaka[1], Ayaka Tabuchi[2,3], Hideki Shirakawa[3], Yutaka Kano[1,3] ([1]*Ctr. for Neuroscience and Biomedical Engineering, Univ. of Electro-Communications,* [2]*JSPS Research Fellowship,* [3]*Dept. Informatics and Engineering, Univ. of Electro-Communications*)

[Aims] Calcium ion ($Ca^{2+}$) dynamics in cytoplasm plays a central role in the regulation of muscle contraction. Although the involvement of intracellular $Ca^{2+}$ buffering by mitochondria has been implicated, *in vivo* mitochondrial $Ca^{2+}$ dynamics during muscle contractions remains unclear. The purpose of this study was to clarify mitochondrial $Ca^{2+}$ dynamics during muscle contraction in male and female *in vivo* mouse models. [Methods] The intact tibialis anterior muscle of adult C57BL/6 mouse was transfected with plasmid vector ($Ca^{2+}$ sensitive fluorescent protein, 4mtD3cpV) by electroporation. Tetanic isometric contractions (100 Hz, 3-second intervals, 50 contractions) were elicited by electrical stimulation. Mitochondrial $Ca^{2+}$ dynamics during contraction under anesthesia were evaluated by *in vivo* fluorescence imaging. [Results] Mitochondrial $Ca^{2+}$ levels increased with the onset of contraction and rapidly returned to resting levels after contraction. There was no significant difference between males and females in mitochondrial peak $Ca^{2+}$ elevation during contraction (males: 13.7 ± 3.2%, females: 18.2 ± 4.3%, vs pre-contraction). Tension curves in response to electrical stimulation were not different between males and females. [Conclusions] This study demonstrates that mitochondrial $Ca^{2+}$ levels increase without delay in response to muscle contraction. Furthermore, the findings revealed an absence of sex differences in mitochondrial $Ca^{2+}$ dynamics during tetanic contractions.

### [3P-138]

### Dorsomorphin, an AMP kinase inihibitor, inhibits skinned smooth muscle contraction

*Masaru Watanabe[1], Yasuyuki Naraki[1], Satoko Mihashi[1], Ryutaro Suma[1], Mizuka Okada ([1]*Laboratory of Physiology, Graduate School of Human Health Scinces, Tokyo Metropolitan University*)

AMP kinase is known to inhibit smooth muscle contraction through inhibition of myosin light chain kinase (MLC kinase) activity (e.g. Horman et al, J. Biol Chem 2008, 283;18505–12). On the other hand, it is still unclear whether dorsomorphin, an inhibitor of AMP kinase, has any effects on smooth muscle contractility. To clarify effects of dorsomorphin on contractile elements in smooth muscle, we investigated the agent effects on skinned (cell membrane permeabilized) smooth muscle contraction. Cell membrane and intracellular $Ca^{2+}$ store of small strips of taenia cecum from guinea pig were permeabilized with beta escin, and Ca ionophore A23187, respectively. Surprisingly, dorsomorphin at 10 micro M and higher significantly reduced $Ca^{2+}$ induced force development of skinned taenia cecum independent of $Ca^{2+}$ concentration. Since the inhibitory effects of dorsomorphin was also observed even when attenuation of MLC-phosphatase activity by tautomycin, a selective inhibitor of MLC-phosphatase, dorsomorphin seems to suppress contractile element activity though inhibition of activity of MLC kinase and/or myosin ATPase. COI:No

### [3P-139]

### The effect of physiologically active compound α on skeletal muscle depends on muscle fiber type

*Ryota Iyama[1], Kurogi Eriko[1], Hayashi Tatsuya[1], Egawa Tatsuro[1] ([1]*Kyoto University*)

Physiologically active compound α (Compound α) have many functions such as angiogenesis. However, the effect of Compound α on skeletal muscle has not been explored. We aimed to elucidate the effect of compound α on skeletal muscle by using transgenic mice (Tg) overexpressing compound α. We used Tg mice (n=16) and wild-type (WT) mice (n=17) at the age of twenty weeks. After anesthesia, gastrocnemius (GAS), extensor digitorum (EDL), and soleus (SOL) muscles were collected. We performed measurement of Compound α level, western blotting and immunohistochemical staining. The Compound α level in GAS was higher in the Tg. The contents of angiogenesis related markers, VEGF and angiopoietin-1, were not different between WT and Tg mice. CD31 expression was higher in the Tg-EDL compared to the WT-EDL. Myofiber type composition was not different between WT and Tg mice. In SOL and EDL, the mean of cross sectional area of muscle fiber (CSA) was not ideal but there was difference in fiber size distribution between WT and Tg mice. It shifted towards a bigger in the Tg-SOL, and a smaller in the Tg-EDL compared to WT. Considering the difference in the CD31 expression and CSA distribution between mice, Compound α can promote angiogenesis and has a hypertrophic effect on slow-twitch and an atrophic effect on fast-twitch muscles. This study suggests that the effect of Compound α on skeletal muscle depends on muscle fiber type.

### [3P-140]

### Peptidoglycan polysaccharide administration decreases skeletal muscle protein synthesis and muscle atrophy in male C57BL/6J mice

*Ryoga Kinoshita[1], Jung Jay[3], Karina Kouzaki[1,2], Yuki Tamura[1,2], Takaya Kotani[2], Koichi Nakazato[1,2] ([1]*Graduate School of Health and Sports Science, Nippon Sport Science Univ.,* [2]*Reseach Institute for Sport Science, Nippon Sport Science Univ.,* [3]*Nippon Sport Science Univ.*)

Purpose: Chronic diseases frequently accompany skeletal muscle atrophy. We recently showed that a single dose of peptidoglycan polysaccharide (PG-PS) administration induces whole-body inflammation and muscle atrophy in female rats. To further characterize drug-induced muscle atrophy, we investigated whether PG-PS induces muscle atrophy in male and female C57/BL6J mice.Methods: Twelve-week-old male and female C57BL/6J mice were randomly divided into the Control (n=10 for each sex) and the PG-PS groups (n=9 for each sex). PG-PS and Saline (50µg/g BW) were intraperitoneally injected. After 3 weeks of a single injection, lower limb muscles were excised to investigate muscle protein mass. Puromycin administration was used to measure muscle protein synthesis.Results: In the gastrocnemius (GM) of male mice, a baseline of protein synthesis in the PG-PS was significantly lower than that in Control. Wet weights of GM, a mixture of slow and fast fibers, were not by PG-PS treatment. PG-PS decreased the wet weight of the soleus, rich in slow fibers, in the male mice. In female mice, the same tendencies were not observed.Conclusion: PG-PS administration decreases skeletal muscle protein synthesis and muscle mass of slow twitch fibers in male mice.

### [3P-141]

### Effects of resistance training on cellular senescence in aging skeletal muscle

*Yung-Li HUNG[1], Ayami SATO[2], Yuka TAKINO[2], Akihito ISHIGAMI[2], Shuichi MACHIDA[1] ([1]*Juntendo University,* [2]*Tokyo Metropolitan Institute of Gerontology*)

Cellular senescence contributes to the process of multiple aging-related pathologies. Exercise training is considered to exert anti-aging effects. Resistance training improves skeletal muscle mass during aging. However, the effects of resistance training on cellular senescence in aging skeletal muscle is unclear. Twenty-two-month-old female rats were divided into sedentary and training groups. The rats in the training groups were trained to climb a ladder while bearing a load for a total of 20 training sessions over two months. After two months, the flexor hallucis longus muscles were collected and analyzed. The senescent cells were identified by using senescence-associated β-galactosidase staining. The results showed that the all and type IIx/IIb muscle fiber cross section area was significantly reduced in the 24-month-old sedentary group compared to 18-month-old sedentary group; however, cross section area increased after climbing training ($p<0.05$). In particular, the senescent cells were dramatically reduced in 24-month-old training group ($p<0.05$). These results suggest that resistance training has senolytic effect on aging female rat skeletal muscle.

### [3P-142]

### Sympathetic modulation of skeletal muscle contractility is altered by age and sex

*Harumi Hotta[1], Kaori Iimura[1], Nobuhiro Watanabe[1], Harue Suzuki[1] ([1]*Department of Autonomic Neuroscience, Tokyo Metropolitan Institute of Gerontology*)

We have recently showed that reflex excitation of muscle sympathetic nerves triggered by muscle contraction contributes to the maintenance of contractility in rat hindlimb muscles. We hypothesized that this mechanism declines with aging with decreasing muscle mass. In the present study, we examined and compared the regulatory potential of sympathetic nerves on skeletal muscle contractility in young adult (4-9 months old) and aged (32-36 months old) male and female rats. The tibial nerve was electrically stimulated to induce tetanic contraction of the triceps sure muscle. The tetanic force (TF) was measured with and without cutting or stimulating the lumbar sympathetic trunk (LST). The reduction in TF amplitude due to transection of the LST was 6.2% in aged rats, which was significantly reduced to about half of the 12.9% in young rats. When the male and female rats were analyzed separately, there was a significant difference between young and aged rats only in males, correlating the degree of muscle atrophy with the decrease in sympathetic contribution. However, there was no significant difference in the increased TF response to LST stimulation at 5-20 Hz. On the other hand, in aged rats, muscle tonus was often increased by LST stimulation alone. The results indicate that the feedback system between skeletal muscle and sympathetic nerves declined in aged rats in association with muscle atrophy, whereas sympathetically mediated increase in muscle tonus, independent of motoneuron activity, augmented. These changes in sympathetic modulation of hindlimb muscle contractility may involve in the reduction of skeletal muscle strength and smoothness of movement during senescence.

### [3P-144]

### Cell-cell fusion triggers the induction of voltage-induced $Ca^{2+}$ release in skeletal myogenesis.

*Taichiro Tomida[1], Yoshinori Mikami[1], Daisuke Ohshima[1], Satomi Adachi-Akahane[1] ([1]*Dept Physiol. Fac Med. Toho Univ.*)

The muscle-specific transmembrane proteins Myomaker and Myomixer were found to mediate cell-cell fusion between myoblasts and govern the irreversible differentiation process leading to skeletal myotube/myofiber formation. However, how cell-cell fusion contributes to muscle cell function remains elusive.This study aims to clarify the physiological significance of cell-cell fusion in differentiating skeletal muscle cells. C2C12 cells were used as an in vitro myogenesis model. Myomixer gene knockout cell line was established using CRISPR-Cas9 (Mymx-KO). Mymx-KO and Mymx-rescued KO cells were analyzed by RNAseq. Myogenic differentiation was induced by DMEM containing 2% horse serum. Voltage-induced $Ca^{2+}$ release (VICR) was evaluated by Cal520 fluorescence by applying electrical field stimulation (20V/cm, 1Hz, 25ms). We found that the VICR was suppressed in Mymx-KO but not in Mymx-rescued KO cells, suggesting that muscle cell requires cell fusion for induction of VICR. RNAseq results demonstrated that the genes related to $Ca^{2+}$ signaling as well as sarcomere/myofibril were upregulated in Mymx-resuced cells. However, the expression level of muscle-specific transcription factors such as MyoD and myogenin was unaltered regardless of whether Mymx was rescued or not. In conclusion, we found a novel regulatory linkage between Myomixer-mediated cell fusion and induction of VICR that is essential for EC coupling.

### [3P-143]

### Neutral sphingomyelinase activation associated factor (Nsmaf) involves in myogenesis inhibition via TNFalpha signaling

Jay Jung[1], Yuki Tamura[1], Karina Kouzaki[1], Takaya Kotani[1], *Koichi Nakazato[1] ([1]*Nippon Sport Science University*)

Chronic diseases induce elevations of systemic inflammatory cytokines and, as a result, skeletal muscle atrophy. Neutral sphingomyelinase activation associated factor (Nsmaf) reportedly regulates tumor necrosis factor-α (TNF-α)-induced signaling pathway. Since inflammatory TNF-α signaling inhibits skeletal muscle differentiation and protein synthesis, Nsmaf might play a role in inflammatory-induced muscle wasting. In the present study, we examined whether Nsmaf affects myogenesis and muscle protein synthesis by using murine C2C12 myoblasts.
In the growth phase, cells were maintained in DMEM and 10% FBS (growth medium: GM). For cell differentiation, the culture medium was switched to DMEM containing 1% HS (differentiation medium: DM). Small interfering RNA (siRNA) was used to induce Nsmaf gene Knock-down (KD). siRNA with a scrambled sequence was used as a negative control. Overexpression (OE) of Nsmaf was induced by transfection of Nsmaf expression plasmid vector. Western blotting was performed to examine protein expression levels. Laser scanning microscopy was used to investigate cell morphology. Even in the GM condition, Nsmaf KD induced upregulation of myogenic differentiation markers, such as myogenin and myosin heavy chain (slow). Nsmaf KD also reduced phosphorylated p38, which is one of the TNF-α signaling molecules. Nsmaf OE in the myoblast had little effect on muscle differentiation. In myotubes, protein synthesis was upregulated by Nsmaf KD. These results indicate that Nsmaf is a novel regulator of myogenesis, possibly via TNF-α signaling.

### Poster Presentation

[3P]
**Circulation**

March 16 (Thu.), 12:10 - 14:10, Poster Room

### [3P-145]

**Relationship between disease phenotype and mutant RyR2 channel activity in CPVT mouse models**

*Nagomi Kurebayashi[1], Masami Kodama[1], Takashi Murayama[1], Masami Sugihara[2], Masato Konishi[1], Aya Miura[3], Hajime Nishio[3], Yukiko U. Inoue[4], Takayoshi Inoue[4], Noguchi Satoru[4], Takashi Sakurai[1] (*[1]Dept Pharmacol, Juntendo Univ Sch Med, [2]Dept Clin Lab Med, Juntendo Univ sch Med, [3]Dept Leg Med, Hyogo Med Univ, [4]Natl Inst Neurosci, NCNP*)

The type 2 ryanodine receptor (RyR2) is a large $Ca^{2+}$ release channel on the sarcoplasmic reticulum and plays a pivotal role in cardiac excitation-contraction coupling. Gain-of-function (GOF) type RyR2 mutations are known to cause lethal ventricular arrhythmias such as catecholaminergic polymorphic ventricular tachycardia (CPVT), and more than 300 of pathogenic mutations have been reported to date. CPVT mutations are found in both familial and sporadic forms, and their onset age of arrhythmia varies from infancy to adulthood. CPVT is usually not associated with anatomical change in the heart, but CPVT cases with dilated cardiomyopathy (DCM) and left ventricular noncompaction (NVNC) have also been occasionally reported. To investigate the cause of these phenotypic differences, we compared phenotypes of multiple lines of mice harboring RyR2 mutations with different degrees of channel activation. We obtained in vivo and in vitro data such as electrocardiogram, $Ca^{2+}$ signals in the isolated cardiomyocytes, [$^3$H]ryanodine binding activity in the microsomes, and gene expression in the heart. Our data suggest good correlations between arrhythmia susceptibility, myocardial anatomic changes and RyR2 channel activity.

### [3P-146]

**Effects of oral minocycline on bone marrow inflammation in restraint stress-induced hypertension**

*Thu Van Nguyen[1,4], Ko Yamanaka[1], Keisuke Tomita[1], Jasenka Zubcevic[2], Sabine Gouraud[3], Hidefumi Waki[1] (*[1]Graduate school of Health and Sports Science, Juntendo University, [2]Department of Physiology and Pharmacology, University of Toledo, [3]College of Liberal Arts, International Christian University, [4]Department of Military Occupational Medicine, Vietnam Military Medical University*)

Minocycline (MIN) has an antihypertensive effect by attenuating both peripheral and central inflammation. The effect of restraint stress (RS) on the central nervous system through activation of myeloid inflammatory cells (ICs) has been reported. However, the effects of MIN on inflammatory factors (IFs) and ICs in the bone marrow (BM) remain unknown.Eighteen male Wistar rats were divided into a group with 3-week RS (ST), and another with stress and oral MIN treatment (SM), besides a control group (CO). To assess the gene expression of IFs, the mRNA was extracted from the BM. The plasma corticosterone level (PCL) was confirmed, and populations of ICs of the peripheral blood were investigated by flow cytometry.Blood pressure and PCL were significantly elevated by RS; however, MIN prevented these responses. Gene expression of inflammatory cytokines (Il1b, Tlr4) was significantly increased in the ST group, but not significantly different in the SM group, compared with the CO group. The number of ICs was also significantly higher in the ST group but not different in the SM group, compared with the CO group.Our findings suggest that MIN inhibited BM inflammation and BM-derived ICs in the peripheral blood. Further investigation is needed to ascertain whether these processes also regulate the central nervous system and blood pressure.

### [3P-147]

**Comparative analysis of myoelastic protein connectin to elucidate vertebrate heart evolution**

*Akira Hanashima[1], Misaki Kimoto[1], Momoko Ohira[1], Yuu Usui[1], Ken Hashimoto[1], Satoshi Mohri[1] (*[1]First Department of Physiology, Kawasaki Medical school*)

The blood pumping function of the heart is an important factor that constrains the activity and size of vertebrates. However, the mechanical properties of the ventricle have not been well investigated. Therefore, we focused on the spring molecule connectin, which determines the myocardial extensibility, and compared it among vertebrates. The results suggest that the elastic region of connectin in mammals and birds, which are homoiothermic and energy-consuming animals, is shorter than that in amphibians, and that the ventricle is less extensible. On the other hand, mammals and birds differ in the way they shorten the spring region, suggesting that the ancestors of mammals and birds, who needed to make their hearts less extensible about 250 million years ago, acquired similar traits through convergent evolution. The coronary circulation, a network of blood vessels within the myocardium of mammals and birds, is absent in amphibians, and blood flow, which is limited during diastole, is inhibited by excessive diastole, suggesting that the appearance of a coronary circulation may have led to the restriction of heart diastole. In mammals, the connectin spring region shortens with growth, suggesting that diastolic dysfunctional heart failure, which has attracted much clinical attention, may be the result of evolutionary changes in the mechanical properties of the ventricle.

### [3P-148]

**A novel calmodulin mutation associated with a severe form of catecholaminergic polymorphic ventricular tachycardia causes robust arrhythmogenicity due to a dominant facilitative effect on the cardiac ryanodine receptors**

*Jingshan Gao[1], Takeru Makiyama[1], Yuta Yamamoto[1], Takuya Kobayashi[2], Hisaaki Aoki[3], Asami Kashiwa[1], Tomohiko Imamura[1], Takanori Aizawa[1], Hai Huang[1], Hirohiko Kohjitani[1], Koichi Kato[4], Futoshi Toyoda[4], Naomasa Makita[5], Seiko Ohno[5], Nagomi Kurebayashi[2], Takashi Murayama[2], Minoru Horie[4], Takeshi Kimura[1] (*[1]Kyoto University, [2]Juntendo University, [3]Osaka Women's and Children's Hospital, [4]Shiga University of Medical Science, [5]National Cerebral and Cardiovascular Center*)

Background: Calmodulin (CaM) is an intermediate calcium-binding messenger protein and three genes (*CALM1-3*) encode identical CaM proteins in human. Recently, missense mutations in one of the three *CALM* genes are identified in patients with long QT syndrome (LQTS) and/or catecholaminergic polymorphic ventricular tachycardia (CPVT). CaM-related CPVT cases are rare and the underlying disease-causing mechanisms is still unclear.Objective: We sought to investigate the arrhythmogenic mechanism of CPVT caused by a novel mutation using human induced pluripotent stem cell (iPSC) models and biochemical assays. Methods and Results: We generated patient-derived iPSCs carrying a heterozygous mutation (c.136G>A, p.E46K) in the *CALM2* gene. To evaluate the effect of *CALM2*-E46K precisely, we utilized additional 3 iPSC lines: healthy control, E46K isogenic control and *CALM2*-N98S (LQTS&CPVT). In electrophysiological assays, *CALM2*-E46K cardiomyocytes (CMs) exhibited significantly frequent early/delayed afterdepolarizations and abnormal $Ca^{2+}$ waves compared to other lines. In $Ca^{2+}$ homeostasis analysis, *CALM2*-E46K CMs showed significantly larger $Ca^{2+}$ leaks from sarcoplasmic reticulum (SR) and smaller SR $Ca^{2+}$ storage than other cell lines. In addition, the biochemical analyses revealed that E46K-CaM showed enhanced binding affinity to cardiac ryanodine receptors (RyR2) than WT-CaM and significantly facilitated RyR2 channel activity. Conclusion: In this study, we for the first time established a CaM-related CPVT iPSC model which recapitulated the severe arrhythmogenic features resulting from dominant facilitative effect of E46K-CaM on RyR2.

### [3P-149]

**Withdrawn**

### [3P-150]

### Measurements of propagation rate of repolarization in a one-dimensional array model composed of human-ventricular cells

*Suzuka Enomoto[1], Yukiko Himeno[1], Ryosuke Hara[1], Hiroto Nomura[1], Akinori Noma[1], Akira Amano[1] ([1]*Ritsumeikan Univ.*)

Conduction of repolarization of cardiac action potentials (APs) was suggested because of the feedback cycle between the increase in the $I_{K1}$ conductance through repolarization and acceleration of repolarization rate at a single cell level. However, it has been difficult in experiments to separate the propagation from the movement of repolarization phase along multicellular tissues in the presence of spontaneous APs. The purpose of this study is to demonstrate propagating repolarization along the single cell array of cell models and measure its rate. We induced quasi-stable depolarization of the cell array by enlarging the $I_{NaL}$ amplitude combined with a decrease in the repolarization reserve, especially the $I_{Kr}$ conductance. The application of a repolarizing stimulus induced propagation of repolarization only when the stimulus amplitude was applied beyond a threshold $V_m$ level. The rate of repolarization was increased by magnifying the $I_{K1}$ conductance, but the rate was less by an order if compared with the conduction velocity of excitation, namely ~ 3 cm/s vs ~ 43 cm/s. The physiological significance of the propagation of repolarization was confirmed by modifying the artifactual preconditioning of the quasi-stable depolarization bit-by-bit toward the physiological one.

### [3P-151]

### Single cell transcriptome analysis of adventitial vasa vasorum remodeling in human aortic dissection.

*Kazuaki Yoshioka[1], Kenji Iino[2], Tomohiro Iba[1], Aya Matsui[1], Hirofumi Takemura[2], Hisamichi Naito[1] ([1]*Department of Physiology, Kanazawa University*, [2]*Department of Cardiovascular Surgery, Kanazawa University*)

Human aortic dissection (AD) is an acute disease characterized by hemorrhage into the tunica media of aortic wall, which can progress to vessel rupture. AD has several causes, but all symptoms present with common features, such as tissue remodeling. Vasa vasorum (VV), defined as vessels of vessels, are small blood vessels supplying nutrients and oxygen to the walls of large blood vessels. Although adventitial angiogenesis is a well-defined feature leading to lesion progression in AD, the cellular heterogeneity within endothelial cells (EC) has not been characterized. To understand the pathogenesis of AD, we aimed to comprehensively characterize the cellular composition of the adventitial VV in AD and to identify molecular alterations in each EC population. In this study, we performed single cell RNA (scRNA) sequencing on adventitial lesions of AD from patients. Clustering analysis of the transcriptional profiles from 7,633 cells identified 10 clusters representing 8 EC types: venous, artery, capillary, post capillary venule, tip cell, proliferating, immature and angiogenic ECs. Comparison with publicly available datasets of human normal aorta revealed differentiation of venous to proliferative immature angiogenic ECs was accompanied by hypoxic response associated with HIF1A upregulation. Our integrated analysis of the scRNA sequencing data with validated by pathohistological analysis could identify VV dysfunction in AD. The immature angiogenic structure was associated with the accumulation of carbonic anhydrase IX (CAIX), a mediator of hypoxia-induced stress response, in the adventitial VV. These data suggest that acute AD would progress from the adventitial region with VV dysfunction which can be valuable target for future therapeutic strategies.

### [3P-152]

### Therapeutic effect of Cordycepin, 3-deoxyadenosine derived from *Cordyceps militaris*, on experimental pulmonary hypertension

*GAOPENG LI[1], XIAODONG LI[1], Lin-Hai Kurahara[1], Kaori Ishikawa[2], Ryo Ishikawa[3], Kazufumi Nakamura[4], Katsuya Hirano[1] ([1]*Dept. Cardiovasc.Physiol., Fac. Med., Kagawa Univ.*, [2]*Dept.General Int. Med., Kagawa Univ. Hospital*, [3]*Dept.Diagnostic Pathology, Kagawa Univ. Hospital*, [4]*Dept.Cardiovasc. Med., Fac. Med., Okayama Univ.*)

Background and purpose: Pulmonary hypertension (PH) is characterized by vascular remodeling caused by augmented proliferation of pulmonary arterial smooth muscle cells (PASMCs). Human telomerase reverse transcriptase (hTERT), a catalytic subunit of telomerase complex, augments cell proliferation, especially when activated by cyclin-dependent kinase 1 (CDK1). Cordycepin is known to inhibit hTERT activity and cell proliferation. We therefore explored the therapeutic effect of cordycepin on PH. Method: Immunostaining was performed in lung tissues from PH and non-PH patients. The proliferation of cultured PASMCs derived from PH patients (PH-PASMCs) was analyzed with an MTT assay. Cordycepin was administered by drinking water to the rats of monocrotaline-induced PH model 11 days after monocrotaline injection. Results: The greater number of cells that co-express CDK1 and h-TERT phosphorylated at T249 were observed in PA of PH lung compared to non-PH lung. Cordycepin inhibited the proliferation of PH-PASMCs. Cordycepin significantly improved remodeling of PA and right ventricles and prolonged the survival of PH rats. Conclusion: CDK1-dependent hTERT activity is upregulated in PA of PH lung. Cordycepin could be a potential strategy for the treatment of PH by mitigating vascular remodeling.

### [3P-153]

### Application of the parameter optimization method to the selective $I_{Kr}$-block induced by E-4031 in the human induced pluripotent stem cell-derived cardiomyocytes (hiPSC-CMs)

*Yukiko Himeno[1], Yixin Zhang[1], Futoshi Toyoda[2], Hirohiko Kohjitani[3], Akinori Noma[1], Akira Amano[1] ([1]*Ritsumeikan Univ.*, [2]*Shiga Univ. Med. Sci.*, [3]*Kyoto Univ.*)

Because of a large variety in the action potential (AP) configuration of hiPSC-CMs, a cell-specific mathematical model is required for quantitative analysis of pharmacological blockage of the cardiac ion channels. We developed a parameter optimization (PO) method based on the pattern search algorithm. The mean square error (MSE) between the model output (AP waveform) and the experimental record (target AP) was minimized by the computer-based PO method. The cell-specific model was defined by a parameter set of ion channel conductances of a baseline hiPSC-CM model, which gave the minimum MSE between the model output and the two target AP waveforms recorded before and after the application of the $I_{Kr}$-blocker to a given hiPSC-CM. Before running the PO method, the output of the baseline model was first fitted to the control AP record by adjusting the individual model parameters manually. For justification of the use of this preliminary-adjusted model as the baseline model, we examined the distribution of MSE, which was calculated by randomizing individual ion channel conductances of the baseline model. We confirmed that there was a single local minimum present in the whole parameter space and that the use of this manually-adjusted parameter set greatly helped to reduce the computational time of individual runs of the PO method.

### [3P-154]

### PDGF receptor and Hippo signaling in pulmonary artery hypertension

*Aya Yamamura[1], Alamgir Hossain[1], Takahashi Rie[1], Iezaki Takashi[1], Hayashi Hisaki[1], Sato Motohiko[1] ([1]*Department of Physiology Aichi Medical University*)

Platelet-derived growth factor (PDGF) and its receptors play physiological and pathological roles in the proliferation, migration, and survival of vascular smooth muscle cells. Pulmonary arterial hypertension (PAH) is a rare disease characterized by pulmonary vascular remodeling. PAH remodeling is due to enhanced proliferation and reduced apoptosis of pulmonary arterial smooth muscle cells (PASMCs). The serum concentration and expression level of PDGF are known to be higher in PAH patients than in normal subjects. We previously found that the expression of calcium-sensing receptors (CaSRs) was upregulated in PASMCs from idiopathic pulmonary arterial hypertension (IPAH) patients. The upregulation mechanism was mediated by the activation of PDGF signals. In the present study, DNA microarray analysis using PASMCs from normal subjects and IPAH patients revealed Hippo signaling as a downstream pathway for PDGF receptors. Hippo signaling is known to be associated with cell life, proliferation, and differentiation. The expressions of YAP (a central molecule of Hippo signaling) and TEAD (a transcription factor) were upregulated in PASMCs from IPAH patients. The treatment with PDGF was increased the expressions of YAP, TEAD, and CYR61 (a fibrosis marker). These upregulations were reversed by siRNA knockdown of PDGF receptor β (PDGFRβ). In conclusion, Hippo signaling is activated following the stimulation of PDGF receptors, contributing to the development of PAH remodeling.

### [3P-155]

### The importance of sympathetic innervation for the postnatal heart development

*Takuro Numaga-Tomita[1], Hiroyuki Kawagishi[1], Tsutomu Nakada[2], Mitsuhiko Yamada[1] ([1]*Shinshu Univ., Sch. Med., Dept. Mol. Pharmacol.*, [2]*Shinshu Univ., Res. Ctr. Adv. Sci. Tech.*)

The sympathetic nervous system is important for the adult heart to adapt to the changes in environmental stresses. Besides that, it has been suggested that sympathetic innervation affects postnatal development of the heart. Several lines of evidence have indicated that sympathetic innervation changes the modes of cardiomyocyte growth from proliferation to hypertrophy and the membrane excitability by altering ion channel activities. However, most of these findings were obtained from the in vitro co-culture of neonatal cardiomyocytes and sympathetic neurons. Therefore, whether it is also the case in vivo remains largely unknown. In this study, we have analyzed the effect of chemical sympathetic denervation on the postnatal murine heart development before weaning (at postnatal day (P) ~20) by treating newborn pups (P0-1) with 6-hydroxydopamine (6-OHDA). Immunofluorescence staining of tyrosine hydroxylase, a marker of sympathetic neurons, demonstrated that the sympathetic neurons were almost completely eliminated in P7 to 21 mice hearts by 6-OHDA treatment. 6-OHDA treatment significantly reduced left ventricular contractility compared with the control at P21. However, neither L-type calcium channel activity nor calcium transients evoked by field stimulation were unchanged in the isolated cardiomyocytes from P21 mice hearts. Fluorescence imaging of T-tubule structures demonstrated that cardiomyocytes from 6-OHDA-treated mice hearts have reduced regularity of T-tubule structure. Furthermore, the protein abundance of alpha-SMA, a vascular smooth muscle cell marker, was significantly reduced in 6-OHDA-treated mice hearts at P21. These results suggest that sympathetic innervation is likely to play critical roles in the postnatal maturation of cardiomyocytes and coronary circulation.

### [3P-156]

### Development of a state transition model to reproduce the inhibitory effect of E-4031 on hERG channels

*Yuna Nakanishi[1], Azumi Sagehashi[1], Hiroto Nomura[1], Yukiko Himeno[1], Futoshi Toyoda[2], Akira Amano[1] ([1]*Ritsumeikan Univ.*, [2]*Shiga Univ. Med. Sci.*)

Inhibition of the hERG channel by drugs was analyzed by comparing the behavior of $I_{Kr}$ obtained from different pharmacological experiments and reproducing it by developing mathematical models. Firstly, we implemented the effect of E-4031 stimulation to the $I_{Kr}$ model, which was implemented in the human ventricular myocyte model (Himeno *et al*., 2015), referring to experimental data reported by Kamiya *et al*., 2006. Secondly, we implemented a dynamic hERG model (Li *et al*., 2017), which was incorporated in O'Hara & Rudy's human ventricular myocyte model (O'Hara *et al*., 2011) and reproduced various drug effects on $I_{Kr}$, for comparison. Kamiya *et al*.'s experimental data suggested the involvement of two time constants. Therefore, our $I_{Kr}$ model was constructed by dividing the inhibitory step into two steps; the initial primed state when the drug acted on the channel and the subsequent step in which the channel was actually blocked. As a result, we were able to construct a model that reproduces the time course of experimental data obtained in guinea pigs. On the other hand, although the state transition of the dynamic hERG model by Li *et al*., could explain the drug-trapping property of E-4031, it would not reproduce the first ~20-second time delay before the drug action onset. The state transition and time course of two different models of $I_{Kr}$ blockade after the E-4031 application will be demonstrated in detail.

### [3P-157]

### Phenotypic analysis of pulmonary vein and atrial muscle-specific Pitx2c conditional knockout mice

*Daiki Seya[1], Takahiro Inoue[1], Shunsuke Baba[1], Toru Akaike[1], Susumu Minamisawa[1]
([1]*Dept. of Cell Physiology, The Jikei University School of Medicine*)

Pulmonary hypertension (PH) is a progressive, intractable disease that causes cardiopulmonary dysfunction due to elevated pulmonary arterial pressure. The pathogenesis of PH varies greatly depending on whether the primary lesion is upstream or downstream of the pulmonary capillaries, and the latter, post-capillary PH (pc-PH) caused by pulmonary vein stenosis/obstruction or left heart disease, currently has not an appropriate animal models to study its pathophysiology and pharmacological effects. In this study, we generated conditional knockout (cKO) mice of Pitx2c, a transcription factor essential for pulmonary vein formation, using Sarcolipin-Cre, in which Cre recombinase is specifically active in pulmonary veins and atrial muscle. The cardiac phenotype in Pitx2c-cKO mice at 8 week-old showed a significant increase relative tissue weights of the right atrium, right ventricle and lungs. Echocardiograms of the same aged cKO mice showed prominent enlargement of the right ventricle, dilated pulmonary artery valve annulus diameter, and decreased left ventricular inner diameter, volume, and mass. A few cKO mice also had elevated right ventricular pressure on cardiac catheterization. These primary findings suggest that the right heart system is overloaded in Pitx2c-cKO due to elevated pulmonary venous pressure and may serve as a model for pc-PH pathology.

### [3P-158]

### Verification of antiarrhythmic drugs using multiple lines of RyR2 mutant mice

*Masami Kodama[1], Nagomi Kurebayashi[1], Takashi Murayama[1], Masami Sugihara[2], Masato Konishi[1], Aya Miura[3], Hajime Nishio[3], Yukiko U. Inoue[4], Takayoshi Inoue[4], Satoru Noguchi[4], Takashi Sakurai[1] ([1]*Dept Pharmacol, Juntendo Univ Sch Med*, [2]*Dept Clin Lab Med, Juntendo Univ Sch Med*, [3]*Dept Leg Med, Hyogo Med Univ*, [4]*Natl Inst Neurosci, NCNP*)

Type 2 ryanodine receptor (RyR2) is a $Ca^{2+}$-release channel on the sarcoplasmic reticulum that plays a central role in cardiac excitation-contraction coupling. Gain of function (GOF)-mutations in RyR2 are related to lethal ventricular arrhythmias, such as catecholamine-induced polymorphic ventricular tachycardia (CPVT). Because conventional antiarrhythmic drugs are not sufficiently effective for treatment of CPVT, development of more effective drugs is needed. To do this, it is necessary to establish a drug evaluation method using model mice. In this study, we employed two electrocardiogram (ECG)-based methods to verify effects of antiarrhythmic drugs, i.e., prevention and termination of arrhythmia, using multiple lines of mice harboring RyR2 mutants. To test preventive effect, β-adrenergic stimulation-induced arrhythmias were measured with and without pretreatment of the drugs under anesthesia using mice carrying mild RyR2 mutation. To evaluate arrhythmia termination effect, spontaneous arrythmias in mice harboring severe RyR2 mutation were measured after treatment with drugs. These methods enable quantitative comparison of the efficacy of antiarrhythmic drugs, proving to be useful for development of antiarrhythmic drugs.

### [3P-159]

### Comparison of Ca2+ transients in cardiomyocytes isolated from quails, snakes and rats

*Ogura Yuhei[1], Sugita Shukei[1], Nakamura Masanori[1], Ujihara Yoshihiro[1] ([1]*Nagoya Institute of Technology*)

Mammalian cardiomyocytes have T-tubule membranes that facilitate rapid changes in Ca2+ concentration, but other vertebrates do not have T-tubule membranes. Although the heart rates of reptiles, amphibians, and fish, which lack T-tubule membranes, are slower than those of mammals, exceptionally, the heart rates of birds are as fast as those of mammals. In this study, we measured Ca2+ transients in cardiomyocytes isolated from avian quails, mammalian rats, and reptilian snakes to gain insight into the mechanism of the faster heart rate in birds, despite the absence of T-tubule membranes. Cardiomyocytes isolated from quails and snakes were significantly narrower than those isolated from rats. When Ca2+ transients in the entire cardiomyocytes were measured using Fura-2 AM, the rate of increase in Ca2+ concentration was significantly slower in quails than in rats, but significantly faster than in snakes. In contrast, the rate of decrease in Ca2+ concentration was significantly faster in quails and snakes than in rats, suggesting that birds and reptiles, which lack T-tubule membranes, may have superior Ca2+ efflux capacity compared to mammals. The enhanced Ca2+ influx capacity of birds during their evolution from reptiles may be the key to their fast heart rates.

### [3P-160]

### Comparative evaluation of passive mechanical properties of ventricles in different habitats using Anura and Serpente.

*Megumi Ito[1], Vu Dang Nguyen[2], Yuhei Ogura[1], Shukei Sugita[1], Masanori Nakamura[1], Yoshihiro Ujihara[1] ([1]*Nagoya Inst. Tech.*, [2]*Univ. of Utah*)

The hearts change their structure and properties in response to changes in mechanical loading. During vertebrate evolution, the transition from aquatic to terrestrial environments may have driven dramatic changes in the structure and properties of the heart, because the effect of gravity on blood circulation is increased. Here, we first investigated the passive mechanical properties and histology of the ventricles of three species of Anura (frogs and toads) with different habitats, *X. laevis* (aquatic), *P. nigromaculatus* (semiaquatic), and *B. j. formosus* (terrestrial). Pressure-loading tests showed that the ventricles were stiff in the order of *B. j. formosus*, *P. nigromaculatus* and *X. laevis*. Histological analysis revealed that *P. nigromaculatus* and *B. j. formosus* had a thick layer of compact myocardium. The second-harmonic generation (SHG) light observation showed that *B. j. formosus* had enrichment of the collagen fibers. These results suggest that the ventricles of Anura become stiff in the process of terrestrialization by thickening of ventricular wall and increasing in collagen. To further test whether the transition from aquatic to terrestrial life stiffened the ventricles, we examined the ventricles of a terrestrial reptile Serpente (*E. quadrivirgata*), which differs dramatically from Anura in body morphology and size. Compared to the ventricles of Anura, the ventricles of *E. quadrivirgata* were asymmetrical and elongated, but were similar in stiffness. These results suggest that the transition from aquatic to terrestrial environment may have been a critical trigger for ventricular stiffening during vertebrate evolution.

### [3P-161]

### CR9 regulates transcription expression as an enhancer of the atrial and brain natriuretic peptide gene in cardiomyocytes.

*Hyejin Jo[1], Ken Matsuoka[2], Hijiri Inoue[1], Osamu Tsukamoto[1,2], Seiji Takashima[1,2]
([1]*Graduate School of Frontier Biosciences Osaka University, Medical Biochemistry*, [2]*Graduate School of Medicine Osaka University, Medical Biochemistry*)

[Backgrounds] Enhancer is a distal-acting *cis*-element that regulates specific gene transcription in a tissue- and temporal-specific manner under developmental stages and pathological conditions. We previously identified a stress-responsive putative enhancer region CR9 (650-bp) from nine conserved non-coding regions (CR1 to CR9) upstream of *Nppb* gene in cardiomyocyte. However, it remains to be solved whether endogenous CR9 element really works as an enhancer and which kinds of transcription factors are involved. [Methods and Results] We systematically targeted these CRs individually using CRIPSR activation (CRISPRa) that employs the nuclease-dead Cas9 protein fused to a transcriptional activator VP64 and sequence-specific single guide RNAs (sgRNAs) to mediate activation on target regions. *Nppb* exhibited significant upregulation upon CRISPRa on the CR9 in cultured rat neonatal cardiomyocytes. In addition, combined targeting on either CR6, CR7, or CR8 with CR9 synergistically upregulated *Nppb* expression. Next, using the biochemical and bioinformatics analysis, we identified several cardiac-specific transcription factors that upregulate *Nppb* expression by binding the CR9 element.[Conclusion] The endogenous CR9 element acts as a hub enhancer for *Nppb* gene transcription in concert with other CR elements and several transcription factors in cardiomyocytes.

### [3P-162]

### Localization of tissue macrophages during the closure of the ductus arteriosus

*Mei Wang[1], Norika Liu[1,2], Susumu Minamisawa[1], Atsushi Nakano[1,2] ([1]*The Jikei University School of Medicine,* [2]*California University Los Angeles*)

ObjectivesOur previous study showed that tissue macrophages derived from hemogenic endocardial cells are essential for the valve remodeling during embryogenesis, suggesting that the local transient hematopoiesis is not about supplementing blood to the systemic circulation but about the local tissue formation. It has been reported that aortic endothelial resident macrophages (MacAIRs) are a unique population of macrophages that seed the aorta shortly after birth. The aim of the entire study is to address whether locally derived macrophages are involved in the remodeling of the ductus arteriosus (DA). The first step is to investigate the presence of macrophages during DA closure, which is required a comparison with the origin of MacAIRs.MethodsIn order to clarify the presence of tissue macrophages during DA remodeling, we investigated DA in wildtype ICR mice neonates of different postnatal breathing time windows (0min, 30min, 2hr, 0.5day, 1day, 1.5day, 2day). Histological immunofluorescent (IF) analysis using CD68, ERG, α-SMA was performed. Results IF analysis revealed that tissue macrophages of DA from earlier time points (0min ~ 0.5day) mainly resident outside of vascular and rarely exist in the smooth muscle layer. After 1day of birth, some macrophages appear to reside in the intimal region of DA.Discussion The appearance of macrophages in the intima of the DA from 1day postnatal suggested that they may be equivalent to $Mac^{AIR}$. We are currently examining whether these macrophages originate from Nkx2-5 lineage. Although further investigation is needed, analyses using early postnatal time points suggested that macrophages present in the intima of the DA may have migrated from outside the vessel.

### [3P-163]

### Withdrawn

### [3P-164]

### Electrophysiological analysis of energy metabolism for mouse sinus node firing rate

*Mayu Takatani[1], Takato Horimoto[1], Tomoe Ueyama[1,2], Shu Nakao[1,3], Teruhisa Kawamura[1,3] ([1]*Coll. Life Sci., Ritsumeikan Univ.,* [2]*Res. Org. Sci. Tech., Ritsumeikan Univ.,* [3]*Ritsumeikan Global Innov. Res. Org.*)

The sinus node is indispensable as the primary pacemaking site in the heart. However, little is known about what energy sources are required to drive sinus node function, whereas contracting cardiomyocytes demand high-rate energy production predominantly from fatty acid and ketone metabolism. In this study, we aim to investigate the energy source of sinus node firing as a heart rate determinant. To analyze the effect of metabolic processes on spontaneous firing of the sinus node, right atrial preparations were freshly dissected from adult mouse hearts, and perfused with physiological Tyrode's solution for subsequent electrophysiological measurements. A right atrial preparation is denervated, thus it is sufficient to analyze intrinsic pacemaking function. We confirmed beating rate remaining over 60 minutes in a perfusion chamber, and it was decreased with hypoxic Tyrode's solution. 2-deoxyglucose, a glucose analogue blocking glycolysis, only slightly affected the beating rate. Rotenone, an ATPase inhibitor, markedly reduced beating rate. In addition, our transcriptomic analysis showed that lipid metabolism-related gene categories were upregulated in mouse sinus node tissues. These results suggest that sinus node firing largely depends on aerobic metabolism driven by fatty acid rather than glycolysis as observed in contracting cardiomyocytes.

### [3P-165]

### Development of a three-dimensional layered cell sheet with elastic fiber formation

*Shota Futagami[1], Shota Tanifuji[2], Takashi Nakamura[2], Yuji Naito[2], Tomoyuki Kojima[2,3], Takuya Naruto[4], Michiya Matsusaki[5], Utako Yokoyama[2] ([1]*The 4th year medical student, Tokyo Medical Univ.,* [2]*Department of Physiology, Tokyo Medical Univ.,* [3]*Department of Obstetrics and Gynecology, Yokohama City University School of Medicine,* [4]*Kanagawa Children's Medical Center,* [5]*Department of Applied Chemistry, Graduate School of Engineering, Osaka Univ.*)

### [3P-166]

### Short term hypergravity alters cytoskeleton organization in HUVECs in response to the following microgravity.

*Moe Shigeishi[1], Susumu Minamisawa[1], Hiroki Bochimoto[1] ([1]*The Jikei University School of Medicine, Division of Aerospace Medicine, Department of Cell Physiology*)

### [3P-167]

### Mechanism of changes in $Ca^{2+}$ dynamics induced by activation of $Ca^{2+}$ handling proteins in the human ventricular cell model

*Miyu Horino[1], Akari Sanechika[1], Atsuhiro Nakagawa[1], Yukiko Himeno[1], Akira Amano[1] ([1]*Ritsumeikan Univ.*)

### Poster Presentation

[3P]
**Endocrine**

March 16 (Thu.), 12:10 - 14:10, Poster Room

### [3P-168]

### Role of Nrf2 in lipotoxic impairment of glucose-stimulated insulin secretion from pancreatic β-cells

*Yuta Kato[1], Daisuke Yokoyama[1], Yuri Yoshimi[1], Kazuma Sugimoto[1], Eri Mukai[1]
([1]*Ritsumeikan University*)

In type 2 diabetes, glucose-stimulated insulin secretion (GSIS) from pancreatic β-cells is impaired. Oxidative stress due to excessive production of reactive oxygen species (ROS) associated with glucotoxicity and lipotoxicity causes the decrease; however, the detailed mechanism has not been clear. Nuclear factor erythroid 2p45-related factor 2 (Nrf2), a transcription factor that regulates genes related to antioxidant, dissociates from regulatory factors such as Kelch-like ECH-associated protein 1 (Keap1) upon oxidative stress and then translocates into the nucleus. In the present study, we investigated the involvement of Nrf2 in the lipotoxic effect on GSIS.
0.5 mM palmitate exposure for 48 hours decreased GSIS and increased intracellular ROS levels in INS-1 cells, a rat pancreatic β-cell line. Palmitate exposure decreased Nrf2 mRNA levels for 6~48 hours and Keap1 mRNA levels for 12 hours. The decrease in GSIS by palmitate exposure was completely restored by ROS scavengers (ascorbic acid + α-tocopherol). Knockdown of Nrf2 by siRNA in INS-1 cells decreased GSIS and increased intracellular ROS levels. The decrease in GSIS by Nrf2 deficiency was only partially restored by ROS scavengers.
These results indicate that decreased GSIS under lipotoxicity is attributed to increased ROS and suggested that Nrf2 regulate GSIS by mechanisms other than the regulation of antioxidant activity.

### [3P-169]

### The effect of the lactational exposure to perfluorooctane sulfonate during aging period

*Ayane Ninomiya[1], Izuki Amano[1], Asahi Haijima[2], Noriyuki Koibuchi[1] ([1]*Dept. Integrative Physiology, Grad. Sch. Medicine, Gunma Univ.*, [2]*Laboratory for Environmental Brain Science, Faculty of Human Sciences, Waseda University*)

The incidence of developmental diseases has been increasing. As one of the reasons, the recent studies has pointed out the perinatal exposure to the environmental chemicals. We previously reported that the lactational exposure to perfluorooctane sulfonate (PFOS) caused the impairment in the cognitive and motor functions in adult male mice. However, little is known about the perinatal PFOS exposure in aged subjects. In the present study, we examined how the lactational exposure to PFOS affects cognitive function, anxiety, and social behavior using aged male mice that were exposed to PFOS (1 mg / kg body weight) during the lactational period. In the visual discrimination test, both control and PFOS groups showed a decrease in the learning curve. There is no difference in % correct between groups. In the object location test, there are no changes in both short- and long-term memory. In the object recognition test, long-term memory was attenuated in the PFOS group. The test battery for anxiety (elevated-plus maze test, light and dark chamber test, marble burying test) detected no difference between groups. In the three-chamber social interaction test, PFOS-exposed mice showed an avoidance to interact with a novel mouse, which was not observed during the young-adult period. These results indicate that the PFOS mainly affects social activity during aging.

### [3P-170]

### Role of the nuclear receptor corepressor 1 (NCoR1) and the silencing mediator of retinoid and thyroid hormone receptors (SMRT) on central nervous system

*Izuki Amano[1], Ayane Ninomiya[1], Reika Kawabata[2], Ritter Megan[3], Anthony Hollenberg[3], Noriyuki Koibuchi[1] ([1]*Department of Integrative Physiology Gunma University Graduate School of Medicine*, [2]*Division of Integrated Oncology Research, Initiative for Advanced Research, Gunma University*, [3]*Division of Endocrinology, Weill Cornell Medicine*)

The nuclear receptor corepressor 1 (NCoR1) and the silencing mediator of retinoic acid and thyroid hormone (SMRT) play critical roles in nuclear receptor(e.g., thyroid and retinoic acid receptors) action as corepressors. Although they are highly homologous and have similar nuclear receptor interaction domains, they have different roles in each organ. Recently, *de novo* genetic variants in nuclear corepressors are found in pediatric patients with neurodevelopmental disorders. Thus, we generated mouse models to understand the role of NCOR1 and SMRT in the central nervous system. We used mice with conditional NCoR1 or SMRT ($NCoR1^{lox/lox}$ or $SMRT^{lox/lox}$) alleles in combination with mice that express *Cre* recombinase in a neuronal specific fashion (Snap25-Ires2-Cre). We found that hypoactivity, social deficits, and mild anxiety behaviors in neuronal specific NCoR1 or SMRT KO mice. In addition, NCoR1 KO mice showed high learning abilities. Next, we performed RNA-sequencing analysis with amygdala from postnatal day 21 to investigate gene expression mediated by NCoR1 and SMRT. We found that 449 genes were upregulated by SMRT deletion, whereas only 8 genes were regulated by NCoR1 deletion. Overall, our data demonstrate for the first time that NCoR1 and SMRT have separate functions in the central nervous system.

### [3P-171]

### Triiodothyronine and estradiol trigger matrix mineralization of MC3T3-E1 through overlapped pathway

*Hiraku Suzuki[1], Sumiyasu Ishii[1], Izuki Amano[1], Noriyuki Koibuchi[1] ([1]*Gunma University*)

It is reported that both triiodothyronine ($T_3$) and estrogen play important roles in bone metabolism. The decrease in these hormones increases the risk of osteoporosis and bone fracture. We previously reported that estradiol induced the expression of calcium-binding proteins including osteocalcin and caused matrix mineralization in MC3T3-E1, osteoblast-like cell line, in lipophilic-hormone free serum-containing culture media. In this study, we examined the mechanism of $T_3$ action on matrix mineralization of MC3T3-E1. $T_3$ induced mineralization with an increase in osteocalcin expression, as shown with estradiol. Such $T_3$ -induced mineralization was inhibited by PMA, a PKC activator. This was also observed with estradiol. Furthermore, both $T_3$- as well as estradiol-mediated pathways is independent from the alkaline-phosphatase (ALP) mediated pathway, because inhibition of ALP activity did not alter the $T_3$- or estradiol-mediated mineralization. These results indicate the critical involvement of these hormones on bone calcification through a novel pathway. Our finding may contribute developing a novel therapeutic strategy for osteoporosis.

### [3P-172]

### The dissociation between the maternal plasma concentration and the placental production pattern of Prl3b1 (mouse placental lactogen II) during late pregnancy.

*Taku James Sairenji[1], Shinnosuke Masuda[1,2], Yuki Fujiwara[1], Noriaki Shimokawa[1,3], Noriyuki Koibuchi[1] ([1]*Dept Intgr Physiol, Med Grad Sch, Gunma Univ, Japan*, [2]*Epigenetics and Metabolism, IMCR, Gunma Univ, Japan*, [3]*Dept Nutr, Takasaki Univ Health Welf, Japan*)

Both pituitary prolactin (PRL) and placental prolactin are essential hormone for mouse reproduction, from maintenance of pregnancy to milk production. One of the most well studied placental prolactin is Prl3b1 (traditionally called mouse placental lactogen II; mPL-II). Our first finding is that there is a significant decrease of maternal plasma concentration of Prl3b1 around 12:00 of gestational day 19(G19). This result contradicts previous studies and our own data which focused on mRNA level of Prl3b1 which show a high expression level until parturition. There are more than 10 members of the placental prolactin family expressed in the mouse placenta during late pregnancy, however there is a lack of study on the quantification on protein production. Thus, we performed a proteome analysis on the whole mouse placental tissue during the 24-hour frame the Prl3b1 decrease, to see the changes of the prolactin quantity. Here we show the results of comparative quantitative analysis by mass spectrometry between the placenta from G18 20:00 to G19 20:00. We detected 7 proteins that significantly increased and 32 proteins that significantly decreased. Prl3b1 did not decrease in the protein quantity. As a conclusion, we report a dissociation between the maternal plasma concentration of Prl3b1 and the production in the placenta during late pregnancy.

### Poster Presentation

[3P]
**Environmental physiology**

March 16 (Thu.), 12:10 - 14:10, Poster Room

### [3P-173]

**Impact of heat stress and exercise load on biological measurement**

*Takashi Maruyama[1], Yoichi Ueta[1] ([1]*University of Occupational and Environmental Health, Japan*)

Heatstroke is caused by the body overheating, dehydration, hyponatremia and poor health conditions. Sometimes it will be a fatal state, so the prevention for heat stroke is important social issue. Usually as a result of exercise or working in high temperature environment, the risk of heat stroke rises. A total of 19 healthy male aged 21–42 years old performed a treadmill exercise load test in an experimental chamber. The environment adjusted to two conditions; high risk condition (temperature 35°C and humidity 50%) and normal condition (temperature 25°C, humidity 50%). During the exercise load test, electrocardiogram and core body temperature (rectal temperature) were continuously measured. The measurement of body weight, urine and blood test were performed at pre and post the exercise load test. The heart rate and core body temperature increased during the exercise and the core body temperature exceeded 38.5°C in 15 cases. Weight change of 1.5% or more was observed in 11 cases, which is an index of dehydration. Analysis of blood and urine tests revealed significant changes in aldosterone, HANP at normal condition (25°C), and Significant changes were observed in total protein, albumin, plasma osmolality, antidiuretic hormone, and urinary aquaporin at high risk condition (35°C). Changes in each index are thought to be caused not only by heat stress, but also by various factors such as exercise, dehydration, and changes of blood pressure.

### [3P-174]

**Discrepancy in movement preparations during hyperthermia due to two different stimulus presentations**

*Keiko Yamamoto[1], Manabu Shibasaki[1] ([1]*Nara Women's Univ.*)

Voluntary force production and neuromuscular function are reduced in a hyperthermic condition, but the neural mechanism remains unclear. We investigated the effect of hyperthermia on the neural activity associated with motor preparation using the electroencephalographic event-related potentials. Two stimulus presentation methods were selected: a method of inducing movement-related cortical potential (MRCP) with self-initiated movement, and contingent negative variation (CNV) with externally-triggered movement. Healthy young volunteers performed a trial before (Pre) and during whole-body heat stress (HS). In the MRCP trial, the amplitude of Bereitschaftspotential (BP, P=0.834) and negative slope (NS', P=0.859) did not change between Pre and HS at any of the electrodes (Cz, Pz, C3, and C4). On the othter hand, the amplitudes of CNV in the late (from 1.5 to 2 s) phases was significantly smaller during HS than Pre at Cz, Pz (P<0.005), and C4 (P<0.05). Neural activity for self-initiated movement is maintained even in the presence of hyperthermia, but that for externally-triggered movement may be inhibited by hyperthermia-induced central fatigue because of cooperation with other brain areas, such as the prefrontal cortex.

### [3P-175]

**Elucidation of the daily torpor mechanism in *Suncus murinus.***

*Yuuki Horii[1,2], Kanako Okadera[1], Shingo Miyawaki[3], Takahiko Shiina[1], Yasutake Shimizu[1] ([1]*Laboratory of Physiology, Joint Department of Veterinary Medicine, Faculty of Applied Biological Sciences, Gifu University,* [2]*Institute for Glyco-core Research (iGCORE), Gifu University,* [3]*Laboratory of Surgery, Joint Department of Veterinary Medicine, Faculty of Applied Biological Sciences, Gifu University*)

The behavior of mammals that significantly reduces their metabolism and lowers their body temperature for several hours within a day is called "daily torpor". Elucidating the mechanism of daily torpor is expected to have medical applications. The house musk shrew (*Suncus murinus*), a mammal that has temperature homeostasis, can perform the daily torpor. The purpose of this study is to determine the conditions under which the shrew will torpor. When shrews were kept at ambient temperatures above 24°C, most animals did not enter daily torpor. However, when the ambient temperature was lowered below 20 °C, all shrews exhibited torpor. In addition, shrews that were lowered in steps from 24 °C to 8 °C in ambient temperature entered the torpor even when returned to a room maintained at 24 °C. This study demonstrates that torpor behavior was observed even without fasting or short photoperiod changes in the shrew, which animal may be a suitable model animal for further elucidating the mechanism of torpor. The elucidation of the mechanism of torpor using *Suncus murinus* may be useful for inducing artificial hibernation states in various species, including humans.

### [3P-176]

**Hypothalamic melanocortin mechanism of age-related decline in brown adipose tissue thermogenesis**

*Manami Oya[1], Kazuhiro Nakamura[1] ([1]*Nagoya University Graduate School of Medicine*)

Thermogenesis in brown adipose tissue (BAT) substantially contributes to whole-body metabolism in rodents and humans. Capability of BAT thermogenesis declines with age, which is a cause of obesity in middle age. However, the mechanism of age-related decline in BAT thermogenesis is unknown. To elucidate the mechanism, we explored any age-related alteration of the melanocortin 4 receptor (MC4R), which mediates leptin–melanocortin satiety signaling to prevent obesity. Immunohistochemistry with an anti-MC4R antibody we generated revealed that MC4R proteins are localized to a specific neuronal organelle in the rat dorsomedial hypothalamus and paraventricular hypothalamic nucleus, which control BAT thermogenesis and food intake. Interestingly, the MC4R-bearing organelle gradually disappeared with age. Specific deletion of the MC4R-bearing organelle using a genetic approach in young MC4R-Cre rats blunted BAT thermogenesis in response to injection of an MC4R agonist or skin cooling, and finally increased adiposity and body weight. These results indicate that age-related loss of MC4R-bearing organelle in hypothalamic neurons reduces the capability of BAT thermogenesis, leading to the development of obesity.

### [3P-177]

**Stimulation of tail suspension activates hypothalamus oxytocin neurons in rats**

*Hirofumi Hashimoto[1,2], Hajime Hoshiai[2], Yumi Chiba[2], Takashi Mizushima[2], Naohiko Anzai[1] ([1]*Dept. Pharmacology, Chiba Univ. Graduate Sch. Med.,* [2]*Dept. Rehabilitation, Dokkyo Medical Univ.*)

Oxytocin (OXT) is a well-known neuropeptide related with uterine contractions and milk ejection reflex. Previous study showed that plasma OXT level decreased and administration of OXT induced muscle regeneration in aging mice. We showed that the fluorescence intensity of mRFP1 increased in aging OXT-monomeric red fluorescent protein 1 (mRFP1) transgenic rats. In this study, we investigated effects of tail-suspended (TS) stimulation in central OXT secreting neurons by Fos-like immunoreactive (LI) activity in rats. Fos-LI cells significantly increased in the supraoptic (SON) and paraventricular nuclei (PVN) in TS group, compared with control group. In double immunohistochemistry for Fos and OXT, OXT-LI cells expressing Fos-LI also significantly increased in the SON and PVN in TS group, compared with control group. These results suggested that the stimulation of TS activates central oxytocin neurons in rats.

### [3P-178]

### Estrogen establishes the sex difference in the rat preoptic area: Involvement of actin dynamics for cell migration

*Tomohiro Hamada[1], Yasuo Sakuma[2] ([1]*Clinical Departments Laboratory, Nippon Medical School,* [2]*Nippon Medical School*)

The volume of the sexually dimorphic nucleus of the preoptic area (SDN-POA) is larger in male rats than in females. It seems that incomplete differentiation of this nucleus, as well as the other sexually dimorphic nucleus, causes gender dysphoria. However, the mechanism for the establishment of sexual dimorphism remains largely unknown, except that estrogen aromatized from androgen during perinatal period cause the masculinization of the nucleus. Transgenic rats were generated that express EGFP under the control of an estrogen receptor (ER) alfa gene promoter 0/B, and this EGFP expression was shown as a live, specific marker for the SDN-POA neurons (0/B-SDN). Recently, we have visualized the nucleogenesis of the 0/B-SDN *in vitro* using the organotypic brain slice cultures and time-lapse imaging. These results suggested that scattering neural migration by estrogen was critical role for the masculinization of the SDN-POA. Further, we examined whether the actin dynamics, through Rac1 pathway, could be involved in the sexual differentiation of the SDN-POA. Rac1 inhibitor prevented the estrogen-induced masculinization of the 0/B-SDN. These results propose the regulation of the neural migration mediated by ERα/Rac1/cofilin/actin pathway is crucial for the establishment of sexual dimorphism of the SDN-POA.

### [3P-179]

### Difference in thermoregulatory responses to cold between group and individual-housed mice

*KEI NAGASHIMA[1], YUTA MASUDA[1], TAISUKE SUGI[1] ([1]*Waseda University*)

[Aim] We tested the hypotheses that, when mice are housed in group in cold environment, they show less activation of the brown adipose tissue and augmented behavioral thermoregulatory response. [Methods] All experimental procedures were approved by the ethical committee of Waseda University. Thirty-two male C57BL/6 mice (7-w age) were housed in four different conditions for 1 month: groups housing (n=8 each) at 27˚C or 22˚C and individual housing (n=16) at 27˚C or 22˚C. Abdominal temperature for all mice was continuously monitored. For group-housed mice, huddling behavior was assessed with video system. After the 1-mo period, cold escape behavior of each mouse was evaluated with a newly developed behavior-assessment system. After the period, all mice were housed at 27˚C for 3 days and then 3-h cold exposure at 18˚C was conducted. The inter-scapular brown adipose tissue was obtained at the end to evaluate the expression of UCP1 and 3 mRNA. [Results and Conclusion] Group-housed mice at 22˚C showed greater number of huddling behavior and similar level of abdominal temperature compared to other mice. The mice showed less activation of cold escape behavior in the behavior-assessment system and less expression of UCP1 mRNA after the 3-h 18˚C exposure. Group housing may change both autonomic and behavioral thermoregulatory response to cold.

### Poster Presentation

[3P]
**Nutritional and metabolic physiology, Thermoregulation**

March 16 (Thu.), 12:10 - 14:10, Poster Room

### [3P-180]

### Some correlation between ornithine and the activation bioactive substances.

*Mai Maeda[1] ([1]*Toua Postgraduate school*)

Background and objectives: Treatment of noncommunicable diseases (NCDs) huge economic effective or insurance system dysfunction that I aimed to improve. World health organization (WHO) releases key facts about NCDs. Cardiovascular diseases account for most NCD deaths annually. The fact attribute deaths followed by raised blood glucose and overweight, obesity. Furthermost they are at higher risk of being exposed to processed food products or unhealthy dietary habits. We intake various nutrients which is from foods and supplements. Under this situation, we need to have alternative diet for healthful or feeling well. Some foods contain ornithine and we take it, improve lipase in our body. The enzyme flow in the blood, yield a chemical reaction in portion of our body. The chemical substances in the body that make fats change into acids and alcohol. This chemical reaction has impact on our body with keeping healthy. Ornithine is arising free fatty acids through stimulating endocrine system of growth hormone (GH). It shows new possible the medical nutrition therapy to lower causing obesity. Shijimi clam could improve lipase and control arising free fatty acids rather than all of foods.Methods: Mature adipose cell controlled Dulbecco's modified Eagle's minimal essential medium. Enzymes are detected by western blot analysis to determine the protein mass. We make sure that free fatty acids and glycerol release to assay system. Analyzed messenger ribonucleic acid with real time polymerase chain reaction. Results: There's some correlation between ornithine and the activation bioactive substances. Hormone sensitive lipase, adipose triacylglycerol lipase, are regulated of the amount and quality concerning modified phosphorylation. Adiponectin is referenced good hormone for some organs, adipose tissue is one of them. The activation of adiponectin in adipose cell, which could inhibit to grow adipose tissue. Bioactive substances are different from DNA and mRNA, needed to examine gene transcription for protein synthesis. Conclusions: We show the new evidence strengthen by ornithine to prevent cardiovascular. Ornithine cycle present in mitochondrion of the liver, ammonia convert into not toxic substances. A person has never been born without physical abilities in the body. Our ability could change whatever food we have. Adiponectin is not less reveal the function than GH. Adiponectin is increasing by adapting ornithine to get help for G-protein-coupled receptors. Obesity would make the burden too heavy for heart, what we call acute obesity triggered arteriosclerosis, or hardening of the arteries. Selection in ornithine supplements or not, which think carefully make up own looks and health. On the other hands, WHO response to the follow. To collaborate to reduce the risks associated with NCDs, and to promote interventions to prevent and control them by individuals and socie

### [3P-181]

### *Transient receptor potential canonical 7(TRPC7) deficiency restrains high-fat-high-fructose diet (HFHF) induced-aortic damage and aging in mice*

*Hao Shen[1], Wen-Li Hsu[2], Chia-Hsiu Wu[1], Yun-Fang Liang[1], Cheng-Hsu Hsieh[1], Yi-Hsuan Chen[1], Yi-Chun Hsieh[1] ([1]*School of Medicine, College of Medicine, Kaohsiung Medical University,* [2]*Department of Dermatology, Kaohsiung Municipal Ta-Tung Hospital, Kaohsiung Medical University Hospital, Kaohsiung Medical University*)

*Background*A Western lifestyle that includes a HFHF-induced metabolic disorder is associated with an increased risk of cardiovascular diseases, which are considered the leading cause of death globally. This metabolic disorder leads to aortic damage via increasing the level of reactive oxidative species (ROS) accumulation and inflammation, triggering pathogenesis of cardiovascular diseases. Our previous study indicated that TRPC7 activation potentiates the cause of ROS elevation, DNA damage response (DDR) and senescence-inflammatory response (SIR), initiating age-related pathogenesis. TRPC7 has been reported as a key initiator mediating myocardial apoptosis, and thereby resulting in the process of heart failure. Metabolic disorder is also correlated with progressive regulation of myocardial inflammation and apoptosis. Although TRPC7 is involved in regulating the process of heart failure, the effect of TRPC7 on the metabolic disorder associated-aortic pathogenesis is still unclear. *Objectives*This study investigated the role of TRPC7 in metabolic disorder associated-aortic pathogenesis. *Methods*We utilized HFHF to mimic the metabolic disorder in C57BL/6J mice. After HFHF feeding for 12 weeks, we sacrificed the mice and recorded their body weight and harvested their aortas. The thickness of the aorta was measured by ImageJ, and the lipid droplets in the aorta were detected using Oil red O staining. The DDR was demonstrated by immunohistochemistry staining with 8-oxo-dG and p16INK4; senescence was identified by staining with senescence-associated β-galactosidase (SA-β-gal) and plasminogen activator inhibitor-1 (PAI-1). Cyclooxygenase-2 (COX-2) was stained to examine the inflammation, and cell apoptosis was determined by detecting caspase-3 expression in the aorta. *Results*Our study indicated that mice fed a HFHF had an increased body weight that was significantly decreased in TRPC7 knockout mice. HFHF-induced lipid droplet deposition in the aorta was restrained with TRPC 7 deficiency. We found that the ratio of adventitia to tunica media thicknesses was increased in mice fed a HFHF, but deficient TRPC7 inhibited the HFHF-induced ratio of adventitia to tunica media increase. Besides, blockage of TRPC7 in the aorta also significantly reduced HFHF-induced DDR, SIR and cell death, while p16INK4, COX-2, SA-β-gal, PAI-1 and caspase-3 were upregulated in the tunica media of aorta of mice fed a HFHF. The 8-oxo-dG was elevated at the endothelium, implicating that DNA damage was occurring at the surface of the vessel. However, mice fed a normal diet, with or without TRPC7, had no pathophysiological effects on their aortas. *Conclusion*Our study illustrated that blockage of TRPC7 inhibited HFHF induced-aortic damage and aging. TRPC7 may play a crucial role in regulating metabolic disorder associated-aortic pathogenesis which initiates the progression of cardiovascular diseases.

### [3P-182]

### Specific expression of $K^+$-$Cl^-$ cotransporter(KCC2) in in the α cells of normal and type 1 diabetes model mouse pancreatic islets

*Chigusa Shimizu[1], Shigeki Okada[1], Shiki Okamoto[2], Hiroaki Masuzaki[2], Chitoshi Takayama[1] ([1]*Department of Molecular Anatomy, Graduate School of Medicine,University of the Ryukyus,* [2]*Division of Endocrinology, Diabetes and Metabolism, Hematology, Rheumatology, Graduate School of Medicine,University of the Ryukyus*)

GABA is an inhibitory neurotransmitter in the mature brain. However, it acts excitatory during development. This difference in action depends on the intracellular chloride ion concentration, regulated by $K^+$-$Cl^-$ cotransporter (KCC2). Sufficient KCC2 expression results in its inhibitory action. GABA is abundant in pancreatic islets, where it acts differentially on the islet cells, and is involved in carbohydrate metabolism. However, the mechanisms underlying the differential action remain unknown. We performed immunohistochemistry for glutamic acid decarboxylase (GAD), a synthetic enzyme for GABA, and KCC2 in normal adult islets. GAD was colocalized with insulin in β cells, whereas KCC2 was expressed in glucagon positive α cells. These results are in line with previous observations that GABA decreases glucagon release but increases insulin release, and suggest that GABA and insulin may work together in reducing blood glucose levels under hyperglycemia. Next, we examined the streptozotocin-induced type1 diabetes mellitus mouse model. GAD and insulin expression levels were markedly decreased. KCC2 was expressed in glucagon-positive cells, whereas insulin- and somatostatin-positive cells were KCC2-negative. These findings suggest that in diabetes model, reduced GABA release may cause disinhibition of glucagon release, resulting in increased blood sugar levels and the maintenance of hyperglycemic state.

### [3P-183]

### Elucidation of the role of thermogenic adipocytes in controlling sucrose intake

*Arata Mori[1], Yasuo Oguri[1], Sho Matsui[1], Satoshi Tsuzuki[1], Tsutomu Sasaki[1]
(*[1]Laboratory of Nutrition Chemistry, Division of Food Science and Biotechnology, Graduate School of Agriculture, Kyoto University*)

Thermogenic adipocytes including brown and beige adipocytes increase energy consumption and regulate glucose metabolism. They also work as metabolic sink and secretory organ. However, the role of these adipocytes in controlling food intake is still unclear. In this study, we used selective b3-adrenoceptor agonist to generate thermogenic adipocytes-activated mice model. We assessed the preference for sucrose in thermogenic adipocytes-activated mice and saline-treated mice (control mice) by using food choice test (regular diet vs. high-sucrose diet). We found that high-sucrose diet intake was significantly lower in thermogenic adipocyte-activated mice relative to control mice. Thereafter, we examined secretory factor that is responsible for reducing of high-sucrose diet intake.Here, we revealed that the administration of selective b3-adrenoceptor agonist dramatically increased plasma FGF21 levels. Of note, our laboratory previously has reported that FGF21 reduces the intake of simple sugar. Taken together, these data suggest that thermogenic adipocytes regulate high-sucrose diet intake via a mechanism involving the secretion of FGF21.

### [3P-184]

### Elucidating the role of the amino acid balance sensing system in regulating food intake

*Kento Yoshida[1], Mai Tsukamoto[2], Sho Matsui[1], Shun-ichiro Asahara[3], Yasuo Oguri[1], Satoshi Tsuzuki[1], Tsutomu Sasaki[1] (*[1]Nutrition Chemistry, Division of Food Science and Biotechnology, Faculty of Agriculture, Kyoto University, [2]Department of Food Science and Biotechnology, Faculty of Agriculture, Kyoto University, [3]Division of Diabetes and Endocrinology, Department of Internal Medicine, Kobe University Graduate School of Medicine*)

The intake of protein with good quality is essential to sustain life. However, how amino acid balance of food is detected and regulates food intake is not yet understood. We performed experiments in cells and mice to determine if the amino acid balance in ingested protein is detected and imbalanced diet is avoided via GCN2 and GDF15, which is activated by amino acid imbalance and a stress-responsive cytokine that induces taste aversion and loss of appetite, respectively. Phosphorylation of GCN2 and its downstream target, eIF2α, was observed in hepatic AML12 cells cultured for 30 minutes in threonine-depleted medium. Mice fed a low-protein diet (LPD) supplemented with amino acids except threonine showed a significant increase in blood GDF15 4 hours after feeding, followed by a decrease in food intake for 2 hours. In addition, mice fed a LPD supplemented with five essential amino acids consumed less food than those fed only LPD. These results indicate that threonine deficiency causes activation of hepatic GCN2 and eIF2α and induces GDF15 secretion, and that feeding of the amino-acid-imbalanced diet suppressed food intake regardless of the kinds and number of limiting amino acids.

### [3P-185]

### The satiety effect of 1,5-Anhydro-d-fructose

Hikaru Monnkawa[1], Yuto Yamaguchi[1], Kenji Torigoe[1], *Masanori Nakata[1] (*[1]Department of Physiology, Faculty of Medicine, Wakayama Medical University*)

1,5-Anhydro-d-fructose (1,5-AF) is a bioactive monosaccharide that is produced by the glycogenolysis in mammalian and is metabolized to 1,5-anhydro-d-glucitol (1,5-AG). 1,5-AG is used as a marker of glycemic control in diabetes patients. 1,5-AF has a variety of physiological activities, but its effects on feeding behavior are unclarified. The present study examined whether 1,5-AF possess effect of satiety. Oral administration of 1,5-AF, not of 1,5-AG suppressed daily food intake. In addition, intracerebroventricular (ICV) administration of 1,5-AF also suppressed feeding. To explore the brain region targeted by 1,5-AF, we investigated c-Fos expression in the hypothalamus implicated in feeding. ICV injection of 1,5-AF significantly increased c-Fos expression in the paraventricular nucleus (PVN). Next, to examine the target PVN neuron subpopulation, we analyzed the expression of neuropeptides in PVN. ICV administration of 1,5-AF increased mRNA expression of oxytocin. Moreover, ICV administration of 1,5-AF also stimulated oxytocin release. Furthermore, the satiety effect of 1,5-AF was abolished in oxytocin knockout mice. These results suggest that 1,5-AF exhibits satiety effects via the activation of oxytocin neurons.

### [3P-186]

### The role of GABAergic neurons in the lateral hypothalamic area and zona incerta in glucose metabolism

*Yu Long[1,2], Kunio Kondoh[1,2], Yasuhiko Minokoshi[1,2] (*[1]Division of Endocrinology and Metabolism, National Institute for Physiological Sciences (NIPS), [2]The Graduate University for Advanced Studies (SOKENDAI)*)

The lateral hypothalamic area (LHA) and zona incerta (ZI) are important regions that regulate feeding behavior. Previous studies have shown that electrical stimulation of LHA changes glucose metabolism in the liver and that LHA neurons project to peripheral tissues such as pancreas and liver, suggesting that LHA and an anatomically close area ZI play an important role in glucose metabolism. However, the role of LHA and ZI neurons in glucose metabolism remains largely unclear. LHA contains similar numbers of both glutamatergic and GABAergic neurons, and ZI neurons are mostly GABAergic in mice and rats. In this study, we investigated whether LHA glutamatergic and LHA/ZI GABAergic neurons control glucose metabolism. We activated glutamatergic and GABAergic neurons in LHA and ZI neurons in male mice using Vglut2- and Vgat-ires Cre mice, respectively, and performed glucose (GTT) and insulin (ITT) tolerance tests. GTT revealed that activation of LHA/ZI GABAergic neurons improves glucose tolerance, while that of LHA glutamatergic neurons does not. ITT also showed that activation of LHA/ZI GABAergic neurons tends to increase insulin sensitivity.These results suggest that LHA/ZI GABAergic neurons regulate not only feeding but also glucose metabolism, including insulin sensitivity in peripheral tissues.

### [3P-187]

### Involvement of the glutamine metabolic pathway in microglial proinflammatory responses and significance of its metabolites

*Itaru Watanabe[1], Teruaki Yamaguchi[1], Haruna Takeda[1], Kodai Nagashio[1], Hajime Yano[1], Junya Tanaka[1] (*[1]Department of Molecular and Cellular Physiology, Ehime University Medical School*)

We have previously reported that extracellular glutamine (Gln) is essential for the proinflammatory response of microglia, and (2) activation of NRF2, a known antioxidant and anti-inflammatory transcription factor, occurs in the background of the proinflammatory response that is suppressed under Gln starvation. Using the mouse microglial cell line BV2, herein, we investigated the metabolism of intracellularly recruited Gln and how it is involved in the proinflammatory response by measuring levels of its amino acid as well as some metabolites during the proinflammatory response. Under Gln-starvation, the observed fluctuations in Glutamate (Glu) content during the proinflammatory response almost disappeared, as did the increased production of glutathione includes Glu as a component. The nitric oxide (NO) production is a typical proinflammatory response and arginine is the raw material of NO. Arginine tend to be decreased during the proinflammatory response, while increased during Gln starvation. Interestingly, marked increases of the levels of alanine, aspartic acid, and ammonia in the medium were observed during the proinflammatory response. We would like to discuss the possible relevance of the excessive inflammatory response of microglia to disease based on these changes in amino acid metabolism, and possible ways to address inflammatory disease by focusing on these changes.

### [3P-188]

### The exposure to dashi through the lactating mother alters appetite of offsprings for oil during their adulthood.

*Shunsuke Fushimi[1], Tsutomu Sasaki[1] (*[1]Laboratory of Nutrition Chemistry, Division of Food Science and Biotechnology Graduate School of Agriculture, Kyoto University*)

[Background] Traditional Japanese foods are eaten over generations despite their low-fat content. Although the ingestion of dashi, a characteristic of the Japanese food, has been reported to have a negative correlation with indicators of obesity, how dashi works remains elusive. Our preliminary investigation showed that the exposure to bonito bonito broth after weaning had no effect on appetite. Therefore, we hypothesized that there is a critical period for dashi to affect appetite, and that it happens prior to the weaning. [Methods] To expose offsprings to dashi prior to weaning, pregnant mice (C57BL6) were exposed to 10% concentrated bonito broth for a specific period (embryonic & lactation, embryonic, lactation). The control (non-exposed) group received water ad libitum. After pups reached adulthood, a licking test was performed to assess appetite in terms of palatability and motivation. To assess the appetite for oil., a 2.5% solution of corn oil was presented in the light phase for 15 minutes to assess the appetite for oil. [Results] Burst size and the number of bursts, representing palatability and motivation, respectively, were significantly increased in the embryonic & lactation and the lactation groups compared to the non-exposed and the embryonic groups. [Conclusion] The exposure to bonito broth through the lactating mother alters appetite of offsprings for oil during their adulthood.

### [3P-189]

### The *in vivo* function of EID1 in lipid metabolism

*Itsuki Takahashi[1], Yusuke Watanabe[1], Tomohiko Sato[2], Mitue Miyazaki[3], Izuki Amano[4], Takeo Nakanishi[1], Noriyuki Koibuchi[4], Noriaki Shimokawa[1,4] ([1]*Takasaki University Graduate School of Health and Welfare,* [2]*Ota College of Medical Technology,* [3]*Hirosaki University Graduate School of Medicine,* [4]*Gunma University Graduate School of Medicine*)

In mammals, two types of adipose tissue have been observed: white adipose tissue, (WAT), which stores excess energy as triglycerides (TG) under the skin and around abdominal viscera, and brown adipose tissue (BAT), which releases stored energy as heat. We recently showed that EP300-interacting inhibitor of differentiation 1 (EID1) inhibits the accumulation of TG in mouse pre-adipocyte 3T3-L1 cells through the downregulation of glycerol 3-phosphate dehydrogenase (GPDH), which is a key enzyme in the synthesis of TG. To clarify the function of EID1 *in vivo*, we generated EID1 transgenic mice (EID1 Tg mice) overexpressing EID1 in only adipose tissues. Interestingly, when these mice were exposed to a cold environment (4 °C, 1 h), the glucose uptake was increased in BAT of the interscapular region, and many genes involved in thermogenesis, such as uncoupling protein-1 (Ucp-1) and peroxisome proliferator-activated receptor γ coactivator 1-α (Pgc-1α), were strongly expressed. We are currently investigating the gene expression mechanism of EID1-mediated thermogenesis by cold exposure and whether or not cold exposure actually increases the body temperature in EID1 Tg mice.

### [3P-190]

### High-intensity exercise training induces oxidative modification of mitochondrial proteins in skeletal muscle

*Maki Takami[1], Wataru Aoi[1], Chinatsu Ando[1], Yoji Kato[2], Yukiko Kobayashi[1], Masashi Kuwahata[1] ([1]*Kyoto Prefectural University,* [2]*University of Hyogo*)

Background: Generation of reactive oxygen species (ROS) is increased in skeletal muscle during exercise. In muscle cells, mitochondria are a major source of ROS, with elevated oxygen consumption. The activity of proteins can be regulated by various oxidative post-translational modifications. The purpose of this study was to examine the oxidative modification of mitochondrial proteins in an exercise mouse model. Methods: ICR mice were divided into two groups, consisting of sedentary and exercise groups. In the exercise group, mice performed treadmill running 5 times per week for 2 weeks. On the next day of the final exercise, gastrocnemius muscles were collected. The level of proteins modified by N-hexanoyl lysine (HEL), 4-hydroxy-2-nonenal (HNE), 3-nitrotyrosine and dihalogenated tyrosine was measured in the muscle. Thereafter, HEL- and HNE-modifications of aconitase 2, carnitine palmitoyltransferase 1, dynamin-related protein 1 and malate dehydrogenase 2 (MDH2) were examined. Results: Oxidative protein modifications of whole lysate extracted from muscle tissues were not significantly different between sedentary and exercise groups. However, HEL- and HNE-modifications of mitochondrial fraction proteins showed a tendency of higher in the exercise group than the sedentary group. Moreover, HEL- and HNE-modifications of MDH2 were significantly higher in the exercise group. Conclusions: Oxidative stress induced by high-intensity exercise training post-translationally modified MDH2 in skeletal muscle, which may cause impaired metabolic system.There is no conflict of interest.

### [3P-191]

### Brain regions related with thermal sensation

*Hironori Watanabe[1], Shibuya Satoshi[2], Sugi Taisuke[1], Kato Issei[1], Kataoka Yufuko[1], Kiyoshi Saito[1], Nagashima Kei[1] ([1]*Waseda University,* [2]*Kyorin University*)

Thermal sensation is induced in the brain via afferent feedback from the peripheral receptors, but it remains controversial which brain regions are relevant. The aim of the present study is to identify brain regions involved in warm and cold sensations by using electroencephalography (EEG). 20 right-handed participants were received local thermal stimulus to the right fingers with the Peltier apparatus while sitting with closed-eyes in a controlled room (26 °C and 50 %RH). The local thermal stimuli were applied 40 times in each condition by paired-thermal stimulus with 15-s of reference stimulus (32 °C) followed by 10-s of conditioned stimulus (warm condition: 40 °C and cold condition: 24 °C). 15-ch EEG signals were continuously measured during each condition. To identify the thermal sensation-related brain regions, independent component (IC) analysis was applied to the preprocessed EEG data. Then, the equivalent current dipole locations were estimated, followed by clustering (k-means method) for the ICs with a dipole residual variance < 15 %. For time-frequency analysis of each target cluster, the event-related spectrum perturbation (ERSP) were compared between warm and cold conditions. Clustering identified some clusters related to thermal sensation whose dipoles were in bilateral premotor and supplementary motor cortices, right-frontal pole and right-somatosensory association area. However, ERSP of the clusters were not different between conditions. The present results suggest that thermal sensation might be induced in the same brain regions regardless of warm or cold sensation.

### [3P-192]

### Comparison of ear canal and rectal temperatures in a hot environment

*Kato Issei[1], Nagashima Kei[1] ([1]*Waseda University*)

[Background] Measuring core body temperature ($T_{core}$) is essential to evaluate thermoregulation. Several body sites, such as the axilla, esophagus, and rectum were adapted to measure $T_{core}$. Accuracy and low invasiveness are important in continuous $T_{core}$ measurement. The present study evaluated the ear canal temperature ($T_{ear}$) as a $T_{core}$ during exercise, heat stress, and fan cooling. [Methods] $T_{ear}$ and rectal temperature ($T_{rec}$) were measured in all experiments. In Experiment 1, a 30-min treadmill exercise was conducted at an ambient temperature ($T_a$) of 35ºC and 65% relative humidity (RH). In Experiment 2, a 70-min treadmill exercise was performed with intermittent fan-cooling at $T_a$ of 35ºC and 50%RH. In Experiment 3, subjects remained seated for 120 min at $T_a$ of 28ºC and 50% RH. Experiment 3 consisted of two trials with different interventions from 60-90 min. One is the trial that continuous fanning was given (FAN trial), and the other is 41˚C hot-water immersion of both legs (HWI trial). [Results] In Experiment 1, there were no significant differences between $T_{rec}$ and $T_{ear}$ during the 30-min exercise ($P$=0.21). $T_{ear}$ has captured during the exercise with a substantial agreement (ICC=0.76, $P$=0.003). In Experiment 2, $T_{ear}$ was significantly lower than $T_{rec}$ at 115-120 min ($P$<0.05). $T_{ear}$ has trail $T_{rec}$ during the exercise of intermittent fan cooling with a moderate agreement (ICC=0.6, $P$<0.001). In the Experiment 3 FAN trial, no significant differences were observed between $T_{rec}$ and $T_{ear}$ during 120-min experimental phases. Continuous fan cooling resulted in a lower ICC value between $T_{ear}$ and $T_{rec}$ compared to the rest phase (ICC=0.75, $P$<0.001; and ICC=0.58, $P$=0.001; respectively). In the HWI trial, $T_{ear}$ was significantly lower than $T_{rec}$ at 80-95 min ($P$<0.05). HWI did not alter the ICC value but lowered the ICC value (ICC=0.44, $P$=0.01) during the subsequent recovery phase. [Conclusions] Ear canal temperature is applicable for monitoring $T_{core}$ increase due to exercise and heat load.

### [3P-193]

### The effect of the ketogenic diet on inflammatory pain

*Kei Eto[1], Masanori Ogata[1], Hitoshi Ishibashi[1] ([1]*Department of Physiology, School of Allied Health Sciences, Kitasato University,*)

Peripheral inflammation, which is caused by tissue damage and several diseases, induces inflammatory pain. In addition, persistent inflammation in the peripheral tissues alters peripheral nerve activity, resulting in plastic changes in the central nervous system. These plastic changes contribute to the induction and maintenance of chronic inflammatory pain. Anti-inflammatory drugs such as NSAIDs are used to treat inflammatory pain, but long-term drug use has problems, such as side effects. Thus, in addition to drug treatment, it is necessary to establish another therapeutic treatment. The ketogenic diet is high in fat, low in carbohydrates, and adequate in protein. This diet has been used to treat epilepsy, and recent reports demonstrate that the ketogenic diet has therapeutic effects on other diseases, such as Parkinson's disease and Alzheimer's disease. The ketone body, which is generated from fat, has an anti-oxidative effect by inhibiting reactive oxidative species. Since inflammation is critical for the induction of abnormal pain, we assumed that the ketogenic diet might alleviate inflammation-induced pain. Injection of formalin into mouse hind paws induces inflammation, which in turn causes acute pain and chronic pain. Thus in this study, we investigated the effect of the ketogenic diet on formalin-induced inflammation pain. Injection of formalin into the dorsal surface of the mouse hind paw cause licking and biting, which indicate spontaneous pain. The number of licking and biting in ketogenic-fed mice was consistent with that of control diet-fed mice. This result suggests that the ketogenic diet cannot prevent acute pain induced by formalin. Next, we assessed the volume of the mouse hind paw one week after the formalin injection. Formalin injection increases the paw volume in control diet-fed mice, while the ketogenic diet prevents the induction of paw edema. This indicates that the ketogenic diet has anti-inflammatory effects on formalin-induced paw edema. Moreover, formalin-induced chronic pain-like behavior, which was induced in both hind paws ipsilateral and contralateral to formalin injection, was alleviated by the ketogenic diet. The present study demonstrates that the ketogenic diet has an analgesic effect on formalin-induced inflammatory pain. These findings may provide a new efficient therapeutic treatment against chronic pain by focusing on the ketogenic diet.

### [3P-194]

### Can homeostasis of peripheral blood components alone shape the metabolic zonation in hepatic lipid metabolism?: a simulation study

*Kensuke Tozuka[1,2], Yasuhiro Naito[1,3], Masaru Tomita[1,3] ([1]*Institute for Advanced Biosciences, Keio University,* [2]*Systems Biology Program, Graduate School of Media and Governance, Keio University,* [3]*Faculty of Environment and Information Studies, Keio University*)

It has been reported that hepatocytes have gene expressions regiospecifically in the hepatic lobule and metabolize heterogeneously across sinusoids, a phenomenon termed metabolic zonation. Metabolic zonation is assumed to contribute to key liver functions, treated as an example of evolutionary optimization of metabolic function. If the assumption is correct, the extant spatial map of gene expressions across sinusoids would be optimized by some evolutionary constraints. In this study, we verified whether the homeostasis of peripheral blood components could be regarded as an evolutionary constraint. By using a mathematical model mainly representing hepatic lipid metabolism and bayesian optimization, we explored a variety of spatial gene expression maps that differ from the map found in the actual human liver. As a result, we found that about a thousand spatial maps of gene expressions can maintain the homeostasis of peripheral blood components. This result suggests that the homeostasis of peripheral blood components alone is not enough to shape the actual spatial map of gene expressions contributed to metabolic zonation. Therefore, there would be additional constraints besides the homeostasis of blood components. We will explore the additional evolutionary constraints shaping actual metabolic zonation by machine learning and mathematical optimization in the future.

### Poster Presentation

[3P]
**Behavior, Biological rhythm, Sleep**

March 16 (Thu.), 12:10 - 14:10, Poster Room

### [3P-195]

### Lactate receptor GPR81 in the ventromedial hypothalamus suppresses whole body fat oxidation during endurance exercise: Implications for brain lactate as a central fatigue signal

*Takashi Matsui[1] ([1]*University of Tsukuba*)

Although fatigue during exercise plays an important role as a biological defense mechanism to prevent overactivity, its neurobiological mechanisms are still unknown. Exhaustive exercise increases brain lactate derived from glycogen, a glucose storage molecule in the astrocytes. Brain lactate serves not only as an energy source for neurons but also as an inhibitory signal for cAMP synthesis via a lactate receptor (GPR81) expressed mainly at synapses. The aim of this study was to examine whether GPR81 in the ventromedial hypothalamus (VMH), a center of energy metabolism, inhibits whole-body fat metabolism, which serves anti-fatigue during endurance exercise. Rats were injected 4-CIN (Monocarboxylate transporter 2 inhibitor) or 3, 5-DHBA (GPR81 agonist) into VMH, and performed 30 minutes of moderate-intensity exercise (20 m/min) in a treadmill metabolic chamber. $O_2$ consumption ($VO_2$), $CO_2$ emissions ($VCO_2$), and respiratory exchange ratio (RER) were measured as indices of whole-body fat metabolism. 4-CIN increased extracellular lactate levels, and did not alter $VO_2$ but increased $VCO_2$ and RER during endurance exercise. 3, 5-DHBA decreased extracellular lactate but increased $VCO_2$ and RER. These results suggest that GPR81 in VMH suppresses whole body fat oxidation during endurance exercise, providing a novel role of lactate as a gliotransmitter in shaping exercise-induced fatigue.

### [3P-196]

### Blood glucose modulates ghrelin-induced orexigenic effect via vagal afferent nerves

*Yuta Masuda[1], Mayuta Toyooka[1], Taichi Nohmi[1], Kento Ohbayashi[1], Toshihiko Yada[2], Yusaku Iwasaki[1] ([1]*Laboratory of Animal Science, Graduate School of Life and Environmental Sciences, Kyoto Prefectural University.*, [2]*Division of Integrative Physiology, Kansai Electric Power Medical Research Institute.*)

Anorexia is a risk factor of health problems including flail and sarcopenia, but its therapeutic has not yet been developed. Ghrelin is well known to be an orexigenic gastrointestinal hormone, and its action is mediated by the vagal afferents. However, its underlying mechanisms remain poorly understood. In this study, we investigated the blood glucose (BG) levels on the orexigenic action of ghrelin and the involvement of the vagal afferents. In ICR mice at 9:30, 2 hours after the start of light phase, feeding behavior were suppressed by satiety and BG were moderately high (120 mg/dl). Intraperitoneal administration (IP) of ghrelin at 30 nmol/kg, but not 3 nmol/kg, into the mice significantly increased food intake and pERK1/2 expression as a neural activation marker in nodose ganglion neurons (NGNs). These effects were completely abolished by subdiaphragmatic vagotomy. Co-administration of ghrelin (30 nmol/kg) and glucose (1 g/kg) did not alter feeding and pERK1/2 expression in NGNs. In ICR mice fasted for 8 hours that exhibited moderate hypoglycemia (80 mg/dl), IP administration of ghrelin at 3 nmol/kg increased feeding and its orexigenic effect was blunted by co-administration of glucose at 1 g/kg. The present study demonstrates that blood glucose modulates ghrelin-induced orexigenic effect via vagal afferents visceral sensory pathway.

### [3P-197]

### GLP-1 release by rare sugar D-allulose reduces anxiety and promotes social behavior via GLP-1 receptor signaling

*Kengo Iba[1], Yusaku Iwasaki[1] ([1]*Laboratory of Animal Science, Graduate School of Life and Environmental Sciences, Kyoto Prefectural University.*)

COVID-19 pandemic triggers 25% increase in prevalence of anxiety and depression worldwide. Vagus nerve stimulation recently became an FDA-approved treatment for brain dysfunctions such as treatment-resistant depression and epilepsy. We demonstrate that release of intestinal hormone glucagon-like peptide-1 (GLP-1) by rare sugar D-allulose (Allu) activates vagal afferents, furthermore, when administered in a time-specific manner, chronic D-allulose corrects arrhythmic overeating and obesity. In present study, we examined whether GLP-1 release by Allu regulates anxiety and social behavior and the involvement of GLP-1 receptor (GLP-1R). We measured anxiety behavior using open field test and elevated plus maze, and social behavior using three chamber social interaction test in healthy C57BL/6J mice. In the light period (resting phase), anxiety-related behaviors were higher and social behaviors were lower than these in dark period. Per oral administration of Allu at 1 g/kg and 3 g/kg at the light period significantly reduced anxiety-related behaviors and increased social behaviors. These effects were blunted in GLP-1R KO mice. Intraperitoneal administration of Allu, which does not induce GLP-1 secretion, did not affect anxiety or social behaviors.
In conclusion, we demonstrate that GLP-1 release by Allu reduces anxiety and promotes sociability via GLP-1R signaling.

### [3P-198]

### Early and late visual deprivation induces irreversible hypersensitivity to mechanical and thermal stimuli independently of anxiety in Long-Evans rats.

Nasim Eskandari[1], Masoumeh Ghaemi Jandabi[1], Hugues Leblond[1], *Mathieu Piche[1] ([1]*Universite du Quebec a Trois-Rivieres*)

Visual deprivation leads to several behavioral adaptations. Although these compensations have been well studied for most sensory modalities, the impact of visual deprivation on the nociceptive system and pain sensitivity is still unclear. In humans, pain sensitivity is increased in early, but not late-onset blindness. In animals, sensitivity to noxious stimulation is increased in both early and late visually deprived rodents. It remains unclear if hypersensitivity developing in visually deprived adult rodents is permanent or reversible. It also remains to be determined if hypersensitivity is caused, at least in part, by increased anxiety. The aim of this behavioral study was to examine whether hypersensitivity to noxious stimuli in early and late visually deprived rats is time dependent, whether it is reversible when animals are exposed or re-exposed to a 12:12 light:dark cycle, and whether anxiety may contribute to or modulate some of these effects. The protocol lasted for 24 weeks and included 46 rats distributed in 5 groups: A) Control rats exposed to a 12:12 light:dark cycle for 24 weeks (n=10); B) Dark-reared rats remaining in the dark for 24 weeks (n=10); C) Dark-reared rats exposed to a 12:12 light:dark cycle at 16 weeks for 8 weeks (n=10); D) Rats exposed to a 12:12 light:dark cycle for 8 weeks, then visually deprived for 8 weeks, and then re-exposed to a 12:12 light:dark cycle for 8 weeks (n=8) E) Rats exposed to a 12:12 light:dark cycle for 8 weeks and then visually deprived for 16 weeks (n=8). Mechanical and thermal sensitivity were examined using the Von Frey and tail-flick tests, respectively. Groups were significantly different over time for mechanical and thermal sensitivity as well as for anxiety (all $p<0.05$). Bonferroni planned contrasts revealed that compared with controls, all visually deprived rats showed increased mechanical and thermal sensitivity at 16 weeks, 17 weeks and 24 weeks (all $p<0.05$), regardless of the timing and duration of the visual deprivation. Bonferroni planned contrasts also revealed that compared with controls, none of the visually deprived rats showed increased anxiety at any time point (all $p>0.05$), and rather tended to or showed lower anxiety. This indicates that early and late visual deprivation increases mechanical and thermal sensitivity, and that these effects are irreversible and independent of anxiety. This is consistent with behavioral compensations reported for other sensory modalities and suggest that mechanical and thermal hypersensitivity may provide an enhanced capacity to avoid noxious stimuli and limit tissue damage in visually deprived rats. These results warrant future studies to determine the brain mechanisms of this hypersensitivity and to clarify if the effects produced by early and late visual deprivation rely on similar or distinct mechanisms.

### [3P-199]

### Synchronization of the exercise timing of two rats in a social wheel cage

Ko Yamanaka[1], *Hidefumi Waki[1] ([1]*Juntendo Univ.*)

While exercising with other highly motivated individuals is known to effectively maintain and improve exercise motivation, the underlying neuronal mechanisms remain unclear. We aimed to establish a behavioral experiment model which could help examine whether social interaction affects exercise motivation in rats. By combining two wheeled cages, we developed a social wheel cage that can change the partition between two rats. Sixteen male Long-Evans rats (4 weeks old), in eight separate pairs, were kept in the social wheel cage for 4 weeks. The partition between the cages was replaced every week either with a wire-mesh (pair condition) or a black acrylic panel (single condition). As an index of the locomotive activity, the number of wheel rotations was measured. The study revealed no significant changes in the locomotive activity between the pair and single conditions. However, cross-correlation analysis revealed higher synchronization of exercise onset timing between the two rats in the pair condition compared to the single condition ($p < 0.05$). These findings suggest that the initiation of exercise by one of the rats triggered the exercise motivation in the other, and induced the synchronization of exercise motivation.

### [3P-200]

### A heart rate study on the internal state induced by distress calls in Japanese house bat, *Pipistrellus abramus*

*Kazuki Yoshino-Hashizawa[1], Midori Hiragochi[1], Motoki Kihara[1], Kohta I Kobayasi[1], Shizuko Hiryu[1] ([1]*Doshisha University*)

Bat's behavior highly relies on acoustic information, including echolocation and communication through ultrasound calls. However, how their internal state is changed by the acoustic information remains unclear. In this study, we approached the internal state in captive Japanese house bats, *Pipistrellus abramus*, using distress calls (DC), which were often uttered under physical stress, as stimuli. Behavioral and heart rate (HR) responses to the DC were measured for evaluating their internal state. Our results showed that the presentation of a DC-uttering bat evoked freezing behavior, one of the major fear responses, and increased HR. These results suggest the DC activated the sympathetic nervous system. Additionally, presenting echolocation call and DC using flip-flop oddball paradigm revealed that HR increased approximately 25 % for only deviant DC. This HR response suggests that the HR did not simply reflect a stimulus but rather included high-level cognition of the global context of stimulus and the DC uttered in the right context might mediate contagion of the stressed state. (COI: NO)

### [3P-201]

### Neural mechanism underlying the wave-like propagation of cerebral vasomotion

*Tomokazu Ohshiro[1], Yoshikawa Takeo[2], Mushiake Hajime[1] ([1]*Dept. of Neurophysiology, Sch. of Medicine, Tohoku University,* [2]*Department of Pharmacology, Tohoku University Graduate School of Medicine*)

Cerebral arteries are known to oscillate in their vessel wall diameter continuously at less than 0.1Hz, a phenomenon known as cerebral vasomotion. The mechanism underlying the vasomotion and its biological significance, however, are not well understood. We have developed a simple and convenient imaging system for visualizing the cerebral vasomotion, in which the blood vessels are illuminated by polarized green light and the reflected light is detected by a CCD camera. Using this system, we found that the vasomotion propagates like ripples over the rodent cortex. To understand the mechanism for generating the curious spatial-temporal pattern of the cerebral vasomotion, we conducted a pharmacological screening and identified Histamine, which abolished the wave-like propagation of the cerebral vasomotion when administered by intracerebroventricular injection. Importantly, *histidine decarboxylase* knockout mice, which cannot synthesize Histamine, exhibited an impaired cerebral vasomotion. Our results suggest that histaminergic neurons play essential roles for generating the wave-like propagation of the cerebral vasomotion.

### [3P-202]

### Levels of Salivary Chromogranin A relate to Fatigability and Physical complaints before and after night Sleep

*Kentaro Taniguchi[1], Okumura Naoya[2], Akito Shimouchi[2] ([1]*Nagahama Institute of bioscience and technology,* [2]*Chubu University*)

Chromogranin A (CgA) is an acidic secretory protein of the granin family, and saliva CgA (sCgA) is regarded as one of psychological stress marker. The aim of this study is to reveal how sCgA levels be associated with fatigability and physical stress during usual daily life. One hundred seventy one adults aged from 20 to 84 years-old (40.7±21.6 years old, 67 men and 104 women) were employed for this study. They collected their own saliva before night sleep and after wake-up during usual daily life. sCgA was assessed by enzyme-linked immunosorbent assay. Cornell Medical Index (CMI) was used as a questionnaire related to physical and psychological conditions including fatigability. High fatigability group indicated significantly lower levels of sCgA after wake-up than those of low fatigability group. Participants with high physical complaints (subtotal of CMI) had significantly lower levels of sCgA before night sleep than those of low group. High fatigability with lowered secretion of sCgA after wake-up could be possibly caused by the recovery of fatigability during night sleep. High physical complaints with decreased sCgA before night sleep might also suggest reduction of sCgA production via physical and/or mental stress during daytime. We found that lowered sCgA before and after night sleep were associated with fatigability and physical complaints, respectively. Further studies are needed with more detailed trend analysis of sCgA.

### [3P-203]

### Input signal regulation of mammalian circadian rhythms using DNA aptamers that specifically bind to melanopsin

*Kazuo Nakazawa[1], Yo Kikuchi[1], Yoshihiro Nakajima[3], Rika Numano[1,2] ([1]*Department of Applied Chemistry and Life Science, Toyohashi University of Technology, Toyohashi,* [2]*Electronics-Inspired Interdisciplinary Research Institute (EIIRIS), Toyohashi University of Technology,* [3]*Health Research Institute, National Institute of Advanced Industrial Science and Technology (AIST)*)

DNA aptamer has attracted considerable attention as oligonucleotide therapeutics, which binds specifically to a biomolecule to modify their function. We screened DNA aptamer of Melanopsin (OPN4), blue-light photopigment in the retina, which plays a key role for input light signals to reset the phase of circadian rhythm in the central clock. First, 15 kinds of DNA aptamer of Melanopsin (Melapt) by Cell-SELEX with eighth rounds were obtained using cells to overexpress Melanopsin on the cell membrane. Subsequently, a functional analysis of each Melapt was performed in *Period2:ELuc* stable cell-line fibroblasts expressing Melanopsin by phase resetting of mammalian circadian rhythms in response to blue-light stimulation as clock gene to monitor *Period2* rhythmic expression with 24 hours period. At the subjective dawn, 4 types of Melapt were identified for Phase Advance by more than 1.5 hours, and 7 types of Melapt were identified for Phase Delay by more than 2 hours, because Melapt may have some effect on the binding domain of Melanopsin to the *trans*-retinal as ligand to affect the intracellular signaling and regulate the transcription of *Period2*. Some kinds of Melapts resulted in a phase shift ability of approximately 2 hours even without photostimulation, because Melapt only may affect the input signaling for phase shift ability partially. Additionally, we verified that these Melapts could modify the function of Melanopsin for phase shift *in vivo Per1::luc* transgenic (Tg) mice similar to *in vitro Per2::ELuc* cell-line. A Melanopsin DNA aptamer successfully regulated the input signal and phase shift ability in both Phase Advance and Delay of mammalian circadian rhythms *in vitro* and *in vivo*.The Melapts allows you to wake up and go to bed early, regardless of sunlight and blue-light of PC and smartphone. It is so adaptable to regulate the phase shift of mammalian circadian rhythms by non-photic signal, DNA aptamers and so on.

### [3P-204]

### Alterations in discrimination learning ability and dopaminergic inputs to the prefrontal cortex in valproic acid-induced rat autism model

*Junichi Tanaka[1], Akihiko Ushigome[2], Makoto Takahashi[1], Yasushi Hayashi[3] ([1]*Naruto Univ. of Educ,* [2]*Teikyo Heisei Univ.,* [3]*Notre Dame Seishin Univ.*)

Prenatal valproic-acid (VPA) exposure of rats on day 12.5 leads to behavioral deficits of sociability, matching similar alterations in human autism spectrum disorder (ASD). ASD-related morphological and functional changes can be found in the prefrontal cortex. The purpose of this study was to investigate alterations in discrimination learning behavior and dopaminergic inputs to the prefrontal cortex caused by prenatal VPA treatment. Pregnant female Wistar rats received 500 mg/kg VPA or saline (control) i.p. on gestation day 12.5. Three-months-old male offspring (VPA and control rats) were conditioned to press a lever under lamp-on conditions for reward acquisition and lamp-off for no reward utilizing a variable interval reinforcement schedule that averaged 15 s. Over a 30-day period, the VPA and control groups showed increased response to lamp-on from 20.3 to 34.7/min and from 24.1 to 37.6/min, respectively. The discrimination ratio (response to lamp-on as a percentage of total response to lamp-on and -off) of the VPA group (68.8%) was significantly lower than that of the control group (83.6%). To investigate the dopaminergic inputs to the prefrontal cortex, in vivo microdialysis was used. In both the groups, increases in the extracellular concentration of DA in the prefrontal cortex during learning sessions were observed. The amount of the increases was much smaller in the VPA group than in the control group. These findings suggest the possibility that reduced dopaminergic inputs to the prefrontal cortex caused by treatment with VPT may be involved in a lower ability in the discrimination learning.

### [3P-205]

### Theta wave synchronization between hippocampus field potential and electroencephalogram induced by restrained water immersion in rats

*Misa Yoshimoto[1], Morimoto Natumi[1], Ikegame Shizuka[1], Miki Kenju[1] ([1]*Autonomic Physiology, Faculty of Human life and Environment, Nara Women's University*)

The hippocampus plays a major role in cognitive memory function and its function is modulated in response to changes in behavioral states. Estimation of changes in hippocampal function leads to cognitive deterioration and impairment prevention. In this study, changes in hippocampal CA1 neural activity, hippocampal field potential, and electroencephalogram (EEG) was examined in rats exposed to restraint water immersion (RWI) stress and whether changes in hippocampal function during RWI could be assessed by EEG. Hippocampal CA1 neural activity, hippocampal field potential, hippocampal regional blood flow, EEG, cervical electromyogram, electrocardiogram, and catheter for arterial pressure were measured after male Wistar rats were chronically implanted with electrodes at least 4 days before the experiment. Rats were restrained in a box filled with water to rats' limbs for 30 min. Immediately after the starting RWI, hippocampal CA1 neural activity, hippocampal regional blood flow, and EEG theta wave power decreased and remained at low levels during the next 30 min of stress. Simultaneously, the synchronicity between the hippocampal field potential and EEG theta power band increased. These results suggest that RWI causes sustained changes in hippocampal neural activity and field potential and that these hippocampal responses may be potentially estimated from changes in the EEG's theta power. No conflict of interest.

### [3P-206]

### The involvement of the bed nucleus of the stria terminalis in the expression of disgusting responses and fearful approaches in the retrieval of conditioned taste aversion

*Emi Kikuchi[1,2], Tadashi Inui[1], Shaoyi Su[1], Makoto Funahashi[1] ([1]*Department of Oral Physiology, Graduate School of Dental Medicine, Hokkaido University,* [2]*Department of Orthodontics, Graduate School of Dental Medicine, Hokkaido University*)

The bed nucleus of the stria terminalis (BNST) plays a pivotal role in anxiety and fear states. The BNST also receives taste and visceral sensations via the brainstem, which are key components of conditioned taste aversion (CTA). CTA causes a reduction in the intake of a taste solution which is a conditioned stimulus (CS) paired with visceral malaise. To clarify whether the BNST is involved in the retrieval of CTA, we investigated the effects of inhibiting BNST neurons via designer receptors exclusively activated by designer drugs on behavioral responses to a CS. Seventeen male C57/BL6 mice received injections of AAV8-hSyn-hM4Di-mCherry (0.5 µl/side) in the bilateral BNST. They were conditioned by pairing 0.2% saccharin solution (CS) with malaise-inducing lithium chloride in a behavioral analysis apparatus. After confirming the establishment of CTA by examining the reduction of CS intake, we assessed the effect of the administration of a designer drug deschloroclozapine (DCZ; 0.05 mg/kg, i.p.; n = 8) on mice's licking and approach behaviors by comparing the vehicle (1% DMSO in saline, i.p.; n = 9) administration. The number of CS licks during a 15-min session in the DCZ-administered mice (374.44 ± 69.23 licks) was significantly smaller than that in the vehicle-administered mice (72.5 ± 41.86 licks) (unpaired t-test; $p < 0.01$). The analysis of lick microstructure based on inter-lick interval revealed that the DCZ administration significantly decreased the mean size of burst licking (5.35 ± 1.78 licks/burst) as compared to the vehicle administration (11.75 ± 2.1 licks/burst) (unpaired t-test; $p < 0.01$). We analyzed approach behaviors by categorizing Entry (moving towards the spout containing the CS) into the events accompanied by licking behavior (Entry-Lick) and ones without licking (Entry-Stop). The probability of Entry-Stop (the number of Entry-Stop / the number of Entry) was significantly augmented by the DCZ administration (DCZ, 66.89 ± 8.82%; vehicle, 34.18 ± 7.2%; unpaired t-test; $p < 0.05$). Since a more aversive taste solution produces a smaller size of burst licking, it is thought that the DCZ-administered mice displayed a stronger disgust response to the CS than the vehicle-administered mice. Since the Entry-Stop indicates that animals approach the CS but hesitate to lick it, the high probability of Entry-Stop in the DCZ-administered mice shows amplified fear against the CS. These results suggest that the inhibition of the BNST neurons enhances disgusting responses and fearful approaches to a CS. The BNST neurons may be implicated in the emotional aspects of the retrieval of CTA.

### [3P-207]

### Effect of differences in feeding rhythm during gestation on offspring behavior

*Tetsuya Shiuchi[1], Shimizu Noriyuki[1], Otsuka Airi[1], Chikahisa Sachiko[1], Sei Hiroyoshi[1] ([1]*Dept. Physiology, Tokushima University Graduate School of Medical Sciences*)

It has been pointed out that the nutritional status during pregnancy may affect not only the development of offspring but also the development of pathological conditions after growth. However, there are many unclear points about the effect of feeding rhythm during pregnancy on offspring. In this study, we investigated whether rearing mice with different feeding rhythms during gestation affects the development and brain function of offspring. After mating 10-14 week-old C57BL/6J female mice, feeding rhythm was changed from day 7.5, and divided 3 groups: The Control group, which allowed free feeding during the dark period, the Morning group, which allowed feeding only during the first half of the dark period, and the Evening group, which allowed feeding only during the second half of the dark period. After weaning, 3-5 male pups were reared per cage, and various analyzes were performed after 8 weeks of age. Compared to the other groups, offspring in the Evening group showed a tendency to be frightened by contact with the fear one after acquiring fear memory in the passive avoidance test and the social avoidance test. There were no significant differences in other anxiety- and depression-like behaviors among the three groups. These results suggest that evening-type feeding rhythms during gestation may affect the emotional behavior of offspring in response to fear.

### [3P-208]

### Increased activity time (α) and activity fragmentation in a novel bipolar disorder (manic state) mouse model using a reverse translational approach

*Wataru Ota[1], Takuya Takahashi[1] ([1]*Dept. Physiol., Yokohama City Univ. Sch. of Med.*)

Bipolar disorder (BD) is a life-threatening mood disorder defined by the presence of one or more manic episodes (*e.g.*, abnormally elevated mood, decreased need for sleep) with/without depressive episodes. However, the biological basis of BD is poorly understood. Recently, we have developed a novel positron emission tomography (PET) tracer for glutamate AMPA receptor (AMPAR) (Miyazaki *et al.*, *Nat. Med.*, 2020). This tracer exhibited the decrease of AMPAR in the cerebellum of BD patients was significantly correlated with the symptomatology score of manic state (Young mania rating scale; YMRS). Based on this clinical data, we generated a novel mouse model in which AMPAR expression is reduced specifically in the cerebellum. We have observed several bipolar mania-like behavior in this model such as lower immobility time in forced swim test and tail suspension test, and increased sucrose preference (hedonia). Since many circadian disruptions are reported in people suffering from BD, we also examined locomotor activity rhythm of the mice and observed increased activity time (α) and activity fragmentation. These results suggest a high validity of this novel animal model and are expected to lead to further understanding of the neurobiology of BD.

### [3P-209]

### Learning schedule for stay and shift in the three-lever operant task in mice

*Mitsugu Yoneda[1], Yahan Sun[1], Yui Kikuchi[1], Takako Ohno-Shosaku[1] ([1]*Dep. Rehab., Health Sci., Kanazawa Univ.*)

To elucidate the neural basis of motor sequencing and skill learning, we established the Yoneda three-lever operant task in mice. In this task, mice are trained to press three levers in a given sequence through trial and error. We previously showed that this task is dependent on the basal ganglia. The basal ganglia are circuits that evaluate the value of action choices. The learning schedule we established uses stay strategy to learn the lever-food relationship in the one-lever task for shaping. In the present study, we investigated whether a 3-lever task could be learned using shift strategy in the schedule for the 1-lever task; 37 male C57BL/6NCr mice were used. The new shift schedule allowed learning of the 3-lever task and the reverse 3-lever task, and we found no differences in performance with both schedules; in the 1-lever task, the shift strategy suppressed bias toward one lever and required fewer experiments than the stay strategy. These results suggest that it is possible to examine 3-lever tasks with two learning strategies, stay and shift, and that this may be useful in elucidating the neural basis of learning. COI: NO.

### [3P-210]

### Withdrawn

### [3P-211]

### Long-term in vivo calcium imaging in orexin neurons with optogenetic activation of dopamine neurons

*Daisuke Iijima[1], Mukai Yasutaka[1], Yamanaka Akihiro[2] ([1]*Nagoya University Research Institute of Environmental Medicine department of Neuroscience 2,* [2]*Chinese Institute for Brain Research,Beijing*)

### Poster Presentation

[3P]
**Pathophysiology**

March 16 (Thu.), 12:10 - 14:10, Poster Room

### [3P-212]

### Glutamate modulates airway smooth muscle tone through umami and NMDA receptors.

*Haruka Sasaki[1], Kentaro Mizuta[1] ([1]*Division of Dento-oral Anesthesiology, Tohoku University Graduate School of Dentistry*)

Introduction: Asthma is characterized by chronic inflammation in the airways, and local glutamate concentration is elevated in inflammatory tissues. Increased concentration of glutamate in airways may affects asthma symptoms. We have identified several glutamate receptors (Umami, NMDA, and AMPA/KA receptor) in airway smooth muscle (ASM). Here, we investigated whether glutamate modulates ASM tone through these receptors. Methods: Effect of monosodium glutamate (MSG) on ASM contraction; mouse tracheal rings suspended in organ baths were treated with MSG (100 μM) ± umami receptor allosteric modulator (IMP; 150 μM), followed by acetylcholine (ACh; $EC_{50}$). (2) Effect of MSG on ASM relaxation; mouse tracheal rings were pretreated with MSG (100 μM) ± either IMP, NMDA receptor antagonist (D-AP5; 50 μM), or AMPA/KA receptor antagonist (CNQX; 10 μM). Then the rings were precontracted with ACh, followed by cumulatively increasing concentrations of isoproterenol (100 pM - 100 μM). Results: Although MSG alone did not affect basal and ACh-induced ASM tone, IMP potentiated ACh-induced ASM contraction. (2) MSG potentiated isoproterenol-induced ASM relaxation, which was significantly inhibited by D-AP5 or IMP. CNQX did not affect the ASM relaxation. Conclusion: Activation of the umami receptor on ASM augments ASM contraction, while activation of the NMDA receptor potentiates isoproterenol-induced ASM relaxation.

### [3P-213]

### Exploring causal genes for bipolar disorder: Downregulation of a mitochondrial functional gene in a large Mendelian-like family.

*Gakuya Takamatsu[1,2], Yoko Manome[4], Kanako Toyama[1,9], Kumiko Yanagi[5], Dimitar Dimitrov[7], Junseok Lee[1,9], Kae Koganebuchi[6,9], Minami Hasegawa[4], Tomoko Hayakawa[1,8], Tsuyoshi Kondo[2], Tomoyuki Takahashi[7], Tadashi Kaname[5], Hirotaka Okano[4], Ryosuke Kimura[3], Masayuki Matsushita[1] ([1]*Dept. of Mol. Cell. Physiol., Grad. Sch. of Med., Univ. of the Ryukyus, Okinawa, Japan,* [2]*Dept. of Neuropsy., Grad. Sch. of Med., Univ. of the Ryukyus, Okinawa, Japan,* [3]*Dept. of Hum. Biol. Anat., Grad. Sch. of Med., Univ. of the Ryukyus, Okinawa, Japan,* [4]*Div. of Regen. Med., Jikei Univ. Sch. of Med., Tokyo, Japan,* [5]*Dept. of Gen. Med., Natl. Ctr. for Chd. Hlth. Dev., Tokyo, Japan,* [6]*Dept. of Biol. Sci., Grad. Sch. of Sci., Univ. of Tokyo, Tokyo, Japan,* [7]*Cel. Mol. Syn. Func. Unit, Okinawa Inst. of Sci. Tech. Grad. Univ., Okinawa, Japan,* [8]*Dept. of Phamc., Jichi Med. Univ., Tochigi, Japan,* [9]*Adv.Med. Res. Ctr., Fac. of Med, Univ. of the Ryukyus, Okinawa, Japan*)

Bipolar disorder (BD) is a major psychiatric disorder characterized by dysregulation of mood and activity, ranging between depressive and manic states. The pathogenesis is little understood. BD is inheritable with estimated heritability for BD of 70–80%; however, genomic variants that strongly contribute to BD are almost unknown. To elucidate the pathogenesis of BD, we focused on one Ryukyuan multiplex pedigree where BD and recurrent depressive disorder follow a Mendelian inheritance pattern, and we performed comprehensive genetic analyses. First, we determined the haplotype sequence linked to mood disorders by combined linkage analysis and whole genome sequencing. Second, we performed RNA sequencing on neurons differentiated from induced pluripotent stem cells (iPSCs) and analyzed allelic imbalances of transcripts in the linkage haplotype. Finally, we found decreased expression of a nuclear-encoded mitochondrial gene in the affected individuals of the family. It might contribute to mitochondrial dysfunction and the development of the disease in the family. (COI: properly declared.)

### [3P-214]

### Molecular mechanism of cerebral edema improvement via IL-1RA released from the stroke-unaffected hindlimb by treadmill exercise after cerebral infarction in rats

*Kana Sugimoto[1], Rina Gono[1], Chihpin Yang[1], Yukie Murata[1], Reiko Nomura[1], Kazuo Harada[1], Teiji Harada[1], Yohei Miyashita[1], Kazuma Higashisaka[1], Ryuichi Katada[1], Hiroshi Matsumoto[1] ([1]*Osaka Univ.*)

Cerebral edema following cerebral infarction can be severe and directly affect mortality and mobility. Exercise therapy after cerebral infarction is an effective therapeutic approach; however, the molecular mechanism remains unclear. Myokines such as IL-1RA are released during skeletal muscle contraction with effects on other organs. We hypothesized that myokine release during exercise might improve brain edema and confirmed the hypothesis using transient middle cerebral artery occlusion (tMCAO) model rats. Rats subjected to tMCAO were divided according to the severity of illness and further assigned to exercise and non-exercise groups. Treadmill exercises were performed at a speed of 2–8 m/min for 10 min from 1–6 days post-reperfusion after tMCAO. Exercise significantly reduced edema and neurological deficits in severely ill rats, with a reduction in AQP4 expression in the ischemic core and increased blood IL-1RA release from the stroke-unaffected hindlimb muscle after tMCAO. Administration of IL-1RA into the lateral ventricles significantly reduced edema and AQP4 expression in the ischemic core. In conclusion, treadmill exercise performed in the early phase of stroke onset alleviated the decrease in blood IL-1RA following ischemic stroke. IL-1RA administration decreased astrocytic AQP4 expression in the ischemic core, suppressing brain edema.

### [3P-215]

### Usefulness of NSAIDs and their physiological mechanisms in relieving COVID-19 vaccine-induced lymphadenopathy

*Misato Aizawa[1], Yuka Fujikura[1], Nanami Hirai[1], Karen Metoki[1], Itsuro Kazama[1] ([1]*Miyagi University, School of Nursing*)

To help end the pandemic of coronavirus disease 2019 (COVID-19), the vaccination still remains the essential tool. However, the COVID-19 mRNA vaccines frequently cause various side effects shortly after the injection. Although they are eventually self-limiting, the use of suitable medications would ease the symptoms. In our survey, among 191 people aged 18 to 22 years who received their third vaccination shots, more than 50% developed fever, headache and generalized fatigue. Additionally, nearly 30% developed lymphadenopathy, which was rarely observed after their first or second vaccination shots. Most of the people with these symptoms used antipyretics, such as acetaminophen and non-steroidal anti-inflammatory drugs (NSAIDs; loxoprofen, aspirin or ibuprofen). The average durations of fever, headache and generalized fatigue were not significantly different among people who took these medications. However, the average duration of lymphadenopathy was shorter in people who took NSAIDs than those who took acetaminophen. In our patch-clamp studies, NSAIDs effectively suppressed the delayed rectifier $K^+$-channel (Kv1.3) currents in T-lymphocytes and thus exerted immunosuppressive effects. Concerning such pharmacological property, the use of NSAIDs would be more beneficial in relieving the vaccine-induced lymphadenopathy which may be attributable to the enhanced adaptive immune response.

### [3P-216]

### Cetirizine and diphenhydramine differentially exert mast cell-stabilizing properties

*Ririka Fujimura[1], Ayano Asada[1], Itsuro Kazama[1] ([1]*Miyagi University, School of Nursing*)

Cetirizine, a second-generation antihistamine, and diphenhydramine, a first-generation antihistamine, are among the most widely used anti-allergic drugs. In addition to longer duration of action and less incidence of sedative side effects, recent clinical studies additionally indicate a higher potency of cetirizine than diphenhydramine in the treatment or prevention of allergic disorders. In the present study, using the differential-interference contrast (DIC) microscopy, we examined the effects of cetirizine and diphenhydramine on the degranulation from rat peritoneal mast cells. Using fluorescence imaging of a water-soluble dye, lucifer yellow, we also examined their effects on the deformation of the plasma membrane. At relatively higher concentrations, both cetirizine and diphenhydramine significantly reduced the numbers of degranulating mast cells. Of note, at 1 mM, cetirizine more markedly reduced the number than diphenhydramine, almost entirely suppressing the degranulation of mast cells. Additionally, 1 mM cetirizine and levocetirizine, another second-generation antihistamine, almost totally inhibited the process of exocytosis in mast cells and washed out the trapping of the lucifer yellow on the cell surface, while diphenhydramine and chlorpheniramine, another first-generation antihistamine, did not. This study provided *in vitro* evidence for the first time that cetirizine more potently inhibited the process of exocytosis in mast cells than diphenhydramine, indicating its higher potency as a mast cell-stabilizer. Such mast cell-stabilizing property of cetirizine could be ascribed to its counteracting effect on the plasma membrane deformation in degranulating mast cells.

### [3P-217]

### Inducing inferior wall myocardial infarction in bullfrog hearts to reveal the mechanisms of ECG changes

*Mizuki Muto[1], Ryo Kuwana[1], Amu Nagano[1], Itsuro Kazama[1] ([1]*Miyagi University, School of Nursing*)

By surgically ligating coronary arteries, animal models of ischemic heart disease have been created in rodents. However, the use of these animals has been restricted to highly specialized laboratories due to technical difficulties. Using bullfrog hearts, we previously reproduced a ST segment elevation in electrocardiogram (ECG), mimicking human ischemic heart disease. In the present study, by inducing subepicardial burn injuries on the inferior part of the frog heart ventricle, we could reproduce typical ECG changes observed in human inferior wall myocardial infarction, such as the marked elevation of the ST segments in inferior limb leads (II, III, aVF) and their reciprocal depression in the opposite limb leads (I, aVL). Due to the decrease in $Na^+$/ $K^+$-ATPase protein expression, the resting membrane potential of injured cardiomyocytes shifted toward depolarization. Such induced electrical difference between the injured and intact cardiomyocytes was thought to be responsible for the creation of "currents of injury" and the subsequent ST segment changes.

### [3P-218]

### Amitriptyline intoxication in bullfrog hearts to reveal the mechanisms of ECG abnormalities

*Amu Nagano[1], Mizuki Muto[1], Itsuro Kazama[1] ([1]*Miyagi University, School of Nursing*)

Amitriptyline intoxication is caused by suicidal or accidental ingestion of this tricyclic antidepressant. The neurological symptoms include lethargy, coma, convulsion and bilateral loss of light reflexes. Regarding the common cardiovascular abnormalities, such as tachycardia and cardiac arrhythmia, clinical studies have shown that amitriptyline intoxication causes the widening of QRS complexes in electrocardiogram (ECG), which eventually leads to fatal cardiac arrest. In the present study, by injecting various concentrations of amitriptyline (2, 5 and 15 mM) into bullfrogs, we actually revealed that amitriptyline caused the widening of QRS complexes in frog hearts. In simultaneous recordings of the cardiac action potential, amitriptyline decreased the slope of phase 0 in the action potential, indicating the inhibition of the inward sodium currents during this phase. Additionally, treatment with sodium bicarbonate quickly restored the widened QRS complexes in the ECG, suggesting the counteraction with the sodium channel blockade by amitriptyline. The dual recordings of ECG waveforms and the action potential in cardiomyocytes enabled us to demonstrate the mechanisms of ECG abnormalities caused by amitriptyline intoxication.

### [3P-219]

### Magnesium potentiates mast cell-stabilizing property of adrenaline

*Ayano Asada[1], Itsuro Kazama[1] ([1]*Miyagi University, School of Nursing*)

Adrenaline is the first-choice drug for anaphylaxis, since it quickly inhibits the release of histamine from mast cells. However, there are several cases that are resistant to adrenaline. Magnesium is one of the essential minerals for human body mainly consumed from daily foods. Besides health promoting functions, such as bone formation, helping to relax muscle and nervous tension, magnesium is known to exert anti-allergic effects. In the present study, using the differential-interference contrast (DIC) microscopy, we examined the effects of adrenaline (1 uM to 1 mM) and magnesium chloride ($MgCl_2$) (1 to 100 mM) on the degranulation from rat peritoneal mast cells. Both adrenaline and $MgCl_2$ dose-dependently decreased the numbers of degranulating mast cells. At relatively higher concentrations, such as 50 and 100 mM, $MgCl_2$ markedly suppressed the numbers of degranulating mast cells. However, at concentrations equal to or lower than 25 mM, it did not significantly affect the numbers of degranulating mast cells. Of note, higher concentrations of $MgCl_2$ additively enhanced the suppressive effect of adrenaline on mast cell degranulation. The results provided *in vitro* evidence that magnesium dose-dependently inhibited the process of exocytosis, and that it additively potentiated the mast cell-stabilizing property of adrenaline.

### Poster Presentation

[3P]
**Drug Action, Pharmacology**

March 16 (Thu.), 12:10 - 14:10, Poster Room

### [3P-220]

**Adenosine released from muscle by intramuscularly injected lidocaine mediates its analgesic effect**

*Teruaki Nasu[1], Masayo Suzuki[2], Kimiaki Katanosaka[1,2], Kazue Mizumura[3] ([1]*College of Life and Health Sciences, Chubu University,* [2]*Graduate School of Life and Health Sciences, Chubu University,* [3]*Department of Physiology, Nihon University School of Dentistry*)

Intramuscular injection of lidocaine (i.m.-Lido) is commonly used to treat chronic muscle pain. The effect even spreads to the contralateral side. Therefore, its analgesic mechanism cannot be explained by conduction block in axons originally proposed. We hypothesized that analgesia is induced by stimulation of adenosine receptor (AR) by adenosine, which is one of metabolites of ATP. To verify our hypothesis, we examined 1) whether i.m.-Lido induced ATP release from the muscle, and 2) effects of adenosine receptor (AR) antagonist on the i.m.-Lido analgesia. For this we used repeated cold stress (RCS) model rats. We measured ATP release from extensor digitorum longus muscle (EDL) isolated and superfused with Krebs solution using the luciferin-luciferase method. I.m.-Lido into EDL significantly increased the ATP concentration in the perfusate in both normal and RCS rats, but i.m. Krebs solution did not. I.m.-Lido induced reversal of muscular mechanical hyperalgesia in RCS rats was diminished by preadministration of AR antagonists (caffeine, rolofylline). These results suggest i.m.-Lido causes ATP release from the injected muscle, and the its metabolite adenosine acts on AR and induces analgesia.

### [3P-221]

**Exendin-4 enhances DRG neurite outgrowth, Schwann cell survival/migration and myelination via activating PI3K-AKT signaling pathway**

*Kazunori Sango[1], Shizuka Takaku[1], Hideji Yako[1], Masami Tsukamoto[1], Naoko Niimi[1]
([1]*Diabetic Neuropathy Project, Tokyo Metropolitan Institute of Medical Science*)

Besides its insulinotropic actions on the pancreatic β cells, the localization of glucagon-like peptide-1 receptor (GLP-1R) at the nervous system suggests GLP-1's neuroprotective activities. The efficacy of a GLP-1 receptor mimetic exendin-4 (Ex-4) toward brain and peripheral nerve injuries and neurodegenerative disorders, such as Parkinson's disease, multiple sclerosis and diabetic neuropathy, has been documented; however, the underlying mechanisms for the neuroprotective activities of Ex-4 remain obscure and define the aim of this study. By immunofluorescence and western blot analyses, we detected the localization of GLP-1R at dorsal root ganglion (DRG) neurons and Schwann cells in 8-week-old female Wistar rats, as well as primary cultured rat DRG neurons and immortalized Fischer rat Schwann cells IFRS1. Ex-4 promoted neurite outgrowth of DRG neurons at 2 days of culture in a concentration-dependent manner (1 nM < 10 nM < 100 nM). MTS and scratch wound assays revealed that 100 nM Ex-4 significantly enhanced survival/proliferation and migration of IFRS1 Schwann cells. These Ex-4 effects were abolished by co-treatment with 25 μM phosphatidyl inositol-3'-phosphate-kinase (PI3K) inhibitor LY294002. Moreover, immunofluorescence and western blot analyses conducted at 21 days of DRG neuron-IFRS1 co-culture revealed that 100 nM Ex-4 significantly increased the number of myelin protein 22 (PMP22)-immunoreactive IFRS1 cells surrounding βIII tubulin-immunoreactive neurites and up-regulated the protein expression of PMP22 and myelin protein zero. Western blotting conducted at 2 days of co-culture resulted in Ex-4-induced phosphorylation of a serine/threonine kinase AKT. These findings suggest that Ex-4 accelerates DRG neurite outgrowth, IFRS1 survival/proliferation and migration, and the myelination process in the co-culture via activating PI3K/AKT pathway.

### [3P-222]

**Estimation of time course of drug action by PK/PD model using a mathematical model of guinea pig ventricular myocyte implemented with $I_{Kr}$ inhibition by E-4031**

*Azumi Sagehashi[1], Yuna Nakanishi[1], Hiroto Nomura[1], Yukiko Himeno[1], Akira Amano[1]
([1]*Ritsumeikan Univ.*)

When analyzing the pharmacological effects of ion channel inhibitors on the heart *in vivo* or *in vitro*, it is difficult to determine at what point during an experiment the response is fully saturated and a steady state is reached. Especially for drugs at low concentrations, it is difficult to follow the time course and to compare the results for each concentration. Therefore, based on the concept of pharmacokinetics/pharmacodynamics (PK/PD), we developed a simulation model to reproduce the reaction time course of a hERG ($I_{Kr}$) channel inhibitor, E-4031. Three PK/PD models, each consisting of two compartments, were generated: *in vivo* (bolus administration), (2) *in vivo* (5-minute intravenous infusion), and (3) *in vitro* (Langendorff perfusion). Drug effects were evaluated using a mathematical model of ventricular myocyte implemented with the $I_{Kr}$ model, which incorporates inhibition by E-4031, to confirm the reproducibility of the experimental data obtained from guinea pigs. As a result, we could discriminate the time until the drug reached the target and the time until action potential duration prolongation was observed, respectively.

### [3P-223]

**Withdrawn**

### [3P-224]

**Microbial dielectric measurement method for drug screening**

*Shingo Murakami[1], Yuuji Kageyama[1], Akira Kimura[1], Akira Ito[2], Tetsuro Horie[3], Genki Ogata[4], Yasuaki Einaga[4], Hiroaki Suzuki[2] ([1]*Department of Electrical, Electronics, and Communication Engineering, Faculty of Science and Engineering, Chuo University,* [2]*Department of Precision Mechanics, Faculty of Science and Engineering, Chuo University,* [3]*School of Life Dentistry, The Nippon Dental University,* [4]*Department of Chemistry, Faculty of Science and Technology, Keio University*)

The effects of candidate compounds on viruses in drug screening have been measured indirectly by infecting cultured cells. To develop a new method to measure their direct pharmacological effects on the virus, by applying the permittivity measurement technique to aqueous solutions containing microorganisms, we developed a non-invasive and label-free method to evaluate the amount of lipid bilayer of microorganisms with permittivity. Using a measurement cell created with a 3D printer and black platinum electrodes with increased surface area and reduced electrode polarization, our method was tested with the measurement of the dielectric constant of yeast, which has a lipid bilayer like viruses but is easy to handle in experiments. The measured dielectric constant of KCl aqueous solution with yeast showed increases in dielectric constants over a broad band of $10^4$-$10^7$ Hz from those without yeast. This result was attributed to the improvement that the effect of electrode polarization on the dielectric constant was suppressed below $10^5$ Hz by our measurement cell, allowing the dielectric properties of the yeast membrane to be isolated. This method is characterized by the ability to measure the direct effects of drugs on microorganisms without culture cells and damage to microorganisms. Therefore, this method is expected to be used in drug screenings in the next pandemic.

### [3P-225]

### Deep learning-based simultaneous prediction of dose-response curves for ion channels

*Jaekyung Song[1,2], Yu Jin Kim[2], Chae Hun Leem[1,2] ([1]*Department of Physiology, University of Ulsan College of Medicine,* [2]*Department of Physiology, Asan Medical Center*)

In the new drug development, CiPA (comprehensive *in vitro* pro-arrhythmia assay) becomes a new paradigm for cardiac toxicity assay. For *in-silico* assay in CiPA, a dose-response curve is required for each ion channel, which can only be obtained experimentally using ion channel expressed in cell lines. CiPA requires data from the multi-ion channels. Since ion channel expressed in cell-line is not quite the same as in natural cell, the experiments must be time consuming and inherently contain errors. We tested possibility that the dose-response curves for the multi-ion channels can be simultaneously identified in natural cells containing the required ion channels. Firstly, we created the pulse protocol to generate the current data instead of the experimental data. We applied O'Hara-Rudy (ORD) human ventricular cell model to the protocol to generate 500,000 data. We trained our deep learning-based conductance prediction network with 300,000 train data and 100,000 validation data, and tested the model with 100,000 data. We call the trained network 'Block Prediction Network' (BPNet). BPNet can identify the conductance with the correlation coefficient, 0.999 for seven ion channels. We then generated current data for each drug using dose-response data for 12 drugs published in CiPA for testing. BPNet can find the same dose-response for each ion channels. Our method has two major advantages. The first is that the drug effect identification can be considerably accelerated. For example, if an experiment is performed five times at four different concentrations in each of the seven ion channels, a total of 140 experiments must be performed. Our method only requires 20 experiments in natural cell and can reduce the number of the experiments to 1/(the number of ion channels). Second, we found we can identify the drug effect on the late sodium without ATX II (anemone toxin II) treatment. Our method needs to be verified with the real experiments, however, clearly showed the possibility to reduce the time, effort, and cost and to change the paradigm of new-drug development.

### Poster Presentation

[3P]
**Medical education, Medical histology**

March 16 (Thu.), 12:10 - 14:10, Poster Room

### [3P-226]

### An attempt to educate students about the “Structure and Function of the Human Body” using VR and AR teaching materials during the COVID-19 pandemic

*Hidetusugu Kohzaki[1] ([1]*Faculty of Nursing, Shumei University*)

[Purpose] The COVID-19 pandemic made it necessary to provide first year nursing students with remote access to lectures on "Structure and Function of Human Body; (SFHB)" via Google Classroom. However, I had doubts about whether such remote lectures would be effective in teaching three-dimensional anatomy, and considered introducing VR/AR teaching materials. Furthermore, with the aim of creating rubrics with VR/AR teaching materials, we conducted preliminary effect measurements and reported them. [Method] Students (77) were asked to give their impressions in an anonymized questionnaire using VR/AR teaching materials, similar to the videos that are used in SFHB, and 38 responded (valid response rate, 49.4%). The evaluation was based on comparing the responses to questionnaires about the teaching materials and class improvement questionnaires, student feedback after each lecture, and the level attained in examinations with those of face-to-face lectures. In addition, the rubric incorporated evaluation scales, evaluation viewpoints, and evaluation criteria. The university is a member of the public transmission security deposit system for educational purposes. This research was reviewed by the research ethics committee of the Faculty of Allied Health Sciences, Yamato University (YHA2015-11).[Results] 1. Similar to conventional videos, 86.8% of the evaluations of VR/AR teaching materials were reported by students as "very interesting" and "interesting." 2. When asked if they would like to use these materials in future lectures and preparations for national exams, 52.6% said they would, and 5.3% said they were unnecessary. 3. In the questionnaire, there were statements such as "because it is easy to understand" and "VR is easier to remember". On the other hand, students expressed opinions such as "printing is enough". The scores in the class improvement questionnaire were 2.95–3.00 out of 3 points for all items. The rubric created from the above achieved "acceptance of VR and AR", "acquisition of knowledge" and "three-dimensional understanding", but "engagement" was 52.6%. On the other hand, the students found "filling in blank maps" difficult. In addition, when we asked about XR teaching materials for subjects other than SFHB, pathology was the most popular, followed by maternity, geriatrics, and physical assessment. [Discussion] A virtual space service called “Metaverse” is expected to increase in popularity. In this survey, students were interested in VR/AR, but the teaching materials used were not necessarily designed for nurses, so "engagement" was 52.6%. According to a survey by the Japanese Nursing Association, 9.2% of students use VR/AR in their lectures, which, while still small. So we decided to use VR/AR teaching materials for nurse training to improve engagement. This research was partially founded by JSPS KAKENHI JP21K10699. I thank Dr. Tetsu Terada, too.

### [3P-227]

### Spiritual intelligence and spiritual practices in students of first year undergraduate course in a medical college in India

*Bhalendu Suryakant Vaishnav[1], Jagdish Varma[2], Anusha Prabhakaran[3], Jaishree Ganjiwale[4], Nirav Vaghela[5], Rutam Vaishnav[6], Smruti Vaishnav[7] ([1]*Professor and Head, Department of Medicine, Pramukhswami Medical College, Bhaikaka University, Karamsad, Gujarat, India,* [2]*Professor, Department of Psychiatry,Pramukhswami Medical College, Bhaikaka University, Karamsad, Gujarat, India,* [3]*Professor and Head, Department of Psychiatry, Pramukhswami Medical College, Bhaikaka University, Karamsad, Gujarat, India,* [4]*Associate Professor Department of Public Health, Pramukhswami Medical College, Bhaikaka University, Karamsad, Gujarat, India,* [5]*Professor, K.M.Patel Institute of Physiotherapy, Bhaikaka University, Karamsad, Gujarat, India,* [6]*Undergraduate student, Pramukhswami Medical College, Bhaikaka University, Karamsad, Gujarat, India,* [7]*Professor and Head, Department of Obstetrics and Gynecology, Pramukhswami Medical College, Bhaikaka University, Karamsad, Gujarat, India*)

Spiritual intelligence (SI) is reported to provide meaning-giving and context-creator functionality, besides enhancing academic engagement and care competence. We studied spiritual intelligence, daily spiritual practices and work satisfaction of 127 students of first year undergraduate course in an Indian medical college. Spiritual Intelligence Self-Report Inventory (SISRI 24) was used which measures overall spiritual intelligence and its 4 components: Critical existential thinking, Personal meaning production, Transcendental awareness, Conscious state expansion. Eighty five percent students had moderate/high SI. Scores: Critical existential thinking (17.0/28), Personal meaning production (12.9 /20) Transcendental awareness (17.8/28), Conscious state expansion (9.8./20). SI was significantly associated with work satisfaction(p=0.006). More than 80% students were regularly undertaking spiritual practices (prayer, meditation, etc). Students expressed that spirituality is helpful in professional growth. Conclusion: First year medical students have moderate/high spiritual intelligence associated with good work satisfaction. Students consider spirituality as essential component of education.

### [3P-228]

### A survey of trends in curriculum evaluation models

*Mitsuo Nagane[1], Fuminobu Tamalu[1], Hajime Hirasawa[1], Kazunori Yoshimura[2], Naofumi Miwa[1] ([1]*Saitama Med. Univ.,* [2]*Nihon Inst. Med. Sci.*)

Curricula are developed in accordance with philosophy and objectives for every educational institutions, and assessed constitutively by curriculum evaluation. Several theoretical curriculum evaluation models have been proposed and applied so far. They include Experimental model, Kirkpatrick’s four model, Logic model, CIPP model. It is of significance for educators to know about trends in current evaluation models. Here, we conducted literature search using Pubmed to find literatures that used four theoretical curriculum evaluation models, and analyzed search results per year for each theoretical model for over twenty years. Search results for every models increased with year, and the search for experimental model obtained the largest number (>10000 per year) among the four models. We investigated the category region of education, and found that the region of “medicine” applied experimental model for curriculum evaluation more frequently than the other regions such as “Nursing” and “Pharmacy”. These results provide helpful information for every educators in charge of curriculum evaluation on future occasions. There are no COIs to declare.

### [3P-229]

### A use of the Nudge theory for improving the attitude of university students towards infection prevention

*Chiaki Itami[1], Kayo Aoba[1], Mitsuo Nagane[1], Naofumi Miwa[1] ([1]*Saitama Medical University, Department of Physiology*)

At Saitama Medical University, students and teachers are required to take thorough countermeasures against new coronavirus infection in face-to-face practical courses. Students are mandatory to care about physical distancing, and avoid the face-to-face conversation. Furthermore, they should wear masks and protective glasses (goggles) or a face shield. However, we often come across the scene where infection prevention of students is compromised. Recently, the Nudge theory, a concept for decision making of consumers, has often been adopted by many private companies as well as public policies. Here, we utilized the Nudge theory in our physiological classes to raise students' awareness of infection prevention. The second year students take physiological practical training classes comprising four themes. We prepared full-size photo panels of teachers and settle them in the entrance of the room. Students voluntarily submitted the questionnaire after completing each theme. We evaluated these self-administered questionnaires using a five-point Likert scale, and found that the presence of the panels slightly increased the Likert score on awareness of infection prevention.There are no COIs to declare.

### [3P-230]

### Development of educational materials to teach children about the pumping function of the heart

Akihiro Hazama[1], *Kei Kakinouchi[2,1] ([1]*Department of Cellular and Integrateive Physiology, Fukusima Medical University, School of Medicine,* [2]*Department of Otorhinolaryngology, Fukusima Medical University, School of Medicine*)

We are trying to educate children about health based on our knowledge of physiology. We have created an educational material that takes advantage of the fact that the volume of one beat of an easily available fuel pump is 80 mL, which is almost equal to the stroke volume of the heart. The idea is to fill a clear plastic container with 5 L of water, which is equivalent to the blood volume of an adult, and pump all of the water out in one minute. This teaching material enables children to understand that the heart pumps out the equivalent of the entire blood volume in one minute, and that although the one-minute pumping process is tiring, the heart is doing it all its life. Two plastic containers filled with equal amounts of water are used to represent the whole body and the lungs. Two children are asked to push each pump for one minute and compare the volume of water in the right and left containers. The children will understand that if the right and left heart pumps do not send out the same amount of water, water will accumulate in the whole body and lungs. This type of teaching material teaches children how the heart works.

### [3P-231]

### Changes in the treatment of physiology in Japanese elementary and secondary education

*Kei Kakinouchi[1,2], Akihiro Hazama[1] ([1]*Fukushima Medical University schoool of medicine department of cellular and integrative physiology,* [2]*Fukushima Medical University schoool of medicine department of otolaryngology*)

We believe that the dissemination of physiological knowledge may help to improve the health of all citizens.In this study, we examined the evolution of education on the physiology of the human body. Before World War II, there was a major course on "Physiology and Hygiene" along with "Animals," "Plants," and "Minerals" in the subject "Natural History" in junior high schools under the old education system. In "Physiology and Hygiene," anatomy, physiology, and hygiene of the human body (including disease prevention, nutrition, and knowledge of individual diseases) were explained in a practical approach. After World War II, the Japanese educational system was reorganized and "Natural History" was integrated into Science along with "Physics and Chemistry". As a result, "Physiology and Hygiene" became one of the fields of Biology. In addition, part of "Physiology and Hygiene" was divided into "Home Economics" and "Health and Physical Education. This presentation will introduce the changes in the elementary and secondary education system regarding "Physiology" and its educational content.

### [3P-232]

### A microprocessor-based low-cost, closed loop electrophysiological data recording system for laboratory training of physiology of medical students

*Yoshiya Matsuzaka[1] ([1]*Tohoku Med Pharm Univ*)

Laboratory training of electrophysiology necessitates costly instruments and/or shielded recording room to obtain noise-free electrophysiological signals (EMG, EEG etc). Yet, the high cost of commercial apparatus often precludes obtaining sufficient number of units for students' training. This is not a desirable situation because it means, in group teaching, only a limited number of students have the chance to directly experience physiological experiments. In addition, the necessity for electrostatic shielding restricts the place where recording is done. Therefore, with affordability and noise attenuation as major requirements, I developed a relatively simple and inexpensive toolkit for medical students' training. The toolkit consists of a microprocessor based task control system that allows users to freely design various stimulus-delivery protocols and simultaneously record electrophysiological signals. To record small-amplitude signals from human subjects, I developed an 8 channel, differential amplifier board that works with the microprocessor. This amplifier recorded clear electroencephalographic signal (EEG) even without electrostatically shielded room and dedicated grounding usually required by commercially available apparatus. Taking advantage of these devices, our medical students were able to directly experience recording clear EEG such as alpha blocking of spontaneous EEG and evoked potentials. This teaching toolkit is expected to serve both in student education and in research project by faculty members.

### Poster Presentation

[3P]
**Study Methodology**

March 16 (Thu.), 12:10 - 14:10, Poster Room

### [3P-233]

***In vivo* large-scale imaging of the mouse brain through novel cranial window utilizing PEO-CYTOP nanosheet**

*Taiga Takahashi[1,2,3], Hong Zhang[4,5], Masakazu Agetsuma[6], Junichi Nabekura[3,6], Kohei Otomo[1,2,7], Yosuke Okamura[4,5,8], Tomomi Nemoto[1,2,3] ([1]*Biophotonics Research Group, Exploratory Research Center on Life and Living Systems (ExCELLS), National Institutes of Natural Sciences,* [2]*Division of Biophotonics, National Institute for Physiological Sciences, National Institutes of Natural Sciences,* [3]*School of Life Science, The Graduate University for Advanced Studies (SOKENDAI),* [4]*Department of Applied Chemistry, School of Engineering, Tokai University,* [5]*Micro/Nano Technology Center, Tokai University,* [6]*Division of Homeostatic Development, National Institute for Physiological Sciences, National Institutes of Natural Sciences,* [7]*Graduate School of Medicine, Juntendo University,* [8]*Graduate School of Engineering, Tokai University*)

*In vivo* two-photon imaging in animal brains with a broad field of view has revealed functional connectivity between brain regions. The "open skull" method has been employed to make a cranial window for observing mouse brains by two-photon microscopy. This method excises a part of the mouse skull and replaces it with a glass coverslip. However, the size of the cranial windows is typically restricted to 5 mm in diameter to avoid the pressure on the brain tissue by a flat glass coverslip. Here, we proposed a large cranial window (over 7 mm in diameter) utilizing polyethylene-oxide coated CYTOP (PEO-CYTOP) nanosheet as a flexible sealing material of a cranial window for *in vivo* two-photon imaging [Takahashi et al. *iScience*, 2020]. PEO-CYTOP nanosheet is ~130 nm thickness and had has a hydrophilized adhesive surface, which allowed for strong adhesion to the brain surface and suppressed bleeding on the surface. Moreover, we prepared large cranial windows utilizing PEO-CYTOP nanosheet and achieved *in vivo* multi-scale imaging of neuronal structures and $Ca^{2+}$ activity at high resolution. The transparency of the large cranial window was monitored over half a year without severe inflammation.

### [3P-234]

**Exploring photoacoustic contrast agents for calcium imaging**

*Yuushi Yamamoto[1], Kyono Daiki[1], Gomi Ryota[1], Shoji Ichiro[1], Murakami Shingo[1] ([1]*Chuo University Faculty of Science and Engineering*)

Intracellular Calcium measurement has been conducted in a number of studies with the Calcium fluorescence imaging technique. However, because the measurement signal of the light is highly scattered and absorbed in the tissues, measurement of intracellular Calcium concentration far from the tissue surface has been difficult with the fluorescence imaging technique. The purpose of the present study is to establish a new Calcium imaging method at depth by examining the photoacoustic effect with calcium-dependent photoacoustic reagents. We constructed a photoacoustic imaging system with a NIR short pulse laser light source, an optical fiber designed to provide efficient positioning of excitation light, and an ultrasonic transducer. A silicon tube type with low photoacoustic noise was selected to contain Calcium and the agents. To examine the feasibility of measuring calcium ion concentrations with the photoacoustic effect, several agents (e.g. Chlorophosphonazo-III, Arsenazo III), which NIR absorbance may change according to calcium concentrations, were selected for the photoacoustic measurement. The agents were dissolved in solutions with various calcium concentrations, sealed in a tube, and measured with the photoacoustic imaging system. The result shows clear but different correlations between calcium concentrations and ultrasound images by photoacoustic effects. This result suggests that an understanding of calcium dynamics at greater depths may be obtained with our photoacoustic imaging method and may lead to a further understanding of calcium signaling at depths.

### [3P-235]

**RFP-dependent Cre recombinase using nanobodies and DARPins**

*Ayumu Inutsuka[1], Sho Maejima[2], Hiroyuki Mizoguchi[3], Ryosuke Kaneko[4], Keiko Takanami[5], Hirotaka Sakamoto[2], Tatsushi Onaka[1] ([1]*Jichi Medical University,* [2]*Okayama University,* [3]*Nagoya University,* [4]*Osaka University,* [5]*Nara Women's University*)

Recent great advances in physiology rely on the regulation of gene expression in specific cells. Various Cre driver lines are available for the genetic manipulation of specific cells in mice. However, the generation of transgenic large animals remains challenging, and it is important to establish specific gene expression regulation techniques using only viral vectors for translational research. Here, we created RFP-dependent Cre recombinases (Cre recombinase dependent on RFP: Cre-DOR) by utilizing Split-Cre reunion via specific binding of nanobodies to red fluorescent proteins (RFPs). The functional binding units for monomeric RFPs are different from those for polymeric RFPs. In estrogen receptor-beta (Esr2)-mRFP1 mice and gastrin-releasing peptide receptor (Grpr)-mRFP1 rats, we confirmed that Cre-DOR can be used for anterograde tracing of the neural projection from RFP-expressing specific neurons. Our results provide a method for manipulating gene expression in specific cells expressing RFPs and expand the repertory of nanobody-based genetic tools. We ultimately aim to create Cre-DOX (Cre recombinase dependent on protein X), a target protein X-dependent Cre recombinase for any given target protein X.

### [3P-236]

**Development of new imaging system for large-scale measurement of synaptic input signals**

*Satoru Kondo[1], Yu Takiguchi[2] ([1]*IRCN, UTIAS, The University of Tokyo,* [2]*Central Institute, Hamamatsu Photonics K.K*)

Recent advances in the optical measurement methods have made it possible to record neuronal activity with high temporal and spatial resolution in living animals. In particular, two-photon imaging technique allows micro-measurements of neuronal functions and structures even at a single synapse level. The current two-photon microscopy uses point-scanning to construct a single frame image and this procedure is a limiting factor for the image acquisition rate, especially for the large-scale imaging. To improve imaging acquisition rate, scanless wide-field excitation could be one of solutions. However, wide-field two-photon excitation with conventional pulsed laser increases the out-of-focus excitation and dramatically decreases the signal-to-noise ratio. To overcome this problem, we developed a new scanless imaging system utilizing the temporally focused pulsed laser. A diffraction grating separates the ultra-short pulse into its constituent wavelengths. Since the objective lens collimates and recombines the separated spectral components only at the focal plane, two-photon excitation occurs within the strictly restricted focal volume.Our new imaging system with temporal-focusing technique will be useful to understand not only the synaptic integration principle but also the dynamic properties of synapses, such as the reorganization of synaptic inputs during development, pathological changes of synaptic inputs in psychiatric disorders, and reorganization of synaptic inputs at learning.

### [3P-237]

**Droplet Electroporation of transfection by nanosecond electric pulse for establishment of iPS cells**

*Rika Numano[1,2], Takeshi Niki[1], Ryuto Shinozaki[2], Minako Matsuo[1], Hirofumi Kurita[2], Wado Akamatsu[3], Kojiro Matsumoto[4] ([1]*Electronics-Inspired Interdisciplinary Research Institute (EIIRIS), Toyohashi University of Technology,* [2]*Department of Applied Chemistry and Life Science, Toyohashi University of Technology,* [3]*Center for Genomic and Regenerative Medicine, Juntendo University,* [4]*Nepa Gene Co.,Ltd.*)

Our group have developed droplet electroporation (dEP) method and device for transfection to reduce cell damage and the amount of necessary materials, cells and transgene. In 2015, the water-in-oil(W/O) droplet electroporation system was previously reported using dielectric oil and an aqueous several micro liter droplet containing mammalian cells and transgene DNA. An aqueous droplet between a pair of electrodes by Coulomb force in dielectric oil and short cutting during applying a DC electric field. Then, the instantaneous short circuit via the droplet facilitates gene transfection by this W/O droplet electroporation during bouncing motion. In this study the stability of performance has been improved according to holding the oil-free droplet between a pair of electrodes and introducing a pulsed electric field due to electric discharge into droplet. Using this dEP device exogenous genes are introduced into mammal cells by exponential nano-second electric pulse with 1-3kV derived from arc discharge. As a result, dEP device succeeded plasmid DNA to transfer into both adherent cells (HEK293) and floating cells (HL60 cells) by approximately 40-60% transfected efficiency. This device takes advantage to delivery some kinds of genes so effective into cell that it is applied to establish iPS cells by transfect Yamanaka's factor into LCL cell lines with high efficiency.

### [3P-238]

### Quantitative Assessment of Microtremors in Mice Using DeepLabCut

*Takamasa Yoshida[1], Toshihiro Hayashi[1] ([1]*Dept Physiol, Teikyo Univ Sch Med*)

Although essential tremor is a movement disorder and is thought to be caused by abnormalities in the cerebellar system, its mechanism is not fully elucidated. Thus, it is required to detect and quantify tremor in animal models of essential tremor to understand the mechanism of essential tremor. In this study, we used a mouse model of tremor provoked by harmaline, analyzed videos of the animals' tremor using deep learning, and examined whether the tremor component could be detected from the videos. We used a system developed in our previous study (Ajima et al., 2021, J. Neurosci. Methods, 351, 109074), in which mice were placed in a 15 cm square acrylic box and their movements were recorded by the tremor detector using piezoelectric elements. Simultaneously, the mice's behavior was recorded by a camera on the top, and the video data was analyzed. Mice were placed in the apparatus before and after harmaline administration, and their behavior was recorded for 5 minutes each. The trajectories of the mice were estimated from the video using DeepLabCut (Mathis et al., 2018 Nat. Neurosci.), and the vibration components derived from the tremor were extracted from the time series of the trajectories. The tremor peak was found from the power spectrum of this time series, and a tremor index was calculated. As a result, tremor induced by less than normal doses of harmaline could also be detected. This provides a new method to evaluate microtremors in mouse models of essential tremor.

### Poster Presentation

[3P]
**Others**

March 16 (Thu.), 12:10 - 14:10, Poster Room

### [3P-239]

### Unpleasant emotions induced by olfactory stimulation decreases cognitive performance with the Stroop color-word test

*Kana Endo[1], Arisa Takeuchi[1], Kaede Morimoto[2], Miu Fujioka[1], Tatsuya Miyaji[1], Ryuichi Kasuya[1], Mitsunori Miyazaki[1] ([1]*Hiroshima University,* [2]*Osaka Health Science University*)

The effects of emotional changes induced by olfactory stimulation on cognitive function have not been fully elucidated. We examined the effects of pleasant and unpleasant emotions induced by olfactory stimulation on cognitive function in 13 young women (21.7±0.2 yrs.). They were exposed to odors (Peach, Isovaleric acid or Odorless paraffine) that elicited pleasant, unpleasant or neutral emotions followed by the Stroop color-word test (SCWT) for cognitive assessment. Odor concentration was set to the lowest level at which subjects could recognize odor's definite character. Emotional changes to each odor stimulus were subjectively evaluated with 11 grades. Skin blood flow in the left cheek was assessed by laser doppler flowmetry to estimate an emotional status. Unpleasant odor stimulus, but not pleasant odor stimulus, significantly lengthened the total SCWT time, whereas Odorless paraffine did not. The unpleasant odor stimulus, but not pleasant odor stimulus, increased the skin blood flow. A significant negative correlation was found between emotional score for unpleasant odor and the total SCWT time. These results suggest that the elicitation of unpleasant emotions decreases cognitive performance in young women. (COI:NO)

### [3P-240]

### a solution replacement technique in microwells for Digital ICA ®

*Yosuke Horiuchi[1], Yoichi Makino[1], Hiroshi Ueno[2], Kazuhito Tabata[2], Hiroyuki Noji[2] ([1]*Technical Research Institute, TOPPAN INC.,* [2]*Department of Applied Chemistry, School of Engineering, The University of Tokyo*)

[Background]These days, there is an increasing need for a highly sensitive detection method of biological molecules in the field of endocrine, immunity, nutritional and metabolic physiology and so on to conduct further analysis of and develop a new remedy for various diseases. We have "Digital ICA®" that combines digital counting using a device with many microwells and an invasive cleavage assay (ICA) that detects target nucleic acids. aM ($10^{-18}$) concentration of DNA can be detected by the method. We are currently developing this method to detect proteins with high sensitivity. To detect proteins, magnetic beads are used and needed to be washed several times in the process. In Digital ICA®, each microwell is closed with oil to be independent on each other. Therefore, it is necessary to replace the solution in the wells after closed with oil to wash each bead. [Methods]We investigated an efficient method of replacing the solution in microwells closed with oil with another solution. And we also tried to detect proteins by this method. [Results/Conclusion] Once the wells were filled with surfactant-containing solution, it could be replaced with another solution even if each well was closed with oil. In addition, the use of magnet seat made it possible to retain the beads in each well while replacing solution. Furthermore, we obtained a data suggesting that this method can detect proteins.

### [3P-241]

### Introduction of Brain/MINDS Data Portal

*Yuki Kobayashi[1] ([1]*RIKEN Center for Brain Sience*)

The Brain/MINDS project was launched in 2014. The aims of this project are overcoming human mental and neurological disorders and advancing information processing technology.The Brain/MINDS project is working primarily with small monkeys, called common marmosets, to elucidate the neural circuits responsible for higher brain functions in primates. Common marmosets are evolutionarily closer to humans than to other mammals such as mice and rats, and have a well-developed frontal lobe, which is responsible for higher brain functions, and a brain structure and function similar to humans. Therefore, it is possible to study disease-related neural circuits in detail in the common marmoset, which is expected to contribute to a more detailed understanding of the mechanisms of human diseases and the development of new treatment methods. In addition, the structural and functional data of the marmoset brain obtained through this project will be made public and shared with researchers around the world, contributing to the development of data-driven science and the advancement of information processing technology.The Brain/MINDS Data Portal has been launched for sharing the data and knowledge being produced in the Brain/MINDS project. The portal aims to provide integrated knowledge for public use and original data for open research and collaboration. In the portal, marmoset reference atlas, marmoset brain MRI data, connectomes of the prefrontal cortex, marmoset gene atlas, marmoset calcium imaging data ,and marmoset brain electrocorticography data are available. We will present the overviews, available dataset, and future direction of the Brain/MINDS Data Portal.

### [3P-242]

### Potential of a new photothermal therapy against amyloid-β protein based on nanodiamonds

*Miwa Shintani[1], Shin-ichiro Yanagiya[2], Hiroki Takanari[2] ([1]*Tokushima University Graduate School of Science and Technology for Innovation,* [2]*Tokushima University*)

As a novel therapies for Alzheimer's disease targeting amyloid-β (Aβ) protein, photothermal therapy (PTT) using metal nanoparticles has been actively investigated. We investigated whether biocompatible nanoparticle, nanodiamond (ND) and NDs with nitrogen-vacancy center (NVND), could be used for PTT against Aβ. ND had little effect on cell viability even when administered at high concentrations to HeLa cells. When ND and NVND colloids were mixed with Tris-HCl (1 M, pH 8.0) containing human Aβ (1-42), the nanoparticle colloids became less stable and aggregated. Irradiation of an ultrashort pulse laser (800 nm, 600 mW) to the mixed sample with ND caused a significant increase in the temperature, while no temperature increase was found in the mixed sample with NVND. Western blot experiments using supernatant of the mixed samples revealed that NVND significantly reduced Aβ in the supernatant, and ND also transiently reduced Aβ from the supernatant immediately after laser irradiation. The results indicated that ND and NVND might be able to removed Aβ from the samples either by interfering directly with Aβ protein or by the photothermal effect of laser irradiation.

### [3P-243]

### Development of data logger with wireless monitor for recording neuronal activity in monkeys

*Ryoi Tamura[1] ([1]*University of Toyama*)

We have recorded neural activity from the hippocampus of freely moving monkeys, sending the recorded signals to the post-processing equipment via a transmission cable attached to the head of the monkey. This recording method often caused troubles such as interference of animal's behavior by the cable. Telemetries and data loggers are practical solutions to overcome these problems. While telemetries are suitable for real-time on-line monitoring of neural activity, stable recording is sometimes difficult when animals actively move around in a large environment. Data loggers, on the other hand, do not suffer from such a problem, but they do not allow on-line monitoring. In the present study we developed a device that combines the advantages of both. The device was divided into three parts: amplification, data storage, and data transmission units. In the amplification unit, input signals (4Ch) were amplified by instrumentation amplifiers, band-pass filtered, and further amplified by post stage amplifiers. In the data storage unit, outputs from the amplification unit were AD-converted by a PIC microcontroller and stored on a SD card. In the data transmission unit, the outputs from the amplification unit were sent to a receiver by a Bluetooth transmitter. The performance of this device was evaluated by recording neuron activity in one animal; as results, we found the stored signals on the SD card were equivalent quality to those obtained with the conventional cabling method, and (2) the signals were faithfully monitored on-line.

### [3P-244]

### The integrated understanding of the brain function -Lesson from the planarian possessing the basic design of the brain-

*Takeshi Inoue[1], Kiyokazu Agata[2], Satoshi Matsuo[1] ([1]*Tottori University,* [2]*National Institute for Basic Biology*)

The brain acts as an information-processing center that integrates complex environmental signals and determines behavioral strategies. However, much remains unmasked about how the brain orchestrates stimulus perception and its association with a corresponding neuronal response. The freshwater planarian is one of the most primitive brain-possessing organisms. Thus, its brain and extraordinal regenerative capacity provide a unique opportunity to understand the central mechanisms of the brain and insight into regenerative medicine. First, we comprehensively investigated the neural and physiological mechanisms of multi-sensory systems and responsive behaviors by combining the quantitative behavioral analysis and RNAi of nervous-related genes. Next, to understand the restoration of brain function associated with environmental signals and brain regeneration, we compared the two groups that regenerated their brains under different environments (normal conditions and light blocked). As a result, the light-blocked group could not recover normal light-responsive behavior, whereas the group with light stimulation could recover normal behavior. We also identified the novel neuropeptide gene of which the expression level was up-regulated by environmental light stimuli. Finally, we found that the novel neuropeptide-expressing neurons interplay the neuronal signal transduction from visual neurons to visual center neurons during brain regeneration. Therefore, we propose that the planarian is the ideal model to reveal the multi-modal system of the sensory-brain-motor circuits and the relationship between morphogenesis and function in the brain from the molecular to the individual level.

### [3P-245]

### The duration of the preemptive analgesic effect of high-frequency transcutaneous electrical nerve stimulation in rats with acute inflammatory pain

*Hideshi Ikemoto[1], Naoki Adachi[1], Takayuki Okumo[1], Tengyang Ni[1,3], Chuluunbat Oyunchimeg[1], Hiroyuki Horikawa[1,2], Shiyu Guo[1], Yanqing Liu[1,3], Tadashi Hisamitsu[1], Masataka Sunagawa[1] ([1]*Department of Physiology, School of Medicine, Showa University,* [2]*Faculty of Arts and Sciences at Fujiyoshida, Showa University,* [3]*Institute of Translational Medicine, Medical College, Yangzhou University*)

In this study, we investigated the duration of the preemptive analgesic effects of high-frequency transcutaneous electrical nerve stimulation (HT, 100 Hz), a non-invasive technique to relieve pain, using rats with acute inflammatory pain. Wistar rats were divided into 5 groups: a Control, a Formalin (For), formalin-injected immediately after the HT treatment (HT + For), formalin-injected 30 min after the HT (HT(30) + For), and formalin-injected 60 min after the HT (HT(60) + For) groups, respectively. Formalin (1%, 50 µl) was injected into the right hind paw. HT was applied for 30 min prior to the formalin injection. The total time spent in pain-related behavior (PRB. licking, flinching and lifting) was quantified for 1 hour after the injection, and then phosphorylated extracellular signal-regulated kinase (pERK) levels, a marker of neural activation, in the spinal dorsal horn was examined by immunohistochemistry. The increased durations of PRB in the second phase and expression of pERK in the For group compared to Control group were significantly suppressed in the HT + For, HT(30) + For, and HT(60) + For groups. In conclusion, the duration of preemptive analgesic effect of HT was at least 120 min after HT treatment.

### Late Breaking Abstracts

Day 1
(March 14, 12:10 - 14:10)

[1LBA] Neurophysiology, Neuronal cell biology - Plasticity
[1LBA] Neurophysiology, Neuronal cell biology - Neural network
[1LBA] Neurophysiology, Neuronal cell biology - Higher brain function
[1LBA] Neurophysiology, Neuronal cell biology - Motor function
[1LBA] Molecular physiology, Cell physiology - Ion channels, Receptors
[1LBA] Molecular physiology, Cell physiology - Others
[1LBA] Oral physiology
[1LBA] Circulation
[1LBA] Autonomic nervous system
[1LBA] Physical fitness and sports medicine
[1LBA] Pathophysiology

### Late Breaking Abstracts

[1LBA]
**Neurophysiology, Neuronal cell biology**
**Plasticity**

March 14 (Tue.), 12:10 - 14:10, Poster Room

### [1LBA-001]

**Length impairments of the axon initial segment in rodent models of attention-deficit hyperactivity disorder and autism spectrum disorder**

*Takeshi Yoshimura[1], Noriyoshi Usui[2], Xiaoye Tian[1], Wakana Harigai[1], Ko Miyoshi[1], Koh Shinoda[3], Junya Tanaka[4], Shoichi Shimada[2], Taiichi Katayama[1] ([1]*Department of Child Development and Molecular Brain Science, United Graduate School of Child Development, Osaka University,* [2]*Department of Neuroscience and Cell Biology, Graduate School of Medicine, Osaka University,* [3]*Division of Neuroanatomy, Department of Neuroscience, Yamaguchi University Graduate School of Medicine,* [4]*Department of Molecular and Cellular Physiology, Graduate School of Medicine, Ehime University*)

The axon initial segment (AIS) is a structural neuronal compartment of the proximal axon that plays key roles in sodium channel clustering, action potential initiation, and signal propagation for neuronal outputs. Mutations in constitutive genes of the AIS, such as *ANK3*, have been identified in patients with neurodevelopmental disorders. Nevertheless, morphological changes in the AIS in neurodevelopmental disorders have not been characterized. In this study, we investigated the AIS length in animal models of attention-deficit hyperactivity disorder (ADHD) and autism spectrum disorder (ASD). We observed abnormalities in AIS length in the rodent models. In ADHD model mice and rats, we observed shorter AIS length in the medial prefrontal cortex (mPFC) and primary somatosensory barrel field (S1BF) neurons. In ASD model mice, S1BF neurons had shorter AIS lengths. These results demonstrate that AIS length is altered in specific brain regions in ADHD and ASD rodent models, and AIS abnormalities may be conserved across species. Our findings provide novel insight into the potential contribution of the AIS to the pathophysiology and pathogenesis of neurodevelopmental disorders.

### [1LBA-002]

**Routine hypercapnic challenge affects the size of phrenic motoneurons of cervical spinal hemisection rat**

*Kenta Kawamura[1], Masaaki Kobayashi[1], Kazuhide Tomita[1] ([1]*Ibaraki Prefectural University of Health Sciences*)

After an individual experiences a cervical cord injury, the cell body's adaptation to the smaller size of phrenic motoneurons occurs within several weeks. It is not known whether a routine hypercapnic challenge can affects this adaptation of phrenic motoneurons. We investigated routine hypercapnic challenge affects the size of phrenic motoneurons of cervical spinal hemisection rat. The rats were divided into three groups: control, training, and intact. Within 72 h post C2 hemisection, the training group began 20min/day, 3 week hypercapnic challenge under awake conditions. After the 3-week challenge, the phrenic motoneurons in all of the rats were retrogradely labeled with horseradish peroxidase, and the motoneuron sizes in each group were measured. The average diameter and cross-sectional area of stained phrenic motoneurons as analyzed were significantly larger in the training group compared to the control group. The histogram distribution was unimodal. Our findings indicate that a routine hypercapnic challenge may increase input to phrenic motoneurons and alter the propensity for motoneuron adaptations. The authors declare no competing of interests.

### [1LBA-003]

**Timing dependence of associative responses on two inputs for medial and lateral dendrites in the rat hippocampal granule cells.**

*Tadayoshi Monden[1], Kazuhisa Kamei[1], Naoki Nakajima[1], Tadanobu Kamijo[2], Takesi Aihara[1] ([1]*Tamagawa University,* [2]*University of The Ryukyus*)

To investigate the association of inputs to granule cells (GCs) in the hippocampal dentate gyrus, we measured the dependence of associative response on the temporal timing between two inputs to medial (MD) and lateral dendrites (LD) by using electrical stimuli in granule cells. In this experiment, phase-differential stimuli were applied to MD and LD in the range of -40~40 msec below firing threshold using rat hippocampal slices, and fEPSPs were recorded from the cell body layer. As a measure of the response characteristics of the two-input association, we calculated the ratio of peak values between the additive waveform of fEPSPs for a single input to MD and LD and the measured waveform of fEPSPs by phase-contrast stimulation. The same experiments were also performed when GABA(A) or NMDA receptors were inhibited by picrotoxin (50 mM) or AP5 (25 mM) and when acetylcholine was filled up by carbachol (0.05 mM). The results in the naive state showed that linear responses were observed only when MD and LD were given inputs simultaneously, and nonlinear responses were observed for the other timings. When inhibitory connections were blocked, the overall trend of associative responses was closer to linear summation than in naive. The same trend was shown when acetylcholine was filled up. On the other hand, when NMDA receptors were inhibited, more nonlinearity was shown than naive at all timings. The same trend was also shown when acetylcholine was additionally filled up. Our results indicated that input association in the granule cells has the properties to detect the input coincidence to MD and LD, closely related to NMDA and GABA(A) receptors.

### [1LBA-004]

**Developmental trajectory of contextual learning:training-induced pathway-specific plasticity at dorsal CA1 synapses**

*Yuheng Yang[1], Yuya Sakimoto[1], Dai Mitsushima[1] ([1]*Department of Physiology, Yamaguchi University Graduate School of Medicine*)

Behavioral battery tests revealed multiple task-dependent critical periods in juvenile rats (Sakimoto et al. *Sci Rep* 2022). Although the learning performance was low in postnatal (PN) 16 days of age that was gradually improved from PN 17 to 20 days, suggesting a critical period for contextual learning with IA task. Using a paradigm of inhibitory avoidance (IA) task, here we further analyzed developmental trajectory of pathway-specific plasticity at dorsal CA1 synapses after the training. We subjected male rats to the IA task and prepared acute hippocampal slices for whole-cell patch clamp experiments, where we stimulated ECIII-CA1 or CA3-CA1 input fibers to analyze evoked excitatory postsynaptic currents (EPSCs). Compared to untrained controls, trained rats exhibited higher AMPA/NMDA current ratios at CA3-CA1 synapses of PN 30, but not PN 16 rats. Moreover, trained rats exhibited higher AMPA/NMDA current ratios at ECIII-CA1 synapses in PN 30 and PN 16 rats. These findings suggested the presence of critical periods in input-specific postsynaptic plasticity occurred after training. Understanding the development of pathway-specific and pre/post-specific plasticity at dorsal CA1 synapses could aid in controlling encoded memory.

### Late Breaking Abstracts

[1LBA]

**Neurophysiology, Neuronal cell biology**

**Neural network**

March 14 (Tue.), 12:10 - 14:10, Poster Room

### [1LBA-005]

### Stepwise formation of spatial context cells in anterior cingulate cortex and its cellular mechanism

*Yuki Murai[1], Ayaka Bota[2], Xinzhi Jiang[1], Akihiro Goto[1], Toru Takumi[2], Yasunori Hayashi[1] ([1]*Kyoto University Graduate School of Medicine,* [2]*Kobe University Graduate School of Medicine*)

It is widely believed that memories are formed initially in the hippocampus and then gradually transferred to various cortical regions, where the memories are stored for a long time. One of the cortical regions is Anterior Cingulate Cortex (ACC). The increase in the expression of c-fos, an immediate early gene product, by the retrieval of remote memories in ACC indicates the involvement of this region in the recall of remote memories. However, the cellular mechanisms of the transfer remain largely unknown. Therefore, we observed the cellular activity in ACC during a learning task by *in vivo* calcium imaging with a head-mounted miniature microscope for 9 days. Consequently, we found the spatial context cells (SCCs), which fire in a spatial context specific manner. The ratio of these cells increased dependent on the learning experience. The inhibition of the hippocampal neural activity with Designer Receptors Exclusively Activated by Designer Drugs (DREADD) suppresses the SCC formation, indicating that SCCs are formed depending on the activity of the hippocampus. As the functions of the hippocampus differ along longitudinal axis, we further studied hippocampal regions required for the SCC formation in detail by individually inhibiting the dorsal or ventral area of the hippocampus with DREADD. Consequently, we elucidated the contribution of specific hippocampus regions to the formation of SCCs. We also recorded the activity of c-fos expressing neurons, considered to be engram cells, in ACC to reveal the contribution of SCCs to the memory consolidation. As a result, the ratio of SCCs in c-fos expressing neurons was significantly higher than that in entire excitatory neurons, which indicates that SCCs are a part of memory engram. These results elucidate that spatial contexts are formed as remote memories in ACC dependent on the hippocampus, and its cellular mechanism.

### [1LBA-006]

### Effects of deep brain stimulation on medial prefrontal cortex in mouse models for psychiatric disorders

*Minobu Ikehara[1], Kazuhiko Yamamuro[1], Yuki Noriyama[1], Yasuhiko Saito[2], Manabu Makinodan[1] ([1]*Department of psychiatry, Nara Medical University,* [2]*Department of Neurophysiology, Nara Medical University*)

Recently, noninvasive neuromodulation by rTMS has been attracting attention as a treatment for psychiatric disorders, and it has been indicated for depression in Japan. In this study, we alternatively used deep brain stimulation (DBS), a neuromodulation technique, to locally stimulate the medial prefrontal cortex (mPFC) in mice because rTMS affects its entire brain. DBS (20 min/day for 6 days)-induced changes in social behaviors, fear memory, depressive symptoms, and activity- and anxiety-related behaviors were tested using the restraint stress model (RS), social defeat model (SD), and juvenile isolation model mice (jSI). RS and SD commonly showed depressive symptoms, which were improved by DBS. Social behaviors were decreased in jSI and SD, but improved with DBS. Next, we examined the effects of DBS on local neural circuits in mPFC using the whole-cell patch clamp technique. sIPSC input frequency to the LayerV/VI pyramidal cells was significantly increased by DBS stimulation (20 min/day for 6 days). An increase in sEPSC frequency and a decrease in sIPSC frequency were also observed in the LayerV/VI PV interneurons. These results suggest that DBS may affect PFC local circuits and ameliorate its relevant behavioral abnormalities in each mouse model.

### [1LBA-007]

### Molecular screening for the formation of axonal branching pattern underlying interaural time difference detection

*Kazuki Furumichi[1], Karin Miyata[1], Ryo Egawa[2], Hiroshi Kuba[2] ([1]*Dept Med, Nagoya Univ, Nagoya, Japan,* [2]*Cell Physiol, Grad Sch Med, Nagoya Univ, Nagoya, Japan*)

Integration of sound information from each ear is fundamental to calculation of interaural time differences for sound localization. In avian species, this integration is based on a unique branching pattern of axons that project bilaterally from nucleus magnocellularis (NM) to nucleus laminaris in the brainstem auditory circuit. However, the mechanisms controlling this branching pattern are poorly understood. In this study, we screened molecules involved in the collateral branch formation of NM axons in the chicken by combining single axon morphometry and perturbation of signaling pathways. Screening experiments using dominant-negative mutants showed that multiple signaling molecules related to Rho family small G proteins contribute to the collateral branch formation in a complex manner. Primary branch for the ipsilateral projection was abolished by inhibition of Rac1 or PAK1 pathways. On the other hand, formation of primary branch from the contralateral projection was promoted by inhibition of Rac1, RhoA, or PAK1 pathways and suppressed moderately by inhibition of Cdc42 pathway. These results suggested that signaling pathways for the collateral branch formation are highly distinct for each side in a single NM axon, providing important insights into the mechanism for formation of the sound localization circuit.

### [1LBA-008]

### Similar functional network structures emerge in the primary motor cortex during quiet wake and slow-wave sleep revealed by calcium imaging and graphical modeling

*Takeshi Kanda[1], Takehiro Miyazaki[1], Kotaro Sakamoto[2], Hideitsu Hino[2], Masashi Yanagisawa[1] ([1]*University of Tsukuba,* [2]*The Institute of Statistical Mathematics*)

Active and quiet wake have distinct effects on brain functions, the underlying physiological events in the cortex are still unknown. Using two-photon calcium imaging and statistical machine learning, we investigated the differences between these two states and the homology with sleep states in local functional connectivity within the primary motor cortex layer 2/3. No large differences were observed in individual neural activity between active and quiet wake. Functional connectivity between neurons, estimated by graphical modeling, was sparse in active wake and dense in quiet wake. Unexpectedly, common neuron pairs were connected functionally to each other during quiet wake and slow-wave sleep. Similarity analysis of functional network structures, calculated by Kullback–Leibler divergence, revealed that, in local cortical networks, slow-wave sleep was similar to quiet wake, while another sleep state rapid-eye-movement sleep was quite different from both active and quiet wake. Our observations suggest that common functional networks emerging in quiet wake and slow-wave sleep support the common offline information processing in the brain.

### [1LBA-009]

### Dopamine neurons convey distinct motivational signals in a self-paced decision-making task

*Hideyuki Matsumoto[1], Kenji Mizuseki[1] ([1]*Department of Physiology, Osaka Metropolitan University Graduate School of Medicine*)

Ventral tegmental area (VTA) dopamine neurons play critical roles in value-based learning and motivated behaviors. However, how dopamine neurons encode and generate motivation is still under debate. In this study, we recorded multiple single unit activities from the lateral VTA while rats performed a self-paced decision-making task in which the available reward amount was systematically changed. Animals showed shorter trial-start latency to initiate trials when they expected larger reward amounts and received larger rewards recently (high reward rate). We found that VTA population activities dynamically and gradually shifted depending on past reward history, presumably representing VTA neural state transitions with reward rate and motivational vigor. We next examined how dopamine neurons represent motivation to invigorate reward-oriented behaviors in the task. We optogenetically identified dopaminergic cell types and their striatal projection targets in each recording session. We found that dopamine neurons encode motivation with distinct firing modes (tonic or phasic activity) depending on whether animals start trials voluntarily or in response to the external trial-start cue. Furthermore, distinct subpopulations of dopamine neurons conveyed distinct motivational signals to different striatal areas. These results shed light on the dopamine dynamics for motivation during adaptive decision-making.

### Late Breaking Abstracts

[1LBA]
**Neurophysiology, Neuronal cell biology**
**Higher brain function**

March 14 (Tue.), 12:10 - 14:10, Poster Room

### [1LBA-010]

**Pharmacological inactivation of the ventral hippocampus attenuates long-trace, but not short-trace, taste aversion conditioning in rat**

*TSENG SZU CHIAO[1], Yasunobu Yasoshima[1] ([1]*Division of Behavioral Physiology, Graduate School of Human Sciences, Osaka University*)

Conditioned taste aversion (CTA) is established by a pairing of a novel taste stimulus (conditioned stimulus, CS) with visceral malaise (unconditioned stimulus, US). Animals can acquire CTA even when aversive US is followed with long CS-US interstimulus interval (long-trace CTA). Excitotoxic lesions of the dorsal and ventral hippocampus (HIP) did not impair CTA with a short CS-US interval procedure (Yamamoto et al., 1994); however, Koh et al. (2009) indicated that lesions of the ventral HIP (vHIP) impaired long-trace CTA. Little is known about how the vHIP is involved in long-trace CTA. To address the issue, we examined the effects of intra-vHIP microinfusions of muscimol (MUS), a GABA-A receptor agonist, before taste CS experience. Male Wistar rats bilaterally received MUS infusions into the vHIP followed by saccharin access as a CS in two-bottle methods. One group of the rats (long-trace group) received an intraperitoneal injection of 0.15 M LiCl as a US three hours after the CS ingestion, the other rats (short-trace group) did the same US immediately after the CS. On the first retention test in the long-trace group, the CS intake in rats with MUS infusions was significantly greater than that in the rats with vehicle infusions, suggesting that inactivation of the vHIP before the CS-US pairing attenuate long-trace CTA. No impairment was found in the short-trace CTA in the rats receiving the same intra-vHIP MUS infusions. The present results suggest that vHIP plays a role in long-trace CTA formation, but short-trace CTA is independent off the vHIP function.

### [1LBA-011]

**Grid cells disconnect from environmental cues to encode goal distance during path-integration**

*Satoshi Kuroki[1,2], Sebastien Royer[2] ([1]*Osaka Metropolitan University,* [2]*Korea Institute of Science and Technology*)

Spatial navigation relies on both landmarks and path integration information. Grid cells in the medial entorhinal cortex (MEC) display periodic firing fields and are believed to provide an intrinsic metric supporting path-integration. However, they are also controlled by landmarks. It is unknown whether they can support path integration and goal-direct navigation in situations requiring the abstraction of environmental cues. To test this possibility, we performed silicon probe recordings of MEC cells in mice that were running on a cue-enriched treadmill belt and had to use path integration to anticipate a water reward delivered after a fixed travel distance (shorter than the belt length). Grid cells showed multiple firing fields, which, at the population level, could be matched to a linear trajectory within a toroidal manifold. Interestingly, grid cell activity was reset by the reward such that the same population code was reused to encode the journeys to rewards, despite the fact that the environmental cue information changed across journeys. Hence, grid cells can disconnect from environmental cues to encode journeys to rewards.

### [1LBA-012]

**Creation of model mice to demonstrate the effects of foods for fondness, a studies using chocolate**

*Riko Sakurai[1], Mizuki Kurakano[1], Fumihro Shutoh[1] ([1]*Facul. Systems Life Engineering, Maebashi Institute of Technology*)

The importance of stress countermeasures is recognized and it is important to discover simple ways to reduce stress. In this study, we focused on the anti-stress effect of ingestion of foods for cheer up and verified it by using model mice. The items for cheer up; alcohol, cigarettes, tea, etc.; are for enjoying the sense of taste and smell and the uplifting feeling when ingest. Besides, since ancient times, the item has stabilized the mind by ingesting it in addition to the staple foods. It is unclear whether these effects are derived from physiological mechanisms or the ingestion experience. Then, we focused on verification by using a model mouse. In this study, we adopted chocolate as the item for cheer up which is liked by many people and recognized for its nutritional value. Purpose of this study is to make mice acquire a preference for chocolate and to examine the effects of ingestion of chocolate on stress. At first, chocolate was gradually given to the mice so that they could learn the taste and eat it willingly. Then, to examine the effect of chocolate on stress, these mice were used to examine chronic social defeat stress. We present these studies that investigated the intake of chocolate and changes in heart rate in mice stressed by chronic social defeat stress.

### [1LBA-013]

**Pareidolia is associated with changes in causal networks between brain regions, including visual areas.**

*Takeshi Kondo[1], Kenji Yoshinaga[2], Takashi Hanakawa[2] ([1]*Faculty of Medicine Kyoto University,* [2]*Integrated Neuroanatomy & Neuroimaging Kyoto University Graduate School of Medicine*)

### [1LBA-014]

**Spontaneous high-frequent firings in hippocampal CA1 neurons: a role of "super bursts" to initiate memory process**

*Junko Ishikawa[1], Koudai Okano[1], Dai Mitsushima[1] ([1]*Yamaguchi Univ.*)

Contextual learning requires hippocampal CA1 neurons, processing spatiotemporal information of experiences. By recording multiple-unit firings of hippocampal CA1 neurons in freely moving male rats, we previously found that episodic experiences induced high frequent spontaneous firings (super burst), followed by an increase in ripple-firings and synaptic plasticity in the CA1. Moreover, we found episode-specific diversity of the features of super bursts, synaptic plasticity, and ripple-firings. Since high-frequency firing of CA1 neurons is well known to promote synaptic plasticity, we hypothesized that the super bursts trigger experience-specific ripple-firings in the CA1. To prove the hypothesis, we developed a new system that immediately detects the onset of the super burst and simultaneously stimulate the hippocampal commissure to inhibit CA1 neural activity. The timing-specific elimination of super bursts successfully prevented changes in the ripple firings caused by the episodic experiences. These results not only support our hypothesis, but also demonstrate the role of super bursts in triggering memory encoding.

### Late Breaking Abstracts

### [1LBA]
### Neurophysiology, Neuronal cell biology
### Motor function

March 14 (Tue.), 12:10 - 14:10, Poster Room

### [1LBA-015]

**Computational model for predicting muscle activity reflects functional connectivity between motor cortex and muscles**

*Kokoro Kawamura[1,2], Takashi Isezaki[3], Michiaki Suzuki[2], Osamu Yokoyama[2], Shin-Ichiro Osawa[1], Kuniyasu Nizuma[1,4], Teiji Tominaga[1], Yukio Nishimura[2] ([1]*Department of Neurosurgery, Tohoku University Graduate School of Medicine,* [2]*Neural Prosthetics Project, Tokyo Metropolitan Institute of Medical Science,* [3]*NTT Human Informatic Laboratories,* [4]*Department of Neurosurgical Engineering and Translational Neuroscience, Tohoku University Graduate School of Medicine*)

Muscle activity depend on the activity of motor cortex that innervates spinal motoneurons. Such anatomical connectivity has been proven by the electrophysiological evidences; evoked muscle activity induced by cortical stimulation. Several studies documented that electromyographic (EMG) activity can be predicted from assemble of oscillatory cortical activities using computational model. However, it is unclear whether the prediction model reflects physiological neural connectivity. The present study is aimed to verify the model predicting EMG activity from cortical activity reflects physiological neural connectivity between the motor cortex and muscles. To assess the strength of neural connectivity between the cortical site and the muscles, we recorded EMG activity of forelimb muscles in behaving monkeys while stimulating a cortical site through the electrode array on surface of motor-related areas. Results showed that the cortical sites where induced high magnitude of evoked muscles activities was located in the primary motor cortex (M1). Then, we recorded signals through the same electrode array in same behavioral condition without cortical stimulation, and predicted the EMG activity from the signals using the sparse linear regression algorithm. The cortical sites exhibiting high weight values for predicting EMG activity were located in the M1, and this result was consistent with the results of cortical stimulation. Moreover, magnitude of evoked muscles activities correlated with values of weights. Thus, weight values obtained from computational model contain information of physiological neural connectivity.

### [1LBA-016]

**Modulation of masseter muscle activity by the cutaneous afferent of hand in the anticipatory stage of eating posture**

*Syusaku Sasada[1] ([1]*Department of Food and Nutrition Science, Sagami Women's University*)

Hand and mouth motor coordination plays an essential role in preventing aspiration while eating. In this study, I investigated whether the cutaneous afferent of hand-pinching food modulates masseter muscle activity in the anticipatory stage of eating, using the cutaneous reflex. The participants were asked to perform three different postures that mimicked the anticipatory stage of eating behavior: pinching food by hand (pinch), biting food without pinching (bite), and both pinching and biting food (pinch and bite). During these performances, electrical stimuli were applied to the superficial radial (SR) nerve on the ipsilateral side of the pinching hand to evoke a cutaneous reflex from the masseter muscle. To analyze the cutaneous reflex, a surface electromyogram (EMG) was recorded on the masseter muscle for 400 ms based on the timing of electrical stimulation. A suppressive EMG response from the masseter muscle (latency: approximately 35–70 ms) was identified following the SR nerve stimulation. The magnitude of the suppressive response was significantly modulated by each posture and was the largest in the "pinch and bite" method. These results suggest that cutaneous afferent of hand contribute in controlling masseter muscle activity in the anticipatory stage of eating behavior.

### Late Breaking Abstracts

[1LBA]
**Molecular physiology, Cell physiology**
**Ion channels, Receptors**

March 14 (Tue.), 12:10 - 14:10, Poster Room

### [1LBA-017]

**Development of a parameter optimization method to determine ionic current composition of a cardiomyocyte model**

*Yixin Zhang[1], Yukiko Himeno[1], Futoshi Toyoda[2], Hirohiko Kohjitani[3], Akinori Noma[1], Akira Amano[1] (*[1]Grad Sch Life Sciences, Dept Bioinfo, Ritsumeikan Univ, [2]Dept Physiol, Shiga Univ Med Sci, [3]Dept Cardiovasc Med, Kyoto Univ*)

Mathematical models of cardiac action potentials (APs) have been widely used to visualize individual currents underlying cardiac APs in a wide variety of experimental conditions. However, most of the parameters of the mathematical AP models have been determined using manual fitting, which might introduce subjective factors and raises questions about the accuracy of the model prediction. To overcome this weakness, we developed a computer algorithm that automatically optimizes those parameters by decreasing the Mean Squared Error (MSE) between the target (experimental) AP waveform and the model output (PO method). The accuracy of the algorithm of the PO method was examined by replacing the experimental AP record with a simulated standard AP waveform. An arbitrary AP waveform was prepared by randomizing several model parameters. Then, the PO method corrected the randomized AP shape to recover the standard AP form by modifying the scaling factors of the model parameters.

### Late Breaking Abstracts

[1LBA]
**Molecular physiology, Cell physiology**
**Others**

March 14 (Tue.), 12:10 - 14:10, Poster Room

### [1LBA-018]

**The initial mechanism of sphingosylphosphorylcholine (SPC), a causative factor of SPC-induced abnormal contraction, around the plasma membrane.**

*Natsuko Tsurudome[1], Yuji Minami[1,2], Katsuko Kajiya[1,2] ([1]*Department of Biological Science and Technology, The United Graduates School of Agricultural Sciences, Kagoshima University, Kagoshima, Japan,* [2]*Department of Food Science and Biotechnology, Faculty of Agriculture, Kagoshima University, Kagoshima, Japan*)

Sphingosylphosphorylcholine (SPC) is a causative factor of vasospasm. When blood vessels undergo vasospasm, vascular smooth muscle cells (VSMCs) contract abnormally and never relax again. However, the mechanism of abnormal contraction caused by SPCs is largely unknown, and neither its prevention nor its treatment has been established. Previous studies have shown that fisetin, a flavonoid from mulberry leaves, prevents SPC-induced abnormal contraction, but the mechanism of this prevention has been unknown. Therefore, we investigated where fisetin inhibits SPC-induced abnormal contraction using surface plasmon resonance. To clarify the relationship between microdomains that are thought to be involved in the abnormal contraction caused by SPC, the expression levels of marker proteins were evaluated by Western blotting. Furthermore, the localization of SPCs was observed using nitrobenzoxazole (NBD)-labeled SPCs to examine their movement around the plasma membrane. As the result, fisetin prevents SPC-induced abnormal contraction by directly acting on VSMCs. However, microdomains did not relate to the mechanism. In addition, NBD-SPC was taken up into the cells via endocytosis and never secrete. Although these results are useful for elucidating the mechanism of SPC-induced abnormal contraction, further studies are needed to clarify how endosomes act on SPC-induced abnormal contraction.

### [1LBA-019]

**Novel intracellular proteolytic pathway required for maintenance of neuromuscular homeostasis**

*Yuuki Fujiwara[1,2], Viorica Raluca Contu[2], Chihana Kabuta[2], Megumu Ogawa[2], Hiromi Fujita[2], Hisae Kikuchi[2], Ryohei Sakai[2], Katsunori Hase[2], Mari Suzuki[3], Ikuko Koyama-Honda[4], Michio Inoue[2], Yasishi Oya[5], Yukiko U. Inoue[2], Takayoshi Inoue[2], Ryosuke Takahashi[6], Ichizo Nishino[2], Keiji Wada[2], Satoru Noguchi[2] ([1]*Department of Child Development and Molecular Brain Science, United Graduate School of Child Development, Osaka University,* [2]*National Institute of Neuroscience, National Center of Neurology and Psychiatry,* [3]*Department of Sensory and Motor Systems, Tokyo Metropolitan Institute of Medical Science,* [4]*Department of Biochemistry and Molecular Biology, Graduate School and Faculty of Medicine, The University of Tokyo,* [5]*Department of Neurology, National Center Hospital,* [6]*Department of Neurology, Graduate School of Medicine, Kyoto University*)

Regulated degradation of cellular components plays an essential role in homeostasis. Lysosomes are largest sites for degradation of virtually all kinds of intracellular macromolecules. Accumulating evidences point out the importance of lysosomal degradation of cellular proteins: Dysfunctions in multiple pathways to deliver cytosolic substrates into lysosomes are related to various diseases, including neurodegenerative diseases and myopathies. however, much of the effort at understanding such pathways has been devoted to studies on "macroautophagy", which entails vast and dynamic rearrangement of membrane structure, and knowledge on other delivery systems and functions of lysosomes per se remains scant. Here, we show that cytosolic proteins are directly imported into lysosomes in an ATP-dependent manner by a mechanism distinct from any known pathways and degraded. We term this novel pathway as "direct-uptake-via/through-membrane-protein (DUMP)". We find that a lysosomal membrane protein, SIDT2, which was previously reported as a putative nucleic acid transporter, is involved in the translocation of substrate proteins in this system. Gain- and loss-of-function analyses reveal that SIDT2 contributes conspicuously to the lysosomal degradation of a wide range of cytosolic proteins in cells at theconstitutive level.Furthermore, we identified a patient of familial neuropathy and myopathy with rimmed vacuoles, harboring a dominant-negative mutation in SIDT2. Sidt2 knockout mice recapitulated typical features of rimmed vacuolar myopathy/neuropathy, which closely resembles observations seen in the patient, including accumulation of cytoplasmic inclusions.These results reveal a previously unknown pathway of proteolysis in lysosomes and highlight the importance of noncanonical types of autophagy in physiology and pathophysiology of human.

### [1LBA-020]

**Investigating the activity of inhibiting the proliferation of human lung adenocarcinoma cell line H1299 by plant extract**

*Kyoichi Takao[1], Hinako Suga[2], Masaharu Nomura[3,4], Noriko Gotoh[3] ([1]*Nihon Univ. School of Medicine,* [2]*Juntendo Univ. School of Medicine,* [3]*Cancer Res. Inst., Kanazawa Univ.,* [4]*Shingo Central Clinic*)

Lung adenocarcinoma, the most frequent type of lung cancer in Japan, is difficult to treat with chemotherapy and radiation, so the development of new preventive and therapeutic methods is expected. Since 2020, we have been searching for plants that inhibit the growth of lung adenocarcinoma. In this study, we focused on the p53-deficient, NRAS gene mutation among the genetic abnormalities that cause lung adenocarcinoma. The aim of this study was to analyze the growth suppression of plant extracts against H1299 cells with these mutations. In addition to H1299 cells, PC-9, A549 and H838 cells were compared in the experiment. These cells were counted after the addition of ethyl acetate extract of plants. The results showed that PC-9, A549, and H838 cells showed growth inhibition. We have examining the effect of the extract on H1299 cell proliferation and cell morphology. We will show these results suggest and discuss the effects of this plant extract on cell proliferation. In the future, we would like to identify natural compounds in this extract that inhibit the growth of lung adenocarcinoma cell lines. This work was partly supported by Extramural Collaborative Research Grant of Cancer Research Institute, Kanazawa University.

### Late Breaking Abstracts

### [1LBA]
### Oral physiology

March 14 (Tue.), 12:10 - 14:10, Poster Room

### [1LBA-021]

**The anion channels may be related to the receptive mechnism of water-stimulated swallowing in laryngopharyngeal region.**

*Chia-Chien Hsu[1], Chihiro Nakatomi[1], Kentaro Ono[1] ([1]*Division of Physiology, Kyushu Dental University*)

The receptive mechanism of water-stimulated swallowing reflex in the laryngopharyngeal region is unknown. Recent findings report the swallowing-evoked nerves were activated by the release of ATP from laryngeal taste bud-like structures. It has long been known that water swallowing is inhibited by NaCl, especially $Cl^-$. We hypothesized that $Cl^-$ channel is involved in the reception of water swallowing in laryngeal taste bud-like structures. In RT-PCR analysis, the expression of ClC-2, 3 were identified in rat laryngeal laminae (arytenoids). Immunofluorescence (IF) staining results show ClC-2 and 3 immunopositive images were seen not only in arytenoid epithelium but also in the taste bud-like structures (CK-8 immunopositive). We have found water swallowing was inhibited after surfactant application on laryngeal mucosa of rats. Therefore, we also performed IF staining after Triton-X-treated rats, and found that ClC-3-positive images were attenuated in a portion of the arytenoid epithelium and in the apex of taste bud-like structures. These results suggest that anion channels expressed in the arytenoid epithelium, including taste bud-like structures, are involved in the reception of water-stimulated swallowing.

### [1LBA-022]

**Rats discriminate differences in particle sizes of microcrystalline cellulose in the oral cavity**

*Chihiro Nakatomi[1], Chia-Chien Hsu[1], Tadashi Inui[2], Kentaro Ono[1] ([1]*Kyushu Dental Univ.*, [2]*Hokkaido Univ.*)

Food texture is an important factor in swallowing, mastication, and palatability. However, the mechanism of texture perception in the oral region has not been revealed. The present study investigates particle perception in rats. There was no innate preference between the particle solution and water. Therefore, we conducted particle preference learning tests using glucose and fructose. It is known that after repeated presentations of glucose and fructose solutions, preference learning to glucose is acquired. Furthermore, preference learning to additives to the glucose solution is also established. Using this method, we performed the preference test by adding f 170 mm and f 20 mm cellulose particles to the glucose and fructose solution, respectively, at a concentration of 1.6% (w/w). After acquisition of preference learning to the glucose solution, rats preferred the f 170 mm solution in a two-bottle preference test for 170 mm and 20 mm solution without glucose and fructose. This result indicates that rats discriminate differences in particle sizes. The present method allows for the study of oral texture perception.
Food texture is an important factor in swallowing, mastication, and palatability. However, the mechanism of texture perception in the oral region has not been revealed. The present study investigates particle perception in rats. There was no innate preference between the particle solution and water. Therefore, we conducted particle preference learning tests using glucose and fructose. It is known that after repeated presentations of glucose and fructose solutions, preference learning to glucose is acquired. Furthermore, preference learning to additives to the glucose solution is also established. Using this method, we performed the preference test by adding f 170 mm and f 20 mm cellulose particles to the glucose and fructose solution, respectively, at a concentration of 1.6% (w/w). After acquisition of preference learning to the glucose solution, rats preferred the f 170 mm solution in a two-bottle preference test for 170 mm and 20 mm solution without glucose and fructose. This result indicates that rats discriminate differences in particle sizes. The present method allows for the study of oral texture perception.

### [1LBA-024]

**Renin-angiotensin system in the geniculate ganglion of diabetic rats.**

*Takeshi Suwabe[1], Yasuo Toshiaki[1], Noritaka Sako[1], Shinpei Takahashi[1] ([1]*Department of Oral Physiology, School of Dentistry, Asahi University*)

It has been reported that a marked increase in taste preference for lower concentrations of glucose and sucrose is exhibited by rats made diabetic with streptozotocin. This finding suggests the possibility that alteration of taste afferent neuron may be involved in certain diabetic statuses. We hypothesized that diabetes activates renin-angiotensin system (RAS) in the geniculate ganglion containing taste afferent neurons and changes the taste preference for glucose and sucrose. Adult male nondiabetic rats received a daily intraperitoneal injection of streptozotocin (STZ) for 2 days. Blood glucose levels of the STZ rats employed in this study was higher than 500 mg/dl. Geniculate ganglion was collected from rats, total RNA was extracted from the geniculate ganglion, and cDNA was synthesized from the RNA template by reverse transcription. Expression levels of RAS-related genes were determined by real-time PCR. Expression levels of angiotensinogen and angiotensin converting enzyme genes were higher in the STZ rats than in nondiabetic rats suggesting alteration of the RAS-related gene expression increases in taste preference for glucose and sucrose.

### [1LBA-023]

**The Effect of Frequency-Regulated Repeated Micro-Vibration on Osteoblast Differentiation in MC3T3-E1 Cells**

*Tada-aki Kudo[1], Guang Hong[2], Kanako Tominami[1], You-Ran Luo[2], Satoshi Izumi[1], Takakuni Tanaka[2], Yohei Hayashi[3,4], Takuya Noguchi[5], Atsushi Matsuzawa[5], Junichi Nakai[1] ([1]*Div. of Oral Physiol., Tohoku Univ. Grad. Sch. of Dent.*, [2]*Div. for Globalization Initiative, Tohoku Univ. Grad. Sch. of Dent.*, [3]*Cell Resource Center for Biomedical Res., IDAC, Tohoku Univ.*, [4]*Grad. Sch. of Life Sci., Tohoku Univ.*, [5]*Lab. of Health Chem., Grad. Sch. of Pharmaceutical Sci., Tohoku Univ.*)

Osteoblasts are crucial for bone remodeling. Physical stimulation is an essential factor affecting the metabolism of osteoblasts and their precursors. However, the role of micro-vibration, which might cause efficient osteoblast differentiation, is mostly unclear. This study aimed to examine the effects of a frequency-regulated repeated micro-vibration (FRMV) on osteoblast differentiation in mouse pre-osteoblastic MC3T3-E1 cells. A waterproof micro-vibrator set in a CO2 incubator was used to apply FRMV to the cells incubated on culture plates. The cells in differentiating medium on the vibrator were exposed to FRMV (50 sec/hour) for 7, 14, or 21 days. Alkaline phosphatase (ALP) activity assay and LDN193189, a BMP receptor inhibitor, were used to evaluate the role of FRMV on osteoblast differentiation in the cells. The results showed that, compared with negative controls, FRMV with the above condition significantly increased the ALP activity of the cells. LDN193189 substantially inhibited the FRMV-mediated upregulation of ALP activity. These results suggest that FRMV might induce osteoblast differentiation via the BMP signaling pathway and offer an effective bone regeneration technique.

### Late Breaking Abstracts

### [1LBA]
### Circulation

March 14 (Tue.), 12:10 - 14:10, Poster Room

### [1LBA-025]

**Visualization of mechanical stress using a new tension indicator**

*Maretoshi Hirai[1] ([1]*Kansai Medical University, Department of Pharmacology*)

To visualize the mechanical stress, we have developed a novel tension-sensing indicator molecule. Whereas conventional tension sensors are based on FRET system, requiring two types of fluorescent proteins and lack quantification, our indicator molecule have succeeded in visualizing tension with only one type of fluorescent protein and monitoring the amount of protein at the same time. By inserting this molecule into the intracellular skeletal proteins αActinin and αCatenin, we succeeded in visualizing the intracellular tension. We found that when this molecule was transduced into NIH3T3 fibroblast cells, the fluorescence intensity was changed with unprecedented sensitivity by adding a myosin inhibitor blebbistatin that attenuates intracellular tension. It was also revealed that the fluorescence intensity of our molecule differs greatly between cell boundaries and cell boundaries, and there is a correlation between the dynamics of cell motility and tension force. Furthermore, with Crispr/Cas9 system, we generated a gene-targeted mouse strain expressing this tension indicator in a cell lineage-specific manner. We confirmed the fluorescent tension signal was consistent with the localization of αActinin and αCatenin in heart and liver, succeeded in tension force in live mice.

### [1LBA-026]

**In-vivo visualization of cardiac ATP levels during baroreflex-mediated arterial pressure changes in ATeam expressed rats**

*Aikitsugu Nishiura[1], Toru Kawada[1], Masamichi Yamamoto[1], Daiki Watanebe[1], Aimi Yokoi[1], Keita Saku[1] ([1]*National Cerebral and Cardiovascular Center*)

Background: We have developed a method of capturing the dynamics of cardiac adenosine triphosphate (ATP) in rats expressed ATeam, an ATP probe. Since sympathetic activation/inhibition alters cardiac metabolism, we examined the impact of baroreflex-mediated sympathetic arterial pressure (AP) changes on cardiac ATP levels. Methods: In anesthetized Sprague-Dawley rats expressed ATeam, we exposed the left ventricular (LV) surface and simultaneously recorded the fluorescence image at 30 frames/s. To regulate the baroreflex, we changed the carotid sinus pressure (CSP) between 60 to 140 mmHg every 60 s. We also examined the impact of acute afterload change by aortic occlusion and heart rate (HR) change by intravenous ivabradine. The ATP levels were assessed as an intensity ratio of red fluorescent protein (RFP) to green fluorescent protein (GFP) under GFP excitation at the same region of interest. Results: The increment of CSP decreased sympathetic activity, AP, and HR with an increase of the RFP/GFP ratio from $1.374 \pm 0.110$ to $1.386 \pm 0.115$ ($P < 0.001$, 10 trials in 5 rats). The aortic occlusion increased AP and decreased the RFP/GFP, whereas ivabradine did not significantly change the RFP/GFP ratio. Conclusion: In rats expressed ATeam, we successfully visualized the changes in cardiac ATP levels under baroreflex activation in the in-vivo condition.

### Late Breaking Abstracts

### [1LBA]
### Autonomic nervous system

March 14 (Tue.), 12:10 - 14:10, Poster Room

### [1LBA-027]

**Sympathoinhibitory and sympathoexcitatory roles of running exercise-excited lateral hypothalamic neurons assessed by rat FosTRAP**

*Satoshi Koba[1], Yuki Yoshimura[1], Kazuomi Nakamura[1], Misako Senoo[1], Karen Shibayama[1], Emi Narai[1], Takeshi Hiyama[1], Tatsuo Watanabe[1] ([1]*Tottori University*)

Targeted recombination in neurons activated by defined stimuli within a limited time window has been established by using a knockin mouse line (FosTRAP mouse), in which the tamoxifen-dependent Cre recombinase $CreER^{T2}$ is expressed from the endogenous c-Fos locus. Here, we newly generated FosTRAP rats via CRISPR/Cas9 system and thereby investigated the distribution of running exercise-excited hypothalamic neurons as well as their roles in eliciting sympathetic outflow. Lateral hypothalamus (LH) of rats that performed 2-h voluntary treadmill running with tamoxifen displayed a greater number of "FosTRAPed" neurons than that of control rats in which tamoxifen was administered during the resting period on the treadmill. Under urethane anesthesia, optogenetic stimulation of cell bodies of the running-exercise-FosTRAPed LH neurons decreased renal sympathetic nerve activity while this optogenetic effect was not seen by stimulation of the control-FosTRAPed neurons. Optogenetic stimulation of axons extending from the running-exercise-FosTRAPed LH neurons in the ventral medulla had a pressor effect. This study suggests the involvement of both sympathoinhibitory and sympathoexcitatory LH neurons during voluntary running exercise.

### [1LBA-028]

**Autonomic responses induced by emotional sports video viewing**

*Kirari Wada[1], Tsugutake Yoneda[1], Hidefumi Waki[1], Ko Yamanaka[1] ([1]*Grad Sch Health & Sports Sci, Juntendo Univ, Japan*)

Appreciation of sports, music, and arts often induce transitory pleasurable feelings. Previously, studies have reported autonomic responses (goose bumps and chills) and brain activity if listening to emotional music. However, the physiological mechanisms underlying sports video viewing-induced emotional changes remain unclear. This study aimed to examine whether sympathetic nervous-related activities are elicited during emotional sports video viewing. Autonomic responses (blood pressure [BP], heart rate, respiration, and skin conductance) were recorded during emotional sports video viewing in seven participants (four men and three women). Participants pressed a button during the time they felt "moved" by watching a sports video (SPO task) or during an instructed time of viewing an upside-down, left-right, and reverse-playback video (CON task). In the SPO task, the average number of button presses per participant was 40.6 ± 11.5 times and the average immediate BP response before pressing the button ($\Delta BP = 0.74 \pm 1.30$ mmHg) was significantly higher than that in the CON task ($-0.78 \pm 0.72$ mmHg; $p < 0.05$). These results suggest that sympathetic activities may increase the feeling of being "moved" during sports video viewing.

### Late Breaking Abstracts

### [1LBA]
**Physical fitness and sports medicine**

March 14 (Tue.), 12:10 - 14:10, Poster Room

### [1LBA-029]

**The legacy effect of exercise on senescence related gene expressions in visceral adipose tissue of young mice**

*Masaki KIMURA[1], Yuuki KANEKO[1], Nanaho ISHIDUKA[1], Taisuke KOREEDA[1], Juntaro MATSUZAKI[1], Yoshimasa SAITO[1] ([1]*Dept. of Pharmacotherapeutics, Faculty of Pharmacy, Keio Univ.*)

[INTRODUCTION] The effects of exercise on senescence related gene expression in visceral adipose tissue and it's maintenance are not fully elucidated. Therefore, we investigated the effects of exercise on senescence related gene expressions in visceral adipose tissue of young mice and it's maintenance after exercise termination, so called “legacy effect”. [METHODS] Male ICR mice (4-week-old) were assigned to one of the following 3 groups, 20-week normal control sedentary condition group, 20-week voluntary wheel running exercise group, or 10-week exercise followed by 10-week sedentary condition group. All animals were housed individually and were fed regular control diet and water ad libitum. At 24-week-old, epididymal fats were collected and weighed immediately after euthanasia.[RESULTS] The mice showed the decreases in senescence and SASP related gene expressions (i.e. p53, p16, and IL-6 mRNA expressions) in visceral adipose tissue in response to exercise and the long-lasting maintenance of these effects was appeared for 10 weeks after stopping exercise. [CONCLUSION] These results suggest that the exercise might ameliorate the senescence related gene expressions in mice visceral adipose tissue and these effects were suggested to maintain for a while after stopping exercise that indicate “legacy effect”.

### Late Breaking Abstracts

[1LBA]
**Pathophysiology**

March 14 (Tue.), 12:10 - 14:10, Poster Room

### [1LBA-031]

**Brain environmental changes associated with epilepsy development and sudden death**

*Yoko Ikoma[1], Daichi Sasaki[1], Ko Matsui[1] ([1]*Super-network brain Physiolosy Graduate School of Life Scieneces Tohoku University*)

Glial role in information processing, plasticity, and healthiness of the brain has been determined. Here, we devised a method to read out as much of the local environment parameters as possible from the detected fluorescence signals with fiber photometry. In this study, plastic change of astrocyte reactions in the lateral hypothalamus was examined using epileptogenesis with hippocampal stimulated kindling model as an extreme form of plasticity. Fluorescent sensors for calcium or pH were genetically expressed in mouse astrocytes. The emergence of an acid response of astrocytes upon exacerbation of epilepsy was observed. The acid response of astrocytes could be the underlying drive for generating the plasticity of epileptogenesis. A rare encounter of sudden epileptic death was studied in detail and compared with a previous seizure of similar magnitude. The difference in the time course of sudden EEG arrest, astrocyte response, and blood flow cessation was described and the boundary between life and death was revealed. Controlling the astrocyte pH could be a new therapeutic target for the prevention of undesired plasticity associated with epileptogenesis and epileptic death.

### [1LBA-030]

**Metabolomic analysis of human urine in perioperative patients**

*Aya Furuta[1], Fumiya Tsutsui[1], Ryota Tsurugaya[1], Kazuma Hyodo[1], Akiho Mashiba[1], Misato Nakatake[1], Kazue Ogata[1], Shinya Kai[2], Shigekiyo Matsumoto[2], Chihiro Shingu[2], Takaaki Kitano[2], Osamu Tokumaru[1] ([1]*Department of Physiology, Faculty of Welfare and Health Sciences, Oita University,* [2]*Department of Anesthesiology, Oita University Faculty of Medicine*)

PurposePerioperative complication is a significant cause of morbidity and mortality in surgery. Early diagnosis and appropriate treatment may improve patients' outcomes. To determine whether a metabolomics approach could be useful in the diagnosis, we applied nuclear magnetic resonance (NMR)-based metabolic profiling to track metabolic changes during surgery. MethodUrine was collected from 40 patients before and after surgery including cardiovascular, pulmonary, and general surgery, and stored at -80°C until NMR spectrometry. Urine samples from 82 healthy controls were also analyzed. Proton NMR spectra were acquired using a Bruker AVANCE III-400 spectrometer. The water peak was suppressed using NOESY pulse sequence. Sixty-four transients were collected for each spectrum. After Fourier transformation, each spectrum was reduced to 85 frequency buckets of equal width in order to reduce the matrix size necessary for principle component (PC) analysis.ResultsScore plots using the first two PCs illustrated significant separation of urine samples between before and after the surgery, by which 49% of variance was explained. The loading plot indicated significant contributions of metabolites in the aliphatic region of chemical shift.ConclusionsOur results indicate that NMR-based metabolomics of urine might serve as a promising approach for the diagnosis, clinical evaluations and prediction of perioperative complications.

### Late Breaking Abstracts

Day 2
(March 15, 12:10 - 14:10)

[2LBA] Neurophysiology, Neuronal cell biology - Neural network
[2LBA] Neurophysiology, Neuronal cell biology - Neurons, Synapses
[2LBA] Molecular physiology, Cell physiology - Membrane transport
[2LBA] Blood, Lymph, Immunity
[2LBA] Respiration
[2LBA] Reproduction
[2LBA] Endocrine
[2LBA] Environmental physiology
[2LBA] Nutritional and metabolic physiology, Thermoregulation
[2LBA] Behavior, Biological rhythm, Sleep
[2LBA] Study Methodology

### Late Breaking Abstracts

[2LBA]

**Neurophysiology, Neuronal cell biology**

**Neural network**

March 15 (Wed.), 12:10 - 14:10, Poster Room

### [2LBA-001]

### Functional connectivity in the limbic and brain stem regions during treadmill exercise in rats

*Shinichiro Ezure[1], Ko Yamanaka[1], Hidefumi Waki[1] ([1]*Grad Sch Health & sports Sci, Juntendo Univ, Japan*)

Athletic performance is affected by cardiovascular response. Although it is known that the limbic and brainstem regions play important roles in the autonomic cardiovascular regulation, how these brain regions interact during high-intensity exercise remains unclear. In this study, Wistar rats (8–9 weeks old) were subjected to a 90-min treadmill running at different exercise intensities (high- intensity, low-intensity, and control groups: 34 m/min, 20 m/min, and 0 m/min, respectively; n = 9 in each group). The number of c-Fos-positive cells was quantified and a pairwise correlation analysis was performed to examine the functional connectivity during exercise. As a result, the number of c-Fos-positive cells in the central nucleus of the amygdala (CeA), the hypothalamic paraventricular nucleus (PVH), and the medullar nucleus tractus solitarius (NTS) increased in the exercise intensity-dependent manner ($p < 0.05$). Furthermore, the number of c-Fos-positive cells showed a correlation between these regions (PVH-NTS, $r = 0.774$, $p < 0.001$; CeA-NTS, $r = 0.84$, $p < 0.001$). Thus, functional connectivity between PVH-NTS and CeA-NTS may influence the cardiovascular response during exercise.

### [2LBA-002]

### Excitatory neurons are incorporated in caudal part of the basal ganglia in rodent

*Yasuharu HIRAI[1], Fumino Fujiyama[1] ([1]*Hokkaido Univ.*)

We found calbindin(CB)-enriched neurons in caudal-lateral part of the basal ganglia of rodents. These neurons were discriminable with the striatal medium spiny neurons, many of which also express CB, by the lack of Ctip2 expression. Farther immunohistochemistry revealed that CB neurons were not GABAergic nor cholinergic neurons. Historically, caudal part of the rodent globus pallidus is sometimes regarded as the basal nucleus of Meynert, that is the part of cholinergic nucleus CH4 of the basal forebrain structure. Therefore, we thought that CB-enriched neurons are taxonomically basal forebrain neurons. Here, we reported that these CB-enriched neurons were able to be labeled in the VGluT2-Cre transgenic mice with adeno-associated virus incorporating Cre-dependent fluorophore expression. Not all labeled neurons were CB-immunopositive, but all negative for striatum and globus pallidus neuron markers. It indicated that both excitatory and inhibitory neurons are intermingled in the caudal basal ganglia, and it could be the factor forming the function of the caudal basal ganglia which differs to the canonical, rostral basal ganglia.

### [2LBA-003]

### Synaptic organization of simple and complex cells in the mouse primary visual cortex

*Yuta Fukuda[1], Satoru Kondo[1], Kenichi Ohki[1] ([1]*The University of Tokyo*)

In the mouse primary visual cortex (V1), orientation-selective neurons are classified into simple and complex cells based on their response properties to the phase of grating stimuli. According to the model proposed by Hubel and Wiesel, simple cells receive input from simple cells with similar phase selectivity and complex cells receive input from simple cells with various phase selectivity. Although this model has been widely accepted for decades, experimental evidence for this model has not yet been fully established. Here, we aim to test this model by mapping the phase selectivity of synaptic inputs on dendrites from simple and complex cells using two-photon calcium imaging in mouse V1. First, we examined the spatial distribution of phase-selective V1 neurons using wide-field epifluorescence and two-photon calcium imaging and found a functional architecture in the distribution of phase-selective neurons across the V1 cortical surface. We then mapped the phase selectivity of dendritic spines from a single simple cell using two-photon calcium imaging. We showed that the simple cell primarily collected inputs from simple cells with similar phase selectivity. Furthermore, we found that the phase-selective inputs were distributed over the dendrites in a manner that spatially corresponded to the cortical phase map. Thus, so far, our data indicate the validity of the Hubel and Wiesel model, at least for simple cells, and suggest that the spatial distribution of phase-selective inputs on dendrites may significantly affect the phase selectivity of neurons.

### [2LBA-004]

### Thalamo-prefrontal circuits responsible for cognitive dysfunction in psychiatric disorders

*Shinichiro Tsutsumi[1] ([1]*RIKEN Center for Brain Science*)

Cognitive dysfunctions in psychiatric disorders such as autism spectrum disorder (ASD) are diffucult to treat, since the underlying neuronal circuit mechanisms are unclear. Functional disruption in thalamo-cortical circuits involving prefrontal cortex (PFC) are thought to be the core culprit for the dysfunction. However, precise circuit substrates and their cellular resolution neural population activity responsible for the dysfunctions remain elusive. To address this issue, we combine an activity-dependent functional tracing and chronic two-photon (2P) imaging of the thalamocortical circuits in psychiatric disease model mice performing a cognitive task.

### [2LBA-005]

### Role of inhibitory neurons in hierarchical information processing of visual prediction errors.

*Reiji Miyata[1], Ryosuke Takeuchi[1], Akinori Sato[1], Kei Ito[1], Masahiro Yamaguchi[1], Fumitaka Osakada[1] ([1]*Nagoya Univ.*)

Animals always predict the environment at the next moment by integrating bottom-up input through sensory organs such as the retina and top-down input such as our own movement, memory, and experience. When sensory input deviates from the prediction, or "prediction error information" is detected, the animal changes its behavior or updates its memory and experience. Research on visual pathways focusing on prediction error has suggested that prediction error information is detected by neural circuits consisting of excitatory and inhibitory neurons in L2/3 of V1. The excitatory neurons send the detected prediction error information to a wide area of the visual cortex including V1, RSP, and M2. However, it is unclear how inhibitory neurons other than V1 are involved in prediction error processing. We constructed a virtual reality (VR) system that can present visual prediction errors to mice and investigated the function of inhibitory neurons by performing wide-field $Ca^{2+}$ imaging on mice expressing GCaMP in a wide range of inhibitory neurons in the cerebral cortex using AAV. As a result, we observed activity in response to prediction error in a wide area of visual cortex including V1, RSP, and M2, where excitatory neuron activity was observed in previous studies. This indicates that cortical widespread inhibitory neurons play an important role in prediction error processing in the visual pathway.

### Late Breaking Abstracts

[2LBA]

### Neurophysiology, Neuronal cell biology

**Neurons, Synapses**

March 15 (Wed.), 12:10 - 14:10, Poster Room

### [2LBA-006]

**NREM-active and REM-active cells in the limbic and cortical regions are differently modulated by fast network oscillations and behaviors**

*Risa Kajiya[1,2], Hiroyuki Miyawaki[1], Kenji MIzuseki[1] ([1]*Osaka Metropolitan University Graduate School of Medicine, Department of physiology,* [2]*Osaka Metropolitan University Graduate School of Medicine, Department of Oral and Maxillofacial Surgery*)

Sleep consists of two distinct states, rapid eye movement (REM) and non-REM (NREM) sleep. Neuros alter their firing activity in response to the brain state changes; however, it remains poorly understood how the firing modulation by sleep states is diverse across neurons and brain regions. To examine how sleep state modulates firings of individual neurons, we analyzed previously obtained 17-hour continuous recordings of single-unit activities and local field potentials in the ventral hippocampus CA1 region (vCA1), prelimbic cortex layer 5 (PL5), and basolateral nucleus of the amygdala (BLA) of fear-conditioned rats. We found that firing rate modulation by sleep state was diverse across cells; more than half of excitatory cells (62%, 72%, and 76% in vCA1, PL5, and BLA, respectively) fired significantly faster in REM than in NREM, whereas considerable fractions of the excitatory cells (35%, 21%, and 21% in vCA1, PL5, and BLA, respectively) fired more in NREM than in REM. Both NREM- and REM-active cells decreased firing activity across sleep in all examined brain regions, which is consistent with homeostatic firing regulation across sleep. However, the firing modulation by the fast network oscillations, such as hippocampal sharp-wave ripples, which support memory consolidation during sleep, significantly differed between NREM- and REM-active cells. In addition, REM-active cells enhanced their firing more prominently than NREM-active cells in response to shock and freezing behavior. These results indicate that NREM- and REM-active cells are differently modulated by fast network oscillations and play distinct roles in the memory process.

### [2LBA-007]

**Edonerpic maleate accelerates recovery of upper limb motor function from spinal cord injury in nonhuman primates**

*Koichi Uramaru[1], Hiroki Abe[1], Waki Nakajima[1], Michiaki Suzuki[2], Osamu Yokoyama[2], Takeaki Yano[3], Nishimura Yukio[2], Takuya Takahashi[1] ([1]*Department of Physiology, Yokohama City University,* [2]*Neural Prosthetics Project, Tokyo Metropolitan Institute of Medical Science,* [3]*Bio Science & Engineering Research Laboratories, FUJIFILM Corporation*)

Glutamate AMPA receptors, which are responsible for excitatory neurotransmission, play a major role in plastic changes in the central nervous system. We have shown that a small compound, edonerpic maleate (edonerpic MA: 1-{3-[2-(1-benzothiophen-5-yl)ethoxy]propyl} azetidin-3-olmaleate) increases the efficiency of synaptic transmission via AMPA receptor and accelerates motor function recovery after brain injury through rehabilitative training. This study was designed to test the efficacy of edonerpic MA in a nonhuman primate spinal cord injury (SCI) model. Six adult monkeys (Macaca fuscata) received a unilateral SCI at C6/7 segment. After the SCI, upper limb motor function was immediately impaired. The animals received rehabilitative training of food retrieval with the affected hand. Edonerpic MA-treated group (n=3) showed earlier improvement in finger dexterity than control group (n=3). Thus, edonerpic MA accelerates the rehabilitation effect to the trained motor function after spinal cord injury. The motor representation of wrist territory confirmed by using intracortical microstimulation was larger in the edonerpic MA-treated group suggesting that edonerpic MA increases the efficiency of synaptic transmission in the residual corticospinal tract on the contralesional side from bilateral motor-related areas.

### [2LBA-008]

**Involvement of mitochondrial reactive oxygen species in AMPA-induced nigral dopaminergic cell death**

*Miki Suzuki[1], Miki Sasaki[1], Mayu Amano[1], Akira Murakami[1], Atsushi Takeda[1], Yuji Hara[1] ([1]*University of Shizuoka, School of Pharmaceutical Sciences, Department of Integrative Physiology*)

Oxidative stress is thought to be associated with neurodegenerative diseases including Parkinson's disease. We previously reported that excessive intracellular $Zn^{2+}$ causes neuronal cell death by hyperactivation of AMPA receptors in the substantia nigra pars compacta (SNpc). To elucidate the mechanism underlying AMPA receptor-induced $Zn^{2+}$ toxicity, we examined a possible relationship between reactive oxygen species (ROS) production and AMPA-induced intracellular $Zn^{2+}$ dysregulation. We first evaluated intracellular ROS and $H_2O_2$ production in SNpc using APF and HYDROP, respectively, and detected the increases in the both fluorescent intensities in response to AMPA injection into SNpc. We next examined mitochondrial functions in SNpc, and found that mitochondrial membrane potential was clearly decreased by AMPA injection. Importantly, removal of extracellular $Zn^{2+}$ using CaEDTA significantly canceled the AMPA-induced toxic effects such as ROS production and mitochondrial dysfunctions. Moreover, AMPA-induced nigral dopaminergic degeneration was suppressed by co-injection of either APF or HYDROP used as ROS scavengers into SNpc. These results suggest that AMPA-induced mitochondrial dysfunction via ROS results in nigral dopaminergic degeneration.

### [2LBA-009]

**Analysis of effects of a new mood stabilizer on adult mouse neurogenesis**

*Naomi Osa[1] ([1]*Shiga University of Medicine, Integrative Physiology*)

Bipolar disorder is one of mental illnesses which impose a heavy burden on our society. We previously reported that classical mood stabilizers, treatment for bipolar disorder, such as lithium, valproic acid and carbamazepine, enhance the self-renewal capability of mouse neural stem cells (NSCs) *in vitro*. Importantly, this enhancement is achieved at therapeutically relevant concentrations in cerebrospinal fluid but not in serum. In addition, chronic administration of mood stabilizers also expanded the NSC pool in the subependymal zone of adult mouse brains. In this study, we investigated whether or not one of new mood stabilizers, lamotrigine (LTG), plays a role in neurogenesis both in vivo and in vitro. We performed a neurosphere assay, by which we can isolate and culture NSCs from the adult brain, and found that neurosphere formation was significantly increased when NSCs were cultured in the presence of LTG as compared to control. Moreover, the proliferation of neural precursor cells was significantly increased in the brain from mice, which were administrated LTG for 12 weeks (10 mg/kg/day), as compared to the controls.These results suggest that the new mood stabilizer also possesses similar pharmacological effects to the classical ones and that those effects on adult NSCs and the neurogenesis plays a significant role in their mood stabilizing action.

### [2LBA-010]

**Effect of polyunsaturated fatty acids on indoxyl sulfate induced neuronal stem cell toxicity**

*masato nakazono[1], Rina Murata[1], Masanori Katakura[1] ([1]*Department of Nutritional Physiology, Graduate School of Pharmaceutical Sciences, Josai University*)

[Objective]Patients with chronic renal failure have been reported to increase risk of cognitive impairment compared to healthy individuals. The causes include an increase in reactive oxygen species (ROS) due to a decrease in renal function and accumulation of uremic substances, but the detailed mechanism has not been clarified. Previous studies have shown that chronic inflammation in the brain due to chronic renal failure reduces neurogenesis and cognitive function. In this study, indoxyl sulfate (IS) was added to neural stem cells (NSCs) to investigate the effects on cell differentiation and proliferation. [Methods]NSCs were collected and cultured from rat fetuses at 14.5 days gestation. IS was added to cultured NSCs, and MTS tests and immunocytochemical staining were performed. [Results and Discussion]IS concentration-dependent decrease in the number of cells in both differentiation and proliferation condition; DHA recovered cell viability. ROS level was increased by IS but DHA decreased IS induced ROS level. Immunocytochemical staining showed IS decreased in both the neuronal marker Tuj-1 and the astrocyte marker GFAP positive cells. [Conclusions]IS suppressed the differentiation and proliferation of neurons and glial cells, and DHA suppressed IS toxicity.

### Late Breaking Abstracts

[2LBA]
**Molecular physiology, Cell physiology**
**Membrane transport**

March 15 (Wed.), 12:10 - 14:10, Poster Room

### [2LBA-011]

### Evolutionary reduction of urea and boric acid transport activity of an Aqp10 paralog in ray-finned fishes

*Genki Imaizumi[1], Kazutaka Ushio[1], Ingo Braasch[2], Ayumi Nagashima[1], Akira Kato[1]
([1]*School of Life Science and Technology, Tokyo Institute of Technology,* [2]*Department of Integrative Biology, College of Natural Science, Michigan State University*)

Aquaporin 10 (Aqp10) is a member of the aquaglyceroporin subfamily of water channels and is permeable to small uncharged solutes such as glycerol, urea, boric acid, as well as water in human. Tetrapod animals have a single *aqp10* gene whereas ray-finned fishes have more than one paralogs for *aqp10* which have been generated by tandem duplication, genome duplication, and deletion. Our previous study showed that a pufferfish paralog Aqp10bb expressed in *Xenopus* oocytes is permeable to water and glycerol but not to urea and boric acid. To understand the evolutionary history that generated this difference, we analyzed the activity of tetrapod, ancient fish, and teleost fish Aqp10s expressed in oocytes. Water, glycerol, urea, and boric acid permeability was commonly observed in tetrapod Aqp10, ancient fish Aqp10a, and teleost fish Aqp10aa, suggesting that this activity is the ancestral characteristics of Aqp10 in vertebrates. In contrast, ancient fish Aqp10b and teleost fish Aqp10bb were highly permeable to glycerol and water but not to urea and boric acid. These results suggest that urea and boric acid transport activity of Aqp10b was reduced in an ancestral species of ray-finned fish.

### [2LBA-012]

### Live-imaging analysis of F-actin actions on intracellular insulin granule behavior

*Shinichiro Ono[1], Hiroyasu Hatakeyama[1], Tomomi Oshima[1], Noriko Takahashi[1]
([1]*Department of Physiology, Kitasato University School of Medicine*)

Insulin secretion is regulated by the recruitment of intracellular insulin granules to the juxtamembrane regions. To elucidate this process and its regulation by F-actin, we conducted live-imaging of insulin granule behavior in rat INS-1 cells expressing insulin-HaloTag. By staining the cells with HaloTag TMR ligand, we observed granular structures of similar size to insulin granules. The movement of these granular structures was captured using a spinning disk confocal microscope at 2 frames/sec at a distance of 1~2 µm above the glass surface and was quantified with mean square displacement. Pharmacological disturbance of whole-cell F-actin dynamics with latrunculin B, cytochalasin D and jasplakinolide indicated negative roles of F-actin on insulin granule movement, whereas inhibition of local F-actin dynamics mediated by formin or Arp2/3 suggested positive roles of F-actin in this movement. Dual-color imaging of insulin granules and F-actin proposed that local F-actin dynamics may directly modulate insulin granule movement. Overall, our findings suggest that the complex actions of F-actin play critical roles in insulin granule behavior.

### [2LBA-013]

### A mathematical model calculating transport of solutes and water at an epithelial cell membrane and tight junction in renal proximal tubule

*Yuto Kunimasa[1], Yukiko Himeno[3], Daiki Thara, Daiki Nishizuka, Akira Amano[3], Junichiro Taniguchi[2] ([1]*Ritsumeikan University Graduate School of Life Sciences,* [2]*Jichi Medical University,* [3]*Ritsumeikan University*)

The transport of solutes and water in renal proximal tubule (PT) has been confirmed experimentally but has not been elucidated quantitatively. We developed a mathematical model to simulate transport of solutes and water in the PT. The luminal membrane of the model contains SGLT, NHE and $K^+$ channels. NaK pump supplied with ATP sufficiently, GLUT, NBC, and $K^+$ channel are placed in the basolateral membrane. $CO_2$ generated from luminal $NaHCO_3$ diffuses into the cell, converted to $HCO_3^-$ and extruded from the cell via NBC. Epithelial cells are connected by tight junction (TJ). Ion transport via ion channels and TJ follows Goldman-Hodgkin-Katz equation. Water is osmotically transported via water channels and TJ, which is accompanied by solvent drag of solutes. Luminal and interstitial concentrations of each solute were assumed to be constant. As a result, solutes and water except $K^+$ were reabsorbed along the PT. $K^+$ was secreted if the luminal concentration of $K^+$ was low supposing early PT. It was suggested that a single epithelial cell model was not sufficient and the development of tubular model was necessary to realize reabsorption of $K^+$ supposing high $K^+$ concentration in the late PT.

### Late Breaking Abstracts

[2LBA]
**Blood, Lymph, Immunity**

March 15 (Wed.), 12:10 - 14:10, Poster Room

### [2LBA-014]

### A Generative Model for Single-Cell Transcriptome with DNA Barcoding Enables Inferring Developmental Trajectories during Differentiation

*Koichiro MAJIMA[1], Yasuhiro KOJIMA[2], Kodai MINOURA[3,1], Teppei SHIMAMURA[1,2]
([1]*Nagoya University Graduate School of Medicine,* [2]*Tokyo Medical and Dental University Medical Research Institute,* [3]*Japanese Red Cross Nagoya Daiichi Hospital*)

Single-cell RNA sequencing (scRNA-seq) enables the comprehensive characterization of cell types and states. However, since RNA-seq destroys cells in the process of analysis, it cannot measure how gene expression changes during dynamic biological processes such as embryo genesis. While recent studies combine scRNA-seq with lineage tracing and provide clonal information between progenitor and mature cells, they still face several challenges. First, although the processes are continuous, the observation can be conducted only at discrete time points due to its experimental cost. Additionally, cells in early state are not progenitor cells because scRNA-seq is performed after progenitor cells divided several times. To address these issues, we developed a new computational methodology that utilizes deep learning to convert single-cell transcriptome observation with DNA barcoding into the latent cell state dynamics consistent with the clonal relationship. This method enables us to quantitatively capture the cell state transitions. We demonstrate how this method can simulate differentiation trajectories in hematopoiesis. The model learned underlying potential dynamics and inferred cell states which are not available during the process from hematopoietic progenitor cells to mature cell types such as erythrocytes and neutrophils.

### Late Breaking Abstracts

[2LBA]
**Respiration**

March 15 (Wed.), 12:10 - 14:10, Poster Room

### [2LBA-015]

### Changes in lipid metabolism and metabolic flexibility in chronic renal failure model rats

*Kazuyuki Urata[1], Masanori Katakura[1], Katsuhiko Yajima[1] ([1]*Department of Nutritional Physiology, Josai University Graduate School*)

[Purpose]Metabolic flexibility is defined as the ability to switch between carbohydrate and fat oxidation. Metabolic diseases are often reported to be associated with decreased metabolic flexibility. There are no consistent results of consistent energy consumption in the whole body in patients with chronic renal failure. In this study, we investigated lipid metabolism and metabolic flexibility in chronic renal failure model rats. [Method] Animals: Six-week-old male Sprague Dawley rats were acclimatized for 2 weeks and then randomly divided into a control (Control Sham, CS) group and a kidney failure (KF) group. Nephrectomy was performed on 5/6, and respiratory quotient measurements and organ were collected 8 weeks after Nephrectomy. [Results/discussion] Nephrectomy reduced renal functions. Hepatic triglycerides in the KF group were significantly decreased, and plasma cholesterol were increased compared with CS group. Metabolic flexibility and fat oxidation from 9:00 to 19:00 were decreased in the KF group compared with CS groups. Therefore, it is possible that the respiratory quotient changes during sleep rather than during active period. [Conclusion] During the progression of renal failure, in the plasma and respiratory quotient throughout the body was increased. TG was accumulated but, in the liver, TG decreased. Therefore, we speculate that metabolic flexibility is reduced in chronic renal failure model rats.

### Late Breaking Abstracts

[2LBA]
**Reproduction**

March 15 (Wed.), 12:10 - 14:10, Poster Room

### [2LBA-016]

### Toxic Effects of Perfluoroalkyl and Polyfluoroalkyl Substances: PFASs in Human Placental Trophoblast through cAMP Signaling Pathway

*Wataru Miyazaki[1,2], Nozomi Kanazawa[2], Haruka Takeuchi[2], Jo Sakuragi[1] ([1]*Hirosaki University Graduate School of Health Sciences,* [2]*Hirosaki University School of Health Sciences*)

Perfluorooctanesulfonate (PFOS) and perfluorooctane acid (PFOA) are widely used as ingredients in several products (i.e., plastic wares, carpets, and fire extinguishers). These are hard to degrade in the environment and are detected in humans ubiquitously because the molecule structures are very stable. The exposure and accumulation of these compounds induce several toxic effects in many organs including the placenta. The toxicities in the placenta by PFOS and PFOA may affect placental function, pregnancy outcomes, and child health, however, how to induce the toxicities are still unknown. In this study, to clarify the pathways of the toxicities in the human placenta, we investigated the effects of PFOS and PFOA in a human trophoblast cell line, Bewo. cAMP inducer, forskolin (FSK), induces syncytialization, cell fusion, and the production of human chorionic gonadotropin in Bewo, but PFOS or PFOA exposure suppressed the gene expressions related to these functions induced by FSK. The suppression could not rescue by exogenous cAMP, IBMX, and isoprenaline. From these results, we hypothesized that PFOS and PFOA may affect the downstream of cAMP signaling pathway, not cAMP induction, and examined the effects of the compounds on cAMP response element-mediated transcriptional activities. These results suggest that PFOS and PFOA may disrupt the functions of the placenta through cAMP signaling pathway.

### Late Breaking Abstracts

[2LBA]
**Endocrine**

March 15 (Wed.), 12:10 - 14:10, Poster Room

### [2LBA-017]

### Influence of somatosensory stimulation on melatonin secretion from the pineal gland in urethane-anesthetized rats

*Nobuhiro Watanabe[1], Harumi Hotta[1] ([1]*Department of Autonomic Neuroscience, Tokyo Metropolitan Institute of Gerontology*)

The pineal gland is an endocrine organ that synthesizes and secretes melatonin, and is innervated by the cervical sympathetic nerve. Somatosensory stimulation modulates autonomic nerve activity and affects hormone secretions. The present study investigated whether somatosensory stimulation influences melatonin secretion from the pineal gland in anesthetized rats. Male Fischer rats were anesthetized with urethane. A microdialysis probe was inserted into the pineal gland, and phosphate buffer saline was perfused at a rate of 1 µL/min. Perfusate was collected every 20 min until 20:00 h (at the beginning of dark phase in vivarium) and the melatonin concentration of the perfusate was quantified by ELISA. As somatosensory stimulation, tactile stimulation on rat's back skin using an elastomer roller (17 mm in diameter, 15 mm in length, 4 g) or pressure stimulation on a hindlimb using a stimulation probe (6 mm in diameter, 10N/$cm^2$) was applied for 20 min. Without stimulation, melatonin concentration was stable over the sampling period (approximately 10% of variability). Tactile stimulation little affected melatonin levels. Pressure stimulation either increased or decreased melatonin levels and the magnitude of the changes was more than 30% in each animal. The present results suggest that pressure stimulation applied to the rat's hindlimb influences melatonin secretion from the pineal gland.

### Late Breaking Abstracts

[2LBA]
**Environmental physiology**

March 15 (Wed.), 12:10 - 14:10, Poster Room

### [2LBA-018]

### Volatile organic compounds emitted from human skin as potential markers of menstruation phase and severity of premenstrual syndrome in young and healthy adult women.

*Toshio Fujii[1], Nozomi Matsuura[1], Yuji Morita[1], Keiko Morimoto[2] ([1]*Research Laboratories for Health and Science & Food Technologies Kirin co., ltd.*, [2]*Department of Health and Nutrition, Faculty of Health Science, Kyoto Koka Women's University*)

(Aim) Volatile compounds emitted from skin (skin gas) can provide information on the physiological status of individuals. We investigated whether the composition of skin gas is affected by the natural menstrual cycle of women, and if there is a correlation between skin gas components and serum levels of female hormones. We also conducted a screen of skin gas to identify potential components related to the severity of premenstrual syndrome (PMS). (Methods) Twelve young healthy women were recruited and underwent blood and skin gas sampling on one day corresponding to each menstrual phase: preovulatory, middle luteal, and late luteal. Skin gas was collected at the cubital fossa and armpit by the Passive Flux Sampler method and quantitatively analyzed by GC-MS. (Results) In skin gas from the cubital fossa, 7 compounds changed significantly and 4 compounds changed marginally during menstruation, as assessed by Kruskal-Wallis test. Six of these compounds were correlated with serum levels of female hormones. We also identified several compounds that might be useful to estimate the severity of PMS. Our findings show the potential of skin gas analysis in monitoring menstruation.

### [2LBA-019]

### Changes in the single capillary blood flow of the finger nail-fold of "Hiesho" subjects during gradual cold exposure examined using videomicroscopic analysis

*Norikazu Ohnishi[1], Uchida Haruna[1], Kondo Yuka[1], Sugawara Keita[1], Nishikawa Mano[1], Yamane Motoi[2] ([1]*Mie Prefectural College of Nursing*, [2]*Aichi Mizuho College*)

Changes in the single capillary blood flow of the finger nail-fold during gradual cold exposure were compared in four subjects who were aware of their increased cold sensitivity (Hiesho) and four who were not (Control). The subjects sat in the climatic chamber in the resting position for 10 minutes while the ambient temperature (Ta) was set at 30°C and then lowered to 10°C in about 40 minutes. Blood flow in a single vessel, the image of which was most clearly observed among visible capillaries prior to the experiment, was measured every 5 minutes as Ta was reduced. Capillary blood flows were lower in Hiesho than in Control at the start of the experiment and remained lower throughout the experiment. Capillary blood flows in Control decreased to the level of those in Hiesyo as Ta fell, while temporal increases were observed in some cases in Control. These findings suggest that differences in hemodynamics in capillary blood flow between Hiesho and Control would be associated with differences in the states of constriction of arterioles and/or arteriovenous anastomoses, as well as peripheral factors, such as vasoconstrictor sensitivity between both vessels, in both groups.

### Late Breaking Abstracts

### [2LBA]
**Nutritional and metabolic physiology, Thermoregulation**

March 15 (Wed.), 12:10 - 14:10, Poster Room

### [2LBA-020]

**Feeding Behavior Analysis in Liver-specific *Acadm* knockout mice**

*Tsugunori Maruyama[1], Sho Matsui[1], Ryosuke Kobayashi[2], Takuro Horii[2], Izuho Hatada[2], Tsutomu Sasaki[1] ([1]*Laboratory of Nutrition Chemistry, Division of Food Science and Biotechnology, Graduate School of Agriculture, Kyoto University,* [2]*Laboratory of Genome Science, Biosignal Genome Resource Center, Institute for Molecular and Cellular Regulation, Gunma University*)

[Purpose] High-fat diet (HFD) is obesogenic because it mainly contains long-chain triglycerides (LCTs) with few medium-chain triglycerides (MCTs). MCT can satisfy appetite with less intake than LCT, and medium-chain fatty acids (MCFAs) have anti-obesity effects. To test if appetite can be specifically stimulated toward MCT, we analyzed feeding behavior of genetically modified mice that is deficient in metabolizing MCFAs. [Method] Because MCFAs are mainly metabolized by the liver, we generated mice with a liver-specific knockout of *Acadm* encoding medium-chain acyl-CoA dehydrogenase (cKO), which is essential for the beta-oxidation of MCFAs. We analyzed the preference for the glyceryl trioctanoate (C8-TG) solution as MCT oil solution and the corn oil solution whose main components are LCTs with two-bottle choice tests over 8 days. [Results and Discussion] Compared with control mice, cKO mice showed significantly lower preference for C8-TG solution, while no difference was observed for the corn oil solution. These results indicate that the body can discriminate between MCTs and LCTs, and MCFA metabolism specifically controls appetite for MCFAs.

### [2LBA-021]

**Transcription factor EID1, which promotes fat accumulation in hepatocytes, modulates fat accumulation in response to different glucose concentrations in the medium.**

*Mitsue Miyazaki[1], Wataru Miyazaki[1], Noriaki Shimokawa[2] ([1]*Hirosaki University Graduate School of Health Science,* [2]*Department of Food and Nutrition, Takasaki University Graduate School of Health and Welfare*)

We reported at this meeting in last year that the transcription factor EID1, which has an inhibitory effect on fat accumulation in adipocytes, can conversely promote fat accumulation in hepatocytes. Since it has been shown that EID1 suppresses transcription of GPD, an enzyme that converts glucose to triglyceride, in adipocytes, we attempted to clarify the relationship between glucose and EID1 in hepatocytes. HepG2, a human hepatoma cell line, overexpressing EID1 were cultured in medium with different glucose concentrations, and fat accumulation at 24 and 48 hours was compared by Oil Red O staining. As a result, more fat was accumulated in the medium with higher glucose concentration. In addition, the intracellular glucose concentration after 24 hours of culture was increased, indicating that the cells cultured in high glucose concentrations took up more glucose. However, GPD expression tended to be suppressed by EID1 overexpression regardless of the glucose concentration in the culture medium, which conflicts with the Oil Red O staining results. These results indicated that EID1 may not affect the conversion of glucose to triglyceride directly in hepatocytes. Further investigation of the pathway of fatty acid to triglyceride conversion in hepatocytes is warranted.

### [2LBA-022]

**Characterization of Beige Adipocyte Progenitor Cells by Single-Cell Analyses**

*Yasuo Oguri[1,2], Tsutomu Sasaki[1] ([1]*Laboratory of Nutrition Chemistry, Division of Food Science and Biotechnology, Graduate School of Agriculture, Kyoto University,* [2]*Beth Israel Deaconess Medical Center, Division of Endocrinology, Diabetes & Metabolism, Harvard Medical School*)

Beige fat cells are an inducible form of thermogenic fat cells that exist within the white adipose tissue in a scattered manner. Increased beige fat biogenesis is reported to have a positive impact on metabolic health. Therefore, beige fat cells could be a potential novel therapeutic target for the treatment of lifestyle diseases. However, the developmental origin of beige fat cells remains unclear due to adipose tissue heterogeneity.Single-cell RNA sequencing identified a unique subset of adipose progenitor cells (APCs) marked by cell surface proteins, including PDGFRα, Sca1, and CD81. Thereafter, we isolated $CD81^+$ cells using FACS and confirmed that $CD81^+$ cells give rise to beige fat cells. To test whether CD81 is required for beige fat biogenesis, CD81-deficient mice were generated using the CRISPR interference (CRISPRi) system. Compared with control mice, the CD81-deficient mice exhibited impaired beige fat biogenesis. Moreover, the loss of CD81 led to diet-induced obesity, glucose intolerance, and insulin resistance. These data suggest that CD81 marks beige APCs and controls whole-body energy metabolism.

### Late Breaking Abstracts

### [2LBA]
### Behavior, Biological rhythm, Sleep

March 15 (Wed.), 12:10 - 14:10, Poster Room

### [2LBA-023]

**The protein kinase A-binding microprotein Akain1 deficiency causes impaired context discrimination in mice.**

Kazuki Fujii[1,2,3], Yumie Koshidaka[2], Mayumi Adachi[2], Yuko Yanagibashi[2], Mina Matsuo[2], Hirofumi Nishizono[4], Nobuyuki Kurosawa[5], Yasunori Aizawa[6], *Keizo Takao[1,2,3] (*[1]Department of Behavioral Physiology, Faculty of Medicine, University of Toyama, [2]Life Science Research Center, University of Toyama, [3]Research Center for Idling Brain Science, University of Toyama, [4]Medical Research Institute, Kanazawa Medical University, [5]Laboratory of Molecular and Cellular Biology, Faculty of Engineering, University of Toyama, [6]School of Life Science and Technology, Tokyo Institute of Technology*)

Cyclic AMP-dependent protein kinase (PKA) plays a key role in the signaling of many G protein-coupled receptors. The specificity of PKA activity is a result of its cellular localization, which is controlled by a family of A-kinase anchoring proteins (AKAPs). AKAPs localize PKA to specific intracellular sites and spatially restrict intracellular signaling events. A-kinase anchor inhibitor 1 (Akain1) is a newly identified PKA-binding single-domain microprotein with a unique function. By competing with AKAPs-PKA binding, Akain1 nullifies the intracellular localization of PKA in cultured cells. Whether and how Akain1 affects the physiologic function of PKA, however, has remained unclear. To address these questions, we generated Akain1 knockout (KO) mice and Akain1 reporter mice. Utilizing the Akain1 reporter mice and anti-Akain1 antibody, we identified that endogenous Akain1 is preferentially expressed in neural tissues. To investigate the function of Akain1 in the brain, we subjected Akain1 KO mice to a comprehensive battery of behavioral tests. In the pattern separation test, Akain1 KO mice exhibited impaired performance in distinguishing between two similar contexts. In the reversal learning task of the Barnes maze test, wild-type mice spent significantly more time investigating the new target compared with the original target, whereas Akain1 KO mice demonstrated no preference between the new and original targets. These findings suggest that Akain1 is critically involved in discriminating between similar contexts.

### [2LBA-024]

**Long-term imaging of organelle $Ca^{2+}$ rhythms in the master circadian clock neurons**

*Sota Hiro[1,2], Tomomi Nemoto[1,2,3], Ryosuke Enoki[1,2,3] (*[1]Department of Physiological Sciences, School of Life Science, The Graduate University for Advanced Studies, SOKENDAI, [2]Division of Biophotonics, National Institute for Physiological Sciences, National Institute of Natural Sciences, [3]Biophotonics Research Group, Exploratory Research Center on Life and Living Systems, National Institutes of Natural Sciences*)

In mammalian animals, the rhythms of behavioral and physiological phenomena, such as sleep-wake cycles and core body temperature, are controlled by the master circadian clock located in the suprachiasmatic nucleus (SCN) of the brain. In the SCN neurons, the circadian rhythms of cytoplasmic $Ca^{2+}$ concentration have been reported, and membrane transport proteins of the endoplasmic reticulum and mitochondria, regulate the $Ca^{2+}$ rhythms (Ikeda et al. Neuron 2003, Morioka et al., Cell Reports, 2022). However, the detailed molecular mechanism of how these intracellular organelles contribute to the cytoplasmic $Ca^{2+}$ rhythm remains unclear. In this study, we performed time-lapse $Ca^{2+}$ imaging in the nucleus/mitochondria and cytoplasm of SCN neurons by expressing spectrally distinct genetically encoded $Ca^{2+}$ probes, GCaMP6s/CEPIA2mt and jRGECO1a. We found a robust circadian $Ca^{2+}$ rhythm in the nucleus in the same phase as in the cytoplasm. Pharmacological experiments showed that $Ca^{2+}$ rhythms in the nucleus and the cytoplasm were regulated by the action potentials through $Na^{+}$ channels but not the ryanodine receptor. In mitochondria, we also detected the circadian rhythm in the CEPIA2mt signal, which was in anti-phase with cytoplasmic $Ca^{2+}$ rhythm. These results suggest that mitochondria might be involved in regulating nuclear and cytoplasmic $Ca^{2+}$ rhythms.

### [2LBA-025]

**Identification of hibernation-induced resetting of the circadian body temperature rhythm in Syrian golden hamster**

*Satoshi Nakagawa[1,2], Yoshifumi Yamaguchi[1] (*[1]Hokkaido Univ., Low temperature institute, [2]Hokkaido Univ., Graduate School of Environmental Science, Division of Biosphere Science*)

Small mammalian hibernators exhibit multiple cycles of hypothermic deep torpor and normothermic periodic arousal during hibernation. Such torpor-arousal cycle is spontaneously terminated in several months even under a constant laboratory condition. Previously, we reported that the basal body temperature (Tb) of Syrian hamster falls about 1°C before onset of hibernation and recovers during hibernation period. In this study we analyzed the circadian Tb rhythm of Syrian hamsters that were maintained under constant short photoperiod and cold (SP-cold) condition. We found that at just after the end of hibernation period, the acrophases of circadian Tb rhythm was similar to that observed in the summer-like long photoperiod and warm (LP-warm) condition in spite of being kept in constant SP-cold condition. Moreover, the acrophases of circadian Tb rhythm was gradually changed, as if the hamsters were transferred from LP-warm to SP-cold. On the other hand, such changes in circadian Tb rhythm did not occur in hamsters that never hibernated under SP-cold condition. These results suggest that the Syrian hamster has an endogenous system which reset the circadian Tb rhythm from winter-like to summer-like form in a hibernation-dependent manner.

### [2LBA-026]

**Reward announcements promote suboptimal choice in mice**

*Hiroyuki Ohta[1], Toshiaki Ishizuka[1] (*[1]National Defense Medical College*)

The current treatment of process addictions, such as gambling and gaming addictions, focuses on psychotherapy, and effective pharmacotherapy has not been established. One of the reasons for this is the lack of appropriate behavioral test batteries for mice to identify the cause of addiction and to conduct pharmacological tests. In response, we have developed a novel operant conditioning task for mice that induces irrational choice. In a two-choice task, one choice (A) is staged to announce a reward, while the other choice (B) is not staged to announce a reward. When the reward probabilities of both options were equal, the mouse preferred option (A) but maintained adherence to (A) even when the reward probability of option (A) decreased. In other words, we succeeded in reproducing in mice the addiction to the "reward announcement," which plays an essential role in forming gambling disorder. This study will allow future neuroscientific studies on the formation of gabmbling disorder and process addiction.

### Late Breaking Abstracts

[2LBA]
**Study Methodology**

March 15 (Wed.), 12:10 - 14:10, Poster Room

### [2LBA-027]

### Development of cell-type specific AAV vectors in the mouse brain

*Naofumi Uesaka[1], Ryo Masumura[1], Mariko Sekiguchi[1] ([1]*Tokyo Medical and Dental University*)

A major goal of the neuroscience field is to understand how each cell generates complex brain functions and how dysfunction of each cell leads to brain diseases. These require defining each type of cell and building genetic tools that can label and manipulate cells in a cell-type specific manner. Recent developments in technologies such as single-cell RNA sequencing have catalogued the gene expression profiles of distinct cell populations and have successfully defined each type of cell based on gene expression patterns. This has led to the understanding that there is a vast range of cell types in the brain, but the access to each type of cell is limited. Adeno-associated virus (AAV) vectors have been shown to be able to transfer genes of interest into neurons and glial cells from mouse to human. Furthermore, AAV has shown possibilities as a clinical tool for treating a variety of brain diseases. By approaching and genetically manipulating specific type of cell with AAV, it is possible to elucidate the cellular mechanisms underlying brain function and to treat a variety of brain diseases. However, AAV vectors which enable us a cell type-specific manipulation are limited. In this study, we will report on our progress of cell type-specific AAVs for the cerebellum that combine promoters and enhancers.

### [2LBA-028]

### Two-photon microscopy image deblurring and super-resolution via deep neural network and generative model

*Haruhiko Morita[1], Shuto Hayashi[1], Yasuhiro Kojima[1], Daisuke Kato[2], Takahiro Tsuji[2], Hiroaki Wake[2], Teppei Shimamura[1] ([1]*Nagoya University Graduate School of Medicine Division of Systems Biology,* [2]*Nagoya University Graduate School of Medicine Department of Anatomy and Molecular Cell Biology*)

Two-Photon Microscopy (TPM) enables deep-tissue live imaging. However, its axial resolution is inferior to the lateral resolution, and this makes it difficult to reconstruct the three-dimensional structure of cellular details such as synapses or microglia spines. Previous studies have been insufficient for improving deep-tissue TPM images, or they are not suitable for live imaging. We built a deep neural network that deblurs and improves the axial resolution of TPM images ("deblurring model"). Since we do not have the true structures of objects in TPM images, the deblurring model was trained in combination with a blurring generative model simulating the blurring process of TPM.For quantitative evaluations, we first adapted our model to simulation data and real images of beads, and we found that our model accurately inferred the true shapes of the objects. Secondly, we adapted our model to images of axons, and we found that the model deblurred images and improved image resolution, resulting in providing more clear cellular shapes.We expect this method enables more accurate evaluations of the three-dimensional structure of the living cells in deep tissue.

### [2LBA-029]

### Estimation of RNA splicing and degradation rates by generative models

*Chikara Mizukoshi[1], Kojima Yasuhiro[2], Shimamura Teppei[1,2] ([1]*Nagoya University Graduate School Of Medicine Division Of Systems Biology,* [2]*Tokyo Medical and Dental University Medical Research Institute Division Of Computational and Systems Biology*)

Single-cell RNA sequencing (scRNA-seq) can provide a variety of biological insights by quantifying mRNA levels at the single-cell level. scRNA-seq could only provide a snapshot of information at one time, but with the development of RNA velocity, we now have dynamic information on how cells are changing. However, until now, the derivation of RNA velocity was limited to the splicing rate and degradation rate of RNA, which must be constant from cell to cell. Since the splicing and degradation rates of RNA vary from cell to cell in vivo, accurate derivation of these parameters will reveal new regulatory mechanisms for splicing and degradation dynamics. We used the RNA velocity equation and variational autoencoder to postulate a model in which splicing and degradation rates vary depending on the cellular state, and estimated those rates using actual data. To prove the accuracy of our estimates, we compared them to the observed degradation rates and checked their consistency with the splicing rates set in the simulation data. This approach allows us to determine the patterns of splicing and degradation rate changes and to identify their control mechanisms.

### [2LBA-030]

### Improvement of CRISPRi for effective gene regulation

*Nodoka Oki[1], Takeshi Kondo[1], Shigetsugu Hatakeyama[1] ([1]*Faculty of Medicine and Graduate School of Medicine, Hokkaido University*)

Genome editing technology based on the CRISPR/Cas9 system enables us to delete specific genes from cells. However, irreversible lack of genes often results in difficulty in cell cloning and cytotoxicity, which limits its utility. A nuclease-inactive Cas9 (dCas9) can be used for various applications including CRISPR interference (CRISPRi). CRISPRi is a superior method for temporal gene knockdown with less off-target effects, less competition with endogenous machinery, and less effort and cost for preparation. Although some improved methods have been reported (Science, 2018, 361, 866-869), overcoming its inefficiency of gene silencing is still challenging. In this study, we tried to improve CRISPRi by the fusion of a transcriptional regulator X to dCas9 (dCas9-X). First, we optimized the domain structure of dCas9-X by comparing 6 truncated mutants of transcriptional regulator X. Next, we assessed whether dCas9-X 1.0, the most effective construct among the tested 6 mutants, outperforms one of current versions of CRISPRi, dCas9-KRAB-MeCP2 (Nat. Methods, 2018, 15, 611-616). As a result, dCas9-X 1.0 showed stronger silencing of target genes compared to dCas9-KRAB-MeCP2 in most conditions. In addition, we demonstrated advantages of dCas9-X 1.0 in specificity of gene targeting and reversibility of gene expression control. These results suggest that dCas9-X 1.0 is superior to some existing CRISPRi platforms and would be useful to evaluate the functions of genes of interest.

### [2LBA-031]

### SSBD:database / SSBD:repository - global sharing of bioimaging data

*Hiroya Itoga[1], Fangfang Wang[1,2], Yuki Yamagata[1,2], Koji Kyoda[1], Yukako Tohsato[1,3], Shuichi Onami[1,2] ([1]*RIKEN Center for Biosystems Dynamics Research,* [2]*RIKEN information R&D and Strategy Headquarters,* [3]*Faculty of Information Science and Engineering, Ritsumeikan University*)

Sharing and reusing microscopy images and biological dynamics data will lead to the discovery of new knowledge in life science; therefore, we have developed SSBD:database since 2013 (https://ssbd.riken.jp). SSBD consists of two systems; one is SSBD:repository, an archive service that quickly shares all kinds of bioimaging data for published or to-be-published papers. The other is SSBD:database, an added-value database that shares highly reusable bioimaging data with rich curator-annotated metadata. It provides previews of microscopy images, access APIs, and visualization of biodynamics data on the web. SSBD:repository is suitable for journal-mandated publication of datasets because it accepts large datasets and issue DOI. Currently SSBD:repository and SSBD:database share more than 8,000 datasets / 26.2 TB of bioimaging data and 696 datasets / 344GB of analyzed, quantitative biodynamics data in the manners of the FAIR principle. Now we are constructing a global sharing system of bioimaging data in collaboration with research groups in Japan, Europe, and North America. The system will share and reuse bioimaging data worldwide in the same search way, standard access methods, and unified data formats.

### [2LBA-032]

### A method for acutely dissociating melanocytes from mouse leptomeninges

*Katsuhiro Nagatomo[1] ([1]*Department of Physiology, Hirosaki University Graduate School of Medicine*)

A well-known function of melanocytes, dark-pigmented cells, is the protection of DNA from ultraviolet light. Fishes and amphibians commonly have translucent bodies at juvenile stages in which extracutaneous melanocytes shade hematopoietic stem and progenitor cells. In mammals, extracutaneous melanocytes are localized in the eye, inner ear, heart, and leptomeninges. Because the skull covers the leptomeninges, UV cannot pass through and reach the leptomeningeal melanocytes. So what is the actual physiological function of the melanocytes in the dark place, other than meaningless UV protection? Unfortunately, however, any reports on their functions could not be found. In this study, I developed a method for preparing single leptomeningeal melanocytes by dissociating enzymatically and mechanically. Although the method might be needed additional adjustment of the dissociating condition, it will be helpful for investigations of the physiological functions of the other extracutaneous melanocytes. (COI: NO)

### Late Breaking Abstracts

Day 3
(March 16, 12:10 - 14:10)

[3LBA] Neurophysiology, Neuronal cell biology - Glia
[3LBA] Neurophysiology, Neuronal cell biology - Sensory function, Sensory organ
[3LBA] Embryology, Regenerative Medicine, Development, Growth, Aging
[3LBA] Muscle
[3LBA] Nutritional and metabolic physiology, Thermoregulation
[3LBA] Behavior, Biological rhythm, Sleep
[3LBA] Drug Action, Pharmacology
[3LBA] Others

### Late Breaking Abstracts

### [3LBA]
**Neurophysiology, Neuronal cell biology**
**Glia**

March 16 (Thu.), 12:10 - 14:10, Poster Room

### [3LBA-001]

**Anti-inflammatory effects of bromovalerylurea mediated by the activation of NRF2**

*Shoko Miyoshi[1], Haruna Takeda[1], Yamaguchi Teruaki[1], Haijme Yano[1], Junya Tanaka[1]
([1]*Department of Molecular and Cellular Physiology, Graduate School of Ehime University Medical School*)

The Kelch-like ECH-associated protein 1–nuclear factor erythroid 2-related factor 2 (KEAP1–NRF2) system plays a central role in redox homeostasis and inflammation control. Oxidative stress or electrophilic compounds promote NRF2 stabilization and transcriptional activity by negatively regulating its inhibitor, KEAP1. We have previously reported that bromovalerylurea (BU), originally developed as a hypnotic, exerts anti-inflammatory effects in various inflammatory disease models. However, the molecular mechanism underlying its effect remains uncertain. Herein, by real-time multicolor luciferase assay using stable luciferase red3 (SLR3) and green-emitting emerald luciferase (ELuc), we found that BU potentiates NRF2-dependent transcription in the human hepatoblastoma cell line HepG2 cells, which lasted for more than 60 hr. Further analysis revealed that BU promotes NRF2 accumulation and the transcription of its downstream cytoprotective genes in the HepG2 and the murine microglial cell line BV2. Keap1 knockdown did not further enhance NRF2 activity, suggesting that BU upregulates NRF2 by targeting KEAP1. Knockdown of Nrf2 in BV2 cells diminished the suppressive effects of BU on the production of pro-inflammatory mediators, like nitric oxide (NO) and its synthase NOS2, indicating the involvement of NRF2 in the anti-inflammatory effects of BU. These data collectively suggest that BU could be repurposed as a novel NRF2 activator to control inflammation and oxidative stress.

### [3LBA-002]

**Development of a method for the gene expression preferentially in microglia using an adeno-associated virus vector.**

*Ayumu Konno[1], Yukihiro Okada[1], Yasunori Matsuzaki[1], Hirokazu Hirai[1] ([1]*Gunma University*)

Microglia, which are immune cells that reside in the central nervous system, are known to provide surveillance and scavenging functions. In this study, we developed the microglia-targeting adeno-associated viral (AAV) vectors containing a 1.7-kb putative promoter region of microglia/macrophage-specific ionized calcium-binding adaptor molecule 1 (*Iba1*), along with repeated miRNA target sites for microRNA (miR)-9 and miR-129-2-3p. The 1.7-kb genomic sequence upstream of the start codon in exon 1 of the *Iba1* (*Aif1*) gene, functions as microglia preferential promoter in the striatum and cerebellum. Furthermore, ectopic transgene expression in non-microglial cells is markedly suppressed upon adding two sets of 4-repeated miRNA target sites for miR-9 and miR-129-2-3p, which are expressed exclusively in non-microglial cells and sponged AAV-derived mRNAs. Our vectors transduced ramified microglia in healthy tissues and reactive microglia in lipopolysaccharide-treated mice and a mouse model of neurodegenerative disease. Thus, microglia-targeting AAV vectors are valuable for studying microglial pathophysiology and therapies, particularly in the striatum and cerebellum. ReferenceOkada Y, Hosoi N, Matsuzaki Y, Fukai Y, Hiraga A, Nakai J, Nitta K, Shinohara Y, Konno A, Hirai H.Development of microglia-targeting adeno-associated viral vectors as tools to study microglial behavior in vivo.Commun Biol., 5(1):1224.https://www.nature.com/articles/s42003-022-04200-3

### [3LBA-003]

**Analysis of the effects of motor learning on oligodendrocyte differentiation and myelination**

*Akari Suzuki[1,2], Shouta Sugio[2,3], Daisuke Kato[1,2,3], Hiroaki Wake[1,2,3] ([1]*Nagoya University Graduate School of Medicine,* [2]*Department of Anatomy and Molecular Cell Biology, Nagoya University Graduate School of Medicine,* [3]*Division of Multicellular Circuit Dynamics, National Institute for Physiological Sciences (NIPS)*)

### [3LBA-004]

**Labeling of microglia focusing on blood-brain barrier permeability**

*Yuki Aoyama[1], Daisuke Kato[1,2], Hiroshi Yukawa[3], Hiroaki Wake[1,2] ([1]*Department of Anatomy and Molecular Cell Biology, Nagoya University Graduate School of Medicine,* [2]*Division of Multicellular Circuit Dynamics, National Institute for Physiological Sciences, National Institutes of Natural Sciences,* [3]*Institute of Nano-Life-Systems, Institutes of Innovation for Future Society Nagoya University*)

Microglia, brain immune cells with ramified and highly motile processes, constantly surveil the parenchyma. Previous study showed that microglia contribute blood-brain barrier (BBB) permeability, and are suggested to be involved in the pathogenesis of psychiatric and neurological disorders. In addition, dysregulation of microglia express pattern recognition receptors (PRRs) expressed in microglia induced signal activation is known to lead to neuroinflammatory responses associated with various neurodegenerative diseases. However, little is known about the effects of microglial PRRs-mediated signaling on the BBB. Previous studies have shown that microglia accumulate around blood vessels and elevated vascular permeability in systemic inflammation induced model mice. In this study, we used *in vivo* two photon imaging of Cx3cr1-EGFP mice to examine how alters the microglia dynamics and the BBB permeability induced by systemic administration of lipopolysaccharide (LPS). We found that the microglia migration didn't decrease significantly after NLRP3 inhibitor treatment, but vascular relative leakage was restored by this treatment. Furthermore, we found that microglia seal blood vessels, captured quantum dots from blood vessels, and microglia relocate after stopping LPS administration. Therefore, in order to track these accumulated microglia in blood vessels in response to inflammation, we are considering tracking the dynamics of microglia by administering magnetic nanoparticles via BBB. These findings are expected to contribute to the development of novel therapies for neuropsychiatric disorders by targeting microglia to regulate the BBB permeability.

### [3LBA-005]

**Application of a novel AAV-mediated microglia-specific gene expression method using microRNA-target sequences and examination of their influence on neural function as controls**

*Nobutake Hosoi[1], Yukihiro Okada[1], Yuuki Fukai[1], Akito Hiraga[1], Yasunori Matsuzaki[1], Junichi Nakai[2], Ayumu Konno[1], Hirokazu Hirai[1] ([1]*Department of Neurophysiology and Neural Repair, Gunma University Graduate School of Medicine,* [2]*Division of Oral Physiology, Department of Oral Function and Morphology, Tohoku University Graduate School of Dentistry*)

Microglia, known as a brain immune cell, has diverse functions in the central nervous system including brain development, neuronal remodeling, and neurodegenerative diseases. In order to examine the mechanisms of microglial functions precisely, microglia-specific experimental manipulation is required. Recently, we have developed a novel microglia-targeting AAV2/9 vector (AAV9 capsid and AAV2 viral genome) using a combination of the microglia/macrophage-specific Iba1 promoter and microRNA target sequences (the sequences against microRNA-9 and microRNA-129-2-3p that are both expressed selectively in non-microglial cells in the brain), leading to transgene silencing in non-microglial cells, although this AAV vector works reasonably only in the cerebellum and the striatum. In this presentation, we apply this AAV vector to live fluorescent imaging of microglial motility and its intracellular $Ca^{2+}$ dynamics in the acute cerebellar slices. In addition, we confirmed that the microRNA target sequences used in this microglia-targeting AAV vector have no effect on neural function, because there was no abnormality in motor behavior nor electrophysiological properties of cerebellar neurons when those microRNA target sequences were overexpressed widely in neurons and/or other cells through the brain with intravenous injection of the blood-brain-barrier-penetrating AAV-PHP.B vector carrying the ubiquitous and strong CBh promoter. These results suggest that our microglia-targeting AAV vector can be a powerful tool for elucidating microglial functions in the cerebellum and the striatum.

### [3LBA-006]

### Brain environmental changes precede neuronal activity shifts to REM sleep

*Yusuke Takahashi[1], Daichi Sasaki[1], Yoko Ikoma[1], Ko Matsui[1] ([1]*Super-network Brain Physiology, Graduate School of Life Sciences, Tohoku University*)

Astrocytes control the local ionic and metabotropic environment in the brain, but they have not been considered an essential component of the neural information circuit. To understand the role of astrocytes in brain function, fluorescent sensor proteins were genetically expressed in the astrocytes of mice. We implanted an optical fiber into the mouse lateral hypothalamus, a part of the brain known to be vital for sleep/awake control and metabolism. A clear change in the optical signals associated with REM sleep was observed. Astrocyte acidification was especially unexpected, as the intracellular solution of cells is highly buffered for pH. This astrocyte acidification may drive the amplification of synaptic signals and may underlie memory formation during REM sleep. Interestingly, changes in the local brain environment detected with the optical recordings preceded the signature change of the ensemble neuronal electrical activity detected with electroencephalogram by nearly 20 seconds. Transition to REM sleep can also be predicted from these local brain environmental changes. This suggests that astrocytes and vascular changes have control of the state of neuronal activity.

### [3LBA-007]

### Elucidation of the invasion mechanism of human-derived glioma cells via L1CAM

*Asako Katsuma[1,2], Daisuke Kanematsu[1], Tomoko Shofuda[1], Naoyuki Inagaki[2], Yonehiro Kanemura[1] ([1]*Department of Biomedical Research and Innovation, Institute for Clinical Research, National Hospital Organization Osaka National Hospital,* [2]*Laboratory of Systems Neurobiology and Medicine, Division of Biological Science, Nara Institute of Science and Technology*)

Gliomas are malignant intracranial tumors and are one of the rare cancers. They show highly invasive and migratory potential, and thereby are difficult to cure with current standard therapies. The analysis of the mechanism of glioma cell migration may lead to the prevention of their invasion and contribute to the improvement of therapeutic outcomes. Glioma stem cells (GSCs) play a central role for glioma formation and maintenance. Although there are many studies suggest biological properties of GSCs, their motility has not been fully revealed. In order to characterize the mechanobiological mechanism of GSCs migration and its relationship to the acquisition of tumor invasiveness, we focused on the mechanobiological roles of cell adhesion molecule L1CAM in GSCs. L1CAM is expressed during nervous system development, and also well-known as a tumor marker and the association of prognosis. We established GSCs from patient-derived glioblastoma tissues using neutrosphere method, and report L1CAM high GSCs and null GDCs. We evaluated their motility by the single-cell tracking method. Migration distance of L1CAM–positive GSCs on laminin was greater than that of L1CAM–negative GSCs. Inhibition of L1CAM function by antibody addition impaired GSCs motility, and conversely, forced expression of L1CAM resulted in promotion of GSCs migration. As haptotaxis, the movements on Laminin and Fibronectin were compared. Migration on Laminin, which has been reported to bind to L1CAM, was significantly higher. As a molecular mechanism, we quickly report that forced expression of L1CAM increased the rate of retrograde actin flow.

### Late Breaking Abstracts

[3LBA]
**Neurophysiology, Neuronal cell biology**
**Sensory function, Sensory organ**

March 16 (Thu.), 12:10 - 14:10, Poster Room

### [3LBA-008]

### Prior vestibular/visual combined stimuli suppress motion sickness symptoms in mice

*Akira Katoh[1], Nanami Nakaya[2], Fumiko Shimura[2], Minoru Kimura[1] ([1]*Tokai University School of Medicine*, [2]*Tokai University, School of Engineering*)

Motion sickness (MS) is a disease that occurs with continuous shaking of the head on a moving vehicle, exhibiting symptoms such as dizziness and nausea. It is mostly accepted that the main cause of MS is a conflict between vestibular/visual perception in the situation when one's surroundings are shaken along with oneself, unlike in daily life. Here we examined the effects of vestibular stimuli by vertical/lateral/fore-aft translation(s) with visual stimulus fixed with head motion on the amount of food intake in mice, in order to establish an experimental system for MS using mice and to develop a method for preventing/reducing symptoms of MS without using drugs. C57BL/6 mice (n = 12) ate significantly less of the regular food (-26%) after 1h of combined vertical/fore-aft translations at 0.5 Hz given with a head-fixed visual stimulus as well as increases of the concentration of the corticosterone in urine, while combined vertical/lateral translations or any of linear vestibular stimuli in the single direction did not change the amount of regular food intake. A fore-aft translation with a head-fixed visual stimulus at 0.5 Hz given for 30 min right before the combined stimuli inducing MS described above suppressed anorexia, resuming the amount of food intake. Our results suggest that vestibular/visual combined stimuli given in advance can relieve MS symptoms, probably by changing eye movements to reduce a mismatch between motion-derived neural signals. This work was supported by the Japan Society for the Promotion of Science (JSPS) Grants-in-Aid for Scientific Research JP21K06847 to AK and MK.

### [3LBA-009]

### Neonatal sex steroids induce a sex difference in pain behavior in mice

*MOEKO KANAYA[1], MARIKO MIYATA[1] ([1]*Division of Neurophysiology, Department of Physiology, School of Medicine, Tokyo Women's Medical University*)

There is a sex difference in pain. Females report more severe pain and longer-lasting pain than males in rodents. We aimed to compare sex steroid actions in neonatal and adult periods on the sex difference in pain behavior. Female mice pups were injected subcutaneously with estradiol benzoate or testosterone propionate within 5 days after birth. We also prepared ovariectomized adult females and castrated adult males. The formalin test resulted that formalin-induced licking behavior was longer in ovariectomized females than in gonadal intact males. However, this sex difference disappeared by neonatal manipulation of sex steroids. That is, the licking time in neonatal testosterone- or estradiol- treated females were significantly shorter than that in neonatal sesame oil-treated females and was similar to that in gonadal intact males. On the other hand, the sex difference was not altered by sex-steroid manipulation in adulthood. These results indicate that neonatal sex steroids induced the male-type pain response in inflammatory pain. Previous reports display an involvement of spinal cord microglia in male pain processing but not in female one. Thus, we next investigated whether the male-specific spinal microglial dependence on pain is organized by neonatal sex steroids. Gonadal intact males showed a decrease of licking time caused by pharmacological microglia ablation using PLX3397 diet, although castrated males did not show the effect of microglia depletion. Therefore, in males, the pain regulation by microglia may work under existence of testosterone. In neonatally testosterone- or estradiol-treated females, there was no effects of microglia ablation on licking time. However, when these females were implanted with testosterone tube in adulthood, they displayed an effect of microglia ablation, inducing a decrease in licking behavior. Taken together, neonatal sex steroids may be requisite for formation of the male-typed pain processing via microglia, which is regulated by testosterone.

### [3LBA-010]

### The dynamics of acetylcholine in the whole cortex of the mouse learning and performing the visual detection task.

*Akinori Y Sato[1], Ryosuke Takeuchi[1], Kei Ito[1], Masahiro Yamaguchi[1], Fumitaka Osakada[1,2,3] ([1]*Lab. of Cellular Pharmacology, Grad. Sch. of Pharmaceut. Sci., Nagoya Univ.*, [2]*Lab. Neural Info. Proc., Inst. Adv. Res., Nagoya Univ.*, [3]*NLS, Inst. Inn. Fut. Soc., Nagoya Univ.*)

The activity of the brain dynamically changes depending on the animal's conditions, such as behavioral contexts and psychological states. The dynamics of neural activity are regulated primarily by neuromodulators, such as acetylcholine, regulating various brain functions including visual function. However, the neural mechanism of the modulation on visual function remains elusive because little research has focused on other brain regions than the primary visual cortex (Pinto et al., 2013). The present study aims to examine the relationship between neuromodulation, visual function, and learning with a focus on the brain-wide effects of acetylcholine. We observed the distribution of concentrations of acetylcholine across the mouse brain using wide-field imaging of a genetically encoded fluorescent sensor of acetylcholine, iAChSnFR (Borden et al., 2020). To measure the visual perception of mice, we developed a visual detection task for head-fixed mice. Wide-field imaging of iAChSnFR from the mice during the task demonstrated that the iAChSnFR signals changed depending on whether the mice licked the spout to report the stimulus detection. The response dynamics showed different patterns in the anterior or posterior cortex regions with higher amplitude in the anterior region, such as the secondary motor cortex. We will present changes in the response to the visual stimulus as the learning of the task progresses.

### [3LBA-011]

### Optogenetic activation of somatosensory afferents in non-human primates by systemic AAV administration

*Akito Kosugi[1], Moeko Kudo[1], Ken-ichi Inoue[2], Masahiko Takada[2], Kazuhiko Seki[1]
([1]*National Center of Neurology and Psychiatry*, [2]*Kyoto University*)

Damage to the central nervous system such as stroke and spinal cord injury causes maladaptive change in the peripheral neural circuit. For example, spasticity, which is common complication of stroke, is caused by hyperexcitability of spinal reflex. Although reducing somatosensory input to the spinal cord can prevent triggering spasticity, no clinical treatment provides selective and reversible manipulation of somatosensory afferents to date. Here, we aimed to develop a method to selectively manipulate somatosensory afferents in non-human primates using optogenetic techniques. First, we investigated the optimal serotype and administration route of adeno-associated virus (AAV) vectors to transduce green fluorescent protein into dorsal root ganglion (DRG) neurons in common marmoset, a small New World monkey. We found that systemic administration through intravenous injection of the capsid variant of serotype-9 (AAV-PHP. B) expressed green fluorescent protein in the dorsal horn of the spinal cord and large-sized DRG neurons selectively. Then, channelrhodopsin 2 (ChR2) was transduced into DRG neurons in the same way. To test whether optical stimulation selectively activate afferent nerve fibres *in vivo*, we performed acute electrophysiological experiments 4−5 weeks after the viral injection. Electrical or optical stimulation were applied to radial nerve and stimulus evoked responses were recorded by cervical dorsal root and wrist extensor muscle. We observed optically-evoked responses only at cervical dorsal root, but not wrist muscle. This suggests that optical stimulation selectively activated afferent nerve fibres. In addition, we confirmed that the expression of ChR2 was localized in the dorsal horn of the cervical spinal cord and large-sized DRG neurons. Furthermore, the expression level of ChR2 and the amplitude of the optically-evoked responses were increased depending on injected viral titer. These results demonstrate the feasibility of the selective manipulation of somatosensory afferents in non-human primates using systemic AAV administration and optical nerve stimulation. This is the first study to demonstrate selective and reversible manipulation of somatosensory afferents in non-human primates. This is an important advancement toward novel gene therapy for abnormal peripheral neural circuit such as spasticity.

### Late Breaking Abstracts

[3LBA]
**Embryology, Regenerative Medicine, Development, Growth, Aging**

March 16 (Thu.), 12:10 - 14:10, Poster Room

### [3LBA-012]

### Generation of *c-Fos* knockout rats, and observation of their phenotype

*Yuki Yoshimura[1], Kazuomi Nakamura[2], Misako Seno[3], Misa Mochizuki[4], Kenji Kawai[4], Satoshi Koba[1], Tatsuo Watanabe[1], Takeshi Hiyama[1] (*[1]Division of Integrative Physiology, Faculty of Medicine, Tottori University, [2]Advanced Medicine Innovation and Clinical Research Center, Tottori University Hospital, [3]Advanced Medicine & Translational Research Center, Organization for Research Initiative and Promotion, Tottori University,, [4]Pathology Center, Central Institute for Experimental Animals*)

c-Fos is one of the Fos family proteins and functions as a transcription factor. Since c-Fos is transiently expressed immediately in neuron after activation, it is widely used as a marker of neuronal activation. Therefore, reporter mice in which reporter gene was inserted in endogenerous *c-Fos* have been used to elucidate neural circuits. But, rat which are larger than mice, have been used as experimental animals in neurophysiology because it is easier to perform surgical treatment to brain regions. Therefore, *c-Fos* reporter rats would be very useful. However, it was difficult to generate gene engineered rats. In addition, despite the deletion of *c-Fos* is known to result in a severe phenotype in mice, there is no report that have demonstrated this in rat. In this study, we generated *c-Fos*-deficient rats using gene editing technology, the CRISPR-Cas system, and analyzed their phenotypes. Rats with homozygous deletion of 1067 bp including the translation start site of *c-Fos* (*c-Fos* KO rats) showed small body size, abnormal teeth and bones, while heterozygous KO rats showed no such phenotypes. The results suggest that even if abnormalities in *c-Fos* expression are observed in genetically modified rats targeting endogenous *c-Fos*, the use of heterozygous rat may not interfere with the required experiments. The generation of *c-Fos* reporter rats is strongly expected in the future.

### [3LBA-013]

### Pax6 expression analysis in anterior pituitary stem cells of adult rodent.

*kanako saito[1], toshiki kameyama[1], Katsuyuki Kunida[1], miho kawata[1], yu kodani[1], akira nakashima[1], hiroshi nagasaki[1] (*[1]Fujita Health University*)

The adenohypophysis is an important organ that plays a central role in regulating multiple endocrine organs by peptidergic hormones. Recent studies have shown, in adult rodents, that sox2 expressing pituitary stem cells (PSCs) are present in the anterior pituitary, and that two types of stem cell niches (marginal cell layer (MCL) and parenchymal) are present. Pax6 is one of the important transcription factors during development of the central nervous system. It is expressed mainly in neural stem cells and is thought to regulate spatiotemporal gene expression during embryogenesis. Although its role during development has been well studied, however, little is known in adult pituitary gland. In this study, we found that Pax6 colocalized with several markers of PSCs in adult rats. As a first step, we examined the localization of Pax6 in detail by immunohistochemistry. We found that Pax6 is expressed more strongly in parenchymal PSCs than in MCL PSCs. Many Pax6(+) cells were present in the ventral side of the parenchyma.
In the future, we would like to elucidate the mechanism of PSC maintenance by studying how Pax6 is involved in two types of MCLs.

### [3LBA-014]

### A Study on Functional Aging in C57BL/6N Mice

*So Min Lee[1], Jeeyoun Jung[2] (*[1]KM Convergence Research Division, Korea Institute of Oriental Medicine, Daejeon, Korea, [2]KM Science Research Division, Korea Institute of Oriental Medicine, Daejeon, Korea*)

In many studies comparing young and old mice individuals, it has been reported that there are significant behavioral differences between the groups, which is called behavioral dysfunctions due to aging. However, relatively little is known about the comparison of behavioral changes from young adulthood to middle age and differences in renal urinary concentration. Here, to investigate age-related behavior and urinary concentration changes in mice, grip strength, endurance, memory, and urine osmolality and ion were measured in 2-month-old and 10-month-old mice. Although there are individual differences in the grip strength, Y maze, and treadmill tests, there was a significant decrease in the middle-aged group compared to the young group. In addition, when comparing the young group and the middle-old group, the volume of urine was low, however, the urine osmolality was high. Also, in urine electrolyte analysis, Na+, K+, and Cl- were higher in the middle-old group than young group. Behavioral and renal function analysis for aging can be affected by individual differences because the rate of aging is different for each individual. Nevertheless, our results confirmed a physical and functional decline in the middle-old group compared to the young group. We suggest that middle-aged health care can delay aging, and further studies on functional improvement of the kidneys, particularly, are needed.

### Late Breaking Abstracts

[3LBA]
**Muscle**

March 16 (Thu.), 12:10 - 14:10, Poster Room

### [3LBA-015]

### Study the effects of Asian herbal medicines for the treatment of sarcopenia patients

Sompot Jantarawong[2], *Yuya Yamaguchi[1], Kazuya Hasegawa[3], Asron Sani[2], Wipapan Khimmaktong[2], Pharkphoom Panichayupakaranant[2], Yutthana Pengjam[2] ([1]*Toho university,* [2]*Prince of Songkla University,* [3]*Teikyo Heisei University*)

Sarcopenia is a disease of regular loss of muscle mass due to the imbalance of protein synthesis and proteolysis process which increases with aging, significantly. Currently, its treatment consists of non-drug and drug therapies, but the use of medication can cause various side effects. Therefore, it is important to research on some effective herbal medicines that can modulate muscle mass. In this study, the inhibition effects of purified curcuminoids, curcumin (Cu), demethoxycurcumin (De), bisdemethoxycurcumin (Bis), curcuminoids-rich extract (CRE), and high water-soluble derivatives of CRE (CRE-SD, CRE-Bin, and CRE-Ter), α-mangostin and ellagic acid on dexamethasone-induced muscle atrophy in differentiation of C2C12 cells was investigated by using various methods such as; MTT and LDH assay for cell viability and cell cytotoxicity, qRT-PCR for gene expression analysis and western blot for protein analysis. The results revealed that the differentiated C2C12 cells treated with Cu, CRE, CRE-Bin, CRE-Ter, α-mangostin and ellagic acid reduced Atrogin-1 and MuRF-1 expression, whereas CRE-SD reduced only MuRF-1 expression. The results of western blot analysis indicated that Cu, CRE, CRE-SD, CRE-Bin, CRE-Ter, α-mangostin and ellagic acid upregulating phospho-Akt, an important protein in mTOR signaling pathway which is crucial pathway for protein synthesis. The current study concludes that Cu, CRE, CRE-Bin, CRE-Ter, α-mangostin and ellagic acid inhibited muscle atrophy by decrease expression of Atrogin-1 and MuRF-1 for inhibiting protein degradation and upregulation phospho-Akt to stimulating protein synthesis. The findings may help the therapeutic potentials as an evidence treating sarcopenia patients.

### [3LBA-016]

### Investigation of the effects of AGEs on the molecular mechanism of protein metabolism in mouse skeletal muscle

*Haiyu Zhao[1], Hayashi Tatsuya[1], Tatsuro Egawa[1] ([1]*Kyoto Univ.*)

Skeletal muscle function is reported to decline with age. Advanced glycation end products (AGEs) generated by the non-enzymatic protein glycation accumulates in the body with age. This study aimed to clarify the direct effects of AGEs on protein metabolism pathway of skeletal muscle by using isolated skeletal muscle of mice. Isolated extensor digitorum muscle from mice was incubated in Krebs buffer with AGEs (1 mg/ml) or bovine serum albumin (BSA) for 2 or 6 h. It was found that AGEs significantly ($p<0.01$) suppressed protein synthesis rate at 6 h compared with BSA, but not at 2 h. In addition, AGEs significantly ($p<0.05$) reduced phosphorylation of mechanistic target of rapamycin (mTOR) $Ser^{2448}$ and 70-kDa ribosomal protein S6 kinase (p70S6K) $Thr^{389}$ compared with BSA at 6 h, but not at 2 h. On the other hand, AGEs did not change the expression of proteolysis signaling markers such as atrogin-1, muscle RING-finger 1 (MuRF1), p62, and microtubule-associated protein light chain 3 (LC3). Collectively, this study suggests that AGEs act directly on skeletal muscle to inhibit protein synthesis signaling, but have no effect on protein degradation pathways in mouse skeletal muscle.

### [3LBA-017]

### Changes of skeletal muscle properties in a mammalian hibernator, Syrian hamster

*Nanaka Matsuoka[1,2], Yoshifumi Yamaguchi[1,2] ([1]*Hokkaido University Graduate school of Environmental Science,* [2]*Institute of Low Temperature Science Hokkaido university*)

Hibernation enables animals to survive starvation or during cold season with low body temperature and chronic immobilization. Syrian hamsters (*Mesocricetus auratus*), a small hibernator, undergoes a transition between two states, deep torpor, a hypothermic state of immobility lasting several days, and periodic arousal, a euthermic state of activity lasting about one day. In non-hibernators, including human, such long-lasting immobility state causes skeletal muscle atrophy and hypoactivity whereas it was reported that some hibernators maintain skeletal muscle function until the end of hibernation. We previously reported biased expression of fast-twitch muscle fiber Type 2b in *Latissimus dorsi muscle* of the Syrian hamster and reduced expression of myostatin in hibernating animals (99th meeting of Physiological Society). Here we conducted detailed analysis and found that atrophy of fast-twitch fibers Type2a/Type2b/Type2x was observed from non-hibernation period to hibernation period, while slow-twitch fiber Type tended to maintain its size. In animals that did not hibernate, the expressions of fast-twitch muscle fiber markers and myostatin that inhibit muscle growth were not reduced. These results suggest that a decrease in fast-twitch fibers may be involved in the achievement of hibernation in the Syrian hamster.

### Late Breaking Abstracts

[3LBA]
**Nutritional and metabolic physiology, Thermoregulation**

March 16 (Thu.), 12:10 - 14:10, Poster Room

### [3LBA-018]

### Prolonged consumption of sugar-sweetened beverages (SSBs) alters sugar metabolism in the small intestine and further contributes to obesity

*Sachiko Sato[1], Arif UL Hasan[1], eiichi Taira[1] ([1]*Iwate medical Univ.*)

It is now well established that excessive consumption of sugar-sweetened beverages (SSBs) is directly associated with increased obesity and obesity-induced diseases such as metabolic syndrome.We examined changes in body weight, feeding behavior, and glucose transportation in the small intestine of mice fed SSBs for certain periods of time. Male ddY mice were divided into two groups, water and sucrose beverage, and kept individually for 8 weeks. Drinks and foods were freely accessible. Body weight, amount of food intake and water consumption were measured over time, and after 8 weeks, glucose tolerance test was performed to check blood levels of glucose and insulin. In addition, gene expression levels related to glucose transportation in the small intestine by qPCR. The results showed that the body weight of the sucrose-group increased significantly compared to the water-group. In addition, the sucrose group consumed less food, while drinking more sucrose beverages.In the glucose tolerance test, the blood glucose level increased rapidly while the insulin level was low, suggesting that the intake of sugar reduced insulin secretion.In addition, the expression of the sugar transporters, GLUT2, GLUT5 and SGLT1 in the small intestine were significantly increased, suggesting that they may alter sugar transportation ability resulting in improving sugar absorption.

### [3LBA-019]

### FGF21-inducing sugars suppress alcohol drinking throough the FGF21-oxytocin system

Sho Matsui[1], Yuma Takahashi[1], Shuhei Morioka[1], Yasuo Oguri[1], Satoshi Tsuzuki[1], *Tsutomu Sasaki[1] ([1]*Kyoto University*)

Alcohol drinking is difficult to treat partly because drinkers' adherence to medications is low. Because alcohol is often consumed with food, studying drinking as an ingestive behavior may provide different perspectives and solutions from substance abuse research. Here, we used genetically oxytocin neuron-specific FGF21 receptor conditional knockout mice to demonstrate that the FGF21-oxytocin system physiologically regulates alcohol drinking. We identified four rare sugars (D-allulose, D-sorbitol, D-tagatose, and D-xylitol) that can significantly induce FGF21 secretion in primary hepatocytes compared to glucose and fructose. The FGF21-inducing rare sugars reduced preference for ethanol through the FGF21-oxytocin system, but not through GLP-1 in mice. Therefore, stimulating the physiological negative feedback regulator of alcohol drinking nutraceutically could be an acceptable alternative to drinkers and complement medical treatment.

### [3LBA-020]

### The Elucidation of the Mechanism that Controls the Level of FGF21 in Plasma and Protein Preference Induced by the Intake of Low Protein Diet

*Mai Tsukamoto[1], Kanako Kouchi[2], Sho Matsui[2], Yasuo Oguri[2], Satoshi Tsuzuki[2], Tsutomu Sasaki[2] ([1]*Department of Food Science and Biotechnology, Faculty of Agriculture, Kyoto University,* [2]*Laboratory of Nutrition Chemistry, Division of Food Science and Biotechnology, Graduate School of Agriculture, Kyoto University*)

FGF21 (Fibroblast Growth Factor 21) is a hormone that has been suggested to modulate protein intake, and its blood concentration increases with a low protein diet (LPD) intake. We examined changes in protein preference over time after LPD ingestion and searched for amino acid (AA) s that inhibit the increase in blood FGF21 levels associated with LPD ingestion. We examined changes in protein preference over time in a food choice test using LPD vs. high protein diet (HPD) after 2 days of LPD intake and found a temporary increase in preference for HPD. In addition, we searched for AAs that decrease FGF21 secretion by replacing culture media of primary hepatocytes with various AA-added media after culturing them in LAA for 24 hours. We found that the FGF21 concentration decreased in the medium His, Met, Phe, Thr, and Trp added. Finally, we fed mice LPD supplemented with these 5 AAs and measured the blood FGF21 levels. A significant increase in FGF21 was observed in mice fed LPD, but not in mice fed LPD with these AAs.
In conclusion, LPD intake increases protein preference and specific AAs are involved in the suppression of FGF21 secretion.

### Late Breaking Abstracts

[3LBA]
**Behavior, Biological rhythm, Sleep**

March 16 (Thu.), 12:10 - 14:10, Poster Room

### [3LBA-021]

### Effects of inflammation-inducing substance (Poly IC) on fetal movement

*Kikyo Nakahama[1,2], Karin Takemura[1,2], Akira Tamaki[2], Kazushige Nakahara[3], Seiichi Morokuma[3], Akiko Arata[1] ([1]*Faculty of Medicine, Dept. of Physiology, Hyogo Medical University,* [2]*Faculty of Rehabilitation, Dept.of Physical Therapy, Hyogo medical University,* [3]*Dept. of Health Sciences, Graduate School of Medical Sciences, Kyushu University*)

Fetal movement has a great influence on fetal development and survival rate. Our previous study found that fetal movements of rat embryos 15-21 days old (E15-21) corresponded to early human pregnancy, revealing similarities between rat and human fetal movements. It has been reported that maternal viral infection increases the risk of psychiatric disorders such as schizophrenia, mental retardation and autism, however, the effects of fetal infection on motor function and differences in symptoms depending on the timing of inflammatory exposure have not been clarified. In this study, we examined whether there is a difference in the motor coordination function and behavior of rat pups depending on the timing of inflammatory exposure during the fetal period. Pregnant rats were intraperitoneally injected with an inflammation-inducing substance (Poly IC) to make infection symptoms. Administration of Poly IC reduced the fetal movements and increased peristaltic movement, suggesting that infection may reduce fetal movements. We examined the relationship between fetal movement and motor coordination planed the following Poly IC administration tests; Group A: 2 administrations on embryonic 18-19 day (E18-19) (n=2), Group B: administration 3 times on E17-19 (n=12), Group C: administration 3 times on E19-21 (n=14), Group D: administration 4 times on E14-17 (n=14), Group E: administration 4 times on E17-20 (n=12), Group F: administration 4 times on E18-21 (n=14), and a total of 7 groups of 20 rat pups in the control group that were not administered Poly IC for comparison with normal development. Then, we compared the motor coordination function and behavior. A fixed-point camera was set up on the above rat pups, and observed the rolling over behavior after birth. On the 2nd and 3rd days after birth, the time required for rolling over was longer in all groups administered with Poly IC than in the control group. In addition, it took more time to rolling over, especially in the group exposed to infection during the last trimester of pregnancy in the order of Group F, Group E, Group D. As for the number of births, a decrease and stillbirths were observed as the exposure time approached the end of pregnancy. The results of this study suggest that the longer the period of exposure of pregnant mothers to inflammatory substances and the closer to the end of pregnancy, the greater the effect on the fetus. It was suggested that fetal movement is an important movement in the early stages of a child's life.

### [3LBA-022]

### Establishment of temperature preference test by assessing water drinking behavior

*Kunitoshi Uchida[1,2], Shori Ishikawa[1], Miyu Akiyama[1], Tetsuya Hosooka[2,3], Azusa Uchida[1] ([1]*Lab. Funct. Physiol., Sch Food Nutr. Sci., Univ. Shizuoka,* [2]*Grad. Sch. Integr. Pharm. Nutr. Sci., Univ. Shizuoka,* [3]*Lab. Nutr. Physiol., Sch Food Nutr. Sci., Univ. Shizuoka*)

Temperature detection and temperature preference are critical for the maintenance of thermal homeostasis and protection from potentially injurious temperature extremes. The methods that measure the time spent on plates with different temperatures such as the two-plate and the thermal gradient tests were usually used to evaluate temperature preference. The problems of these methods are that a temperature gradient is generated between the plate and the air layer, and small animals are susceptible to this temperature gradient. Therefore, we believed that development of the new temperature preference methods by assessing parameters other than plate temperature. In this study, we established the temperature preference test by assessing water drinking behavior modifying the two-bottle taste preference test. We found that mice in a normal environment (23°C) avoid drinking water more than 40°C. Moreover, while mice in the normal environment (23°C) preferred 10°C and 30°C water equally, mice in a hot environment (35°C) prefer to 10°C water compared to 30°C water. Thus, this temperature preference test could be used to evaluate temperature preference depending on ambient temperature changes.

### [3LBA-023]

### Identification of sleep-regulating genes by suppressor screening using *Sik3sleepy* mutant mice

*Chika Miyoshi[1], Noriko Hotta-Hirashima[1], Satomi Kanno[1], Miyo Kakizaki[1], Aya Ikkyu[1], Masashi Yanagisawa[1,2,3], Hiromasa Funato[1,4] ([1]*WPI-IIIS, University of Tsukuba,* [2]*University of Texas Southwestern Medical Center,* [3]*TARA, University of Tsukuba,* [4]*Department of Anatomy, Faculty of Medicine, Toho University*)

We conducted a large-scale screening of randomly mutagenized mice with EEG/EMG-based sleep/wake monitoring, and established the *Sleepy* mutant pedigree (Funato, Miyoshi et al., Nature 2016). *Sleepy* mice have a gain-of-function mutation in the *Sik3* serine/threonine kinase gene and show an increased non-REM sleep time and depth. Recently we demonstrated that the signaling pathway LKB1-SIK3-HDAC4 regulates the quantity and quality of non-REM sleep by acting in the hypothalamus and cortex, respectively (Kim, Miyoshi et al., Nature 2022). However, the upstream and downstream components for the SIK3 pathway in sleep regulation have not been completely elucidated. Here, we randomly induced a second mutation by administering the mutagen ENU to *Sik3*$^{sleepy}$ male mice, and attempted to identify mutations that can rescue the hypersomnia phenotype in *Sik3*$^{sleepy}$ mice.

### [3LBA-024]

### Anxiety-like behaviors induced in the chronic stage of pain in the partial sciatic nerve rats

*THU NGUYEN DANG[1], Son Nguyen Tien[1], Shiori Narimatsu[1], Naoto Fujita[1], Norimitsu Morioka[2], Susumu Urakawa[1] ([1]*Dept. of Musculoskeletal Functional Research and Regeneration, Graduate School of Biomedical and Health Sciences, Hiroshima University,* [2]*Dept. of Pharmacology, Graduate School of Biomedical and Health Sciences, Hiroshima University*)

A full understanding of the role of pain progression and emotional disorders has not been elucidated. Here we investigated the progressive time course of neuropathic pain in the development of emotional behaviors. Eight-week-old male Wistar rats were used in a partial sciatic nerve ligation (pSNL) model. The elevated plus maze (EPM) and open field (OF) tests were used to assess emotional-like behaviors every two weeks until 8 weeks after surgery. We confirmed that the pSNL rats reduced the mechanical withdrawal threshold in the surgical hind paw after surgery in the von Frey test. Those rats displayed anxiety-like behaviors in the EPM (decrease in time spent, number of moving and head dip in the open arm; decrease in the ratio of rearing in the open arm to rearings in the closed arm) and the OF (decrease in time spent in the central zone; increase in the ratio of rearing in last 5 minutes to rearing in first 5 minutes) from 4 weeks after surgery. No significant difference in these parameters was observed at 2 weeks post-surgery. In the EPM, at eight weeks post-surgery, there is a significant decrease in the total number of rearing and an increase in the inactive duration in pSNL rats, suggesting a depressive-like behavior. These findings indicate that pSNL rats develop anxiety-like behaviors in the chronic stage of neuropathic pain, which is not shown clearly in the acute phase.

### Late Breaking Abstracts

[3LBA]
**Drug Action, Pharmacology**

March 16 (Thu.), 12:10 - 14:10, Poster Room

### [3LBA-025]

**2-Ethylpyrazine, a coffee aroma component, increases locomotor activity in mice**

*Madoka Tsujimoto[1], Junichi Tanaka[2], Yasushi Hayashi[1] ([1]*Notre Dame Seishin Univ.*, [2]*Naruto Univ. of Education*)

Previous studies revealed that the aroma of Brazilian coffee beans (Brazil aroma) enhances alertness and cognitive function in humans. We also found that Brazil aroma increases the locomotor activity of fatigue-loaded mice by behavioral analysis. To investigate the components related to the function of Brazil aroma, we performed a correlation analysis between Brazil aroma components and mouse locomotor activity. Several components that could be related to the increased locomotor activity were found. Based on these results, in this study, we attempted to clarify the effect of each aroma component on mouse open-field behavior. Additionally, we measured the plasma catecholamine levels after the component exposures. Male ICR mice aged 7-9 weeks were used. 2-Phenylethanol, 2,3,5-trimethylpyrazine, 2-ethylpyrazine, β−damascenone, and 2-ethyl-3,5-dimethylpyrazine were selected as coffee aroma components. In the open-field test, mouse behavior was observed for 60 min by using the video tracking system under the aroma exposures. Plasma catecholamine levels were measured by HPLC-ECD system. Of the components tested, 2-ethylpyrazine enhanced locomotor activity and defecation. Plasma catecholamine levels increased after 2-ethylpyrazine exposure. These results show that 2-ethylpyrazine is an essential component of Brazil aroma, which enhances alertness in animals.

### [3LBA-026]

**Comparative study of metabolome and transcriptome profiling on lung cancer cells with different sensitivity to statins**

*Tomoko Warita[1,2], Nanami Irie[3], Yaxuan Zhou[3], Jiro Tashiro[4], Akihiro Sugiura[4], Kana Mizoguchi[2], Kiyoshi Ohtani[1,2,3], Takuro Ishikawa[5], Katsuhiko Warita[4], Zoltán N. Oltvai[6] ([1]*School of Biological and Environmental Sciences, Kwansei Gakuin University,* [2]*School of Science and Technology, Kwansei Gakuin University,* [3]*Graduate School of Science and Technology, Kwansei Gakuin University,* [4]*School of Veterinary Medicine, Tottori University,* [5]*Department of Anatomy, Aichi Medical University,* [6]*Department of Pathology and Laboratory Medicine, University of Rochester*)

Statins are potential therapeutic agents for attenuating metastasis. However, the mechanisms that mediate the effects of statin, the homeostatic responses of tumor cells to statin therapy, and the modes underlying the antitumor effects of statins remain unclear. To uncover the effects of statins on cancer cells in vitro, we performed transcriptome and metabolome analyses on atorvastatin-treated, statin-resistant and statin-sensitive lung cancer cells. Results of Gene Ontology and pathway enrichment analyses showed that after 24 h of atorvastatin treatment, the expression levels of cell cycle- and DNA replication-related genes were significantly downregulated in statin-sensitive cancer cells. Similarly, altered levels of substances of purine and polyamine metabolism, glycolysis, and the pentose phosphate pathway indicated cell type-specific metabolic changes, including those in the levels of intracellular redox state. In turn, hydrogen peroxide treatment potentiated the growth inhibitory effect of atorvastatin in mesenchymal cells but not in epithelial lung cancer cells. Thus, innate differences in cell type-specific baseline metabolite levels and the capacity for drug treatment-induced stress responses could determine the susceptibility of cancer cells to statins.

### [3LBA-027]

**Free radical direct scavenging activity of sitagliptin as an antioxidant**

*Misato Nakatake[1,2], Kazue Ogata[2], Ryohei Umeda[3], Hiroki Takanari[4], Osamu Tokumaru[2] ([1]*Oita University Graduate School of Welfare and Health Suiences,* [2]*Department of Physiology, Faculty of Welfare and Health Sciences, Oita University,* [3]*Department of Advanced Medical Sciences, Oita University,* [4]*Institute of Post-LED Photonics, Tokushima University*)

PurposeSitagliptin is one of the representative DPP-4 inhibitors. Recently, it is reported that sitagliptin is preventive against oxidative stress and inflammation. However, detailed mechanism is not fully described. We aimed to study free radical scavenging activity of sitagliptin against multiple free radicals. MethodsFree radical scavenging activity of sitagliptin was evaluated against nine species of free radicals by electron spin resonance spectroscopy with the spin-trapping method. From dose-response curves, reaction rate constants with free radicals examined were estimated. Antioxidative activity against lipids was assessed by TBARS assay. ResultsSitagliptin significantly scavenged the following three free radical species in dose-dependent manners; hydroxyl radical, ascorbyl free radical and singlet oxygen. Sitagliptin did not scavenge *tert*-butoxyl radical, *tert*-butyl peroxyl radical, DPPH, superoxide anion, nitric oxide and tyrosyl radical. Sitagliptin significantly inhibited oxidation of lipids in mice brain tissue. ConclusionsSitagliptin dose-dependently scavenged multiple free radicals including hydroxyl radical. We speculated that the direct free radical scavenging activity of sitagliptin might contribute to its antioxidative activity *in vitro* and eventually *in vivo*.

### Late Breaking Abstracts

[3LBA]
**Others**

March 16 (Thu.), 12:10 - 14:10, Poster Room

### [3LBA-028]

### Comparison of PDGF-BB Concentrations between Platelet-rich Plasma and Platelet–poor Plasma Derived from Rats

*Haruka Takemura[1,2], Takayuki OKumo[1,2], Taro Kimura[1,2], Hideshi Ikemoto[1], Yasunori Takayama[1], Naoki Adachi[1], Koji Kanzaki[2], Masataka Sunagawa[1] (*[1]Department of Physiology, Showa University School of Medicine, [2]Department of Orthopedic Surgery, Showa University Fujigaoka Hospital*)

Platelet–rich plasma (PRP) has been kept eyes on regenerative medicine, which is prepared by single or double centrifugation procedure. Previous studies suggest that platelet–derived growth factor–BB (PDGF-BB), which is said to be rich in PRP, contributes to tissue repair activating migration and proliferation of the mesenchymal stem cells, and angiogenesis. In this study, PDGF-BB concentrations in PRP and platelet–poor plasma (PPP) were measured comparatively by ELISA using rats' whole blood. Three 9-week-old male Wistar rats were used. Under general anesthesia, 8 ml of whole blood was aspirated from the common iliac vein. Of that, 4 ml was used to prepare PRP and the remaining 4 ml as PPP. For PRP preparation, double centrifugation procedure was applied (400×*g* for 7 min, and 2000×*g* for 10 min). On the other hand, PPP preparation was performed by centrifugation at 1000×*g* for 20 min after the blood was steadily set for 30 min. As a result, PDGF-BB in the PRP group was 2.02 times more concentrated than that in the PPP group ($p < 0.05$). In conclusion, PRP is thought to be effective for tissue regeneration thanks to highly concentrated growth factors.

### [3LBA-029]

### Comparison of TGF-b Concentrations between Platelet-rich Plasma and Serum

*Takayuki Okumo[1,2], Atsushi Sato[3], Haruka Takemura[1,2], Hideshi Ikemoto[1], Yasunori Takayama[1], Naoki Adachi[1], Masataka Sunagawa[1] (*[1]Department pf Physiology, Showa University School of Medicine, Tokyo, Japan, [2]Department of Orthopedic Surgery, Showa University Fujigaoka Hospital., [3]Department of Orthopedic Surgery, Showa University Koto Toyosu Hospital*)

Platelet–rich plasma (PRP) has been kept eyes on regenerative medicine, which is prepared by single or double centrifugation procedure. Previous studies suggest that tissue growth factor-b (TGF-b), which is said to be rich in PRP, activates migration and proliferation of the mesenchymal stem cells, which contributes to tissue repair. In this study, TGF-b concentration in PRP and serum was compared measured by ELISA. Nine patients (6 males and 3 females, mean age: 36.7 years) were subjected in this study. Leukocyte-rich PRP was prepared using PEAK® PRP SYSTEM (DePuy Synthes Mitek Sports Medicine, Raynham, Massachusetts, USA). On the other hand, whole blood was steadily set for 30 minutes and centrifuged at 1000×*g* for 20 minutes for serum preparation. As a result, TGF-b in PRP was 18.87 times more concentrated than that in serum ($p < 0.001$). In conclusion, PRP is thought to be effective in tissue regeneration thanks to highly concentrated growth factors.

### [3LBA-030]

### PTBP1 drives c-Myc-dependent gastric cancer progression and stemness

*Tengyang Ni[1,2], Haibo Wang[2], Shiyu Guo[1], Masataka Sunagawa[1], Yanqing Liu[2] (*[1]Department of Physiology, School of Medicine, Showa University, [2]Institute of Translational Medicine, Medical College, Yangzhou University*)

Background Gastric cancer (GC) tumorigenesis and treatment failure are caused by cancer stem cells. Polypyrimidine tract binding protein 1 (PTBP1) is now being considered as a therapeutic target for tumor progression and stem cell characteristics. Methods PTBP1 expression in GC samples was detected using tissue microarrays. Proliferation, colony-formation, spheroid formation, and stem cell analysis were used to examine PTBP1's role in tumorigenesis and stem cell maintenance. In AGS and HGC-27 cells with or without PTBP1 deficiency, ubiquitin-related protein expression and co-precipitation assays were performed. Results We identified that PTBP1 was aberrantly highly expressed and represented a novel prognostic factor in GC patients. PTBP1 maintained the tumorigenic activity and stem cell characteristics of GC *in vitro* and *in vivo*. PTBP1 directly interacts with c-Myc and stabilizes its protein levels by preventing its proteasomal degradation. Conclusions By preserving the stability of c-Myc through the ubiquitin-proteasome pathway, the oncogene PTBP1 supports stem cell-like phenotypes of GC and is involved in GC progression.

### [3LBA-031]

### Study on the effect and mechanism of aqueous extract of Chinese herbal prescription (TFK) in treating gout arthritis

*Haibo Wang[1], Tengyang Ni[1,2], Shiyu Guo[2], Masataka Sunagawa[2], Yanqing Liu[1,2] (*[1]Yangzhou University, [2]Showa University*)

Objective: To provide an experimental basis for the development of high-effective and low-toxic natural compounds for the treatment of gout arthritis. Methods: Gouty arthritis model and hyperuricemia model were used to test the anti-inflammatory effect of the Tong Feng Kang (TFK) compound. An automatic biochemical analyzer was used to measure the levels of serum uric acid and urinary uric acid, and a protein thermal fluorescence chip was used to measure the expression of gout-related immunoinflammatory factors. ELISA assay was used to detect the effect of TFK compound on macrophage activation. The hematoxylin-eosin staining of the organs were examined to observe tissue damage. Results: Compared with the model group, the TFK compound had the effect of reducing swelling and anti-inflammation, and could significantly reduce the levels of blood uric acid, urine uric acid, the expression of inflammatory cytokines and the number of neutrophils in the hyperuricemia rats, while inhibiting the activation of macrophages. Conclusion: These findings indicate that TFK compound inhibits the enrichment of neutrophils and the activation of macrophages in the early stage of inflammation.

### [3LBA-032]

### Architecture and function of terminal Schwann cells in mechanosensory corpuscles

*Sviatoslav Bagriantsev[1] (*[1]Yale University*)

Mammalian Meissner corpuscles and their avian analogs corpuscles of Grandry detect transient touch and vibration, but their ultrastructure and mechanism of function are poorly understood. The sensory core of corpuscles contains the terminal neurite(s) of the mechanoreceptor afferent surrounded by Schwann cell-derived lamellar cells. The afferent is thought to be the sole touch-sensing element within the corpuscle, whereas the role of lamellar cells is unknown. Here, we present a high-resolution three-dimensional ultrastructure of an avian Meissner (Grandry) corpuscle in intact skin acquired using the enhanced focused ion beam scanning electron microscopy (FIB-SEM), followed by machine learning based segmentation and reconstruction. We show that the afferent splits within the corpuscle into several disk-shaped endings layered between flattened lamellar cells. Each lamellar cell contains numerous exocytotic dense core vesicles and forms large-area contacts with adjacent afferent disks. Together with single-corpuscle RNA sequencing and electrophysiology, our FIB-SEM data reveal lamellar cells as secretory sensors of touch. We developed a method for direct electrophysiological recordings of afferent activity from an individual corpuscle in the skin. Using this approach, we show that activation of a single mechanosensitive lamellar cell is sufficient to trigger action potentials in the afferent, providing the first evidence that lamellar cells in corpuscles are active detectors of touch. These results reveal comprehensive architecture of Grandry corpuscle and demonstrate a dual sensory mechanism comprised of a mechanoreceptor afferent and lamellar cells. This work is supported by grants from NSF (IOS-1923127) and NIH (1R01NS097547).
